# The Establishment of Hypertrophic Cardiomyopathy Diagnosis Model via Artificial Neural Network and Random Decision Forest Method

**DOI:** 10.1155/2022/2024974

**Published:** 2022-09-15

**Authors:** Shuanglei Li, Zekun Feng, Cangsong Xiao, Yang Wu, Weihua Ye

**Affiliations:** ^1^Division of Adult Cardiac Surgery, Department of Cardiology, The Sixth Medical Center, Chinese PLA General Hospital, Beijing 100037, China; ^2^Division of Pediatric Cardiac Surgery, Department of Cardiology, The Sixth Medical Center, Chinese PLA General Hospital, Beijing 100037, China

## Abstract

Hypertrophic cardiomyopathy is a hereditary disease characterized by asymmetric ventricular hypertrophy as the key anatomical feature. Currently, there exists no effective method for the early diagnosis of hypertrophic cardiomyopathy. In this analysis, we incorporated multiple GEO datasets containing RNA profiles of hypertrophic cardiomyopathic patient tissues, identified 642 differentially expressed genes, and performed GO and KEGG analyses. Furthermore, we narrowed down 46 characteristic genes from these differentially expressed genes using random decision forests and conducted transcription factor regulation analysis on them. Using 40 genes that showed overlap between the training set and the verification set, the artificial neural network was trained, and the final MPS scoring model was constructed, and a receiver-operating characteristic (ROC) curve was drawn. We used the MPS model to predict the verification dataset and drew the ROC curve, which demonstrated the good prediction performance of the model. In conclusion, this study combines a random decision forest and artificial neural network to build a diagnostic model for hypertrophic cardiomyopathy to predict the disease, aiming at early detection and treatment, prolonging the survival time, and improving the quality of life of patients.

## 1. Introduction

Hypertrophic cardiomyopathy (HCM) is a common inherited heart disease characterized by unexplained left ventricular hypertrophy. HCM occurs in about 1/500–1/200 of the general population [1, 2]. Among the HCM patients, about two-thirds have left ventricular outflow tract (LVOT) obstruction, known as hypertrophic obstructive cardiomyopathy or obstructive hypertrophic cardiomyopathy (OHCM) [3]. For patients with drug-refractory OHCM, myectomy is the primary treatment for alleviating LVOT obstruction [4, 5]. Previous studies have shown that myectomy can change the natural clinical course in OHCM patients, and postsurgery, most patients can achieve a life expectancy similar to normal people of the same age [6]. However, some patients still experience long-term adverse events after the surgery. Studies have identified several preoperative clinical risk factors to optimize patient risk stratification and management [7–9]. Preoperative risk factors included increasing age, increased preoperative N-terminal probrain natriuretic peptide levels, and increased left atrial diameter. Identifying high-risk patients and implementing early interventions are of great clinical significance for patient management. Therefore, it is essential to explore the relevant risk factors which affect the long-term prognosis of patients with hypertrophic cardiomyopathy to optimize postoperative clinical management strategies.

HCM can lead to adverse outcomes such as sudden arrhythmic death [10], heart failure [11], and atrial fibrillation which may lead to embolic stroke [12]. Significant progress has been made in understanding HCM, but its pathogenesis is still not fully understood. As a result, several patients do not respond well to the treatments resulting in clinical symptoms and substantial reductions in life expectancy. Several traditional diagnostic methods for hypertrophic cardiomyopathy exist such as dynamic auscultation of cardiac murmur changes, the discovery of myocardial hypertrophy and left ventricular outflow tract obstruction using an electrocardiogram (ECG), and cardiac magnetic resonance imaging (CMRI) [13–15]. However, genetic testing to probe for the presence of disease-causing genes and mutations offers tremendous advantages over traditional methods. It could screen first-degree relatives or fetuses even before birth, taking the diagnosis of HCM to a new level. As genetic testing guides the diagnosis of HCM, identifying asymptomatic HCM patients before the onset of clinical disease and subsequent treatment has become a class I recommended aid in European and North American guidelines [16–18]. Therefore, comprehensive analysis of the gene signature associated with HCM and exploring the pathogenesis of HCM at the molecular level play an extremely important role in the diagnosis, prevention, treatment, and prediction of the prognosis of the disease [19].

Deep learning methods are emerging as an important tool in clinical diagnosis. We hypothesized that a deep learning method based on the characteristic gene signatures of HCM could be used to develop a diagnostic model for HCM. Towards this goal, we used random decision forests and artificial neural networks. A random decision forest is a classifier that contains multiple decision trees and is a supervised learning method. It evaluates the importance of variables when deciding on categories, making it well suited for feature filtering. On the other hand, an artificial neural network is an algorithmic mathematical model that simulates the behavioral characteristics of animal neural networks and parallel distributed processes. It is similar to a biological neuron structure and, like any neural network processing framework, has an input layer, an implicit layer, and an output layer. In this study, we aimed to develop a diagnostic model for hypertrophic cardiomyopathy using random decision forest and artificial neural networks to understand disease pathology and aim at early detection and treatment. The diagnostic model could thereby help prolong the survival time of patients and improve their quality of life.

## 2. Materials and Methods

### 2.1. Gene Expression Omnibus (GEO) Data Download

GSE36961 and GSE141910 were downloaded from GEO (https://www.ncbi.nlm.nih.gov/geo/) for the construction of the diagnostic model and for verifying the performance of the model, respectively. The training dataset GSE36961 consisted of transcriptomic profiling of 145 surgical myectomy tissue samples, as shown in Table 1, which included 106 HCM and 39 normal cardiac tissue samples. HCM samples were used as the case group and normal cardiac tissue samples were used as the control group in the subsequent analysis. The validation dataset GSE141910 included 366 samples in total (see Table 1 for details). To ensure consistency with the training set samples as much as possible, in this study, we selected 28 HCM samples as the case group and 166 nonfailing donors as the control group.

### 2.2. Screening Differentially Expressed Genes

The gene expression data were analyzed by using the R software package limma to obtain the differentially expressed genes between HCM and normal myocardial tissues in the training dataset GSE36961. The differentially expressed genes between HCM (case) group and the normal myocardial tissue (control) group in the training dataset were screened based on the multiple of difference (fold change [FC] > |1.5|) and significance (*p* value < 0.05). Using these cut-offs, a total of 642 differentially expressed genes were identified, which included 250 upregulated and 392 downregulated genes.

### 2.3. Enrichment Analysis

Enrichment analysis of differentially expressed genes was performed using the R-package clusterProfiler, which uses the Gene Ontology (GO) and KEGG pathway databases. Through GO analysis, we obtained the biological process (BP), cellular components (CC), and molecular function (MF) of differentially expressed genes. Through KEGG analysis, we obtained the abundant signaling pathways involved with the differentially expressed genes. *p* < 0.05 was defined as statistically significant enrichment. The top 10 pathways or subgroups of gene enrichment were selected to draw the bubble map.

### 2.4. Random Decision Forest Screening for Characteristic Gene

A random decision forest is a classifier that contains multiple decision trees and is a supervised learning method. It evaluates the importance of variables when deciding on categories, making it well suited for feature filtering. We used the randomForest package in R to construct a random decision forest model and screen the characteristic genes.

The filtering steps used are as follows:

1. The differentially expressed genes were used to train the random decision forest model, and the genes with the negative characteristic importance index (mean decrease accuracy) are deleted to obtain a new expression matrix
2. The random decision forest model is retrained with the new expression matrix, genes with negative feature importance are deleted, and the step is cycled until the feature importance no longer has a negative value. This results in a primary characteristic gene set
3. The above two steps are repeated 10 times resulting in 10 primary characteristic gene sets. The genes that appeared more than three times were screened out as the final set of characteristic genes

### 2.5. Analysis of Transcription Factor (TF) Regulation of Characteristic Genes

The human TF regulatory network data were downloaded from the TR TRRUST database (https://www.grnpedia.org/trrust/), and the TF regulatory network data of characteristic genes were obtained. The regulatory network diagram was drawn using Cytoscape software.

### 2.6. Training and Effectiveness Evaluation of Neural Network

An artificial neural network is an algorithmic mathematical model that simulates the behavioral characteristics of animal neural networks and parallel distributed processes. It is similar to a biological neuron structure and, like any neural network processing framework, has an input layer, an implicit layer, and an output layer. We used the nnet package in R to train the feedforward back-propagation neural network. Nnet is an R-package used to fit a single hidden layer neural network. To facilitate the construction of the MPS model, we selected the number of hidden nodes as size = 1. The maximum number of iterations was set to maxit = 200.

The back-propagation neural network we used is an important branch of neural networks. The learning of neural networks depends heavily on back-propagation, and the back-propagation of error is similar to learning from error. The errors are corrected by themselves in each iteration until a convergence point is reached, and the learned weights and deviations were obtained. The purpose of backpropagation is to correct the weight of each layer and minimize the overall error of the output layer, both to optimize the overall loss function *L* derived as follows:

(1) $$\begin{array}{c} \cos t=L\left(W,B\left|\left(x_{1},y_{N}\right)\right.\right)=\frac{1}{N}\overset{N}{\underset{i=1}{\sum }}L_{i}\left(W,B\left|\left(x_{i},y_{i}\right)\right.\right). \end{array}$$

Starting from the objective function, the local gradient of each node (operation) in each layer is solved layer by layer. According to the following chain rule:

(2) $$\begin{array}{c} \begin{array}{c} y=f\left(u\right),u=f\left(x\right),\\ \frac{\partial y}{\partial x}=\frac{\partial y}{\partial u}\cdot \frac{\partial u}{\partial x}. \end{array} \end{array}$$

The partial derivative of the cost function for a neuron in the whole network from the following derivation:

(3) $$\begin{array}{c} \frac{\partial C}{\partial w_{j}}\approx \frac{C\left(w+\varepsilon e_{j}\right)-C\left(w\right)}{\varepsilon }. \end{array}$$

The partial derivatives products of the cost function flowing through all fingers of this parameter are superimposed so that the partial derivatives of all parameters can be obtained by one operation, that is, the gradient of the cost function. The gradient is transferred layer by layer from back to front.

### 2.7. Constructing MPS Molecular Diagnosis Model

Although the prediction of the neural network model is very good, the hidden layer and the nonlinear activation functions make the interpretability of the model low. We deleted the deviation in the model and the weights of some hidden layers and obtained a linear model on the characteristic genes, which enhances the interpretability of the model.

## 3. Results

### 3.1. HCM Expression Data and Preprocessing

We used GSE36961, a transcriptomic profiling database from HCM patients downloaded from GEO as a training dataset. Another dataset GSE141910, which included transcriptome profiling from nonfailing donors was used as the verification set and preprocessed to obtain gene expression data. We performed data pretreatment as follows: the downloaded dataset is used as log2-transformed quantity-normalized signal intensity. First, the probe is mapped to the gene, and the empty probe is removed. In case multiple probes correspond to the same gene, we selected the probe with the highest median and used it to calculate the expression value of that gene.

### 3.2. Screening Differential Genes

**Figure 1:**
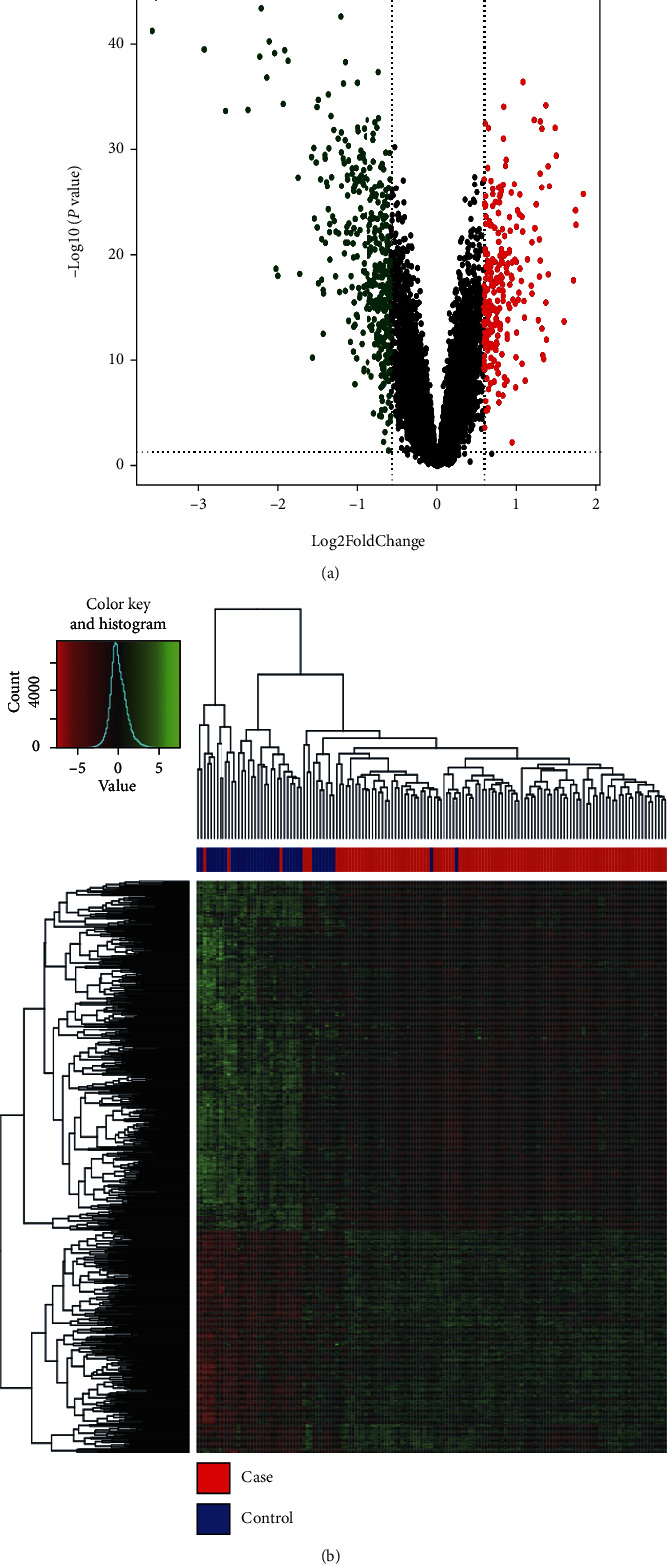
Differentially expressed genes. (a) Volcano plot. The abscissa is log_2_FoldChange, and the ordinate is -log_10_*p*value. The red and green spots are the genes with FC > 1.5 and *p* value < 0.05, and the green spots are the genes with FC < 1.5 and *p* value < 0.05. (b) Heatmap display of differentially expressed genes. The expression quantity is converted into *Z*-score, and the color in the graph changes from red to green, indicating that the expression changes from low to high. In the strip chart on the upper side of the graph, red represents HCM sample, and blue represents control sample.

A total of 642 differentially expressed genes were identified by comparing the HCM tissue samples and the control tissues from the training dataset using limma in R as shown in Figure 1. Supplementary table 1 provides details of the differential genes, where up, down, and stable in the group column, respectively, correspond to upregulated and downregulated and unchanged genes. The heatmap was drawn according to the expression values of the differential genes.

### 3.3. Enrichment Analysis

**Figure 2:**
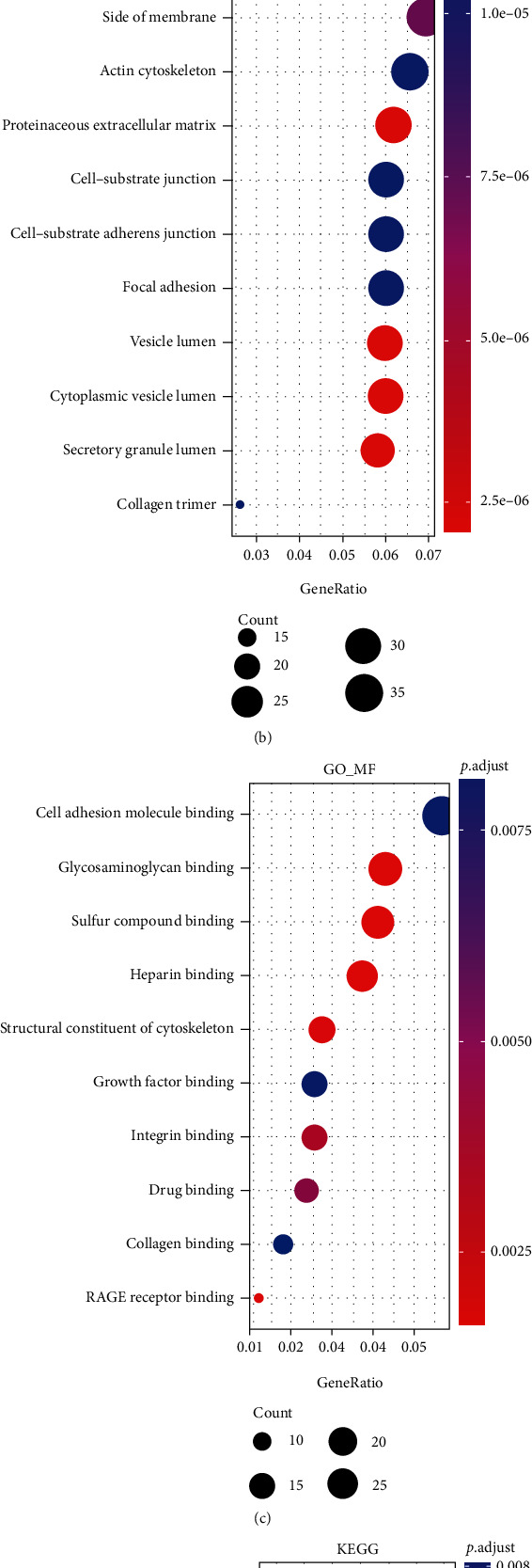
Function and pathway enrichment analyses of DEGs in HCM. (a–c) Significant Gene Ontology terms of the DEGs, including biological processes (BP), molecular function (MF), and cell component (CC). (d) Significant KEGG pathways of the DEGs associated with HCM.

Next, we performed enrichment analysis on 642 differential genes as described in the “Materials and Methods” section, using the clusterProfiler package in R. We performed GO and KEGG analysis on the differential genes and screened out the top 10 pathways to draw a bubble diagram, as shown in Figure 2. In the KEGG analysis, we found that feature genes are associated with apoptosis, JAK-STAT signaling pathway, and HIF-1 signaling. Studies have shown that the formation of pressure-stressed cardiac hypertrophy is accompanied by apoptosis of cardiomyocytes, resulting in progressive loss of effective contractile function units-cardiomyocytes, the enhanced compensatory function of viable cardiomyocytes, and adaptive hypertrophy of cardiomyocytes, deposition of extracellular matrix, and reactive interstitial fibrosis. As the degree of hypertrophy progresses, the increase of myocardial cell apoptosis further reduces the number of myocardial cells, the overall contractility of the myocardium decreases, and the increase of fibrosis reduces the compliance of the left ventricle, and the myocardial contractility cannot exert its due blood ejection. Therefore, a vicious circle is formed, resulting in the decompensation of myocardial function and the occurrence of heart failure. Apoptosis can be seen in the conversion of cardiac hypertrophy to heart failure and plays an important role. Therefore, understanding the mechanism and influencing factors of cardiomyocyte apoptosis is of great significance for blocking or delaying the occurrence and development of cardiac hypertrophy. The JAK/STAT pathway is involved in many important biological processes such as cell proliferation, differentiation, apoptosis, and immune regulation. However, little research has been done on its role in cardiac hypertrophy. Previous studies have also shown that the HIF-1 signaling pathway plays a role in cardiac hypertrophy, and the knockdown of HIF-1*α* attenuated HIMF-induced cardiomyocyte hypertrophy.

### 3.4. Selecting Characteristic Genes to Relate to That Diagnostic Model by Random Decision Forest Screening with Large Sample Data

**Figure 3:**
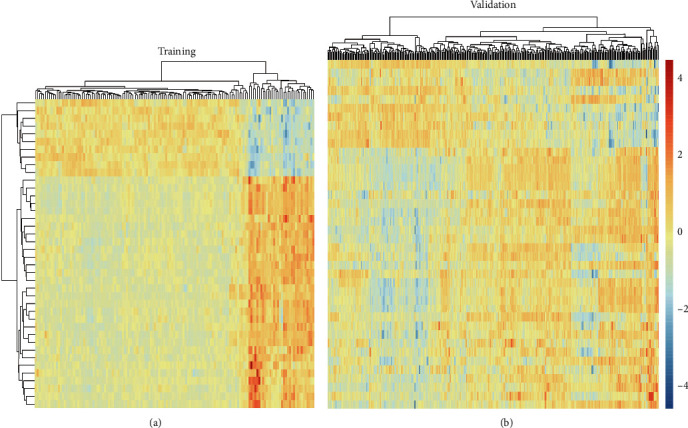
Heatmaps of training and verification set expression data.

We then proceeded to narrow down the characteristic genes from the differentially expressed genes. We selected a total of 46 characteristic genes according to step 4 of the “Materials and Methods” (see Supplementary table 2). We drew the heatmap of the expression levels of these characteristic genes in the training set and the verification set, with the abscissa representing the sample and the ordinate representing the gene, as shown in Figure 3.

### 3.5. Transcription Factor (TF) Regulation Analysis in Characteristic Genes

**Figure 4:**
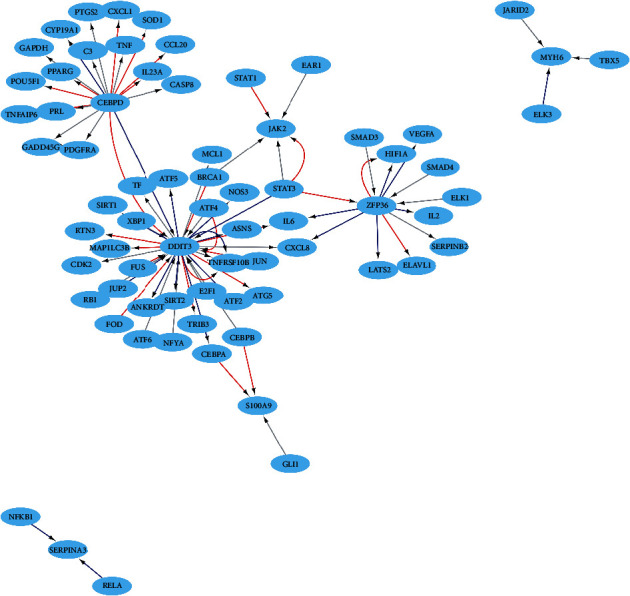
Transcriptional factor regulatory network diagram. The arrows pointed from TF to the target gene. The color of the line was red for activation, blue for repression, and gray for unknown.

To probe the possible biological functions of the included 46 genes, we constructed TF regulatory networks. The result shows that myosin heavy chain 6, *MYH6*, is one of the core genes in the network. Myosin plays an important role in the hypertrophic process of the heart as well as in regulating cardiac function, and myosin heavy chain, *MYHC*, is a key component in the function of myosin. The expression of its encoding genes, *MYH6* and *MYH7*, is implicated in regulatory processes governing cardiac function and is also significantly altered in various myocardial diseases. Further, both genes have been reported as causative genes for hypertrophic as well as dilated cardiomyopathy. Although the two *MYHC* types differ widely in their expression in adults, the ratio can be altered under certain disease conditions indicating that both *MYHC*s may have roles in the development and progression of adult myocardial diseases. Therefore, the study of these two *MYHC* molecules and their genes, *MYH6* and *MYH7*, will give us further insights into cardiac hypertrophy and potentially help us improve the diagnosis and treatment of various other cardiomyopathies (Figure 4).

### 3.6. Construction of an Artificial Neural Network Model

We then sought to construct an artificial neural network model to develop the diagnostic model. We found that six of the 46 genes screened out according to the random decision forest algorithm did not exist in the verification set GSE141910. Therefore, we excluded these six genes from the input variable of the neural network model resulting in 40 genes. It is a prerequisite to standardize the data before constructing the neural network model. Hence, we used the standard deviation method to convert the expression amount to *Z*-score, and used it as the model input (Gene_Score). According to the constructed neural network model, Gene_Weight of each gene was obtained, as shown in Table 2.

### 3.7. Evaluation of Diagnostic Efficiency

The diagnostic model formula was calculated based on the characteristic gene and gene_weight, and the receiver-operating characteristic (ROC) curve was used to evaluate the diagnostic efficiency.

**Figure 5:**
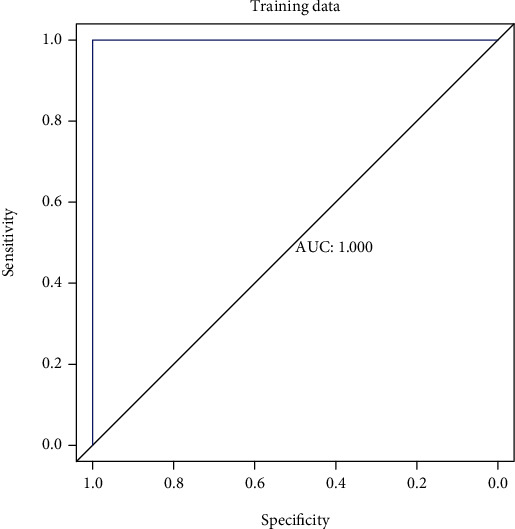
Receiver-operating characteristic (ROC) curve of the training set.

The product of Gene_Score and Gene_Weight of each gene was added up to obtain the MPS value of each sample, as shown in the following formula. The ROC curve was constructed based on the MPS value of the samples and whether they were ill. AUC = 1 may be due to the small sample size (Figure 5).

(4) $$\begin{array}{c} \text{MPS}=\Sigma \;\left(\text{Gene}\_\text{score}*\text{Gene}\_\text{Weight}\right). \end{array}$$

### 3.8. Verification of the Diagnostic Model

**Figure 6:**
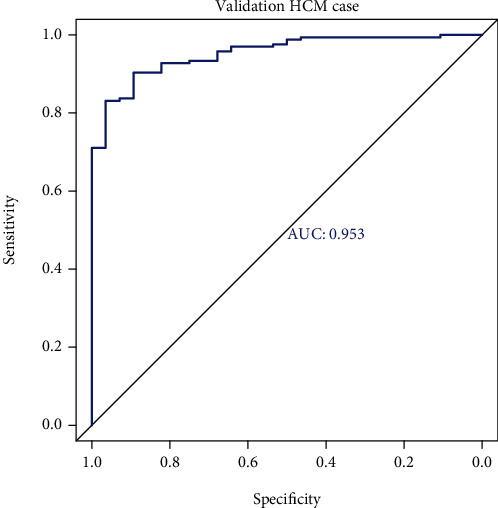
Receiver-operating characteristic (ROC) curve of the validation set.

Using the GSE141910 dataset as the verification set, the disease samples in the GSE141910 dataset included tissues from dilated cardiomyopathy (DCM) and peripartum cardiomyopathy (PPCM) patients in addition to HCM. We selected only the HCM samples and compared them to the control samples and calculated the MPS value of each sample based on the obtained gene_weight and expression value of each gene. According to the MPS value and the disease status of the samples, we made the ROC curve (Figure 6), and the area under the curve (AUC) was calculated to be 0.953.

### 3.9. Prediction Value for Other Types of Cardiomyopathies

**Figure 7:**
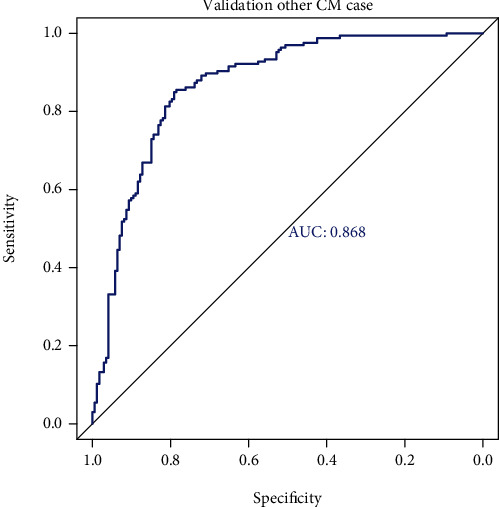
Receiver-operating characteristic (ROC) curve of peripartum cardiomyopathy (PPCM).

DCM and PPCM, like HCM, belong to the category of cardiomyopathy; hence, they might also have similarities in gene networks associated with them. We also used our model to assess whether we can predict DCM and PPCM. Towards this goal, we selected DCM, PPCM, and control samples from the GSE141910 dataset. As described above, the MSP value of each sample was calculated based on the obtained gene_weight and expression value of each gene. According to the MPS value and sample illness, the ROC curve was made (Figure 7), and the AUC was found to be 0.868.

## 4. Discussion

HCM is the most common inherited heart disease characterized by asymmetric hypertrophy of ventricular walls, and its incidence rate is about 1/500 [20]. Although the advances in diagnosis and treatment in recent years have improved the prognosis, the annual death rate is still as high as 1%. At present, hypertrophic cardiomyopathy remains one of the major causes of sudden cardiac death and heart failure in young people and athletes, emphasizing the importance of early identification, especially in high-risk patients [21, 22]. Genetic testing shows its superiority over other diagnostic methods due to the possibility of early detection and high specificity. In particular, the advancement of next-generation sequencing technology has made the detection of gene signatures rapidly thereby enabling screening of first-degree relatives, risk stratification, and prognosis judgment. To date, a total of 27 pathogenic genes and more than 1,500 pathogenic mutation sites for hypertrophic cardiomyopathy have been found, but no consistent conclusion has been reached on the relationship between genotype and phenotype. The majority of the research focused on one or a few classes of genes such as the sarcomere gene and ignored the phenotypic effects of other pathogenic genes, thus biasing the results [23]. In this study, based on the next-generation sequencing data of tissue samples from patients suffering from hypertrophic cardiomyopathy, an artificial neural network and random decision forest method were used to establish a risk prediction model. The accuracy rate of the model in the training set is 100% and the accuracy in the test set was 95.3%. The model accuracy rate and the changing trend of the loss function value of the training and the test sets were similar. This indicates that the model established by the artificial neural networks obtained the generalization ability through the learning process and can classify the new data with the same characteristics as the modeling data.

Currently, deep learning is the most widely used machine learning method in medical research [24, 25]. Due to the huge, complex, and disordered medical data, the traditional machine learning method is not competent for developing models based on medical data [26, 27]. In deep learning, methods such as depth neural network (DNN) and convolutional neural network (CNN) are generally used. Unlike the single-layered structure of traditional computer regression analysis, the neural network is a complex multilayered perception model, which includes three layers, i.e., an input layer, a simulation neuron layer, and an output layer. Unlike traditional regression analysis, neural networks can analyze nonlinear data because of their data processing ability. As long as appropriate input and output layers are selected, and a large amount of clinical data are learned and debugged through the network model, a functional relationship between the input and the output layers with an association relationship that is infinitely close to reality can be found [28, 29]. The use of successfully trained network models has a great role in promoting clinical prediction and treatment.

Conventional clinical diagnosis of HCM is based on other unexplained left ventricular hypertrophy determined by ECG or CMRI [30–32]. Despite the prevalence of hypertrophic cardiomyopathy, the majority of affected individuals may still be undiagnosed, and many have not experienced substantial symptoms or a reduction in life expectancy. Therefore, the coverage population of clinical diagnosis is much lower than that of the diseased population. Compared with traditional disease diagnosis methods, molecular diagnostics such as genetic testing has many advantages. Gene testing is a major tool for early diagnosis of HCM that could potentially prevent poor disease prognosis in contrast; the current testing methods, in general, could only find the disease once it manifests as symptoms and could categorize the disease as early, medium, and so on based on severity. Gene detection, therefore, has the potential to prevent the prognosis of diseases, and in comparison, the traditional physical examination methods cannot play such a role. Further, the traditional physical examination is very passive and cumbersome as many diseases have no obvious early signs or symptoms. In addition, in many instances, once the disease progresses to a certain stage, a lack of medical and surgical tools could fail to prevent adverse effects leaving the patients with substantially reduced quality of life and life expectancy.

In this study, for the first time, we put forward a new model for the diagnosis of hypertrophic cardiomyopathy utilizing transcriptomic profiling and gene strategies in combination with a random decision forest and artificial neural networks. Previous studies have mainly focused on the role of a single gene in the pathogenesis of hypertrophic cardiomyopathy. Our diagnostic model included the differentially expressed genes thereby delineating the genetic features of hypertrophic cardiomyopathy more comprehensively. Our model could update our understanding of the diagnosis and treatment of hypertrophic cardiomyopathy significantly.

The limitation of this study is that the number of samples is small, which could compromise the validity of the study results. Therefore, in future experiments, expansion of sample size is required to verify the predictive value of the model. Also, the age group and ethnicity/race of the study population could mean that the generalization of the results needs to be tested in other population groups. Further, in the future, the scope of the data collection could be expanded to test the hypothetic diagnostic model put forward in this paper.

## 5. Conclusion

In this analysis, we screened out a total of 642 differentially expressed genes from an RNA seq database of HCM and normal patient tissues and conducted GO and KEGG analysis on the differentially expressed genes. Furthermore, we narrowed it down to 46 characteristic genes from the list of differentially expressed genes using random decision forests and conducted transcription factor regulation analysis on the characteristic genes. There were 40 genes with an intersection between the training set and the verification set among the 46 characteristic genes. We used these 40 genes and trained the artificial neural network and constructed the final MPS scoring model and drew the ROC curve. Then, we used the MPS model to predict the verification set and drew the ROC curve, which validated that the model had good prediction performance.

In the era of the constant evolution of healthcare and information technology, several data science and other information technologies are used to personalize healthcare and enhance patient interactions. With the rapid development of computer science and technology, artificial intelligence (AI) and deep learning technology will continue to develop and be applied in the field of cardiovascular disease research. In the future, diagnostic research should involve a large sample size, multiple centers, and diverse population demographics of patients with hypertrophic cardiomyopathy. This would allow AI, machine learning, and deep learning to play a greater role in the diagnosis and treatment of hypertrophic cardiomyopathy.

## Figures and Tables

**Table 1:** Dataset information.

Dataset ID	Platform	Clinical factors	No.
GSE36961	GPL15389	Cardiac tissue, HCM	106
		Cardiac tissue, control	39

GSE141910	GPL16791	Dilated cardiomyopathy, DCM	166
		Hypertrophic cardiomyopathy, HCM	28
		Peripartum cardiomyopathy, PPCM	6
		Nonfailing donor	166

**Table 2:** Feature gene weights.

Gene	Weight	Gene	Weight
ZNF415	-0.3444	MT1X	0.2538
ZFP36	0.4579	MT1M	0.5366
ZDHHC9	0.4951	MT1A	0.4001
TUBA3E	0.2818	MGST1	0.3361
TUBA3D	0.1690	METTL7B	0.4440
TUBA3C	0.1202	MAP3K6	0.3573
TSPYL2	0.7612	LYVE1	0.2352
TKT	0.2494	JAK2	-0.6003
TIPARP	0.2653	IVNS1ABP	-0.6081
SORBS2	-0.4329	INPP1	0.5683
SOCS1	0.5096	IFITM2	0.2351
SERPINA3	0.1677	FCN3	0.3213
SAP18	-0.2174	DYRK1B	-0.3370
S1PR3	0.2383	DDIT3	-0.5131
S100A9	0.1687	CHRDL2	0.6157
RASD1	0.5417	CHN1	-0.1588
RANGAP1	0.5581	CEBPD	0.1254
PRKCD	0.5690	CDC42EP4	0.6638
PHLDB2	-0.7297	C1R	0.1597
MYH6	0.3718	AP3M2	-0.1000

## Data Availability

The data used to support the findings of this study are included within the article.

## Supplementary Materials

Supplementary 1 Supplementary table 1: list of differentially expressed genes.

Supplementary 2 Supplementary table 2: transcriptional regulatory network of the feature genes.
