# Supplementary material for: The Establishment of Hypertrophic Cardiomyopathy Diagnosis Model via Artificial Neural Network and Random Decision Forest Method
Source: Mediators Inflamm. 2022 Sep 15;2022:2024974. doi: 10.1155/2022/2024974 (PMC9500244; doi:10.1155/2022/2024974)
Supplement: Supplementary 2 — Supplementary table 2: transcriptional regulatory network of the feature genes. [file 2024974.f2.pdf]

ranscriptional regulatory network of the feature gene

TF	Target	Mode of Regulation
ATF3	DDIT3	Activation
ATF4	DDIT3	Activation
ATF4	DDIT3	Unknown
ATF6	DDIT3	Unknown
BRCA1	DDIT3	Activation
BRCA1	JAK2	Unknown
CEBPA	S100A9	Activation
CEBPB	DDIT3	Unknown
CEBPB	S100A9	Activation
CEBPD	C3	Unknown
CEBPD	CASP8	Unknown
CEBPD	CCL20	Activation
CEBPD	CXCL1	Activation
CEBPD	CYP19A1	Repression
CEBPD	DDIT3	Activation
CEBPD	DDIT3	Repression
CEBPD	GADD45G	Unknown
CEBPD	GAPDH	Unknown
CEBPD	IL23A	Activation
CEBPD	PDGFRA	Unknown
CEBPD	POU5F1	Activation
CEBPD	PPARG	Activation
CEBPD	PRL	Unknown
CEBPD	PTGS2	Unknown
CEBPD	SOD1	Activation
CEBPD	TNF	Unknown
CEBPD	TNFAIP6	Activation
DDIT3	ANKRD1	Repression
DDIT3	ASNS	Repression
DDIT3	ATF2	Repression
DDIT3	ATF5	Repression
DDIT3	ATG5	Activation
DDIT3	CDK2	Unknown
DDIT3	CEBPA	Repression
DDIT3	CXCL8	Unknown
DDIT3	IL6	Activation
DDIT3	MAP1LC3B	Activation
DDIT3	MCL1	Unknown
DDIT3	NOS3	Repression
DDIT3	RTN3	Activation
DDIT3	SIRT1	Repression
DDIT3	SIRT2	Repression
DDIT3	TF	Unknown
DDIT3	TNFRSF10B	Activation
DDIT3	TNFRSF10B	Repression
DDIT3	TNFRSF10B	Unknown
DDIT3	TRIB3	Activation
E2F1	DDIT3	Unknown
ELK1	ZFP36	Unknown

ELK3	MYH6	Repression
ESR1	JAK2	Unknown
FOS	DDIT3	Activation
FUS	DDIT3	Activation
GLI1	S100A9	Unknown
JARID2	MYH6	Unknown
JDP2	DDIT3	Repression
JUN	DDIT3	Activation
NFKB1	SERPINA3	Repression
NFYA	DDIT3	Unknown
RB1	DDIT3	Unknown
RELA	SERPINA3	Repression
SMAD3	ZFP36	Unknown
SMAD4	ZFP36	Unknown
STAT1	JAK2	Activation
STAT3	DDIT3	Repression
STAT3	JAK2	Activation
STAT3	JAK2	Unknown
STAT3	ZFP36	Activation
TBX5	MYH6	Unknown
XBP1	DDIT3	Unknown
ZFP36	CXCL8	Repression
ZFP36	ELAVL1	Activation
ZFP36	HIF1A	Activation
ZFP36	HIF1A	Repression
ZFP36	IL2	Repression
ZFP36	IL6	Repression
ZFP36	LATS2	Repression
ZFP36	SERPINB2	Unknown
ZFP36	VEGFA	Repression
