# Supplementary material for: The Establishment of Hypertrophic Cardiomyopathy Diagnosis Model via Artificial Neural Network and Random Decision Forest Method
Source: Mediators Inflamm. 2022 Sep 15;2022:2024974. doi: 10.1155/2022/2024974 (PMC9500244; doi:10.1155/2022/2024974)
Supplement: Supplementary 1 — Supplementary table 1: list of differentially expressed genes. [file 2024974.f1.pdf]

List of differentially expressed genes

	logFC	AveExpr	P.Value
TUBA3E	-2.17304548	8.363578315	5.20338E-47
CEBPD	-2.308038963	9.424201214	8.39688E-46
TUBA3C	-2.037360093	7.246174711	1.4436E-45
ZFP36	-2.239531917	9.024541888	3.0698E-45
RASD1	-3.514337543	7.99396139	4.02417E-45
TUBA3D	-2.204856437	9.813431346	4.17833E-44
CDC42EP4	-1.213302811	10.06790815	2.50981E-43
SERPINA3	-3.561409692	8.55788027	5.87007E-42
AXUD1	-2.104494512	8.411439364	5.87929E-41
S100A9	-2.914536944	7.743202495	3.38416E-40
MYH6	-1.914546911	12.77222076	3.98515E-40
FCN3	-2.038046435	8.212979154	7.67333E-40
MT1M	-2.224301096	7.028446005	1.62439E-39
LYVE1	-1.873189921	7.416424672	3.99476E-39
MAP3K6	-1.155977199	9.207012969	5.36836E-39
PRKCD	-0.72957472	7.201549463	4.84733E-38
CD163	-2.136505796	7.465009139	1.57654E-37
YPEL2	1.072454464	8.805297951	3.99272E-37
S1PR3	-1.008836077	7.691426132	4.8107E-37
C1R	-1.182223545	6.970479508	5.6455E-37
FPR1	-1.370626969	6.506236067	6.12488E-36
FCER1G	-1.495600746	7.576523546	1.97707E-35
FKBP5	-1.933816352	8.65775758	4.91261E-35
PIK3IP1	1.355199621	8.980673974	6.51063E-35
AP3M2	0.830505016	8.991696339	8.88162E-35
MYC	-1.510731995	7.368986977	9.46979E-35
MT1X	-2.371958845	9.910865144	1.7682E-34
S100A8	-2.648366209	7.920906097	2.21018E-34
MAFB	-1.338778522	8.025339956	6.88789E-34
CMTM7	-0.725549695	7.412721572	1.08283E-33
SLA	-0.877378017	6.421984927	1.57998E-33
JAK2	1.210077892	8.484025396	1.63052E-33
SCN2B	1.286297808	8.349879992	2.23165E-33
FES	-0.771121807	7.381166846	2.72645E-33
DYRK1B	0.602407455	6.888180985	3.61924E-33
ZDHHC9	-1.006841473	7.540044801	8.49788E-33
GALK1	-0.744362326	8.947532995	8.62259E-33
PROS1	1.472143696	11.42441666	9.14043E-33
CHN1	0.643360271	7.924035647	9.54348E-33
NFKBIZ	-0.920983962	7.583803482	1.05989E-32
C7ORF41	1.305974386	10.41049993	1.10903E-32
TIPARP	-1.302047694	8.875520985	1.40521E-32
SLCO4A1	-1.001282684	6.802616432	1.90852E-32
LOC653888	-1.207531946	7.399348544	2.4529E-32
CORO1A	-0.791800135	6.693470252	3.27835E-32
ALOX5	-0.898132552	6.986170737	5.95125E-32
CTSC	-1.248884441	6.80407804	9.48529E-32
SORBS2	0.826650963	11.54301883	9.58156E-32
CD97	-0.794429808	8.295105548	1.24872E-31

XLKD1	-1.160006048	6.745885563	1.32389E-31
STAT3	-1.118871766	9.195537113	4.51318E-31
SEC14L1	-0.524071749	7.205474165	6.18392E-31
MT1A	-1.549496452	10.84912039	7.32315E-31
PIM1	-1.363769495	7.407739261	7.91323E-31
ARPC1B	-0.965696735	6.950221746	1.30937E-30
IFITM2	-1.208189748	11.83859754	1.95083E-30
HCK	-0.637205809	6.639667803	2.02825E-30
EBI2	-0.586869459	6.386732572	2.26629E-30
WAS	-0.93873277	7.182494252	2.59955E-30
ITGB2	-1.413178278	8.807851365	3.44755E-30
SMOC2	1.487051563	9.433749692	3.96851E-30
F13A1	-1.578700179	7.405951536	5.17769E-30
CD248	-0.983489191	8.208457754	5.46332E-30
RANGAP1	-0.941230803	8.828720144	5.82774E-30
LCP1	-1.410086347	8.333819562	7.22172E-30
IFITM3	-1.186065314	11.67757043	7.44107E-30
SMYD2	0.860853624	10.36040251	9.93609E-30
HCLS1	-1.116174517	8.265719717	1.12376E-29
SLCO2B1	-0.911506246	8.130770898	1.37913E-29
IL18R1	-0.790626412	6.568124843	1.37933E-29
BTG2	-1.520996291	8.574959065	1.67594E-29
CHRDL2	-1.171336546	6.374890146	2.00064E-29
MGST1	-0.680607023	6.40046017	2.35837E-29
C10ORF71	0.853147399	7.263092124	3.51359E-29
IRX6	1.384343762	8.771585266	3.89684E-29
INPP1	-0.695696565	8.759646446	4.59567E-29
IL13RA1	-0.833352749	8.959267621	5.40967E-29
MGC42367	-1.024647194	7.761970783	5.60507E-29
SAP18	0.634844476	8.834469954	5.67646E-29
CHST7	-1.071284973	8.898629868	6.24156E-29
MFSD10	-0.662730927	9.599808274	7.27124E-29
BCL6	-0.932343623	8.796081776	8.91972E-29
LMOD2	1.284989823	9.4494294	1.90957E-28
NFKBIA	-1.072534097	10.53437933	1.9649E-28
TUBB2C	-0.961446534	10.4743106	2.91559E-28
LOC653879	-1.133854342	7.076305456	3.10481E-28
MNS1	0.471203766	6.849638238	4.17063E-28
C1QC	-1.330656624	8.210266395	4.24884E-28
SLC15A3	-0.894326938	8.294544527	4.36807E-28
MT2A	-1.746983526	10.47381887	4.66428E-28
NNMT	-0.907491226	7.974207221	4.92524E-28
CYBA	-0.983796872	9.136413151	5.10426E-28
C1ORF162	-1.042522118	8.283033764	6.5787E-28
C1QB	-1.468626129	8.319736068	6.6257E-28
RHBDF2	-0.58288311	6.988903593	6.72398E-28
DOCK2	-0.869684446	7.221842642	6.86734E-28
LAPTM5	-1.130703788	7.385334012	6.89481E-28
ZNF415	0.589720867	8.423383405	7.02878E-28
ZGPAT	-0.417441877	7.157060395	8.74824E-28
HEG1	0.674588054	9.036105752	9.49413E-28

LRRC8A	-0.961047305	7.618568549	9.87789E-28
CEBPB	-1.012413536	11.24876265	1.02412E-27
CD14	-1.393619597	9.923432956	1.31884E-27
C5ORF4	0.567046604	6.955149441	1.54607E-27
FGD2	-0.705767428	6.739171033	1.61004E-27
CD68	-0.938087946	8.50494957	1.70772E-27
MBP	0.962895477	8.87691728	2.1029E-27
ZNF627	0.470003847	7.844395869	2.1911E-27
S100A10	-1.035596719	11.42912271	2.30731E-27
MASP1	0.825194106	9.912881394	2.34796E-27
TKT	-1.039250448	7.909001107	2.50017E-27
VAMP8	-0.932000332	8.669879639	2.5604E-27
TSPYL2	-0.687601527	7.035684514	2.68272E-27
TDRD9	-0.72255815	6.723318543	2.95006E-27
SRGN	-1.206380635	8.949063704	3.09603E-27
ALOX5AP	-1.385100966	8.200514581	3.30815E-27
FRZB	1.399855478	8.986840756	3.3142E-27
IVNS1ABP	0.783412027	8.824739812	3.59153E-27
LILRA5	-0.770152322	6.376438545	3.74536E-27
C14ORF132	1.303669979	7.776039798	4.30289E-27
FAM110B	0.768827203	8.683677421	4.39978E-27
SLC12A9	-0.60054858	7.820734667	4.588E-27
HAVCR2	-1.051982114	7.765469828	4.90016E-27
MTCH1	-0.505081482	12.0517783	5.06177E-27
GPR56	-0.884996235	8.183013453	5.18907E-27
CCND2	0.697046073	12.37862069	5.99107E-27
ARHGAP4	-0.555250377	7.152700881	7.43232E-27
CAPN1	-0.63387474	9.6856311	7.87998E-27
MVP	-0.809736216	7.869034202	8.25956E-27
LYPLAL1	0.803544412	9.815284858	9.16409E-27
OSR1	-1.053812231	6.965551149	9.68795E-27
CD209	-0.989748877	6.857377991	1.1776E-26
SYF2	0.499227447	9.405887961	1.24378E-26
CYP2J2	0.924832746	12.34107539	1.36096E-26
IFI30	-0.797417176	6.514332373	1.44544E-26
ACE2	1.82110406	9.10628885	1.8575E-26
SORBS1	0.786983887	10.04951485	1.93456E-26
DDIT3	0.697526189	8.101093941	1.9389E-26
LILRB5	-0.555867219	6.622068527	1.96233E-26
ATPIF1	0.790723056	10.46682943	2.03038E-26
PRICKLE1	1.034180661	9.627772368	2.10037E-26
HES6	-0.7542047	7.001334758	2.1183E-26
SEMA4B	-0.677630682	7.244327872	2.40908E-26
RASSF1	-0.542187956	6.825196403	2.59222E-26
M6PRBP1	-1.130238422	9.645433947	2.62608E-26
C8ORF47	0.607278657	6.839954068	2.9408E-26
NKD2	-0.805368887	6.946162309	3.09309E-26
GPR172A	-0.757787242	7.572592125	3.10746E-26
KIAA1853	0.781712947	6.910965551	3.13663E-26
OSMR	-0.620299918	6.259879896	3.41836E-26
PUS1	-0.577005826	7.262991181	3.80964E-26

ZNF187	0.350627277	7.204474357	6.45774E-26
TCEAL8	0.455277927	9.386098843	6.64019E-26
TUBB	-0.831171496	9.593267409	6.64997E-26
INTU	0.468743219	7.000295224	1.06842E-25
GNA15	-0.647094662	6.530537041	1.07714E-25
ANKMY2	0.730871513	9.406718349	1.08035E-25
SELS	-0.730888293	9.873210322	1.17429E-25
SDSL	0.777364068	8.616526934	1.18556E-25
UCHL5IP	-0.409211141	7.839014021	1.20945E-25
GATA2	-0.490551869	6.585213111	1.49355E-25
HMGN2	0.595019783	9.43373208	1.52523E-25
VPS41	0.412825558	8.667257542	1.61413E-25
C5ORF23	1.236415014	8.704239079	1.82608E-25
GRASP	-0.614334688	6.772534552	2.06756E-25
YPEL1	0.606775759	7.580901137	2.09399E-25
CNDP2	-0.507135997	10.62173537	2.16028E-25
LOC148915	0.6963943	10.12284792	2.56086E-25
SVIL	0.605639124	10.7703781	2.76954E-25
MAP3K8	-0.670904136	6.817151127	3.39781E-25
MIDN	-0.969821259	8.803575999	4.37374E-25
SGK	-1.371773743	9.133653258	5.01284E-25
RASL11B	1.724164568	10.08212131	6.56625E-25
IRX2	1.003778858	7.290923729	6.79066E-25
PDE5A	0.765944838	7.586638624	1.16141E-24
MPP3	-0.813163841	7.349614494	1.23934E-24
C20ORF27	-0.479489405	7.522275042	1.34227E-24
TUBA1B	-0.697747869	13.1190396	1.3518E-24
PLCE1	1.027332419	7.895222524	1.57553E-24
CSNK1D	-0.554541951	7.768791334	1.7702E-24
IMPDH1	-0.487130218	7.218401159	1.82265E-24
VSIG4	-0.897448968	6.932149756	2.01319E-24
FCGR2A	-0.677676775	7.018134223	2.38012E-24
GFPT2	-0.941566814	6.843550946	2.40653E-24
KIAA0141	0.611702749	9.130519678	2.47449E-24
DCUN1D3	-0.6332991	7.721418571	2.50343E-24
DDIT4	-1.316253099	9.361341096	2.5407E-24
LOC255783	-0.65466918	9.395411481	2.61304E-24
LRRC49	1.059504238	11.22239707	2.65557E-24
SLC16A9	0.903819037	7.231852171	2.80649E-24
EGLN2	-0.400906152	9.18625526	2.88126E-24
PPARG	-0.462090459	7.06948157	3.26832E-24
PLEK	-0.781365718	7.08194882	3.55709E-24
DUSP1	-1.544017941	10.35509095	3.87523E-24
IRAK3	-0.657308689	6.711133782	4.0471E-24
ADAP2	-0.808101872	7.719722425	4.1326E-24
SLC39A1	-0.665371687	8.810862213	4.13613E-24
FGR	-0.738872676	6.706411419	4.25316E-24
PLTP	-1.036818148	8.13160404	4.30236E-24
TERF1	0.422646876	6.764317126	4.34264E-24
MARCO	-0.830394715	6.445852003	4.46545E-24
FERMT3	-0.478398667	6.720215332	4.75304E-24

SLC25A28	-0.557681881	9.769833397	6.02186E-24
C1QTNF1	-1.297475834	8.458476318	6.56044E-24
TRMT5	0.655602959	9.630876333	6.70847E-24
ARS2	-0.42929386	9.093393383	7.09412E-24
SLC7A1	-1.113469682	8.02702406	1.14379E-23
CCDC28A	0.608692733	8.756387956	1.27098E-23
RARRES1	-1.352698156	6.661289737	1.30649E-23
LOC730534	0.65265905	10.80886652	1.34476E-23
SERPINA5	-0.835768842	6.818580725	1.42139E-23
SFRP1	1.730116233	9.941031749	1.51578E-23
C21ORF34	0.678687241	8.733035219	1.56186E-23
TOMM40	-0.639266186	10.24689436	1.65996E-23
SLC47A1	0.662445153	7.558569044	1.71342E-23
SMAP2	-0.62261482	8.358573974	1.73265E-23
BCL3	-0.677738028	7.039637327	1.76766E-23
LTBR	-0.865396197	7.455398929	1.99214E-23
DUSP23	-0.675250141	9.424201164	2.26281E-23
TYROBP	-1.139684762	8.503822961	2.36623E-23
CRYM	0.99151093	8.814403132	2.38282E-23
HOPX	-1.508235272	7.061728991	2.71605E-23
DUSP26	0.718799999	10.56587005	2.97481E-23
BATF3	-0.568633925	6.474354993	3.03255E-23
PLAUR	-0.573948498	6.352908115	3.16456E-23
PDIA3P	-1.051998892	7.025640968	3.23774E-23
RGS4	1.21952652	7.669459237	3.27798E-23
MS4A7	-0.766349345	7.132862818	3.33643E-23
LOC644128	0.527163514	7.783389157	4.02371E-23
CCND1	0.813543156	11.98855885	4.04219E-23
ICK	0.453458229	6.950397801	4.33714E-23
TUBA1C	-0.978286622	11.16644877	4.87253E-23
ITGAM	-0.567811378	6.700383363	5.07241E-23
SNRPB	-0.710146623	9.277489694	5.38898E-23
DDOST	-0.860626208	9.226315635	5.69594E-23
EDNRA	0.911514912	8.69934266	5.7462E-23
TJP1	0.500510896	9.7612249	6.25064E-23
COL21A1	1.067366116	7.803279422	6.71988E-23
CAPRIN1	0.422628976	8.625869056	6.75603E-23
ECGF1	-1.276095936	8.222504989	6.9422E-23
ERRFI1	-0.773853627	8.03749842	7.51748E-23
METTL7B	-1.35576942	6.805915976	7.62163E-23
PLEKHF1	-1.143556811	8.468329286	7.89458E-23
PHLDB2	0.763003593	8.802285407	8.87815E-23
SH3BGRL3	-1.09046293	8.30054016	9.29193E-23
C19ORF10	-1.24273035	7.30400264	1.0127E-22
GLA	-0.510625611	7.908476819	1.05813E-22
MS4A6A	-1.013317788	7.849406978	1.10153E-22
FLJ20021	0.425359297	7.460420277	1.37285E-22
HRASLS	0.549011572	8.025682833	1.47357E-22
CDC42EP2	-0.574235357	7.35718097	1.51128E-22
NCF4	-0.324602641	6.413000851	1.57519E-22
EIF4G1	-0.521447319	8.953610857	1.62159E-22

SLC11A1	-0.575441244	6.211710152	1.76631E-22
FRMD5	0.843333192	8.446543812	1.77091E-22
OMA1	0.558718317	7.980936635	1.7964E-22
LOC642031	-0.690506969	8.106709744	1.85129E-22
EPB41L5	0.422791249	7.259535226	1.89049E-22
DHX37	-0.611440582	7.170513524	2.11276E-22
VPS45	0.483354557	9.367984565	2.40536E-22
KLF6	-0.848226833	8.906031643	2.78619E-22
SLC2A1	-0.769695298	8.451792362	2.80818E-22
SEC61A1	-0.676211895	10.2087779	2.95695E-22
ZNF20	0.310118111	7.070929037	3.01E-22
HLTF	0.504277155	8.030296238	3.15714E-22
SLC39A3	-0.394130384	7.8909051	3.43742E-22
ENO1	-0.822814533	11.09503374	3.5503E-22
LOC201140	-1.453454217	6.851446313	3.58562E-22
KYNU	-0.412633364	6.318266231	3.70785E-22
ATP1A2	1.273976716	9.949108075	3.71877E-22
EML3	-0.611712377	8.723004204	5.19827E-22
LOC158376	-0.534273135	6.873467382	5.62033E-22
ATP6V1G2	0.29518451	6.709886098	6.0306E-22
TEAD4	-0.855167766	6.898112366	6.3615E-22
KLHDC9	0.7688161	7.583966995	6.47125E-22
FBXO38	0.377698531	8.222887882	6.50498E-22
TBXAS1	-0.621545819	6.74652871	6.50741E-22
TMEM189	-0.465957171	7.471676425	6.79914E-22
SLC1A5	-0.518203108	6.567261318	6.87646E-22
GATM	1.185078875	8.251836408	7.0639E-22
TNFRSF1A	-0.751442563	8.720666438	7.5033E-22
SERPINH1	-1.154716849	7.854073361	7.60761E-22
ELL2	-1.127652352	7.287942019	7.6512E-22
ARPC3	-1.406895783	8.51904283	7.98037E-22
LOC387763	-1.062458753	8.120588864	9.05744E-22
COL6A2	-0.712040902	7.903847781	1.02854E-21
HYAL2	-0.59336157	7.293705121	1.03055E-21
ATP1A1	-0.912096987	10.12442544	1.06391E-21
ORC3L	0.41017041	8.347241188	1.2271E-21
GSDMD	-0.615561818	8.154640072	1.24369E-21
RASSF9	0.450344215	6.649434783	1.29625E-21
CRBN	0.526727571	9.443684586	1.3156E-21
LCN6	-0.909306775	6.678009936	1.34967E-21
CD53	-0.351805196	6.308850369	1.74858E-21
ACVR1B	-0.313831008	6.971566612	1.7626E-21
ZNF787	-0.617415712	9.420159528	1.89348E-21
ZYX	-0.81995149	8.923872746	2.27014E-21
HM13	-0.308063696	6.461046431	2.33577E-21
RGS19	-0.489530588	6.935296121	2.5536E-21
C3	-0.418116475	6.291601613	2.76799E-21
RDH10	-0.615993113	7.375351294	2.80134E-21
KLHL12	0.38806161	8.761153863	2.82563E-21
C5ORF41	0.601776628	8.470670069	2.95917E-21
FZD2	0.802476968	7.63476304	3.12571E-21

LOC729843	0.550550094	9.149389645	3.17002E-21
PARVG	-0.474134534	6.617227832	3.36154E-21
FKBP2	-0.683030888	7.223201095	3.37089E-21
CYBB	-0.769901601	7.044877476	3.40161E-21
ENDOGL1	-0.856565406	7.961245865	3.56633E-21
SLC37A4	-0.443518287	8.144205366	3.70548E-21
PTPN11	0.598306059	10.97223735	3.80219E-21
DIO2	0.90243037	8.014166304	3.90478E-21
ARHGAP24	0.553052263	7.468563248	4.12954E-21
NDUFA4L2	-0.670362421	8.807442229	4.23338E-21
ASPSCR1	-0.382109877	8.430638528	4.32362E-21
PGD	-0.47171617	8.396056419	4.47772E-21
ATP6V0C	-0.41069345	10.43362802	4.63846E-21
BEST1	-0.366440287	6.44944861	4.67153E-21
C19ORF51	0.483682897	6.779097886	4.68064E-21
MERTK	-0.503129201	7.395599607	5.22565E-21
GPR4	-0.82936927	7.07733018	5.39443E-21
PAFAH2	0.515053104	7.373984582	5.49067E-21
TMBIM1	-0.672390575	8.473453436	5.6334E-21
GRM1	0.808947767	7.130029822	6.0619E-21
CTSB	-0.574660103	10.60391429	6.19447E-21
SYNPO2L	0.897387194	13.05911338	6.49748E-21
TGFBR2	-0.536906266	9.996725322	7.11221E-21
CHSY1	-0.63798806	9.012796117	7.58323E-21
NLGN2	-0.476608762	6.943758959	7.5974E-21
HEPH	0.606329638	7.207210737	7.7503E-21
TNKS1BP1	-0.471061244	6.959802032	7.96971E-21
AGXT2L1	-1.169369052	6.85345771	8.02408E-21
ALG3	-0.44290921	7.147132736	8.11736E-21
LRRC1	0.383949591	6.828831661	8.22619E-21
NUP62CL	0.632593907	6.983759505	8.96446E-21
HTRA1	0.851145274	10.19139514	9.87036E-21
RBPMS2	-0.525136791	13.09125169	1.01207E-20
TRABD	-0.437807179	9.644511181	1.07486E-20
DCLK1	-0.424506487	6.888276979	1.15401E-20
MKNK2	-0.572107705	9.836604907	1.21332E-20
FKBP11	-0.853579611	7.395827334	1.25478E-20
COL16A1	0.835536729	8.355090188	1.28794E-20
NAP1L3	0.784069995	7.26780656	1.33693E-20
RAB28	0.517709434	8.086156915	1.37253E-20
KIAA1191	0.415095093	9.988376367	1.46795E-20
DARC	-1.013155528	7.48208902	1.54241E-20
MME	0.393519291	6.492026448	1.69623E-20
MAPK13	-0.426610369	6.318561984	1.73775E-20
C21ORF51	0.472721969	7.961192053	1.81942E-20
TMBIM6	-0.471042151	11.10156415	1.82273E-20
NBPF20	-1.056817388	7.740492235	1.93203E-20
HSPA1L	0.35950397	6.992076084	2.05841E-20
HPCAL1	-0.469564631	8.516727287	2.09858E-20
DNAJC21	0.285935387	6.995152833	2.21598E-20
JAG2	-0.468630094	7.497928386	2.4245E-20

NPL	-0.456041541	6.371082422	2.47425E-20
TSPYL3	0.339437702	6.796494315	2.50381E-20
ALS2CR13	0.503554667	7.853384791	2.5272E-20
HMGB2	0.695734754	8.69464473	2.95492E-20
LUM	1.129268262	10.27276589	2.96715E-20
ATL3	-0.528647006	6.878970303	3.05186E-20
PLA2G2A	-1.35136974	6.710933342	3.09579E-20
VWC2	0.968802057	9.198583298	3.22068E-20
RRBP1	-0.586759348	8.251882154	3.56717E-20
S100A13	0.625445662	8.777518315	3.82502E-20
HFE2	1.285898159	9.376935388	3.84222E-20
RPN1	-0.568667488	10.46584417	4.00181E-20
HGS	-0.634571583	9.548890024	4.18951E-20
ECM2	0.805650453	7.96410746	4.47529E-20
NIPA2	-0.4617481	8.188534972	4.47713E-20
EXTL1	0.71385808	7.373856125	4.55234E-20
FAM125A	-0.44749581	8.310473379	4.71457E-20
CBX7	0.720065928	9.567321263	4.78488E-20
SLC35F1	0.990000349	8.449775371	5.0886E-20
C14ORF179	0.665759857	9.239369231	5.25679E-20
BATF	-0.368947113	6.225517124	5.42589E-20
C5ORF46	0.936961438	7.971629226	5.71992E-20
KIAA0644	0.815768774	7.93214698	6.19474E-20
ENTPD6	-0.543435689	8.163743057	6.66753E-20
SNAI2	-0.581893743	7.159209233	6.76598E-20
OKL38	-0.922587577	7.658780676	7.02385E-20
APCDD1L	0.884280701	7.346388572	7.50876E-20
MEIS1	-0.500625315	7.77559716	7.59016E-20
UNC84B	-0.513784522	11.43293856	7.69141E-20
RNF149	-0.478521363	8.200219209	7.80583E-20
ZNF211	0.382506613	7.876082553	7.82509E-20
C1ORF142	0.67373745	7.857619468	8.61214E-20
HCST	-0.70634771	7.605437516	8.69581E-20
C2ORF64	0.630970646	9.223774295	8.93737E-20
LOC728473	0.778204057	9.864266715	9.25339E-20
DYNC2LI1	0.315328247	6.746771296	9.41405E-20
LOC731486	-0.349777007	6.377395909	1.06061E-19
FBP1	-0.546154439	6.325396466	1.10431E-19
RGMA	-0.768341897	8.770499366	1.14539E-19
N6AMT1	0.396731035	7.219870403	1.14935E-19
C19ORF59	-0.665850084	6.126910453	1.22757E-19
BTBD14A	-0.588953345	7.037241134	1.27717E-19
ASAH1	0.671071525	7.921382198	1.28982E-19
CSF3R	-0.537235457	6.482600019	1.31594E-19
CIDEB	-0.313487048	6.509137758	1.32924E-19
APBB1IP	-0.411287662	6.914888989	1.36481E-19
LPCAT3	-0.700616233	7.958737808	1.3762E-19
EPB41L3	-0.533040854	7.191335445	1.4224E-19
LMNB2	-0.537777414	7.705437481	1.42252E-19
HBP1	0.460077406	9.041397547	1.48164E-19
PLK2	0.605839951	6.953970854	1.5501E-19

SERPINA1	-0.483730861	6.387051166	1.56345E-19
IL18BP	-0.499132944	6.502262282	1.60785E-19
KCNK12	-0.698191549	6.271947639	1.68129E-19
KLHL34	0.861720272	7.851503201	1.71936E-19
CDR2L	-0.671644583	9.031781075	1.7492E-19
OSGIN2	0.5210365	7.7048958	1.75919E-19
STK38L	0.661257546	7.769945116	1.7787E-19
LILRB2	-0.238619557	6.220557979	1.88644E-19
SLC30A1	0.465104737	7.283390205	2.02113E-19
C21ORF63	-0.562372507	7.621109736	2.16664E-19
FBXO32	1.035651406	10.09198904	2.16772E-19
SERPINE1	-2.020118049	7.055587978	2.19675E-19
LIN9	0.752122043	8.040589079	2.49393E-19
CTPS	-0.545919342	7.04662763	2.62689E-19
PLAU	-0.727533559	7.647648917	2.64678E-19
ZMYND12	0.576286173	7.011557144	2.84584E-19
GPRASP2	0.452785864	7.764745099	2.95884E-19
HK3	-0.456941295	6.276644364	2.96269E-19
CD59	-0.762134303	8.244701974	3.15309E-19
NR2C1	0.386359077	7.605874313	3.17395E-19
ADCY3	-0.690300616	9.217262608	3.47448E-19
LDHA	-0.936015427	11.75617719	3.69021E-19
FAM107A	-0.913216949	9.948520577	3.90694E-19
PKD1L2	0.63723996	6.871830317	3.98334E-19
C1RL	-0.501304247	6.724542349	4.04785E-19
MED16	-0.454657406	8.087932943	4.1796E-19
RNASE2	-0.624561082	6.243482781	4.18168E-19
PNRC2	0.409634828	9.3809012	4.22765E-19
TICAM1	-0.373011152	7.680057413	4.23859E-19
FXR1	0.392541873	9.622587166	4.29703E-19
LNX2	0.444160094	7.719238202	4.52266E-19
C4ORF41	0.272514037	7.914818377	4.80502E-19
FKSG30	-0.913500179	8.482071091	4.88709E-19
PPP1R3C	0.868920767	11.97268637	4.88714E-19
TRAK2	0.622100346	11.33118654	4.92153E-19
MDFIC	0.679763349	7.602637947	5.24042E-19
THRA	0.39949005	7.427619707	5.24605E-19
CEL	-0.626699358	6.782093375	5.48417E-19
OBFC2A	-0.640169169	6.909985643	5.78728E-19
ZMYND19	-0.386806491	7.591408043	5.93154E-19
MTHFR	-0.599939449	7.39543948	6.00883E-19
KTELC1	-0.6111983	7.989761149	6.13458E-19
LOC285556	-1.727003546	6.788190465	6.64381E-19
ACTB	-0.871282128	11.60902774	6.71872E-19
EMILIN1	-0.619024333	7.192822514	7.27793E-19
HSPA2	1.3869027	9.078256802	7.44311E-19
AMZ2	0.465549911	9.904974966	7.47363E-19
PRKCQ	-0.776557147	8.034744324	7.56368E-19
PTBP1	-0.564157551	10.68093201	8.27359E-19
FAM102A	0.288292369	6.847258176	8.33355E-19
LGMN	-0.498767489	8.863110977	9.54242E-19

STXBP2	-0.509257959	6.28421529	9.58158E-19
AIF1	-0.669699197	7.328221559	9.66801E-19
FYCO1	0.796351867	10.7015661	9.77657E-19
ARF3	-0.440146201	7.448592955	9.98356E-19
PDK4	-1.998476938	10.21842091	1.02187E-18
TNNT1	0.925408338	8.06580085	1.02685E-18
ISG20	-1.087698086	7.504957892	1.07577E-18
FCN1	-1.281077078	7.148845468	1.12747E-18
KLF4	-0.753885263	7.035842626	1.18399E-18
GRB2	-0.345057182	7.414079702	1.21894E-18
THBS4	1.274954436	11.38449204	1.22575E-18
PHGDH	-0.727314942	8.51266329	1.2326E-18
MTE	-0.875648522	8.167733677	1.23724E-18
LILRA2	-0.474382858	6.173547602	1.30346E-18
TEAD2	-0.486919804	10.39354452	1.31947E-18
ARPC4	-0.568472594	8.098495413	1.35851E-18
RAC2	-0.689974287	7.13371563	1.40911E-18
IFI16	-0.680892959	7.850421705	1.42928E-18
SLC31A1	-0.548464739	7.418391203	1.46148E-18
SBNO2	-0.377145292	6.512097795	1.5046E-18
IGSF5	0.809052642	9.044116142	1.52687E-18
UBN1	-0.534512636	8.422004514	1.52831E-18
FAM46A	-0.538916807	10.65680994	1.54743E-18
TMEM140	0.79598168	9.184559038	1.55478E-18
APOBEC3F	0.287533072	6.909597472	1.58527E-18
LMNA	-0.512936592	9.506594952	1.67799E-18
C17ORF70	-0.519095598	8.931322987	1.71609E-18
TMEM74	0.745286571	6.725443536	1.72234E-18
ZNF385A	-0.288436765	6.323906745	1.75292E-18
ARRDC2	-0.391090331	6.534945639	1.76891E-18
SOX4	0.670785888	7.514014396	1.77701E-18
LOC654264	-0.536766316	6.262159339	1.7845E-18
GPNMB	0.935495942	10.66371931	1.79294E-18
POLR3H	-0.322963463	6.581256041	1.8134E-18
C5AR1	-0.505073827	6.48867394	1.8289E-18
EYA1	0.485170699	7.083120907	1.8315E-18
FCGR1B	-0.384912789	6.354194109	1.89561E-18
GLRB	0.61034537	7.914696417	1.9108E-18
MKL1	-0.410813113	6.831506926	1.96836E-18
C22ORF25	0.652496514	8.790438251	1.99071E-18
TPD52L1	0.779128129	8.872300613	2.00423E-18
ZNF189	-0.587134489	7.864208273	2.00668E-18
SLMAP	0.525195303	12.05883683	2.0382E-18
GRRP1	-0.559704692	7.800291321	2.10205E-18
MST150	-0.351536963	6.714122518	2.20598E-18
ZMPSTE24	-0.501087565	9.972064503	2.29359E-18
TNFRSF1B	-0.5745177	7.064466642	2.29553E-18
CD276	-0.454217251	6.76117876	2.4153E-18
CFD	-1.453993694	10.71739242	2.42204E-18
OMD	0.78724288	7.654714394	2.51101E-18
PPP1R14B	-0.853186393	9.621784995	2.53573E-18

GFOD1	-0.79021959	9.53338219	2.60014E-18
CENPA	1.699105761	7.752729554	2.69099E-18
ASCC3L1	0.373237594	7.403749334	2.72532E-18
C14ORF135	0.681137737	9.404951284	2.89428E-18
PCK2	-0.236834894	6.25762166	2.98157E-18
TSPAN32	0.765822454	8.42706855	3.04192E-18
SERPINI1	0.446867781	7.137291118	3.06194E-18
TM7SF2	1.050733175	10.01304247	3.08705E-18
TMEM14C	0.372714827	11.60536582	3.13366E-18
S100A11	-0.80914981	8.027354802	3.26357E-18
SIRT5	0.458363534	8.510552217	3.29959E-18
KIAA1429	0.339677219	7.699653565	3.35648E-18
LOC401397	0.787062505	11.60726742	3.4612E-18
LOC399942	-0.843959613	7.523422031	3.54382E-18
TMEM51	-0.476773445	8.258860026	3.83685E-18
LYN	-0.587527301	7.613209173	4.29103E-18
SGIP1	0.740798009	7.585311167	4.549E-18
SPI1	-0.343378632	6.54556995	4.75804E-18
RHOG	-0.554144393	8.350689817	4.80807E-18
RCOR3	0.500819658	9.256304111	4.90632E-18
STEAP3	-0.344361226	6.539875034	5.13188E-18
MGST3	0.462651896	13.38988442	5.18869E-18
APOLD1	-1.494669491	8.427634231	5.2058E-18
PYGL	-0.38152672	6.93626996	5.40491E-18
LOC643783	0.295957936	6.437500292	5.50027E-18
FZD1	0.51589518	7.299123716	5.69579E-18
MUC1	-0.318225667	6.319434741	5.76183E-18
HSPA4L	0.395441058	7.100730838	5.89648E-18
CCNYL1	-0.338638232	6.74207753	6.14985E-18
TMEM149	-0.49102382	6.873181864	6.15518E-18
ZNF536	0.507030681	6.923373587	6.38631E-18
S100A12	-0.613598635	6.172056021	6.4652E-18
ZNF616	0.301194713	6.808612527	6.54914E-18
ATP2A2	-0.760917615	12.41340482	6.61496E-18
C10ORF110	0.816287167	7.614752671	6.78781E-18
KCNA5	0.907252933	7.938057613	6.97048E-18
DNAJC4	0.349264018	7.099758127	7.09894E-18
C12ORF47	0.503348395	9.872704226	7.20246E-18
RGS1	-0.894146769	6.436164755	7.39257E-18
ARHGAP9	-0.409911043	6.469857076	7.46853E-18
ID3	-0.676707614	9.973402335	8.09062E-18
LOC648024	-0.829891529	7.87633999	8.15144E-18
FRMD3	0.682747021	8.686544635	8.2519E-18
IER3	1.176232337	9.223657174	8.68971E-18
C13ORF15	-0.798395499	11.05528709	8.69196E-18
EDC3	0.704947836	8.673258433	8.97481E-18
GARNL3	0.587752146	7.947466615	9.02463E-18
FAM129B	-0.516806822	8.573541765	9.0458E-18
RNASE1	-0.706189214	9.329227513	9.24406E-18
SMTNL2	-0.583227975	6.485768757	9.50439E-18
SNX13	0.462684929	7.99075626	9.86656E-18

FGD3	-0.465276903	7.074243345	9.972E-18
C1ORF105	-0.399427336	6.386479365	1.05541E-17
PDGFRB	-0.66891769	9.72524823	1.0639E-17
CD33	-0.377098094	6.292472066	1.08969E-17
SLC7A5	-0.929917676	6.979620293	1.11241E-17
FAM46B	-1.073498976	8.051599923	1.20299E-17
PUF60	-0.419130603	10.89966979	1.23521E-17
CLIC1	-0.883362519	9.716498784	1.24816E-17
FPR3	-0.497672501	6.608298151	1.36074E-17
B4GALT5	-0.533720507	9.581337671	1.37998E-17
GCA	-0.564612526	7.59768294	1.38052E-17
SOCS1	-0.630781111	6.41253047	1.45932E-17
SLC31A2	-0.506841589	7.950730072	1.46529E-17
SNF1LK	-0.994204823	6.846204153	1.46697E-17
LOC642897	0.562602208	8.094655208	1.49346E-17
KLHL13	0.42160115	7.041218572	1.55299E-17
YARS	-0.52945853	9.261331831	1.56627E-17
TMCO3	0.60465623	8.536052135	1.59056E-17
BRD8	0.302102035	7.512025613	1.60075E-17
CACNA1H	-0.413455024	7.060044022	1.66001E-17
BDNF	0.543270869	6.812731114	1.70616E-17
COMMD1	0.491027642	11.1368411	1.82308E-17
FLJ35801	-0.417800048	6.423568913	1.85466E-17
USP46	0.59919467	8.344532391	1.86609E-17
MLKL	-0.383664402	6.694300099	1.93319E-17
PHF3	0.470368931	8.903001549	1.97616E-17
TMEM56	0.633408587	7.695508394	2.03507E-17
ATPBD4	0.343288602	7.130626786	2.05629E-17
LHFPL2	-0.654792497	7.637478516	2.06946E-17
TRIM39	0.238576841	7.332363594	2.09758E-17
DRAM	-0.433627785	7.234390787	2.15667E-17
MYADM	-0.576553209	9.802801934	2.15916E-17
LILRA3	-0.357624159	6.161228139	2.19086E-17
XPO4	0.77295963	9.988280595	2.24445E-17
TTYH3	-0.404492853	6.636528712	2.30517E-17
CCL2	-1.436967943	8.394104274	2.31723E-17
PTCD2	0.410371548	7.504266989	2.33654E-17
GPBAR1	-0.404934751	6.55403612	2.56154E-17
RNF20	0.55299308	9.730055893	2.66906E-17
UNQ2541	-0.42280156	6.145817989	2.73442E-17
ZNF30	0.385442163	7.409824719	2.7546E-17
ARPC5L	-0.608634664	9.187141238	2.77444E-17
ADFP	-1.158514398	9.550061749	2.80172E-17
HRK	0.548790566	6.486890906	2.83017E-17
SOX18	-0.871028238	10.652518	2.86689E-17
CXCR7	0.820615502	9.899600272	2.89857E-17
IL15RA	-0.207496146	6.184521417	2.93843E-17
ITPR3	-0.635472617	9.204273678	2.96264E-17
MAGOH	-0.581521127	7.355142734	2.98087E-17
NEDD9	-0.552408047	6.975573223	3.01603E-17
FLJ23584	0.407092178	7.069080203	3.11555E-17

KIAA1303	0.24294168	6.492138726	3.16179E-17
UPF3B	0.323076276	7.522501415	3.19606E-17
FLJ27354	0.488020438	7.254795776	3.20127E-17
ZNF16	0.245719652	6.87448468	3.28784E-17
PYCARD	-0.423258023	7.03471858	3.40617E-17
GADD45G	-1.231088708	9.573987199	3.46239E-17
LRP5	0.41330811	8.064765018	3.47132E-17
BBS4	0.472789229	8.184333234	3.59633E-17
ST8SIA5	0.711023904	7.795842141	3.69578E-17
CSK	-0.515290513	8.188939891	3.70717E-17
ZNF502	0.229356497	6.76750764	3.8807E-17
EIF4ENIF1	0.324261673	8.345234488	3.90168E-17
PTHR1	-0.610088272	8.167869394	3.9055E-17
TLL2	0.598733568	6.891761471	3.91833E-17
TAF15	1.020381594	9.63275622	4.00383E-17
C6ORF111	0.487045573	8.660147725	4.01627E-17
FURIN	-0.459437601	7.052534819	4.09552E-17
POLR1E	0.527164039	8.352488794	4.12229E-17
SUSD1	-0.457268091	7.023932184	4.16374E-17
NT5DC3	-0.511097983	7.171617144	4.29299E-17
NBPF10	-0.682541661	6.839014909	4.33329E-17
TUBG1	-0.421980371	8.200730962	4.34022E-17
PAFAH1B3	-0.424642098	6.703313307	4.53643E-17
ARHGDIA	-0.621536556	8.838313148	4.54654E-17
LY96	-0.912912928	8.134008324	4.8203E-17
LMCD1	-1.431687064	9.35588141	4.83181E-17
SPTBN1	0.520929831	9.944769561	4.95123E-17
PROM1	1.185860835	8.682228608	4.98042E-17
FADD	-0.413299629	8.099238089	5.3427E-17
ZNF671	0.373200532	7.907548301	5.44327E-17
LOC374395	-0.406121138	10.0712225	5.6126E-17
C14ORF28	0.555356791	7.819144365	5.65595E-17
ETS2	-0.573209584	8.53930326	5.68086E-17
LARP7	0.416322119	9.15077002	5.7428E-17
NINJ2	-0.594077135	7.672593466	5.88002E-17
FGF1	0.347090661	6.724967638	5.92825E-17
C9ORF24	0.593025277	8.444622334	5.94177E-17
ADAMTS9	-0.977980024	7.791870076	6.02784E-17
LOC647000	-0.586348594	10.745267	6.15462E-17
CCR1	-0.329142898	6.255820054	6.16711E-17
MORN4	0.396939552	6.793972459	6.17431E-17
HMOX1	-0.495647314	6.37284608	6.39291E-17
LRSAM1	-0.406042617	7.679381236	6.93534E-17
CXCL2	-0.830697034	6.733744309	7.48665E-17
GLIPR1	-0.507212952	6.617044467	7.71351E-17
MFN2	0.594411067	11.38076346	7.76803E-17
CCND3	-0.448104143	10.75394143	8.25893E-17
RBM20	0.823559513	11.21083464	8.32432E-17
NLRP3	-0.252057618	6.254072254	8.41422E-17
MOAP1	0.575112695	9.534782456	8.49409E-17
SKP1A	0.347835901	9.119569927	9.72498E-17

UBE2J1	-0.557659858	7.852577044	1.00905E-16
COL4A5	0.552630416	7.661007126	1.08522E-16
BTK	-0.452004357	6.314905838	1.08918E-16
SLC7A7	-0.590315739	7.222942328	1.12005E-16
CX3CR1	0.487440568	6.835194018	1.12629E-16
WARS	-0.711361248	9.655084481	1.13941E-16
SPOCK1	-0.983287601	8.254305933	1.18556E-16
CAPN3	0.437275576	6.778481638	1.20565E-16
TSC1	0.499058449	8.466388694	1.24167E-16
SLC2A6	-0.433750952	6.994345402	1.26503E-16
C3AR1	-0.379341799	6.330710335	1.37152E-16
KIAA1468	0.252918377	7.417021123	1.45712E-16
PPM1G	-0.432347229	9.261876275	1.57746E-16
PDGFRA	-0.689360336	8.410832716	1.61927E-16
MYD88	-0.349683652	6.781480844	1.67995E-16
PPCS	0.40772888	9.602271881	1.69188E-16
TIMP4	-0.854768313	7.770136009	1.71591E-16
NOL6	-0.419823428	7.855588232	1.71902E-16
PMM1	0.337200137	9.18761892	1.72221E-16
C5ORF37	0.268731682	7.179430887	1.73962E-16
TUBA1A	-0.615394793	11.06033385	1.74026E-16
NMNAT3	0.416206627	7.759932237	1.79087E-16
MYCN	0.336300791	6.360801185	1.79362E-16
ZNF277	0.502502606	8.516665013	1.8586E-16
SNCA	1.012257804	8.096976655	1.89594E-16
SFRP4	0.947146334	6.987260789	1.90165E-16
LANCL1	0.413923494	10.1017887	1.98047E-16
PRDX6	-0.455173553	11.93640386	2.04158E-16
AQP9	-0.6591233	6.541413637	2.07735E-16
UNC45B	0.408243645	7.188125149	2.10171E-16
PLSCR3	-0.520299358	10.35556757	2.11958E-16
NOL1	-0.465319167	7.440569193	2.22532E-16
FCGBP	-0.818559275	6.706677405	2.23141E-16
GAB1	0.339693408	6.790331386	2.23985E-16
ZFP90	0.380953402	8.374795622	2.25739E-16
LRCH4	-0.452119108	6.952891225	2.28391E-16
P2RY11	-0.345660272	6.715253112	2.28916E-16
IL4R	-0.349031856	6.549022994	2.29109E-16
MAP2K1	-0.757100528	9.148082085	2.34017E-16
CPT1B	0.75426414	9.422929995	2.36961E-16
GABRB1	0.821748304	7.73275612	2.41612E-16
TGFBR3	-0.645174858	8.847316943	2.48466E-16
RDH5	-0.862937151	8.273152798	2.52392E-16
PNPLA4	0.408769929	7.009180901	2.55403E-16
MLLT11	1.084993822	11.00966006	2.55999E-16
GRK4	0.34579148	6.854785462	2.65919E-16
HLF	0.655439023	7.786693779	2.72693E-16
TMEM42	0.48794428	8.710357933	2.79489E-16
ELL	-0.294057964	7.246604345	2.82198E-16
EIF4A1	-0.732779329	11.61029812	2.84224E-16
LFNG	0.557299214	7.206335626	2.9355E-16

SLC44A3	-0.432031245	6.693232632	2.99515E-16
MMP9	-0.438864948	6.111823159	3.10972E-16
DCUN1D2	0.597448274	7.861901887	3.11012E-16
KIAA1370	0.523200231	8.175566096	3.11148E-16
PLA2G15	0.54685491	8.260128389	3.19275E-16
CCDC109B	-0.825030125	7.999999341	3.25616E-16
REXO1	-0.318687981	7.246160779	3.41884E-16
NPTX2	-1.049951791	6.754437362	3.46564E-16
PTPRM	0.467490734	10.33253806	3.53944E-16
CLIC6	1.355220831	9.624450586	3.59014E-16
SAMSN1	-0.222151215	6.144566842	3.60594E-16
CTF1	0.357713735	7.036617048	3.69614E-16
ZNF564	0.319920101	7.792321137	3.7469E-16
MDP-1	0.349083607	7.959745915	3.76385E-16
FLJ21986	0.616904211	9.008624956	3.77434E-16
PVR	-0.754558275	7.132487111	3.78518E-16
TAP1	-0.712357831	9.181414069	3.79482E-16
GAK	-0.347473795	7.701124146	4.01008E-16
C10ORF97	0.389173236	8.769709211	4.02001E-16
CD300C	-0.257524958	6.140951916	4.02424E-16
ASB3	0.224447318	7.434967504	4.08774E-16
PSMB8	-0.39198003	7.218708187	4.10408E-16
SLC16A3	-0.804721344	7.644357259	4.17216E-16
YTHDC1	0.326481851	9.338327902	4.17292E-16
MTHFD2	-0.573860838	7.793608296	4.19823E-16
VARS	-0.335167826	6.997261015	4.20852E-16
UCRC	0.379868673	9.240478549	4.2932E-16
TMEM71	0.974712916	9.442847766	4.32305E-16
FAM89A	-0.356289208	7.343679942	4.39444E-16
CDKL3	0.239484534	6.918735377	4.58546E-16
LRRC32	-0.666837333	9.03272418	4.61923E-16
CCDC41	0.504485305	7.520626463	4.68097E-16
AKAP8	-0.340759217	7.828723923	4.75952E-16
ZNHIT3	0.321558459	9.11940212	4.84048E-16
PDE4D	-0.755201743	7.962111688	4.99121E-16
CD86	-0.243930249	6.466879358	5.0307E-16
FASN	-0.738146786	8.415772863	5.03928E-16
SYK	-0.444065078	6.711685912	5.08066E-16
DCLK2	0.52282381	7.408523169	5.08496E-16
MYH7B	0.89303973	9.232718383	5.34914E-16
STEAP4	-0.415219584	6.197529607	5.37596E-16
MS4A4A	-0.335657445	6.454623624	5.44965E-16
SH2D3C	-0.329551333	7.281077354	5.72338E-16
PDZD2	0.501230749	8.754140365	5.84517E-16
CRSP2	0.256618971	6.771647892	5.93597E-16
C11ORF74	0.476464608	9.77861106	6.10371E-16
SPPL2A	-0.642187703	9.049958313	6.17312E-16
CHMP5	0.501997013	9.162648246	6.34521E-16
TPD52	0.198839746	6.549648873	6.35422E-16
ABL1	-0.312161869	7.688130068	6.42732E-16
ANKRA2	0.443914922	8.124001489	6.50476E-16

RCN1	-0.537839941	8.789050397	6.57905E-16
CDKN2D	-0.341482807	6.837733329	6.65674E-16
DNAJC15	0.51767096	9.360619553	6.83359E-16
U2AF2	-0.373980633	8.661571814	6.9937E-16
DPY19L2	0.679470301	8.619515521	7.12609E-16
CLDND2	0.516007147	7.797247313	7.15002E-16
NAMPT	-0.877879963	7.459401123	7.23214E-16
ZNF664	-0.572940143	6.618458525	7.41402E-16
RRAGB	0.331732357	7.645314631	7.49414E-16
CCDC121	0.292875874	6.826026635	7.57596E-16
SEC23B	-0.422154144	7.528931562	7.74971E-16
TMUB1	-0.485933401	8.241022834	7.75095E-16
PDE7A	-0.585833732	7.697242169	7.77218E-16
FMO4	0.512487751	7.553734199	7.77824E-16
PVRL2	-0.360304613	7.361968869	7.82456E-16
RNASE6	-0.501397516	6.642434524	7.8712E-16
C14ORF147	-0.364654144	8.175863424	8.05281E-16
CAP1	-0.607724767	10.0473434	8.44231E-16
NXT1	-0.49393077	8.487506522	8.6408E-16
OXCT1	0.639192833	9.786647706	8.8427E-16
FAM164A	0.380296677	7.264015728	9.04006E-16
H2AFY	-0.315741917	9.867806503	9.40194E-16
SLC25A22	-0.401048102	6.700639786	9.92863E-16
PAAF1	0.367857468	8.120100382	1.00645E-15
TMED9	-0.684590619	9.483598994	1.01107E-15
IGFBP4	-0.778915473	9.229406183	1.02532E-15
CTSF	0.583216376	8.540245948	1.0391E-15
EMP3	-0.662175818	9.685941686	1.06727E-15
TRIM8	-0.417836815	10.29540838	1.0832E-15
ATP1B2	0.408095745	7.33540694	1.09282E-15
RCC2	-0.535551971	8.978650105	1.11114E-15
RAMP1	-0.756338533	9.708930958	1.12127E-15
TYRP1	0.90626692	9.519295848	1.12173E-15
DUSP28	0.614030668	9.367032726	1.12978E-15
SMARCD2	-0.411410717	6.77788788	1.13804E-15
RIN2	0.420166811	9.42897373	1.1597E-15
ZBTB4	0.491514634	10.10161363	1.16696E-15
H2AFZ	-0.535783793	10.66411576	1.21703E-15
GPSM2	-0.529083998	6.363509366	1.23791E-15
SLC2A12	0.778882051	8.721583289	1.24542E-15
C6ORF130	0.34354344	8.622363166	1.25904E-15
C7ORF44	0.365318803	10.53015053	1.26106E-15
LOC650832	-0.893221492	9.082089421	1.28226E-15
TFDP2	0.369563026	7.45325574	1.30883E-15
MDK	0.29748827	6.723427762	1.31167E-15
LOC606724	-0.269054721	6.229508404	1.32334E-15
GMPR2	0.269125873	8.43539092	1.33512E-15
NPR3	0.719636172	7.260156062	1.33774E-15
KIAA1394	0.611448264	7.569108955	1.34492E-15
APBB3	0.487974295	8.610218675	1.36804E-15
SKAP2	-0.294295908	6.509622479	1.39057E-15

MAP2K5	0.339918263	7.316607223	1.42757E-15
HLA-DRB4	-0.839372003	8.562945259	1.44105E-15
BAIAP2L1	-0.271993049	6.433846867	1.50237E-15
FTL	-0.569764551	12.54645628	1.50353E-15
WDR48	0.413157823	8.383211409	1.50673E-15
PTP4A2	-0.408788903	9.13938087	1.59025E-15
GYS1	0.502483893	9.089739594	1.59162E-15
PPRC1	-0.471965713	7.910943404	1.64168E-15
ESRRB	0.325641238	6.668755001	1.66951E-15
MUM1L1	0.695280801	7.514298518	1.69948E-15
MTP18	-0.731276068	8.038412783	1.71105E-15
TMEM16A	0.570305137	8.034467854	1.76069E-15
PSTPIP2	-0.315445448	6.537662466	1.76447E-15
LOC730525	-0.642857892	9.446514397	1.87813E-15
HNRNPAB	-0.44832728	10.28232298	1.92306E-15
S100A3	-0.656185898	6.515507866	2.01534E-15
KLHL3	-0.733718447	9.510377973	2.04529E-15
RAB3IL1	-0.5116838	8.399259066	2.08563E-15
FAIM2	-0.46043403	6.392373518	2.10632E-15
DPH2	-0.468263404	7.667373983	2.10705E-15
POP5	0.403006664	7.980228442	2.12622E-15
PPWD1	0.296261614	7.593880297	2.17329E-15
SSPN	0.635788486	9.268240258	2.24211E-15
BOP1	-0.49108525	9.543896165	2.2635E-15
ZDHHC18	-0.338464541	6.729527731	2.40744E-15
RASSF2	-0.416600868	6.772575895	2.44854E-15
VPS8	0.500366892	8.501988664	2.49583E-15
SLCO3A1	-0.33392481	7.533603296	2.49606E-15
KIAA1949	-0.502484666	7.767724541	2.5047E-15
TP53INP1	0.540428964	7.767595157	2.52036E-15
FNBP4	0.582774908	10.20066046	2.62116E-15
C17ORF58	-0.355595772	7.589838976	2.66404E-15
POR	-0.554259952	7.485951782	2.72915E-15
PRMT2	0.421427328	8.363918442	2.75885E-15
SLC38A3	0.266186908	6.278550416	2.77633E-15
ST8SIA2	0.747469569	7.507589219	2.80382E-15
ZEB1	0.475366016	7.264958035	2.89805E-15
C12ORF44	-0.47804904	8.43217818	2.94384E-15
ZBTB25	0.288265426	6.787588427	3.01023E-15
ACYP2	0.533768824	9.309040034	3.06233E-15
LETMD1	0.355229302	7.556186209	3.12644E-15
STAB1	-0.569278733	7.596117275	3.26473E-15
ZNF17	0.319534805	8.017816062	3.33666E-15
SLC36A4	-0.464486291	7.023070879	3.38642E-15
MPHOSPH8	0.375751378	8.456544889	3.40742E-15
LOC723972	0.600642428	7.926472175	3.42998E-15
MYOM1	0.537880966	13.99956863	3.44538E-15
TESK1	-0.425297419	8.078832019	3.60903E-15
AGTR1	-0.317441857	6.371687991	3.76533E-15
CFL1	-0.496449793	11.09790102	3.77097E-15
LOC647346	0.646528388	8.63615768	3.81776E-15

ZMAT1	0.155077596	6.182963369	3.87818E-15
RAB7B	0.344651269	6.563104617	3.93021E-15
KCNJ2	0.655152139	9.065962003	4.04362E-15
NSL1	0.263475043	7.695319719	4.0917E-15
ICAM2	-0.545535968	9.819824135	4.20235E-15
PLAG1	0.232503235	6.682414678	4.24517E-15
CAV3	0.473138886	7.391689692	4.32989E-15
EIF2B4	-0.325160054	9.49047814	4.37667E-15
XPC	0.39201578	8.358883614	4.46886E-15
DPM1	0.397944058	10.10958607	4.54552E-15
DYRK4	0.32175251	8.70117578	4.58228E-15
CRLF3	-0.246028182	6.852422828	4.62368E-15
GALNT17	0.471821755	7.092180001	4.71929E-15
OCIAD2	-0.457044957	8.305168984	4.90293E-15
MFSD1	-0.404396883	9.194672527	4.94485E-15
OGFR	-0.324698098	7.344659692	4.96804E-15
LOC441019	-0.370072732	6.423188999	5.14781E-15
LEFTY2	0.546687367	6.841402399	5.15726E-15
TRIB1	-1.019853839	9.491518728	5.26078E-15
PLA2G4C	0.628800218	10.11825753	5.26339E-15
BAX	-0.317240899	6.627445807	5.5493E-15
GGT5	-0.369182853	6.533711934	5.85588E-15
FLAD1	-0.243177629	8.069622889	5.88909E-15
SLC40A1	0.48072056	8.240389262	5.91874E-15
BLM	-0.485515974	6.573034362	5.94499E-15
GATAD2A	-0.546083794	8.659548604	5.97711E-15
FRMD4A	-0.519848918	8.165099401	6.18069E-15
CLEC10A	-0.218047902	6.273404496	6.37117E-15
LOC643888	0.341212714	6.987421449	6.41641E-15
EFR3A	0.373718466	10.11314701	6.49127E-15
RWDD3	0.386495697	7.328798363	6.50039E-15
SCRN1	0.642066573	8.694226959	6.52925E-15
ST3GAL1	-0.469724271	9.089977672	6.57203E-15
KIAA0427	-0.573183337	8.32181886	6.58897E-15
ZCCHC5	0.219115238	6.217909938	6.6061E-15
IK	0.358412342	9.22297254	6.7091E-15
RNF145	-0.403866641	7.613259785	7.16375E-15
SOCS3	-0.358688541	6.216214423	7.52118E-15
FLJ20273	-0.272590195	6.091013531	7.54844E-15
PRR16	0.51664988	8.005867122	7.54895E-15
LLGL1	0.445096387	8.266125531	7.58561E-15
ITGB4BP	-0.623047999	8.975047691	7.67559E-15
LDLR	-1.006436628	8.691374264	8.31086E-15
TCTEX1D2	0.488563059	7.931333972	8.53573E-15
SDCCAG1	0.364122857	8.002802593	8.98216E-15
SHISA2	0.442202074	6.417426955	9.07741E-15
ZBED4	-0.293229126	6.839757154	9.1769E-15
KIAA1217	0.373197979	7.90476543	9.17707E-15
FMOD	1.091335288	8.009102118	9.69344E-15
M160	-0.38637614	6.628836974	1.00977E-14
STAMBPL1	0.432473564	7.703254767	1.03334E-14

SCO2	-0.439167187	8.855239757	1.04826E-14
ZFP82	0.352519529	7.489827442	1.08144E-14
C14ORF106	0.367533016	7.877547555	1.10541E-14
ZNF404	0.352762984	7.004784051	1.11838E-14
TAF1	0.27310669	6.662536474	1.12166E-14
MTRF1L	-0.249628048	8.913911169	1.15736E-14
RCBTB2	0.285626867	7.363725144	1.16477E-14
MATN2	0.739024682	9.03653346	1.16705E-14
SLC25A27	0.391308712	7.223725901	1.20864E-14
ZRANB2	0.34083484	9.323529294	1.27072E-14
SAT1	-0.853226493	10.98271084	1.27277E-14
DOPEY1	0.35848237	7.216769146	1.29448E-14
MAP6	0.179883604	6.393600507	1.32412E-14
ZNF615	0.397577601	8.355885769	1.33302E-14
ISCU	0.539235965	12.03745565	1.33845E-14
GMPPB	-0.309354476	6.926590061	1.3548E-14
SLC24A6	-0.384798835	7.846286303	1.35556E-14
LMTK2	0.358042246	6.966098566	1.38612E-14
CLEC4G	-0.359633934	6.219849593	1.39696E-14
SERTAD1	-0.77807523	8.628465717	1.39737E-14
TUBB4Q	-0.606713667	7.701653721	1.40102E-14
TMEM126B	0.341644283	10.24462792	1.46857E-14
C9ORF125	0.377286632	7.643794548	1.48446E-14
CCDC25	0.30436323	8.931591041	1.50807E-14
MPDU1	-0.37765723	7.053943154	1.51988E-14
LRRC39	0.882307466	8.988726398	1.52253E-14
TBC1D16	-0.341533046	7.339011716	1.52381E-14
SH3GL1	-0.304605233	6.98836941	1.55567E-14
ISLR	0.331548368	6.584214056	1.55858E-14
SLITRK4	1.260442169	7.741740727	1.60076E-14
E2F4	-0.389701735	8.483122751	1.6133E-14
SCUBE2	0.509327601	6.607964521	1.70803E-14
RP9	0.345329739	9.47803074	1.74719E-14
LOC730063	0.364431488	7.300454207	1.80204E-14
RAB8A	-0.406981013	8.321658705	1.847E-14
PRRX1	0.633854147	9.409699187	1.86057E-14
BRMS1	-0.464509276	8.600451817	1.86272E-14
FST	-0.314394788	7.357340626	1.8635E-14
SPP1	-1.116975344	7.098378908	1.88002E-14
CDKN2AIP	0.302344995	7.418958791	1.91716E-14
SEPSECS	0.219174687	6.686248581	1.91985E-14
TMSB10	-0.550884186	12.40921712	1.9822E-14
GLDN	0.741650053	8.088220807	2.02735E-14
ATPAF1	0.456930768	10.53708311	2.04855E-14
CATSPER2P1	0.231395913	6.686143256	2.05791E-14
TRIM41	0.383193129	7.615245921	2.06314E-14
PSPH	0.238799442	6.804722621	2.10722E-14
APOA1	1.584427169	9.75381417	2.17025E-14
BBS2	0.47474764	9.042648681	2.23657E-14
PITPNM1	-0.471845565	8.738606106	2.24759E-14
LOC402221	-0.662496212	7.391900371	2.25786E-14

ZNF816A	0.314070837	7.21550526	2.32972E-14
C17ORF62	-0.39575335	7.612778119	2.4668E-14
COL7A1	0.585047803	8.543720847	2.47703E-14
SLC11A2	-0.391944501	8.669473514	2.47877E-14
SLC3A2	-0.332711029	7.170048534	2.48358E-14
FAM65A	-0.381315997	10.06433984	2.50548E-14
C1ORF24	0.583514608	12.53253942	2.5145E-14
CYP4B1	-0.621546372	6.566040756	2.60197E-14
IHPK3	-0.679875839	7.751782136	2.67202E-14
FAM98C	0.309010244	7.036722345	2.72913E-14
HCFC1	-0.332505145	9.82235437	2.73814E-14
WDR1	-0.437372463	10.27847961	2.7582E-14
ZNF433	0.320116807	7.116713256	2.85196E-14
PFN1	-0.430217935	11.58782453	2.90487E-14
NDP	0.498104882	6.734385066	2.94038E-14
TMEM2	-0.497361569	6.997731689	3.09826E-14
LGTN	0.318823062	8.226057119	3.17535E-14
TNFRSF14	-0.484006821	9.405992942	3.18458E-14
MYOZ3	0.767026291	8.401723576	3.2027E-14
BIRC3	-0.471273405	8.4704322	3.26115E-14
ALDH5A1	0.651831909	8.407852616	3.34318E-14
LAT2	-0.195509256	6.198100956	3.41562E-14
BCAT1	-0.466876285	6.353946569	3.42132E-14
MAP2K3	-0.502275735	6.893563625	3.51344E-14
KDELR2	-0.522264954	8.675093313	3.62717E-14
ALDH4A1	0.389323877	7.76776592	3.70142E-14
TGIF1	-0.303196758	6.5066062	3.71571E-14
IFNGR1	-0.469346764	10.52171531	3.73665E-14
ADD3	0.433725779	8.653261652	3.80829E-14
GADD45A	0.746804545	10.44911309	3.81801E-14
SGCG	0.787795206	10.07206295	3.89632E-14
GJA1	0.748921889	10.8577887	3.9556E-14
RAB5A	-0.516719958	8.220439506	4.08703E-14
DPH5	0.300642844	8.684277851	4.10064E-14
PEX1	0.387467156	7.454270235	4.11472E-14
SERTAD3	-0.260819921	7.003554691	4.22321E-14
WWC1	-0.722668501	6.197381729	4.26736E-14
THAP6	0.339148052	6.992140045	4.33145E-14
B3GNT8	0.339350875	7.061189069	4.39387E-14
ARHGEF19	-0.286141612	6.583289439	4.53914E-14
STS-1	-1.040639768	6.886805842	4.5743E-14
ZNF248	0.390866369	7.168421687	4.62357E-14
ARMC4	0.308531565	6.310977639	4.64204E-14
TMEM43	-0.582179881	9.732461656	4.64711E-14
ADAL	0.389393663	7.34237258	4.74771E-14
MYOZ2	0.715833163	10.51331378	4.78758E-14
MT1G	-0.681174086	6.767657698	4.9471E-14
IL17D	0.590950398	7.768033159	5.0677E-14
RGS2	-0.804874888	9.221843188	5.07098E-14
LOC492311	0.479072242	8.892833564	5.10562E-14
AKR7A3	0.286249745	7.761025497	5.12799E-14

LRFN3	-0.380954339	7.898261334	5.52447E-14
OLFML2A	0.687493215	9.140369628	5.60788E-14
CBR4	0.392104003	8.753368345	5.61719E-14
C20ORF26	0.786628816	8.598929197	5.70228E-14
ID1	-0.685264884	9.033752248	5.84843E-14
DNAJC19	0.531100989	8.531476387	5.92225E-14
COL4A3BP	0.417509236	7.876149207	6.00748E-14
KBTBD3	0.266531527	6.927928998	6.13208E-14
MFNG	-0.420881564	7.491036128	6.1445E-14
ZKSCAN4	0.249336371	6.990353581	6.26598E-14
TNFAIP8L3	-0.832471082	6.75995232	6.35574E-14
TCIRG1	-0.268953239	6.707433335	6.36591E-14
LONRF2	0.271603473	6.772593888	6.44007E-14
FBXO8	0.328128684	8.802015564	6.48415E-14
ITGA5	-1.053960692	8.78861633	6.52053E-14
ST3GAL2	-0.300533372	6.617692836	6.61678E-14
LIX1L	-0.357751344	7.940927946	6.65172E-14
MN1	0.44827806	7.694644488	6.71665E-14
HYI	0.333417203	6.834505032	6.7394E-14
PTRH2	-0.311174294	7.650393138	7.04222E-14
RNF103	0.556278967	10.73052983	7.04654E-14
ANKRD13A	-0.817699235	8.857674137	7.07975E-14
SPATA7	0.279650964	7.104332969	7.44404E-14
CTNNA3	0.305950626	6.743437971	7.54601E-14
KANK1	0.444934119	8.914765358	7.58468E-14
TNPO1	-0.282352392	7.510440588	7.81206E-14
LOC401431	0.311074019	6.830425108	7.97777E-14
U1SNRNPBP	-0.366568457	7.624892316	7.98113E-14
C10ORF11	-0.416426374	6.668134122	8.00202E-14
CDC37	-0.313306371	12.47860484	8.45457E-14
ST13	0.382296777	10.09443508	8.58296E-14
SLC25A43	-0.380437563	7.037356191	8.61494E-14
ALPL	-0.649066159	8.456244723	8.76454E-14
JMJD1C	0.29831129	8.050550098	8.78081E-14
BAZ2B	0.422710119	9.047533729	9.11676E-14
SNAPC5	0.425055721	7.660398029	9.2121E-14
THAP9	0.273595758	6.374458807	9.35645E-14
PEMT	-0.26196247	7.32167107	9.42186E-14
ANPEP	-0.482266066	6.383035579	9.69995E-14
NCDN	0.241178994	6.778493634	9.7404E-14
PMS2L5	0.30033645	7.400026895	9.83009E-14
TPM3	1.306328046	8.288219449	9.91105E-14
WDSUB1	0.493513816	8.467574009	1.00251E-13
SCO1	0.420646859	10.45039886	1.06882E-13
DAND5	0.514521962	7.444269144	1.08438E-13
DBC1	0.787263459	6.872628568	1.08673E-13
WBP1	0.319890288	7.995162965	1.09607E-13
RGS11	0.48931277	7.641606972	1.09936E-13
PARP2	0.2471326	7.656576831	1.14232E-13
BHLHB9	0.312358931	7.184449131	1.16998E-13
ECOP	-0.262745183	6.594745709	1.17595E-13

AZIN1	0.560390743	9.240539936	1.18908E-13
LRWD1	-0.317180712	7.449592468	1.19325E-13
RABGGTB	0.306450554	9.9894153	1.21247E-13
XBP1	-0.598571246	10.12297368	1.22094E-13
TRIM5	-0.262930511	6.691980418	1.25259E-13
MRFAP1L1	0.243837845	9.083211681	1.27125E-13
AP1B1	-0.305186106	7.472879082	1.27632E-13
CCPG1	0.451832896	9.478826434	1.27916E-13
PGA5	-0.263585224	6.051596436	1.28411E-13
EDEM2	-0.402557318	7.46884403	1.2983E-13
PTDSS1	-0.806505606	10.01270264	1.33274E-13
PQBP1	-0.279772898	7.224271373	1.34207E-13
ZDHHC5	-0.284239908	8.095641244	1.3524E-13
CLK4	0.392572106	7.438836846	1.37693E-13
RANBP10	0.321772336	7.562008869	1.37712E-13
ARHGEF6	0.57681945	11.06755743	1.38929E-13
SMARCD3	0.392953715	8.653127731	1.38955E-13
FAM131A	-0.320814932	8.27632342	1.39537E-13
SETMAR	0.396875619	8.803242778	1.39641E-13
PICALM	-0.720497811	8.014656997	1.41399E-13
G6PD	-0.34089879	6.636357966	1.41627E-13
C9ORF114	-0.367820335	7.612278847	1.43506E-13
THSD4	0.381460149	7.176908633	1.44602E-13
RNF214	0.214356725	7.020369687	1.46338E-13
DAGLB	-0.507502557	7.705502284	1.51115E-13
TMEM150	-0.333405039	8.156586496	1.53436E-13
SOX15	0.641484519	7.40838528	1.53581E-13
RUNX1T1	0.208737876	6.569105811	1.62142E-13
CBR3	0.480101112	7.216476121	1.63706E-13
KIAA0040	-0.265010955	6.583665022	1.6794E-13
FLJ10986	0.518566129	9.140835274	1.68229E-13
SIGLEC10	-0.557485822	6.183071346	1.70932E-13
TTC14	0.368188737	7.751906563	1.71189E-13
IFIT2	0.69094878	8.150475366	1.74514E-13
IL17RB	-0.253080263	6.064877478	1.76449E-13
RGS6	0.318329973	6.852904943	1.76817E-13
ZNF407	0.296720046	7.611310141	1.78948E-13
ZSCAN5A	-0.287318735	6.718354502	1.81092E-13
TBC1D8	0.436397932	8.552265002	1.81969E-13
RIPK2	-0.422675208	7.64878304	1.84853E-13
CRIPT	0.500484609	9.293746507	1.86005E-13
KCNE1	0.513553655	6.5838864	1.87689E-13
LOC63920	0.329887555	7.076847298	1.93695E-13
C9ORF119	0.413694034	8.468976335	1.94791E-13
IL7R	-0.254377429	6.225812501	1.96448E-13
FAM152B	-0.380919353	8.935188573	1.96804E-13
C19ORF22	-0.439452456	8.639082988	1.9774E-13
PIK3R1	0.530802641	8.626717827	2.00488E-13
NAAA	0.281703652	7.282634113	2.01807E-13
STOM	-0.456167154	10.18829539	2.03381E-13
GTF2F2	0.309827591	10.36415867	2.03748E-13

ELA2	-0.276680882	6.151353413	2.07999E-13
ZNF323	0.356784739	7.195311334	2.12966E-13
AGBL5	0.306155352	7.185389236	2.1326E-13
UPF2	0.228790304	8.540071233	2.26126E-13
C3ORF23	0.232036136	6.75203756	2.29215E-13
WDFY2	-0.259817888	6.842719977	2.30831E-13
KIAA0831	0.519793632	8.587712088	2.32327E-13
C20ORF177	0.592611622	9.364086734	2.34049E-13
TUFT1	0.463649728	7.514073764	2.35313E-13
SH3GL2	0.992589821	8.710811027	2.37901E-13
SLC25A18	-0.361656895	6.437245615	2.41402E-13
HSPA6	-0.939502097	6.962799309	2.42448E-13
SERPINB8	-0.521963614	6.40689679	2.43004E-13
ATP1B3	-0.617943596	8.782647047	2.43238E-13
PCBD1	0.348868037	9.509568523	2.49516E-13
PGBD4	0.166335033	6.453775571	2.50127E-13
VSTM2L	-0.518501155	6.46333812	2.58644E-13
TUBB2A	-0.477349008	6.708781087	2.6758E-13
KIAA1383	0.278628625	6.73360754	2.73964E-13
PLP2	-0.454236932	6.990683481	2.74429E-13
C10ORF26	-0.343934602	7.612391402	2.76178E-13
SERPINB1	-0.628963054	8.132001348	2.88267E-13
SNX1	0.290909115	7.463897115	2.95581E-13
SFRS9	-0.356854969	9.561157288	2.95724E-13
IAH1	0.441044141	10.35467672	2.97742E-13
WDR4	-0.322701684	7.02560875	2.98253E-13
TEX12	0.306688564	6.428185771	3.02429E-13
FOS	-1.436188744	7.422432308	3.0853E-13
PILRA	-0.188473744	6.423902316	3.11066E-13
CNKSR3	0.468078769	9.413999096	3.12325E-13
ATM	0.329189404	7.478456987	3.12848E-13
SPATA2L	-0.391336761	6.896818695	3.13801E-13
GAS2	0.332091197	6.818800134	3.15307E-13
ZNF598	-0.339984461	7.721031881	3.19711E-13
AK3L1	0.338625511	8.332849834	3.41293E-13
NUDT4	-0.177485587	6.191100197	3.41659E-13
ZFP3	0.319043735	7.682344192	3.42098E-13
FAM129A	0.648064839	11.40336356	3.4234E-13
TMEM30B	0.613945458	6.750468706	3.43596E-13
AKR7A2	0.332439213	10.52420791	3.453E-13
ENAM	0.488114272	7.058714451	3.48924E-13
ALDH1A3	-0.748272859	6.813119162	3.50134E-13
FUNDC1	0.438652607	8.723392433	3.50947E-13
ZMYND11	0.301669467	9.804606991	3.53965E-13
DHX40	0.23294802	7.235753375	3.54583E-13
VGLL4	0.351568619	9.618025672	3.5535E-13
RYR2	0.873003299	11.5751854	3.67092E-13
HSPB7	-0.457897353	13.3895532	3.69858E-13
AMPH	0.413178931	7.163577474	4.03714E-13
LOC541471	0.309974894	6.392425632	4.13654E-13
ITGA3	-0.420037655	7.648761178	4.17743E-13

C7ORF53	0.514620762	7.21411185	4.24817E-13
PAPD4	0.355998687	8.740183592	4.30557E-13
LOC88523	0.411872937	10.72531984	4.35561E-13
NP	-0.731353207	7.145353999	4.45969E-13
KIAA1712	0.310684943	7.99610118	4.61093E-13
GJA4	-0.457693371	8.291162717	4.68814E-13
C1ORF175	0.530719102	7.142038072	4.70354E-13
FNDC1	0.757184777	7.710921286	4.75992E-13
SRPRB	-0.479343702	9.463488911	4.78084E-13
CNTNAP1	-0.480508487	6.949567506	4.83269E-13
DOK5	0.455064473	6.871934111	5.04174E-13
TXLNA	-0.36827012	9.283843204	5.05749E-13
TOB2	-0.339059605	7.191077799	5.10696E-13
PKD2	0.307784645	7.805732082	5.11753E-13
LPCAT1	-0.391028749	6.817493745	5.19474E-13
KLHDC6	0.555842761	9.049444752	5.22223E-13
NDEL1	-0.32748811	9.192463317	5.22415E-13
C11ORF1	0.411703363	8.087401897	5.26872E-13
FNIP1	0.351709548	7.737667891	5.42933E-13
CAMK2B	0.403332325	9.390579616	5.48343E-13
MSX1	-0.585992	8.141172222	5.64856E-13
LONRF1	-0.361737634	7.122286396	5.67541E-13
C4ORF18	0.425588971	9.341746277	5.77367E-13
ZZZ3	0.31449361	8.541194002	5.78592E-13
LOC390975	-0.279861032	6.393709456	5.79325E-13
ZNF706	0.263196064	7.559235855	5.81814E-13
LCN12	0.327414187	6.482028551	6.07204E-13
RARB	0.268843755	7.068488834	6.076E-13
CTSK	0.619830742	7.761376578	6.09558E-13
PRKCB1	-0.223862838	6.151853956	6.1321E-13
KLF2	-0.643351776	11.2926599	6.23364E-13
ERCC5	0.322209865	8.770126713	6.44984E-13
COX15	0.300067723	7.606665516	6.5335E-13
MKRN2	0.433682047	9.811140586	6.75888E-13
BYSL	-0.459422656	7.593662378	6.82255E-13
COL6A3	-0.696795722	10.18444755	6.99805E-13
STXBP6	-0.699845706	7.974673253	7.00915E-13
GPR22	0.596341234	7.889432436	7.02758E-13
GGH	-0.289474996	6.451540641	7.18588E-13
ZNF544	0.269232879	8.132606189	7.18647E-13
ATP2C1	0.303196051	8.059730502	7.44184E-13
CHST13	-0.288657526	6.402190044	7.54436E-13
WDR46	-0.455505813	7.749740738	7.64753E-13
BST2	-0.748710886	8.226700304	7.74225E-13
TCF21	-0.561782231	7.218721842	7.99188E-13
NDFIP1	0.307692467	7.963765818	8.10273E-13
RBPJ	-0.208745309	6.668034749	8.11021E-13
ARHGEF12	0.318045107	7.843510564	8.40261E-13
LOC644760	0.594914511	8.008989782	8.49493E-13
GPR137C	0.258282674	6.64460843	8.63528E-13
PFDN1	0.358190456	9.354285042	8.74785E-13

SCAP	-0.353308962	9.169167461	8.82712E-13
C1ORF63	0.399062117	7.84823215	8.83275E-13
CASQ1	0.874748517	8.686830805	8.86561E-13
LIN7B	0.566830276	8.627248621	9.25071E-13
SLC1A7	0.474602647	6.561659288	9.39011E-13
RNF165	0.59635635	7.44997661	9.40761E-13
CYFIP1	-0.253551037	7.264133037	9.41469E-13
RBM24	0.505073106	9.850065261	9.5205E-13
MGEA5	0.387658585	11.2551719	9.53478E-13
OSCAR	-0.200836881	6.554964472	9.58839E-13
UBXN2A	-0.396877179	8.440895437	9.68637E-13
TM9SF1	-0.217935722	6.880974618	9.75976E-13
FAM59A	-0.337750063	6.715941756	1.00412E-12
PGBD3	0.242194783	6.562403018	1.00559E-12
ELF4	-0.294434293	7.202606928	1.04701E-12
ZNF540	0.335998741	7.025257882	1.06575E-12
MPPE1	0.421916453	8.717322516	1.07485E-12
CPNE8	-0.346370697	7.286024839	1.07562E-12
GTPBP6	-0.426780085	10.54912631	1.0763E-12
KIAA0922	0.387367347	7.850401876	1.078E-12
TIMM44	-0.35168941	8.156950129	1.07831E-12
PROK2	-0.289220895	6.108276693	1.10029E-12
CDK2	-0.286747151	7.959895709	1.11383E-12
CA3	1.362298452	7.223133583	1.12195E-12
CENPB	-0.319252376	10.86163897	1.14504E-12
TMEM49	-0.459195959	9.002170444	1.19194E-12
TMEM167B	0.333919458	8.846744991	1.19829E-12
FBXL22	0.370960842	6.564361709	1.20258E-12
ANKRD46	0.410057936	8.932038735	1.24399E-12
MSRB3	0.382383424	9.624476968	1.2571E-12
BAZ1A	-0.356531239	6.84475174	1.302E-12
GLT25D1	-0.455621651	8.899000681	1.38672E-12
C12ORF39	0.41851675	7.148774261	1.38923E-12
IFITM1	-0.669740207	10.4345323	1.40306E-12
PDK3	0.288380439	6.991347237	1.42624E-12
MYO9B	-0.295178306	7.286420927	1.43585E-12
NUP62	-0.278419066	9.134584815	1.44325E-12
KCNAB1	0.272727301	6.883111451	1.47719E-12
B4GALT3	-0.33401181	8.719190779	1.49415E-12
TMEM144	0.494959914	7.925602726	1.50194E-12
SLC25A44	-0.282272401	8.884157603	1.55525E-12
ZBTB47	0.549085852	9.298744909	1.63506E-12
GNL1	-0.291610691	7.62208768	1.66734E-12
SEC15L2	0.148572542	6.299298798	1.66961E-12
TNFRSF6B	-0.279185718	6.432171688	1.68473E-12
PFKFB3	-0.387975463	8.010246436	1.68817E-12
LOC440589	-0.440077525	13.26915102	1.70444E-12
ANXA2	-0.6756556	9.95694366	1.7252E-12
CMBL	0.624459815	9.548612093	1.72729E-12
LOC651380	0.379479902	6.989393218	1.73998E-12
LOC391692	-0.326763067	6.65317272	1.74507E-12

CX3CL1	-0.540705613	8.462774573	1.78027E-12
PSIP1	0.391831135	7.486334588	1.78457E-12
IL8	-0.741904287	6.478884079	1.81684E-12
VPS13A	0.355731464	7.318321525	1.84041E-12
WDR70	-0.353697202	7.635052183	1.86842E-12
CDKN1A	-1.094740062	8.144431354	1.86876E-12
GRIA3	0.251539119	6.401912382	1.94196E-12
HDAC5	0.195826339	6.501748836	1.94329E-12
HSF1	0.428764858	6.973424482	1.94387E-12
FBXO3	0.373755517	8.023534573	1.9681E-12
ELF1	-0.356610094	9.061931079	1.99817E-12
LOC196549	0.742949799	8.129297782	2.00273E-12
QSOX1	-0.455538602	7.497295829	2.04196E-12
FLJ10781	0.581374131	7.162370935	2.04651E-12
PLEKHH2	0.417912557	6.663505368	2.09782E-12
SIPA1	-0.34357839	7.825546861	2.10665E-12
C1QA	-0.481586194	6.842562838	2.13974E-12
IRAK1	-0.517505874	8.248827817	2.14411E-12
CTSS	-0.279660445	6.346305818	2.15159E-12
C14ORF45	0.231107525	6.731454243	2.15286E-12
OSTF1	-0.395368949	8.58583546	2.16532E-12
DUT	0.274284563	7.658736898	2.1768E-12
LOC648500	0.328507622	6.701124464	2.1783E-12
MID1IP1	-0.551821487	7.908065836	2.23146E-12
KAL1	0.457253396	7.554770656	2.31278E-12
ZFP106	0.398048268	12.5624918	2.34272E-12
TFE3	-0.278344697	6.691598856	2.35355E-12
FAM20C	-0.225546567	7.025737402	2.39223E-12
NFKB2	-0.213615507	6.478862326	2.42102E-12
LEPREL2	0.443013743	7.462595922	2.61959E-12
TRAK1	0.321450971	7.973290122	2.66419E-12
CCM2	-0.344894299	8.459535286	2.69486E-12
CRISPLD2	-0.660178551	9.123552006	2.70476E-12
CYP2U1	0.279878086	6.754998342	2.70852E-12
ANTXR2	0.422684325	9.411886079	2.78199E-12
ACADM	0.462234423	11.83484409	2.80894E-12
PROX1	0.67217842	8.434822053	2.82673E-12
SCD	-0.951477555	7.855180814	2.82909E-12
MFAP4	0.747117462	10.18903797	2.84661E-12
KIAA1737	0.422489338	9.955394696	2.86954E-12
NCOA7	-0.425870272	7.877697223	2.90483E-12
MAEL	0.422065874	7.003025818	2.94266E-12
PHACTR4	-0.348937219	7.81119586	2.95267E-12
OSBPL2	0.394953999	8.320957973	2.99621E-12
IL1RL1	-0.773520453	6.28035773	3.01729E-12
SH3D19	0.479278288	10.06236713	3.03822E-12
ZC3H12B	0.240950115	6.421066175	3.07099E-12
ACAD8	0.35185175	8.34921781	3.17412E-12
MTMR10	0.353250048	7.882159238	3.18636E-12
DYNC1LI2	0.312634932	11.25147323	3.20817E-12
POLA2	-0.285872457	7.294665754	3.22383E-12

ATF3	-0.729194903	6.824173878	3.25766E-12
ATF7IP2	0.356001909	7.887678302	3.29593E-12
SLC35A2	-0.198383379	7.082027463	3.34696E-12
PSMD3	-0.386438116	7.108310955	3.34793E-12
FAM13A1	0.240997482	6.697158423	3.36193E-12
SLC30A2	0.704347179	7.381217831	3.45707E-12
CDKN1B	0.449071531	8.505513721	3.46733E-12
PREPL	0.410189829	8.889018332	3.4732E-12
ZNF541	0.577865331	7.295932211	3.55559E-12
C20ORF24	-0.310865555	10.99013303	3.63692E-12
KHDRBS3	-0.396316859	8.022910825	3.65685E-12
GOLSYN	-0.333934929	6.578237277	3.65915E-12
DKFZP564O0823	0.624406989	10.76781323	3.72113E-12
ZNF763	0.299765214	6.619169126	3.76099E-12
NR2C2AP	-0.318862336	6.873903935	3.77022E-12
RABGAP1	0.449168898	9.736422058	3.82548E-12
LIAS	0.403713724	8.127101483	3.8608E-12
LOC285908	0.329520561	6.841517503	3.87562E-12
DARS2	0.309808828	7.539911756	4.02909E-12
NME1	-0.637158234	8.493027949	4.05169E-12
ACTG1	-0.566040363	11.89831485	4.06812E-12
TNIK	0.368560266	7.415580578	4.08273E-12
CTSG	-0.657468402	6.854876723	4.1033E-12
PACSIN2	-0.373399811	10.55986502	4.11109E-12
C17ORF91	-0.606904819	9.201327289	4.14322E-12
SOCS2	0.632340892	9.136884897	4.17941E-12
ABHD11	0.310065642	7.042514322	4.2294E-12
ZNF302	0.360539996	8.837905718	4.25317E-12
SLC9A3R1	-0.568071546	8.011942041	4.30298E-12
COPZ2	0.395746829	7.801057982	4.30308E-12
OTUD4	-0.279747051	6.868993863	4.34681E-12
UBB	0.38118468	13.28838855	4.41848E-12
IKBKG	-0.375791951	8.70231137	4.57074E-12
IRF8	-0.36191806	7.096363652	4.58124E-12
ORC5L	0.189260859	6.996128341	4.60656E-12
EPAS1	-0.416140789	11.23850158	4.65121E-12
ZCCHC7	0.356247328	8.096409115	4.71334E-12
GPATCH4	-0.447109475	7.287882005	4.73949E-12
ALDH6A1	0.446190709	8.600515694	4.84704E-12
NOLA1	-0.372036187	7.982094899	4.88054E-12
ORC2L	0.290106418	8.144231662	4.89278E-12
PROCR	-0.31865745	6.79773196	4.93668E-12
MARK3	-0.304583645	7.562008403	4.966E-12
ORAOV1	-0.34012484	7.502097569	5.00263E-12
RAI2	0.267742926	7.33188003	5.09516E-12
NT5C2	0.466561454	9.522773677	5.10189E-12
SLC25A39	-0.338095554	9.68219904	5.20271E-12
TARS2	0.313863198	7.283481145	5.27616E-12
ACOX2	0.606715788	9.686415607	5.47482E-12
RBMX2	0.16742977	7.179912603	5.58817E-12
ETV6	-0.272969312	7.547047362	5.61158E-12

MAP4	0.682223993	8.574691231	5.64761E-12
THNSL1	0.306818841	7.120306185	5.94322E-12
NFYC	0.330221137	8.776025593	5.97884E-12
RGS17	0.243376449	6.506330672	5.9974E-12
RNF144B	-0.409456096	7.412215661	6.05215E-12
HTR2B	0.536228869	6.776895295	6.23445E-12
ZMYM4	0.342724214	8.028796795	6.24286E-12
CASP4	-0.222469567	6.67686657	6.25118E-12
CMTM8	-0.426133225	7.902438104	6.40753E-12
DIXDC1	0.337860344	7.437556898	6.42105E-12
CCDC130	-0.288071693	10.2218401	6.4896E-12
ADHFE1	0.432484079	9.712805763	6.51249E-12
TCEAL7	0.274672306	6.63946558	6.52629E-12
SURF4	-0.468623872	8.973839517	6.56343E-12
CCDC66	0.264589438	7.171259271	6.56732E-12
NSDHL	-0.441501631	8.472983554	6.61273E-12
LOC730024	0.20368381	6.425140358	6.66861E-12
CST3	-0.354436129	12.55809658	6.75379E-12
NRSN2	-0.315419329	7.422839198	6.76027E-12
KLF12	0.215576975	6.897878151	6.94294E-12
ZNF518A	0.305558451	7.301277681	7.06039E-12
ATAD3A	-0.369736528	7.07668396	7.15439E-12
GFM2	0.34579446	7.073687278	7.30091E-12
RNF220	-0.264599789	8.602509804	7.33742E-12
OPN3	-0.202649151	6.340774173	7.38475E-12
GRAP	-0.136477851	6.308870177	7.56651E-12
LEAP2	0.253757527	6.73931601	7.61468E-12
PPP3CC	-0.376996579	8.95250812	7.96304E-12
CCDC91	0.397883547	7.86083245	7.9839E-12
EPHA3	0.245726439	6.488043102	8.0915E-12
CAPZB	-0.25784856	8.951545134	8.12608E-12
SLC6A9	-0.130129544	6.202912734	8.58805E-12
LOC203547	-0.23083313	9.1816898	8.62894E-12
DPAGT1	-0.334916167	7.616289168	8.70194E-12
VAT1L	0.623929526	7.640321758	8.92726E-12
TINF2	-0.440761091	9.141756759	8.93494E-12
FBXO31	0.397926314	9.545588624	9.03091E-12
SOX17	-0.431115593	6.94888959	9.12684E-12
SHISA5	-0.378589733	9.299888523	9.28076E-12
LITAF	-0.691391324	9.477502518	9.33911E-12
ARHGEF3	0.381809255	9.317855539	9.35615E-12
CNNM4	0.381900434	7.155380787	9.38433E-12
IGBP1	0.346320847	8.897995764	9.49354E-12
PSCD4	-0.415567494	7.013481783	9.52697E-12
ARAF	-0.278822126	9.512647811	9.87155E-12
C19ORF54	0.280689537	7.132129171	9.8787E-12
SLC6A1	0.266053256	6.542670224	9.93743E-12
C9ORF61	0.439488212	8.50095189	1.00102E-11
RGS5	0.527425568	10.62275405	1.01004E-11
MTHFD2L	0.423018724	8.205470188	1.02809E-11
HBEGF	0.733478452	9.814438749	1.04534E-11

SLU7	0.343391071	8.26375548	1.0626E-11
C17ORF85	0.23343933	7.473762954	1.06536E-11
MAFG	-0.17274372	6.802550059	1.07178E-11
EVC	0.160408498	6.479780372	1.08357E-11
AQP10	0.245515882	6.223987197	1.08686E-11
NMB	-0.373952349	6.647448362	1.10011E-11
SMARCE1	0.286620791	8.45479183	1.11646E-11
FLJ35258	0.635304579	7.610940078	1.11693E-11
TOR1AIP1	0.275988399	9.318051446	1.12436E-11
EBI3	-0.301899294	6.30217536	1.13555E-11
C8ORF40	0.466690765	10.28870883	1.13664E-11
CCDC112	0.260250473	6.974221606	1.14715E-11
C6ORF91	0.376756913	6.690291242	1.15863E-11
TSPAN12	0.519511283	8.172815146	1.18016E-11
GLOD4	0.287334197	8.769155342	1.18689E-11
SCAMP2	-0.298795255	6.741369457	1.1984E-11
NIP7	-0.37962563	7.898621678	1.22393E-11
ACADL	0.276631289	6.239126316	1.26622E-11
ZNF135	0.312222926	7.222531854	1.30313E-11
IL6	-0.602685835	6.431536327	1.31526E-11
CORO1B	-0.277056618	7.176992499	1.32915E-11
PIAS2	0.172007446	7.006441866	1.33271E-11
ITPKC	-0.315085446	6.594449313	1.36429E-11
TCEAL4	0.238970877	10.15371212	1.3669E-11
CMIP	-0.432750047	7.223755833	1.3755E-11
ATPAF2	0.278197727	7.919802285	1.37582E-11
LOC121952	0.473286298	7.097601507	1.3833E-11
EIF3B	-0.362114861	10.23889044	1.40205E-11
ARV1	0.386240628	8.992353587	1.4226E-11
NHLRC3	0.25894939	7.35836323	1.42898E-11
DNAJC13	0.243734521	7.975215934	1.43653E-11
CLPTM1	-0.332720525	9.461962124	1.44391E-11
NAT10	-0.3242828	8.880959265	1.45434E-11
UNQ1940	0.518466754	6.787783528	1.45448E-11
ZSCAN18	0.438446102	10.8621623	1.48673E-11
CD300A	-0.240363393	6.25629441	1.50486E-11
FAM92A1	0.358992593	7.886037473	1.50683E-11
VGLL2	0.502522683	6.429378039	1.51337E-11
C1ORF165	0.283191515	6.783872512	1.51604E-11
SEPT9	-0.293757108	11.05447111	1.53256E-11
TIMP1	-1.057636209	11.7269992	1.53281E-11
LOC400963	-0.400036977	13.0099513	1.53433E-11
INTS9	-0.221027215	8.704345516	1.5879E-11
ARMCX2	0.390795879	9.660545973	1.59742E-11
LCMT1	0.205034811	9.819108061	1.62137E-11
ATP6V1F	-0.536913607	10.49567014	1.64144E-11
PRKCH	-0.522221132	8.605017133	1.64574E-11
HARS2	0.28771987	9.719855441	1.65952E-11
TSC22D1	-0.580257223	9.543897354	1.65996E-11
C1ORF25	0.211469456	7.267616455	1.70534E-11
THUMPD3	0.183812273	6.87531585	1.71679E-11

TIMELESS	-0.267102506	6.732779222	1.7809E-11
RNF126	-0.277426529	8.000029155	1.79702E-11
YWHAQ	0.372293228	12.47102111	1.79767E-11
GLT25D2	0.348673972	7.078475839	1.84687E-11
CCL11	-0.313600748	6.266374003	1.87792E-11
TRUB1	0.386179649	6.693744875	1.89493E-11
TP53INP2	0.513562037	10.10344167	1.93112E-11
PFDN4	0.30087364	7.121213825	1.93617E-11
TNFAIP3	-0.30193663	6.988927593	1.95863E-11
IL18RAP	-0.270714174	6.295127063	1.96692E-11
ZNF630	0.200635182	6.575993933	1.96812E-11
MT1F	-0.533683035	7.337827105	2.00469E-11
C10ORF35	0.405794873	7.975447306	2.00621E-11
SLC38A10	-0.179723965	6.606533403	2.03205E-11
C10ORF125	-0.289780309	6.394358288	2.03428E-11
KCNIP2	-0.277438104	6.14302694	2.05771E-11
EIF4A2	0.3372489	12.74923248	2.11962E-11
CDC42EP1	-0.207996017	6.596489428	2.12862E-11
TTF1	0.222778631	7.290027615	2.13189E-11
TUBB3	-0.516400415	7.011783744	2.13357E-11
CAMK1D	-0.161075293	6.145510338	2.16349E-11
FCGR1A	-0.320391055	6.210707516	2.19916E-11
MAPK7	-0.143057207	6.499778036	2.21136E-11
RAB23	0.403160016	7.836682023	2.23331E-11
UAP1	-0.525199911	7.82059622	2.24358E-11
ENAH	0.265180501	7.075752668	2.26948E-11
NEK1	0.308246191	7.766017112	2.28689E-11
LOC652458	0.321776893	6.968994102	2.31271E-11
PRIC285	-0.533377359	8.243770149	2.31706E-11
PDIA4	-0.439436851	6.658841648	2.35371E-11
CSDC2	-0.54882135	10.42422209	2.36777E-11
RAB5C	-0.345759148	7.666576363	2.39498E-11
FBXL2	0.234090801	6.523010284	2.41136E-11
ANXA5	-0.596589385	10.50722004	2.41782E-11
LOC649143	-0.720276226	7.20431858	2.43856E-11
TNMD	0.543873513	6.727085207	2.47222E-11
SPNS1	-0.414954662	8.456914815	2.47446E-11
CDH19	-0.430818128	7.473001401	2.63954E-11
UBE2W	0.230842193	7.002599147	2.66764E-11
TSSC4	-0.428962722	8.181489666	2.679E-11
FAM120B	0.316674485	9.868607981	2.68495E-11
MYO1G	-0.356620763	6.47177787	2.72126E-11
APP	0.808510501	9.384388515	2.72758E-11
DNTTIP1	0.324844733	8.692365567	2.7569E-11
PPP1R9A	0.309751288	7.039184986	2.7611E-11
LOC199800	-0.235167362	6.451817353	2.76633E-11
SPTAN1	0.43853074	9.717554765	2.78569E-11
FBXL6	-0.189358289	6.703385772	2.78751E-11
AKR1C4	-0.490570143	7.664045052	2.812E-11
ARHGAP1	0.409415437	10.5484087	2.84692E-11
FYB	-0.237296981	6.334185952	2.87108E-11

CCDC86	-0.522846089	7.510527549	2.88296E-11
MTMR15	0.321807045	7.604085146	2.89998E-11
CD40	-0.184419277	6.289690988	2.9113E-11
HERC5	0.553979067	8.40148262	2.93822E-11
IL1R2	-0.467473542	6.174175418	2.97482E-11
CLCC1	-0.214001991	6.574590695	2.9788E-11
IFT88	0.19418212	6.75461453	3.06497E-11
CPEB1	0.172432372	6.196010889	3.06905E-11
LOC441408	0.478458319	8.485961729	3.06969E-11
ACOT1	0.50587862	8.69565516	3.0771E-11
BRP44L	0.449887522	12.72819821	3.10102E-11
C11ORF60	0.256804607	7.603464703	3.20971E-11
RECQL	0.320647324	7.504122284	3.2882E-11
C21ORF7	1.315687381	8.424081811	3.30811E-11
C2ORF34	-0.265274954	6.829388832	3.32164E-11
MGC40489	-0.185765791	6.452357891	3.35327E-11
LIPG	-0.636297864	6.224990203	3.45212E-11
ARD1A	-0.396449134	8.52664635	3.45382E-11
VIL2	-0.373542857	11.41865322	3.48855E-11
BIRC6	0.22601175	7.397733751	3.54112E-11
PSCD1	-0.328780363	9.274269746	3.56221E-11
TTLL7	0.313762627	6.611326448	3.58532E-11
PCMTD2	0.507679316	8.97338438	3.6052E-11
DCTN6	0.319215951	8.882314238	3.66094E-11
ZNF653	-0.321000243	7.671947777	3.70808E-11
HIST1H2AC	0.594700563	8.216338978	3.72222E-11
KIAA1598	-0.334864758	7.236273741	3.7242E-11
TNFRSF10B	-0.260926451	7.064998262	3.72841E-11
NFE2L3	-0.208969347	6.346920741	3.75626E-11
ZNF235	0.183600323	6.598823685	3.75677E-11
ARRDC3	0.2703069	6.74958456	3.82801E-11
CLASP1	0.332979657	9.621636605	3.8481E-11
C19ORF24	-0.360080562	7.149137502	3.87933E-11
CD37	-0.217881955	6.241808376	3.87989E-11
MAEA	-0.333116368	7.518826846	3.92579E-11
FLJ20444	0.254098856	7.04270898	3.95213E-11
C9ORF6	0.252458542	7.768747023	4.01808E-11
C10ORF76	0.555876923	8.602506585	4.06884E-11
RCOR2	0.455436383	7.360567516	4.10015E-11
SPIN3	0.18508828	6.69589576	4.13492E-11
KIAA0194	-0.284855585	8.244467964	4.16164E-11
SSU72	-0.319140008	11.66953986	4.19822E-11
LRRC25	-0.208485302	6.151294381	4.20085E-11
RFTN1	-0.430239111	10.19916314	4.20698E-11
C7ORF36	0.262849897	7.746636337	4.20735E-11
VPS37B	-0.348443296	7.95773749	4.22321E-11
ZNF177	0.341563792	7.307013863	4.22874E-11
LOC285550	0.310310451	6.987346348	4.26735E-11
TMEM100	-0.4796344	7.080919284	4.26977E-11
KCNA4	0.283517065	6.477776071	4.39355E-11
LOC649679	-0.265054602	6.513261083	4.49734E-11

BID	-0.225800602	6.724333185	4.51298E-11
GNB3	0.363707677	6.595957108	4.5498E-11
GPR116	-0.405284754	10.80859189	4.55381E-11
DDR1	0.296092175	6.91857969	4.57868E-11
IQCG	-0.778136282	6.713112295	4.60303E-11
PCDHB17	-0.18746835	6.481120471	4.62994E-11
TSPAN13	-0.519496579	7.922965089	4.65456E-11
TRMT11	0.194812576	6.846885834	4.67092E-11
JUNB	-0.711922941	7.311142579	4.72004E-11
LOC91561	-0.332589436	12.89438127	4.76431E-11
TXNDC1	-0.400718123	8.389457405	4.76516E-11
LOC730273	0.267133615	6.645146589	4.7674E-11
ALS2	0.342545492	10.09486284	4.80635E-11
THYN1	0.370725846	8.276720985	4.81954E-11
PMM2	-0.42495372	7.354907069	4.85285E-11
C13ORF33	-0.551064672	6.714246084	4.93582E-11
LOC728014	-0.336199892	7.569539679	5.00672E-11
IGSF1	0.577188888	6.832877352	5.01105E-11
C6ORF153	-0.290180016	9.429974263	5.05037E-11
C1QTNF7	0.235144215	6.355117824	5.07672E-11
MT1E	-0.617584869	9.665542426	5.15471E-11
ARHGAP12	0.268880749	7.072801292	5.22373E-11
BCL9	0.284906599	7.766519877	5.29721E-11
AXL	-0.447156407	7.954870938	5.30474E-11
MXRA5	0.985612124	10.30705692	5.31509E-11
ZNF233	0.283654241	6.717426005	5.34434E-11
GBGT1	-0.301390618	6.67206182	5.37122E-11
PAPSS2	-0.437112122	7.227384761	5.40319E-11
THAP10	0.402633795	7.785979995	5.45259E-11
CPEB3	0.415699486	8.268058988	5.51702E-11
RAB24	0.226552888	7.492025771	5.55439E-11
DPP3	-0.253390163	6.581464224	5.59647E-11
SLC22A5	-0.525280035	7.655742179	5.61486E-11
LY86	-0.342445846	6.696713538	5.67413E-11
CORIN	-1.565963151	8.155973961	5.73866E-11
C14ORF159	0.542392353	10.87584945	5.75992E-11
ADAM15	-0.391506802	9.323410035	5.87058E-11
MYL1	0.324694891	6.387452741	5.87166E-11
DAK	0.180906529	6.569653664	5.91678E-11
RSC1A1	0.208717683	6.746376087	5.93419E-11
PCCB	0.388458108	11.16854672	5.93442E-11
C3ORF54	0.405093188	7.741325272	6.02292E-11
ATP13A1	-0.338827232	7.124662649	6.02922E-11
MBIP	0.218399027	7.206319517	6.11625E-11
DHX32	0.305104127	10.26516859	6.18982E-11
IRF6	0.675669887	7.619448204	6.19235E-11
GABRE	0.480749754	7.523529279	6.25898E-11
PTGER2	-0.225134697	6.184135037	6.3417E-11
HAPLN1	0.505637163	6.640359358	6.41174E-11
SPG11	0.400848584	9.184709802	6.44141E-11
TRIM33	0.267527478	8.142527843	6.47683E-11

TPM4	-0.430919296	6.830665236	6.54155E-11
SEMA4D	-0.27633299	6.87385047	6.55961E-11
EPB49	0.340497011	7.475805351	6.5871E-11
PPP1R1A	-0.565718387	8.211769211	6.64704E-11
HAMP	-1.018311737	6.365078952	6.66135E-11
LOC201175	-0.199788314	6.296353312	6.75818E-11
SLC5A6	-0.384521526	7.40556895	6.77739E-11
LYZ	-0.730492991	6.993296621	6.80665E-11
SMARCA4	-0.3170111	9.594328856	6.81927E-11
SENP5	0.33062477	7.286399037	6.98796E-11
F2RL3	-0.257479409	6.333197464	7.14058E-11
KIF5B	0.500253746	9.159116187	7.24101E-11
PSTPIP1	-0.222013481	6.116778602	7.29577E-11
RGL1	0.352736693	10.21036023	7.50467E-11
TSPYL1	0.307496143	9.533057616	7.54333E-11
CCL13	-0.443764317	6.489227469	7.55583E-11
LOC100008589	1.327965284	12.35183562	7.67139E-11
KLHL36	-0.337542463	7.718792774	7.78055E-11
GNB1	-0.257331394	11.15789887	7.79445E-11
EDNRB	-0.322368802	6.932526695	7.85299E-11
NLGN1	0.47655432	7.838847583	7.85449E-11
SETD8	-0.249807246	7.534183568	7.86108E-11
SBK1	0.449871112	6.984825853	8.056E-11
ALG13	-0.260109925	6.99012109	8.12439E-11
GPX1	-0.356553519	9.7125607	8.23554E-11
PKD1	0.45963559	8.284265035	8.2582E-11
EIF2C2	-0.521615501	8.95358341	8.27142E-11
LOC441461	0.252483571	6.678570895	8.38688E-11
TSKU	-0.45595329	7.697708394	8.45121E-11
NAPB	0.360320016	8.207648244	8.49831E-11
ARHGEF17	-0.379534231	7.580685798	8.51739E-11
RNF146	0.356004316	7.571810196	8.60864E-11
C2ORF33	0.311727	8.371052403	8.62397E-11
C21ORF69	0.220743967	6.293957091	8.62602E-11
NOTCH3	-0.406737575	8.702596711	8.6291E-11
SERTAD4	0.477597923	7.451477253	8.63478E-11
ING4	0.26978484	6.990838081	8.64426E-11
FERMT2	-0.375694761	10.92340294	8.8101E-11
MPZL1	-0.301591494	7.074954076	8.85E-11
CRYZL1	0.237053632	7.766839297	9.07365E-11
SLC26A4	0.391755264	6.393289789	9.08018E-11
ZNF23	0.234183666	6.939122447	9.14902E-11
STK19	-0.357025539	7.982976042	9.32865E-11
GFPT1	0.369348079	8.415967831	9.3529E-11
ZNF76	0.34400235	7.527454858	9.53478E-11
MAN2B1	-0.287867941	6.745794438	9.65488E-11
FHL3	-0.361655822	7.734481112	9.67387E-11
ZNF503	-0.390909476	7.188568164	9.72629E-11
LAMA2	0.365872921	9.53798021	9.75505E-11
ERMAP	0.18839378	6.686099502	9.89905E-11
KIAA0460	0.304248234	6.976210094	9.9269E-11

TMEM79	0.264743157	7.062821409	1.01227E-10
TSC22D3	-0.532662171	8.018269738	1.03483E-10
C14ORF139	-0.281882818	6.91223887	1.04005E-10
GCOM1	0.587714009	9.584802919	1.06698E-10
KLHL24	0.712235002	10.49068589	1.07065E-10
LPAR4	0.225390329	6.703779026	1.07255E-10
PDCD6IP	0.196378559	7.636799895	1.08489E-10
KIDINS220	0.397359596	9.296655022	1.08625E-10
SDF4	-0.289311686	8.703171791	1.09199E-10
C15ORF24	-0.260285493	10.62027045	1.09427E-10
SSR1	-0.346810477	9.268413888	1.09914E-10
C20ORF127	-0.53993722	8.051353392	1.12469E-10
CAP2	0.469864996	11.7900262	1.12786E-10
C2ORF28	0.254268215	11.05931413	1.12811E-10
GPR3	-0.334318012	6.321896979	1.14282E-10
TTC8	0.214199217	6.8887685	1.14541E-10
FAM133A	0.333936917	6.305655916	1.14808E-10
GABARAPL2	0.351884138	11.18517529	1.15152E-10
RCCD1	0.165888382	6.386560213	1.15894E-10
C16ORF30	-0.420955814	8.384915834	1.16451E-10
TTC37	0.440189887	8.900645237	1.17201E-10
PITPNC1	-0.330265735	6.747954563	1.1739E-10
BACH2	0.130344554	6.30811869	1.18351E-10
FBXO21	0.318969065	9.720467153	1.19703E-10
LEF1	0.286023537	6.459702605	1.20795E-10
ATXN3	0.20097995	7.194504912	1.21063E-10
HEXA	0.262637685	6.732608174	1.22677E-10
NIN	0.225164534	7.152800436	1.23422E-10
PDE1C	0.445347422	8.339596329	1.24114E-10
RAB8B	-0.478158894	8.000429766	1.26638E-10
PLSCR1	-0.332120393	6.320712975	1.26745E-10
FBXO18	-0.29248507	8.90279107	1.29007E-10
GLCE	0.283270701	7.874827764	1.29933E-10
RPN2	-0.510356149	7.59371331	1.33154E-10
ZNF230	0.200885737	6.622590061	1.33256E-10
RTKN	-0.17366079	7.138651532	1.35039E-10
MRPS14	0.212261123	6.806719923	1.36634E-10
EPHB4	-0.376930943	7.808455418	1.36735E-10
JAM3	0.33711121	10.4700262	1.38701E-10
SLC25A25	-0.228727441	6.769382596	1.41992E-10
RPL29	-0.563588645	6.92278539	1.42265E-10
TRIM45	0.383463847	7.466967607	1.43876E-10
PTPN1	-0.64043883	9.612522955	1.44429E-10
MMP23A	0.543149696	8.015325994	1.45881E-10
LIME1	-0.327006514	7.093805151	1.46014E-10
SDF2L1	-0.600284191	9.530478549	1.49667E-10
SPRY1	-0.416136497	9.17117509	1.49689E-10
TOB1	0.479231917	9.392507648	1.52467E-10
KIAA2010	-0.242382395	7.742953367	1.54381E-10
SLC2A3	-0.872771436	8.756763397	1.56468E-10
ATP7B	0.207665147	6.849050089	1.56849E-10

PDE4DIP	0.249556382	6.577319148	1.56888E-10
TRPC1	0.337079536	7.982747932	1.5778E-10
MYO19	-0.12925953	6.260194195	1.58407E-10
PCMTD1	0.356734979	9.468898914	1.59899E-10
PER3	0.575989374	8.005963976	1.61207E-10
C1ORF212	-0.410811449	7.60245094	1.61914E-10
HESX1	0.195606187	6.178108144	1.61967E-10
C1ORF183	-0.177122672	6.366780272	1.63925E-10
GSTA4	0.298686658	7.740908979	1.65031E-10
TBC1D4	0.349747469	9.070701762	1.66045E-10
ACLY	-0.321468123	8.442460291	1.66242E-10
GALM	0.293093951	7.652677111	1.66432E-10
RECK	0.260287885	7.273464092	1.68642E-10
RELB	-0.280127839	6.556102718	1.69266E-10
APBA3	-0.262119454	7.940251703	1.69615E-10
IRX3	-0.608374829	11.89689993	1.71455E-10
ADAM11	-0.381984639	6.299085073	1.71508E-10
RASSF7	-0.316970575	7.16531277	1.71679E-10
OLFM1	-0.255289403	6.981332483	1.72984E-10
POLR3GL	0.308619633	10.89622214	1.73067E-10
NOD2	-0.220913494	6.050471568	1.73411E-10
KRIT1	0.235442939	7.454599057	1.73584E-10
PUS7	-0.376983341	7.479784162	1.77294E-10
ADC	0.276296972	6.628062534	1.77939E-10
LEPROTL1	0.332566956	9.82091905	1.7894E-10
JOSD1	-0.373740591	7.570328621	1.79915E-10
C10ORF33	0.464620779	7.704749248	1.85675E-10
UBL7	0.208218087	7.662402857	1.88461E-10
RWDD2A	0.278634556	7.996952416	1.88595E-10
ACADSB	0.398414148	7.264317725	1.9956E-10
FAM58A	-0.305499945	9.029892213	2.0072E-10
SND1	-0.350677307	8.88769639	2.01697E-10
HERC6	0.546208405	8.556019182	2.01756E-10
LOC728037	0.52529231	8.258677668	2.11682E-10
ATP1B1	-0.333355753	11.12188322	2.15739E-10
LOC652097	1.059502107	9.390681396	2.16634E-10
LOC644739	-0.209729343	6.652599798	2.22948E-10
LOH3CR2A	-0.50372202	6.485097286	2.23782E-10
KCTD18	0.268801338	7.09810488	2.32032E-10
LOC653186	-0.253545576	6.281773272	2.33974E-10
GRAMD1A	-0.438482133	7.949375786	2.34965E-10
MYO5A	-0.35069992	7.029185258	2.35099E-10
DPY19L4	0.311657979	8.089555809	2.35951E-10
ABCF2	-0.241767323	7.169876301	2.3873E-10
LMO7	0.397393818	8.336377345	2.41051E-10
TCF25	-0.276215381	9.957581652	2.46652E-10
DNAJB12	-0.144280465	6.983166902	2.50506E-10
DOCK11	-0.289283979	6.998118978	2.50674E-10
C3ORF52	-0.686357189	6.828114529	2.51821E-10
AGAP3	-0.221011794	6.974369981	2.55408E-10
FDFT1	0.258715747	10.23554093	2.56839E-10

POLR2G	0.297977843	9.701201074	2.56866E-10
C4ORF38	0.209462192	6.651294713	2.57883E-10
P2RY2	-0.242863472	6.616593763	2.59738E-10
WEE1	0.448095158	9.611504623	2.61226E-10
BEX1	0.762793733	8.520845003	2.62474E-10
MRPL35	0.320004056	9.664075843	2.62934E-10
UGCG	-0.393788554	7.313850026	2.63737E-10
ZNF654	0.172910404	6.530381388	2.65109E-10
MPP6	-0.209888042	6.212614415	2.67161E-10
PTN	0.287972155	8.594787246	2.69477E-10
HACL1	0.269861145	9.192578416	2.69605E-10
C4ORF29	0.480624688	7.651959885	2.70028E-10
GPATCH1	0.216259097	7.257625986	2.70128E-10
PXDNL	0.593876724	8.120388526	2.70786E-10
ACTN2	0.342691938	11.74488596	2.73841E-10
ZNF366	-0.316463107	6.759510881	2.79749E-10
FAM38A	-0.439090296	8.23713562	2.82827E-10
BIVM	0.318436573	8.898644592	2.85856E-10
EXOC2	0.230797756	7.470146836	2.86662E-10
SEMA6B	-0.151536627	6.479850031	2.90633E-10
MURC	0.523748953	9.829534297	2.91193E-10
ANK2	0.35966093	7.817881014	2.94411E-10
DET1	0.227228853	7.790882289	2.94707E-10
CBX3	0.22943249	7.604199933	2.95709E-10
IKIP	-0.232642002	6.369169973	2.97116E-10
DOCK8	-0.209623897	6.336902723	2.97516E-10
NUP37	0.281471385	9.486772202	2.98552E-10
ZNF626	0.17976989	6.911870037	3.00298E-10
GRK5	-0.441994062	8.045624526	3.00481E-10
AP2S1	-0.257703544	11.48379361	3.03638E-10
LARP5	0.379299525	9.693985876	3.08297E-10
EFHD2	-0.36041338	9.270799966	3.09815E-10
RASD2	-0.359912123	7.396185866	3.11445E-10
LYRM2	0.431191234	9.317486053	3.13595E-10
RFX5	0.250245716	7.669858365	3.14139E-10
CHI3L2	-0.224145726	6.164317093	3.15374E-10
DDX39	-0.419159533	8.556223984	3.15645E-10
SLC35A1	0.265537113	8.918061546	3.21521E-10
OSGEPL1	0.28590895	7.045455921	3.31173E-10
ARHGEF9	0.330679056	6.839543775	3.32058E-10
VPS16	-0.205812231	7.614243354	3.32285E-10
ANKRD6	0.295511161	7.408479323	3.36675E-10
DOCK1	0.264436264	7.640630708	3.36757E-10
SEC61B	-0.346560369	10.59239115	3.36786E-10
AFF3	0.259164496	6.741042346	3.36816E-10
MRTO4	-0.415222176	7.394496167	3.41127E-10
RABGAP1L	0.68226153	8.227766981	3.41332E-10
EIF4EBP3	0.384959554	6.804322955	3.42367E-10
ABCG2	0.196634407	6.147843353	3.45301E-10
QPCT	0.360791859	7.186682195	3.492E-10
GEMIN8	0.194360491	7.058917513	3.49363E-10

POLR1C	-0.299624454	7.904905937	3.49466E-10
AFMID	0.320118273	7.173560442	3.49869E-10
NCAM2	-0.268315564	6.423694393	3.50091E-10
FANCL	0.17828921	6.780898757	3.60117E-10
RARA	-0.256141701	6.883153542	3.64782E-10
USP54	-0.22876082	6.384402138	3.6627E-10
C1ORF188	0.65236973	8.832394438	3.66517E-10
RAB6IP1	0.264819278	11.09899306	3.6652E-10
GLT8D2	0.371296796	7.8248889	3.71759E-10
PFAS	-0.294372696	7.439589503	3.72481E-10
NDUFA8	0.269931283	13.1195335	3.73687E-10
C19ORF12	-0.263125803	8.457958244	3.74293E-10
CAT	0.409947691	9.364060848	3.76068E-10
TXNDC5	-0.336272122	7.787509242	3.8253E-10
AMY2A	0.331367395	7.176696428	3.8725E-10
UBAP1	-0.285368382	8.754367367	3.89992E-10
TAP2	-0.280490108	6.681711847	3.90573E-10
HCCS	0.272662692	8.445171383	3.93372E-10
VDAC3	0.318340781	12.89867696	3.96804E-10
STARD7	-0.351404667	10.53512573	3.97927E-10
IL11RA	0.391131195	9.633891247	3.98413E-10
UNG	-0.380445742	7.233899077	4.01118E-10
AMHR2	0.485171411	6.679158655	4.02072E-10
UBTD1	-0.303642884	8.303124073	4.08243E-10
C10ORF32	0.370525395	8.898550992	4.09975E-10
ESM1	0.481377431	6.520127617	4.12917E-10
PRR6	-0.535717855	8.101641974	4.18947E-10
SH3TC1	-0.307785763	6.706174613	4.19205E-10
LOC648147	0.251842471	6.347592961	4.21599E-10
SPAG16	0.263762373	6.823236015	4.31214E-10
SNTA1	0.399912091	10.39748177	4.31627E-10
NUDT6	0.363939704	7.36017766	4.32314E-10
ORAI3	0.209481253	6.495559178	4.32734E-10
ZNF337	0.305182494	7.836843558	4.34966E-10
MGC27348	0.263893415	6.863244337	4.45009E-10
TMLHE	0.347503173	8.375189051	4.48473E-10
PELI1	-0.382429185	7.271617656	4.5286E-10
RAPH1	-0.251289482	6.798977812	4.54843E-10
CXCL16	-0.356866448	7.15013168	4.57564E-10
IRF7	-0.332173508	6.863592518	4.62566E-10
TMEM165	-0.424180102	6.978510799	4.74274E-10
MLH3	0.152826351	6.60692078	4.82628E-10
AZGP1	-0.660350194	7.427974965	4.84336E-10
PHYH	0.424141286	10.68735851	4.87642E-10
SPCS2	-0.323101679	9.337089333	4.89612E-10
KPNA2	-0.331111046	6.68230939	4.9196E-10
PLEKHB2	-0.317049982	7.38821709	5.00863E-10
FOLR2	-0.41478324	7.01205962	5.01E-10
SFRS2IP	0.241545708	7.342358774	5.01623E-10
AOX1	-0.31353801	6.166369749	5.04959E-10
ZNF594	0.329568758	7.361048785	5.05776E-10

CAND2	0.483352537	9.263813048	5.0799E-10
BMP6	0.390617262	6.833969922	5.08708E-10
DIDO1	0.138922866	6.798670195	5.11683E-10
SREBF1	-0.28405255	6.656605192	5.11839E-10
FABP5	-0.461903335	8.518083393	5.17906E-10
RRP9	-0.295575627	6.228249055	5.27973E-10
CHCHD6	0.337577116	7.773909515	5.29618E-10
ENO2	0.580371506	7.424367326	5.29935E-10
VPS13D	-0.265379032	6.849154396	5.35475E-10
PRDX1	-0.267194478	11.36688622	5.36489E-10
MMP25	-0.152523753	6.229120742	5.36645E-10
CCRK	-0.150138534	6.484077427	5.38825E-10
AUP1	-0.255595931	7.057693913	5.44466E-10
CCRN4L	-0.280238751	6.647593944	5.45953E-10
SPC24	-0.272721007	6.839441637	5.46082E-10
SULF1	0.742652132	8.168956379	5.50241E-10
SDCCAG3	-0.269137164	7.520556327	5.52657E-10
B3GNT2	0.333411631	7.311735092	5.55835E-10
DNAJB6	0.259569885	10.30796132	5.5802E-10
FCGRT	-0.350377992	9.140617523	5.61126E-10
APITD1	0.207593373	7.02772972	5.62577E-10
UBE2Q2	0.362784708	9.598714831	5.62796E-10
PPP1R3A	0.517174537	8.59911925	5.65546E-10
HOMER1	0.423180557	7.707972027	5.68674E-10
ACYP1	0.277417853	7.706338569	5.70313E-10
MGST2	0.354703928	10.99547405	5.70616E-10
IL6R	-0.147951111	6.151057622	5.71817E-10
MRPS36	0.333320409	8.514402589	5.80398E-10
PMPCB	0.304261304	10.47108823	5.87414E-10
SRI	0.205414244	7.607612799	5.93022E-10
FLJ46082	-0.191928916	6.185061153	5.98296E-10
SLAMF8	-0.342722151	6.148426287	6.00503E-10
ATXN10	0.160400943	7.04699083	6.09799E-10
CYP39A1	0.200187374	6.467680814	6.17916E-10
APLP2	0.345459401	8.967096274	6.28112E-10
CXORF57	0.389705946	6.885752551	6.34061E-10
RPP40	-0.379727214	8.368447438	6.34867E-10
GIT2	-0.152970122	6.550823369	6.41427E-10
MGC87042	-0.341695305	6.46839109	6.41795E-10
CD44	-0.673614732	8.267690237	6.4183E-10
DUSP16	0.231960963	6.757905805	6.52964E-10
KCTD5	-0.190170136	7.978709219	6.55896E-10
LOC344595	0.23087121	6.831039306	6.64852E-10
CYR61	-0.927353692	8.739183348	6.80161E-10
LOC201229	0.204038093	6.592088079	6.84076E-10
BASP1	-0.471767319	7.378746704	6.89891E-10
SLC25A19	-0.357076993	7.390050054	6.92096E-10
NPC1	-0.367862808	7.700015912	6.93313E-10
FAR2	-0.296001542	6.578878272	6.97791E-10
PKP2	0.490567675	11.15050741	7.04039E-10
YWHAH	-0.41745947	11.47777414	7.10502E-10

UBE2Q1	-0.302187666	7.21487456	7.18102E-10
GEM	0.582757535	7.563934564	7.21036E-10
FOXS1	0.439473897	6.779515707	7.25882E-10
USP13	0.419747546	10.38932106	7.28027E-10
CCDC147	0.236039921	6.911844271	7.29676E-10
IGFBP5	0.551640927	11.99858235	7.33746E-10
DTX2	-0.232793146	6.987581744	7.45044E-10
TAF9L	0.301256748	7.517552788	7.45631E-10
DMTF1	0.212818918	8.301117422	7.49303E-10
GALT	0.301383656	8.151663853	7.55882E-10
HIP1R	-0.12029691	6.24615596	7.63231E-10
LDB3	0.598094398	9.045555461	7.7136E-10
SEMA5B	0.21924428	6.558102603	7.79246E-10
C9ORF21	-0.396728222	7.366705474	7.84547E-10
KIAA1143	0.148630491	6.601389438	7.92716E-10
TXNDC14	-0.288155296	9.935504702	8.05135E-10
RASSF4	0.289597228	7.908144247	8.0816E-10
TNFRSF21	-0.393065132	8.25593187	8.09155E-10
CLEC16A	-0.350714245	9.130490424	8.10222E-10
CASZ1	-0.314325694	8.560279877	8.13395E-10
CCDC6	-0.488516512	9.296958956	8.19713E-10
TNRC6A	0.258975824	7.305920964	8.20826E-10
SEMA6D	0.097004189	6.183439868	8.21785E-10
LOC283953	0.236788191	6.226192882	8.22445E-10
IMP4	-0.404601339	10.42788267	8.24903E-10
ZNF167	0.116637195	6.310047291	8.36912E-10
C17ORF60	-0.206723839	6.24232624	8.48545E-10
RRM1	0.289537573	9.937417985	8.49373E-10
CLTA	-0.240907348	8.582651788	8.51626E-10
ZFP2	0.225891088	6.621365562	8.80105E-10
ZNF740	0.194458414	6.361291891	8.84252E-10
C1ORF117	-0.656667951	8.333284669	8.89038E-10
LARP6	-0.383151811	9.34674081	8.96406E-10
MYH9	-0.567584088	11.00579126	8.99617E-10
PRKAB2	0.42289661	8.684839304	9.06541E-10
ATHL1	-0.273317682	6.622742046	9.17377E-10
TOR3A	-0.340796926	7.214753999	9.22884E-10
GMFG	-0.363617673	7.581970899	9.26552E-10
FICD	-0.297790316	6.913902105	9.40386E-10
CADPS	0.223865934	6.586175454	9.44179E-10
APRT	-0.294290592	8.150120952	9.52031E-10
HIF1AN	0.292812916	8.055632923	9.70836E-10
ISG20L1	-0.585031061	7.488431599	9.75193E-10
ESAM	-0.423010675	9.115281907	9.82264E-10
GPAM	0.427520718	7.976416086	9.83402E-10
SUB1	0.443985321	9.468692936	9.98352E-10
TOM1	-0.321866344	8.572845589	1.0189E-09
SNX12	0.336454999	6.637841919	1.02893E-09
NUP205	-0.283914586	8.058903455	1.03682E-09
DOK6	0.351348136	6.655787952	1.04141E-09
CKLF	-0.295216349	7.621979408	1.04985E-09

SLC25A11	0.461083603	8.830381588	1.05205E-09
PSME4	-0.317209129	9.997415688	1.05803E-09
SRBD1	0.227712318	7.712826024	1.06339E-09
OSBPL3	-0.184599374	6.463299042	1.06559E-09
RHOB	-0.566868522	8.726623803	1.07273E-09
S100A16	-0.461447455	8.506230447	1.08065E-09
OGFRL1	-0.144978227	6.35890324	1.08681E-09
CMAH	-0.228440009	6.715366634	1.09083E-09
SLC35F2	-0.212361457	6.245075496	1.0963E-09
ZNF839	0.245526153	6.819686714	1.10668E-09
HEY2	-0.929281652	9.306629852	1.11144E-09
KBTBD7	0.260730352	6.848035385	1.11356E-09
MKL2	0.292874242	7.240761865	1.12283E-09
TNFRSF10A	-0.224638535	6.228517782	1.12926E-09
RFWD2	-0.251401507	8.760175017	1.13011E-09
LIMCH1	0.450375422	11.2275501	1.13159E-09
MUSTN1	-0.8614957	8.061765408	1.15846E-09
AACS	-0.217000554	7.424180608	1.19676E-09
TTC3	0.279880735	9.165463947	1.20763E-09
PLEKHB1	0.420688416	7.321573519	1.20926E-09
PTPN21	0.276744287	6.920049297	1.20942E-09
KCNS3	0.282724066	6.915246771	1.2318E-09
NFE2L1	0.495551695	11.06870537	1.24399E-09
B4GALT1	-0.304188282	7.502994225	1.24526E-09
ADRM1	-0.2698532	9.23398014	1.24616E-09
TEX261	-0.239750063	7.392734204	1.24745E-09
ZNF641	0.256747925	7.249091874	1.25779E-09
TCTA	0.265617348	7.706230892	1.26619E-09
C9ORF10OS	0.249763934	7.630858698	1.28875E-09
OPTN	0.391570578	10.27644368	1.30378E-09
PFKFB4	-0.223217741	6.630868554	1.3227E-09
KIAA1128	0.342899284	9.871724097	1.32823E-09
ANP32C	0.316676199	6.634969215	1.3436E-09
PLCD3	0.308926684	7.04201924	1.36066E-09
HMGCL	0.315511099	8.099333804	1.36679E-09
DDO	0.297977738	7.026205434	1.36771E-09
MGC35361	0.201506666	6.826769557	1.37366E-09
SPIRE1	0.306494832	10.93304026	1.384E-09
DDX59	0.209904635	7.172996986	1.39706E-09
EFCAB7	0.183457662	6.517518115	1.40262E-09
VAMP1	0.155365217	6.539165844	1.41431E-09
KCTD12	-0.384575101	7.796885305	1.42145E-09
CCDC92	-0.300143478	10.46749137	1.4262E-09
FHOD3	0.493183434	11.5127532	1.43714E-09
TXNDC13	-0.359903916	8.873017249	1.45052E-09
GAS1	-0.472425227	7.824073776	1.45462E-09
PAICS	-0.299790253	8.402017711	1.46082E-09
THOC5	-0.26381421	8.40237591	1.46646E-09
GTF2H5	0.380193208	10.08892076	1.46649E-09
HSPA5	-0.526684737	8.983517261	1.48251E-09
CPT2	0.501505529	9.563445903	1.49024E-09

BMP4	0.295066502	6.941686009	1.49379E-09
GLIPR2	-0.510755713	9.06585033	1.49811E-09
PPIL1	-0.452519078	8.495629899	1.50803E-09
WTIP	0.317871206	7.69923744	1.51362E-09
FTSJ1	-0.204048435	6.794134635	1.5141E-09
MNDA	-0.220490163	6.201686295	1.51887E-09
CRELD2	-0.33522341	9.517278134	1.529E-09
TUB	0.259583649	6.854370514	1.53771E-09
FOSL1	-0.481212528	6.363154173	1.54172E-09
TFPI	-0.473501255	8.242614094	1.57426E-09
ZNF83	0.312204845	7.438345859	1.59123E-09
UBA3	0.474564191	8.92920842	1.60163E-09
C10ORF78	0.292083758	7.363426195	1.60325E-09
PSMB6	-0.224887877	12.24489518	1.63686E-09
FBXO4	0.135350621	6.554312764	1.65196E-09
ZNF471	0.206353077	7.002311038	1.66076E-09
MOBKL2A	-0.218697714	6.952069147	1.66226E-09
PFN4	0.234048282	6.65820285	1.66715E-09
DBNL	-0.300653255	9.000074198	1.68627E-09
IL1R1	-0.352978617	6.523920471	1.687E-09
RASL11A	-0.247233006	6.390341859	1.69052E-09
AGRN	0.279632927	6.815212037	1.69935E-09
FAM173B	0.256662522	7.244720496	1.70816E-09
MARCH5	-0.198650541	6.965300322	1.70868E-09
GNB1L	-0.28549757	6.528728539	1.71013E-09
DPYSL4	0.761521665	10.17730147	1.71694E-09
TUBB6	-0.596916425	8.732511062	1.72108E-09
FAM96B	-0.342198331	10.37782039	1.73343E-09
HTRA3	0.279483042	6.666858828	1.73362E-09
CADM1	0.458361892	9.023142047	1.73615E-09
MYH10	0.46197324	10.76845641	1.76042E-09
IRX5	0.558680123	8.943681785	1.76322E-09
MOBKL2C	-0.197312176	7.143386594	1.77923E-09
NDST1	-0.340271367	6.626765742	1.78286E-09
CA14	-0.41053023	6.443558487	1.81025E-09
CCDC45	0.245920052	8.276497141	1.84761E-09
TPSAB1	-0.446983485	6.813460508	1.85382E-09
ATG4D	-0.198460051	6.795893825	1.86211E-09
MYL6B	0.235033476	10.30678299	1.86217E-09
SEMA5A	0.369053921	7.849505748	1.8778E-09
BOLA2	-0.293000916	8.710964683	1.88333E-09
IPO11	0.28785471	9.400998971	1.88482E-09
MGC4172	0.28493414	8.395405058	1.91805E-09
EMR2	-0.313947697	6.483937652	1.93218E-09
LOC149448	0.368036778	8.693935185	1.9372E-09
STARD8	-0.396562256	7.213657824	1.95746E-09
FRMPD4	0.217892002	6.531361423	1.95941E-09
TAF6L	-0.284801395	8.124822058	1.9806E-09
ADAMTSL5	0.315475376	7.361814284	2.01617E-09
CSF1R	-0.559415261	8.966517262	2.04744E-09
ZDHHC8	-0.281377393	10.4227584	2.0565E-09

P2RY5	0.336394023	8.078600984	2.05706E-09
TRMT1	-0.292335459	7.647561128	2.09827E-09
DCAKD	0.321514926	9.74115349	2.11464E-09
OBSL1	0.292965304	6.591652496	2.12254E-09
PTPN12	-0.301770188	8.467573241	2.14057E-09
STX12	-0.27979788	8.152288565	2.14058E-09
POLR3F	0.233064118	7.23743178	2.14823E-09
DEGS1	-0.31852315	9.783825465	2.14898E-09
PAOX	-0.152713131	6.784651553	2.18637E-09
LOC91461	-0.476524034	7.811491974	2.18719E-09
NNT	0.439998086	9.898996338	2.22839E-09
SNORA64	-0.200712143	6.387094043	2.23663E-09
OGDHL	0.469526946	6.736733949	2.24352E-09
RAD51AP1	0.265113253	6.597710232	2.25882E-09
CLCN7	-0.267500124	10.30444892	2.26044E-09
ING3	-0.20384468	6.776002994	2.31067E-09
C20ORF46	-0.469526611	7.756309163	2.36528E-09
PRDX4	-0.306042868	9.997858012	2.37251E-09
CPSF4	-0.219085076	8.883999392	2.37894E-09
TESSP2	0.489355529	6.977696225	2.40212E-09
PPP2CB	-0.322423447	8.94655819	2.40816E-09
LOC651309	0.353164551	7.229531165	2.42606E-09
GAST	0.211845329	6.696315237	2.4558E-09
GHITM	0.267372281	13.4042496	2.46475E-09
RAB27A	-0.127842774	6.214459673	2.46536E-09
TRAF6	0.149195127	6.826343478	2.46553E-09
MRPS6	-0.389203493	10.87037079	2.49328E-09
LOC388681	0.462766729	9.755945295	2.49517E-09
ZNF204	0.386056695	6.742712416	2.5032E-09
ADPGK	-0.191448862	7.212957688	2.52328E-09
AASS	-0.287831868	7.430189886	2.57013E-09
SLC9A9	-0.251656107	6.577822516	2.58224E-09
ATF5	-0.32897945	7.640032303	2.60222E-09
SYT15	-0.197407493	6.952078109	2.60235E-09
RCE1	-0.18161941	6.59691227	2.60316E-09
RP5-1022P6.2	0.307171357	7.53902143	2.6163E-09
MLLT10	0.2877231	7.529529213	2.61677E-09
LTA4H	0.205934083	10.11311689	2.61832E-09
CORO6	0.396305164	9.123966272	2.66856E-09
TMEM88	-0.367753475	8.649750627	2.67347E-09
LOC92270	0.250789587	6.880205326	2.67673E-09
LACE1	0.188314776	6.501284445	2.67968E-09
UBE2H	0.481445099	8.429117494	2.68379E-09
C1ORF50	0.256596032	9.0526076	2.69156E-09
PIGP	0.338157012	9.311192298	2.69896E-09
PURA	0.254089374	7.569293997	2.70933E-09
TMEM154	-0.161714061	6.356200929	2.7309E-09
FRAS1	0.184844077	6.583318986	2.74388E-09
PPP2R5A	-0.311360303	9.104349223	2.77362E-09
RPUSD1	-0.253521771	7.034312688	2.79421E-09
FBXW11	0.263616619	8.757009073	2.82419E-09

SLC39A11	-0.217936616	6.71895405	2.82419E-09
SCYL1BP1	0.226427401	7.41790508	2.82434E-09
GADD45B	-0.690657941	7.438546057	2.84555E-09
PTEN	-0.285949722	8.097833809	2.85465E-09
TNFAIP6	-0.373862325	6.842021659	2.89742E-09
RAP1GAP	-0.464096101	7.3063338	2.94397E-09
SVEP1	0.500531354	8.910364599	2.97915E-09
ANKAR	0.133037584	6.287935999	2.98794E-09
COMMD8	0.352583576	8.106038607	3.00306E-09
ABCB4	0.269535992	6.848524035	3.02023E-09
GRINL1A	0.147243924	6.738242422	3.02287E-09
FKBP9L	0.278976203	9.382723599	3.03852E-09
VPS26B	0.307191569	8.683522525	3.08426E-09
FPR2	-0.135713518	6.048724694	3.09161E-09
ENPEP	-0.243451993	6.512099538	3.12219E-09
VPS52	0.236815969	7.997202735	3.1262E-09
TOP1MT	-0.155218762	6.376331634	3.14959E-09
RNF219	0.21156911	7.700946401	3.1685E-09
NCRNA00153	0.263755999	6.80984456	3.16947E-09
NEXN	0.324814754	11.76881529	3.17444E-09
GCNT2	0.331982491	7.343292473	3.1855E-09
TMEM158	-0.648090893	7.115744196	3.22638E-09
JMJD1A	0.317805059	7.665799056	3.23856E-09
ADPRHL1	0.574677573	10.30899723	3.24855E-09
PTGES2	-0.259667685	9.943570396	3.27839E-09
LOC653904	-0.183233743	6.222330439	3.28801E-09
CHIC2	-0.478928207	8.850015605	3.29795E-09
TMEM11	-0.282808048	7.391620527	3.29901E-09
FAM69B	0.2362855	7.637454181	3.31342E-09
GPR34	-0.191173364	6.249963565	3.3386E-09
TNC	-0.818372261	7.323277986	3.35491E-09
ABTB1	0.333401075	8.236085576	3.393E-09
ITGB3	-0.219408685	6.147819902	3.42187E-09
KIAA0672	-0.328522017	7.901291585	3.42494E-09
HTATIP2	-0.428816931	8.100294831	3.43009E-09
TTPAL	-0.228179258	6.880105538	3.44604E-09
GOPC	0.289531031	7.80571642	3.45666E-09
PKIG	0.358998078	7.142861124	3.457E-09
LOC440733	-0.273581402	13.25223996	3.46044E-09
FXYD5	-0.402592982	8.101720329	3.5126E-09
TLN2	0.422856713	8.488586989	3.5453E-09
RPL37	0.220307382	7.001628106	3.56401E-09
ARID5A	0.246323745	6.548915023	3.56602E-09
BRD7	0.208529807	8.577160051	3.56936E-09
C11ORF46	0.377534796	8.629643469	3.57332E-09
LYSMD4	0.236820578	7.6669111	3.57788E-09
SPHK1	-0.398597915	6.433299029	3.58164E-09
KAZALD1	0.377364667	7.254558117	3.64633E-09
ASNS	-0.547640193	7.75304932	3.66135E-09
FLJ40142	0.18349444	6.90337416	3.66614E-09
KIAA1160	0.205922701	8.043639293	3.699E-09

MRPL40	0.28646217	9.737437539	3.76371E-09
SYVN1	-0.270445851	7.878153159	3.77137E-09
CTPS2	0.184578327	7.175204413	3.79932E-09
MAGEE1	0.230729933	7.032035824	3.83429E-09
SBF1	-0.297893958	8.504227908	3.83785E-09
PKNOX2	-0.248861031	6.662477091	3.86641E-09
HNRPUL1	-0.245227492	9.407279577	3.88603E-09
CLCF1	-0.523634972	6.614535414	3.89515E-09
HLA-B	-0.41468141	10.25664859	3.933E-09
FLJ14213	0.260918087	6.565613163	3.93758E-09
TGFA	-0.157838307	6.160710821	3.9405E-09
ZNF684	0.388593053	7.513848832	3.95716E-09
PIP5K1C	-0.316934088	8.415813596	3.99676E-09
ZFPM2	0.299922176	7.602427851	4.00633E-09
ALPK1	-0.244831084	6.480003101	4.02934E-09
ALDH16A1	-0.21070966	6.612037257	4.09478E-09
RAB15	-0.637967909	7.350469852	4.10796E-09
SMPDL3A	-0.308036399	6.909614708	4.16122E-09
NDC80	0.169636885	6.331140977	4.22067E-09
INTS2	0.360924423	7.876343111	4.23246E-09
UCHL1	0.840837896	7.439063275	4.24311E-09
LOC652002	0.372062647	7.123970586	4.25111E-09
TMEM147	-0.266426364	10.59958727	4.25654E-09
THOP1	-0.3095927	7.914667594	4.28799E-09
C17ORF61	0.295677008	11.12713461	4.29253E-09
PSMD8	-0.344003285	10.3102996	4.34629E-09
THOC4	-0.310446775	7.597430993	4.34753E-09
PAK1	0.381795335	6.959315271	4.36361E-09
PDE6B	0.204265889	6.261461003	4.3979E-09
RAG1AP1	-0.290130217	7.143570585	4.45513E-09
LRIG3	0.178740082	6.401321101	4.47776E-09
ACAT1	0.343394584	12.84478122	4.48642E-09
LOC643961	0.345846046	6.341521813	4.50301E-09
LAMA5	-0.346380722	10.79394675	4.61637E-09
TNFAIP2	-0.270722455	6.53332786	4.62652E-09
SF3B1	0.161541457	8.389924925	4.71404E-09
STK40	-0.378039293	9.12494096	4.72576E-09
FLJ10081	0.255279518	8.919897617	4.73767E-09
PRKX	-0.198337833	6.631605521	4.7647E-09
PBX1	0.234006752	7.76045098	4.78124E-09
NOV	0.465945645	7.391217104	4.79324E-09
RALB	-0.295057991	10.22452025	4.86695E-09
SRGAP1	-0.363093109	6.833959574	4.87494E-09
TXNDC12	0.266978302	9.881152057	4.87974E-09
RABL4	0.309634546	8.140903129	4.90622E-09
CARM1	-0.33548982	9.790279902	4.90897E-09
EHD1	-0.315117719	10.16916179	4.96898E-09
CLASP2	0.422241805	7.999994523	4.98104E-09
TADA1L	0.249546373	7.559300273	5.02636E-09
NANS	-0.318430986	7.923307476	5.05287E-09
LOC652164	0.258973622	6.521240928	5.06398E-09

BHLHB3	0.311089175	7.319128264	5.10504E-09
IFIT1	0.620947135	8.338211054	5.12888E-09
ACAD10	0.374818435	8.040753275	5.13021E-09
MTMR4	0.403800411	8.399415737	5.14992E-09
FAT	0.317348576	9.631418555	5.17359E-09
PPFIA4	0.454076478	8.254178763	5.23767E-09
ZMIZ2	-0.184452184	7.080301919	5.25553E-09
DHRS7	-0.328072787	9.780674132	5.43728E-09
BSDC1	-0.404886411	7.816326445	5.44715E-09
JMJD1B	0.313748764	7.89804726	5.59326E-09
PTPRR	0.184179281	6.523155498	5.59843E-09
PDIK1L	0.223125774	7.186087071	5.62862E-09
MTMR2	0.156512053	7.088641548	5.65338E-09
POLE4	-0.356777072	10.34557933	5.66598E-09
STX19	0.224125545	6.552228257	5.6903E-09
RAD17	0.118526401	6.50124835	5.7045E-09
C14ORF133	0.250153107	7.399279356	5.72915E-09
LOC440503	-0.221989305	6.259172434	5.77776E-09
EMP1	-0.554425906	9.886848515	5.7842E-09
RAGE	-0.278774588	8.848779594	5.79264E-09
NPHP4	-0.144904225	6.359960711	5.85815E-09
ABCG1	-0.226326105	6.568833934	5.86568E-09
MXI1	0.182515406	6.78050242	5.86965E-09
DBP	0.416793824	6.934588169	5.87169E-09
ATP1B4	0.350127491	6.319106116	5.91499E-09
IFNAR2	-0.157241164	6.872261709	6.08548E-09
PPP1CA	-0.283628917	8.525296401	6.09223E-09
NOL8	0.206984629	7.71347862	6.15019E-09
NAP5	0.202990206	6.35975655	6.2844E-09
COL9A1	0.198401992	6.256308448	6.30972E-09
PTPN4	0.2620251	8.055953892	6.31198E-09
RFC1	0.195548103	7.934925256	6.33219E-09
BMP1	-0.126510828	6.351035336	6.39808E-09
SDCBP	-0.443408187	8.941977479	6.40254E-09
KIAA0889	-0.176802917	6.486173324	6.42917E-09
FCGR2B	-0.188048448	6.173907307	6.43975E-09
C15ORF17	-0.340933562	8.805012735	6.4611E-09
SLC36A1	-0.268735158	6.493131854	6.51573E-09
PSD4	-0.159968618	6.139671543	6.52072E-09
REPS2	-0.128338242	6.228695065	6.52224E-09
SH3RF1	-0.303585427	7.186014703	6.6517E-09
FKBP4	-0.322347343	7.686721092	6.65809E-09
C1ORF213	0.137535283	6.248072189	6.68075E-09
CD55	-0.490589173	9.489743872	6.85514E-09
SLC9A1	-0.422150712	9.001254355	6.90858E-09
CASD1	0.279033005	7.220975108	6.93557E-09
TMEFF2	-0.280667894	6.080637496	7.00516E-09
C20ORF200	0.288978467	6.604852541	7.0148E-09
RAB35	-0.334886394	7.68299042	7.03221E-09
HEATR1	-0.356351198	8.05165169	7.03326E-09
AGL	0.299144851	8.088095995	7.08585E-09

WDR74	-0.2664461	7.897679755	7.14072E-09
RAB26	0.223243378	6.555795752	7.22014E-09
LAD1	-0.619216879	6.755271794	7.31535E-09
SLC4A2	-0.281815874	7.295212431	7.43141E-09
GTF2E2	-0.346997943	9.420820548	7.43695E-09
WDR51A	-0.215886506	6.737522755	7.46073E-09
ZCCHC9	0.305043594	8.40613721	7.5219E-09
SYNM	0.361513618	10.69682302	7.59866E-09
MRPL10	0.262271269	7.86738364	7.68189E-09
LOC644615	-0.379627999	7.481384343	7.68209E-09
YWHAZ	-0.339344882	10.23777871	7.69027E-09
GRWD1	-0.250247299	8.077055081	7.78049E-09
EXOC7	-0.219241464	8.101437133	7.83721E-09
RNASET2	-0.305305203	9.917180422	7.85334E-09
HNRPH3	0.329121054	8.189990878	7.87665E-09
ARFGAP3	-0.295730685	7.242950242	7.94223E-09
DUS3L	-0.299202729	7.47587027	7.9596E-09
RRS1	-0.32379906	7.793701543	7.97505E-09
SDCCAG10	0.230167548	9.603748527	8.01108E-09
FAM43B	-0.354185816	7.008637246	8.04566E-09
ICA1L	0.123275029	6.297293285	8.17903E-09
NLRX1	0.288502527	8.184655056	8.22025E-09
LOC646168	0.276903111	6.564987701	8.22613E-09
ZCWPW2	0.182199444	6.67890881	8.31194E-09
COMP	1.09908573	7.09204958	8.49124E-09
AEBP1	0.871540912	8.631734564	8.65583E-09
AARS	-0.269374181	10.95709071	8.70171E-09
ZCRB1	0.290767824	7.099815491	8.76515E-09
SYTL2	0.354064234	7.017330972	8.7971E-09
TNNI3	0.338425229	13.77489635	8.84187E-09
HSD17B10	-0.246650431	8.344942641	8.86179E-09
FAM70B	-0.127731899	6.406465724	8.87919E-09
ARNTL	-0.283343733	6.671585278	8.91209E-09
LIPT1	0.237623288	7.314130888	8.98133E-09
MED12	0.218500056	7.26809627	9.0045E-09
GON4L	0.155045841	7.120754082	9.01944E-09
CARKD	0.272210983	8.829851501	9.13783E-09
AMY1C	-0.601542267	10.99836613	9.23323E-09
STAG3L4	0.39046222	7.785208685	9.28029E-09
ANAPC10	0.25832568	8.100431783	9.29108E-09
REST	0.130673686	6.338517674	9.33392E-09
ELF3	-0.396940223	6.131540384	9.40024E-09
ABHD5	-0.490190389	7.570944914	9.47767E-09
NEK6	-0.210772237	6.307335942	9.48657E-09
RYR3	0.202238708	6.230223683	9.53046E-09
MIER2	-0.224355936	6.509080321	9.53807E-09
SLC6A6	-0.403218114	6.434037032	9.62617E-09
PPARGC1B	0.42885292	7.665754108	9.69869E-09
PTGER4	0.40663483	8.214464398	9.79078E-09
SLC30A9	0.272293921	10.07444184	9.8586E-09
UACA	0.131985932	6.465195817	1.00381E-08

TMEM205	-0.265647969	11.16032424	1.00384E-08
LGALS9	-0.213329121	6.588529855	1.00445E-08
PEX7	0.445000812	8.42235078	1.01895E-08
ERCC6	0.183785337	6.393446154	1.02036E-08
CC2D2A	0.219217529	6.596320347	1.02253E-08
C3ORF1	0.322140814	8.269294077	1.02996E-08
GNL2	-0.408120535	9.808041376	1.03841E-08
PTPN3	0.713848668	8.318202996	1.03937E-08
C10ORF104	0.427136442	9.062901465	1.03963E-08
TARBP1	0.230689753	7.197827285	1.04265E-08
MTHFD1L	-0.391112754	6.970973857	1.04643E-08
FAM108C1	0.369976766	8.437679339	1.08146E-08
F8	-0.208754873	6.786436542	1.09144E-08
SASH3	-0.206192962	6.439757229	1.09832E-08
PRCC	-0.150681676	6.952449573	1.13755E-08
PDZD4	0.157034326	6.306135019	1.14155E-08
OR51E1	0.30978261	6.781336114	1.14946E-08
NACA	0.177161875	12.67279931	1.14947E-08
RAB32	-0.409115768	7.664178458	1.1508E-08
ASRGL1	-0.194052519	6.046578133	1.15286E-08
ST6GALNAC4	0.268262937	7.642739637	1.176E-08
MGAT1	-0.30880272	9.428390517	1.17992E-08
INADL	0.303901604	7.206028145	1.18332E-08
SOX11	-0.312804134	6.260757396	1.19491E-08
CRNKL1	0.180708468	6.774658246	1.19788E-08
CIRH1A	-0.364617628	8.776903973	1.20736E-08
ARHGEF18	-0.27650352	8.71734172	1.22237E-08
LPHN3	0.253465747	6.459060397	1.22533E-08
FAM73B	0.273038487	7.973934055	1.22934E-08
IMPACT	0.150602111	6.342754961	1.23497E-08
SCYL3	0.141418068	6.7948271	1.24429E-08
C6ORF125	0.322789251	11.52096408	1.24509E-08
ATP6V0A1	-0.369274803	9.296281528	1.24963E-08
C20ORF166	0.35646	6.967859996	1.25027E-08
TBC1D7	0.386896966	9.124701768	1.25726E-08
PLCG1	-0.279494758	7.277411876	1.26193E-08
YPEL3	0.422825854	8.931468753	1.26852E-08
LOC285359	0.255089752	6.455685246	1.27126E-08
ZC3H14	0.101125732	6.559681429	1.27238E-08
PI4KAP1	-0.246114893	6.829157741	1.27705E-08
PAK2	-0.267509196	7.753415734	1.27745E-08
KLHDC2	0.442603516	10.86429836	1.2913E-08
CMTM6	-0.246355521	7.587614784	1.29528E-08
STK38	-0.359394634	8.491771356	1.2976E-08
WFDC1	-0.235032031	6.252592986	1.30756E-08
CHMP4B	-0.310319515	9.771756017	1.30849E-08
NRAS	-0.19878341	7.737591594	1.31032E-08
ALS2CR8	0.167426884	6.272647144	1.33167E-08
FAM179B	0.459436517	8.091054324	1.3531E-08
AKR1A1	-0.20078389	8.738479165	1.36117E-08
OPA1	0.25884281	8.739281836	1.36362E-08

PFKFB2	0.502773406	7.803338273	1.39277E-08
LOC643319	-0.362661516	6.605533298	1.4008E-08
CROP	0.39097729	8.388044886	1.40413E-08
DIP2B	0.239127685	8.733906226	1.41648E-08
TMEM116	0.446889781	9.064795084	1.41774E-08
RHEB	-0.308725102	10.53847855	1.42284E-08
PRKCSH	-0.191207622	7.556182972	1.44119E-08
MPHOSPH9	0.174821483	6.981878173	1.44889E-08
FAM50A	-0.32010303	10.55767525	1.4643E-08
MAP4K4	0.228956932	8.083476169	1.46467E-08
FLJ45244	0.260302869	7.234763879	1.47011E-08
PTTG1IP	-0.226995888	11.27943575	1.47594E-08
C17ORF44	0.193960932	6.53011507	1.49529E-08
GMPPA	-0.248907171	7.414168875	1.49921E-08
RRAS	0.3716811	11.02935291	1.50195E-08
TMTC1	-0.306074829	7.307609904	1.50725E-08
AMD1	-0.461331385	9.415940072	1.51342E-08
IGSF21	-0.179754969	6.238881423	1.51662E-08
IL10RB	-0.255719465	8.455675117	1.51916E-08
CD93	-0.474587902	10.34022303	1.5205E-08
PDSS1	-0.221938558	7.009857244	1.52524E-08
ENG	-0.383924478	9.663527425	1.52675E-08
PDZRN3	0.314757038	7.121693523	1.53009E-08
S100A6	0.40069368	11.79649005	1.53615E-08
MYL7	0.668343616	13.38597689	1.53817E-08
C11ORF17	0.239564155	8.259293883	1.53955E-08
MMP23B	0.53809875	9.414621483	1.55558E-08
OSBPL9	0.263887749	8.808801069	1.56849E-08
SGPP2	-0.495451919	6.707885144	1.57378E-08
VRK1	0.189630779	6.838860285	1.58324E-08
EIF6	-0.334223969	7.881635626	1.58732E-08
ATF4	-0.3968728	10.60341052	1.5919E-08
LOC653377	0.311516482	10.50546122	1.6087E-08
NFE2	-0.190730105	6.195090839	1.61001E-08
FNDC4	-0.191979204	6.399640976	1.61047E-08
APOM	0.18913251	6.68163165	1.62318E-08
PLEKHG3	0.26557909	7.569579778	1.64759E-08
SLC38A2	-0.660521721	9.375680205	1.65439E-08
AP2M1	0.254764107	9.874818116	1.65489E-08
HSZFP36	0.231697526	7.366158952	1.6584E-08
LZTS1	-0.236942126	6.605910905	1.66428E-08
WDR62	0.50576087	7.525594342	1.67266E-08
CLNS1A	0.26898904	9.720255251	1.67931E-08
CCNG2	0.242263148	6.649882977	1.68678E-08
AP3S2	0.201423361	7.462657356	1.69259E-08
FGF7	-0.233793034	6.199723511	1.70083E-08
AMT	0.492055166	8.702265025	1.70318E-08
ST8SIA4	0.135590391	6.338110279	1.71144E-08
PREX1	-0.20965794	6.337838531	1.72177E-08
FZD9	-0.311189973	6.232901967	1.74671E-08
LRRN3	-0.361226408	6.49784418	1.77012E-08

TRIM52	0.211656226	6.804795546	1.78963E-08
MGRN1	-0.295202984	7.312434035	1.82039E-08
THBS1	-1.040279616	9.126107567	1.82903E-08
TMEM55A	0.402982624	8.394832881	1.82993E-08
C12ORF26	0.301340086	8.04874861	1.84884E-08
OSGEP	-0.259716153	7.894305722	1.8679E-08
GCLC	0.239881489	7.268194295	1.868E-08
LOC648470	0.355736218	7.146398701	1.89456E-08
PRR13	-0.275855797	10.50891333	1.89573E-08
CLK1	0.289764875	8.640426867	1.92163E-08
PRPF4	-0.282512603	8.512149535	1.92944E-08
TSPAN9	0.372923556	12.9859479	1.93641E-08
EFTUD1	-0.216814914	7.099294526	1.95109E-08
HINT3	0.162205958	6.603661087	1.95907E-08
GOLPH3	-0.266706402	9.013095655	1.98072E-08
C7ORF23	-0.252485627	7.441216056	1.98254E-08
ST3GAL3	0.202500665	7.639191485	1.99011E-08
LOC338758	0.254706593	7.16278631	2.00347E-08
ABCC8	0.388952714	6.945226336	2.00797E-08
LOC731895	0.180151592	6.325590684	2.02484E-08
LST1	-0.15421521	6.41225686	2.03603E-08
FSD2	0.841948403	10.11375411	2.0364E-08
ABCE1	-0.290285618	8.025198539	2.04704E-08
BCAS4	0.201677127	6.861457354	2.06225E-08
ABCB6	-0.239269406	7.704310591	2.06362E-08
PDPN	-0.378148386	7.208899324	2.07362E-08
ACOX1	0.206487914	6.854355236	2.09097E-08
BP75	0.234701055	7.086197488	2.1076E-08
C15ORF58	0.185482238	6.490685921	2.11178E-08
GBAS	0.400302469	10.67480589	2.13921E-08
CITED2	-0.452967598	10.05457322	2.14066E-08
LOC647108	-0.181759398	6.337606027	2.14444E-08
YTHDF1	-0.227696058	8.836696983	2.14737E-08
ALDH7A1	0.344329443	9.346291563	2.14939E-08
TMEM115	-0.238729045	8.567086502	2.16179E-08
MT3	-0.396659281	6.882211226	2.19676E-08
UFSP2	0.309766038	9.138935215	2.19915E-08
ZNF426	0.201237931	7.614402984	2.20208E-08
GPT	-0.426476377	8.887567184	2.20459E-08
PCDH1	-0.111832039	6.178456194	2.21273E-08
ZNF350	0.172875459	6.772634881	2.23583E-08
HAS2	-0.086274359	6.12782981	2.24193E-08
DDIT4L	0.247630023	6.637482288	2.24792E-08
FREM1	0.342312	6.412470097	2.25083E-08
PANK1	0.337609615	7.767342827	2.27642E-08
C16ORF35	-0.195613415	7.54090671	2.27781E-08
CYGB	-0.367164494	8.523635803	2.30773E-08
ZNF512	0.260569719	8.471321483	2.32296E-08
C17ORF53	-0.223596227	6.533509891	2.34699E-08
CCDC55	0.268567887	7.799526463	2.34728E-08
BMPR1A	0.208951124	7.461789556	2.44163E-08

MAFF	-0.269374546	6.811549763	2.44762E-08
DUSP5	-0.673559166	8.211783084	2.49241E-08
PLOD3	-0.308163076	9.281623839	2.50212E-08
GLIS3	-0.107297788	6.281457769	2.50866E-08
EVL	0.339243806	10.72497428	2.53114E-08
TNNT3	0.547380573	7.789764173	2.55805E-08
VAMP5	-0.373083527	9.942266309	2.56028E-08
ARPC1A	0.258979227	10.58503957	2.56554E-08
MRPL45	0.216086173	9.064072021	2.58616E-08
C9ORF9	0.323143468	7.270882559	2.59627E-08
VNN2	-0.147992413	6.256427367	2.6055E-08
FLOT2	-0.320779372	10.97956641	2.60562E-08
SYT11	0.425119436	9.941511182	2.61716E-08
PYCR2	0.22697284	8.960131711	2.64436E-08
LOC644310	0.391752041	9.063675221	2.66828E-08
NGF	-0.376598745	7.293557275	2.67292E-08
DHRSX	0.233283211	6.887094691	2.67351E-08
DDX21	-0.452802342	8.410023392	2.69724E-08
LOC388588	0.525416659	9.175413767	2.69748E-08
VIP	0.221273165	6.710649571	2.71231E-08
DNAJB11	-0.414554855	10.2972247	2.71453E-08
PLEKHA2	0.310136454	8.384431563	2.71849E-08
KBTBD4	0.17352973	6.996079404	2.71854E-08
ZXDA	0.07972566	6.194722714	2.7266E-08
HOXB1	-0.178723959	6.313901387	2.73073E-08
ABI3	-0.242590072	6.756676406	2.77554E-08
ROGDI	0.338938335	8.357201936	2.77781E-08
HNRPM	-0.232071284	9.961533523	2.79466E-08
IQWD1	0.250629998	10.32229851	2.83259E-08
GPR44	0.312276482	6.743704903	2.83904E-08
TNFSF10	0.489468928	8.343782254	2.84862E-08
STX2	-0.336479473	7.779468215	2.85199E-08
HDAC3	0.175386063	8.534348113	2.85437E-08
NTN4	0.356605017	7.997194233	2.85626E-08
DUS4L	0.29471768	8.129698292	2.85817E-08
LOC93622	0.23272375	7.677330054	2.87025E-08
ZNF550	0.148275308	6.810542122	2.87726E-08
ARMCX6	0.229410443	7.667354262	2.88967E-08
UPP1	-0.290492882	7.766695586	2.90102E-08
HAGH	0.294104472	10.81902286	2.92477E-08
DYRK2	0.316906674	8.177290817	2.92708E-08
ALS2CR4	0.302430623	8.505433327	2.95182E-08
GPRIN3	0.218715673	6.671585496	2.95231E-08
FLJ10213	-0.246649271	7.121228587	2.96896E-08
EFEMP1	-0.538833655	7.862396228	2.98934E-08
CDKN2C	0.28448575	7.007721405	2.99553E-08
ZNF605	0.218049017	6.95243805	3.01037E-08
ZNF493	0.161023234	6.630951454	3.02553E-08
PTPN6	-0.132999956	6.280014892	3.0592E-08
ALG9	-0.31748777	7.721767156	3.06763E-08
SEPW1	0.288782408	11.9299042	3.07368E-08

DNAJC24	0.307175861	7.835247125	3.21788E-08
C1ORF190	0.279252205	7.2155066	3.24481E-08
CTRC	-0.175742643	6.965272424	3.25108E-08
CHRD	-0.185319761	6.594635649	3.28266E-08
PEX16	-0.20181573	7.573340783	3.28844E-08
HMG20B	-0.262298912	9.188296041	3.30174E-08
SLC25A30	0.268067547	6.812841228	3.31158E-08
ZNF133	0.18487082	7.114658624	3.33783E-08
PLA2G7	-0.162280249	6.078486712	3.41049E-08
PERP	0.565257775	8.103203888	3.4413E-08
SATB1	0.230388776	6.682377363	3.44263E-08
BCL2	0.369822599	7.285107471	3.46538E-08
PCDHGB4	-0.172545666	6.49766034	3.51449E-08
TUBB2B	-0.368678214	6.993724173	3.52856E-08
RNF150	0.391294644	10.45502061	3.5754E-08
KBTBD5	-0.693082666	6.4061514	3.5898E-08
PHF21A	0.253477852	9.663837165	3.60625E-08
CP110	0.238642723	8.321108823	3.62986E-08
EDA2R	0.158036732	6.137200089	3.64678E-08
CNOT6	-0.204851111	7.650044965	3.65454E-08
KRTCAP2	-0.24839952	10.93741548	3.66144E-08
ZNF385D	-0.333771033	7.012127319	3.66489E-08
LEPREL1	0.980876795	8.583054668	3.67858E-08
RBM16	0.201780425	7.4304962	3.69464E-08
DGAT1	-0.220150384	7.653651028	3.71874E-08
KCTD15	-0.181469218	6.765885564	3.72496E-08
PDE1A	0.542775291	7.898534149	3.73793E-08
KLHL21	0.379930592	8.169527132	3.7524E-08
PPAN	-0.279932409	6.914151262	3.76522E-08
CRADD	0.342261229	9.937357697	3.79273E-08
PDIA6	-0.364181917	8.147787442	3.80402E-08
RALGDS	-0.479110295	8.989380637	3.83813E-08
FLVCR2	-0.270119019	6.861771962	3.84847E-08
ROD1	-0.276654513	8.810959005	3.85292E-08
DDX56	-0.243410092	8.302090795	3.88052E-08
LOC400214	0.178868913	6.408931538	3.88516E-08
ZNF701	0.196689463	6.572037164	3.88746E-08
SLC25A38	0.276462781	7.515363196	3.90485E-08
C20ORF54	-0.189868516	6.424022532	3.91548E-08
KRBA2	-0.269740315	6.495058029	3.93077E-08
ACSL5	-0.18103126	6.812384951	3.94539E-08
RBMS1	-0.237790673	8.274215042	4.00708E-08
NT5C3	0.252003007	8.600337584	4.01277E-08
LACTB	-0.28157423	8.205912831	4.03624E-08
PHKB	0.218674464	8.340932503	4.08499E-08
PTPRU	-0.228155618	6.276012493	4.14968E-08
TBC1D2	-0.183789525	6.467393364	4.16684E-08
SOBP	0.210807456	7.464802554	4.17335E-08
IL20RA	-0.197648418	6.355189463	4.17566E-08
NOS3	-0.492449966	7.881295983	4.17811E-08
SEMA3F	-0.281042282	6.777844097	4.19569E-08

RGS16	-0.177744556	6.270133101	4.25166E-08
SH3PXD2B	-0.277804281	6.397640339	4.26243E-08
IFT122	-0.194693013	6.795109411	4.26907E-08
HIF1A	-0.31157114	7.339507798	4.28267E-08
ARID4A	0.150485656	6.795135722	4.29369E-08
RNF14	0.234329509	9.270219424	4.29962E-08
UBE2L6	-0.38079985	8.328810469	4.31019E-08
MYOCD	0.194891901	6.560580592	4.31632E-08
CBY1	0.275138277	8.095368649	4.34857E-08
KBTBD10	-0.402678492	10.45685799	4.36829E-08
LOC651302	0.291977676	7.091637339	4.38519E-08
NCAPD3	0.227864894	6.870135782	4.44194E-08
NIPSNAP1	0.323748566	7.890292275	4.44715E-08
SLCO5A1	-0.370436942	6.850697633	4.48407E-08
RPS6KA1	-0.159912352	6.204395915	4.50585E-08
ECE2	-0.199354286	6.201583532	4.58513E-08
TPR	0.404325228	8.619877884	4.59445E-08
DNAJC18	0.169298753	6.915549532	4.61186E-08
SRP14P1	0.216551775	11.43625262	4.65549E-08
B3GALTL	0.468746598	8.26758341	4.67315E-08
MED22	-0.245558004	8.54236567	4.69822E-08
CIP29	0.203848744	10.9700213	4.7693E-08
PTGES	-0.231874197	6.208359738	4.78765E-08
SLC22A4	-0.293092367	6.529073602	4.93216E-08
LOC150763	-0.120781957	6.531528788	4.9665E-08
SMAD9	0.220380953	6.420393619	4.99329E-08
JAZF1	0.311908677	8.800456823	5.00879E-08
TXLNB	0.483479732	7.014502681	5.03454E-08
TATDN3	0.264710766	8.465550471	5.17338E-08
NECAB1	0.182605286	6.399993296	5.1738E-08
DENND2D	-0.177821468	6.256914708	5.18896E-08
POLR3D	-0.22729894	6.493877138	5.25292E-08
YIF1B	-0.21846422	7.660045685	5.30395E-08
GUCY1A2	-0.170077559	6.295250967	5.35655E-08
TM4SF18	-0.364375576	7.650543817	5.36095E-08
COL23A1	0.422241056	7.943878727	5.37445E-08
NOSTRIN	-0.310666857	7.724843864	5.3798E-08
TNNI3K	0.644932394	9.809525796	5.381E-08
GPHN	0.21311012	7.484286291	5.38294E-08
PRKCZ	0.192267697	6.974040789	5.39751E-08
MPL	0.270393102	6.866260472	5.42593E-08
UBE2Z	-0.171036313	6.847837397	5.45595E-08
TDRD6	0.279448212	6.962157693	5.4613E-08
CYP11A1	0.171088643	6.733390777	5.53592E-08
SRRM1L	0.221735338	7.415343024	5.5361E-08
HERC1	0.256746121	8.960608854	5.54574E-08
SLC7A8	-0.131919292	6.338027672	5.63047E-08
LOC650737	0.292835783	7.241567125	5.66211E-08
TNIP1	0.26132199	9.094879346	5.69536E-08
GPA33	0.199841272	6.601548019	5.70697E-08
CACNB3	-0.250996435	7.161609148	5.71552E-08

DSC1	0.528857319	7.228857185	5.71675E-08
SRR	0.200463763	6.63903189	5.8325E-08
KATNB1	-0.226397626	7.654851445	5.86974E-08
GYLTL1B	0.19996595	6.398071007	5.87293E-08
ACOT7	-0.231764398	6.881886383	5.92593E-08
FIS1	0.283957986	10.7767839	5.96735E-08
VPS29	-0.275673649	10.70096543	5.99535E-08
TLR8	-0.126181548	6.094878135	6.0046E-08
SPATA18	0.215523367	6.490043782	6.04322E-08
KIAA1009	0.154504233	6.64992299	6.06326E-08
RORC	0.2603172	6.606729991	6.06518E-08
CABC1	-0.43316483	10.99827564	6.10341E-08
PDE4B	-0.247454023	6.432086695	6.11038E-08
SCRIB	-0.158566305	7.725226523	6.12929E-08
MARK1	0.211288414	7.826153893	6.14179E-08
JTV1	-0.228964274	10.513919	6.21333E-08
CSPG4	-0.333297008	7.512769573	6.2452E-08
M6PR	-0.249577367	8.935034858	6.29959E-08
BEX5	-0.194641172	6.25904453	6.3021E-08
FLJ20125	0.275530081	7.22276383	6.34422E-08
LOC285074	0.257981643	7.931241234	6.34845E-08
P704P	-0.315387408	6.504862881	6.40795E-08
C10ORF57	0.202844153	7.264191012	6.43678E-08
ARHGDIB	-0.359576016	11.37401789	6.45796E-08
FECH	0.278793646	7.381137355	6.46585E-08
METTL1	-0.248340548	6.410066626	6.49946E-08
RNH1	-0.261537873	8.888694208	6.57791E-08
GRM8	0.245919589	6.532326225	6.67722E-08
DNMT1	-0.324507376	9.455923357	6.68543E-08
SFRS5	-0.370361486	10.51401441	6.77206E-08
SH2B1	0.244685718	7.230098358	6.85161E-08
SLC4A7	-0.253345793	7.782857153	6.85245E-08
PRICKLE2	0.296320405	8.334706593	6.86012E-08
ADA	-0.306086028	7.63597039	6.89551E-08
BRF2	0.214872122	7.920081251	6.94084E-08
HLA-C	-0.272017901	6.639397437	7.0145E-08
CCL21	-0.479832101	6.721761092	7.02466E-08
IDS	0.188522543	8.57607349	7.03589E-08
SUV39H1	-0.257981294	7.505014709	7.06588E-08
TAGLN2	-0.471562085	8.833417904	7.0836E-08
FLJ10246	-0.152781742	6.182568048	7.10224E-08
UQCR	0.222995195	7.505417631	7.12774E-08
WDR66	0.189612648	6.291324856	7.13681E-08
MCOLN1	-0.279727077	7.868730355	7.18357E-08
DHX15	-0.298565522	10.06775175	7.19655E-08
LOC643452	0.170297154	7.315025099	7.19952E-08
PIP4K2B	0.172226456	6.663159441	7.22472E-08
ASB5	0.309477567	7.053747621	7.22963E-08
GPR146	0.20613909	6.852923067	7.23733E-08
CCDC90A	0.319415281	9.698303676	7.2606E-08
CAMK2A	0.343174187	7.205800551	7.381E-08

MRPL46	0.288570681	10.53471053	7.49012E-08
GCLM	-0.245189819	7.101441456	7.52164E-08
SLAMF1	-0.188097281	6.046580036	7.56493E-08
CEBPG	-0.309890022	8.318543103	7.60768E-08
SHPK	-0.242311268	7.268956355	7.61583E-08
SNORD68	-0.227080605	6.740760476	7.7077E-08
COPE	-0.197507852	9.12976347	7.71648E-08
OAF	-0.393010768	8.653212716	7.72583E-08
AP4B1	0.208948652	8.365190961	7.74374E-08
TSPAN17	-0.258571257	9.193797418	7.7839E-08
XPR1	0.426067086	9.234009239	7.79173E-08
ABCF1	-0.263597043	9.141698642	7.79489E-08
MAPK9	-0.202700209	7.203951017	7.89799E-08
MRPS27	0.226169976	9.567227026	7.9264E-08
ZFR	0.325980217	9.618779384	7.92822E-08
ZNF442	0.156874098	6.651013361	7.99698E-08
PPP1R3D	0.223760147	7.306540032	8.02745E-08
CCDC115	0.247570512	8.18155603	8.05033E-08
SEC24D	-0.304275636	6.92655012	8.0531E-08
HDGF	-0.218695745	9.011761425	8.07141E-08
ZNF197	0.133203978	6.608164206	8.12339E-08
SUGT1	0.262734055	8.127414601	8.16986E-08
FOSL2	-0.1698531	6.415563665	8.21507E-08
CKAP2	0.282466474	6.946502224	8.23985E-08
STARD13	-0.157849716	7.152542807	8.25313E-08
PECI	0.270235682	10.26445596	8.36302E-08
ZNF717	0.165518314	6.324420215	8.40347E-08
RALGPS1	0.316793063	7.863983058	8.41389E-08
COMMD10	0.341020591	8.798731405	8.42613E-08
FAM122B	0.191024777	7.513781656	8.46618E-08
NUDT5	-0.308498103	7.445031266	8.5089E-08
GRINA	-0.251958595	8.478679061	8.53553E-08
ASCC2	-0.394135611	10.02577035	8.55863E-08
S100P	-0.213581387	5.978845586	8.56821E-08
CFH	0.560491762	7.826122687	8.58384E-08
MAOB	0.444923396	9.029913417	8.59336E-08
TRIM23	0.226560125	6.879860475	8.64352E-08
MSN	-0.477787486	10.53419217	8.68718E-08
TTC23	0.163532158	7.246882808	8.71409E-08
LOC731950	0.137019006	6.740416061	8.72326E-08
C5ORF30	0.180551347	6.715538851	8.74888E-08
NPTXR	0.160718058	6.619573992	8.79467E-08
RPL34	-0.3163904	7.958143498	8.81896E-08
ZFP36L2	-0.292343434	7.301716722	8.82584E-08
COMMD6	0.282211405	10.5395371	8.85386E-08
SLC16A5	0.339899087	7.694687943	8.8749E-08
CCDC141	0.519782999	8.09003913	8.98493E-08
PYGB	0.480330836	10.5061492	9.03319E-08
SRPX	-0.382743365	10.5329874	9.04675E-08
LOC400027	0.273172181	9.924733337	9.15007E-08
COPS4	0.300462382	9.680312016	9.17371E-08

RRP12	-0.567071994	7.801004994	9.19166E-08
ABHD10	0.351636906	8.139280875	9.20772E-08
PPP1R1C	0.445355213	7.510178268	9.29458E-08
VASN	-0.393782918	8.229273548	9.32877E-08
POGK	-0.21937337	8.777916412	9.36306E-08
LOC728452	-0.217988083	6.427497742	9.44206E-08
SMARCA2	0.345717131	9.229934746	9.51546E-08
ENOX2	0.154515237	6.894262496	9.59471E-08
ZNF10	0.204616715	7.10565574	9.62784E-08
PRO0628	-0.270810382	6.498419396	9.67569E-08
LOC649049	-0.389876887	11.27588904	9.70096E-08
STK3	-0.366832815	8.629635182	9.76211E-08
SLC8A1	0.176216262	6.645989578	9.77471E-08
LTBP3	0.251611553	7.488278246	9.80192E-08
LOC728519	-0.194073497	6.409420895	9.8469E-08
CXCL6	-0.239139653	6.153223661	9.90459E-08
RPS6KB2	-0.213895677	9.15415192	1.00448E-07
TOMM40L	0.257828014	7.562440621	1.0052E-07
C1ORF83	0.161105097	6.852219713	1.01249E-07
LOC400890	-0.206905499	8.279646681	1.01299E-07
KIAA0240	0.233514446	8.811655032	1.01353E-07
EFHD1	0.374396939	10.15777661	1.02006E-07
C20ORF116	-0.233165846	8.97337062	1.0215E-07
HDC	-0.273617739	6.309317251	1.02527E-07
PEX19	0.235625783	8.367424553	1.03493E-07
ZFP37	0.189516684	6.826920042	1.03616E-07
JUND	-0.34390784	12.25049876	1.03669E-07
LOC145814	0.370677588	6.822975422	1.04264E-07
WFDC3	0.116326157	6.282533312	1.05441E-07
MYNN	0.175132064	7.173695881	1.06086E-07
ADAMTS5	-0.300297084	6.40039788	1.06738E-07
LONP2	0.163287981	8.262525332	1.07203E-07
ZNF610	0.176393091	6.187331156	1.07353E-07
CEP68	0.257987877	7.065768425	1.08024E-07
C6ORF162	0.200584391	6.953751395	1.08328E-07
ZNF175	0.177978592	7.156680067	1.1015E-07
DAZAP1	-0.396200086	7.534918207	1.10402E-07
LILRB4	-0.123884527	6.191456821	1.10513E-07
ZNF271	0.201776251	8.002925684	1.11522E-07
CTNND2	0.266115813	6.811797895	1.11649E-07
C7ORF54	-0.226195517	6.883998908	1.11865E-07
TIMP2	0.323681053	9.132835893	1.12089E-07
LOC645378	0.193557417	6.628095266	1.12611E-07
LIPA	-0.30786625	8.818145075	1.1268E-07
NRM	-0.189784455	6.352689098	1.12685E-07
RAB4B	-0.169816554	7.123286705	1.13216E-07
AGPAT1	0.187040841	6.758164383	1.14195E-07
TPST2	-0.311070825	8.85396205	1.14682E-07
C22ORF40	0.155546367	6.687969213	1.15759E-07
LOC653171	-0.246179133	7.54992032	1.16218E-07
RALBP1	0.226519218	8.514327755	1.17761E-07

SEH1L	-0.214671174	6.990212867	1.18646E-07
RPS2	-0.473196734	11.96131927	1.19203E-07
NVL	0.134145468	6.8741296	1.19777E-07
POLR2C	0.294067302	8.793959598	1.19955E-07
HSPC111	-0.394412736	7.205006133	1.20118E-07
RBM39	0.341624915	8.083168733	1.20375E-07
ATP6V1D	0.250416081	11.54183927	1.20495E-07
DYNC1H1	0.317716334	10.20857695	1.2055E-07
DBT	0.247612855	9.28460252	1.20935E-07
LOC646849	0.395525955	9.416994722	1.22687E-07
MRPS16	-0.271551718	9.308155535	1.22998E-07
TK2	0.193383237	6.856565257	1.23929E-07
VPS37C	-0.323464864	10.32226451	1.25704E-07
HDAC9	0.123465385	6.215372364	1.25924E-07
CDC2L6	0.230357923	8.359061616	1.2647E-07
SCRN3	0.199360776	6.838975209	1.26902E-07
PPP2R3A	0.290623951	7.879300868	1.27128E-07
LASP1	-0.301136979	10.39181589	1.27142E-07
LRG1	-0.19035228	6.260377856	1.27333E-07
MYST4	0.191247853	6.768896468	1.28E-07
GABARAPL1	-0.354682812	10.8839212	1.28569E-07
PIN4	0.176625055	6.699841712	1.29477E-07
RIC3	0.223184958	6.886041107	1.29702E-07
NPM3	-0.329093023	7.735932316	1.29731E-07
RHPN2	-0.252993205	6.833556214	1.3004E-07
CRIP3	0.31818351	6.612209956	1.30206E-07
HADH	0.4067285	12.49813917	1.31425E-07
MYOM2	0.482763263	13.64135863	1.31514E-07
CREB5	0.207945883	6.746028942	1.31929E-07
EDN3	0.126942673	6.073207764	1.33018E-07
GRAMD4	-0.379413173	8.683167824	1.33208E-07
C6ORF203	0.367259569	7.256990871	1.34312E-07
CDC25B	-0.192357964	6.853871321	1.34506E-07
CYB5D2	0.279645055	7.994813863	1.34758E-07
NCBP2	-0.220759065	7.524522661	1.35954E-07
CMYA5	0.309846642	13.1922424	1.36405E-07
KIAA0232	0.253158295	7.344049604	1.37028E-07
SIRT4	0.261807522	7.585008011	1.40168E-07
MTA2	-0.25499117	7.575895548	1.42541E-07
BAG3	-0.421617918	11.41232545	1.44549E-07
CPA3	-0.312972015	6.51364895	1.44907E-07
ZNF680	0.2930662	7.381164344	1.4584E-07
C3ORF60	0.214334786	10.15483564	1.46798E-07
DACT3	0.446106866	9.188715841	1.47777E-07
FBXW5	0.21745189	8.006030628	1.47896E-07
C1ORF2	-0.194648296	7.581336976	1.48278E-07
TTC12	0.20971167	6.503694562	1.49151E-07
ZNF285A	0.218498974	7.487151673	1.50327E-07
PIK3C2B	0.256387688	7.792198311	1.50509E-07
F5	-0.468229447	6.624886652	1.50627E-07
OLFML3	0.391957838	7.642157839	1.5122E-07

MAD2L2	-0.284166954	9.215952655	1.53408E-07
GUCA1C	0.767261785	7.897708234	1.55339E-07
ZNF224	0.164246014	6.789009842	1.55994E-07
ADORA3	-0.206122584	7.699909489	1.56585E-07
FKRP	0.191550086	7.435953412	1.57032E-07
DLD	0.424005308	10.64854238	1.58828E-07
GFOD2	-0.215990913	6.998152045	1.59101E-07
MBOAT7	-0.26012367	6.96957332	1.59787E-07
DC2	-0.383605784	9.054156425	1.60777E-07
LYPD1	0.176574373	6.536560678	1.61508E-07
OXA1L	0.312748993	9.907247073	1.61845E-07
CECR6	0.21056587	6.688107352	1.62471E-07
CCDC8	0.202452074	7.016405151	1.64218E-07
TRPV2	-0.26360884	6.921267003	1.67527E-07
GPIHBP1	-0.424324209	7.364609084	1.67908E-07
TMEM135	0.272154566	7.043137037	1.68006E-07
GAL	-0.311413049	6.075850055	1.68349E-07
PHF5A	0.285553586	8.633347893	1.68568E-07
IRF2BP1	0.201970701	6.972667787	1.69938E-07
KLF15	-0.331326078	7.972686752	1.70046E-07
LGALS8	0.284626004	8.908741873	1.71865E-07
CSNK2A2	0.229864546	9.269702098	1.72326E-07
CD69	-0.140898506	6.125455382	1.72458E-07
C11ORF31	0.140550514	6.49194113	1.73179E-07
PLGLB1	0.16579789	6.426039621	1.74881E-07
ANTXR1	0.335020393	8.365574573	1.7519E-07
ABHD2	-0.251972418	6.619926588	1.75332E-07
GNA11	-0.309707129	9.36525356	1.7849E-07
NIPBL	0.143747899	7.47292144	1.80721E-07
ARIH2	0.346873696	10.23938732	1.80997E-07
CETN2	0.249233278	10.75635785	1.88764E-07
FASTKD1	0.316948544	8.728016314	1.89445E-07
FGD6	0.217974283	6.729188514	1.90354E-07
HLA-DRA	-0.488886748	10.59682585	1.90559E-07
MSC	-0.310594243	6.784680032	1.90864E-07
AP2B1	0.209057919	7.068084497	1.92205E-07
APOL3	-0.242849417	7.004904322	1.92701E-07
ANGPTL4	-0.497220084	6.472881133	1.92986E-07
C12ORF62	0.306557235	10.25514035	1.94019E-07
SCOC	0.275252128	9.26803297	1.94582E-07
FEM1C	-0.310043941	9.85565606	1.99433E-07
TRK1	-0.402601304	6.48754254	1.99742E-07
MAP4K3	0.244525095	7.513033119	2.00528E-07
GGNBP2	0.199709092	9.360736625	2.03803E-07
DOLPP1	-0.162756581	7.157563	2.04325E-07
CYBRD1	0.376545055	9.822389856	2.04702E-07
WBSCR18	0.267531846	7.357516072	2.05584E-07
TBC1D15	0.206984789	8.606899564	2.12663E-07
LOC147804	0.819519496	7.440059617	2.15177E-07
TTC30A	0.19896326	6.790197161	2.1713E-07
POPDC2	-0.313487712	10.94815553	2.19216E-07

SRGAP3	0.232468475	6.847607058	2.19376E-07
STRA13	-0.274256175	9.013767315	2.19663E-07
LOC641705	0.253073934	6.948062427	2.22036E-07
MANBA	0.286747743	8.114242856	2.25635E-07
HSPC171	-0.247139389	7.809371672	2.25762E-07
CARHSP1	-0.350180197	9.183797908	2.26543E-07
SSR2	-0.224470227	7.645118404	2.2749E-07
HIPK2	-0.593682467	9.024136541	2.29626E-07
MRGPRF	-0.2031528	6.544097951	2.30721E-07
NCKAP1L	-0.265869537	6.307669139	2.33844E-07
MRPS33	0.256668036	9.660945935	2.34849E-07
PODN	0.446627484	8.874463758	2.35419E-07
IER5	0.373637844	8.343922358	2.37313E-07
GAB3	0.3061441	7.293764118	2.38479E-07
ASB15	0.397809948	7.285984879	2.38968E-07
SPAG11A	-0.131465084	6.32806269	2.40283E-07
EP400	0.182205258	8.263004562	2.41152E-07
HIST1H4C	0.31087619	11.53665297	2.42473E-07
C17ORF90	0.183896183	8.614095374	2.43084E-07
DDX24	0.288906373	10.15865716	2.4339E-07
EEF2K	-0.346289826	9.125664563	2.43552E-07
APLP1	0.193394462	6.4174912	2.43679E-07
ARMCX3	0.239111353	7.462269846	2.43953E-07
IFT172	0.225835339	7.075970308	2.46311E-07
TCFL5	0.259286446	7.453711929	2.47936E-07
MAD2L1	0.132582122	6.306811226	2.50181E-07
DCUN1D5	-0.254929184	9.035352297	2.50854E-07
NXNL1	0.259497358	6.732650746	2.51056E-07
P2RX5	-0.269725886	6.275885314	2.51441E-07
CLPTM1L	-0.337440746	8.98676618	2.52114E-07
ZNF827	0.317262523	8.208622929	2.53072E-07
LZIC	0.16928931	7.297066226	2.56508E-07
NBN	0.147369625	7.197054998	2.56832E-07
C1ORF35	0.192911338	7.411352157	2.59249E-07
SNORA33	-0.203816234	6.554196281	2.59899E-07
SHMT1	-0.138753636	6.834308455	2.60941E-07
POMT1	0.156407791	7.395351555	2.61025E-07
AFAP1L1	0.233327414	8.536690188	2.61756E-07
SMCR7L	-0.198728803	8.268956376	2.63009E-07
WDR45	0.183905628	7.435474163	2.64283E-07
MPHOSPH10	0.278412605	7.970157561	2.6558E-07
DTX3	0.245666194	7.245466043	2.66979E-07
ENPP5	0.247679713	6.768895552	2.67602E-07
IL32	-0.431804686	7.548461967	2.68179E-07
FAM134B	0.463006404	10.18276844	2.69075E-07
FAM100A	-0.221062271	7.134228836	2.71144E-07
FUT4	-0.176838756	6.7527268	2.73384E-07
PGA3	-0.082404433	6.129414949	2.74214E-07
C9ORF102	0.213886356	7.401045256	2.75498E-07
ZNF704	0.16165029	6.597944747	2.83616E-07
ACTR6	0.311567582	8.996894717	2.84079E-07

CCDC53	0.255019341	9.678952202	2.8486E-07
CD38	-0.211004961	6.070740458	2.88614E-07
ARHGAP19	0.18399111	7.109397216	2.88825E-07
C17ORF87	-0.166144615	6.188051096	2.8894E-07
CFB	-0.512961409	6.966312687	2.90901E-07
C2ORF76	0.193636537	6.886830712	2.91136E-07
MAP2	0.13582931	6.291556943	2.91267E-07
ZFP30	0.193643225	6.419515124	2.91919E-07
PDIA5	-0.231243801	7.01474563	2.92787E-07
RAMP3	-0.324287855	7.82301304	2.9542E-07
PHC1	0.181256826	7.139106632	2.96802E-07
MRPL49	0.194027553	7.68292146	2.96811E-07
USP48	0.129489255	7.008154546	2.96933E-07
SGSM1	0.356520816	7.865359059	2.97442E-07
LOC441268	-0.182149446	6.248298899	2.98618E-07
PCBD2	0.247399137	7.3822402	2.99678E-07
WDR5	-0.126156879	6.770084249	3.00181E-07
NSF	-0.19996906	7.987820481	3.06737E-07
C18ORF10	0.187317286	7.619084479	3.06927E-07
PDHB	0.27729412	12.56083176	3.07238E-07
TCTEX1D1	-0.2119428	6.911527771	3.10625E-07
SEC31A	0.307257091	9.247903479	3.13819E-07
QTRT1	-0.249290119	6.818166372	3.14011E-07
USP28	0.228035341	6.826739217	3.16856E-07
PTPN14	0.192592677	6.702315495	3.17508E-07
ADAT3	-0.17158604	6.289931242	3.18243E-07
PARVA	-0.228922143	7.350246221	3.1943E-07
TFRC	-0.688439975	10.61377384	3.19698E-07
FBXL14	0.161277754	6.290329403	3.21011E-07
ATF6	-0.284579709	8.523643288	3.22591E-07
NOPE	-0.170205294	6.416699207	3.24315E-07
ADCK2	-0.253333359	7.81484891	3.25308E-07
PRMT1	-0.300726893	9.326319841	3.27115E-07
WWC2	0.173048584	6.687777207	3.27169E-07
GPS2	-0.269687523	8.328577754	3.29923E-07
PER1	-0.294952156	6.361781995	3.29926E-07
ADD1	-0.180980509	10.75588816	3.30774E-07
MGA	0.151068293	6.801624247	3.31233E-07
BZRAP1	0.254826607	6.880907024	3.31245E-07
C17ORF80	0.236320281	7.233344824	3.31849E-07
PPP1R10	0.317119142	7.388464333	3.31943E-07
CRY2	0.421896613	8.962694418	3.32013E-07
ADRA1B	-0.289364962	7.069485025	3.32804E-07
CMTM3	-0.207190658	7.393984711	3.33075E-07
NEBL	0.345383709	9.70901722	3.35346E-07
GNG11	0.419501222	10.82742865	3.35505E-07
PARP11	0.116877336	6.508262624	3.36794E-07
NUB1	0.213297268	8.463347777	3.36967E-07
TNFSF13B	-0.291816277	6.849197064	3.37941E-07
CHRAC1	-0.164732264	7.064836983	3.38925E-07
ADH5	0.417273609	7.219504077	3.39164E-07

DBI	0.257926914	11.48767436	3.39958E-07
ACAD11	0.30078052	9.555355242	3.41384E-07
GOLPH4	0.379857466	9.011101534	3.43373E-07
FAM48A	0.110599146	6.576538904	3.44059E-07
LOC57228	-0.154749951	6.422177401	3.45003E-07
APEX1	-0.251315927	9.549693178	3.45407E-07
ELK1	-0.293871334	8.536810543	3.46721E-07
LOC653566	-0.304374962	9.083769615	3.47292E-07
GIMAP5	-0.343084437	7.769696085	3.5186E-07
GAP43	-0.294051665	6.419689901	3.52778E-07
NENF	0.243438294	11.71496468	3.55662E-07
RBP1	-0.361047342	8.21208564	3.57255E-07
LOC731878	-0.286360329	8.365484084	3.57419E-07
SH3PXD2A	0.274743711	10.55185164	3.59532E-07
ANO5	0.197411715	6.461835787	3.59617E-07
SLC4A4	0.166627718	6.758870657	3.60051E-07
CHN2	0.354761093	8.040484674	3.60519E-07
TIGD7	0.237832778	7.873013271	3.62772E-07
SEPT5	0.437541192	9.365072197	3.669E-07
RBM4B	0.199174823	8.078636901	3.67129E-07
CHST14	0.196216318	7.174341583	3.67138E-07
C9ORF103	0.265578046	7.983188398	3.72399E-07
TPRG1L	0.284491035	10.63799394	3.74844E-07
RABL3	0.259880249	7.332236929	3.77365E-07
BTD	0.153925809	6.915106498	3.7786E-07
PLAC9	-0.404517077	9.623082531	3.81658E-07
LOC644590	0.277633478	7.188121223	3.82283E-07
RAI1	-0.235451528	7.855838022	3.82567E-07
SLC25A1	-0.255006373	7.690664642	3.84918E-07
WDR34	0.285222225	8.176310849	3.85315E-07
STK10	-0.179912633	6.540281687	3.8586E-07
FOXO3	-0.285179895	9.17233624	3.87707E-07
CCNL1	-0.201856938	7.48720963	3.87804E-07
FNBP1	-0.205979512	9.615888951	3.90949E-07
C18ORF22	0.172076937	6.986776342	3.94076E-07
GNAI2	-0.257326466	9.996329429	3.95425E-07
CDC7	-0.1937569	6.243648611	3.97874E-07
FXYD1	0.37240626	8.76375603	3.9811E-07
C6ORF48	0.249789462	8.996534216	3.98322E-07
ZNF667	0.134530031	6.528027579	3.99275E-07
MAD1L1	-0.151152632	6.490998068	4.02166E-07
TRQ1	-0.217950675	6.244207313	4.03003E-07
DDX42	0.189584862	8.580069129	4.21052E-07
EIF3I	-0.311958979	10.04889937	4.23462E-07
HSD17B14	0.276791903	6.559411685	4.25283E-07
PPP1CB	0.262932827	9.624405723	4.2819E-07
HDDC2	0.235204282	11.58737691	4.31085E-07
NSMCE4A	0.235980808	9.842291866	4.31198E-07
CTSL1	-0.221250409	10.92994972	4.34365E-07
TAX1BP1	0.191679317	11.51144945	4.36154E-07
SRD5A2L2	0.388866684	12.53721582	4.38126E-07

PIGW	-0.145898506	6.344623025	4.39348E-07
SEPT8	0.16099616	6.404616473	4.40604E-07
IFI35	-0.329946286	7.958865057	4.4195E-07
TACC1	-0.273714756	11.27138995	4.44852E-07
CXORF23	0.185165936	6.491798272	4.46326E-07
DOCK9	-0.291582931	6.870710856	4.4761E-07
TWSG1	0.274635008	8.387715214	4.50252E-07
CHAC2	-0.194157043	6.143187563	4.50485E-07
CORO7	-0.166384933	6.876145422	4.52894E-07
CALB2	-0.11596904	6.243502718	4.57023E-07
ZNF439	0.187519427	7.130579109	4.58207E-07
C1ORF149	0.21034714	7.362649953	4.59725E-07
ZBTB43	-0.28533628	8.522719616	4.60163E-07
SYT17	0.150538305	6.199295966	4.60736E-07
EGLN1	-0.2691615	7.408247983	4.63415E-07
SNRK	0.245922843	9.184961995	4.65423E-07
MESP1	0.360543072	7.400887999	4.65935E-07
CCL19	-0.376592074	6.598529578	4.72299E-07
WBSCR22	-0.252921626	9.522417278	4.73288E-07
TMEM9	0.212674366	7.809071618	4.73625E-07
PSAP	-0.202661258	13.1486324	4.73721E-07
CAMP	-0.186425177	6.098205337	4.7401E-07
HIRIP3	0.230839324	8.912124568	4.75432E-07
VASH2	-0.1992821	6.098465686	4.81389E-07
EPN2	-0.169254718	7.147287041	4.83796E-07
LSM12	-0.155997403	7.254177766	4.89915E-07
KIAA1754	-0.373336584	9.668613281	4.9289E-07
C1ORF74	0.241610988	6.924367526	4.94044E-07
SHISA3	-0.35127655	6.661244652	4.95591E-07
RAD21	0.431630587	9.023139462	4.95843E-07
LAMC2	-0.212294923	6.386100203	4.97369E-07
IRF2BP2	0.291615888	8.107509385	4.97982E-07
LOC151579	-0.313429976	8.458410324	5.01139E-07
ZDHHC16	-0.274341767	8.671721869	5.05233E-07
C16ORF79	0.232343713	7.165094593	5.0718E-07
CLDN15	-0.137441156	6.462595598	5.0831E-07
MEOX2	0.407076657	6.922497005	5.13405E-07
PAQR4	-0.304514881	7.699176726	5.16004E-07
PHF1	0.211703949	7.451888182	5.17527E-07
RTN1	-0.101662088	6.206041592	5.20679E-07
MUTED	0.141575418	7.380041758	5.21749E-07
YAF2	0.180711158	7.941132347	5.22271E-07
ZNF226	0.220661534	7.025968363	5.24776E-07
C11ORF75	-0.465649654	8.447178671	5.28718E-07
PRPF19	-0.234015438	7.957813191	5.29205E-07
HEXB	0.284348052	11.41880248	5.30522E-07
TMEM18	0.137804341	6.659458238	5.32866E-07
JUB	0.159945344	6.263216579	5.34446E-07
ARG2	-0.459550191	6.579342288	5.35874E-07
LOC728411	-0.193612441	6.391028367	5.37195E-07
CYP46A1	0.292577786	7.217230812	5.40577E-07

ITGAE	0.296454133	8.639875158	5.40764E-07
LOC652624	-0.348320231	11.38374015	5.44217E-07
SIAH2	0.24444068	7.604887093	5.44904E-07
SECISBP2	-0.210786041	7.887351017	5.47207E-07
PTGFRN	0.381617905	9.039526924	5.49661E-07
TMEM120B	-0.110049544	6.138969276	5.51707E-07
EIF3A	0.322552271	9.957501734	5.53901E-07
C22ORF13	-0.250378316	10.81456052	5.55728E-07
C11ORF52	-0.219448943	6.212214357	5.58575E-07
C2ORF30	0.228013173	9.549564682	5.59632E-07
CEP63	0.164079298	7.534267118	5.61925E-07
NID1	-0.228704656	6.735745532	5.62973E-07
ECE1	-0.153317831	6.389094136	5.63405E-07
HSPA12A	-0.180547132	7.303107946	5.70813E-07
IL12A	-0.175299725	6.286948631	5.7159E-07
CDC42SE1	-0.150821182	6.90729321	5.72421E-07
TRA2A	-0.244604673	7.731971979	5.73304E-07
DUSP2	-0.218128886	6.349056226	5.74312E-07
E2F3	-0.236498972	8.535554875	5.76711E-07
FLJ39827	-0.293710782	7.448286878	5.78874E-07
OXR1	0.349620755	9.796041041	5.8034E-07
RPSA	-0.321805483	11.7554484	5.80572E-07
UNC93B1	-0.20905089	6.784965004	5.80956E-07
EVI5L	-0.241198261	8.947859683	5.82423E-07
C6ORF108	0.260720596	8.373509519	5.82497E-07
SRPK3	0.34010236	8.431433188	5.85887E-07
PLCXD1	-0.462088484	6.869238762	5.86924E-07
IRAK1BP1	0.152591663	6.666258325	5.87332E-07
ADIPOR2	-0.241255079	9.284555928	5.87718E-07
PPAT	-0.213332342	6.670869951	5.88841E-07
ZNF408	0.148541878	6.514236605	5.89695E-07
KLHL7	0.390729554	8.749421201	5.91529E-07
CAMLG	0.250505244	10.81586757	5.93229E-07
SNRPC	0.215349957	9.412542726	5.96431E-07
UBIAD1	-0.285046266	7.257616368	6.00159E-07
SNAPC2	-0.181871425	7.600016109	6.00938E-07
ANKRD39	0.213551377	7.958841261	6.01408E-07
APEG1	0.22980935	7.260304516	6.0252E-07
ZNF484	0.113720616	6.798966181	6.04529E-07
ANGPTL1	0.127598415	6.234879153	6.07471E-07
EZH1	0.126977095	6.673031866	6.0942E-07
RBBP6	0.149772447	6.87267879	6.1172E-07
MNAT1	0.268971366	7.233834516	6.12935E-07
SLIT2	-0.294797294	7.42165866	6.15079E-07
NRIP1	-0.279788719	8.283272991	6.17291E-07
CKAP5	0.206395919	9.213357216	6.18171E-07
HSD3B7	-0.203669139	6.604616137	6.20365E-07
SCARB2	-0.240924062	11.33064391	6.20452E-07
EPHX2	0.473383737	8.086448518	6.20463E-07
C7ORF49	-0.187283927	7.394914652	6.22129E-07
GNA13	-0.250026014	7.915943931	6.23066E-07

C21ORF70	-0.227193405	7.710029726	6.27281E-07
VPS33B	-0.164509758	7.071882116	6.33275E-07
ZNF254	0.103603554	6.322592771	6.37461E-07
TDG	-0.300517876	8.750333761	6.38806E-07
SLC39A8	-0.196644595	6.343065181	6.40681E-07
AGFG2	-0.105559662	6.3731791	6.41259E-07
NELF	-0.235260445	7.390990786	6.41273E-07
TRAPPC3	-0.215897442	9.50451091	6.4145E-07
ADCK5	0.155838137	6.805250024	6.44118E-07
CHGB	-0.380759433	6.594869468	6.50488E-07
PCF11	0.249365842	7.52674493	6.53988E-07
TRPC4AP	-0.245302566	8.679415684	6.54075E-07
POLR3B	0.184936437	8.331782379	6.54279E-07
ADSS	-0.233893621	7.446513289	6.63022E-07
NAB1	0.253909555	8.352499387	6.63531E-07
KIAA0649	0.242654184	7.496663917	6.64803E-07
LSAMP	-0.515958036	7.151256389	6.70339E-07
CHFR	-0.248139368	8.71628889	6.80434E-07
SLC46A3	0.264828735	8.515683766	6.8275E-07
STAG2	0.154631345	7.073432397	6.87792E-07
FLJ12684	0.310450589	6.90957084	6.92505E-07
SCGN	-0.272350241	6.305174647	7.01452E-07
DHX9	-0.120632593	6.582700809	7.05375E-07
RBM41	0.117252871	6.518509784	7.06293E-07
PDLIM1	0.264631084	11.21659992	7.12452E-07
E2F5	-0.236721544	6.858082674	7.16047E-07
CAPRIN2	0.202292332	7.748694304	7.16352E-07
NOTCH1	-0.312343635	8.867259531	7.1641E-07
IKBKAP	0.107378205	6.442555085	7.16542E-07
ATP5L	0.256781737	12.53013992	7.17025E-07
EBNA1BP2	-0.317240098	10.06247719	7.21347E-07
ERF	-0.202029346	7.251792405	7.21413E-07
MYBPHL	-0.15575795	6.055695213	7.22398E-07
C19ORF42	0.264738844	8.093397261	7.27591E-07
GPN2	-0.232675263	8.749637299	7.29007E-07
TMEM189-UBE2V1	-0.215016011	6.80056136	7.32355E-07
CBLB	-0.339098323	9.34668647	7.35024E-07
C21ORF2	-0.178884556	8.027315173	7.37603E-07
TXNDC4	0.124450968	6.472716368	7.38468E-07
C20ORF149	0.349438458	7.865564437	7.39879E-07
MCCC1	0.285163627	9.70049059	7.47729E-07
FGFR1OP	0.151317184	6.878455035	7.53719E-07
RUNX3	-0.139929999	6.417895976	7.56121E-07
ZNF256	0.193214095	7.868468756	7.6058E-07
PDGFD	0.280299756	7.840578441	7.62051E-07
METTL5	0.255477406	9.871465992	7.62553E-07
SH3RF2	0.541917515	12.11424289	7.67908E-07
EDF1	0.1732932	11.305374	7.71124E-07
C10ORF137	0.209228428	7.813841432	7.74438E-07
FRY	0.357355145	7.718706159	7.78009E-07
BRP44	0.223148187	11.278825	7.79513E-07

TNFAIP1	-0.292020892	8.339681402	7.82713E-07
ATP5S	0.325713356	8.417289655	7.88137E-07
TMCC1	0.175995862	6.636948149	7.94532E-07
FLJ41603	0.308988	8.216600182	7.98651E-07
FGF18	0.603291829	8.000333034	7.98866E-07
VCP	-0.236641512	10.33334531	7.98869E-07
GABBR2	-0.255215853	6.23827189	8.00994E-07
METAP2	0.209772762	10.49870381	8.01068E-07
CPD	-0.269301559	8.762908858	8.01521E-07
ZFHX3	-0.26311335	7.936470757	8.04169E-07
LOC653972	0.292550872	7.061639572	8.12274E-07
XIRP1	-0.690556944	11.04011461	8.14139E-07
C21ORF59	0.150356206	7.448319329	8.18236E-07
GLUL	-0.093172186	6.188114996	8.23583E-07
PRELID1	-0.384564588	7.970784562	8.29439E-07
UHRF1	-0.181440482	6.448530852	8.30181E-07
RASSF5	-0.177406517	6.60232663	8.36231E-07
PDLIM5	0.384462066	11.52144732	8.48397E-07
HVCN1	-0.18705658	6.608638958	8.53232E-07
SLC9A4	0.205185498	6.679687928	8.54735E-07
NAT9	-0.270336684	7.72246958	8.62213E-07
HDHD3	-0.260247752	7.266167359	8.67691E-07
ZNF287	0.139246408	6.166237153	8.6873E-07
PRRG2	-0.176867402	6.39084078	8.70475E-07
UNC84A	0.252750324	9.77218051	8.71597E-07
SNRPF	-0.293239198	9.951513077	8.73007E-07
SRA1	0.263905552	7.133012527	8.76671E-07
RNF170	0.225056162	7.418485498	8.76774E-07
HLA-E	-0.429727553	8.852327641	8.82538E-07
C20ORF103	0.286696956	6.282550641	8.83772E-07
KCNK3	-0.205307763	6.723615455	8.86775E-07
C12ORF57	0.228372681	11.42051631	8.87867E-07
LOC653994	-0.400918124	7.386458184	9.02368E-07
SH3GLB1	-0.340373707	10.05857035	9.03307E-07
FLJ22167	0.166326605	6.706771418	9.07515E-07
RPL39L	0.286866428	8.045982726	9.08133E-07
GBA	-0.173693453	7.367220912	9.08645E-07
GADL1	0.165763156	6.161148211	9.16882E-07
VPS24	0.284967724	8.727968271	9.18037E-07
NPHP3	0.206520995	7.69873111	9.23097E-07
ZNF613	0.135422553	7.155468601	9.29959E-07
FKBP3	0.32576038	9.170968478	9.33799E-07
LOC646463	0.325251155	8.138132347	9.3684E-07
PARVB	-0.251119963	7.63643639	9.39761E-07
SGCA	0.278398184	9.381639801	9.50271E-07
CD47	0.238167611	7.965914074	9.55134E-07
MAF	-0.228809769	6.877994012	9.72595E-07
DMKN	0.272052175	6.804716176	9.73007E-07
SUMO3	-0.240154767	11.14996862	9.73922E-07
SAMD4A	0.353377499	9.717111827	9.76488E-07
SNX21	0.159397435	6.449219205	9.84023E-07

TOMM34	-0.285945415	8.434464546	9.84025E-07
RNF125	-0.167069437	6.324836072	9.85824E-07
PPP6C	-0.187369884	9.98939791	9.90548E-07
NME1-NME2	-0.413492911	9.624767105	9.94365E-07
ASGR2	-0.084746361	6.09079424	9.96661E-07
SNORA10	-0.153630181	6.488300997	9.99733E-07
C6ORF57	0.243475966	7.869951437	1.0025E-06
CXXC5	0.410422858	9.915398083	1.00606E-06
ANAPC13	0.260665128	11.7300435	1.00642E-06
RWDD4A	0.181795078	9.092868507	1.00676E-06
TFB1M	0.244789352	8.378315785	1.00681E-06
E2F2	-0.164983913	6.101096069	1.01948E-06
DHFRL1	0.137941951	6.747767342	1.02227E-06
IL27RA	-0.258567018	7.078810451	1.02286E-06
NR2F6	-0.256398641	8.132413071	1.02983E-06
ZFAND6	0.244308976	8.549709799	1.03112E-06
ZCCHC3	0.200617428	7.16267804	1.03584E-06
LETM1	-0.207731087	6.900937996	1.0373E-06
MGC12965	0.287357996	6.644435902	1.03968E-06
C18ORF51	0.50554981	7.56672818	1.03986E-06
TUSC4	0.19863023	9.402724158	1.04423E-06
AURKA	-0.210316802	7.372677562	1.04842E-06
RAB11FIP3	0.261690971	9.78584833	1.05221E-06
ZMYM2	0.182360263	7.265902348	1.05833E-06
U2AF1	-0.249943121	7.850665266	1.06345E-06
CCNB1IP1	0.234455312	8.920183727	1.06403E-06
GSN	0.35454385	10.05086214	1.07E-06
NSMCE1	0.19018594	9.060980787	1.0813E-06
LOC647970	0.13976473	6.305233125	1.08368E-06
ACVRL1	-0.268675808	7.217763448	1.08539E-06
THOC6	-0.234129589	7.224968999	1.08739E-06
ZFYVE21	-0.345134783	9.65314815	1.10207E-06
RCN3	-0.346546154	7.126940402	1.10467E-06
SLC25A13	-0.298296289	7.485333815	1.12441E-06
MMP28	0.12203989	6.358207374	1.12627E-06
MLSTD1	-0.127174166	6.290780794	1.13152E-06
SNORA70	-0.24538931	7.966918835	1.13722E-06
CCNI	0.215664548	13.10666441	1.13741E-06
MAP2K1IP1	0.19401503	9.165459855	1.13871E-06
PENK	0.767737868	7.360787319	1.13919E-06
SRRM1	0.19749309	9.840808718	1.14346E-06
GABPA	0.184339768	6.80782878	1.14438E-06
STEAP1	-0.092995935	6.111097872	1.14674E-06
FLJ39653	0.233963287	6.978083912	1.14846E-06
HRIHFB2122	-0.170165659	6.71905534	1.14914E-06
RPL3L	0.353621477	11.32485614	1.15059E-06
LOC729101	0.213894144	7.436439357	1.15266E-06
ZNF365	0.099108616	6.152409619	1.16371E-06
SNRP70	-0.206712382	9.190010029	1.1717E-06
SLCO2A1	-0.61415268	8.282284517	1.17413E-06
TMEM67	0.380102736	8.08476391	1.17631E-06

LAT1-3TM	-0.140017301	6.422520872	1.17971E-06
RNF130	-0.21379974	8.512448499	1.18128E-06
LOC644743	-0.337949739	7.460554636	1.18255E-06
AP1M1	-0.231097923	9.752820831	1.18732E-06
KHSRP	-0.26529045	8.073260113	1.20155E-06
EI24	-0.224977355	9.321471226	1.20189E-06
RBM12B	0.251362286	7.402259502	1.20703E-06
C19ORF18	0.237100586	6.933679746	1.21547E-06
PDE2A	-0.225564816	6.772296378	1.2216E-06
KIAA0564	0.260912447	7.507531872	1.22257E-06
LOC339344	-0.198586836	9.336545373	1.23534E-06
TMEM47	0.379218576	10.08803873	1.23994E-06
LRRC10	0.229434054	6.426175377	1.24429E-06
NECAB3	0.272107161	7.680378691	1.24631E-06
FOXC1	-0.443048526	9.247659951	1.24882E-06
LOC285929	-0.2506179	6.448898524	1.25046E-06
UBE2J2	-0.122004398	6.41808567	1.25541E-06
CETN3	0.277315041	7.466735961	1.2569E-06
SLC22A18	0.29257594	7.483068316	1.2659E-06
ZNF181	0.276154734	7.218527217	1.27371E-06
TMEM183A	-0.264292405	9.606811034	1.27685E-06
FAM167B	-0.151249942	6.196081024	1.27801E-06
DNAJA4	-0.485311582	11.71734031	1.28357E-06
ARMCX1	0.29416481	8.570661922	1.29661E-06
LOC643446	-0.183693452	6.820554062	1.30123E-06
NGLY1	-0.203665328	8.305931151	1.30167E-06
C18ORF1	0.20328754	6.776933119	1.30361E-06
STAG3L2	-0.29192051	8.062670514	1.31909E-06
FOLR3	-0.283308691	6.113949178	1.33147E-06
FAM104B	0.151621279	7.100839605	1.33305E-06
CLEC1A	-0.220560335	7.022258829	1.33391E-06
RBBP7	0.314575117	9.204310203	1.3417E-06
C1ORF225	0.318959056	7.910042346	1.34361E-06
LOC401019	-0.217390647	13.27375104	1.34865E-06
OPA3	-0.213030813	7.01727379	1.35655E-06
ALDH1A2	0.346060008	8.32082205	1.35839E-06
PLAGL1	0.150259002	6.698114401	1.36369E-06
IVD	0.332454813	9.483154986	1.36779E-06
FIS	-0.145283452	6.367827146	1.37481E-06
PLCB2	-0.192758108	6.37091749	1.38288E-06
KIAA0196	0.204802364	8.929713713	1.38333E-06
EGFL7	-0.147550685	6.93569067	1.38901E-06
HIST1H4H	0.364140291	7.845663084	1.38914E-06
SUOX	0.248625012	8.64236124	1.40522E-06
TIAM2	0.22431967	6.811342587	1.40727E-06
ZNF703	-0.088779018	6.077750357	1.42714E-06
CDC123	-0.232125979	9.002355778	1.42749E-06
RRAGD	0.272994567	11.03261775	1.4278E-06
FBXO9	0.114543717	6.297775436	1.43061E-06
FAP	0.473261671	7.018487486	1.43268E-06
DERL2	-0.256259329	7.852806963	1.43673E-06

DECR2	0.287745124	8.212786597	1.43828E-06
ZNF18	0.200377977	7.273314253	1.43833E-06
KIAA0528	0.315786872	9.122974858	1.44147E-06
BMP2	-0.203344922	6.693858013	1.44638E-06
CAV1	-0.282572258	11.13586683	1.45476E-06
TMEM80	0.151755826	6.72578385	1.45617E-06
NUPR1	0.417421952	8.154145485	1.468E-06
C9ORF123	0.225094318	8.459281941	1.46813E-06
LOC389203	0.25427818	7.197658268	1.47028E-06
ORMDL3	0.335815926	7.81394191	1.47513E-06
EIF4E3	0.244288062	9.161645394	1.4761E-06
RAB9A	-0.25094397	9.35063697	1.48058E-06
IPO4	-0.250138355	6.906899777	1.48626E-06
CBFA2T2	0.098429051	6.523664205	1.48677E-06
BBS12	-0.256534055	6.996289558	1.48685E-06
PLXNB1	-0.232997865	7.611481361	1.4896E-06
HIG2	-0.350415459	6.569869973	1.49593E-06
PCDH18	0.287269195	7.442134548	1.4971E-06
ANXA2P3	-0.199965994	6.35724504	1.50612E-06
FAM45A	0.26611677	8.699858888	1.51071E-06
SMC4	0.160698319	7.121004381	1.51606E-06
HPS1	-0.132563283	6.432173224	1.51827E-06
PIGZ	0.203169027	6.982184773	1.52671E-06
MGAT4A	-0.183173985	6.9298362	1.53385E-06
PELP1	0.23049753	8.319473818	1.53616E-06
GARS	-0.280336553	9.630601724	1.55454E-06
OSM	-0.128004189	6.050562378	1.56639E-06
C11ORF45	0.203607941	6.657424303	1.57071E-06
TTC33	0.240343455	7.148930658	1.57087E-06
C11ORF38	-0.209915238	7.125875391	1.58003E-06
RAB40B	0.422441313	9.972034862	1.58718E-06
CDH23	0.169723197	6.670299099	1.59063E-06
COQ3	0.298127764	9.980038009	1.60146E-06
CCR2	-0.080559616	6.139243126	1.61085E-06
TMEM183B	-0.259674694	9.561538413	1.61339E-06
COG6	0.232143802	7.955359324	1.61765E-06
LEMD2	-0.174162162	7.958987401	1.62247E-06
SKP2	0.110348882	6.926985399	1.63213E-06
PACSIN3	0.34504048	9.71847721	1.64523E-06
PDS5A	-0.183458164	7.539917475	1.66892E-06
AGTPBP1	-0.256238964	7.969684867	1.68426E-06
UQCRQ	0.210906765	14.0409431	1.68678E-06
CCDC32	0.289269058	9.612294068	1.69034E-06
FBXO30	0.229211154	7.972683917	1.69234E-06
HEATR2	0.203615054	7.669123073	1.6928E-06
PIGO	-0.101093409	6.597044255	1.69493E-06
KIAA1244	0.215228786	6.410524005	1.69717E-06
KIAA1267	0.170131884	8.48223748	1.70784E-06
PIAS4	-0.233133292	8.649454713	1.71947E-06
BMF	0.100562385	6.122953106	1.73846E-06
WDR13	-0.196317515	10.08081106	1.74603E-06

SRPR	-0.210936613	8.280608747	1.74833E-06
SUPT3H	0.12933877	6.644333109	1.76399E-06
PLD3	-0.19756196	8.196777973	1.76487E-06
ELK4	-0.09961229	6.232190911	1.77624E-06
POLR2D	-0.218091815	7.381488894	1.77777E-06
ZNF561	0.19684384	6.632856235	1.78771E-06
CFI	-0.261489425	6.523670145	1.78973E-06
UBAC1	0.248451978	11.31564634	1.79476E-06
PMVK	-0.29750052	7.34089619	1.79816E-06
TCEB3	-0.200948742	8.059312121	1.81127E-06
LTB4R	-0.175498736	6.141160122	1.81837E-06
PTGDS	-0.423120137	13.18295568	1.82778E-06
TPMT	-0.24000311	8.428479389	1.83077E-06
SNX10	-0.211128543	6.185509695	1.83915E-06
F2RL2	0.138498791	6.136250954	1.84588E-06
CD300LG	-0.454049124	8.79678015	1.85361E-06
UBE2O	0.225354684	7.596739696	1.86576E-06
CCBL1	-0.217290587	7.659121803	1.86593E-06
RPLP0	-0.424842856	10.77952388	1.87261E-06
ZSWIM6	-0.248931668	7.829908564	1.88895E-06
AXIN1	-0.266099251	7.572900125	1.89722E-06
KIAA1107	0.126442758	6.246229084	1.90169E-06
TJAP1	0.210309578	9.79194463	1.91405E-06
DHTKD1	0.29332249	7.0817891	1.91656E-06
AMY2B	0.319266758	7.133144079	1.92424E-06
POLB	0.266167752	9.343490445	1.9425E-06
ZFP161	0.161548812	7.617921257	1.94379E-06
BOLA1	0.185824872	6.887092694	1.94795E-06
C20ORF43	0.156463372	11.07065716	1.96165E-06
SHE	-0.287888711	7.617709101	1.96818E-06
TRAPPC2L	0.305119462	10.3670031	1.97301E-06
C2	-0.17012162	6.549249332	1.99825E-06
LRRC51	0.131793005	6.567280712	2.00743E-06
RAB38	-0.207780763	6.185211421	2.00769E-06
RIC8A	-0.211969955	8.85143601	2.02119E-06
CNBP	0.258239715	11.21773097	2.02254E-06
TMEM159	0.405651977	8.722106649	2.02694E-06
CKAP4	-0.273321379	10.01736496	2.03021E-06
SETBP1	0.279429573	7.245697912	2.03653E-06
C10ORF67	0.208812374	6.682767152	2.05651E-06
KIAA1271	-0.140826461	6.356520127	2.05805E-06
ILF3	-0.150467078	7.883822169	2.05815E-06
TLR4	-0.162060941	6.535152904	2.06735E-06
WDR45L	-0.218430914	8.046577249	2.06932E-06
EHD3	0.276433173	6.715275444	2.07243E-06
C16ORF72	-0.1853503	8.492685959	2.07299E-06
CRYBG3	0.151773512	6.301768897	2.07991E-06
C15ORF40	0.146302295	7.298935889	2.08941E-06
ARNT2	0.19100705	6.6422217	2.09876E-06
LOC641825	0.331612261	7.371910874	2.10302E-06
KCNIP3	-0.135352663	6.67616538	2.10552E-06

PH-4	0.174441232	7.607824742	2.11484E-06
ZNF507	0.14237738	6.323742698	2.1269E-06
HSF4	0.181655226	6.481427958	2.12995E-06
ANK1	0.249040852	6.850785152	2.13331E-06
DAB2	-0.332273153	8.509371433	2.15103E-06
ABCB7	0.28314935	9.007257342	2.1598E-06
AQP4	-0.208648546	6.175338921	2.16355E-06
AKAP8L	-0.279598987	7.575236384	2.19517E-06
OSAP	0.178926237	6.416088713	2.20213E-06
PSMB9	-0.243000233	6.83700935	2.2142E-06
SLC16A14	0.146206421	6.535321395	2.22608E-06
EFTUD2	-0.229800687	9.02821118	2.24315E-06
MSRB2	0.214527272	12.7463955	2.24611E-06
SLC41A3	-0.185904353	8.783561166	2.25081E-06
FARP1	0.359276601	7.444688232	2.25764E-06
C15ORF59	-0.314646583	8.639809131	2.25882E-06
FAM81A	-0.208393753	6.23913336	2.26729E-06
ESD	0.205205913	9.861526678	2.2723E-06
CEP290	0.177799518	6.669057556	2.27249E-06
CPE	0.431287884	8.979632744	2.28372E-06
KLRG1	0.147253573	6.610242229	2.28703E-06
TUBE1	0.322637346	7.384752784	2.29401E-06
HIC2	-0.1828703	7.031214776	2.31662E-06
ELOVL5	-0.377676156	8.056770435	2.32044E-06
GPR124	0.262267482	7.112737398	2.32436E-06
COQ6	0.225831066	8.482586912	2.32525E-06
LOC283174	-0.225357005	6.821073872	2.34268E-06
CETP	-0.143296114	6.394553678	2.34563E-06
LOC643293	0.106024153	6.257815182	2.35581E-06
CCDC51	0.191119106	7.407364588	2.36948E-06
FHIT	0.207618465	6.922466562	2.37245E-06
ITFG2	0.202028736	8.04181352	2.37804E-06
FAM155B	-0.165633597	6.376047976	2.40617E-06
SMAP1	0.173262719	7.942012415	2.41298E-06
VGLL3	-0.18919604	6.319804678	2.41985E-06
C16ORF87	0.248628029	7.890724103	2.4201E-06
RARRES3	-0.496556978	8.197334892	2.42893E-06
WDR59	0.223954234	9.271776414	2.45994E-06
FBL	-0.220945767	9.666532481	2.46026E-06
C2ORF18	-0.220273573	6.997678511	2.47683E-06
HS3ST2	-0.12112252	6.160133029	2.49374E-06
ESRRAP2	-0.240539879	9.326268856	2.49903E-06
SETD3	0.176758627	10.20115817	2.51633E-06
ZNF234	0.175715237	7.176827082	2.52482E-06
SNRPA1	-0.259751078	9.936237789	2.52607E-06
SNCAIP	0.239118187	6.962620694	2.55699E-06
ZDHHC15	0.17326701	6.44941573	2.5589E-06
ZNF491	0.106357268	6.405863495	2.56133E-06
ZER1	0.220802499	7.552378316	2.56151E-06
ZNF12	0.180979321	6.738164249	2.58163E-06
ZNF25	0.289422062	9.294582843	2.58571E-06

C11ORF71	0.230171999	6.902394785	2.59142E-06
EDN1	-0.352833398	6.953398971	2.59797E-06
SLAIN1	0.178777924	6.77240041	2.60757E-06
C3ORF33	0.20024336	6.646977412	2.62056E-06
PTRH1	-0.293987202	7.697857571	2.62917E-06
GNMT	-0.185807683	6.148215101	2.62958E-06
MED23	0.122968005	7.098453502	2.63595E-06
LRRCC1	0.186445807	6.798300839	2.64734E-06
CD52	-0.302022115	6.985311901	2.66153E-06
TSNAX	0.262312626	7.699966838	2.66158E-06
KCMF1	-0.135383383	6.807205733	2.6625E-06
MYO9A	-0.202570117	7.997230087	2.66775E-06
LOC645863	0.154581296	6.41218332	2.67097E-06
LOC440349	-0.144119034	6.408476203	2.67252E-06
AGPAT9	0.323617806	9.637084055	2.67682E-06
KRCC1	0.252911233	8.417847655	2.67957E-06
CHKB	0.217196751	7.957809478	2.68099E-06
S100A4	-0.440440614	10.92224018	2.69159E-06
PNPLA7	0.498006398	8.880082771	2.70525E-06
HKR1	0.176657657	7.331691674	2.70609E-06
FAM57A	-0.205464105	7.473795876	2.71846E-06
CEP57	0.182006243	6.750191437	2.7226E-06
CPNE3	0.273869998	10.62108839	2.72908E-06
A4GALT	-0.435605593	8.775687938	2.75045E-06
RTN4R	0.147197058	6.338311835	2.76007E-06
BHMT2	-0.178181189	6.464856154	2.77232E-06
DOCK10	-0.210278937	6.987389935	2.79765E-06
LINGO4	0.301004676	6.976628958	2.81276E-06
GDF10	0.194431873	6.251550236	2.8237E-06
CHMP2A	0.161659032	9.556310887	2.84145E-06
KPNA4	0.269214876	11.1724788	2.84892E-06
KIAA1688	-0.189969605	6.864170902	2.85705E-06
CCDC136	0.404167796	7.980447919	2.86575E-06
PEBP4	-0.578551761	8.948643673	2.86984E-06
BBS1	0.213851142	7.674554497	2.88781E-06
DYRK3	0.124530708	6.512325744	2.90955E-06
TMEM177	-0.186305923	7.702596337	2.94608E-06
STIL	0.233246081	7.232797651	2.96796E-06
MRPL1	0.292442574	8.637126814	2.97102E-06
LAMA4	0.315551352	8.420100152	2.97968E-06
ACBD5	0.182681523	6.472705846	2.98521E-06
CDCA2	-0.424665678	7.083354296	2.99599E-06
LOC648057	0.255950005	6.826868101	3.00597E-06
GNRH1	0.160080149	6.928290032	3.01692E-06
SLC38A7	0.199570394	6.815018672	3.02341E-06
CIR	0.270480983	7.568882853	3.05667E-06
SH3BGR	0.273700451	7.33413958	3.06603E-06
HIBCH	0.306187707	8.962105122	3.07552E-06
C18ORF8	-0.192654324	8.088138439	3.09097E-06
MAT2B	0.187219657	9.550159107	3.10535E-06
TMUB2	-0.124375075	7.58303678	3.15078E-06

RBBP9	0.134562351	7.149273114	3.16552E-06
SDC2	0.244843032	7.493411237	3.17022E-06
NPHP1	0.075487273	6.278541879	3.18652E-06
RNF24	-0.212760379	6.93507849	3.18965E-06
GNPNAT1	-0.19870341	6.247986416	3.19066E-06
SAMD13	0.128445761	6.262209582	3.19137E-06
YIPF1	-0.180156428	8.345328241	3.19414E-06
TMEM198	-0.160937214	6.354385956	3.20156E-06
UBTD2	-0.117732166	6.383479331	3.20234E-06
FKBP14	-0.338770474	9.191591834	3.2218E-06
DPF3	0.279774452	7.472696538	3.22835E-06
ZNF71	0.407603035	8.77332479	3.23E-06
MRPL52	0.191057395	8.04859243	3.23632E-06
PLEKHA5	0.474734562	8.254517443	3.24778E-06
AKAP11	0.158330059	7.621474636	3.24883E-06
KIAA0152	-0.280115347	10.033861	3.25119E-06
SRPK1	-0.17346478	8.256244697	3.27217E-06
EDIL3	0.227225293	6.33522583	3.27537E-06
LOC652545	-0.273320984	8.462344665	3.27871E-06
LRRC8D	-0.207473722	6.571837548	3.28225E-06
YIPF3	-0.264239397	9.603378796	3.29171E-06
DBN1	-0.317640436	7.455345689	3.35011E-06
HECW2	0.378660896	8.44340745	3.41589E-06
RIOK2	0.194362756	8.11991856	3.41708E-06
C14ORF2	0.290206831	9.636941674	3.41994E-06
LOC642299	0.230839528	7.105571898	3.4483E-06
FAM50B	0.277265163	8.39761267	3.46945E-06
LEPR	0.199618999	6.635496485	3.48562E-06
CRISPLD1	0.10807434	6.129072431	3.4956E-06
ZFYVE16	0.156846507	7.369036874	3.50691E-06
MAGED2	0.176014987	7.014003837	3.53055E-06
U2AF1L4	0.163058509	7.217354623	3.5421E-06
CHODL	0.182332918	7.086451275	3.5486E-06
MARCKSL1	-0.344289003	7.949922177	3.58647E-06
SGPL1	-0.132307117	6.658501557	3.59145E-06
C1S	-0.398773441	8.053278233	3.6005E-06
FAM33A	0.168365812	7.358695563	3.6231E-06
SNAP23	0.248365375	7.86293133	3.62988E-06
EIF4A3	-0.171695942	9.366449142	3.64158E-06
GNL3	-0.290334175	7.857558137	3.65077E-06
KCNT2	0.133372134	6.181932355	3.6687E-06
MMP15	0.309891804	7.497859137	3.67353E-06
GOLGA4	0.209140687	8.344351526	3.69039E-06
NDRG2	-0.475253298	9.297911673	3.70028E-06
GCNT3	-0.209532942	6.078860861	3.70129E-06
RPAIN	0.210460031	10.23065512	3.70285E-06
LOC650695	-0.135239929	6.08807188	3.80633E-06
THY1	0.629629857	7.15821396	3.81354E-06
LMNB1	-0.126814981	6.10000153	3.8224E-06
CADPS2	-0.231671326	7.012261774	3.82992E-06
BTRC	0.19347267	6.81280145	3.84324E-06

ANKRD23	0.366599015	6.916821346	3.87638E-06
PAWR	-0.212264566	6.739060358	3.8818E-06
CRKL	-0.211440487	10.3604246	3.89197E-06
CDC5L	0.247975959	8.254409752	3.90897E-06
C20ORF194	0.146520122	6.8871312	3.90958E-06
GTF3C3	0.181213192	8.415152277	3.91365E-06
C4ORF27	0.168107517	8.24310369	3.91831E-06
TGFB1I1	-0.223575976	7.09738342	3.93832E-06
GPR162	-0.218610994	7.398761282	3.93895E-06
ACVR2A	0.144662171	7.122610983	3.97037E-06
PPP2R3C	0.207566034	8.929204982	4.01941E-06
FOXK2	-0.112196996	6.282670643	4.05801E-06
ELP4	0.168032663	7.303977805	4.07311E-06
PGLS	-0.270801492	8.352777072	4.07369E-06
EP300	0.177624047	7.577614215	4.07413E-06
PLXNA3	-0.249823284	6.767285137	4.08529E-06
WIPI1	0.297334355	9.883590209	4.09763E-06
C11ORF54	0.203795159	8.089888809	4.1045E-06
GRB10	0.156220564	7.249761844	4.14627E-06
SNORD108	0.134165896	6.168208663	4.14671E-06
BCAM	0.087392491	6.313945473	4.15276E-06
PANK2	-0.135836148	7.536848773	4.1546E-06
CLEC5A	-0.186063001	6.271306276	4.15915E-06
ANXA1	-0.381173717	9.896294897	4.1719E-06
ZNF462	0.288819439	8.71170752	4.1934E-06
LSM7	-0.269881727	9.778756317	4.19665E-06
NMD3	0.232807581	8.676761118	4.24323E-06
MGC71993	-0.189379139	10.28828842	4.25948E-06
PNMT	-0.155707997	6.344155799	4.27718E-06
PLEKHG1	-0.219535799	6.732465453	4.28392E-06
FABP3	0.383018331	11.29832356	4.3026E-06
TP53I13	-0.370930809	8.933926871	4.31725E-06
TRIM32	0.154126251	7.489337054	4.33422E-06
TES	-0.137161875	6.268546197	4.33917E-06
SLC25A16	0.16418245	6.627028145	4.34473E-06
RB1CC1	0.267637271	9.459333672	4.35922E-06
PDXP	0.230028169	9.740999602	4.3687E-06
MLH1	0.311281662	8.857357191	4.37347E-06
KIT	0.322298789	7.143298111	4.3859E-06
DVL3	0.293386239	8.545228503	4.39352E-06
ZBTB7B	-0.157278563	6.386701704	4.39491E-06
HINT2	0.239570849	10.15375009	4.3975E-06
FLJ39632	0.102582558	6.183240218	4.40367E-06
HNRNPU	-0.244463465	8.287321485	4.40796E-06
CRSP9	0.140039669	6.99349351	4.4097E-06
HSP90B1	-0.352105391	12.01183315	4.41772E-06
ZC3H6	0.195065649	7.049174613	4.42943E-06
LOC402176	-0.184269938	6.289997047	4.48719E-06
MFSD7	-0.158524079	6.597223559	4.49669E-06
LMAN2L	0.229972034	8.583521301	4.51542E-06
CAMK2G	0.147674715	7.06053021	4.54003E-06

FDX1L	0.177947238	7.959079794	4.57523E-06
EIF5A	-0.335907641	10.09786762	4.58718E-06
HAS3	0.114934236	6.149153697	4.60345E-06
TCEA1	0.202509121	7.221669685	4.60689E-06
ITSN1	-0.198400489	8.102996528	4.63688E-06
PRKAA2	0.344395153	7.451691064	4.67452E-06
MTERF	0.115669088	6.532634954	4.68139E-06
SLC43A3	-0.141644952	6.774429297	4.68402E-06
PKIA	0.262262072	10.788681	4.70169E-06
DHX33	-0.172825058	7.325551812	4.70959E-06
B3GAT3	-0.174599504	6.581474779	4.73436E-06
SHROOM4	-0.341879042	9.971078844	4.74217E-06
ATP11C	-0.133003174	6.393088475	4.75174E-06
LOC144481	0.166812128	6.234658964	4.75411E-06
SHPRH	0.114450082	6.590370527	4.76697E-06
YOD1	0.210137327	7.299766696	4.76902E-06
RAPGEF3	-0.169908296	6.745156732	4.8074E-06
PPP1R8	0.10076281	6.728054663	4.80861E-06
CYB561D2	-0.162059155	7.255876525	4.82723E-06
DDX23	-0.201327589	7.825937556	4.83048E-06
VTN	0.46761135	8.741802437	4.85038E-06
HDAC2	0.227236045	9.640893898	4.89796E-06
CORO1C	-0.321985752	8.138814496	4.90248E-06
THAP1	0.167496081	7.217630797	4.91456E-06
FBXL20	0.177344627	7.453692114	4.92125E-06
NAP1L1	0.217943827	8.364325214	4.93189E-06
CHMP1B	-0.230316935	9.101183024	4.9379E-06
HEXIM2	0.172725453	6.646690905	4.95857E-06
SELE	-0.202572419	6.29582129	4.95954E-06
STBD1	-0.317488788	7.599608789	4.9924E-06
ABCA5	0.118593706	6.358639187	5.04682E-06
IARS2	0.238831347	9.915757664	5.06366E-06
PCDHB15	-0.146178587	6.127009784	5.07495E-06
C1ORF216	-0.184405207	6.825445171	5.11515E-06
MTCP1	0.231341857	10.60119236	5.14958E-06
CCDC43	0.246734731	7.643218864	5.17261E-06
GNPTAB	0.17667316	7.300325863	5.17757E-06
C1QL1	-0.152009253	6.266596806	5.18109E-06
BNIP3	0.268948033	11.80340992	5.20832E-06
LZTR1	-0.238081953	9.069926242	5.22785E-06
PLEKHO1	-0.388566975	8.921189551	5.2652E-06
BMP7	-0.283447418	6.369391931	5.2912E-06
SPAG7	0.251743674	9.83100307	5.30069E-06
CYP1B1	-0.65519443	8.593168422	5.3259E-06
NPEPL1	0.248265583	8.943086196	5.33631E-06
BMPR2	0.161095772	8.997967635	5.34815E-06
SPATA5L1	0.21259081	7.560089164	5.35442E-06
PSMB3	-0.168253311	11.17138538	5.35827E-06
LOC642333	0.346299065	9.598939561	5.36478E-06
PSME2	-0.40617863	9.016190206	5.3734E-06
OSR2	-0.080747532	6.110540731	5.38052E-06

RUNDC2A	-0.118010802	6.179540127	5.38797E-06
AIG1	0.354405137	8.515631386	5.39933E-06
ADSSL1	0.341059023	9.23639619	5.39942E-06
PPP1R14A	-0.30239409	9.254383179	5.42789E-06
REPS1	0.150766677	6.713442766	5.44828E-06
TMEM27	0.163448036	6.357611613	5.44858E-06
AHCY	-0.223968407	9.379270016	5.45842E-06
C14ORF131	0.243718444	8.671866293	5.46038E-06
SRRP35	-0.150269835	6.066415575	5.47392E-06
EVI2B	-0.189461918	6.43708252	5.48535E-06
RPS6KA2	-0.244704186	8.522724333	5.50104E-06
MYL5	0.267012036	7.147755202	5.51135E-06
SH2D5	-0.187484664	6.43517709	5.51938E-06
SEPP1	0.385502014	8.35794281	5.52794E-06
CHUK	-0.180366326	7.882389399	5.52892E-06
GPSM3	-0.234859212	6.24617326	5.54528E-06
WDR67	0.220282876	7.156474889	5.56766E-06
SCG2	0.406799549	6.34081482	5.5987E-06
SEC14L2	-0.166159343	6.017590835	5.61653E-06
APCDD1	0.374281751	8.078393002	5.65019E-06
SCRN2	0.175174947	6.992070727	5.67025E-06
TOR1AIP2	0.117622001	6.524841667	5.69147E-06
CNOT4	0.161367057	7.462917245	5.75143E-06
ATG5	0.186053374	7.777025238	5.75956E-06
PDGFC	0.251780929	7.771669355	5.76594E-06
C20ORF132	0.086007495	6.345605641	5.76754E-06
UBE1C	0.317766367	7.306568848	5.77164E-06
PPIL3	0.260509569	8.192125965	5.78993E-06
CXXC1	-0.175241593	8.526258465	5.79512E-06
SCAPER	0.250722733	8.142243825	5.81575E-06
LOC645566	0.184855497	6.193600267	5.86241E-06
DVL2	0.152653003	7.304313984	5.86483E-06
GABRB3	-0.070906822	6.122694043	5.87669E-06
U2AF1L2	0.297509246	8.760474951	5.90362E-06
ASS1	-0.267829963	8.097952732	5.90691E-06
CEACAM3	0.149951913	6.49857015	5.94503E-06
POSTN	0.624928263	7.80432931	5.94605E-06
LPCAT4	-0.296504868	6.991856568	5.98363E-06
HAND2	0.259922531	7.46913511	5.99872E-06
LOC644436	-0.199471679	6.193469002	6.00823E-06
WDR55	-0.182747992	6.820387496	6.03849E-06
STK33	0.19561922	7.320473502	6.04378E-06
STX4	-0.338243696	8.912508229	6.1066E-06
APOE	0.605571013	9.011193494	6.1271E-06
SMARCB1	-0.190020186	7.589524145	6.12858E-06
TTC13	-0.145878096	7.105578305	6.15579E-06
PNPLA6	-0.174993761	8.468396519	6.18592E-06
PTTG1	-0.302888693	6.599709753	6.20586E-06
PTHLH	0.263732392	6.325987278	6.21632E-06
CNN1	-0.698453179	9.308810118	6.21958E-06
SIVA	-0.230091836	9.248132321	6.22829E-06

ACOT8	0.18220785	7.31337459	6.23502E-06
ANKRD26	0.193994666	7.231776616	6.23705E-06
RN7SK	-0.242877765	7.544488412	6.24392E-06
BET1	0.16599762	7.334582659	6.2443E-06
EIF2A	0.203967744	9.925959388	6.24446E-06
TAX1BP3	0.204332898	10.75852882	6.26198E-06
USP10	-0.229612926	7.747004295	6.29974E-06
KIAA0247	-0.17611895	7.838515923	6.34972E-06
C18ORF21	0.175215398	7.948950579	6.35113E-06
GFM1	0.218184235	10.12633843	6.41619E-06
CASC4	0.173198432	7.013051002	6.42781E-06
HEYL	-0.457001879	9.857875113	6.43074E-06
SNX11	-0.135367498	7.439427912	6.43081E-06
BEX2	0.401459124	7.234679121	6.44118E-06
P4HA2	-0.254385397	8.533066804	6.45576E-06
KCNMA1	0.102149125	6.268370925	6.46347E-06
PPP1R3E	0.159671527	6.646261746	6.47021E-06
LRRC47	0.180087972	9.325987835	6.47953E-06
LUZP1	-0.256858143	9.787384221	6.48664E-06
C20ORF107	-0.100082995	6.279118134	6.49607E-06
LOC642780	0.084406484	6.264720998	6.49998E-06
USP38	-0.421073488	9.392593583	6.51626E-06
ZNF148	0.394456218	9.457370263	6.51743E-06
FLJ43663	0.282652394	7.123994557	6.52751E-06
ARPP-19	-0.202551345	8.236156189	6.59704E-06
PFAAP5	0.265729422	7.989707811	6.62866E-06
MPDZ	0.285688023	9.389910921	6.66085E-06
SNUPN	0.160824112	8.058393247	6.72566E-06
FLJ21438	-0.202900248	6.986758977	6.72799E-06
LOC642921	-0.144356781	6.314426909	6.74891E-06
SDHAP3	0.097528683	6.371500083	6.75605E-06
ME2	0.276208849	9.338697737	6.79538E-06
LONP1	0.239067048	9.818867662	6.80377E-06
SDHD	0.40273875	10.81736601	6.82189E-06
C9ORF16	0.248670963	7.014922691	6.84266E-06
TPP2	0.112967635	7.00864342	6.85058E-06
HRH2	0.115828334	6.072726442	6.85297E-06
SLC25A20	0.281436274	8.312890183	6.85877E-06
CUX2	0.201543189	6.021775679	6.91458E-06
RING1	-0.169775467	10.12538422	6.94485E-06
DHX35	0.190986325	7.507436821	6.97942E-06
PI4KB	-0.146638588	8.145563373	6.99422E-06
PPP2R1B	0.096285103	6.633179109	7.00023E-06
GOT2	0.27549259	12.41408151	7.03591E-06
LOC647054	0.133001197	6.546059155	7.03616E-06
RASL12	-0.245783257	8.826881071	7.0365E-06
ZNF425	0.133115204	6.306589196	7.09344E-06
LOC649604	-0.178204009	6.388499242	7.10931E-06
CHCHD5	0.20991071	8.448217984	7.11176E-06
USP22	-0.11660963	6.380798898	7.12209E-06
VWF	-0.247975401	13.03337409	7.12623E-06

HEBP2	0.268422511	11.73391124	7.12918E-06
C1ORF128	-0.230180693	9.738263017	7.14725E-06
TAF1C	-0.190833707	8.212025895	7.19785E-06
LOC339804	0.44781391	7.754029208	7.21335E-06
ANP32A	0.385825277	7.069310621	7.21933E-06
DHRS4L2	0.27617331	8.009108847	7.2216E-06
CDK5	0.228706652	7.142954305	7.29501E-06
PARP9	-0.250551803	7.538549225	7.33283E-06
RBM5	0.180639809	10.21384697	7.36623E-06
YIPF4	0.352311082	8.562299232	7.37421E-06
GMDS	-0.208247665	8.547643871	7.39235E-06
GATA6	0.182349633	6.544359437	7.40954E-06
RPGR	-0.183135355	6.457517751	7.41208E-06
PPP1R11	-0.195038838	10.66987169	7.41612E-06
TPPP3	0.164517091	6.73489517	7.43432E-06
UBTF	0.097475911	6.439390671	7.43743E-06
C3ORF15	0.138785144	6.799681279	7.44566E-06
PLEKHA6	0.29758898	7.156098744	7.48114E-06
NME5	0.206619716	6.667789127	7.49642E-06
ZFAND1	0.173452642	7.726943705	7.51658E-06
GSPT1	-0.28262055	10.99727795	7.5797E-06
DKFZP564O0523	0.155375802	6.670928582	7.65245E-06
ZSCAN4	0.132828106	6.245398108	7.66822E-06
SYNE2	0.286963868	7.588826715	7.67182E-06
ENSA	-0.201869335	7.137009261	7.72471E-06
ATE1	0.079724224	6.348027534	7.7401E-06
WNT2B	0.122163725	6.703464316	7.82609E-06
GTF2B	0.139304013	7.798390498	7.83028E-06
TMEM182	0.564810295	9.207178442	7.84806E-06
LOC728635	0.251712454	7.693311383	7.8582E-06
ZNF517	0.085165369	6.306946023	7.87724E-06
TPPP	-0.220559966	6.373453428	7.88774E-06
RBM26	0.196110711	7.290526036	7.88952E-06
ENPP2	0.365902732	7.096411455	7.92288E-06
ZFYVE26	0.203517599	8.513383194	7.92661E-06
MGC18216	0.264907012	8.164763627	7.92794E-06
LOC648249	-0.309046013	9.889540183	7.95041E-06
CISD1	0.180097116	12.32484724	7.95161E-06
LOC651029	-0.29181732	6.940288773	7.98746E-06
DKK3	0.401185032	11.71459575	8.00398E-06
MCM2	0.186785309	6.883659724	8.00407E-06
JPH1	-0.317441485	8.357419538	8.00572E-06
CCDC131	0.154328472	6.887172869	8.06668E-06
AOAH	-0.161488408	6.320637573	8.08619E-06
CNIH4	-0.378477689	9.608850028	8.15147E-06
RIPK1	-0.174585547	7.427938866	8.1773E-06
NUDC	-0.162978236	11.22809088	8.22595E-06
FGL2	-0.281497411	7.670210037	8.26987E-06
LZTFL1	0.192132043	7.234208683	8.2822E-06
KIAA0261	0.236059776	7.537283903	8.29213E-06
DYNC1LI1	0.27635758	7.132449925	8.3242E-06

ACTR5	-0.191303047	7.026659669	8.33388E-06
TPM2	-0.228322804	11.63143974	8.34007E-06
LOC401238	-0.164536608	6.398229419	8.38935E-06
ARMET	-0.378527822	10.42335366	8.41271E-06
TRADD	-0.137073119	6.524541142	8.42785E-06
RENBP	0.251261642	8.307070443	8.43324E-06
NPR2	0.143553661	7.160139061	8.51308E-06
TMEM62	-0.194794124	7.825770713	8.52605E-06
GLRX2	-0.245906449	8.173686208	8.56759E-06
AK3	0.275444874	11.43370532	8.57331E-06
ARGLU1	0.258228175	10.45202592	8.58261E-06
LOC653308	0.195014269	7.076768066	8.58958E-06
TEF	0.336365986	7.682216781	8.60545E-06
BBS9	-0.524633285	7.49542781	8.76443E-06
SLC19A3	-0.138745311	6.158138482	8.79371E-06
ENDOG	-0.255482364	8.466374547	8.89897E-06
ANKRD32	0.183739731	6.75986404	8.92641E-06
C9ORF23	0.267069502	7.836426873	8.92717E-06
TSPAN4	-0.226495511	7.721729879	8.93355E-06
LOC440093	-0.333834159	8.007552811	8.96862E-06
MFHAS1	-0.188986791	6.646822639	9.00709E-06
STMN1	0.114996527	6.531106733	9.03767E-06
CTBP2	-0.09032764	6.258666393	9.0388E-06
ZBTB48	-0.157068444	7.719842578	9.04724E-06
LOC643911	0.392583261	8.423167924	9.05456E-06
ARHGAP11A	-0.102354424	6.209444371	9.16824E-06
DIABLO	-0.151337787	9.410910242	9.16991E-06
LEPRE1	-0.199078158	7.182313763	9.20019E-06
NSUN3	0.133282947	6.854591771	9.23901E-06
PDXK	-0.194437871	9.623276219	9.25545E-06
ASL	-0.095116135	6.25092879	9.2996E-06
SNAPC3	0.173593059	7.541912946	9.30709E-06
AXIN2	-0.280841542	8.257864392	9.31643E-06
PNN	0.261904391	9.181427729	9.40682E-06
SCARA5	-0.464816143	7.440804596	9.48233E-06
ELOVL4	0.251616146	7.157032142	9.5689E-06
ZNF644	0.141336395	7.076565041	9.57297E-06
EPDR1	0.43841618	11.09565953	9.68954E-06
ERN1	-0.141288336	6.705575482	9.75384E-06
CDC42BPB	0.221829697	8.123318989	9.84153E-06
PNO1	-0.277477128	7.308778491	9.8952E-06
TNNC2	0.124594865	6.112900887	9.93013E-06
GLTSCR1	-0.147518968	7.663282996	9.9576E-06
C11ORF48	-0.256958007	8.265095008	1.01108E-05
LOC648605	0.128245167	6.594751842	1.01264E-05
SRM	-0.270563476	8.542383042	1.01266E-05
HMG20A	0.187647869	7.268505282	1.01715E-05
DERL1	-0.261283848	9.049812939	1.01945E-05
SIRT1	0.238022506	7.87063066	1.03153E-05
LOC644096	0.131950035	7.400138017	1.04077E-05
C4ORF14	0.165384731	8.68146049	1.0423E-05

ISCA1L	0.217290543	8.651972611	1.04245E-05
HOXD12	0.185213885	7.963724239	1.05588E-05
UBR1	0.189520048	6.627269959	1.05653E-05
RHOT2	0.249850229	8.557212287	1.06305E-05
CARD8	-0.268025431	7.471520882	1.06642E-05
ELOVL6	-0.490827841	6.790172972	1.07967E-05
CSF1	-0.071527502	6.081274861	1.08457E-05
SCAMP3	-0.176651733	9.441708335	1.08635E-05
NBEAL2	-0.191595968	6.865143582	1.0872E-05
IL24	-0.253211114	6.077172207	1.09244E-05
GPR64	-0.153278809	6.312873999	1.09609E-05
C17ORF75	0.316637778	7.023348451	1.10034E-05
TRIOBP	-0.191888682	8.757274943	1.10102E-05
TMEM70	-0.197379374	6.969355635	1.10474E-05
MAP1LC3B	-0.294686697	10.12414343	1.10559E-05
CDK2AP2	-0.42268176	7.876146909	1.10569E-05
SLC39A14	-0.307480484	9.636454775	1.10649E-05
ACADVL	-0.245900497	11.59903604	1.10807E-05
LOC399888	0.136249552	6.526622363	1.12324E-05
UROD	0.202787036	10.42162356	1.12656E-05
ACSM3	0.149348961	6.388866958	1.13786E-05
ATP5G3	0.262588285	8.219861221	1.14781E-05
MAN2C1	0.227461222	8.148303662	1.15596E-05
COMMD3	0.212940838	11.46808036	1.15958E-05
DDX17	0.292198612	8.986512603	1.1616E-05
DHRS7B	-0.190396289	8.169396248	1.16703E-05
C17ORF63	-0.19178765	7.398745746	1.17062E-05
CXORF56	0.156969794	6.677246328	1.17797E-05
LOC642489	-0.338115443	8.93635301	1.18313E-05
CCT2	-0.44377926	9.714069673	1.18448E-05
OR8H3	-0.147056301	6.549169027	1.19083E-05
ZBTB8OS	0.125820537	6.821336431	1.19416E-05
ATP6V0A2	-0.147442955	7.48314881	1.19588E-05
HNRPC	0.309551633	7.857006718	1.19685E-05
YRDC	-0.306567652	9.669188143	1.19908E-05
MYOT	-0.800006423	9.398312314	1.20013E-05
CLEC4GP1	-0.107143755	6.078890644	1.20298E-05
ZNF444	0.237291861	6.990898169	1.20433E-05
AKR1C2	-0.290258415	7.336288798	1.20859E-05
HECTD3	-0.188113951	7.551361462	1.20956E-05
ZNF529	0.223196532	8.586360092	1.21408E-05
HSPBAP1	0.151842326	7.19490651	1.21704E-05
CD74	-0.324249088	9.604955277	1.21744E-05
SCARNA9	-0.175202591	6.455856447	1.21864E-05
TTC17	-0.19632567	7.485221071	1.22078E-05
TPO	-0.130238539	6.153623345	1.22802E-05
PADI4	-0.141310292	6.133367913	1.23269E-05
MST1	0.212498647	7.12021272	1.24049E-05
PIK3R4	0.147305599	6.868370222	1.24723E-05
PTTG2	0.201905811	8.242334129	1.24871E-05
ZNF451	0.131780042	7.190779925	1.25106E-05

G6PC3	-0.203891147	7.667211694	1.26211E-05
AP3D1	-0.163973879	8.145112115	1.26285E-05
IDH2	-0.314068034	11.79279149	1.26513E-05
AGT	-0.252138681	9.036667602	1.26656E-05
MACF1	0.212958463	8.723170986	1.26907E-05
NEDD1	0.107232792	6.442527851	1.27166E-05
SLC27A1	0.311747387	8.86759593	1.28433E-05
BCL2L12	-0.136253192	7.392064791	1.28996E-05
TPCN1	0.186951549	6.742101904	1.2927E-05
IRS2	-0.457617891	8.520042268	1.29904E-05
PAQR6	0.079133766	6.105672256	1.29904E-05
MLXIPL	-0.114504016	6.246007932	1.2995E-05
LOC440160	0.207173816	6.379605653	1.30469E-05
LOC387867	-0.318274316	11.41878081	1.31085E-05
LOC650152	-0.362839042	10.45352994	1.31092E-05
CDS2	0.230469806	8.596274791	1.32173E-05
CNPY3	-0.129864169	6.823722576	1.32578E-05
IL3RA	-0.143886855	6.489960361	1.32927E-05
TP53	-0.129109192	6.293229727	1.33397E-05
SNORD36A	-0.15652376	6.380583766	1.33947E-05
LRRFIP2	-0.209652883	9.210436333	1.34094E-05
PTTG3	-0.212822083	6.51901183	1.34674E-05
GHR	0.340988228	8.579531437	1.34962E-05
RASIP1	-0.297211787	8.633125006	1.35272E-05
CCDC111	0.160721457	6.888521505	1.35422E-05
LOC729905	-0.15400423	6.438790722	1.35456E-05
EPN1	-0.201132741	11.82291694	1.35802E-05
C9ORF164	0.253918802	7.368780548	1.35938E-05
AGK	0.196860482	9.039623197	1.35969E-05
STK32B	-0.158526338	6.35382894	1.36125E-05
TMEM57	-0.125255544	6.846565694	1.36328E-05
NHP2L1	-0.185356178	9.816799334	1.36964E-05
TAGLN	-0.519428324	10.22267939	1.37334E-05
DOK7	0.254125464	7.688768525	1.38038E-05
SALL2	0.173506179	6.834175314	1.38482E-05
MRPL53	0.265910407	9.839462524	1.3887E-05
GRHPR	0.219298609	8.82999705	1.39124E-05
CEP120	0.156404427	6.442466552	1.39636E-05
PPM1F	-0.278420834	9.381821357	1.39791E-05
LOC643720	0.137014526	6.165463988	1.40261E-05
HERC4	0.128695066	7.395518226	1.40954E-05
IIP45	-0.148290506	6.606992359	1.41282E-05
IKBKE	-0.112731867	6.176481746	1.41347E-05
SF3B4	-0.227758002	9.313157213	1.41984E-05
SGCE	0.307220299	8.869209678	1.42919E-05
NME3	0.231538203	9.267361726	1.43112E-05
LOC727751	0.123957455	6.310194481	1.43133E-05
SLC27A6	0.388900335	7.453403855	1.43426E-05
AP1G1	-0.20731152	7.598358406	1.43615E-05
MRCL3	0.371469304	14.65730292	1.43756E-05
TACC2	0.301306814	10.15447	1.43871E-05

CDKN2AIPNL	-0.319703689	11.78575296	1.44823E-05
PCDHB4	-0.167833855	6.628659931	1.44875E-05
NTNG2	0.14563954	6.152792102	1.45047E-05
ABCA2	0.15975082	6.451332938	1.45611E-05
VAV1	-0.114966222	6.198181485	1.45618E-05
DHRS12	0.110156647	6.714724691	1.45899E-05
SERINC2	-0.143611012	6.040757271	1.46825E-05
PMS2	0.085700194	6.411307035	1.4768E-05
C16ORF63	0.194476588	9.761878478	1.47699E-05
GBP2	-0.351668164	8.776216583	1.47792E-05
LOC645550	0.1947511	6.53249936	1.4789E-05
SPRY2	-0.313485822	9.700577911	1.50013E-05
RANBP1	-0.225727883	9.129066482	1.50157E-05
ABHD1	0.217971998	6.951790192	1.51305E-05
GALNT11	0.348378023	8.443687737	1.53693E-05
LOC652455	0.17592223	6.597661822	1.53858E-05
FAM171A1	-0.209790106	8.507349521	1.53951E-05
CENPM	-0.103206759	6.131160176	1.55772E-05
B9D2	-0.197418184	7.181376545	1.57256E-05
C6ORF115	-0.280129402	7.087267658	1.57684E-05
LOC389137	0.158673715	7.3806445	1.58387E-05
SPHKAP	0.564248657	10.43082443	1.58441E-05
MEF2C	0.29190039	7.968337485	1.58743E-05
MEPCE	-0.23061886	8.863138276	1.58971E-05
LOC647856	-0.28594206	10.69321855	1.59371E-05
IFI27	-0.33759123	10.92910186	1.5946E-05
LOC642267	0.100096875	6.161741326	1.60038E-05
ANKZF1	0.106918022	6.741890835	1.60783E-05
SLC20A1	-0.32700383	8.667062199	1.62091E-05
GLYCTK	-0.107083512	6.553594133	1.62601E-05
FJX1	-0.235812751	6.635334741	1.65497E-05
UST	-0.168143907	6.555984101	1.67466E-05
BAK1	-0.228099605	6.813059526	1.68093E-05
FLJ20718	-0.162603554	7.29364662	1.68214E-05
SMARCA5	0.223555203	7.724884982	1.68398E-05
ZKSCAN3	0.128083569	6.59029697	1.68529E-05
RNPC3	0.162064211	6.947189316	1.6968E-05
HRSP12	0.218313987	8.042431883	1.69711E-05
KLF9	-0.30731822	11.3586261	1.69781E-05
COLEC11	0.250623648	6.841509116	1.70575E-05
FGF9	0.247451125	8.676883641	1.70693E-05
VHL	-0.121002634	7.423776806	1.70731E-05
MICB	-0.163924531	6.512515779	1.70815E-05
EVI1	0.333832221	8.707814268	1.70888E-05
DLAT	0.259548966	9.882049502	1.7107E-05
LOC651881	-0.114783913	6.2863814	1.71205E-05
EIF2S3	0.23167565	10.26002141	1.72076E-05
UCHL3	-0.304730605	8.912332417	1.7373E-05
GPBP1L1	0.153205878	7.645535697	1.74114E-05
DRD4	0.236511565	6.598037053	1.74146E-05
TRAPPC2	0.116507959	6.930226702	1.74658E-05

PLA1A	-0.238893028	6.480901706	1.75181E-05
LOC654069	0.120534097	6.199079402	1.7562E-05
TLCD1	-0.141469949	6.158491118	1.76502E-05
SLAMF6	-0.109072404	6.156523293	1.76875E-05
PMS2L2	0.130908292	6.365678179	1.77026E-05
TIA1	0.170901833	7.943446972	1.78051E-05
SPRYD3	-0.214601923	9.459045203	1.78964E-05
SHMT2	-0.272335513	9.461381689	1.79165E-05
CSGLCA-T	-0.150857785	7.043060741	1.79259E-05
LOC654346	-0.126321774	6.153413969	1.79316E-05
FXR2	-0.305398999	8.320972609	1.79541E-05
THOC2	0.167398378	7.851430989	1.79608E-05
TTC5	0.14941176	7.25030591	1.80013E-05
POLE3	-0.243207157	10.44540071	1.80455E-05
ZNF510	0.114789297	6.42830558	1.82308E-05
LOC400406	-0.233553784	6.47446412	1.831E-05
KIAA0133	-0.140801553	6.630934463	1.83416E-05
CNN2	-0.227340467	7.867636457	1.84304E-05
C1ORF168	0.423066643	7.454389467	1.84348E-05
TERF2IP	0.233520729	9.401644145	1.84581E-05
LOC731314	-0.198456103	7.237211043	1.84605E-05
CD63	-0.512909448	8.788861917	1.85448E-05
COBRA1	-0.167810385	7.737970186	1.86723E-05
C6ORF145	-0.209662645	6.416460187	1.87521E-05
POLE2	-0.122249285	6.257356176	1.88467E-05
KCNJ8	-0.250003505	9.671904269	1.88661E-05
CALCOCO2	-0.316921181	8.285390502	1.88718E-05
LOC648749	0.201009916	6.551989232	1.88814E-05
YEATS4	0.256330381	7.982555786	1.89778E-05
AGA	0.188090733	7.284510611	1.90182E-05
NUPL2	0.158408189	8.389539658	1.90333E-05
C9ORF142	-0.172736225	8.860791037	1.91674E-05
SLC38A8	-0.193944631	6.182685419	1.92196E-05
ZFAND5	0.205954783	12.03635124	1.92245E-05
KIF7	0.167933073	6.61024947	1.9244E-05
ANKRD29	0.194784536	6.949839376	1.93077E-05
CCDC104	0.158091296	7.439437017	1.93563E-05
KIAA1324L	0.15834955	6.884585113	1.93626E-05
ATG4B	-0.202809197	7.260102921	1.93685E-05
SP100	0.14956403	6.925984439	1.93796E-05
MAP4K2	-0.192303294	8.714045279	1.94224E-05
LOC650215	-0.231629768	7.930633679	1.94496E-05
INPP4B	0.302950765	8.162047622	1.94995E-05
LRRC2	0.379967526	8.947298579	1.95306E-05
GATA4	0.22799999	7.700248195	1.95375E-05
CYBASC3	-0.154528056	7.092444021	1.95479E-05
KIAA0319L	0.152651037	7.446372076	1.95499E-05
GRIPAP1	-0.291067462	8.882208574	1.95616E-05
LRRC3B	0.076245533	6.104431739	1.9635E-05
SLC2A11	0.113147611	6.691510355	1.98257E-05
EGR1	-0.72408262	8.488727696	1.98507E-05

EMD	-0.208151208	10.31393026	1.99104E-05
RHBDL1	0.161168819	6.473074232	1.99173E-05
HIST1H4E	0.167028558	6.468859648	1.99465E-05
MGC16169	0.172764341	7.393301048	2.00795E-05
GUCA1B	0.151088047	6.51808812	2.01484E-05
LOC728689	0.228267921	7.073633557	2.01577E-05
DDAH1	0.50539273	8.299700322	2.01737E-05
C14ORF153	-0.303588225	9.865563296	2.02358E-05
TRIM56	-0.241793487	7.587600222	2.02856E-05
DYSFIP1	-0.079318551	6.085066383	2.03003E-05
MLL	0.177443642	7.030238328	2.03374E-05
NMRAL1	-0.441846586	8.599322781	2.0351E-05
LOC653226	0.226662359	10.91573745	2.04947E-05
FTO	0.20734218	7.949863893	2.05112E-05
UTP18	-0.183857728	6.807401887	2.07775E-05
CHD6	0.224181437	7.152476593	2.07885E-05
PRUNE	0.228127612	8.354294009	2.08073E-05
HLA-DRB6	-0.408140426	7.988851437	2.09075E-05
ATP6V1A	-0.220683923	9.252912466	2.09949E-05
C5ORF13	0.429311247	9.819474661	2.10903E-05
RASGRP2	-0.132130576	6.50465863	2.11196E-05
PLEKHA4	0.339154585	8.053155759	2.11841E-05
C18ORF55	0.19548183	11.11890328	2.1211E-05
TRAM1	-0.238235459	11.19906769	2.12638E-05
GOLGA6B	0.164333794	6.530768	2.12867E-05
C3ORF18	0.29139631	7.477479332	2.13298E-05
TMC6	0.306074442	8.329310816	2.13712E-05
C14ORF180	0.459071155	9.460729801	2.13725E-05
SLFN11	-0.197209996	8.258050533	2.13998E-05
HLA-DMA	-0.389144244	9.698931992	2.14378E-05
IGSF6	-0.183806499	6.253375257	2.15132E-05
ECD	0.129044379	7.155470069	2.15434E-05
LOC221442	0.209122812	6.991675801	2.15631E-05
OXSR1	-0.226816944	9.643094846	2.16108E-05
LOC645937	-0.14888596	6.736444614	2.16964E-05
MKRN1	0.189659757	11.06098221	2.17561E-05
ZKSCAN2	0.181523522	7.856353799	2.17833E-05
TFF3	0.39707575	7.553332907	2.17855E-05
PDE6A	0.122169788	6.183062727	2.18877E-05
LOC653082	-0.163626857	6.258515171	2.19342E-05
S1PR4	-0.115343865	6.194343574	2.20946E-05
WDR27	0.125334731	6.398016944	2.2112E-05
S100PBP	0.079065658	6.736335044	2.21809E-05
RBMS3	0.12484526	6.642795399	2.22567E-05
LOC201725	0.157500264	6.692703106	2.22657E-05
MAMDC2	0.387072407	8.323415473	2.22754E-05
PCDH20	-0.584662639	7.049332018	2.22759E-05
SNORD4A	-0.129375835	6.266141702	2.2288E-05
JMJD6	-0.120042538	6.466255696	2.23314E-05
CDK6	-0.254001317	7.855801125	2.24983E-05
EXOC6	0.090177239	6.762429132	2.25134E-05

BCDIN3D	0.147973844	7.739722508	2.25446E-05
MYO5B	0.092940532	6.151861458	2.28071E-05
CXCL1	-0.177016278	6.14082363	2.28125E-05
CPNE4	-0.699600245	7.87995378	2.28188E-05
EBPL	-0.240997804	7.441794099	2.28772E-05
CCNDBP1	0.256584472	9.918120653	2.29738E-05
VDAC1	0.188184897	12.3072244	2.30418E-05
LOC643031	0.26451811	13.69949142	2.31187E-05
IL1RN	-0.104623131	6.187518748	2.31702E-05
MICALL2	-0.10519123	6.36753231	2.32025E-05
RPS5	-0.224514478	12.40257359	2.32073E-05
LRRC20	0.323392639	9.01931273	2.32337E-05
TUBG2	0.206044859	8.138903054	2.32701E-05
SERHL	0.066155487	6.24998773	2.33225E-05
RBMS2	0.214892731	7.276528261	2.35177E-05
TRIM24	0.135413588	7.081637208	2.35829E-05
ERCC1	-0.19073498	9.390740451	2.36802E-05
REXO2	0.159782267	11.96310506	2.37625E-05
ZNF200	0.093876119	6.688338326	2.37964E-05
HCG4	-0.306052466	7.737984559	2.38112E-05
SERTAD2	0.208928032	9.944466818	2.38149E-05
FKBP1A	-0.250427319	8.574808734	2.39906E-05
NME4	0.244879964	9.642832577	2.4027E-05
NFKBIB	-0.12833747	6.746690768	2.41446E-05
IMMT	0.205760029	10.87189081	2.42099E-05
HELLS	-0.085159326	6.037689186	2.43376E-05
B4GALT7	-0.209310578	7.983661822	2.43939E-05
LOC650826	0.139870808	6.480709104	2.44879E-05
PPHLN1	0.12718491	7.619313581	2.4494E-05
MRPS26	0.195989878	9.687768848	2.4533E-05
CSNK1E	-0.186619054	9.108989843	2.45752E-05
LOC153561	-0.183635413	6.845836299	2.45852E-05
PDE3B	0.166573463	6.589765159	2.47775E-05
CABP1	-0.067575084	6.101219314	2.47942E-05
ITGAX	-0.109369477	6.155166596	2.48714E-05
WIBG	-0.147734233	7.088168204	2.50529E-05
TPD52L2	-0.23108145	9.818190425	2.50564E-05
TCAP	-0.39584082	12.75881558	2.5059E-05
ARL1	0.169775741	9.923974238	2.50921E-05
PLCB4	0.191838148	6.855689423	2.51514E-05
C2CD2	-0.198090624	7.511435331	2.52599E-05
CHMP4A	0.198851483	8.908006927	2.5268E-05
RAPSN	0.150280249	6.726900034	2.53724E-05
COQ5	0.278632895	10.29718376	2.54404E-05
POMZP3	0.398608135	6.798226113	2.5484E-05
ZBED5	0.24257206	9.071368623	2.54857E-05
EMILIN3	0.192220438	6.507680232	2.55488E-05
TXN	-0.254097653	10.61617035	2.55604E-05
TCEA2	0.176780371	7.482046147	2.56404E-05
NR4A3	-0.140943496	6.101120892	2.60158E-05
KCNN3	0.131696648	6.455730696	2.60337E-05

SLC35E3	0.195177232	8.114282671	2.61214E-05
C10ORF54	-0.312618641	7.929517232	2.61229E-05
NRBP2	0.271297348	8.616157771	2.61716E-05
BCKDHA	0.247634854	10.50002489	2.61774E-05
TUSC3	0.233699413	8.112040869	2.62244E-05
DNLZ	-0.291185524	9.804191322	2.62715E-05
FCER1A	0.408860778	7.242949066	2.63978E-05
UCK2	-0.478910834	7.777243223	2.64589E-05
MTHFS	0.208754096	8.296166717	2.65832E-05
MBD5	0.077105261	6.311719701	2.66599E-05
TMEM139	0.174668444	6.323301092	2.66696E-05
WBP2	-0.224081283	12.39594507	2.67786E-05
TBRG4	-0.189500656	8.22255142	2.70566E-05
MRPL36	-0.200118257	10.56947419	2.71845E-05
TGFBI	-0.384227133	10.35901771	2.73439E-05
SLC29A1	0.287427857	8.358069914	2.73859E-05
LOC647834	0.157239071	6.879277191	2.7421E-05
SNORD36C	-0.153796955	6.240191788	2.74391E-05
KIAA0195	0.199297279	7.506187992	2.74975E-05
GALNTL4	0.30284665	8.161342104	2.75739E-05
UBE2D3	-0.202923804	10.09125505	2.75864E-05
CATSPER2	-0.338465688	10.63488474	2.76506E-05
JUP	0.24701658	9.11067444	2.76696E-05
CGNL1	0.502020131	10.38395395	2.77822E-05
SOX6	0.087657902	6.136455143	2.78723E-05
XRN2	-0.20004403	7.401311671	2.79964E-05
HDGFRP3	0.214702222	7.81135302	2.81449E-05
FYN	0.119276275	7.415519291	2.81899E-05
QKI	0.170556126	7.232848268	2.82256E-05
LCN2	-0.151479637	6.063513975	2.82535E-05
C11ORF65	0.107224616	6.305436899	2.83076E-05
GPAA1	-0.207796665	8.692639315	2.83183E-05
ZNF259	-0.296038651	7.395572896	2.83355E-05
KLHL4	0.08258651	6.199604236	2.83505E-05
SNX16	0.171874559	7.003712299	2.83927E-05
MRPL11	0.144530592	9.213486777	2.83947E-05
RP2	-0.185978085	6.583578751	2.84052E-05
USP12	-0.18220809	6.111111053	2.8488E-05
PRDX3	0.328958038	11.28767791	2.84882E-05
PLN	0.330043209	10.43330644	2.84894E-05
CACNA1B	0.358857222	8.46338235	2.86384E-05
PACS1	0.278971947	7.865711813	2.86735E-05
TLE4	0.12376291	7.200163903	2.891E-05
LACTB2	0.358136145	9.889550161	2.89841E-05
DCTN1	0.225070546	9.02622462	2.90198E-05
MRPL3	-0.223458488	9.750835034	2.90344E-05
CAPN2	-0.126858519	6.958698205	2.90847E-05
C10ORF119	-0.14354594	6.510243913	2.91102E-05
LIMS1	-0.244031532	8.498680725	2.91532E-05
LOC390705	0.216331896	7.748970835	2.91541E-05
PAIP2	0.25402765	9.664648043	2.91641E-05

LOC650111	-0.129053325	6.338618252	2.93866E-05
COQ10A	0.411040666	10.83806206	2.94105E-05
COASY	-0.154814252	8.209345954	2.94308E-05
ASB13	-0.134788709	7.349443026	2.94417E-05
WBP4	0.165391098	9.254550189	2.94523E-05
RHBDL3	-0.194574714	6.35755567	2.94607E-05
ITGA2	-0.241983121	6.378624873	2.97754E-05
ANXA7	0.199597455	9.755679809	2.97999E-05
BAT1	0.201331688	8.831915437	2.98753E-05
TNFRSF25	0.188091988	7.202385437	2.99067E-05
MYL3	-0.225099391	14.20952614	2.99699E-05
NUAK2	0.081380102	6.073690536	2.99917E-05
KLHL11	0.085992656	6.243960403	3.00844E-05
DDX3X	-0.25955238	9.293852132	3.00882E-05
USP15	0.238632119	8.125331254	3.01289E-05
SMARCC1	-0.263354357	7.949363945	3.01361E-05
THAP4	-0.136293294	6.358279546	3.02491E-05
PEBP1	0.314009188	10.74747863	3.02563E-05
FGFR3	0.210354049	7.029120869	3.03167E-05
LOC51057	0.125603921	6.323332647	3.04827E-05
PTPRD	0.185616461	6.924382989	3.05642E-05
SH3GLB2	0.208216434	9.578011704	3.05891E-05
LOC388524	-0.213537883	12.09646143	3.06353E-05
LOC729776	0.236132372	9.052531659	3.07714E-05
TXNL2	-0.246354416	8.575108623	3.07794E-05
LOC643665	0.12981686	6.534747282	3.08991E-05
TTLL12	-0.214750282	7.191587621	3.09651E-05
ATG7	-0.144639352	7.902167997	3.09674E-05
C3ORF34	-0.300800955	10.65228614	3.11351E-05
BCL9L	-0.206476109	6.680507089	3.1224E-05
PAK1IP1	-0.224492771	7.408612315	3.12792E-05
ZBTB17	-0.218740368	7.687471357	3.13055E-05
COL20A1	0.132247089	6.522406137	3.13313E-05
KIAA0430	0.189998701	9.808037285	3.15977E-05
ZNF441	0.118194502	6.172299313	3.16164E-05
FNTB	0.191371371	8.486541845	3.17138E-05
TLR2	-0.114600084	5.963211546	3.17608E-05
C7ORF50	0.218190208	11.19329945	3.18577E-05
IQGAP1	-0.22463916	7.706999009	3.1987E-05
DMAP1	0.17038881	8.537780009	3.20209E-05
HIGD2A	0.327546667	11.50870344	3.21159E-05
KLHL28	0.167999505	7.303622752	3.21369E-05
EXOC1	0.094713855	7.224878789	3.21712E-05
CIDEC	-0.383835164	6.342252793	3.23698E-05
THTPA	0.143989018	6.471397578	3.25362E-05
ERLIN2	0.091887055	6.830739206	3.25459E-05
CCNJ	-0.147217926	5.895549109	3.25884E-05
AQP7P2	0.341838817	8.529686202	3.26084E-05
BBX	0.23677243	9.033744814	3.26663E-05
GSTP1	-0.169170751	11.56720716	3.27025E-05
TCF20	-0.069589607	6.819559138	3.27198E-05

OXCT2	-0.368303447	7.533521106	3.28004E-05
KIAA1618	0.141677329	7.282247354	3.28062E-05
FAM14A	0.355899577	8.823372698	3.29369E-05
CALD1	0.207115621	9.961637384	3.29737E-05
BCL2L1	-0.282557642	9.417965201	3.29897E-05
UBR5	0.122797773	7.802877373	3.30885E-05
CEP350	0.22358981	7.862713765	3.30972E-05
NET1	0.174724177	7.445947681	3.3201E-05
RETSAT	-0.379592124	6.94239597	3.33779E-05
TK1	-0.131960303	6.298648806	3.33939E-05
NMT2	0.168585259	7.879834222	3.3418E-05
DEF6	-0.1421465	6.401166724	3.3451E-05
MTX1	-0.107714649	7.631821848	3.3661E-05
RPIA	-0.206036837	8.519516734	3.41311E-05
APEX2	-0.150009657	7.828867913	3.43659E-05
MTX2	0.181629006	9.678971101	3.43709E-05
LOC648304	0.10403956	6.279365364	3.44956E-05
LRBA	0.178943374	7.886689222	3.45084E-05
STK17B	-0.092237185	6.118739869	3.45636E-05
TUBB4	-0.114635471	6.200311098	3.46525E-05
LOC285053	-0.266486138	12.19212557	3.47142E-05
FAM40B	-0.244875225	7.559491554	3.47906E-05
DKK1	-0.259504172	6.137425891	3.48589E-05
PGR	0.106103623	6.381411087	3.48832E-05
MED27	-0.127935989	7.028838162	3.48973E-05
TEK	-0.252584444	8.963712364	3.49083E-05
SGMS2	-0.283986274	6.42981682	3.4961E-05
SP4	0.204886631	7.13772487	3.50041E-05
BMS1	-0.185188285	8.656814789	3.50087E-05
C14ORF93	0.147210731	7.570092737	3.50433E-05
C16ORF80	0.245479838	9.070229988	3.52834E-05
HMBS	-0.144409228	7.615969677	3.53467E-05
ZFP91	0.142999186	9.959845422	3.54813E-05
PSMC3IP	0.080596693	6.156199687	3.55923E-05
ZNF428	-0.374110978	10.20958026	3.56454E-05
ALG8	-0.116694116	7.540833574	3.5737E-05
PPP1R12C	0.283137565	7.609926462	3.57728E-05
SOD2	-0.241676098	8.791239075	3.61971E-05
IGFBP3	0.521583226	9.27609558	3.62673E-05
ARC	-0.172009301	6.289023623	3.64259E-05
ERMP1	0.316968268	7.475051632	3.65061E-05
LOC652815	0.216200007	6.709907928	3.67271E-05
COX17	0.195355104	12.46809691	3.67472E-05
CXORF45	0.165466437	7.200258175	3.69068E-05
BRPF3	-0.201496453	8.225433741	3.69185E-05
AP1G2	-0.096762533	6.262258865	3.69255E-05
SCYL1	-0.154624932	9.87343688	3.70928E-05
DUSP19	-0.316756539	10.98115387	3.7184E-05
KCNC4	0.07403996	6.157676539	3.72803E-05
LOC647210	-0.157583407	6.498269746	3.72868E-05
LOC149134	0.176748728	6.709663684	3.74386E-05

MUT	0.220597761	8.403045995	3.74679E-05
SAFB	-0.18694172	9.208508894	3.74916E-05
TOP2B	0.209241917	10.35530838	3.75016E-05
KIAA0556	0.202867734	7.898761017	3.76777E-05
ASIP	0.557113277	9.515075376	3.78041E-05
DSEL	0.113922494	6.300986209	3.78879E-05
PGS1	-0.142315702	7.801730722	3.81742E-05
PIGK	0.212296147	7.527354848	3.8177E-05
DHX34	-0.108410916	6.584207964	3.82068E-05
AKNA	-0.15082575	6.71582335	3.82887E-05
TBX15	-0.123974529	6.24384007	3.83084E-05
SNORD14B	-0.113035936	6.080204706	3.83265E-05
PALLD	-0.189746337	12.18792331	3.85546E-05
RNF11	0.150977074	7.348326514	3.85603E-05
TNFSF4	-0.095469215	6.252588009	3.87117E-05
PCNA	0.16097577	7.329871818	3.87903E-05
RPL7L1	-0.228884971	11.57082034	3.88642E-05
P4HB	-0.206702283	10.86663634	3.88695E-05
RUVBL2	-0.235110721	7.875549986	3.91298E-05
SRP14	0.190003067	12.4048025	3.95028E-05
MAPRE2	0.25516037	10.57050537	3.95218E-05
CTHRC1	0.46650225	7.949652208	3.95284E-05
CYP2R1	-0.153115664	7.096015809	3.9671E-05
XPO6	-0.169571721	7.630012349	3.97423E-05
P8	0.372934897	7.913755395	3.97477E-05
TRIB3	-0.244690436	6.68432574	3.98273E-05
ASXL1	-0.134757917	7.346644213	4.01259E-05
ALKBH2	-0.183782452	7.501497256	4.04648E-05
WDR69	-0.147304584	5.983221572	4.05838E-05
ARL15	0.129274904	6.829305786	4.064E-05
C16ORF58	-0.219214158	9.15974831	4.0699E-05
TLL1	-0.170584749	5.963586717	4.09836E-05
MOSPD2	0.112963217	6.746724457	4.1073E-05
AVEN	-0.205805444	7.760507013	4.17862E-05
FLJ36070	0.237918238	6.965825507	4.181E-05
RNASEL	0.176104698	7.311360363	4.20277E-05
SIRT7	-0.115546642	6.224037658	4.23927E-05
MESDC1	-0.156929369	6.385485943	4.26205E-05
LAMB2	0.381475754	10.10305687	4.26464E-05
GAS7	-0.215486098	7.225718594	4.26482E-05
SP110	-0.183669023	7.457398069	4.26492E-05
ODF2	0.126209806	7.120048796	4.26604E-05
KRT10	-0.243876439	9.502323429	4.27795E-05
ZFP1	0.148009193	6.516765925	4.27909E-05
TBL2	-0.152083558	7.143851783	4.27933E-05
MTERFD1	-0.221860268	9.036469592	4.28501E-05
LOC646567	-0.312727098	8.426012326	4.33513E-05
C12ORF45	-0.193468322	7.427308844	4.37578E-05
FLJ46230	-0.14620253	6.476905136	4.39201E-05
MICAL1	-0.165332518	6.689571914	4.39935E-05
C6ORF173	-0.138916396	6.509169435	4.40105E-05

NUP85	-0.15492992	8.283680786	4.4085E-05
DCUN1D1	0.148479241	6.808938937	4.41634E-05
RPL18A	-0.199204974	13.33280196	4.45516E-05
XRCC2	-0.306066277	8.657671776	4.46062E-05
GMEB2	-0.15792553	8.234261705	4.46444E-05
UBR2	0.140890775	8.427577191	4.46924E-05
TUBA8	0.242902493	6.850026534	4.4792E-05
ABCA10	0.177053279	6.458040423	4.49761E-05
DCTN4	0.218794228	8.60205762	4.50079E-05
GNPAT	0.136282421	9.328854473	4.51739E-05
C6ORF47	-0.294062911	7.547479399	4.56037E-05
FABP6	-0.155429486	6.275951015	4.57262E-05
P2RY6	-0.081562418	6.324221901	4.60365E-05
INPPL1	-0.219764155	9.737745814	4.61083E-05
TMEM14B	0.295649417	11.2085798	4.62177E-05
PTGS1	-0.152597967	6.579638256	4.65105E-05
EFHC1	0.129833585	6.653697594	4.65806E-05
PTPRE	-0.231940184	8.056921912	4.65936E-05
C12ORF49	-0.133108328	6.259691334	4.66389E-05
DNAH1	0.144727675	6.69823435	4.69427E-05
TMED5	-0.363303392	9.301139469	4.69742E-05
ANKRD40	0.135071052	7.012491768	4.70353E-05
FANCG	-0.173593444	6.84782503	4.70943E-05
HOOK1	-0.195153056	6.591430554	4.71355E-05
CHCHD7	0.201059326	8.247233095	4.71522E-05
DENND1B	0.117679001	6.119032507	4.72668E-05
C9ORF86	-0.188883258	8.023949607	4.76634E-05
YARS2	-0.123016175	8.713722757	4.77773E-05
PPAPDC2	0.118457664	6.796868311	4.82212E-05
IRAK2	-0.231320996	6.892718323	4.83226E-05
C6ORF1	0.097890391	6.550041599	4.84234E-05
SUPT16H	0.206748288	9.187806927	4.84651E-05
PUS10	0.094736943	6.223029831	4.87838E-05
SMCR7	0.102977657	6.201682991	4.89374E-05
ANGPTL7	0.16356321	6.394061703	4.94926E-05
TBX3	0.188988399	6.884741455	4.95447E-05
EPPB9	0.19331779	6.405283799	4.95688E-05
ITFG1	0.22760735	8.670781849	4.96098E-05
CASQ2	0.222988819	13.59459826	4.9712E-05
OAT	0.193846204	8.872006004	4.9734E-05
TRIM29	0.190259027	7.511729128	4.982E-05
PCNP	0.210572119	10.58930854	4.98467E-05
RLN2	0.089332765	6.08861551	4.99069E-05
LOC644254	0.218040203	6.411022295	5.03216E-05
SMC3	0.13341967	7.732539805	5.03317E-05
WDR35	0.118462473	6.731020378	5.0489E-05
ANO10	-0.146006783	6.877614598	5.06058E-05
ZNF137	0.114889743	6.419313572	5.07319E-05
CSNK1G2	-0.321075993	9.524497214	5.09312E-05
PAN2	0.156005582	7.835784761	5.1163E-05
EIF3C	0.255174398	7.197726478	5.13709E-05

TIGA1	0.250078563	9.586406994	5.13988E-05
ARCN1	-0.22789574	9.982403387	5.14003E-05
C15ORF15	0.204052787	10.75742463	5.14538E-05
MAP3K7	0.159635135	8.103432573	5.15077E-05
CBARA1	0.190413781	7.93492534	5.16255E-05
ANO7	-0.086681224	6.345383253	5.17135E-05
SPR	0.234272504	8.086255083	5.20534E-05
LOC441108	0.101931014	6.103350792	5.2065E-05
KLHL30	0.229737065	7.999609934	5.20844E-05
GAS8	0.248141805	7.77242983	5.21667E-05
C1ORF181	0.173876406	8.334403189	5.24075E-05
CLEC7A	-0.069207768	6.108302435	5.246E-05
C1ORF163	-0.15495775	7.19514286	5.25094E-05
PTX3	-0.187784153	5.983427566	5.26477E-05
TMEM86A	0.168165728	6.366001722	5.29094E-05
SOX13	-0.180624862	7.392990682	5.33278E-05
OAZ1	-0.134809712	14.10520209	5.38662E-05
LOC653505	-0.287806241	9.671444161	5.39214E-05
C3ORF45	0.38554456	7.463248645	5.39984E-05
ZCWPW1	0.197472178	7.480040176	5.4042E-05
ANXA2P1	-0.488348669	9.007658026	5.41672E-05
NCOA2	0.174942758	6.356027841	5.41811E-05
R3HDM2	0.163012801	7.023008842	5.42689E-05
ZSWIM5	0.136985976	6.365149902	5.44942E-05
PPP1R16B	0.259010007	7.469288576	5.48538E-05
BEXL1	0.269758311	9.356588924	5.50653E-05
CPXM1	-0.138445198	6.234053803	5.55406E-05
CDGAP	-0.205537495	6.809988566	5.56056E-05
LCMT2	0.172486775	7.497217884	5.59951E-05
RAB7A	0.177422414	11.88859616	5.61671E-05
KIF1A	0.331073752	6.647990628	5.63124E-05
IFT81	0.086195176	6.284965442	5.63372E-05
COL5A3	-0.187148226	6.441308691	5.635E-05
PSMD2	-0.216681226	9.880236736	5.6475E-05
TCEAL2	0.256131989	6.616333281	5.64907E-05
LOC728153	-0.155386354	7.36688681	5.65439E-05
UTX	0.205332996	7.143183283	5.66362E-05
NXN	-0.184130009	7.388523908	5.68728E-05
PMP22	-0.202391809	10.36696655	5.73618E-05
SNORD46	-0.149526368	6.069632984	5.74284E-05
NOL14	-0.178106432	7.575096435	5.7464E-05
LOC648984	-0.153998967	6.434555305	5.75174E-05
MEGF9	-0.104150796	6.144401713	5.78958E-05
C15ORF57	0.239412665	7.174742302	5.80083E-05
CWF19L2	0.161783475	7.64888236	5.81315E-05
C1ORF122	0.215124751	9.077416971	5.82176E-05
ESRRA	-0.193687407	8.91669745	5.82258E-05
N4BP2L1	0.115520557	6.540606585	5.82703E-05
POLR2L	0.199406154	7.692799844	5.83917E-05
NAPEPLD	0.194593955	6.789467815	5.84763E-05
ALDH3B1	0.092125458	6.338462152	5.89938E-05

SLC6A18	0.130757632	6.383186475	5.90489E-05
BXDC1	-0.261668981	9.9005104	5.93735E-05
KIAA0391	0.150857241	8.807459012	5.9467E-05
ZMYM1	0.158096863	7.190925502	5.95598E-05
PCNX	-0.15904661	8.698280796	5.97213E-05
FOSB	-0.591727222	6.906219586	6.00981E-05
SYNJ2	0.151576891	7.133051449	6.01108E-05
PI4K2B	-0.108313788	6.414906065	6.03011E-05
C7ORF20	-0.092001732	6.727097132	6.06041E-05
TBCC	0.179741638	7.860280162	6.07409E-05
TRIM68	0.134024925	8.37279718	6.08895E-05
CSTF3	0.111101275	7.704608958	6.09371E-05
TCEB1	-0.219918092	10.74046156	6.11979E-05
BRPF1	-0.145364793	8.934742974	6.14401E-05
CENTA1	0.252367527	9.079875976	6.2021E-05
LOC401115	-0.385170996	8.568151362	6.21919E-05
IRX4	-0.260576412	8.57640509	6.23326E-05
SRXN1	-0.285620267	8.079253851	6.25026E-05
KIAA0892	0.22162335	7.99813148	6.26632E-05
WDR91	0.125986191	6.58476599	6.27652E-05
CASP1	-0.166994238	7.171619624	6.28852E-05
NDUFB4	0.09355506	6.254105624	6.29623E-05
MCM4	0.169055992	7.629456809	6.31093E-05
HPSE	-0.092841723	6.131004774	6.31455E-05
SLC25A5	0.258089016	12.35221701	6.35578E-05
DUSP3	0.280096034	12.0008992	6.43005E-05
RTN4IP1	0.219550241	8.468581182	6.4456E-05
FLJ39639	0.13102059	6.841784386	6.45591E-05
KIFAP3	0.267214999	9.703833084	6.46411E-05
UBD	-0.218971346	6.123658148	6.4678E-05
B4GALT4	-0.109794216	6.891859062	6.49274E-05
NKX2-5	-0.274981098	9.949721294	6.50588E-05
MAPK3	-0.181464441	9.141120825	6.50813E-05
LOC641978	0.106416043	6.396192384	6.51063E-05
GMIP	-0.139742935	6.125316644	6.56181E-05
GCNT1	-0.083466122	6.21198931	6.56527E-05
GTPBP8	0.191302285	8.272085698	6.58144E-05
CD4	-0.12003613	6.327051393	6.58831E-05
C3ORF38	0.160329902	7.150443199	6.60036E-05
C21ORF122	0.177177043	6.869501541	6.63895E-05
TCN2	-0.198807359	7.044695361	6.6512E-05
CENTG2	-0.194283446	7.731250322	6.67085E-05
LASS6	0.241738607	10.70953822	6.6733E-05
FLJ23834	-0.222712972	6.085045139	6.67453E-05
REEP1	0.251546502	8.906842138	6.67991E-05
CHEK2	-0.072290864	6.197433919	6.69595E-05
RAB6A	0.152872723	7.579492208	6.73086E-05
ITGA10	0.319053495	7.208410556	6.73134E-05
HIST3H2A	0.383313831	7.840981705	6.73424E-05
ZNF266	0.098224942	6.928137976	6.7972E-05
PRPF40B	0.104674849	6.361445528	6.81452E-05

JTB	-0.237093061	11.67862839	6.84752E-05
MXRA7	0.188223828	8.672356542	6.8534E-05
METTL9	0.084376686	6.724087747	6.9036E-05
C3ORF19	0.163318098	8.34907158	6.90705E-05
LOC644869	0.155683183	7.436609521	6.91124E-05
TLR5	-0.153356143	6.608253748	6.91651E-05
RXRA	-0.221781474	9.788437386	6.9245E-05
RFTN2	0.227588543	7.504599629	6.93982E-05
CTSH	-0.220161399	8.744902497	6.95503E-05
CENTD1	0.284482189	7.456028728	6.98786E-05
KIAA0251	-0.100417045	6.259296789	7.06626E-05
SAV1	0.177789544	7.242355705	7.0699E-05
RUFY2	0.073652035	6.436577781	7.09268E-05
UBE3C	-0.143290538	9.374408683	7.11063E-05
GANAB	-0.241570025	7.413580092	7.12832E-05
LYPLA2	-0.129997835	7.196013326	7.13917E-05
SFRS2	-0.176785345	10.83815556	7.15453E-05
ADO	0.204834504	8.680798631	7.17269E-05
FMO3	0.251040224	6.878660207	7.17964E-05
RNASEN	0.139963372	7.6190997	7.18088E-05
C8ORF55	-0.256213368	8.994250515	7.18593E-05
DBNDD2	-0.24268447	12.42614176	7.19631E-05
LOC85389	-0.129148263	6.182404882	7.22077E-05
MON1B	-0.150363437	6.824011142	7.222E-05
C14ORF149	-0.268536199	7.316014615	7.28897E-05
RGS18	-0.140561416	6.41160877	7.31411E-05
FLJ35429	0.175058685	6.632278664	7.34579E-05
SEMA4A	-0.118487632	6.339562124	7.38888E-05
REV1	0.417804563	10.02490911	7.3972E-05
NDUFV1	-0.20985595	11.50188883	7.40102E-05
STAM	0.286456908	8.948462209	7.40821E-05
TOP3A	-0.128237437	6.905600229	7.41118E-05
C21ORF91	0.097972715	6.560899345	7.4151E-05
TRIP13	-0.146588952	6.121753102	7.44687E-05
LOC26010	0.367522692	9.49205428	7.46715E-05
NQO1	-0.198775252	7.25403607	7.47519E-05
TTLL4	-0.109394213	6.270770551	7.52164E-05
LYRM5	0.267863845	10.33644644	7.55119E-05
SCAND1	-0.16900059	8.446208285	7.55758E-05
TCTN1	0.1635035	7.934247716	7.57094E-05
LOC730083	0.110731811	6.152769531	7.58742E-05
SHANK3	-0.237825254	9.583582732	7.6675E-05
ADCY1	-0.166887863	6.588626681	7.68596E-05
ATP5G1	-0.212534484	11.52748066	7.69342E-05
COL9A2	0.119973004	6.209127224	7.69773E-05
WDR92	0.136402069	7.322477098	7.7143E-05
JARID1B	0.123470128	7.968381002	7.75769E-05
EXOC4	0.12124713	6.931603218	7.76981E-05
DNAL1	0.12943231	6.951793991	7.78334E-05
SNHG3-RCC1	-0.178282244	6.130484937	7.78839E-05
SMCHD1	0.112903782	6.46771514	7.80151E-05

LOC401284	0.11317715	6.122220879	7.80666E-05
LDLRAP1	0.139637939	6.696223661	7.83446E-05
SSBP2	0.28457945	9.42266917	7.84588E-05
AKR1C3	-0.329381497	9.514150262	7.88651E-05
ZBTB16	-0.471235147	8.472837445	7.88905E-05
PPARGC1A	0.283184875	10.65904619	7.89148E-05
SLC16A12	-0.220065466	11.63858494	7.9119E-05
LOC645018	-0.301440885	7.573143509	7.91884E-05
CCDC77	0.147995373	7.431092859	7.9263E-05
MTX3	0.143773685	8.006524937	7.92746E-05
ASMTL	0.1608943	8.319721724	7.95589E-05
GALE	-0.098922064	6.319138501	8.00667E-05
VEZT	0.231077726	8.119858321	8.01044E-05
SCN3B	0.113911224	6.335738148	8.02173E-05
MLYCD	0.28217689	9.47761112	8.07733E-05
MMD	-0.266336118	7.957100846	8.07867E-05
UGDH	-0.331028435	8.058620256	8.09558E-05
NUFIP2	-0.167344575	7.895839744	8.10478E-05
EDARADD	0.114924493	6.355353217	8.11277E-05
ELP2	0.150121341	8.748639124	8.11568E-05
C22ORF30	0.103126886	6.233989417	8.12644E-05
PPAPDC3	0.386715079	10.58038593	8.14745E-05
HADH2	-0.156583246	7.896718148	8.15745E-05
DNALI1	0.149271019	6.882706239	8.17975E-05
ARIH1	0.143878496	7.96596758	8.184E-05
BTG3	-0.222131391	7.381310348	8.18527E-05
KIAA0802	0.133914188	6.305363661	8.23352E-05
STAMBP	0.143806184	8.646805686	8.24906E-05
RIOK3	0.152575674	9.015894748	8.26235E-05
AMY1A	-0.277431441	6.674340393	8.26708E-05
RPS15	-0.212052945	11.309441	8.28236E-05
PSD3	0.183513444	7.347656553	8.33566E-05
TMEM63A	-0.14694351	6.679477692	8.33856E-05
EBF3	0.218044107	8.222299099	8.38222E-05
TFCP2	0.146726652	8.210728496	8.40796E-05
LOC440928	0.095831797	6.238091542	8.41692E-05
DGCR6	0.253340426	10.60155964	8.41881E-05
GMEB1	-0.10371197	6.718675746	8.44115E-05
MRPL55	0.141683292	9.040857843	8.46696E-05
NDST2	-0.13293245	7.688038713	8.47532E-05
EPC1	0.148281324	6.570155231	8.47909E-05
MOCS2	0.208751393	7.275085919	8.48382E-05
MRPS35	0.262092476	9.026765415	8.49718E-05
LOC653479	0.185738957	7.397638225	8.49887E-05
NMNAT1	0.152478826	7.036812666	8.5272E-05
STAT6	-0.17333576	7.56182863	8.5557E-05
SLC2A14	-0.116644335	6.139564581	8.56901E-05
SPRYD4	0.175161016	7.891065884	8.56955E-05
PITX3	-0.157649881	6.152982456	8.58361E-05
PIK4CA	-0.160701328	8.301050599	8.61855E-05
VCAN	-0.45328719	8.85731958	8.64003E-05

ZNF416	0.126604813	6.891990091	8.70115E-05
JUN	-0.287714693	10.29899996	8.72154E-05
SRRD	-0.176864654	7.044581413	8.72752E-05
HIVEP1	0.1268596	7.011109114	8.73937E-05
AUH	0.301080157	9.091024912	8.7594E-05
AZI2	0.171149358	7.719345296	8.7731E-05
ALKBH3	-0.197357504	7.763493061	8.84111E-05
RAB43	0.0782273	6.555981695	8.87672E-05
SENP6	0.11998442	7.39718911	8.89642E-05
LMBR1	0.174809758	8.118660715	8.89746E-05
KIAA0776	0.13711392	6.563763726	8.94063E-05
LY6E	-0.451155055	8.163755176	8.9642E-05
PARK7	0.161796062	11.85915049	8.97631E-05
TMEM37	-0.108500505	6.269104053	8.97827E-05
LOC727935	0.113597794	6.535070446	8.99972E-05
PCYOX1	0.294658559	9.397471304	9.04429E-05
LOC644390	0.186394891	6.400403577	9.05578E-05
DCK	0.096086079	6.770144668	9.13542E-05
AKAP6	0.197128455	7.810797916	9.13885E-05
PFTK1	-0.157746425	6.712713172	9.14326E-05
LRAP	-0.274841551	12.08103084	9.14463E-05
C1ORF218	0.254170503	8.387910836	9.15739E-05
NUCB1	-0.170547786	12.61936198	9.16891E-05
LOC644863	0.199850071	12.40661942	9.22259E-05
MYO1F	-0.124430917	6.200820511	9.24492E-05
NT5DC2	-0.218902125	8.177761927	9.32011E-05
ADNP	0.130370475	7.395724588	9.36418E-05
PIK3CD	-0.096500717	6.64653323	9.37606E-05
PKN2	0.165413106	6.909041869	9.43143E-05
OGT	0.192898357	9.136750988	9.44019E-05
C8ORF4	-0.335099314	7.984358225	9.44141E-05
PCNT	-0.271165244	9.215047269	9.70032E-05
ATP5SL	0.17613879	10.58008468	9.70386E-05
FOXC2	0.151901016	6.976946144	9.72168E-05
HCG9	0.190989264	6.247084943	9.72778E-05
C7ORF63	0.133314026	6.864026834	9.75535E-05
TMEM216	-0.159371187	7.624929118	9.7857E-05
MCTP1	-0.093438241	6.110764312	9.97034E-05
MGC20983	0.129571189	6.529391563	0.000100418
ECM1	-0.192177885	7.210810974	0.000100749
DST	0.140682532	6.536466911	0.00010083
AATF	-0.231656791	8.342084564	0.000101105
SOCS4	0.10991448	7.192265182	0.000102033
GOLGA8A	-0.197341424	6.54236235	0.000102033
CYB5R1	0.259089982	11.00883337	0.000102426
SETD2	0.137444475	8.808897221	0.000102588
ADH1A	-0.617993322	8.785709049	0.000102663
NOL5A	-0.260155645	8.760345571	0.00010312
RBM28	0.174044046	7.759890301	0.000103163
AMMECR1	-0.083618682	6.224400316	0.000103353
LRP3	0.199024783	9.118613903	0.000103455

RASAL2	0.12662423	6.595214298	0.000103472
ZNF658B	0.081766128	6.408647726	0.000103537
KCTD14	-0.092342765	6.092479138	0.000103664
SPAG9	-0.150614092	7.87835907	0.000103773
ANKRD37	-0.320317953	8.095848019	0.000103813
COL14A1	0.189170345	6.833444371	0.000103825
LOC202051	0.220822958	7.316520025	0.000104019
MGC72104	0.257944754	8.303338248	0.000104203
TRIM22	-0.294376734	7.437965529	0.000104977
PVRL3	-0.142448481	7.034381339	0.000104994
C20ORF39	0.341277669	8.624524829	0.000105031
RPL8	-0.159308447	12.33055474	0.000105322
ZMIZ1	0.207110955	10.62759817	0.000105689
STX5	-0.14892465	8.365719519	0.000105818
AS3MT	0.278178665	7.498369832	0.000106173
RYBP	-0.267902541	8.156652535	0.00010653
NFU1	0.232917977	10.15247831	0.0001068
RNF41	-0.210742618	7.145221972	0.000107377
ZNF136	0.0726305	6.308426468	0.000108023
C21ORF124	0.108965208	6.411670858	0.000108086
ADAMTS1	-0.39629335	8.664461804	0.000108213
UTP14C	0.174481652	8.61084275	0.000109436
C2ORF49	0.176780855	8.030248029	0.000109706
CSNK1G3	0.092382705	6.836320733	0.000109901
CXORF38	-0.151712843	7.234105468	0.000110097
LOC651575	0.14735681	7.234093348	0.000110411
GTF3C1	0.277453019	8.018326084	0.000110435
PACS2	-0.142986519	7.431840035	0.000110505
HSPA1B	-0.373148145	10.80002308	0.000110649
HOXC11	0.18944072	6.685198135	0.000111047
DMPK	-0.253544049	7.972317398	0.000111342
MOCOS	-0.121116928	6.274307368	0.000111503
NDUFB5	0.286925451	12.69246442	0.000111515
ZNF691	0.114163291	6.550042779	0.000111619
MED20	-0.210040835	9.799405047	0.000111649
MYOZ1	0.320841748	6.707191938	0.000111726
ZNF14	-0.327793544	10.23590523	0.000112028
HMGA1	-0.160600017	6.753728484	0.000112325
LOC644284	0.134048016	6.491873469	0.000112326
E4F1	-0.18839426	9.115816802	0.000112776
MAP3K1	0.239896892	7.991295646	0.000112813
AK2	-0.161920364	10.09663461	0.00011286
IMPDH2	-0.284474839	9.352256708	0.00011327
MARVELD2	-0.070904618	5.99648999	0.000113564
PDHX	0.246691161	10.38608158	0.000113604
VPS11	0.138436247	6.977391291	0.000113714
FAM23B	-0.130149783	6.262129744	0.000114075
C14ORF102	0.13008787	7.721204824	0.000114078
TGDS	0.120855219	7.387974532	0.00011431
FLNC	-0.342914009	13.31933655	0.000114787
LOC642732	0.14122622	6.501238786	0.000115141

IFNGR2	-0.179749242	10.60785719	0.000115145
CHRM2	0.111096868	6.399469025	0.000115373
CBFB	-0.214687055	7.332194673	0.000115981
ZNF438	0.155905222	7.506578843	0.000116111
HIST1H1A	0.130765728	6.658084652	0.000116282
MS4A6E	-0.105331041	5.98571932	0.000116637
CYHR1	0.179039051	6.929925909	0.000116861
GTF3C2	-0.100258042	7.945991412	0.000117109
NOLC1	-0.173934328	6.895468	0.000118417
LOC653066	-0.108955152	6.406846867	0.000119081
BEX4	0.269857832	9.243662623	0.000119221
DCP1A	-0.156235971	7.346770742	0.000119441
UBL4A	-0.199397418	8.783355253	0.000119497
C10ORF6	0.205987018	9.014874547	0.000119739
MOG	-0.144458995	6.261241107	0.000119844
GRAMD3	-0.185383848	8.013807509	0.000119866
MICAL2	0.206675005	7.473714779	0.0001201
GIMAP8	-0.295224708	8.817728183	0.000120309
C9ORF127	0.150058049	7.306310811	0.000120709
THRAP5	-0.118672574	6.372557488	0.000120964
GTDC1	0.095595517	6.77335176	0.000121142
PPP1CC	-0.223195795	11.42278035	0.000121175
LAP3	-0.268385037	9.147010177	0.000121448
C3ORF58	0.146407582	7.05548744	0.000122069
PRKRIP1	0.130139587	7.739184637	0.000122187
NRIP3	0.182351207	6.733913826	0.000122321
PECAM1	-0.267733102	8.875626209	0.000122462
ZNF543	0.112710526	6.590671439	0.000122489
PPA1	-0.241920221	11.43012141	0.000122714
LOC729148	-0.22827783	8.88960901	0.00012286
ZC3H3	-0.169824505	8.36694687	0.000122983
ADORA2B	0.132421045	6.584997322	0.000123202
FIGNL1	0.083178657	6.865957951	0.000123328
SHKBP1	-0.132762483	6.69082478	0.000123971
CXCL10	-0.414303577	6.675227316	0.000124203
FLJ22222	0.126395521	7.272855107	0.00012434
LOC645100	0.170497021	7.083206569	0.000124863
EFHA2	0.136046513	6.384782138	0.000124895
HLA-DRB3	-0.355664966	7.665932261	0.00012518
GSDMB	0.148590222	6.923338065	0.000125215
C4ORF30	0.135021942	7.903727108	0.000125242
CPLX3	-0.109661945	6.069127929	0.000125278
OXSM	0.155237032	7.130651739	0.000125363
LOC440704	-0.266866621	9.701061097	0.00012551
SFRS4	-0.179798308	10.35068055	0.000125633
ZNF2	0.091198745	6.441948302	0.000126017
RPL3	-0.226151423	12.56999692	0.000126226
FZD7	0.188572072	7.991075178	0.000126971
NDUFB7	0.183234051	12.40791408	0.000126973
DNER	-0.162150902	6.120690421	0.000127147
LOC653394	0.09686179	6.469710868	0.00012794

DKFZP686I15217	0.090464055	6.533269629	0.000128103
SERPING1	-0.281031329	7.868006148	0.000128358
LOC651149	-0.375240483	8.561579866	0.000128409
RAB13	-0.245549494	6.762786756	0.000128514
STRBP	-0.184050975	7.052335662	0.00012898
SHC1	-0.232289684	8.476874271	0.000129194
LOC653342	-0.126837144	6.0824073	0.000129571
COPZ1	-0.165072036	8.713876551	0.000130394
GTF2E1	0.109708636	7.180582605	0.00013047
GSTK1	0.196231728	11.2718068	0.000130761
ALDOA	-0.207594509	11.97465954	0.000130968
LOC652826	-0.197926958	7.494510465	0.000132009
CAPN5	-0.170013005	6.990146458	0.000132474
LOC653354	0.136414527	6.979613536	0.000132569
BIN3	-0.14272844	6.546675876	0.000133309
PUM1	0.136581879	10.3240619	0.00013385
ARPC5	-0.293763795	9.886903482	0.000134359
TOR1B	-0.151800382	7.23803638	0.000134486
PPM1A	0.173907707	6.981698465	0.000134715
LOC196993	0.112507043	6.348114951	0.000134906
VPS35	0.251718945	10.47410391	0.000135162
RGR	-0.084678769	6.361680307	0.000135552
NAGA	-0.121604764	6.323566223	0.000136816
MAGT1	-0.295764234	9.918128826	0.000137357
ZNF688	0.118327566	6.881993362	0.000137572
PMEPA1	-0.273150149	7.893996178	0.000137639
PLCZ1	0.124415885	6.218455723	0.000137809
PCDH9	0.178329296	6.756591092	0.000138395
C14ORF43	0.133990509	7.179833023	0.00013856
OSBP2	0.246343872	7.06891718	0.000138695
EPM2A	0.173427928	7.032147055	0.000139147
AURKAIP1	-0.16979509	12.46675745	0.000139349
CACNA2D2	0.094106021	6.474621394	0.000140078
ZNF687	0.102667002	6.20237173	0.000140219
LOC653158	-0.17780886	6.843195048	0.000140278
CCNG1	-0.269187017	8.371246136	0.000140838
YTHDF3	0.149245574	8.104454266	0.000140843
C12ORF34	-0.142568597	6.382198073	0.000141541
FAM83D	-0.170189409	6.11701979	0.000141999
PMS2CL	0.049680531	6.22872533	0.000142195
NUDT2	0.271047386	8.336962948	0.000142629
CDK5RAP2	0.201656936	8.464530307	0.000143366
RILPL2	-0.151449485	7.009512133	0.000143945
LOC124512	0.169339854	9.344397757	0.000144305
SSFA2	0.182878382	7.244191089	0.00014438
FADS3	-0.171444479	6.643033327	0.00014506
RHBDD1	-0.109662164	7.656152834	0.000145234
LSM4	0.2258153	9.938953153	0.000146032
CUL4B	0.117031469	6.867645144	0.00014605
CBS	-0.346952141	7.577785515	0.00014635
SUSD2	-0.331283703	7.557538945	0.000146371

LEO1	0.128363141	6.84717223	0.000146476
RFXANK	-0.122458309	7.39649858	0.000146525
POLR2I	0.1743728	10.7533389	0.000146713
ZNF418	0.141437703	6.914482028	0.000147126
FAM114A1	-0.125387435	6.443771934	0.000147135
MED25	-0.222273828	7.89903019	0.000147216
CLN8	-0.136041885	6.713139414	0.000147382
LOC401357	-0.161744545	7.006203152	0.000147391
GRB14	-0.205299109	7.080192284	0.000147804
HEATR3	-0.10253953	6.737918345	0.00014897
SUPT5H	0.22079292	9.503485658	0.000149042
PPIL4	0.106084801	6.27867893	0.000149161
SEL1L2	-0.491400526	6.953252308	0.000149841
HK1	-0.177939637	10.42481935	0.00015014
C12ORF52	0.138385174	8.019208535	0.000150663
NELL1	0.133080505	6.566081194	0.000152083
FAM10A7	0.217335104	7.140285285	0.000152123
ATP6V1H	0.161326934	8.245006611	0.00015217
PFDN6	-0.250868577	7.284707428	0.000152886
NEK7	0.298743998	6.991287939	0.000153205
TBCCD1	-0.090663199	6.524408595	0.000153278
ATP6V1E1	0.158072227	11.67936973	0.000153524
COPS8	0.14756496	9.019018424	0.000154197
IGF2R	-0.194607188	10.68105722	0.000154264
COL4A6	0.136929616	6.57113086	0.000154658
SSH1	-0.153141188	6.990919903	0.000155182
CUL4A	0.183376834	9.886550176	0.000155614
NRP1	0.176777732	9.041473114	0.000156523
DNM1L	0.152482588	8.703102639	0.000156793
CCDC132	0.129431574	6.600713067	0.000157385
PEG3	0.174811552	7.558197935	0.000157433
KRR1	0.137372704	6.789639736	0.000158231
PPP2R2B	0.164808403	7.455160443	0.000158402
PARP16	0.135300416	6.576283983	0.000159111
SELT	0.139048804	8.210235276	0.000159155
NKIRAS1	0.174630461	9.213437558	0.000159507
TSEN2	-0.156538504	7.720610173	0.000159731
GRN	-0.181104168	9.150381207	0.000159739
RAB40C	-0.208007698	9.520648978	0.000160612
GPC1	0.241699824	8.671842039	0.00016093
PHF12	0.071272496	6.506722246	0.000161058
THRSP	-0.312762553	6.119876815	0.000161277
NETO2	-0.247215545	6.914553747	0.000161342
CPNE1	-0.133825122	7.223895138	0.000161579
HSPB6	0.44043809	11.83405565	0.000161815
PPP1R14C	0.263427757	10.23067157	0.000161845
C20ORF196	0.12896711	6.77398132	0.000162061
CENTG3	0.159426607	7.117960831	0.000162286
SLC41A1	0.27347771	10.45567821	0.000163177
STGC3	0.141173829	6.418320748	0.000163382
CCDC90B	0.153799982	9.342384308	0.000163545

HSGT1	0.123020194	6.64396559	0.000164095
STAU1	0.165843065	10.21739795	0.000164434
C21ORF119	0.177304029	7.33419214	0.000164895
FIP1L1	0.158599675	7.32685246	0.000164953
C9ORF169	0.118287134	6.173743871	0.000164968
ALS2CR16	0.110878135	6.256009416	0.000165327
CD274	-0.126746594	6.18454218	0.000165333
STAT5A	-0.194136465	7.641104274	0.0001655
SH3BP2	-0.109765795	6.22465608	0.000165591
KCTD20	-0.194311648	8.53680804	0.00016586
FOXRED2	0.124908541	6.677390833	0.000165875
RBP7	0.311028559	8.702943679	0.000165881
TM7SF3	0.187825489	7.3054448	0.000165901
CPAMD8	-0.140857716	6.302285508	0.000166244
SMARCD1	-0.120479778	7.758833462	0.000166505
MCFD2	-0.17281098	7.51675732	0.000166532
CLIP3	0.309710816	8.248855352	0.000166555
HHEX	0.186015328	6.82052362	0.000166629
CYB5A	0.269359712	9.703865353	0.000167267
TJP2	0.196291083	7.21024241	0.000167822
C1ORF198	0.283940077	8.501982858	0.000167867
APPL1	0.228407514	8.151970267	0.000168407
MED8	-0.116779413	7.256214533	0.000169313
DEXI	-0.226713498	10.91850641	0.000169387
MAPK6	-0.236023007	8.248979599	0.00016957
MKS1	0.119322216	6.523693519	0.000170429
CCDC7	0.058736225	6.118813348	0.000170556
NCAM1	0.144135411	7.199351988	0.000171378
LOC728417	0.090302502	6.48964982	0.000172579
IDI2	0.114735101	6.244684789	0.000173545
PSMD10	0.15356369	8.001227778	0.000173849
TRIM2	0.299713549	8.055042837	0.000174101
SLC44A2	0.171200958	10.0226822	0.000174982
MECR	0.102521707	6.533810307	0.000175361
GDF15	0.128902779	6.357894517	0.000175493
C20ORF30	0.136805921	10.5045406	0.0001757
KRAS	-0.110016544	7.043808293	0.000176681
YPEL5	0.210753934	9.820373823	0.000176837
KIAA0020	-0.208912342	8.505772701	0.000176956
DLK2	0.141901342	6.438228858	0.000177175
PLEKHH1	-0.117321996	6.443836489	0.000177712
C14ORF85	-0.268620698	11.47968661	0.000178301
AEBP2	0.11349287	7.448438251	0.000179117
ABCC3	-0.130058817	6.221548943	0.000179259
EIF4EBP1	-0.152237927	6.326368785	0.000179533
LBR	-0.074752976	6.323223336	0.000179701
DSG4	0.104845387	6.359698846	0.000179773
HDGF2	0.115217546	7.866492556	0.000179941
FN1	-0.100369688	6.137592625	0.000180612
AKR1D1	-0.19678631	12.11866731	0.000180821
ZNF673	0.122058866	6.40420992	0.000181153

CSF2RA	-0.21715802	8.960437489	0.000181464
NPY6R	0.232767588	9.459037576	0.000181619
LOC90826	0.138746299	7.189597048	0.000183045
CCBL2	-0.091735243	6.720201154	0.000183061
SCARB1	0.159009217	7.011659259	0.000184085
LOC648581	-0.131091347	6.907540224	0.000184487
BECN1	0.14378406	8.528256122	0.000184962
LOC644322	-0.391324181	8.651775795	0.000185055
ARHGAP28	0.067811952	6.109392795	0.000185769
MSTN	0.115511	6.201081302	0.000185934
MYL9	0.249016125	10.2354434	0.000186967
C1ORF186	-0.113252076	6.092516718	0.000187011
CPSF1	-0.165935209	8.154542056	0.000187027
LOC652541	0.127381911	6.799746987	0.000187214
C5ORF29	-0.15293865	6.325838293	0.000187616
KCNK1	-0.443341157	7.66320109	0.000187627
PQLC1	-0.136463408	10.21947392	0.000187978
ATAD1	0.179176927	9.135469743	0.000188016
TOMM20	0.213077832	9.736854883	0.000188588
TIGD2	0.101001516	6.656948877	0.000191819
GCS1	-0.148488093	6.790134749	0.000192104
MGC70857	-0.155096072	7.798947412	0.000193628
AQP7	0.274168949	7.724859293	0.000193763
HLA-DMB	-0.324347317	8.895074213	0.000193903
RICTOR	0.155199328	6.503311184	0.000194139
L2HGDH	0.189035835	7.74962376	0.000194447
GLO1	0.200091566	11.10744581	0.000195978
PPOX	0.143495988	7.769638702	0.000196968
CENPN	-0.263152592	7.102226606	0.000197304
SUCLA2	0.277451463	10.10153693	0.000197431
FBXO40	0.182575067	6.292168216	0.000198338
NDUFS1	0.249807098	7.815901257	0.000198483
C13ORF34	-0.126540228	6.175584518	0.000199579
CCL8	-0.392844419	6.963754791	0.000200014
MAP1A	0.419393807	8.553758813	0.000200707
CCT7	-0.137038895	10.52917787	0.000201516
PNKD	0.124004285	7.44778399	0.000201927
BTBD1	0.286057734	8.665908355	0.00020201
DNAJC3	-0.149213124	6.292263331	0.000203426
C6ORF35	0.092266218	6.268883958	0.000203445
LAS1L	-0.155062271	6.810027627	0.000203696
EEF1E1	-0.212385736	7.720975118	0.00020389
CDO1	0.235810554	7.382970659	0.000204208
ZNF341	-0.126868851	6.681570198	0.000204615
SLC36A2	-0.124926759	6.069916635	0.000206022
RHOJ	-0.165580519	7.571994351	0.000206226
NLRC4	-0.107415926	6.177295022	0.000207255
PRPF31	0.19312114	9.178884915	0.000207673
AASDH	0.145624012	7.311213016	0.000207792
PCDHGA12	-0.159154363	6.442593513	0.000208294
USP31	-0.134615048	6.05233901	0.000208756

WDR40A	-0.137910693	8.101554723	0.000209572
ZNF546	0.107810159	5.998990178	0.00020968
HLA-DQB1	-0.284652643	6.713819686	0.000210266
LCN10	-0.11920945	6.0255868	0.000210899
CHST3	-0.234082004	8.13579908	0.000211245
SLK	0.207788722	8.822857137	0.000211363
NUP133	0.18127603	8.469713905	0.000211744
WDR8	0.126589883	8.134812105	0.000212234
MPZL2	0.185935577	7.34242992	0.000212285
ZNF140	0.176801897	6.699499279	0.000212549
GYG2	-0.069997517	6.02595808	0.000214572
CTBS	0.126906935	7.294391048	0.000214768
SH3KBP1	-0.402685724	9.553223335	0.000214789
HSPA14	-0.129378719	7.989031572	0.000215096
ERCC2	0.148572903	6.893627012	0.000215129
TP53I11	-0.109156436	6.414639868	0.000215866
CTDSPL	0.202221386	9.890123237	0.000215965
TLR6	-0.075142129	6.20524766	0.000216588
TBL1XR1	0.226338087	7.35539186	0.00021662
MRPL54	0.211615394	9.924183361	0.000217525
LOC390354	-0.283926835	12.01165825	0.000217578
EIF1B	0.348769782	11.57295348	0.000217855
LOC134997	-0.267971329	10.81287182	0.00021825
IQGAP2	0.191059631	6.961259705	0.000219548
C5ORF35	0.271209025	8.069133657	0.000220122
DIRAS1	-0.327071954	7.232339495	0.000220535
CREB1	-0.231402601	11.39676671	0.000220967
HLA-H	-0.288349587	9.853336556	0.000221581
PCBP2	0.136360385	11.71845742	0.000221625
LOC651316	0.153782173	6.527782663	0.000222003
SLC4A1AP	0.154337768	7.277775277	0.000222294
DHRS3	0.210478224	9.291983192	0.00022343
GCC2	0.068705587	6.167683197	0.000224089
NOTCH4	-0.223434463	7.769449166	0.000224131
C12ORF65	0.207570897	7.445712172	0.00022471
FGFRL1	-0.168141655	7.695050098	0.000224765
NR3C2	0.200608111	8.036776764	0.000227507
ZNF160	0.115254242	7.430670308	0.000228665
C12ORF4	0.151482917	7.453520055	0.000228817
ISOC1	0.30188083	9.880737249	0.000228837
LOC727848	-0.088813214	6.25370018	0.000229201
LOC647389	-0.24372079	8.316837776	0.000229876
GOLGA1	0.14862051	7.485095093	0.000230009
HSCB	-0.128052565	8.023342108	0.000230459
P2RY1	0.094714797	6.251609503	0.000230661
DPY30	0.15434244	8.879986927	0.000230983
SYDE2	0.104721292	6.575569273	0.000231455
ZBTB33	0.145635564	8.698238534	0.000231478
PHB	-0.115915742	9.691590515	0.000231796
ESR1	0.127395437	6.299203757	0.000232061
YY1AP1	-0.105815558	8.220928621	0.000232144

C3ORF37	-0.127029735	8.064219096	0.000232171
C10ORF59	0.160221179	7.414828094	0.000232656
MAPRE1	-0.179867115	10.39269797	0.00023277
POLDIP2	0.125108479	8.260828822	0.000232868
TMEM44	-0.145234059	6.720626477	0.000232995
ISCA1	0.227582544	8.736834496	0.000233165
FLJ42875	0.10969231	6.484570738	0.000234596
GMCL1	0.131356795	6.807214964	0.000235747
GAB2	0.141136339	7.225024636	0.00023707
PPP2CA	0.151993527	11.49227561	0.000238261
ICA1	-0.16637616	8.929891896	0.000238662
TUSC1	-0.149850418	7.165282034	0.000239106
JPH3	0.104047848	6.4816094	0.000239513
C15ORF52	0.275758389	11.21188434	0.000240469
ROCK1	0.164345072	6.528723568	0.000240484
PSCDBP	-0.116715791	6.110117947	0.000241385
MGC13057	-0.160893332	7.358650564	0.000241765
TUBA4A	-0.397931274	10.51807443	0.00024227
HSPG2	0.143121051	6.667651526	0.00024246
VEGFA	0.22110042	7.835257111	0.000242623
COL6A1	-0.245790086	9.42535504	0.000243549
ACO1	0.144536374	11.08996311	0.000243625
BCL7B	-0.125347787	8.509380049	0.000243634
ATG4C	0.108478263	6.708713864	0.000243809
KIF15	0.102032226	6.231447993	0.00024401
LOC653157	0.173542513	6.414342543	0.000244024
OSGIN1	-0.101306617	6.245286968	0.000244244
MAFK	0.118394022	6.232724898	0.000246457
FZD8	-0.14016533	6.570936953	0.000246479
PLOD2	-0.246631856	7.964232251	0.000246801
D4S234E	-0.387536964	7.344409561	0.000246863
STIP1	-0.263594981	9.330633033	0.000247712
STX16	0.177166556	9.258644886	0.000247968
C19ORF61	-0.136670044	6.608912979	0.000248249
RAPGEFL1	-0.124881984	6.667211479	0.000248823
SPOCK2	-0.345176505	8.071955034	0.000249521
C18ORF34	0.188665473	6.312602554	0.000249542
UPF3A	0.1435812	7.844110827	0.000250748
WISP1	-0.099159674	6.130707895	0.00025176
C13ORF1	0.154590943	7.833540709	0.000252754
NEURL2	0.188124072	7.501848403	0.00025304
PIBF1	0.111196322	6.791270342	0.000253678
SEPN1	-0.217154583	8.018903902	0.000253801
GALNTL2	-0.254178392	6.470288389	0.000254821
EMCN	-0.238287577	7.682641385	0.000255149
PML	-0.044297341	6.211809298	0.000255734
LOC651398	0.086245414	6.146234372	0.000256149
RPS3	-0.22201017	12.18019545	0.000256389
VPS39	0.201414558	7.405090662	0.00025681
KIAA1219	-0.111678131	7.124355381	0.000257203
USP49	-0.360113541	10.20446722	0.000257538

ABHD4	0.119465598	6.73057528	0.000257549
DNASE2	0.202045504	9.387863882	0.000257942
LOC650683	0.096989214	6.271413021	0.000258112
PARP3	0.150030671	8.119751833	0.000259974
NDUFS2	0.263511368	8.305911356	0.00026006
C7ORF10	0.171856762	7.077656942	0.000260423
LOC728744	-0.108448104	6.098719964	0.000260448
INPP5D	-0.135642516	6.531409367	0.000262804
ZDHHC7	-0.121993354	9.614519906	0.00026353
BCO2	0.212196834	6.630349426	0.000263977
KCNK6	0.185821336	7.099623623	0.000264052
ZNF334	0.07375652	6.16935788	0.000265139
XIRP2	0.588422606	11.0412158	0.000265418
SYPL2	-0.220817045	7.309603688	0.000266062
ZMYND17	0.196513549	6.511344709	0.000266084
LOC643722	-0.085119831	6.041009972	0.000267148
LOC727762	0.157299042	6.881208918	0.000267439
NUP93	-0.161108583	8.222863845	0.000267564
C16ORF57	-0.181281492	8.017709745	0.000267588
LASS2	-0.196999159	7.859701277	0.000267716
HNRNPUL2	-0.191041294	8.491577489	0.000268658
MPST	-0.121908802	7.100634911	0.000269976
ANKDD1A	-0.130765865	7.376042908	0.000270294
SNRPN	0.188492336	10.97221158	0.000272589
IRF5	-0.092688863	6.135925293	0.000272889
TTC35	0.202030172	6.9057749	0.00027359
ZNF383	0.117376087	6.548394548	0.000273849
ZNF275	-0.107330692	7.2970995	0.000273966
LTF	-0.108587713	6.098604935	0.000274871
LOC730316	-0.141958427	10.11782448	0.000274995
ARMCX5	0.11851823	6.740878718	0.000276306
EHHADH	0.255194567	7.545747158	0.000276505
REV3L	0.135196827	6.722681634	0.000276565
DLG4	0.204591626	7.208008044	0.000276919
LOC646144	0.059303823	6.212110442	0.000277174
OBFC2B	0.186508151	7.522617216	0.000278
CCR4	0.09089438	5.877431035	0.000279613
ZNF606	0.114001472	6.69032838	0.000280091
KCNS1	0.118507261	6.199456398	0.000281692
ZFP112	0.18089395	6.75467867	0.000282268
PSTK	0.097081486	6.755062767	0.000283013
TMEM93	-0.128704851	10.03613492	0.000283407
ARSG	0.109543932	6.614678963	0.000283411
FLJ35220	0.104212491	6.431803121	0.000283824
SRPK2	-0.221875882	6.875696481	0.000283909
VSIG8	0.103937703	6.311744653	0.000284128
NUDT1	0.126207444	7.653106144	0.000284154
MED11	0.124581709	6.571925356	0.000284937
LOC645743	0.111014946	6.426634338	0.000287069
YEATS2	0.139191622	7.763232265	0.000287894
LRCH1	0.084499817	6.277437023	0.000289697

NCAPG2	-0.119577829	6.204987942	0.000291842
NANP	-0.102124747	6.200800745	0.000294018
TUBGCP5	0.227282704	7.465654253	0.000294689
HPS4	0.043099071	6.18314235	0.00029546
PCDH12	0.202672743	6.670547384	0.000295709
NXPH4	0.077485203	6.125531846	0.000296342
RBM38	-0.301112346	7.862806904	0.000296586
SAAL1	0.156005631	8.373192313	0.000296912
RAPGEF1	-0.145845338	8.540247796	0.000297189
ARHGAP10	0.226309843	8.994160188	0.000297431
LOC388474	-0.1354957	13.81335734	0.000297574
CD109	-0.124175842	6.241714672	0.000298
LYPD2	0.123105913	6.389893655	0.000298384
GMFB	-0.228581769	9.484280454	0.000298671
TTLL1	0.099926348	6.423959931	0.000299035
FAF2	-0.172026595	9.307334794	0.000299175
COX7A2	0.154678296	13.57767657	0.000299194
LOC646531	-0.221523129	12.35826927	0.000299539
TBCE	0.139793387	8.665748605	0.000301188
LOC652968	-0.123625253	6.99670101	0.00030185
SETDB2	0.278705328	8.081502032	0.000301854
GNPDA1	0.155364711	9.412394265	0.000302831
GSTM5	0.135638998	6.327700053	0.00030345
ZNF549	-0.263913273	11.21944619	0.000304405
KATNAL2	0.094262063	6.38066773	0.000304409
NUCKS1	0.265631594	7.522581114	0.000305571
ZNF7	0.135313288	7.45154275	0.000305645
ACOT4	0.149968137	6.620037848	0.000306706
YBX1	-0.1459143	11.49630028	0.000306707
KIAA1328	0.126906031	6.632975534	0.000308597
LOC550112	0.106269595	6.439491711	0.00030884
HABP4	0.160418467	8.399593304	0.000310223
EPHA2	-0.147253351	6.405934944	0.000310529
ILF2	-0.214538689	10.00907546	0.000310571
TRO	0.163116224	7.005807374	0.000310949
NMI	-0.198745943	7.260577873	0.000311389
ARF1	-0.1146303	9.88948019	0.000311735
NPAT	0.161557225	6.53022696	0.000312183
NR2C2	-0.162041655	6.820915819	0.00031235
LOC341457	-0.248773163	12.69186859	0.000312642
FLJ22374	0.143186336	6.932403635	0.000312668
HK2	-0.346414406	7.355359987	0.000313285
CPXM2	0.4196779	7.872768802	0.000313523
BCL11A	0.165693863	7.421518038	0.000314696
FAM80A	0.149182389	7.006693192	0.000314911
TMEM106C	0.293503377	8.869914843	0.000315007
POLR2J3	0.163949006	8.852764626	0.000315075
C7	-0.491004879	8.969459028	0.000315347
BAI3	-0.152660543	6.493961634	0.000315512
ZNF577	-0.203156556	7.919215677	0.000316584
OCRL	-0.113563879	7.390294923	0.000316587

MFAP1	0.177603379	8.081161971	0.000316706
C6ORF199	0.124127427	6.472926829	0.000317117
PLEC1	-0.0907287	6.615640678	0.000318201
TMEM17	-0.292555215	10.53055674	0.000319033
PHF10	0.110699454	7.257336126	0.000319758
DDX50	0.138784181	8.488223607	0.000320924
TSR1	-0.133578937	7.304164747	0.000322071
SCMH1	-0.10615541	7.76362593	0.00032299
PFKM	0.338419606	11.67113455	0.00032305
LOC348262	-0.204541432	8.510324203	0.000323126
ARPC2	-0.13943264	10.12591709	0.000323389
FAM186B	0.087681592	6.144920668	0.00032431
AMOT	0.149376576	6.99167891	0.000324386
CHST10	0.142556881	7.217747242	0.000324812
TMEM104	-0.123861528	6.252764309	0.000324923
C14ORF19	0.165344084	6.369605358	0.000325001
APAF1	-0.107660189	6.966156198	0.00032622
LOC442501	0.065460855	6.106087498	0.00032687
BCOR	0.138574822	7.015136894	0.000327347
FLJ46309	-0.299205925	10.10757217	0.000327996
KIF21A	0.216412273	8.42011933	0.000328688
NCALD	-0.179784276	8.119272243	0.00032969
TNRC15	0.126870507	8.305334665	0.00033247
LOC644039	-0.164444256	13.40091638	0.000332753
FLJ37464	-0.144364113	6.435776363	0.000333057
GSPT2	0.166760111	8.774698134	0.00033588
NDFIP2	-0.208234573	9.65663542	0.000336389
NEDD8	0.104644651	12.12243483	0.000336891
CSDE1	0.160793785	8.076254372	0.000336934
LOC402560	0.203854869	8.392690931	0.00033703
PLXDC2	0.261247888	7.703806829	0.000337598
ALDH1L2	-0.101942838	6.208083067	0.000338161
SBF2	0.102587337	6.65784709	0.000338335
LOX	-0.153657946	6.341257351	0.00033956
LTA	0.088979712	6.227887029	0.000339888
C16ORF56	-0.196992573	7.624983041	0.000340939
TIGD1	0.083541579	6.227625222	0.000342568
IL2RA	-0.098154397	6.034218727	0.000342664
GLRX	0.426501757	9.793786259	0.000342911
HSPC268	-0.258980968	9.559771572	0.000343065
ORC4L	0.11882748	6.596420713	0.00034551
MTG1	-0.107096935	6.525595312	0.000345753
NDUFC1	0.155705719	10.4817167	0.000345942
GPR161	-0.080284924	6.129921195	0.000347925
FUCA2	-0.104508951	7.481917857	0.000350706
SERPINE2	0.549077184	11.54764238	0.000351183
PLDN	-0.176943992	8.972047514	0.000351233
PGBD5	0.165110823	6.894982509	0.000352365
C2ORF63	0.179614983	6.327642811	0.000352865
ARL6IP5	0.15447663	11.50380449	0.000353747
TNFSF13	-0.077312612	6.116411664	0.000354213

UBE3B	0.207532122	7.64631983	0.000354716
TMED3	-0.280630816	10.35502322	0.000356042
VPS37D	0.126566563	8.010484011	0.000356325
VGF	-0.1696214	6.172912153	0.000356592
LOC347544	-0.243724659	10.93635163	0.000357134
LYPD5	-0.083574663	6.132292849	0.000357192
DPP7	-0.175737209	8.067287398	0.000357959
PTPN18	-0.089289096	6.213383858	0.000358158
C6ORF85	0.136815927	7.249182964	0.000358603
TSPAN8	0.220779127	6.194834473	0.000359202
NUDT9P1	0.108517635	6.279598521	0.000360319
CYB561	-0.081609033	6.606899314	0.000361126
NAP1L2	0.087132254	6.424153747	0.000361167
EBF2	-0.085860846	6.252732827	0.000361549
RNMT	0.194624118	9.377983948	0.000361853
MOCS1	-0.118236466	8.384451652	0.000362073
C6ORF117	0.164723596	6.515212192	0.000362573
INSC	-0.080983861	6.154181	0.000366093
ZNF669	-0.163121031	7.011609141	0.000366493
NIT2	0.172666228	8.925311292	0.000367639
PLEKHN1	0.145506761	7.206073649	0.000370192
TLN1	-0.171390286	9.019186261	0.000370694
MCAM	0.142829398	6.504331048	0.000371786
ST6GAL1	0.135703282	8.274907142	0.000371848
ZNF668	-0.163455985	7.540667361	0.000371856
NAT14	-0.127419084	7.221702445	0.00037198
ALG5	-0.184773702	8.338516636	0.000372042
ZNF34	0.127314583	7.107114989	0.00037207
NIF3L1	0.123508258	9.593620225	0.000375499
C7ORF26	-0.146453953	8.055234448	0.000375856
SRP72	-0.168989934	7.749590562	0.000375976
SRP9	0.191514373	10.77899222	0.000376995
LOC399744	-0.104417127	6.321653478	0.000377472
SCCPDH	0.143257963	7.25146138	0.000378247
TMEM129	0.174245019	6.709174561	0.000378304
SPG3A	0.095551856	6.494098724	0.000379232
APIP	0.173633558	8.386339767	0.000379796
RAB17	-0.2632714	7.279587926	0.000379872
NEU1	-0.168323639	7.869454133	0.000380236
FAM174A	0.187622359	8.079890365	0.000380241
NECAP2	-0.16930518	7.9472175	0.000380326
DKFZP686O24166	0.113393068	6.478323386	0.000380476
AHSA2	0.340064373	7.338722104	0.000380623
PMPCA	0.149070053	9.561936864	0.00038154
RORA	0.088294161	6.567897342	0.000385208
DNAJC5	-0.13941766	6.991567527	0.00038608
BRD3	0.214277632	8.642407405	0.00038724
C8ORF58	-0.098131451	6.422827566	0.000387727
CLPP	-0.14353312	8.709272895	0.000389148
C11ORF67	-0.27412852	10.31467092	0.000389433
LOC729466	0.12169272	13.69256036	0.000390368

TMEM106B	0.255334946	7.756501486	0.000390551
ASPM	0.122762396	6.397742568	0.00039064
CCR6	-0.177104024	12.39515882	0.00039099
IDH3G	-0.144164511	8.661418797	0.000391574
TMEM161A	0.127277547	6.846915669	0.000392884
LOC643466	0.111269503	6.325953963	0.000393003
IL1B	-0.112138309	6.125413705	0.000393083
TRAPPC4	-0.173862011	9.382684661	0.000393098
TTC32	0.179340408	7.464690436	0.000395242
PAK6	0.192904881	6.670532129	0.000395464
GNAO1	-0.107165585	6.275824597	0.000395848
ARL2BP	-0.165382202	10.19036273	0.000396312
ZNF662	0.112150966	6.45744652	0.000396344
TM6SF1	-0.141165673	6.887293343	0.000396739
UBC	0.158317881	13.76376127	0.000396795
WDR24	0.12138276	6.810174966	0.0003969
LOC123688	-0.113038155	6.661731025	0.000397289
TCF4	0.221583092	8.836167364	0.000397369
FUBP3	0.155866495	8.400208619	0.000398627
ABAT	0.13869711	6.317196295	0.000398872
B3GALNT1	0.213494523	8.847705726	0.000398924
C12ORF30	0.109713392	7.176045925	0.000401025
C5ORF39	0.136351585	7.140220296	0.000401269
SSR3	-0.189626849	7.411659909	0.000401545
HEATR6	0.15079335	7.115928865	0.000402428
LOC642755	-0.170999094	11.03607997	0.000403524
GLUD1	0.133640658	10.04864226	0.000404649
C6ORF151	-0.160837138	6.640202293	0.00040486
C10ORF84	0.10075197	6.692381218	0.000404882
FCER2	-0.092351	6.06926987	0.000405355
TMEM178	0.18209249	6.834566821	0.000405696
REP15	-0.145739909	6.482245923	0.00040578
AKTIP	-0.190625902	9.761287835	0.000405866
BNIPL	0.052390398	6.245996103	0.000406076
NIPSNAP3A	0.147777296	6.980113266	0.000406091
DAP3	0.153724547	9.993595943	0.000406546
SNORD13	0.345578058	7.845441294	0.000406659
FAM158A	0.128533394	7.764044033	0.000407858
NAGK	-0.125376711	9.252475962	0.000408465
TMEM192	0.125868098	7.004355722	0.000411699
STK4	-0.169296187	8.685468991	0.000411762
CANT1	-0.147756923	8.051230479	0.000412692
MTMR14	0.168163765	8.562366879	0.000412805
SLC25A36	0.231276199	8.248571184	0.000414244
CPNE5	-0.251124116	6.236508148	0.000414869
SNRPA	-0.134425607	7.783034913	0.000416854
LOC400879	0.081643086	6.169340785	0.000417827
TMEM54	0.180160372	7.256978831	0.000418218
PGRMC1	-0.16333999	9.366014184	0.0004186
MYL6	-0.247589279	12.58900797	0.000419688
TRUB2	0.137584782	7.30973119	0.000419726

FAM107B	0.23828088	7.2613172	0.000421341
ULK1	0.208807945	9.677417613	0.000421861
SH3BP4	-0.161465001	7.90095033	0.000422036
SIL1	-0.138260976	8.445557346	0.000422627
RNF34	0.14416185	8.445035528	0.000422973
IL10RA	-0.113942064	6.441424382	0.000423174
FBXL10	0.111536233	7.644519474	0.000423332
COG7	0.116066671	6.802017014	0.000424159
LAMP1	-0.138568214	11.14201686	0.000424724
C1ORF123	0.185793013	9.917560933	0.000426681
NCF1	-0.113736833	6.161063812	0.000427729
MAST4	-0.20495858	9.66328587	0.000429325
P2RX4	-0.148385802	7.038596158	0.000429416
LOC728772	-0.067142467	6.352334404	0.000431219
LOC653610	-0.161980266	6.385924087	0.000432115
NLRP7	0.104743175	6.371429281	0.00043298
ASF1A	0.20426963	7.999585383	0.000433114
MATR3	0.11622186	8.6885702	0.000433673
LOC653506	-0.214700212	10.87368036	0.000438088
FAM13C1	0.076917024	6.182069371	0.000438094
ATXN7L3	-0.164865648	7.785939759	0.000439177
EDN2	-0.096125717	6.040198948	0.000440612
MMP2	-0.101660219	6.690021112	0.000446851
VPS36	0.198342778	9.023580594	0.000446949
UBE2A	0.180870955	9.142721991	0.000448276
SIGLEC16	-0.135203616	6.20970475	0.000450659
CDK10	-0.106446564	7.571557219	0.000450732
TC2N	-0.12627081	6.194902748	0.000451819
NUDT3	0.163305314	9.495652485	0.000452906
ZSCAN12	0.101880756	6.309426226	0.00045317
ZNF679	0.100710913	6.120488462	0.000453945
TMEM218	0.134177995	7.61458516	0.000457074
LOC642852	0.131260554	6.288699463	0.000460843
SLC7A6	0.178350872	8.071942415	0.00046199
AASDHPPT	0.199206014	8.37884803	0.000462689
MSH2	0.096838801	6.334859882	0.00046375
C19ORF4	0.191077017	6.592656728	0.000465078
CRYZ	0.285271506	10.00184204	0.000466199
HSF2	-0.190759744	8.048810988	0.000467043
KIF13B	0.161516262	7.686963338	0.000467248
AKAP12	-0.177638267	6.658204548	0.000467381
LOC654201	0.095771377	6.079270914	0.000470209
NAP1L5	0.2270611	7.757366358	0.000472347
FAM78B	-0.121348017	6.395944557	0.000474434
DIS3	0.100905385	6.465178401	0.000474902
PRMT5	-0.151244356	8.382912289	0.000476272
LOC441034	-0.125035011	13.51130371	0.00047703
SLFNL1	-0.104995228	6.084483472	0.000478964
LOC644162	0.257901172	7.899513735	0.000480527
TATDN2	-0.161648859	7.450169305	0.000480954
CYP2S1	-0.06706461	6.183477715	0.000481163

ANKRD57	-0.163535616	8.028753994	0.000481213
STT3A	-0.195325432	9.008082675	0.000482848
LPPR4	0.096477777	6.13711725	0.000482992
MGC61598	0.260061379	7.727701944	0.000483181
ASH2L	0.137168424	9.276479235	0.000484097
RALA	-0.163055307	9.916999442	0.000485913
FLJ44076	0.08763846	6.11743639	0.000486555
C9ORF72	0.092585623	6.761581899	0.000487801
WDR21B	0.114758878	6.697558687	0.000489592
HRASLS3	-0.184771367	11.27957092	0.000490494
HP	-0.098027921	6.115513232	0.0004922
LOC642412	0.150101997	6.970275152	0.000492529
PTPRA	0.070456611	7.070894858	0.000496206
UBE4B	0.202319199	8.65376659	0.000497167
FAM108A3	-0.128506754	7.373765373	0.000498254
BCAR3	-0.297842678	8.079740773	0.000499387
C11ORF68	0.146434373	7.44178663	0.00049954
ADI1	0.405861349	8.061842272	0.000500342
CCDC128	0.253978835	7.472160026	0.000500663
COL10A1	0.176321065	6.140842109	0.00050182
CXCL12	-0.227097902	8.77196414	0.000502183
VIPR2	0.25122233	7.535853226	0.000502579
FLJ20254	-0.219549553	6.917905901	0.000503819
SLC7A2	-0.21786119	7.360295418	0.000506809
LSM3	0.190810748	10.3783068	0.00050704
AHNAK	0.15562732	10.21434743	0.000511324
ANAPC4	0.131608158	8.63670284	0.000511606
CCDC101	0.111863526	7.253105179	0.000512003
RAB30	0.106120254	6.368265648	0.000513219
ALS2CL	-0.067887985	6.183339035	0.000513431
ASB16	0.100026965	6.212355327	0.00051401
CLIC3	-0.241626918	6.749185341	0.00051404
ADCY4	-0.219724	8.62922394	0.000514286
SPIN1	0.137770045	7.26899352	0.000515554
LOC729603	-0.221231487	12.90359156	0.000516626
RCBTB1	0.118493429	6.859826539	0.000517735
WHSC1	0.062979473	6.12488812	0.000518117
SLC1A3	-0.292088893	9.675603849	0.000518364
CLEC2D	0.2487611	10.70210605	0.000518787
MFAP3	-0.145147457	7.099247013	0.000519746
LOC645196	0.098650262	6.382069342	0.000520252
TTC1	0.200132904	9.151451836	0.000522454
MYBBP1A	-0.159792469	6.469166077	0.000522521
NMNAT2	0.080439794	6.337254766	0.000523883
E2F8	0.104450652	6.047861152	0.000524783
SF4	0.12409252	8.055425763	0.000527658
ST7	-0.086263805	7.906222316	0.000529601
CDCP1	-0.050791734	6.127185307	0.000529819
C5ORF44	0.153729522	6.792816504	0.00052994
PXMP3	0.15066764	8.036806252	0.000531262
RPS19	-0.183094528	12.88968937	0.000531456

LMBRD2	0.06003304	6.333512784	0.000531777
ADAMTS7	-0.144628323	6.251813857	0.000532815
C2ORF29	0.14034968	10.4866821	0.000533019
CA1	0.088126344	6.011877648	0.00053714
MDH2	-0.122702756	13.58937257	0.000538728
GPSM1	-0.22053151	8.957165291	0.000539136
KLHL6	-0.090381541	6.189609112	0.000539315
HCFC1R1	0.189482835	9.530179924	0.000539569
LOC649422	0.18268789	6.815784728	0.000540197
C6ORF205	0.089814462	6.574245999	0.000540361
GRK6	-0.082725218	6.669477954	0.000540934
LYNX1	0.05937949	6.072666743	0.0005412
RPS18	-0.209046728	12.07646867	0.000542556
NSMAF	-0.136683139	8.956017315	0.000544628
SIGLEC5	-0.072937781	6.110898204	0.000546328
DDX5	-0.146381017	11.48917631	0.000546789
C11ORF51	0.188607739	7.533002217	0.000548121
MYBPH	-0.147108712	6.247121634	0.000549165
C10ORF141	-0.104259308	6.508140243	0.000551359
SERP1	-0.154598566	7.199266696	0.000551397
NFRKB	0.099892082	6.503886399	0.000553609
KALRN	0.070291216	6.116032457	0.000553809
CEACAM1	-0.134594641	6.993069853	0.000554447
AKAP1	0.260080886	7.741681547	0.00055547
GRPEL1	-0.168764958	9.390153424	0.000557732
HSPBP1	-0.094990055	6.564847338	0.000558146
LRRC42	-0.173980642	8.618199724	0.000558607
WDR73	-0.13082174	7.020278243	0.000559267
C16ORF59	-0.104000494	6.272813902	0.000561584
SENP7	0.144978987	7.52467219	0.000564084
STK36	0.166333768	7.92719583	0.000566356
TUBD1	0.208199358	7.01321028	0.000567868
CLYBL	0.182803676	8.561141565	0.000568576
LMOD1	0.135319135	6.602540872	0.000569162
IGSF22	-0.090853503	6.039098722	0.000570055
MAGED4B	-0.078017265	6.298224962	0.000571036
DEFB103B	-0.087109228	6.054302348	0.000572307
TANC1	0.197009279	9.327507437	0.000577514
PMS1	0.12204622	6.480900403	0.000579433
DPY19L1	-0.148547337	9.14578036	0.000580611
TBL3	-0.138179916	6.913705127	0.000582786
MIB2	-0.202635087	7.796155202	0.000583357
PEX6	0.225611569	7.310903892	0.000583754
LRP8	-0.086997336	6.137586201	0.000584114
ALG1	-0.111911141	6.442401426	0.000586274
PIK3R2	0.180752839	10.36912433	0.000587725
UBE2T	0.20559828	7.755767805	0.000587837
LOC284023	0.119982408	6.994052875	0.000587851
SC5DL	0.208827443	9.182687147	0.000590085
BCAR1	-0.186594437	7.850864357	0.000590589
ADARB1	-0.124037394	7.562555232	0.000593879

DKK2	0.104715336	6.248961372	0.000595103
CALCB	-0.150938338	6.151770101	0.000595985
KIAA1012	0.152804991	8.913372587	0.000598079
IL17RA	-0.132038571	6.405830216	0.00059841
RIPK3	-0.108993297	6.719873281	0.000599112
CARD11	-0.115283451	6.976630351	0.000599145
LOC339123	-0.155516577	11.29802019	0.000601587
MAP4K1	-0.08501271	6.328734948	0.000604142
NDUFC2	0.134281552	6.651447056	0.000605135
ZNF580	0.095277204	6.749418194	0.000610037
NAT1	-0.058695381	6.265216897	0.000610154
STC1	-0.379096757	7.160871221	0.000610174
KIAA1202	-0.175931053	6.811466726	0.000610542
SDCCAG8	0.105618466	6.849586418	0.000610984
PIGU	-0.140218836	8.394341083	0.000611905
SUSD3	-0.090548463	6.270784456	0.00061365
RHOBTB1	0.094541448	6.30006872	0.000613875
SPCS3	-0.219315571	7.253799947	0.000614851
POLA1	0.126164087	7.279412492	0.000615529
ADAR	-0.124240877	8.886213435	0.000615983
SAMD8	0.127043662	6.404202244	0.000616798
FKBP9	0.079883103	6.255381226	0.000617081
SMAD5	0.149949648	8.265821442	0.000618054
ZSWIM3	0.050731387	6.203760837	0.000620885
EIF2S2	-0.101352322	6.634519739	0.000621869
PHLPPL	0.103309307	6.497048929	0.000621885
EID1	0.169051935	7.469830637	0.000623795
LOC442421	-0.13327788	6.378795697	0.000624409
C19ORF53	0.144313973	10.12620714	0.000624445
RPL26L1	0.177273674	8.833850292	0.00062599
HELZ	0.165294505	7.90548577	0.000626507
CD36	0.256003203	11.42037982	0.000626657
RAB33B	0.282425277	7.694504591	0.000631108
LOC285016	0.318125486	6.906584091	0.000634768
C3ORF21	-0.157289024	7.65252966	0.000635641
ABHD14B	0.140400635	6.672094311	0.000637722
ENPP4	-0.178918234	7.794622584	0.00063826
SFXN2	0.107759651	6.626375246	0.000639839
TIMM8A	0.193339499	7.299845893	0.000642267
ABLIM1	0.171114002	11.51719504	0.000642519
GPR83	0.082518247	6.171315715	0.000643152
PIF1	0.084359875	6.195750431	0.000643554
BCAT2	-0.153624449	8.534048092	0.00064378
COL27A1	-0.125514733	6.273065825	0.000644891
LOC647326	-0.089526332	6.073588435	0.00064548
PEX11B	0.145545537	9.642765206	0.000646583
C10ORF58	-0.128900317	11.31667078	0.000647588
MALL	-0.242038633	8.205021869	0.000648633
PIPSL	-0.212109289	8.517705155	0.000652627
SMO	0.101828462	6.604417477	0.000653005
KBTBD11	-0.217580086	8.742841084	0.00065377

MGC33846	0.126499303	6.236767797	0.000659308
CRIM1	0.290127182	8.077099676	0.000660657
JMJD5	0.095969225	6.145946872	0.000661027
LARGE	-0.190091435	9.777486604	0.000663398
PEX13	0.09679274	6.961684273	0.000664305
MAP6D1	0.138696719	7.626677822	0.000664456
UNC50	0.125763689	10.2618236	0.000668611
RABL2B	-0.086809289	7.175362722	0.0006707
KIAA0363	-0.167421155	7.089869956	0.000671821
HOXB7	-0.113565641	6.136234172	0.000672459
COIL	0.102564224	7.954013342	0.000673333
ZBBX	0.105013429	6.62455412	0.000675494
RPA3	0.173089116	9.508372295	0.000675495
CDS1	-0.11742128	6.091122286	0.000675746
LOC643011	0.110094592	6.776110769	0.000676197
GOLGA2	-0.173230977	7.660549731	0.000676435
SMC6	0.197963015	6.752327464	0.000682403
PCOLCE2	0.273258807	11.12835111	0.000685079
METT10D	0.1219902	6.974474381	0.000685233
PSME3	-0.101591411	8.080307298	0.000685543
TTC18	0.119728423	6.427069159	0.000686538
AFF4	-0.248724005	7.587823684	0.000687783
SR140	0.128811495	7.963514322	0.000688439
ZNF573	0.183319482	7.589746016	0.000689155
C12ORF10	-0.131827099	9.301144238	0.0006896
FEZ2	0.155266761	10.07845153	0.000689807
ANKRD50	0.185055375	7.731761056	0.000691231
SYNJ2BP	0.250194093	9.702193904	0.000692659
BTBD12	-0.149932669	7.097898726	0.000692862
SS18	0.113368857	8.226633542	0.000695535
RHOA	-0.172407452	11.24832665	0.000696955
LOC643509	-0.180656234	12.6807459	0.000697943
PAN3	0.153724689	7.961682033	0.00070019
GTF2H1	0.179239423	7.193089859	0.000700918
DHPS	0.116313433	8.968356175	0.000702939
ODF3L2	-0.105429065	6.31051395	0.000705626
LOC731777	0.194001337	7.854632938	0.000705716
EEA1	0.107948823	6.517112504	0.000706578
CFL2	0.202684808	7.372035149	0.000707141
HSPB1	-0.218241934	14.00589844	0.000708692
TTF2	-0.143568838	7.132893234	0.000709038
FAM127B	-0.109920898	7.956317484	0.000709102
LOC136143	-0.175864549	8.277905155	0.000709659
OGN	0.266375603	6.93832029	0.000710309
BACE2	-0.143014539	7.111716835	0.000710857
FGF12	-0.654953529	8.197015462	0.000712215
FGFR1	0.081621562	6.255975433	0.000713338
LOC649169	-0.123651795	6.547561512	0.000713947
SLC22A16	-0.099350381	6.025601405	0.000714723
MGAT2	-0.162241735	7.448137549	0.000716871
LOC730092	0.085203409	6.404478528	0.000718547

ST14	-0.102224783	6.057782967	0.000722819
PLD1	0.249899344	8.200876831	0.000722863
POU5F1P1	-0.067888895	6.05975304	0.000729761
TNIP2	-0.167395364	6.390248869	0.000730199
UCP2	0.26351264	7.362766505	0.000731648
SLC35A5	0.176638707	9.061954971	0.000735034
MGC12966	0.161591411	8.07446631	0.000736721
HLA-DOB	-0.0912274	6.190171981	0.000737598
ZNF432	0.108602528	6.823452135	0.000737665
CTR9	0.117074966	6.928886499	0.000738339
ZNF513	-0.107176665	6.797199674	0.000740241
SLC16A4	0.085170738	6.301508481	0.000741336
ITPKB	-0.158402305	7.600769216	0.000741878
CHST1	-0.157050183	6.625392419	0.000742061
SPARCL1	-0.200550706	12.42252984	0.000743131
HSDL2	0.267310604	10.22059326	0.000744093
LIG3	-0.072051681	6.580507219	0.00074454
PLOD1	-0.191395592	10.50257783	0.000746402
ZNF260	0.122813374	7.339145985	0.000747213
PHLDA2	-0.117655442	6.142838661	0.000747301
QDPR	0.220484414	10.47118155	0.000747325
C3ORF64	0.189782212	7.779281733	0.000749479
GHDC	0.111664071	6.96801612	0.000754134
NOL12	-0.118101579	7.495048119	0.000760377
PCBP3	-0.104153541	6.144895983	0.000761792
ATRNL1	0.100072384	6.172028606	0.000765507
RSBN1L	0.137811921	6.977626654	0.000766427
ARHGEF4	0.089359877	6.10736365	0.000767321
RGN	0.176621	7.167441444	0.000768828
ZNF585A	0.107604081	7.07019425	0.000769352
PHF2	-0.11772827	7.521809638	0.000770982
LGI1	0.07348062	6.063184061	0.000771182
CASP7	-0.075361863	6.348729588	0.000772701
COPG2	0.235982784	6.791264512	0.000773224
CSHL1	0.066402545	6.356579738	0.000774051
ALG2	-0.064493219	6.231942867	0.000775709
TSPAN18	-0.324587292	8.153467719	0.000779534
FTSJD1	0.114663152	7.285395502	0.00078051
LOC654174	-0.097643854	6.298230263	0.00078185
PSAT1	-0.105729628	6.11650458	0.000784474
RAB12	0.164951475	6.72284146	0.000784936
DEPDC1B	-0.063936843	6.026934563	0.000786596
KIAA0513	-0.111786504	6.692525793	0.000787345
SOX8	0.223422844	7.390242507	0.00078982
ARHGAP30	-0.103531821	7.00302823	0.000790229
FAM39E	-0.189367279	10.59276751	0.000790726
VIT	-0.174429818	6.478627739	0.000792072
HIST2H4A	0.130022622	6.701397517	0.000793468
PCIF1	0.09087324	6.745910884	0.000796255
C1ORF170	-0.235921132	7.239141119	0.000796326
EBF1	-0.187247866	7.727137357	0.000798758

MARCH1	-0.071965084	6.069896545	0.000798763
ANXA6	0.107765791	7.438745002	0.000803753
ECT2	-0.19924235	7.084946357	0.00080533
SETD1B	0.106538573	6.708625436	0.000805991
PSMA4	-0.111596077	9.873664425	0.000807277
LYG1	-0.088418228	6.235431205	0.000809421
C1GALT1	0.178980169	6.928289777	0.000810447
MSR1	-0.088241593	6.118734672	0.000812687
SLC26A9	0.256469666	6.847141025	0.000814106
CCDC21	0.266150829	8.771140963	0.000819187
SF3A3	-0.141533562	9.307676294	0.00081983
TRIM47	-0.185604722	7.189719146	0.000820013
LOC346887	-0.151366106	6.493133895	0.000820345
NXF1	-0.136923415	8.8926719	0.00082048
PIP3-E	0.136202098	6.463967379	0.000822009
LOC653884	0.132337589	7.656538052	0.000822449
CR1	-0.053179	6.025249247	0.000829412
MGP	-0.361612701	12.24350673	0.00083074
PLAC8	-0.090160711	6.229150054	0.000830858
DIS3L	0.191148409	9.749964412	0.000831332
UBL3	0.151299558	9.803179565	0.000831382
CCDC56	0.11892658	10.00749694	0.000831907
NTN1	-0.198883244	6.913123609	0.000835109
C1ORF97	0.212282786	8.008718591	0.00083567
CCL23	-0.093382943	6.372312613	0.00083797
KIAA0859	-0.114039173	8.41691096	0.000838088
EAF2	-0.094214536	6.400319411	0.0008426
PJA1	0.107994653	6.958480416	0.000843101
CRELD1	0.161502528	8.337135804	0.000843439
LOC652671	-0.069703246	6.151176676	0.000843471
GART	-0.119258887	7.678079049	0.000843898
LOC339879	0.073428502	6.014969752	0.00084614
LOC644584	-0.138753252	6.623843202	0.000849129
RIN3	-0.116067403	6.286290589	0.000849518
LOC283547	-0.096986492	6.19456616	0.000849965
IMPA2	-0.229667233	8.540139188	0.000850911
LRRC33	-0.138381123	6.595975834	0.000851359
EID2B	-0.241093559	10.37482408	0.000852598
CLN3	-0.094328491	6.905343796	0.000852804
RBM22	0.125378932	10.63109576	0.000853829
RNF217	0.178740679	7.179333976	0.000855708
LRRC28	0.129335452	8.028255137	0.0008565
TERC	-0.121738614	6.137070857	0.000857415
LPPR2	-0.167488301	6.698511284	0.000858057
HLA-DQA1	-0.375944522	7.552628844	0.000859371
ZNF32	0.109401981	7.805327433	0.000859588
CNOT7	0.10363488	8.052985107	0.000860055
MED28	-0.143426454	8.243238934	0.00086332
LOC554203	0.228035124	7.161328871	0.00086519
TGM2	-0.291515836	9.815858821	0.000866694
TCP11L2	0.082662353	6.310630608	0.000867948

DTNA	0.140557207	11.36462029	0.000868984
PIGA	-0.149413311	7.186486235	0.00086969
YIPF5	0.140339233	7.047759409	0.000870557
SLC29A2	-0.213656867	7.432794895	0.000872769
SFRS7	0.184293562	8.981840006	0.000873077
LOC642197	0.16399163	9.419584477	0.000875765
LOC648366	-0.079577429	6.227228844	0.000876457
ITGA9	0.180584063	7.16452219	0.000879399
RNF40	-0.108349393	6.840640806	0.000882676
PELO	-0.169717817	9.72002435	0.000882804
LOC440345	-0.123093214	6.579965565	0.000883163
COG3	0.14235013	7.201127606	0.000883443
PROSAPIP1	-0.136439079	6.795636888	0.000884545
KPNA1	-0.146074491	7.941305578	0.000884888
TTC26	0.105025552	6.610207592	0.000886834
PALB2	0.09120276	6.685557756	0.00088795
TRPV1	0.060633612	6.184400509	0.000887978
KIAA1407	0.080479032	6.243874505	0.000889223
SOLH	-0.122601405	6.482619151	0.00089148
RPL22	0.224708545	10.97124724	0.00089431
CRSP6	0.103066554	6.316045779	0.000894566
CDT1	-0.100855187	5.984452562	0.000896435
HRB	-0.103661756	6.543053233	0.000902239
EPSTI1	-0.290828387	7.353591904	0.000907533
SNTB1	0.127328974	7.120762187	0.00090973
LRP10	-0.203113837	8.552612442	0.000910544
LOC131873	-0.165192467	6.326805611	0.000911849
CCDC127	0.177600786	7.451753579	0.000912239
IGFBP6	-0.275119081	9.445826957	0.000915816
PNMA1	0.153138255	7.195227566	0.000917564
C9ORF40	-0.121561196	6.771226663	0.000921242
ETV4	0.080779314	6.247893569	0.000921491
PSME1	-0.206620274	9.704548137	0.00092309
NPSR1	0.11108004	6.913753937	0.000925717
PARP10	-0.137223447	6.596297236	0.000926106
ZBED1	0.195489982	10.5673042	0.000927782
FLJ38482	0.203444263	8.287041462	0.00092903
IRF9	-0.215317092	9.282947179	0.000929356
LOC400657	0.164646363	7.763692554	0.000935795
RPAP3	0.115969959	7.291544527	0.000936197
PCSK6	-0.05295165	6.106573001	0.000937338
BRD4	-0.070129549	6.310394787	0.000939136
FEZ1	0.222334774	8.406793323	0.000939561
SMARCC2	0.143683153	7.303590538	0.000941254
C1ORF85	-0.150474278	8.873367088	0.000943274
LOC647958	0.11768893	6.1794526	0.000944613
DPP9	-0.17977662	8.794881387	0.000945112
C16ORF75	0.134845234	7.911560687	0.000949916
PABPN1	-0.192919858	8.883766513	0.000950076
ALKBH5	-0.138731028	12.63824504	0.000950102
MYO1C	0.148847811	7.762090493	0.000952167

HLA-F	-0.232797764	8.469511001	0.000952566
RAD51	-0.087130271	6.832995513	0.000953722
LOC644852	-0.137979323	6.563395869	0.000954686
C21ORF33	0.18810758	10.85715633	0.000955206
SP3	0.118870006	7.121127923	0.000955615
MRPS21	0.162615593	10.24654031	0.000960035
KIF13A	0.099955066	6.279756879	0.000966906
CALCOCO1	0.16856093	8.205536564	0.000972456
MPI	0.15994275	7.102023248	0.000973263
KLHL20	0.147107023	7.532156987	0.000973805
GUCY1A3	0.256165129	9.201590673	0.000974455
RPAP1	0.11541229	6.788036297	0.000978621
IRAK4	-0.107491659	6.336855732	0.00097974
POLR3A	-0.099996363	7.605511022	0.000980107
C3ORF70	0.138936576	7.078831566	0.000982933
GPR160	-0.088066623	6.140137569	0.00098313
CCNE2	0.071454974	6.149352511	0.000985146
BMP2K	-0.063755415	6.538423716	0.000992754
LDHB	0.201617079	12.70232893	0.000994991
NDUFAF2	-0.169855405	10.04675927	0.000996654
PSMC4	-0.197568318	8.192467976	0.000996672
AQR	-0.099144261	6.946942248	0.000998312
EIF3EIP	0.26822837	10.91211923	0.001000523
SCG5	0.130543937	6.327369177	0.001000771
PMS2L1	0.056941927	6.354628791	0.001006293
MAN1A2	0.070789319	6.573998355	0.001008083
CUGBP1	-0.121347273	6.828529489	0.00100926
NUSAP1	0.126355151	6.443760534	0.001015315
LOC389033	-0.097629276	6.307048662	0.001015672
VIM	-0.248432641	11.65299602	0.00101633
PRNP	0.16325948	11.71800383	0.001016989
PXT1	-0.074521873	6.030179965	0.001018134
KIAA0367	0.288212294	6.615745285	0.001018265
NBEA	0.22103698	7.877729198	0.00101848
C21ORF128	-0.12118413	6.496328189	0.001018487
PPP1R3B	0.133708334	6.516452435	0.001019499
ICAM3	-0.19863783	8.640491325	0.001022711
RAN	-0.197712353	8.494485455	0.001023818
HYOU1	-0.205915318	7.670612907	0.001025516
PPP3CB	0.154530446	10.14739624	0.001029004
GGA1	-0.18378926	11.8654527	0.001031165
KIAA1211	0.052348643	6.128498506	0.001031599
HFM1	0.061188245	6.166248768	0.001032366
RTN3	0.156527812	7.787313225	0.001032989
PCDH17	0.346988688	8.198838312	0.00103952
MGC3207	0.178679355	8.018775554	0.00103994
DCTN2	-0.116977654	11.2251892	0.001043327
KLK11	-0.073471627	6.142885194	0.001044877
LOC653602	-0.118441443	6.177703447	0.001045391
TNK2	-0.139183546	7.285091904	0.001046289
NUAK1	0.189307082	10.31105838	0.001046941

SEZ6L	0.098336437	5.974793084	0.001046973
ANKRD12	0.15377284	8.390778974	0.001048189
SNHG11	0.109251615	7.130828109	0.001048478
MRPL16	0.252003243	8.570190638	0.00105058
C14ORF172	-0.142994434	6.549045636	0.001051534
FLJ44124	-0.252803707	10.8159505	0.001053883
TMEM38A	0.171750781	6.930659911	0.001056375
SFRS12	0.155381273	7.805413393	0.00105802
PDGFB	-0.095887808	6.701765101	0.0010591
SHCBP1	-0.180343427	11.56057087	0.00105994
PALM	-0.176775188	7.789914136	0.001060115
TROVE2	0.098359794	8.682651799	0.001061216
TP73L	0.128355072	6.17991219	0.001063001
PRDM11	-0.081676397	6.042960662	0.001063031
CACHD1	-0.079579073	6.359797981	0.00106342
USP9X	0.112932921	8.437412625	0.001064612
ZNF649	0.105245469	6.909544774	0.001069203
LOC283683	0.179664751	7.117065799	0.001069456
EDA	-0.04517612	6.165292798	0.001071211
C9ORF30	-0.199971614	7.903679754	0.001072026
FAIM	0.064979538	6.208432639	0.001074157
TP53RK	-0.142353147	7.163075501	0.001076008
ETFA	0.215307165	11.97547416	0.001077337
FLNA	-0.150103941	6.280215008	0.001078351
ZNF85	0.120183168	6.05146579	0.001079039
ITGAV	0.17088748	10.60657091	0.001079608
FTHL7	-0.167013875	12.56410338	0.001080036
SEMA6C	0.2300517	7.655349592	0.001080053
PTPRN2	0.083294203	6.362323272	0.001080681
ZNF518B	-0.159534503	8.273618702	0.00108387
ZSWIM4	-0.12022577	7.027238836	0.001084336
TMF1	0.092505464	6.744941957	0.001084969
RN7SL1	-0.155806746	14.29050442	0.001085076
OSBPL7	0.093689026	6.822356792	0.001087008
CD34	-0.19006182	8.926006877	0.001087119
TRAPPC6B	0.143123251	7.364060676	0.001090003
RALY	-0.162375489	9.424613547	0.001091245
TBC1D19	0.117710855	7.102084769	0.001093199
CHRNA5	-0.228612739	9.565613032	0.001094822
MPPED2	0.159020705	6.565582066	0.001095198
RPL12	-0.185263488	11.8076169	0.001100646
SEMA4C	-0.128235914	6.286933804	0.001100895
RABGGTA	-0.089657986	6.490096151	0.001104351
VASP	-0.156944318	6.336877227	0.001105597
PTGS2	-0.136705126	6.463664008	0.001106228
SNORD100	-0.089157368	6.222092952	0.001108043
MBD6	-0.158243447	8.492714569	0.001108053
KIAA1553	-0.063642277	6.12007964	0.001111006
ZNF165	0.081843245	6.433650693	0.001114311
GGPS1	0.101660226	8.231312965	0.001115582
MGC33556	-0.109090371	5.922324278	0.00112198

NRCAM	-0.103447087	6.00391128	0.00112269
XTP3TPA	-0.166275652	8.801624683	0.001122697
MTIF2	0.178151066	9.148568678	0.001125883
IL1RAP	-0.122252277	6.427506183	0.001126571
ARHGEF11	0.068312338	6.721802776	0.001127079
MAP1S	-0.136060929	7.307855701	0.001127476
PACSIN1	0.285025002	8.114315392	0.001134429
CSE1L	-0.114818055	8.731770255	0.001137658
EVI2A	-0.07141115	6.216392138	0.001144193
ZNF772	0.119777065	7.173671681	0.001148557
C12ORF5	-0.149820259	7.083138413	0.00114964
HOOK2	0.188735831	8.962502062	0.001152413
RGS10	-0.139169351	6.852372996	0.001157977
LRRN4CL	-0.13803762	6.37837876	0.001158093
PLA2G5	-0.184205073	8.270715108	0.00116081
IQCC	0.137107899	6.560423568	0.001161881
LOC134466	0.103744591	6.117533518	0.001162183
PRPF18	0.143045901	7.594362204	0.001162247
SPECC1L	0.166560559	10.32788911	0.001163382
HEBP1	0.175003749	9.411531295	0.001168901
OPLAH	-0.205435947	8.910885557	0.001169368
HIST1H1C	-0.275258146	10.27814261	0.001170863
BZW1	-0.056458487	6.187507561	0.0011742
ZNF91	0.287367615	9.815224865	0.001178369
A2BP1	-0.157313556	6.932652955	0.001180007
BCAP31	-0.196914569	9.953383721	0.001181209
TRIP4	0.10964873	7.781755388	0.001186131
LOC653717	-0.149187201	6.57036891	0.001186152
ABRA	-0.325423052	6.97631744	0.001188113
LOC731366	0.080389576	6.192092544	0.001192232
SERINC1	0.179847047	9.499204183	0.001192312
LOC648570	-0.068196291	6.008594276	0.001192552
CASK	-0.092116452	6.813019197	0.001195279
CHCHD4	0.17982889	9.988551191	0.001201069
CUEDC1	-0.188348035	9.916362604	0.001201452
CAPNS1	-0.134852527	10.78911235	0.001204722
DYNLT1	0.209016545	10.12324815	0.001204879
LOC653583	0.127505424	6.562344654	0.001205573
SLC16A7	0.168051291	6.997609231	0.001209668
C1ORF54	0.254481912	9.402363697	0.001210511
TINP1	0.178949981	10.41931239	0.001211068
SNTB2	-0.139808566	7.458045053	0.00121297
TSPAN3	-0.162357526	9.368159569	0.001213912
FKTN	-0.192883083	11.48632909	0.001213959
SUPT4H1	0.129191629	8.93798326	0.001220364
UBQLN4	-0.152631567	9.230670939	0.00122065
FAT3	0.117571089	6.238750153	0.001221973
SLC30A5	0.108633217	7.964775359	0.001224512
GJC1	-0.185600004	11.95739757	0.001226163
WDR43	-0.161641941	6.663243324	0.001226897
ASPH	0.199794598	7.340017194	0.001228841

PTH2	0.1192436	6.246210407	0.001230672
APOL1	-0.076881649	6.177074411	0.001230775
LAG3	-0.087951287	6.228869869	0.00123666
C17ORF39	0.078763267	7.523082248	0.001245974
CTDSPL2	0.07676514	6.786585196	0.00124641
PHYHD1	-0.132160536	6.573774955	0.001247583
C1ORF53	0.225134537	8.605091489	0.001248841
C4ORF42	0.089714828	6.28952252	0.001250222
GPR180	-0.145541048	6.85219149	0.001250298
MYO1D	0.229295962	7.112782796	0.001250924
CCDC14	0.126180681	8.13907749	0.001251037
C20ORF7	0.156794731	8.662614414	0.001252187
TAOK1	0.263304096	7.562518332	0.001252988
LOC646574	0.108739379	6.341080643	0.00125836
CDV3	-0.223533069	9.016569734	0.00126032
DACT2	0.164389929	6.373117804	0.001260676
TSG101	0.115672324	10.37726744	0.00126087
FAM83B	-0.078901996	6.039818395	0.001263135
TM2D1	0.166531701	9.757369101	0.001269517
TM4SF1	-0.198121449	12.02058061	0.001270813
LOC652837	0.183204667	6.562655316	0.001274383
PPM1J	0.110890945	6.529216794	0.001274725
VSIG2	-0.088558379	6.387628147	0.001274955
LOC651143	-0.136079124	6.93078918	0.001275446
RNF5	-0.11008188	7.74935465	0.001281919
ETFB	-0.181501997	11.15016505	0.001286849
C14ORF108	0.124105184	8.523202768	0.00128877
ACACB	-0.269950151	10.12939619	0.001290051
STOML1	-0.133698324	7.558492328	0.00129134
ASCC3	0.119156197	7.107744265	0.00129292
CD320	-0.148288659	7.937095016	0.001294679
SLC19A2	-0.099536288	6.563114008	0.001299008
SNORD56	-0.090610895	6.424490784	0.001299216
NCOA3	-0.116208195	8.202575676	0.001300224
VEGFB	0.160121987	10.64486421	0.001300467
ADH1B	-0.278010956	6.868448621	0.0013015
RARS2	0.14908719	8.585940629	0.001305438
GLTPD1	-0.186502182	7.774496405	0.00131061
ZNF767	0.112751727	7.315550886	0.001311915
HIST1H2BK	-0.206274637	8.958028411	0.001312234
EGF	0.102249196	6.517656062	0.001315194
PARD3	-0.116734944	6.31464569	0.001316444
C9ORF80	-0.196300173	11.37465554	0.001320329
FOXD2	0.096863967	6.166624692	0.001321159
PRRG1	-0.160961661	7.950832009	0.00132274
SIGLECP3	0.081673908	6.155683767	0.001323129
ITLN1	-0.376097315	6.621436936	0.001325494
PDE1B	-0.085746388	6.164338331	0.001329524
FAM109B	-0.127256586	6.58540465	0.001330782
FCHO2	0.180799721	7.193422485	0.001334244
LOC727849	0.061630894	6.121161357	0.001339783

ZNF658	0.127022501	6.427308257	0.001343669
LOC644544	-0.091103084	6.079493015	0.00134607
DKFZP586H2123	0.255331418	7.604763926	0.001346383
BEAN	-0.062650025	6.053920077	0.001352757
ACTR8	0.12290878	7.024683396	0.001353075
MRPL44	0.138243157	7.76603808	0.001354413
C8ORF33	-0.105122614	8.68092134	0.00135475
DNAJB9	-0.205976287	9.542579289	0.00135595
ISG20L2	-0.166138983	7.419839477	0.001361211
MT1H	-0.096228089	6.080423312	0.00136476
SH2B2	-0.08612949	6.049020616	0.001364891
TAF4	0.154106998	8.146779732	0.001366267
LOC130773	0.134110577	7.256323898	0.001366732
MED10	-0.177404563	9.136137753	0.001367872
TRAFD1	-0.242548744	7.901614086	0.00136826
SNORD35B	-0.10485545	6.293280901	0.001372151
OLFM4	0.108568435	6.248953041	0.001373606
RABEP1	0.18627831	9.250023086	0.00137573
APOO	0.191199321	9.333120374	0.001376199
FXYD2	-0.045602698	6.100510268	0.001376339
C1ORF19	0.143832323	9.246193234	0.001376707
KIAA1542	-0.133116953	6.115398619	0.00138131
ANGPTL2	0.302927777	9.832598268	0.001383811
EFNB2	0.256033084	9.287637202	0.001384955
MS4A2	-0.094527393	6.193862173	0.001389744
HLA-DPA1	-0.287132438	9.823149048	0.001394662
ZNF362	0.164318132	9.03296107	0.001396093
DLL1	-0.267819586	7.873340754	0.001396752
MCM5	-0.124676755	7.050574714	0.001397707
LOC653489	-0.215833524	8.662195139	0.001398661
LOC401317	0.285514548	7.105945931	0.00139902
CYB5D1	0.159748085	8.551652481	0.001400913
GAS2L1	-0.055387272	6.378701521	0.001403232
LOC651064	0.155981193	10.25203288	0.001405393
ZNF397	0.089269554	6.435986001	0.001408183
COPS6	-0.118283818	8.54565592	0.001414204
CDRT1	-0.053208809	6.110063623	0.001416373
LOC642947	-0.186374534	11.89634484	0.001417395
GLT8D3	0.089352573	7.093135171	0.001418357
C3ORF59	0.133352618	7.569228244	0.001422889
INDO	-0.380965836	6.445561268	0.0014239
C19ORF43	-0.225008762	9.058188199	0.001424094
PCGF5	0.15911832	6.94293213	0.00142632
FIG4	0.102844182	7.599510542	0.001427297
QTRTD1	-0.093805338	6.458068418	0.00142953
CTDP1	-0.06654	6.580933567	0.001430416
COL13A1	0.131730292	6.565506493	0.00143329
DYNLRB1	0.108138075	9.93568218	0.001435428
LOC648059	-0.12653458	6.803696523	0.001435796
ZNF431	-0.092673576	6.423305686	0.00143819
JRKL	0.081252286	6.37864154	0.001440208

ZNF747	0.095103785	6.361974615	0.001440262
CHRNA3	0.093007607	6.41849296	0.001447435
LOC286297	-0.117364095	6.572779538	0.001450025
ZFYVE1	0.072584287	6.557197202	0.001450487
C17ORF71	0.158742418	7.057920407	0.001451204
MTRF1	0.117489523	6.804976375	0.001455452
LOC645888	0.093344513	6.361708538	0.0014562
SMAD7	0.126513609	6.945938406	0.001458689
ST6GALNAC6	0.199703395	8.340363715	0.001460219
HIST1H3D	0.098245231	6.465746116	0.001461699
REC8	0.099887341	6.726348538	0.00146464
CCDC117	0.114553566	7.417813168	0.001466244
LOC651790	0.078713731	6.101938478	0.001469637
GSTT2	0.23978597	6.916624026	0.001470638
FBXW7	0.15545844	8.122841529	0.001471626
AYP1P1	0.162464957	8.40008984	0.001472237
PTGDR	0.075006406	6.218189121	0.001473117
LCN1	0.116025307	6.247406976	0.001475646
ARHGEF1	-0.094133543	6.295159959	0.00147754
IDH1	-0.157882331	7.964555813	0.001478063
WHSC2	0.150713802	6.574566603	0.001479873
MXD4	-0.188410998	10.29485511	0.001486272
LOC441196	-0.077706543	5.98287625	0.001488113
KIAA0355	-0.161154116	9.700763029	0.001490085
YIPF6	-0.125487224	9.046804378	0.001495051
CLTB	-0.156984899	10.76563838	0.001495137
MRPL4	-0.102832243	7.018023208	0.001502503
SOD1	0.154903415	12.9525343	0.001504086
DEFA1	-0.094977945	6.052807732	0.001506108
MAX	-0.069463164	6.731608582	0.001506841
BRWD1	0.092114069	7.397203794	0.001507523
MYRIP	0.053953635	6.164909658	0.001508144
SH2B3	-0.161312691	9.516920065	0.001510075
PODXL2	0.106569465	6.472246862	0.001510768
RNF135	-0.065960166	6.567725602	0.001514201
C11ORF42	-0.084365231	6.028444207	0.001514308
IFRD2	-0.189966537	7.694956246	0.001515314
COQ7	0.138534854	7.588914374	0.001515337
SLC25A15	-0.084504212	6.736491606	0.001516621
HYPK	-0.215595205	10.00642272	0.001522015
WDR68	-0.103007295	8.578418565	0.001522097
MCL1	-0.180912833	8.366324342	0.001525371
FLYWCH1	0.120762953	6.726062927	0.001529778
LHFP	0.169038125	9.581078198	0.001531511
LTC4S	0.139468939	6.710468524	0.001534155
GLTP	-0.147900643	10.44180315	0.001534403
ARMC10	0.153656664	8.288621684	0.001537666
SIGLEC9	-0.083246666	6.110568755	0.001538429
SEC23IP	-0.106055468	7.470837923	0.001538863
TMEM87A	-0.171403434	10.91441415	0.001541711
C4ORF36	0.086219849	6.234768506	0.001550336

SCARF2	-0.096418713	6.301467554	0.001552113
CREB3L2	-0.15116327	9.088252773	0.00155497
LOC441775	-0.195887382	11.84713345	0.001557902
DCBLD1	-0.091884138	6.557422375	0.001561507
SOHLH2	0.090505219	6.206879281	0.00156239
RAMP2	0.153806237	7.650971701	0.001564853
MGAT4B	0.137780994	7.622801503	0.001565764
SEMA3E	-0.244833891	9.292180649	0.001568478
PBLD	0.052239354	6.209240256	0.001572571
SLC25A14	0.119321673	7.148284322	0.001578592
CYP26B1	0.194710516	6.860034562	0.001579854
PPP1R3F	-0.141428759	9.038743209	0.001583289
SAA1	-0.089525211	6.15754866	0.00158371
LOC652672	-0.151148498	7.72599545	0.00158601
PRKD1	0.12271719	6.878470802	0.001588692
C8ORFK36	0.061181885	6.110186033	0.001590474
UQCC	-0.160577515	9.156583131	0.001593981
SIGLEC1	-0.077960198	6.084161158	0.001595402
C19ORF28	-0.12062071	6.292765754	0.001603182
RAB34	-0.153924441	7.37575117	0.001604133
SCARA3	0.095148362	6.358013922	0.001604775
CKM	-0.154988628	14.33693673	0.001616368
RNF215	0.125905563	6.39680748	0.001616683
ZNF823	0.103762033	7.078625655	0.001616738
FAM101B	-0.086712686	6.42736784	0.001618772
HIST1H3H	0.075465265	6.111147466	0.001620716
LOC645098	0.085424032	6.126629611	0.001621375
SUPT6H	0.130130184	7.166062726	0.001621701
ZNF283	0.100717298	6.493117947	0.001621854
ERH	0.220950701	9.271910775	0.001621949
IFT57	0.122894398	7.378205385	0.001622686
RBM3	-0.132995277	6.612643851	0.001624454
RPP21	-0.137049062	10.17037546	0.001624805
EEF1G	-0.257181225	11.66790296	0.00162555
FEM1B	-0.104017377	6.658609525	0.001630072
NLRC5	-0.093048646	6.42097747	0.001631317
COG4	0.135473999	7.544347811	0.001632044
LOC653650	0.074679272	6.260401451	0.00163731
VIPR1	-0.171711681	7.296747714	0.001638196
KIAA0999	-0.146517385	8.54869667	0.001640884
SLC19A1	-0.105440467	6.185854326	0.001643361
CREBZF	0.159509962	6.938076184	0.001648269
FLJ45248	-0.109989896	6.238807706	0.001652371
STARD3	-0.140865429	6.406349773	0.001654739
ZNF443	0.084758325	6.539759727	0.001657806
NY-SAR-48	0.062103638	6.570973085	0.001663964
C15ORF38	0.108149504	7.18235985	0.001672312
ZBTB3	0.186227942	6.96513085	0.001672984
CCDC74A	0.083685453	6.22010177	0.001674893
TM9SF2	-0.176831296	9.726416422	0.00167561
C21ORF24	-0.196303266	11.68625455	0.001680386

PILRB	0.124365217	6.740626965	0.00168258
CNTNAP3B	-0.057617671	6.098636269	0.001683419
VENTX	-0.10864249	6.785358279	0.001685871
CAD	-0.10556473	6.463203146	0.001690197
SIX5	0.167045858	8.790084168	0.001693568
PLAT	0.246778761	7.271542149	0.001693659
C12ORF60	0.13212333	6.781009876	0.001695369
ZNF134	0.106269021	7.178075059	0.001696642
FMNL2	-0.094041318	6.379721124	0.001699879
SEPT2	0.147546393	10.51305747	0.001706789
PURB	-0.14311102	9.311274467	0.001716079
BCR	-0.050695805	6.313742918	0.001717525
NFIA	0.160184051	9.089631397	0.001724161
TBC1D5	-0.09931151	6.232844462	0.001730293
SFRP5	-0.085449223	5.9774072	0.001735069
BRCA1	0.074363807	6.400345306	0.001735094
LRRC48	0.047055911	6.158637833	0.001735144
C9ORF66	0.108709477	6.657047083	0.001735624
ACP6	0.172706469	7.230836717	0.001738009
LOC148137	-0.085064979	6.263523944	0.001739405
LOC730994	0.138015105	7.230151141	0.001741127
NUDT13	0.101280718	6.635453449	0.001742047
TCP11L1	0.088115483	6.533523555	0.001747884
OR4D1	0.104857792	6.189237358	0.00175054
POP7	-0.142806632	8.044764623	0.001750593
NOD1	-0.129281616	7.169250399	0.001755419
LOC197350	0.109075867	6.320150524	0.001756456
PKN3	-0.133025948	6.779975945	0.001760563
FREQ	-0.221092375	6.951866893	0.001762098
BAT3	0.127275488	8.704971945	0.001770604
PEPD	-0.133189521	9.25554139	0.001775697
NEIL2	0.176050444	8.045248382	0.001776428
TMEM132A	-0.107562629	6.488775335	0.001780022
PAFAH1B2	0.110782	6.661144563	0.001781069
OGG1	-0.076926855	6.541545506	0.001782989
TNFAIP8	-0.112953422	6.344719218	0.001783396
ATP13A5	-0.113351568	5.930345314	0.001789694
C17ORF69	0.12140672	6.591797096	0.001793751
GPR65	-0.08121671	6.303693354	0.001797748
GPR84	-0.083391314	6.171123795	0.00179998
LOC643287	-0.290357453	7.943193187	0.001800993
LHX2	-0.089492597	6.007391028	0.001802892
FAM26F	-0.098259481	6.437274251	0.001805848
KL	-0.116155275	6.64565601	0.001806105
TPRKB	-0.166097868	7.549266721	0.001806935
LOC729196	-0.076231666	6.071087161	0.001808829
TBCB	0.220209614	9.687818557	0.00180906
MGC3032	0.07255543	6.584725052	0.001809113
HHAT	0.104601672	6.128482301	0.001810842
AIFM1	0.184587947	9.32541964	0.001817134
HGD	0.068595583	6.161392018	0.001818849

MR1	-0.119429927	6.374258489	0.001823114
PSMG2	0.150522924	9.755724366	0.001826566
SLC38A1	-0.227564929	8.845955399	0.001827778
RAB9B	0.240824994	8.945455967	0.001832337
WNT5B	-0.074896938	6.352505454	0.001833849
LOC205251	0.237255158	9.99058324	0.001835239
LOC375190	-0.098460076	6.197267257	0.001835845
LOC401623	0.101457813	6.238451708	0.00183814
TM9SF3	-0.224086574	7.91725001	0.001838894
PDK2	0.149189582	7.892257487	0.00183901
FAM8A1	0.136672006	7.513508758	0.001841142
RETN	-0.150305547	6.00430833	0.001842158
ZNF628	-0.076435327	6.122605464	0.001842802
FAM113A	-0.119995903	6.814335595	0.001843052
IQCA1	0.083060059	6.184407969	0.001846529
LPXN	0.124002295	8.197999012	0.001849564
CXXC6	0.082895328	6.132216714	0.001853039
GAL3ST3	0.105612646	6.360611319	0.001853226
CIB1	-0.155106146	11.60738272	0.001854478
C14ORF142	0.18098894	8.205636655	0.001855624
SIRPA	-0.192369605	8.947417249	0.001857512
EARS2	0.157088344	7.517975938	0.001857703
CCL26	-0.138511656	6.299843549	0.001859446
KLHDC5	0.183311688	8.044229918	0.001868113
ZNF652	-0.185699006	9.763394396	0.001873946
MGC16703	-0.157665175	12.93912702	0.001879104
ZNF367	0.080533883	6.143798151	0.001880006
GSK3A	0.103564462	6.67719405	0.001886003
CCS	-0.105185239	7.301177786	0.001890529
VWA1	-0.101600881	6.519954644	0.001892581
PRPF4B	0.069905925	6.61185902	0.001897701
C2ORF24	0.148324756	8.108693748	0.00190636
RAB1A	-0.124729479	8.519302535	0.001906803
C12ORF43	-0.096566681	6.993534058	0.001914001
ZDHHC17	0.133156855	7.988285466	0.001918847
TP53BP2	-0.139991414	7.538578364	0.001920048
LOC727726	0.083176841	6.824553716	0.001920493
P2RXL1	-0.082519048	6.035286832	0.001922997
MRS2	0.1702504	7.642676304	0.001923239
RPL27	-0.140104162	13.10377199	0.001927487
GCHFR	-0.162825886	7.074217085	0.001930822
THBD	-0.167550228	6.302109802	0.001931401
DJ222E13.2	-0.08818372	6.147384869	0.001931865
NGFR	-0.15639812	6.102323448	0.00193435
HSD17B8	0.215819667	8.540591331	0.001938825
CHD5	0.084966348	6.126075471	0.001939373
HOXA4	-0.089888421	6.023187884	0.00194093
C20ORF29	-0.124584137	7.045489521	0.001945741
SLC23A2	-0.062274455	6.276768331	0.001950279
LHCGR	0.125584916	6.158460772	0.001951746
PID1	-0.188726106	7.318463646	0.001953063

MALT1	-0.106187425	6.827633759	0.001953464
KLHDC1	0.058559977	6.128199887	0.001954561
KRTAP20-2	0.084714548	6.380162734	0.001955113
MIER1	0.092386561	7.536991125	0.001956447
FAM71E1	-0.096787551	6.602466791	0.00195936
CHMP2B	0.163893375	8.553460136	0.001959411
AMACR	0.123837595	6.665178733	0.001963358
DUS1L	-0.137469469	6.829330901	0.00196968
PHTF1	0.170231206	7.195253022	0.00197073
ADAM23	-0.228350158	8.35238273	0.001978324
USF2	0.159415964	8.374642462	0.001981906
ARL5A	0.125525497	10.36952326	0.00198957
LOC441246	-0.177046069	12.68261547	0.001989571
IL1RL2	-0.085395631	6.07275742	0.001990151
PLXNA1	-0.151542801	7.754667639	0.0019921
PLD2	-0.111773879	6.4865538	0.002003686
LOC653453	-0.080521796	6.042534098	0.002003974
CENPO	-0.057580579	6.119579703	0.002013166
LOC652844	0.097802883	6.17431392	0.002014009
KCNS2	-0.083583138	6.126731075	0.002015402
ZNF436	0.132611617	6.480837952	0.002016463
ITPA	-0.157219728	7.355846855	0.002020493
GABBR1	0.093295163	6.81364194	0.002023402
ODZ3	0.174843718	7.064242604	0.002024053
CA13	-0.128467381	6.403834331	0.002025134
LOC653513	0.389338091	9.607271951	0.002031921
POL3S	-0.080582616	6.123200398	0.002039717
CCDC49	-0.095569814	7.36660082	0.002042417
C1ORF77	0.12098377	9.653047216	0.002043887
PRAF2	-0.105121247	6.581541182	0.002049583
MND1	-0.055623548	6.06085957	0.00205467
C5ORF25	0.069069647	6.400657432	0.002061692
LOC643224	-0.06357265	6.317099062	0.002063469
DFNB59	0.081246851	6.290586688	0.002069965
WWP2	0.066466221	6.724473739	0.002072321
NLRP8	-0.280572082	10.21471767	0.002072521
LANCL2	0.122161576	7.738579324	0.002073653
SYNGAP1	0.086546212	6.247216734	0.00207822
HSN2	-0.077767022	6.155228502	0.00207838
FLJ90757	0.109206955	6.660457716	0.002080096
PPP2R3B	0.186232213	8.462807064	0.002081217
CCDC81	0.087111636	6.123277872	0.002085153
CASP9	0.09743975	7.098414932	0.002088362
MBD3	-0.145212563	7.377623266	0.002091052
ATP10D	0.081890953	6.241272414	0.002092015
PLAA	-0.076518767	6.472466185	0.002094795
NDN	0.20569081	10.86874738	0.002095687
C3ORF17	-0.088748786	6.824678915	0.00209791
TXNDC16	0.097651231	6.277045958	0.002099879
MOSC1	0.320520933	7.32081392	0.002101585
KCNA7	-0.11283017	6.052544451	0.002106706

GLB1	-0.105656654	8.223286976	0.002109985
DIP2A	0.055604834	6.573529755	0.002112908
MAK10	0.087691445	6.929280261	0.002122237
IHPK1	-0.112888001	8.019259664	0.002123972
TTC19	-0.150459281	8.797626973	0.002126219
GPX3	-0.184371414	12.37530002	0.002127022
NDUFA9	0.162413232	11.28955862	0.002128485
XK	-0.173707774	7.49317181	0.002132018
SEPHS1	0.105299957	8.269804508	0.002132517
LOC401093	-0.179116066	7.145895984	0.002132533
STRN	0.112778488	8.035837461	0.00213283
ZNF599	0.059447944	6.127932878	0.002135055
CDC42	-0.130979849	7.052607374	0.002138284
C9ORF78	0.113473968	10.09037393	0.002139488
PPP1R15A	0.207796995	10.93643719	0.002140274
FZD6	0.185022406	6.920298093	0.002142228
MARCH2	0.127088422	8.083559656	0.002155849
WDR47	0.118576882	7.018522429	0.002164246
GUSBL2	0.116138742	7.000480851	0.002164287
RPS19BP1	-0.189009539	8.655524291	0.002164632
DNAJC25	-0.125026185	7.631146709	0.002170261
AKIRIN1	-0.167440423	8.117847537	0.002171514
C9ORF150	0.110242283	6.314011038	0.002177145
LOC344405	0.09931544	6.607779322	0.002177392
PRMT7	0.169265865	6.956333473	0.002178624
CIAO1	0.093835471	9.732274411	0.00218552
GARNL4	-0.196596173	7.800410205	0.002189152
C2ORF42	0.14245829	7.678277562	0.002194022
PCBP1	-0.120783686	11.70815543	0.002194557
XRN1	0.116123805	7.577518185	0.002200225
SARM1	0.096374786	6.585129907	0.00220168
TTC27	0.130771698	8.366558425	0.002211755
MAL	-0.254385277	7.529732994	0.002213193
KIAA1324	-0.09140361	6.028438657	0.002226618
PTPRO	-0.072668589	6.144500508	0.00222897
NARF	0.150577137	8.785225772	0.002229989
SF3B14	0.139662397	9.751005426	0.002233731
CHD1	-0.174885858	8.385200599	0.002236999
PRKAG1	-0.232259475	8.387831806	0.002238332
RIMS3	-0.091072295	6.6693562	0.002240251
C8ORF37	-0.198362859	10.67839821	0.00224527
LOC554206	0.094871749	6.639198581	0.002245503
SLC39A10	-0.174244429	6.733386667	0.002247096
C8ORF45	-0.242144941	9.551033415	0.002248771
SPIN4	0.124975429	6.625092677	0.002250399
UROS	0.174526822	11.22190118	0.002251179
FHDC1	-0.070056965	6.18396744	0.002253269
ARHGAP23	-0.15361708	8.170077518	0.002256397
C19ORF23	-0.108516274	6.366423264	0.002263208
PCDHB2	-0.127850964	6.659429521	0.00227626
PHF17	-0.080946287	7.137738664	0.002284962

GALIG	0.170176194	7.087734537	0.002288591
RNF141	0.192065412	7.183259681	0.002297903
NICN1	0.180131319	9.445388285	0.002304738
MYH4	-0.115541359	6.392191806	0.002305671
TMEM209	0.119527193	7.749798175	0.002313521
ACTN4	-0.174237211	8.474977165	0.002317782
FVT1	-0.146347558	8.775828633	0.002325667
IL4I1	-0.06005179	6.030807009	0.002328385
LOC646195	-0.117409857	13.87689598	0.002329837
CAMK1	-0.134613318	7.149506343	0.002329923
PON2	0.126116778	9.468414505	0.002334883
KRTAP10-12	-0.071191746	5.929974397	0.002336931
APOL6	-0.079840316	6.05671669	0.00234231
KCNIP1	0.056240518	6.05441666	0.002344566
DHX36	0.099832085	7.875535879	0.002344728
LOC652388	-0.164346539	7.0173569	0.002349157
MOSC2	0.218140525	7.679603838	0.002350236
MRPS25	0.188530927	8.670476779	0.002356782
ITIH4	0.086078934	6.169052947	0.002356982
CLDN9	0.059418366	6.299601183	0.002359823
PGBD2	-0.077711126	6.166158169	0.002362419
MYO3B	-0.132785582	6.792968616	0.002366748
DPM2	-0.086401993	6.709184654	0.002367686
XRCC4	-0.120182768	6.100005554	0.002369393
C14ORF80	-0.092128312	6.735757294	0.002375866
GNAI3	-0.139889016	7.027204841	0.002376659
SPTLC1	-0.202216664	9.224591762	0.002380706
MGC72080	0.174140467	7.449244862	0.002382245
LOC728358	-0.054878715	6.016363259	0.002387757
RNF122	-0.165831887	6.482904199	0.002398516
SP140	-0.053973247	6.04621291	0.002398706
SPATA20	0.276695074	9.693153586	0.002401915
SELI	-0.125839425	6.893426669	0.002402918
GNAT1	-0.062891369	6.385516667	0.002404731
SPHK2	-0.135045888	8.492047625	0.002410265
DJ341D10.1	-0.123147163	6.891818465	0.002411285
JMY	0.091534306	6.441918448	0.002412148
MAL2	-0.111346897	6.151691563	0.002414342
GPI	0.174667643	10.7950864	0.002418661
ZDHHC12	-0.123109124	6.640803325	0.0024187
ANGPT2	-0.375772831	7.248215019	0.002418871
MTMR3	0.101173257	7.430549563	0.002421317
VBP1	0.121891003	10.31810621	0.002434951
SIAH1	0.095501741	8.354428645	0.002445021
RPL18	-0.14693086	13.26648256	0.002458912
AADACL1	-0.198834249	8.785461451	0.002462019
IKBKB	-0.111600268	6.790428061	0.002470679
ZW10	-0.130969257	8.442831583	0.002475312
C14ORF32	-0.168494858	8.915863575	0.002478668
TTC25	0.089479317	6.425240851	0.002482396
XRCC6BP1	0.228393906	7.26536036	0.002488952

RNASE3	-0.095350809	5.899140765	0.002489847
ISYNA1	-0.141004739	7.267134618	0.00249111
GLT8D1	0.10434923	6.732719854	0.002497365
RAB11A	-0.1492313	10.63432165	0.002499095
HIPK1	-0.041329973	6.005076052	0.002499704
ZNF720	0.088728226	6.209174376	0.002507967
UNC5B	0.104271226	6.577246929	0.002510548
RUSC1	-0.139736801	9.102765534	0.002510619
BRSK1	0.184756726	7.548731987	0.002517322
TGFB2	0.176108896	6.473699774	0.00251811
TBC1D20	0.097935743	7.452359711	0.002519473
LOC730820	0.130548647	7.358174432	0.002521307
CHM	0.105472189	7.458485921	0.002523472
TYRO3	-0.128455809	6.625489827	0.002524229
LGI2	-0.166359191	6.461699482	0.002524347
ORAI1	-0.12889065	6.654969938	0.002525917
NQO2	-0.320547605	7.902594859	0.002544303
CDH15	-0.078320719	6.16549724	0.002547468
ZNF276	-0.094154777	6.215298079	0.002564265
KIAA1641	0.065064595	6.304659608	0.002571655
APOBEC3C	0.090738019	6.511706995	0.002573139
GBP1	-0.253858687	7.110096732	0.002580466
DPH3	-0.155757589	7.38672736	0.002586845
LOC441061	-0.139898685	6.372276143	0.002590097
MRPL21	0.16661537	9.76893259	0.002591733
FLJ36644	0.078158211	6.33503857	0.002601197
NFKB1	-0.132962379	10.24118514	0.002621042
RMND5B	0.107562337	7.518661916	0.002621474
TCEB2	0.122833898	11.18178861	0.002633047
AP3B1	-0.13869103	9.575649034	0.002636172
DOK3	-0.076224571	5.979370455	0.002655018
TRIM3	-0.061429972	6.433051109	0.002656947
TRIM61	0.106502625	6.534917401	0.002658222
SMC2	0.149646294	7.192364498	0.002665705
ZNF394	-0.19409561	9.541138428	0.002667027
PAPOLA	-0.221831075	10.04553443	0.002667033
LOC731007	0.162120558	7.351547477	0.002682581
BAG4	0.104040983	6.519851508	0.002687217
LOC643396	-0.10353278	6.286771286	0.002691707
LOC643872	0.075903501	6.068739822	0.00269588
LOC645688	-0.172735725	9.926965202	0.002704106
LRP4	-0.126667412	6.699425996	0.002704671
H2AFY2	0.142342469	9.418453599	0.002706584
FRK	0.135166322	6.558307119	0.00271179
PRICKLE4	0.15391021	10.37753443	0.002715488
LOC647195	-0.088521869	6.322117219	0.002722833
LOC146517	-0.156887401	8.240737393	0.002724453
LOC158301	-0.13423204	6.926568109	0.002726813
PBXIP1	0.104493292	6.592182433	0.002727534
SLC25A45	0.054273265	6.157847557	0.002727542
SEC11A	-0.123571307	10.57409532	0.002727885

TMEM33	-0.112234409	6.67734043	0.00273835
TDRD1	-0.172826936	8.472225739	0.002743573
ACTR10	0.135598976	9.591149371	0.002744706
EXOSC4	-0.170945443	7.626771158	0.002745549
RTCD1	0.154358664	8.914604007	0.002750365
TTC30B	0.088097185	6.199793475	0.002751465
FZD3	0.093387558	6.479624976	0.00275148
LOC731002	0.09746223	6.138317331	0.002752903
ZNF589	0.087295109	6.557911657	0.002754876
ELOVL1	-0.154985779	6.81207164	0.002755937
MRPL19	0.230672046	7.684770438	0.002766968
GARNL1	0.109105614	8.110635216	0.002770897
C20ORF52	0.175951734	11.87790923	0.00277395
LOC646276	-0.100227095	6.196996001	0.00278541
GNG7	0.236280836	9.117210692	0.002786262
ITK	-0.095320629	6.414314972	0.002790533
USP14	-0.101356538	7.756637514	0.002794201
C1ORF144	-0.145536906	9.774479974	0.002809715
POLR2B	0.173073846	8.815592703	0.002818668
TSFM	0.146259999	7.622761281	0.002831517
CYFIP2	0.175417979	8.01096162	0.00283373
FLJ14107	-0.078472907	5.996833736	0.002833859
FAM119B	-0.100334193	6.865620443	0.002836001
FADS2	-0.106449583	6.145959429	0.002836099
LOC731835	0.080745063	6.445932013	0.002839516
FAM49B	-0.16584234	6.867014519	0.002847145
LOC650831	-0.063246035	5.999041555	0.002847886
PLS1	0.07151498	6.376746167	0.002854943
ZNF558	0.091826109	6.944278352	0.002859398
DPY19L3	-0.092404728	6.158985452	0.002859648
NONO	-0.110487787	9.698434772	0.002861103
CYC1	-0.137453407	12.89398424	0.002861982
LOC649801	0.213728084	6.504803513	0.002865023
FRS3	0.175268409	8.195190739	0.002866976
LOC652881	0.129542047	6.481861163	0.002867156
DKC1	-0.16614772	9.394540377	0.002868221
LOC732371	-0.089710778	6.160075786	0.00287181
GNA12	-0.092907211	7.254674098	0.002871958
CORO2A	-0.051273127	6.047674284	0.002876383
OCIAD1	-0.148254635	10.93922512	0.002886199
DENR	-0.132988616	9.160864756	0.002890553
ZNF217	-0.125322549	7.049371009	0.002900235
SPRED1	-0.209320113	9.063046992	0.002906343
KIAA0753	0.098893871	6.477804037	0.002910158
SBDSP	0.205762932	9.078292421	0.002912465
MGC39584	-0.068250943	5.910997746	0.002913918
SPAG1	0.051954154	6.323374259	0.002926993
LPAR1	-0.168342297	7.43441823	0.002937147
KIAA1671	0.152115249	8.100607257	0.00293817
CSAD	0.119305887	7.179111206	0.002939663
ZEB2	0.152093587	8.284571672	0.002945072

SCNN1D	-0.136974142	6.960399385	0.002948524
LARS2	0.117892177	7.395121226	0.002951281
CD84	-0.085799281	6.212122553	0.002952928
LOC387762	-0.096488012	6.196611709	0.002957162
UQCRC2	0.198853211	10.57680076	0.002958907
HOXB5	-0.085511128	6.276281593	0.002961518
TMED4	0.167569078	9.242799422	0.002963107
FAM98A	0.167973856	10.06726074	0.002966067
DDX11	-0.043571152	6.159601644	0.002968196
APM-1	0.110204639	6.462877653	0.002969468
ELOVL2	-0.113777248	6.354669559	0.002971183
TEX264	-0.13537983	7.615168731	0.002971629
PREP	0.119545553	8.390002967	0.002980555
GPD1	-0.093967608	6.156761079	0.002983027
PABPC1	-0.168018408	9.705870328	0.002984173
DAPK1	-0.105705641	6.384081748	0.002987262
PMS2L3	0.101797627	6.425012371	0.002988972
GK	-0.110282524	6.991325132	0.002999996
ATAD3B	-0.08200412	6.230139299	0.003003482
PIGH	-0.155134394	7.3333066	0.003006801
CHL1	-0.064639312	6.119652514	0.003025325
MDH1	0.126525477	14.10641055	0.003026297
SSH2	-0.121355404	8.587544411	0.003026603
LOC729759	0.071784587	6.107964452	0.003026802
SIGLEC7	-0.052020274	6.15938269	0.003028278
ZFAND2A	-0.242349803	8.097319415	0.003046948
SDHA	0.231425962	11.66482596	0.003047222
ZNF596	0.055511746	6.044520299	0.00305012
RHOD	-0.192383611	8.594592941	0.003050847
SCGB1D2	-0.19290694	6.372988818	0.003051752
LOC642859	-0.056180183	6.046604531	0.00307026
RNF144	-0.166860177	8.501399028	0.003076307
PEX11G	0.144502276	6.272092637	0.003078075
IKZF5	0.132920245	6.977098455	0.003080398
DYM	-0.101531207	8.879502634	0.003083439
PPM1B	0.108246143	7.878335413	0.003086368
LOC730051	0.16049175	7.441682951	0.003098992
CREB3L4	0.084046183	6.080881261	0.003110014
HDHD2	0.232615581	8.289914441	0.003114104
TUG1	0.134210001	10.87371845	0.003121551
TNFSF8	-0.078969183	6.194591736	0.003122128
FAM10A4	0.123727776	8.51616727	0.003123893
CNTN4	0.054312502	6.102750231	0.003128167
FAM168A	-0.073523706	6.076946936	0.00313229
HPS5	0.093684797	7.168462193	0.003144819
INMT	0.247615573	8.979512486	0.003145232
TCF7L2	0.097642601	6.384304088	0.003152795
C18ORF26	0.086462408	6.228209043	0.003156504
LOC728888	-0.162547898	10.82952253	0.003166148
ABCG5	0.088003704	6.60139095	0.003171149
GLI3	-0.085029861	6.449401384	0.003171313

LOC152195	0.091377302	6.48406254	0.003172088
KCNQ3	-0.100372864	6.661849001	0.003181117
CCDC47	-0.112860029	6.473132714	0.003182916
HIVEP2	-0.120791846	7.664699956	0.003187283
CALN1	-0.073746598	6.870448735	0.003187499
BTNL2	-0.083701619	6.270085237	0.003188198
ZNFX1	-0.157589878	8.500388718	0.003191312
NAV1	0.225701925	8.467772555	0.003204362
C10ORF41	-0.080569013	6.278566224	0.003206142
SENP2	0.103766509	7.998709409	0.003207839
XYLT1	-0.159122268	6.244607603	0.003210902
COL4A3	-0.084785519	6.379934086	0.003214284
SLC1A4	-0.140273544	6.892457806	0.003215982
CACNA2D1	0.064265855	6.160824382	0.003227034
HDHD1A	0.163661921	8.590828918	0.003229463
SPIN2B	0.064270184	6.275241413	0.003235804
C1ORF58	0.086862583	6.159552141	0.003238227
MIB1	0.084152283	6.487747169	0.003253894
GIYD1	-0.101114561	6.862093059	0.003255559
PUM2	0.128709199	9.283871073	0.003264404
COMMD9	0.134147131	8.028963885	0.00326734
ALS2CR12	0.117894096	6.069180495	0.003268868
LOC440731	-0.086566924	6.632665485	0.003289596
LOC493754	0.101663219	6.620405542	0.003289599
PHF11	-0.118622765	9.133018888	0.00329237
WTAP	-0.089049292	7.304108616	0.003293898
ECHS1	0.171055954	11.4190208	0.003299174
ECHDC1	0.183583104	7.080489385	0.003307545
ABCA1	0.243220896	9.011111864	0.003307663
SLC26A6	-0.086382869	6.693422301	0.003308912
RHAG	-0.068335426	6.070060941	0.003309531
UGP2	-0.174326995	10.0713678	0.00331613
MGC4677	-0.344684277	8.421226258	0.00331768
TIE1	-0.119777999	7.146211079	0.003318187
MAP1LC3A	-0.171203332	9.495450136	0.003320884
ELP3	0.0923223	9.003731603	0.003326152
LOC644137	-0.070799012	6.002708062	0.003331484
ARID1A	-0.132597395	8.577034668	0.003351053
KIAA1715	-0.114867434	7.332367865	0.003355823
RABGEF1	0.088902975	7.120937563	0.003358148
LOC441191	0.103061871	6.550374952	0.003369841
AIFM2	0.108679348	6.673172289	0.003370316
TNNC1	0.148154285	14.49325852	0.003376363
CCDC23	0.168292428	9.349708558	0.003378034
DISC1	-0.041330304	6.077034705	0.003378702
ZNF593	-0.212553537	8.829667803	0.003381276
NRK	0.125861382	6.713092465	0.003382774
ACTG2	-0.446862615	7.715219919	0.003384714
MEOX1	0.196053544	7.658718783	0.003394081
WRNIP1	0.157251001	9.01988966	0.003396587
POT1	0.070468236	6.310728385	0.003405463

COL22A1	0.130552208	6.224525902	0.003408502
CDCA7L	0.203270108	7.199979458	0.0034096
RNPS1	-0.139578313	9.007108032	0.003414611
LOC732075	0.108452867	6.525872672	0.003420983
FAM21C	0.052139722	6.350304856	0.003441861
TNS1	-0.166228306	8.523400072	0.003449124
CCDC48	0.149882356	6.181130851	0.003452724
CXORF34	-0.088355524	6.757815718	0.003458655
LOC728723	0.078274618	6.126232263	0.003464441
GPR68	-0.155255749	6.352787624	0.003474149
CDKN1C	-0.192194554	7.923822424	0.003478801
RABAC1	-0.208529915	9.347175599	0.003482488
VAMP3	0.143457633	9.939593249	0.003510556
BUD31	0.194752267	9.529759943	0.00351343
RSAD1	0.151198276	7.636168968	0.003520746
GPKOW	-0.143247314	7.652527738	0.003525244
MT1JP	-0.071505008	6.176918759	0.00352525
FLJ30092	-0.050998015	6.157849397	0.003529865
LRRC6	0.120384323	6.323718783	0.003544779
ZNF57	-0.142346633	6.623414084	0.00354507
LOC645241	0.073079212	6.143351678	0.003545434
DNAJC9	0.10756826	8.59467147	0.003569515
HCN2	0.073602225	6.167898711	0.003580979
ERBB2IP	0.061696946	6.413434495	0.00358503
KIAA1333	0.086703998	6.233114517	0.003585219
ANGPTL5	0.172989417	6.54804785	0.003586326
RPL36A	-0.098748061	6.372412661	0.003587677
AFG3L1	-0.046753075	6.376313988	0.003587817
SEPT7	0.2807981	7.978412929	0.003590391
NHLRC2	-0.132655603	7.260306836	0.003601823
C3ORF26	-0.151366882	8.706447358	0.003608919
CABLES2	-0.132788129	6.950924766	0.003609994
RNF19A	0.092761524	7.708502356	0.003612813
PHKG2	-0.111347411	6.758900789	0.003618497
CRYAB	-0.151184099	14.51487924	0.003620143
KIF17	-0.079935761	6.416535094	0.003621622
RAVER1	-0.10440197	7.143744243	0.003623827
LOC652242	0.072219039	5.95385333	0.003624763
CCR7	-0.085491762	6.088613504	0.003625702
SCAMP5	0.091297731	6.544087445	0.00363515
C13ORF7	0.172799239	7.159093765	0.003636964
SYN2	-0.081641281	6.279669148	0.003638461
LOC646588	0.093949857	6.180536223	0.003642793
TMEM14A	0.147197886	7.796112213	0.003652802
SEC16A	-0.128414856	8.77794923	0.003655221
IQCK	0.104614352	7.866864564	0.003655308
MYST1	0.12058539	8.628691598	0.003655492
MFSD2	-0.081080977	5.995709286	0.003658258
ITGA7	-0.147911929	7.478823399	0.003664668
ZC3H4	0.122687568	8.737308554	0.003673208
ZBTB38	0.16480653	6.811611493	0.003678356

SPAG8	0.080980943	6.181990302	0.003681702
PKD1L1	0.065783985	6.401485469	0.003702618
TOX4	0.136719845	7.493214426	0.00370981
DTNBP1	-0.075253672	6.815702551	0.003720507
RUNX2	0.055721263	6.086401203	0.003722413
CDC42SE2	-0.120115547	7.279269677	0.00372274
CYTL1	0.133773422	6.122287172	0.003722744
CTH	0.058111102	6.262062324	0.003726584
STK39	-0.191108074	9.579341152	0.00373258
CD58	-0.145431068	8.486290477	0.003742762
SLC16A6	-0.074802187	6.086559846	0.003744461
MICALL1	-0.102517442	6.484867502	0.003759928
NARG1	-0.121826246	7.409013897	0.003760022
TSLP	0.056622477	6.226836295	0.003763747
FMO2	0.273367335	9.135452085	0.003776109
ATL2	-0.111355391	7.247633682	0.003791816
SLC9A5	0.109363829	6.078818495	0.003795671
PTGIS	-0.27803032	7.731812404	0.003819024
B3GALT4	0.11615167	7.070661453	0.00382267
LOC642443	0.170496029	6.46114585	0.003824252
TST	-0.163942447	11.28076178	0.003834432
DGUOK	-0.105813623	9.012823828	0.003837311
C6ORF142	0.119971578	10.47694771	0.00384018
KIAA1881	-0.298382782	10.20720379	0.0038457
PTP4A1	-0.142374165	6.669199231	0.003848656
FARSA	-0.122550206	7.385866927	0.003850593
CSGALNACT2	-0.120772995	6.076825477	0.003852806
MGC2752	-0.093551695	6.571912649	0.00386036
SMOX	-0.054009637	6.356450079	0.003864582
FGFR4	-0.046225285	6.064729552	0.00387634
SCRG1	0.285287124	7.238908292	0.003876745
LOC647450	-0.176962142	6.461927204	0.003883248
PLEKHO2	-0.155694613	6.791597097	0.003885738
LOC642393	-0.073575673	6.366575472	0.003904947
NEUROG2	0.077505023	6.253805996	0.003910107
EXTL2	0.095803472	7.349946462	0.003920382
PCTK3	0.164956925	7.883934287	0.003922717
LOC644511	-0.180831341	10.76981712	0.00393662
LOC651285	-0.067478098	6.142042737	0.003937704
PPTC7	-0.231896953	8.854997987	0.003946152
KPNB1	-0.162559861	11.07290136	0.003959313
LOC401218	-0.10143831	7.348646993	0.003961337
LOC647677	0.058136193	5.948913323	0.003967595
KIAA0652	0.100235793	6.340305075	0.00398415
LCP2	-0.069253748	6.225901215	0.003988913
XG	0.083689306	6.031691875	0.003990857
RMND5A	0.100241334	6.390933802	0.003995981
ATXN2	0.103305046	10.26332679	0.004000555
DPP4	0.14006287	6.632437749	0.004039232
NR3C1	0.065668137	6.612858025	0.004048558
LOC642423	0.080596638	6.264644221	0.0040689

C16ORF13	0.110399241	7.841009249	0.004070617
DNAJC2	0.097759952	6.215147601	0.004074219
LOC641700	-0.154323832	7.035775425	0.004076615
CDAN1	-0.179230136	10.57915008	0.004083659
BLZF1	-0.239525905	10.48943445	0.004092494
TBX1	-0.035182635	6.043253833	0.004102055
MAPKAP1	-0.109170093	7.823938854	0.004114818
NR4A1	-0.06603258	6.063874202	0.0041219
ANKRD44	-0.128680178	6.455647466	0.004128291
C21ORF81	0.127190777	6.40383005	0.004131554
SYTL4	-0.106711853	6.819852941	0.004135135
GNS	-0.132322469	12.21746454	0.004136788
RBKS	-0.112459068	7.238466197	0.004137414
CRMP1	-0.067747696	6.445686351	0.004142166
STARD5	-0.077038833	6.194824165	0.004152261
FLT1	-0.106880607	6.06392364	0.00415696
FLJ20699	0.144372889	8.189529096	0.004177802
USP25	0.134559086	6.864827443	0.004178256
LAMC3	0.144270187	6.409991413	0.004178267
HSPB3	-0.183328917	12.65227834	0.004179419
ZNF614	0.09536701	7.607432616	0.004195311
SELK	-0.158039604	9.627290369	0.004201485
CYP4V2	0.302026847	8.906498386	0.004211424
NRGN	-0.139392882	6.407345854	0.004212086
ACBD3	0.140480233	9.023765328	0.004223859
LOC644670	-0.083477266	6.403624477	0.00423202
PRKAR1A	0.190061189	11.09179949	0.004240371
DEDD	-0.082792977	7.381156434	0.004255249
TBP	0.087813763	7.918093556	0.004273628
RG9MTD3	-0.065207466	6.408360161	0.004273628
ZC3H7A	0.096858586	8.293719946	0.004278393
LOC650128	-0.140192474	7.133636073	0.004280427
LOC646609	-0.113825358	6.228247921	0.00428071
ADPRHL2	-0.189156117	7.232521188	0.004282369
C9	0.065534915	5.974878902	0.004299397
ZWILCH	-0.073574511	6.51950801	0.004300365
C20ORF72	0.138502147	8.76067596	0.004305936
TSPAN5	0.160814008	6.913882826	0.004306116
RUNDC1	-0.109827366	7.966351272	0.00430678
RAB37	0.039632658	6.197802097	0.004326589
JMJD2A	-0.101093944	6.154024777	0.004329087
CNTROB	0.081562948	6.441511759	0.004329584
HPDL	-0.078299696	6.093923239	0.004330036
MGC3731	-0.142582984	9.150049537	0.0043415
FLJ44112	0.076634666	5.97766166	0.004343316
KIAA0746	-0.123637453	6.904804394	0.004354261
RFWD3	-0.10863604	7.174416621	0.004362097
PPARA	0.087353848	6.899966499	0.004368519
TRIM25	-0.118859706	7.142931028	0.004371835
SLC22A15	0.129455148	7.204998554	0.004372676
FAM110A	-0.057883637	6.183037318	0.004373634

WRN	-0.097571425	7.432232354	0.004375126
DPYSL3	0.267546491	10.63508454	0.00437676
LOC651337	0.088532552	6.154126697	0.00437769
NADSYN1	-0.111574329	7.449006604	0.004387326
GPATCH2	0.095614912	6.792992929	0.004388139
CENPQ	0.127294572	6.613486219	0.004389584
CST6	-0.064666044	6.062903209	0.004400595
PLEKHM2	-0.151907983	10.5581464	0.004403191
HNRPK	-0.151136575	9.176362673	0.004404338
NUDT4P1	-0.073492182	6.006841279	0.00440435
GORASP1	0.127558329	8.482095665	0.00440983
TMED2	-0.206968272	9.255702968	0.004410915
RNF111	0.109608227	6.214210108	0.004414543
NCOA5	-0.121687693	7.707080409	0.004418167
USP16	0.106849936	8.722082313	0.004418566
FGF13	0.053230915	6.080022898	0.004429039
STX7	0.12181389	7.253159158	0.004433515
NUDT12	0.083302626	6.179233571	0.004434043
MAP7D3	0.109715736	6.685119723	0.004434792
SUMO1P3	0.170891018	7.689214525	0.004437804
BAPX1	0.091999102	6.05890517	0.004438227
C1ORF156	0.098925842	6.198818674	0.004438282
CTAGE5	0.051896007	6.195835838	0.004439127
PSMA5	-0.133820767	10.60181806	0.00443987
NAGLU	-0.134290936	7.00259955	0.00444126
CRYGS	0.100763239	6.858560647	0.004445719
C6ORF89	-0.12934371	7.21028192	0.004447945
TMPRSS13	-0.076769958	6.075442895	0.00445211
SERPINF2	-0.077978823	6.105908835	0.004464433
ACAT2	-0.117297903	6.953012023	0.004465088
NEU4	-0.089263976	6.029355516	0.0044661
CROCC	0.088920728	6.849112807	0.004485351
C5ORF28	-0.219910104	10.20664086	0.004497869
DNASE1L3	0.173457034	6.548024155	0.004500732
LOC651872	0.088391425	6.372284299	0.004509774
TFEB	0.088890484	6.519773018	0.004510859
ALDOC	0.267213692	12.10328602	0.004511815
C13ORF25	-0.087316768	6.282866501	0.004518982
NOC4L	-0.048312875	6.546651303	0.004522739
FOXO1	-0.108058793	8.748751878	0.004529733
CSDA	-0.173767088	13.33794771	0.004537966
LOC653438	-0.114337132	7.753253423	0.004539551
LOC643310	0.145226123	9.803134529	0.004540916
LOC730272	-0.070244083	5.973720178	0.004544523
ARRDC4	-0.183841676	6.876414099	0.004548105
TRAPPC6A	0.14714295	6.912697603	0.004567292
DEFB122	0.066843433	6.060467309	0.004573003
ARFGEF1	0.100515375	7.767500728	0.004599019
FAM104A	-0.144924112	8.749111838	0.004599542
OSBPL1A	0.152653554	8.910818378	0.004608381
MFSD8	0.090001503	7.079786498	0.004613323

TNRC6B	0.123885465	7.733733104	0.004613951
CAPG	-0.083251311	6.297746017	0.004620328
LOC283050	-0.080686468	6.004357648	0.004626065
DGKI	0.088875171	6.114542261	0.004629018
C20ORF59	-0.083684764	6.037396875	0.004633262
HDAC8	-0.094433937	6.676541544	0.004633393
LOC648852	-0.263514164	9.258000493	0.004636665
LOC153364	0.091149515	6.600023124	0.004647303
RFX2	-0.090202124	6.232697287	0.004651587
PLIN	-0.154779522	6.101367037	0.004655518
CYB5R2	-0.081485753	6.293600523	0.004656019
OSBP	0.111207183	10.29995658	0.004656926
LOC388654	-0.244420547	11.77668985	0.004659692
KCNG1	-0.058108692	6.191306663	0.004663284
LRRC40	0.085452391	6.598305463	0.004663768
CCL7	-0.091648217	5.953507247	0.004665692
OPHN1	-0.081835253	6.139768657	0.004666668
PRPS1	-0.111630712	8.842668368	0.004667762
LMO4	-0.176457023	9.584212733	0.004676895
LOXL3	-0.098280058	6.507924703	0.004679451
ROCK2	-0.139250678	12.95387209	0.004685669
ZNF250	0.083418767	6.7769164	0.004692849
NGFRAP1	0.240677789	9.999735358	0.004694481
LOC645013	-0.075648613	6.037270041	0.004703232
ERP29	-0.114062314	9.873979583	0.00470741
EEF1D	-0.14601844	9.942891887	0.00471177
ING2	-0.112314755	7.161077239	0.004714439
HEMK1	0.076249377	6.498995129	0.00472622
DMD	0.071893657	6.714477521	0.00472921
EPB41L1	0.062789596	6.266904252	0.004735617
CSNK1A1	-0.129747851	7.948119653	0.004737617
UFD1L	0.069682339	6.740575416	0.004742296
NFX1	0.137569869	8.196161937	0.004745106
LOC441763	0.383699225	10.55800299	0.004746477
LOC642477	0.101649103	6.300370891	0.00476816
TOP3B	0.137104868	8.030581909	0.004768411
RPS21	0.146933878	9.764979345	0.00477243
LOC652489	0.142033095	8.00667968	0.004773074
GRIK2	-0.050896665	6.123681856	0.004807306
PPP1R12A	0.138781601	7.293140684	0.004807681
C4ORF34	0.119777462	8.795231049	0.004812206
C22ORF28	-0.10683173	9.428788415	0.004814532
FLJ10996	0.100974122	6.238267952	0.004816053
ASB10	0.29764267	10.20499535	0.0048202
SAP130	0.090570251	8.135588647	0.004820572
AFAP1L2	0.097487116	7.00154365	0.004822799
RRP7A	-0.21599182	8.669467089	0.004831648
PRRT1	0.083767104	6.404513219	0.004833355
DNAJC28	0.051788826	6.186799432	0.004835585
TM2D2	-0.114966496	7.335778161	0.004840559
PPP1R15B	-0.081768143	6.19885227	0.0048518

PDCD2	-0.131476757	8.474346332	0.004853298
LOC649509	0.062404635	6.032827592	0.004853302
ARMC1	0.140714223	8.736128428	0.004856685
RMND1	0.09123781	7.153254073	0.004864394
OLFML1	0.176999887	7.41181126	0.004876634
TMTC4	-0.058370701	6.427277162	0.004880912
CSN1S2A	0.070545987	6.087116073	0.004887852
SMPX	0.205894575	11.72019454	0.004890315
C6ORF72	0.189868216	7.638930238	0.004895208
CDC34	-0.172209626	8.552737803	0.004900293
ZNF768	-0.179201983	6.477881633	0.004902731
ACTR2	-0.152933737	10.3027904	0.004907393
MRPL34	0.230103982	10.35781882	0.004908978
CXORF26	-0.145152328	8.625567736	0.004918196
IPPK	-0.094087914	6.66369819	0.004923701
RELL2	-0.072140023	6.05415887	0.004925595
UBE4A	0.174143895	8.887979317	0.00492945
APTX	0.08590521	7.714778918	0.004930643
FAM116A	0.181163226	8.167260637	0.0049312
AKR1B10	-0.079026378	5.963756615	0.004936501
LMTK3	0.142563332	6.716103266	0.004943326
LOC646109	-0.066334274	6.050332996	0.004946042
MON2	0.081836799	7.730962852	0.004962738
VTI1B	0.169751178	8.175689145	0.004967599
DPYD	-0.145272328	6.777347273	0.00496812
TAPT1	0.109444534	7.669741065	0.004970711
WDR77	0.114303571	7.583877464	0.004980113
SNORD38A	-0.091826762	6.112395433	0.004982928
RNASEH2A	-0.103489408	6.619241507	0.004998502
FIGN	0.081059216	6.371086805	0.00500646
RPS27	-0.124481582	13.38782046	0.005010635
KCNMB1	0.329236667	8.083733047	0.005010719
KIF3C	0.097241903	6.227601297	0.005018688
FBXO6	-0.12410646	7.568898542	0.005021717
EPM2AIP1	0.20121002	9.213097438	0.005043741
AOF2	-0.102474559	8.564957329	0.005063501
CLEC12A	-0.061699222	6.161415027	0.00508395
ADAM22	0.04484164	5.97159861	0.005098713
EIF4B	0.142541695	11.52793966	0.005116091
HNRNPD	-0.137198732	11.30353297	0.005119498
TRAF3IP1	0.075536568	7.082567295	0.005133578
CD82	-0.095459783	7.08094554	0.005138762
CLCNKB	0.082581612	6.317483098	0.005140719
LOC652264	-0.153849582	7.160932569	0.005153778
NSFL1C	0.131190103	9.105762465	0.005154282
ATP13A2	-0.0992254	6.628270395	0.00515584
ZNF501	0.078796807	6.116033648	0.005165351
SSX4B	0.053577508	6.124466327	0.005178167
ITM2C	0.147209781	8.374067442	0.005193091
LOC730358	0.127774322	7.02848686	0.005200534
UBQLN2	0.183507649	10.10252333	0.005229886

IFT74	0.176729002	7.802050434	0.005233544
HNRNPH2	-0.158771459	6.837798073	0.00523981
C7ORF47	-0.132583377	7.945451768	0.005250353
LOC642816	0.043576077	6.03025885	0.005251559
LOC650037	-0.082395623	6.077155191	0.00525446
KLHL2	-0.154999987	7.128003604	0.005263319
RBM14	-0.139856525	9.415349216	0.0052726
C8ORF32	0.162241632	8.4753033	0.005272739
TAF1B	-0.106761939	6.70828049	0.005274671
KATNAL1	0.117471033	7.683422516	0.005279843
TF	-0.17961139	6.26642047	0.00528101
NES	0.316822072	10.30710528	0.005288331
HBZ	-0.074861608	6.102461422	0.005297685
BOC	0.09044602	6.203867007	0.00530016
SLC25A17	-0.087548009	6.498686846	0.005303424
CSPG4LYP1	0.06629858	5.993143648	0.005320052
OBFC1	0.142097012	9.331879522	0.005330419
CLIC4	-0.188488843	10.19175155	0.005337327
RAD54B	0.05385686	6.15654144	0.005348507
ELMOD2	0.113709449	7.028093089	0.005359532
RNF5P1	-0.173618132	8.97880171	0.005363171
COL5A1	-0.299852164	8.732012927	0.005365454
RNF19B	-0.116086788	6.642620814	0.005391744
PSMC3	-0.095685912	10.70814464	0.005413741
GOLGB1	0.238636078	8.617503877	0.005416923
C11ORF63	-0.082892514	6.499195727	0.005419213
TMEM194	0.08264479	6.483219236	0.005425369
C1ORF96	0.070465774	6.168986295	0.005445329
AKR1B1	-0.177825634	11.22060472	0.005473124
ANKRD24	0.080372923	6.19098824	0.005479921
PDE4C	-0.185820202	11.91050717	0.005505109
P4HA1	0.160538549	7.946581928	0.005518594
WBSCR17	0.105564136	6.784337249	0.005527346
SEC22A	-0.090455999	7.961527497	0.005533517
CHX10	-0.073756459	6.130424598	0.005537451
LOC644029	-0.136875938	11.66523911	0.005543646
PGAM5	-0.07823602	6.986878894	0.00554971
SNORD43	-0.065911856	5.947899967	0.005550339
IMMP2L	0.12674432	7.893748926	0.005555861
MCF2L2	0.05858143	6.242880759	0.005559688
GAMT	0.17583637	9.359070138	0.005561539
MYH3	-0.081816275	6.237209612	0.005589073
PGM3	-0.130715128	6.944431893	0.005589537
WDR18	-0.219677802	9.209110841	0.005589959
C3ORF55	-0.072700696	6.007757186	0.00560152
LOC388002	0.072044513	6.138298798	0.00560362
TXNRD1	-0.262400166	8.900556294	0.005609204
C9ORF3	0.161196481	8.984288025	0.005611306
N-PAC	-0.146354995	8.447088992	0.005613616
FLJ45337	0.082841728	6.655193856	0.00561627
DAB2IP	0.062123563	6.35871643	0.005634871

ZNF479	0.076852997	5.983341683	0.005638077
LDHD	0.189725337	7.859095392	0.005642414
POMP	-0.154997505	11.18038606	0.005645131
TMEM143	0.216938052	9.000172507	0.005652861
LRRC58	-0.114372584	6.859287794	0.005661344
OR1M1	-0.064983865	6.027735921	0.0056654
HOXA5	-0.083063878	6.011987151	0.005667371
YIPF7	0.08490933	6.237734979	0.005673383
RELA	-0.097440275	6.966404454	0.005675587
PTPN2	-0.070188438	6.351329095	0.005679349
RASA1	0.093950467	7.868081405	0.005702994
ARHGAP21	0.14241228	10.26512464	0.005704541
FAM116B	-0.116282524	7.057381106	0.005705612
LOC407835	-0.118643325	8.08024013	0.005731048
LOC653119	-0.099540078	6.222969735	0.005731134
C6	-0.673928539	9.401276545	0.005745495
ENTPD4	-0.143922775	7.395507934	0.005763231
RFESD	0.086451271	7.050931462	0.005774944
CHIC1	0.049395957	6.22534011	0.005775437
TAS2R9	0.063460242	6.085648814	0.005778922
PLEKHA1	-0.13914943	9.552391209	0.005783791
DZIP3	0.075053993	6.39754524	0.005785672
SNRPE	0.056642188	6.175939274	0.005792725
PITRM1	0.125723152	9.232324331	0.005795388
PALM2-AKAP2	0.05199083	6.023817887	0.005795448
USP7	-0.18117677	7.536565883	0.005797137
CCDC109A	-0.102880203	7.134789916	0.005800123
HSBP1	-0.15127981	10.09430729	0.005804001
TNCRNA	-0.118266252	6.504332421	0.005805353
CLOCK	0.14399167	7.645835584	0.005827151
C17ORF81	0.062986794	7.180676839	0.005829551
ALDH3A2	0.132640268	9.285157224	0.005829993
SLC2A9	-0.048837885	6.198198482	0.005831117
UNC13B	0.113584726	6.870825141	0.00583148
SLC35B1	-0.134829078	10.36939353	0.00583421
LOC387841	-0.113592613	12.58743659	0.005836159
NOC2L	-0.117071249	6.569885059	0.005857754
SNF1LK2	-0.088548044	6.672692524	0.005859002
CD48	-0.117862348	6.340081875	0.005866569
IGF2BP3	0.123802146	6.536350811	0.005876817
TTC38	0.194499725	7.840668506	0.00587762
DUSP18	-0.100613593	6.69660249	0.005879378
NFATC1	0.096435883	6.403046096	0.005880783
FANCI	-0.089153821	6.219834737	0.005883424
HOXB4	-0.076084709	6.135265904	0.005892901
PXDN	-0.317082963	7.884261925	0.005893404
ZNF213	-0.092686445	7.202364666	0.005898925
FBXW2	-0.114345388	7.467214724	0.005903185
GPR126	-0.076009584	6.126193264	0.005904398
SP2	0.105881525	7.656227173	0.005907399
NF2	-0.047469874	6.16190718	0.0059075

HLA-A	-0.166688445	11.94086231	0.005918336
LOC285033	-0.051116651	6.235695109	0.00592306
C21ORF123	0.066256347	5.997915673	0.005948161
C16ORF68	-0.175306811	9.19598051	0.00595508
GIMAP6	-0.139360586	7.926981425	0.005973413
TOM1L2	0.124649434	7.476306066	0.005980251
GATA5	0.178769219	6.445770101	0.005987947
KCNT1	-0.238617553	7.138746759	0.005991608
LOC653291	0.075927644	6.05961794	0.005993689
LOC729446	0.095785405	7.589376502	0.006008203
LOC650251	0.146272255	6.804113392	0.006014223
LOC285216	-0.076178447	6.132394904	0.006017768
LYL1	-0.154025347	8.794128765	0.006020707
MRPS12	-0.134503436	9.011671832	0.006033943
TIAM1	-0.077463854	6.169528934	0.006034763
KCNF1	0.090769063	6.169973376	0.006038455
EXT2	-0.105537828	7.673068906	0.006043794
SEC61A2	0.094372352	6.892366271	0.006058081
RTF1	0.082039985	6.631266302	0.006072925
NME2	-0.077514143	6.765738382	0.006080038
RNF7	0.148969145	10.07560872	0.006083281
SPIRE2	-0.078641655	6.157346388	0.006099327
LOC653907	-0.065798476	6.062357019	0.006101558
DEFB107A	0.071288757	6.362575314	0.006114519
ZNF26	0.089518957	7.101214647	0.006121961
ARRDC5	-0.076990847	5.967133609	0.006144738
LOC647570	0.076188431	6.431218935	0.006147879
TRIM28	-0.11109302	7.929873716	0.006151193
KLF10	-0.078873289	6.476878604	0.006165183
RCN2	0.164717606	8.508496243	0.00617697
ZFP36L1	-0.264175946	9.076742294	0.006180758
ZDHHC14	0.219427765	7.970209291	0.006192374
KRT8	-0.164486111	7.799983226	0.006195028
PRKRIR	0.142909017	9.583951531	0.006195727
PRND	0.12658402	6.132460311	0.006198376
NEK3	0.064597801	6.490281821	0.006208149
SPINK1	-0.288569241	6.326552355	0.006211503
FAM73A	-0.219272535	8.224114415	0.006213687
SLC25A46	0.137017604	9.258227742	0.006214017
BUB1	0.094037531	6.124200798	0.006214928
CDR2	-0.148550395	7.635591728	0.006224965
LOC653147	0.132773913	8.832664374	0.006251931
DDX51	-0.228810724	10.73903539	0.006259967
SESTD1	0.133621249	7.708629035	0.006285534
ZCCHC14	0.126450921	8.311970147	0.006287014
ODC1	0.213672269	9.965846598	0.006292201
TTMA	0.049147879	6.034872848	0.006307629
GPR89C	-0.081710048	6.740958113	0.006324677
PHLPP	0.088372039	6.474579131	0.006326097
HSD17B4	0.145313878	10.29922915	0.006327285
RPUSD4	0.154062296	10.80923776	0.006330967

C21ORF55	-0.170571502	12.79676785	0.006337277
LOC643356	-0.062686453	6.013726005	0.00634967
FLJ11292	0.119958003	6.767255226	0.00636136
COMMD4	0.090991822	8.203438554	0.006392317
STXBP1	-0.070507915	6.757270944	0.006399811
TMIE	0.06927086	6.086574671	0.006432817
TRPC6	-0.082514612	6.46576417	0.006444979
XKR8	0.059747287	6.859672715	0.006451297
GDF9	0.071560653	6.205956783	0.006463173
PPM1E	0.227958965	6.796688975	0.00646623
MYO7A	-0.090174982	6.492690004	0.006466363
NPPC	-0.108588914	6.216528897	0.006471746
LOC728006	0.117068771	8.710551957	0.006482651
PPARD	-0.058497786	6.45471808	0.006489688
NUP35	-0.090274859	6.731924899	0.006519548
NPPA	0.931967862	12.41831102	0.006521991
C1ORF159	-0.072555387	6.522581136	0.006526337
KRTCAP3	0.111305858	6.509731702	0.006543775
LTV1	-0.163728375	8.078029107	0.006544332
FGF11	-0.102769086	6.482554997	0.006563628
BAD	-0.092957521	7.412907747	0.006566546
SNURF	0.205971175	10.3864453	0.006566833
ELMO3	-0.069544136	6.167854155	0.0065766
PRLH	0.06934526	6.030509726	0.00657831
CREB3L3	0.062417672	5.923075152	0.006583391
ZNF225	0.069084718	6.477779044	0.006592247
CALHM2	-0.125847168	6.735438331	0.006599193
UBXN4	0.133769347	10.16943757	0.006609466
SETD4	0.081647139	6.890506559	0.006610696
ZNF775	0.083691649	6.61332567	0.00661529
NANOS3	0.099841037	6.975657844	0.006617842
FUT6	-0.078689301	6.495256424	0.006621394
MGC27345	-0.092294581	6.482305722	0.00662497
CAMTA1	0.119098876	6.821540124	0.006625982
NUDCD1	-0.082923001	6.22458794	0.006651289
EBAG9	0.12020597	8.620693865	0.006657516
C3ORF39	0.116699891	7.840390383	0.00665813
RILP	0.108482607	7.185060908	0.006660131
C18ORF56	0.080877766	6.194209165	0.006665923
ZNF384	0.083767654	6.613993581	0.006668848
CD200R2	0.071896928	5.939387389	0.006669277
ZNF69	-0.203548762	10.17035207	0.006669423
CDC14B	0.109154586	7.75415815	0.006674149
STRN4	-0.080872401	6.851614152	0.006686658
WWC3	0.130357677	8.400803492	0.006699296
CORO2B	-0.06542476	6.416256385	0.006705551
S100A2	0.088359703	6.842713272	0.006716817
FHOD1	0.131629424	7.884984162	0.006732713
CLSTN1	0.124228147	8.615004942	0.006737282
PDCL3	0.096620975	8.766319803	0.006743649
RHBDL2	-0.199058899	7.633803408	0.006745605

LOC642277	-0.067172409	6.046619264	0.00675991
LOC646667	0.066219657	6.09194865	0.006783826
MTMR11	0.108464062	7.088940654	0.006810651
LOC388282	0.047122965	6.227453448	0.00682602
TMEM128	0.145890396	6.648992907	0.006829808
OR2A9P	-0.063348502	6.227385748	0.006834873
FEM1A	-0.15356453	10.57450741	0.006835039
PLGLA	0.054917631	6.125840991	0.006844279
NISCH	0.11732385	9.268223388	0.006845367
FLJ40473	0.074214602	6.168039551	0.006854025
DGKA	-0.055103225	6.111289115	0.006866443
RUNDC2C	-0.112933328	7.118635075	0.006870305
TNFSF15	-0.119590749	7.184453939	0.006889115
TSPO	-0.10740519	10.91027938	0.006921552
GRIP2	0.066460613	6.212396621	0.006929631
C21ORF62	-0.115082515	5.991444883	0.006935071
FKSG24	-0.102001345	6.703643465	0.006938053
CXORF36	-0.064169868	6.093781332	0.006959952
MGC15763	0.11148715	7.054713997	0.006964937
ACSS1	0.259686915	8.978803599	0.006972932
MTMR12	0.11665517	6.959066245	0.006983764
ZNF184	0.083556574	6.200722567	0.006995388
NDUFS3	0.123664771	12.46848923	0.007005045
SCPEP1	0.183659196	9.433363658	0.00701367
CD302	-0.113787606	6.994710361	0.007022861
KCNJ12	-0.160929551	6.624732712	0.00703322
GPR89B	-0.103209471	7.029728175	0.00704757
IGSF3	-0.191630778	7.637411083	0.007057185
CHCHD3	0.140769231	9.458225655	0.007062285
RBM45	0.118543098	7.331411924	0.007092195
MTR	0.184684657	8.277056148	0.007093014
STXBP5	0.102439211	7.400664735	0.007099076
ANGEL2	0.103222255	8.865152059	0.007099088
C3ORF36	-0.068543471	6.226140841	0.00710213
LOC727909	0.062989325	6.037945796	0.007106729
TXNDC9	0.18539111	8.103433853	0.007131292
KCNMB2	0.048826295	6.215794627	0.007144027
C1ORF71	-0.152372226	9.107413068	0.007146489
TM2D3	-0.084263362	9.175266665	0.007157456
LOC644961	-0.069827494	5.961130743	0.00716172
C8ORF44	0.075715191	6.393953174	0.007183256
DSCR5	0.121224623	6.187340463	0.007189383
MARCH3	-0.240644942	9.263816176	0.007193958
NAB2	0.12884416	6.566585989	0.007194562
C15ORF23	0.11273291	6.69587718	0.007197371
PPEF1	-0.09748983	6.113577073	0.007200011
ISL1	0.063758941	6.095180065	0.00722503
LOC646019	-0.064874918	6.048308139	0.007229347
MCEE	0.18191939	10.11790372	0.007235306
DKFZP451M2119	0.090010471	6.369782367	0.007242997
LOC728492	0.171385898	10.39624438	0.007254097

TRAF3IP2	-0.104697485	7.491359655	0.007257808
FIBCD1	-0.113431576	6.508372031	0.007271533
LOC641693	0.069011279	6.152761792	0.0072729
LOC652773	0.193366518	7.921152134	0.007279798
FANCC	-0.077871086	6.164425782	0.007291338
LOC644935	0.101143819	8.073450115	0.007297276
AADAT	0.041097346	6.233873961	0.007311869
ORC6L	-0.135246887	13.03882178	0.007312934
C4ORF39	0.080221677	6.290328941	0.007319596
C17ORF49	-0.116856372	8.714649272	0.007323396
ZC3H8	0.171024038	6.761347344	0.007327546
WRB	0.14580217	8.342941156	0.007331306
TAF8	0.090216053	6.249604932	0.007336159
NCAN	0.081366575	6.758798626	0.007336875
TRIM27	-0.08616717	6.917418891	0.007345801
PDCD10	0.128493828	7.718213901	0.007347946
LOC644537	0.063711032	6.041746885	0.007353876
TIMP3	-0.227253242	9.613905764	0.007354179
MAGMAS	-0.122272773	10.18605154	0.007356561
B2M	-0.162282772	12.39179885	0.007357415
RAD51C	0.100817641	7.927978083	0.007360501
RQCD1	0.187816192	8.909532994	0.007373334
FGFR1OP2	0.146646736	6.935360543	0.007373367
CTSA	-0.136407568	8.502327261	0.007398596
ZNF264	-0.114827384	6.833687333	0.007400711
LOC391771	0.061791884	6.120641852	0.007405085
ST6GALNAC1	0.107789713	6.383835026	0.0074465
RRN3	0.207327939	7.047465699	0.00746068
SYP	-0.072047271	6.083341698	0.007463334
C22ORF32	0.112461722	7.350836778	0.00749138
APOB	-0.226046169	7.122542252	0.007499875
LOC646119	-0.060218569	5.959581548	0.00751062
FBXO34	0.108584432	8.199376909	0.007515789
NIP30	0.098763482	7.414294498	0.007516731
EZH2	-0.045955738	6.107132975	0.007523373
ARL6	0.043045356	6.0605854	0.007524371
LOC399900	-0.178206874	12.69185407	0.007548857
SH3YL1	0.119564265	7.140620927	0.00756964
PMCH	0.069243781	5.955375296	0.007580582
MPEG1	-0.067951566	6.300530635	0.007582899
KLF13	-0.167657191	8.98830188	0.007594101
GRPEL2	0.124646663	8.608651933	0.007600592
C3ORF30	-0.047013032	6.212768145	0.007623011
C5ORF42	0.05515666	6.251929203	0.00762989
ATAD2	0.162640797	7.225631301	0.007631664
RBM4	-0.094505851	7.250920938	0.007665611
ARL3	0.105965715	6.906557074	0.007665811
C14ORF94	0.160518938	7.377249363	0.007667941
MRPL14	-0.138316574	10.00168529	0.007675323
LOC648706	0.082469957	6.152673508	0.007678632
ZNF322A	0.083656623	6.443749711	0.00768145

UBL5	0.166032547	9.713648824	0.007700453
SERPINF1	0.232301268	8.722721735	0.007711662
C1QTNF6	-0.062596017	6.165938963	0.007717629
USP3	-0.108694768	8.121346833	0.007720807
TMEM66	0.112737363	12.10564152	0.007723654
LOC641896	-0.065658766	6.117949229	0.007727473
MYLIP	0.183646876	8.525982468	0.007738957
DOT1L	-0.083576565	6.259629391	0.007739249
PHF14	0.070466783	7.036824526	0.007741727
SEL1L	-0.088256465	6.7523988	0.00776119
ARHGAP26	-0.084544447	6.495512491	0.007780128
RPUSD2	0.111712641	8.947876164	0.007803041
LOC439957	-0.070480609	5.964167748	0.007804378
TMEM136	0.128004667	8.769030674	0.007805064
TIMM17B	0.112049977	8.393532872	0.00781014
CNOT10	0.086627858	7.263442372	0.00781136
C16ORF86	0.102330126	6.761144737	0.007820861
SSX5	0.061781186	6.119702443	0.007835185
LOC646317	-0.070057971	6.236981851	0.007844852
PRPS2	-0.077968013	6.647813228	0.007845884
CENPJ	0.092809628	6.883304112	0.007855246
ACTR1A	-0.107553909	9.923731068	0.007860907
VAPB	0.209874777	6.538837112	0.007876889
TMTC2	-0.072076386	6.25111798	0.007921254
XPOT	-0.110644978	6.457588486	0.007940639
LPCAT2	-0.090515923	6.358790207	0.007954536
ACOX3	-0.094981289	8.525643119	0.007958927
CRTAC1	0.166497771	6.455463066	0.007965672
KCNH2	-0.109014717	7.415077191	0.007965694
ORMDL2	-0.112784584	6.20756173	0.007977577
KIF9	0.042392967	6.051950996	0.007979426
UTP11L	-0.17286528	8.932307612	0.007989392
IWS1	-0.109168402	8.18916916	0.007996181
C6ORF120	-0.103862522	6.708532928	0.008001558
LOC388181	0.076098851	6.083592242	0.008005919
ADH1C	-0.163865536	6.492224321	0.008009768
FLRT3	0.059404483	6.087404391	0.008011027
TRIM21	-0.113698715	6.827144788	0.008014217
MRPS34	-0.156624278	6.93748119	0.008027436
C9ORF116	0.067422129	6.397373415	0.008028101
WDR61	0.108061358	9.40378042	0.008031721
CCR5	-0.056306492	6.056870273	0.008039016
C6ORF222	0.089749542	6.599974927	0.008048934
ZNF227	0.098505735	6.663508516	0.008049628
C19ORF25	0.087027629	6.951878759	0.008055823
PIK3AP1	-0.128640616	6.328980871	0.008088852
HIST1H2BC	0.097858736	6.331867024	0.008117756
MAPK8IP1	0.158410912	6.455986156	0.008124573
TRNAU1AP	0.05598141	6.545515862	0.008130099
BCAS3	0.076023662	6.310262943	0.008132
REEP5	0.120942393	11.57257247	0.008132418

APBA2BP	0.099468702	6.259208274	0.008135323
KLRB1	0.107023614	6.388458105	0.008143564
ERCC3	0.105696442	7.863723366	0.008145762
LOC652634	-0.1686003	7.406330266	0.008150749
SFXN5	0.100244905	6.783243034	0.008165222
BRMS1L	0.113231462	7.347515476	0.008185841
PITPNM2	-0.076954447	6.517804664	0.008193189
LOC654260	-0.093183279	6.806939153	0.008212662
GPR137B	0.151441063	8.541643687	0.00823946
IFIT5	0.118723521	6.774141442	0.008243589
ZNF154	0.081670889	6.887922545	0.008246703
RIF1	0.091037362	6.844207977	0.008248133
LOC728844	-0.081634905	6.06171284	0.008248314
LOC648695	0.131452166	7.74330824	0.008248336
HDAC4	0.191030596	7.902649646	0.008248803
DYNLT3	0.158130459	8.019955094	0.008259955
MDM4	0.097808851	6.446724614	0.00827803
DHX30	0.089219966	8.029165876	0.008278991
P2RY8	-0.127384848	6.631269763	0.008279961
BAIAP2	-0.053837711	6.424192093	0.008283195
P2RY14	0.058284556	6.122288933	0.008305566
AP2A2	-0.110583593	7.054573275	0.008308281
LOC144983	0.050492246	6.204762989	0.00836289
ZNF608	0.103752145	6.560737463	0.008367551
STARD3NL	-0.110643775	7.489535664	0.008376558
ZNF555	0.079300409	6.318656297	0.008386802
TFPT	0.097821779	7.75740546	0.008395191
NDUFV3	0.108530538	6.994957791	0.008396117
PIP5K2A	-0.063699271	6.17082369	0.008416907
TTLL5	0.08527476	7.449173414	0.008426221
SKIV2L2	0.108406273	8.71255749	0.008431734
PHF20L1	0.077380464	7.353763554	0.008448519
NT5C1A	0.091596932	6.196633094	0.008449352
CKB	-0.240075842	10.15887467	0.008461591
SAA2	-0.073006759	6.031187568	0.00847453
LCAT	0.103410411	7.029105444	0.008479608
PRAM1	-0.080579547	6.137327953	0.008483509
NCOA1	-0.093279158	8.143036243	0.008494283
HISPPD2A	0.064007821	6.521207743	0.008501131
JAM2	0.203734408	8.8456884	0.008503643
AGPAT2	0.131188545	8.053607722	0.008531544
F12	-0.066156639	6.192322312	0.008544189
ACIN1	0.112165134	8.390264314	0.008547417
NOL11	-0.13860772	9.039085607	0.008552277
MMS19L	0.092785316	9.542028824	0.008556263
INPP5B	-0.092232755	6.899703759	0.008557625
DAG1	-0.116487383	9.036419831	0.008571307
ANKRD9	-0.175429517	11.05046745	0.008583454
LOC642678	-0.131693184	6.632076887	0.0085959
FAHD2A	0.129051201	7.400211219	0.008600362
MAPKAPK5	-0.087481097	7.878559857	0.008601816

ZNF519	0.046735527	6.138766029	0.008625224
OPN1MW	-0.050555784	6.023492173	0.008629253
SUMO2	0.149196464	9.460136402	0.008643338
C21ORF56	-0.125998226	6.627705686	0.008651326
PCMT1	0.140291723	12.1941609	0.00865525
C19ORF47	-0.070597979	6.262444615	0.008658964
CKS1B	-0.135853326	8.393394141	0.008663011
LOC649555	-0.194249287	8.127369467	0.00868641
TXNL4A	-0.07543272	6.322165676	0.008695476
EWSR1	-0.081533871	9.392755655	0.008699083
KIAA1704	0.064790363	6.238095668	0.008703547
LEP	-0.186505672	7.4894739	0.008711336
DEF8	0.082113956	8.114925904	0.008751582
PPFIBP1	-0.168907354	8.428014009	0.008767116
ZDHHC24	-0.07678401	6.588804982	0.008782006
CEP170	0.043786849	6.191809002	0.008792614
RAE1	-0.094862141	7.531693074	0.008810691
ATOH8	-0.165260557	7.536131377	0.008827872
SNAPIN	0.185672288	7.177407941	0.008839941
MKX	0.103982775	6.061064282	0.008852383
SLC7A6OS	0.111645578	6.726510025	0.008859273
CNNM3	0.106666841	7.324235905	0.00887041
OR4P4	-0.069932855	6.188198367	0.008892101
PDE6C	-0.050117337	5.931595957	0.008906075
CDK5RAP3	0.121986644	8.43796464	0.008907975
ABCC4	-0.105758669	6.543693343	0.008912859
PIGM	0.128458095	7.115978581	0.00891813
PPM2C	0.22094983	7.848329319	0.008921839
DYNLL1	0.19416524	10.49676207	0.00893225
LOC654042	0.047235817	6.246312718	0.008933808
FBXO28	0.096416504	7.907308986	0.008935495
PHF8	0.055846281	6.059085842	0.008969296
GGA2	-0.113306268	7.921319523	0.00897927
MMP16	-0.044843149	5.956570773	0.008979534
TASP1	0.084361875	6.710623863	0.00898565
LOC647543	-0.277635564	7.400358493	0.009004329
ARRB2	-0.06833066	6.265558826	0.009004596
LOC643242	0.064056811	5.979463057	0.009011742
ELFN2	0.06825784	6.047410818	0.00901207
KIAA0241	-0.07343254	6.423817257	0.009018381
SUCLG1	0.129501666	11.70564814	0.009022217
OR2Y1	-0.065528503	5.949086155	0.009045819
ELN	-0.126248408	6.412342247	0.009052011
NARS2	0.141818865	7.894869317	0.009054903
AGPAT3	-0.06296587	6.184495487	0.009060711
LBH	-0.136921545	8.280393903	0.009061558
C6ORF204	-0.171936841	6.798327657	0.009063327
TRIM55	-0.242771427	9.235514451	0.009086078
RAB3GAP1	0.120131677	11.06637981	0.009086169
CPSF2	0.082913517	7.748934817	0.009089845
PHC3	-0.055153784	6.306281119	0.009095119

LOC643668	0.180343601	9.42818178	0.009098586
LRMP	0.083296401	6.374380045	0.009101833
SELL	-0.085509639	6.173185849	0.009122089
ANKRD28	-0.080968771	7.189118849	0.009128017
C10ORF4	0.036356157	6.235599312	0.009129419
NIPA1	-0.120912092	7.343984915	0.009138571
SIP1	0.108639976	7.557588831	0.009154131
C20ORF45	-0.145169671	8.289745179	0.009167072
USP33	0.105961	6.795708819	0.009177093
OR56B4	-0.066313975	6.433117155	0.00920925
EIF3G	-0.12043102	10.83855342	0.00921297
SELENBP1	-0.229737326	7.76594679	0.009242287
TMEM60	0.10499986	8.960944511	0.009251641
LOC442060	0.057830811	5.97240906	0.009253051
BCL7C	0.096394684	7.851234396	0.009263415
LOC647766	-0.069081727	6.132040817	0.00926483
TP53AP1	0.07903797	6.695683072	0.009266897
SCGB1D1	0.078023105	6.409595331	0.009279418
VWA5A	-0.074778494	6.267424554	0.009280032
MRAS	0.180473058	8.660278944	0.009299567
SP5	-0.079045451	6.408666317	0.009302982
RNASEH2B	0.231093987	8.091490345	0.009304896
FLJ23152	0.07002449	6.490470877	0.009314343
C10ORF47	-0.056165882	6.14542763	0.009331566
GIPC1	0.09759048	8.513368684	0.009348517
THUMPD1	0.116008885	7.75580569	0.009352442
PDIA2	0.079718212	6.314261388	0.009353334
ABCD3	-0.127493553	7.66472534	0.0093725
RBAK	0.075909606	6.456831066	0.009380714
DDX1	0.137051056	11.38291274	0.009384321
LOC651546	0.056256183	6.033665986	0.009407596
PARN	0.11040227	7.936199352	0.009422617
TSPAN31	0.095746359	9.429462184	0.009446156
LOC728944	0.1221052	7.718973941	0.009451831
KCND2	-0.062230998	5.974352015	0.009462844
LOC389257	0.176620677	6.782826339	0.009463888
EBP	-0.199154628	7.783272139	0.009472129
LOC96597	0.06780537	6.004286298	0.009472971
ACTR1B	0.093516542	8.979728349	0.009482957
EIF3J	-0.129247546	8.024251596	0.009488257
PTGIR	-0.075547669	6.265237186	0.009490704
KY	0.087062251	6.948856447	0.009513412
ALDH2	0.160164569	10.69770966	0.009514843
TTTY5	0.048974244	6.130757266	0.009516842
LOC145837	0.064873715	6.147526219	0.009529243
GKAP1	0.175817925	7.495604716	0.009545177
BTBD15	0.147277955	7.201138158	0.009565296
RBBP8	-0.079626286	6.857160839	0.009572482
AES	0.111144561	10.20750679	0.009609747
LOC644336	-0.072859902	5.985894962	0.009613774
UTP15	-0.089439188	6.775978303	0.009615234

KLHL22	0.144397611	8.932385067	0.009616947
CCDC3	0.193023923	7.329323085	0.009618509
FLJ46358	0.068716188	6.105520208	0.009641075
SLC20A2	-0.15145848	9.295303679	0.009652347
PNPT1	-0.146053467	11.66212351	0.009673559
ST3GAL4	-0.189642776	7.657285663	0.009681124
RANBP6	-0.173048562	7.141079254	0.009681233
SLC25A40	0.103082578	6.776958222	0.009685717
C1QTNF9	0.11669063	6.303668437	0.009686168
LOC642372	-0.071780475	5.957345069	0.009699122
HDAC1	-0.079845211	10.15271582	0.009713728
CPEB2	0.106636919	7.039338256	0.009720511
FLOT1	-0.109691494	10.17046344	0.009727764
XKR6	0.054847692	6.31630653	0.009729452
ZNF364	0.190083791	10.96865912	0.009746802
DERPC	-0.07014111	6.235458486	0.009768532
TRA1P2	-0.206121026	7.353335624	0.009776075
DDX54	0.174927047	7.89339996	0.009777906
SCN4B	0.177559706	6.935994987	0.009780834
LRRC59	-0.088643845	6.272644948	0.009792789
TMOD4	0.070185888	6.421423452	0.009796683
ICOS	0.067819892	6.2664882	0.009802881
YWHAB	-0.151067701	10.71273091	0.009824164
PCDH19	0.084412956	6.519253644	0.009838911
DNAJC5G	0.082762829	6.323388061	0.009839229
C22ORF36	0.095521646	6.66706784	0.009839489
LOC648399	0.132120188	6.660689191	0.00984034
NR0B2	0.121884772	6.378960439	0.009855413
MID1	0.137205818	8.661144707	0.009857011
AFF1	-0.107356605	7.493750246	0.009860863
LOC650369	-0.217947353	8.969990908	0.009875908
PSMA2	0.103002237	8.299520867	0.009884598
FBN3	0.058746521	6.158929003	0.009901707
KLRG2	-0.076135728	6.332527311	0.009905158
TLE3	-0.085548406	6.343895386	0.00990861
SSRP1	-0.101245861	6.688527748	0.00991744
C6ORF150	-0.065549694	5.928058063	0.0099268
PIWIL4	0.053357571	6.227399323	0.009935422
GTF2IRD2P	0.09247472	6.694007029	0.009943604
WNK1	-0.119412698	9.511894509	0.009945396
SPHAR	0.201577198	7.700244654	0.009949308
SHOX2	0.050004988	6.369905168	0.009960667
LOC643431	-0.100553142	6.282564084	0.009971082
MPP4	-0.058028065	5.918758078	0.009983999
HS3ST3A1	-0.211333947	6.875159879	0.009987746
LOC402057	-0.10898122	12.01403283	0.009995051
ADAM8	-0.068671378	6.092398658	0.010002273
WWP1	0.143656502	10.02602233	0.010008963
LOC653182	-0.057929751	6.079563081	0.010010022
LOC90586	-0.223307894	9.749864808	0.010035026
DEFA3	-0.074215329	6.012280682	0.010042117

HRCT1	-0.137276152	6.482426776	0.01008108
MFSD3	-0.153821568	9.360583412	0.010119378
SLC28A2	0.058544335	5.974896229	0.010121472
RPL13L	-0.140683032	7.595310321	0.010126527
GPBP1	0.089002124	8.334150218	0.010142001
NAV2	-0.147176086	8.029467879	0.010155298
TRIM74	0.079172725	6.226061947	0.01016171
EIF4H	-0.126932048	10.15834668	0.010168591
EXOC3	-0.116438982	8.103443589	0.010179502
DDX31	-0.054632829	6.481234292	0.010191391
CLCN2	0.067766711	6.293441179	0.010195712
SFRS15	0.126742768	7.729706297	0.010201105
ACP5	-0.068873907	5.963165457	0.010212815
LYSMD2	-0.204184148	8.295646088	0.01021579
NXF2B	-0.045130351	6.073930875	0.010216651
PPIG	0.115274521	7.146663027	0.010223499
PGA4	-0.070539507	5.917999925	0.010265497
C19ORF50	-0.125916037	8.092581902	0.010296011
BZW2	0.110365152	11.77345239	0.010304452
MOXD1	0.198861515	7.29755384	0.010334158
PARP4	-0.124689996	9.304073275	0.010342776
PDZRN4	0.050296086	6.005530816	0.010356092
ANKRD11	-0.101022817	8.594683245	0.0103589
ADAMTS13	0.048509814	6.121428649	0.010367659
DNAI2	-0.114393858	7.093678794	0.010375713
REL	-0.058331306	6.108929597	0.010382557
C22ORF39	0.125935706	8.357253511	0.0103867
CADM3	-0.064668123	6.154778326	0.010390548
MAP2K2	-0.149774578	9.597129498	0.010414101
PA2G4	-0.178940991	8.517236201	0.010422914
CHD1L	-0.093245065	6.92426383	0.01046447
ATP5F1	0.127822397	12.03975657	0.010464802
FAM49A	-0.067332279	6.317386314	0.010483767
NARS	0.170057539	10.91942509	0.010502299
FBXO5	-0.068683579	6.282589421	0.010509649
ZHX2	0.171697146	8.803123902	0.01051244
LOC644538	0.096878451	6.174805727	0.010517439
APPL2	-0.115869946	8.147832932	0.01054458
HIGD1B	0.164788641	8.385549215	0.01056103
AFTPH	0.074412059	6.590733011	0.010563186
RIPK5	0.181407357	7.595010448	0.010598475
NOVA1	0.055311415	6.179459792	0.010608573
DACT1	0.070536048	6.345537577	0.01061011
CLCNKA	0.170597049	7.696728437	0.010632523
KIAA1571	0.081251459	6.210948677	0.010638183
PABPC5	0.042945034	6.036183563	0.010690545
FTH1	0.191318543	10.43814552	0.01069586
SPOP	-0.125289751	9.59681891	0.010698635
LGALS3BP	-0.226934273	9.533062469	0.010700994
PARK2	0.101320372	6.752386742	0.010705338
CDC14A	0.03141318	6.129919866	0.010706883

LOC23117	-0.179271967	8.195237577	0.010711209
PPP2R1A	0.121993775	11.59248502	0.010716403
GNB2L1	-0.150398606	13.33337586	0.010736277
ARSD	0.077688688	6.906486109	0.010742576
LOC644251	0.133372363	6.416854993	0.010742972
EXOSC1	0.084238437	8.343426417	0.010749569
FLII	-0.132803505	9.284850152	0.010749739
PRKCI	-0.098117839	6.953888637	0.010751401
LMO2	0.168933923	7.702727976	0.010766683
NF1	0.079557845	6.563852402	0.010774211
LOC652493	-0.177602318	6.594792526	0.01078236
SLC44A4	-0.157504593	11.08404569	0.010806339
C16ORF53	0.115444409	7.677140986	0.010815055
YES1	-0.197659199	8.480931347	0.010816462
GNAI1	0.075456459	6.515765026	0.01082837
FLRT2	0.111126592	6.592098294	0.010828457
NKIRAS2	0.09627804	7.078792448	0.010851936
TMEM110	-0.069539662	6.235241356	0.010852661
PRKAB1	-0.081733943	7.29850206	0.010871527
PRTFDC1	0.148501891	6.860047612	0.010872989
RBCK1	-0.0844149	8.116166821	0.0108837
CREB3	-0.124271675	6.601213158	0.010930535
TAZ	0.092650832	7.225967154	0.010932936
ULK2	0.136409656	6.753525244	0.010935987
LOC648710	-0.075255316	6.082376148	0.010936655
BRE	0.100254609	8.874664085	0.010968549
PPP1R13B	0.166631586	10.19040176	0.010970382
PLA2G4F	-0.11063751	6.171790609	0.010972462
RND3	0.201707571	9.214318183	0.010978257
PSMA3	-0.131466576	9.132041827	0.010994597
ANKRD10	-0.127638193	8.400612439	0.011034581
PAIP1	0.04709927	6.345250602	0.011037196
LCOR	0.134287991	7.765269549	0.011071038
LRP1B	0.083239905	6.091412673	0.011078389
RHOXF1	0.062892747	6.049957499	0.011078541
FAM100B	0.110259766	7.106510337	0.011088313
GANC	-0.064251835	6.110050904	0.011095174
LOC652479	-0.088970901	6.233183183	0.011096268
DNMT3B	0.032908053	6.258658168	0.011097328
SLC5A7	0.068785124	6.03089785	0.011117284
FN3K	0.073400749	6.465410464	0.011124806
GCC1	-0.099984049	7.75614519	0.011128621
C11ORF82	-0.077120726	6.149999992	0.011132088
SOX9	0.217591644	8.23842884	0.011134834
LOC441179	0.062152214	6.193712911	0.011155384
PRPSAP2	0.08984547	8.040530376	0.011159572
PI3	-0.06902463	6.048537527	0.011171272
ZNF251	0.060803357	6.45098116	0.011173325
WSB1	0.145342609	8.654511179	0.011182766
FRAP1	-0.098592623	8.519187335	0.011199515
MEN1	-0.043248281	6.206680312	0.011205885

ATRIP	-0.072466774	6.969424449	0.011211704
TGFB3	0.19953826	8.34538971	0.011225361
PAPOLG	0.069857396	6.448596264	0.011231918
JAG1	0.156460085	8.274233813	0.011302266
LOC644530	0.05812906	6.180433977	0.011306558
KIF1B	-0.115709152	8.648677459	0.011306803
SORBS3	-0.08061021	7.239847879	0.011314623
EHMT1	-0.040238135	6.203661214	0.011316567
ASPA	0.079292201	6.120716749	0.011325303
NOMO2	-0.122623303	9.357761075	0.01133116
CAST	-0.202155831	9.378134342	0.011334052
TMEM22	-0.103966293	6.865642852	0.011343379
LOC642578	0.055141727	6.045215033	0.011347848
SERBP1	-0.115087558	8.938577331	0.011358022
SAR1B	0.109956868	8.952199401	0.011405091
RPS17	-0.121875868	12.73199923	0.011405483
C16ORF62	0.076512498	6.553654134	0.011438147
PRRT3	0.095617564	6.4313339	0.011474779
COX6C	0.084303059	13.63555876	0.011476067
UBE2I	-0.090726768	8.706547383	0.011488583
BPHL	0.154697395	9.007750777	0.011514981
FLJ32154	-0.060166559	5.953833176	0.01153243
SH3BP1	-0.068607164	6.194382651	0.011543393
B3GNT6	0.155197734	8.384031598	0.011557394
LOC150223	-0.071392634	6.169933109	0.011569345
RPRD1A	0.099813937	8.462222351	0.011597181
LOC493869	0.206925139	7.444683693	0.011597284
ID2	0.195485175	8.807321638	0.011601072
LSM8	0.100767164	7.071003253	0.011607705
PRDM16	0.053664593	6.337684333	0.011627107
ZNF533	0.212430955	6.534987589	0.011659476
PHKA1	0.094416775	7.104204625	0.01168007
PSMA7	-0.065152965	6.252593894	0.011704301
AMDHD1	-0.080787405	6.334581678	0.011710191
TMSB4X	-0.125480658	13.11100532	0.011712087
PHIP	0.108973416	8.601339259	0.011733692
ITPKA	-0.163464112	6.871415084	0.011770793
TWF1	-0.056886996	6.261202456	0.011791182
SFRS1	-0.123060737	10.2589804	0.011800088
TMED10	-0.198198062	9.242811553	0.011816991
RANBP3	-0.059119436	7.487222874	0.011817093
TMCO6	-0.068329624	6.473380463	0.011823783
RPLP2	-0.100311186	14.1528484	0.011836337
LOC642398	0.106550319	6.508000244	0.011846113
NPFFR2	0.0474805	6.052408545	0.011876087
SMAD3	-0.1126427	7.837749298	0.01191328
RNPEP	-0.127303342	7.485402712	0.011927825
NPTX1	-0.036506955	5.947607567	0.011937534
ZNF155	0.064822166	6.224986937	0.011942345
LOC649711	-0.068331218	6.179654506	0.011956424
LOC649209	-0.072508579	6.383016787	0.011979476

PDCD2L	0.100392093	7.489821253	0.011994403
KCNG2	-0.042825025	6.160663489	0.012006823
POLE	0.084076475	6.732398191	0.012045359
LOC644466	0.058705919	5.994106896	0.012052715
GCK	0.046378822	6.086178232	0.012054587
LOC645683	-0.119639583	13.19763761	0.012059511
GAPVD1	-0.115194872	9.415811849	0.012063363
LOC730360	0.06940986	6.201675202	0.012103619
BUD13	0.080742321	7.038532488	0.012118048
PPA2	-0.125689834	10.34278854	0.012118769
CA5B	0.100032759	6.49265196	0.012122084
FBLN5	-0.168091011	8.439070523	0.012143932
LOC641710	0.070735069	6.533847399	0.012151312
DNAJB1	-0.20588337	8.198407292	0.01215312
ETNK2	-0.086346836	6.149207384	0.012156486
C7ORF28A	0.081912976	7.268976124	0.012164134
LOC646342	-0.055812843	5.909974556	0.012176271
SLC35C1	-0.168358929	6.612886141	0.01218018
FLJ20160	-0.113794076	8.074673285	0.012188848
ABO	-0.058455635	6.026852303	0.012188962
MTA3	0.078645109	8.023573395	0.012223142
FCRL1	0.063698117	6.092391621	0.012247064
WASF2	-0.14425738	8.039303686	0.012260752
GYG1	-0.11651433	11.30570652	0.01228466
ADRA2C	-0.043613496	6.155457522	0.012294521
RASGRP1	0.072998214	6.293161412	0.012298933
SIM2	-0.046230424	6.043605725	0.01231717
GPR132	-0.060947269	6.121779617	0.012354878
CCNC	0.107771951	7.550788079	0.012382108
C1ORF210	0.048344989	6.205904008	0.012422025
TICAM2	-0.116902488	7.314651529	0.012430451
GP1BA	0.102003708	7.349276897	0.012431679
ATP8B4	-0.140815728	7.443967951	0.01245747
MSTP9	0.073549588	6.413450814	0.012466918
CCDC59	0.147301289	9.700518323	0.012494275
GTF2A2	0.141229008	10.12771062	0.012501648
AAA1	0.034469311	6.084934627	0.012507958
POLM	-0.085661055	6.900575899	0.012521674
ZC3HAV1	-0.10770398	7.393772357	0.012524014
ASGR1	-0.128118075	6.357696114	0.012528156
DCTN5	-0.169618784	8.282656875	0.01253742
FANCD2	-0.042128209	6.126553361	0.012543285
KIAA1797	0.104023778	7.71225331	0.012543995
ZCCHC8	0.09338964	7.246804761	0.012570047
LOC645600	-0.082082071	6.360982967	0.012571008
DKFZP761P0423	-0.116405046	7.283370754	0.012574062
SAP30L	-0.13916019	8.605782111	0.01257636
ZBTB20	0.149658092	9.820113297	0.012580048
MUSK	-0.09784911	7.119069632	0.012606
BAT5	-0.090343746	8.12698244	0.012613863
LOC648099	-0.108017432	6.851215857	0.012622989

ANKRD43	0.05697341	6.100151751	0.012624737
C1ORF26	0.14884511	6.382615627	0.012630499
BBS5	0.041947867	6.129419955	0.012632611
C9ORF58	-0.140563743	7.279816709	0.012644009
PTPN13	0.073922948	6.445816255	0.012661936
LOC401052	0.08260399	6.890434795	0.012669429
ZNF193	-0.079018808	7.017898943	0.012675513
CTCF	0.11164715	7.920008966	0.012681144
FARSB	-0.063099516	6.221783023	0.012685261
AMDHD2	-0.113781766	6.808524959	0.012702236
OR51B2	-0.058617447	5.986672397	0.012707429
MLX	-0.092329594	7.143149158	0.012717789
S100A1	0.212476152	10.7624373	0.012720803
MAGEA6	0.046473584	6.155806112	0.012722655
MELK	-0.064703095	6.077541997	0.012751843
DOC2B	-0.073162088	6.18473571	0.012793453
FAM44B	0.111626304	9.815762511	0.012806038
TMEM59	0.107081304	12.04746835	0.012815764
PTGFR	0.070554021	6.130526183	0.012817456
ERO1L	-0.140701855	7.41915596	0.012828384
LOC149837	0.068057449	6.319714402	0.012829164
LOC400721	-0.144025933	13.57093597	0.012878335
N4BP1	-0.082787802	6.846272443	0.012935439
TCF12	0.091299348	7.684845262	0.012943315
TGFBRAP1	-0.128487474	6.95292144	0.012964309
LOC652699	0.063445502	6.211576119	0.012968441
LOC646900	0.176890264	7.732001898	0.012969289
PRKCE	-0.090490653	6.854485024	0.012983048
LILRB1	-0.143886014	11.01621548	0.012991034
NEK8	-0.063840936	6.164619955	0.012994606
CBWD6	-0.0555947	5.88860881	0.013010814
EIF5	-0.169958852	7.950086772	0.013020264
GATC	-0.081526563	6.266731762	0.013020583
LOC347376	0.197101727	8.39094983	0.013023427
LOXL4	-0.274053471	7.137477499	0.013033381
PCTP	-0.077569294	6.874078881	0.013034404
LOC730077	0.102262776	6.646112786	0.013039852
CES4	-0.060927875	6.115558784	0.013058936
LOC642660	0.061536066	6.054357899	0.013070261
METTL3	0.0895233	8.109768785	0.013073751
FLJ32252	-0.157242789	6.305645277	0.013094108
C17ORF48	0.094934756	6.803543797	0.013102809
SLC35E1	-0.228329671	9.126599125	0.013128817
SHC4	0.088663872	6.57774609	0.013149674
EMX1	-0.043526415	5.995013608	0.013154356
C10ORF39	0.084592418	6.601454102	0.01321486
CAPN7	0.081305891	7.221456598	0.013218178
HSD17B11	-0.116185737	7.277727788	0.013219652
MDM2	-0.031035333	6.011798434	0.013226173
EPS15	0.095717445	7.850676874	0.013229001
RPL27A	-0.119066203	13.18449758	0.013233613

ZNF429	0.072791467	6.081550153	0.013238801
ZNF607	0.042596877	6.252484112	0.013245325
ABHD3	-0.075754496	6.652456609	0.013251455
LOC648494	0.13286364	6.91012883	0.013281077
SYT4	-0.07005871	5.963349269	0.013287973
CSTB	0.130514068	10.98421001	0.013295498
CENPE	-0.045916398	6.136238365	0.013295593
LIMK2	-0.03417583	6.19180727	0.013308085
SULT1A2	0.093208411	6.483704976	0.013326088
APOOL	-0.09071306	6.785337193	0.013371981
RGS7BP	0.083038602	6.334212292	0.013385726
DAD1	-0.141153707	11.07266838	0.01339265
RAB33A	0.145343846	6.515976227	0.013400977
HNT	0.093891034	6.414254995	0.013402131
SEC11C	-0.186596211	8.196331574	0.013439818
C8ORF31	-0.061575111	6.10912226	0.013443299
C16ORF90	-0.047403219	6.016334644	0.013465492
LOC653895	0.074055651	6.195226671	0.013466073
C1ORF103	-0.047876783	6.014757892	0.013470775
CMAS	0.09698144	9.38033557	0.013518966
FER	-0.076511855	6.665413478	0.013526106
DENND5B	-0.174406949	8.871734551	0.013534302
PSMD14	-0.128040243	9.645892935	0.013535418
ATP5A1	0.124923802	14.20401372	0.013576835
FASTKD3	0.081445065	6.829571683	0.013579338
LOC650706	0.066351582	5.997191886	0.01358365
AHCTF1	-0.069682594	7.487608915	0.013629952
ASB1	-0.201043248	9.075144027	0.01366572
ZNF695	-0.055272487	5.966885984	0.013673267
RNF25	0.081373684	6.563879586	0.013692294
LOC653214	-0.059645862	5.981299748	0.013735897
ASF1B	-0.064878644	6.085881837	0.013746245
FCHO1	-0.066343231	6.083431695	0.013768363
ARG1	-0.065945726	5.999155505	0.013795922
PCYT2	0.113406554	7.580773916	0.013806251
C1ORF176	-0.170362436	8.607499205	0.013809783
CDC27	0.063914705	6.21207366	0.013820783
CRAT	0.092134636	7.110667035	0.013820929
CFLAR	-0.136883871	10.12451451	0.013822582
MYCBPAP	0.077715907	6.397147223	0.013826498
PIGV	0.08190604	7.266451723	0.013894292
DCDC5	-0.067912469	6.426847227	0.013913649
LSM1	0.128386475	11.5094549	0.01392849
BNIP3L	0.112141604	7.950293149	0.013940562
LOC389672	-0.153462317	7.289745911	0.01395997
LOC387934	-0.282860152	7.826620612	0.014007823
PSCA	-0.058675627	6.134091626	0.014040334
METTL10	0.047580552	6.281565446	0.014094246
CLK2	0.053692696	7.136253622	0.014102221
PPAN-P2RY11	-0.077073518	6.295546673	0.014103968
PNPLA2	0.169133091	9.245527445	0.014110475

FRMD6	0.095989207	7.130965366	0.014141836
C1ORF152	0.063678556	6.337264656	0.014147484
L3MBTL	0.03942598	6.10467292	0.014156935
RAC1	-0.091576894	9.988043173	0.014157578
THSD1	0.048814747	6.242076544	0.014158081
SDK1	-0.050431074	6.120593892	0.014175705
GALNTL1	-0.227407119	9.437799287	0.014186184
LOC642771	-0.077681354	6.436730283	0.014189458
ITGA11	-0.11073687	6.660245125	0.014206791
C4ORF32	0.155648204	7.353858091	0.014248782
UBE2F	-0.140297336	10.70762849	0.014256465
LOC642787	0.066337889	6.383244992	0.0143171
C7ORF55	0.174382641	9.403640624	0.014318525
KIF1C	0.223923557	8.672346005	0.014337672
ZNF713	0.046036475	6.397875519	0.014338181
C19ORF48	-0.060526902	6.714990297	0.014341401
KIAA0323	-0.10351671	7.040905175	0.014341602
CACNA2D3	-0.089560937	6.329930448	0.014350473
FAHD1	-0.143928579	7.96448312	0.014372564
SLC35A4	-0.10660674	8.815045108	0.014387589
ANKRD54	0.096341387	7.44123476	0.014389965
TOR2A	-0.057686835	6.045840029	0.014422074
TLR1	-0.099351221	6.30543417	0.014431613
RNF208	0.059157247	6.241625755	0.014440097
ZNF347	0.062142666	6.359239776	0.014457668
BSG	0.19995124	11.23279044	0.014474701
PRSS27	-0.059653305	6.105841176	0.014475307
ORAI2	-0.059553235	6.080744006	0.014483948
GCAT	-0.173882162	8.899440623	0.01449817
RHOBTB2	-0.107680411	7.114216578	0.014508784
LOC440132	-0.068650253	6.065411104	0.014511866
MDN1	0.082362645	6.53898326	0.014524404
LRRC55	-0.064975989	6.010431892	0.01453076
LOC645466	0.160933758	7.739540469	0.014543595
ANKH	0.076974465	6.186069633	0.014549922
ZNF294	0.089319364	7.559965274	0.014564147
IL2RG	-0.058861486	5.96509359	0.014580228
GUF1	0.114065485	7.28641214	0.014594577
ZNF202	-0.07813002	6.278519675	0.014599747
DLEU1	-0.09824756	7.323382874	0.014614897
RANBP2L1	0.074465578	6.181077706	0.014625697
LEAP-2	0.063763135	6.123439799	0.014646511
RP11-529I10.4	0.10991588	7.984823174	0.014648369
LOC644012	0.074629934	6.264408184	0.01465616
RPESP	-0.12896953	6.587808927	0.014666057
POFUT1	-0.151900447	9.216087262	0.014677931
BSN	0.072309112	6.323834803	0.014687571
TMEM219	0.13693454	9.321836432	0.014690142
COX7B	0.112860819	12.45995204	0.014704888
TMSB4Y	0.123529492	6.157247863	0.014709165
TNFAIP8L2	-0.05325717	6.069545411	0.014727525

LOC644380	0.072191552	6.257166932	0.014728873
LOC441087	-0.181730448	12.84825778	0.014734728
LOC652404	0.06322106	6.106219841	0.014738375
PGM5	0.115057135	10.75846479	0.014767145
FOXP1	0.080407723	6.327531489	0.014777889
DCTN3	0.111858655	9.908479882	0.01477931
RASGEF1C	-0.038891546	6.077909165	0.014788555
RGS3	0.033793982	6.182140288	0.014796385
MEF2D	0.129228271	9.373553696	0.014825561
LOC646304	0.057402024	6.239670137	0.014867847
SOD3	0.125976422	6.855209352	0.014880993
CHPT1	-0.151844702	9.816723752	0.014892571
TPK1	-0.080539353	6.844806736	0.014895972
ACPP	-0.063293638	5.986756901	0.014897486
XPA	0.085788987	6.322579737	0.01491415
LOC554208	0.115027837	7.05292962	0.0149162
KIF2A	-0.093450453	6.824308485	0.01492667
LOC651723	0.048311032	6.085046664	0.014952044
KIAA0664	0.163588936	9.121902722	0.014952557
GOLPH3L	0.090891387	7.086611159	0.014973737
C9ORF5	0.133215097	9.813169431	0.015012047
HIST1H2BL	0.068343799	5.983636374	0.015025235
ZC3H12A	-0.105220636	6.424461967	0.01503632
NTRK2	0.064215538	6.322560347	0.015041735
KIAA1199	0.141401924	6.249486983	0.015073571
PRRG4	-0.086541385	6.641425726	0.015083288
DEFB118	0.063431203	6.150040073	0.015083619
GPR177	0.113274636	8.704356798	0.015084409
DUSP11	0.068039891	7.569238851	0.015094534
LOC653227	0.060503903	6.057275267	0.01510677
KCNH3	0.063769592	6.200709425	0.015119996
SLC6A19	0.068376592	5.979317342	0.015137772
KREMEN1	0.064479313	6.679255778	0.0151562
FAM160A2	0.099644731	7.643581473	0.015161807
LOC440396	0.049491629	6.203112784	0.015161987
SQRDL	-0.106797357	7.194768903	0.015162882
HAX1	0.119929525	9.150264065	0.015185519
HIBADH	0.153043996	10.62077625	0.015188595
RTP4	0.095752629	6.594756093	0.01519816
VCPIP1	0.070217245	7.699074717	0.015207776
C17ORF74	0.074505784	6.529592665	0.015209714
LASS1	0.060952104	6.188661409	0.015243346
LOC642113	-0.163029414	6.500070467	0.015245094
CLTCL1	0.095982226	6.415437286	0.015254335
MAP3K12	0.118329073	6.33233416	0.015261335
RBM25	0.10725185	9.119620486	0.015270724
LOC653748	0.038721881	5.990898442	0.015279153
LOC220729	0.056141399	6.356114522	0.015296386
CBX2	0.060737729	6.610506461	0.015297867
PANX1	-0.132583142	6.928947868	0.015303531
MCART1	-0.197771497	10.8111796	0.015314111

ALDH18A1	-0.111714174	7.343947582	0.015319029
SUDS3	0.080713189	6.37089064	0.015374169
C1QTNF2	0.065208996	6.203049845	0.015401222
UQCRH	0.10624302	12.69209553	0.015414995
KNTC1	-0.087455259	6.661234262	0.01543604
ZFX	0.124185252	7.069474733	0.01543988
LOC728069	-0.139507454	6.803519977	0.015443678
KIRREL	-0.070770957	6.107028194	0.015468846
ATP7A	0.074350459	6.444986812	0.015477836
ATF7IP	0.060254589	6.187466546	0.01548019
PDCD1	-0.091357734	6.149519591	0.015484634
MYH14	0.079748042	6.661084121	0.015487342
EPS15L1	0.100798448	7.521303	0.015492587
ITGAL	-0.075329857	6.219941071	0.015497586
RFC3	0.048044231	6.446442425	0.015528332
LOC81691	0.101514521	7.227839824	0.015545728
LOC651407	0.062590876	6.137860279	0.015548227
FAM36A	0.178890057	8.126933907	0.015574847
FAM80B	0.075760779	6.487481189	0.015607476
THEM4	0.102442824	6.471218889	0.015665649
GMPR	0.185805836	8.583534519	0.015679156
SLC44A5	0.062463089	6.050326634	0.015699364
DDR2	0.099059782	6.829016577	0.015699481
ATP6V1G1	0.1181086	10.88393969	0.01574875
SH2D1A	0.106371984	7.247900786	0.015755698
RPS6KA5	0.108428782	6.802460441	0.015759432
ZFAND2B	-0.111759967	8.31633063	0.015762646
TWIST2	-0.039425834	6.161298601	0.015804592
LAT	-0.034199159	6.134375842	0.015804722
MRPL39	0.131409027	8.891541026	0.015808157
SLC12A8	-0.065469712	6.23236778	0.015822546
CCDC102B	0.072074381	6.048634078	0.015825071
FBXO16	0.063862978	6.111339021	0.015831392
HSD11B1L	0.052896966	6.667999071	0.015834494
FAM76B	0.101230777	6.804568127	0.01583551
RDH16	0.065818745	6.064731294	0.015843282
OTUD7A	-0.044070248	6.132532851	0.015854226
CCL24	-0.057553547	5.905469904	0.015855818
AKAP7	0.100210284	6.971620954	0.015860967
KIAA0101	-0.10599052	12.51927106	0.015865349
WDR5B	0.066714406	6.221610311	0.015866395
LOC644268	-0.057955824	5.947631792	0.015907538
LOC652623	0.07551969	6.206737147	0.015914652
ANAPC7	-0.095719996	6.756951356	0.015945357
RAP1A	0.070711373	6.719424389	0.015958115
RBM9	-0.125737035	8.535536316	0.01598226
PCBP4	0.076042607	7.668456466	0.016010259
DKK4	-0.068284851	5.986467339	0.016016618
CNN3	-0.145484043	10.68518192	0.016037901
LOC645070	0.058903281	6.113664534	0.016063002
FALZ	0.072405448	6.275058886	0.016108884

HIAT1	-0.087654433	7.015109636	0.016114172
GABARAP	0.126819341	7.434936373	0.016125416
HNRNPF	-0.08459626	6.592780001	0.016153886
TMED10P	-0.106505377	11.40020666	0.016158798
IL6ST	-0.05958788	6.162571214	0.016161534
MMAB	0.063463256	6.554139816	0.016182552
SNRPG	-0.131243592	11.99614847	0.016183601
SNPH	-0.120652091	6.209924539	0.016207026
CCDC97	0.089095633	7.446729636	0.016207785
PROM2	0.084652903	6.378097636	0.016240845
GDPD3	0.061690178	6.134210842	0.016250109
NT5C3L	-0.107328243	7.984924148	0.016261986
CLDND1	-0.098926516	9.68539169	0.016262202
ZBTB46	0.093006204	7.562385616	0.016275314
LAIR2	-0.052950809	6.246117126	0.0163203
FTHL12	-0.17299953	11.46820509	0.016324771
LOC653803	0.076547783	6.311983926	0.016334271
DSC2	0.11984775	8.268823015	0.016339752
LILRB3	-0.144040936	12.49168081	0.016340538
RAD51L3	0.045497781	6.341796965	0.016368126
SVOP	0.144006913	6.542591311	0.016369128
LOC649775	0.059700182	6.018120438	0.016390588
LOC220686	-0.090573652	7.287136136	0.016398882
DDX28	0.149726322	7.973221943	0.016411417
TMEM134	0.125769034	9.376491462	0.016436461
C19ORF56	0.164517823	10.43816945	0.016439001
SYNPR	0.072717319	5.97862933	0.016449581
USP9Y	0.202892537	6.469325541	0.016469195
SLC13A1	-0.044885664	6.094554288	0.016496406
HBXIP	0.102561357	11.05126303	0.016502995
BTG1	-0.12411374	10.88633064	0.016504134
MRPS17	-0.10307681	9.519124368	0.016534083
GPC4	-0.18708554	7.395979173	0.016589913
PUS7L	0.082575648	6.552518154	0.016599085
OR3A4	0.1021882	7.518439893	0.016599351
SELM	-0.174325251	9.139490014	0.016640418
TAF7L	-0.058202251	5.883435934	0.016680923
KRT19	-0.075525278	6.001605662	0.016681039
METTL2A	0.057076745	6.441072559	0.016683241
SHROOM2	0.070108769	6.498398308	0.016683805
GBP5	-0.087886874	6.191587175	0.016698244
CECR1	0.263271452	8.729103511	0.016698858
NDUFA11	0.106840254	12.12455446	0.016730448
COMMD5	-0.063539898	7.790430917	0.01673061
LOC727825	-0.110238873	7.568845983	0.016741904
PLA2G2D	-0.118573024	7.224424412	0.016755507
MED24	0.107119629	8.026176223	0.016795396
C17ORF28	0.07211043	6.529358829	0.016798516
LOC646483	-0.14669455	11.54740793	0.016808268
CDRT4	-0.074059629	6.819899831	0.016809348
PSG6	-0.034656763	6.04688442	0.016821283

SLC24A1	0.071354475	6.433549608	0.016832431
CD8A	-0.065560745	6.213068356	0.016857055
ACTR3	-0.231402103	8.594275351	0.016879357
LOC650885	0.066222943	6.296778484	0.016891029
FSD1CL	0.081482251	6.151658343	0.016910811
ABI3BP	0.229023352	7.828300676	0.016918265
GBE1	-0.166456303	9.935564674	0.01692468
MRPS24	-0.115199835	10.94351477	0.016928128
NCOR1	0.079697986	7.089004919	0.016930948
NLRP12	-0.045233808	5.986098266	0.016966637
CPVL	0.175715818	10.92030848	0.016984638
MAP2K4	-0.077739202	7.708571238	0.016998303
OSBPL11	-0.117886253	7.115884991	0.01701657
HDDC3	0.103597254	7.777525998	0.01701958
CCDC137	-0.064751751	6.244980359	0.017027331
LOC653071	0.210852155	7.723681243	0.017027504
MGC48637	0.04424901	6.125561113	0.017066086
ZBTB5	0.080469461	7.04983677	0.017067062
TRIM16L	0.0885942	6.193928267	0.017067583
PEX5	-0.126286885	10.06086975	0.017077517
C19ORF41	-0.053106561	5.974070819	0.01708136
MYPN	0.195012971	9.960655834	0.017100728
LOC651816	-0.142632861	8.199933971	0.017106316
FAM130A2	0.102075466	6.401732222	0.01713204
SFRS10	-0.16727769	9.287233993	0.017132541
C19ORF39	0.065993678	6.356529075	0.01714803
SLC6A12	0.059890904	6.278208222	0.017153532
ZBTB45	-0.129982136	7.127058702	0.017189026
PRPS1L1	-0.041807731	6.155890407	0.017195877
THOC1	0.086626224	7.852068461	0.017207437
KLHL23	-0.084519018	6.529595142	0.01723271
SSBP4	-0.086534887	7.260560457	0.017245034
DENND1A	0.049300669	6.510388056	0.01725735
CCBE1	-0.191185474	8.211759677	0.017278875
PCDHB8	-0.06226339	5.993727415	0.01729021
LOC645004	-0.047404052	5.924860341	0.017301723
TMED1	-0.105145791	9.270369426	0.017306794
NKX1-2	-0.06328964	5.970151432	0.0173536
SLC38A4	-0.061968555	6.023590429	0.017410781
PGM2	-0.097239977	7.09392452	0.017411269
MCART2	0.046319755	5.982266343	0.017420892
C5ORF33	0.118745966	7.15655572	0.01743287
LOC387882	0.113237163	6.381475932	0.017439528
SPEF2	0.048618639	6.096629524	0.017448302
LOC653338	0.058781564	6.061131003	0.017481494
WFIKKN1	0.062665686	5.94275806	0.017493439
PDLIM3	-0.29715099	10.99368441	0.017494715
LOC730031	0.070226956	6.362937013	0.017505875
TTC39C	-0.06557741	6.364488251	0.017506454
IGFBP2	-0.300867748	8.686723699	0.017514287
DKFZP564J102	0.100843004	6.749770961	0.017517991

LOC643821	0.047907659	5.98302882	0.017539475
CENTD2	0.08857046	6.556418381	0.017554258
TBC1D3B	-0.04005795	6.12162878	0.017555597
MIA3	-0.045223484	6.271045582	0.017558738
LOC202181	-0.041863173	6.123826362	0.017571592
CCDC74B	-0.055512907	6.042502295	0.01759736
TNFRSF9	-0.07421819	6.147506899	0.017602647
TFPI2	-0.088192057	6.213794171	0.017663559
STK35	-0.158747654	6.78368056	0.017670269
TDP1	-0.141448519	8.486251702	0.017707749
BBS10	0.151845954	6.520120167	0.017711337
SLC6A15	0.043547031	6.214359705	0.017718287
USP5	0.102925049	9.650064912	0.017745616
FRMD7	0.078889544	6.033101359	0.017752333
CISD2	0.085735555	7.084170693	0.017756498
C11ORF87	-0.057054948	6.037307306	0.017786098
THAP2	0.045629795	6.235027019	0.017789292
PSMC1	0.075843431	12.05310255	0.017794702
LOC613037	-0.197983146	9.935817293	0.01781171
OR2L13	-0.048001343	6.006519751	0.017814657
MS4A14	-0.055274704	6.234788831	0.017841512
HUS1B	0.073447102	6.847063263	0.017848188
MRPS11	0.094563801	9.850318307	0.017860398
HLCS	-0.045249353	6.337081946	0.017874735
KCTD3	-0.07256661	8.01277396	0.017882671
CCDC106	-0.165659904	8.918774208	0.017953764
MORN2	0.117424227	7.981371078	0.017973731
GTPBP2	-0.060388214	6.403939516	0.018046789
LRRC14	-0.080441356	7.419596594	0.018053996
ADRB1	-0.175993352	6.848132698	0.018060333
TDRD3	0.087463992	6.802620081	0.018077115
LOC650263	0.069012951	6.044860709	0.018110577
LOC728715	-0.094035614	6.554663718	0.018113389
LOC645058	0.141415041	11.12703713	0.018130801
NDUFA7	0.113437993	10.31760191	0.01813877
TBC1D17	0.113051698	6.988799026	0.018190853
TLK1	0.121507624	7.371980188	0.018197187
MORC3	0.081097829	6.768580939	0.018213304
C7ORF57	0.059298191	6.060603904	0.018256252
COX11P	0.057223843	6.218362438	0.018293265
IL15	-0.049889722	6.516867215	0.018299378
SUCLG2	0.12467463	10.37586882	0.018329676
ARMETL1	0.091331272	6.569972878	0.018364101
FBXO22	0.056581863	6.807445727	0.018370083
BMP5	-0.145764901	6.559159338	0.018378353
RRP1B	-0.07615248	6.570680117	0.018379193
FLJ39061	0.041886904	6.156499334	0.01839096
LOC606495	0.062829863	6.170835629	0.018399769
CCL18	-0.082922214	5.99246645	0.018437962
OR2B2	0.067385995	6.377082624	0.01846316
FAM131C	-0.047971911	6.224378212	0.018463376

UBAP2L	0.196074025	7.787967121	0.01847516
NOLA3	-0.106342133	11.22381075	0.01847851
IRS1	0.115988224	7.314338817	0.018499885
EHD4	-0.139804922	9.893456535	0.018538287
PRSS35	0.073189265	6.59215996	0.018540095
LRFN5	-0.066495791	6.182369892	0.018580458
LOC646766	-0.133753445	11.8712084	0.018600626
LOC652607	0.056639097	6.078001166	0.018606186
LY6G5B	-0.057189763	6.022660038	0.018626523
SRRM2	-0.134137353	10.6386801	0.018694283
SAMD14	0.076958491	6.584154474	0.018710064
USP2	-0.105005192	6.932231347	0.018710087
WBSCR16	0.062233719	6.846824059	0.018760036
IPP	0.140851615	6.986874457	0.01877327
KIF20A	-0.082533007	6.128075012	0.018789116
LOC653344	0.12083407	7.787770731	0.018794229
SNORD55	-0.08181533	6.222179533	0.018807672
SOX7	0.136845336	7.407360174	0.018810657
OSBPL8	-0.12207515	7.479889822	0.018824555
GGCT	-0.102971049	7.643925368	0.018828259
LOC644075	0.075664002	6.042397755	0.018828381
POLG2	0.135319751	6.516689527	0.018854477
RCP9	-0.184668233	9.224819557	0.018859361
ZNF185	-0.066045499	6.497393834	0.018885785
GLT6D1	-0.067283799	6.108483199	0.018888106
RASSF6	-0.04205803	6.362733483	0.018908487
LOC653108	0.134576994	7.084958851	0.018919626
LOC645983	0.050070357	6.001504574	0.018922893
CLMN	-0.066464395	6.252226325	0.01896104
HHLA3	0.042031397	6.122217143	0.018978376
KCNK17	0.100587544	6.396188507	0.018998762
PRPF6	-0.085299588	6.601626294	0.019014826
PISD	-0.118345871	7.236346229	0.019022794
CGI-96	-0.09453889	6.471721749	0.019044731
CRHBP	0.071916372	6.398267375	0.019046226
APC	0.08478829	6.166170388	0.019055189
SAFB2	-0.128567623	8.401778181	0.019062963
GNB2	-0.117896518	7.089605595	0.019084019
LOC647596	-0.059717841	6.061299012	0.019096631
MTA1	0.155996018	10.27232028	0.019115118
GEN1	0.040985912	6.12051408	0.019126939
TBL1X	-0.104078474	8.253532651	0.019136106
NFYA	0.049148674	6.265311771	0.019137201
CITED4	-0.172654539	8.395718882	0.019165542
PAP2D	0.051297932	6.008978246	0.019167084
MGC62100	0.068459977	6.225670924	0.019184185
NAPRT1	0.128692153	8.91125536	0.019202705
VPS13C	0.096703236	6.853523773	0.019227041
INHBE	0.102647919	6.016948482	0.019234677
LOC134145	0.069592424	6.091471676	0.019274064
ACCN2	-0.046761685	6.26096331	0.019286665

IL10	-0.173014769	9.548465952	0.019295125
HCN3	-0.05862619	6.351200861	0.019304352
PDCD7	-0.110125075	12.41882336	0.01934512
C14ORF11	0.097423174	6.217203183	0.019347822
LOC642559	-0.059931371	6.139543774	0.019378316
DNCL1	0.170430992	11.40494762	0.019382912
SSH3	0.087075764	7.055912167	0.01940514
EIF2AK2	0.122022856	8.956127264	0.019415917
UGCGL2	-0.081835515	7.751560565	0.019417865
RPS6KA6	-0.093644831	6.425117609	0.019419856
POPDC3	0.13198275	8.052773284	0.019431017
RNF6	-0.053572862	6.263712585	0.019466677
GATS	-0.141909952	7.244922458	0.019472554
ZNF770	0.082588338	8.438211588	0.019501212
PVT1	-0.050997584	6.00964267	0.019522322
NACAP1	-0.133779496	6.469863713	0.019548042
C1ORF41	0.111024773	7.862588255	0.019553375
B3GNT5	-0.073759527	6.126413036	0.019560019
TMEM170A	0.14515168	7.04487947	0.019572122
SHB	0.127521286	7.624661907	0.019586814
ZNF420	0.082286732	6.183590105	0.019589566
C9ORF95	0.159024965	7.300567431	0.01961127
DTWD2	-0.185956969	8.943483704	0.019614177
SEC14L5	0.332819662	7.808899068	0.019618452
MTCH2	0.087458036	6.794045661	0.019621151
ARSB	0.086325358	6.854061754	0.0197517
SLC10A7	-0.036913272	6.22056702	0.019796735
C2ORF47	0.121426383	9.485189231	0.019819019
GZMK	-0.078221425	6.200855947	0.019837479
SRGAP2	0.069173068	6.910849389	0.019842987
HNRPH1	-0.126240823	7.520437295	0.019857814
TMEM69	0.147563832	8.566086372	0.019873074
IFRD1	0.181513886	7.636626532	0.019874748
ILVBL	-0.122506152	9.689191712	0.019897031
LAMB3	-0.058350952	6.357564488	0.019986988
LOC643652	0.05585469	6.067401819	0.020004857
LRRC8C	-0.101031039	6.565983231	0.020016863
WDR79	-0.116076715	6.696704111	0.020022137
FFAR2	-0.052681664	6.096324461	0.020026107
ABI2	0.052666872	7.114610153	0.020044921
C19ORF33	-0.186283332	6.756606214	0.020059133
LOC641926	-0.073443628	6.070828416	0.020092409
MRPL47	0.122027433	8.413357871	0.020110867
CDC14C	0.078396317	6.312493731	0.020119523
LOC646434	0.057857883	6.28776894	0.02014391
PIM3	-0.091351448	6.823132228	0.020157497
C22ORF26	0.05911827	6.124777822	0.020170927
ENO3	0.173510937	12.86883267	0.020171973
LOC653269	0.052183551	6.084237884	0.020191232
MAGEF1	-0.110507484	6.914074252	0.020208377
UCP3	0.072300083	6.314409821	0.020231674

MCM10	-0.048378744	6.079905637	0.020235259
MCM8	-0.159024535	9.595751611	0.020247288
CLDN5	-0.252697355	8.744964038	0.02026203
HECTD2	0.056208336	6.350860017	0.020271579
BAG5	-0.069383508	7.865672525	0.020308231
LOC729559	0.104270812	7.230192002	0.020311663
HIST1H2BG	0.09578075	6.593545187	0.02033725
C20ORF108	0.132781558	8.703623404	0.02034658
LOC652816	-0.054993862	6.201453516	0.020349659
ZP3	0.250719153	7.306029884	0.020372549
UBXN1	-0.08530681	10.38406031	0.020392421
HSPA12B	-0.135766791	8.196769383	0.020394372
LOC375295	0.049992764	6.25237094	0.020401814
MLPH	0.271385635	9.21923409	0.020422238
FAM62B	-0.129106733	11.22442117	0.020428248
PHOSPHO2	0.066977792	6.367937193	0.020440954
IKZF2	0.044652793	6.371064502	0.020461909
LOC645508	-0.063309172	6.050111695	0.020464972
ZHX1	0.079290764	6.576352446	0.020466652
ANKRD27	0.082890777	6.716769131	0.020471021
KATNA1	0.092892579	8.590996656	0.020472085
MPND	0.079968203	6.681643293	0.020489328
PXMP4	0.090680604	6.737355929	0.02049104
CA8	-0.145476592	6.843269866	0.020508559
C9ORF46	0.139440002	9.833007943	0.020516199
LOC649095	-0.068227166	6.207043275	0.020547195
EIF3H	0.09174017	10.67051436	0.020567609
SLC46A2	0.093427894	6.970263143	0.020572594
TMEM8	0.140570002	8.140941529	0.020601559
MORG1	-0.096159698	6.91757103	0.020608602
GPR175	-0.078484164	7.463948149	0.020700677
GGCX	-0.078027321	6.977520246	0.020711838
ALOX15B	-0.044915472	6.162909996	0.020729438
EPR1	-0.065075681	6.077052187	0.02073133
PER2	0.163335568	7.643595186	0.020837254
CLP1	-0.074312781	7.184380681	0.020855168
SRL	-0.13333161	12.72258643	0.020868702
SLC5A8	-0.15320346	7.110579792	0.02088755
SNN	-0.150406666	7.00413975	0.020905625
MAD2L1BP	0.097941745	7.726129501	0.020919294
LOC441528	-0.065531801	6.069645494	0.020925844
TYRO3P	-0.073735197	5.983116572	0.020939566
KLHL25	-0.085998928	6.127544694	0.020945013
YIF1A	-0.170419188	9.270319906	0.020965912
SCN5A	0.046404517	6.30169191	0.020966207
ATF2	0.075029658	6.682982208	0.020982622
PITX1	0.068025975	6.133681747	0.02098904
LOC652595	-0.147782455	8.558721749	0.021010794
LOC654161	0.09753474	7.012874514	0.02101559
MCM3APAS	-0.062418265	6.154379821	0.021020212
RAI16	0.127652035	7.225966107	0.02102705

RLN1	-0.055879503	6.005973621	0.021029157
PSMC6	0.137797612	9.491418752	0.021084736
PPFIA2	0.056391772	6.009703899	0.021112594
C9ORF106	0.063158494	6.215616188	0.021212633
PTPRZ1	-0.059377462	6.17702773	0.021213134
JAK1	-0.169424975	8.528572393	0.021232569
NKX3-1	-0.153552703	7.241213077	0.021237014
FAM139A	0.053252143	6.040867381	0.02124407
TSR2	0.15839044	8.863250445	0.021257466
TOPORS	0.049835858	6.611933396	0.021290971
MBD4	-0.174325256	9.946322249	0.021329278
LOC654074	0.132776834	6.788778988	0.021334116
COQ9	0.157365053	11.20351529	0.021409156
H2AFJ	-0.115562586	8.139572186	0.021416351
SLC13A4	0.071859911	6.226865837	0.021445586
CNKSR1	-0.060560725	6.320294321	0.021456713
NBEAL1	0.060667028	6.034074037	0.021457704
C6ORF62	0.091462237	8.048252881	0.02146354
ERAP1	0.04768618	6.351361377	0.021476204
UBE2M	-0.080650836	11.64998195	0.021498058
ZNHIT1	0.082211937	10.09165951	0.021537225
PCSK1N	0.08181003	6.122210762	0.02155356
TRIM26	0.077498696	7.518275061	0.021562266
GM2A	-0.155853311	6.943357067	0.021585796
PMF1	-0.103251604	7.554838528	0.02160602
BIRC5	-0.05632639	6.036273756	0.02161354
NKAIN3	-0.096931315	6.917776751	0.021625676
HBA2	-0.450593203	10.67368867	0.021627243
TIRAP	0.036992714	6.430475151	0.021633785
LOC648149	0.064494908	6.206030671	0.021663975
PPIB	-0.123623066	6.295499377	0.021674099
CCDC149	-0.078609862	6.401341137	0.021677272
LOC197135	-0.123558821	6.421094503	0.021681378
TIMM23	-0.07087673	10.33734916	0.021735458
DEFB109	0.056503474	6.069807899	0.021740478
CD200	0.056641176	6.458015873	0.021748822
MC1R	-0.085217051	6.752918677	0.021773466
ITIH5	-0.115590753	11.95495651	0.021775621
SDCBP2	-0.039654655	6.105544781	0.021781941
AHSG	0.095472101	6.366984631	0.021791991
HPS6	-0.110019225	9.479215813	0.021797516
FBXL3	0.091606101	6.737933611	0.021814151
DLL3	-0.046476501	5.932991118	0.021829963
ZNF576	-0.061969542	7.054092893	0.021851692
TBCD	-0.090769236	7.903090759	0.021878326
ACCN5	-0.059774069	6.016804522	0.021910551
LOC642287	0.058346533	6.024242438	0.021918462
DNAJA2	0.073374324	10.76299759	0.02198657
MFSD5	-0.084887402	8.170817457	0.022014528
C7ORF25	0.086447984	7.498800306	0.022016493
LOC652577	-0.058136145	6.006131137	0.022079031

CDC40	0.080025045	8.885741225	0.022150807
CAMK2D	0.121591186	7.869560297	0.022152753
CHAC1	-0.071923056	6.251483504	0.022205554
ICOSLG	0.10332661	7.961022379	0.022212063
RNF38	0.113640067	7.994404822	0.022231674
BICD2	-0.147222175	8.655165931	0.022243998
OR5J2	-0.057815273	5.890006981	0.022245874
TECTA	0.064611272	6.551519681	0.02226454
DHCR7	-0.105078481	7.472565507	0.022279247
GPR89A	-0.080896796	6.585553289	0.022353417
MSH3	-0.110050239	12.99802033	0.022378369
LOC116236	-0.082561322	6.680369832	0.022395345
CDH5	-0.144703061	9.371143077	0.022409792
LOC653801	0.053936623	5.904319809	0.022411623
CENPK	-0.068519147	6.151293286	0.022414852
HSD17B7	-0.152148109	11.26150326	0.022437615
PRR14	-0.1290273	8.745533687	0.02245431
HNRPR	-0.126791259	10.06003001	0.02246022
DTX3L	-0.079774493	6.288375819	0.022464477
FLJ45717	0.07178199	6.332951792	0.022477578
LOC646447	0.057988116	6.067747344	0.022489265
GLI1	0.075357887	6.636471206	0.022509925
BCL2L2	0.155623483	9.153924933	0.022539589
KIR2DS1	0.044116552	6.034492143	0.022542117
COL1A1	-0.378987321	8.543471008	0.022552129
TSGA14	-0.050279285	6.321802531	0.022556539
GLG1	-0.092429315	10.75435255	0.022557433
IL17RC	-0.039505568	6.369809675	0.022560536
ARF4	-0.119633271	9.700185586	0.022570439
FMNL3	0.056388835	6.402962848	0.02258633
CD79A	-0.038443579	6.166030294	0.022609994
GYPC	0.11584123	9.946228474	0.022618137
LOC653326	0.055150981	5.930032302	0.022632726
NANOS1	-0.055043117	6.212734577	0.022661781
KIAA1751	-0.160204352	8.31986932	0.022678141
ANKRD22	-0.048445769	6.077582362	0.022681437
TMEM39A	-0.086727739	7.027881994	0.022687433
TTLL6	0.052122949	6.098889281	0.022688668
LOC650968	0.055503419	6.139657345	0.022690466
ARG99	-0.066762757	6.122730463	0.022770145
MNT	0.088296683	7.603113969	0.022770814
RAB18	0.098674122	7.24486161	0.022778611
LOC654113	-0.057639375	5.964543086	0.022786415
DDX26B	0.046100215	6.153399906	0.022797552
CNR2	0.055697498	6.01771796	0.022828336
TEX10	-0.072456076	7.543277252	0.022828398
GAL3ST2	0.079340918	6.545432762	0.02283744
CDKL2	0.045745632	5.969542618	0.022838396
C6ORF163	0.05973386	6.162684641	0.02292252
LOC391322	0.051682019	6.188826498	0.02294936
C8ORF76	-0.089074889	9.184888984	0.023023126

LOC645447	0.033012637	6.046615738	0.023054013
EGR2	-0.204744202	7.057331514	0.023061664
ZNF430	-0.127512995	11.22542416	0.023066448
TBC1D3	-0.067032145	6.028545984	0.023090426
BFSP1	0.066283494	6.002134462	0.023090999
MBTPS2	0.068940685	6.384249338	0.023114926
FAM122A	-0.049154562	6.518017828	0.023122242
RARG	-0.055362239	6.233206601	0.023127053
HOXB2	-0.134485574	6.87851231	0.023146407
NEDD4L	0.137783271	8.459028699	0.023187116
HS2ST1	0.095678701	6.805486496	0.023202417
ITGA2B	-0.076514642	5.912305536	0.023218377
RDBP	-0.090405788	7.490065996	0.02324836
CYP2C9	-0.062402017	5.950959637	0.023262867
MAPKAPK2	-0.149419947	7.933002642	0.023274455
JMJD4	0.083784444	6.661769171	0.023294649
OR6B1	-0.069135899	6.229894462	0.023302958
MORC2	0.096197479	7.418460749	0.023309713
STX8	0.125659353	8.519154968	0.023320974
ARHGAP29	0.069025173	6.281920565	0.023327233
DOK2	-0.066522916	6.050452965	0.023329062
RPL17	-0.134993643	12.19458521	0.023342586
UNQ9370	0.077412002	6.06071768	0.023344319
LOC650566	0.059747893	6.02365013	0.023380406
GAS6	0.132113977	10.33966178	0.023395986
ZHX3	-0.120204318	7.467201977	0.023396655
F8A1	0.08841226	7.63076113	0.023402652
PIGT	-0.093962668	10.00520299	0.023412334
SCN9A	-0.045401966	6.330182094	0.023418732
FAM65B	0.180056862	7.194945784	0.023450605
ZNF174	0.058281975	6.624442924	0.023461336
SCHIP1	0.141320533	10.09334489	0.023465496
TMEM20	0.083417799	6.517292865	0.0235158
C10ORF111	0.056730954	6.119729017	0.02353596
GPLD1	0.039308178	6.021457377	0.023538153
SGMS1	-0.073250308	6.470216809	0.023572257
EML4	-0.118659136	7.60767773	0.023580706
PMAIP1	-0.042738239	6.111822689	0.023640124
C5ORF5	0.128247722	9.107633552	0.023716067
C10ORF108	-0.077203796	6.152797825	0.023757384
C8ORF38	-0.113025785	8.125493611	0.023772086
MRPL20	0.125433059	9.882187677	0.023776594
PCGF2	0.090410028	7.702554611	0.023830283
IFFO1	0.113669152	8.344376122	0.023831659
ZSCAN21	0.069017696	7.1034726	0.023893541
ZPLD1	-0.064429253	5.912355136	0.023951155
KDELC2	-0.107001318	8.160642982	0.02406743
CAPS2	0.066067118	5.99938289	0.024079973
LOC352909	0.074821107	6.03881251	0.024101426
RNF113A	0.149220935	7.584638429	0.024124103
LOC643831	0.065224481	6.520145609	0.024133949

LOC643774	0.0625209	6.026767011	0.024136047
ACE	0.037129419	6.071378303	0.02416432
C22ORF29	0.083591411	6.936838868	0.024175412
LOC646676	-0.053346023	6.184276607	0.024177865
SYNJ1	0.077674245	6.843130113	0.024257436
ADIPOQ	-0.061896415	6.28562807	0.024290408
LOC647135	-0.032318425	6.130596125	0.024303295
C17ORF95	0.096947526	7.039322737	0.024303454
FSIP1	0.103670194	6.353862397	0.024308702
FBXO48	0.044449983	6.121177884	0.024310978
OR14I1	0.055780756	5.998802309	0.024328959
ELF2	0.066909681	7.768092604	0.024330378
CNTNAP3	-0.08598218	6.12176784	0.024351905
FSCN1	0.192293224	9.918385312	0.024379341
TMEM191B	0.039461226	6.15188108	0.024415731
SLC39A13	0.081928648	6.443620699	0.024428356
EXOSC3	-0.092710536	7.923162187	0.024431868
RAB27B	0.040481537	6.151756881	0.024463301
EIF2AK4	-0.126873707	8.363413783	0.024483618
RPL13A	-0.232350704	9.105001118	0.024516165
ZNF317	0.081224953	7.556740728	0.024541945
RBX1	0.089484621	11.14420185	0.024602479
DDB2	0.076865893	6.286331406	0.024638882
LOC650577	0.058893983	5.98104273	0.024654875
COL4A4	-0.126451049	6.36951403	0.024672365
C2ORF7	0.125876907	9.469578602	0.024709378
NARFL	0.066265296	7.686699704	0.024754538
YIPF2	-0.077060941	6.994792814	0.024768456
DFFB	-0.058738138	6.382799285	0.024772359
GLE1	0.155486091	8.174373599	0.02478464
SCML2	-0.064001459	6.484195996	0.024808574
APBB2	0.12271377	7.302360932	0.024814323
HCG2P7	-0.167046257	10.47415745	0.024819594
PIK3R3	-0.107859629	6.404654641	0.024825339
KLRF1	0.070563154	6.159235381	0.024828957
LRRC24	-0.060846367	6.012990238	0.024830107
FAR1	0.089552628	8.134464304	0.024866638
LRRC34	0.088094763	6.004382495	0.024895254
GHRL	-0.061644238	6.08163249	0.0248974
LOC650003	-0.079649499	6.104138741	0.02490578
CCR9	0.063427288	5.977915596	0.024926786
LSDP5	-0.159641696	7.320198845	0.024934114
VARS2	0.135131948	8.689283489	0.024967064
HMGB4	-0.039675147	6.026270233	0.024969401
TAS2R10	0.068169298	6.231243271	0.02497575
GCH1	-0.054525215	6.115769153	0.025003988
C14ORF124	-0.152332569	7.496270547	0.025004181
MYO1B	-0.149835888	7.917039641	0.025016901
CREM	0.130772998	6.436905571	0.025021842
CP	-0.054738448	6.077146866	0.025072091
LOC643959	-0.051485428	6.11252007	0.025086032

LOC650193	-0.05279627	5.944540249	0.025097153
LPIN2	-0.056890647	7.056436698	0.025102826
LIN54	0.070741766	6.860825492	0.025153907
TSSK6	0.066315303	6.159911778	0.025161733
NR1H2	-0.102541387	8.822430945	0.025179411
RFC5	0.060335728	6.905236281	0.025185223
LOC643790	0.06914141	6.477738288	0.025196815
LOC730479	0.055443451	6.047007189	0.025241875
IL12RB1	-0.033998479	6.061787021	0.025244389
NMT1	-0.125884142	7.218009175	0.025258308
MATK	-0.047693446	6.192354732	0.025266524
CREG1	0.121960629	9.458296017	0.025275254
LOC646437	0.059911863	6.384644101	0.025308563
LOC651326	-0.067262003	5.929928784	0.025312154
CCT5	-0.101249869	6.839361939	0.02534147
TP53I3	0.091916274	6.951036685	0.025351008
LOC729882	-0.050484399	6.106132479	0.025377655
METT5D1	0.09347511	6.846155873	0.025390692
LOC162073	-0.090814158	6.33961648	0.025423747
C5ORF45	0.048004675	6.482468013	0.025432537
LOC729137	-0.056778294	6.254662494	0.025445913
PSMD12	-0.191835111	10.24645504	0.025461329
SLC25A23	0.137467307	11.10583307	0.025495077
LOC729675	0.058015456	6.176319946	0.025512707
IARS	-0.117968576	8.032372624	0.025535246
LOC642156	0.054482935	6.209676009	0.0255567
MBOAT2	0.08487975	6.769506062	0.025580755
RHOT1	0.092805262	8.394211274	0.025582541
HES4	-0.144756735	11.12806493	0.025592269
C15ORF37	0.051066782	6.057156545	0.025612612
BDH2	0.080173809	6.164015794	0.025654672
C1ORF59	-0.078519381	6.796399896	0.025670277
UHMK1	-0.080406405	6.351343821	0.025675281
ZNF625	0.050956972	6.297363149	0.025678294
SLC25A42	-0.11490084	8.534163119	0.025683512
LOC645015	-0.060370791	6.196621933	0.025730955
SV2A	0.065009843	6.168069464	0.025739969
ATP6AP1	-0.14680531	11.29630632	0.025757815
LOC653450	0.11073106	7.443381149	0.02577278
HR	0.043746166	5.94839068	0.025783714
LOC387790	0.0819899	5.981133497	0.025790912
CCDC71	-0.112822919	6.269627822	0.025801042
RASA2	-0.061515544	6.30485006	0.025837008
SEC24B	0.059439697	6.934863932	0.0258474
UBE2L3	-0.097129196	8.216621353	0.025856114
SETDB1	0.08542448	7.273048427	0.025884527
MITF	0.085227617	7.243239095	0.025891698
LOC643995	-0.051731561	6.220138271	0.025921049
LOC646858	0.053570008	6.087819094	0.025925665
ALKBH7	0.122841526	9.442764836	0.025934888
HERC3	0.106134946	7.640139452	0.025936469

APH1B	-0.088035044	6.867349322	0.0259375
LOC653383	-0.104450085	7.019249772	0.02593917
SLC27A3	-0.201167797	7.590414079	0.025941481
KCNRG	0.057208794	6.178888619	0.02594442
LOC644150	0.07521079	6.429704799	0.025968643
CPT1C	-0.082184941	6.553625793	0.025998251
WNK3	-0.043122044	6.123939018	0.026004156
SPATA13	-0.11551359	6.71153527	0.026004973
MRPS10	0.100743836	10.52466643	0.026045578
DCP2	0.091904379	8.135825446	0.026096055
C6ORF201	0.056127429	5.987455863	0.026124753
PLEKHJ1	-0.107298127	6.881675561	0.026126053
LOC391767	0.057665368	5.989973674	0.026139567
PTGER3	0.043522789	6.115155147	0.026177129
LOC127406	-0.058890237	6.069780264	0.026205298
ACAP3	-0.083608477	6.293823321	0.02621005
ZNF236	0.055345251	6.328592482	0.026290836
DYSF	-0.137802774	9.263222148	0.02631776
LOC400464	0.079118289	6.559172399	0.026367544
MRPS9	0.134534112	10.06941776	0.026374456
ADAMTS17	0.061366118	6.282906192	0.026375159
UBQLN1	0.09240391	9.033902736	0.026424368
ZMYM3	0.070486317	6.386382273	0.026427988
ARFIP1	0.07078321	7.011697117	0.026485315
LOC652140	0.057210255	6.21198037	0.02653977
DOLK	0.075502834	7.543888611	0.026573389
ZNF835	0.054702989	6.036598091	0.026582954
C22ORF31	0.061549557	5.94426542	0.026586271
CLEC2B	-0.108689531	6.699423216	0.026596881
CD72	-0.061931521	6.293918758	0.026608919
ZNF566	0.044974386	6.274607845	0.026609314
POM121	0.049196755	6.099319261	0.026637398
LOC652889	-0.056517211	6.124060637	0.026682302
LOC654032	-0.043179975	6.013120081	0.026686571
PLS3	0.111517123	9.454295153	0.026725908
TESSP5	0.109957245	6.285807895	0.026728927
PTPN22	-0.029456938	6.035970764	0.026779949
TNFRSF4	0.176381358	7.137274155	0.026783352
C15ORF5	0.084375061	6.392198849	0.02681717
GINS3	0.106408812	7.818045118	0.026943559
DAPP1	-0.151546745	10.42514607	0.02695024
PIM2	-0.083320541	6.728374431	0.026954935
GRIN3A	-0.062901061	6.120417831	0.026998369
DAXX	-0.087945592	6.810714295	0.026999571
TSN	0.132816906	6.51110094	0.02701571
ENC1	0.159135809	7.335660282	0.027041736
DNAL4	0.102037716	8.608359859	0.02707617
SSR4	-0.112341786	10.89139572	0.027078568
WWTR1	-0.08145701	6.274691352	0.027103809
FUT8	0.061469595	6.517560112	0.027108295
B3GNT9	0.059810548	6.123523135	0.027119146

RPUSD3	-0.092236901	8.675915457	0.027153945
AHDC1	-0.087742358	6.760935881	0.0271751
FAM118A	-0.127285093	6.393826696	0.027183905
RICS	-0.073701322	6.726835456	0.027221691
PICK1	-0.129097586	7.724361404	0.027252649
NOC3L	-0.151457257	8.3561939	0.027270699
EIF3K	0.087471162	12.07089613	0.027275267
TCF15	-0.127034882	7.231716354	0.027288242
MSL2L1	0.054674169	6.708377614	0.02729556
PCYT1B	-0.049497686	5.951192368	0.027297713
LOC646938	-0.061429637	6.414206486	0.027316929
SFXN4	-0.084017615	9.335978279	0.0273221
USP1	0.073108966	7.01110157	0.027341302
NCOR2	-0.102614073	9.186747117	0.027362
SERF1B	0.119019788	8.016427118	0.027365556
CLIP4	0.147177798	8.221078707	0.027366554
LYG2	-0.06483922	6.041437352	0.027368225
SCP2	0.087133163	6.825962562	0.027372402
NDUFB1	0.126868685	7.33178209	0.027397554
NNAT	-0.040341862	6.152792251	0.027398063
CMC1	-0.105662903	8.465518584	0.027420167
LOC255326	-0.064221433	6.31224998	0.027428514
KLHDC8B	-0.142421469	10.76132062	0.0274551
C6ORF159	-0.04455055	6.064905612	0.027463751
YY1	-0.132418564	8.692873551	0.027469597
CENPC1	0.075307983	6.287478279	0.027490318
VASH1	-0.165283863	7.884800959	0.0275129
SNORD34	-0.052263008	6.072699849	0.027534321
RPS6KA4	-0.123478114	7.099097304	0.027545195
LOC642418	-0.052245519	5.998051199	0.027572048
C2ORF27	0.058621288	6.028085425	0.027576169
CST2	0.123975949	6.012439379	0.027635866
ABCA11	0.072820865	6.474794412	0.027642571
TMPRSS12	0.048714793	6.150497142	0.027683621
POLG	-0.093422847	7.620161031	0.027699016
PRELP	0.061403876	6.421060991	0.027714137
GSTTP2	-0.058703068	6.460822808	0.027746165
ZNF532	0.088842928	8.122995239	0.027782566
NTSR2	-0.031651557	6.049874064	0.027790396
LOC648639	-0.059631309	5.950554816	0.027799598
ALMS1	0.079798604	6.647889209	0.02782149
FARSLB	-0.114424474	14.12410866	0.027837758
LOC643870	0.134295885	7.281487272	0.027866496
LSG1	-0.107432351	7.112672601	0.027967095
UAP1L1	0.097392757	7.008946028	0.02797457
TMEM91	-0.102853982	7.398082184	0.027995005
ALAS2	0.04198793	6.119145523	0.028030294
ANXA11	-0.096087725	8.750888923	0.028087218
C7ORF59	0.245739542	8.902231594	0.028131492
SRD5A1	0.063556865	6.340694085	0.02822146
TRIM37	-0.06131345	6.86022315	0.028252805

LOC646134	-0.053938581	6.076460951	0.02826568
RDM1	0.043894449	6.044692595	0.028290597
C1ORF107	-0.046670728	6.20857709	0.028293986
IGLL1	-0.199994282	6.660260653	0.028350816
LOC389517	-0.121717138	9.871798546	0.028359035
ABR	0.108463825	9.423814027	0.028364276
CALR	-0.133613934	8.87095495	0.028372382
SPG7	-0.110917115	9.162846805	0.028470072
FMNL1	-0.059858598	6.225433244	0.028475091
ZNF319	-0.069789576	7.8166402	0.028500919
LOC730015	0.073575937	6.134615981	0.028528129
NCL	-0.098748133	9.823267965	0.028530375
PCGF6	0.076250826	7.297766935	0.028574744
JARID2	-0.140932034	7.909436195	0.028580087
LOC648814	0.070833178	6.289198529	0.028589091
LOC644243	0.050014153	6.11131492	0.028670604
SCGB2A2	-0.05690189	6.003693162	0.028680059
NEK9	-0.058336874	6.164681797	0.028694554
RECQL4	-0.056589006	6.025330151	0.028701515
FAM22A	0.038198597	6.175437267	0.028721363
GABPB2	-0.118523723	11.11209037	0.028750732
VPS4B	0.08741579	8.504755816	0.028764614
HERC2P2	-0.029059906	6.189327406	0.028790365
SDS	-0.052336575	6.031108045	0.02879114
CNTN6	-0.056028727	6.451346052	0.028829311
ERV3	0.088445276	6.302524065	0.028846478
TRIM71	-0.040119409	6.064853608	0.028865604
LOC646890	0.04605498	6.085742558	0.028885683
BET1L	0.095975123	6.359167369	0.028951631
CRABP2	0.088487482	6.449040138	0.028956831
GALR2	-0.068055542	5.950743274	0.02896509
LRRC50	-0.068993907	6.099195248	0.028990474
SHRM	0.191741466	8.815581264	0.029053359
CTGF	-0.322172029	10.88258146	0.029063474
FAM5C	-0.084682959	6.331554078	0.029066186
NT5DC1	0.119034445	7.690751642	0.029078611
TAC3	0.040753624	6.270654619	0.029098244
OAS1	-0.090964037	6.352975136	0.029142856
STELLAR	-0.052403638	5.953271825	0.029145519
NHEDC2	-0.047115595	6.255457772	0.029150485
BAGE3	-0.067193837	6.042190161	0.02915327
DGAT2	0.0799845	6.363158814	0.029171765
PRPH2	0.06605083	6.446847536	0.029196095
LOC388789	0.090474324	10.30350319	0.029259722
CTSZ	-0.149830651	7.492410233	0.029269063
NOMO1	-0.125592172	8.564857462	0.029333088
COX4NB	-0.087816312	7.572210997	0.029333839
FARS2	0.096942919	8.978448395	0.029374651
ALG14	0.081411983	7.000833983	0.029388814
FRAT2	0.108931478	8.076813874	0.029414114
ASTN1	0.057846516	6.273434769	0.029431672

ZNF750	-0.054193738	6.159165557	0.029448352
CTNND1	0.030312166	6.254251275	0.029460846
C14ORF151	-0.069681993	6.505313814	0.029468087
C14ORF173	-0.108729308	9.158000238	0.029478661
CUL5	0.169939289	9.585971148	0.029515433
KRT126P	-0.046114223	6.346567232	0.029533202
OR11H6	0.060059963	5.984359906	0.029551559
LOC643121	0.059278415	6.171958438	0.029558803
DOCK5	-0.065534863	6.461272854	0.029583169
SUMF1	0.091838161	7.900351178	0.029621077
HMHA1	-0.086956661	6.202191553	0.029645922
PGM2L1	-0.072399046	6.446107473	0.029683146
TMEM39B	-0.072681144	7.180098174	0.029748636
TRIP11	0.113294944	8.178250785	0.029789913
TCF3	-0.072249079	6.691111061	0.029800153
PDS5B	0.066557504	7.230128612	0.029836194
FXYD4	0.07002547	5.950478606	0.029871539
NKPD1	-0.036061069	5.969321186	0.029912882
ACSL3	-0.082221932	8.177913185	0.029913119
ATMIN	0.090624805	7.292392658	0.029927666
SCNM1	-0.077584745	7.454203072	0.029970962
HCFC2	0.082999474	6.871649855	0.029987856
LOC389435	-0.118096879	13.4541187	0.030007954
SYTL3	0.134065036	7.638495838	0.030024842
KIAA1683	0.09895786	6.309359281	0.030112185
STXBP3	0.091199607	7.604530053	0.030143383
C5ORF38	0.056150327	6.126353677	0.030177614
CDH22	-0.049214144	6.1353464	0.030188205
LOC653764	0.140873901	8.149161603	0.030217434
IDI1	0.113843214	7.429379481	0.030222711
RWDD1	-0.094680212	10.71410135	0.030234452
LOC646254	-0.065995279	6.073700181	0.030257586
NSUN5C	-0.065986555	6.751572214	0.030285038
C14ORF145	-0.071498891	6.111484129	0.030316371
SHBG	0.056948408	6.367950664	0.030326897
RSPH3	0.077475283	7.402509581	0.030330308
F11R	-0.102204011	6.708760374	0.030363091
NAT6	0.089974696	7.002162768	0.030384638
LOC643265	0.051884821	6.002096036	0.03039561
CHST9	-0.190925365	6.405447225	0.030395794
LOC642486	0.060414314	6.362431814	0.030497942
RSF1	0.086422647	6.258635721	0.030506585
C6ORF105	-0.050916498	6.047481456	0.030511488
FBXL17	0.15139572	6.439670712	0.030529827
FLJ20323	-0.074861905	7.959980661	0.030546292
NRG2	0.041709429	6.119720706	0.030599261
RPS10	-0.112958707	13.29104108	0.030609926
TSP50	0.133858234	6.122277447	0.030646931
LOC643842	-0.07062505	6.043900273	0.030656124
C1QL4	-0.054611053	5.914564193	0.030686687
LRRK2	0.04449768	6.170525944	0.030699386

SEPT11	0.153400142	8.732250088	0.030717287
SATL1	0.102181282	6.509426284	0.030744274
TPST1	-0.081722678	7.125740499	0.030802227
MDGA1	0.063000581	6.417971069	0.03083302
NID2	0.145287268	6.994642022	0.030842075
LOC653275	0.049220103	6.041685865	0.030866129
FAT4	0.068714495	6.222807004	0.030879474
LOC646268	-0.047134119	5.901645871	0.030924128
HIP2	-0.072318086	7.543292755	0.030924478
LOC728468	0.059149628	6.132093504	0.030942437
IFNAR1	-0.087342514	7.971561521	0.030947276
LOC440900	0.06087155	6.289186471	0.031045974
PCDHB19P	-0.13154928	7.491846539	0.031052224
FH	0.153282713	11.76955037	0.031076714
DSN1	0.085210156	6.78054611	0.031169906
IGJ	-0.079486741	6.101345091	0.031175902
TM7SF4	-0.054297095	6.031567822	0.03118693
PHCA	-0.108710744	7.513785383	0.031193778
DIP2C	0.113375357	8.324693562	0.031223723
RAB3A	0.078047947	6.155130207	0.031239836
NUPL1	0.032367193	6.258541429	0.031245371
SGK269	0.050879245	6.042912	0.031292197
SLC22A18AS	-0.062420091	6.439405007	0.031309791
PCDHB13	-0.059717327	6.335508415	0.031342971
LOC388276	0.056612048	6.089908296	0.031363424
MUC2	0.074347947	6.14218156	0.031410287
ZNF75A	0.10107477	7.090548094	0.031414856
PPP1R13L	0.149620359	8.942164291	0.031419024
TOX	-0.061674017	6.017938795	0.031421837
POLL	0.065847	6.314485124	0.03149173
TRAPPC9	0.105362263	7.315374042	0.031565702
DLG2	0.073165717	6.527690286	0.031570501
GDPD5	0.071337892	6.501587141	0.031599613
ZNF621	0.072732251	7.716357742	0.031602108
LOC653471	-0.036351203	6.241403036	0.031616976
PIK3CA	0.07301703	6.461420806	0.031632961
RYR1	-0.043350927	6.164459422	0.031633372
LOC145853	0.121885596	9.349430486	0.031633695
ZNF146	-0.113596773	7.950749081	0.031640411
PRDM2	-0.036888843	6.332440076	0.031658484
ANKRD30B	-0.138007697	10.92701225	0.031679554
PLAC4	-0.066527397	6.03714539	0.031684664
LOC646562	0.06121872	6.173229168	0.031714732
ARHGEF7	-0.063858841	7.966577911	0.031730537
KCTD13	-0.076773476	6.799464406	0.031738588
XPNPEP3	-0.143624477	12.01850322	0.031739819
FLJ45445	-0.040524154	6.122636858	0.031753285
MTERFD3	0.043220274	6.253520763	0.031764081
PDRG1	0.091654088	7.949812294	0.03177629
SLC39A9	-0.06885832	6.318647166	0.031809408
UGCGL1	-0.082718308	6.874020658	0.03183528

ANKRD2	-0.366860285	8.748405523	0.031862008
CDCA4	-0.044296254	6.711806673	0.031881449
SLC34A3	0.056437418	6.124644134	0.031886229
FOXI2	0.059216423	6.151314833	0.031897332
FEZF2	0.068451756	5.946544941	0.031902832
RNUXA	-0.073692805	7.9267575	0.031916518
FLJ36492	0.075773531	6.239487076	0.031943971
DUSP27	0.078169189	6.388125625	0.03197173
CATSPERB	-0.057150013	6.071399306	0.032019842
METTL4	0.087553996	7.02947241	0.032024747
UBOX5	0.040667274	6.475218275	0.032061568
LOC643345	0.044897501	6.125253681	0.032072302
CYLN2	0.101336918	6.926158227	0.032102533
MAPK12	0.106521618	6.8433858	0.032103336
C7ORF28B	0.167510537	7.99841316	0.032153905
SPRR2B	0.056356516	6.141463142	0.032166562
RASGRP3	0.137183971	9.180022835	0.032196625
ZNF548	0.069749091	7.613710491	0.032200827
RNF43	0.071199994	6.435527292	0.032215328
C6ORF224	-0.060089264	6.09005312	0.032230143
LMO1	-0.118361168	6.215632971	0.032263302
ALDH8A1	-0.035520429	6.112180972	0.032266891
COL25A1	-0.038145864	5.959465342	0.032327546
C20ORF142	-0.079362996	6.754382489	0.032337624
CXORF39	-0.075268213	6.413595761	0.032403226
LGALS7	-0.192158809	8.309500605	0.032405364
UBQLNL	-0.067262475	6.248823415	0.032453031
RPL14	0.250963474	8.716707005	0.032481188
CLCA4	-0.058651988	5.98437909	0.03254065
LOC731186	0.050533465	6.109201969	0.032541048
CASP3	-0.132859347	7.831355775	0.032553576
ZNF629	0.132133636	6.881653093	0.032586264
TEX13A	-0.056503098	6.353101209	0.032586833
CLTC	-0.136259346	7.208706504	0.032612722
GDAP2	-0.049351535	6.236574042	0.03262073
DCLRE1A	0.088415562	7.294958212	0.032622259
LOC643997	-0.170267409	7.808863351	0.032704632
LOC643913	0.052711215	6.503747672	0.032706078
PARP14	-0.149541578	7.449906537	0.032714742
FLJ46210	-0.054700574	6.019927417	0.03271773
LOC158160	-0.106144799	6.980946025	0.03273838
BPNT1	0.072475319	6.681951376	0.032739219
LYSMD1	0.080277723	6.854103509	0.032777975
S1PR5	0.059997881	6.174543775	0.032781992
APEH	0.141730807	9.38871364	0.032808486
PKM2	-0.108351699	8.788986747	0.032825009
KIAA0082	0.093186496	6.314790834	0.032917593
C6ORF211	0.106664768	7.006574723	0.032939309
LOC731096	-0.107148441	12.522525	0.032951869
SNAPC4	-0.0891694	8.540197973	0.032963314
SENP8	0.089153192	6.228864178	0.033020935

LOC642502	-0.151413377	7.43735626	0.033023156
LOC646871	0.049168082	6.024427316	0.033036178
PYCR1	-0.060621385	6.189820215	0.033047036
FEN1	-0.067934181	7.429001138	0.033049076
LOC652326	0.066528651	6.514520792	0.033057873
ZNF132	0.059222825	6.392675715	0.033067823
LOC646825	0.058861538	6.08465846	0.033087035
NFIB	0.122946652	10.8398646	0.03309273
FRAT1	0.045948238	6.387731493	0.033118787
PPP1R7	0.077434452	9.000432373	0.033118846
ACSM2A	0.049626731	5.940696441	0.033163533
HHATL	0.170085075	10.4015928	0.03319224
LOC400120	-0.065788063	6.559211721	0.033204671
ZNF764	0.069486895	6.33322467	0.03322725
ANKRD20A1	0.134659625	7.192531833	0.033231624
LOC651655	-0.055151395	5.876227196	0.03326108
NOTUM	-0.038454709	6.169963617	0.033279716
LDLRAD3	-0.071512354	6.10103324	0.033298591
SERPINB4	-0.05656199	5.905729135	0.03331021
LOC152084	0.05242463	6.175846171	0.033315778
RNF123	0.095328192	7.959285186	0.03338092
ZNF521	0.124478619	6.987592709	0.033389882
GVIN1	0.13395647	7.153712203	0.033439346
SSTR5	-0.063524099	5.987592861	0.033466231
FLJ32065	0.051135794	6.153713914	0.033476493
TUBGCP4	-0.067005469	7.473655825	0.033479339
PTPLAD1	-0.063311013	7.690820988	0.033486164
RAET1G	-0.042550536	6.118211493	0.033500072
LOC732450	0.079687924	6.37006815	0.033530289
NOL3	0.118506633	8.432167099	0.033532701
MED6	0.108670259	9.857446837	0.033532805
LOC388578	0.058574906	6.088816079	0.03358889
SP140L	-0.052076876	6.298385218	0.033631677
ABLIM3	-0.159637634	8.371579328	0.033632166
CD99L2	-0.105634393	10.45400647	0.03363722
TBC1D24	0.048538991	6.233145846	0.033645686
CGRRF1	0.11357548	8.572005721	0.033648963
LOC652265	0.053922803	6.129203513	0.033661373
CTTN	-0.109604669	8.957752542	0.033695462
LOC650006	-0.052594789	5.910525644	0.033745443
EGFLAM	-0.107616926	7.771313416	0.033772661
IL17RD	-0.14312488	8.202293709	0.033822748
SYPL1	-0.108272802	9.000186024	0.033857725
CITED1	-0.073957095	6.05912863	0.03387028
QPRT	-0.185996098	8.785989158	0.033874978
FLJ20489	0.096854034	7.256021985	0.033930645
RGAG1	-0.063392824	6.269112675	0.033954461
KRTAP6-2	0.067501757	6.458431241	0.033966128
PKP4	0.097365845	8.581725957	0.033984701
MKI67IP	-0.132052538	8.543962451	0.034025734
FLJ40125	0.054199032	6.22447425	0.03407427

ZNF454	0.083403652	6.14837094	0.03408141
LOC645362	0.112002125	7.078635083	0.034082855
LOC440970	0.057717556	6.096257096	0.034098759
LOC642775	0.070553853	6.287580311	0.034104593
SEC24A	-0.110485893	6.594658713	0.034114795
ANAPC5	-0.052786466	10.0601844	0.034139835
TRIM58	-0.051988789	6.143490773	0.03420749
KCNB1	-0.068695225	6.258112011	0.03424557
C4B	0.059131659	6.071274634	0.034279336
HNRPA1L-2	0.137170392	8.707813972	0.034302335
LOC648900	-0.050486752	6.029237071	0.034314357
KRTAP10-2	0.047192537	6.222222203	0.034321812
OR10H3	0.049959245	6.021717154	0.034330952
ZNF570	0.054280238	6.309541964	0.034339568
TCEAL3	-0.094895104	10.05726445	0.034344187
LOC647656	-0.049216221	5.987182019	0.034353537
HIST1H2AE	0.0711604	6.195097292	0.034369378
CHST6	0.078128488	6.284652727	0.034374431
LOC646089	-0.053908239	6.047264146	0.034385509
LOC113230	0.082992152	6.69072467	0.034398478
TSC22D4	0.096708623	7.259710987	0.034419611
TRIO	-0.087398505	6.9747805	0.03446264
BOLA3	0.120938668	10.70791808	0.034465847
NPAS3	0.033248047	6.051962713	0.034478782
RECQL5	0.037267751	6.414259293	0.034529
BSCL2	-0.091928299	8.617748324	0.034548263
SLC13A5	-0.060918197	6.016952273	0.034637656
TRPC7	0.049165843	6.053579428	0.034639166
TRIM43	0.062861053	5.933246878	0.034648378
SCLT1	-0.063040229	6.221118614	0.034658697
SOX12	0.051594638	6.243032932	0.03468305
EPS8	-0.147786129	7.536835414	0.03471277
SOX14	0.057457697	6.326335555	0.034718507
LOC642889	-0.054908429	6.047076704	0.034719043
SH3TC2	0.032539425	6.198999622	0.034733688
OS9	-0.134242837	6.904551544	0.034743558
LOC728308	-0.055873403	6.130117635	0.034771301
LOC339742	0.031890988	6.119881564	0.034780183
MEA1	0.090864612	7.749686847	0.034791484
IFT140	0.06358208	6.49705248	0.03479357
SLC9A6	-0.053835811	6.955681986	0.034795355
MAN1B1	0.1002109	8.83137263	0.034801428
ANAPC2	-0.050753507	6.186893462	0.034875297
GPR107	-0.056853545	6.15114793	0.034905349
TMEM107	0.07674625	6.436479586	0.034923651
CTGLF1	0.057475811	6.447343541	0.034941412
CCDC37	-0.049750709	6.050174417	0.034950201
TMEM87B	-0.054288583	6.27017273	0.034955017
SLC25A12	0.140360809	9.420536703	0.034956232
LOC644112	0.053069396	6.01072089	0.035001678
SAPS1	-0.07991629	7.15819577	0.035013894

LOC643150	-0.059800581	6.023437661	0.035022517
ZNF846	0.05341933	6.099685541	0.03504865
NUDT15	0.072999705	7.377105013	0.035068813
LOC728895	-0.068660309	6.179509891	0.035098545
C16ORF45	0.129388383	7.490263256	0.03515245
HN1	-0.111743048	6.77323367	0.03518001
LOC652246	0.056517911	5.909565548	0.035188196
SLC29A3	0.0671668	6.306754909	0.035190884
CHCHD8	-0.089536028	7.401541263	0.035210702
ST5	-0.068451768	7.122965842	0.035229685
KRT16	0.052684609	6.371772763	0.035257814
APOB48R	-0.055614588	6.063837924	0.035267974
FAM91A1	-0.059325752	6.266315736	0.035276541
KIF20B	0.069267945	6.923612021	0.035276992
NAGPA	0.077465335	7.117463288	0.035296057
LRIG1	0.125841366	9.726636254	0.03531697
ISY1	0.059493795	8.199549244	0.035336801
LOC732425	-0.08171248	6.606553275	0.035395221
EMILIN2	-0.240688763	9.227399949	0.035399702
FERMT1	0.070697678	6.111707797	0.035410492
DNAJB5	-0.137793493	7.629081185	0.035412216
LOC646743	0.074310974	6.061692821	0.035422192
LOC401280	0.052545545	6.238173418	0.035428953
DHX8	0.072296301	6.593352954	0.035432899
CT47.7	0.039325245	6.146984864	0.035436734
MLL5	0.049448846	6.766471595	0.035438144
MOSPD3	-0.052720392	6.724788534	0.035457573
UNC13A	0.035770491	6.284874987	0.035479987
GSR	0.072188631	7.339288186	0.035487652
MSL3L1	-0.062223436	6.735698771	0.035559838
SORT1	0.184529538	10.3178887	0.035571532
COL15A1	-0.160170119	9.789873191	0.035634895
RFXAP	0.042303158	6.575515272	0.035636919
GLMN	0.07426822	7.191923533	0.035644562
LOC388969	0.087572932	7.149547365	0.03568721
CCK	0.054579311	5.966204775	0.035708582
HCG18	-0.058023343	6.142959949	0.035782186
ZNF295	-0.101204363	6.996661685	0.035789409
TTLL3	0.052083812	6.623444287	0.035831714
MTUS1	-0.092775537	7.451652742	0.035833539
FIBIN	0.095533268	6.49460741	0.035867319
SEC24C	-0.078181557	8.766680615	0.035872595
PRPH	-0.060965571	6.543239594	0.035873374
PRKAR1B	0.111571366	6.522929986	0.035883176
ECAT1	-0.061968119	6.011974014	0.035896317
PPP4R1	-0.074752589	8.833122189	0.035920307
RBPMS	0.068279492	7.515209717	0.035945221
FKBP8	0.076031839	6.716365066	0.035965239
LOC645895	-0.149110938	13.1606745	0.035966545
LOC646324	0.069484503	5.943528277	0.035973933
AGPAT4	0.050368362	6.450996145	0.035980723

BAIAP2L2	0.058207361	6.459173782	0.036005732
TRIM38	-0.078971887	6.64482539	0.036019619
KCNH6	-0.173412358	10.72245028	0.036040143
CPLX2	0.033517521	6.050391922	0.036057026
LOC646519	-0.057140487	6.252173019	0.03610195
LRRC37B	0.05637681	6.604548631	0.036102982
C1QTNF5	0.16253466	8.430288242	0.036112642
MYCT1	0.07322894	6.61148717	0.036117936
RFPL4B	-0.040110315	5.994588045	0.036129126
LOC441124	-0.056369129	6.430961622	0.036160284
PANX2	-0.055021641	6.036541043	0.036181754
LOC653741	0.052016493	6.115289711	0.036187183
LOC441584	-0.056331617	5.974111083	0.036188253
PTPLB	0.120573971	8.954787345	0.03621347
NEIL1	0.056422621	6.500090208	0.036214337
LOC646210	0.054939609	6.207408666	0.03622714
PRDM6	0.052998877	6.324839902	0.036242748
VEGFC	0.134076592	7.14470734	0.036258765
CGA	-0.077118002	6.104625048	0.036338003
LOC646561	0.046817415	6.066986476	0.036377139
CRTC2	-0.085482683	6.461093876	0.03654851
CHMP7	-0.098552775	6.869552764	0.036605465
PLCG2	-0.13496882	7.950757798	0.036631336
LOC647536	-0.049551086	6.019767277	0.036635703
C11ORF73	0.104245424	8.151163627	0.036681547
PSORS1C1	-0.071406057	6.782982325	0.036706765
FIT1	0.18840888	11.2798301	0.03672733
IL18	-0.128648131	12.57683762	0.036729413
KIAA1543	-0.037687646	5.996850533	0.036744136
C1ORF112	-0.061669414	6.387797955	0.036761045
SIRPG	0.079417199	7.22009537	0.036776746
LOC642342	-0.10962872	6.647273961	0.036872178
PGAP1	0.053057681	6.40956583	0.036875376
FLJ21075	0.053115641	6.091158711	0.036889776
ZNF665	0.059926447	5.872244476	0.036947575
ARHGEF2	0.11037244	9.466824168	0.036956541
ZNF511	0.09047205	9.635659199	0.036996294
LOC642302	0.052960725	6.063110635	0.037064714
CASC2	-0.049681988	6.612742916	0.037105851
LOC284948	-0.05428586	6.075852028	0.037120434
SLC17A7	0.055702245	6.303880886	0.037125481
RFC4	0.065089848	7.77437188	0.037163621
C11ORF39	0.056305865	5.998561268	0.037249966
RBL2	0.078631211	8.635476945	0.037254814
ANKRD36B	0.059463253	6.733295834	0.037258815
LOC653232	-0.098273064	12.47561254	0.037260755
CIAPIN1	-0.078327197	7.725226484	0.037262096
CHD7	-0.078431986	7.991001364	0.037263055
KDELR3	0.116785132	7.33478996	0.037271811
ASAP3	-0.179719972	8.435957371	0.037320917
EPC2	0.092990008	6.416048047	0.037355034

AMIGO2	0.05580077	6.389557734	0.037357517
C7ORF27	-0.109786597	8.708362052	0.03737689
MT1B	0.053833002	6.132166097	0.037436136
SULF2	0.090862722	7.577065175	0.037437108
MED21	0.160816769	6.904752363	0.037456075
C9ORF163	0.054641256	6.08993681	0.037488264
NCKIPSD	-0.084449689	7.11647161	0.037501563
CCDC15	0.04408835	6.266136448	0.037505349
LOC650898	0.094110227	6.431462321	0.037515114
FLJ44606	0.049239285	6.357375116	0.037518726
TRAPPC5	-0.123250542	9.697739979	0.037569795
HLA-A29.1	-0.610021456	8.525485034	0.037587211
FAM177B	0.05448792	5.971594034	0.037622026
ZBTB44	0.093018056	6.980485022	0.03767926
EIF1AX	-0.181584763	8.064872769	0.037686305
LOC654202	0.065906521	6.1234588	0.037718944
MLLT6	-0.087635873	9.200646384	0.037788865
GOLGA7	0.06853389	8.393028886	0.037806053
VCX-C	0.066042485	6.27387619	0.03786686
BAZ1B	0.070594302	7.36229943	0.037869905
LOC646781	0.044434096	5.937964103	0.03791153
ZACN	0.078948855	5.914212957	0.037984324
TYSND1	-0.067119819	7.126118025	0.03800741
HGFAC	-0.062679981	5.988749359	0.038014537
NIPSNAP3B	0.141869424	6.429974886	0.038050777
OR51T1	0.074974557	6.623762281	0.038054841
HBB	-0.402073721	11.08023742	0.038068767
SF3B3	-0.11132623	8.792860238	0.038126105
C20ORF111	0.110355742	9.221597988	0.038130342
LOC154907	0.052015695	5.91240218	0.038154765
BANF1	0.097979701	9.880986225	0.03815578
SIN3A	0.09158274	8.664214086	0.038182637
NR4A2	-0.214217366	6.521122338	0.038187995
CCDC78	-0.042251103	5.998675995	0.038200027
TNFRSF10D	-0.07607736	5.999821642	0.038258975
ELMO2	-0.042889128	7.398352946	0.038263791
LOC643714	0.048293683	6.091264855	0.038295149
PTPRQ	-0.054339699	6.291052192	0.03845234
NDST4	0.051591876	6.025841561	0.038455889
NFAT5	-0.102136697	6.751477915	0.038534425
LOC645893	0.062117127	5.929969987	0.038586132
ETF1	-0.111003269	9.501192977	0.038641781
LOC646123	0.079140317	6.369108986	0.038664284
LOC401895	0.056064486	6.186936093	0.038725993
RDH8	0.046307932	6.225683077	0.038735064
LOC653629	-0.051546209	6.277954565	0.038740646
ADAM10	0.072237976	6.917856634	0.038790507
CAV2	-0.127744738	9.60677837	0.038815915
LUZP4	-0.048228579	5.911344384	0.038880343
SEMA3C	0.189642738	7.978974831	0.038921871
PDE10A	-0.040497967	6.039474159	0.038982143

ZNF781	0.038433345	6.183891871	0.039016801
DNM2	-0.087154269	7.787483646	0.039035381
CD46	0.09774533	8.603118757	0.039084115
LOC652287	0.054622292	6.21959707	0.039093309
C14ORF129	0.073580633	7.435487185	0.039231249
TTLL11	-0.062517952	6.323217173	0.039252199
SEDLP	-0.106558357	7.89676841	0.039253618
LOC643145	-0.063022523	5.901844467	0.039334811
LOC392364	-0.050645508	6.063736292	0.039341444
LOC643596	0.051547745	5.996201832	0.039386114
CLDN1	0.070053249	6.418110037	0.039396595
TFAM	0.102122962	8.692785902	0.039404096
RNMTL1	0.097362073	9.459597585	0.039412296
VAMP7	-0.122615023	8.893392481	0.039412369
SLC44A1	0.105784668	9.508831886	0.039530601
ZNF524	-0.112462272	7.730301117	0.039552302
OR1A1	0.052537158	5.978626095	0.039585988
CCL17	-0.057425258	5.982311577	0.039635371
PAF1	0.133500621	9.262251959	0.039644945
IPO5	-0.129101654	8.08902999	0.039650262
LOC653204	-0.066330289	6.169344427	0.039718259
MGC70863	-0.059394291	6.332450711	0.039719651
LOC729264	0.076484332	6.330499297	0.039740902
ZNF434	0.077511662	7.402235733	0.039744031
NPB	0.057577225	6.039859306	0.039778955
LOC653333	0.060129496	6.283011	0.039783647
CCDC134	-0.054976244	6.231137027	0.039805231
ATP6V1B2	-0.110107391	10.63572989	0.039829997
LOC653498	0.052278131	6.061618822	0.039887599
GNA14	-0.066918612	6.27013714	0.039910244
ZCCHC6	0.080774316	6.619859019	0.039935803
BAI2	0.061143866	6.066963049	0.039961969
ZNF675	0.050932001	6.025611117	0.039993851
ETV7	-0.054712685	6.084599181	0.040003052
DNASE1	0.050760679	6.129111805	0.040005449
NIT1	0.107320065	7.332204361	0.0400172
LOC641699	-0.05034814	5.938524608	0.040034716
SNORD35A	-0.061469476	6.138904606	0.040047945
GPX4	-0.087008239	11.9751005	0.040155283
SPA17	-0.09092809	6.876202513	0.040156579
ABCC10	0.07936067	6.465225933	0.040166822
NUDT14	-0.130120336	9.152521325	0.040201591
SAT2	0.109022287	8.732550419	0.040205449
PARL	0.065794046	9.926610842	0.040226653
EPHA8	0.044355087	6.298896146	0.04022941
KRT72	0.056228582	6.246776319	0.040266934
LOC643262	0.050358313	6.002622765	0.040294023
SYTL1	0.053259939	6.080638377	0.04029596
FAM53B	-0.141848436	7.754928196	0.040303962
AMY1B	0.065715991	6.603235371	0.040344855
TTYH1	-0.054941779	6.199820747	0.040369204

DNAH9	0.032440101	6.025900546	0.040422549
SMURF2	-0.059722713	6.228665536	0.040451361
SLCO1A2	0.026227381	5.983539986	0.040462968
PDCL	0.06477575	6.538194661	0.040504887
HIST1H2BI	-0.047904088	5.890788085	0.040579579
CEP152	-0.040850844	6.144151221	0.040595931
LOC647839	0.05549906	5.969883245	0.040624926
SFRS17A	-0.08509461	8.775605805	0.040626939
THOC7	0.10673173	11.230263	0.040629521
SNORA32	-0.058718886	6.216828929	0.040687408
FZD4	-0.096050838	9.433715569	0.040695662
LOC654163	0.055484216	5.970490729	0.04075239
ASXL3	0.049203941	6.020597379	0.040783737
LOC647816	0.04632415	6.012015368	0.040848674
LOC645875	0.047260157	6.112368868	0.04090084
LOC652694	-0.083921011	6.335645053	0.040927428
LOC647129	0.045349424	6.040086697	0.040942721
TBC1D14	0.096413987	9.065932483	0.040965518
KIAA1602	0.113860218	7.750353412	0.041006648
LOC441177	0.051397861	5.918325584	0.041036729
SYCE1	0.033584267	6.135853506	0.041047253
GPR114	0.058553127	6.270257495	0.041097286
PAPLN	0.079213956	6.257814995	0.041101658
EFEMP2	0.109281535	10.19564201	0.04111303
LOC90624	0.133698998	7.596155589	0.041127514
LOC643000	-0.054355215	6.004549834	0.041198895
LOC650914	-0.058264822	5.941393304	0.041210696
LOC651730	0.055357003	5.991989686	0.041216939
ZNF320	0.089930094	6.605696575	0.041261356
LOC642698	0.052895552	5.983154379	0.041282912
ABCA13	-0.058888813	5.991995591	0.041293311
PATE	0.054131127	6.302991405	0.04130951
TOMM7	0.076830087	13.2820507	0.041324892
ITPRIPL2	-0.286746236	8.916086442	0.041330883
LOC646723	-0.13746987	6.89108258	0.041340388
UBE1DC1	0.06516629	7.088520176	0.041530406
SFMBT1	0.054743662	6.908824993	0.041546966
RBED1	0.078500867	6.761618698	0.041572776
LOC145820	-0.065763451	6.435307673	0.041596416
CHGA	-0.053008244	6.08152798	0.041642438
LOC285047	-0.052966818	6.011184947	0.041678982
NRG3	0.055690319	6.100841068	0.041692845
ATP5H	0.067222166	13.16361257	0.041769712
LOC643334	0.046021655	6.050870227	0.041791172
CXCL9	-0.232906083	6.56615765	0.041793992
LOC389293	0.173886422	8.78503798	0.041823217
ADIG	-0.055475426	6.233555868	0.041831599
SCN3A	0.090155365	8.290059928	0.041883066
LOC650526	-0.053085422	6.144007947	0.041888303
CCNF	-0.070881074	6.459697448	0.041891152
RPL10A	-0.097929322	12.26156566	0.04193899

SPPL2B	0.035926807	6.202323552	0.041953884
LOC650548	0.05280365	6.021564858	0.041962391
TAF5L	-0.055354858	6.103158668	0.042016827
PCGF1	0.092651619	7.516736314	0.042017279
ATP13A3	-0.062919224	6.319055395	0.042053896
LOC653086	-0.138197984	8.559776828	0.042055338
ODF2L	0.047129729	6.46135295	0.042100053
CBWD5	-0.108493113	7.438162433	0.042155586
SDR-O	0.042921545	5.988293306	0.042191731
ETV1	0.073269579	6.241329059	0.042197549
C16ORF33	-0.109380319	10.45423309	0.042210343
ZNF207	-0.081572874	9.543377829	0.042237693
GNGT2	-0.049677687	6.270233764	0.042242684
LOC643740	-0.049127345	6.242203051	0.042256401
GFRA2	-0.079033388	7.468224682	0.042285347
LOC647954	-0.085275035	6.688525563	0.042301714
TIPRL	0.067844714	7.848436553	0.042341555
KIAA1279	0.103098174	8.677270229	0.042359047
PARS2	0.064817648	6.425786077	0.042380548
KLHL26	0.054509641	6.578592953	0.042391368
C2ORF32	-0.077693474	6.800154725	0.042394433
C20ORF100	-0.148519606	7.769136262	0.042434827
SIDT1	-0.053388223	6.033004703	0.042459795
PEX3	0.065750659	6.21286729	0.042515899
LOC389983	0.053422577	6.045938751	0.042552014
TSPAN11	-0.035998051	6.00983613	0.042562085
SNORD64	-0.055236282	6.010521796	0.04256735
CMTM5	0.050714815	6.08075599	0.042599718
LOC728825	0.077988147	6.645775121	0.042623933
LOC646111	0.049612655	6.004411959	0.042627969
RORB	-0.1442631	6.451496612	0.042673577
DQX1	0.056995325	6.085790286	0.042675758
C14ORF79	0.070283417	6.662464631	0.042681343
NAE1	0.077453935	8.527482776	0.042684867
FPGT	0.050345494	6.04894897	0.042701371
ADSL	-0.103989449	10.5620253	0.04272905
ERBB3	-0.047257886	6.35713708	0.042768661
TOX3	-0.048429826	5.948057706	0.042804833
C2ORF40	0.155814671	6.943838899	0.042822983
MPFL	0.057776812	6.421587973	0.042840428
PRKG1	0.040965344	6.107031833	0.04288168
ZNF419	0.105161788	7.724392901	0.042955846
LOC441440	-0.047680883	5.967370185	0.042982129
SYT5	0.049811762	6.089979137	0.042992661
JARID1C	-0.056794473	6.284563724	0.043010411
TCHP	0.071299558	6.690059695	0.043163435
RGP1	0.057709241	6.415524128	0.043198862
MMEL1	-0.03261923	6.050667697	0.043213234
PSMD4	0.09954694	9.800506729	0.043236301
MAP1B	-0.220196525	7.711816553	0.043236497
LOC649456	0.059150367	6.381982839	0.043237393

SDPR	0.160965797	10.07634536	0.043268821
NAG18	-0.09075076	13.17050824	0.043269568
LOC730012	-0.051815867	6.058723112	0.04327152
DRAP1	-0.115370799	9.93751314	0.043276913
TMEM84	0.053652146	6.129886468	0.043303287
ARL4D	-0.05427547	6.302994697	0.043326895
DKFZP586I1420	-0.059980662	6.750147617	0.043343251
FLJ43752	0.050249218	6.188584358	0.043443187
LOC729310	0.055583736	6.227906615	0.043478002
RBP4	-0.061584874	6.051927593	0.043508221
LRRC31	0.060138518	6.303500054	0.043608075
CNPY4	0.049189265	6.784201228	0.043644734
FLJ45831	0.049621011	6.015703057	0.043684521
LOC644457	0.049385265	6.168370612	0.043704554
ZNF460	0.050049915	6.510936288	0.04371818
LOC643453	-0.055214042	6.192893731	0.043729554
LOC554175	0.036990688	6.069191262	0.043776277
DOPEY2	-0.096052869	7.947232551	0.043778618
ATP5J	0.104341109	13.19775651	0.043806861
RSBN1	0.099275132	8.665779012	0.043826471
G6PC	0.038879071	6.019421503	0.04383645
METTL8	-0.056751439	6.223256941	0.043842728
LOC652204	0.047796735	6.068220511	0.043848115
N4BP2	-0.132306548	8.501391005	0.043922313
LOC651986	0.05210701	5.925312078	0.043940237
LOC442382	-0.054091815	5.966840158	0.043986539
SIGLEC12	-0.034647399	6.174858116	0.043994763
FZD5	-0.059882284	6.398571332	0.044005142
NCAPD2	0.080330723	7.343818228	0.044011608
LOC643382	0.056695939	6.191476677	0.04401182
SIX3	0.046342057	6.11782711	0.044065823
LOC441046	0.057381018	6.406063461	0.044091384
UBE2V2	-0.1026093	8.03230532	0.044105016
MOSPD1	0.133487195	7.107668376	0.044125667
NUDT18	0.089560603	7.149651462	0.044138991
LOC651850	0.045795697	6.126024933	0.044275683
MX2	-0.154676642	7.318402186	0.044284302
RHOC	-0.099429655	10.87952926	0.044293883
FCRLB	0.071592175	6.605092204	0.044338997
TSSC1	-0.090971986	8.02759608	0.044343092
MSH5	-0.02642513	6.241076018	0.044352497
HIST1H2BH	-0.047207402	6.146261189	0.04440173
C6ORF54	-0.065430328	6.1455591	0.044428701
AGXT2L2	-0.05479484	7.212974523	0.044471026
LOC728352	0.076093149	6.004171724	0.044513044
PES1	-0.12167492	6.522009616	0.044537643
KRT6B	0.031784994	6.091548661	0.04455959
KIAA0256	0.088633818	8.653688976	0.044615699
MCMDC1	0.052714796	6.231002181	0.044678925
SNORD56B	0.050204431	6.038649601	0.044697702
AKT2	0.065315375	6.279424473	0.044704811

LOC652857	-0.04731261	5.921216012	0.044792807
FLYWCH2	0.078046715	7.464774742	0.044798464
P15RS	0.095843756	10.08000148	0.04481561
OR10G7	0.056213886	6.184583575	0.044840248
LOC390259	0.047549671	6.074698348	0.044842503
ZNF35	-0.066174537	7.22931407	0.04485341
C18ORF32	0.189403747	7.525785512	0.044907835
LOC441996	0.053774257	6.085915948	0.044952105
FLJ20628	0.100408657	7.458318463	0.044967408
EIF2C4	0.055966796	6.747855523	0.04497922
LOC652046	0.04820537	6.106244185	0.045007065
KRT3	0.053668828	6.268109167	0.045028405
ICT1	0.159897839	8.796228097	0.045045533
HMGB1	0.110600396	7.175508223	0.045056012
CACNA2D4	-0.04469817	6.22974981	0.045119446
CTNNB1	0.078223266	7.061059059	0.045171799
SPATA2	-0.059380316	6.673487269	0.045212765
ATP6V0D1	-0.105130616	9.206650008	0.045215585
MCF2	0.093281245	6.244704413	0.045239126
GOLGA8B	0.153388008	8.387893119	0.045263508
PPIA	-0.071813399	6.889146701	0.045264293
C4ORF19	0.054317749	6.440621052	0.045277302
GPT2	-0.148863374	8.25607002	0.045328747
GOLGA3	0.097841259	9.825135013	0.04538992
BCS1L	0.075149132	8.648978012	0.045390451
DENND3	-0.089426613	6.945236635	0.045427355
PCYOX1L	0.080762961	6.179106386	0.04546068
CMYA3	0.206474537	7.059048568	0.045513743
HP1BP3	-0.065912693	6.534400425	0.045537131
ARSK	0.061571415	6.482317146	0.045657327
LOC651745	0.052962996	6.062579973	0.045698039
LOC645364	0.065201484	5.90523479	0.045699417
FAM153B	-0.112963704	7.42070279	0.045707603
CAMKK2	-0.061380244	6.166145124	0.045750798
PLEKHA3	0.10326037	7.378455852	0.045753136
RPL28	-0.073923817	6.696441787	0.045844343
CUTA	0.078537743	9.779001112	0.045869097
F8A3	-0.056111283	6.113297584	0.045886246
DCDC2	0.051204344	5.981127327	0.045919689
LOC153684	0.048813271	6.243629864	0.045959196
CSRP1	-0.160958601	9.148994703	0.046020377
LOC653197	0.053924487	6.090464019	0.046177836
MX1	-0.215476609	10.11955358	0.04618008
TLX2	-0.039826663	5.953542929	0.046252233
OR2M7	-0.048810073	5.94820974	0.046375997
SAMHD1	-0.056457498	6.10543012	0.04642069
D2HGDH	0.126751114	9.101884046	0.046458634
TSHZ1	0.084587133	7.856500659	0.046469131
CACNA1E	-0.049230232	6.021508789	0.046536571
RPS9	-0.118465969	11.95263807	0.046539745
ZNF572	0.048976942	6.103776472	0.046553764

CLEC9A	0.034665546	6.087541476	0.046556731
VPS25	0.087695225	8.081285573	0.046563232
PFDN5	0.101614966	11.76297341	0.046569236
OR10Z1	-0.044416003	6.025620747	0.046661767
ZNF142	0.13446829	7.090997906	0.046692459
LOC643686	0.049268501	6.127378906	0.046737106
LOC645752	0.058895292	6.120310836	0.046753093
CXXC4	0.123106301	6.396584575	0.046768372
BRI3BP	-0.046780639	6.507584279	0.046788532
LOC642666	-0.041553253	6.047901589	0.04679161
CNFN	0.063148665	6.549526599	0.046826718
LOC645535	0.051934536	6.391920513	0.046897354
COX6BP1	-0.051726007	6.105631754	0.046915079
ZNF791	0.102051451	7.652233304	0.046941143
PIGB	0.066722184	6.866835497	0.046962223
TMEM48	-0.058936881	6.345253096	0.046988893
BAT2	-0.091765802	8.062597401	0.046988994
AMZ1	0.044536432	6.191192424	0.047037468
NKRF	0.069234118	7.237855173	0.047056852
CC2D2B	0.049093875	6.104524896	0.047119383
C11ORF80	0.099307381	6.768488183	0.047150944
RPL39	-0.10815991	12.86439678	0.047205605
TSPAN7	-0.121054622	7.971161184	0.047207397
LOC643430	-0.054688135	6.013576661	0.047212567
MARCH8	0.034228262	6.079023637	0.04725477
RAB42	-0.057625722	6.208418339	0.04725769
KIAA1033	0.109404343	6.31751431	0.047259449
AP2A1	-0.040378222	6.598560454	0.047267057
NPAS2	-0.117967368	7.739097325	0.047273916
MADCAM1	0.035751121	6.110396072	0.047306927
EIF3IP1	0.047491885	6.255744107	0.047322962
SLC2A13	-0.056036802	6.066336053	0.047351172
FAM114A2	0.054052213	6.755030223	0.047363841
FASTKD2	0.090148645	7.934169324	0.047370409
LOC646585	0.056016815	6.082869923	0.047421346
SASH1	-0.098429801	8.753680488	0.047426901
SLC4A8	0.036497411	6.144410484	0.047467049
PRPSAP1	-0.089928819	9.629798285	0.047533283
SFRS8	0.073011985	7.368532854	0.047537415
TMEM77	0.074396511	7.457820945	0.047537492
LOC388237	-0.034295424	6.201824519	0.047559444
ATP6V0B	-0.094086952	9.847525528	0.047566561
LOC286208	0.126526293	7.178506039	0.047568614
PPIH	0.120676951	8.173506839	0.04759419
TMEM176B	0.142102189	7.00336944	0.047630002
APOA4	-0.046968157	5.919339593	0.047642566
LOC440055	-0.139363611	10.30988467	0.047670625
ARSJ	0.073082135	6.330437565	0.047723861
CD99	-0.16288262	8.88660287	0.047732833
EXOD1	0.052013967	6.184443198	0.047733042
TMEM117	0.103826795	7.114410325	0.047775893

C20ORF86	0.059643521	6.20096479	0.04781617
LOC731102	0.060653505	6.180897451	0.047840195
C6ORF114	-0.072261292	6.056127532	0.047881649
LOC649639	0.077607275	6.522520293	0.047891007
TOLLIP	0.073983701	7.600879651	0.048015089
FAM103A1	-0.081039772	7.76619775	0.048067524
SLC12A6	0.040363111	6.14718762	0.048156648
CCDC124	-0.079254402	7.409385816	0.048158345
FLJ10490	0.051409037	6.185486995	0.048195762
PHF6	0.03223934	6.085523911	0.048251107
TRAM1L1	0.060239293	6.177727251	0.048271364
TFIP11	0.06089422	7.539772596	0.048334267
LOC654103	-0.092309626	8.475984674	0.048356117
PRY	-0.044571311	6.02747122	0.048357731
ARMCX4	0.057992534	6.306682825	0.048362248
NSUN2	-0.10055781	9.88829526	0.048422643
SNX17	-0.080010424	9.714665878	0.04842795
HMGN3	0.051249196	6.325732071	0.048454434
OR10A5	-0.052669948	5.826301121	0.048507413
CPNE6	0.050895238	6.127632854	0.04867718
LOC642953	0.044816419	6.05903017	0.04871474
THAP3	0.055988661	6.20720739	0.048733391
C16ORF14	0.160982671	9.100210428	0.048882501
LOC643138	0.05452541	6.138542049	0.048888173
TAS2R38	-0.055149546	6.263455132	0.04891266
CD207	-0.054609979	6.02221469	0.04892702
RNF4	-0.060308271	7.436739966	0.048936671
MAN2A1	-0.061596991	6.589457387	0.049047418
CCDC24	0.072935902	6.439207841	0.049071325
LOC645602	0.055217099	5.951623593	0.049103029
FAM39DP	-0.070392947	8.09356472	0.049142993
HLA-DPB1	-0.114066502	6.592576958	0.049148175
CRYL1	0.106959676	7.617493869	0.049159041
FAM131B	0.072033441	6.420414559	0.049185664
KHK	-0.028878755	6.165360086	0.049189126
GAS2L2	0.039487224	6.075563824	0.049213938
OR5M10	0.040749915	6.115365116	0.049261639
FSTL1	-0.177678266	9.587451685	0.049277399
C21ORF42	0.045900504	6.181839307	0.049280931
GALNT10	-0.042138679	6.519072895	0.049291785
LOC126536	-0.044901209	6.182029617	0.049493194
C20ORF20	-0.086684231	9.247891814	0.049510942
FLJ41170	-0.050180274	6.041608033	0.04951463
NPC2	-0.101690247	12.31905501	0.049520477
LOC647006	-0.046552779	6.035542948	0.049553899
PXMP2	-0.143125243	9.053026999	0.049561628
PPIL6	-0.0669605	6.308454224	0.049607083
ESPNL	0.167535044	6.364960662	0.049631247
KRT17	0.063509035	6.098368136	0.049651079
LOC440737	-0.115653111	11.14524364	0.049655558
LOC643951	-0.044393264	6.041943055	0.049703374

STH	0.047793364	6.37884059	0.04980546
LOC650321	0.12377906	6.515270486	0.049809421
COX5A	-0.078500564	13.81251154	0.049939396
ZNF574	0.049494328	6.289751304	0.049978276
LMF2	-0.072656488	7.864514222	0.049978618
C19ORF2	0.067235228	10.07479197	0.050003981
ZNF771	0.034584399	6.190626033	0.050037381
LOC652586	-0.04649733	5.972004121	0.050051713
LOC644094	0.056401921	6.287503127	0.05005537
MTERFD2	0.053971832	6.497402387	0.050119544
MS4A8B	0.050875505	6.02902595	0.050122695
LOC653689	-0.05510621	6.214314274	0.050182418
SAMD9	0.119209891	7.303373737	0.0501892
NELL2	0.051227954	6.04729554	0.050288144
CDC20	-0.084288262	6.514857972	0.050294665
LBX1	0.033103108	5.959102009	0.050453494
LOC653643	0.051560685	6.172525702	0.050480741
LIMS2	0.12603133	9.070867297	0.050482832
CLCN5	-0.048803513	6.135034955	0.050528185
CCDC40	0.035743664	6.102626059	0.050549387
ITGA8	0.095375039	6.199846345	0.050637576
LOC645914	-0.047772249	5.887432879	0.050658449
C17ORF59	0.072818914	6.74553415	0.050676663
ZFPL1	-0.060727026	7.058420835	0.050705738
LOC641804	-0.051805966	6.206229424	0.050766244
LOC647691	-0.080920775	6.642236359	0.05076778
LOC375010	0.047478198	5.931750678	0.050787
VPS37A	0.080381868	8.635371681	0.050847796
ART4	-0.076167622	6.823901089	0.050850995
LOC641741	0.044246073	6.05446557	0.050856701
ARL14	0.057413997	6.219134775	0.050863766
SEZ6L2	0.035515537	6.107457657	0.050870045
WDR85	0.057087685	6.537963613	0.050872134
RABL5	0.058491638	6.184540768	0.050933292
LOC728248	0.048635815	5.934360819	0.05094715
LOC644744	0.049135919	6.020447015	0.050978182
LOC644624	0.028561894	6.164295256	0.051116661
SFPQ	-0.134190836	6.665829472	0.05112743
YME1L1	0.108482452	8.724167363	0.05115064
MAGED1	0.073612968	9.754371077	0.05120233
NUDT21	0.079404968	7.467415515	0.051238691
LOC652814	0.048175445	6.120215193	0.051305014
OPN1SW	-0.051589509	6.131927194	0.051347636
KPNA3	0.095291085	9.085330111	0.051356433
MRPS28	0.116027225	9.904114167	0.051365995
GAA	0.091888792	7.443950486	0.051383602
C9ORF130	-0.108628555	7.791749657	0.051383724
LMO3	0.081434859	6.309884275	0.051393352
ARFGEF2	-0.054965421	6.60941844	0.051394945
SSBP3	-0.060271865	6.673980241	0.051424172
CCDC72	0.085102345	11.54458838	0.051462019

ADCY7	-0.059255475	6.672509142	0.051497431
TTC24	0.054165738	6.080673844	0.05151299
CD164	-0.17410189	6.970254259	0.051597122
ETS1	-0.124824981	10.01882037	0.051661496
PDE9A	-0.038865583	6.378040887	0.051746165
C19ORF31	-0.098692496	13.49864192	0.051787766
LRIG2	-0.057848965	6.922770336	0.051839788
RHOF	-0.071066731	6.287797734	0.051851748
TMEM68	0.052445396	6.316851941	0.051860998
TRIM36	0.027495661	6.148248314	0.051936683
MRPL24	-0.069116502	9.89181229	0.051958887
SEPX1	0.104204633	10.4169595	0.052000874
ADCYAP1R1	0.046056358	6.027571002	0.052048874
POU5F1	0.040558268	6.015350073	0.052075288
LOC643817	0.047658031	6.154555407	0.052127303
CAPN14	0.054533926	6.146865325	0.052133948
INO80B	-0.061618752	6.807300788	0.052162194
LOC641815	-0.047233896	5.960574756	0.052177344
NEB	0.187495823	6.485486214	0.052179344
PTMA	-0.273664374	8.151996365	0.052199446
METRNL	-0.094934303	8.318880118	0.05222734
DNAH10	0.032711423	6.069959681	0.052292609
FLJ32679	0.027694373	6.087798491	0.052312336
LOC402160	-0.038279832	6.039552626	0.05231489
LOC642432	0.073267822	6.299510979	0.052395869
FLJ22662	0.092432083	9.194870167	0.052408631
NSBP1	0.042470202	6.301388429	0.052422349
CD247	0.096116065	6.649837832	0.052444304
CTSO	0.160449699	8.21415487	0.052468446
HAGHL	-0.063783792	6.527549292	0.052472461
LOC441873	0.06377912	6.026622952	0.052521398
CIDEA	-0.065619954	6.128209437	0.052533039
SLC30A7	-0.089525496	7.775696704	0.052560158
SAE1	0.084752126	11.14241039	0.052568764
SCGB1D4	-0.049242202	5.960302241	0.052726257
DAPK2	0.10323461	7.066467756	0.052748054
SNX27	-0.08810094	9.694196516	0.0528119
ACVR1	-0.104060644	9.323075659	0.052873238
BRIP1	0.06048393	6.239736611	0.05287351
C9ORF64	0.074145863	7.04714951	0.052883643
SYMPK	0.107436575	6.891716181	0.052932822
CSNK2A1	-0.080289387	7.698397591	0.052950252
FBXW9	-0.070674246	7.021001198	0.052973831
NDUFA5	0.122526499	6.430087157	0.053035911
EPGN	-0.073202547	7.000045675	0.053039954
NHS	0.030379237	6.195188936	0.053057765
RBM18	0.093554824	8.143520575	0.053065031
SNORA66	-0.053509366	5.992863157	0.053067319
ZMAT3	-0.131465503	10.6412226	0.053098062
CNTN5	0.032924176	5.987293766	0.053269696
VPS28	-0.080475481	9.200183951	0.05333542

EYA3	-0.031713725	6.102714672	0.053379718
LOC646358	0.051530018	6.220199685	0.053383753
NUP54	0.079022664	7.806259216	0.053440283
LOC285941	-0.054181396	6.233775936	0.053467544
LOC643687	-0.052376513	5.968295844	0.053476278
IL9R	-0.046767456	5.996273608	0.053488813
LOC653145	-0.025930161	5.969278798	0.053515186
HIPK3	0.063029708	6.55131187	0.053540906
C21ORF45	0.065100519	6.256031848	0.053560983
NR1H3	-0.087661611	7.903699573	0.05364598
C14ORF4	-0.141895965	8.742405355	0.05367942
IL25	0.04339329	6.125552758	0.053686057
LOC389895	0.04813253	6.125624952	0.053709298
GEMIN6	-0.077375141	7.800872713	0.053729286
LOC730740	0.091276143	11.20743043	0.053764359
BTN3A2	-0.095313661	6.887452672	0.053770469
OR2L3	0.053026881	6.266806147	0.053775113
SCN10A	-0.052691263	6.039221491	0.053821593
WISP2	-0.175922923	7.010752811	0.053839594
LOC644276	-0.04734572	6.226186176	0.053880719
KEAP1	-0.071186326	8.380771967	0.053889433
ADAMTS8	-0.09568755	6.173742182	0.053889906
LOC652152	0.047956642	6.095266821	0.05393728
SMN1	0.043791573	6.306621988	0.053997497
LOC400304	0.152939726	7.157726455	0.054059414
CGGBP1	-0.082542865	8.814556268	0.054070018
FBXO10	0.038310035	6.243520577	0.054070714
CGB7	0.040052738	6.074958029	0.054109183
KIR2DL1	-0.037136168	6.052459988	0.054129581
ZNF721	0.105729083	8.031516417	0.054160417
LOC643329	0.046262644	6.033526347	0.054161203
VAV3	-0.065014289	6.371574298	0.054229621
SEPT4	-0.096540397	7.942010131	0.054275213
SMARCAL1	0.074007088	7.717134632	0.054322639
LOC653886	-0.04647586	6.166799785	0.054330733
AHSA1	-0.129088839	9.339420494	0.054344711
ATOH1	-0.05150352	6.053381063	0.054359244
ENTPD3	0.042915988	6.177102396	0.054394664
ST7L	0.0456593	6.410636939	0.054432145
LOC654000	0.038232241	6.379240339	0.054577169
LRRC67	0.060958677	6.041502475	0.054585465
MSLN	-0.06770434	6.019668961	0.05462436
SUMO1	0.073924454	6.308819088	0.054644675
C1ORF102	0.053363982	6.503279052	0.054652112
PSMB5	-0.104543281	10.28426199	0.05469588
AUTS2	0.09186036	8.307126774	0.054700751
WDSOF1	-0.0856526	6.480314615	0.054718199
COL4A2	-0.185505509	8.027044495	0.054799562
STAM2	0.074763022	7.017826604	0.054810149
ZNF280D	0.052529894	6.898162061	0.054821537
ATP2B4	-0.120528795	9.789722452	0.054834997

FLJ43763	0.045869858	5.966591548	0.054854554
CARD9	-0.070448999	8.098439815	0.05490388
LOC646129	-0.048001204	6.020434484	0.054965743
LGALS9B	-0.04080767	6.282725603	0.055000304
LOC727927	-0.04881654	6.260272601	0.055047664
RBMY2EP	-0.038627079	6.026017583	0.055051275
MGC52000	-0.110230759	8.210475238	0.055060453
FLJ21865	0.078752247	7.387213213	0.055118842
ALDH9A1	0.095687665	12.00995115	0.055160369
LOC653107	0.023904729	6.06047321	0.055169472
MED13	0.040731627	6.26600323	0.055259393
C6ORF192	0.089525005	7.350462656	0.055355145
TSPYL5	-0.100970671	8.389083084	0.055371361
GRIN2D	0.05765522	5.975942875	0.055426564
NCF1B	0.044579712	5.94446076	0.05548469
FOXM1	-0.039995128	6.025780765	0.055602795
CLN6	-0.056175596	6.352362274	0.055637227
OR2A2	0.048582511	6.111733832	0.05564457
LOC650325	-0.049275698	6.104722081	0.05568852
RPLP0P2	0.050325496	6.039561663	0.055803192
TMEM31	-0.049023528	6.229010132	0.055828109
LOC642821	0.050802734	6.080326124	0.055829988
PFKP	-0.144298175	10.58417637	0.055836548
ARID3A	-0.081191927	7.15117755	0.055864091
ZNF818	0.053063644	6.004265921	0.055868123
DCHS1	0.070621186	6.814879923	0.05591498
RBMX	0.103585046	8.9005933	0.055915725
ZDHHC13	0.051495567	6.604696914	0.056012532
GLB1L	0.058071814	6.393393446	0.056054881
RSU1	-0.098232143	8.378657976	0.056080734
USP41	0.030863536	6.216433338	0.056152341
LOC645927	0.045206349	6.106486688	0.056217144
THSD1P	0.042938617	6.144405375	0.056218554
LOC642757	0.046238261	5.989373972	0.056222453
GPR50	0.05300662	6.032139903	0.056225815
TSPY1	0.038984908	5.96147061	0.056228304
C9ORF96	0.052242737	6.081442087	0.056242865
MGC57346	0.064142294	6.962577536	0.056273606
LRRC44	0.044785507	6.031212375	0.056279933
TRIM63	-0.203476587	9.878936849	0.056344445
LOC728005	0.037692364	6.054142073	0.056419349
CDK9	-0.100894141	7.715640572	0.056433955
SEC14L4	0.065747146	6.048757198	0.056503504
CYYR1	-0.09511152	8.528473615	0.056524239
CHRFAM7A	-0.029286066	6.043087089	0.056554523
PNPLA8	-0.115183082	7.79843826	0.056611771
TOM1L1	0.055681936	6.169629868	0.056674317
TCP10L	0.056951459	6.818284385	0.056712224
AAK1	0.092383989	7.017296939	0.056775849
PRODH2	-0.058043003	5.989502524	0.056806038
GABRP	0.049788408	6.229032513	0.056833656

MINPP1	0.084588355	6.859540456	0.056879319
PDCD5	-0.099276799	9.813112639	0.056886521
CNOT3	0.085969484	7.055907893	0.056906841
NOL7	-0.150961774	11.43677793	0.056919516
SUSD4	-0.098710884	6.195573937	0.056945856
LOC643109	-0.051996901	6.112101673	0.056971476
SERINC3	0.066857634	8.213899004	0.057036263
SALL1	-0.052450057	5.971455374	0.057040894
HES5	0.115926992	6.8043744	0.057043621
ALKBH8	0.058678631	6.486703246	0.057136601
TBX4	-0.049340496	5.951952954	0.057158203
C19ORF6	-0.093185146	7.900415889	0.057163153
RCAN2	0.112883905	12.03502966	0.057190759
EXOSC7	0.112389473	8.688802337	0.057199814
POP1	-0.073730945	6.295267967	0.057215828
LOC390712	-0.045245175	5.903865532	0.05731455
HYDIN	0.014755761	6.080702406	0.05735381
LOC645351	0.058301441	6.258504689	0.057356403
ACSL1	0.191026473	10.17022915	0.057365191
LOC644403	0.046679069	6.184323631	0.057424283
KIN	0.056850727	6.203280351	0.05751429
ZNF28	0.038903408	6.282650033	0.057551571
KIAA1377	0.047742397	6.10934684	0.057560113
LOC652565	-0.052431201	6.450310138	0.057588
LASS4	0.078266032	6.599294224	0.057630204
NRXN2	0.057260512	6.375334697	0.057652184
DENND2A	0.101308071	8.334343277	0.057660936
BRUNOL6	0.058411968	6.475793932	0.057661925
DGCR2	0.101216626	8.11004872	0.057675676
LOC643222	0.042309128	6.074627749	0.057678968
OR6W1P	0.052836515	5.990571569	0.057681781
DMRT3	-0.047784657	5.951665079	0.057733602
MESDC2	-0.047703419	6.179136042	0.057740444
ATF1	0.080840516	6.833688383	0.057750086
APBB1	0.038801919	6.086828211	0.057756186
EPHB3	0.060391208	6.589638106	0.057763482
KRTAP10-4	0.048880725	6.066679886	0.057767826
CEP70	0.073034577	6.917701139	0.0577715
NKAPL	0.038398757	6.216149005	0.057804285
C1ORF89	0.063424893	6.547346728	0.057806591
HTR6	0.071834395	6.223797507	0.057819354
PELI3	0.063315145	6.290576552	0.057845123
MS4A13	-0.032831104	6.006301731	0.057887602
SLCO1C1	-0.035762141	6.069452949	0.057935827
ZNF559	0.100170686	8.011624984	0.057990952
DGKQ	-0.069079917	7.256630573	0.058034479
CDC73	0.048608469	6.357339081	0.058048051
ITIH2	0.049419858	6.095263914	0.058106526
FLJ33544	0.045695903	6.182687555	0.058121373
SGSM3	-0.070029629	6.795949819	0.058150797
LOC645848	0.051631898	6.269163973	0.058188408

PDP2	-0.038741501	6.307034989	0.058196748
LOC647571	0.042055059	6.097789637	0.058283411
C10ORF73	0.143840248	7.515167264	0.058318574
LOC256374	0.044848133	5.941075438	0.058343267
LOC730456	0.074465411	5.974416811	0.05837545
NTRK1	-0.03037303	6.180821417	0.058376314
SERPINB6	-0.079784987	11.21754782	0.058421878
CCDC18	0.04433142	6.229499195	0.058428813
GK2	0.08999424	7.711943198	0.058429928
LOC729047	-0.047602814	5.941076382	0.058434753
SLC6A5	0.045244834	5.953878579	0.058445535
GORASP2	-0.085274238	9.218230523	0.058469172
ALKBH6	0.060816938	7.565971025	0.058469753
EFCBP1	0.115803309	7.053383458	0.058518685
KIR2DS3	0.045539643	6.021663553	0.058541292
EIF2B5	-0.073604893	9.008044204	0.05854182
LOC647340	0.06485842	13.11611214	0.058565862
GTF2H4	0.09624723	7.652834005	0.058572861
FAM29A	0.042213269	6.050643322	0.058587787
LOC646817	0.065215476	6.253591934	0.058649998
MAN2B2	0.119708172	8.82659604	0.058666521
RSPRY1	-0.112754861	8.654367822	0.058695273
ZNF212	-0.079179152	7.518001095	0.058771005
LOC647974	-0.046376868	5.866557997	0.0588242
C10ORF53	-0.026848209	6.025516931	0.058893588
CXORF40B	-0.065067197	7.36930424	0.058910638
C15ORF29	0.088748712	6.560696712	0.058942247
DHRS9	-0.209842732	6.738639466	0.058988959
NLRP14	-0.042121641	5.934852873	0.059037003
FAM86C	0.052945673	6.761656885	0.059042493
CPXCR1	0.033756034	6.095754876	0.059115219
HIST2H2AB	0.052856522	6.164848235	0.059158077
CCDC44	0.072350945	10.20109136	0.059170131
SLC9A7	-0.050029653	6.29723874	0.059191311
ZUFSP	-0.061292756	6.946872815	0.059220651
C1ORF86	-0.07092415	8.032945812	0.05923482
BCAS2	0.077298428	6.393448103	0.05925475
ERAL1	-0.098240785	9.017742874	0.059335417
PIAS3	-0.057515784	6.765182827	0.059351097
KIRREL3	-0.052314927	5.993578917	0.059400346
LOC285733	-0.054575616	6.124361083	0.05943873
ZNF483	-0.082296778	7.058839782	0.059444826
LOC729715	0.047485728	6.258629188	0.059448265
ATG16L2	-0.073911599	6.588590265	0.059453106
LOC645218	0.033521403	6.117685467	0.059526922
LOC442251	-0.046791854	5.991895723	0.059530291
ABCC13	-0.027952485	6.197744833	0.059585809
FLJ34503	0.072255824	6.226759992	0.059634931
PPAPDC1B	-0.066187767	6.74112963	0.059658872
LOC649923	-0.088500932	6.070805048	0.059675636
ZNF222	0.061097282	6.289438787	0.059721536

ABCC5	-0.127384713	8.425704731	0.059737386
ZFP64	0.033479261	6.370497637	0.059789093
DNAJA3	-0.089569114	10.6187537	0.05980371
LOC645048	0.043527015	5.956507353	0.059825832
LOC730262	-0.032791335	6.071901221	0.059843018
RNF128	0.029915301	6.337814891	0.059853352
FLJ10916	0.266574604	8.977507095	0.059899761
GNE	-0.134315821	8.378800901	0.059931517
RBMY1E	-0.035644531	6.028573865	0.059961315
KIAA1539	-0.103888733	7.83197704	0.059974881
C16ORF84	-0.049065965	6.336349093	0.060031061
PPFIA1	-0.045172276	7.870537173	0.060031953
LOC652637	0.051445347	6.213685453	0.060079166
EYA2	-0.025890412	6.128959434	0.060090655
LOC142937	0.048783223	5.979964517	0.060130981
CELSR2	0.076638175	6.555337335	0.06019295
LOC642602	0.040262835	6.145445932	0.060271419
SLITRK6	0.047773054	6.136290455	0.060277827
SKIV2L	-0.109334741	7.473594807	0.060328235
CEP27	-0.139678818	8.663152135	0.060386246
WDR51B	0.091991005	7.062233102	0.06041358
C10ORF116	-0.147892191	10.83000459	0.060425118
P76	0.099680913	7.587409661	0.06050102
AMBN	0.023970581	6.0357746	0.060508339
KRI1	0.055246001	6.636981668	0.06056544
LOC283932	0.075390789	6.8652606	0.060569934
LOC646009	0.049152348	6.00533223	0.06057059
CCR3	-0.034705293	5.960696482	0.060610163
PRR19	0.047718306	6.179644694	0.060670971
SERPINB2	-0.037886848	6.016731691	0.060797876
LOC644026	0.060002676	6.016255371	0.060852063
C11ORF57	0.06778657	6.796182735	0.060857651
LOC387820	-0.095260273	9.314302169	0.060931976
GALNT1	-0.059310086	6.740823558	0.060945772
CD300LF	-0.054622342	6.106688448	0.060977846
GPR108	-0.07426512	7.049348967	0.060995367
ARL16	-0.155389028	9.227827756	0.061011381
H1FX	0.120482225	8.488946094	0.061022026
OR51V1	-0.039632077	5.879738534	0.061129978
KIAA2018	0.053136173	5.994923224	0.061145828
ANKS1B	0.036903283	6.207838221	0.061249924
FTSJ2	0.081856787	6.532576389	0.061285309
ALPP	-0.14208972	11.21252573	0.061290414
MPPED1	0.037298551	6.056587685	0.061318172
PRKAR2A	-0.091274766	7.152580304	0.061344362
RAD50	0.056656821	6.728063134	0.061348704
CROT	0.068212896	7.02111867	0.061350976
RBL1	0.036316113	6.215986583	0.061374416
RFC2	-0.037693513	6.545797574	0.061378391
LOC642809	-0.064857616	6.124443508	0.061449778
PRKD2	-0.059083553	6.501457647	0.061484339

LOC401068	-0.042518384	6.096174723	0.061513644
TMEM30A	-0.095738871	7.698574168	0.061540198
OR4F5	0.032702713	6.040392029	0.061548736
LOC644413	0.048855837	5.963291894	0.061556292
MRPS30	0.102099865	9.801723732	0.061596796
RPL21	-0.182755577	10.98676737	0.061616407
MET	-0.094509419	6.623750436	0.061647184
ERC1	0.063433597	6.410285738	0.061648453
RC3H2	0.078542054	7.251816914	0.061674261
HYAL4	0.049074104	6.14801966	0.061741711
RPL31	0.155525246	11.32764853	0.061763894
PQLC3	0.089450651	8.208070214	0.061847684
TYW1	0.074914826	7.357541955	0.061872623
RSHL3	-0.042866758	6.156998355	0.061910157
SMEK3P	-0.043679509	5.975443394	0.061932353
PEX10	0.043540479	6.197618771	0.061957944
HRG	0.033281343	6.001864923	0.061966562
SUZ12P	0.024571422	6.103230107	0.061988912
LOC649260	-0.070755239	6.590616909	0.062031293
DPT	0.215779973	8.008550952	0.062039348
FAM177A1	-0.091770944	11.63369591	0.062071975
DMRTA1	0.060138229	6.166828006	0.062078419
LOC642104	0.047497056	5.941399568	0.062082153
NECAB2	0.05357238	6.260958211	0.062110223
LOC732172	0.057763509	6.591600965	0.06213022
FAM169B	0.043722674	5.967556085	0.062132929
LUZP5	0.046216275	6.007539958	0.062171838
LOC391092	0.04930087	6.037074133	0.062234114
LOC652616	-0.0467178	6.126747796	0.06224176
HACE1	0.068672461	6.504251998	0.062265558
LOC652344	0.043932243	6.023473443	0.062271823
LOC647200	0.047340156	6.080786261	0.062317345
IER5L	0.072698251	6.706750257	0.062326978
LOC132706	0.049210664	6.074912576	0.062332267
LOC651017	-0.048857536	5.948710764	0.06245791
MRAP	-0.048205154	6.061752849	0.062475647
LOC645336	-0.044848329	6.054313767	0.062503889
FLJ11151	-0.058972406	6.351867936	0.062580924
FKBP1B	-0.046920006	6.11329868	0.062591525
LOC651686	-0.044606687	6.112773944	0.06261103
CCDC84	0.085648993	8.101213515	0.062649412
POLR2K	0.070452752	6.821675668	0.062692011
LOC388564	0.06160572	6.292209416	0.062706716
LOC641992	-0.102709251	6.901702981	0.062753157
ZNF3	0.039215895	6.414984701	0.062817237
GRIK4	0.047347573	6.007889951	0.062818742
H19	-0.215165273	11.18964567	0.062849134
SLIT3	-0.104863202	6.602592365	0.062866846
BOAT	-0.04829066	6.390545259	0.062879804
RTP1	0.050992903	6.225739268	0.062892159
STRN3	0.128478477	9.283514846	0.062926412

SS18L1	0.086273454	6.791616227	0.062928844
WFDC11	0.053679549	5.985006201	0.063065512
PLXNA4B	0.055660699	5.943135053	0.0630743
PPP1R16A	-0.124619078	9.735668595	0.063083443
KANK2	0.152070127	9.34730312	0.063087912
MRGPRX1	0.040461921	5.914687404	0.063141524
TREM1	-0.072390684	6.20704574	0.063181059
FLJ45256	-0.041574092	6.236795656	0.063196845
IZUMO1	-0.04177393	6.026476855	0.063204183
LOC643550	-0.059747663	6.200631275	0.063212113
LOC653238	0.021951609	6.006606428	0.063237173
FTHL8	-0.190121478	10.6148154	0.063261418
UBXN11	-0.029532452	6.387005487	0.063284145
FLJ10324	0.057066463	6.558443789	0.063383283
SULT1C3	0.046322523	6.188666015	0.063428521
TNKS	0.044943621	6.191721053	0.063442303
LOC642212	0.050031814	5.944972426	0.063451816
DEFB131	0.050963116	6.476733303	0.063477691
CPLX1	-0.144907204	6.913343101	0.063507842
LOC731432	-0.075577157	6.34631014	0.063508291
C1ORF132	-0.066471007	6.012268841	0.063547328
ANXA4	0.074177363	6.372070107	0.063592161
LOC645968	0.152449659	7.77313821	0.063672866
ATP2B1	0.055737164	6.380073227	0.06373524
ALDH1A1	0.135380539	7.78252655	0.063761028
DUXAP3	-0.145192417	9.956020917	0.063806429
LOC645197	0.037134603	6.155842028	0.06381343
KIAA1622	-0.050977564	5.978507256	0.063827416
KITLG	0.090400344	6.740273379	0.063866135
CCDC144A	0.031624232	5.988348569	0.063871755
VPS18	0.074446	7.435773926	0.063907049
GLRA1	-0.051481129	6.01347095	0.063924056
BAT4	0.060533526	6.586954902	0.063928379
FAM149A	0.103711308	7.06831633	0.063945332
LOC645436	-0.114765525	10.70791353	0.063957854
LOC642424	-0.050784683	6.001835559	0.063959703
SNORD116-2	-0.048156692	6.049536244	0.063980481
VLDLR	0.099421786	8.925323304	0.064070338
HES1	-0.11374886	6.881277292	0.064093747
CAPZA1	-0.099427526	6.557181137	0.064232913
USP6NL	0.045100119	6.793005627	0.064235324
LOC344382	0.049544789	6.230107447	0.064239196
COQ10B	-0.078355972	10.42710806	0.064279633
C15ORF33	0.039946225	5.990113582	0.06431496
SLC10A3	-0.077863564	8.720791414	0.064342956
LOC652783	-0.054140474	5.952438922	0.064380367
LCT	-0.037434684	5.951906275	0.064472287
MAGEB4	0.041500226	5.896460195	0.064513848
LOC653564	0.049047285	5.928155588	0.064519147
PRIM1	0.040829872	6.55986566	0.064523432
LOC645367	-0.034828206	6.038425284	0.064540995

AHR	-0.111920854	10.48585961	0.06455131
LOC653034	-0.050403476	6.438556962	0.064557262
LOC649987	0.043753975	5.999029494	0.064594423
LOC644577	-0.051331231	6.00190909	0.064633331
LOC644343	0.111714485	6.664469953	0.064702864
PRDM5	0.047058691	6.080132803	0.064725876
VCX	0.062965762	6.095771437	0.064800988
LOC388494	-0.045055514	6.020996359	0.064836242
MTM1	0.06190862	7.134206147	0.064876363
S1PR2	0.048263649	6.068871536	0.064889385
LOC641806	0.046850841	6.37405479	0.064909034
HEY1	-0.113758624	8.582333414	0.064921131
GINS2	0.081042022	6.560405806	0.064933421
TSSK1B	0.047502567	6.248427728	0.065113428
GIYD2	-0.043076899	6.060906148	0.065117483
LAMA3	0.031826418	6.351681806	0.065119006
HOXC4	-0.032754587	6.072843051	0.065123331
LOC650137	-0.037114818	6.01292592	0.065125689
WDR25	0.096823696	6.776461177	0.065130571
GCDH	0.078879847	7.883852201	0.065130602
FOXI1	0.03532462	6.205523659	0.065140349
SYNPO	0.065911179	6.059428888	0.065157934
EPHA7	0.043812787	5.97310633	0.065277388
SPPL3	0.07047931	6.606903666	0.065306288
ATP6V0E1	0.062447574	11.40339875	0.065387297
LOC255809	-0.044602754	6.154674323	0.065396798
LONRF3	-0.035811297	6.053797703	0.065457067
ARF5	-0.083752304	9.017337175	0.065463997
C10ORF115	-0.049646022	5.818732783	0.065472163
LOC649632	-0.041316499	6.018181499	0.06557049
KCNMB4	0.097313186	7.038831853	0.065615261
GNLY	0.062110834	6.293878699	0.065686555
LOC645582	0.04828074	6.072363979	0.065827737
OR2J3	0.040925089	6.014427758	0.06583066
LOC646783	0.041287099	6.31531231	0.065869521
AFG3L2	0.196945981	10.41290052	0.065871787
LOC652373	0.046179088	6.091115972	0.066016585
RIT1	0.078595245	6.882265472	0.066184746
CRB3	-0.028704144	6.180937163	0.066227018
ZC3H18	-0.061264675	6.72211644	0.066239601
LOC441655	0.047445881	6.303473685	0.066278827
TBC1D8B	-0.025328323	6.063965448	0.066297972
NKX6-3	0.067030762	6.370273507	0.066319381
MATN1	-0.054404871	6.017242424	0.066329219
SAMD1	-0.066233509	6.719001844	0.066333067
LOC441511	0.085615582	6.850213675	0.066358939
DHRS4	0.087331502	9.426509814	0.066373957
PIAS1	0.07572213	8.141103363	0.066384514
ATP11B	0.093723209	7.528735589	0.066464199
FLJ46380	0.096443535	6.65213147	0.066485964
HRH4	0.042786091	6.122298143	0.066493388

RRAD	-0.24270882	9.31512256	0.066522114
LOC643002	0.043618784	5.997779527	0.066545234
DKFZP761E198	-0.057259341	6.253064789	0.06663428
SLC25A26	-0.10420683	7.336572936	0.066650449
MAPKAPK3	-0.104829417	12.22908684	0.066706187
COPS2	0.103708611	6.865242182	0.066736982
LASS5	-0.078850366	7.210492419	0.066782369
LOC643043	0.032512156	6.125235858	0.066784607
DSCR6	0.057917503	6.070482837	0.066821503
CD1B	-0.049093877	5.984709713	0.066839154
OR9G4	0.041661538	5.95019094	0.066852165
MOBP	0.025427672	6.014091006	0.066882897
LOC647384	-0.033909866	6.049242752	0.066955957
LOC650363	-0.043479215	6.13209671	0.066969027
LOC645669	0.042748657	6.07820426	0.067055636
LOC646746	-0.033060584	6.37824608	0.067058836
FLJ26850	0.058149436	6.312419646	0.067129491
TRIM50	0.034736331	6.081845734	0.067151094
SPRR4	0.047882206	6.152520683	0.067152049
ILK	0.061228108	10.29037868	0.067184817
PPID	0.071822473	6.627973807	0.067196515
GALNS	-0.068796657	6.482010903	0.06722249
CCDC65	0.053462898	5.861817626	0.067263114
ZC3H10	0.097206609	6.82913949	0.067340915
PELI2	0.071346025	6.765445137	0.06739248
FLJ45224	0.089902539	6.563370963	0.06740219
WDR20	-0.052708417	7.249453323	0.067432299
LOC554223	-0.030055587	6.098535173	0.067514418
FLJ42291	-0.049483742	6.027706948	0.067576936
LOC284064	0.043000588	5.985549901	0.06760307
ZXDC	-0.043978804	6.784489124	0.06763911
LOC402508	0.047026186	6.057387887	0.067666843
C21ORF67	0.076919761	6.362120554	0.067736547
NKAP	0.061197535	6.611798889	0.067739281
WHDC1L1	0.024079631	6.070522872	0.067755655
SNX5	0.052668724	7.395014271	0.067788618
C4ORF44	0.033784997	6.143858344	0.067842458
CPZ	0.030384794	6.072335755	0.067850817
LOC387941	0.046434254	6.113282234	0.067879807
LOC645711	0.040644382	6.144062456	0.067916998
DGCR5	-0.04699442	6.030283326	0.06792259
CNOT6L	0.039391114	6.277630685	0.068016054
CA12	-0.037547862	6.145026925	0.068043672
LOC338797	-0.047564026	5.934194859	0.068133199
CAMSAP1	0.071847224	8.400484991	0.06813474
LOC148766	-0.043409852	6.069658146	0.06814252
FABP7	0.062306571	6.242849975	0.068144095
LOC646309	-0.048540318	6.119459361	0.068158224
SLC34A2	0.033384821	6.064950649	0.068158931
PDDC1	0.076173115	6.790171152	0.068162892
GRIA2	0.046128624	5.995188271	0.068184161

CEP250	0.039008865	6.331588281	0.068235573
RNF175	-0.07622338	6.318497928	0.068252079
TMC7	0.053532232	6.375002004	0.068273755
INO80C	0.062577917	8.49972234	0.06830303
GOSR2	-0.076459357	7.180655493	0.068315167
INTS4	0.043923975	7.005247243	0.068324226
NRIP2	-0.058228684	6.567123878	0.068367222
CXORF1	0.044388869	6.156732527	0.068391444
TLE6	0.082556958	6.892917341	0.068393138
ANKRD55	-0.050777184	5.970830553	0.06843435
HMG2L1	0.043639866	6.395130376	0.068482313
TBC1D3G	-0.067653086	6.417000904	0.068495798
DSCR3	-0.109253227	8.765602422	0.068521424
DMC1	-0.14647525	10.28902639	0.068525142
C18ORF2	0.039191668	6.043119365	0.068615328
RNF26	0.073962914	6.990310391	0.068648949
LOC284821	-0.133260519	12.72955941	0.068701084
ZDHHC21	0.05327587	6.315596776	0.068813246
PRAMEF16	0.049783511	6.03329817	0.068819396
PHLDB1	-0.093550496	10.17454597	0.068821135
RCL1	-0.071263953	6.973831734	0.068849678
CASC1	0.080971514	6.291629917	0.068959827
C6ORF160	0.16669279	10.15533299	0.068983113
CBLN2	-0.083773534	6.164433398	0.068985028
SMC5	-0.051187534	5.938222741	0.068986784
SDHALP1	-0.076167731	8.019861714	0.069008835
KIF21B	0.034379694	6.062131913	0.069035273
IGSF2	-0.043273937	6.2406311	0.069051458
BXDC5	0.080774501	8.475127455	0.069106447
WDR22	0.095960815	10.73263818	0.069144287
LOC120376	-0.051248672	6.011082838	0.069147248
SPRN	0.046011705	6.39807544	0.069159219
C15ORF56	0.049480161	6.021186066	0.069174769
LOC402694	-0.149438135	11.44839693	0.069183269
FAM21A	0.061646211	6.337092941	0.069300868
CDA	-0.100995381	6.951683747	0.069304273
PCDHGC4	0.034821122	6.063564661	0.069327011
C20ORF135	-0.04552094	5.986774608	0.069382152
TMEM188	-0.093605936	8.857792195	0.06938716
RPS6KC1	-0.047705026	7.014173413	0.069388897
INTS1	-0.068717913	8.222901222	0.069455352
CAPN11	-0.057868696	6.279025686	0.069537572
GTPBP4	-0.121510824	9.815410285	0.069545585
LOC400968	-0.044229849	5.893219188	0.069561542
QRICH1	-0.067095385	8.086397337	0.069563119
FAM19A4	0.031162187	6.212239405	0.069601094
RBM7	0.112892458	7.147750431	0.069603745
ANXA9	0.051349082	5.971072134	0.06960855
CSNK1G1	-0.044259278	6.585768456	0.069628273
MRPL12	0.099247182	10.21163262	0.069660476
CLRN1	0.050893009	6.5303679	0.069704622

PTCD3	0.045804446	6.274485928	0.069706135
HNRNPC	-0.071749101	7.854234437	0.069830597
SRF	-0.094070585	10.92512999	0.069850623
PITPNA	-0.072470131	7.781092141	0.069852993
B4GALT6	-0.059846071	6.241288268	0.069857386
INTS10	-0.065288953	8.726977986	0.069891021
BAALC	-0.044101889	6.347630478	0.069896082
LOC652392	0.046963916	6.071141016	0.069920857
SHANK1	0.049085527	6.006935653	0.069943756
LOC648638	0.091925485	8.094547396	0.069989109
MTHFD1	0.067638293	7.069930845	0.070040269
LOC641772	0.029096245	6.09337584	0.070078513
KRTAP10-7	-0.045752745	5.917677385	0.070094894
CWC15	0.075597938	10.10120269	0.070117039
RPA1	0.104655046	9.257688295	0.070120402
CUGBP2	0.064775624	6.957399448	0.070202365
RPPH1	-0.057893466	6.358421146	0.070233839
LOC255374	-0.045013577	6.191799729	0.070237379
DBR1	0.063041221	6.55832443	0.070266008
LOC649404	0.059480903	5.911979707	0.070267562
LOC552891	0.052070527	6.392683676	0.070286635
COG2	0.061203792	8.771693968	0.070314714
ZNF324B	0.054529609	6.21544013	0.07031988
AAAS	0.058762705	6.880830554	0.070349338
ITGA1	-0.088814766	6.858228144	0.070403093
C7ORF11	0.061068082	7.173871488	0.070425949
NRAP	-0.14663064	10.35760273	0.070467513
PPIL5	-0.033345694	6.555920074	0.070521045
LOC647103	0.043668672	6.125590052	0.070566491
PRSS8	0.072889145	6.031104177	0.070673581
RNF44	0.084819621	7.128548619	0.070676672
LOC200420	0.056876197	6.110817431	0.070678345
CDH1	-0.041374151	6.008330552	0.070681431
LOC647919	0.046586135	6.138720971	0.07073418
GRIK3	-0.044329041	6.111001711	0.07075803
LOC653127	-0.049608521	6.142818503	0.070770543
LRRC15	-0.087618426	7.246749701	0.070818476
VPS72	0.072156855	7.995900595	0.070852665
SNX14	0.06004588	7.311662702	0.070919615
CACNA1D	0.050795884	6.346177574	0.070928876
LOC647121	-0.043195246	6.077769447	0.071024045
CLEC3B	0.160566009	9.210296417	0.071136556
LOC652251	0.048840872	6.006310826	0.071142485
DUX4C	0.045188301	6.143678786	0.071145096
NUP107	-0.077346803	8.302219458	0.071215856
TESC	-0.204089	9.973883859	0.071228169
EXOC3L	-0.049035419	6.188789857	0.07125016
HYMAI	0.041924813	6.054475528	0.071266348
LOC401915	0.049841731	6.007065389	0.071279251
CXORF24	0.039402681	6.059916853	0.071294849
LOC650008	-0.048318409	6.189297514	0.071304391

SULT1A1	-0.122339816	8.870670415	0.071305019
SCNN1B	0.055234195	6.340539235	0.071377899
VAPA	0.049082068	6.78673916	0.071428383
FLJ40722	-0.122047474	7.680588968	0.071431707
LOC390378	-0.053878943	6.003151828	0.071531908
SIGLEC11	-0.045860952	6.093246635	0.071536515
TUFM	0.074956226	10.47877372	0.07166485
POLR2J	-0.090157973	6.92592877	0.071672363
H2BFWT	0.054767272	5.962793314	0.071719896
DNASE1L1	0.069067552	7.380547999	0.071735526
NBPF3	0.037361962	6.540564157	0.071766934
ZNF568	0.035125652	6.179979709	0.071801405
FGF2	0.055034628	6.591363309	0.071837994
HSPE1	-0.111932073	10.95787495	0.07184062
CSMD1	0.033001444	6.17241332	0.071870013
CHES1	0.142421917	10.0016784	0.071889307
RGNEF	0.027870493	6.175989331	0.071991749
SNX33	0.067095692	6.996721738	0.072094884
TBC1D22B	-0.068288615	6.831320232	0.072103148
LOC642154	0.041575928	6.281738207	0.072131315
GAL3ST4	-0.084025659	6.149208809	0.072221541
ZNF414	0.049226575	6.473571384	0.072246351
ATG9A	-0.079279189	7.737455349	0.072257305
WWOX	-0.03279698	6.156341903	0.07227105
FAM43A	0.193250293	9.388789007	0.072278183
LOC730426	0.041560204	6.11948974	0.072294509
LOC644655	0.042680959	5.922347059	0.072297977
LOC91661	0.061173839	6.294870769	0.072377967
HSH2D	0.048085575	6.015428453	0.07242629
SEPT10	0.051995151	6.754270955	0.072449679
C14ORF126	0.050821795	6.199213232	0.072459935
LOC143666	-0.046526621	6.526211034	0.072472761
LOC645032	0.049485697	6.119152493	0.072473739
NFIX	0.132965491	10.39229183	0.072483444
FTHL3P	-0.152463322	9.599009089	0.072510647
WNT5A	-0.053274408	6.186629473	0.072513727
OR6N2	0.050931122	6.027912433	0.072522203
ATXN1	0.095244448	7.759766409	0.072549857
SFT2D3	0.055645314	6.677207835	0.072564114
LOC648851	0.051605583	5.912127333	0.072635683
FAM7A1	0.061009213	6.192912842	0.072637782
LOC642387	-0.039829287	5.943270514	0.072688721
C3ORF62	0.046363419	6.583888012	0.072732353
SPON1	-0.193155356	8.261118689	0.072734465
PLAC8L1	-0.046542672	6.148831804	0.072748499
CAMK2N1	-0.140094268	8.929084365	0.072776203
GBA3	0.032235957	5.959476115	0.072836
SNORD25	0.049154716	6.2595155	0.072851317
SNORA25	-0.106577077	6.703116284	0.072854368
RPL35	-0.119978303	12.8507892	0.072862342
DYNLL2	0.098173356	9.256123374	0.072875572

PLA2R1	0.036266032	6.205705243	0.072899591
APH1A	-0.081482526	8.235991586	0.072904477
PLSCR2	-0.042568062	6.080640796	0.072928874
ADAMTSL4	-0.069641898	6.741477879	0.072930218
UBE2N	0.108350685	10.01257649	0.072969621
HCG27	0.053992534	6.346266345	0.072995679
STAT5B	0.075295251	7.757881522	0.073012424
LOC390367	0.042563526	6.164171095	0.073164428
NHEJ1	0.044973811	6.147424941	0.073238254
DSE	-0.079810304	7.247421683	0.073254185
FAM5B	0.046049396	6.238937157	0.07329687
LOC647166	0.048694896	5.974495571	0.073308629
LOC646507	0.047597879	5.982597269	0.073326178
CLCN3	-0.066384985	8.017389159	0.073373331
CNTN2	-0.046646286	6.024202931	0.073375249
PADI3	0.044020345	6.029068442	0.073398217
LOC642855	0.058249849	6.222393632	0.073401419
WDR89	0.033893319	6.180986951	0.073532542
MTIF3	0.141321684	10.3801065	0.073534412
ZFP28	0.043502081	6.207663719	0.073543775
GPHB5	0.046498597	6.308037032	0.073727781
GALNT3	-0.104044333	7.648125406	0.073730673
C10ORF12	0.05194137	6.259621567	0.073750538
LOC653766	0.053145919	6.117044318	0.073788175
PIWIL2	-0.028114698	6.004585749	0.073838408
UBR4	-0.088221354	9.391531199	0.07392076
FLJ10357	0.056268055	6.491412389	0.074008938
FASTKD5	-0.078921227	9.250559749	0.074012112
GTF2IRD2B	-0.090856224	8.461604891	0.074027046
LOC390466	0.100825871	8.228686307	0.074067388
FLJ43980	-0.051455848	5.915643795	0.074123671
LEREPO4	-0.044578721	5.988773788	0.074148689
LOC652126	-0.047916777	6.028869664	0.074206098
DUOXA2	-0.042373991	6.091116552	0.074221616
C8ORF22	-0.046395147	5.98331201	0.074230062
PDE8A	0.057242426	7.31970424	0.074274868
TRPM4	0.139249907	8.164850645	0.074276404
COQ2	0.107262854	9.654509377	0.074350627
TDRD7	0.078486374	8.345931656	0.074375569
C13ORF18	-0.032398462	6.086325473	0.074432293
LDHC	0.082942865	6.256312397	0.074462539
CDH17	0.081123015	6.097396677	0.074471322
TUSC5	-0.047046171	6.068337581	0.074474571
ACSL6	0.023118051	6.049022933	0.074513413
LIN37	0.051396577	6.704268332	0.074585336
USP53	0.112958629	7.174223008	0.074614645
RPS15A	-0.086984608	12.32330219	0.074616927
C6ORF103	-0.041711378	6.068517534	0.074624988
GRIA1	0.046331813	6.104133469	0.074630318
TELO2	-0.06607571	7.059990208	0.074704267
SLC17A4	-0.047701011	5.951694558	0.074765761

SLC7A10	-0.050062781	6.149069052	0.074794058
KRTAP13-3	0.046129998	6.056394839	0.074833482
QSER1	0.048026922	6.244739752	0.074844218
SLC17A2	-0.05598716	6.064305356	0.074893986
BHLHB5	0.073525274	6.37584207	0.074895635
RFXDC1	-0.050031662	6.180077315	0.074927668
LOC651556	-0.045143728	6.210412763	0.074982265
VSIG1	-0.063633849	6.025711846	0.074991646
EPN3	0.142817675	6.916274301	0.075001577
ARHGEF10	0.085685717	9.377126732	0.075078413
CAPN12	-0.036174804	6.191068657	0.075195004
FBXO27	0.050678004	6.077750125	0.075282258
PLCL2	-0.122423556	9.577295432	0.075311722
HMG1L1	0.147936178	8.098515372	0.075419583
LOC644227	-0.04690563	5.943485575	0.075462484
LOC642669	0.047094877	6.124763464	0.075498259
DEPDC4	-0.033557887	6.086078788	0.075569295
GNG5	0.096797572	9.422918317	0.075579227
C10ORF132	-0.04593852	6.045071057	0.075616178
CBLN1	-0.149677331	6.718338876	0.075648099
LOC400506	0.065546288	6.587465382	0.075648639
LOC642282	-0.07746148	6.394831786	0.075719726
PNKP	-0.082357528	8.732126719	0.075735162
CSTF2T	0.075390007	7.470063029	0.07575126
RNF121	-0.055457397	7.988175809	0.075765849
LOC649957	0.055243812	6.233156499	0.075770266
HSP90AA1	-0.105495755	11.97481235	0.075800793
CST9	0.081465613	7.78026559	0.075819608
LOC652517	-0.045148574	5.949947893	0.075852193
LOC648415	0.045428319	6.11939818	0.075873336
TMEM148	-0.0424548	6.026672421	0.075874644
NRSN1	-0.045017369	6.015987224	0.075914503
TNXA	-0.056261094	6.340117278	0.076007442
LOC645312	-0.042949718	6.130917578	0.076043769
LOC648997	0.078373546	6.067786264	0.076117941
USMG5	0.33878269	8.530083281	0.076139967
SLC35B4	-0.065966447	6.373412332	0.076145052
LOC647104	-0.077971089	6.693175172	0.076213784
LOC652534	0.038733701	6.104516922	0.076215185
LOC652704	0.050088504	6.170733151	0.076247095
PIGF	0.067563761	7.193236201	0.076251117
RAB3IP	0.073838878	8.504843386	0.076277925
LOC729021	-0.076198673	6.847680981	0.0763072
SUPV3L1	0.066319766	8.099905781	0.07633773
RBM44	0.046369131	6.358572909	0.076350548
CORT	0.057446028	6.089233773	0.076374654
LOC391811	-0.0924361	11.58179328	0.076375127
C14ORF143	0.046711592	6.394866756	0.076402561
LOC389748	-0.042722167	5.989870259	0.076427088
RAD52	0.033827673	6.131526172	0.076453977
SYT7	-0.063650015	6.465862096	0.076474542

GTSF1	-0.104945443	6.132899226	0.076509822
LTB	-0.057719186	6.29337319	0.076556331
HOXA2	-0.034212152	6.086957149	0.076582726
IL3	-0.047445046	5.975173716	0.076603309
STT3B	0.093192896	7.621866843	0.076732326
ETV5	-0.092334856	7.215032257	0.076733532
LOC647592	0.047183938	6.132977803	0.076737326
C6ORF106	-0.072889613	7.474897835	0.076793114
OR2D2	0.044634125	6.021399243	0.076834836
TEKT4	0.056793891	5.934322398	0.076869918
LDOC1	-0.066220968	6.864031887	0.076898149
CDC42EP3	0.1242656	7.454912404	0.076912743
PGM1	0.099702137	12.6566895	0.076915246
VN1R1	0.059459598	6.354457233	0.07702743
MARS2	-0.049087384	6.618811352	0.077090528
RPL24	-0.095867672	13.19525612	0.077092368
ZNF682	-0.140178327	9.50266774	0.077134651
TRMT6	-0.078428562	6.306848536	0.077141951
TNFSF9	0.054253507	6.396234857	0.077147254
RDH13	0.105232892	8.29983812	0.077163006
LOC646266	0.043881042	5.990360296	0.07717544
RFP	-0.056300576	6.867245484	0.077184233
LOC653778	-0.086164356	8.055705525	0.077184795
DNAJB14	0.045618214	6.732606592	0.077205093
LOC285382	0.075817999	7.132791755	0.077207151
LOC441582	-0.045973812	6.043432809	0.077210234
TEKT3	0.055783993	6.090374229	0.077283133
MFSD11	0.100209782	8.836177205	0.077286596
LOC642321	0.040983093	6.125362046	0.07734735
UBA6	-0.099282007	7.273575076	0.077395359
ZSWIM2	0.040684147	5.997215006	0.077399362
PI4K2A	-0.072563213	6.817780486	0.077449484
LOC643201	-0.025478628	6.120237316	0.077466275
LOC402251	0.165669869	11.28230963	0.077501647
CABP7	-0.041343807	6.261648861	0.077543736
DEK	-0.129462015	9.264039041	0.077561164
APOC3	-0.049347393	5.994697118	0.077590415
ASMT	0.044691574	6.034288374	0.077722607
LIF	-0.05625989	6.105466041	0.077747534
LOC644979	-0.122473459	7.219787654	0.077859022
OR13C8	-0.047854114	5.95537166	0.077930225
NXF5	0.042706429	6.075083636	0.077951915
ADCYAP1	-0.051414772	6.290064156	0.077994676
LOC647778	0.049740653	6.303384329	0.078007886
LOC652898	0.045164965	5.971013263	0.078052618
ANP32B	0.063010443	11.43925474	0.078061251
LOC643176	-0.048745115	6.299136781	0.078099704
DENND2C	-0.08313242	6.705601843	0.078160127
TTK	-0.044699081	6.01228736	0.078215501
GPR42	0.046112929	6.002161999	0.078242461
NOTCH2	-0.041293856	6.242030353	0.078249918

PON1	0.055895425	5.973405221	0.078275965
C15ORF44	0.036274996	6.520330543	0.078301614
RPH3A	-0.045016345	6.222868947	0.078365419
SLC22A17	0.060188818	7.108458384	0.078388725
FAM24A	0.04557503	5.981442138	0.078427906
TMEM16B	0.053150661	6.467864764	0.078432697
OR6C76	0.04978474	5.930426025	0.078434205
DCT	0.041970271	6.093280255	0.078471528
SNORD83A	-0.040715317	5.939000104	0.078544891
ME3	0.070557601	7.387026789	0.078627128
TSTA3	-0.070414823	7.127936275	0.078781445
ELF5	-0.030321845	6.002398313	0.078782774
FAM109A	0.093003176	7.174372346	0.078795563
ZNF681	-0.032851719	6.000517704	0.078798059
OSTBETA	-0.037878027	5.959927645	0.078932163
LOC388755	-0.043274247	6.153661331	0.079009139
LOC651073	0.050546602	6.184981097	0.079038503
FAM162A	0.21215303	10.05192876	0.0790408
NPPB	0.677930546	12.34261697	0.079063704
GPN1	-0.090122004	8.962356851	0.079072534
SNORD109B	0.047445854	6.302181076	0.079083886
RUSC2	-0.107950128	7.142043203	0.079118269
LTBP4	0.107449273	8.64949692	0.079165686
USP11	0.078440955	7.702718509	0.079236978
LOC730510	-0.039986717	6.268384053	0.079341382
LOC644412	-0.048306955	5.98342383	0.079341975
POLR3C	-0.06528571	8.988624195	0.079360581
PRM1	-0.043178607	6.110099338	0.079472603
LCE5A	0.042245029	6.211924539	0.079473711
RSHL1	0.048914262	6.21808517	0.07951801
C22ORF9	-0.032194568	6.191244019	0.079533088
TMEM98	0.106315214	8.041275619	0.079588973
NKX2-8	-0.069039813	6.034764988	0.079622919
CACNA1I	0.032186972	6.096134897	0.079623239
OR7E37P	-0.047318966	6.136105687	0.079628202
AAMP	-0.091640627	7.34035444	0.079652941
LOC440978	0.034703307	6.155840097	0.079675689
CDKN2B	0.03958033	6.448659301	0.079697736
PTPRC	-0.024937703	6.055871534	0.079698267
RALGPS2	-0.025814935	6.035020563	0.079769737
AP4S1	0.053664075	6.675359912	0.079915155
DNAJC8	0.073712802	11.39564281	0.079939931
LOC728653	-0.072674914	6.728740838	0.079947567
SLC4A5	-0.101635717	7.702678578	0.079955667
MEGF8	-0.056913096	6.29409507	0.080002903
PSMD13	-0.065743869	6.733356814	0.080014217
CHMP6	-0.050664738	7.634345669	0.080030172
FAM115A	-0.138554492	10.97792764	0.080061461
TMEM151A	-0.028089926	6.064804506	0.080129823
VEPH1	-0.04593412	6.041762047	0.080203388
ASB14	0.056500656	6.085687408	0.080217436

LOC651333	-0.04461117	6.036052757	0.080239024
C15ORF27	-0.042790839	5.945625032	0.080270541
ELL3	0.054974258	6.333447401	0.080321101
BPY2	0.031515151	5.962667347	0.08032401
ADAMTSL1	-0.023582369	6.179164875	0.080363752
UTP14A	-0.062938959	7.706180265	0.080393638
CCL4	0.039470498	5.999313069	0.080437949
BRSK2	-0.055645462	6.060262537	0.080449949
LOC643669	0.032710975	6.215567151	0.080485508
LOC653050	-0.029507017	6.146890547	0.080494715
C9ORF117	0.04105404	6.126055064	0.080530136
FBLN7	0.061982764	6.213755136	0.080547805
LOC96610	-0.063140951	6.798743073	0.08055264
SFT2D1	-0.093584748	9.899658907	0.080582467
LOC642573	0.041082763	6.215114092	0.080589042
LOC646302	0.057846416	6.046862451	0.080595862
CLCN6	0.057218949	7.734430163	0.08072276
FNDC5	-0.185681829	10.07975587	0.080760473
RNF216	-0.104773431	8.09893174	0.080766467
HSPC047	-0.046370501	6.411805876	0.080801341
EEF2	0.074362519	13.12358535	0.080846951
NOMO3	-0.09002872	6.618831117	0.080854489
SREBF2	0.072838887	6.676388786	0.080856843
LOC442448	0.043206742	6.252861384	0.080924774
GDE1	0.104438062	8.977507734	0.080927891
PIGN	0.065762685	7.8986771	0.080971569
SEP15	-0.099681419	10.1438082	0.081079154
LOC283392	0.047313767	6.09623444	0.081094404
ZNF800	-0.076946862	7.223983906	0.081258657
DKFZP451A211	0.157262543	9.882796312	0.081266718
MFAP3L	0.029686464	6.009795219	0.081267207
APOA2	-0.045670828	6.077414007	0.081299481
GPM6B	-0.102240449	7.019741986	0.081317933
AGGF1	0.058993833	8.011715219	0.081387801
IFNA21	-0.045372443	6.260842142	0.081399646
C7ORF43	-0.063003898	6.549535312	0.081418902
FAM58B	-0.040717734	6.080077428	0.081488378
PRKACB	0.025115596	6.116911596	0.081515855
TCF23	0.043740506	6.167075345	0.081575121
MYO18A	0.09715736	7.849545343	0.081674738
LOC643311	0.05271833	5.915434254	0.081679477
LOC642236	0.073215273	7.479621125	0.08169248
FCF1	0.058932479	6.899213693	0.081704928
LOC652278	0.048390715	6.170232367	0.081732021
CUEDC2	0.07431917	9.822158187	0.081785016
LOC644642	-0.084503702	6.938682396	0.081818646
PHF23	-0.064736785	7.641953149	0.081893404
LOC644764	0.041831153	6.216985396	0.081918876
LOC647415	0.047808892	6.04721671	0.081942388
ZNF813	-0.047695191	6.160020619	0.081985528
TMCO4	0.052055338	6.505568408	0.082028668

ZNF778	0.044182835	6.25308646	0.082068973
LOC254559	0.04719631	6.036022923	0.082110187
RAP1B	-0.068030892	6.771643167	0.082175728
LOC653807	0.046591384	6.005811133	0.082204226
C21ORF57	-0.093474489	7.705319142	0.082214754
LOC653480	0.043933193	6.166057156	0.082231641
LOC646533	0.040272252	6.05909418	0.082333775
CYP20A1	-0.04510518	7.14530039	0.082378651
OR8D2	0.043511169	6.105252109	0.082400413
LOC644264	0.047535957	6.061275332	0.082461429
LOC645151	0.037222192	6.070844988	0.082464645
TTYH2	-0.065269427	6.794260194	0.082547987
MBD2	-0.029926205	6.15951934	0.082574799
CCDC107	-0.082585776	7.811961	0.082589985
LOC646348	0.045422415	6.251527827	0.082595735
LOH12CR1	0.057440641	6.556205817	0.082597913
LOC388780	-0.042042624	6.166988501	0.082781188
ZNF423	-0.04911209	6.567591445	0.082806483
ATP2B3	0.025482728	6.015925897	0.082852402
C5ORF22	0.055430269	6.930926102	0.082875632
FLJ36208	0.045305049	6.348706947	0.082998638
LOC643032	0.045369589	5.985036105	0.083017828
NAP1L4	-0.105641134	10.60675744	0.083047029
ADAM21	0.057821213	6.616583297	0.083049268
HEPACAM	-0.031773658	6.089168101	0.083232647
LOC388153	-0.036517624	6.056377117	0.083366673
C3ORF42	-0.028549135	6.04684628	0.083427936
CEP135	-0.059146686	7.067503543	0.083459875
LOC645478	-0.042644495	5.965193802	0.083511006
RPL6	-0.070627296	12.61319105	0.083585335
CNNM2	-0.031049288	6.115583194	0.083585492
APOBEC3G	0.063812805	6.498106348	0.083669869
DDX18	-0.083669393	9.30471491	0.083700148
ZNF623	0.033449521	6.118743721	0.083708283
LOC389833	-0.103681439	6.678312585	0.083725156
DTD1	-0.071621827	7.6809104	0.083769378
LOC642178	-0.047259288	5.914172753	0.083831155
RPP25	-0.057395025	6.387541476	0.083858829
XRCC1	0.063692849	7.146695107	0.083913508
LOC642653	-0.039705477	6.020187449	0.083917279
LOC654002	0.082122184	5.983691808	0.083947321
ZNF514	0.063328325	7.14860719	0.08395012
SYT13	-0.151892441	6.844425901	0.083986105
MKRN3	-0.044162605	6.085981089	0.083993035
ZNF702P	-0.060777118	6.50370331	0.084063193
LOC647308	0.043840448	6.043413359	0.084066135
DC36	0.041411186	6.276983921	0.08407261
LOC652570	0.040870874	5.873596839	0.084120361
FILIP1L	-0.136441758	8.299940672	0.084185105
GSC2	0.049428834	5.927248715	0.084278993
PHOX2A	-0.044853932	6.069948656	0.084288729

KIAA1600	0.082533746	8.733278054	0.084329188
KLK1	-0.045425886	6.261074295	0.084330809
SEMA7A	-0.036814031	6.076603219	0.084377257
PCOLCE	-0.127212286	8.659700259	0.084526786
SATB2	-0.049480522	6.166472671	0.084559083
LOC731365	-0.085982263	12.76109136	0.084561194
CDK8	0.059685022	6.760068829	0.084605684
LOC389607	0.04526645	6.073130096	0.084839285
STX17	0.045504871	6.226121029	0.084843377
LOC642558	-0.031942413	6.013952168	0.084855709
LCK	0.023852448	6.202391611	0.084982474
C8ORF77	0.048135855	6.313232914	0.08501314
WDR72	0.046322814	6.043792253	0.085024635
APOBEC3D	-0.045588673	6.104748268	0.085025341
LOC647910	0.058728227	6.225014437	0.085076211
LOC440359	-0.138988437	11.09248906	0.08507746
FAM62C	0.050319079	6.1379578	0.085084901
HSPA1A	-0.160357891	11.47102832	0.085088578
LOC643018	-0.044643475	6.103797047	0.085180856
ZNF445	-0.046524384	5.981965468	0.085202068
C8ORF59	0.085140701	9.97170999	0.085209848
SEMA4G	0.043675637	6.228492041	0.0852477
LOC440067	0.042000491	6.040967432	0.085263288
LOC648278	-0.045102951	5.973443254	0.085295419
C4ORF12	0.046530485	6.176344283	0.08536391
HYLS1	0.05485461	7.125923118	0.085482732
LCE1A	0.047638326	6.007212909	0.085492106
COL8A2	0.075675712	6.924167311	0.085512753
LOC651429	0.044401245	6.411255691	0.085582414
LOC654230	0.040412015	6.053075118	0.085591108
LOC643085	0.039644967	5.972959017	0.08560071
LOC645210	0.040528908	6.129407044	0.085654443
NUP88	-0.078893881	9.36193273	0.085837079
VSNL1	0.12308409	7.451017048	0.085884798
RAC3	-0.048941952	6.346892235	0.085925397
LOC652357	0.037109668	6.10795058	0.085936668
LOC400796	0.042308443	6.05642387	0.085948231
LOC644066	0.037674625	6.055134723	0.085999836
FLJ37201	0.045879726	6.11329751	0.086104153
FRMPD2L2	0.021115185	6.10673689	0.086115412
CNIH	0.097965966	9.403648592	0.086141313
PDE6G	-0.043935	6.45616637	0.086152202
LOC644294	0.047833121	5.967603509	0.08615977
DNAJC11	-0.066129117	6.995931174	0.08636753
OR5W2	-0.050944882	5.936488726	0.086372273
FBN1	-0.083747304	6.629367916	0.086394513
FLJ40292	-0.049386173	5.961102968	0.086397735
AURKAPS1	-0.049865216	6.258671443	0.086401025
GTSCR1	-0.045213905	6.097594255	0.086402125
CCDC102A	0.084498996	8.13740313	0.086530266
NEU2	-0.037424679	6.021243982	0.086602774

LOC646114	0.035921504	6.123242487	0.086615548
LOC643837	-0.047349844	6.085040137	0.086618134
GNG12	-0.128909974	7.434607445	0.086663413
SAPS2	0.06973614	7.045949372	0.086667674
LOC652578	-0.038389923	5.980551953	0.086677065
CCDC103	0.043038593	6.123111017	0.086735119
ALDOB	-0.062215927	6.601638436	0.086738561
LCA5	0.045769003	6.321639369	0.086791254
WBSCR27	0.050891885	6.175383118	0.086879818
PLEKHF2	0.049573549	6.540309027	0.08689626
LOC442028	0.056516101	5.831965066	0.086994856
LOC650840	-0.049691351	6.350091454	0.087069442
HSD17B13	0.037664782	6.105924655	0.087121225
ITGB4	0.036700348	6.262476173	0.087139483
ARL13A	0.046081797	6.089591266	0.087181318
LOC652614	0.042460577	6.194151318	0.087203296
TLK2	0.098506325	7.944675488	0.087224627
FLJ16369	-0.047818515	5.90164916	0.087300986
AIRE	-0.118104702	10.9454358	0.087378049
C10ORF10	-0.18197584	8.802562849	0.087385688
LOC652568	-0.044108829	5.983138324	0.087541093
APOBEC2	-0.150510742	11.48509554	0.087569803
TNRC6C	-0.034978266	6.109909977	0.087591344
IDH3B	-0.077887243	11.20863924	0.087618706
AKT1S1	0.056480156	6.400773993	0.087719735
WNK2	0.11462246	7.440304429	0.087782835
SCGB3A1	0.05409754	6.157687674	0.08780878
LOC441737	0.050274215	6.13482024	0.087814487
IL16	0.035711028	6.250091402	0.087831626
TBCA	0.080150829	12.23121662	0.087846894
LOC644705	-0.044860803	6.019258147	0.087857533
VPS53	0.047664677	6.182725202	0.087924183
OR4C16	-0.053206653	6.075243915	0.087926646
LOC646128	0.037011696	6.195531222	0.087948636
LTB4R2	-0.036747951	6.017431577	0.087979493
MBTD1	-0.082899658	7.187325458	0.088019904
IL17B	0.042940357	6.094533224	0.088103663
FBXO44	0.029670524	6.130894002	0.088131299
C2ORF77	0.058015101	6.036818855	0.088260424
ZNF41	0.031406735	6.099330462	0.088274307
ERP27	-0.047817717	6.211749945	0.088281842
LOC158381	0.042165976	6.337835028	0.088302485
CLIP1	-0.109341098	8.912416562	0.088320876
MRPS23	0.080363486	9.061490811	0.088329186
HINT1	-0.090691256	11.96686311	0.088329871
ARID5B	-0.109381312	9.66831792	0.088405135
LOC401622	0.060850024	6.661416895	0.088475949
OR4K15	0.029360632	5.980696742	0.088483709
STK11	0.094518072	6.708171783	0.088510105
INPP5F	0.030943333	6.496389438	0.088531711
C1ORF56	0.046074432	6.377250395	0.088594371

LOC646422	-0.036811452	6.067368078	0.088667828
LYPLA2P1	-0.035841016	6.390645749	0.088678413
C1ORF189	-0.040321885	6.033738809	0.088686122
FAHD2B	0.099062772	9.173534476	0.088730531
INTS8	0.097990576	7.700382017	0.088744031
LIG1	-0.049896974	6.477578966	0.088744233
CCDC85A	-0.087165367	7.113815412	0.088782731
NPLOC4	-0.095643207	8.222814887	0.088865884
SNX4	-0.099237348	8.435234491	0.08886895
ASCL2	-0.051100455	6.493664067	0.08900516
LOC283970	0.029521486	6.161148855	0.089055004
PLEKHM1	-0.062061926	7.46028024	0.089104224
PLEKHM3	0.04859606	6.002836246	0.089187226
OR6C4	0.053675139	5.884042984	0.089215905
SUMO1P1	0.028659307	6.029859829	0.089273574
LOC391045	-0.05853379	6.05699034	0.089274068
RBP3	-0.050150616	5.945311332	0.089325618
TRIP12	0.094060558	9.232304515	0.089326534
LOC402644	-0.190974059	7.914544233	0.089389184
LOC653276	-0.041063073	6.151846476	0.0893945
NUP153	0.061834665	7.802077214	0.089408546
LOC653125	0.032354179	6.167896249	0.089431397
LIN52	0.073902599	6.713335376	0.089444638
GPR25	0.050406919	5.986509019	0.08946408
LOC652342	-0.041197093	6.068748718	0.089513284
LOC650905	-0.038526885	5.982782788	0.089547295
LOC652796	0.038770567	5.996942618	0.08958937
ABT1	-0.072458814	6.247316362	0.089625874
ANKRD47	-0.077389333	7.319670212	0.089655666
LOC442454	0.099775711	12.72406829	0.089689216
PCGF3	0.05005233	6.150546447	0.089697916
LOC652453	0.045245971	5.978610689	0.089799812
SULT1C4	0.04625808	6.270870029	0.089828797
LOC645322	0.041785548	6.024250186	0.08989864
GALC	0.073292208	8.076521373	0.089904924
BGLAP	0.048139299	6.777862142	0.08991964
CBX4	0.106224808	7.590948378	0.089991177
FCAR	-0.101757972	7.932110616	0.090026033
PMCHL1	0.049701	6.281361899	0.090080146
LGALS1	0.1062131	12.87151154	0.090177841
KAT2A	-0.073673617	8.70207013	0.09028489
TMEM162	-0.041202898	6.00598911	0.090287042
STAG3	0.051515589	6.541497314	0.090312843
GIP	-0.036040854	6.001908172	0.090335426
LOC643977	-0.056586503	6.245324125	0.090367969
STOML2	-0.062653678	10.91021144	0.090405704
NAGS	0.056451693	6.256438694	0.09044889
MPP2	-0.09629302	7.115865435	0.09049807
MSRA	0.079314922	9.850707646	0.090605891
SDHB	0.081923951	12.56034904	0.090611479
SCML1	-0.053364209	6.842600797	0.090698592

SLC1A1	-0.051289262	6.163120534	0.090737696
SPACA5B	-0.035736724	6.045489293	0.090744211
C16ORF82	0.045615806	5.961349628	0.090772665
OR5AR1	0.029009509	6.057626097	0.090822058
LOC654253	0.06841787	6.090581574	0.090837986
COL8A1	0.163540879	8.581433343	0.090845135
KIAA1660	0.035988551	6.078486815	0.090862785
LRRC37A	-0.047474732	6.217447287	0.09086522
NME7	0.065351655	7.302048071	0.09102641
DCAMKL3	-0.041802295	6.0813713	0.091033893
COL19A1	0.030416639	6.067269649	0.091074408
CYP7B1	0.047870705	6.084391876	0.091116075
C4ORF16	0.044077581	6.25750314	0.091126547
MYL4	-0.372316161	9.957156378	0.091154379
C1ORF115	-0.120951136	8.503933862	0.091170248
SLITRK3	0.039689216	6.048542491	0.09118443
HNMT	0.055087735	6.91480952	0.091290467
OR13C3	-0.041741511	5.994171561	0.091330467
ZBTB6	0.064321732	6.2131887	0.091357388
C1ORF100	0.04425289	6.094533316	0.091374131
SLC2A4	0.070807173	6.61159177	0.091405407
C1ORF125	-0.034706264	6.000354656	0.091408071
MPRIP	-0.108741623	10.31962983	0.091443845
MMP14	-0.044062466	6.238302004	0.091485785
DSCAML1	-0.04421261	5.971162433	0.091558855
TNFSF12	0.057895284	7.61690407	0.091655658
LOC286239	0.0410692	5.86938779	0.091656036
NOX4	0.17636406	7.617109106	0.091809279
F2RL1	-0.126761989	6.794077861	0.091861382
MGC57359	0.021451702	6.151407071	0.091881337
PSMC5	-0.096741532	10.55921575	0.09188968
RPS27L	0.091625909	10.82565758	0.091980469
LOC442572	0.036834824	6.067075932	0.092018413
LOC647107	0.04196208	6.05179132	0.092110949
OR2AG1	0.045859949	6.183555555	0.092113197
PCDHGA8	-0.048602165	6.056952606	0.092131078
FAM63A	0.090521521	8.560003205	0.092133345
LOC643422	0.042484833	6.322155569	0.092150608
LOC644634	0.025312492	6.272010606	0.092241676
OR7A10	0.044002693	6.145202517	0.092250125
ASTE1	0.057936398	7.115876071	0.092254291
JAKMIP2	-0.048513937	6.043081719	0.0923563
LOC645435	0.046884364	6.022924627	0.092375264
ASPHD1	-0.028482279	6.029191609	0.092392482
SUGT1P	-0.046218114	5.976985891	0.092453445
BCAN	0.030812602	6.275243595	0.092537433
LOC652068	-0.044137342	5.978626014	0.092544566
MGC3020	0.053976002	6.484422617	0.092595154
LOC647333	-0.035005856	5.984652276	0.092613281
ECHDC2	0.087209649	11.4369155	0.092620457
FUSIP1	0.045881313	6.748241277	0.092624933

MGC40168	-0.04124704	5.975820401	0.092639346
PBOV1	0.028171445	6.08389604	0.0926606
LOC644242	0.042321938	6.275517281	0.092688093
DZIP1	0.037039858	6.053785167	0.092758049
LOC440456	-0.021569895	6.116353674	0.092762277
LRCH3	-0.058395025	6.919765903	0.092773249
HOMER3	-0.090608872	6.997781684	0.092778324
ASB4	0.035241898	6.055803532	0.092792906
LOC644886	0.042812134	6.018810863	0.092827644
MRPL50	-0.119318365	9.605427058	0.092871556
ATRX	0.037580817	6.087953745	0.092877282
ZNF324	0.066911228	6.96750978	0.092891373
RUNX1	-0.076723416	6.403216276	0.092921721
MYSM1	0.050888513	6.125289683	0.092947681
CCDC113	0.048958091	6.148057564	0.093035805
RAB22A	-0.06467425	8.66400644	0.093056538
AGPAT6	-0.077779316	6.545563537	0.093056903
PLD5	0.158348347	6.214126441	0.093057792
LOC143188	-0.042118628	6.003799428	0.093059054
ATG10	0.065739813	7.813568358	0.093149242
IFNA10	-0.047889025	6.13885191	0.093164196
CYP24A1	0.044541866	5.937952265	0.093304833
STS	0.046210282	6.311051387	0.093344728
GSX2	-0.041614306	5.886086427	0.093404812
SLC13A2	-0.048495447	6.144156337	0.09341229
PPP3CA	-0.061509417	6.815469962	0.093439237
CD5L	0.052930568	5.927118032	0.093536872
ZNF280B	-0.047604702	6.069394012	0.093538748
LGR8	0.040941097	5.942641322	0.093541233
SMAD2	-0.027512649	6.291331988	0.093609089
ANGPT1	-0.081008569	6.828625752	0.093620783
SPRR1B	0.049888957	6.412131887	0.093693873
LOC646374	0.040868226	6.055683847	0.093717119
PTK9	0.068853608	6.200336829	0.093731047
BRAP	0.070504843	5.938922969	0.093773929
ETAA1	0.059290777	6.212182881	0.093789288
MUCDHL	-0.043257639	6.106765281	0.093793958
HOXD9	-0.04958052	6.017051844	0.093950115
PIN1	0.061796967	11.2480276	0.093954326
SNX25	-0.043180685	6.925646194	0.093955409
APOH	-0.041467034	5.925476652	0.09395754
SPESP1	0.050913337	6.350230308	0.094072331
SLMO1	0.033520745	6.400153725	0.094108051
ABCC11	-0.026289363	6.10574482	0.094117481
TMBIM4	-0.06345461	11.51209032	0.094130381
GPR82	-0.029871641	5.98804824	0.094131852
PCSK1	-0.033321541	6.03904533	0.094209663
LOC285479	-0.044971643	6.175748772	0.094240357
NPHS2	0.047594112	6.113300577	0.094253958
ZNF821	0.066683996	7.086429448	0.094276158
KIFC1	-0.046599711	6.081427269	0.094357403

PHACTR1	-0.125595612	7.422226594	0.09440316
LRRC37A4	-0.029589374	6.288197167	0.09450102
SGTA	0.084320987	7.702385844	0.094627781
GUSB	-0.062738448	8.214121779	0.094696364
HBBP1	0.041387957	6.151121221	0.094868447
LOC441601	0.038925151	6.067640201	0.094869098
LOC728863	-0.057023856	6.208063195	0.094883177
LOC644424	-0.046555291	6.326689018	0.094887244
GJD3	-0.070274678	7.429489743	0.094960907
TMPRSS6	-0.051597143	5.938966981	0.094963464
XPNPEP1	0.053134307	10.04957228	0.095021269
CD3EAP	-0.046183716	6.453471966	0.095112758
TMEM45A	-0.101283981	6.75057951	0.09524233
LOC653497	0.040298006	6.279749029	0.095243235
MBOAT1	-0.053303032	6.491969163	0.095272629
LOC442366	-0.042829822	6.282301322	0.095316037
ARMC9	0.064569672	6.283311359	0.095348003
LOC440053	-0.037644139	5.926007702	0.095351213
LOC653876	0.041324664	6.375321792	0.095385892
MRPL41	-0.098237496	12.01774123	0.095440125
ZNF219	0.089526413	8.059269094	0.095458151
H2AFX	-0.060152422	6.304057987	0.095504252
BEGAIN	0.091541542	7.285525291	0.095513441
LOC732111	0.045630881	6.225882273	0.095534655
LOC648841	0.03970777	6.065733522	0.095582678
LOC374920	-0.044912127	6.490706992	0.095607372
FLJ41200	-0.069458033	6.195102121	0.095627335
NOL4	-0.030933593	6.131578574	0.095739475
PRSS7	0.046620895	6.68378645	0.095805057
LOC650803	-0.076638111	6.276228809	0.095817814
FAM22B	0.039468727	6.149128259	0.095817986
FLJ11235	-0.086802086	7.278964964	0.095823125
USP21	0.038201171	6.878487927	0.095863731
EFCAB2	0.08477886	6.154342045	0.096055907
NADK	-0.058503024	7.010461108	0.096068449
C17ORF42	0.041354836	6.083990837	0.096083856
LYST	0.031227999	6.314277627	0.096145739
LOC651483	0.050955617	6.0894561	0.09619565
LOC728789	-0.036270282	6.082615644	0.096198752
WDR37	0.078977958	8.926015018	0.096205255
LOC728936	0.04017849	6.099733625	0.096262756
RPL36AL	-0.089793905	12.2964524	0.096388059
LOC646064	0.048280243	6.415078257	0.09644897
SULT2B1	0.038177424	6.708981	0.096468898
NEFM	-0.049446992	6.145596958	0.096485091
CHRNE	-0.044630395	6.290479677	0.096566117
LOC643346	0.0380671	6.005806924	0.096627845
LOC644266	0.036536193	6.15288881	0.096630267
CD40LG	-0.034805334	6.005885281	0.096669683
FLJ44005	0.043753721	6.162427557	0.096690735
PCSK5	0.097179117	7.906934846	0.096692597

TRIM4	0.063399479	7.798446714	0.096753451
OR7A5	0.039842092	5.980867808	0.096759838
XAB2	0.086473722	8.411385767	0.096794119
LOC653874	0.094540364	6.674086916	0.096876937
TRPM6	-0.046793695	6.062655605	0.096877676
THEG	-0.034481064	6.245267096	0.096921937
ZNF826	-0.044349244	6.108316101	0.096964369
ADAMTSL3	0.101480698	6.897043103	0.096984419
RTL1	0.029963346	6.028174895	0.096988052
UTP6	-0.092609688	8.774689253	0.097091575
RPS7	-0.125493022	7.814176132	0.097168954
WDR19	0.079095044	7.836975547	0.097225585
KIAA1609	-0.04129405	6.190932726	0.097253181
RACGAP1	-0.044066146	6.23165917	0.097288654
C16ORF54	0.050787714	6.196755897	0.097297183
LOC642397	0.023750776	6.026546583	0.097305273
LCN9	0.040856119	6.010617864	0.097377866
DEFB121	-0.049845683	5.935482399	0.097403177
CYSLTR1	0.050779642	6.304473034	0.097415225
PCDHA3	-0.02613101	5.97850366	0.097434787
SLC41A2	-0.049609501	6.086011504	0.097447173
OR4F21	0.029569388	6.056567688	0.097479177
GJA10	-0.045635906	5.952259817	0.097506797
EEF1B2	-0.06660094	10.73151463	0.09751768
GALNT13	-0.041265108	6.069900657	0.097528896
PHPT1	0.067180642	11.65514945	0.097536495
LBA1	0.060157255	6.716363429	0.097609044
THBS3	0.093826696	6.763708158	0.097610008
MON1A	-0.049032692	6.470416087	0.097730033
ASH1L	0.042030523	6.303351387	0.097732933
LOC729744	-0.026104621	6.095313231	0.097811569
LOC648827	-0.047916398	6.169126499	0.097812681
LOC642373	-0.063835393	6.369374975	0.097842619
ZNF280A	0.047278616	6.219587638	0.097862997
LOC642968	0.039538749	6.135001691	0.097873139
HSFX1	-0.02645326	6.158704518	0.097900393
LOC127295	-0.105099797	10.21760318	0.097903439
LOC146439	0.055153211	6.227484657	0.098007193
OR4X2	0.04544248	5.948318169	0.098023329
AKAP13	0.088372382	10.09336694	0.098050415
LOC654010	-0.043401351	6.000514893	0.098056192
CATSPER3	0.064172068	6.068018601	0.09811846
ATP5I	0.077728613	12.51940048	0.098146484
GLP2R	0.038707128	5.945221045	0.098170503
IMP3	-0.058043263	11.0415682	0.098228538
LOC391378	0.041643316	6.072136857	0.098253114
CDC42BPA	-0.050230026	6.889897961	0.098274301
ASB11	0.055467428	6.348049031	0.098328659
LOC388210	-0.037126078	6.120357293	0.09836177
MUC16	0.044909305	6.163080158	0.098422802
VANGL2	-0.067411543	6.605374746	0.098424072

LOC652553	0.045545227	5.948127909	0.098493452
SLC5A10	-0.022786933	6.030274114	0.098501254
IRS4	0.043413453	6.447350157	0.098503753
KPNA5	-0.062592118	6.532978466	0.098652346
LOC652674	-0.036942547	5.999305534	0.098663072
CAPS	0.052682013	6.585599831	0.098727588
CRYGB	0.05650715	5.99277159	0.098768407
SPTB	0.049665016	6.794119061	0.0988058
LOC647855	-0.038483955	6.075256301	0.098818381
AMOTL2	0.060258573	6.083742421	0.098822189
LOC652620	-0.052180643	5.921615845	0.098889457
RPRC1	-0.067402589	12.16670555	0.098941703
NCF2	-0.067943901	6.139761142	0.098981579
ART1	0.06014366	6.223850044	0.099005608
PODXL	-0.094683675	8.625089256	0.099026556
SMURF1	-0.041303983	6.271386564	0.099040797
LOC647122	0.036647382	6.118956267	0.099041576
OR5K4	0.031479152	6.164685962	0.09906604
RBM34	0.090651198	8.259284018	0.099115121
LOC340113	0.045178242	6.208248622	0.099135047
INHA	-0.080897619	6.263731852	0.099136183
CT47.1	0.029177871	6.055700331	0.09920489
MOGAT2	0.042643537	6.137624304	0.099284124
LOC642183	0.041736569	6.267601451	0.099309964
ALPI	0.045822657	6.248872186	0.09932036
CES1	0.141508359	7.095863275	0.099362877
PPT1	-0.107840616	10.18777545	0.099384762
LOC643258	0.042480475	5.947543786	0.099391233
EDEM3	-0.052334986	6.819604398	0.099403375
COX6B1	0.082046927	12.98307901	0.099407766
RY1	-0.074167021	9.422078638	0.099447275
NSD1	0.034479986	6.547094128	0.099447793
RAB41	0.044825479	6.046145477	0.099452506
ZBTB10	0.046731221	6.204394764	0.099484976
SLC14A1	0.100333141	7.236773726	0.099601732
LOC642933	0.033172511	6.030766807	0.099625433
CCDC60	-0.044594989	6.10582959	0.099641908
HOXC6	0.028165162	6.279437185	0.099648245
FGD4	0.038208057	6.212121522	0.099769792
LOC642834	-0.043321667	5.979751397	0.099879007
C17ORF79	0.086641487	9.088333503	0.100003395
SNTG1	-0.035701643	5.936579983	0.10003916
FRMD4B	0.027414934	6.062267733	0.100048647
MRRF	-0.04825958	8.245980528	0.100067939
SKIP	-0.040225008	6.350201795	0.100080524
DGKD	-0.083365346	6.539662189	0.1000813
PROSC	0.078414392	9.516461738	0.100100727
TRERF1	-0.027053104	6.199442168	0.100143141
LIPI	0.046238311	5.944638322	0.100164545
TTRAP	0.088356542	6.410283695	0.100220514
PRKACG	0.046196681	6.292983826	0.100232068

LOC644297	-0.074689677	6.352931962	0.10027169
LCE3D	0.042464704	5.9423863	0.100277922
C16ORF47	0.041268433	6.251525883	0.100314656
LOC646699	0.039130199	6.085765652	0.100327862
FBXO39	-0.037207456	6.004376279	0.100379834
CRP	-0.044546553	6.032106917	0.10039859
LOC645931	0.051089836	6.08493505	0.100435543
SMAD4	0.086092845	9.499881681	0.100490371
ALCAM	-0.128735081	6.91869311	0.10066059
LOC653889	-0.035385934	5.938249892	0.10075565
PPM1H	-0.07234902	6.870296396	0.100818481
KRTAP3-2	-0.024718053	6.096392642	0.100852308
TMCO1	-0.072194984	10.24929897	0.100886787
KLHL8	-0.067858615	8.082373789	0.100903237
SDC4	0.104271408	8.795486312	0.100934499
LOC399939	-0.039448626	5.890755128	0.100935196
TMEM125	0.048319618	6.149446345	0.101045649
LOC390594	0.04341649	5.970020455	0.101066479
LOC642795	0.041855113	6.206671715	0.101082551
CSPG5	0.040958418	6.091365759	0.101102407
SNORA7B	-0.058290223	6.902779785	0.101127701
QSOX2	-0.064364604	7.39762737	0.101131027
ATPBD3	-0.067796555	6.594353473	0.10118406
LPL	0.118363486	12.80783756	0.101213026
HOXC5	-0.040193414	6.078643343	0.101246782
NAALADL1	-0.055040608	6.594080968	0.101256274
MRC2	-0.051757324	6.334874508	0.101259398
TMEM109	-0.066067098	9.653927012	0.101271265
NOLA2	-0.080535555	8.829208784	0.10129562
IQCF1	-0.045581697	5.949167047	0.101306078
CASC3	0.094091275	8.102454334	0.101335489
TRRAP	0.076331352	9.33227148	0.1013692
HRC	0.111280788	12.87388381	0.101440097
LOC650361	0.039390345	5.985621935	0.101476516
LOC654194	-0.184052581	9.992748413	0.101558402
INVS	0.030254471	6.429511245	0.101654015
CLDN14	-0.06552648	7.657134514	0.101744227
CXORF27	-0.049513107	6.161891017	0.101763692
EEF1A2	-0.075197443	13.58421045	0.101775842
IKZF1	-0.052076338	6.226977612	0.101855877
IGFL3	-0.051901752	6.419458656	0.101857898
LOC645904	-0.033167798	6.193106172	0.101913169
LOC653217	0.047095988	5.910705711	0.101924692
FBXO2	-0.031456061	6.177593749	0.101958282
ATXN7L1	0.047688277	6.139550915	0.101973609
MUTYH	-0.03834427	6.639975644	0.10205762
CD2BP2	-0.070400857	9.849828958	0.102063246
EXOSC9	0.068794686	7.784400663	0.102089664
ZNF624	0.037996074	6.125800374	0.102190591
SAPS3	-0.08512486	8.683756159	0.102289615
OR51L1	-0.040650041	5.956862638	0.102303531

LCE2C	0.046986137	6.048277018	0.102312948
LOC647009	0.060777995	7.068621209	0.10238741
LOC653265	0.036582811	6.283897682	0.102401503
OR1Q1	-0.044706192	5.915213673	0.102468509
C1ORF185	-0.036380006	5.995001877	0.102472761
MCOLN3	0.037240762	6.275254295	0.102488153
OR5D16	0.032568304	6.169707527	0.102490506
ZNF655	-0.041636749	6.907702483	0.102590981
CAB39L	-0.043841382	6.82828448	0.102597541
DIRAS2	0.030351624	6.027278596	0.102683256
RBM33	0.077556424	8.617389995	0.102689112
CYLC2	-0.04789397	6.388023359	0.102716711
CCNA1	0.0578133	6.289817792	0.102716949
LOC643401	-0.025178906	6.063048194	0.10276625
TCP1	-0.097184481	8.671640274	0.102842708
CLDN18	0.02629875	6.288840859	0.102846979
PDGFRL	-0.134968089	8.1026666	0.102873737
FAM71D	-0.036450042	6.020242377	0.102880834
SMAD1	-0.029667839	6.049961403	0.102888111
LOC283584	0.038916349	6.045306948	0.102891124
SMPD3	0.055026313	6.266326634	0.102951324
FLJ40113	0.022844032	6.217914168	0.10297441
CS	0.094791401	10.90666193	0.103031115
ZNF195	0.056685216	7.126028226	0.103046636
KLRD1	0.027038179	6.111157639	0.103054928
MAPK4	-0.144375181	7.750929894	0.103113028
TBC1D9	0.118431785	7.964217965	0.103247373
BLVRA	0.063401334	9.504513552	0.10326998
MFF	0.266235119	7.625511342	0.103280306
MMD2	-0.042140068	5.925545458	0.103288551
LOC652667	-0.040706955	6.074731856	0.10329017
DLST	0.127194261	8.994299965	0.10351899
GML	-0.067242176	6.095808488	0.103538324
DES	0.106705627	13.38668345	0.103568606
LOC649583	-0.040814367	6.085187354	0.103569824
LOC441081	-0.096297953	6.464154158	0.103590562
FLJ11783	0.057268151	6.207881435	0.103644082
C9ORF126	-0.041954095	6.060673582	0.103668786
SNHG10	0.069973229	6.443850396	0.103697212
LOC649975	0.044360922	6.113675998	0.103745661
MGC16075	-0.039835126	6.0370686	0.103749821
LOC646982	-0.037505914	6.091536888	0.103764247
GPR133	-0.040305201	6.131868718	0.103830325
ITM2A	0.11116429	10.00898414	0.103868302
LOC654187	0.046702962	6.217644099	0.103970592
C5ORF48	-0.042042795	5.938737584	0.103995523
MED15	0.04212439	6.642663726	0.103996592
KRTAP10-11	0.063645043	6.923049508	0.104021864
TFAP2A	-0.03991037	6.539102586	0.104060796
CCNT1	0.049353292	6.724176123	0.104066555
OR5AP2	0.045740467	6.045222641	0.104112108

PFKFB1	0.043329809	5.983802455	0.104134403
C2ORF52	0.030471754	6.155295707	0.104145706
LOC646036	-0.042876809	5.953356116	0.104152827
LOC439992	0.045510041	6.092814843	0.104175884
GRAMD1C	0.045795477	6.08810988	0.104286774
LOC651894	-0.100362994	11.63489952	0.104312726
KCNE4	-0.047871228	6.039818924	0.104338646
LOC286411	0.032644031	6.140420966	0.104396266
GPR115	-0.043575465	5.969140796	0.104450796
GPR20	-0.046888462	6.204209021	0.104459795
HERC2	0.060835881	8.420777901	0.104518131
SLC27A5	0.06969127	6.448549308	0.104518887
C9ORF144	-0.048660461	6.081854685	0.104652722
SRP19	-0.095609587	8.798078622	0.104662906
LOC401321	-0.092022462	7.368410208	0.104674367
TSEN34	0.066335518	9.284004438	0.104798429
RBM15	0.070056555	8.555704103	0.104827212
C1QTNF4	0.054333377	6.325782155	0.104850431
CHRNA6	0.047128348	6.506601933	0.104858608
TCN1	-0.046381038	5.943901646	0.104870629
ZNF562	-0.084522793	7.68702732	0.104918645
BCL2L11	0.025769773	6.081507169	0.104946788
ANKS3	0.04895793	6.453044623	0.104964438
LOC90835	-0.05539359	6.475916108	0.104976455
LOC643690	0.044628202	6.047864248	0.104992778
LOC650546	-0.025435189	6.158445918	0.105086951
SARS	-0.099942204	8.24431808	0.105211655
LOC729764	0.126892572	7.40429551	0.105215906
BIRC7	0.037799718	6.087257067	0.105399085
LOC441178	-0.040440643	5.930468513	0.105430026
CRAMP1L	0.057310375	7.488954947	0.10559211
LIPC	-0.032300049	6.04284187	0.105664347
HECTD1	-0.078652361	10.50374964	0.105711339
ANKRD58	-0.066930549	6.001704676	0.10573862
PTPN23	-0.049751338	6.574238641	0.105882829
PLUNC	-0.042050444	6.008772079	0.105933269
LOC648293	-0.031731328	6.119040688	0.105967792
HTR1B	-0.039351789	5.900813968	0.106040299
ZNF45	0.052802401	6.829110474	0.106081797
C14ORF177	-0.042099401	5.99738533	0.106134267
TMEM55B	-0.069832455	7.734851791	0.106160945
OR5T1	-0.038339543	5.869110492	0.106172867
FAM53C	0.078939712	9.496366939	0.106258382
ADAMTS3	-0.064370019	6.013181343	0.10627825
ACSS3	0.058956624	6.183391467	0.106278347
TAAR2	-0.04858651	6.142845652	0.106336326
ART3	0.134870849	10.80895139	0.106382242
LOC388177	0.036033272	6.114067507	0.106417243
GIPR	-0.040903491	5.94127019	0.106496225
LOC652543	-0.045335277	5.960215409	0.106664781
STRAP	-0.129189081	9.801442189	0.106720941

LOC650140	-0.045711123	6.06280634	0.106742164
TREX2	-0.040656032	5.972106093	0.106777544
MSH6	-0.09412411	9.220761974	0.106855349
IQCD	0.042127266	6.086016326	0.106885642
FLJ45300	0.041872714	6.097023352	0.106888632
LOC653451	-0.035479256	5.976830564	0.106912639
PCCA	0.116737452	9.04707856	0.106929991
SFN	-0.038665278	5.964504916	0.106931328
C4ORF35	0.043213518	6.038851768	0.10697165
CCL3L3	-0.089601423	6.314177302	0.107017763
ATP9A	0.086320769	8.878130946	0.107041838
CA2	-0.100051364	8.518527092	0.107204482
SDHC	-0.093654517	9.055690822	0.107283521
APLN	0.115481244	6.801595336	0.107334221
TRY6	0.061429543	6.017841335	0.107474856
C1ORF109	-0.057163189	7.139668808	0.107545738
LOC645609	0.081942627	7.467541045	0.107563326
LOC641849	0.113874924	7.465289921	0.107599988
DEGS2	0.039073685	6.049755197	0.107624911
MST4	0.060260306	6.896171885	0.107641731
ZNF90	-0.037955096	5.987927906	0.107669275
LOC729185	0.03806078	6.073461617	0.107793101
HIST3H2BB	0.038135889	6.152570512	0.107862003
FSD1L	0.042175626	6.35647548	0.10802792
EXO1	-0.026781063	6.061464094	0.108080851
LOC339692	0.046310031	6.267648118	0.108186545
LOC645590	-0.034894759	6.11390921	0.108236187
LOC644423	0.052809594	6.212181439	0.108245635
KDELR1	-0.068863152	9.284554668	0.108251954
MRC1L1	-0.045576628	5.996030342	0.108279601
ZAP70	0.029893474	6.167959547	0.108289254
STAT2	-0.090755774	9.637439541	0.108294045
LOC727761	-0.063802696	6.942101189	0.108395803
SNORA65	-0.037000191	6.240733084	0.108396894
CT47.6	-0.031733839	6.097727264	0.108398359
CNGB1	0.05334629	6.42791429	0.108410284
DDX19B	-0.046563389	8.036033804	0.108519098
LOC647038	-0.038202698	6.026981434	0.108543606
LOC643922	0.052146301	6.064804565	0.108612397
DEDD2	-0.089949834	8.507733728	0.108628162
DDX60	0.071535249	7.067541814	0.108645285
MCTS1	0.155457748	8.08111124	0.108845712
WDR93	0.037406932	5.967508661	0.109010911
MTF1	-0.060583107	7.323169135	0.109046532
PKMYT1	-0.028210939	6.048133127	0.109066597
LOC91664	-0.047200118	6.326901927	0.10907079
ACVR1C	0.043689679	6.01699108	0.109113723
C16ORF7	-0.064029826	7.658138029	0.109118966
FBXO25	0.032506397	6.304606458	0.109151916
KRTAP13-4	-0.041409533	6.106589258	0.109195032
LOC440361	0.043916414	5.947141324	0.109251041

LOC642820	0.043917213	6.149061157	0.109268752
GJA3	0.091237681	7.63515526	0.109349065
LOC388161	0.031043772	6.179605162	0.10935211
NAG	0.090338033	7.659007637	0.109359273
POLR2J4	-0.022443804	6.200718053	0.109401748
LOC644519	0.045539693	5.973704482	0.109448682
COPS3	-0.062796313	10.70014665	0.109488613
RFXDC2	-0.045984668	6.230211763	0.109530103
ZNF642	0.041179518	6.253562324	0.10955073
LOC645342	-0.039547875	5.916752453	0.109591458
LOC492303	-0.046750427	5.993521025	0.109660079
LOC390688	-0.043836589	6.12470585	0.109708498
CSF3	-0.027014256	5.993176236	0.109732554
LOC652051	-0.062710043	6.018883885	0.109742879
MIZF	0.039456642	6.853544957	0.109769152
TAS2R45	0.045372517	6.102559961	0.109901944
CCDC88A	0.059327691	7.876866099	0.109965305
MBL1P1	0.048149106	6.414086441	0.109989493
AICDA	0.04932348	6.076365125	0.109997414
RREB1	0.031303481	6.371617179	0.110008229
LOC646482	-0.038430344	6.108666706	0.110017663
ARTN	-0.022710456	6.032287267	0.110046822
CDC45L	-0.05672712	6.196039432	0.110054782
ZNF330	0.092259335	10.05425644	0.110079509
FLJ14166	-0.055049979	6.174867847	0.110130875
ZNF121	-0.050804278	6.110540568	0.110162057
LOC653057	0.043640334	6.099199154	0.110182096
SLC45A4	-0.063327119	7.273986966	0.110232717
VPS33A	0.049613288	7.084466637	0.110242213
ASB6	0.04218191	6.573983699	0.110263172
SLC26A11	0.02800415	6.260782916	0.110306164
CYP4Z1	-0.020090488	6.010751615	0.110344531
LOC652767	-0.042314815	6.010979631	0.110462701
CDH24	0.028186071	6.100591409	0.110583789
USP29	0.043118696	6.004514433	0.110647914
ZNF268	0.029377644	6.266915338	0.110778562
LOC643008	0.041744527	6.30157241	0.110784871
GATA3	0.04266917	6.215872105	0.110848137
LOC652726	0.08499224	6.571190685	0.110943403
MPV17	0.08614595	8.675292767	0.11096993
MAT2A	-0.100681328	11.36558707	0.111016214
TNRC4	0.04065544	5.959139512	0.111046754
KHDRBS1	0.065036084	10.62814279	0.111055166
PPM1K	0.105381839	8.5294008	0.111071322
PDGFA	0.043951936	6.355012556	0.11109544
UBE2D2	0.041698774	7.399576693	0.111135725
GPR21	0.040448677	6.22725644	0.11115334
FLJ42133	-0.043802481	6.101423801	0.111231587
PRKCA	0.10993043	7.730923047	0.111346827
STX10	-0.069950434	8.650569908	0.11137208
LOC440297	0.067199279	6.188310719	0.111391127

FCRL5	-0.030910728	6.183868504	0.111402855
LOC649057	0.037580184	6.068033244	0.111416454
CLSPN	0.054365345	6.105443375	0.111483155
HIST2H2AC	-0.107934947	8.783678945	0.111534625
LCE3A	0.049806925	5.925568008	0.111537495
LRP12	0.05467764	6.589618292	0.111537519
LOC143543	0.073403687	7.33900618	0.111547288
ABCF3	-0.06789146	7.15992777	0.111576932
REEP6	0.06510881	6.827576488	0.111618741
LOC374491	0.035869203	6.171206148	0.111625826
DDEF2	0.109851065	7.600440908	0.11164339
LOC400958	0.047029753	6.510365801	0.111666958
LOC652606	-0.049414754	6.018045835	0.111728378
LOC729751	-0.029895454	6.072557625	0.111781231
TMC8	-0.043681801	6.066877226	0.111817444
RAB1C	-0.038897504	5.99207371	0.111913499
LOC285141	0.045864068	6.136117599	0.111920499
ZNF263	0.087230146	8.047778989	0.111937936
ICMT	-0.076137411	7.801217167	0.111988993
RASGRF2	-0.043791038	6.054041061	0.112037699
ZMYND10	-0.042517856	6.06187892	0.112065377
LOC728876	-0.053069553	6.088388018	0.112090646
LOC651357	0.035582725	6.04707648	0.112109821
EDEM1	-0.106239611	6.714907916	0.112112383
BTN3A1	0.051645475	6.608207384	0.11240612
IL5RA	-0.033023676	6.32864341	0.112437727
SLC6A17	-0.044172579	6.056438445	0.112569895
C20ORF82	0.048442348	6.337079651	0.112579634
RRM2B	0.089624191	7.139920204	0.11258065
DENND4A	-0.036049081	6.461236426	0.112625651
GPR52	-0.031973556	5.970240335	0.112682191
PIK3R5	-0.049788653	6.272244239	0.112791552
SPSB3	-0.084718302	9.924435126	0.112974367
XPO7	-0.063912334	7.664474586	0.113045849
CALM3	-0.098220818	11.61666444	0.113049064
YBX2	-0.039979769	6.24105591	0.113053341
LOC115648	0.049005566	6.257482802	0.113079843
CLSTN2	-0.051396358	6.484741771	0.113104091
SLC4A10	-0.040706543	5.963214718	0.113133283
NECAP1	-0.065876884	8.822155014	0.113224309
DAZL	-0.028330954	5.971459685	0.11334104
LOC652197	0.037175889	6.05485711	0.113397838
DCD	0.033169502	6.065174986	0.113442753
LOC144776	0.036959986	6.187191189	0.11349201
FAM106A	-0.029128933	6.038354604	0.113492674
POLR2H	-0.1234449	8.843888696	0.11357951
MAMLD1	0.095041762	6.678184156	0.113598038
DCST2	0.042366786	5.932613229	0.113623078
EN1	0.038825766	6.161572398	0.113631107
LOC653262	0.03929505	6.155323681	0.113635922
CDCA8	-0.040391578	6.237576311	0.113638333

LPHN1	-0.050079511	6.689136317	0.113644347
PASD1	-0.039486425	5.986495077	0.113725581
TWF2	0.114712912	9.440031152	0.113742466
SURF1	0.076657153	9.033818927	0.11376273
NLF2	-0.078301382	6.478341603	0.113773317
C14ORF112	0.079569208	9.489471889	0.113795841
TBXA2R	-0.032761737	6.139964398	0.113824566
BCL8	0.038977977	6.014505451	0.113866774
LOC390192	0.05180131	6.111846004	0.113957593
TAS2R49	0.040191375	6.152569472	0.114048211
ANKHD1	0.045918658	6.875518491	0.114102199
ZCCHC17	0.05959738	8.302805846	0.114103743
PSMA1	0.085970893	10.00619442	0.114153381
GOLGA5	0.067375974	8.592099681	0.11416318
LOC728518	0.073039371	6.22816905	0.11417523
CCDC82	0.037778945	6.030083482	0.114219682
ADCY2	0.048847859	6.604756977	0.114221954
COX19	-0.07383108	7.857264799	0.114235891
LOC387804	-0.032559781	6.082179723	0.114259848
ATG12	0.067097177	8.277029321	0.114340085
NUDT17	0.042313971	6.182687113	0.114378509
CT45-1	-0.020518217	5.98153032	0.114544392
LOC653604	0.032732523	6.182422663	0.114606718
REEP4	-0.041945964	6.414270594	0.114607726
DOK4	0.070042721	6.94609855	0.114634755
PIR	0.065198518	7.339664238	0.114654823
BAMBI	0.140536604	10.30954947	0.114668613
LOC652722	0.038891156	6.054730298	0.11486449
LOC654135	-0.05130329	6.388780797	0.114915402
FBXL15	0.051968396	8.050966583	0.114959017
LOC389669	0.03697524	5.905459962	0.115033396
FKBPL	-0.047650858	6.297540177	0.115226194
SLC30A3	-0.126314326	8.354411059	0.115255606
HIST1H3J	0.04351293	6.103305352	0.115273341
TYW3	0.098950534	7.690880953	0.115343203
ADRA1A	-0.03416578	6.302073733	0.115382328
LOC644556	0.038562558	6.111491917	0.115388415
GTPBP10	0.036939621	6.252367549	0.115428024
TTC31	0.048917099	7.771400597	0.115435844
LOC643980	0.032548849	6.081953108	0.115533404
LOC647065	-0.038645222	6.031771135	0.115555649
ZBTB26	0.042305274	6.088970877	0.115602487
XRRA1	0.050110972	6.189800226	0.115622784
LOC643403	0.050360526	6.133540502	0.115642699
NPAL1	-0.043563089	6.042710448	0.115684275
TPH2	0.042239856	6.020851191	0.11569634
PDE11A	-0.024175405	6.016574214	0.115712353
LOC651059	-0.034114417	6.046173785	0.115777304
LOC647339	0.03797586	6.042852327	0.115805087
LGALS12	-0.048801808	6.243178539	0.115805391
C6ORF78	0.039959583	6.200390849	0.115824874

MLF2	-0.066559273	9.11334807	0.115888318
HIST1H2AI	-0.061557224	6.578485604	0.116008866
LOC648526	0.050981299	6.184952066	0.11606098
GFRA1	-0.02915092	6.160672185	0.116097173
LOC653059	0.057201107	6.183069754	0.116107721
FLJ25778	0.061182987	7.18144276	0.116144071
PRC1	0.062652569	7.036295419	0.11615057
PM20D2	0.071556707	7.011558544	0.116270104
RAP2C	-0.079592529	7.354881028	0.116276705
MGC39900	0.031879749	6.348293023	0.116316246
SV2C	0.041801051	6.066960882	0.116353256
INSRR	0.04030146	6.066300079	0.116371921
LOC653733	0.039450897	6.043739315	0.116434141
KHDC1	0.05868081	6.31549916	0.116464594
MTHFSD	0.049358285	6.680005477	0.116642702
XAGE1E	0.048128246	6.146148799	0.116653511
BTLA	0.031780626	6.230443534	0.116688455
PC	-0.030439512	6.222321512	0.116760593
LOC653420	0.043786532	6.00654566	0.116787771
ZIM3	0.044080665	6.062140864	0.117079705
LOC642778	0.040383067	5.867828984	0.117302344
USP4	0.04609481	7.598161493	0.117358723
LOC645851	0.043986123	5.974959882	0.117370425
LOC730919	0.044880506	6.168812896	0.117443246
GOT1L1	0.042413905	6.045634877	0.11744403
CDKN3	-0.064708421	6.474651677	0.117539713
LOC388122	-0.042008372	6.370552922	0.117553671
CASP2	-0.058569009	8.251245555	0.117597985
HSPC152	0.077418014	10.50906082	0.117618872
ANKFN1	-0.053614995	5.885196857	0.1176908
LOC647269	0.038090269	6.087539447	0.117704972
LOC440313	0.045705719	6.200244822	0.117737793
PPP2R5D	0.046422574	7.156383684	0.117815538
STX3	-0.059091836	7.723722338	0.117834675
RDX	0.072317942	8.909298552	0.117835187
LGALS9C	-0.051981919	5.76170589	0.117987708
PDLIM7	0.128218331	9.489935365	0.118003441
RCAN1	0.152346042	8.886962302	0.118010943
LOC643226	-0.0256414	6.092826562	0.118030181
CAPN13	0.055937479	5.930938473	0.118051146
IL7	-0.032603933	6.016742569	0.118096682
SMU1	0.102528789	7.033621116	0.118127067
ACAP1	0.052594452	6.566166921	0.118169791
TSGA10IP	0.041635485	6.179812986	0.118186116
ADIPOR1	0.064238581	9.034006028	0.118237074
CYCS	0.095923386	9.102319026	0.118346666
FLJ12949	0.05041626	6.837732842	0.118383425
BCL6B	0.152658684	6.890849756	0.118395734
LOC93463	0.038862687	6.046518228	0.118442312
BTBD9	0.069435992	7.482447063	0.118449393
TSEN54	-0.051722564	6.558605772	0.1184964

SLC9A3	-0.044727829	6.0317772	0.118531878
LOC642468	0.035873552	6.362303583	0.11861854
LOC441956	0.025978484	6.125399127	0.118633108
RAI14	-0.164499835	8.558559738	0.118645015
TTTY21	-0.031904646	6.029018248	0.118832311
RASGEF1B	0.053971512	6.342761157	0.118852941
LOC646508	-0.044215221	6.674317652	0.118893409
LOC643962	-0.026603901	5.982628969	0.118916901
LOC641989	0.041714082	6.453232089	0.118988523
C12ORF59	-0.046919021	6.001595632	0.119074091
GP5	0.046476156	6.025547148	0.119134298
LOC645981	0.037304967	5.990536331	0.119159267
KLF3	-0.032034809	6.167497878	0.119170571
NTF3	0.073132595	6.639803399	0.119250024
UCKL1	0.080461138	9.727488369	0.119293778
LOC730686	0.043355075	6.266157855	0.11930897
CD83	0.056764131	6.650308687	0.119309784
BTG4	-0.036555476	6.003741874	0.119353045
MGC29506	-0.05694813	6.341757806	0.119493265
ARRDC1	-0.044588279	6.256578171	0.119508198
LOC645732	0.035903784	6.13495937	0.119518464
EVI5	-0.089661332	8.308638073	0.11967315
LOC440926	0.058254361	11.59835421	0.119699321
FAM155A	-0.036426067	5.955960732	0.119702181
OR51S1	0.041860869	6.012788368	0.119770763
ZNF699	0.033412079	6.565141775	0.119806146
SNORD15A	0.043547215	5.962167277	0.119829155
EHMT2	0.033502455	6.251740244	0.119838616
HMGN1	0.081639677	10.41684061	0.119897055
UTS2D	0.027660594	6.047997336	0.119907745
RSPO2	0.033092743	6.464304645	0.119941812
RPL41	-0.080689797	13.29328618	0.120008711
PDZD7	0.037844755	5.935108353	0.120110757
MUC12	0.030843062	6.198810245	0.120122727
LOC400831	0.043590433	6.295037903	0.12023149
OTOR	0.031210493	6.093766881	0.120286748
DEAF1	0.035663289	6.575404106	0.120347482
SEC16B	-0.052005532	5.991374258	0.120380928
KCNA10	0.034387239	5.934498343	0.120394274
LOC644919	0.048741154	6.346747677	0.120415185
USP19	-0.046602855	6.363711434	0.120423855
BPTF	0.039595995	6.762534803	0.120471883
NXPH1	0.040298158	6.411811814	0.120475215
LOC647022	-0.041781872	5.962261921	0.120483301
C12ORF53	-0.042929561	6.034289055	0.120512423
TTC21A	0.046199163	6.286640319	0.120552525
MPO	-0.038728919	6.043962846	0.120566461
LOC653419	0.036662554	6.167581427	0.120603206
LOC728351	-0.040838921	6.211599739	0.120616138
MSI1	0.044323859	6.337069351	0.120689018
WDR57	-0.062847498	7.974102689	0.120698368

GZMA	0.078585317	6.590274981	0.120710149
TRIM67	-0.046427115	6.169757733	0.120729971
UIMC1	0.065657456	10.15304989	0.12081991
TMEM26	-0.05226185	6.271991389	0.120830619
LOC648196	-0.040779145	6.205013333	0.120897551
SETX	0.082775535	7.182941718	0.121060824
SPRY4	-0.06272993	6.963567586	0.121075272
TRIM34	0.02210935	6.01568801	0.121105336
C1ORF120	0.034869498	6.084261549	0.121139231
LOC644250	-0.13412932	9.222428482	0.121143317
ADAM17	-0.040387416	7.028430053	0.121210372
LOC648622	-0.090581974	11.64074147	0.121432805
METTL2B	-0.045782123	6.273746904	0.121530515
LOC653734	0.039127367	6.01498265	0.121555968
LNPEP	0.042881716	6.896754877	0.12157773
ABCA4	0.062909545	6.433252099	0.121673544
LOC646428	-0.040039182	5.984615117	0.12168422
GJB3	0.024733702	6.066878159	0.121801632
LOC643626	0.042325693	5.993023004	0.121829635
DOM3Z	0.05616012	7.478213304	0.121832969
TESK2	0.049453428	6.481447242	0.121888487
CEACAM21	0.050012377	6.375306775	0.121890795
C7ORF45	0.046973246	6.21794117	0.12220409
LOC727759	-0.039001946	6.322620615	0.12226138
GABPAP	0.018195172	6.133327714	0.122309071
MGC10701	-0.033741543	5.986432989	0.122500801
HEL308	0.043817005	7.015339106	0.122612839
PPP4R1L	-0.027025605	5.981000972	0.12261847
A2M	-0.130942234	10.8434455	0.122653718
LOC641955	0.037741802	6.121116521	0.12276904
LHX1	-0.037937718	6.314358431	0.122793579
LOC732316	0.043699018	6.057616551	0.122801539
CIZ1	0.042334144	6.372690307	0.122806224
RNF39	0.034317857	6.118738851	0.122818085
KRT6A	0.042470903	6.042009981	0.122840746
LOC645168	-0.044182137	6.238579031	0.123035259
EXPH5	-0.040099954	6.127245998	0.123076538
LOC653495	0.042506303	5.932192784	0.123106752
BLCAP	0.107450904	8.870277068	0.123204217
PPAP2C	-0.027875025	6.137385918	0.123281634
LOC401233	0.138559456	6.906201442	0.12328492
RAB11FIP5	-0.098730731	8.583683546	0.123305594
ATP5C1	0.093981197	12.16534656	0.123324845
PPP1R14D	-0.045247418	6.421038218	0.123339067
ACP1	0.08967374	10.01496564	0.123408895
LOC391157	-0.039185175	5.962185291	0.123409641
PLA2G4A	-0.041885317	6.200441526	0.123428316
LOC645302	0.033291555	6.029804073	0.123437388
DIRAS3	-0.093455729	6.646212461	0.123499267
TCF7	0.019049785	6.046336202	0.123515883
PRLHR	-0.036006183	6.022549105	0.123547073

LOC253724	0.025146265	5.967393494	0.123605503
LOC338799	0.039083987	6.256382076	0.123639322
RPL29P2	0.050146211	6.580604682	0.123652013
SON	0.033886507	8.403638616	0.123689412
FUK	0.057766921	7.85094944	0.123710496
KRTAP12-1	0.041388679	6.208575446	0.123730164
LOC284757	0.181289215	7.027492755	0.12375665
RPL13	-0.125453062	8.418801711	0.123756809
PRG2	-0.034788565	6.131182194	0.12395235
ZNF486	-0.122830005	12.17336425	0.123957806
LOC644120	-0.037277243	6.064659633	0.123967675
LMLN	0.053679012	6.320602494	0.123994539
LOC202459	0.047115742	6.405182356	0.123997532
SLC12A1	-0.038425293	6.03864971	0.124033396
BARHL1	0.039289597	6.062976601	0.124110558
LOC649365	0.061243247	6.422469328	0.12413183
ZCCHC13	0.038154422	5.966009564	0.124224446
LOC644334	0.035042961	6.183056925	0.124284908
CNGA1	-0.057760005	6.226599457	0.124340447
STK25	-0.074829604	8.578838483	0.124369241
CHR415SYT	-0.047431799	5.961045465	0.124414208
SLC6A2	0.045432228	6.313207244	0.124450339
C7ORF42	-0.066815574	7.83615457	0.124464339
NT5C	-0.066318139	9.832159872	0.124490624
MYL2	-0.115462683	14.76423862	0.124509088
LOC283202	0.02771754	6.102358744	0.124556221
TSPAN16	0.034615975	6.081333631	0.124561608
LOC643847	0.027734103	6.027432693	0.124612499
LOC650122	0.039693482	6.054197632	0.124627054
PEX26	0.030147865	6.208366981	0.124698652
POLD4	-0.071108605	7.225851356	0.124712341
KRTAP10-1	0.038539592	6.00218996	0.124739281
FLJ34931	0.060894464	6.320938266	0.124752578
LOC646366	0.025304225	6.035147987	0.124763125
CTXN3	0.03025439	5.977841749	0.124771485
LOC283788	-0.038631132	5.979086303	0.124777797
CRYGA	0.036408278	6.36694821	0.124830188
LOC648509	-0.071356198	6.705396911	0.124845037
PCDHA12	0.026797171	6.02182515	0.12490117
SPACA1	-0.039939812	6.239427141	0.124919949
LOC643253	-0.042933626	5.935255113	0.12504328
PIWIL3	0.040053419	6.011023758	0.125043507
LOC643235	-0.039274535	6.062884145	0.125073733
HOM-TES-103	0.045171971	6.311519362	0.12514067
SNAP29	-0.060695404	7.667778078	0.125155795
UBE2G1	-0.07605212	9.298915012	0.125183723
LOC644362	0.039148223	6.100113006	0.125190562
RCVRN	-0.045390139	6.180665526	0.125207076
OR2Z1	0.042578744	6.175447818	0.125230117
AK5	0.027046498	6.015276048	0.125232912
ABCD4	0.022771914	6.135292078	0.125280823

CLEC17A	0.039228567	6.026317831	0.125363854
MYST3	-0.076937265	8.639674415	0.125419214
RBBP5	0.048329947	7.199962296	0.125481882
PAPPA	-0.10870441	7.161408055	0.125499839
FLJ40852	0.040956078	6.410313019	0.125514234
OR3A3	0.037138006	5.943102668	0.125515656
LOC643581	0.035092135	6.11645393	0.12552993
GSTO2	0.052489587	6.448444437	0.125575684
LOC440080	0.021032639	5.991001243	0.125619445
LOC652571	0.033617125	6.07147678	0.125654605
SGSH	-0.079761034	8.07226696	0.125718636
WNT11	0.045775513	6.309519055	0.125753384
RPL11	-0.060935698	13.46343954	0.125805878
LOC652011	-0.038066618	5.903336386	0.125910384
EIF2AK1	-0.05673374	10.11663517	0.125914539
FLJ46838	-0.034129113	6.32140057	0.125941608
FLJ20309	0.040281628	6.141825382	0.125958163
GPR109B	0.025572922	6.22868175	0.126004576
KLHL32	-0.040346675	6.032033229	0.126077473
KCNAB2	-0.028049082	6.200684676	0.126092597
GFRA3	-0.060277197	6.460678475	0.126114057
UNQ9433	-0.043950405	5.97620521	0.1261257
LOC645705	-0.042281175	5.982407167	0.126180999
LOC647286	0.025456002	6.022743278	0.126292594
LOC644973	0.038323806	6.110583602	0.126326788
CXCR6	-0.040117359	6.071180605	0.126364649
GSS	-0.063615964	7.626413452	0.126379132
LOC641694	-0.046132804	5.901098494	0.126396004
LOC642891	0.039501807	6.05334533	0.126419525
CRIP2	-0.092608011	11.96823486	0.126421963
KIAA0090	-0.064782675	8.131483014	0.126425751
KRT24	-0.036938579	6.318521845	0.126605337
DKFZP686C2281	0.039959319	6.03375015	0.126620472
CHST4	-0.036702522	5.991831404	0.12665683
RNF185	0.053192071	6.916824775	0.126659434
TADA3L	0.039906781	7.104726365	0.126661037
OR14J1	-0.03771188	6.062342923	0.126714048
ZNHIT2	-0.071511755	6.760033872	0.126724052
LOC728564	-0.068761724	8.309729327	0.126730581
NKG7	0.102276872	6.774771448	0.126746302
HMGB3	-0.052293131	6.512969783	0.126791247
TP53TG3	0.050555615	6.264234552	0.126814873
LGALS2	0.067703121	6.214514916	0.126920243
LOC728729	-0.037713448	5.984809811	0.127016874
LOC730432	0.067279974	8.981260404	0.127275132
PRR12	0.041222689	6.280054286	0.127275767
RAF1	-0.055206661	9.870499811	0.127276508
SPATA19	0.046848737	6.072836516	0.127301201
SYN3	-0.030091248	6.040997264	0.127302242
CXORF50	-0.036145074	5.982924443	0.127375558
CLEC4F	0.041864756	6.089729826	0.127397442

PCDH24	0.04442174	6.561127869	0.127474967
LOC642644	0.055232772	5.990386259	0.127497168
ABCC6	0.022027265	6.089249249	0.127526608
LOC644008	-0.04090792	5.963283147	0.127716152
LOC440820	-0.046146248	5.952550349	0.127730084
ZIC2	-0.040272886	6.083396456	0.127731267
SPATS1	-0.040300731	5.917942581	0.127750596
COX7A1	0.071199652	14.14286209	0.127756748
NLRP9	0.037052736	5.964832646	0.127758869
LOC646243	0.028132057	6.092198496	0.127805625
LOC653609	0.037854256	6.018834881	0.127870424
TBKBP1	0.042340693	6.342567738	0.127904142
MED30	0.071904487	8.500911209	0.127916823
C1ORF106	0.067001224	6.450285549	0.128036962
TMEM132C	0.033803632	6.067883333	0.128086257
ZIC5	0.039570952	6.195091995	0.128115785
MRVI1	-0.034799226	6.190191721	0.128151935
ZWINT	-0.023589314	6.087292153	0.128325032
SBNO1	0.04784953	6.374025839	0.128345364
SSX1	0.02454474	5.991319128	0.128385378
ELK3	0.038975886	6.250220394	0.128473744
SAC3D1	-0.070919015	7.064516879	0.128500489
RSRC2	0.068969824	8.347963311	0.128577955
LOC642290	-0.03847224	6.148689687	0.128632996
ADAM2	0.046744825	6.253645307	0.128757379
RCHY1	0.046161939	6.516684201	0.128789659
REXO4	-0.106061664	7.636344455	0.128814746
C19ORF36	-0.027182931	6.108997848	0.128873637
GPR35	0.042454602	6.036860262	0.12923187
KIFC2	-0.083677627	6.567942931	0.12925572
PKD1L3	0.046835156	6.129942454	0.129302113
FAM24B	0.028590338	6.224116321	0.129363572
SCGB1A1	-0.044337326	6.055362133	0.129389439
HPGD	0.060585128	6.402037	0.129488426
OSBPL6	0.045428076	6.715278247	0.129488477
MGC39372	0.065736573	6.300483912	0.129533993
GABRA4	0.038236038	6.111042348	0.129538114
RIMS4	0.040520604	6.373992945	0.129624128
LOC643558	0.020173317	6.17134832	0.129698581
EIF2C3	0.030940834	6.457617622	0.129770133
LOC647947	0.038263887	6.29686518	0.129919705
RGS14	0.040924491	6.358460605	0.130028603
C6ORF206	0.037842307	6.016270924	0.130211461
LEMD3	0.066100762	8.588787905	0.130217905
H3F3A	0.046598186	12.63510353	0.130252014
FLJ43950	0.021276856	6.158244885	0.130305174
FAM166A	0.040546478	6.107034149	0.130345595
PRR11	-0.043982805	6.080818848	0.130353233
KLKB1	0.086093653	6.524014764	0.130373854
WDR63	0.046927909	6.0848446	0.130375451
LOC642716	0.036779388	6.16135595	0.130417669

LOC642954	0.035142226	6.112940572	0.13042972
SCRT1	0.025245456	6.208419188	0.130452506
RPL36	-0.083861194	10.58499931	0.13047637
FLJ38969	0.03742496	6.16701904	0.130511114
MMP19	-0.025242605	6.132408337	0.130544472
GABRA1	0.03654055	6.008990452	0.130547837
ENTPD2	0.033184186	5.975006548	0.130568049
TSSK2	0.042182286	6.00654615	0.130588509
HOXD3	0.04949477	6.763423192	0.130706869
LOC644725	0.041929054	6.252989133	0.130774709
EFNA4	-0.042847544	7.147894316	0.130800957
C12ORF35	0.055175915	7.042552725	0.130848239
CHEK1	-0.037085103	6.360727168	0.130934534
C20ORF152	-0.044124352	5.964795871	0.131049004
ZFY	0.074903938	6.249961058	0.131132608
LOC647274	-0.035754474	6.192102751	0.131140213
SAMD10	-0.042585384	6.25091324	0.131249615
LOC652209	-0.037538603	6.001579845	0.131337722
FAM83E	0.03985842	5.958004263	0.131374273
LOC389102	0.036718183	6.333707852	0.131431977
NDUFA12	-0.072416985	12.08638643	0.131441474
C20ORF94	0.051281177	6.719812923	0.13145305
C14ORF100	0.060425159	8.216776053	0.131480535
LOC646717	0.035184321	6.067371581	0.131485423
SEC62	-0.054254756	7.251980717	0.131497445
BRCC3	-0.049675551	6.902759229	0.131600577
CICE	-0.068484381	7.768665931	0.131738213
LMX1A	0.020811715	5.987725094	0.131780083
LOC649199	0.040956304	6.05612526	0.131841998
OR5K2	-0.042531427	6.116411422	0.13188046
LOC441326	-0.030527685	5.994983442	0.131900087
LOC642250	-0.13220677	11.33360036	0.131921126
NLP	-0.061479491	6.38728078	0.132007564
CCL3	-0.049439679	6.166446765	0.132056083
LOC647466	-0.036212855	5.960575773	0.132101165
LOC641716	0.035711192	6.161081153	0.132188
NFATC4	-0.041580649	6.134748458	0.132191076
SNORD14A	-0.114548637	6.819038297	0.132237641
FOXP2	0.031074397	6.089639931	0.132337903
LOC643389	-0.038267149	6.227436132	0.132548405
PCM1	0.079334909	9.657624637	0.132608159
TXNDC2	0.025647503	6.087890326	0.132668505
ATP5O	0.07090392	13.03953181	0.132669108
KRT75	0.041230078	6.028578961	0.132706932
LOC653463	0.040535296	6.249015228	0.132714697
GDAP1	0.030867644	6.076346203	0.132755329
CARD14	-0.025696299	6.036879381	0.132864707
FAIM3	-0.107975161	7.215722636	0.132908031
TNRC9	0.039776613	5.856939309	0.132961386
LOC643482	-0.035032332	6.143536198	0.132999303
SLC4A9	-0.046771996	5.972224636	0.133015658

LOC644188	0.049064075	5.989034817	0.1330552
LOC646431	-0.040813522	6.100822596	0.133092174
LOC284067	0.039793721	6.197028467	0.133146268
C6ORF184	-0.023806228	6.06797648	0.133147143
NDUFB6	0.068548952	11.19616958	0.133155659
UBLCP1	0.082915383	8.446488879	0.133157444
CRKRS	-0.053339931	6.634664352	0.133263838
LOC729393	0.038020793	6.255870361	0.133362294
LOC653651	-0.043435573	5.966864534	0.133433231
LOC650721	-0.037014771	6.054511103	0.133503826
INSM1	0.040575683	6.527977443	0.133556632
MGC15705	0.037197729	6.105752565	0.133581417
MRLC2	-0.080067144	11.90742788	0.133696902
OR10AG1	-0.042308188	6.021460911	0.133788246
NSUN6	0.088000388	6.544196938	0.13383797
KCNN4	-0.068985682	6.241247928	0.133839994
KIF26B	-0.03826086	6.272816046	0.133881067
IPO8	0.143852819	7.746439948	0.133903654
LOC653781	0.035563735	6.328447896	0.134001395
LOC654109	0.056026345	5.930054139	0.134016028
CYCSL1	0.094639978	10.54982981	0.134076108
LOC196913	-0.039443898	5.943488343	0.134083492
LOC388923	0.032905195	6.067993355	0.134232584
POU2F2	-0.037516536	5.932141478	0.134258924
LOC145783	0.046263551	6.532311963	0.134345177
LOC652481	-0.097455789	8.533337415	0.13436166
GALNT12	-0.036061829	6.470160981	0.134404364
SEMA3D	0.037333082	5.940106828	0.134429641
PTPRK	-0.098726676	8.403370474	0.134515368
BSND	0.04473424	6.2671161	0.134515422
HIATL1	-0.034391909	7.036901807	0.134586569
LOC647566	-0.040092928	6.053160692	0.134594715
LOC442590	0.039355648	6.065149022	0.134603004
LOC648377	0.035597836	6.059055093	0.134633749
UNKL	0.030279626	6.356362155	0.134736546
FBXW8	0.034475531	6.68240849	0.134737626
ZNF215	-0.034153238	6.00992114	0.134791756
HAO2	0.03216695	6.011347755	0.13490679
LOC642969	0.041766294	6.321535002	0.134957985
SNX3	0.079043205	11.49117085	0.134982866
CDC42BPG	0.038584278	5.940819523	0.135026366
NTRK3	-0.029539508	6.174949286	0.135034884
FAM27L	-0.041947377	5.992202251	0.135302159
FLJ37078	-0.068451599	6.509590152	0.135421746
KLKP1	-0.042057792	5.996353709	0.135502857
KLC1	0.074543999	9.025228136	0.135531077
SH2D4A	-0.054532573	6.574122449	0.135600699
C5	0.051297873	6.959264134	0.135626177
ANAPC11	0.057838713	8.363677902	0.135629495
LOC651060	0.041623958	6.265526464	0.135672979
LOC285176	-0.078809149	12.79181912	0.135683986

HPRT1	0.05891999	9.006078128	0.135733782
OR5B21	-0.039112283	6.08418557	0.135748623
PSMB1	-0.079105028	12.07546041	0.13575578
SLC17A8	0.03785817	5.95777318	0.135769491
SULT1B1	-0.037152866	6.054930743	0.135846751
VDR	-0.031009804	6.001944667	0.135865737
ZC3H15	0.080134885	8.074148513	0.136019476
PTBP2	-0.059477256	7.690291427	0.136056628
TNS3	0.076418709	10.06307338	0.136058967
LOC650132	-0.048178317	6.133080184	0.136078616
GBA2	-0.049305689	7.293617459	0.136098954
SKI	-0.047340624	7.075175322	0.136200656
RNF148	-0.040381998	6.238489465	0.136264421
MRPL48	0.087297743	7.729874607	0.136303155
STOX2	-0.097670696	8.711040413	0.136321651
NLRP1	0.03323381	6.147923034	0.136368496
CLEC4C	-0.031568279	6.156409299	0.136401864
LOC728990	0.03760821	6.109707406	0.136435098
STRA6	0.034870988	5.996571434	0.136551936
RNASE8	-0.037211855	5.988257237	0.136569928
LOC644065	-0.040214129	5.925274361	0.136598482
NCOA6IP	0.04683369	6.858644811	0.136601082
FCRL2	-0.023894234	6.091930704	0.136646255
LOC643923	-0.028196908	6.07745434	0.136647279
AARSD1	0.075766796	8.524333152	0.136669087
C20ORF11	0.09969462	9.265982718	0.136678188
RIC8B	0.101905619	7.264224157	0.136776811
AKAP9	0.056885166	6.174485429	0.136815008
LOC647169	0.023325647	6.039274377	0.136845596
ANO4	-0.038521829	6.12912088	0.136853188
SEC31B	0.038062271	6.110490389	0.13722912
SNORD4B	-0.038762837	6.178266765	0.137286361
LOC649712	-0.03526152	6.199088175	0.13733014
C1ORF76	-0.042747546	6.533754029	0.137333082
SPINT4	-0.040483441	5.882100254	0.137508155
HCRTR2	0.042921787	6.290349912	0.137524326
RABL2A	-0.036887431	6.534115576	0.137589818
FGFBP2	0.142996512	6.947476946	0.137623858
LOC642903	-0.040998685	6.126587297	0.137629111
RPS16	-0.070159473	14.1664512	0.137643089
PHLDA1	0.210542352	8.876913244	0.137652913
SHFM1	0.067587048	10.92756332	0.137678924
EED	0.049490994	6.631985079	0.137679895
CD3E	0.035668407	6.196407608	0.137738616
TAAR8	0.050041688	6.197808395	0.137775381
SCGB1C1	-0.03382188	6.175602273	0.137807796
FAM123A	0.025787148	6.163824994	0.137812709
GNG4	-0.0365162	6.013945777	0.137820636
STOX1	-0.026203259	6.081750508	0.137854976
CDH2	0.074186362	13.15046374	0.137863914
LOC653092	-0.031924554	6.065291005	0.137909576

NT5C1B	-0.029949194	5.955398222	0.137920632
DUSP12	-0.060831901	8.265171648	0.137929656
KIAA1875	0.053114051	6.588997594	0.137963732
OR11H4	0.04040839	6.120700303	0.138074437
UMOD	-0.030876097	6.081089428	0.138099589
SNORD54	0.039356759	5.982075463	0.138152738
ZNF321	-0.029487222	6.120229184	0.138242089
CD79B	-0.069059772	7.402919968	0.138396248
LOC644799	0.125224537	9.139130228	0.13863336
SCIN	-0.040875751	6.004094559	0.138642711
LOC133185	0.040683204	6.336241062	0.138643596
CLEC6A	0.046984268	5.947618123	0.138731101
NUP160	0.050786114	8.072271949	0.138746808
EIF2B1	0.060259588	8.711729933	0.138946489
GLI4	0.047451402	6.581014073	0.138960312
KLRK1	0.047538692	6.232650688	0.139048147
RFPL3S	-0.027042812	6.043106413	0.139104162
LRRC41	-0.06497573	7.954109071	0.139227162
LOC130678	0.035347261	6.144197898	0.139245163
LOC442316	-0.036087733	5.980966034	0.139313808
MCHR1	0.049781485	6.047348707	0.139377247
ARX	0.036908174	6.214083987	0.139413562
LOC387856	-0.026617975	6.233074146	0.139470199
SCYL2	0.080692377	8.690181896	0.139542341
USP24	0.079735881	9.733045014	0.139594294
TMEM53	-0.048734054	6.430200101	0.139606727
LOC653663	0.022724948	6.124783151	0.139643477
UQCRB	-0.099278902	10.07682896	0.139707394
NPAS4	-0.03947399	6.018781426	0.139717669
LOC653103	-0.050215722	7.757068366	0.139739393
LOC646663	0.038931962	6.19930574	0.139743704
BEST3	-0.039027037	6.094246363	0.139798835
LOC652745	0.039726728	5.977721837	0.139909049
TUBGCP2	-0.07254715	8.818686457	0.140099048
LOC645126	0.034671046	6.06291253	0.140212083
C15ORF48	-0.022720013	6.014086729	0.140242285
APOD	0.213107016	11.89705196	0.140279455
MARCH7	0.099043563	10.38305007	0.140301539
GPR61	-0.042100287	5.981403673	0.140321911
PI15	-0.053994815	6.843149852	0.140374002
LOC376693	-0.039365856	5.9589771	0.140395468
LOC400752	0.033229814	6.032506159	0.140472461
LOC652439	0.035993117	6.014523778	0.140499741
LOC440092	-0.048178981	6.084773903	0.14052198
LOC647080	0.03775837	6.183690524	0.140590595
MRPL13	0.098204117	9.380659505	0.140617985
C20ORF38	0.03372305	6.134441279	0.140633619
LOC648133	0.03772607	6.109276973	0.14085687
LOC651829	-0.04211919	5.952268808	0.140859206
POU4F2	-0.037704673	5.983900617	0.140954209
OLFML2B	-0.089687493	6.469852013	0.140974178

LOC644167	-0.038847942	6.026213492	0.141041396
PKP3	0.034590809	6.096283518	0.141064033
DGAT2L6	0.038304907	5.993291914	0.141142612
TMEM45B	0.045396137	6.113117631	0.141161497
CCDC70	-0.043142282	6.105158839	0.141174582
TMEM4	-0.076302022	7.904078669	0.141223559
LOC646449	-0.030764817	6.105994621	0.141226901
ENOSF1	0.101158	8.002923367	0.141249691
SLC39A7	-0.068896113	6.347222639	0.141290616
LOC643695	0.039696181	5.965333907	0.141310299
LOC650162	0.040658208	6.102441974	0.141374204
LOC644500	-0.034310262	6.214201299	0.141383924
JMJD2B	-0.034959345	6.056460197	0.141402379
ZDHHC19	-0.038595731	6.071240821	0.141503755
LOC645644	0.036328959	6.18356162	0.141535699
LOC649351	0.041449416	6.182320433	0.141549479
LILRA6	-0.022187603	6.101439993	0.14156881
LOC650528	0.034070704	6.139094285	0.141601095
TRIMP1	0.040913268	6.090656293	0.141606986
OSTM1	0.053412577	6.979073473	0.14168867
MYO1E	-0.055920103	6.181417517	0.14170183
PTCRA	-0.025996111	6.084231111	0.141800343
FAM9C	0.03241357	6.037592415	0.141975656
NAT5	-0.059814119	10.28070745	0.14200827
C16ORF48	-0.05722079	8.330656627	0.14211021
LOC401097	0.041655627	5.967486548	0.142435738
ANKRD30A	-0.022004269	5.947687676	0.142513116
GRK1	-0.036084006	6.125285777	0.142525039
PRKRA	-0.101300431	10.05157856	0.142534836
BATF2	-0.05493234	6.357033577	0.142650672
EPB41	-0.034964612	6.032423465	0.142740597
LOC643304	0.041048496	6.291876279	0.142745594
KGFLP1	0.042600683	6.24823124	0.142762203
LOC651721	0.038164765	5.945105254	0.142811186
KIAA0226	-0.055727831	6.381270213	0.142831098
ZNF650	-0.085752275	8.812596269	0.142874328
NOD3	0.034457913	6.142098313	0.14287687
THEM5	0.037827063	6.107135873	0.142891533
CNGA3	0.020503081	6.112334942	0.142926919
PLAGL2	0.059272016	7.441166043	0.142997236
PAQR8	0.063976124	7.172134378	0.143177516
LOC647041	-0.032938564	6.336490703	0.143240478
LOC440356	0.03542124	6.07306106	0.143360996
ROBO1	0.027348878	6.218316038	0.143392625
TRIM6	0.04206292	6.313993183	0.143417143
F10	0.073628503	6.66340555	0.143509789
ZNF571	0.029023499	6.18265389	0.1435415
LOC654118	0.032360649	6.105228509	0.143720464
PTPLAD2	-0.127778095	8.960716265	0.143759807
TPRX1	-0.042428712	5.980145086	0.143827699
TH	0.026495261	6.013411528	0.14385121

LOC731158	0.039896674	5.991383647	0.143920369
EVX1	-0.052705775	6.039734129	0.143939248
LOC642447	0.036012495	6.217426092	0.144003002
LOC440233	-0.035395729	5.947899716	0.144019743
LOC388946	-0.029384375	6.048981069	0.144091314
PEX11A	0.06625528	6.70171041	0.144215831
LOC440509	0.045203803	6.640066164	0.144256166
DPEP2	0.055484324	6.337473067	0.144261634
CHRNA4	0.039543521	6.17540446	0.144319711
LOC643854	0.030826268	6.118170657	0.144320263
OR5K3	-0.026126965	6.009222238	0.144380903
CHRM5	0.036684636	5.98620343	0.144392037
LOC651905	-0.037453676	6.037872843	0.144437353
LOC643154	-0.034073468	6.074687083	0.144451218
USP39	-0.054588007	8.668884822	0.144483319
HJURP	-0.039767846	6.129085655	0.144527426
PWP1	-0.058783818	8.581205831	0.144557136
FRMPD1	0.042734325	6.339248634	0.144646841
MYLK2	-0.043614038	6.170053154	0.144866349
FLJ38379	-0.021316047	5.955118588	0.144876389
STAG1	-0.057252025	7.733352368	0.14493037
SNX6	-0.051762643	7.792840958	0.144934333
IL28B	0.104510707	9.946185608	0.145088528
ETV2	0.072523087	6.117528	0.145100177
C14ORF37	-0.065934627	6.644573737	0.145112129
BHLHB8	0.040326116	6.106957144	0.145173122
PTPRF	-0.049298098	6.767371637	0.145173435
NLK	0.100298503	7.471209848	0.14522508
CILP	0.175489985	8.671228563	0.145247274
LOC650148	0.03779875	6.025265608	0.145435662
BIRC2	-0.058455756	8.775247764	0.145436735
CHMP4C	0.074536522	6.379059179	0.145477704
LOC641801	0.037760734	6.110857384	0.14554949
C18ORF19	0.105783032	7.834474772	0.145572656
LOC730818	0.033218839	6.112264605	0.145594977
CAMK4	-0.029949097	5.952082754	0.145624425
DKFZP434N035	-0.027979962	6.082314906	0.145724052
TEX11	0.026188792	6.027795068	0.145736401
CEACAM16	-0.032202083	6.092024282	0.145755473
LOC643869	0.038739994	5.987631547	0.14578818
FAM173A	0.07118315	8.503237645	0.145827453
LOC643256	-0.036180178	6.219251846	0.145849542
LOC644992	0.030789958	6.092355812	0.145908896
ARL17P1	0.097121742	6.945412037	0.145913682
SMPDL3B	0.024011722	6.005418657	0.145915632
TXNDC17	-0.073851327	10.90277132	0.145986279
TRIM69	0.039159146	6.372633116	0.146130545
ZNF98	0.034901745	6.201559951	0.14614459
TNFAIP8L1	0.104483532	7.236815519	0.146208836
SLC35D3	-0.039638509	5.996567586	0.14623157
CACNA1C	0.09113733	7.86414914	0.146255616

C3ORF57	0.028193501	5.958312403	0.146294573
LSM14B	0.046705715	6.682533381	0.146294743
C19ORF70	0.057966398	11.89688226	0.146331063
HNRNPA0	-0.082990009	9.698838123	0.14634838
C1ORF114	-0.051776872	5.990435803	0.146471401
LOC652784	0.04473319	5.984164044	0.146503408
OR6V1	0.037791806	6.315721432	0.146511541
OBP2B	-0.036194999	5.888544332	0.146680967
NCF1C	-0.054883784	6.476522856	0.146773511
LOC644763	-0.038111758	6.277773351	0.146808371
SERPINA10	0.045369803	5.995541154	0.146829843
SOS1	-0.050089305	6.610282853	0.146832327
NMUR2	-0.037339505	5.939583222	0.146865786
LOC649037	0.038345965	5.86067068	0.146895967
GCSH	0.059630997	6.992688459	0.146943362
LOC650812	0.041565015	5.921515169	0.146981109
COL2A1	0.034443941	6.134414767	0.147060995
RGL4	-0.039126583	6.237366162	0.147120677
LOC650708	0.034426167	6.223640199	0.147160105
PWWP2A	0.045398624	6.783577796	0.147222052
LOC642712	0.03811698	6.291328497	0.147258059
DLK1	-0.275104274	8.114182434	0.147303468
LOC201164	-0.080674893	6.823265569	0.147306924
PDAP1	0.056773246	6.027876916	0.147349391
CCBP2	0.058200771	6.303008132	0.147391651
PBX4	-0.040022643	6.11486126	0.147413948
MGC5457	-0.102594043	6.836902908	0.147515468
GLCCI1	0.042000117	6.542222159	0.147575913
OR51I1	0.034871112	6.084451595	0.14781297
C8ORF49	0.049872266	6.480312514	0.147836062
NAT13	-0.089143646	7.188395052	0.147901268
HADHA	-0.095948895	9.860276829	0.147927496
LOC645262	-0.032162716	6.083735223	0.147938662
CYS1	0.025262415	6.069928501	0.147947078
XKRY	0.038539169	5.969219932	0.147987547
SNIP1	0.07162083	7.310798445	0.148076057
CFP	-0.04204267	6.149388028	0.148090465
PCP4	-0.062200032	6.594142745	0.148133042
LOC651192	0.036888954	6.023185776	0.148140748
OR9I1	0.040047849	6.200189956	0.148215735
C2ORF25	0.082742497	10.54761595	0.148291523
LOC646388	0.033617282	5.967546516	0.148335522
C11ORF41	0.02936611	6.07199361	0.148361716
ZFHX4	-0.040893702	5.950612047	0.148380373
EIF3M	-0.068650576	9.796382373	0.148382594
LOC650428	0.034987613	6.139952008	0.148434553
TXNL1	0.119980294	7.707571715	0.148488773
LOC652883	-0.039361227	6.209913212	0.148532079
LOC647520	0.038694438	6.36200597	0.148588168
LOC643147	-0.03567209	5.970646099	0.148619348
PCDHB3	-0.029856957	6.200096713	0.148663792

LOC285900	-0.076116823	10.92133266	0.148671524
FAM134C	-0.075608273	6.148330762	0.148747121
RET	-0.031042179	6.204797026	0.148778434
LOC644974	0.033275767	5.995478123	0.148817943
CEP55	-0.044387022	6.300809718	0.148927634
TTPA	0.026204948	5.892290004	0.148975604
KLB	-0.040482009	6.246998711	0.149011012
PCDHGC3	-0.023381745	6.250358414	0.149022356
LOC650274	-0.028312579	6.019500189	0.149190543
FLJ12993	-0.031829925	6.258334988	0.149191627
LOC646256	0.036959915	6.051255474	0.149241448
FBLIM1	-0.037644054	6.337414226	0.149242819
ECEL1	0.033460142	6.104198037	0.14925611
LOC390349	-0.041573212	5.948611185	0.149273898
LOC647037	0.072727374	7.927116589	0.149304303
NBPF12	0.044796782	6.146176789	0.149342049
LOC649385	0.037570541	6.076233725	0.149369291
FAM18B	-0.059005946	7.628220568	0.149412136
CST9L	-0.03628549	5.91886188	0.149426781
TPX2	-0.041321671	6.08194233	0.149484571
LOC650985	0.031421154	6.104587179	0.149632758
ZNF565	0.028326816	6.76857537	0.149634219
GLS	-0.069780573	8.34185886	0.149673027
LOC730196	0.03607826	6.041638816	0.149692995
CNTN3	0.054490622	6.242045347	0.149759147
LOC643416	0.038210119	5.896098295	0.149790073
LOC646625	-0.036621908	5.915069114	0.149839778
LOC730243	0.04866856	6.158001107	0.149932224
IKZF4	0.052310375	7.524659217	0.149938553
CHST2	-0.065915278	6.158778482	0.149945965
BNIP2	0.06270806	9.170325329	0.150041233
LOC642419	-0.039647775	6.122924511	0.150059019
C9ORF156	0.06082874	7.54817818	0.150091861
BAGE2	-0.040803309	6.057283253	0.150245542
B4GALT2	0.033584281	6.183264375	0.150340595
CCL28	0.02126806	6.122487508	0.15036786
TARDBP	-0.050297237	6.704910746	0.150387485
GTF2H3	0.060487984	6.904856559	0.150575746
PRRC1	-0.069789043	8.131916887	0.150619665
OR4D2	-0.026015803	6.088277699	0.150666079
NFYB	0.050326366	6.87520818	0.150712956
KIF5A	0.032255051	6.118967991	0.150773597
LOC644450	0.033399103	6.19609539	0.150815469
FLJ36157	-0.034950616	6.03000478	0.15081982
LOC399898	0.042383041	6.041458552	0.15089517
FBXO15	0.041082126	6.297597598	0.150942375
SNIP	-0.042854215	5.919194872	0.15101089
LOC146325	-0.044883065	5.961089697	0.151072864
KRTHB6	0.055446979	5.923310292	0.151155509
SESN3	0.035967941	6.188658169	0.151164667
FAM168B	0.106670224	7.724550022	0.151236885

AVPR1B	0.051954699	5.965902232	0.151254239
LOC652435	0.033089573	5.989938385	0.151275793
CEP110	0.041209113	6.584953008	0.151389821
LOC441031	0.035987501	6.025895335	0.151391111
POLD1	-0.053974719	6.684823254	0.15141384
SLC9A10	0.073124641	6.733509244	0.151499884
ABCB9	-0.05988373	6.896034726	0.15150624
LOC653674	0.042424665	5.884902948	0.151610135
LOC727791	-0.035395544	6.086118365	0.151673257
R3HDM1	0.045418117	6.762809749	0.151673857
ZNRF2	0.044862817	6.2442816	0.151737023
C9ORF131	0.031372979	6.168339835	0.151747848
LOC644204	-0.035812591	6.074075592	0.151774788
KIAA2022	0.039048589	5.951234081	0.151815696
LOC650684	-0.037944118	5.931594312	0.151936551
IPF1	0.038538506	6.108899479	0.151937466
LOC644808	-0.037168054	5.945234323	0.151983722
LOC641941	0.03671871	6.23686084	0.152082008
SH2D6	-0.034331048	5.983228008	0.152085399
RTN4RL1	-0.056763106	6.213132356	0.152113361
PAM	0.062980685	13.56855397	0.15217231
LOC645454	-0.040413264	5.859530376	0.15222491
KBTBD8	-0.092070451	7.346663193	0.152265853
OR6K2	0.032716294	5.977307051	0.15230096
NFS1	0.033398526	6.626847358	0.152450725
GAD2	-0.035041797	6.097346272	0.152465744
DNAJC22	0.039177271	6.158821346	0.152615447
SSX2IP	-0.053919704	6.652658435	0.152662314
CCL15	-0.048256347	6.382148781	0.152683851
NOTO	-0.037215164	6.131713308	0.152755244
ABCC1	-0.033265744	6.255614819	0.152766327
OR7A17	0.047833716	6.06208666	0.15278294
LOC642788	0.040141231	6.320398497	0.152835852
LOC388199	0.039575087	5.933990168	0.152856886
SQSTM1	0.170930026	10.94630066	0.152866766
CHST12	-0.063144332	7.758873349	0.152906446
INS	0.03740742	6.202073834	0.152919173
HNRPA1P4	0.113299716	9.75254535	0.152927963
PCDHGB6	0.045774643	6.85652337	0.152952713
TTTY20	0.036277264	6.000147912	0.153055124
LOC647819	0.032475793	5.949328849	0.153155995
KLF11	0.083404481	8.602719221	0.153224261
UGT2B4	-0.153112359	6.805553613	0.153261388
DHH	-0.045137311	6.126443346	0.153274585
LOC648226	-0.037637357	6.121056038	0.153329134
NUDT22	0.06381031	8.372089472	0.153384406
KIAA0692	-0.015002476	6.041107428	0.153431099
GNRH2	-0.024397622	6.013728217	0.153450622
SPACA4	0.038582955	6.156390736	0.153477242
RAD23A	-0.067335511	10.28429773	0.15352938
C6ORF138	0.042883414	6.556424741	0.153565277

WIF1	0.054904555	6.076077422	0.153708323
LMAN2	-0.126034329	6.618892002	0.15388452
LOC390231	-0.045995803	5.985615972	0.153910332
MAOA	0.116872713	11.2274091	0.153924539
PAQR7	-0.049781923	6.463751708	0.15397502
LOC645971	-0.032894437	5.904622859	0.154049972
GGA3	-0.03427363	6.757346064	0.154067229
FGF17	0.036788706	6.081614612	0.154130595
LOC402538	-0.037887016	6.064882781	0.154148339
SAP30	-0.116429583	9.014141764	0.154150525
XRCC6	-0.053592854	10.13306326	0.154194353
ST6GAL2	0.034958219	6.003975707	0.154237614
DNAJC1	-0.04961304	6.400562816	0.154257073
LOC647785	-0.03741439	5.976894246	0.15430838
MMP1	-0.04001117	6.113693175	0.154360682
LGALS3	-0.156134081	9.394408777	0.154385403
CYP8B1	-0.035772489	6.018734077	0.154441195
ZSCAN16	0.041489438	6.981050956	0.154449273
GZF1	0.043236467	6.574294443	0.15463806
LOC442206	-0.0305779	5.914861047	0.154645993
KIAA0773	0.028394936	6.07566546	0.154655787
C9ORF93	0.024205261	6.171869265	0.154722671
EIF2AK3	-0.069938436	6.562051503	0.154740586
PCSK7	-0.087772891	8.429938999	0.154808372
POLR3K	0.057082807	7.65710004	0.154826276
ZBTB37	0.039199634	5.938582645	0.154830689
ADAM6	0.028961706	6.115123005	0.154971207
DUSP22	0.052090769	9.326151401	0.154981234
GTF3C6	0.078424767	8.850256383	0.155051754
SEC14L3	-0.037421328	6.033787565	0.155075358
AFAP1	0.045394115	6.61417673	0.155187584
ERGIC1	0.081994827	7.335178105	0.155219795
LOC402217	-0.036157934	6.032453706	0.155248702
ADAMTSL2	0.117794318	8.164955389	0.155273363
BRDG1	-0.03461977	5.894256346	0.155291407
ATP2B2	0.01715291	5.994722286	0.155393577
BIRC8	-0.032633608	6.064896137	0.155448534
LOC647044	0.042614066	6.22237259	0.155486877
CDC2L2	-0.082486029	6.802633117	0.155576812
RERE	-0.073918318	9.256795666	0.155582113
COMMD2	-0.081362355	7.736569464	0.155610501
AMFR	-0.086218129	7.992776442	0.155640595
C20ORF173	0.035749064	6.06190511	0.155648735
SLC9A8	0.055069898	6.964421933	0.155687207
DRD1IP	0.0384726	6.040802853	0.15569612
ADM	0.163437728	10.07829621	0.155741222
TRIM64	-0.038344633	6.123618161	0.155770514
LOC642187	-0.04173681	5.991856275	0.15580564
LOC654165	0.037710187	6.063887424	0.15581653
ST8SIA1	-0.031203669	6.137975066	0.155820218
TH1L	-0.051488418	8.874163674	0.155859444

TAS2R43	0.036144081	6.223080063	0.155950816
PRNT	0.039004239	5.955081877	0.156027135
C14ORF68	-0.03660045	6.056922806	0.156092085
LOC132241	-0.068168416	7.328115054	0.156103871
LOC729008	-0.063397866	6.46431261	0.156164338
LOC643814	-0.040066478	5.997448149	0.156167303
SERINC5	0.037096653	5.971661484	0.156215515
LOC347487	-0.036340827	5.928457221	0.156263112
RAB3C	-0.037236709	6.04517198	0.156328509
LOC283710	-0.032977699	6.159265269	0.156400934
FBXO7	-0.039046276	7.40914484	0.15647462
FLJ43276	-0.03421392	6.025088248	0.156482033
HIST2H2BE	0.115993711	10.01629437	0.156529599
KRTAP10-10	0.025253244	6.245793428	0.156535593
KLRA1	0.034352504	6.140767648	0.156545709
MAML2	0.030368221	6.149322571	0.156581462
INCENP	-0.026433882	6.199002528	0.156612518
ACRBP	-0.04101171	6.07823566	0.156634441
FLJ40712	-0.035942838	6.211659741	0.156684311
IRX1	-0.068780647	6.769507126	0.156716682
LOC648223	0.037389192	6.268091281	0.156722904
C16ORF50	-0.025913454	6.003116441	0.156742942
FTHL2	-0.113482155	9.164410748	0.156763891
CES3	-0.027812717	6.049169417	0.156812736
LOC165186	0.047298583	6.125681932	0.156837133
CDH16	-0.039022549	6.196506009	0.156866957
LOC388532	-0.111736093	11.7086797	0.156920903
MAGEA2B	-0.041485748	6.044209895	0.156959555
SOS2	0.055049037	6.744468045	0.156993548
SPACA5	0.03330348	6.038120251	0.157015525
SNORD32A	-0.035333154	6.211780981	0.157044312
LOC647254	0.034942301	5.943478021	0.157044726
LOC646615	0.036863546	6.136459013	0.157052688
LOC643621	0.032431286	6.107895827	0.157092023
LOC441212	0.032707365	6.107950214	0.157139624
LOC652764	-0.041174862	6.133142325	0.157147286
TMEM213	-0.041344481	5.943852846	0.157182773
EFNA5	0.04435245	6.284294456	0.157214902
ACTL6A	-0.0493085	8.534053781	0.157272329
SPSB1	-0.069250169	6.982416394	0.15732701
MGC26718	0.037224764	6.033900383	0.157447693
TMEM195	0.035083864	6.173370696	0.157459128
LOC644313	0.034025299	6.191896954	0.157499589
PRRT2	0.078065938	6.909106857	0.15765041
LOC399978	0.036380571	6.012230285	0.157655575
LOC648829	0.030352109	6.036780557	0.157659821
OR1C1	0.037383599	6.080590454	0.157690041
C1ORF124	-0.03028894	6.601762217	0.157746448
LOC649389	0.034327012	6.136407732	0.157765455
HHCM	0.036122279	6.010980538	0.157838891
LOC652526	0.036536515	6.032449079	0.15784077

KRT74	0.046524864	5.955788425	0.157967987
LOC390414	0.048176214	6.21031816	0.158160886
PRELID2	-0.028662044	6.04909836	0.158172116
SDF2	-0.054743832	9.268388941	0.158298732
LOC728636	0.035598188	6.043841997	0.158350563
FLJ41821	0.033885678	5.988724756	0.158393099
PLCH2	-0.04193692	6.559349764	0.158486614
SAMD11	0.029524592	6.377361639	0.158600739
CRYBB1	-0.034581581	6.060093033	0.158696985
MXRA8	0.044835581	6.422525971	0.158741452
PET112L	0.122506648	9.590369674	0.158757493
C16ORF74	0.038915488	6.095151386	0.158834067
SPTLC2	-0.037612326	6.813715943	0.158884802
IBTK	0.106835588	9.6429251	0.158969883
CBWD1	0.022090027	6.046097168	0.158971666
LOC642245	0.04057737	5.869701541	0.158980411
SOX2	-0.028038088	6.045743907	0.159024573
NGRN	-0.064091719	11.50807869	0.159218222
PLK1	0.05242221	6.948881118	0.159271457
LOC648987	-0.050832012	5.97086741	0.159345184
ZDHHC1	0.052473126	7.311751945	0.159453986
CDKL4	-0.035708594	6.115445485	0.159586322
LOC644602	0.038308531	6.095508925	0.159594397
PRDM8	-0.056002268	6.835659068	0.159612475
ABCB10	0.072076808	7.587687973	0.159781592
LOC643475	0.024033232	6.083356598	0.15980082
LOC652640	-0.0376925	6.265437748	0.159831352
TCTEX1D4	0.027933099	6.068237427	0.159840184
KIR2DL3	-0.02610092	6.044739685	0.159853547
CIRBP	-0.094102486	11.76029198	0.159919848
GRM3	0.033376209	6.068505994	0.159963598
PROL1	0.031763355	5.982941964	0.160102281
OR5H15	0.036522701	5.955803945	0.160176117
ARMC6	-0.046671592	6.853759534	0.160179138
LOC643060	0.034718638	5.965806693	0.160326808
TMPRSS3	0.020351185	6.126870227	0.160348901
LOC643161	0.037951224	5.992374848	0.160386247
TMCC2	0.042860177	6.078015671	0.160402089
PAQR3	0.067372327	7.345915021	0.160431815
ZNF214	0.030103469	6.140081336	0.160501813
LOC339746	0.030852588	5.980732924	0.160509946
POLRMT	-0.065431727	8.292399021	0.160628929
TIMM50	-0.036630003	5.96118284	0.160629692
LOC641704	-0.037201063	5.966249461	0.160670721
HADHB	-0.098761004	11.04047103	0.160734283
LOC442597	-0.034811162	6.238019021	0.160754141
OTOP3	-0.036759615	6.103670214	0.160774335
LOC645089	0.034361682	5.964285194	0.160872472
RANBP3L	0.028475008	6.099238289	0.160893298
RHCG	0.04296179	6.150406935	0.160903327
C2ORF67	0.040271714	6.216479979	0.160995148

AKT1	0.058629102	10.13828356	0.161051285
BICD1	0.028312453	6.19768562	0.161157837
ATP6V1E2	0.04936898	6.710525808	0.161185055
MCHR2	-0.035972842	6.217057968	0.161228042
LOC646097	0.032107796	6.083231714	0.161238687
RIT2	-0.036006571	5.899344089	0.161287248
LOC644005	-0.038841228	5.913584268	0.161300857
IRF1	-0.128217259	8.434941948	0.161326522
DIMT1L	-0.064075043	10.1313725	0.161397605
ODZ2	-0.034283113	6.282031529	0.161489643
CGB8	-0.033460812	6.162338355	0.161568384
SORCS2	0.047389604	6.161984303	0.161604427
LOC647673	0.145050568	9.484404758	0.161634508
C1ORF137	0.036317904	6.155146608	0.161674255
DNMT3A	-0.021881961	6.14790945	0.161714762
LOC646100	-0.048676289	6.352304222	0.161827382
LOC653841	0.030438713	6.068912007	0.161889883
KIAA0408	-0.06976493	6.780445265	0.161911681
TBCEL	-0.059323989	6.036110946	0.161945734
KIAA0907	0.062489996	8.590172032	0.161948984
LOC651603	-0.030899285	5.917271684	0.162010709
TIGD6	0.035044279	6.476106133	0.162162323
OR4K2	0.025791192	6.055754079	0.162235652
LOC649667	-0.033951354	5.953229572	0.162289823
ANKRD16	0.034858004	6.541941541	0.162348299
HAS1	-0.039723128	6.005121882	0.162354034
MEX3C	-0.0879621	7.721792511	0.162472974
LOC203510	-0.032345699	6.086781007	0.162483415
LOC647468	-0.034230579	5.97555735	0.162505846
DOHH	-0.069176425	6.540239202	0.162606244
DUSP21	-0.036781673	6.004277214	0.162640763
NR6A1	0.02812381	6.114021776	0.162688734
LOC645254	-0.033342732	6.051775088	0.162690731
RAB11FIP2	0.04367817	7.984694836	0.162805893
DNASE2B	0.022885805	6.06019894	0.162810779
C8ORF16	0.043742642	6.248672922	0.162858628
LOC642003	0.036700724	6.265378617	0.162890209
C5ORF24	0.050201071	6.841077466	0.162901749
LOC730109	-0.023815003	6.102103896	0.162910448
ARHGAP18	-0.054274702	6.323849289	0.16296596
CNP	0.035079986	6.190645149	0.163013129
LOC644669	0.033204422	5.989197187	0.16305125
WDR7	0.037244556	7.347514632	0.163110743
C10ORF2	-0.068006987	6.691998535	0.163125301
SSX9	-0.028082053	6.147032794	0.163202708
TMEM86B	-0.03892681	6.475388829	0.163328612
IFI44L	0.160870901	8.215260072	0.163342831
LOC730347	-0.01773065	6.046324179	0.163345167
DLG5	0.044634161	6.196071178	0.163385409
RHBDD3	0.081400861	6.718149613	0.163457039
RNF169	0.043650007	6.352446613	0.163540747

KCNB2	0.036431857	6.046411318	0.163607966
SOCS5	0.034797415	6.522107405	0.163660783
FAT2	0.037229053	5.909114508	0.16367025
EPRS	0.065251865	10.17208732	0.163676083
JOSD2	-0.071658861	6.82401429	0.16374031
LOC652664	-0.034824858	5.979401931	0.163886947
BPIL3	-0.035074907	5.972735484	0.163963674
TAL2	0.052581427	6.121256931	0.163989176
C15ORF32	-0.034914037	5.930141256	0.16402965
ASNA1	0.046615787	6.127348676	0.164035117
IL1F8	-0.021715162	6.026215064	0.164053827
STARD4	0.045523707	5.986165177	0.164070032
ITGBL1	0.053728314	6.278449667	0.164088117
RBM10	-0.048680053	9.352387708	0.164149601
SPDEF	-0.033904001	6.024198465	0.164162598
GABRB2	-0.025897472	5.968877272	0.164174994
H2BFM	-0.03837468	6.287861821	0.164183543
SERGEF	0.072755739	7.860446267	0.164212219
C1ORF116	-0.042672525	6.345641259	0.164237775
SNHG8	0.040236019	6.364877278	0.16425303
RESP18	-0.035483071	6.030331732	0.164256558
CCDC96	0.034610138	6.32528198	0.164397265
LOC646155	-0.040259596	6.027705396	0.164434039
NR2F2	-0.063574742	7.549726033	0.164462294
C12ORF41	0.050816209	9.499363657	0.164564239
PDE12	-0.054607331	7.154763684	0.164720317
SERPINB3	-0.023327723	5.924338698	0.164813799
SIPA1L1	0.054076537	6.431173919	0.164862792
CCDC50	-0.067109555	8.518634204	0.164878431
TTC16	-0.040758565	6.816081996	0.165006008
TMEM41A	-0.082315881	6.950189813	0.165060956
DHFR	0.039577566	6.117438084	0.165067896
PMFBP1	-0.037417195	6.06759844	0.165088693
MEG3	-0.120581182	8.210940932	0.165109543
TAS1R1	0.024541738	6.013082357	0.165114639
ZIC1	-0.044242689	5.974862498	0.165136384
HTRA2	-0.051848616	7.320670669	0.165186279
HAR1A	0.035948092	6.102709012	0.165245686
TMEM95	-0.037436566	5.95370582	0.165269082
LOC652523	-0.033891178	6.053392969	0.165270574
C17ORF97	0.132623276	7.603507002	0.165336285
NPM1	-0.037459326	6.149528242	0.165448025
COMT	-0.070220354	9.651366941	0.165483935
ARSI	0.036446424	6.003743498	0.165497244
CYP11B2	0.041489041	6.112542431	0.165520293
LOC389523	0.049913418	6.239226849	0.165547679
LOC648999	-0.034227936	5.864080484	0.165703535
MC3R	-0.037591292	5.935540725	0.165714046
PRG3	0.035604881	5.990125206	0.165755921
LOC650771	0.034273053	6.040209319	0.165837361
OR4K1	0.031249805	6.132373856	0.165839803

LOC643844	-0.028141167	6.014252829	0.165877087
LOC645712	-0.033553673	5.944434985	0.165908209
FMR1NB	-0.035590119	6.011189502	0.166031803
LOC124685	0.037413061	5.908351275	0.166158267
LOC728554	-0.084538939	7.926984161	0.166197086
ARRB1	0.050893575	7.399887248	0.166307506
AP4M1	0.038874185	6.553229261	0.166378778
KTI12	-0.064466035	6.917979688	0.166399998
MLL4	-0.075129612	6.983958686	0.166440839
YAP1	0.085157406	9.064620075	0.166447696
BRD2	-0.066204181	9.967628265	0.166481983
LOC645648	0.064949672	6.000120813	0.166554191
FAM117A	0.041216218	6.335223871	0.166635764
BAP1	-0.050972275	7.495629667	0.166686713
NHLH2	0.02565894	6.074941398	0.166797228
LOC652759	0.032543922	6.021233163	0.166816845
NSUN4	-0.049315687	8.040610202	0.166940283
RANBP2	0.05310791	6.488207359	0.166988255
LOC646194	0.051323222	5.912362356	0.167054744
FSCB	-0.025246562	5.986617526	0.167069784
ZNF43	0.026230577	6.033589821	0.167091534
HOXC8	0.034126395	6.06190763	0.16709754
ARL2	0.057373852	11.0729282	0.167156582
LOC645116	-0.035135383	6.029568958	0.167169556
C9ORF7	0.040271315	7.310751869	0.167193042
TKTL1	0.129017195	7.926719918	0.167204991
OFD1	0.044492068	7.182688704	0.167206115
LOC400145	0.03450298	6.2929901	0.16728177
NUP188	0.087578386	7.50766596	0.167292082
LOC648667	-0.03695358	5.995593446	0.167329407
NCAPG	-0.048315336	6.1535618	0.167336062
LOC644873	-0.038176274	6.091771698	0.167435317
LOC644098	-0.035157335	5.979353625	0.167439601
ZSWIM1	0.055289909	8.123917042	0.167520377
UCK1	0.050296667	6.597092971	0.167626833
C2ORF65	0.045782496	6.526561543	0.167635764
MYO10	0.06256683	6.787797743	0.1676379
LOC652227	0.034888211	6.216956459	0.167717906
PIGC	-0.042897655	7.868838474	0.167724608
C21ORF90	-0.035377509	6.13542399	0.167742526
LOC645330	0.033477803	6.130634677	0.167781029
MAPK14	-0.022539808	6.222351328	0.167884543
LOC440927	0.091262301	12.12188728	0.16794448
MGC16025	0.034181666	6.081623197	0.167946291
SPINK7	-0.032183994	6.015286388	0.168108217
ZDHHC4	0.036226355	6.122336658	0.168137527
PARP12	-0.094412315	7.709621301	0.168138109
LOC387940	0.034787585	6.07505022	0.168152941
LOC652696	-0.033327442	5.947344486	0.168319393
WFDC2	0.025037253	6.317587478	0.168338678
NPM2	0.036520186	6.248776534	0.168357205

LOC284672	0.035018407	6.05757975	0.16836649
AMN1	0.051019477	6.648948351	0.168372511
KRTAP17-1	0.033333009	6.000147816	0.168388431
TCEB3C	0.033601512	5.965007321	0.168436761
GTPBP3	-0.044236569	7.227163699	0.168439848
PCDHGA11	-0.02571744	5.971173605	0.168444205
CAB39	-0.059483959	10.75264222	0.168462468
LOC647854	0.034162975	6.125734877	0.168488847
LOC651126	0.035760843	6.166654973	0.168538229
LOC653920	-0.03679782	5.942958828	0.168558808
LOC401957	-0.036086886	5.937613324	0.168606418
GNB5	0.036840681	6.376870325	0.168652804
TRPM1	-0.035477184	5.92635686	0.168693472
LOC644710	-0.035068974	6.11620246	0.168704772
PLAC2	0.041918362	6.676702202	0.168734575
PRAMEF10	0.018775787	6.035897217	0.168750355
YDJC	-0.043148188	6.431662986	0.168752829
LOC387723	0.03865377	5.910050149	0.168911018
MGC87631	0.034870124	6.138912928	0.168941727
C6ORF65	-0.043947693	6.105611424	0.16896167
LOC648745	0.041297987	5.882272717	0.16904479
LOC653188	0.036726511	6.133371565	0.169179423
SPATS2	-0.042318286	6.512231369	0.169262818
OVOL1	-0.022620903	6.028901349	0.1692745
TREML2P	-0.039726475	6.063082239	0.169323737
LOC648003	-0.029484874	6.076466788	0.169358731
SMG5	0.074166846	9.200899723	0.169406103
SHROOM1	0.041718146	6.165493367	0.169481875
LOC649493	0.032499651	6.239065436	0.169487881
LOC643072	0.031298027	5.986615961	0.169633025
LOC392563	-0.030548922	6.033935353	0.169646622
OR2AG2	0.040516053	6.081314532	0.169660841
PAPPA2	0.027850815	6.026259361	0.169668757
LOC643266	0.037224964	6.108914377	0.169670792
TAS2R1	-0.029973552	6.081160354	0.16968806
GOLGA8F	-0.035815518	6.022697166	0.169712973
WDR81	-0.102236954	6.285021244	0.169810225
SPATA22	0.04962841	6.122450644	0.16992581
TMEM108	-0.052012353	6.564468848	0.169929306
LHX3	0.023264115	6.132327362	0.169953661
C9ORF167	0.055444496	7.131213534	0.169965657
C10ORF99	-0.03747444	6.072959425	0.170033753
FUBP1	0.039195487	6.63847955	0.170035867
RPS26L1	-0.096895593	11.31731571	0.170044521
FLJ46361	0.024700725	6.08275109	0.170062333
LOC388344	-0.091462074	6.755272613	0.170103173
SF3A1	0.034861564	7.013591182	0.170201288
OAS3	-0.073501357	6.594860371	0.170242218
OR10W1	-0.024468673	6.094305013	0.170254551
KRTAP24-1	0.03193067	6.038148808	0.170366141
POU6F1	0.043855112	6.38666909	0.170366459

FLJ35816	0.036880334	6.043522669	0.170380961
NTHL1	0.103885132	7.669034671	0.170382373
CDK7	0.101801639	7.575022672	0.17062366
CEP72	0.032993025	5.999936933	0.170626658
C9ORF14	-0.038918246	5.972328481	0.170671235
IL1F6	-0.031861278	5.945449675	0.170738091
CXORF40A	0.051612517	8.219575987	0.170785121
HTR3A	-0.022538585	6.101824369	0.170803303
GSK3B	-0.06816194	8.269764022	0.170883943
PLA2G4D	0.026121007	6.09160693	0.17088939
NR2F1	0.045330698	6.142339289	0.17094469
RPL26	-0.096002482	11.00184206	0.171000796
PCDHA4	-0.042074032	6.179592078	0.171118316
TERF2	0.050254937	7.696974337	0.171204566
LOC650203	-0.034768394	6.081090818	0.171215212
PKD2L1	-0.032835801	5.972543717	0.17132182
LOC647710	0.036551812	6.415501047	0.171341069
RGMB	0.052116938	6.680721303	0.171353463
OR10H1	0.029885176	5.894279055	0.171429754
OR4F16	-0.032772785	5.986009718	0.171446142
TMEM81	0.045886565	5.998217493	0.171453796
OXGR1	0.025114522	6.1297788	0.171522514
FLJ45983	0.04844944	6.051724329	0.17157907
ZNF24	-0.044542563	6.346535646	0.171608538
PAX5	-0.027794308	5.929158878	0.171628267
GPR174	-0.027720596	5.976707345	0.17163238
LOC100101121	0.038696607	6.004280978	0.171818108
FLJ43860	-0.019004977	6.074236458	0.171819234
RP13-360B22.2	0.038195969	6.147657293	0.171942752
LOC441150	0.055966452	7.044748787	0.171950874
LOC644989	-0.032295085	6.081268969	0.17200363
IQCF3	-0.034892175	5.977285936	0.172032432
UBA52	-0.050786172	12.61397138	0.172088796
AMPD3	-0.022998341	6.131626816	0.17231988
C1ORF110	0.039628875	6.085509102	0.172327848
LOC642916	0.041671365	6.346432767	0.172355078
LOC402569	-0.035594145	5.9899423	0.172370013
LSM10	-0.05657232	8.733909635	0.172433113
LOC730953	0.039556694	6.193821301	0.172606537
LOC650061	-0.036550163	6.019266631	0.1726234
LOC644841	-0.036461212	6.158944894	0.172738691
KRTAP10-3	-0.042195772	6.022254972	0.172803056
TBPL1	-0.079617614	8.745598887	0.172824508
LOC391766	0.031154243	6.066663869	0.172850549
CPM	-0.023542449	6.465498311	0.172943852
LOC730049	-0.029802585	5.926453001	0.173024526
FAM135A	0.047034564	6.993566728	0.173045878
C20ORF55	-0.048838058	6.310955734	0.173091991
ESCO1	0.055416212	6.915553879	0.173109327
BTBD7	0.033935861	6.993119401	0.173133914
GPR55	-0.039236897	6.01049522	0.173235848

FLJ25037	0.036760818	6.038014702	0.173328313
LANCL3	-0.030033435	5.966404616	0.173443936
MRPS7	0.056358878	10.21064517	0.173470934
KIAA1486	-0.035008211	6.08475311	0.173481887
MAGEB5	0.03579876	6.054141958	0.173529624
PNRC1	-0.040255253	6.161031116	0.173849168
MGC4294	0.039332565	6.093168099	0.173895268
LOC645045	0.034835204	6.079579578	0.17391731
FLJ44048	0.042298984	6.033519607	0.173918767
ST3GAL6	0.08722734	9.726545262	0.173925164
QARS	0.054612163	11.43564911	0.173926844
SGEF	-0.030459333	6.078507294	0.174064136
HPS3	0.048401695	6.652368609	0.174070461
MGC102966	0.038371217	6.379663099	0.1740761
OMG	-0.039815331	5.942494096	0.174118272
STYX	0.028663582	6.127126125	0.174185234
LALBA	-0.032541324	6.030492306	0.174233539
POLDIP3	0.041319233	7.512849675	0.174397361
C10ORF51	-0.034608454	6.102330774	0.174414571
LOC441925	-0.035100576	5.987496803	0.17445677
UMPS	-0.035555919	5.963526961	0.17448875
CHAF1B	-0.048410373	6.113975433	0.174502411
LOC649683	0.037050309	5.995713494	0.174535842
BIK	-0.035226251	5.930341143	0.17455079
AOC3	-0.071067253	6.139737177	0.174561319
FAM86A	-0.046836128	6.688921263	0.174667251
ANO6	-0.064251149	8.835196341	0.174684481
CYP4X1	-0.057119706	6.684632905	0.174741009
LOC643608	0.035697887	6.048561821	0.174770459
LOC442204	0.021812351	6.01661593	0.174823766
KIF5C	-0.030898975	6.286950868	0.174997621
COMTD1	-0.124523024	7.941998509	0.1751248
KIAA0895	-0.048618661	6.464603072	0.175216882
SSNA1	-0.063066047	8.320271187	0.175249637
LOC643169	0.049589446	6.472602402	0.175319426
SS18L2	-0.07784997	10.33005649	0.175344907
LOC651752	-0.038792006	6.118823211	0.175433525
LOC642355	0.038795743	6.019603479	0.175465012
TMEM50A	-0.046335717	6.756571047	0.175471258
CXCL11	-0.036829722	6.104708964	0.175527659
UBE2D1	0.040772755	6.162310172	0.175557535
LRRC29	0.030959484	6.325498479	0.175648202
ILDR1	-0.039311615	5.936383173	0.175688359
DRGX	-0.032906798	6.127342344	0.175740879
ATPBD1B	0.06144756	7.939866339	0.175778681
LOC653524	0.030647952	6.360852215	0.175795617
LOC642863	0.03666621	6.024354618	0.175816613
WDR87	0.025791183	6.110807035	0.175824017
LOC644291	0.024015523	6.090751288	0.175839156
LOC645282	0.038830488	6.151808944	0.176009581
OR4A16	-0.032875047	6.041888978	0.176034683

ZNF500	0.046213571	6.938400376	0.176100764
DCTD	-0.044870119	8.574768347	0.17620055
LOC641742	0.049443937	6.126581836	0.176272524
LOC440368	-0.032520972	6.063859084	0.176312686
LOC729732	-0.033248	6.176559147	0.176353987
OGDH	-0.074576792	9.132108094	0.176379026
SPAG4	0.042115667	6.227065034	0.17641174
LOC641744	-0.040299253	5.982729575	0.17656712
GPR137	0.067897469	9.12390755	0.176578542
FLT3LG	-0.047462393	6.36258179	0.176603791
SPINK2	-0.036752787	6.051199927	0.17664652
NOP5/NOP58	-0.08509088	8.311896267	0.176678752
KRT32	-0.052328519	5.979051175	0.176693565
BCL2L10	-0.038595554	5.995788087	0.17670787
PSMAL	0.036221545	6.004745104	0.176761402
MRGPRX2	0.025223186	6.057936405	0.176848626
GULP1	-0.036695432	6.181720511	0.17689002
HSD17B12	-0.078771261	10.38909688	0.176917669
C1ORF66	0.051661489	7.4310832	0.176953131
LOC644656	-0.041205314	5.906237734	0.177002081
ELAVL4	0.038205248	6.115190468	0.177014174
THOC3	-0.054639025	6.81109861	0.177048389
ZNF619	0.038609112	5.960566532	0.177086767
LOC644986	0.034643571	6.033903839	0.177095978
PROZ	0.036501601	5.971316988	0.177112444
CENPF	0.043818859	6.211535335	0.177147027
GLYATL2	0.041634573	6.017761289	0.177176233
RP11-49G10.8	0.038342278	6.145582388	0.177279254
TNFRSF11B	-0.181852295	7.74437248	0.177414386
HIST1H2BE	0.034562528	6.183042692	0.177447397
LOC650780	0.041516546	6.499603288	0.177498676
LOC644286	0.033697386	6.15430691	0.177548473
DNMBP	0.030817265	6.043358906	0.177651814
UCN3	0.037033751	6.143909441	0.177665172
TRY1	0.036430614	6.12793538	0.177832193
LOC645141	0.033737659	5.96721198	0.177856699
KIAA1549	-0.031803256	6.0424206	0.177909968
LOC401237	-0.035047427	6.316084915	0.177951262
LOC645958	-0.037093118	5.848754856	0.177965451
C10ORF91	0.025755662	6.056688671	0.178139794
SPAG11B	-0.023920055	6.117603529	0.178165659
KRTAP5-3	0.037182255	5.927040975	0.178239276
MRPS5	0.061291589	9.381205818	0.178356551
KIF19	-0.033965944	5.973660894	0.178385571
SCARNA15	-0.035465798	5.964027594	0.178425944
LOC646568	0.034463715	6.283108107	0.178434028
C9ORF85	0.040875435	6.174677337	0.178434754
LOC390110	0.032815851	6.155566662	0.178485468
LOC646008	0.040302458	6.131784441	0.178511999
UNC5CL	0.033251905	6.003594423	0.178741652
MID2	0.026173915	6.501938869	0.178758369

LOC643761	-0.042974615	5.902564984	0.178847608
HAL	-0.022818794	6.001835839	0.178873944
LOC392196	-0.033948554	5.921034518	0.178914334
LOC645718	0.036308773	6.085621516	0.178967357
NSUN5B	-0.043472027	6.962360522	0.179088394
COLEC10	0.035331365	6.042534123	0.17912552
LOC647181	-0.032328268	6.093624304	0.179140046
LOC647513	-0.040275292	5.930552911	0.179195857
LOC644516	0.029997389	6.13034014	0.179255729
FXYD6	-0.074054481	8.423204254	0.179280178
DDX19-DDX19L	0.046067953	6.568396048	0.179292214
LOC139735	0.030653368	6.10101143	0.17930784
LOC644144	-0.040110647	6.450161611	0.17940108
FKBP15	0.046723647	6.953495796	0.179477746
ACTN1	-0.131687667	10.42025528	0.179513238
LOC388481	0.033200954	6.042892188	0.179515611
PDK1	0.038464887	6.537583935	0.179586589
CEACAM7	0.035664422	5.957153593	0.179648542
PRED57	0.043101622	6.154819044	0.179746823
RBMY1A3P	-0.024987954	6.032185787	0.179754584
LOC642989	0.12547874	9.821580543	0.179792273
BPGM	0.028226107	6.55198542	0.179821162
ZNF587	0.023920939	6.085082301	0.17990016
FLJ13611	-0.033120846	6.133873531	0.17995932
LOC642934	0.138984038	9.658615857	0.180028639
PREI3	0.061129174	8.401932031	0.1801456
LOC654258	0.031506727	6.006022913	0.180154559
WDR78	0.023615389	5.995428016	0.180177919
LOC641823	0.037684401	6.380612363	0.18026397
OR2A5	0.044116239	6.669172713	0.180327647
RPL7	0.096465923	9.505148944	0.180330932
LOC647347	0.042530899	5.977010873	0.180351906
FLG2	-0.025069459	6.011666791	0.18054584
XKR3	-0.033061848	5.888022371	0.180585871
ALPK3	0.068237073	7.071501606	0.180721093
DNAI1	-0.032933021	6.096386722	0.180723123
SYT2	-0.033060587	5.917383504	0.180726838
ATP6V0A4	0.019585063	6.027441701	0.180742887
DISC2	-0.036084818	6.131221758	0.180753595
SNORD18C	-0.034107685	5.955059978	0.180950505
DUSP7	-0.032627527	6.086613081	0.180988338
LOC646813	-0.036342961	6.215176718	0.181060538
NUDT8	-0.041065307	6.238200825	0.181112464
GGT3P	0.040465438	6.300901878	0.181134395
MAT1A	-0.037594435	5.904898773	0.181193028
MIPEP	0.138704073	9.034800628	0.181297551
LOC652831	-0.033476074	6.052118165	0.181416838
SERPINA4	-0.042836599	6.112713993	0.181468385
LOC646734	-0.040554013	6.095460996	0.181490639
GIT1	-0.048699804	6.828559483	0.181516335
MRC1	-0.023569751	6.027790299	0.1815282

LOC644175	-0.035945283	6.027349779	0.181554333
STXBP4	0.032533593	6.296921176	0.181644616
MYB	-0.032841438	6.174814947	0.181652208
LOC644621	0.035134357	6.00298825	0.181754097
CHMP1A	0.09081017	8.32086287	0.181758039
TMOD2	0.029126778	5.967153461	0.181802821
LOC644948	0.037637537	5.971220553	0.181854971
CALCA	-0.055890698	6.171647452	0.182005501
CARD6	-0.043580206	6.174250335	0.18207289
LOC285442	0.030130395	6.018046821	0.182143915
KIR2DL5B	-0.023641348	5.966027848	0.182148203
MGC50722	-0.034169237	6.282539269	0.182186842
EPX	0.064474853	5.990068755	0.182241471
C11ORF72	-0.029221873	6.049829674	0.182317504
ZNF296	-0.037148059	6.208788005	0.182352664
PREB	-0.082171486	7.342171916	0.182357957
SMC1A	-0.037750801	6.211642227	0.182438918
LOC442180	0.042659388	6.17242512	0.182596667
LOC649292	0.033004605	6.088797294	0.182619111
LENG1	-0.04829023	6.822986451	0.182671798
FLJ40411	0.03214521	6.072673202	0.182729001
LOC651511	-0.057065054	5.990285211	0.182757043
OR2A25	-0.029006314	6.103426546	0.183009561
LOC440918	0.03101626	6.168925787	0.183047081
C12ORF61	-0.034476699	5.958989875	0.183074551
ZNF75	0.041859158	6.978579749	0.183150138
MICALCL	-0.040182195	5.998339595	0.183214137
TAS2R41	-0.034730474	6.164303178	0.183262637
SRFBP1	-0.04926043	6.896831194	0.183332976
ATP9B	0.046631314	6.379584936	0.183384723
C10ORF55	0.050373841	6.197259676	0.183386232
LOC284417	-0.035414392	6.225688322	0.18345828
ELA2B	0.038588906	6.287158693	0.183543555
GLYATL1	0.035672448	6.200058425	0.183627345
LOC401180	0.027106005	6.295322028	0.183663481
TFCP2L1	0.034167535	6.025594675	0.183694647
MYBPC1	-0.077607302	6.624703332	0.183720949
GGTLC1	0.02123392	6.259811912	0.183728107
LOC442124	-0.035857486	6.171345409	0.18374652
EPHA5	0.025190102	5.9681319	0.18375249
CCL16	0.048225468	6.203713654	0.183759367
TMEM52	-0.038173112	6.113285114	0.183856976
LOC650342	0.032566589	6.097120504	0.183935651
LOC642148	0.030556311	6.051335	0.183943304
LOC644086	0.053749366	6.513753191	0.183946159
PADI1	0.035559088	6.133785318	0.183959507
LOC642399	0.036645671	6.248511239	0.183978683
ASZ1	-0.024233761	5.912537941	0.18400449
KRTAP19-3	0.039280417	5.93076292	0.184098999
LOC646570	-0.031874396	6.093335322	0.18416745
LOC647225	0.036389853	6.192239439	0.184213933

MMP24	0.032896185	6.187414617	0.184215773
AMMECR1L	-0.075897759	7.676044641	0.184260278
MAML1	0.027181785	6.409629342	0.184333791
CGB1	-0.024517475	6.048079347	0.184337332
FOXJ2	-0.063433654	10.32628078	0.184397022
THAP8	0.038494562	6.067726015	0.184415941
TAF7	0.06739563	9.188527159	0.184419732
TMEM166	-0.053722468	6.354943111	0.184438291
PSMA6	-0.051171573	11.38531091	0.184457929
NKX6-2	0.032826437	6.119888885	0.184511316
TIAF1	0.071337357	9.949935299	0.18453922
LOC652501	0.02921454	6.00059574	0.184608535
SVOPL	-0.035298675	5.928740901	0.184644969
TNFRSF10C	0.037473238	6.652860247	0.184718316
LOC643868	0.030956307	5.898523325	0.184735602
AGBL3	0.03241822	5.997431444	0.184735958
BTBD3	-0.056615835	7.655083246	0.18476251
LOC647460	-0.032719329	6.229327242	0.184791634
GMCL1L	-0.033518106	5.901674922	0.184813514
C14ORF78	-0.07730896	6.54605423	0.184821562
LOC643273	-0.036239244	6.153696418	0.184861555
LOC643248	0.03913906	6.024063566	0.18488732
LOC442560	0.034344435	6.114429793	0.184946062
LIPJ	-0.035961699	6.20791153	0.185006642
KRTAP5-4	0.034871698	5.969067062	0.185014373
LOC645078	0.037121092	5.934521964	0.185145489
PDZD8	-0.048587455	6.927875617	0.185361547
CASP12	0.035895678	6.018583079	0.18540763
DEPDC7	0.020403305	6.055578867	0.185461874
LOC651197	-0.038524813	6.034564339	0.185492381
KIAA1161	0.048535718	6.106613308	0.185504024
KRT36	0.032643591	6.099996804	0.185529074
GABRA3	0.040521099	5.986674266	0.185530212
LOC650804	-0.033967758	6.0296856	0.185593894
NUDCD2	0.060439193	6.968914701	0.185661695
AGR3	0.025694217	6.102289997	0.185662563
PRAMEF7	-0.018947464	6.128095248	0.185676503
SC4MOL	0.080370714	7.366441656	0.185692527
FLJ32255	-0.03481511	6.150114413	0.185695469
SEZ6	-0.043714811	5.992324969	0.185743592
LOC653570	-0.032821316	6.01318901	0.185764789
KLF1	0.033080095	6.40225486	0.185876947
LOC652869	0.03327846	5.960607513	0.185936932
ZDHHC23	-0.0500752	6.183326707	0.185996746
LOC646190	-0.032947331	5.984424595	0.186024852
CYP2D6	-0.028296683	6.196888549	0.186100655
DSTN	0.054885649	11.35393354	0.186177356
LOC643344	0.033782984	5.934989987	0.186287769
LOC652078	0.049344607	6.177754941	0.18634048
ATPBD1C	0.042427581	6.057348687	0.186343295
RAD9B	-0.038710249	6.099874428	0.186397903

SCN1A	0.033955727	5.98988239	0.186425688
LOC642210	-0.074583187	13.84811113	0.186632499
BEST4	0.035082613	6.070377896	0.186661155
LOC648645	0.03762078	6.049162368	0.186857601
NANOS2	0.034475675	6.007007122	0.186867226
LGI3	-0.031871833	5.938478499	0.186924113
LOC645545	-0.032017364	6.017962677	0.186940039
LOC650095	-0.044263642	5.966923918	0.186975748
ESPL1	0.043208027	6.874482213	0.186993006
LOC652153	0.030091466	6.043287255	0.187165733
ZDHHC11	-0.101588434	7.013288477	0.187181628
AZU1	-0.032705372	5.976705938	0.18721236
TMOD1	-0.051279604	11.82589794	0.187230535
CHRM4	0.035519296	5.929605637	0.187258469
GIPC3	0.028861041	5.914614973	0.187308676
LOC441876	-0.071527813	13.72767943	0.18739704
ZNF711	0.031046248	6.072336999	0.187503149
PCP2	-0.032206993	6.150519928	0.187539644
LOC441709	-0.040943395	5.950559545	0.187568841
C6ORF128	-0.031802305	6.214696395	0.187591351
LOC652882	-0.029897263	5.964226476	0.187613441
LOC648148	0.047056428	6.027272773	0.187757672
LOC650770	0.033381872	6.017119799	0.187791502
LOC643738	0.029229865	6.146562693	0.187865562
HMBOX1	-0.070540599	7.255561519	0.187866393
C10ORF65	0.123445037	8.452910209	0.187905725
MSMB	-0.019952636	6.027129059	0.187975631
UTY	0.054907959	6.228668023	0.187984027
LOC649826	-0.032504987	6.131477091	0.187995218
OR2M4	0.0333149	6.091035479	0.188008302
LOC642521	0.033872688	5.993462055	0.188057311
C6ORF148	0.043708966	6.354909175	0.188148226
IRF3	-0.0443005	7.341667972	0.18819206
CACNA1S	-0.044447227	5.916632528	0.18823194
NRN1L	-0.033017233	6.174400442	0.188239209
LOC650517	0.019010768	5.981320586	0.188256669
OR4C15	0.023836749	6.109515533	0.188263509
LOC648205	-0.035096783	6.045372422	0.188270544
SRCAP	-0.056819176	6.366726553	0.188343224
IGSF10	0.061395097	6.152018884	0.188363256
OTUD5	-0.056682832	6.480292616	0.188391622
LOC644536	-0.033144926	6.070051842	0.188416795
LOC642620	0.030267529	5.926881301	0.188446356
LOC647488	-0.03586455	6.258212907	0.188457795
FUCA1	0.093568819	8.384096629	0.188546836
CCL27	0.035801913	6.073904233	0.188548645
FLJ21767	-0.022536473	6.050276493	0.1887473
COBL	0.105680958	8.255600886	0.188849678
MIOX	0.031191094	6.023395969	0.18900912
ZNF578	0.028361192	5.917909065	0.189019833
LOC652147	0.034744977	5.955718169	0.189080987

LOC648982	0.029733834	6.188131005	0.189124362
MFAP2	-0.043273965	6.063242308	0.189146294
CLCA2	-0.034319435	5.975506518	0.189153197
RBBP4	0.043327272	6.917127787	0.189176109
MGC50273	-0.022032484	5.974991811	0.189240537
LOC647531	-0.045489085	5.96556678	0.189282198
VWCE	-0.031645369	6.117887847	0.18929855
MT1P2	0.039823914	5.886898011	0.189314616
LOC641743	-0.034517661	6.116139137	0.189376271
PRDM10	-0.03529092	6.879114889	0.189462807
RPL32P3	-0.024801525	6.076753849	0.189528586
LOC646442	0.029924002	6.107748444	0.189627301
LOC646149	0.034244458	6.028842079	0.189692001
STK32A	-0.035770637	5.943942935	0.189784021
FBXL5	0.064313221	8.220365996	0.189899775
LOC647028	0.035181667	5.988668503	0.189913596
FLJ45721	0.039570328	6.004474232	0.189965144
MYH15	-0.037766838	6.012052161	0.189971907
ZNRF1	0.042400693	6.088141539	0.190079796
C2CD2L	-0.037009989	6.353697787	0.190217047
C17ORF92	-0.032361912	6.091056223	0.1902599
LOC732165	0.095661052	8.296466842	0.190271258
LOC644033	-0.078694726	7.981725196	0.190336058
RAB5B	0.054577276	10.81244288	0.190366817
LOC644768	-0.02426622	6.019236383	0.190416101
SLC8A2	-0.035504513	6.178946781	0.190505997
LOC653352	-0.020648093	6.172703738	0.190520892
KCNQ2	-0.027381336	6.203286663	0.190543623
WDR60	0.069505425	8.973229388	0.190562078
LOC653200	0.031521556	6.047578878	0.190737123
CNNM1	0.037088433	6.477864629	0.190796005
KRTAP5-6	-0.029896448	5.937382142	0.190960144
OR56A4	0.030942336	5.993119243	0.19101789
CDH7	-0.021687615	6.166357233	0.191070617
C9ORF27	-0.035102754	5.865402026	0.191087689
LOC653632	0.044785023	5.96389041	0.191097927
LOC645289	0.065768927	6.967426458	0.191227262
LOC644631	-0.033779531	6.141627567	0.19136499
RWDD2B	0.045518	7.163056988	0.191369339
KCNJ11	-0.044081369	6.205030391	0.19137923
LOC653887	0.030487565	6.19000477	0.19143285
HDAC10	0.037528562	6.16596227	0.191438401
TFAP2D	-0.035452012	5.999736454	0.191450668
TUT1	0.039528163	6.902362024	0.191526136
UHRF1BP1L	-0.02718527	6.460603582	0.191542914
ZNF557	-0.07690391	7.143082351	0.191674984
VMO1	-0.037368839	6.455409109	0.191745773
ITGB5	-0.058830056	9.920434638	0.191826868
TET3	-0.037009625	6.111341124	0.191857245
FER1L4	-0.025520053	6.089933248	0.19189532
GCET2	0.026925839	6.068630461	0.191923805

LOC645553	0.032862081	6.022456035	0.191972465
LOC650584	0.028980297	6.063663836	0.191982944
LOC650463	-0.034098126	6.075450596	0.191992292
ZNRD1	0.045639633	7.543666753	0.192032804
CHML	0.042824172	6.814279288	0.192093689
KIAA0565	-0.016166675	6.065578229	0.192137832
LOC728601	-0.032871773	5.881844578	0.192195663
LOC728910	0.029494385	6.221090827	0.192274488
ITGB1	-0.065713112	11.09851236	0.192330048
CLDN22	-0.03503706	6.015670389	0.192354422
LOC643570	0.034612012	6.099087805	0.192434228
NUP50	0.02201503	6.421769249	0.192463016
ZNF646	0.035844986	6.195979253	0.192496533
LOC440776	0.051313645	6.282216676	0.192502811
MGC45922	0.02609072	6.000909837	0.19252179
GUK1	0.049991858	10.61113123	0.192526686
TMEM190	0.036104242	5.978680902	0.192542344
ADRA1D	-0.034615198	6.271134475	0.192608596
MAB21L1	0.04414666	6.117655223	0.192628271
JMJD2D	0.047528228	6.10834504	0.192703349
FLJ23754	-0.034003574	6.198103482	0.192763577
LOC652708	0.033159832	6.019561362	0.192804943
TMIGD1	0.033709106	6.210724838	0.192822976
LOC646227	0.031110707	6.085305204	0.192830906
CD164L2	-0.033578545	5.912466038	0.192929385
C12ORF36	-0.031798847	6.041345411	0.193102448
COG1	0.052922401	7.522082417	0.193162321
LRRC8B	-0.034725089	6.07798497	0.193209165
KIAA1604	0.055330041	6.318590678	0.193264621
SNORD16	0.035582836	6.115611685	0.19328345
ZNF789	0.029201085	6.630996462	0.193320509
LOC644647	-0.030362172	5.979393757	0.1933339
MOBKL2B	0.039749712	6.152359658	0.193581098
LOC283278	-0.02975112	5.950385698	0.193604256
ZBP1	-0.051875218	7.820447921	0.193619126
BLNK	-0.034098375	5.923675671	0.193640232
MYOHD1	0.035794914	6.169889551	0.193643599
FLJ42393	-0.042553281	6.256260984	0.193674706
LOC440525	-0.035471443	6.067725811	0.193686215
PATZ1	0.027193934	6.050532645	0.193713818
SUCNR1	0.030840128	6.079633731	0.19383357
MYCBP	-0.034459109	6.245267263	0.193840159
CACNG7	-0.035797019	5.982135237	0.193898384
LOC652713	0.031554881	6.230140463	0.193979767
LOC642471	0.032722524	6.113248592	0.19400455
OR1L6	-0.038534592	5.947714067	0.194008652
LOC339240	0.02926503	6.00500329	0.194070378
LOC653905	0.028418008	6.075171336	0.194089632
CAMTA2	0.043854142	6.28421784	0.194138671
LOC401087	0.03203784	6.01163327	0.194246273
KRTAP5-5	-0.032460676	5.897031293	0.194296949

FLJ31813	-0.031484931	6.117924375	0.194297605
LOC650433	-0.033843817	6.237254802	0.194313518
LOC200493	0.036750264	6.230655922	0.194319739
DOCK4	-0.039936692	5.933778712	0.194393551
AGXT2	-0.029463431	6.003892132	0.194420105
GTF2IRD2	0.047996822	6.67469026	0.194422108
SC65	-0.055075052	7.034302309	0.19443698
NAT12	0.056239722	6.77148554	0.194449902
C2ORF39	-0.034963306	6.003365317	0.194470569
MFSD9	-0.0315719	6.180474641	0.194550227
PSG5	-0.023067152	5.937930077	0.194584896
RAB4A	0.077716674	8.602571655	0.19462594
LOC642181	-0.040356204	5.982295994	0.194742408
LOC155060	0.031482077	6.25440289	0.194751254
LOC649021	-0.038953049	6.033532054	0.194764138
ANKRD41	0.027020947	6.420107921	0.194786599
LOC653155	0.028856522	6.063685731	0.194938409
PADI2	-0.055217429	6.045382498	0.194999447
SFRS14	0.070649739	8.996696388	0.195038347
CDH6	0.031725051	6.408429531	0.195042975
PLXNA2	-0.045137826	6.565168518	0.19504717
KLHL18	0.047056886	6.894871984	0.195163118
PDE4A	-0.045968008	6.302937237	0.195308423
C20ORF96	0.029809001	6.090217925	0.195330159
LOC196752	-0.078247864	6.782735715	0.195333016
LOC642343	0.028716779	6.069327481	0.19539396
LOC647331	-0.028670285	6.161575969	0.19539467
LOC375748	0.036358079	6.074764743	0.195621153
LOC441136	0.042022723	6.429387334	0.195626547
LRRC23	0.054667609	6.85531504	0.195695357
DKFZP434K191	-0.061908738	7.388738868	0.195705001
LOC644193	-0.034011184	6.161010712	0.195867473
FLJ10803	0.041026519	6.343765355	0.196041065
RBM17	0.050948587	9.39835852	0.19606673
LOC646426	-0.033370551	6.300575603	0.196090109
F7	0.021196992	6.138407991	0.196148654
FLJ43987	0.032778902	6.01050694	0.196149613
CCDC12	-0.059759664	9.261617829	0.196158014
AGBL4	-0.034743957	5.944843168	0.196215909
TMEM163	0.043865524	6.219298171	0.196260141
RSPH10B	0.016485017	6.109814784	0.196261316
LOC389676	0.039711132	6.10234121	0.196452402
LOC646345	0.028968923	6.104831444	0.196456401
LOC653563	-0.031413361	6.048440226	0.19645958
CT45-4	0.018361909	6.029115791	0.196489773
PLEKHG5	-0.019073662	6.035659311	0.196506635
LOC647079	-0.025597191	5.949878608	0.196675783
LOC730249	0.029415897	6.194133153	0.196695351
LOC158730	-0.028069191	5.927674348	0.196699693
LOC341604	-0.02949227	6.125613891	0.197082964
LOC651562	0.03489709	5.964266597	0.197159696

IQCE	0.034928591	6.185276947	0.197160012
OR13G1	0.034444656	6.056318618	0.197168341
LOC653355	-0.051907661	6.153441819	0.197185055
DDHD2	0.054201466	8.254620883	0.197202714
LIPL4	0.023042266	6.087756169	0.197247773
EMX2	0.025988116	6.150897376	0.197274696
RPL15	0.068048294	11.27301134	0.19728207
LOC654335	-0.029812181	6.12333518	0.197314293
LOC644116	0.03390142	6.205159059	0.197338532
ROS1	0.026913833	6.022646295	0.197385449
LOC340156	0.042273993	6.247329925	0.197392759
LOC646589	-0.033987116	6.057718934	0.197461772
KIR3DL1	-0.023832728	6.080837758	0.197495665
GRIK1	-0.022962739	5.976736161	0.197523812
CD5	-0.029630419	6.07467386	0.197655904
RNF112	-0.036427687	6.571316481	0.197705232
HERV-FRD	0.038560831	6.336136	0.197708932
STARD10	-0.083484244	9.03050344	0.197723123
NCKAP1	-0.086367101	8.53462318	0.197747159
LOC649200	-0.031257288	6.016512542	0.197776118
TSPYL4	-0.061048751	6.933756308	0.197806007
LOC641721	0.028854852	6.009811823	0.197839664
LOC646970	-0.033023123	5.874839968	0.197871802
LOC647606	-0.053218961	6.191246522	0.198007624
FLRT1	-0.035868105	6.067392685	0.198015284
LOC644001	-0.038223028	6.207365232	0.198021341
LOC646835	0.030354376	5.968098507	0.198102199
LOC651344	-0.03494678	5.994277008	0.198191386
KCNJ5	-0.048421746	6.472176937	0.198238915
LOC653392	0.032368325	6.237297808	0.198300607
FAM151B	0.035434444	6.180287803	0.198415081
ALPK2	0.11854981	10.97731185	0.198480386
DRG2	0.045297104	7.551874855	0.19853785
NR5A2	-0.031945849	6.292509666	0.198636785
LOC647406	-0.033610743	6.183400094	0.19868577
MGAT5B	-0.027251245	6.111062839	0.198723517
LOC260339	0.022476347	6.090900759	0.198776468
LOC653225	0.035387087	5.923013307	0.198813504
SLC5A1	-0.143247852	9.84496806	0.198853713
ART5	-0.033165368	6.063044698	0.198866997
LY6G5C	0.022703591	6.144189068	0.198893798
ZXDB	0.039328295	6.587937391	0.199201089
DEFB137	-0.03141356	5.942004295	0.199224023
RTP2	-0.036080924	6.148041655	0.199393874
CABLES1	0.051136255	7.154734935	0.199509282
LRRK1	-0.031126941	6.111583341	0.199523952
ASAH3	0.027476547	6.052979368	0.199614118
PPP3R1	-0.087890583	8.430822099	0.199615311
CLK3	0.035520234	7.874707441	0.199630649
LRRTM1	0.026015598	6.116087574	0.199671852
LOC645967	-0.044001465	6.075682398	0.199722075

LOC649431	-0.03359829	5.996883382	0.19979854
SOCS6	0.057384813	6.212911626	0.199854209
LOC641726	0.033954797	5.930628905	0.200007367
LCE3B	0.032236491	5.991528416	0.200051936
MYCBP2	0.056258774	8.56835548	0.200070145
HIST1H2BD	0.08202558	9.098368794	0.200170837
MTMR1	-0.02228187	6.156448687	0.200205669
LILRA4	0.059318171	6.001689256	0.200242473
LOC647060	0.03993186	6.137438103	0.200265337
OR4K5	-0.028800183	5.996609844	0.200353578
GOT1	0.071536728	12.87722458	0.200468827
LOC654117	-0.031337372	6.171207538	0.200548109
LOC643491	0.030200867	6.021182657	0.200628097
LOC648145	0.027662092	6.161881825	0.200764135
KLRC3	-0.019872937	5.965191377	0.200781536
TSHB	-0.033056369	5.982302749	0.200814082
BAGE4	0.026940982	5.934593456	0.200825005
LRRC37A3	-0.032924264	5.895098954	0.200836978
LOC645783	0.025730079	6.008439409	0.200840905
TMEM119	0.131433481	7.028008972	0.200886945
LOC646939	0.026782123	6.071555506	0.200887079
LIPF	-0.032209501	6.292389795	0.200997138
LYRM4	-0.063713657	6.760098061	0.201025704
CWF19L1	-0.060882321	9.317275238	0.201051101
OR10H2	0.028548993	5.94058888	0.20109911
MTFR1	-0.08214198	7.772409368	0.201119123
MIA	0.053047308	6.062122435	0.2011496
ARMC8	-0.033109204	6.599175555	0.201150544
LOC440348	-0.074199622	8.14291259	0.201186369
LOC644083	-0.034049206	6.085448155	0.201198446
MORN3	-0.038193232	6.028677876	0.201249712
CHSY3	0.056615014	6.585948372	0.201258588
ARL6IP4	0.060882236	8.091261929	0.201388845
LOC390033	0.027212472	6.055223016	0.20141091
HES3	0.030968512	6.125589071	0.201415383
GNPDA2	0.045683676	7.312769958	0.201455055
DNAJA1	-0.092684749	10.69014515	0.201530779
SYT16	0.053361407	6.141649934	0.201596503
FLJ23172	0.030366438	5.893374043	0.201685299
ESX1	0.03264243	6.033494621	0.201743288
LOC283585	0.027699231	6.085735148	0.201809217
LOC728395	-0.034422918	6.098495684	0.202037578
WDR26	0.050589381	8.141593033	0.202079614
AMIGO1	0.042905891	6.313645995	0.202108631
MRFAP1	0.051911501	11.40496419	0.202232244
C16ORF70	0.041127938	6.936142105	0.20229343
AGTRAP	-0.069153062	6.910106404	0.202362459
LOC390714	0.046317211	6.091639259	0.202374176
CLEC14A	-0.086101417	9.980054682	0.202430076
LOC649821	-0.066053491	11.63803569	0.202458482
CTBP1	-0.049036513	8.458381569	0.202473071

C19ORF44	0.044653469	7.00491108	0.20256611
IL17F	0.034072561	5.955923911	0.20263085
LOC440993	0.087826542	6.651158678	0.202680916
LOC652271	-0.028997529	6.192597632	0.202843967
CA9	0.039440532	6.250998729	0.20293144
NMU	0.034358378	5.876772535	0.202942195
RP1	0.024399811	6.012967864	0.20311638
FGF3	-0.035541567	6.072936068	0.203198332
LCORL	0.023886319	6.005103124	0.20322424
PRR7	0.041297481	6.140141033	0.203230982
LMAN1	-0.051027349	6.571060537	0.203243334
EPHX1	-0.095071538	7.268987148	0.203258942
MGAT5	0.073393853	9.027612726	0.203359965
PAGE1	-0.031689264	6.005407612	0.203376247
LAMP3	-0.024418192	6.050319301	0.20340108
FAM126A	0.025096649	6.181521688	0.203478448
NAT15	-0.095449398	6.86207053	0.203506307
TSNAXIP1	0.049124511	6.017048866	0.203544842
DKFZP434B061	0.023017059	6.05159971	0.203675709
LOC644672	0.043055396	6.163860766	0.20369146
PSITPTE22	-0.025524915	6.020423043	0.203758847
CLIP2	0.038513662	6.555157127	0.20377817
KRT23	0.027522513	6.433535847	0.203810785
LOC650689	0.031385898	6.220750165	0.203831538
AP3S1	-0.066197895	8.132620971	0.203896834
LENEP	-0.03301304	5.987881025	0.204006894
MGC52282	0.034089004	6.058374586	0.204332091
ZNF579	0.048590774	6.529517828	0.204420479
ADAMTS16	-0.032860319	6.145873902	0.20442854
C20ORF165	-0.032007835	6.108046419	0.204452687
LOC643904	0.025086199	6.016104478	0.20446154
TDGF1	0.021210705	5.98068093	0.204481576
SFMBT2	0.047652334	6.58645446	0.20454159
PIWIL1	-0.028499307	6.205089628	0.204572803
LOC644097	0.034299997	5.919985524	0.204576181
C13ORF3	0.034410687	6.10946908	0.204589641
EFCAB5	-0.020193923	6.016362861	0.204612083
LDB2	-0.078552513	9.942723103	0.204612366
TFAP4	0.036511806	6.322468599	0.204621096
LOC730417	0.019152718	6.258154736	0.204643887
C20ORF185	-0.030367225	5.994374522	0.20468416
CLN5	0.051657057	7.889903604	0.204727572
SCUBE3	-0.033175575	6.211869825	0.204730244
FER1L3	0.0891995	8.478335075	0.204787762
SKIL	-0.022520278	6.038918457	0.204896096
LOC643895	-0.031470685	5.92218417	0.204964544
ZNF585B	0.027794509	6.208052272	0.204969354
DUSP6	0.074762202	7.092599905	0.204996335
CRYBA1	0.034496294	5.992845901	0.204997015
SNCG	-0.044705598	6.373818156	0.205039983
TGM3	-0.038689616	6.234081841	0.205177162

RNF31	-0.046617161	7.608836972	0.205217889
LOC651141	-0.030864128	6.001465324	0.205222887
SPOCK3	-0.033028447	5.876891549	0.205255305
NAPA	0.056525982	6.846620205	0.205256174
RAD54L	0.042976255	6.113018019	0.205269685
ALK	-0.029305544	6.204676544	0.205325215
LOC650167	-0.022292164	6.064564581	0.205417957
FBXL7	0.083908529	6.508534113	0.205455214
LOC645212	0.03586248	6.375725597	0.205511705
SLC5A2	-0.024065083	6.033818264	0.205547691
LOC391358	-0.029392048	6.073307606	0.205550142
GPATCH3	0.057720131	6.738513222	0.205801642
IQCH	0.02119095	6.00435875	0.205812493
LOC139587	-0.028563721	6.025898041	0.205939772
LOC653199	0.024719131	6.307006151	0.205995186
KRT26	-0.025706141	6.034932316	0.206026683
EDG1	0.0740113	8.590164079	0.206068333
WNK4	-0.02932573	5.997952635	0.206074233
MANSC1	-0.037036379	6.715850586	0.206159643
MORC4	0.041536912	6.301143043	0.206203538
ZNF527	0.03423432	6.249911804	0.206230489
PREX2	-0.020592827	6.006050754	0.206287709
ROR2	-0.033333425	6.287810487	0.206346574
LOC440742	0.02990845	6.017293545	0.206451454
METTL6	0.037089234	6.461736899	0.206494462
FSBP	0.028485335	6.125798163	0.206498761
NAPG	0.07346466	6.797418576	0.206598013
TSPYL6	-0.037598613	6.067225222	0.20660888
C14ORF115	0.043303817	6.086381476	0.206608947
SEC61G	-0.068190204	11.75138654	0.206652993
INO80E	-0.043140764	7.583302478	0.20666843
LOC652789	0.03535641	6.222102569	0.206679183
LOC645520	0.025055181	6.068079463	0.206712228
ARFGAP1	-0.055863511	7.046519352	0.206717138
LOC644347	0.033131589	5.888172284	0.206827262
LOC652490	-0.028901012	6.053004327	0.206913863
LOC649859	-0.029288249	6.12271635	0.206921401
DDN	-0.047775816	6.260916738	0.207002918
LOC651914	0.031953739	5.926050136	0.207035062
LOC730877	0.030142647	6.321489365	0.207036963
MAGEB16	0.033958797	6.027145156	0.207065297
C1ORF147	-0.041019252	6.177204832	0.207122649
LOC651115	-0.034727188	6.041005426	0.207182203
C2ORF15	0.028854154	6.069802874	0.207240214
HIST1H2BN	0.034340983	6.064086123	0.207306716
CDC42EP5	-0.080648948	8.329123096	0.207315225
LOC440354	0.05402767	7.631556859	0.207489023
LOC645073	0.03525862	6.043257998	0.207494483
NEFH	-0.033270295	6.048695741	0.207552892
PTPRB	-0.068572283	7.976141413	0.207616864
RPL22L1	-0.038537635	6.155822429	0.207639563

ZNF746	-0.041200046	7.748709053	0.2076636
PYY	0.033391848	5.962933465	0.207767197
LIFR	-0.071429871	7.00658411	0.207781243
LOC643718	0.028126045	5.975772982	0.207783154
WASF3	0.058228374	7.782920952	0.207987594
DSCAM	0.024554746	6.513525473	0.208057708
STYXL1	0.077958241	7.792444944	0.208082749
GCG	0.035595914	6.199739261	0.208109608
SPARC	-0.090000392	10.95467325	0.208125017
LOC649680	0.039677228	6.054208053	0.208223127
C8ORF78	0.0325421	5.915681752	0.208233023
IL23A	0.030609793	6.017762662	0.208308276
NASP	-0.034673549	7.040658752	0.208357765
RAXL1	-0.089814101	8.68034277	0.208361546
LOC730411	-0.027881492	6.033931615	0.208368787
CYB5R3	0.053208058	12.2677465	0.208373949
HCN4	-0.041611611	6.333291344	0.208446721
CT45-6	0.030627989	6.077823047	0.208490395
G3BP1	-0.078009074	7.521944151	0.208523359
S100G	0.031339482	5.908340022	0.208526058
NUDT11	-0.027074124	6.218590357	0.208537367
ZNF804A	-0.035074175	6.117232007	0.208563772
LOC652400	0.031702177	6.261946597	0.208600084
WDR54	0.071708733	7.784560984	0.208642663
HIST1H3F	0.053741867	6.14277779	0.208661715
STAG3L1	0.04125833	6.837399238	0.208683279
OR1L1	0.031204884	6.004647475	0.208734958
PTP4A3	-0.104636047	10.12034045	0.208808634
CNIH2	0.018677103	6.082010507	0.208852063
LOC402279	-0.033147559	5.910183206	0.208902981
LOC654256	0.029637667	6.192706006	0.208903915
SSB	0.050595153	8.435796945	0.208995478
THPO	-0.028355395	6.046348216	0.208996487
SLC1A2	0.055905032	6.218887084	0.209120252
AMTN	0.031584423	5.891321919	0.209196014
TNFRSF18	-0.020666339	5.990866605	0.209415593
PRTG	-0.018543475	6.002721968	0.209433624
TNFRSF8	-0.024311253	6.116125525	0.209479063
LOC728505	0.038615602	6.374739202	0.209480351
AMH	0.031550291	6.045245496	0.209490741
LOC652645	-0.032142071	5.924200336	0.209557815
WDR40B	-0.02114871	6.12432773	0.209686222
LOC646774	0.038179864	6.289748979	0.209693468
MT4	-0.036015814	5.822380316	0.20975682
PLK3	0.054528453	6.042711499	0.209791229
CD24	0.05606117	6.267868798	0.20981983
HIST4H4	-0.028820592	5.979382574	0.209870262
C4ORF6	0.032910744	6.10430783	0.20989868
CLEC4A	0.032464709	6.468013736	0.210052628
ZNF581	0.054524393	7.575627235	0.21010449
ANKRD13D	-0.016566822	6.243165341	0.210148023

LOC650494	-0.034407088	6.021992455	0.210258189
LOC441127	0.030594222	6.074632594	0.210302973
LOC652680	-0.028404996	6.00701154	0.210374261
BCHE	-0.068984011	6.771938125	0.210393326
NAT8	-0.03262511	6.092431631	0.210397097
CHDH	-0.034657911	5.907092908	0.210408896
OR52H1	0.033127372	6.128692079	0.210411173
LOC644916	0.03275736	6.014743028	0.210443244
CCNE1	-0.029677945	6.438566707	0.210480355
LOC643318	0.027877522	6.035085456	0.210497908
C2ORF37	0.038771575	6.249383771	0.210599792
LOC727948	0.13582301	7.872692989	0.210605182
ZBTB11	0.070573778	6.539962362	0.210634175
LOC442406	-0.026372815	6.047992962	0.210653108
TRPC3	-0.033261922	6.040334446	0.210700242
PTPDC1	-0.03681129	6.710089307	0.210722939
MOCS3	0.029656368	6.211412558	0.210748011
TAF9	0.073342957	7.297831318	0.210748945
STX1A	-0.035440665	6.343539741	0.210808332
HYAL1	0.071928683	9.193352843	0.210930159
CMTM4	-0.029717445	6.792730096	0.210980138
LOC653579	0.029881087	6.144209988	0.211005317
GDI2	-0.06893402	9.776614956	0.211047524
ANP32E	0.046537856	5.966682929	0.211056268
C15ORF60	-0.042615302	6.103652305	0.211059567
GPR63	0.0338946	6.093352328	0.21110076
LGI4	0.065867287	6.269658959	0.211199915
C21ORF66	0.038945413	7.262592636	0.211204393
DKFZP434B0335	-0.062510871	6.913307984	0.211247126
ZNF331	0.058656786	6.989497517	0.211269702
FAM71F2	0.030931524	6.045603566	0.211281233
SLC12A5	0.02809769	6.142810688	0.211353469
LOC732372	-0.039045342	5.916418662	0.211391406
RTDR1	-0.029453553	5.935348504	0.211441946
NPY	-0.030379725	6.093577047	0.211588551
LOC644196	0.022974534	5.969797989	0.21159138
LOC641765	0.024714244	6.245461595	0.211608161
FAM118B	0.040296424	6.842585093	0.211704674
ZFP41	0.026858021	6.050479301	0.21178216
LOC650329	-0.029900922	5.990492752	0.211809528
TRPC2	-0.025758559	6.011645015	0.211885521
FLJ45832	0.030778753	6.207821977	0.211891097
ULBP3	-0.034154854	6.225008069	0.211907742
AOC2	0.017750639	6.093928518	0.211972245
LOC441119	0.033655964	6.025170835	0.212051142
PSG7	-0.024011225	5.933013991	0.212070624
C12ORF24	0.078678994	7.484463903	0.212183515
HCG3	0.045366938	6.002905108	0.212185007
LOC388813	-0.031576285	5.979911847	0.212245536
C10ORF93	0.019307034	6.011376452	0.21229654
LOC642167	0.029930076	6.178265184	0.212324988

TP73	0.037873429	5.9832952	0.212368037
LOC647049	0.0302242	5.977808291	0.212417604
SPNS3	0.031884405	6.060127478	0.212516465
GGN	0.031166393	6.021157296	0.212579032
LOC254398	0.030441109	6.15373403	0.212608338
FOXP4	-0.026839487	6.263182355	0.212609164
LOC647843	-0.026675541	6.046607233	0.212609318
LOC645227	-0.02857911	6.038497963	0.212646902
LOC644931	-0.105206997	7.158043805	0.212804694
NEK5	0.030581559	5.923782668	0.212847379
ADH7	0.03066687	5.96432239	0.212849299
ATP5J2	-0.036712044	11.92375113	0.212882544
IDH3A	0.07651098	10.22232847	0.212908206
NRD1	-0.054285914	10.55262876	0.212973168
FOXO4	0.116505738	8.175797084	0.212986703
LOC650562	0.032292902	6.174053146	0.21306774
MAP3K11	-0.053249888	7.493348634	0.213122373
SFRP2	0.049134016	6.049642241	0.213203104
FEV	0.044879114	6.045197705	0.213274556
CRTC1	0.035744395	6.404109281	0.213365059
LOC730256	0.036716311	6.570199371	0.213534687
LOC652612	0.032918816	6.088678019	0.213652063
DUX5	0.03871739	5.900241292	0.213737515
PPT2	0.022339796	6.49476071	0.213748417
LOC648127	-0.02839645	5.993886975	0.213787977
LOC647519	0.030388269	5.981989121	0.213801489
LOC650498	-0.030697038	6.052804632	0.213884073
CARS	-0.057134389	7.282407593	0.213916916
LOC283999	-0.031791335	6.33308963	0.214273546
FAM161B	0.034381771	6.079747429	0.214299323
RANBP9	0.060165992	6.545911453	0.214410729
SGCB	0.083662035	7.753150995	0.214655387
LOC646132	-0.032572494	6.16779616	0.214685721
PRDM4	-0.064415743	8.085599141	0.214695228
DPM3	0.063847968	8.933945997	0.214806996
ANKRD13B	-0.036100855	6.170288252	0.214816245
LOC646772	-0.03116297	6.033298477	0.21485808
LOC728481	0.079576738	11.88382777	0.214921613
TMEM156	0.040569677	6.417123228	0.21495791
GPR157	-0.029391834	5.962493191	0.215172201
LOC645551	-0.029315003	6.044639518	0.215264044
KIAA0586	-0.043767403	7.446511434	0.215470228
LOC643696	0.029126974	6.079791273	0.215718644
TBPL2	0.032645938	6.193941073	0.215800379
CEACAM20	0.032097842	5.962270956	0.215801715
LOC649817	0.03104898	6.05286893	0.215805599
GNL3L	-0.082090923	8.458015057	0.215815054
LOC642185	-0.030697975	6.169416501	0.215828643
C10ORF37	0.023438407	6.064590863	0.215863544
SLC12A2	0.061703861	7.415313588	0.215886259
SLC4A3	-0.111080952	8.877252213	0.215935668

LOC650961	0.036092866	6.054303408	0.215948718
SLC24A5	0.032880733	5.948921827	0.216017285
SRP68	-0.054334561	9.43145074	0.216176051
LRRTM3	-0.024140379	6.007087512	0.216275002
KIFC3	0.050219046	5.98265293	0.216315237
NCOA6	0.048169012	8.917561344	0.216467342
CENPL	-0.035002736	6.379478172	0.2165281
PAX6	0.037187022	6.162638179	0.216533572
TXNL4B	0.047281272	6.607281581	0.216536238
ZNF143	-0.061051239	7.489673878	0.216556099
IL17REL	-0.032014499	6.148286437	0.216582764
GLRX5	-0.052777763	12.38181766	0.216736023
LOC645186	0.034384147	6.118265862	0.2169113
LOC644172	0.027923983	6.272145569	0.216942466
SCYE1	0.03067446	6.075695688	0.216984003
NR5A1	-0.029538449	6.02481602	0.217050889
PSKH1	-0.037792163	6.218404215	0.217110313
TM9SF4	-0.043643403	8.545647347	0.217126091
SOAT1	-0.039900221	6.436950948	0.217226478
C6ORF27	0.032480552	6.238530291	0.217291095
RAB20	-0.052450482	6.789557729	0.217325551
CDC37L1	-0.078757537	6.523917739	0.217330014
C10ORF49	0.044775152	6.270053989	0.217500462
LOC643225	-0.026982611	5.940502754	0.217501428
LDB1	0.048745717	6.164454322	0.217593904
TMEM164	0.05934775	6.997141059	0.217660857
MYT1L	0.021215535	6.02038579	0.217665466
KRT1	0.030628277	5.924356213	0.217691001
ACOT11	0.098085055	8.955369508	0.217778416
LOC402066	-0.038075196	6.645101033	0.217817427
LOC340228	0.035821674	6.042863236	0.217861598
LOC389365	0.036820605	6.192060151	0.217935729
ACN9	0.069987549	8.597025555	0.217958217
C20ORF160	0.056216448	8.30932818	0.217968603
OR5AN1	-0.029159501	6.09651978	0.218065638
LOC283487	-0.034725575	6.245891456	0.2181912
IFNA8	0.052333949	7.048252034	0.21825499
LCE1B	-0.035150696	6.118256576	0.218309676
ZNF345	0.025813493	6.224198906	0.218311454
LRAT	-0.032003454	6.056793578	0.218349135
SLC5A12	-0.018234604	5.961834085	0.218367524
SULT1A4	0.040934254	6.942327123	0.218379062
OR2T29	0.029365402	6.011456436	0.218389225
LOC399746	-0.028502536	6.028761992	0.218446161
PSMD9	-0.033544677	6.693719539	0.21850597
RBM15B	-0.055378836	6.367315361	0.21853891
EPS8L1	0.019354129	6.046869818	0.218594782
SEMA3G	0.070011676	6.360765464	0.218620899
XKR7	-0.029359818	6.07561791	0.218624444
DBNDD1	-0.059339881	6.932628163	0.218635657
LSM5	0.069414935	9.608287247	0.218662633

TBR1	0.030766717	6.005704718	0.218714817
ACSF3	0.03585927	6.114653193	0.218816269
LOC390876	-0.042108813	6.350358642	0.218818693
RNF17	0.019525709	5.98498855	0.218948747
C1D	0.026335318	6.158255707	0.219005144
DAZ2	0.022753374	6.054173174	0.219023045
IMPAD1	-0.065729978	8.112505741	0.219051784
LOC644168	-0.031072609	6.241975927	0.219105391
TLX1	0.036344186	6.108072841	0.219162968
LOC645839	-0.029262141	6.014474139	0.219275187
ARVP6125	0.031876199	6.243699259	0.219325233
LOC643125	0.028880064	6.073844506	0.219425796
LOC644769	0.030611797	6.04207152	0.219477067
LOC400197	0.030752856	6.172104769	0.21947897
ZFAND3	-0.049282825	8.174158088	0.219554228
LOC644427	0.033596484	6.010021439	0.219597516
SLC2A10	0.040260333	6.72858515	0.219679238
CCDC138	-0.032728768	6.125605691	0.21975456
HCP5	-0.075458757	8.784061059	0.219756459
MEI1	-0.02082471	6.124666415	0.219771156
RASGEF1A	-0.041355284	5.832870737	0.219824354
DACH2	0.030506491	6.053672398	0.219923199
CDRT15	0.030318868	6.026359941	0.219944841
LOC641719	0.034985995	5.930567793	0.220159062
C14ORF125	0.052910704	6.302281733	0.220334445
IFNK	0.042556412	5.909716236	0.220338737
L3MBTL3	0.022620826	6.204019993	0.220371329
LOC646359	-0.027820418	6.073799208	0.22058595
GPC6	0.045251997	7.505046927	0.220596519
TNFSF14	-0.067479308	8.017835216	0.220756995
TMCC3	-0.036928737	5.9982998	0.22078102
LOC642879	-0.030034583	5.989187664	0.220954187
LOC401137	0.02035595	5.980904627	0.221046157
UBAC2	0.062027643	8.800286828	0.221065036
LOC388565	-0.030568681	6.288956113	0.221083015
MCOLN2	-0.041512684	6.175497288	0.221104153
LOC650724	0.031128019	6.139705507	0.221124545
IFRG15	-0.0350337	6.143375962	0.221185463
LOC255620	-0.023588009	6.065485582	0.221260549
AMAC1	0.038618012	6.184531425	0.221329806
P2RY4	0.041586587	6.049276084	0.221362131
SLC39A5	0.042674744	5.966728124	0.221462049
LOC654056	0.032729979	5.858459459	0.221464597
CAPN10	0.021348894	6.39145541	0.221499414
LOC648576	0.026932087	5.959578532	0.221546242
C8ORF48	0.041084373	6.812173907	0.221611004
LOC643146	0.027063812	6.099492536	0.221670097
CNIH3	0.032691841	6.096652669	0.221790015
FLJ20433	-0.039827233	6.363730388	0.221791564
TUBB8	0.031698995	6.012721601	0.221812952
LOC643505	0.038307109	6.748105382	0.22184824

C14ORF70	-0.031581182	6.036244606	0.221880114
DPPA3	-0.020552129	6.031894592	0.221968334
IL22RA2	0.026738728	6.042745918	0.222033043
LRP1	-0.080182203	6.710889891	0.222093832
FLJ35776	-0.02272124	6.01204834	0.222170362
LOC652183	-0.033194893	5.92990578	0.222181451
PRDM13	-0.032724896	6.01237381	0.222188392
POU5F2	0.037623073	6.159999511	0.222200471
LOC650919	0.038235176	6.580656446	0.222205015
SLC16A8	0.033068321	6.070829632	0.222240581
LOC652808	0.028861649	6.077708188	0.222249984
GALNT4	-0.044773754	6.39013429	0.222264076
MIF4GD	0.111633805	7.296297788	0.222305467
YTHDF2	-0.048785695	8.56496124	0.2223198
LOC649318	0.029310895	6.162074781	0.222351848
KIAA1522	-0.041880047	6.55617128	0.222404228
LOC643012	0.036216864	6.084253664	0.222411911
LOC402269	-0.029312058	6.03318369	0.222674532
LOC652886	0.030832186	6.277419834	0.222723534
ADARB2	-0.030562571	6.179124657	0.222741098
LOC649214	0.022228239	6.0671889	0.222931713
LOC643805	-0.033887302	6.101594306	0.222940021
ZKSCAN5	0.023509675	6.27477015	0.222994518
C10ORF18	0.031024847	6.133825624	0.223078375
LOC649203	-0.03096029	6.228916663	0.223212508
C3ORF65	0.021071112	5.962680503	0.223219154
LOC641810	-0.031966274	5.937809681	0.223572581
MAPRE3	0.05776171	8.548329287	0.223577075
AQP6	0.036623715	6.906850377	0.223589264
GATA1	-0.031282737	6.012213532	0.22372702
LOC653349	-0.017224345	6.120765391	0.223864708
LOC647147	-0.033015448	6.009261301	0.223954301
LARP4	0.080011658	8.179518434	0.224146291
FLJ32867	-0.02821437	6.090411031	0.22416628
SMN2	0.050912361	6.293499922	0.224208802
LOC645261	0.031724395	6.327724789	0.224214766
LOC729378	-0.032643298	6.098290872	0.22429591
LOC642563	0.032483758	6.104419151	0.224354539
YY2	-0.029330325	6.104039302	0.224432229
AP3M1	-0.045847327	7.616874455	0.224469732
LOC651004	-0.030891871	6.156064719	0.224586177
WNT9A	0.03000312	6.236600952	0.224756388
MTO1	0.040877559	7.790474166	0.224930539
LOC647546	-0.028931226	6.096874833	0.224936812
PSG4	0.020441599	6.00695944	0.225006479
LOC648217	-0.027992409	5.948725772	0.225056195
LOC222967	-0.01638346	6.117826494	0.225112185
CYB5R4	0.050636297	7.345111516	0.225130996
KLHDC4	0.058378133	7.305222017	0.225142784
LOC644876	-0.031319943	5.977964693	0.225181291
CSTF1	-0.023626934	6.075793512	0.225247204

BAT2D1	-0.05254021	8.540937283	0.225249644
LOC650254	0.033026298	6.007892264	0.225255742
LOC653829	-0.067102618	7.040077769	0.225285076
SF1	0.03284484	6.617909392	0.225292514
REEP2	-0.058457357	6.461759682	0.225312978
FLJ42177	-0.028992268	6.154112105	0.225348237
AMBP	-0.037704552	6.248126414	0.225406008
NME2P1	0.031164992	6.130058308	0.225419111
TRH	-0.031733908	6.129954727	0.225491854
PLVAP	-0.081182669	6.691385267	0.225583953
TMEM199	0.051098164	7.307532448	0.225677955
LOC648210	-0.071962405	10.63751542	0.225678639
HEMGN	0.016780393	5.977322672	0.225727832
LOC441208	0.063775511	6.505880144	0.225782963
L3MBTL4	0.029418184	5.98682448	0.225867623
LOC731999	-0.034788428	6.486298195	0.225933264
UGT2B7	-0.159418916	7.002775749	0.225967946
GLT1D1	0.051669368	6.029655145	0.226029568
TNPO2	-0.051886114	9.493459615	0.226062452
LOC652838	-0.030673433	6.097631172	0.226261291
LOC653618	0.030053956	6.025917931	0.226431143
LOC654192	0.04419722	6.182398649	0.226433904
KIF11	0.025626945	6.221890599	0.226688116
LOC647474	0.024581091	6.293332195	0.226746092
SMPD1	0.041056779	7.02355902	0.22674733
ARHGDIG	0.049039993	6.551881674	0.226767766
LOC221710	-0.035655874	6.58734762	0.226853163
LOC348840	0.056903633	6.088349881	0.226891616
BAIAP3	0.034555548	5.995869611	0.226955789
LOC646554	0.035436238	5.962601901	0.226970461
LOC653641	0.022671334	6.063754173	0.227162239
LOC643341	-0.026767995	6.094126574	0.2271651
OR4C46	0.03295023	6.13289819	0.227253886
PROKR2	0.02958123	6.023534272	0.227282822
LOC729786	0.03307912	6.027846374	0.227326378
LOC401252	0.02942685	6.100163359	0.227420448
LOC650235	-0.029652435	6.182628987	0.227478341
LOC401101	0.031542158	5.963587318	0.227603998
PRIM2	0.049230993	6.356454005	0.227674822
ACY3	0.028127675	5.943310308	0.227737491
NCSTN	-0.064584021	10.44905318	0.227861763
IDE	0.054060305	7.334140155	0.227899338
DCUN1D4	-0.043420479	8.0928765	0.227964238
LOC388503	0.018546388	6.070392088	0.228066874
PRH2	0.03027919	6.117517935	0.228099211
CD300E	0.030839622	6.164690435	0.228101271
ZCCHC11	0.043826379	7.324582104	0.228103353
LOC442388	0.025736375	6.074179099	0.2281619
LOC653576	-0.024954683	6.033345723	0.228266486
C3ORF31	-0.062357553	8.51560889	0.228269684
LOC651231	-0.03046324	6.072170177	0.22830383

KIR2DL4	-0.020777516	6.100791678	0.228322463
OCA2	0.033752033	5.945681934	0.228336748
CYORF15A	0.13365786	6.800999246	0.228450987
OR7D2	0.042201119	6.014870578	0.228592134
LOC644452	0.033186466	6.160671736	0.228597026
ANKRD35	-0.044699852	6.765581524	0.228603124
FAM90A1	-0.038321665	6.052543807	0.228625395
C16ORF46	-0.030730347	6.088106168	0.228701347
ERCC8	0.020155223	6.270963685	0.228727664
LOC651007	-0.027204896	6.258370257	0.228791104
CD81	-0.041618289	13.89679794	0.228891871
GATAD1	0.038048993	6.882573036	0.228973794
LOC653507	0.02836323	5.939331869	0.229037464
TCHH	0.032079199	6.427434381	0.229053263
C8ORF46	-0.052022386	6.504155303	0.229138506
LOC643937	0.033853415	6.28815074	0.229153657
XKR4	-0.059245039	6.430852775	0.229167008
LOC642767	0.026975481	5.98825523	0.229176456
LOC649060	0.029320982	6.038776782	0.229296495
LOC654254	-0.033602537	6.087568394	0.229321393
LOC646926	0.028153135	6.095527707	0.229422409
FGF5	-0.022601279	6.168264976	0.229447552
LEKR1	0.029667676	6.270310027	0.229484078
FAM44A	0.03299164	6.025136294	0.229500115
LOC642592	0.028241368	6.003723461	0.229555508
AKAP2	-0.050651764	6.567316412	0.229594352
LOC651150	0.027070629	6.088935847	0.229656354
BTBD11	-0.018516891	6.131308752	0.229781913
GNG10	0.078462355	7.894342976	0.229784616
A26C3	-0.019651676	6.177860508	0.229923807
LOC643808	0.035104914	5.932997093	0.229961656
CDC23	0.04983878	8.565214057	0.229976614
SUMF2	-0.052104604	8.925805614	0.230051653
TFDP1	-0.041306022	9.044652528	0.230053542
LOC641996	-0.034584577	6.314824569	0.230085603
LOC646627	-0.021491782	6.003363089	0.230120822
PARC	0.061369869	6.335784808	0.230122747
SPRR2C	0.031268787	5.972872654	0.230180354
SLC28A1	0.025380275	6.089678249	0.230337728
BCCIP	-0.056505792	8.464223279	0.230415946
LOC388849	-0.01896026	6.051717514	0.230431023
TMPRSS11D	-0.034634352	6.0589092	0.230526324
LOC644360	0.043779989	6.086328575	0.230532887
C22ORF23	0.03238903	6.62179801	0.23053412
LOC644003	-0.029506785	6.058205466	0.230643129
CERKL	-0.016010589	5.949687755	0.23072533
RFT1	-0.037723423	6.201617909	0.230883796
TAF12	0.047784978	7.939751157	0.230894592
SLC25A34	0.135069306	7.700623053	0.23101362
F9	0.030963984	6.204600038	0.231018558
ARID2	0.037687118	6.896837222	0.231084785

KIAA1530	0.027041612	6.555063203	0.231130564
PCAF	0.058334496	6.390759027	0.231145923
PYDC1	0.030100155	6.056120524	0.231220629
LOC339674	-0.019891097	6.031555526	0.231230982
LOC641365	0.042627249	6.286489459	0.231256161
PPP1R2P3	0.032207768	6.001535155	0.231258898
TRAM2	-0.053921633	7.655932455	0.231307888
LOC644884	0.031371353	6.190408616	0.231390181
C4ORF23	0.031515622	6.496788895	0.231403357
C1ORF129	-0.034221065	6.171028888	0.231451001
FAM81B	0.036677358	6.130029063	0.231531708
LOC285768	0.027792377	5.977975175	0.231571392
LOC654252	0.029415925	6.107982347	0.231683601
STX11	-0.081695488	7.866780113	0.231746421
CHD9	0.064299377	9.215730639	0.231781609
LOC388963	0.027131658	6.062095246	0.231803103
MRPS31	0.045896074	9.619645638	0.231831331
SGTB	-0.023000455	6.098427086	0.231957875
LOC643949	-0.099763523	8.982553817	0.231976307
LOC402617	-0.032588806	6.044861778	0.232007212
ELMOD1	-0.041431724	6.28093322	0.232057605
COX11	0.046597372	6.807630107	0.232120813
C19ORF35	-0.030838001	6.133051442	0.232327328
FAM83C	0.031304833	6.102880785	0.232474467
ZNF446	0.041761714	7.095580857	0.232493219
FLJ46300	0.027046208	6.243709999	0.232508598
MEGF11	-0.031977749	5.896926593	0.232656534
LOC284422	-0.026807081	6.05891536	0.23266546
LOC220433	0.115632773	10.20268076	0.232699894
WDR41	-0.069013193	8.025394084	0.23275885
ITPR1	0.061143384	7.703452121	0.23278168
RGPD7	0.034515279	6.062759288	0.232861685
LOC645294	-0.029582094	5.932847406	0.232908808
LOC643131	0.031015642	5.954399691	0.232929356
LOC643070	0.028892391	6.08162571	0.232951399
LOC728516	-0.03094196	6.027273956	0.232976421
ZNF582	0.044342995	5.996726445	0.233019353
F2R	-0.067218363	13.52518577	0.233050671
PRAP1	0.031587533	6.125415046	0.233175083
LOC728285	0.033443621	5.988966029	0.233202044
LOC388915	-0.025499423	5.859825811	0.233210705
ZNF620	0.031416522	6.101486996	0.233231801
ZNF609	-0.040761889	6.653915976	0.233271017
LOC388312	-0.029328903	6.051241753	0.233360224
LOC728811	-0.141985781	6.90454516	0.233452471
LOC647711	0.025395887	6.043628031	0.233471816
LOC651892	0.026196478	6.170339102	0.23350479
LOC643662	0.031134095	6.111414849	0.233513285
CASKIN1	-0.030159807	6.036346024	0.233582716
DISP1	0.072453767	7.720210441	0.233630184
GLT8D4	-0.03702221	6.055391148	0.233919277

MRPL23	-0.05847001	10.6533619	0.233938346
RPS6KB1	0.057514089	8.910254448	0.233973413
MAPK10	0.02013179	6.226719081	0.233993018
DEFB104B	0.026334028	5.956684694	0.234040587
UFC1	-0.067003713	9.769505278	0.234076763
CTSD	-0.070949412	9.916319248	0.234165403
LOC647208	-0.034234237	5.99390704	0.234216167
LOC642995	0.015327894	6.080200077	0.23435114
LOC651503	-0.032216281	6.241478262	0.234354574
DDI1	0.049326877	6.115023346	0.234513683
LOC649978	0.028216686	6.125171662	0.234536413
CCDC35	0.033982579	6.219214667	0.234555648
DYNC2H1	0.058548373	6.65702083	0.234621835
C1ORF21	0.059244271	8.40692175	0.234661895
LOC650853	-0.031438942	6.345758667	0.23468592
LOC651738	-0.034459548	6.335723456	0.234688577
LOC441205	-0.027424313	5.910226957	0.234722234
VIL1	-0.029715485	5.960962412	0.234737809
LGR5	0.03143318	6.105525685	0.23480032
FLJ45684	0.027962956	6.017649584	0.234868938
PDCL2	-0.033937848	5.909250012	0.234869713
DMRTA2	-0.031448996	6.095899036	0.234922302
VKORC1	-0.05414874	10.13450214	0.234928539
FMR1	0.032508186	5.959141934	0.234972743
OR13A1	0.029734479	6.077272865	0.235123956
MCRS1	-0.058643932	9.580680341	0.235137995
MAP4K5	-0.038450928	7.256855874	0.235238232
LOC613206	0.028814046	6.344221252	0.235239637
OLIG2	0.029300145	6.038420813	0.235367587
GPM6A	0.021323811	6.289654065	0.235395212
ZNF597	0.03048177	6.263095954	0.235415286
LOC651731	0.030380153	5.93293118	0.23557847
LOC651202	-0.122186277	9.332187896	0.235610717
LOC729758	0.028137493	5.990193104	0.235665753
ZNF480	0.07903324	7.409895906	0.235668584
TCBA1	-0.030118249	5.965432428	0.235679526
SIRPB1	-0.034575337	6.035364928	0.235681797
MGC70870	-0.032412671	6.091613596	0.235728109
LOC651314	0.027493807	6.169737415	0.235781114
LTBP1	0.022989102	6.171729861	0.235819343
RPS6	-0.051183551	12.83327295	0.236012604
ODF3L1	0.032385784	6.179285375	0.236016173
TMPRSS11A	-0.028830443	6.035301377	0.236032954
OR9A2	0.028613979	6.128688862	0.236092704
GPR112	-0.037123975	6.060170222	0.236099388
EXOC5	-0.031522371	6.464470653	0.236136833
LOC648041	0.03443389	6.082220633	0.236164162
AQP2	0.032826594	6.144540342	0.236167079
FAM126B	0.036176535	6.819958369	0.236232417
LOC645899	-0.075529439	13.14004572	0.236240854
TMEM89	0.036916977	6.201809872	0.236262211

SERPINB5	-0.033461711	6.11897651	0.236301847
LOC649910	-0.029533339	6.147320637	0.23634301
CALM2	-0.054824516	12.57005784	0.236432829
HSD11B1	-0.052004472	6.64502885	0.236485647
C8ORF41	0.092365678	7.420029004	0.23654408
UNC45A	-0.041910794	7.580391012	0.236559363
LOC389671	-0.029007796	6.09654609	0.236602234
PSMG3	-0.041004811	6.994515736	0.236614327
COG8	-0.0320576	6.407999428	0.236679416
LOC647328	-0.033382269	6.408015205	0.236714988
EIF3E	0.054700812	12.89856984	0.236753517
DNAJC27	0.060544743	8.363006863	0.236766823
MAK16	-0.080593137	8.115058213	0.236929821
SMG1	-0.05345567	7.951123243	0.236948423
KRTAP10-8	-0.031737806	6.070663475	0.237069822
HBG1	-0.067100794	6.242704536	0.237093347
LOC648943	0.02643358	6.059327765	0.23722734
C4ORF17	-0.028603212	5.977044691	0.237302306
PTPRG	-0.072478695	6.698280123	0.237369325
KIAA0317	0.028588144	6.024934411	0.237539565
LOC440706	0.038169818	5.997056494	0.237580629
CCL20	-0.055464671	6.106477091	0.237581529
ANKRD13C	-0.041417625	6.985558789	0.23779541
PARG	0.025182273	6.271990995	0.237817471
LOC642987	0.028805567	6.090696742	0.237870171
XAGE1B	0.020647522	6.064392802	0.237890884
HS3ST3B1	0.02935955	6.204251437	0.237900397
LOC388900	-0.029138496	5.917330713	0.237928524
SELO	-0.043031641	8.079986323	0.23794924
TMEM161B	0.055683855	6.113768605	0.238143313
PTPN7	-0.023670894	6.188122867	0.238167274
C1ORF141	0.020736996	6.014833678	0.2382148
LOC441870	0.029267326	5.999238121	0.238250293
PIGQ	-0.064261777	7.847718207	0.23825594
LOC646279	0.026645919	6.062329301	0.238272947
LOC113386	0.082351974	7.755541822	0.238307321
UBXD6	-0.041605556	6.249971327	0.238321347
C22ORF33	0.049936918	6.194294386	0.238355466
RPL38	-0.051718365	13.63701482	0.238506318
GJA9	0.036379915	6.107288884	0.23851458
CDIPT	0.063682362	6.569308263	0.238578291
CHAF1A	0.033591237	6.278419984	0.238793347
CLDN12	-0.059772589	8.360768498	0.238805023
DNHL1	0.02803968	6.232829743	0.238960686
LOC440577	-0.027046329	6.265578972	0.238967912
BTN3A3	-0.049092693	6.822190245	0.238994922
HIT-40	0.030371586	6.165289591	0.239127274
ANXA13	0.01757216	6.001043077	0.2391792
SOX1	-0.03013525	5.942711432	0.23921118
MGC33407	0.027812638	5.956060323	0.239312891
GPR37L1	-0.031402002	5.960477645	0.23934425

LOC645686	0.028335397	5.991812541	0.239431086
LOC389787	0.106189751	12.73084757	0.239451735
ADAM20	0.034270604	5.966519047	0.239516389
CSTA	-0.027253534	6.022754583	0.239550572
LOC400713	0.051057818	6.830760488	0.239563364
ZBTB41	0.04667833	6.441890123	0.239591497
RAPGEF6	0.043706581	7.162232787	0.239638047
LOC643350	0.029805163	5.977794742	0.239652997
LOC653146	-0.029833944	6.068153313	0.239672764
HDAC11	-0.056292497	6.556557705	0.239782268
LOC389842	0.032067891	6.236485658	0.239800965
LOC651848	-0.028523771	6.099128105	0.239898317
LOC645576	-0.027496071	6.014893894	0.239931472
HIST1H2BA	-0.027010717	5.962314386	0.23995239
UBL4B	0.028567654	6.059231987	0.240158832
LOC390561	-0.030782911	6.027377885	0.2401777
KCNH1	0.02170137	5.995669916	0.240182251
LOC647089	0.036111234	6.113274989	0.240208062
NME6	0.037433746	7.232565604	0.240253512
KRTAP19-2	0.027022023	5.952053155	0.2403943
LOC650599	0.033420228	6.088148348	0.240419937
OCEL1	-0.0684195	8.299893145	0.240523065
LOC402562	0.031425071	5.991690243	0.240590281
LOC652111	0.027515092	6.153131362	0.2406074
ZMAT5	0.034719474	7.346012649	0.240763541
C21ORF86	-0.024718827	6.043537011	0.240801387
DAZ1	0.032632346	6.022627361	0.240856743
GJA8	-0.028757788	6.131501683	0.240887835
CABYR	0.023610725	6.04726909	0.241042959
ZNF556	0.04621916	6.074133898	0.241066993
IL5	-0.022037849	6.089090874	0.241123827
LOC648392	0.028238131	6.100610872	0.241126387
RTEL1	-0.024784754	6.169163018	0.241155566
ZNF223	-0.093837762	8.594910686	0.241162445
KLHDC3	-0.072542045	10.25674973	0.241174712
FLJ30058	-0.050878029	7.344691247	0.241180971
TAF3	0.020027318	6.197911873	0.241186789
DYNC1I1	0.086733299	8.753270164	0.241211528
LOC644652	-0.02642908	6.187075451	0.241221706
IGF2AS	0.040249624	5.943183058	0.241297665
LOC646010	-0.028471626	6.055896276	0.241302485
KIAA1666	-0.021626156	6.308675757	0.241349207
TMPRSS11B	-0.029416567	5.985449698	0.241357451
IL22	0.018598489	6.027951237	0.241386611
LOC653037	0.034886979	6.082308305	0.241424797
GPR6	0.025070172	6.154639669	0.241493659
LOC648590	0.0269625	6.143224786	0.241538664
C12ORF71	-0.028816334	6.134697817	0.241552381
LOC645012	-0.026708439	5.909658005	0.241800256
LOC651102	-0.034962812	5.982245453	0.241926539
LOC440995	0.031927866	6.103837112	0.241943133

LOC650712	-0.030159486	6.000009215	0.241984733
LOC646675	-0.117382962	8.622241535	0.242057159
LOC646198	0.031880356	5.918023121	0.242078483
KIAA1922	0.029124672	6.072438591	0.242135802
KRT73	0.028559705	6.05404299	0.242138577
C1ORF172	0.026990374	6.102524909	0.242162384
FLJ43582	0.027393892	6.081259857	0.242220387
LOC387873	-0.030029664	6.055241746	0.242285956
ATXN8OS	-0.027358456	5.982080928	0.24234747
SLC5A11	-0.027897809	6.037472873	0.24238183
UGT2A1	0.020822083	6.029329175	0.242456442
LOC645363	-0.029225726	5.970476383	0.242485402
LOC649428	-0.033330023	5.925291603	0.242542891
DOK1	-0.044111482	6.075550304	0.242748101
APPBP2	0.039330074	8.046474403	0.242861315
LOC389830	0.028140732	6.057803493	0.243012771
LOC730130	0.030526462	6.128004019	0.243057255
C16ORF5	0.035554765	6.457399362	0.243122758
SLIC1	-0.033325657	6.0089616	0.243139032
METT11D1	0.036831739	7.732187223	0.243259879
PHF19	0.026282203	6.71042683	0.243309243
LOC642629	0.026283263	5.930516948	0.243359487
LOC402483	0.042275733	6.086450541	0.243394922
TFAMP1	-0.02819509	6.024873669	0.243461235
LOC652200	-0.027463891	5.916292327	0.243502515
PRKCG	0.02870379	6.150864785	0.243535659
BDP1	-0.030540906	6.256106441	0.243581721
TGOLN2	-0.071037783	10.07730686	0.243614781
LOC653533	-0.031481818	6.082785096	0.243692799
TNFSF12-TNFSF13	0.03221405	6.428104804	0.243745557
SNRPD1	-0.039455382	6.775167801	0.243840098
LOC389641	-0.025610853	6.068227044	0.243875722
CPSF3	0.048737088	8.933942462	0.243949183
KIR3DX1	-0.030782954	6.071657721	0.244039393
FBXL21	0.033720343	6.219798194	0.244087482
KIAA0514	0.020911103	5.952415168	0.244095248
LOC651430	-0.02997829	6.077212752	0.244102592
IFNA17	0.029312007	5.89182929	0.244123992
PRR3	0.042551731	6.288406629	0.244164302
AGAP1	-0.021349087	6.053535248	0.24422952
KRT86	-0.029212508	6.003886421	0.24423799
PBRM1	0.025464124	6.737488114	0.244291805
FLJ90650	-0.030281961	6.065345317	0.244436532
ACSM5	0.047005349	6.293973762	0.244468156
LOC644468	0.037691681	5.941325219	0.244757549
FAM174B	-0.069734718	7.907566827	0.244866517
CHURC1	0.214676465	9.767410767	0.244939052
LOC643770	-0.026838368	5.991143213	0.245064122
CYLC1	-0.02029372	6.132000133	0.245126291
LOC651728	0.031519734	5.953002072	0.245279753
STCH	-0.048085086	6.503676963	0.245344716

LOC442020	0.031334815	6.033335899	0.245386281
PLCXD2	0.035019499	5.893251076	0.245458474
FAM128A	-0.045659819	6.077047975	0.245481916
DMWD	-0.082203795	7.205122317	0.245492548
ATF7	-0.031247799	6.184267272	0.245582005
SLC2A4RG	0.035097466	7.43280923	0.245624535
APLNR	0.120432951	8.807117497	0.245639831
LOC729602	-0.034593583	6.193187435	0.245659286
MGC33894	0.027084967	5.962468136	0.245735138
AADACL3	0.025174267	6.029658549	0.245763002
CPNE7	-0.015313358	6.023033619	0.245840896
GRIN2C	-0.038951394	6.2878217	0.245869196
WHDC1L2	0.016777784	6.13046345	0.245901165
MGC42105	-0.034876789	6.096328429	0.245973716
RAP2B	0.022648842	6.139759572	0.246005964
SCML4	-0.025667117	5.949988469	0.246247222
LOC124216	-0.028936941	6.165741374	0.246473581
LOC728183	-0.02801233	5.995805619	0.246819675
LOC149224	-0.029807447	6.058155076	0.246882576
CATSPER1	-0.032288076	6.013858872	0.246894621
LIX1	0.031627761	6.168433024	0.247002881
RNF212	0.031740018	6.131243256	0.247008611
GNG3	0.039623641	6.035774825	0.247093469
GMNN	-0.05148903	6.881425351	0.247111927
IL23R	0.020626092	5.973240169	0.247134113
NPAL3	0.058188553	8.369536858	0.247179739
CD28	-0.032024757	6.132559605	0.247282328
TOMM22	0.071050269	9.105884906	0.24730302
ACAA1	0.069824401	9.358002312	0.247359038
CCDC88B	0.032359061	6.39995224	0.247397621
SNW1	0.050255257	7.077527328	0.247415372
LOC652288	-0.028952233	5.913301263	0.247465983
LOC401410	0.031992082	5.988028284	0.247501684
LOC650314	-0.031268624	5.971190737	0.247664025
SLC39A4	-0.023815171	6.19025159	0.247708149
USH1G	0.03503253	6.433733792	0.247722881
LOC644558	0.030749441	6.209704962	0.247769447
C20ORF133	0.028214573	6.134110435	0.247773473
RNF180	0.029951751	6.405025627	0.247815657
KCNA1	0.028475426	5.976944309	0.247938325
PGAM2	-0.107309468	10.79829578	0.247951443
MYEOV2	-0.033516494	6.126718005	0.248037521
MORN5	-0.030568307	5.947092076	0.248085011
LOC653325	-0.029340787	6.248246883	0.248085479
LOC642433	-0.028282461	6.012912323	0.248252246
CLDN2	0.028245504	6.067068709	0.24835342
PRDM9	0.021718236	6.338108784	0.24857266
MTRR	-0.058118429	7.501226463	0.248751818
SLC2A5	-0.111343182	7.262612589	0.248895186
TRIT1	-0.06653424	8.418300526	0.248936894
PRB4	-0.029799019	6.082253633	0.248942331

LOC653962	0.05014666	5.933351707	0.248957353
LOC651272	0.027728463	6.069945283	0.249010964
LOC652127	0.030326319	5.941715324	0.249088683
RAET1E	0.022642665	6.146368593	0.249171632
USH2A	0.014798157	6.127975276	0.249214608
VAX1	0.030379894	6.006550314	0.249245293
LOC653280	-0.027798613	6.053030311	0.24925184
SSTR4	-0.027769548	6.031644477	0.249267161
LOC345630	-0.031437908	6.035186314	0.249532755
GTPBP1	-0.029309566	6.337698355	0.249600319
VCX3A	0.020684527	6.160216703	0.249747255
DUXA	-0.033656874	5.950470964	0.249914746
LOC441251	0.036438114	6.230620499	0.249922126
LOC646491	-0.032191473	6.458007807	0.249946082
C19ORF66	-0.052071935	7.138885522	0.24996088
TFAP2C	-0.022929302	6.046807411	0.25008932
SNAPC1	-0.053157858	7.580155786	0.25009256
HPR	0.157756242	8.503642292	0.250226553
LOC652850	0.02980879	6.107770076	0.250327966
MIA2	-0.022113519	5.949622329	0.250435558
LOC645992	0.02699449	5.934662682	0.250458249
LOC728621	0.026615739	5.945130093	0.25056915
XKRX	0.029893046	6.017982543	0.250690908
RAD1	-0.039221152	6.511284342	0.250763548
ADAM30	0.028734044	6.026291795	0.250778749
C16ORF3	-0.033017723	6.160972871	0.250857852
LOC649317	0.023657471	5.972175531	0.250885504
COL24A1	0.046616406	6.400785471	0.251003476
PTPRS	-0.027339549	6.244910626	0.251015532
LOC649956	0.027272773	6.145067962	0.251052771
TMEM202	-0.02113591	6.142447418	0.251149796
DHDDS	0.040845226	7.555146953	0.251199079
LOC644399	0.049537905	6.153159171	0.251298973
LOC645869	0.03090405	6.00415166	0.251346605
HOXC13	0.036988312	6.157984783	0.251347707
MGC27121	-0.031010777	6.106942173	0.251393475
ANXA10	0.027744129	5.977629413	0.251411385
CD6	0.036196998	6.266162749	0.251488432
LOC388720	-0.039547176	14.30609134	0.251504548
LOC116412	-0.021755908	6.112100908	0.251610373
LOC642730	0.037950687	6.002587392	0.251611749
HPCAL4	-0.060624985	6.067375757	0.251675529
PCDHB10	0.02363858	6.054645399	0.251750014
PFN3	0.044256742	6.034656557	0.251756211
CYP3A5	0.02950029	6.038374653	0.251777398
NPY2R	0.026850541	6.093803576	0.251802332
LOC345643	0.030128607	6.136994284	0.25201823
LOC441771	0.028304095	6.031297702	0.252050895
RAB3D	0.028619685	6.127289241	0.252099333
LOC641805	0.028179888	6.0184017	0.252140092
LOC642380	-0.023311166	6.200579412	0.252261452

FAM75A2	0.030521197	5.949902443	0.252287644
MSI2	-0.030015236	7.20573458	0.252357443
C8ORFK32	0.025836795	6.059598734	0.252488212
LOC649948	0.027472352	6.059510009	0.252563883
HOXD1	0.033657626	6.025707327	0.252705383
LOC648705	-0.036555551	6.147100858	0.25285401
GRIA4	-0.020547807	5.97033352	0.252875356
LOC652513	0.03511052	6.043213555	0.25288889
TMEM132E	-0.03068487	6.025797704	0.25298452
AK7	-0.034850375	6.023721323	0.253065275
OCLN	-0.016153643	5.995464446	0.253095582
POLR1A	-0.030984677	6.158347343	0.253099864
C9ORF31	0.028387399	5.97655735	0.253149417
TIMM8B	0.073700919	8.322938875	0.253203375
ZMYM5	-0.02981349	6.504818399	0.253287053
RBM11	0.023621293	6.216577702	0.253349956
XGPY2	-0.022492885	6.036644768	0.253419101
RNF138	-0.051020539	6.5562968	0.253507627
LOC642505	-0.029719719	6.123296615	0.25354062
CUZD1	0.026755095	6.167208876	0.253545301
GRAMD2	-0.033010483	5.989455978	0.253602681
ANKRD33	-0.072506017	9.393710437	0.253745423
LOC650261	0.029571047	6.075275733	0.253770473
RTP3	0.038800891	5.895252821	0.253775048
KRTAP4-3	0.026772442	5.999137265	0.253861849
LOC389901	0.044591815	6.644855066	0.253882233
LOC642961	0.028901269	5.970363993	0.2539471
CLU	0.018355028	5.983766402	0.253956833
LOC646997	0.027731116	5.986112439	0.253965422
ERVK6	0.028798448	6.039047004	0.254037778
HMCN1	0.037925249	6.177563664	0.254074224
FAM38B	0.036473631	6.28065668	0.254126938
LOC283755	-0.017985169	6.022191682	0.254190437
C8ORF15	0.026845771	6.181026467	0.254256677
KDR	0.035188434	6.480697306	0.254286189
UGT3A2	-0.027610049	5.957077535	0.254395353
LOC650311	-0.034429746	5.96697672	0.254443205
NDUFAB1	-0.080202271	13.1004843	0.254518488
LOC643953	0.027064333	5.986600918	0.25457508
SLC45A1	-0.022009546	6.253435914	0.254663592
KCNJ10	0.033805271	6.473665169	0.254720943
RAD51AP2	0.031215611	6.114204825	0.254736069
C9ORF153	0.02835046	5.977747777	0.254763377
GADD45GIP1	-0.040625027	6.496890162	0.254777823
REM1	-0.051944691	6.938299392	0.254863606
KERA	0.02028018	6.126942318	0.254864451
LOC652369	-0.033231935	5.944401516	0.2548855
LOC644477	0.041429665	6.008777499	0.254912463
TTC39B	-0.020092762	6.054698689	0.254951303
LOC652819	-0.029044667	6.038653512	0.254968155
LOC646334	-0.032373184	6.160409934	0.254990008

INE1	-0.030300844	5.967280737	0.255007484
LOC440352	0.028991554	6.316625541	0.255041805
LOC651125	-0.032861892	6.061095772	0.255074519
LOC728095	0.025297158	6.063222223	0.255082662
FUZ	-0.044406336	6.765803295	0.255093856
LOC642583	0.030457907	6.032942264	0.255154225
FLJ35848	-0.020864553	6.027625607	0.255171422
RARRES2	-0.089263136	7.521072667	0.255193673
LOC653324	0.056195838	6.393404169	0.255327109
LOC392197	-0.029938136	6.021463426	0.255336891
LOC649091	0.026839036	6.082663431	0.255354944
SSTR2	-0.083653056	8.052701382	0.255372969
C4ORF21	0.026467401	5.975617195	0.255406136
RKHD1	-0.029159801	5.929452415	0.255503862
TARS	0.06995002	9.022196438	0.255507521
LOC650543	-0.02567671	6.09061098	0.255536022
MGC5139	0.046308303	6.194313374	0.255570932
TRIM72	0.041852131	6.032185279	0.255665716
LOC652594	0.032680381	6.033158916	0.255712425
ZNF138	0.023587431	6.372334527	0.255749208
LOC441617	-0.028669363	5.984386417	0.255797323
RNPEPL1	0.047128184	6.465089066	0.255873426
COX8A	0.052386565	13.59443439	0.255923132
LOC730194	-0.029671476	5.976223392	0.255960295
TNNI1	0.026675716	6.055966413	0.255967114
MAGI1	0.026049809	6.325306243	0.255971055
LOC728229	0.029805576	6.24961477	0.256075938
UNC119	0.054632055	6.557873389	0.2560813
RPL4	-0.062886327	11.83112507	0.256116765
LOC642453	-0.028374606	6.047631668	0.256153686
LOC285444	-0.027313185	6.148464175	0.256154569
LOC728654	0.02737387	6.174425505	0.256208318
APOBEC3A	-0.027580999	5.954327329	0.256297283
RPL35A	0.066570717	13.1843951	0.256299195
STATH	0.022166467	6.108990331	0.256334664
MPP5	0.050316721	7.680437811	0.25635214
LOC642591	-0.028832298	6.02421246	0.256426658
PPARBP	-0.048650366	7.178260296	0.256551345
RPS6KL1	0.034061833	6.676388437	0.256583681
LOC653135	0.019563138	6.119253459	0.256616411
C1QBP	-0.047734264	10.62162466	0.256635763
ZNF586	0.045194658	7.429870587	0.256715086
KENAE	0.033120955	6.183233752	0.256730798
FLJ45974	-0.027048083	6.07569436	0.256822875
LY6G6D	0.034769212	6.109915079	0.25682994
COL18A1	-0.067602318	8.139626142	0.25683969
C3ORF14	0.055603077	7.185284994	0.256949996
LOC644767	-0.028194055	5.934274961	0.257176099
CEMP1	0.044396271	6.090600043	0.257201527
LOC648432	-0.025912396	6.120484616	0.257205423
NFAM1	0.052444491	6.091729955	0.257310039

ACHE	0.017407636	6.137278138	0.257355077
RAD9A	0.04198608	6.709892964	0.257358618
DRD1	-0.038694247	5.955876685	0.25748237
ARHGEF10L	0.034295068	6.335973394	0.257506373
LOC342931	-0.026557514	5.98246856	0.257609123
OR1S2	-0.018998136	6.065037434	0.25778137
LOC642168	-0.029191413	6.098857769	0.257790876
SRD5A3	-0.033463398	6.332682311	0.257872759
MGC46336	0.028361728	6.082941587	0.257943878
LOC645836	-0.026793054	6.08398005	0.257957501
LOC647748	0.025706396	6.157122882	0.258046094
LOC649128	-0.023725056	6.033417337	0.258052451
MFI2	-0.019667867	6.287938612	0.258109281
LYZL2	-0.029743705	6.048836344	0.258133521
ZAN	0.019772073	6.072629573	0.258260966
LOC440550	-0.025878878	6.019115731	0.25839847
LOC441773	0.029586868	6.100692491	0.25850441
TMOD3	-0.028710409	6.163127701	0.258530791
SLC10A6	-0.029605935	5.901249209	0.258562519
GFI1	-0.029203628	6.036723803	0.258574624
IFNW1	-0.021924588	6.170139649	0.258600067
IQSEC1	-0.053343822	8.650992598	0.258609474
SPATA3	0.021982025	6.104779856	0.25865238
LOC642278	-0.02867602	6.154088755	0.258658092
LOC648253	0.025551906	6.069948835	0.258766272
ZNF469	-0.023959874	6.04314659	0.2590458
WDR42B	-0.026281719	5.887341457	0.259052881
LOC645153	0.031327008	6.231033088	0.259091232
RLF	0.058525599	6.910981591	0.259128211
ZSCAN1	0.056422838	6.526226222	0.259142535
MARVELD3	0.018546575	6.058502483	0.25915812
LOC642608	-0.027785618	6.101344729	0.25918939
MBL2	-0.031135093	5.960693502	0.259192484
LOC649063	-0.02450256	6.027233184	0.2592714
RNF190	-0.026686549	6.01496015	0.259304189
LOC285407	-0.026686005	6.261451573	0.259408431
LOC649571	0.027388126	6.282433799	0.259449796
LOC649800	-0.027482835	5.91758142	0.25948272
RPL19	-0.046591981	12.99925502	0.25951705
PSRC1	-0.02016581	6.215674565	0.259530131
FIGLA	-0.028810794	6.102388462	0.259632803
FAM7A3	0.01789125	6.034306273	0.259660125
LOC644591	0.019434695	6.172049869	0.259672333
LOC441476	0.032843734	5.99847624	0.259716303
C21ORF29	-0.027738323	6.006763653	0.259781635
LOC647413	-0.029089152	5.951564083	0.259785915
ASAM	-0.043231588	6.408609774	0.259845538
PRMT3	-0.087495858	8.77453135	0.259956319
XAF1	0.07846363	7.918871976	0.260000234
ADORA2A	0.055592375	6.899434949	0.260037211
LOC653696	0.030400548	6.258583553	0.260047686

LOC647058	-0.026850499	5.966001139	0.260053969
VCAM1	0.080285945	7.090334712	0.260137199
TCERG1	0.026832655	7.397466905	0.26014704
LOC642981	-0.032009837	6.296124864	0.260199951
ZFYVE27	0.03623565	6.312486968	0.260227779
LOC646399	-0.03354265	6.170557816	0.260263721
DEFB134	-0.031857926	5.979543495	0.260281676
LOC285697	-0.022807421	6.115404564	0.260359876
LOC648257	-0.026540285	5.913557875	0.26041595
SFXN3	-0.038818732	6.266558365	0.260423926
THEM2	0.072613231	9.807610415	0.260523461
BTNL8	-0.021511479	5.888070578	0.260528277
OR13J1	-0.027084296	6.012712448	0.260553005
LOC440030	0.029779285	6.133098376	0.260560006
COL4A1	-0.118379231	10.9102459	0.260677383
PI16	0.15673936	9.115933402	0.26070566
HOOK3	-0.029967638	6.522537687	0.260836708
MTSS1	-0.071299102	8.371990936	0.260849541
NRF1	-0.016764482	6.072350511	0.260867401
FLJ44653	0.027979304	6.098737262	0.261023412
ACADS	-0.064660682	8.511004473	0.261047381
LOC646439	0.031368145	6.099711922	0.261060935
LOC730302	0.028305459	6.122277965	0.261112459
PLEKHG4	0.032723249	6.158220072	0.261230985
MAN2A2	-0.059292277	7.299887332	0.261237896
OSBPL10	0.051779523	6.93183873	0.261460302
DUX4	-0.024910715	5.969551282	0.261461619
FLJ39739	-0.013789016	6.036866474	0.26150756
POGZ	-0.025137558	6.51808496	0.261621085
CYP27B1	-0.031297932	6.091795739	0.261670621
LOC643700	0.028249193	6.006010335	0.261746575
SLC9A3R2	-0.077625221	6.833076415	0.26188143
SLC1A6	0.033516032	5.929203387	0.261887528
LOC642428	0.027935559	6.065328768	0.261955658
LOC653852	-0.027919022	5.936336053	0.261988401
TMEM97	0.041373212	7.623877192	0.262031119
LOC643943	-0.026697592	5.93928219	0.262120734
LOC647374	-0.026036572	6.0393511	0.262144363
CPSF6	0.028654456	6.414207344	0.262210504
LOC647797	0.029773812	6.067049948	0.262217375
CIB3	-0.02529664	6.166360002	0.262217964
MYH7	-0.099423667	14.73954364	0.262221444
LOC651319	-0.030459422	5.936157729	0.262308073
LOC643772	-0.030233887	6.282002154	0.262358934
CDH11	-0.107732261	7.864259725	0.262386673
B3GN-T6	-0.053788954	5.948584519	0.26254643
ATN1	-0.035791119	6.91956435	0.262632795
DYX1C1	0.016843608	6.005566929	0.26265837
LOC643132	-0.023633436	6.136032351	0.262669066
CLDN11	0.085834294	7.002644476	0.262697181
LOC650214	0.026268117	5.944374665	0.262698964

SLC47A2	0.035183014	5.983795456	0.262725753
LOC649009	-0.021635628	6.266804327	0.262736982
LOC389816	0.017353654	6.011987488	0.262741387
CST7	-0.032690795	6.458773595	0.262757993
LOC652282	0.025204459	6.098516037	0.262794885
DKKL1	0.029878608	6.344426779	0.262846186
GPR19	-0.021816903	6.049813821	0.262862424
LOC643930	-0.029001232	5.884216512	0.26288418
OR4C11	0.026181014	6.090499632	0.262920196
LOC283440	0.028308754	6.103108276	0.26295831
CTRB1	0.036736388	5.907015309	0.262971267
LOC391656	-0.071583037	11.93239973	0.263004013
MAZ	-0.019728005	6.258562098	0.26300459
FBXL4	-0.029287013	6.0183962	0.263021568
KCNJ13	0.027632044	6.083482864	0.263038024
C18ORF25	0.054236391	9.28408253	0.263039807
LOC649991	0.029673675	6.132340134	0.263165287
LOC440848	0.028142687	6.003073667	0.263252768
STAU2	0.039044032	7.341737943	0.263260026
ZNF322B	0.028903078	6.102619783	0.263373444
UGT2B17	-0.064420082	6.31300429	0.26339051
TNF	0.038566878	6.23888297	0.263392821
LOC643853	-0.03169273	6.122942313	0.263396839
LOC127099	0.029510766	6.037724875	0.263436072
LOC441811	-0.033068653	5.877045496	0.263604329
LOC441098	0.03111565	5.949959372	0.263652214
C1ORF194	-0.026187508	5.992414801	0.263713469
FLJ33996	0.02797218	6.274185258	0.263795386
RHOQ	0.055965566	12.31195547	0.263948343
PIP5K2B	-0.079957922	10.1005639	0.26398012
C2ORF60	-0.032354634	6.010187249	0.264125938
C17ORF76	0.026817402	6.14495859	0.264136774
GPER	0.061072482	7.394168666	0.264177406
RUNDC3A	0.035139101	5.984686341	0.264352951
C16ORF85	-0.027591234	6.004708826	0.264399284
TMIGD2	0.03034691	5.845526374	0.264445426
ASCC1	-0.053855893	9.909768141	0.26448055
DSG2	0.059005538	7.207133463	0.264605594
ARNTL2	-0.032333035	6.008613995	0.264637123
FAM108B1	-0.039727037	6.803996713	0.264682147
ROBO3	-0.037207805	6.674194082	0.264804139
LOC653387	-0.021092276	6.034143626	0.264856783
DPYS	0.029546904	5.965715149	0.264925775
FLJ41423	-0.059763316	8.735164473	0.264937247
ACPT	0.040819214	6.805462876	0.264948164
SAMD12	-0.033266616	5.95076919	0.265044415
AP1S1	0.047553263	6.960352937	0.26511215
CDH13	0.089387237	9.489567269	0.265186797
LOC652100	0.026630179	5.982446084	0.265205355
LOC647213	0.022998462	6.000743869	0.265211868
LOC646067	-0.03011918	6.225073058	0.265214428

LOC648170	-0.026524275	5.997931463	0.265236038
UGT2A3	0.019576922	5.973920184	0.265252615
CBX5	0.085881686	8.487211437	0.265257079
NEUROD2	-0.021026687	6.040992256	0.26527201
PTPN20	-0.023410247	6.062839222	0.265347844
CLUAP1	-0.071967597	12.39701655	0.265378956
NT5E	-0.045611559	7.083579816	0.265390071
LOC646512	0.026451616	6.048654153	0.265426243
AMICA1	-0.04613047	6.687154342	0.265437133
BRCA2	-0.033381304	6.062230278	0.265556564
LOC646299	-0.029052329	6.05390827	0.265565842
B3GALT6	-0.054230729	7.648045261	0.265602985
APOA1BP	-0.051156123	10.09572868	0.265624369
LOC51149	0.029518482	5.980692539	0.265670806
SRPX2	-0.100646399	7.530150943	0.265723141
DKFZP781N1041	0.026138308	6.110549429	0.265755613
LOC651530	-0.02713154	6.020355632	0.265768691
TTC29	-0.023570058	6.051231481	0.26580776
DGKK	-0.030453377	5.986990376	0.265866398
ARVCF	0.043213656	6.30843091	0.266089219
SLC38A5	-0.046620313	6.269800907	0.266121161
LOC653316	0.049171447	6.172895506	0.266149768
TTTY6B	-0.019422014	6.011660481	0.266152971
LOC339902	-0.02771511	5.978999557	0.266156719
INPP5A	0.046472167	9.052015837	0.266227107
LOC653577	0.025526196	6.126066729	0.266238946
FLJ32569	0.020085948	6.096987713	0.266245448
TMEM92	0.025682595	6.036042293	0.266311622
TMEM121	-0.026905009	6.011122461	0.266373305
LOC151234	-0.029840597	5.931569225	0.266450618
LOC440829	0.037889816	6.269447587	0.26649106
AR	0.054240766	6.737246872	0.266508414
MAST1	0.027748819	5.933057753	0.266608728
C20ORF134	-0.025573149	6.010420623	0.266695
LOC653319	-0.020300476	6.154406465	0.266729614
LOC643599	-0.02597741	5.917737944	0.266732213
CSAG3A	-0.021256801	6.101089383	0.266733743
MAPK8IP3	-0.0483946	7.403782739	0.266792646
LOC651617	0.025807903	5.976008093	0.266843184
LOC650568	-0.032579537	5.896194862	0.266886023
LOC730118	0.0279174	6.152695602	0.266932221
LOC729826	0.020336658	6.143574017	0.266936146
SLC30A10	0.028348704	6.163849943	0.266974058
ANO8	-0.042300531	6.702692883	0.267057414
SNORD22	0.030585343	6.422808744	0.267189547
ABHD13	0.021022631	6.273211503	0.267286418
C12ORF40	-0.017400629	5.977193049	0.267303389
CDYL	-0.028629826	6.54094262	0.267313985
FLJ45994	0.025465494	6.087170071	0.267425549
LOC595101	-0.029817274	6.265312929	0.267565081
PPP2R2D	-0.036538443	8.0639689	0.267577715

CTTNBP2NL	-0.033536407	6.5117916	0.267743594
LOC653434	0.027047637	6.06985202	0.267812371
LOC200810	0.032839443	6.293539647	0.267814834
TMPRSS7	0.031191859	6.272736793	0.267825105
IMPA1	0.07384902	9.062486149	0.267880932
OR1E1	-0.045345228	6.558178027	0.267886601
LOC651554	-0.031770674	6.033369339	0.268067888
PITPNB	-0.052619067	7.793106214	0.268132679
ENOX1	-0.052409107	5.960653202	0.26818447
ABL2	-0.019761888	6.198132272	0.268186655
CDKAL1	-0.037469905	6.574187192	0.26819729
LOC649192	-0.030252021	6.340870616	0.268223019
KLC4	0.022809627	6.082798823	0.268238266
KIAA1109	0.030572815	6.199230684	0.268379102
C8ORF71	-0.026568171	5.995422155	0.268380595
PGC	-0.029945596	6.004037429	0.268394012
MEF2A	0.104427074	7.850557056	0.268396435
FLJ21839	-0.030909666	6.095344461	0.26846214
LOC644668	-0.033563433	5.949799802	0.268464177
LOC650930	-0.035116181	6.06963513	0.26853537
LHPP	0.058240742	7.461721451	0.268634868
ACOT2	-0.10152194	8.167564282	0.268650935
FLJ30851	-0.020566138	6.150384632	0.268686537
TBC1D21	0.038994934	6.0329113	0.268706257
GSTA2	0.024441577	5.979665544	0.268745472
ZNF100	-0.021240089	6.025773885	0.268763789
LOC653820	-0.019996769	6.242040612	0.268856144
PAK4	0.027365687	6.839446507	0.268861708
CSNK2B	-0.050939544	10.27231029	0.268895859
MECP2	0.036611088	6.627129046	0.268947527
SYNGR4	-0.029222186	6.062847639	0.268998423
LOC90120	-0.038997184	6.570961086	0.269001642
TMEM19	0.067295645	6.471577187	0.26904923
ADK	0.044125423	7.591956702	0.269083567
LOC728556	0.058298693	8.749447339	0.269165783
CYP4A11	0.027711962	6.105770146	0.269211076
LOC644734	-0.027530611	6.101079921	0.269213965
LOC731670	-0.029360685	6.100774028	0.269256114
LOC646903	0.032109725	5.908192569	0.269315994
DPP10	-0.020852495	6.011133438	0.269513389
WNT9B	-0.040078944	5.960358187	0.269517183
ASAH3L	0.023853062	6.115693482	0.269519911
LOC652469	0.028828809	6.040358034	0.269549696
LOC645276	0.048924849	6.010787864	0.269565788
LOC647309	0.019185514	6.070270233	0.269709281
LOC652761	0.033801051	6.079145601	0.269731779
UBFD1	-0.050800591	7.666205876	0.269748486
ODZ1	-0.029624639	5.934222335	0.269844659
LOC285588	0.027057882	6.101158744	0.269919559
LOC645427	-0.028917479	6.020308406	0.270052452
EHBP1	-0.042039533	8.441173843	0.270095638

KIAA1843	-0.02945993	6.077162559	0.270134452
C12ORF29	-0.057690221	7.774346967	0.270180549
MYOM3	0.087478085	11.24610606	0.270201249
LOC727805	0.032141261	5.959608812	0.270277175
LOC642978	-0.023963004	6.077145022	0.270524978
ATP6V1G3	-0.021373779	6.058518093	0.270544396
LOC644135	-0.031482231	5.886139996	0.270701335
LOC642503	0.029808991	6.211563248	0.270804787
LOC645481	-0.029538049	6.052291191	0.270830807
C2ORF16	0.030515618	6.100317643	0.270841892
LOC643582	0.026751114	5.99302184	0.27090393
FHL2	0.11153978	12.92268411	0.270932744
ODF1	0.026865208	6.018227478	0.270937029
FLJ20972	-0.026437467	6.042028453	0.27093754
CEBPE	0.028603765	6.197929523	0.270951442
LOC653423	0.027174558	6.069457505	0.271014099
KRT27	0.027619349	6.014762986	0.271045516
ADAMDEC1	-0.022012487	6.052274301	0.271162171
LOC653094	-0.029831974	5.999442128	0.271266304
LOC340598	0.025142315	6.14323012	0.271419138
HLX	0.053157999	6.89758235	0.271490983
OR7D4	0.027502135	6.06692787	0.271518829
LOC649848	0.028062438	6.04702821	0.271684048
LOC649908	-0.030628173	5.992063632	0.271824617
ATP5D	-0.055851662	11.43932349	0.271849703
C2ORF50	0.038740298	6.234155068	0.271906073
ILKAP	0.037802305	7.331943194	0.271906761
SNORA62	-0.034195361	6.502604681	0.272244742
GZMH	0.062816647	6.908836277	0.272356772
PAGE5	-0.018344374	6.037562264	0.272433266
LHX9	0.022221476	5.96304329	0.272484457
LOC643878	-0.025919886	5.984486083	0.272502001
TAF1L	0.039909025	6.916413824	0.272517683
GSTCD	0.050986357	7.822770921	0.272583388
FAM134A	-0.034064904	7.154116828	0.272625359
LINGO2	-0.030888133	6.003732023	0.272635149
TRPM5	-0.027107782	5.928110662	0.272635967
LOC650717	0.031681403	6.345171626	0.272648329
DTX1	-0.038981552	6.204925071	0.272787841
CMA1	-0.025403909	6.095250763	0.272809133
GALR1	-0.022824488	6.066004396	0.272905946
LOC440567	0.078409505	13.48012762	0.273020113
TBC1D3C	-0.025318616	6.093247489	0.273048698
TTTY3	0.026073399	6.003311319	0.273075484
NCBP1	-0.036876858	7.744703945	0.273102227
LOC649945	0.03507083	5.997459858	0.27311655
LOC642411	-0.025576906	6.199978611	0.273130445
C14ORF128	0.03560701	6.429623036	0.273132091
TDRD10	0.034620662	6.401622831	0.273398176
LIMA1	0.062214881	7.477519879	0.273482553
RINT1	0.03371061	7.108185797	0.273494048

CSPP1	0.036395916	7.164911635	0.273519833
LOC646917	-0.026764569	6.086103695	0.273526476
LOC646162	-0.028731772	5.932618284	0.273584272
KIR2DS4	0.047398719	5.987362611	0.273593489
LOC643559	-0.028599346	6.168843354	0.273622378
TMED7	0.05781315	9.402572646	0.273626486
LOC652856	-0.029402637	6.065740947	0.27378142
ZNF467	-0.06204191	7.532648447	0.273876909
EXOSC6	-0.062686068	8.511870207	0.273887727
RAD18	-0.018337196	6.125066916	0.273895055
SLN	-0.190295772	8.0503999	0.274116918
LOC652647	0.057778083	6.73616906	0.27414004
LOC642759	0.038994572	5.91955344	0.274162723
RAB6B	0.073938355	8.993967412	0.27434265
ACBD4	0.051348305	6.181537867	0.274486001
LMBRD1	0.060089158	9.568955016	0.274553494
LOC643697	-0.022914428	6.08139339	0.274588018
IAPP	0.036167316	6.299242346	0.274593693
TRIM16	-0.017850342	6.055363888	0.274633407
LOC440895	-0.019531938	6.21534035	0.274726935
LOC220429	0.029447645	6.148418118	0.274753927
PCDHA5	0.033912758	6.79894652	0.274818965
MCM3	0.038760344	9.236528395	0.274858551
LOC644157	-0.029920534	5.983801256	0.274880679
ZYG11B	0.059346345	10.61145863	0.274961317
OR4S2	-0.033670549	6.008951078	0.274964049
PNMA6A	0.032618724	6.177696234	0.274998801
DDHD1	0.0218074	6.060076586	0.275099827
PAH	-0.019094118	6.001592831	0.27523788
LOC645966	-0.026598423	6.223113453	0.2753468
FLJ33708	0.026978838	5.933547174	0.275461895
LOC645487	-0.026860319	6.120680986	0.275604551
LOC645676	0.018283843	6.163599434	0.275679237
SLC38A9	-0.036162613	6.789633528	0.275707515
LOC728081	-0.035888609	6.143748481	0.275746825
LOC645441	0.01848408	6.027940611	0.27576711
ZNF326	-0.023420045	6.187193017	0.27604165
HERPUD1	-0.077570755	9.623160158	0.27612253
LOC644953	0.027679843	5.927089094	0.276149638
KBTBD6	0.03882572	6.280296799	0.276198363
FLJ12355	0.029954249	6.171788124	0.276220922
EPHB2	-0.025161578	6.077525512	0.276242449
FOXD4L1	-0.021881887	6.077340902	0.276301207
LOC644828	-0.026013515	6.169223226	0.276313252
LOC641994	0.02730755	6.110383907	0.276425188
TYK2	0.042309558	9.58876596	0.276456873
MEFV	-0.02216915	6.093537231	0.276464158
FLJ41841	0.033035069	5.928760505	0.276596295
PCDHB11	0.020793357	5.998870865	0.276612265
LOC729486	0.019622303	6.146810933	0.276669403
RAX	0.031564422	5.974494851	0.276759544

DEFB1	-0.039290814	6.240662913	0.27686755
LOC653673	0.037254276	6.096423024	0.276893978
HYAL3	-0.032607012	6.432251803	0.276919996
RAB7L1	0.052627694	7.58617602	0.276930138
TTC4	0.056239511	9.95118359	0.276980441
LOC649937	-0.024720431	5.995748375	0.277227263
SPEF1	-0.04456789	6.056711412	0.277243142
LOC644679	0.027051822	6.247156606	0.277381882
GPR149	-0.024359728	6.158397318	0.277473536
PKP1	0.022034508	6.11459154	0.27758697
LOC653899	-0.025993213	6.030539549	0.277718233
POTE2	-0.020088842	5.998248983	0.277744592
LOC652828	0.023957499	6.087135803	0.277766167
SMPD4	0.032908857	6.6269962	0.277790986
LOC652765	-0.028100902	6.023855441	0.277839548
LOC646103	-0.016786725	6.286589012	0.277851653
C1ORF180	0.028649765	5.929602917	0.27786715
LOC390595	-0.032738573	5.914236391	0.277884596
FUS	-0.034732391	7.093303202	0.277895078
LOC643950	-0.025782381	6.115905571	0.277909699
SPRYD5	0.027538227	6.494011616	0.27795157
SYAP1	-0.050708359	7.619521064	0.277977021
B3GALT5	0.016141089	6.016980058	0.277993642
FAM12B	-0.032112227	6.147673905	0.278023984
SCTR	0.037669061	6.310821313	0.278057686
ZNF512B	0.045642022	7.140274168	0.278188453
LOC649159	0.023872257	6.147052914	0.278204928
LOC644580	0.040390488	6.030378761	0.278334227
PLEKHA7	0.09068001	8.112168766	0.278360701
SLC30A6	0.023818089	6.226533247	0.278388881
PCDHA13	-0.019853997	6.035051129	0.278398871
SNRPD2	0.047286179	11.69706741	0.278419304
P2RY10	0.026499775	6.107455756	0.278506659
LOC440748	-0.0184188	6.104646843	0.278588427
LOC645494	0.025151924	6.071647222	0.27859888
LOC283157	0.040571341	6.282368867	0.278688642
FBLN2	0.090534943	8.777800227	0.27871985
LOC649935	0.026222043	6.043931434	0.278803975
LOC652903	-0.040339219	6.363814218	0.278909917
MAF1	0.047556457	8.56535811	0.278916067
SIGIRR	-0.041888541	6.634776368	0.278997836
HSPA8	-0.081594555	12.3157978	0.279119119
LOC642350	0.02701492	5.973730657	0.279263538
GLUD2	0.035062053	6.868997969	0.279268279
ERBB2	-0.050818533	8.333455154	0.279320352
LOC644316	-0.027861643	6.357708058	0.279493753
ZNF808	0.027232668	6.079366189	0.279563332
C19ORF21	0.031129332	6.417376818	0.279597318
DKFZP761O2018	0.024902889	6.057040298	0.279609587
LOC648044	0.027805492	6.151744595	0.279720256
OR51F2	-0.018370736	5.944237996	0.279748212

C1ORF64	-0.054037582	6.319190628	0.279928272
SPTBN5	0.036097705	6.320643567	0.279939912
FLJ34969	0.027918754	6.011949823	0.27995153
LOC643143	0.027308396	6.112170115	0.280066004
KIAA0947	0.029125527	6.657211601	0.280119078
TBC1D26	0.022419585	5.997748592	0.280136136
RPS12	-0.068941432	13.15559224	0.280209838
LOC644779	0.031294983	5.980101353	0.280239127
LOC649088	0.026240929	6.103887132	0.280291172
LOC730357	-0.02712032	6.151646471	0.280300728
LOC642452	-0.025045743	6.209270606	0.280330516
TRIM46	0.027751868	6.061746504	0.280335564
LOC649133	0.033199485	6.058833465	0.280371559
LOC654244	0.053154859	6.669183504	0.280387385
KCNC1	0.02786975	5.970661703	0.280407731
ZNF473	0.026541399	6.901746943	0.280514827
OR6C1	0.027572242	5.853720224	0.280529633
OR2T11	0.030538209	6.304689697	0.280534643
LOC647873	0.027591634	6.162561967	0.28058585
ARMC7	0.038330477	6.986603644	0.280722851
GOLGA2L1	0.034503095	6.149624115	0.280764631
DNAH2	0.025668826	6.028845102	0.280867891
BRWD3	-0.028983437	6.099957742	0.281053387
WDFY3	0.022922572	6.145503947	0.281520317
LOC643197	-0.030309252	6.017203338	0.281659297
FAM18B2	-0.034494032	6.618697543	0.281700585
VRK3	-0.031192033	7.514995294	0.281768663
POLR2F	0.042223394	11.30486176	0.281800227
ZNF281	0.052949253	7.539878083	0.281832103
THG1L	0.040853327	6.530485728	0.282114703
BOK	-0.044303324	7.345323539	0.282177213
LOC647888	0.02639581	5.994627395	0.282225989
SIT1	-0.030594149	6.326985476	0.282349301
CER1	0.019079517	6.059441696	0.282413763
NEK11	0.019712568	6.19343348	0.282424282
CDH9	0.025219359	6.305893105	0.2824285
LOC440925	0.027534165	6.145903234	0.282442603
LRRC38	-0.088195278	6.619240721	0.282511789
AVP	-0.019413826	6.288098458	0.282556259
MORF4L2	-0.074848283	10.2947106	0.28258034
FAM164C	0.056255252	7.336589874	0.282625288
LOC653242	0.050220448	7.118890929	0.282628972
ARL5B	0.02688537	6.401090967	0.282662534
LOC652663	0.023571895	6.125056611	0.282763184
WDR16	0.01975246	6.034178582	0.282798103
LOC158830	0.034005074	6.058048254	0.282822862
LOC388275	0.062061267	9.269259003	0.282963469
C20ORF71	0.01615879	6.073428018	0.283088195
FOXA2	0.019174307	5.922649699	0.283225304
LOC646628	0.040176781	5.976947898	0.283407022
NRG1	-0.014063717	5.995521876	0.283426339

POLR2A	0.055022274	9.503839536	0.28346166
LOC390933	0.018594306	6.215015277	0.283467646
LOC646494	0.027052286	6.262441236	0.283644588
WDR21C	0.029773746	6.21513427	0.283682318
LOC643834	-0.021363094	6.027501654	0.283707054
ASB9	0.069272107	6.510991032	0.283730364
LOC650410	0.024415884	6.048166648	0.283794972
ZNF284	-0.019902132	6.063034218	0.283962627
SEC22C	0.038048446	7.972354304	0.284157803
LOC440334	-0.027316028	6.051791771	0.284180725
SDK2	0.034097608	6.379946973	0.28437436
ZNF232	0.046348019	6.748657974	0.284419927
CYP4Z2P	-0.013025358	6.120609066	0.284453893
LSP1	-0.02324568	6.368078976	0.284495938
PCDH7	0.063720179	6.75279261	0.284546628
FLJ16734	0.026116293	6.328394261	0.284727926
SLC7A4	0.029622788	6.256247694	0.28474553
LOC441682	-0.027918529	6.189680203	0.285029002
LOC389904	-0.02797524	6.139638523	0.285121662
ALAS1	0.032836008	7.036987363	0.285167257
PPP1R2	0.056485858	8.624669048	0.285176857
LOC647176	0.029280182	6.317870393	0.28527463
LOC643912	0.042047824	6.08901626	0.285329969
LOC653481	-0.027907784	6.036236928	0.285365026
RAB11FIP1	-0.099545313	7.918915885	0.285379561
LOC649455	-0.026524668	5.975866638	0.285389475
SLFN12L	-0.025726714	5.976470512	0.285486918
CCRL1	0.016433847	6.056381019	0.285554237
LOC644646	-0.022694795	6.081736998	0.285566246
ZNF93	0.030849742	6.960798418	0.285582949
LOC644539	0.023898065	6.048644726	0.285696083
LOC652468	-0.026192255	5.885853639	0.285725649
DFNA5	-0.077331698	8.181644173	0.285784948
FBXO11	0.032929975	8.278413554	0.285839612
LOC402670	-0.027976672	6.139998384	0.285842662
LOC648907	0.024726952	6.641697156	0.28588708
KCNQ5	0.018364749	5.934338068	0.286138941
MAS1L	0.025607767	6.12947566	0.286155457
PPCDC	0.0286978	6.031301095	0.286195579
LOC642671	0.026051882	6.020788359	0.286339817
APOL2	-0.027971855	6.485809064	0.286435801
LOC402693	0.027779821	6.235517259	0.286534899
LOC650553	-0.026325228	5.984132748	0.286572489
LOC647461	0.027112936	5.971620874	0.286584276
ZNF592	-0.032543875	6.241342854	0.286584642
LOC650098	0.02642724	6.109699487	0.286612528
CLEC2A	0.024921762	6.092399719	0.286617288
THSD7B	0.022478443	6.087452063	0.286677406
CHRNA10	-0.042091642	6.05336563	0.286822742
BCL2A1	0.033008783	6.415590552	0.286832862
GBP7	-0.029420848	5.995480391	0.286963815

SH3BP5L	-0.067223088	7.190026009	0.286979511
IGFBP7	-0.083245164	12.17299665	0.287183183
LOC646845	-0.023540605	6.013134545	0.287201945
ANG	-0.057144539	7.747317659	0.28725294
MRPL43	0.054141619	8.48597188	0.287365782
MAGEC2	0.019010376	6.094937397	0.287492214
C13ORF28	0.027478601	6.132183578	0.287604563
LOC439949	-0.029514049	6.102187402	0.287672934
SLC35F3	0.017574637	6.006800217	0.28767536
C15ORF21	0.018120676	6.160035948	0.287885979
FAM53A	-0.040212199	6.238141359	0.287903551
ACBD6	0.035549896	7.827815399	0.287952542
MRPL51	-0.040966054	11.39411316	0.288050343
GJC2	-0.047174423	7.354260088	0.288054624
C1ORF88	0.036544863	6.410911858	0.28813617
LOC727851	0.020620227	6.024627782	0.288146541
LOC645273	-0.025341474	6.068838437	0.288209832
RAB25	-0.023382489	6.157426499	0.288244846
CCDC17	-0.030386134	6.348380499	0.28831946
UNK	0.026508288	5.98023012	0.288320325
OR10AD1	-0.028901112	6.025970539	0.288338591
GPATC4	0.025580474	6.113333439	0.288340583
ATP11A	0.020041621	6.009348031	0.288340791
NTS	-0.019549821	5.983101056	0.288367509
TACC3	-0.033056615	6.332766685	0.28840275
PDE3A	-0.057722657	7.368984889	0.288405582
LOC400986	0.041802651	6.35934032	0.288490131
LOC648167	-0.023805603	5.980329093	0.288695373
LOC389394	-0.024818377	6.005258739	0.288755844
TEX9	0.018474313	6.070669636	0.288763017
KRTAP27-1	0.020506572	6.13492949	0.288803027
LOC646262	0.025660868	6.261469654	0.288803263
LOC652522	-0.023446534	5.973371511	0.288879648
NFATC3	0.018711792	6.72770078	0.288893758
TEX15	0.028094034	5.907227424	0.288911669
HTR7P	0.03614399	6.003447769	0.289067267
LOC647941	0.025711383	6.120738884	0.289211505
MIAT	0.029948336	5.994532039	0.289294871
FLJ16323	0.025525829	6.165669049	0.289333501
THEX1	-0.030713909	5.972836208	0.28946288
COPB2	-0.056552247	9.151947486	0.28957404
FOXD4L3	0.035682101	6.040109499	0.28963824
TM4SF5	0.033299491	5.832723443	0.289687127
TXNIP	0.109437799	11.64753576	0.28974925
OR51E2	0.029763877	6.278021288	0.289816255
GPRC5C	-0.035544917	7.798918105	0.289819698
LOC646197	0.080887439	8.903716739	0.2899039
RNF167	-0.044269128	8.183188958	0.289975009
RXFP3	-0.024286908	6.108506703	0.289999838
AVPR1A	-0.030891078	6.037893405	0.290010049
C14ORF119	0.048253762	6.114201172	0.290012973

LOC647868	-0.024621781	5.946809754	0.290013938
ZNF490	-0.029459718	6.012261771	0.290076555
GAGE8	0.034122498	5.939705744	0.290094053
GLIPR1L1	0.027033473	6.284603628	0.29011151
LOC390211	-0.024611547	6.019240962	0.290198828
FIZ1	-0.029952675	7.077493802	0.290227693
TMEM138	-0.037312412	7.768558729	0.290241047
RBM23	-0.044739907	8.984244401	0.290262836
OR4C6	0.027977102	6.045977806	0.290347672
LOC388476	0.023697028	6.049513657	0.290354565
MSH4	0.027548898	6.149650731	0.290464154
LOC650794	0.017695288	6.028202318	0.290467894
RTTN	0.069575924	8.838644316	0.290520325
LOC389599	0.069334052	8.501654401	0.290613708
SCAND2	0.01616984	6.277922476	0.290652652
LSS	-0.029884313	7.37328074	0.290659411
FLNB	-0.066751602	8.778974788	0.290841175
DRD2	0.021837626	5.984284997	0.290919437
KRT76	0.026386457	6.049380933	0.291018807
SNX7	-0.047256412	7.070501883	0.29105723
ZNF677	-0.027871108	6.075849802	0.291216453
LOC644635	0.029676162	6.177760992	0.291220802
PALMD	0.031821961	6.275641264	0.291247595
WIT-1	-0.035714761	6.307796191	0.291258886
LOC728939	-0.025187521	6.208415814	0.291328758
LOC646748	-0.028242614	6.029073021	0.291387664
LOC651441	-0.030524209	6.140264664	0.291404023
C14ORF48	-0.027068912	6.227530598	0.291404228
LOC732162	0.019265031	6.034113276	0.291428952
QPCTL	-0.025501541	6.121249576	0.291440483
MXD1	-0.030692946	6.905098375	0.291441085
PHF15	-0.051666538	7.860912679	0.291617194
CYP51A1	-0.081735219	7.445577639	0.291673162
ALX3	0.019248665	6.109477647	0.291695169
ZNF117	-0.0224052	5.972972813	0.291791506
FLJ25006	0.029220916	6.49904492	0.29180032
LOC729647	0.02762733	6.217882482	0.291812909
CDC2	-0.022947737	6.193894249	0.291843098
TRIM15	0.01651316	6.000523009	0.291909182
HNF4A	0.016022718	6.075074409	0.291952074
LOC651561	-0.03408638	5.90159101	0.292058115
SCNN1A	-0.025466836	6.066220334	0.292158383
CA7	0.019193148	6.002585133	0.292184448
BTBD6	-0.067243569	9.286790671	0.292371689
UBP1	0.039962718	9.208655378	0.292377267
SLC14A2	-0.025044833	6.206361744	0.292406284
LOC284402	0.024860795	6.025890923	0.292406306
SPRR2A	0.028301703	6.060420743	0.29247857
LOC645708	-0.031067011	6.06043076	0.292503704
SLC5A5	-0.026452684	5.937331862	0.292524331
SNRPD3	0.052940142	7.434311805	0.292566797

SET	0.105526247	9.535711548	0.292580933
SCAF1	-0.032126804	6.414284704	0.292684963
ROM1	0.044442648	7.075607778	0.292793992
C18ORF20	-0.029626641	5.99452393	0.292798221
ATP2C2	-0.03142965	5.938789164	0.292845731
SETD6	-0.0383031	5.831193432	0.292860482
LOC653800	0.031661256	6.376207819	0.293260902
LOC652906	-0.025752416	6.064347074	0.29327798
LSM2	-0.072649616	9.87329744	0.293303783
LOC728219	-0.031548182	6.036737896	0.293304693
RNASE13	-0.028994404	6.026677385	0.293308955
LOC728919	0.024745123	6.176068331	0.293314204
LOC730862	0.025150304	6.04424936	0.293576839
ELA1	-0.03068354	5.928206071	0.293614563
KRT80	-0.04841111	6.197961635	0.293648547
LOC401447	0.019049006	6.070931255	0.293948197
LOC643149	0.024030455	6.052495876	0.293958099
LOC647344	-0.029460535	5.904544166	0.294010959
EEF1A1	0.065857087	13.51060084	0.294169725
ING5	-0.017365909	6.122892678	0.29417972
HSPB9	-0.028286362	5.976096003	0.294261195
RPS23	0.140477814	10.66773325	0.294320389
ISG15	-0.132706686	7.883125001	0.294336519
LOC643326	0.027065536	6.127470184	0.294510756
FSIP2	-0.026373355	6.029026726	0.294515251
LYZL4	-0.024627033	6.003519931	0.294552297
C6ORF58	0.026500181	5.931159402	0.294667671
NDUFS7	0.055602282	12.48062535	0.29475685
PTOV1	-0.044126568	9.934262899	0.294925333
LOC400965	-0.032050818	6.124988607	0.29506722
SLC12A7	0.028684794	6.08502774	0.295069398
JAK3	-0.028354834	5.953321261	0.295102356
LOC647050	0.02706021	6.300295293	0.295173129
LOC728912	0.017408281	6.023934505	0.295187783
GDF7	-0.0259271	6.13697579	0.295240323
LOC652389	0.030539845	6.290392561	0.295267126
LOC642711	-0.027479587	5.986884932	0.295307411
SYTL5	0.037660433	6.216939784	0.295320591
ARHGAP6	-0.013592708	6.012373977	0.295422563
GHSR	-0.020193994	6.058583575	0.295456301
TAS2R14	0.026769135	6.075906845	0.295541726
LOC648981	0.027192557	5.895089976	0.295624593
FBXO22OS	0.024098085	6.118725072	0.295685727
PACRG	0.0547928	6.63413042	0.295722184
LOC727987	-0.053758791	6.252894048	0.2957329
PSENEN	-0.034922828	7.103393102	0.295744612
LOC654123	-0.019266954	6.051924076	0.295747436
DRP2	-0.025005461	5.847455352	0.29577171
ECSIT	-0.052288532	8.227527067	0.29578346
TMEM130	-0.027997022	6.058724882	0.295798019
LOC649540	-0.027272055	6.148552349	0.295870512

SERHL2	0.024023266	6.162667186	0.29596263
LOC649851	0.027935124	6.286101615	0.295977464
PAPD1	0.042857333	8.447520162	0.295984552
LOC650869	-0.026103349	5.953834312	0.296039583
LOC643090	0.027347198	5.93929347	0.296054413
LOC646090	-0.02370739	6.101212253	0.296099948
LOC644921	-0.026283911	5.944721737	0.2962647
KISS1R	-0.028944562	5.999757001	0.29627005
HSD17B7P2	-0.027654087	6.171178849	0.296298112
LOC653657	0.026725302	5.926496995	0.296346112
LOC653272	-0.029461744	5.971566796	0.296352845
LOC121981	0.024313544	5.983127529	0.296411954
LOC644305	-0.023130257	5.92129923	0.296423714
TAAR5	-0.026608043	6.058408054	0.296443689
LOC649305	0.033617231	6.068607771	0.296580979
C2CD3	0.024509565	6.119015678	0.296732232
LOC347292	-0.049727917	8.286633967	0.296782642
LOC647783	-0.02628884	6.019390992	0.2967969
NR2E3	-0.015899049	6.046280413	0.296829267
IFP38	0.082814034	8.014124529	0.29691069
LOC651825	0.023358033	6.015101886	0.296988306
LOC652730	0.027352502	6.160979418	0.296991135
LOC650114	0.025524204	5.971776935	0.297058023
LOC644548	0.01750193	6.134501961	0.2971242
PRKG2	-0.028041769	5.993596732	0.297158438
FLJ25404	-0.025896106	6.122847673	0.297179382
LOC642144	0.02206087	6.100292989	0.297334123
SUV39H2	0.03352888	6.780448729	0.297398626
FAH	-0.062693204	7.564325225	0.297497413
MDGA2	0.024926404	6.078388903	0.297518721
ZNF785	-0.031846264	6.263058095	0.297545859
OR8A1	-0.024946086	6.071913419	0.297587057
GHRH	0.028307452	6.016298573	0.297606836
LOC652781	0.024682574	6.058221899	0.297611757
KRTAP9-5	-0.025630094	5.986666652	0.297844781
LOC389286	-0.077408973	8.289848714	0.298040325
OR10K2	-0.029128142	5.922110382	0.298096272
POLR1D	-0.053207642	10.46152377	0.298155096
PLCXD3	0.082627414	9.979563579	0.298234613
LRRC27	0.028856481	6.028827557	0.298440408
LOC645724	0.021864389	5.973335574	0.298530645
PIK3CB	0.05126452	6.974477399	0.298608452
LOC653425	0.027128939	5.980382999	0.298709776
LOC646665	-0.029296963	6.253434777	0.298787717
ENPP6	0.027519599	6.048035459	0.298860243
RUVBL1	-0.038391805	7.603276466	0.298974871
TBC1D9B	-0.037363678	7.403194748	0.298998458
LHX8	0.027408705	6.000798821	0.299010734
OR8K5	-0.019866429	6.149588364	0.2991336
LOC652255	0.023954494	6.082294791	0.299147418
LOC645385	-0.058058805	10.62689896	0.299148799

DNAJB8	0.03183938	6.309411443	0.299338457
LOC653601	-0.025741668	6.063898626	0.299345212
GAD1	-0.016173832	6.010540654	0.299387188
PTPLA	0.056663594	11.50316412	0.299423513
HDX	0.029283643	6.250957961	0.299436256
OR5L1	0.018138421	6.000755651	0.299477594
LOC651468	0.027018485	6.116815699	0.299517897
CRB1	0.018302846	6.06884876	0.299538693
LOC645799	-0.019986498	6.049990783	0.299656796
MGC40499	0.027021725	6.20821771	0.29967064
SYN1	0.044231076	7.13495796	0.299722377
LOC641733	-0.028638867	6.002946656	0.299765973
LOC645651	0.027094927	5.991152108	0.299810495
C2ORF48	-0.030074363	6.326912502	0.29985711
BMPR1B	-0.030577699	5.965453153	0.299861146
C1ORF220	0.025675513	5.964042299	0.299986642
PRR8	0.036444062	6.23204739	0.300086954
LOC92017	-0.032177288	6.440457037	0.300174497
KIF2B	-0.022759015	6.070662802	0.300263333
OR5H6	0.024537245	5.982515133	0.300290689
LRRC4C	0.032253151	6.286182248	0.3004015
LOC653104	0.024213597	6.063631733	0.300440823
LOC441257	-0.019194979	6.050283875	0.3004897
KCNN1	0.036536587	6.049140204	0.300736962
ENOPH1	0.054599173	8.988352054	0.300739927
LOC647707	-0.025056294	5.937543985	0.300849556
SESN1	0.053900759	9.223606229	0.30088503
LOC392395	-0.022562861	6.011900621	0.300934454
LOC641950	-0.023445355	5.998020489	0.300977588
TACSTD2	0.03344415	6.125002553	0.301120725
SNAG1	-0.026964228	6.069132675	0.3013685
NKTR	-0.048320417	8.898204056	0.301391593
ARFIP2	0.058118329	6.024728094	0.301442352
LOC652618	0.030127076	6.030990729	0.301470478
C20ORF106	-0.019771929	6.053781555	0.30149807
ERGIC3	-0.052182111	9.21017678	0.301684152
C20ORF117	-0.034115018	6.72767714	0.301699792
OTOA	-0.017412401	6.00890345	0.30170994
DDX46	-0.049395699	8.231585917	0.301742737
DEADC1	-0.023909172	6.167293126	0.301837043
SLC6A20	0.016108228	6.158253734	0.301850049
C3ORF24	0.017838054	5.988738658	0.302056218
LOC286187	-0.03092095	5.849004545	0.302130489
TRPM8	-0.017412777	5.974891487	0.30229803
AURKC	0.018165454	6.111318725	0.302336575
GPR75	0.025529358	6.096974486	0.302410816
PI4KAP2	-0.047802964	8.144647364	0.302450499
PNMA2	0.056088228	7.100363108	0.302460524
LOC401351	0.028327264	6.05620805	0.302488411
SLC8A3	-0.016160523	6.094281547	0.30250966
UGT1A7	0.026595693	5.987887863	0.302513862

CLK2P	-0.01726816	6.088907904	0.30256576
CACNG3	0.027278229	6.120176165	0.302571947
C2ORF21	0.019265366	6.228937423	0.302593414
LOC643272	-0.025531176	6.177546802	0.302672676
ATP6V1B1	0.056528662	6.577634252	0.302730435
DDX41	-0.041120036	7.563700119	0.302898666
OR10X1	-0.028014451	5.903428618	0.302961082
LOC652790	0.0250017	6.527033763	0.302971325
LOC728340	0.025246326	5.942565694	0.302971708
TRAF7	-0.026429173	6.469606731	0.303008762
MATN4	0.016869426	5.976269196	0.30309193
ERLIN1	-0.045198266	6.309886323	0.303221152
TTC28	-0.024023095	6.122651963	0.303254997
LOC647391	0.027323543	6.058989161	0.30330601
GJB4	0.038558623	5.970566674	0.303309088
IGFN1	-0.02743173	5.939811218	0.30334878
ABHD12B	-0.015141665	6.061148619	0.303355753
PPP1R12B	0.0803629	7.529430695	0.303385107
LOC646897	-0.036896067	6.461808626	0.303386227
LOC652685	0.037692564	7.144142152	0.30339388
LOC649327	-0.02546411	5.989425011	0.30367411
ERAP2	-0.143967291	7.253052942	0.30369598
LOC644131	0.073843173	7.299860419	0.303724125
LOC643623	0.018397671	6.116321058	0.303734369
LOC649497	-0.029659451	6.109782931	0.303748995
LOC652860	-0.023843639	5.996445155	0.303783907
TDRD5	-0.025705074	5.935087711	0.30391801
CDRT15P	0.034727173	6.029284134	0.303925943
LOC283553	-0.024700677	6.015802195	0.304041433
ZNF182	-0.023354373	6.475285846	0.304079706
NLRP2	0.030314392	5.904272727	0.304138149
LOC388022	-0.026834318	5.930083918	0.304170007
EIF2B2	0.049767409	8.897521184	0.304251195
LOC642149	-0.030400346	6.03876416	0.304505192
ZFYVE20	0.034950261	9.888526487	0.304516811
LOC554235	-0.026490515	6.097130349	0.304582489
CDKL5	0.015296942	6.075555277	0.304616765
H2BFXP	-0.022643502	6.025605878	0.304646789
KIAA2013	0.04650059	8.478548629	0.304737325
FRMPD3	-0.033409341	6.222872053	0.304763193
LARP1	0.044112334	7.853998647	0.304862585
LOC654106	0.03294709	5.974483127	0.304865222
LOC647983	-0.026203362	5.935313406	0.304976434
LOC650030	0.021174556	6.301205842	0.304981182
GSDM1	0.045896406	6.792748689	0.3052204
OTOL1	-0.024731041	6.023385088	0.305254568
LOC646303	0.029361832	6.183695606	0.305263857
LDHAL6B	0.019105875	6.078978373	0.305277499
LOC283874	0.033311957	6.569947111	0.305320314
HHIPL2	0.037753226	6.011661172	0.305489296
LOC731548	-0.028681334	6.077131252	0.305579746

LOC645284	-0.063404479	7.113224705	0.305592973
LOC652235	0.023890903	6.01553168	0.305644638
GTF2IP1	0.050102115	8.024458108	0.305670701
LOC342892	0.025894427	6.226749408	0.305707161
P2RX7	0.037956023	7.646602055	0.305733583
SAC	0.033830315	6.289609624	0.305747566
LOC439951	-0.023611185	6.079672078	0.305788352
LOC648496	0.027455558	6.053336873	0.305814599
ADAM5P	0.028911428	5.990754885	0.305958647
NKD1	0.029419317	6.080783158	0.305976686
SEMA3B	0.047766093	6.745490557	0.305982806
LOC347475	-0.025312995	5.945395373	0.305993031
RIOK1	-0.027035202	7.00761702	0.306014969
LOC652557	-0.024343936	6.204340051	0.306077905
GPRC6A	0.024442127	6.130857517	0.306078367
LOC650733	0.022861303	6.012652975	0.30608424
PVRL1	-0.015420556	6.116303249	0.306097095
ANKRD45	-0.0280068	6.145475144	0.306239037
PAX2	0.017174151	6.120398836	0.306403268
LOC652697	0.033743622	6.049814935	0.306442163
GRHL2	0.02829062	6.061947693	0.306467105
EVX2	0.022476714	5.975573966	0.306468284
OCLM	0.024816848	6.090702022	0.306500655
OR5AC2	-0.024052882	5.916547962	0.306506099
DYRK1A	0.043857282	8.195690146	0.306548698
SRP54	-0.033849241	9.084496274	0.306639446
BTAF1	0.047504772	8.300460834	0.306660403
FANK1	0.02691378	6.23000928	0.306676829
HUWE1	-0.057074484	8.080432023	0.306762815
MEX3B	-0.033231731	6.421267813	0.306807215
LOC646484	0.019195595	5.957348883	0.30688308
LOC647718	0.020245126	6.183869625	0.306905626
LOC649563	0.016553861	6.066127648	0.306933602
C6ORF154	-0.026893396	6.152666343	0.306934341
GLS2	0.018661395	6.014194168	0.306938734
VN1R5	-0.026558801	6.139149164	0.307065849
TLX3	-0.031499091	5.976401301	0.307119682
LOC728608	-0.032198551	6.34206515	0.307125458
C13ORF39	-0.029260902	5.933982339	0.307194642
LOC643007	-0.08657542	7.285562072	0.307298902
LOC642374	0.025666969	6.075508314	0.307318094
LOC651169	0.030310615	6.280296031	0.307330868
KAAG1	-0.026766525	6.052908297	0.307372735
LOC392489	0.024971014	6.199579269	0.307381884
LOC653353	-0.030222583	5.98809816	0.307455004
DR1	0.05423502	7.989461213	0.307591862
ANKFY1	-0.050611379	7.289044318	0.307597023
LOC652451	-0.023236838	6.015123887	0.307646776
PEA15	-0.059216523	12.05617341	0.307784178
TIMM22	0.056236216	9.712224232	0.307850472
LOC391165	-0.02755005	5.887375706	0.307908332

CBLC	0.023538267	6.114357992	0.307948659
HEATR5B	0.057874774	8.50195131	0.308116048
IFI44	-0.085322652	8.444764065	0.308121157
LOC642369	0.027036308	6.382285901	0.308244674
STX6	-0.034149926	7.703379674	0.308333016
LOC644749	0.025593616	6.141850529	0.308444147
FLJ38377	0.027924032	6.054640534	0.308473092
ATP13A4	0.028474377	6.016992115	0.308476183
MLF1	0.104182808	8.02881616	0.308482108
LOC646803	0.02452631	6.112859731	0.308534761
KLF16	-0.029191108	5.989669687	0.308550261
DGKB	0.01947237	6.040414935	0.308711154
PAX8	-0.017165397	6.109720048	0.308760205
LRRC45	0.023610084	6.389766649	0.308794528
C9ORF43	0.03321334	6.252560479	0.308803199
LOC654159	0.024808282	5.946559235	0.308817636
LOC641947	-0.034349196	5.94561635	0.30891448
LOC645871	-0.024765645	6.012036775	0.308965596
AOF1	0.023676775	6.026205246	0.30897765
GABRR1	0.027998336	5.967481702	0.30909356
LOC729269	0.024744393	6.061938151	0.309121695
LOC654092	-0.028249164	6.092875773	0.309165829
HMGN4	0.047701275	9.679328457	0.309192767
ACRC	-0.043752777	6.931065514	0.309348811
LOC647366	-0.015833049	6.000706163	0.309474628
LOC255649	0.018413806	6.015750514	0.309615919
CMPK1	0.052928493	8.966605614	0.309658116
LOC650557	-0.019354819	6.031471254	0.309750896
GJB6	-0.020538688	5.939473043	0.309768394
WDR86	0.03956849	6.362003348	0.309885242
LOC648271	0.024422464	6.04303708	0.309910077
FAM90A10	-0.027641016	6.003815268	0.309944873
CD1E	0.021142184	6.270410134	0.309960881
C8ORF34	-0.031502612	6.657399885	0.310000786
OR1F2P	-0.024274552	6.049723552	0.310044579
UNCX	-0.025795695	5.995344296	0.310207102
LOC651864	-0.027834039	5.986941617	0.310313139
EXT1	0.06135193	7.680905082	0.31033847
TFB2M	0.057056973	8.702626423	0.31037261
HIST1H4L	-0.027366158	5.881828853	0.310577355
LOC440795	-0.021879341	6.061223196	0.310650014
KCNK9	-0.029174182	6.120296762	0.310752807
C10ORF114	0.036590986	6.17599357	0.310778887
SDC4P	-0.028305251	6.184447004	0.310836614
LOC651091	0.026026264	6.055723594	0.310879655
AP1GBP1	-0.020626167	6.159542181	0.310924744
ATP8B2	-0.034089402	6.445313099	0.310939042
LOC652238	-0.023173474	5.915473591	0.31105846
LOC642153	-0.027198048	5.95386543	0.311136484
FKSG49	0.028537704	5.987020338	0.311160636
NRBP1	-0.04461977	7.391263002	0.311174265

LOC440291	-0.025432461	6.079533866	0.311225745
LOC441548	0.021896139	6.020756989	0.311262927
LOC642781	-0.024045852	6.109007141	0.311302928
CHD4	-0.051278179	9.491534814	0.311436328
FAM20A	-0.026777395	6.307234084	0.311467287
SCRT2	0.02779205	6.021275473	0.311481429
HLA-DQB2	0.030254193	6.136352873	0.311631235
LOC644680	0.025515139	6.001095675	0.311659622
VPREB3	0.032856391	6.25397221	0.311668588
LOC653149	0.026224798	6.169499681	0.311713179
LOC644666	-0.026453486	6.209681397	0.311840965
C14ORF169	0.059691643	7.164142263	0.311846449
LOC387885	0.02573249	6.254266091	0.311915846
LOC648790	0.02490612	6.004146946	0.311933917
MEX3D	-0.035820947	6.601613671	0.312105243
LOC283104	-0.025031864	6.137330628	0.312117871
LOC646796	0.028747448	6.028481109	0.312225232
LOC619207	-0.015685756	6.009591716	0.312231423
ZNF784	-0.043304587	7.188135835	0.312291959
NXPH2	0.027910065	6.019745862	0.31231962
LOC255130	0.025746986	6.090843171	0.312440819
C9ORF140	-0.028251873	5.969099626	0.312469974
C3ORF10	0.052194862	9.129517914	0.312492791
VWA2	0.022628965	5.976648653	0.312504586
HS6ST1	0.024032947	6.374698075	0.312598564
RSPO3	0.039090837	6.867578025	0.312609246
HCG4P6	0.026748299	6.073878869	0.312681573
HUNK	-0.025987841	6.009824158	0.312683012
HTR1F	0.029090172	6.017828081	0.312719924
LOC728117	0.027775542	6.062567324	0.312748542
CADM4	-0.032181674	6.39096558	0.312750219
ABTB2	0.056654455	7.104891091	0.312792822
LOC653735	-0.02963227	5.973122656	0.312890094
LOC286260	-0.022714673	5.986262837	0.312920502
LOC652510	-0.026822776	5.943452538	0.312925862
NFE2L2	0.057211677	9.099105637	0.312930687
FZR1	0.036304536	6.818114288	0.313025795
LOC643918	0.017815211	6.067996984	0.313116365
KRTAP4-2	0.025967778	5.940726171	0.313211808
LOC650107	0.023838123	6.052134463	0.313302212
SLC6A13	0.036351651	7.179771336	0.313353586
LOC652787	0.022233634	6.109649745	0.313399012
LOC644717	0.023555797	6.124733304	0.313487594
IFI6	-0.097759181	10.10474667	0.313600218
USP40	0.027776421	6.351664874	0.313687954
APOBEC3H	-0.023925993	6.044049734	0.313700869
LOC650412	0.024697979	6.113735575	0.313732322
LOC643990	-0.024209266	6.020843981	0.313761975
MLLT4	0.018342781	6.18373097	0.313768748
LOC732424	0.025896223	6.098770389	0.313844202
SCAMP4	-0.028337157	6.523842549	0.313845897

CLEC3A	-0.019197119	6.059728927	0.313889551
LOC652022	-0.024458657	6.02705546	0.313895013
CYP3A4	-0.023346262	6.024586042	0.313900243
PSMB10	0.053156779	10.95404907	0.313979728
POMGNT1	0.045186545	10.73352397	0.31401727
TRPM7	0.028262974	6.340880891	0.314027297
NUTF2	-0.051694278	7.064965451	0.314070003
TG	0.028224323	6.140823715	0.314201669
LOC648080	-0.02525269	6.038222468	0.31423898
ACP2	-0.067566036	7.242395094	0.314324996
FAM176B	0.049558225	7.11916047	0.314331633
TAS2R4	0.027197798	6.070005983	0.314358101
AGRP	-0.025625603	6.055734333	0.314371183
C1ORF126	0.027423802	6.002381629	0.314407894
KIAA1632	0.01945666	6.35856587	0.314446837
PRDX2	-0.05781917	10.79999292	0.314448489
LOC388242	-0.022570648	6.051010653	0.31449892
LBXCOR1	-0.022586576	6.074740624	0.314501947
HKDC1	-0.025076631	6.050497805	0.314526084
LOC650716	-0.024825456	6.009699894	0.314555455
SGK2	-0.015162493	5.993726982	0.314641916
PCDHGC5	0.018105281	6.000467839	0.3148564
LOC649813	0.024321051	6.074573319	0.314934902
S100A5	-0.030478079	6.163320636	0.315115259
EFCAB3	0.026938369	6.107754707	0.315196534
CPS1	-0.037056755	6.611883879	0.31524542
RPTN	0.024072405	6.071958514	0.315366969
CFHR5	-0.030059286	6.018165743	0.315387441
OR2H1	0.023786608	5.913656564	0.315388543
LOC441546	-0.026221657	6.047942923	0.31540877
AMAC1L1	0.02353184	6.085441343	0.315470821
LOC644325	-0.023527429	6.044816046	0.315505431
ENDOD1	-0.051520833	7.310956453	0.315517995
ATP8B3	0.022783699	6.128317961	0.315638986
ZMAT2	-0.038643107	8.639325965	0.315685879
MUC7	-0.025446072	5.95288717	0.315702669
C13ORF27	-0.056104278	10.5020962	0.315706205
LOC401242	0.025588122	6.150082729	0.315805547
CD70	-0.027790472	6.214228568	0.315876891
OR13H1	-0.024435879	6.042113354	0.31593805
LOC642637	-0.025012572	6.095326209	0.315964018
LOC731408	0.024565884	6.004434986	0.3159921
LOC645291	-0.019887627	6.058160985	0.315996399
PPIL2	0.015657181	6.14596784	0.316158222
LOC728747	0.024710728	5.965923289	0.316188134
LOC651167	-0.023649302	6.02895911	0.316204534
DEFB124	-0.029241277	6.200091935	0.316225697
RGPD4	0.019743737	6.02757485	0.316229338
VAMP2	0.093208074	7.922065526	0.316231078
PCDHA1	0.014437339	6.047991566	0.316245548
RSPH1	0.02613598	6.05852712	0.316325899

XYLB	-0.02426001	6.115693698	0.316343823
RHOXF2B	0.01834013	6.110390277	0.316391029
C1ORF57	0.060290073	9.396080168	0.316446902
LOC643965	0.028602527	5.919267037	0.316531651
LOC642338	-0.025830297	6.005283752	0.316548182
LOC727866	-0.027886958	6.291191129	0.316574232
CLLU1OS	-0.027260571	6.070024981	0.316699629
SIN3B	0.043903542	7.232498141	0.316756723
MAGEA3	-0.01964449	6.124932913	0.316777813
ATP1A3	0.042596882	6.652309439	0.316946713
EPB41L4B	-0.030892688	6.481953973	0.316989588
DKFZP434I0714	0.027746925	6.144252521	0.317029525
C20ORF175	-0.034431464	6.281949687	0.3170874
LOC647348	-0.024006493	6.17219379	0.317127598
HNRPLL	0.045639997	6.912972833	0.317156536
HMGCS1	0.046374435	8.873573918	0.317193355
GPR37	-0.025175575	6.063725189	0.317290484
HBQ1	-0.027149431	5.976611298	0.317406861
LOC649162	0.024631113	6.05343137	0.317519863
NBPF11	0.039491607	7.98898905	0.317529086
LOC646804	0.024550002	6.147796115	0.317540947
OR11H1	0.019116876	6.043339736	0.31755797
TRPA1	-0.022609079	6.006540928	0.317571817
C3ORF44	-0.025954055	6.001663149	0.317605606
OR5AU1	-0.023660302	6.052974956	0.317622908
LOC647283	0.024636543	5.900915649	0.317686675
PSMF1	0.03781066	9.78295743	0.317811951
LOC645811	-0.025097433	5.979066211	0.317834055
LOC646350	0.022957606	6.000987107	0.317911592
LOC441795	0.024393536	6.047344035	0.317988935
SMARCAD1	0.032054821	6.972548261	0.318006002
CLEC12B	-0.029175043	6.110803531	0.31800839
LOC641875	0.025551086	6.017605083	0.318226714
LOC646098	0.027035824	5.951359191	0.318420955
LOC652821	-0.027643141	6.351325962	0.318436495
LOC90113	-0.025841145	6.124013284	0.318853272
LOC441759	0.028530894	5.971772214	0.318889749
OR2T34	0.026583612	5.96125574	0.318910535
CCDC34	0.058922923	7.50805864	0.318961438
FGF23	0.022971004	6.341814625	0.318980775
VMD2L3	0.024137212	6.059886886	0.319022215
LOC729408	-0.023338136	6.020203464	0.319082368
THAP11	-0.051395058	9.96787487	0.319117719
SYNGR1	0.032992201	7.245363451	0.319283681
ENPP7	0.02208266	6.132823516	0.319323685
TCL1A	0.025367153	5.983377157	0.319348087
GRIN2A	-0.036813859	6.124814236	0.3193908
CRABP1	0.029787622	6.013049878	0.31940737
LOC649857	0.028510772	5.962090074	0.319470538
UNC5A	-0.030368589	5.947181353	0.319502631
LOC644348	0.026453254	5.944047187	0.319561367

LOC402635	0.029528518	5.90494668	0.319571925
ACTL7A	0.0259664	6.019613517	0.319587871
ZNF805	0.036675239	6.987384052	0.319715274
LOC646033	0.027464334	5.984558012	0.319718804
LOC644827	-0.029969774	5.949074847	0.319720286
RAPGEF4	-0.064452432	6.675131135	0.31978022
C6ORF12	-0.017035538	6.16577447	0.319924851
TFAP2E	0.028115744	5.989652986	0.319927427
MGMT	-0.06322331	9.196233416	0.320006835
LOC647359	0.022710688	6.449764327	0.320046832
LOC645293	0.022726965	6.032298879	0.320053691
CYP21A2	0.024572437	6.312839103	0.320086875
LOC139116	0.02581821	5.901493774	0.320215389
ZNF19	-0.025391763	6.037970243	0.320300002
GREB1	0.015659147	6.224691966	0.320392536
LOC650698	-0.029568891	6.303780159	0.32052747
HIST1H4D	0.026100333	5.998372452	0.320537414
DNAJC7	0.063208548	8.851825811	0.320613639
SGPP1	0.02857859	6.15562451	0.320642113
C9ORF29	0.030805058	5.988427311	0.320646208
ZBTB24	-0.026961035	7.346356618	0.320688184
TRPV4	0.016791928	6.074159188	0.320714421
LOC645349	0.022858347	6.254166015	0.320726495
LOC440337	0.026337602	6.172276405	0.320766821
GIPC2	-0.022277374	6.073278066	0.320803255
LOC388621	-0.088919111	11.10045679	0.320961765
LOC387826	-0.025386065	6.213071501	0.320977043
KRTAP4-1	0.02761451	5.989038157	0.32105132
LOC643882	-0.055708956	6.959003211	0.321075341
OR10G4	0.025057226	5.984418017	0.321267552
COX18	0.018688114	6.46498732	0.321275383
LOC646908	-0.026449539	6.024673596	0.321460153
RGL2	0.046248441	7.265687465	0.321625285
C6ORF79	0.025335119	6.004703136	0.321676748
SCN7A	0.037113117	6.571738638	0.321769725
LOC284801	-0.02286897	6.03996766	0.32182165
CYORF14	0.120905961	6.741261343	0.321871281
FNDC7	0.022739407	6.033330161	0.322040522
H1F0	-0.078688725	10.52415695	0.322080811
TTC21B	-0.015604757	5.98883384	0.322153012
LOC644814	-0.032626357	6.029454458	0.322162967
WIPF1	-0.023573878	6.287122518	0.322168191
LOC648064	-0.022037818	6.130815364	0.322173774
TAPBP	-0.045076284	8.365457937	0.322178605
OTOF	-0.016005786	6.016405156	0.322231745
HIST3H3	-0.024667669	6.040500805	0.32234394
LOC441126	-0.027308034	6.371499826	0.32237386
USP42	-0.038647468	7.404835868	0.322426301
LOC653123	-0.028754343	5.956568936	0.322429165
KRT12	-0.025986047	6.093063162	0.322565717
FLJ22269	-0.02956401	5.995997503	0.322613283

CCNB2	-0.036376121	6.090028641	0.322676651
ATG4A	0.029767723	7.516537632	0.322724885
LOC643580	0.023103499	6.092936948	0.322860016
KCNN2	-0.061344275	6.969789579	0.323024128
SLITRK5	0.023946538	6.044981421	0.323058557
FLJ44881	-0.02322456	5.983756256	0.323088624
LOC649812	-0.028466577	5.977260729	0.323174585
GPSN2	-0.044535437	9.721123112	0.323204584
DCC	0.022508598	6.045441829	0.323218323
ZNF828	-0.040411674	8.127431408	0.323218787
MRM1	0.033106862	6.338810387	0.323335697
LOC645304	0.028177548	6.048595832	0.323360607
LY9	0.016566557	6.013871926	0.323469929
LOC642528	-0.025835523	6.110069799	0.323560816
LOC653165	0.022638012	6.047321595	0.323576721
THAP7	0.024041556	6.790699635	0.323642132
HOMEZ	-0.038511024	6.072927865	0.323662804
KCNH7	-0.018780813	5.956794268	0.323930202
TMEFF1	-0.027755278	6.32522528	0.323995952
TNR	0.0239161	5.974351172	0.324021899
C3ORF56	0.030619681	6.018284908	0.324039593
FLJ45202	0.033147991	6.36396686	0.324058727
OR4D5	0.022087337	6.061472071	0.324083344
SULT1E1	0.034190614	6.02484885	0.324174139
ORC1L	-0.023055752	6.083845549	0.324287741
SPANXN1	-0.024292096	5.992646158	0.324317873
PAK7	0.020968948	6.213502802	0.324384211
CUTC	0.054284329	8.927263931	0.324523972
LOC653539	0.013270784	6.036408584	0.324528024
LOC651769	0.029403274	6.067957563	0.324548467
PRICKLE3	-0.026733692	6.233050718	0.324671404
DCDC1	0.023688686	5.958213241	0.324696611
LOC651044	0.02768454	6.08127173	0.32470408
CRTAM	-0.024819838	5.963214526	0.324726827
LOC648066	0.022853945	6.036867218	0.324786975
LOC652274	0.027344331	6.129851125	0.325055266
LOC648763	-0.018219957	6.039370753	0.32506637
LOC649362	-0.028026172	6.131167824	0.325109794
RRAS2	0.102290148	8.881546452	0.325183566
KPTN	-0.023502295	6.065169191	0.325194358
LOC644978	0.029954861	6.141394685	0.325296566
DGKG	0.060460112	6.842119879	0.325527345
VANGL1	0.026023525	6.194927984	0.325561573
LOC648966	0.026285393	6.071597265	0.325574221
MED9	-0.062333238	7.638466064	0.325810296
RBMY1B	-0.025094217	5.970141528	0.325851186
ZNF333	0.019867414	6.538260419	0.326014405
LOC644267	0.02506117	5.925494789	0.32607826
PHACTR3	-0.061762827	6.5138009	0.326196216
LOC645099	0.014050771	6.009246709	0.326220035
LOC728004	-0.034985128	6.035977802	0.326386457

SIKE	-0.029920494	6.12532405	0.326406563
CTLA4	-0.02296627	6.070333737	0.326485336
LOC645956	0.028100744	6.051403111	0.326665562
ST18	0.027979316	5.900836245	0.326692114
TMEM75	-0.025021953	6.033175508	0.326708957
QRSL1	0.033342597	6.345757762	0.326854612
POLQ	0.027995106	6.33098779	0.32686383
BACE1	0.029228523	6.436051121	0.326925096
LOC652070	-0.02550204	6.002664209	0.327024372
UBE2S	-0.023106666	5.917609083	0.3270811
ADRA2B	0.037729169	6.947571438	0.327128197
LOC649784	-0.023953821	5.999650758	0.327339741
FLJ39660	0.031712112	6.195588028	0.327465259
FAM90A12	0.020734559	6.001679606	0.327479894
LOC646719	0.018509888	5.918112471	0.327546375
LIPL2	0.029896196	6.03356279	0.327587412
LOC731640	-0.072927323	11.61919119	0.327643713
LOC652579	0.024731671	5.949918449	0.327667865
POLR1B	-0.026985781	6.365610595	0.327709237
LY75	-0.02852458	5.920572111	0.327791149
TNN	-0.024785379	6.010986559	0.327821111
LOC393076	-0.028490313	6.158654285	0.32784956
LOC284964	0.023647961	6.059822133	0.327877937
LOC643237	0.021854979	5.985416643	0.327904605
LOC647485	-0.024004526	5.940417719	0.327984454
FAM10A6	0.024933727	6.004469152	0.328047902
LOC644554	0.023272176	6.019760242	0.328050886
OR4K14	0.027199743	6.104611733	0.328232857
PLXDC1	0.031837944	6.362252979	0.328339868
SNORD48	-0.028229701	6.132399003	0.328362088
LOC387895	-0.019244966	5.968785082	0.328376839
ANRIL	-0.022957143	6.042260002	0.328407565
ATIC	-0.050836036	9.952153251	0.328423287
PPP1R2P9	-0.022919726	6.032087796	0.32843448
ZNF474	0.018088582	6.168992982	0.328455152
HAT1	0.060640051	7.894800173	0.328455232
KLF7	0.032180697	6.146334455	0.328460133
LOC732150	0.024534339	6.147248585	0.328499744
PLEKHA9	-0.04797553	7.378386409	0.328505655
DAP	-0.046928951	7.758317237	0.328517698
GPR151	-0.026851804	5.977037577	0.328628738
TMC2	0.032517691	6.072971023	0.328643785
FLJ41766	-0.02322405	6.226593674	0.328648199
LOC649217	-0.025833405	5.988148838	0.328786146
FAM181A	0.023410724	6.088176021	0.328808133
XCR1	0.017685952	6.131787435	0.328811639
OR4B1	-0.018637343	6.186718728	0.328828243
LPAL2	0.01208495	5.987249959	0.328936434
ARMC5	0.044825456	6.295169908	0.328952267
LOC648403	-0.02524445	6.130881122	0.329029801
FLJ46154	0.025007116	5.988706807	0.329171863

LOC440105	0.027652267	5.941256867	0.329197422
PCDHGA4	-0.017885419	5.979678175	0.329229595
ZNF660	0.034708386	6.455799339	0.329289877
ZNF396	0.022142097	6.36885423	0.329336717
MGAT3	0.037811649	6.328798913	0.329422715
ALDH1L1	-0.087360426	8.127975156	0.329442309
ZNF542	-0.034712487	6.231870633	0.329538178
LOC644058	-0.023216685	5.964339928	0.329551905
GSG2	-0.024695311	6.025187276	0.329570731
AGPS	-0.046086851	7.751615357	0.32959666
SART3	0.038996899	6.482728072	0.329608358
AMOTL1	-0.028982703	6.242501819	0.329612944
LOC653206	-0.017737052	6.130819409	0.329752839
AMAC1L2	0.015868019	6.149053849	0.329775035
C19ORF26	-0.040663294	6.080345128	0.329776006
TBC1D10A	-0.042150251	6.287276913	0.329839871
ALS2CR14	-0.050899788	7.439686323	0.329893301
LOC646740	0.023017885	6.084175772	0.329963779
LOC402096	-0.023651997	5.930691159	0.329979082
KIAA1862	-0.044939917	7.160978936	0.330020029
PFKL	-0.03431128	7.165083112	0.330056036
LOC400499	0.019910715	6.145258592	0.330063822
AIM1L	-0.01535149	5.997159698	0.33021306
TRPT1	0.054016745	10.23400581	0.330214487
LOC642576	0.023799495	6.127305666	0.330217402
SMAD6	0.080565247	8.834377552	0.330291239
CXCL5	-0.017047571	6.014718124	0.330443739
CYP4F12	-0.043187162	6.545557071	0.330490227
RANGRF	0.058455275	6.429071645	0.330551255
LOC644682	-0.023845602	5.984940962	0.330609326
FMO6P	0.025477429	6.542596643	0.330624842
IL20RB	0.026147457	6.184904102	0.330667517
LOC645663	-0.028016775	6.187992291	0.330726262
WDR3	0.023814049	6.21691112	0.330944537
LOC647247	-0.028564579	5.894885031	0.330994674
ASTN2	0.028531639	6.392018946	0.331019725
FAM14B	0.042983503	6.312046322	0.33105042
LOC400696	-0.032372747	5.926538239	0.331052821
SCD5	-0.032702251	7.213534977	0.331134061
LOC650155	-0.025524977	6.316472959	0.331168072
OR2B3P	0.016970772	6.149803398	0.331202647
LOC642684	-0.022185779	6.149762287	0.331292125
PRB3	0.018055983	6.177216294	0.331463258
DHODH	0.023724425	6.27179714	0.331525123
RGS9	-0.019108833	5.90510654	0.33152953
KSR2	0.01480769	6.229147467	0.331544184
NUDT16	0.053070942	8.369058056	0.331686824
RHEBL1	0.039825464	6.103990808	0.331730216
HPD	-0.034774314	6.002140233	0.33181155
LOC652179	-0.033204423	6.026474399	0.331871248
DDEF1	0.053330578	8.776972992	0.331945597

LOC440104	0.024722648	6.066577986	0.331974322
EID3	-0.041139817	6.150316329	0.332083259
ITGA4	-0.026360819	6.261202762	0.332143564
ANKS6	-0.023265697	5.947175652	0.332146628
HS3ST6	-0.026906396	6.063634655	0.332176042
LOC645504	0.02330006	6.054403018	0.332233297
ZNF595	0.026485498	5.807034217	0.332303053
LOC644816	0.03061639	6.434085623	0.332424883
FCRL3	0.017032043	6.191733875	0.332525645
LOC651195	-0.021547483	6.05413189	0.332552373
MAN1A1	-0.044235593	7.507466565	0.33257444
GPR142	-0.026487524	6.079289261	0.332600308
OR2A1	-0.020843714	6.306269085	0.332674785
RGAG4	0.027825881	6.334096147	0.332684396
FAM90A6P	0.013158649	6.110684231	0.332850622
TNP2	-0.027609109	5.890890242	0.332882331
FLJ25076	-0.024680006	5.924228715	0.332916502
TMEM207	0.048235705	7.034199049	0.333006626
PDPR	-0.06276336	7.872608546	0.333057682
LOC645458	0.018982745	5.98515663	0.333064475
TCEAL1	0.034122915	7.465753498	0.333076102
FGFBP3	0.027131122	6.218866293	0.333088542
TEKT5	0.021714242	5.951347888	0.333104506
ZNF553	-0.030274695	6.072440169	0.333179958
OR13D1	-0.027828697	5.944062306	0.333183671
C9ORF97	-0.037412746	6.895977501	0.333198308
LOC646731	-0.023609303	6.005872945	0.333293247
CFC1	-0.018736396	6.107086842	0.33335578
DYDC2	-0.026323087	6.296032768	0.333380106
S100A7L2	-0.02547177	6.424303437	0.333433812
SLC23A1	0.025514711	6.548423598	0.333493645
LOC643423	0.03327566	6.502675687	0.333584165
MYF6	-0.018690796	6.137470145	0.333741025
FAM22G	-0.025119342	5.960283241	0.333764989
NR1H4	0.024720223	6.108445154	0.333771957
SLC35F5	0.05302216	8.056322035	0.333910201
ZNF449	0.02393214	6.177164266	0.333985709
UROC1	-0.028757348	6.081455512	0.334122415
LOC642950	0.028541525	6.031725052	0.334129272
CABP5	0.026383199	5.915404481	0.334135305
LOC646096	-0.027279585	6.019094387	0.334136458
CDH12P	0.026881692	6.105385146	0.334267126
OLFM3	0.028329044	5.887633543	0.33430832
FBLN1	-0.091059618	9.38947624	0.334353912
PAGE4	0.029053705	5.992620747	0.334356951
RGPD5	0.012006702	6.046221697	0.334380749
HIST2H2AA3	-0.067103525	9.123666983	0.33442661
PPP4R4	-0.0161103	5.989369267	0.334428572
LOC136288	0.025953053	6.040309352	0.334478225
DLX3	0.033080583	6.223296216	0.334488985
LOC728504	0.025575802	5.984347679	0.334619101

LOC645279	0.025802801	6.090070364	0.334621206
NDUFA4	0.03892307	14.12959995	0.334669213
TTLL9	0.030603077	6.14110716	0.334828896
KLK15	0.014828612	6.029506018	0.334832971
LHFPL3	0.036579703	6.626607089	0.334853914
LOC649270	0.014424279	6.160253077	0.334899978
RBMY1F	0.026928009	5.981313133	0.334958876
BPESC1	0.032772631	6.127713565	0.334964334
MRPL28	-0.036751469	7.936578317	0.334967346
HMGCR	0.045622091	7.714368328	0.334971643
AP1M2	-0.039626955	6.05344075	0.33498535
AVIL	0.025688635	6.480376187	0.335001845
IQCB1	-0.023309254	6.816095389	0.335066396
DNCL2A	0.022835591	6.04055306	0.335102118
DIO1	-0.015038369	5.944034349	0.335111688
OTUD6B	0.042858912	7.461771201	0.335115667
LOC728403	0.027590728	6.0457143	0.335200057
LOC653115	0.054112517	6.688055076	0.335265896
LOC641929	0.024082938	6.066519588	0.335396621
LOC645172	0.024670096	6.125239013	0.335442063
KIAA1772	0.045365958	6.482459338	0.335557247
TAAR9	-0.026578034	6.114550182	0.335614635
LOC642702	0.023671062	6.010140068	0.335636148
JDP2	-0.047825029	6.480785483	0.335715829
ITGB8	-0.026022015	6.105870785	0.335785578
C19ORF62	0.032935384	8.856083005	0.335956206
NPBWR1	-0.027060635	5.952912837	0.336225275
SNAP91	0.15629893	7.054277172	0.33627566
TFR2	0.02432238	6.067565767	0.336317748
CCR10	-0.026420571	6.249919749	0.336451181
KIF23	0.017829565	6.071707584	0.336517518
PLD4	-0.030831035	6.208321993	0.336620271
LOC646933	0.024084676	5.908235563	0.33666084
GBX2	-0.023656661	6.315344604	0.336663684
HCG22	-0.023605896	6.08988089	0.336689352
LOC441282	-0.015638682	6.052488657	0.336832403
COL11A2	-0.015819385	6.130452667	0.33692481
ARGFX	-0.021063793	5.925412655	0.337040194
LOC648600	-0.027636549	6.21072456	0.337040313
KRT84	-0.023683132	6.125503905	0.337044684
FAM7A2	0.01409558	6.011828357	0.337074201
LOC732416	0.025051653	6.221094719	0.337103611
FLJ44817	0.02517932	5.95468815	0.337174726
XRCC5	0.043575375	8.132254273	0.337198911
SULT1C2	-0.020200485	6.040477814	0.337251581
LOC648364	-0.050457733	6.023332818	0.337347888
NUP155	-0.024494031	7.163783879	0.337348916
LRP6	0.028397912	6.516539738	0.337385206
COPS7A	-0.034553047	10.64070893	0.337414645
OR10R2	-0.02572522	6.021086883	0.337566991
LOC392215	0.019735941	5.985868781	0.337620888

TAGAP	-0.014589542	6.019490681	0.337675311
SLC6A4	-0.022057541	6.04141036	0.337747971
LOC651777	-0.030297354	6.221780668	0.337753881
B3GALT1	0.023821599	6.260705204	0.337768591
PLXNB3	-0.020278578	6.055907293	0.337780493
LOC391276	-0.022310382	5.999105444	0.337799089
LOC651268	-0.026551268	6.100734657	0.337816585
VCL	0.061651195	11.0075903	0.337942846
C4ORF37	-0.016818459	6.124328168	0.337985081
CLECL1	-0.025831799	6.026133926	0.338002182
LOC642265	-0.02563601	6.067424849	0.338107637
HILS1	-0.026493904	6.202544444	0.338124872
APBA2	0.022222869	6.004740154	0.338181498
FBXO41	0.020622207	6.225998392	0.338284265
CLGN	0.077541996	7.547365498	0.338296283
CXORF58	-0.022918311	5.976999411	0.338312964
ERAF	-0.024395308	6.113815558	0.338446554
DMXL1	0.053472018	6.406761165	0.338449134
LINS1	-0.018402969	6.334455165	0.338638501
CAMKK1	0.020524535	6.23566792	0.338702501
LOC647336	-0.027283529	6.006606935	0.338743746
RPS25	-0.041291585	14.16950304	0.338854278
LOC645194	-0.024104364	6.037190568	0.339135641
LOC651381	0.025408047	5.937402824	0.339213076
LOC645879	0.025008981	6.054576033	0.33930658
ADNP2	0.043222543	7.772795013	0.339322068
LOC652160	0.027446312	6.066721745	0.339407557
UGT1A9	-0.023418534	6.033041223	0.339456301
ATP2A3	0.02655529	6.191172733	0.339907645
ECHDC3	-0.075732511	10.39580672	0.339952019
SPCS1	0.050137639	11.23858761	0.339952419
NPY5R	0.019305292	6.108181436	0.339977505
LOC126520	0.035929925	6.179316657	0.340049543
LOC652405	-0.023559583	5.946675532	0.340083796
C16ORF55	-0.02612414	5.942260371	0.340145775
LOC646855	0.032222448	6.108976098	0.340203513
LOC284701	0.014045682	6.108497257	0.340235693
SHD	-0.022074481	6.024762153	0.340270965
KAT5	0.028441391	6.617776621	0.34030412
LOC650475	-0.023926676	6.087841506	0.340312113
PIK3C3	0.045613227	6.611582363	0.340395952
KCTD7	0.030089317	6.215346983	0.340448533
LOC730076	0.027146656	5.968970558	0.340647089
SST	-0.02655303	5.908990432	0.340882141
GLYAT	0.01645625	6.026007068	0.340882608
MGC24103	-0.045072814	6.707148639	0.341055005
LOC644057	0.023918104	6.213347716	0.341058982
OR52I1	-0.023080506	6.043402578	0.341162502
LOC645333	-0.023706902	5.9603914	0.341278448
LOC649092	-0.022133452	6.076620991	0.341323053
RPA4	-0.026392418	6.259541447	0.341383961

LOC653249	0.015256578	6.017441138	0.341402224
LOC641908	0.020586855	6.061342356	0.341468354
LOC651031	-0.022835719	5.972795837	0.341667428
SMA4	-0.047874237	7.135640815	0.341719302
LOC648963	0.027667972	6.1868649	0.341754988
LOC645659	0.02870801	6.38853425	0.341793589
GYPA	-0.020271031	5.891187368	0.341828187
RPE	0.022815387	6.512381451	0.341881255
OR8K3	-0.025773452	5.955283298	0.341902829
LOC653073	0.022623566	6.438067073	0.341922352
DHRS1	0.061390234	8.357512519	0.341928825
LOC645179	0.025218384	5.961867998	0.342023848
LOC442057	0.025422284	6.325435963	0.34204355
LOC401498	-0.024454943	5.913495987	0.342102313
DEC1	-0.027679635	5.985958163	0.342207839
TSPAN33	0.047963724	7.790561445	0.342241121
LOC731898	0.026174793	6.027729661	0.342287125
MICAL3	-0.024701554	6.041114814	0.342329449
CRHR2	-0.023144239	5.935659736	0.342501257
FBXO43	-0.017861937	6.110395505	0.342527695
CASKIN2	-0.037286178	6.14382936	0.342536853
OLA1	0.015401089	6.112605416	0.342547471
RPL23AP7	0.058414187	7.606581853	0.342609314
ALOX12B	-0.026171986	6.009247777	0.342640882
LOC652086	-0.021917909	6.037530536	0.34272735
LOC285299	-0.025358077	6.11242104	0.342752016
LOC648329	-0.023781457	6.118223788	0.342752111
MEP1A	0.026437794	6.258480469	0.34279132
TMEM175	-0.060574472	8.446387568	0.34281427
FLJ35773	0.024959357	6.159331423	0.342831087
OR51A4	-0.027452738	5.865191205	0.342869761
LNP1	0.035677802	6.041669863	0.342934099
UTP20	-0.013732824	6.073375428	0.342949874
LOC644285	-0.025901775	6.012248533	0.343046855
LOC650509	0.023291291	6.225330031	0.343082531
LOC389370	-0.024961093	6.111707529	0.34313197
LOC389813	0.019857963	6.057480364	0.343177607
HSD11B2	0.029546253	6.212853176	0.343199313
LOC138652	-0.021493899	6.177260069	0.343346964
MAGEA9	0.023917432	6.043094304	0.34338263
LOC728565	-0.042379556	7.155209008	0.343434007
LOC389457	0.022420802	6.118861583	0.343489296
IFIT1L	-0.025357255	5.939372453	0.343662082
LOC648796	0.023917403	6.062187509	0.343669396
SLC6A3	-0.024672277	6.0311064	0.343878665
ZNF385C	0.024728571	6.002232422	0.344152983
LOC646492	0.027776066	6.017256528	0.344184525
LOC652743	-0.025892615	6.005963186	0.344228184
LOC644090	0.023954453	6.109208505	0.344304746
DPH1	0.026923416	6.56598345	0.344395708
LOC645317	-0.03672493	12.52716789	0.344456581

LOC652529	-0.025622562	6.018272881	0.344503967
BTF3L4	0.037738883	8.427279503	0.344586343
LOC647154	-0.024744444	6.016571283	0.344616013
LOC440917	-0.020890876	6.01552335	0.344637734
CDX1	-0.026086426	6.001035988	0.344740586
LOC647960	0.021860578	6.054377063	0.344803252
LOC728103	0.024405689	5.966165417	0.34484944
MUC4	0.016481843	6.071782506	0.344853375
ERICH1	0.038325284	8.098630511	0.344997685
NEDD4	-0.017950924	6.526212304	0.345058117
DEFA6	-0.031625188	5.866612839	0.345200139
OR6C65	0.019890757	6.046035074	0.345230732
PRLR	0.02170855	6.072809193	0.345421788
EEFSEC	-0.028942888	6.498887091	0.34554052
CASP8AP2	-0.021910651	5.913834025	0.345604652
LOC642347	0.024244872	6.171139352	0.345604761
KCNJ14	-0.015744238	6.225536489	0.345635286
OPRK1	-0.023755869	6.261464969	0.345650435
OR4A15	0.032008277	6.142763475	0.345651909
LOC641311	0.025554518	6.002362391	0.345695548
MAGEA10	-0.016873245	6.043355133	0.345793911
LOC650185	0.021108913	6.170534877	0.345867148
PRPF3	-0.066373435	8.152009117	0.345907514
BMP3	-0.033780056	6.134004126	0.346053019
LOC648480	0.027637857	6.002274412	0.346066571
WDR12	0.030684669	8.450875311	0.346097431
SLC5A9	0.024645125	6.08004045	0.346131346
PSG2	0.02009296	5.999281167	0.346184407
LOC729837	0.027441244	6.386985923	0.346206264
CYP17A1	0.025437496	6.08038229	0.34623149
KRT15	-0.024311057	6.060479009	0.346302789
FAM46D	-0.024358197	5.953057818	0.346321853
RFX6	-0.023605405	6.087086112	0.346379782
CHST5	0.020964201	6.012999955	0.346650724
LOC645636	0.02209948	6.200908957	0.34666385
GPC2	0.034760204	6.246379964	0.346828344
HSPA4	0.069128204	8.889719943	0.346941931
DVL1	0.022430799	6.414296095	0.346969379
C11ORF84	-0.038935423	6.006098633	0.347009729
LOC652805	-0.028484471	5.99220899	0.347122251
SPIN2A	0.025284898	6.141397065	0.347134844
GRIN1	0.023108694	6.563634343	0.347139421
LOC652566	0.025620084	6.207089414	0.34715357
CEP192	0.057753137	7.414536197	0.3473387
APOBEC1	0.024792667	6.154499079	0.347375603
USP51	0.026688867	6.055497216	0.347521983
METTL7A	-0.088422968	7.943516966	0.347624671
LOC641802	-0.029399475	6.090508999	0.347664975
LOC653591	0.025822396	6.202486575	0.347908514
C1ORF38	-0.023815425	6.110858534	0.347909381
FAM96A	0.048194691	9.8338156	0.348005194

FGF16	0.024605752	5.897903235	0.34801939
LOC391282	0.016631685	5.920687493	0.348081267
LOC644099	-0.02332239	6.0390888	0.34812984
PDE8B	0.072673978	7.006019791	0.348180809
KNG1	-0.025243569	6.050037722	0.348203536
SLC25A10	-0.064592463	6.871485315	0.348288784
DAZ4	0.014703146	6.081040873	0.348289349
DDA1	-0.039085347	10.59721215	0.348341023
TCF19	-0.018715569	6.096619518	0.348377222
LOC441261	-0.02699326	5.939718691	0.34838464
LOC731444	0.025392973	6.175932635	0.348405731
LOC284577	0.023780357	6.18732976	0.34844098
ZNF804B	-0.023353044	5.985362231	0.348476501
LOC643006	0.021875973	6.013322425	0.348686691
GPR144	0.024901991	6.009449014	0.348732914
LOC402117	-0.025368638	5.962180294	0.348792941
CC2D1B	-0.024691332	6.501263522	0.348879036
CD7	-0.025911311	6.260246364	0.348883365
ROPN1	-0.063094097	6.726477436	0.349074676
C18ORF45	0.032854039	6.354147053	0.349139166
MFRP	0.023953976	5.923386883	0.349193656
NAPSB	0.032637681	6.341983587	0.349286928
LOC440934	-0.048483227	6.450054163	0.349332357
MMP7	-0.01736898	6.004360577	0.349400473
LOC650298	0.061609983	7.854982224	0.349484146
NEUROD4	0.024060979	6.072363444	0.349490771
CCDC28B	-0.058353727	7.830772896	0.349561176
HLXB9	-0.026748012	6.157621797	0.349586561
LOC727778	0.024557471	5.924774258	0.34966834
LOC441249	0.023911313	6.128007983	0.349681283
C7ORF34	0.023791714	5.963929773	0.34968773
CLDN4	0.031852695	6.162989108	0.349852942
SQLE	-0.04222153	7.038982763	0.349891134
OR2W3	-0.023051251	6.220898485	0.349897691
ANKRD21	0.023736063	5.937364718	0.349987949
GRHL1	0.014740912	6.081766089	0.349997679
OTOP2	0.024963425	6.034322236	0.349997903
LOC644043	0.02476267	6.236811383	0.350063805
LOC653917	0.024082904	6.246825334	0.350066297
LOC649546	0.02054737	6.018650348	0.350118615
FLJ16171	-0.023500366	5.910098696	0.350177928
RNF32	0.014303548	6.01243129	0.350271236
CLRN3	-0.024161384	6.21805648	0.350339996
CLPX	0.033326852	8.358676204	0.350390495
LOC648039	0.021582548	6.229771681	0.350402036
GATAD2B	0.031501524	7.764082	0.350416269
FMN2	0.026391502	6.018652368	0.350488702
LOC653623	0.023924795	6.153200159	0.350510104
LOC651979	0.023808785	6.275065351	0.35059896
HAP1	-0.014629464	6.116763052	0.350639912
RLBP1	0.025036548	6.115254938	0.350712817

LOC654134	-0.021879118	5.888848487	0.350728655
SNF8	-0.062233041	8.487902122	0.350872766
CRISP1	0.01611467	5.926887883	0.351042177
LOC644975	-0.02259662	6.055439329	0.351133862
LOC401876	0.02067634	6.11894034	0.351142577
LOC728832	-0.020963955	6.049336668	0.35117373
C6ORF170	-0.040094771	7.248603887	0.351188383
SNCB	-0.014278969	5.99882442	0.35119985
LOC643355	0.026412397	6.066643144	0.351253232
PRPF38A	0.020675932	6.800941368	0.351345678
DDX47	0.032774618	8.913337015	0.351359611
LYCAT	-0.062177163	8.834160235	0.351415077
LOC730027	0.02911624	6.158532995	0.351621289
ZNF786	-0.062946918	9.122847606	0.351684466
LOC648700	0.023422364	6.050414512	0.351706618
GTF3C4	-0.029110551	6.082996804	0.351712146
HMHB1	0.028845481	6.034986133	0.35175752
SFT2D2	-0.024120962	6.102865167	0.35187184
CRYGD	-0.021876229	5.961394525	0.351880343
GOSR1	-0.023273358	6.706884823	0.352081975
C6ORF191	-0.022246745	6.05841483	0.35209465
PROC	0.022712546	5.973470023	0.352293676
TMEM131	-0.051965156	9.868459795	0.352368838
LOC643152	-0.023868238	6.001317304	0.352484009
CCDC63	-0.021916156	6.091292212	0.352675551
LOC400707	0.030929161	6.05593395	0.35287495
LOC649723	0.032472536	5.894266364	0.352901695
ZNF670	0.018925578	6.147765847	0.35291778
TLE2	-0.045590247	8.195251987	0.352927002
LOC442283	0.025510093	6.176628711	0.353046602
LOC644059	0.024326053	5.977377414	0.353075332
PTF1A	-0.026440476	5.97842784	0.353216674
LOC644845	-0.025015538	6.140727551	0.353315774
LOC642313	0.015269339	6.103647444	0.353475964
MRPL2	0.05956666	8.201069247	0.35395809
SEPHS2	-0.042000061	8.078853467	0.354002188
B3GNTL1	0.028043019	6.35769895	0.354090355
LOC644234	-0.023682352	6.190845177	0.354126644
FLJ43806	0.024998737	6.0560374	0.354199269
DHDH	0.020984555	6.157911247	0.354238223
OR2A14	-0.02604685	6.139714095	0.354335101
LOC440341	-0.046157508	8.746269407	0.354355269
UBA7	-0.049706817	7.796084834	0.35441554
LOC644319	0.02284623	6.15450139	0.354476755
ITPK1	0.051145044	8.244418931	0.354609231
ICAM4	0.015434169	6.109165255	0.354673355
KRT222P	0.025862538	6.086011701	0.354686727
LOC642985	0.018774318	6.071509408	0.354723212
SP8	-0.014527419	6.171882183	0.354736642
SHH	0.029854929	6.273192755	0.35498481
FOXD1	0.021931657	5.975531699	0.354989456

LOC644044	0.024025205	6.264933595	0.355145578
KCNJ3	0.025972314	6.02227007	0.355166497
LOC651668	-0.02126832	6.045502695	0.35519252
LOC653759	-0.023359082	5.935180365	0.355313939
LOC652793	-0.024939998	5.997027648	0.355349721
MEP1B	0.025909464	6.318286797	0.355399445
LOC728116	-0.026446085	6.126867437	0.355421889
MRPL42	0.035233722	7.721556721	0.355447753
LOC285095	0.023055871	6.154157341	0.355462718
LOC649150	0.082413955	11.69313087	0.355590586
LOC402641	0.022053992	6.0427596	0.355662183
CHD8	0.066419342	8.067620239	0.355830902
SERAC1	0.017829911	6.332121324	0.355965968
LOC441874	0.023196686	6.048815577	0.355996201
LOC390877	0.021857335	6.098569589	0.355999982
BLID	0.02384983	6.104495199	0.356071401
NOL10	0.027115769	6.82435917	0.35609212
HTR7	-0.019279689	6.193636219	0.356179199
LOC340094	0.024192405	6.144119773	0.35620253
ISCA2	-0.037662687	8.120531638	0.356319195
RTN2	0.025191253	7.086421112	0.356408133
AVPI1	-0.058398358	9.277582	0.356454857
LOC646920	0.023756426	6.205507366	0.356526963
TRHR	-0.021508966	6.050578379	0.356749617
ASCL3	0.021308459	6.044133795	0.356751877
H2AFB1	-0.022996737	6.097444871	0.356755756
SLC25A32	-0.028832476	6.12301757	0.356761715
KCTD10	-0.065229042	8.002420833	0.356842046
TRIM13	-0.04296233	7.880798138	0.356892461
LOC642939	0.022900085	5.992893701	0.356897027
CHORDC1	-0.045030787	6.719262612	0.35696539
LOC648684	-0.02535016	6.424290263	0.356971358
PEX12	0.016380443	6.111247522	0.357116234
IGF1R	0.039297808	6.467205312	0.357123316
LOC653867	-0.024189896	6.128723138	0.357234366
CD19	-0.02832455	6.080454936	0.357309926
TNNI2	-0.025932692	5.995036466	0.35731397
LOC651987	-0.027016974	6.201697434	0.357419118
TGM7	0.034427167	6.234507304	0.357489726
LOC343296	0.022416642	6.126919027	0.357503845
OTOS	-0.020491652	5.951194784	0.357524884
UBQLN3	-0.025503486	6.626488354	0.357592559
LOC646946	0.021148585	6.011326767	0.357692723
SLC43A1	-0.039462407	6.125776003	0.357783811
SH3GL3	0.024268221	6.067647059	0.357879137
LOC729957	-0.020640167	6.018940756	0.35788257
FLJ40504	-0.029387071	6.278734849	0.357899086
FLJ31945	0.017816772	6.118168843	0.357910034
EIF5B	-0.066401334	9.499048962	0.357924416
ESR2	0.016841714	6.064079052	0.358054029
LOC255025	0.030472055	6.002412626	0.358370644

LOC644421	0.020927667	6.216662832	0.358551598
SPSB4	0.03543124	6.174124413	0.358651827
LECT2	0.028264996	6.010585341	0.35872801
MGC13005	0.018986189	6.135035359	0.358731181
LOC642680	0.023209478	5.983183299	0.358860049
DLEU2	0.016304791	6.123679098	0.35887317
FHL1	0.101142042	12.39710839	0.358874207
C2ORF58	0.033260144	6.123647448	0.358885093
C3ORF48	-0.021890514	6.054411592	0.358918102
LOC652466	0.025884784	6.092786335	0.358940567
LOC650815	0.043921882	6.049895623	0.358997802
GPR123	0.02261711	5.971427594	0.359078145
SNED1	-0.014839124	6.042494611	0.359098838
OR1L8	0.027087853	6.181528038	0.359188012
FLJ40448	-0.034504405	6.425805868	0.359246876
CYP19A1	-0.017715088	6.074634261	0.359252473
BCL2L15	-0.016063966	5.996661053	0.359292945
UBE2C	-0.023944021	6.156845198	0.359298296
C10ORF28	0.040882312	6.774757902	0.359306009
LOC649765	0.025571095	6.178181394	0.359329946
LOC650455	0.025873823	6.009092931	0.359334889
ZBED3	0.029406756	6.126739057	0.359618452
PLA2G2F	-0.021920517	6.050567228	0.359678934
NUMA1	0.058403203	7.343936143	0.359715899
LOC401525	-0.026584085	6.136064515	0.359742173
BXDC2	0.041225028	8.314913492	0.359820464
IRGM	0.019939916	6.175104328	0.359855291
WNT10B	-0.030642608	6.014944517	0.359892345
LOC652396	-0.019590617	6.068070335	0.359925564
ERG	-0.014860815	6.184210733	0.359927237
CD226	-0.024114783	5.932385905	0.35993076
KCNC3	-0.021520225	5.976321988	0.359961863
LOC647630	-0.024849647	6.209036592	0.360093443
C1ORF91	-0.026047175	6.235391536	0.3601285
CNGA2	-0.024652408	6.091417999	0.360174145
LOC652341	-0.020889087	5.904120438	0.360224906
PSEN2	-0.031798783	7.92873654	0.360226753
LOC643734	0.02607466	5.951329251	0.360398606
LOC648803	0.018477194	6.104923724	0.360593021
CYP4F3	0.015635977	6.01631638	0.360629647
C5ORF34	0.015732012	6.166532024	0.360738965
CNTLN	0.025400595	6.550870378	0.360886767
CCDC148	-0.023930493	5.965008145	0.360943947
LOC646580	-0.022788079	6.014072617	0.360955319
MPG	-0.014325832	6.118197305	0.361052445
TFF1	-0.024713184	6.001470876	0.361055069
BFSP2	-0.028408887	6.099858016	0.361202623
FLJ16124	-0.023366939	5.935594665	0.361221529
RIN1	-0.030837299	6.534909919	0.361330939
MAGEB6B	0.02574945	6.068953839	0.361404315
LGALS4	0.023557189	6.177348785	0.361445515

DOC2A	0.018335619	6.013167116	0.361507924
WDR23	-0.037741392	7.350824854	0.361518679
HCCA2	-0.03267372	7.648853726	0.361531881
LOC645529	0.022699625	6.052180734	0.361673441
ZP4	-0.022912617	5.912389963	0.361729676
LOC646443	-0.02128848	6.105319816	0.361788141
LOC653653	-0.019529811	6.025150727	0.361803219
ADAM12	-0.01734374	6.017291953	0.361848589
FAM87B	-0.022094426	6.099530274	0.361862023
TTTY6	0.018837887	6.110746473	0.361904945
ADCK4	-0.057374366	6.562598785	0.362039365
SFTPB	0.017431044	6.142752991	0.362103526
MYOC	0.062548764	6.610743814	0.362193487
HNRPUL2	0.037506608	8.556645036	0.362297198
PSD	0.023264523	6.081417441	0.36231133
LOC643988	0.025436188	6.147906537	0.362500332
RBM35A	0.025402026	6.024610611	0.362535781
WNT3	-0.025234686	6.470577565	0.362539751
GPR87	0.021167363	6.110716863	0.362549206
GSTA5	-0.023613817	5.888691524	0.362565955
RGS20	0.016454281	5.993798762	0.362593835
LGR4	0.037064587	6.475993805	0.362706774
DDX19A	0.021012489	8.386880822	0.362727397
RASL10B	0.030124447	6.436983881	0.362905353
CTAGE1	0.018496084	5.986864898	0.363049451
LST-3TM12	0.023048833	6.008336888	0.363089886
GABRA6	0.025691478	5.993808512	0.363105875
LOC651876	0.02057016	6.308437917	0.363140371
ETFDH	0.07284615	10.87924747	0.363448116
LOC650942	-0.022063082	6.006267237	0.363451749
LOC645039	-0.019843172	6.112219233	0.363526202
C10ORF113	-0.023133798	5.900986445	0.36374803
GPR182	-0.029974815	6.166584647	0.363759442
C10ORF90	0.025488803	5.973647076	0.363802596
LOC728160	0.015015965	6.009860357	0.36381896
SEMA3A	-0.031337751	6.329532176	0.363922403
FOXA3	-0.025869715	6.023287289	0.363973198
CCDC68	-0.019910263	6.478843869	0.364111089
ERCC4	0.019043843	6.244313027	0.364136154
OR8G5	0.02154162	5.99766913	0.364499558
LOC652202	-0.022949219	6.017527535	0.364688168
LDLRAD1	0.024033968	6.122413298	0.364712945
LOC648792	0.023777216	5.888339207	0.364776158
PKHD1	0.017232846	6.248689947	0.364811311
LOC143941	-0.021650275	6.108294348	0.364855766
RPRML	-0.06768504	6.596107442	0.36487803
NLGN3	0.023263965	6.002216382	0.36492919
DCP1B	-0.022903975	6.225291867	0.365007909
GUCA1A	0.022876141	6.367419429	0.365045609
LOC648752	-0.026459435	6.073024232	0.365095518
DHX58	-0.035518176	6.542077999	0.365188466

TAS2R48	0.02368306	6.045134162	0.365206168
CFHR4	0.023440258	5.947517491	0.365222469
MOGAT3	0.021760842	5.958649928	0.36539871
SLC37A1	-0.029468341	6.610186461	0.365428312
ZDHHC3	0.039866988	7.622741618	0.365493961
CSN2	0.023491794	6.052065732	0.365528326
LOC650020	-0.026322053	6.267534642	0.365930682
LOC642594	0.021408485	6.059278892	0.36602743
FSTL4	0.016564589	6.114969733	0.366129016
NTN2L	-0.023212546	5.944337327	0.366166355
ZNF683	0.025768073	6.311252149	0.366176426
LCE1D	0.023694283	6.133523774	0.366199899
KIAA1627	-0.055671248	7.499571528	0.36621869
CCDC123	0.02945065	6.470319667	0.366240911
LOC644277	0.026237197	6.145196579	0.3662832
FGL1	-0.01206898	6.182505393	0.366336483
BPIL2	-0.026224598	5.992244693	0.366412627
XAGE1D	-0.026537173	6.054789658	0.366503529
DCN	-0.062466517	12.12713102	0.366513449
WNT8A	0.01639004	6.034643989	0.366524008
MCC	-0.029541432	6.482746191	0.366549231
TATDN1	-0.049167853	8.041714884	0.366593195
ZNF358	-0.048582605	10.13444419	0.366647034
LOC389142	0.021806658	6.159626335	0.366762428
UNC5D	-0.025065704	5.826308513	0.366810802
LOC497256	0.019670493	6.070222561	0.366815855
LOC143678	-0.021233742	6.040941718	0.366827232
OTC	0.032473965	6.023595534	0.366879858
LOC441135	0.022274085	5.946847291	0.366880124
FMO9P	0.019011217	6.002898201	0.366909171
TTTY19	-0.01721021	6.127311141	0.366996167
PKIB	-0.017635518	6.100732288	0.367120327
LOC390364	0.023496657	5.981531435	0.367262428
LOC644278	-0.020380601	6.077321971	0.367296769
LOC651353	0.020946786	6.080407049	0.367431582
TTTY18	0.014843173	6.060816403	0.367461587
CPN1	-0.023530238	6.016230228	0.367586191
LOC653093	0.022346561	6.166201843	0.36761633
PRAMEF5	0.022790036	6.449608185	0.367784211
MED4	0.048296718	8.557452828	0.36778972
LOC389111	-0.020495423	6.130770589	0.367806371
LOC643083	0.027515742	5.926757638	0.367883921
LOC338667	-0.02436705	6.176977726	0.36798804
LOC651390	0.021392932	6.026597305	0.368185081
TPTE2	0.015419838	6.001624585	0.368208669
FUT11	0.036747278	6.051096467	0.36828625
HBM	-0.024178842	6.105613135	0.368511404
HMGCS2	-0.22050721	7.65923639	0.368540923
MLNR	0.024378321	5.99659542	0.368564997
MTMR6	0.065183772	8.593541316	0.368615056
LOC652670	-0.023571677	5.896072059	0.368933086

LOC642082	0.043011548	7.791452493	0.368967793
SFRS2B	0.030487572	8.48495284	0.36898818
DSCR10	-0.023392946	6.364002961	0.369131017
LOC643841	0.025736839	6.131265562	0.369203687
LOC647456	0.023071431	6.41324167	0.36926177
SLC24A3	0.040054375	6.214980127	0.369271256
LOC728643	-0.110004911	8.33954242	0.369592594
C12ORF32	-0.035807394	6.651739564	0.369634066
RELT	0.022052342	5.941401258	0.369750376
PCDHB1	-0.022244027	5.90393365	0.369766902
OR13C4	-0.023102679	6.027991604	0.369855629
LOC642574	0.028660218	6.099411919	0.369873115
LOC649067	0.034094084	6.168937023	0.369901559
MCM7	0.031992162	7.95128553	0.369920263
CCDC105	0.022185604	6.025575276	0.369922494
GPR103	-0.023845984	6.117562981	0.369945272
SLC38A11	0.040754025	6.519374655	0.370032031
LOC648716	-0.015838531	6.141001423	0.370073133
LOC390372	0.02425954	6.414775845	0.370100757
LOC643433	0.046754952	11.33151921	0.370121787
ANKRD49	0.030401448	7.222073805	0.370344618
LOC441114	-0.023734449	6.082558169	0.37036633
LOC644280	0.028737762	6.041760621	0.370522381
TEX13B	-0.023046042	6.121272852	0.370601677
LOC440295	-0.024295647	5.942774182	0.370620923
CCDC54	-0.023404411	6.001725861	0.370705239
COLEC12	0.053414393	7.808644342	0.37074643
LOC652651	-0.022539193	6.077472762	0.370752746
LOC649227	0.021300445	6.179036352	0.370945879
BANK1	0.024165815	6.125492786	0.371065773
FAM171B	-0.037369977	6.134420697	0.371135782
LOC645079	0.030210872	6.479111534	0.371294306
EIF5A2	0.023430656	6.560692417	0.371350459
LOC285500	0.021103649	6.020175831	0.371362105
CENPI	0.015461733	6.071710294	0.371381469
LOC641751	-0.020437662	5.990477629	0.371559295
DENND1C	-0.020948192	6.08731604	0.371647802
HOXA9	-0.01611801	6.011164127	0.371672983
LOC647579	0.022365355	6.113685801	0.371695018
TMEM38B	0.049255356	8.147176931	0.371778662
MUC17	0.02242471	6.0301607	0.37191984
FLJ42986	0.017560921	6.064742251	0.372085456
OR4F4	-0.015241955	6.018216271	0.372095419
LOC644326	0.020270923	6.054942863	0.372117066
SPANXN5	-0.021668885	5.930236166	0.372135056
SYS1	-0.035204338	7.469422212	0.372144615
C1ORF92	0.023713407	6.174786709	0.372245947
LOC644964	0.020491829	5.968033049	0.372261765
CHRNG	-0.023827087	6.068403736	0.372393551
ATP4A	-0.029532895	5.999130415	0.372418427
C15ORF20	0.036121293	5.944389078	0.372505652

LOC651809	0.02172248	6.046600007	0.372600553
ZNF253	0.023698817	5.994815163	0.372633284
KCNJ6	-0.022541183	6.090001553	0.37282258
PWWP2B	-0.033988981	7.158213322	0.372954297
ZMYND15	0.036176314	6.416315288	0.372984256
LOC147975	0.020195545	5.946539652	0.373015038
POU1F1	-0.019707038	5.849949733	0.373049282
IFITM5	-0.025059601	6.054875345	0.373092292
TMEM35	0.026903618	6.147930903	0.373213463
ZNF648	-0.024801742	5.977611307	0.373252687
RLTPR	-0.020229233	6.004190338	0.373416549
KIAA0329	-0.025890561	6.1813495	0.373522799
LOC652552	-0.020838056	6.126112579	0.373751842
CH25H	-0.075433823	6.429294542	0.373976374
DHRS8	-0.021556759	6.237227796	0.373991796
C6ORF136	-0.049654862	9.506861944	0.374005996
LOC647244	-0.029047786	5.920236395	0.374167458
GAB4	0.023066293	6.042827283	0.374206655
CREBBP	0.028396459	7.868772555	0.374233506
NFATC2	-0.016641773	5.933669527	0.374254454
KRT33B	-0.024015564	6.064129728	0.374288829
GP6	0.0193587	6.306898674	0.374400744
TRMT2B	-0.026301358	5.934463936	0.374422102
LOC653584	0.020726196	5.998064196	0.374429196
LOC641860	0.028573911	6.166487956	0.374442734
LOC345051	-0.019604009	6.063521822	0.37449676
MICA	-0.043104167	6.605977608	0.37451707
SOAT2	-0.022076724	6.070112408	0.37451891
LOC642580	-0.021273116	6.002818888	0.37489262
LRP11	0.033246658	7.093596704	0.374927285
LOC651553	0.02483078	6.043838582	0.374939207
LOC643900	-0.02178317	6.166544081	0.37496674
LOC388160	-0.018422963	6.080124891	0.375093045
LOC643233	0.034658481	5.971859573	0.375124802
LBX2	-0.022086178	6.064420734	0.375209926
C10ORF122	-0.021210376	6.138436092	0.375259945
LOC647191	0.026130911	6.152819131	0.375381524
ACACA	-0.03235841	7.698879526	0.375405968
NXF4	-0.017644262	6.061805646	0.375470884
CDK5R1	0.026852248	6.347137802	0.375535643
ZNF554	-0.02145304	6.018290674	0.375540005
INF2	0.03808286	7.226958239	0.375759303
TANK	0.03606146	6.871808868	0.375874838
FLJ25715	0.022262701	6.139287117	0.375964017
ANKRD1	0.197415538	11.75414562	0.376199009
ALPPL2	-0.026271056	6.208711972	0.376247115
LOC643721	0.022252883	5.93444604	0.376248073
PLCD1	0.028038065	6.668563415	0.376254252
UHRF2	0.033450355	6.823121647	0.376291905
INA	-0.022130004	6.028728491	0.376348744
LOC644302	-0.024227628	5.846503277	0.37637879

LOC650836	0.025855133	5.930679246	0.376400658
LOC651012	0.018125348	6.028513773	0.376458345
LOC644225	-0.021842879	6.029239347	0.376535693
IL28A	-0.02648975	6.062204928	0.376576474
LOC652294	0.024739879	6.116748804	0.376788173
LOC641714	-0.024744014	5.91525615	0.376808096
OBSCN	0.075262261	9.04910781	0.376831922
UBE2E2	0.039161021	9.864580236	0.376979691
LOC651965	-0.020321131	5.992389347	0.37700513
SSX6	0.016095619	6.007047676	0.377014981
LOC652134	0.025420596	6.362677995	0.377022194
LOC653240	-0.016575624	6.123003928	0.377092467
LOC649548	-0.044543237	12.7153119	0.377276743
MPP1	-0.049347554	7.659259879	0.377305177
LOC653817	0.024134038	5.935063093	0.37742094
TMEM137	-0.058791564	6.766131181	0.377562604
MYO6	0.025810348	6.441509933	0.37759382
FAM90A20	0.024449612	5.949504844	0.377614124
LOC645634	0.02317334	6.08615571	0.377644452
ZNF33B	-0.045719969	7.534241542	0.377670149
LOC643533	-0.020133964	6.040175662	0.377774749
LOC642425	-0.024336874	5.961482888	0.377790413
CPA2	0.020009501	6.010466388	0.377824143
FLJ41046	0.023740565	6.192470853	0.378024668
MXD3	-0.027171705	6.386667829	0.378203728
LOC442329	-0.023690043	6.248072787	0.378251889
LOC647648	-0.027088181	5.894171376	0.378309446
NHLRC1	0.022077169	5.985779465	0.378513364
SLC45A3	-0.033836348	6.614444055	0.378708712
LOC643594	-0.023235573	6.046748667	0.378735269
LOC642626	-0.021049843	6.037126762	0.378735819
LOC731052	-0.021994312	6.006251444	0.378742452
LOC644923	-0.027255661	6.035250035	0.378749017
TM4SF19	-0.012178652	6.016238197	0.378865491
TEX28	0.02534828	6.296150471	0.379166099
ITPR2	-0.025353879	6.600653295	0.379206758
LOC644351	-0.021981177	6.143454017	0.379261311
RALYL	0.063045411	6.485587632	0.379300004
PHYHIPL	0.02812633	6.066694278	0.379406095
ADCY8	0.025954513	6.037906513	0.379415874
LOC644126	-0.020850185	6.038002428	0.379627925
LOC652476	-0.020956581	5.93401307	0.379868549
HTR2A	-0.044724513	10.68946266	0.379941562
INSM2	-0.020982324	6.032312061	0.379968845
LOC648569	0.019834679	6.186080462	0.380034448
CSRP3	0.097889452	11.70563518	0.380123273
TRMT12	-0.035119627	7.811127296	0.380214704
FGD5	0.042877787	8.065181357	0.380295544
LOC644981	-0.023847207	5.997359248	0.380305154
VNN3	-0.013728261	6.023802333	0.380494062
LOC643624	-0.02089052	6.344189408	0.380508605

LOC645627	0.025005743	6.122594728	0.380587715
PTDSS2	-0.056204368	7.510524967	0.38067808
LOC339047	0.010644891	6.079960278	0.3807869
LOC642160	0.022431048	6.030111854	0.380809304
LOC642103	-0.028698489	5.948772561	0.380858649
LOC729370	-0.025919571	6.171365828	0.380861106
CTSE	-0.017531005	6.29210767	0.380884678
RBM	-0.024436724	5.963096287	0.380924453
KIAA0953	-0.021352383	6.148148683	0.381055945
LOC642049	-0.021139835	6.030959394	0.381121713
CLSTN3	-0.023057999	6.092412969	0.381364836
MAGI3	-0.021296563	6.394972047	0.381437281
TEP1	-0.022175465	6.02440201	0.381442748
C14ORF82	-0.042938411	6.881570099	0.381580551
DISP2	-0.025054868	6.227172194	0.381689
DONSON	0.022362915	6.679855937	0.381717552
SCG3	-0.015557024	6.076064178	0.381808771
LOC644785	0.025318596	6.011567829	0.381810789
LOC731049	-0.047565024	8.955769304	0.381975389
OR51A7	-0.021837572	6.099921995	0.38202966
LOC643492	0.021463587	6.199940624	0.382210498
LOC642268	-0.018112793	5.994397383	0.382231723
TMEM65	0.032327111	6.319759132	0.38244568
STYK1	-0.016344085	6.000293834	0.382457863
NPTN	0.055127447	10.43007068	0.382459346
TM4SF20	0.021080575	5.95020981	0.382526049
MGC40574	-0.020810644	5.930103615	0.382766887
LOC645891	-0.027824109	6.002022932	0.382795791
PTGR2	0.041333554	7.680953606	0.382851559
LOC646698	-0.023357017	5.999102612	0.383041749
LOC644359	-0.019699262	6.096570875	0.383049521
LOC730274	-0.023769692	6.050125288	0.383094553
AADACL4	-0.031007927	6.00924245	0.383174709
C11ORF76	-0.014754674	5.926021108	0.383239229
CDC26	0.056095363	10.48222374	0.383375302
LOC652491	-0.027432214	6.057595724	0.383426889
TMEM85	-0.033124583	10.33383177	0.383467623
FHL5	-0.044530126	6.599657114	0.383472662
LOC648050	0.025868646	5.942562511	0.383576956
MEPE	0.024693919	6.108157577	0.383619865
RDH12	-0.025554088	6.044703824	0.383692654
DKFZP686E2158	0.024407649	6.346315905	0.383694022
POLS	0.032273031	8.156340917	0.383697804
PCNXL3	-0.031992708	7.115292729	0.383730102
VAC14	-0.01665632	6.3142127	0.383790715
RRAGC	0.036002938	8.339258555	0.383848919
LOC728066	0.021335208	6.021402926	0.383898569
FABP1	0.019522666	5.945217362	0.383969025
PRSS23	0.067476791	7.525325402	0.383973893
LOC651196	0.022724875	6.110762988	0.384025109
LOC643236	-0.02042749	6.095377229	0.384049159

GYPB	0.021896299	5.894395515	0.384177994
LRDD	0.021025661	6.651527322	0.384181584
PNCK	-0.057878362	7.901043368	0.384441583
MARCH6	0.076961983	9.229619879	0.384471978
SIM1	0.014703706	6.058569601	0.384489396
KIF16B	0.027138388	7.113620207	0.38460488
LOC642198	0.02596532	6.003787386	0.384609576
REG1P	0.027413649	5.954973545	0.384727306
ITCH	-0.032877595	7.975181116	0.384779976
LOC643120	0.025266051	5.918515272	0.384795993
LOC643313	0.017803108	6.205556057	0.384821554
TBC1D3E	0.024375053	6.012380715	0.384848589
LOC646501	0.020600793	6.000732918	0.384908761
RPS20	-0.036947875	13.01558512	0.385125014
LOC642772	-0.021439755	6.118401067	0.385187278
LOC650788	0.036062757	5.972859744	0.385217918
TTC39A	-0.022760166	5.964446234	0.385245475
CYP2C19	-0.022946488	6.093197893	0.385296361
HOXD11	0.028702996	6.092834935	0.385345396
TIGD5	-0.046436273	8.233299885	0.385462142
SFTPA2	-0.020542148	6.102084749	0.385476809
DCLRE1B	-0.024362037	6.05312176	0.385514278
PRKACA	-0.02914783	6.485742382	0.385605935
LOC642295	0.017320442	6.328463146	0.385626634
LOC390547	-0.023488991	6.142071354	0.385630043
LOC648174	0.023587631	6.16706941	0.385757419
LOC647295	0.023522673	6.197045582	0.385767312
LOC439985	-0.021531441	6.052395739	0.385864279
TOPBP1	0.047464429	7.868804107	0.385932844
CHST8	0.019685713	6.046450241	0.385953948
FLJ35785	0.012646955	6.099078126	0.385996356
CREB3L1	-0.022653686	6.220947619	0.386034172
OR5AY1	-0.022006713	6.010719111	0.386058404
LOC652043	-0.020098886	6.045773203	0.386148701
FLJ38773	0.020657271	6.031554865	0.386168335
PYGO1	-0.022589447	6.038867197	0.386248549
LOC644056	-0.020897826	6.148751242	0.386342857
LOC644843	-0.021864646	5.944553792	0.386376243
SCN2A	-0.012851792	6.03101774	0.386438947
DGKH	0.024461236	6.117988362	0.38656481
GPATCH8	0.02873854	6.224665873	0.386569385
TSGA10	0.013617122	6.014280684	0.386587306
HAPLN3	-0.037502551	6.28384926	0.386611631
LOC149643	-0.024317254	6.038963099	0.38668499
RNASE12	-0.023220196	5.972701534	0.38670669
OR5H1	0.023683487	6.058015761	0.386738678
SLC15A1	-0.021636117	6.016796976	0.386858935
C1ORF131	0.033610564	8.130777285	0.386867543
LOC441792	-0.021906152	5.880828556	0.386940828
LOC441907	0.022099518	6.100690818	0.387015469
LOC653968	0.019652726	6.009736389	0.38713763

LOC649770	-0.02143172	5.947482547	0.387162037
LATS2	0.038027348	6.984183387	0.387163532
MKI67	0.023770206	6.095923995	0.387192371
LOC644408	0.021883103	5.93018002	0.387413956
LOC400927	0.024079815	6.070589701	0.387488958
TBX5	-0.021917626	6.234471299	0.387529779
TAS2R3	0.023212782	6.101540063	0.38758642
FAM130A1	-0.027337674	7.3008138	0.387631782
KIAA1984	-0.025871856	6.373407016	0.387633937
TAF4B	-0.024822724	6.188624054	0.387952932
LOC643630	-0.020697898	6.088543856	0.387964139
HIGD2BP	0.014169895	5.920703074	0.388205288
PBK	0.0249573	6.038493692	0.38821284
DEFB125	0.026914786	6.311916454	0.388348067
LOC653252	-0.020416716	6.05243748	0.38836566
LOC728499	-0.053365902	7.663175099	0.388386706
LOC390570	-0.02243172	5.983844299	0.388548814
LOC642980	0.018633882	6.208264703	0.388566469
TMC5	0.037405572	6.023986217	0.388570323
TMEM123	0.043298879	9.402105502	0.388651512
EVC2	0.025027609	6.362605939	0.388740631
LOC648069	0.025225633	6.204000049	0.388824015
GJD4	0.020095392	6.054460185	0.388827567
FA2H	0.020523866	5.983262443	0.388930916
LOC730995	0.042097376	6.391560293	0.389026882
TMEM105	0.023742662	6.129601462	0.389203621
PHLDB3	-0.031527073	6.047213911	0.389225834
C18ORF16	0.020338215	6.051734166	0.38934191
LOC123876	-0.022639003	5.928743341	0.389401687
LOC253573	0.026078069	6.117133303	0.389495001
KU-MEL-3	-0.022767946	5.972805747	0.389496371
LRRFIP1	-0.065611801	10.19268284	0.389505714
ALOXE3	-0.021234644	6.153091049	0.389623638
DKFZP686O1327	0.018063808	6.03241012	0.389626606
GTF2F1	0.034326855	7.923988123	0.38971818
CLDN8	0.018936263	6.010852625	0.389720488
LOC651476	-0.029653062	5.849575201	0.389832387
LOC649396	0.0259688	6.104567304	0.389850658
CA4	0.108331209	9.901964439	0.389864271
FLJ30375	-0.022276428	6.01262129	0.389957304
LOC649025	-0.019137673	6.172106323	0.389973238
LOC401010	0.020410843	6.449197135	0.389982672
GSTA1	-0.019168847	5.976163905	0.389986356
LOC643391	-0.025870356	6.015653298	0.39030027
LOC649749	-0.020680807	6.060375573	0.390323607
ANKK1	-0.01952291	5.922663943	0.390349986
RPS4X	0.051196385	11.02425986	0.39037034
NOS1	0.019857391	6.008857135	0.39038178
GPR88	-0.022444301	5.991024409	0.390462769
LOC652231	0.024158023	6.091695634	0.3905345
LOC650146	0.02381397	6.052661275	0.390576521

ABCC12	-0.026763352	6.530237456	0.390722538
OR9A4	-0.025887059	6.026222462	0.390750276
CDX4	0.027658693	5.955006937	0.390796846
PIGL	-0.022571457	6.265342041	0.390843906
BAGE	0.024754741	6.424262545	0.390850403
LOC646496	-0.019807794	5.998015717	0.390866881
SGOL2	-0.016676622	6.103412313	0.39088877
KIAA1166	-0.026330505	6.385093992	0.391047633
GBL	0.027985042	7.511015204	0.391054367
EMR1	0.022083269	5.989138425	0.391057618
KRTAP15-1	0.024742877	5.951232376	0.391142908
ZNF709	-0.021740945	6.041391072	0.391155102
LOC645201	-0.023802031	5.949506916	0.391202581
VPREB1	-0.018307856	6.070234114	0.391230974
C1ORF65	0.021823851	5.939186705	0.391353442
SNRPB2	0.026689292	9.771315602	0.391435903
LOC650850	0.023042491	6.116584692	0.391464732
SLC29A4	-0.034484902	6.509175574	0.391599586
LOC644422	0.044731578	6.972527583	0.391613356
C17ORF82	-0.024586302	6.164632218	0.391659168
LOC731000	0.013814496	6.087349167	0.391677022
DPF1	-0.023236416	6.196201093	0.391741745
OR5AS1	0.019208088	6.111325701	0.391952039
NCOA4	0.045208739	12.70009862	0.391999164
LOC650239	-0.013393061	5.947870395	0.392036195
C1GALT1C1	0.031792476	7.071505311	0.392048051
DLX6	0.02127758	6.003539256	0.392105166
IPO7	-0.057005671	6.906676428	0.39213639
GABRA5	-0.014628923	5.973617831	0.392213889
LRRC7	-0.021671709	5.926868104	0.392264742
DKFZP434I1020	-0.017149647	6.097939588	0.392275573
MAP3K4	-0.035477257	7.845674228	0.392323521
CCDC120	0.026601119	6.69517104	0.39242412
LOC651936	0.021732937	6.328060384	0.392424273
ZSCAN20	0.023785697	6.141188744	0.392470751
DENND4C	-0.032642958	8.16937798	0.39248907
OR5B12	0.019353432	6.052062274	0.392584635
LOC119358	0.021483505	6.141907451	0.392621945
MDS1	-0.030530816	6.058827916	0.392645224
LOC388965	-0.021580522	6.062457962	0.392711249
LOC642439	0.019450531	6.123438733	0.392732628
FIGF	0.052171923	6.394936317	0.392870384
STC2	-0.071602623	7.33597569	0.39296193
LOC646226	-0.026182274	5.938090036	0.39297879
SLC30A8	-0.020805875	6.080465313	0.392982527
CCDC33	-0.025391215	6.257753185	0.39299748
ZNF705A	0.019360231	5.982251774	0.393070119
TMTC3	0.030769901	6.501316024	0.393114359
LOC647971	-0.020361598	6.348313065	0.393155141
LOC387761	-0.022734756	5.96294864	0.393333848
DNAJB13	0.030210709	6.136631266	0.39336908

CRISP3	0.020767788	6.053066066	0.393434273
LOC728127	0.024075602	6.717409906	0.393489068
POU3F2	-0.015909134	6.049884577	0.393494688
C9ORF71	0.020311848	6.012827726	0.393496463
SHQ1	-0.035680499	6.933893309	0.393529201
GPR15	-0.021995533	6.012174094	0.393669946
CYP26C1	-0.025275336	6.03749971	0.393720682
GRTP1	-0.020475848	6.107848304	0.393734258
LOC650268	-0.02272696	6.129872002	0.39382399
ZNF382	-0.022585471	5.967433497	0.393845602
SMYD3	-0.032793485	7.556223849	0.39387143
FLJ33360	-0.023346375	5.988010969	0.39387644
LOC650238	0.021606467	5.927149377	0.39391096
INHBC	0.01927341	5.971624501	0.393916908
MAGI2	0.03096328	5.986298609	0.394186496
HMG4L	-0.02448789	6.074374211	0.394198952
PDZK1	0.013037747	6.00448857	0.394295003
LOC653680	-0.026765022	5.980509906	0.394313616
LOC647131	0.021915722	6.076763098	0.394322991
LOC650651	-0.021495044	6.081886681	0.394344256
LOC646037	0.0185457	6.093741313	0.39436148
ZNF563	-0.033922637	6.396626753	0.394564068
FLJ45537	0.017830043	6.021074666	0.394609075
LOC390856	0.018220059	6.073936951	0.394675028
HIC1	-0.024996742	6.491919283	0.394705052
LOC644571	-0.023283894	6.129984625	0.394779633
PHF7	0.012338132	6.143384965	0.394861009
HOXC10	-0.022711569	6.149899329	0.394881649
USP26	-0.030682625	6.592422219	0.395058828
EDAR	-0.019756301	5.988544342	0.395084361
LOC441869	-0.020234681	5.93779381	0.395092384
LOC652806	0.021019574	6.232426341	0.395152465
LOC731035	0.030002387	6.063304447	0.395170829
NOB1	-0.031517214	6.633235278	0.395224103
VEZF1	-0.036172014	10.77072434	0.395247688
UPK1A	0.02399297	6.207200257	0.395257424
TMEM16D	0.019763452	5.991740896	0.395333481
LOC728089	0.022199107	6.201127178	0.395333651
TRNT1	-0.02684829	6.733094048	0.395428093
LOC647591	0.022720879	6.052328088	0.395450026
PROKR1	0.023597474	5.975157314	0.395466288
LOC730804	-0.017479871	5.912110561	0.395665674
TNNT2	0.037905394	13.54784282	0.395745273
LOC649189	-0.019440292	6.405540222	0.395757056
DSG3	-0.019541105	5.996390549	0.395762608
MAGEB10	0.025206117	5.904654434	0.395805445
LOC728269	0.016203104	6.045453155	0.395848225
FLJ35934	0.023875733	6.184594803	0.395854284
OR2T8	0.029229814	5.952172426	0.396055188
LOC647832	0.013743447	6.037845223	0.396145624
CDYL2	-0.021307253	6.274822915	0.396180792

C1ORF94	-0.020840932	6.180642173	0.396228801
TAC1	0.013638676	6.005516826	0.396258752
UXS1	0.042233514	6.13174148	0.396284894
LOC653894	-0.01949619	5.969746526	0.396331392
PDCD4	-0.043005272	9.333593401	0.396372754
IL33	0.047830932	7.340974083	0.396414916
KRTHA5	0.019867708	6.012645114	0.396425171
AKT3	-0.012351568	6.126525313	0.396581209
C4ORF11	-0.027150713	6.00034135	0.396646872
CD2	-0.031087321	6.169115304	0.396844135
PFDN2	0.039373905	6.113014443	0.396849875
C20ORF141	-0.021566261	6.147253583	0.396959076
LOC652540	-0.020668984	5.934116047	0.396977115
GK5	0.031288088	6.852729383	0.397055103
LOC647911	0.029961495	5.953633986	0.397067173
TBX2	-0.063736497	7.696906175	0.397135861
C6ORF155	-0.022039888	6.130568358	0.397212522
HIST1H2AH	-0.021810545	5.969408439	0.397335757
LOC646029	-0.022509926	6.071393184	0.397355087
CDY1	-0.012884788	6.045913396	0.397471714
KRT34	-0.019961318	6.028821554	0.397635229
LOC645029	0.021781609	6.091710715	0.397656862
LOC651291	0.020493858	6.043081962	0.397666904
NUDT10	-0.016693938	5.938979541	0.397732085
UPK1B	0.028842299	5.966551802	0.397867471
PTMS	-0.060169389	8.51818146	0.397918765
ATR	-0.01307984	6.015010543	0.397968977
GSTM2	0.06923325	10.13437312	0.398028551
LOC647011	-0.02562883	6.121396578	0.39808837
WFDC13	-0.024505211	5.937633415	0.398117717
CYP2B7P1	-0.020144449	6.021774981	0.398182173
APOL5	0.022421506	6.130218811	0.398228051
LOC400163	-0.027293184	5.909256202	0.398243003
CINP	-0.032624217	6.725849266	0.398279872
FAM178B	0.020180955	6.197900132	0.398310291
LOC653412	-0.020073748	5.950941173	0.398332926
USP8	0.047641331	7.702913951	0.398525006
LOC643467	-0.019703805	6.093192077	0.398603892
ACSS2	-0.040954855	9.970631869	0.398642206
C17ORF45	0.048561647	11.44660153	0.398700729
LOC643210	-0.020419867	6.031702303	0.398712297
ASXL2	-0.037765475	8.736779973	0.398726088
BMP15	0.021955785	6.109574886	0.398735396
MGC25181	0.030242262	5.925980989	0.39874117
OR2F2	-0.020909951	6.074926569	0.398766014
NUDT16L1	0.070970139	8.309029056	0.398869616
LOC648490	0.023969375	6.412027337	0.398949787
LOC390282	-0.021510976	5.992480168	0.399035319
C3ORF41	-0.020675853	6.007525309	0.399084241
PABPC1L2B	-0.019604343	6.075104176	0.399087051
NUMB	-0.026892384	8.810555093	0.399173199

C9ORF25	0.027154379	7.014893101	0.399223816
TXNRD2	0.033363453	7.151205875	0.399260201
CRLF2	-0.01246093	6.088335702	0.399388209
LOC653047	0.015142709	6.135094246	0.399393259
KSR1	-0.022679261	6.142385313	0.399535874
DICER1	-0.023501436	7.120353338	0.399656033
COX7C	0.039266425	14.36993423	0.39966401
LOC648289	0.021629198	5.976283637	0.399682816
LOC92154	0.025062805	6.057519181	0.399705914
LOC441868	0.02234019	6.160842389	0.399784483
LOC643791	-0.020809022	6.016523263	0.399795738
ANAPC1	0.019802012	7.21912429	0.399841891
LOC440905	0.01544608	6.209439365	0.39991503
FLJ46347	-0.022085562	5.994605048	0.399924096
LOC400174	0.021973434	6.085840882	0.40006149
EXDL1	0.02518704	5.979462695	0.400100111
WDR33	-0.028725181	7.74287247	0.400164073
ESF1	0.024340968	5.981214201	0.400457698
LOC645781	0.016721196	6.022626217	0.400556469
FNTA	0.036366883	9.146673255	0.400695413
LOC389118	0.032822869	6.059915314	0.400704036
FATE1	-0.026965356	6.075966228	0.400791961
FLJ38723	-0.022504291	6.06494125	0.400844247
LOC440786	0.024305395	6.07230217	0.40084438
LOC643424	0.022080809	6.103417244	0.40085416
PRPF8	-0.051218786	10.02505915	0.400858133
CCDC99	-0.027831952	6.827993367	0.401175142
LOC652682	-0.023173179	6.098775584	0.401184682
LOC440131	0.020631147	6.122742044	0.401259228
C10ORF46	-0.02878078	6.208365278	0.401310094
C6ORF59	-0.024178581	6.164924899	0.401390681
LOC643598	0.020472419	6.059556495	0.401424159
LOC653039	-0.018757529	6.076829852	0.401455982
MRPS15	0.035377488	10.76402626	0.401470798
HIPK4	-0.020664158	6.038320741	0.401507781
LUZP2	-0.024083522	6.408407407	0.401521671
CSPG4LYP2	0.02024193	5.973190377	0.401683859
SLC35B3	0.032727186	7.544069139	0.401791536
ATG3	0.038749505	8.404872707	0.401822878
C12ORF54	-0.024490963	6.001838569	0.401909836
SYT14	-0.018879059	6.06382881	0.401963665
TXN2	0.047882885	6.364325047	0.402014585
RC3H1	0.035144284	5.960422901	0.402030171
LOC647651	-0.019412924	6.376601398	0.402065406
LOC646278	-0.022225065	6.063800233	0.402068889
ZCCHC24	-0.049565795	9.530349982	0.40207977
LOC645084	-0.028371206	6.009959377	0.40210845
LOC651062	0.018956292	6.002966691	0.402188655
LOC652224	-0.022737401	5.957773245	0.402238383
CTGLF3	0.047154493	9.230191356	0.402301133
DUSP5P	-0.015780841	5.987369194	0.402357145

SPOCD1	0.028455669	6.279109561	0.402414178
ELA3B	-0.021196641	6.164886371	0.40243122
LOC654128	-0.019770569	6.157364571	0.402480239
TCTN2	-0.023428495	5.982455141	0.402518272
IL4	-0.014941005	5.997291173	0.402550146
PHF13	-0.041281735	7.956350655	0.402765555
LOC653857	0.012994583	6.066265352	0.402880344
LOC729777	-0.022787112	6.006702676	0.402903331
LOC646012	0.016383907	6.219364322	0.402944759
LOC728763	0.018460507	6.002438872	0.403004209
LOC646452	-0.021247257	6.022081308	0.403044015
SPG20	0.028148255	7.202747181	0.403044333
DGCR14	0.025086342	6.673832837	0.403068781
CCNT2	-0.02068501	6.711195958	0.403135956
RUFY1	0.018119613	6.839449066	0.40321442
LOC731641	0.019158328	6.02706138	0.403245714
ANKRD34A	-0.023074542	6.486457131	0.403273089
LOC652715	0.018795662	5.949576489	0.403304696
SORCS1	-0.042369947	6.440029879	0.403320119
DEFB32	-0.016466298	5.97444985	0.403374161
LOC650556	-0.020751879	6.062598831	0.403384841
IER2	-0.039170103	6.263837179	0.403496611
LOC343927	0.023461843	5.924022523	0.403666312
SULT1A3	-0.023528026	6.939943029	0.403675757
LOC645465	-0.019726154	6.247562599	0.403794182
FAM105B	-0.036399563	7.692525088	0.40384715
LOC648362	-0.030588994	5.880661194	0.403958122
LOC644800	-0.022143056	6.034064216	0.404024933
LOC646708	-0.022061707	5.999939548	0.404050425
BDKRB2	-0.027803032	6.107861477	0.404140385
C6ORF129	-0.043159205	8.526197222	0.404199674
LOC400682	0.012981198	6.063649003	0.404296477
CTSL2	-0.026468338	6.330417883	0.404300945
KRTAP7-1	0.019197228	6.096806186	0.40439603
NSUN7	-0.015526413	6.124458812	0.404648321
LOC642797	0.020458153	5.980545639	0.404686166
SORD	-0.021549444	6.284560486	0.40472218
MSTO1	-0.048033464	7.401551903	0.404801495
MGC40069	-0.023043679	6.116310068	0.404861167
BBC3	0.026938036	6.06476696	0.404923168
TNFRSF12A	-0.135386379	9.623620957	0.404947008
CSTL1	-0.024306078	6.011888926	0.40496758
GLTPD2	-0.020092638	5.937970559	0.405053319
PPP4R2	-0.022691902	6.129788264	0.40505949
LOC643216	-0.028018372	6.022896933	0.405116605
AQP12A	0.024709333	6.266840126	0.405132452
LOC647704	-0.018676741	6.113279425	0.40523627
FAM127C	-0.032628456	6.748570796	0.405325044
LOC648489	0.017997254	6.056381406	0.405547065
LOC729254	-0.019985398	6.187819364	0.405591298
TDO2	-0.014932333	5.951190789	0.40560248

LOC644774	0.074444856	9.732370064	0.40580917
GALNT9	0.011605977	6.076825702	0.405882021
PSMA8	0.023014926	5.941563691	0.405938854
F13B	-0.020749097	5.935242514	0.405946172
LOC728760	-0.013842265	6.019596798	0.405951795
NLRP4	0.024023965	6.005024588	0.406044418
IGFL1	-0.022493527	6.054146141	0.406071449
MARCKS	0.055113456	10.06973204	0.406155949
LOC650580	0.040140259	6.727577805	0.406297691
LOC343052	0.032364667	6.070436899	0.40636282
FLJ12688	-0.022796285	6.182828115	0.406420006
LOC644781	0.019093633	6.146642927	0.40659162
CDH12	-0.020168622	5.936441494	0.406630985
MRP63	0.02818983	7.362244286	0.406643212
LY6H	-0.017402044	6.247765914	0.406698783
LOC644611	-0.027451469	6.102775819	0.40676453
PTRF	-0.075249845	11.42269205	0.406910576
LOC642504	0.019873329	5.935204264	0.406916751
LOC646300	-0.01527947	6.069628671	0.40692057
TRR1	0.019387639	5.999564502	0.406920928
PTGR1	-0.07001405	8.466613826	0.407027999
LOC644184	-0.020373239	5.985145387	0.407052807
LOC729003	-0.019467133	5.9394587	0.407089504
C10ORF129	-0.014797572	5.970216277	0.407094685
LOC649417	-0.022025382	6.332128676	0.407102402
HMX1	0.020680416	6.103342001	0.407168951
LOC646566	0.022321956	5.964484236	0.407353592
FLJ40244	0.023242822	5.847194442	0.40735842
UPB1	-0.036366436	6.498034618	0.407369366
PVRIG	-0.022416737	5.963806429	0.407371062
PLA2G10	0.023318308	6.0777653	0.407385687
CCDC150	0.020455617	6.037117075	0.407411169
LOC440607	0.020049781	6.076764014	0.407449195
KRTAP10-9	-0.014684688	6.199558375	0.407480425
FBF1	0.014932487	6.101823789	0.407596009
LOC654155	0.032542975	7.119530562	0.407746846
TBC1D29	-0.024862626	5.870855242	0.407822609
CDCP2	-0.021462742	5.99677709	0.407914779
LOC651746	0.02518557	5.937639826	0.407998542
IFLTD1	0.02164647	5.937010329	0.40805194
KIF18B	0.014785064	6.036032105	0.408223717
C5ORF15	-0.036990982	10.25413211	0.408278661
CHCHD2	-0.03949616	12.87858375	0.408292438
C20ORF3	-0.062765075	7.691575952	0.408302875
LOC644999	-0.020725524	6.136164557	0.408316114
GSTM1	0.062115554	9.722820777	0.408423136
CEP76	0.023113233	6.170764513	0.408514799
OR1D2	0.023233138	6.16046248	0.40856577
LOC642782	0.013344254	6.030449818	0.408605026
PCDHGB3	0.021052217	6.082878736	0.408638075
WDR75	0.031332276	8.964072381	0.408645059

TYR	-0.023790669	6.021156169	0.408696951
C6ORF174	0.023000067	5.976085187	0.408789382
LOC644844	0.024143284	6.447807276	0.408810538
ARPM2	-0.020561246	5.976814514	0.408812152
TPRXL	0.013610488	5.982306677	0.40894264
DNAJC12	0.047883891	7.268062815	0.408948547
GDAP1L1	-0.022479962	6.177953932	0.409013582
LOC128322	0.021581064	6.230371345	0.409113091
LOC642655	-0.023215876	6.004955188	0.409118111
EML5	0.014585748	6.042986007	0.409127867
LOC391509	-0.0298203	6.055196678	0.409213468
SARS2	0.063630044	7.692632433	0.409236002
FUT3	0.020770167	6.068267652	0.409543679
COCH	0.021210464	6.296612361	0.409696665
TRIB2	0.050185172	7.87768421	0.409719486
LOC651401	-0.018183533	6.084292019	0.409877349
LOC440498	0.039905779	6.295986124	0.409886835
WDR64	0.019825536	6.111425967	0.409895607
TTC6	-0.019938991	5.953338754	0.409951882
GRID2	-0.022835408	5.978316429	0.409999362
LOC650036	0.03344689	5.984374647	0.410029612
ZNF22	-0.028478565	8.982282128	0.410099803
OR52B6	0.021745497	6.216467333	0.41043624
AHRR	0.024019721	6.028576253	0.410456972
AIP	-0.041698561	8.779561318	0.410520067
AIPL1	-0.015474901	6.198811837	0.410542159
PANX3	0.022213785	6.23446032	0.410543646
LOC654023	-0.021164248	6.142579458	0.410626912
LOC645574	-0.030210801	6.264835429	0.410630111
C17ORF68	0.01921299	6.859011511	0.410633126
GP9	-0.021264869	5.922127613	0.410638865
FLJ13305	0.030973706	6.539832868	0.410657461
BHMT	0.021739441	5.86292694	0.410680832
CISH	0.068298173	6.982265891	0.410775649
C1ORF55	-0.039692793	7.805750007	0.41083558
PLCD4	0.022919008	6.129471791	0.410890231
CPT1A	-0.016092377	6.421901081	0.410893413
RP11-45B20.2	0.01702361	6.18240421	0.410904371
TMEM133	-0.034652372	6.143800196	0.411128723
RGS13	0.016349273	6.097409126	0.411173237
DRD5	0.019132437	5.995908694	0.411346526
FAM122C	-0.021231462	6.109221935	0.411481139
LOC652441	0.025454424	5.962603067	0.411551173
GABRG3	-0.020810868	6.03958352	0.411600929
LOC642588	0.020076473	5.961300579	0.411621403
UNC13D	-0.027044117	6.31052903	0.411634618
OR2T5	0.02182831	5.964150972	0.411641435
LOC642334	0.014311928	6.056057916	0.411658228
CTRB2	0.019662366	6.133418817	0.411703336
LOC643719	0.018803151	6.224352078	0.411776903
TMC3	-0.022843788	6.210196462	0.411867341

PPM1M	-0.040472301	7.999419665	0.411893892
LOC644366	-0.031902223	6.162205207	0.411917209
USP35	0.024391266	6.42548533	0.41197503
LOC648794	-0.019832671	6.017320278	0.412005497
KRTAP4-4	-0.020854948	5.978228539	0.412285271
TMEM34	0.030956631	6.90555999	0.412360259
JSRP1	-0.0287461	6.071344789	0.412411472
TIFAB	-0.015070528	6.048955533	0.412438253
C21ORF74	-0.01913025	6.048472078	0.412584713
FLJ34870	-0.017358035	6.066963275	0.412657412
GSTTP1	-0.022365297	5.960781069	0.412690639
FAM113B	-0.030768022	6.604296615	0.412723318
MTMR8	0.022454171	5.991913498	0.41283887
LOC441241	-0.025336635	6.592412774	0.413048741
KIAA0406	0.023002628	6.850193385	0.413084765
LOC389791	-0.022798389	6.237068658	0.413112767
RLBP1L2	-0.022034476	6.055578893	0.413121936
C8ORF17	0.020480605	5.991944286	0.413127837
C21ORF93	0.01854783	6.151306781	0.413242926
ARL13B	0.023683781	6.12336602	0.413315323
RPL32	-0.040599524	11.58525415	0.413365244
FLJ90680	-0.017587445	6.285178286	0.413368974
LOC643412	0.018285812	6.114028007	0.413409825
FLJ27465	0.016954408	6.192315206	0.41352587
DCST1	-0.024783767	6.137178221	0.413704143
LOC651974	0.021117082	6.026528429	0.413705002
LOC653830	-0.023100761	5.794740424	0.413773672
LOC652222	-0.026037566	6.036598061	0.413824803
TMEM63C	0.028764459	6.315765718	0.414068154
ACTA1	-0.061049568	14.72133392	0.414131145
ZNF395	-0.046653315	8.471660323	0.414163045
LOC651638	-0.022254649	5.948253373	0.41437342
OGFOD2	-0.023349949	6.315236164	0.414407246
LOC642456	0.024111729	6.318959085	0.414568495
IQCF2	0.021587813	6.048125401	0.414643931
C1ORF118	-0.027274917	6.04731628	0.414675979
LOC387720	-0.021484632	6.117816758	0.414724962
LOC652065	0.022532564	5.952878139	0.414791025
LUC7L2	-0.023529289	6.855854383	0.415043537
LOC643935	0.023549911	6.231609036	0.415056155
CD2AP	-0.032864836	6.616710102	0.415058938
UCN	0.025306595	6.38057404	0.415122561
EFNB3	0.037682386	6.287505599	0.415377287
PDE7B	-0.079815931	8.655518931	0.415386466
PCDHGA9	0.018233073	6.166837572	0.415405223
GLTSCR2	0.033755635	12.31216958	0.415443771
IFNA5	0.024459427	5.937423454	0.415445773
LOC650976	-0.021114073	6.108437806	0.415563501
C12ORF48	-0.02290866	6.447182294	0.415584601
ZCCHC4	0.022879184	6.292006839	0.415616291
JPH4	0.022249223	6.074791866	0.415759631

LXN	0.050678037	7.416114296	0.415810248
CD3G	-0.019559577	6.126415279	0.415877075
LOC653216	-0.01665648	6.031085045	0.41590784
LOC390332	0.022150827	6.046868287	0.41596727
ALG11	-0.036074491	6.569666366	0.415984723
LOC284371	0.024711633	6.202279831	0.416043973
CRIP1	0.080762643	9.561809013	0.416077127
LOC389199	-0.020330208	6.037640578	0.416111492
COX6A2	0.044399067	12.54205414	0.416114276
FRMPD2L1	-0.014972318	5.92624158	0.416208408
CBX1	-0.045722675	6.553540949	0.416254515
C3ORF25	-0.020622941	6.208251317	0.416296925
MEMO1	0.039389831	7.94213973	0.416369752
LOC651123	0.02769833	6.006496765	0.416431611
SLCO4C1	-0.022280855	6.065119404	0.41649277
MGC12760	-0.029016063	6.454588237	0.41651259
CHST11	0.027607879	6.008208885	0.416565758
CDADC1	-0.02056038	6.311570096	0.416727828
DNAH3	-0.020278628	6.054395263	0.416796871
LOC645964	0.022351015	5.917047371	0.41681976
WDR40C	-0.022864081	6.034551523	0.416865648
FCRL4	0.021702736	5.828849132	0.416887317
HCRTR1	0.021142961	6.040549269	0.41695917
OR2L1P	-0.015320088	5.957333068	0.417032449
DPRX	-0.029844049	6.153002514	0.417047628
LOC727815	0.01501466	6.065148356	0.417170794
LOC641901	0.021694569	6.024632408	0.417172436
XYLT2	-0.03146456	8.663732594	0.417280043
SPATA5	0.013675123	6.153255413	0.417318079
EIF1AY	0.406850301	8.797682912	0.417384055
SLC22A13	0.021156553	6.142384472	0.417617096
NRL	0.020200506	6.056046354	0.417713923
LOC646191	-0.025145704	6.047230391	0.417735001
LOC257358	0.02076226	6.033729179	0.417802638
LOC647993	-0.028407993	6.006984482	0.417811911
PRKY	0.066538247	6.343685738	0.417812068
OR6C70	0.020842213	5.926540205	0.417838232
C2ORF62	-0.022463196	6.0331482	0.417932404
FAM3A	0.045802795	9.908200237	0.417996874
LOC642351	0.020979049	6.138744539	0.418010113
TBK1	0.02987728	8.378742452	0.418105117
FLJ43692	-0.034218238	6.550466701	0.418117814
LOC652331	0.01887224	5.9641401	0.418131434
TTBK1	0.018775165	6.044894365	0.418164196
FLJ46363	0.019835564	6.206014052	0.418196422
CUL3	-0.024773955	6.200069559	0.418236732
FAM3B	-0.016490577	5.971968925	0.418244397
PKN1	0.045959986	6.972035374	0.418290261
IL17RE	-0.016212488	6.011729576	0.418361049
LOC651916	0.025499761	6.183613247	0.418361909
PRDM15	0.01815493	6.049219677	0.418487004

ST6GALNAC3	-0.041606902	6.695942722	0.418539878
LOC648245	0.017716203	6.13503257	0.418828647
LOC651647	-0.021273542	6.061332332	0.418844371
LOC643035	0.028591501	6.743701101	0.418857957
OR12D3	0.016893669	5.915441059	0.418942681
OR4S1	0.022333274	6.141211186	0.418960569
LOC642904	-0.021105424	6.086122298	0.418964164
LOC728145	0.016713248	6.086560962	0.418966544
LOC643883	0.021594991	6.033964157	0.418972235
ZNF300	-0.020997981	6.087591466	0.418991562
LOC651701	-0.020309363	6.054152929	0.419000286
CICK0721Q.1	0.019122236	6.114386438	0.419001582
LOC651055	0.019413572	6.054403472	0.419171775
ZNF530	-0.018515526	6.185564034	0.419277993
MIP	-0.028658162	5.82172763	0.41931606
OMP	0.021338676	5.893400668	0.419381905
MVK	0.017970712	6.640684681	0.419385835
LOC645974	-0.025369054	6.091050246	0.419389195
CACNG8	0.016255364	6.193763905	0.419556054
C11ORF66	0.020632878	6.227123967	0.419635277
HIST1H1D	-0.019449687	6.164060103	0.41966275
KIAA1545	0.045975309	7.766288618	0.419840714
FPGS	0.014857839	6.129640621	0.41987421
LOC652742	-0.020038165	6.011047848	0.420119498
TIMM17A	-0.024052582	6.631534732	0.420150154
CCNB1	0.022567336	6.114433194	0.420156825
LOC400388	0.019184937	6.214693181	0.420185561
FLJ42842	0.020740085	6.020806112	0.420280218
RPP38	-0.01891478	6.417854197	0.420313702
LOC650659	0.018613463	6.019890885	0.420381106
VHLL	-0.018039935	6.036512135	0.420430526
HIST1H3G	-0.022943323	6.248061332	0.420435003
LOC642864	-0.021182005	5.94079805	0.420446567
SYCP3	-0.014397143	6.159751413	0.420475596
WDR49	-0.013462365	6.123952947	0.4205266
SEMA6A	0.042624504	8.206992737	0.420732027
TALDO1	-0.029509496	9.815189344	0.420787105
LOC653773	0.05788453	10.97807919	0.420798499
LOC653715	0.020561993	6.155583712	0.420825114
LOC644638	-0.024354202	6.213426622	0.421043464
MED13L	-0.028761149	6.50821419	0.421048546
LOC647844	0.019795176	6.110773053	0.421086453
CCDC57	0.015274849	6.085749086	0.421294016
LOC642134	-0.020051764	6.04043868	0.421303604
P2RX3	0.014893343	6.03525445	0.421307399
LOC652437	0.023913319	5.964435554	0.42144297
ABCA6	-0.04356231	7.581030393	0.421471573
LHFPL5	-0.018902761	5.896929246	0.421536219
GBP3	-0.047744671	8.3396398	0.421611843
ADAMTS10	-0.02097385	6.140794251	0.421614077
CLIC5	0.090981682	9.972854564	0.421638752

ZBTB1	0.021691356	6.063349758	0.421658142
LRP2BP	-0.024144233	6.290963897	0.421660326
C9ORF62	0.028668876	6.567114086	0.421697081
NCR1	-0.025202514	6.011930011	0.421697664
CCNA2	0.024641935	6.287449807	0.421751853
SIRT2	-0.033784007	7.012576665	0.421853257
MMRN2	0.035112146	6.427718234	0.421923195
LOC641834	-0.017769582	6.245696027	0.421990446
WFS1	-0.066669263	10.49136118	0.422098822
LOC391004	-0.021154199	5.967302549	0.42212137
LOC652508	0.023138834	6.414742487	0.422166327
CSAG2	0.020457507	5.898649814	0.422170369
LOC643958	-0.020281162	6.025527289	0.422170828
SYT9	0.022346312	6.09338423	0.422196148
SLC16A11	-0.019784391	5.984058788	0.422217446
LOC651952	0.02019426	6.126356467	0.422234229
LOC732107	-0.019265846	6.111480057	0.422257983
LOC649635	-0.022568088	6.000395026	0.422322932
CERK	-0.03187219	9.593110613	0.422349401
LOC644295	0.025887209	6.186485593	0.422428608
LOC348021	0.022449752	6.140529773	0.422450318
FABP5L7	-0.021104634	6.097771501	0.422477299
TMEM61	-0.021520213	6.11439068	0.422521496
TREH	-0.022030313	6.142323011	0.422637765
TRIM40	0.02162376	6.018422118	0.422664147
LOC643325	-0.018790196	6.141612285	0.422665141
ENTPD8	0.012164175	5.989266277	0.422704581
HBG2	-0.051751173	6.172335624	0.422784465
LENG8	0.022552431	6.435013313	0.422902016
FLJ32682	0.017755548	6.010002279	0.422979017
NKX2-6	0.018697032	6.088721796	0.423028475
USP36	-0.034555404	6.607112888	0.423045503
LOC646381	0.021225703	6.212352591	0.423058191
ANKS1A	0.043121101	9.437545415	0.423069108
LOC729487	0.021107665	6.159938887	0.423107507
LOC647275	0.022438994	6.043053661	0.423125214
PRPF38B	0.02106549	6.661497971	0.42317589
RBM42	-0.037695311	8.84256236	0.423185051
C20ORF114	-0.020255806	6.07894976	0.423242383
LOC644404	0.023768881	6.091680297	0.423249327
TET2	0.021555995	6.078783944	0.423258769
ATXN7	0.02648156	5.991698147	0.423317459
ACSM4	0.01735685	6.008707032	0.423413968
LOC644912	-0.020168289	5.951156788	0.423450293
LOC391859	-0.02047231	6.208727809	0.423463079
NUBP2	0.029753942	6.956261994	0.423508632
LOC645721	-0.022761553	5.968590621	0.423605785
UBASH3A	-0.018265929	6.094414232	0.423620511
LOC652879	0.019247411	5.998117844	0.423625587
LOC642105	0.021033256	6.116407139	0.42369499
KCTD4	0.021735546	6.001328685	0.423753215

C10ORF61	-0.019873279	6.760046823	0.423810488
LOC650761	-0.016537781	6.032184388	0.423833076
LOC391037	-0.01739988	6.084638368	0.423846527
LOC728534	0.018801056	6.081514889	0.423851443
LOC653305	0.022338461	6.061807784	0.424087511
LOC643936	0.014373889	6.026137492	0.424144901
LOC652533	0.028083378	6.041918678	0.424199317
TSPAN2	-0.02064995	6.009534116	0.424207893
LOC653683	0.017822383	6.056895701	0.424318008
LOC650116	0.03472694	7.226234697	0.424370936
GDPD4	-0.019493461	6.257813612	0.42455003
LOC645451	0.021398625	5.939237672	0.424550893
ST3GAL5	-0.028160208	6.631070346	0.424575917
LAMC1	0.032089546	11.71606562	0.424739834
RDH14	0.036558609	10.74399362	0.424888817
HDAC7A	0.024850549	6.404356029	0.424978339
LOC648274	-0.020185327	5.951784784	0.425007305
LOC440268	-0.018903095	6.023160385	0.425051022
THSD7A	0.027997744	6.102464548	0.4250762
SH2D2A	-0.023398529	6.290950518	0.425197869
LOC401914	-0.019779796	6.174182655	0.425303591
PMP22CD	0.019382591	6.131048196	0.425319081
RPS6KA3	0.017878202	6.519141383	0.425356734
SLA2	-0.014504223	6.026931304	0.425363495
LOC729919	-0.021002758	6.080726157	0.425372729
LOC649896	0.023882756	5.962908329	0.42541609
RBM43	0.020810062	6.079746631	0.425450708
LOC643748	0.02150827	5.975846388	0.42556241
FLJ44477	0.021114472	6.263850722	0.425587354
C6ORF52	0.024051227	6.330033759	0.425625119
LOC649578	-0.021677566	5.987664712	0.425664695
ZNF567	-0.01577895	6.194870682	0.425693131
LOC652628	-0.022361012	6.373892675	0.425705703
SLITRK1	-0.016457374	6.040523254	0.425774888
C9ORF36	0.015134505	6.018445821	0.425778477
LOC646537	-0.026237148	6.042181649	0.425888685
DULLARD	0.051549774	7.329894181	0.425961053
DDI2	-0.013749832	6.126271917	0.425989096
LOC643055	0.01758091	5.936361615	0.42611732
RD3	0.029470578	6.100593562	0.426153383
RSRC1	0.036274716	7.203098399	0.426297355
HMX2	-0.02128261	6.128568615	0.426355019
FYTTD1	-0.048829908	8.390745379	0.426492252
LOC730340	-0.018491533	6.095329287	0.426642835
LDHAL6A	0.023255304	6.261586338	0.426661507
EPB41L4A	0.029735251	6.917960229	0.426711828
LOC652792	0.022584662	6.082104586	0.426718959
LOC642681	0.018934245	6.023711784	0.42680498
FFAR3	-0.021003078	6.303910959	0.426807825
OR4C12	0.015057887	6.071229569	0.426813491
LOC730833	0.026857538	5.99811653	0.42682355

LOC647666	0.017705863	5.933494472	0.42683083
OCM	0.020929063	5.984837768	0.426883385
DPYSL2	-0.036196893	11.34532398	0.426892198
EVPL	0.018876017	6.084009218	0.42702799
LOC649397	0.017559076	6.316106496	0.427249904
LOC400221	-0.022618578	5.918574509	0.427593676
GPR39	0.020243796	6.024044804	0.427622173
LOC651896	0.01953715	6.052676775	0.427649928
GGT7	0.030169341	6.40007359	0.427784744
C9ORF110	-0.033074149	6.046114129	0.427889341
NUF2	-0.014791064	6.001233064	0.427961322
LOC644690	-0.019107657	5.967550868	0.42796852
LOC392467	0.016628771	6.058160743	0.427969733
LOC644607	-0.024805356	6.203075103	0.427981583
LOC642340	-0.025619063	6.017479399	0.427986021
LOC649443	-0.020553148	6.102048461	0.428167734
FAM26A	-0.019968772	5.933079345	0.428200039
D21S2056E	0.029542938	5.946787215	0.428259612
C1ORF111	0.020666299	6.12073402	0.428260461
ZNF780A	0.019948802	6.090819689	0.428320713
FLJ44815	0.021625665	6.113299513	0.428342189
C20ORF123	0.027682055	5.94895895	0.428456841
TNFSF18	0.021512738	6.100103903	0.428526668
LOC440013	0.012108977	6.02838608	0.428573656
LOC646604	-0.020025684	6.137609916	0.42860176
LOC729355	0.020414566	6.28143587	0.428699063
AFF2	0.021864175	6.174273219	0.428763541
MS4A10	-0.020144725	5.919326239	0.428791751
LOC651760	-0.01680177	5.972748414	0.428812204
FOXR1	-0.010947853	6.015187243	0.428932013
TMEM127	-0.031019038	7.225019416	0.428952141
LOC652448	-0.018158808	6.053488185	0.42899145
ZNF44	0.019006015	6.029908689	0.429045115
CCDC38	0.01915895	6.034287886	0.429074994
LOC401923	0.01952517	6.00219595	0.429108371
LOC650459	0.019648756	5.946672466	0.429199745
ACRV1	-0.015741919	6.136311302	0.429239941
LOC653582	-0.019506076	6.097980918	0.429309063
ZNF286A	0.025030336	6.294239909	0.429367394
FOXF1	-0.028514064	6.072621469	0.429442132
LOC150577	0.027467614	6.041664119	0.4296589
KRT4	-0.020961832	5.995241113	0.429977063
FLJ34047	-0.031340858	6.174335499	0.430287993
LOC731283	-0.021675261	5.918064159	0.430399084
MAGOHB	-0.025263731	6.051178899	0.430506624
LOC653380	0.021347769	6.072790794	0.43061259
ESRRG	-0.042532673	7.944496072	0.430612711
ARHGAP27	-0.02894406	6.139244112	0.430637833
C20ORF118	-0.018385799	6.210209337	0.430640764
LOC648438	0.016894759	6.044973563	0.430642008
IL28RA	-0.031101101	6.591643323	0.430647824

VSTM2	0.021301057	6.094185852	0.430677592
GTF2A1L	0.012815178	6.097023489	0.430717474
TRAF1	0.016337732	6.392404981	0.430778518
UBE2NL	-0.022053009	5.939979763	0.430832411
RPH3AL	-0.035516583	6.474870116	0.430875292
SAMD4B	-0.037969137	6.922551479	0.430882599
LOC650180	0.020550281	6.057519223	0.431148109
LOC643203	-0.023168812	5.8854851	0.431324931
PTH	0.01663261	5.929875338	0.431346146
VSX1	0.011375421	6.01393606	0.431490537
LOC645306	-0.028200469	6.04295845	0.431492929
MAGEB18	0.02319332	6.068018244	0.43149372
PIP5KL1	0.024685282	6.211857716	0.431637816
SLC26A7	-0.01535049	6.048413062	0.431662143
LOC649073	-0.017540265	6.120338206	0.431703005
GPRC5B	0.031725948	6.996917748	0.431720019
KIAA2026	-0.025051758	7.188869533	0.431885026
GNG2	0.029111338	6.677829875	0.431912022
KRT20	-0.013062172	6.073399525	0.431952218
DCI	0.05428085	10.04115946	0.432079308
MEGF10	0.016078572	6.341314433	0.432082865
C15ORF51	-0.018720149	5.990017096	0.432111818
C12ORF67	0.023442949	6.276545226	0.432151087
FLJ45964	-0.02334357	5.999430327	0.432157151
DIAPH2	0.023635756	7.113627331	0.432199101
LSM6	-0.038889052	6.515511416	0.432203084
LOC647156	0.020311809	6.025221071	0.432315659
LOC643563	0.017562719	6.058753654	0.432441704
PCNXL2	0.013191529	6.119920121	0.432458125
LOC651293	-0.023437125	5.932725149	0.432464088
LOC648855	0.018488678	6.018448052	0.432474263
LOC653750	0.021719157	6.213371199	0.432543187
DNM1	-0.032327327	6.162837476	0.43254776
MDFI	0.036185007	5.934550837	0.432609517
LOC727735	-0.010910186	6.052030815	0.4327315
CDH10	0.021510386	5.944017683	0.432748063
OR52A5	0.017498515	6.583344965	0.432749938
UBE2R2	0.033342225	6.29337463	0.432964922
LOC646864	-0.017228675	6.055060013	0.433063675
LARS	0.030043357	9.460552916	0.433073891
GALNAC4S-6ST	-0.055654289	7.270002135	0.433087063
LOC641785	-0.018887027	6.019963058	0.433170018
ARL8A	0.027262791	6.111933542	0.433259497
LOC646011	-0.019096251	5.966423512	0.433341497
BRAF	0.023990874	6.354679054	0.433341507
LOC643261	0.018920276	5.920304779	0.433346448
LOC220416	0.020435484	6.039172208	0.433447063
MPZL3	0.025991353	6.18966106	0.433570832
FCHSD2	-0.039856419	8.400204814	0.433582778
MMP17	0.011340684	6.055510015	0.433613574
HTR4	0.012161128	6.156562829	0.433653718

LOC647726	-0.019289694	6.17696402	0.433708414
AKAP10	0.024879002	6.57695743	0.433876148
LOC647480	-0.02010871	6.000176595	0.433963371
LOC650468	-0.021291832	5.941105535	0.433972765
LOC648732	0.017263943	6.109203001	0.434099882
LOC649856	-0.01877647	6.023918519	0.434156172
LOC650922	-0.029739123	5.847182482	0.434165575
FGF8	0.014245208	6.033300318	0.434186755
DNAHL1	-0.026307099	5.872737915	0.434236905
IL31RA	0.020494686	6.231144179	0.434242195
SLC39A12	-0.019335617	6.08426438	0.434346974
TIFA	0.028393915	6.56447939	0.434365205
NLGN4X	0.039726808	6.872220304	0.434381728
NAF1	-0.025899404	6.549267834	0.434501921
PYGO2	0.028904015	6.453047868	0.434670994
RSL1D1	-0.041105808	9.585039154	0.43479986
SERPINB13	-0.024107556	5.890206669	0.434883087
LOC642014	0.018841101	5.945349526	0.434969727
LOC169834	0.018699857	5.96134655	0.43504616
KCNK18	0.020103668	6.199971267	0.435053608
OR5F1	-0.015183093	6.075350825	0.435083585
SLC18A2	0.017059155	5.974509389	0.435106792
LOC729973	-0.017758055	5.975807527	0.435169372
LOC648556	0.017821785	6.136564049	0.435178171
C8ORF30A	-0.012457014	6.104544367	0.435221595
LOC650200	0.021133448	6.074235615	0.43524563
GRAP2	-0.022433322	5.88286512	0.435321188
PKHD1L1	-0.025713129	6.236873814	0.435433471
KLK14	-0.019237821	6.03071093	0.43553443
LOC643194	-0.020375812	5.939240622	0.435546396
MUC6	-0.012347311	6.160903303	0.435601932
ANKRD20B	0.013374117	5.939579367	0.435664432
LOC390483	0.017734361	6.128058672	0.435816754
LOC642366	0.025917742	5.995732182	0.435903385
LOC389031	0.029517472	6.24829651	0.435911457
ZBTB8	0.027736454	6.549727354	0.43595032
LOC654096	0.031271035	6.161100284	0.436052708
PTPN9	-0.015848341	6.318111849	0.436192285
LOC643441	-0.0121837	6.048793418	0.436227186
LOC643142	-0.018251605	5.95276292	0.436287681
CDK5R2	0.020028689	6.156936731	0.436339903
JARID1A	-0.033272231	7.316351254	0.436355165
EXOSC8	0.032955173	8.848200686	0.436367054
TTTY13	0.01488276	5.948043651	0.436385677
LOC652191	-0.022515391	6.047524751	0.436462449
LOC651576	0.017602679	6.086198936	0.436465426
LOC651959	-0.019973088	6.28351396	0.43648005
PRSS12	0.023300341	6.115881191	0.436513706
PRR17	-0.018292805	6.003666921	0.436577554
LOC644105	-0.012992328	6.153965747	0.436626962
CDC2L5	0.025543503	7.736326429	0.436697483

LOC646552	0.034828679	6.006025723	0.436734987
NDRG4	0.050767169	10.93763583	0.436741715
FNDC3A	-0.052181429	7.47987708	0.436855436
LOC650029	-0.029610804	7.560740403	0.436912728
P2RX6P	0.022363515	6.136365762	0.436937955
LOC642033	-0.040393735	7.129960218	0.437050972
GLP1R	-0.022073897	6.072765296	0.437056479
IL1F9	0.019411	6.020955528	0.437171919
HOXA11	0.022598478	6.049699551	0.437173924
LOC653234	-0.015112072	6.222924838	0.437212972
AGR2	0.027728534	6.063415835	0.43726175
ADAD1	-0.019653025	5.870881768	0.437322248
DMGDH	0.015472721	6.121858943	0.437351737
C14ORF1	0.034305358	6.053038476	0.437358526
LOC650285	0.021011195	5.996874466	0.437513209
LRRC8E	-0.020344328	6.234618287	0.437519902
ZNF663	0.020546166	6.121376266	0.437561411
LOC340549	0.026525082	6.178195684	0.437633807
CLDN3	0.024329833	5.986807203	0.437641128
ADCY5	0.057307883	7.663239803	0.437716973
C10ORF72	0.022387465	6.363915217	0.437719783
LOC338328	-0.018425687	6.003784356	0.437767385
FAM22F	0.017631038	6.35609964	0.437846927
CRIM2	0.023375557	5.89656027	0.437946012
MOBKL1A	0.042875078	6.307739785	0.438005062
WFDC5	0.015350332	6.111595476	0.438012425
SPATA17	-0.024800964	5.968675089	0.438170365
PCDHGA3	0.014376797	6.186953464	0.438272124
LOC389124	-0.017863956	6.448052837	0.438323649
SEC23A	-0.031832392	6.86063538	0.438349884
C19ORF57	0.014859612	6.204105203	0.438357397
IL2RB	0.039615534	6.20699853	0.438368119
KIAA0738	0.021405042	6.094276989	0.438432979
LOC653261	0.019483603	6.089444743	0.438443068
OR4N5	-0.017627564	6.011724813	0.438576486
LOC645671	-0.021669237	6.279606993	0.438613675
LOC642361	0.025683255	6.930320229	0.43865022
ONECUT3	0.020056931	5.997117317	0.43865296
DKFZP779B1634	-0.019429794	6.014130435	0.438713111
CXORF61	0.020513446	6.055051242	0.438774126
CEP170L	-0.011098274	5.943453608	0.438782356
SLC26A2	-0.029591228	6.811976731	0.438999738
LOC654085	-0.024605984	5.907858695	0.439132199
SH3MD4	0.019559528	5.984979273	0.439245049
DMRTB1	-0.019925323	6.114267724	0.439265722
GP2	0.02728878	6.055124656	0.439415743
SNTG2	-0.022625134	6.137165162	0.439422482
TRIP10	-0.052367793	8.203181347	0.439578972
LOC653051	-0.018571562	6.090860728	0.439627181
LOC648189	0.019927526	6.296270561	0.439655035
FLJ42418	0.017697045	6.166704695	0.439663098

ADAM29	0.023821373	6.134104006	0.439756481
LOC728734	0.033912449	7.50912674	0.439845175
DMBT1	0.01463862	6.070185047	0.440024973
INOC1	-0.026437031	6.935426827	0.440034567
C10ORF31	0.019300897	6.154753466	0.440060263
LIPE	0.037934691	6.948307599	0.440099264
LOC652843	0.018277908	6.171088828	0.440148169
SYNE1	-0.012509907	6.189715192	0.440280356
PDC	-0.012078879	6.082962288	0.440289649
LOC643275	-0.011645181	6.036654176	0.440300548
LOC642120	-0.019684322	6.086309755	0.440310042
PCDHB14	-0.021789845	6.354720705	0.440373116
LOC652226	0.020246908	6.287689742	0.440379092
SSSCA1	-0.030454104	7.119147289	0.440552206
CHTF18	0.024949811	6.363712382	0.440710189
KLHL15	-0.023907804	6.071104785	0.440858104
TPRG1	-0.019031301	6.055845047	0.440884521
LOC645088	-0.018579088	6.026884774	0.440971649
TMPO	0.01351824	6.147646273	0.44099767
C2ORF78	0.020548694	6.127077018	0.441045084
LOC654208	-0.017384937	5.980754738	0.44111328
MED26	-0.021305486	6.512490926	0.441119301
LOC649486	0.018996895	6.091001763	0.441322913
ING1	0.01345646	6.19951366	0.441496617
WDTC1	0.033151935	6.022358868	0.441496933
IGF2	0.026542247	6.48549774	0.441519203
MAPK11	-0.016549794	6.247242696	0.44162699
PIH1D2	0.019534094	6.018852004	0.441761041
LOC644133	-0.019984154	6.105345958	0.441909364
C6ORF107	-0.018628428	6.246212251	0.441963869
LOC342979	0.016302625	5.910514067	0.44200808
NOM1	-0.01661329	6.044356453	0.442042565
LOC651621	-0.020132143	6.276013403	0.442099055
LOC653937	-0.022010169	5.99226563	0.442100731
LEFTY1	-0.019567092	6.028995063	0.442152234
LOC727944	0.028309374	6.016440524	0.442265546
LAGE3	0.032787137	9.546502918	0.442328562
LOC645789	0.01746868	5.897365661	0.442546717
LOC641767	-0.019332295	6.085675986	0.44265931
FREM2	-0.014927318	6.153773691	0.442796058
TNXB	-0.015999857	6.133636451	0.443165973
ZNF622	-0.061068113	10.74340002	0.443245555
LOC643357	0.054276418	10.3212299	0.443265513
LOC399753	0.010965818	6.032865494	0.443484004
LOC651427	-0.019092593	6.037348615	0.443539363
LOC440157	-0.03667552	6.978341359	0.44357669
LOC648343	0.071037534	7.677307641	0.443637544
ELAVL1	-0.029849955	6.824976095	0.44368603
LOC649125	0.016888074	6.07302566	0.443701434
LOC642140	-0.021307105	5.957680795	0.443727643
PPP2R5E	0.041267259	9.574029453	0.44390914

LOC642119	0.016828943	6.008644781	0.443995449
SLC12A3	-0.018433863	5.895913087	0.444058782
LOC645745	0.017490868	6.094161029	0.444087507
DPY19L2P2	0.016159393	6.19177657	0.444089212
SP1	-0.028466637	7.776358635	0.444098907
CEP164	-0.027461356	6.71464375	0.444101963
DUX1	0.01976485	6.157674044	0.444168739
LOC647407	0.019322463	6.014976592	0.444440817
CANX	0.036891983	9.19427584	0.444543076
SIRT6	0.024123865	6.217380881	0.444564816
TFEC	-0.013374138	6.063511746	0.444658411
SUNC1	-0.017322162	6.326038211	0.444666675
PCDHB6	0.014772593	6.018762322	0.444722857
GPR77	-0.019863218	5.898202181	0.444790705
LOC648897	0.028445465	6.022828032	0.444877222
LOC642849	0.016411614	6.025063396	0.444936209
LOC344875	0.020074559	6.082269767	0.445030701
CENTD3	-0.038790065	9.429596076	0.445096404
DLEC1	0.015467952	6.181501949	0.445118165
FLJ20581	0.0150834	6.10025465	0.445131314
AGC1	-0.021724074	6.251253073	0.445131573
FAM47C	-0.015486069	6.121805426	0.445355665
LOC389120	0.01813942	6.032501505	0.445509897
USH1C	0.016044084	5.953041291	0.445562059
POU3F4	0.020616738	6.26514717	0.445575368
LOC286094	0.019785401	5.951841374	0.445740268
C13ORF35	-0.018240598	5.966077897	0.445759483
FOXN3	0.016772831	6.186191414	0.445782406
ZNF829	-0.010995726	6.072359879	0.445867816
C5ORF21	0.031354218	9.399414233	0.445897704
GAGE5	0.040622388	6.076357658	0.445994028
LRRN2	0.024094198	6.635501675	0.446071945
LOC641522	0.021516702	6.370857501	0.446101246
ERC2	0.022341436	6.121240428	0.446169468
SNX2	0.034901043	8.653757598	0.446262204
LOC644524	-0.019758159	6.034628391	0.446266961
GEMIN7	0.016319585	6.04443947	0.446576733
LOC648659	0.040952081	6.588256036	0.446699017
SEPT14	0.016760024	6.015150863	0.446715664
OPN5	0.014829243	6.093313997	0.446940288
GCM2	0.018697797	6.037883543	0.446964648
GPR179	0.019806947	6.230994063	0.446980846
SWAP70	0.040504802	9.072144644	0.447002074
SPINK8	0.015837761	5.935436875	0.447068293
CDC25A	0.016487189	6.146430941	0.447152718
LOC648751	0.018001918	6.115441069	0.44715788
LOC400578	0.014447233	6.014176828	0.44719622
SPATA16	-0.023486426	5.942047738	0.4473291
C1ORF201	0.026876616	6.090915362	0.447679673
LOC643397	0.020078075	6.086303387	0.447745663
LOC387683	-0.020408078	6.357805189	0.447766058

SNORD30	0.023899746	6.229792151	0.447777287
TRHDE	-0.018050358	5.962209549	0.447801122
SSX2	-0.009413483	6.063956924	0.447810826
TXNDC8	-0.017991727	6.038032934	0.447831148
WDR52	-0.019350709	6.522603071	0.447914625
OR8H1	-0.012718542	6.062926723	0.44802065
LOC285735	0.019122793	6.018880146	0.448116694
C19ORF29	-0.018016552	6.664362117	0.448131253
LOC440407	0.018445426	6.056492456	0.448149163
LOC647742	-0.018462461	6.124504315	0.448201334
LOC649965	-0.019118681	6.000256836	0.448230795
HSD3B2	-0.019747663	6.125654454	0.448300023
LOC652324	-0.023843169	6.59786119	0.448331499
MLLT3	-0.024030567	6.237664841	0.448334354
LOC645287	-0.019750985	5.896277535	0.448349156
LOC644944	0.017340711	6.004147751	0.448354999
LOC642740	0.023255391	6.236923538	0.448637339
LOC645682	-0.019125399	5.970989897	0.448653775
C1ORF67	-0.020046199	6.087498559	0.448740945
KIAA0146	-0.026602854	6.704835806	0.448883447
HTN3	0.015814305	6.158610413	0.448889279
MARCH4	0.019902211	5.984888425	0.448906096
PTER	-0.032811327	6.349327964	0.448945018
EGR3	-0.022773121	6.53324252	0.448998651
CHCHD9	-0.036116702	11.20995947	0.449064463
LOC644224	-0.019727421	6.113980704	0.449144046
LOC647891	-0.016063829	6.179599592	0.449334345
MAP9	0.028891949	6.304594416	0.44941728
CTAG2	-0.027912038	6.093693285	0.449502981
RAVER2	0.044760567	8.429182534	0.449503957
ARL9	0.020835949	6.042018995	0.449517681
KIAA0174	0.025372781	10.3504067	0.449588373
TACSTD1	-0.028033581	6.257525731	0.449630734
KRTAP11-1	-0.018978868	6.022395047	0.449706177
RNF138P1	-0.019618788	6.018906896	0.449730186
OR11G2	-0.02067294	6.150072321	0.449817718
FAM20B	-0.065008577	8.947986781	0.449862326
LOC648481	0.020837895	6.049763957	0.449868424
STK31	-0.010439084	6.060073961	0.449881235
KLHDC7B	-0.020386366	5.956321501	0.449889033
FLJ90036	-0.020231815	6.02853049	0.449914663
CACNA1F	-0.0195946	5.997602526	0.449938684
LOC648054	-0.018772093	6.019168229	0.450150382
DKFZP564C196	-0.019278572	6.190145546	0.450188896
LOC652139	-0.01741292	5.8751352	0.450336188
IRGC	0.019097784	5.997960632	0.450481084
APOBEC4	0.017295471	5.924130918	0.45054796
LOC729260	-0.018122599	6.116527754	0.450585499
ID4	0.028170378	6.043828415	0.450620377
BOLL	-0.009590924	5.999820689	0.450634082
LOC729476	0.026241867	5.915161825	0.450687273

FLJ37673	-0.019710716	6.062240239	0.450715997
LOC255187	0.016737551	5.986505465	0.450745144
LOC120824	-0.019325855	6.047885925	0.450805138
SLC39A2	-0.018584012	6.014343253	0.450851507
FLJ36116	0.018920781	6.158563475	0.450956454
TRPM3	-0.011953746	6.068685977	0.450961202
TOP1P1	-0.015621024	5.963157434	0.451202953
LOC649452	0.020888077	6.064067744	0.451299143
ABI1	0.039632621	6.934380531	0.451314829
LOC651454	-0.017957694	6.09090555	0.451408579
LOC644689	-0.025487407	6.160163063	0.451604211
PEX14	-0.026350474	7.15854475	0.451658741
LOC648668	-0.019152223	5.994754437	0.451699206
SGK3	0.020472628	7.070455476	0.451726194
LOC650638	0.02257753	6.098475937	0.451824904
PZP	0.027035239	6.633754686	0.451901432
AHCYL2	0.023508957	6.685873548	0.451987776
LOC651886	0.020303499	6.032067289	0.452055946
LOC441378	0.0219938	6.165957514	0.452148864
LOC653219	-0.017378009	6.230663746	0.452183651
ZIC4	0.018097711	6.057440681	0.452187874
LOC652679	-0.034084303	5.901228886	0.452190491
LOC731496	-0.019889832	6.175188275	0.452217186
OR10G3	0.022562058	6.077731296	0.45225636
LOC645995	0.021464108	6.010866381	0.452269844
C3ORF46	0.018497787	6.267024379	0.45227795
NLRP10	0.016474085	6.244788757	0.452420758
ZNF509	0.027818975	6.636677955	0.452513226
C14ORF178	0.016221754	5.894693168	0.452553203
PDF	-0.015222694	6.147137279	0.452597046
MGC10997	0.034136822	9.766660621	0.452598749
LOC284441	-0.019849229	6.109629167	0.452678451
GUCA2B	-0.019618987	6.108725069	0.452706539
LOC650823	0.019671065	6.145436974	0.452710879
DSP	0.046392444	9.093478034	0.452818373
LOC644592	-0.02074839	6.0597838	0.452848975
MTDH	-0.039079428	8.533927475	0.452926174
LOC440082	0.016782967	6.117865318	0.453001871
CTAG1B	0.017437815	6.094879314	0.453103648
CADM2	-0.02890563	6.120542519	0.453185131
RCOR1	0.021315202	5.971264307	0.453248646
WFDC10B	-0.015568844	6.133846898	0.45329454
LARP2	-0.031466043	7.519415663	0.453312637
IGHMBP2	0.02085504	6.667510886	0.453373786
ZPBP	-0.019711461	5.997644829	0.45345368
LOC647509	-0.021037871	6.02580714	0.453594759
PRMT8	-0.018708825	6.101159067	0.453596895
MCART6	0.014196121	6.12904978	0.453660316
UTP3	0.039752358	7.334960122	0.453773058
LOC347411	-0.020255384	6.059576979	0.453778598
LOC653555	0.018754738	5.997623212	0.453807029

LOC653562	0.01717359	6.029442798	0.454025167
RNF12	-0.013168446	5.979300878	0.454034615
LOC402715	-0.018047988	6.035780854	0.454057065
ZNF304	0.041565212	6.351069088	0.454073822
ZNF470	0.021013923	6.364808377	0.454195227
LOC149950	0.022039023	6.449389577	0.454202288
DPRXP4	-0.013352223	6.112950248	0.454285597
EIF2S1	-0.031526401	7.836150195	0.454306624
ZNF75D	-0.039045605	6.56378034	0.454454297
WBSCR19	-0.009121747	6.092394145	0.4545039
PIP4K2C	-0.027356785	7.020844837	0.454527926
CCDC58	0.048681461	7.964545559	0.454660085
LOC646829	-0.017724756	6.006036446	0.454879792
CHRNB1	0.032122542	7.191389056	0.454902139
LOC645662	0.018686061	6.182207902	0.454921737
KMO	0.022372105	6.01177222	0.455211345
KLK2	-0.015669108	6.171292263	0.455289445
C1ORF99	0.012975204	6.012415445	0.455322971
LOC642109	0.02099415	6.354366581	0.455324955
CUL2	0.023444809	8.92904349	0.455454785
LOC653514	0.0167212	6.067206461	0.455463583
C9ORF109	0.018918993	6.195651688	0.455503561
DKFZP434P211	0.018540031	6.217763549	0.455731707
MGC35440	0.020196411	6.167846637	0.455793474
USP43	-0.023677798	6.110172118	0.456097874
EPS8L3	-0.015167005	6.129916139	0.456106152
LOC650323	0.018399135	5.909561537	0.456168871
C9ORF135	0.01966238	6.033759929	0.456175788
ZNF70	-0.022232153	5.952178308	0.456194235
GNRHR	0.020044312	5.929366453	0.456289608
ZCCHC10	0.015026093	6.232443524	0.456390504
LOC643429	0.018824271	5.985137773	0.456393944
LOC648629	0.018751307	6.10707685	0.456396635
LATS1	-0.013201613	5.988268373	0.456466036
LOC653746	-0.024103082	5.925125146	0.456538101
FFAR1	-0.016662582	6.283149446	0.456546185
RAB9P1	-0.017505937	5.903219192	0.456592911
MAP3K10	-0.026703052	6.09926784	0.456692081
TRPM2	-0.013593819	6.212286289	0.456752547
CHKA	0.020808286	6.922449317	0.456828532
LOC649891	-0.018250209	5.978730961	0.45702584
LOC400759	-0.036282733	6.242502741	0.457105996
CLEC4M	0.016958543	5.946931199	0.457138657
GREM2	0.017269337	6.019931187	0.457151563
C17ORF78	0.019195868	6.022082371	0.457233192
LOC649280	-0.021691542	6.0618968	0.457276166
LOC643920	0.017598734	5.982056286	0.457307878
TAS2R7	-0.01846574	5.944801265	0.457317425
LOC441050	0.030528279	6.830276762	0.457342878
OR2C1	-0.02127932	5.942443329	0.457381039
LIG4	-0.017392511	6.381784581	0.457410552

LOC644499	0.019099829	6.278536496	0.457440083
LOC644748	0.016689523	6.16682728	0.457440675
LOC645165	-0.019771219	6.510261476	0.457504486
FLJ40288	-0.020848496	6.077672587	0.457572832
OR8U8	-0.01835573	5.958128861	0.457592877
FLJ20464	-0.017685552	6.003225752	0.457675251
BRD1	-0.02380685	6.94047612	0.457766629
GNAT3	-0.012614347	5.946628853	0.457796787
PNPLA5	0.021249328	5.962088136	0.45794563
GRM6	-0.018496826	5.925008477	0.458027694
LOC652798	-0.018336895	6.119918281	0.458051701
NDUFA3	-0.041512214	12.6264697	0.45806793
LOC390387	-0.011349466	5.958726834	0.458159477
DLGAP3	0.019737333	6.002251306	0.458162114
KLF5	0.037787795	6.218822687	0.458392756
FOXN1	-0.019530308	6.082285153	0.458560396
LOC645655	0.020343835	5.962869704	0.458592512
SLAMF7	0.022821693	6.070845787	0.458597263
LOC653297	0.02002539	6.185339308	0.458754213
LOC728012	0.014306373	6.123988715	0.458809143
SLC7A9	0.024843964	6.769930537	0.458821333
WSCD1	0.03657098	6.31703094	0.458839032
LPP	-0.059516594	10.35902163	0.45884483
LOC284428	-0.014219339	6.045760984	0.4589243
LOC441362	-0.020942024	5.917335393	0.459005806
LOC400301	0.017959409	6.043767871	0.459080535
LAMP2	-0.023668658	9.058466994	0.459138537
LOC642426	0.019709544	6.104001223	0.459232878
MACROD1	0.059006503	9.106488827	0.459267092
HIST1H2AJ	-0.017027791	6.064076115	0.459463154
JRK	0.013180541	6.282068979	0.459467719
PSCD2	0.020125219	6.959565228	0.459816357
SFRS3	0.031455348	7.315027444	0.459825182
TBRG1	-0.018688802	6.1207894	0.459840891
LOC649416	0.017403921	6.169356215	0.459845266
TCP11	-0.022392956	6.168808234	0.459864885
PDPK1	0.022009631	7.669612887	0.459876712
CUL7	0.020348167	6.255193757	0.459905985
PCDHAC2	0.012115759	6.091984376	0.459933605
OR9Q1	-0.019584166	5.845028869	0.459950252
TPPP2	0.018079921	5.987384198	0.460003013
KIAA1210	-0.021040572	6.141181465	0.460011615
OR13C5	0.020598376	5.879351838	0.46002828
SPINT2	0.058587668	7.36681085	0.460127477
FLJ40296	0.009812106	6.027863336	0.460176369
PGPEP1	-0.032457249	6.16586313	0.460222439
PCID2	0.032237276	11.09783797	0.460519847
LOC402571	0.021116035	6.099822746	0.460621107
LOC647163	0.020293659	6.016313628	0.46064232
ALG12	-0.022957213	6.611180762	0.460745434
SAMD6	0.012040161	6.095478142	0.460823993

EAF1	-0.042803091	6.970584169	0.460861278
LOC651635	0.016446564	6.14798077	0.460868176
LOC651186	0.02471767	6.095107461	0.460907686
FAM83H	-0.021555673	6.24278788	0.460913832
HORMAD1	0.011911058	6.028013046	0.46097879
PPIE	-0.023632292	7.79147107	0.461136962
LOC644504	0.01721247	6.023560558	0.461142811
C11ORF61	0.017682555	6.679496407	0.461267194
ZFP14	0.026386843	6.255807216	0.461285104
KCND1	-0.025990168	6.135462347	0.461324822
ARHGAP20	0.021917871	6.077379061	0.461447639
IMAA	-0.055348621	13.07178259	0.461476633
TREML4	-0.019175473	6.098883564	0.46154821
GDI1	0.036379182	8.646473225	0.461703434
UBR7	0.026901339	7.624419583	0.461762477
RPS3A	0.049246869	12.5614705	0.461789481
MEF2B	0.016672546	6.290197563	0.461862702
CLINT1	0.035091069	9.359461685	0.461955623
LOC648394	0.018365882	6.304760727	0.46197113
RIG	0.016587005	5.986166611	0.461997358
EXOC3L2	0.017766623	6.023669166	0.462109807
INTS3	0.033149823	8.917836077	0.462125508
LOC647869	0.01714153	5.993454299	0.462253009
LOC653105	0.011867562	6.071149077	0.462264388
LOC646448	0.019087868	6.077785076	0.462322216
LOC649768	0.016541935	6.250661728	0.462324546
LOC283551	0.017876073	5.973260124	0.462362493
LOC389316	0.016945706	5.984674751	0.462414972
LOC441376	-0.017439449	5.966634071	0.462470371
LOC731159	-0.019048031	5.932547339	0.462518105
LOC645444	-0.016359548	6.091694343	0.462812121
WDR38	0.013760442	6.279459015	0.4629121
ZBED2	-0.018808164	5.95681256	0.463102405
LOC643461	-0.020869248	6.216217019	0.463148962
ZNF692	-0.023184651	7.199378772	0.463214376
LOC652082	-0.018573609	6.348867267	0.463372285
MYO1A	0.018221853	6.422305842	0.46369093
PRSSL1	-0.019305563	5.986818414	0.46373147
RDH11	-0.026966129	8.760304213	0.463816283
LOC649374	-0.01641644	6.114987669	0.463836439
LOC645635	0.018317358	6.093212655	0.463856956
LOC653515	-0.017530802	6.096169371	0.463861981
NTNG1	0.013728186	6.012337558	0.463956114
LOC645755	-0.020994878	5.965385829	0.463977159
PGK2	-0.012928597	5.979520734	0.464048626
LOC652801	0.017055036	6.041100056	0.464089146
ADAM19	-0.077069563	8.500696874	0.464235857
LOC342346	0.018011011	6.334501807	0.464373941
SERPINB9	-0.015361717	6.131074211	0.464394612
RASEF	0.019899815	6.042559196	0.464399779
NPC1L1	0.020774071	6.165861228	0.46445796

FGF20	-0.013616841	6.138447029	0.464466074
LOC644993	-0.016249264	6.092993407	0.464527805
LOC730713	0.013047512	5.988512546	0.464530022
HOXB13	-0.018479173	5.96632801	0.464563221
LOC648735	-0.018452343	6.025785638	0.464715664
LOC730704	0.021159044	6.167417819	0.464779832
SLC10A5	0.017839902	5.981594742	0.464802149
VPS4A	-0.028212183	7.937808579	0.46486237
OVGP1	-0.025767588	7.178039769	0.46490215
LOC375323	-0.017235022	6.128196678	0.46490776
ALDH3A1	0.024901121	6.016624327	0.465048647
LOC653257	-0.014641151	6.097838266	0.465230481
LOC642480	0.02108246	5.959356765	0.465322657
LOC346950	0.050070622	7.192879897	0.465427774
LOC650253	-0.028113872	6.072916561	0.465430209
LOC647323	0.017821276	6.070938265	0.465445435
LOC653492	0.017260545	6.04101442	0.465489578
DEFB110	-0.017055814	6.047798556	0.465494846
NUP214	0.032993602	8.07254787	0.465494927
LOC648998	0.016322416	6.050034509	0.465517861
HELT	0.01781139	6.035614293	0.465553374
TTC36	-0.017540915	6.106460396	0.465753201
OR2T4	0.020920344	5.957408587	0.465762489
LAMA1	-0.010616513	6.11877404	0.465796361
LOC652067	0.021100602	6.129663852	0.465879156
C16ORF71	-0.018373015	6.039708392	0.466002797
LOC642736	0.018001732	6.061146737	0.466149507
ELA3A	-0.012414181	6.033722081	0.466157262
NUDT19	0.019147859	6.338641381	0.466166168
ATXN7L2	-0.024176381	6.629002442	0.466257563
LOC440944	0.020209864	5.960181332	0.466294527
HNRPA2B1	0.030079295	11.67990576	0.466363951
LOC652495	-0.018446784	6.027212663	0.466463146
NLRP13	0.016589754	5.995895965	0.466493509
MGC15634	0.021477026	6.331402585	0.466552698
SERPINB12	-0.018983992	5.883734772	0.466563271
ADAMTS6	0.021667854	6.255052583	0.466589667
LOC642093	0.018971728	5.977398754	0.46668114
CRYGC	-0.017677674	5.990023615	0.466716051
WDR90	0.017281585	6.304192203	0.466785719
LOC392559	0.019277128	6.012615553	0.466892778
LOC649886	0.017872601	6.199188506	0.466898876
FLJ16165	0.020012782	6.093024448	0.466918694
LOC642216	0.017184022	6.019654451	0.467021685
LOC649383	-0.017791442	6.123113892	0.467030158
MYH1	-0.011450398	6.042212874	0.467077836
ZNF238	-0.012395157	6.206435271	0.467102419
LOC730344	-0.016053906	6.047249806	0.467113388
KRTAP8-1	-0.017310533	6.030137876	0.467307648
TMPRSS4	-0.01679543	6.182674261	0.467517047
TXNDC11	-0.027926803	7.356274722	0.467697646

LOC441344	0.015293248	5.965271293	0.467766483
LOC646365	0.020938794	6.095453144	0.467766653
OR8I2	-0.022687907	5.930183184	0.467820784
NUBPL	0.044443133	10.38753394	0.467846887
LOC653314	-0.027881735	12.34791331	0.467877677
LOC647741	-0.020415391	6.29361507	0.467882039
FBXO17	0.012051577	6.129564869	0.468032174
FLJ43870	0.019765377	6.034468483	0.468143772
FAM76A	0.032998934	6.557929483	0.468176715
PRAMEF17	-0.017366371	6.161641181	0.46820571
PON3	0.025986484	6.601092235	0.468241084
CART1	0.020001127	5.895490957	0.468385499
ZFP95	0.027370941	5.989849188	0.468415939
LOC150786	0.018438174	5.926329949	0.468473116
LOC642514	0.020004023	6.167132476	0.46849636
TECTB	-0.019755878	5.965873576	0.468519508
FAM89B	0.03244298	8.634340196	0.468579653
C2ORF61	0.023739343	5.957179904	0.468641771
KIAA0492	-0.025579457	6.407198914	0.468646331
DNHD1	0.022542118	6.568644788	0.468667472
LOC645818	-0.018192293	6.042556632	0.468672851
LOC654203	-0.0168564	6.203162957	0.46867903
C5ORF32	-0.045058821	9.63303581	0.468809216
FLJ33651	0.018400075	6.025436442	0.468828667
KIR2DS5	0.017281464	6.066934574	0.468867582
LOC646458	-0.018465324	6.097850272	0.468917727
LOC642450	-0.016245594	6.098679759	0.468968008
HGF	-0.01287214	6.092999278	0.469086795
C4A	-0.024710833	6.083603357	0.469269113
SPAG5	0.017102717	6.29977893	0.469276678
SGCD	0.042658498	7.758498814	0.469286138
VPS54	0.016911256	6.728965545	0.469378629
TARSL2	-0.034680988	7.222900679	0.469392789
LOC253820	-0.019013849	6.005238488	0.469523937
SERPINB11	-0.017040176	6.169241788	0.469584241
CPNE2	0.020880989	6.297804965	0.469625938
PLEK2	-0.018556701	6.118674199	0.469794531
PLA2G12B	-0.019272587	6.268942389	0.469863624
C2ORF53	0.016995962	6.17472416	0.469978324
SPATA6	-0.01590612	6.257597217	0.470021457
MYF5	0.013898718	6.154843955	0.470191534
ARPP-21	-0.010683971	6.014301252	0.470223607
ABCD1	0.03027608	6.916327116	0.470252668
LOC649041	0.020475493	6.023740496	0.470287418
ZNF766	0.036356904	6.21671382	0.47044875
SFI1	-0.011521706	6.14991096	0.470524227
HLA-DQA2	-0.016966143	6.211188033	0.470559676
LOC644171	-0.014945412	5.971112362	0.470716782
LZTS2	0.0210176	7.050489763	0.47077436
C17ORF57	-0.017441273	6.085392696	0.470838009
LOC338579	-0.011335691	6.04762799	0.470845845

LOC643283	-0.01792175	6.016091753	0.470948188
LOC286016	-0.041002573	9.739336178	0.471082886
NEU3	0.029817809	6.078811468	0.471254639
SH3BGRL2	-0.036588705	7.872252105	0.471348068
LOC644898	0.026422229	6.131345112	0.471461929
ASNSD1	0.039158681	9.759822104	0.471496012
CMTM2	-0.019810668	5.933337941	0.471547863
C7ORF29	0.019679762	6.520835105	0.471735643
ABCA7	-0.014301217	6.134894323	0.471784273
STIM2	0.035318494	6.674951014	0.471811776
FAM120C	0.022343285	6.071916388	0.472016283
MAP3K9	-0.027487065	6.088392758	0.472099564
PLCB1	0.031282953	6.779040767	0.472132825
CST1	0.02546876	6.083958464	0.472141716
LOC652071	-0.037240435	13.03108182	0.472263792
LOC400708	-0.021304528	6.192995066	0.472485843
ZCCHC2	0.017841583	6.005448365	0.472651066
FGFR2	0.020039718	6.049816098	0.472713036
MGC34761	0.01869432	6.237510576	0.472806258
LOC646183	-0.018905783	5.947944242	0.472907211
WIPF3	0.017959694	6.01127102	0.473048694
LOC648447	-0.017568688	5.964482903	0.473095534
LOC649754	0.013545307	6.537587449	0.473112066
LOC642280	-0.013867193	6.010185829	0.473114677
LOC649296	-0.02010131	6.079299791	0.473141674
DYDC1	-0.012784442	5.972983537	0.473258329
MAGEB3	0.029732825	6.111786932	0.473362638
ASB17	0.013235817	6.072355284	0.473379203
OR5M8	0.013244853	5.98330008	0.473414631
LOC644429	0.017910509	6.063364788	0.47346483
MGAM	-0.014200893	5.978573755	0.473545884
LOC652433	0.016039319	6.010378089	0.473555434
LOC730920	-0.019063256	6.523501434	0.473562739
OR5M9	-0.017358327	6.044909924	0.473564256
KIF24	-0.01690624	6.150614783	0.473579755
MYBPC3	-0.049680625	14.3174638	0.47362236
FLJ42102	-0.01826257	5.89416571	0.473777471
PSORS1C2	-0.016343291	6.094213441	0.473780925
LOC646869	0.017239485	6.247016546	0.473851162
LOC391475	0.020104679	6.360011751	0.473871764
FLJ42289	0.018535016	5.991556232	0.473889481
PIB5PA	-0.050740618	7.972428605	0.474058351
HS3ST5	0.021942003	6.009465126	0.474123254
LOC651729	-0.02223817	6.13396044	0.474187506
RXRG	0.016621414	6.272396918	0.474248332
LOC643363	0.018720085	6.005755878	0.474270314
WHDC1	-0.026590122	6.596668102	0.474359511
LOC649702	-0.021984226	6.07696539	0.474369122
LOC652698	0.025449333	6.388877178	0.474427353
BLR1	-0.020346487	5.953694594	0.474454219
LOC133609	-0.014578045	6.031682121	0.474542469

LOC441931	-0.018756748	6.112460917	0.474618528
CNTFR	-0.01442744	6.161078997	0.474757669
DEFB126	-0.016002295	5.893644467	0.474861581
KRTAP6-3	-0.016670234	5.995069608	0.475077871
LOC642219	0.02671832	5.914661219	0.475106754
LOC642962	-0.018312441	6.150403116	0.475130686
LOC642133	0.016397983	6.075647749	0.475193276
DNAJB7	-0.017290109	6.01300205	0.475260429
LOC646280	-0.020553338	6.072360259	0.475298228
INDOL1	-0.020977112	6.326589716	0.475333828
PKDREJ	0.023630949	6.055125041	0.475389266
DDX53	-0.017518996	6.067231912	0.475396533
KCNE3	0.020338339	5.984859441	0.47539929
LOC652548	0.019432556	5.902826685	0.47549704
LOC127011	-0.022597206	5.953033351	0.475555425
LOC650861	0.019990858	5.962398217	0.475634656
C1ORF108	-0.022731521	6.108624069	0.475675643
SMNDC1	0.051053574	8.368087688	0.475712741
RBM6	0.033658736	8.424800828	0.475756598
LOC653381	-0.042747962	9.148027704	0.475853331
LOC644681	0.019650874	6.16930677	0.475922019
ADORA1	-0.039047122	6.712798415	0.475932013
LOC653752	-0.031939647	6.929551341	0.476014119
LOC645929	0.01961859	5.922577817	0.476098853
LOC441812	0.018509092	5.927504368	0.4762936
GPR153	0.018934026	6.344432018	0.4763377
LOC648972	-0.016971308	6.105167918	0.476420426
CAPZA3	0.018612629	5.997649558	0.476447859
IVL	0.029933027	6.028483472	0.476453976
LOC339809	-0.020755781	6.147542617	0.476508124
POU3F3	0.021259027	5.93880188	0.476576565
SUMO4	0.01881396	6.115144583	0.476583122
CLPB	-0.017782934	6.171709088	0.476606531
SAA4	0.02052532	5.972042081	0.476656939
C9ORF53	0.016997831	6.060552872	0.476758404
NMS	0.015876294	6.064361646	0.476765031
TMCO5A	0.029742876	6.001870497	0.476877429
LOC649360	0.02052387	5.822881949	0.476900889
LOC728334	0.017244295	5.999464229	0.476937232
CRYBA2	0.014070329	6.133654245	0.47701071
FLJ90231	0.01200313	6.019420947	0.4770925
LOC652353	0.01639808	6.108670875	0.477100772
LOC648704	-0.023786291	6.080987156	0.477135731
GDF5	0.017887097	6.045905684	0.4771797
LOC391817	0.019356372	6.215939351	0.47727502
LOC652905	0.017232569	6.182079012	0.477349059
LOC730034	0.021551642	6.053697617	0.477376343
CCDC11	-0.018243584	6.06922	0.477380699
SLC25A21	0.017113879	5.999148064	0.477404617
KRT39	-0.012106674	5.980969055	0.477433218
GHRHR	0.014622891	5.92745357	0.477439376

LOC647681	0.016048994	6.118759493	0.477593427
LOC646498	-0.012234734	6.106932361	0.477833117
GNAS	0.027135234	12.10058629	0.477904115
CYP7A1	0.015821853	5.993383499	0.477933967
ASPRV1	0.018908642	6.26151316	0.477971867
TMEM5	0.044183043	10.12701063	0.478110095
FLJ25758	-0.018305905	6.045764833	0.478224447
GLYBP	-0.018530738	6.14240687	0.478339541
LOC648657	-0.017856547	6.13928726	0.478363514
ZC3H5	-0.039513068	7.673112199	0.478440607
LOC646990	-0.018326394	6.438318759	0.478651904
PYCRL	-0.020235544	6.016841461	0.478726223
C2ORF54	0.017957393	5.983332214	0.478736526
LOC649174	0.017872776	6.227721959	0.478794289
HUS1	0.014203725	5.993085277	0.478893081
LOC730809	-0.017508034	5.990634503	0.478913207
FLJ33387	-0.018452632	6.075340102	0.478957079
ALAD	0.045028951	7.806562636	0.479101429
LOC647003	-0.017211158	6.10530161	0.479136994
LOC650373	0.015264809	6.068203107	0.479146005
LOC400768	0.019020037	6.14684042	0.479171486
BCAS1	-0.014004807	5.962740928	0.479282202
ABCA9	-0.041647242	7.104690111	0.479402145
LOC646363	0.018774911	6.07010015	0.479425065
LOC652213	0.019847174	6.105385372	0.479444547
RAG1	0.017760659	6.104397552	0.479462164
ADRBK1	0.020951098	5.96072925	0.479498228
LOC649853	-0.019831253	6.130044924	0.479503189
FAM105A	-0.03500048	6.138578755	0.479618247
C6ORF26	0.016820375	6.159018212	0.479689768
PHACTR2	0.046328358	8.702541547	0.479712478
LOC730132	-0.016226995	6.152660886	0.479738971
CHPF	0.048927368	8.676820352	0.479927886
FLJ14816	-0.018397792	5.978138986	0.48012059
LOC649580	-0.018777106	6.473578309	0.480126411
LOC647197	0.017299333	6.076092647	0.480267561
SCN8A	-0.018871227	5.916295332	0.48038398
LOC653118	0.016366892	6.07022478	0.480445307
FBXW12	0.018083597	5.977851585	0.480451843
UBE3A	-0.025600319	9.634967788	0.48054837
LOC652608	0.026475196	6.262708699	0.480573555
LOC646173	0.01819593	5.984478102	0.480593626
EREG	-0.018568115	6.219337808	0.480614367
UTS2	-0.009614023	6.003200203	0.48061587
GLIS1	0.017463038	6.031434054	0.480671932
LOC642597	0.014849348	6.049234095	0.480706414
IFT80	-0.015719529	6.018525125	0.480802399
FDX1	0.028535496	7.114509254	0.480901937
LOC646401	0.017048092	6.048721	0.48095423
GALNT7	0.016378515	5.905641523	0.481052629
LOC440248	0.018198424	6.220343577	0.481064805

PCYT1A	-0.01870931	6.196824001	0.481084835
LOC728044	0.016931904	6.031464396	0.481102693
KRTAP1-3	0.017835416	6.040725	0.481114542
FAU	-0.030080905	12.75523713	0.481158013
LOC652599	0.017174039	6.09435665	0.481185808
PJA2	0.05482632	11.21206697	0.481254608
URG4	-0.023983595	7.937767223	0.481259434
HTR3E	0.017470599	6.036508944	0.481348331
SURF6	-0.033006605	8.605221732	0.48143925
LOC650392	-0.016790135	5.977420825	0.481514373
OR2M3	-0.013790465	6.092724991	0.481528337
COX6A1	0.04746192	9.870755207	0.481533521
CALU	-0.054787279	10.17109975	0.481557294
LOC641852	0.019449309	6.103334428	0.481640089
FLJ42709	-0.018463577	6.055753119	0.481795623
C1ORF32	-0.020593719	6.151652744	0.481967366
OR5A1	0.018626049	6.009127419	0.481993861
LOC644091	0.022309348	6.103746524	0.48204773
ST6GALNAC2	-0.031989249	6.216549309	0.482165507
C11ORF58	0.037577661	6.55736719	0.482318366
KIAA0527	0.012253624	5.974878072	0.482351763
LPIN3	-0.020216283	6.391121336	0.482448092
SI	0.014731612	6.074113229	0.482467361
C22ORF34	0.018912305	6.050999614	0.482478537
NFXL1	0.026063097	7.005761914	0.482484492
PTPRJ	-0.017636368	6.190765419	0.482683401
SPANXE	0.012141276	6.043525476	0.482739542
LOC155100	-0.015659975	6.12340528	0.482858864
AMELY	-0.016509805	6.167013001	0.48305518
RRP15	-0.044209031	8.861697152	0.48323816
C6ORF118	-0.016056355	6.002705895	0.483314326
LOC653052	-0.019216217	6.089842264	0.483622869
MAP1D	0.02954182	6.15818565	0.483636521
LOC645489	0.035942971	8.055947387	0.483651021
MYH11	0.083938406	9.745801575	0.483780107
LOC649406	0.015256808	5.982442093	0.483813317
LOC650144	0.027980825	6.016497521	0.483958384
C21ORF41	0.009174376	6.042998413	0.48400254
LOC651467	0.015005453	6.001169507	0.484174743
ETNK1	-0.015178591	6.450384034	0.484252054
HSPCAL3	-0.021382483	6.097494309	0.484425819
LOC164380	0.016916792	5.865845531	0.484493607
LOC401131	0.018858025	6.010893519	0.484553287
DTL	-0.020541779	6.118114862	0.48458259
ATP10B	-0.018411149	6.113204019	0.484617294
LOC650436	0.019906154	5.828695641	0.484630639
PRDM1	0.019082939	6.367646412	0.484701827
LOC642769	0.018815733	6.180717323	0.484755232
PRCP	-0.028415164	10.56880629	0.48482966
MAP3K3	-0.028156054	7.080663337	0.484925138
PDIA3	-0.023674167	6.053047952	0.484962347

GUSBL1	-0.031152972	7.51513452	0.484993666
OR6C2	0.018445194	5.914625739	0.485033068
DACH1	-0.03233768	6.666025723	0.485136066
TMEM32	-0.033132154	9.531110259	0.485202414
LOC729335	0.013602638	6.103098541	0.485235738
LOC651957	0.019009598	6.115436793	0.48533246
LOC645396	-0.017025153	5.997542282	0.485454368
ITGAD	0.022479031	6.019065561	0.485455131
SFRS6	0.033993009	10.87132486	0.485459212
SLC22A2	0.012784651	6.012413171	0.485466158
LOC652701	0.019747541	5.998305305	0.485476788
LOC643684	-0.014140032	6.057415828	0.485491435
WDR36	0.028037514	7.974301323	0.485560194
SERPINA9	0.01385738	6.076102127	0.485568078
SF3A2	-0.030674025	8.426051279	0.485593708
ACTA2	-0.075421392	12.14685093	0.485673521
CHRNB2	-0.020688316	5.932411338	0.485724826
SLC25A35	0.018107497	6.246229684	0.48573251
DNAH5	-0.019133961	6.187194568	0.485796898
LOC652703	0.018822651	6.119766521	0.48588534
CHAD	0.016191103	6.126359697	0.48592553
DGCR8	-0.040457147	7.526731591	0.485931271
AMBRA1	0.022255134	6.193425755	0.486002729
C14ORF156	-0.03221337	12.6051945	0.486007999
LOC648527	0.015244252	5.950937762	0.486052082
SPO11	-0.011024344	6.17006285	0.486159137
LOC151121	-0.01926657	6.20585374	0.486237239
BPY2B	0.01754563	6.098274629	0.486277114
PTK6	0.015365124	6.410679417	0.486307613
LOC728094	-0.018463748	6.019267986	0.486524078
RAB10	-0.027634321	10.94365292	0.486553688
C6ORF94	0.016511683	5.945021357	0.486601027
LOC340511	0.018346957	6.062247361	0.486705034
FLJ36032	0.016309756	6.02686823	0.486819263
OR5D18	-0.014198539	6.020392455	0.486869317
SLC17A5	-0.018917084	6.19113645	0.486889023
CEPT1	-0.018853414	7.472900322	0.486948396
DPP8	0.020740176	7.317820921	0.486965841
PSPN	-0.018218534	5.943391593	0.487093166
GRM2	-0.023220936	6.580642138	0.487099992
OR6C74	0.018326094	5.98509245	0.487169445
B3GAT2	0.016035941	5.993542774	0.487240631
STMN4	-0.01654662	5.981494136	0.487319553
TREM2	-0.018288234	6.138245529	0.48732206
KRTAP13-1	-0.014015272	6.13152666	0.487409913
MAP3K2	0.018664635	6.56936373	0.487621052
C14ORF23	0.018386377	6.00950258	0.487664054
SERF1A	-0.016678408	6.287727181	0.487740243
FRMD1	-0.019133496	5.919560012	0.48783688
VPS26	0.051539131	7.173416943	0.487841304
FAM45B	0.018055479	6.231728101	0.487904866

DUPD1	0.016624024	6.03048013	0.487929882
RXFP4	-0.018277045	6.06363161	0.487930721
LOC732275	0.01779484	5.999769698	0.488083583
C4ORF45	0.016096385	6.087733782	0.488101854
ACO2	0.047988217	11.2609856	0.488133432
DTX4	0.018292912	6.005478831	0.488150928
EXOSC5	-0.032077134	7.861252517	0.488214304
CDK5RAP1	0.019681173	7.983596494	0.48826422
TNP1	-0.017340379	6.223214221	0.48834188
ZNF80	0.017633406	6.268034441	0.488424855
ZG16	-0.015220331	6.023772032	0.488472868
LOC649447	0.034326757	11.44847376	0.488501653
LOC650657	-0.019170796	6.148366352	0.488530874
DKFZP686D0972	0.020756893	5.85270253	0.488648076
C21ORF88	0.020069327	6.140828496	0.488651482
STON1	0.030691727	6.635313322	0.488697785
SNX26	0.029467866	7.540815092	0.488702677
C6ORF185	-0.017820007	5.969234018	0.488746448
LOC641738	0.014946094	5.971762255	0.488754041
LOC389300	0.015812622	6.05838092	0.488771337
LDLRAD2	-0.019380805	6.131758997	0.489084977
SNHG3	0.018316712	6.107030192	0.489095054
LOC255275	0.016130016	6.047469428	0.489135317
C6ORF61	0.024293308	6.505866438	0.48933019
ACOT6	0.016470479	5.949238986	0.489393875
FAM124B	0.051500473	7.048010713	0.489418169
TSPAN14	-0.044499712	6.516468035	0.489576035
PEX5L	0.012909808	6.075686754	0.489582064
B4GALNT4	-0.02180102	5.858466819	0.489743932
LOC440309	0.01948102	6.108341511	0.489924844
HHLA1	0.019284252	6.156428194	0.489944818
LOC645690	0.015557972	6.073287161	0.489948427
EPHA6	-0.007676336	6.06359737	0.48995216
LOC440421	0.018132067	6.211780425	0.489989776
IL13RA2	-0.025128277	6.023616054	0.489989832
TUBGCP3	-0.019034564	6.144115034	0.48999092
APOA5	-0.021036453	6.007956401	0.490057363
SPANXN2	0.021862234	5.891131421	0.490093367
LOC90925	0.016333553	6.088960171	0.490108881
OR7G1	-0.020300873	6.092072786	0.490280614
GPR26	-0.024556224	6.051598594	0.490286464
BMP8A	-0.015746617	5.994190616	0.490392434
LOC645401	-0.019988401	6.259720371	0.490398125
PDCD1LG2	-0.019887722	5.84181398	0.490448545
LOC644088	0.014980626	6.068452719	0.490483015
SHARPIN	-0.046289325	6.833090876	0.490663189
MAB21L2	0.014214906	6.126253279	0.490785107
LOC284215	0.016904455	6.034722008	0.490818785
KRT81	0.024137397	6.143438629	0.490896112
LOC652416	0.019727044	6.045655551	0.490926251
C16ORF89	-0.031853311	6.077222656	0.490955831

DEFB128	-0.019294957	6.135724166	0.491020693
RNF113B	-0.016593398	6.318607795	0.491056939
DUX3	-0.017777747	6.163970808	0.491075287
LOC651345	0.00907066	6.019931474	0.491120326
LHX5	0.019667943	6.191667897	0.491195774
OR10A6	0.017430609	6.054035195	0.491196888
FLJ35946	0.019086552	5.893113447	0.491310635
PCDHB12	-0.020376715	6.309605801	0.491314531
LOC649341	-0.014587278	6.047059487	0.49133894
NAALADL2	0.018434636	6.145808067	0.49138638
HTATSF1	-0.017964321	5.949848457	0.491391845
C6ORF68	0.016472241	6.039645117	0.491394571
PALM2	0.020169924	6.23884243	0.491405305
KCTD2	-0.027167873	6.784730341	0.491435097
PTAFR	-0.027203073	6.185880393	0.491460947
PPP2R5B	0.026227861	7.293535277	0.491546332
PCDHGB7	-0.012456716	5.980166797	0.491579975
FAM90A8	0.017416327	6.032850321	0.49164051
LOC651675	-0.01675383	5.977939416	0.491799202
HIST1H3A	0.017647957	6.426545078	0.491815782
KRTAP5-10	0.017919045	5.98794791	0.491915853
LOC644014	-0.022750901	5.887908831	0.491984571
CEACAM5	0.017649681	6.0968727	0.491986716
PCDHGA5	0.01222474	6.115626993	0.492044499
PAX3	-0.011344555	5.960644486	0.492104859
SLIT1	0.016458251	6.034273058	0.492119306
LOC727787	0.015437985	6.126816344	0.492158322
LOC728003	-0.014369246	5.911041438	0.492160849
TUBGCP6	0.016801268	6.728993438	0.492244076
PPP2R2C	-0.025011363	6.365132405	0.4922889
TNFRSF17	-0.019032716	5.920931613	0.492331588
RPL10L	0.021780282	6.430940126	0.492372468
LOC653692	0.017071398	6.086069509	0.49240995
CYLD	0.022176202	6.777324319	0.492619796
LOC645268	-0.016357083	5.970129972	0.492680157
PRR5	-0.013347146	6.374110936	0.492693875
GNAT2	-0.020318546	5.973600098	0.492805987
LOC652669	-0.019190699	6.297778407	0.492837363
TEX101	-0.021076878	5.94520116	0.492921485
ZNF492	0.012720863	6.157237875	0.492989651
FAM129C	0.017345651	6.057619957	0.493024401
FLJ31222	-0.016814824	6.00504612	0.493060352
SLC10A1	-0.01798535	5.916659885	0.493098286
LOC645177	-0.017699777	6.087567363	0.493187273
KCNJ15	0.011025478	6.094714081	0.493224137
UGT1A8	-0.018965546	5.889069257	0.493487245
LOC646686	-0.017927761	6.182051589	0.493493549
FLJ41352	-0.01016255	6.030096035	0.493551816
LOC644629	-0.017517375	6.240532406	0.493557299
LOC653411	0.02132669	6.084986983	0.493733455
OC90	-0.017709375	5.977114123	0.493743488

LEMD1	-0.017436547	5.953963188	0.493848113
LOC647943	0.019936643	6.172912819	0.493851836
LOC388458	-0.019245258	6.173189386	0.493874156
UPK3B	0.009724006	6.084387674	0.494069786
PGGT1B	-0.025646991	6.775905325	0.494080067
LOC646043	-0.030504181	6.201785235	0.494105565
CDKN2A	0.011152797	6.065971753	0.494302248
C12ORF23	0.055486185	7.768044609	0.49441864
LOC645164	-0.015896577	6.114932928	0.494443025
BHLHB4	0.01789253	5.944045072	0.494573516
LOC652854	0.01583502	5.970349525	0.494828623
LOC400095	0.015854117	5.969339056	0.494841721
LOC286310	0.014877124	6.583596944	0.494854882
OR5D13	0.016461613	6.071740681	0.494858688
MRO	0.018319632	5.952023265	0.494880423
ABHD6	-0.025174406	6.921285731	0.494909617
LOC652272	0.018007049	6.093039482	0.494956424
GNPTG	0.03109622	8.745709077	0.494971647
C11ORF16	0.017952839	6.232752645	0.495009557
LOC440871	-0.017803495	6.007824252	0.495185788
TGM5	-0.009280636	6.022555464	0.495205622
VDAC2	0.026328838	6.204883236	0.495263317
LHX4	-0.018499277	6.207267884	0.495338122
FLJ42258	0.013437502	6.218160731	0.4954052
LOC440551	-0.023178944	6.03936197	0.495430979
PHC2	-0.023938915	7.039973614	0.495464213
LOC441864	0.024280635	6.080268226	0.495468106
CTDSP1	0.02693945	8.21392383	0.495554944
BMP2KL	0.017714085	6.168509851	0.495608329
LOC647533	-0.014966343	5.986292439	0.495625876
LOC650995	0.018559109	6.051619366	0.495682408
KIAA1529	0.017995589	5.913198812	0.495708098
HOXA10	-0.011200701	5.987761711	0.495745235
SENP3	-0.032797704	6.163243427	0.49604451
LOC646249	0.016135373	5.949876045	0.496131825
SPEM1	-0.019582495	6.138185754	0.496306931
TJP3	-0.016827238	6.059551067	0.49651545
HAAO	0.017434616	6.311167056	0.496576121
CBLN4	0.018911217	6.096308289	0.496626249
LOC283849	0.024691555	6.192359505	0.4967784
SOX10	-0.021740085	6.296390601	0.496793366
KBTBD9	0.01680083	6.338742109	0.496831481
PGK1	-0.044474041	10.11392781	0.496835234
DNAJC16	-0.017071669	6.209038455	0.49686815
LOC643100	-0.019052056	6.07332029	0.496926174
FXN	-0.018025558	6.75639639	0.497041464
TCF1	-0.020304144	5.894529871	0.497089574
ERMN	-0.010235315	6.016053519	0.49713574
LOC653885	-0.019168605	6.176477822	0.497142063
OR2H2	0.022985986	6.704856399	0.497262478
NAT11	0.023654268	6.350262265	0.497293106

LOC648974	0.015744356	6.082648091	0.497376954
ZFP42	0.017192171	6.04492752	0.49738826
LOC441698	0.019723783	6.057436615	0.497652823
MAST3	0.022804	6.906245144	0.497654469
LOC388259	-0.015064248	6.058898401	0.497677179
SULT2A1	0.017343414	6.027534221	0.497712502
ROPN1L	0.01766915	6.279919374	0.497876406
LELP1	-0.01571602	6.087512907	0.497954299
FAM150A	0.016931366	5.982142692	0.497954606
OSTN	0.016744054	6.005364729	0.497970186
LOC647540	0.015894365	5.971160179	0.498327598
FAM133B	0.037938974	8.072839683	0.498359003
LOC642112	0.021217011	6.066453399	0.498392148
MKKS	0.02301538	8.513359453	0.498413653
L3MBTL2	-0.022112155	7.316208407	0.498552335
LOC648840	-0.017066011	5.949982058	0.498590877
LOC645955	0.015192024	6.141992042	0.498594773
OR52B4	-0.016944709	5.915393566	0.498630633
LOC442261	0.018420037	6.107629964	0.498641499
OR2M2	-0.016379153	6.04953923	0.498651929
MANEA	-0.014654761	6.285669441	0.498670627
LOC653418	-0.017464955	6.01965485	0.49885866
OR6C3	-0.017766065	6.176744922	0.499115432
NCR3	0.017457826	6.030892074	0.499186471
FLJ37587	-0.017886954	6.100267557	0.499219375
DNASE1L2	0.018094379	6.085476052	0.499239328
TADA2L	-0.012467008	6.333510599	0.499245918
FLJ16793	-0.022046956	6.284834957	0.499295536
WDFY1	0.036667672	9.085775049	0.499368834
LOC652456	0.015325262	6.242162256	0.499407143
ZNF77	0.024952367	6.596646132	0.499437202
BARX2	0.019386918	6.14063364	0.499506369
FABP2	-0.020311938	6.263357417	0.499562912
UNQ6975	0.018062552	6.048799346	0.499719669
LOC730241	0.01838778	6.169164257	0.499745642
EXDL2	0.028129161	7.822641522	0.499762032
LOC645585	-0.017985963	6.006832081	0.499770568
SLC15A4	0.028516056	8.552828971	0.499776381
HECW1	0.01910551	5.990509258	0.499816112
KIAA0100	0.037900512	6.380777452	0.499845015
LOC642497	-0.016927107	5.985965571	0.499898649
LOC158825	0.017191303	6.074586196	0.500060593
LRRC36	0.016843261	5.988919757	0.500065087
LOC441233	-0.016758861	6.024804275	0.500068831
CASP6	-0.019824438	6.549263061	0.500071861
LOC389786	0.017762699	6.068229843	0.500092351
LOC554226	0.012780749	5.930300424	0.500099606
NUBP1	-0.029468781	8.404424277	0.500171297
OR6S1	0.011520354	5.989405905	0.500310572
ERVWE1	-0.012570984	6.156255919	0.500381608
NMBR	-0.014861283	6.037456127	0.500435465

LOC644640	-0.018291604	6.22797129	0.500440166
LOC652148	-0.016276713	6.014637926	0.500586738
LOC732313	-0.015825485	6.03692391	0.500624776
LOC652633	0.022517055	6.233254737	0.500752222
CPNE9	-0.025704983	6.021601413	0.500769894
ZNF547	0.017885588	5.954456144	0.500845148
SMTNL1	0.018269667	6.221231821	0.500846954
HMP19	0.023066888	6.327764699	0.500853213
ZNF498	-0.023363432	6.082751669	0.50090614
LOC644419	0.016285247	6.053661422	0.500907116
TMCO2	0.020731958	5.909896566	0.500909612
LOC374973	0.014936794	6.070418296	0.5009438
LOC652515	0.016399705	6.136225296	0.500988182
SLC16A10	-0.042728452	7.932945265	0.501056292
LOC650887	-0.017924478	5.96554601	0.501251174
LOC652034	0.019442502	5.907801714	0.501279172
LOC646110	-0.015867493	6.051937416	0.501280254
OR7G2	0.029799665	5.887483889	0.501367953
LNX1	-0.019480681	6.284255304	0.50137528
EPHA4	-0.049841447	7.663799486	0.501465395
LOC652635	-0.018789133	6.024391015	0.501649857
ACAD9	-0.023600608	8.411520853	0.501692499
LOC442132	0.025893916	5.929745569	0.501789907
LOC650668	0.015740656	6.020154952	0.501923053
GFAP	0.018270717	6.103477014	0.502073052
LOC646691	-0.015349615	6.087062286	0.502089957
ZNF780B	0.016772324	6.091979908	0.502164739
LOC646976	0.016098651	6.095285244	0.502215613
CEND1	-0.016192583	6.249891949	0.502350647
CCDC89	0.025001186	6.57589956	0.502408961
CXCR4	0.034731164	6.328459314	0.502426519
OR4N2	0.018294296	6.071729469	0.502467386
NDUFA13	0.030843633	11.7320794	0.502484688
OR4X1	-0.016867084	6.067367971	0.502495458
CLC	-0.022227498	6.241293427	0.502515041
C7ORF13	-0.019753097	6.456663805	0.502528256
FAM47A	0.018702647	6.049564786	0.502628259
C15ORF54	-0.020538595	5.970146545	0.502632437
LOC402232	-0.016471639	6.296137114	0.502646836
RFPL3	-0.016421496	6.086892355	0.502828338
KRTAP4-8	-0.020093562	6.332961832	0.502861239
SERPINB10	0.015252469	6.150450539	0.502977823
CAGE1	0.015023739	6.200300342	0.502979935
UBE2V1	0.010260997	6.205538253	0.503169283
GRPR	-0.012191325	6.052812392	0.50318406
LOC646730	-0.018840091	6.020177377	0.503267748
SGSM2	-0.038860159	10.28779649	0.503350641
AVPR2	0.020632485	6.208263995	0.503454205
TBL1Y	-0.013244935	6.103549983	0.503465324
NPVF	-0.015849005	5.991054169	0.503498183
ZDHHC2	0.0280143	6.631335027	0.503517727

ADAM33	-0.012282955	6.325765106	0.503593089
NXNL2	0.012315777	6.09699068	0.503755764
WSB2	0.050126604	8.761086349	0.503873246
LOC651580	0.016523802	6.115831434	0.503892006
HAPLN2	0.019077164	6.34316123	0.503900293
LOC647234	-0.016259612	6.066258632	0.503970762
PLSCR5	-0.016995412	6.064782361	0.503986726
COL11A1	0.010552242	6.089142542	0.504013168
FN3KRP	-0.035389236	8.462776359	0.504078474
LOC649940	0.014902492	6.025747997	0.504123407
LOC643619	0.016842008	6.091466147	0.504154868
LOC645537	-0.019088773	5.946561935	0.504192291
TTC23L	0.016368924	6.162252536	0.50422204
LOC221981	0.011864591	6.113581184	0.504467946
LOC652871	-0.019958728	6.133763373	0.504481093
LOC646335	-0.016899811	5.959883965	0.504586538
C9ORF165	0.02075923	5.975001901	0.50458654
PADI6	0.016816541	6.125723205	0.504617069
NALCN	0.018965576	6.132798685	0.504700125
NACA2	0.024303254	5.901827221	0.50476347
FAM162B	-0.048722531	8.646518548	0.504855962
OR6K3	-0.015777504	6.032469517	0.504878045
GCUD2	-0.020484909	6.058702205	0.504980201
LOC649878	-0.016502528	6.044391496	0.504995702
SH3BGRL	0.032857482	7.665237237	0.505216283
LOC641912	0.018915993	6.110940384	0.50526757
LOC642132	0.017936122	6.04623038	0.505273322
LOC652678	-0.01932806	6.013048389	0.505475915
FSHB	0.016344074	5.99295572	0.505492244
ZNF391	0.021926894	5.863909182	0.505496727
LOC643855	0.016234792	6.153936728	0.505536309
PCDHB5	0.026881602	6.587452139	0.505591294
ACSBG1	0.024244915	6.222389548	0.505651684
LOC642403	-0.01727132	6.283720339	0.505699732
LOC650120	0.016394667	6.045597435	0.505850521
NR1D2	0.038398769	6.99122939	0.505859438
LOC388955	0.019176126	6.110693492	0.505873573
LOC642111	-0.023049201	5.867587085	0.505944439
MLC1	0.012027942	5.982012254	0.506097125
CYP2E1	-0.021066392	6.33299474	0.506216237
LOC646947	0.018121925	5.903583896	0.506296383
LOC651578	-0.017081617	6.08236921	0.506383845
KRTAP19-4	0.01708959	6.029545623	0.5064072
TNFRSF13C	-0.039345084	8.199984063	0.506437449
LOC147710	-0.014386747	6.096376255	0.506460254
LOC642539	0.015507665	6.177122787	0.506477248
LOC650673	0.015969459	6.10261905	0.506484561
ASB7	0.013052746	6.076024483	0.506532952
LOC389888	0.017497546	6.039142373	0.50668109
ADH6	0.015503386	6.256292762	0.506710028
NBPF9	-0.00858036	6.154555895	0.506769629

GABRQ	-0.015452538	5.936020365	0.506884737
LOC652115	0.01631107	6.161700854	0.506982767
FAM90A2P	-0.008472382	6.126464671	0.507082652
RAB14	0.021006133	6.362346954	0.507109877
LOC652195	-0.019464541	6.360489776	0.507257978
ADCY9	-0.032744222	8.18494123	0.507268126
IQGAP3	-0.019681243	6.461236702	0.507276787
TAF11	-0.017627115	6.290671198	0.507325272
LOC650439	0.015698114	6.008744913	0.507583838
FASLG	0.01748021	6.106041081	0.507619228
C7ORF46	-0.020623904	6.25001385	0.507655937
ANKMY1	0.011922513	6.399836845	0.507745896
LOC652799	-0.0184531	5.955210929	0.507750638
GJB2	0.023558681	6.137235157	0.507857282
SCNN1G	0.01196147	6.073822916	0.507960658
PCDHGB2	0.015561845	6.094493888	0.50801717
SPN	-0.020942591	6.616395177	0.508152625
LOC401442	0.026242416	5.940192487	0.508231008
ZNF346	-0.020417699	6.290716032	0.508422671
LOC651558	-0.021901122	6.413308346	0.508422682
RPS13	-0.030244867	12.46895899	0.508424945
WHSC1L1	0.01107009	6.290353025	0.508549665
EOMES	-0.021038587	5.93357721	0.50865474
SESN2	0.017917099	6.197588648	0.508675632
LOC644677	-0.016022991	6.071343102	0.508781784
PABPC3	-0.018938749	6.058240205	0.50880192
LOC283711	0.016446319	6.269391803	0.508835269
LOC642383	-0.014942749	5.937775025	0.508873477
TRIML1	-0.01849061	6.403624354	0.508893213
LOC648791	-0.024722352	6.298153697	0.508916123
LOC643037	-0.018574981	6.01304369	0.508966558
LOC728498	0.012138718	6.103564772	0.50897542
LOC645162	-0.015161685	5.943454641	0.509065932
LOC392583	-0.017217771	6.036440102	0.509262236
ZNF672	0.029650051	9.269113679	0.509355204
MAPT	0.04650767	6.903260149	0.509362343
VPS13B	-0.008742683	6.185366945	0.509374115
TRIM44	0.029441945	9.60997931	0.509379878
LOC645785	0.017599245	6.118858145	0.509386573
POP4	0.038405591	7.506294288	0.509434832
RPL9	0.034611505	11.97315587	0.509480987
LOC643809	0.020273886	6.125201735	0.509543768
MMPL1	0.015399423	6.242828938	0.50957445
LOC653636	-0.016068616	6.067096933	0.509591118
LOR	-0.018709393	5.956961691	0.509592173
C13ORF31	0.026230497	6.268296911	0.509640963
LOC729558	0.021021695	6.293783597	0.509666016
ADM2	-0.02403738	6.816543755	0.509822692
LOC440570	-0.016317076	6.051234557	0.509839389
LENG9	0.017179351	6.049877074	0.509866775
LOC645355	-0.015548461	6.329276652	0.509873111

LOC154449	-0.015655883	6.029933617	0.509893016
BCLAF1	0.024848346	8.819755048	0.509914764
SRD5A2	0.020543204	6.131378623	0.510111449
CAMSAP1L1	0.041274595	8.767891282	0.510141192
C17ORF37	0.023927705	6.889944727	0.510149071
C6ORF146	0.018670845	5.992959788	0.510266697
NANOG	-0.017247107	5.994934022	0.510360711
LRIT1	0.016077481	6.0548678	0.510550156
SLC25A24	0.014957152	6.373164138	0.510551233
SLC10A2	-0.01742084	6.050273685	0.51055127
LOC642155	0.016834587	6.085635082	0.510662159
LOC729175	-0.017736321	6.173618547	0.510777748
ARHGAP17	0.026447726	8.65700828	0.510828774
KIAA1919	-0.01762812	6.013712689	0.510838278
LOC388885	0.01695884	5.953280586	0.510869892
LOC401911	-0.013422027	6.015684491	0.510877997
LOC649210	-0.015885364	6.376512857	0.511188372
LOC647836	0.016783848	5.978242293	0.511289473
C13ORF26	0.012527181	6.044290398	0.511419348
LOC731438	-0.018549812	5.91086129	0.511631501
GSG1L	-0.023748333	6.509706285	0.511640789
LOC643749	0.017475382	5.99228387	0.511696134
FAM99A	0.014477584	5.952520317	0.511699627
PRM2	-0.01778471	5.880820568	0.51174613
LOC645113	0.017481698	6.075061127	0.511858893
LOC653136	0.016124381	6.203151374	0.511884352
RTN4RL2	-0.016335378	6.072283386	0.511935554
SPOPL	0.024719809	6.693771164	0.512143838
PIK3C2G	-0.017006807	5.919801256	0.512177277
FLJ32658	0.017618464	6.004540318	0.512235204
PCP4L1	-0.017012462	5.972963214	0.512389022
PIGX	-0.03676068	6.619436795	0.512458933
PLEKHG7	-0.015879751	6.264515723	0.512481776
POLR3G	-0.010554213	6.02705726	0.512548673
HS6ST2	-0.011658815	6.041394378	0.512717592
MED19	0.034855672	8.30114417	0.512736297
LOC646677	-0.040649194	6.699483734	0.512817223
WNT7A	-0.017295238	6.063129443	0.512827669
BCORL1	-0.020388972	6.318948226	0.512914441
LOC648040	0.019560325	5.974861885	0.512927259
CHIA	-0.012322217	6.01977299	0.512958567
ICF45	0.019649176	6.336224057	0.512961247
ARHGAP25	0.014823251	6.433581677	0.512962359
LOC401206	-0.030910641	14.17150235	0.512966018
CLDN20	0.02068731	6.468091449	0.512973119
LOC441551	0.015178152	6.089860065	0.512977998
LOC390998	0.018201148	6.062894525	0.513192183
SMG6	0.024746286	6.279093295	0.513223242
USF1	0.030732317	7.071083609	0.513225981
FLJ44186	0.015414851	6.097564991	0.513256486
CRYAA	-0.017344466	6.114955281	0.513281419

LRRC4B	-0.013238547	5.953180116	0.513354838
MALAT1	-0.017856095	6.041009441	0.513389123
OR2L2	-0.017200305	5.94941581	0.513419547
LOC651006	0.018039582	6.124001822	0.51343942
LOC650038	0.013567343	6.013655444	0.51357784
KIAA1524	-0.011360666	5.994264002	0.513682539
LOC283340	-0.017181755	5.978349675	0.513699658
TBN	0.020945149	6.397793176	0.513739856
PGLYRP3	0.010844164	6.086424607	0.513745226
PTPRT	-0.010479683	6.091798367	0.513756264
INCA	0.014224846	6.021626778	0.51379152
IL8RB	-0.016432341	6.236012943	0.513819379
TRIM6-TRIM34	-0.012282188	5.93665744	0.513849411
CXCR5	0.013404782	6.274828376	0.513982532
CRISP2	0.019141989	6.180396779	0.514012368
LOC613266	0.019969348	6.001493951	0.514048933
MYLK	0.018904326	6.592871767	0.514050847
CXCL17	0.01808819	6.088665634	0.514142445
IL34	0.023660197	6.276944835	0.514157073
EPOR	0.021054007	6.418128088	0.514267796
LOC652710	-0.022585626	5.899660516	0.514341747
GSTO1	0.024915169	12.27962338	0.514442134
ALKBH1	-0.019376116	7.122526696	0.514515327
PBX2	0.036454313	7.417722924	0.514537482
EIF4E1B	0.022432723	6.129773336	0.514612007
LOC644010	0.009525309	6.074646726	0.514657256
C9ORF122	0.020575167	5.929347006	0.514669089
VAT1	-0.039425174	7.940768329	0.514706679
LY6G6E	0.018002441	6.043649318	0.514798467
LOC647784	-0.01129193	5.950897253	0.514884526
SLC17A1	-0.021270216	5.911167815	0.51489151
XAGE5	-0.017640738	5.885188467	0.514944455
IQSEC2	-0.016675432	6.333966821	0.515017102
UBE1	-0.034575996	9.371754966	0.515092417
LOC642370	0.023744173	6.025386414	0.515092955
LOC641788	0.023427534	6.310222454	0.515094722
LOC652142	0.015934337	5.992219914	0.515104735
THSD3	0.010056157	5.992966416	0.515346307
C1QTNF8	0.021815853	6.114058786	0.515454049
LOC648898	0.009173626	6.021420627	0.515464386
LOC643213	-0.016734063	6.194436215	0.515566755
PDHA1	-0.048602817	11.28030724	0.515724984
LOC648130	-0.024919762	5.994022506	0.515795642
OR52M1	-0.016186789	5.989937621	0.515830504
LPAR5	-0.046331173	7.742351256	0.515910536
LOC645448	-0.015120959	6.016239279	0.515954008
FAM82A2	0.041745019	8.042866414	0.516002384
MEST	-0.010084519	5.991570971	0.516218105
KRTAP5-9	-0.017175143	6.063944825	0.51628863
LCE3E	-0.019392975	6.063272594	0.516342939
C11ORF24	-0.028573433	7.711382611	0.516508064

COLQ	-0.057896	7.233538876	0.516519798
RPRM	-0.016710915	6.001045103	0.516551437
C3ORF27	0.017606548	5.964404088	0.516666371
ZNF718	-0.015514001	6.294162504	0.516716241
SAGE1	-0.010966	5.991077375	0.516758663
LOC644617	0.027617735	6.258355053	0.516826425
PURG	0.018868179	6.461724369	0.516848101
IMPG2	0.018082024	6.441899306	0.516909763
ELAC2	-0.026417289	8.210126083	0.516968455
MAP3K14	0.026935187	6.746078739	0.517018282
E2F7	0.022055822	6.06913635	0.517116004
TSPAN19	-0.014630524	6.171320742	0.517201526
AFM	0.019327138	5.826549786	0.51723671
LOC651315	-0.018927037	5.958759256	0.517308923
GPR158L1	0.015440862	6.093485731	0.517314548
FKSG83	-0.015977559	6.092271367	0.517350385
ZNF440	0.016218859	5.977184715	0.517370735
TCERG1L	-0.018237356	5.920579268	0.517693366
GPR12	-0.016188227	6.047718621	0.517775623
LOC727994	0.017918786	6.065562761	0.517802787
CXORF21	0.012736406	6.041485775	0.517814503
TCOF1	-0.009539757	6.075922507	0.517833109
LOC647363	0.018576222	6.620671123	0.517865753
C6ORF70	-0.024268212	7.092194843	0.517943399
ACD	-0.021966268	7.030941191	0.517983391
SLC16A1	0.016808763	6.138260681	0.518019103
LOC646863	0.014595014	6.230883788	0.518066817
RPAP2	0.026783433	7.879518332	0.518129827
LOC648237	0.014748415	6.072284067	0.518256671
EFHB	-0.013511985	6.138400467	0.518299539
LOC642650	0.016533428	6.037594773	0.518354926
B3GALT2	0.059606461	7.26732709	0.518375278
LOC642354	0.015397171	5.977833654	0.518388208
ATAD3C	-0.016713691	6.20357518	0.518407084
LOC650406	0.017513528	6.211385259	0.518411699
OR5B3	0.020251523	5.917997386	0.518506139
LCAP	0.012517595	6.172730159	0.51856616
HSFY2	-0.007923031	5.989591047	0.51857987
LOC652692	0.016507136	5.953770115	0.518666933
ST7OT1	0.020201901	6.24326585	0.518680235
C11ORF70	-0.013755711	6.255796255	0.518703581
LAIR1	-0.051383867	13.77766634	0.518914667
SPINK5	0.018017469	6.200376636	0.518937867
LOC642677	-0.016478448	6.073822014	0.519039955
GNAZ	0.033607113	6.120946452	0.519123731
LOC643331	-0.020800992	5.895369424	0.519195257
LOC643069	0.016337391	6.097335773	0.519201507
LOC648365	0.018401457	6.0471166	0.519469816
LOC652281	0.023448767	6.326061081	0.519471426
GPR172B	-0.014681314	6.036031174	0.519488933
WBP11P1	0.008493649	6.031893016	0.519540141

PPBP	0.022635728	5.956243381	0.519678971
ZNF267	-0.020207367	5.896392468	0.519751288
SLC13A3	0.013483484	6.338183613	0.519836141
STRADB	-0.043258808	9.309039299	0.51985532
TMEM103	0.017775928	7.924061675	0.519867701
HCN1	-0.019145394	6.041122921	0.51988089
C9ORF47	0.017864848	6.094364273	0.519883579
LOC644574	-0.018451566	6.141141468	0.519924641
LOC643576	-0.01491795	6.042636189	0.51994438
LOC283682	-0.017280558	6.00872769	0.519953364
LOC148709	-0.016729372	6.043259326	0.519973019
LOC341112	0.018729889	6.15315578	0.52004981
RPS28	-0.059222756	11.89714944	0.52020525
OR9G9	0.01554398	6.035490869	0.520436861
LOC643910	0.019329686	5.985570049	0.520553946
SACM1L	0.046339569	8.398773095	0.520596918
TRIML2	-0.018080575	6.039327084	0.520609384
LOC643130	-0.014751474	6.078249801	0.520679534
LOC387927	0.017879326	6.302260517	0.520684901
BCL10	-0.013899728	6.446214869	0.520718613
MRPS2	0.025198027	8.576380336	0.520726482
LOC392617	-0.016552591	5.968659804	0.520819988
APBA1	-0.011025789	6.035434156	0.520850292
SYNGR2	0.014275682	6.082220415	0.520866634
PRF1	0.028250793	6.318723133	0.520906733
C14ORF138	0.018723767	7.146268008	0.520960583
MCPH1	0.031025722	7.017494158	0.521004073
WNT16	-0.01339722	6.380091482	0.521018918
BCL7A	0.01586286	6.111558887	0.521161296
LOC648521	-0.015694212	5.909800126	0.521197966
KCNK13	-0.026204978	6.1456534	0.521235025
TESSP1	-0.016242275	6.035448619	0.521245287
LOC642568	-0.015555282	5.8925317	0.521517457
FANCM	0.024994801	6.013656309	0.521654149
LOC650058	-0.015556385	6.156265927	0.521782946
GAS5	0.021073588	6.011871732	0.521787228
LOC728193	-0.015497223	6.247041196	0.521798447
C11ORF36	0.015324146	6.07513675	0.521822034
DDB1	-0.018579144	10.17080663	0.521840885
ZNF452	-0.012953367	6.040168575	0.521849047
LOC644736	0.014709538	6.02896329	0.52191321
MRPL15	0.042745999	11.13144683	0.521934592
OTUB2	-0.009941213	6.149608566	0.522102435
GLRA2	-0.016026896	5.978029176	0.522114408
IFNB1	-0.01965205	5.899546196	0.522196077
IL8RBP	-0.015335564	6.009759172	0.522225203
LOC124220	0.019643631	5.903233926	0.522245597
LRRC18	0.02024066	6.057789909	0.522267554
LOC440434	-0.013592643	6.042189106	0.522371024
PDZD11	-0.011906603	6.161500203	0.522432202
MS4A3	-0.009934099	5.958864776	0.522531587

LOC345537	0.017076337	6.055858292	0.522573533
LOC399829	-0.017805771	6.091916333	0.522586124
LOC644762	0.033581402	8.371332456	0.522590671
LOC389151	0.014803033	5.972712942	0.52261047
TTTY12	0.017887374	5.967952587	0.522655495
PARD6B	-0.017699664	6.172801201	0.522706621
PTPRN	-0.019030569	5.990436416	0.522716739
LHFPL1	0.026576429	6.013893603	0.522840218
LOC643075	-0.016764368	5.949858611	0.522970307
RAB11FIP4	-0.018886451	6.15944299	0.522974313
C17ORF67	-0.027620012	5.963837861	0.523002009
LOC200261	0.015925655	6.110490791	0.523051389
LOC643302	0.01645079	5.87842825	0.523102877
LOC643435	-0.016573716	6.039071333	0.52312087
LOC649255	-0.015623747	5.947967154	0.523195987
LOC641938	0.017311756	6.134691184	0.523220865
VCY1B	-0.016421073	5.989756619	0.523331685
LOC284293	0.0170886	6.11294226	0.523364202
LOC652155	0.019277818	6.204523794	0.52354976
LOC730226	0.016318869	6.41658179	0.523558694
FLJ37543	0.019336993	6.051243527	0.523559486
LOC642121	-0.016961484	6.014646931	0.523607576
OR7E156P	0.017368253	6.795485082	0.523851643
NLRP11	0.015792867	5.972191953	0.523864758
LOC652597	0.022239635	6.053248306	0.52393208
SPIB	-0.015909419	5.999857643	0.52399837
SPSB2	-0.03811282	6.635860571	0.524029961
LOC646697	-0.016793045	6.186103139	0.524073383
LOC401497	-0.018665735	6.362083564	0.524213926
OR2F1	-0.017768646	5.980732905	0.524264419
ENTPD7	0.021054854	6.076094573	0.524350982
HSPC159	-0.035094157	6.936573976	0.524446688
CCKAR	0.015952575	6.085389394	0.524480697
SCN4A	0.018855268	6.154207098	0.524510661
SNAI1	-0.01545645	5.940956356	0.524559158
LOC650147	0.01603491	6.124321141	0.524876667
LSM14A	0.023226374	9.869490931	0.52502878
OR10P1	-0.022095126	6.000628018	0.525033091
OR52E4	0.015508252	6.067767515	0.525147279
TMEM63B	-0.039679799	6.854500757	0.525419012
LOC440005	0.018943027	5.875680917	0.525426605
LOC442117	-0.01633722	5.981233031	0.525452263
LOC284260	-0.015488452	6.023263881	0.525522223
LOC642861	-0.017315418	5.933891709	0.525535339
LOC727855	0.015255261	6.152057424	0.525568771
PGRMC2	0.031235579	10.6061185	0.525581576
LOC442229	-0.015905542	6.108141688	0.525652017
TSSK3	0.017935209	6.505215864	0.525676363
CPA1	-0.018462723	5.975130541	0.525700082
LOC642726	-0.012633506	6.416261128	0.525747585
LOC392275	-0.009822905	6.075655682	0.525778244

CACYBP	-0.024016328	7.660961988	0.52578415
LOC727732	0.012232463	6.094448972	0.525849499
LOC347364	0.01564453	6.200806446	0.525901848
PSG11	0.014190516	5.976462542	0.526012289
CACNA1G	-0.007675029	6.011937884	0.526045683
LRIT3	-0.015661839	5.984925111	0.526067718
ZNF761	0.016013625	6.220879968	0.526093024
KIAA1957	-0.015855931	6.106720242	0.526149663
LOC730242	0.015236505	6.070584956	0.526158534
LOC647212	0.015568445	6.063093014	0.526247259
LOC649246	-0.014741452	6.160878046	0.52635369
LOC654172	0.011078051	6.043149348	0.526364512
LOC647447	0.007438083	5.994071492	0.526471047
PBEF1	-0.016402907	6.287439615	0.526515345
LOC653676	-0.015308208	6.143325627	0.526632538
CGB5	0.012324508	6.089927833	0.526657021
LOC644100	0.010811301	6.099632507	0.526810174
TSPAN6	0.020441245	6.906916066	0.526815189
LOC648868	0.014411591	6.072505876	0.526822978
SMR3A	-0.016449196	6.01444575	0.527078287
INTS7	-0.022832373	6.768785054	0.527087502
EIF4G2	-0.019218161	11.63187648	0.527114847
SPATC1	0.011029114	6.135536175	0.527119804
BRUNOL5	-0.015518394	6.061014672	0.527156782
FXC1	0.022971914	7.849987949	0.527173677
C19ORF45	-0.018749856	6.029978905	0.527317752
KIAA1644	-0.015924153	6.14422391	0.52740697
DDX52	0.022571621	7.129264667	0.527435263
HPX	0.018785115	6.103563322	0.527456553
METRN	-0.034552125	7.062649459	0.527469166
MGC16291	0.013609972	5.981699589	0.5274976
LOC653310	-0.01739266	6.018813985	0.527528236
CSN1S1	-0.013497797	5.993247691	0.52761764
GIF	0.015444773	6.084267845	0.527654586
CMPK2	-0.021557506	6.083536476	0.527662005
LOC642035	0.015942369	6.125110239	0.527668351
LOC646296	0.017066117	6.202605183	0.527693138
KRTAP23-1	0.015240414	6.080867169	0.527720588
LOC648894	-0.016475052	6.010268785	0.527795122
PYHIN1	0.010384653	6.149934562	0.5278141
LOC652408	-0.0152929	6.111190823	0.527934564
RAP2A	0.03859087	8.75641484	0.527990045
LOC641959	0.016331983	6.365837792	0.528160373
EFNA1	-0.04132266	9.326566623	0.528197366
LOC647521	0.016906312	6.308149782	0.528202054
LOC652471	-0.014351905	5.986464561	0.528205729
LOC645143	-0.014455773	6.072989632	0.528211942
GIGYF1	0.017678175	6.483833163	0.528274203
LOC399937	0.009971626	5.988202951	0.528275657
LOC345222	-0.016740587	6.064148119	0.528435498
LOC642223	0.015537797	6.048869356	0.528536885

DLX5	0.017630473	6.091233797	0.528597244
FLJ46481	-0.015715784	6.402609492	0.528661703
TNS4	-0.018283534	6.223953013	0.528677485
LOC653853	-0.011870355	6.064151367	0.528691844
LOC654127	0.016013132	6.054863531	0.528770239
C6ORF81	-0.025000905	6.796043123	0.52879512
SMOC1	-0.013253863	6.001603083	0.528798248
OTOG	-0.015582745	6.090657679	0.528827401
KIAA1285	0.027103323	6.299464461	0.528859606
IGSF8	0.018219885	6.253196292	0.528886469
TACR1	0.01021763	6.059666765	0.52892122
KRTAP4-11	0.016882048	5.977563771	0.52900858
BRDT	-0.010884539	5.940725131	0.529038361
SNX20	-0.011464836	6.084805125	0.52910916
OTUB1	0.017408984	7.031605873	0.52913074
PF4V1	0.016190917	6.347372547	0.529134863
UBE2E1	-0.029061469	9.839567848	0.529238994
LOC641848	0.084664254	8.461903737	0.529309531
C15ORF41	-0.053344418	10.45651571	0.529386125
LOC648625	0.015041502	6.06087457	0.529388154
MYH16	0.011436901	6.174181517	0.529394656
LOC644431	-0.016447824	6.0745157	0.529601842
LOC645411	-0.01515926	6.277968487	0.529637602
IFNA6	-0.017909151	5.90273977	0.529671445
NT5M	0.026654008	6.631709346	0.529747764
CSH1	0.01013658	5.980189789	0.52978198
EPB42	-0.017463701	5.975263736	0.52980492
LOC732156	-0.015446907	6.132428882	0.529897265
LOC648931	0.015454543	6.133103724	0.530001761
LOC647529	-0.020576524	5.980000071	0.530075583
RPL37A	-0.036391941	12.19421497	0.53014347
LOC390205	0.018536747	6.104368132	0.530165528
CRNN	-0.040565653	8.183375416	0.530198621
LOC728302	-0.017440417	5.993400788	0.530202711
FCGR2C	-0.018094749	5.892549435	0.530300068
GSTA3	-0.011791247	6.108840746	0.530437954
LOC256223	0.01535334	6.008743276	0.530532842
SLC24A4	0.010076296	6.180612227	0.530549218
LOC646818	-0.016838812	5.893543461	0.530602828
T	0.011851943	5.977246328	0.53062877
EHF	-0.015601702	6.22096208	0.530826804
LOC651481	-0.015651842	5.88334465	0.530845074
LOC649661	0.021961264	6.814543071	0.530851682
DECR1	0.038237741	12.21565559	0.530852595
SLC35A3	-0.023870335	7.383819042	0.530872502
MDM1	0.01314844	6.206745655	0.530951578
LOC651536	-0.016776594	6.171772769	0.530974296
STON1-GTF2A1L	0.014794594	5.980937194	0.531098726
LOC727942	-0.016666686	5.998211687	0.5311615
CCL22	-0.021547333	5.917130734	0.531256132
LOC642943	0.019532836	6.165858197	0.531286119

MMACHC	-0.033083923	7.554634257	0.531287215
KRTAP5-8	-0.01650345	5.91663129	0.531293189
SLC10A4	-0.012588504	6.060618521	0.531297674
GKN2	-0.018140691	6.052452047	0.531315283
LOC644946	0.012816012	6.049057692	0.53137931
TSHZ2	0.035357657	7.024778586	0.531422448
LOC731236	-0.016300711	6.101186879	0.531499212
AANAT	-0.01593957	5.917709032	0.531551268
POLI	-0.018284272	6.019793467	0.531598429
LOC653129	-0.017108856	5.991638809	0.531657504
PCDHGA2	-0.011446459	5.997782538	0.531766363
TACR2	-0.016883114	6.079749405	0.53180223
MAGEA8	-0.012917942	6.080439849	0.53187196
COX5B	0.037154989	13.90639196	0.531918907
AMIGO3	0.01675546	6.214336131	0.53193042
LOC730050	-0.023561938	6.402134671	0.53197485
SMC1B	0.017602224	6.153934118	0.532062528
KIR3DL2	0.017674385	6.060929869	0.532102784
SHOC2	0.04607665	7.377587756	0.532150829
YJEFN3	0.018918725	6.182409822	0.532158813
LOC401507	0.015791051	5.863126901	0.532456123
CYTH1	0.029486157	7.409460899	0.532493742
C10ORF64	0.019797774	6.157679622	0.532556333
OR2D3	0.0168616	6.028574864	0.532606142
LOC654133	-0.015019395	6.048488894	0.532698929
CACNG6	0.010797955	6.125273738	0.532727046
LRRC26	0.017549959	6.130804159	0.532802587
PAFAH1B1	-0.046138555	9.595693797	0.532822782
ASPHD2	-0.016924339	6.145224909	0.532919125
AGPAT5	-0.026823789	6.530964172	0.53299617
CYP4F11	-0.021487125	6.172013109	0.53305507
LOC728056	0.016470397	6.012822885	0.533258484
HIST1H1B	0.013189429	6.001551513	0.533306235
PLK4	0.017548893	6.333203337	0.53331619
SPANXA1	0.0112478	5.993536434	0.53338465
OR5H2	0.017899876	5.942185738	0.533429344
LOC401286	-0.01614616	6.082898815	0.533458376
DFNB31	0.015973817	6.18611155	0.533539685
LOC646914	-0.01532983	5.974499074	0.533741762
WDR53	0.021666119	6.714397292	0.533770241
FLJ12334	-0.017563922	5.999937709	0.534071401
DAAM2	0.032325748	7.273620994	0.534173076
LOC646154	-0.015467421	6.112503234	0.534210778
FLJ32214	0.017378046	5.955540348	0.534464304
FLJ44060	0.014878399	6.010980121	0.534654239
LOC647998	-0.017299906	5.991717889	0.53470266
ZNF410	-0.025300324	7.697235086	0.534708382
RNF133	-0.014640437	6.080649245	0.534726976
ASPN	-0.016515338	6.035603997	0.534939271
PEG10	-0.01176242	6.037328151	0.535033896
LOC650795	0.017216398	6.071692834	0.535049672

HLA-DRB5	0.18085049	7.020385809	0.535073878
API5	0.021282974	8.640567891	0.535111489
SPDYC	0.016479128	6.176970485	0.535209945
LOC644528	-0.014422084	6.206537527	0.53522083
MPZ	0.028513806	6.122629502	0.535353962
CCIN	-0.013847245	6.039569693	0.535392884
TAGLN3	0.023702195	6.246741234	0.535588026
LRRC56	-0.018115159	6.132348048	0.535594617
DOCK3	0.022177203	6.494086129	0.535658189
SPRR1A	-0.020959384	5.948294935	0.53570088
MANBAL	-0.022215084	8.285028916	0.535723427
LOC644836	0.015923225	6.195676037	0.535855536
LOC727820	0.018308431	6.591614751	0.535880908
UCP1	0.014900043	6.087910205	0.535893258
LOC644320	-0.01370031	5.941443629	0.535952355
DUSP8	0.041463243	7.466622066	0.535958633
IQSEC3	-0.012955766	6.174474666	0.535992249
GPR81	0.012060402	6.030715172	0.536059067
LOC645908	-0.01719873	5.892865214	0.536069398
LOC644809	0.016172576	6.162396278	0.536092052
LOC653089	0.014994458	5.977388811	0.536286864
OR5R1	-0.016341653	6.100740023	0.536298695
LOC654342	0.023349512	6.530427404	0.536339428
PTPRH	-0.024848705	6.334902983	0.536382773
CEP97	0.016111413	5.906827683	0.536431048
LOC388948	0.01415001	6.131158326	0.536512788
CYTH3	0.019674006	7.34703269	0.536678301
LOC651450	0.017853178	6.132196108	0.536752763
FAM83A	0.010741098	5.974683075	0.536755335
ZRANB1	0.041162076	7.977891922	0.536830212
CNTD2	-0.015017574	6.148065745	0.536969096
VILL	0.017825894	6.307755887	0.537013295
C10ORF30	-0.018925018	6.089419269	0.537033714
TAF13	-0.021054984	6.521143674	0.537112122
CABP4	-0.014872388	6.059743368	0.537126293
LHFPL4	-0.016535782	6.088434357	0.537132087
YWHAG	-0.032950524	9.83741924	0.537147366
SLC30A4	0.012074578	6.004206151	0.537234471
C1ORF173	-0.014528303	6.065436182	0.537265094
ZNF257	0.013480371	5.971310426	0.537301394
PFN2	-0.036256288	7.878635624	0.537405495
LOC649442	0.016882785	5.951938342	0.537465139
LRRIQ4	-0.019627988	5.917445362	0.537763791
LOC147645	0.016991092	6.10763531	0.537841121
STK16	-0.014256779	6.226842294	0.537880016
NPAS1	-0.017847771	6.112886641	0.537905192
C21ORF129	0.018609863	6.036662401	0.537906718
EDG4	0.019061558	6.533299201	0.537984335
LOC647264	-0.011844098	6.032911865	0.537992619
FBXO24	-0.00930091	6.057188787	0.538052594
HMGCLL1	0.008957562	6.088583734	0.538135019

HLA-G	0.043475779	7.332215333	0.538160977
LOC646720	0.014945361	5.990289197	0.538226712
ATP6AP2	-0.032427344	10.00507861	0.538241287
GPX5	0.010441058	6.077792817	0.538523899
TLR10	-0.009779957	6.108771104	0.538546731
LOC342900	-0.010646826	6.047118655	0.538564538
LOC645144	-0.016026194	6.030017656	0.538590345
N4BP3	-0.017276107	6.091194196	0.538626764
MRPL9	-0.025213034	7.552255344	0.538720245
C1ORF31	-0.041417976	7.344075226	0.538745904
KIAA0774	-0.047624631	7.568992457	0.538865708
PDZD3	0.018756715	5.968442007	0.538882024
JARID1D	0.125185119	7.053736321	0.538917728
BZRPL1	-0.0152638	5.92287855	0.538934371
SNORD116-4	-0.018222829	5.919505448	0.53895395
LOC440287	0.017463741	6.027856769	0.539023045
FSD1	0.007337606	6.123766268	0.539048208
ADRBK2	-0.020086069	5.972734361	0.539084485
UTS2R	-0.016399753	6.099715129	0.539166001
LOC391025	-0.01537587	6.087342627	0.539238849
LOC391209	0.015752954	6.027614554	0.539243297
LOC653180	-0.014967667	5.90771252	0.539253158
RSAD2	0.051720488	6.860130743	0.539336589
UTP23	0.019335264	6.962542715	0.539337878
SCGB3A2	0.018486981	5.936221839	0.539360115
LRRC57	0.019446053	6.966722124	0.53942473
CGN	-0.014885881	5.990222944	0.5394508
LOC648211	-0.015146046	6.17132936	0.539517182
PCDHA2	-0.011700313	6.055234631	0.539557089
C4ORF28	0.018760935	6.061214452	0.539774389
LOC646999	0.014937384	6.053953426	0.539789304
MLLT1	0.017222824	6.162105767	0.53983446
LOC649700	0.014967916	6.114315765	0.539920596
LOC652707	0.016702154	6.157285029	0.539927135
SNX31	0.018346858	6.243985826	0.540068066
MYH8	-0.015046366	6.033798024	0.540113693
OR1L3	0.017786265	5.931243967	0.540193988
FCRLA	-0.019232222	6.128775019	0.540240239
LOC653799	0.014370824	6.178200244	0.540246963
IGF2BP2	-0.037644745	8.297202054	0.540311879
KLHDC8A	0.019799569	5.948368997	0.540323387
BLOC1S1	-0.050710461	9.22069785	0.540431139
CYP1A1	-0.035675174	6.185258749	0.54053394
LOC651586	0.013916768	6.069957793	0.540574724
EFHA1	0.033433996	10.63879859	0.540620026
A26A1	-0.012561534	6.064079419	0.540641678
C11ORF2	-0.030455583	10.68856804	0.540816414
LOC645951	-0.016575974	6.045817193	0.540924063
LOC653468	0.009927332	6.171718427	0.540932288
LOC649762	-0.014045199	6.113843726	0.540978549
SNORD26	-0.015054929	6.029592198	0.540982863

SECTM1	0.023322102	6.174867561	0.54103317
PAIP2B	-0.017592644	6.133332847	0.541076384
LOC645128	-0.015918478	6.225682335	0.54107985
PM20D1	0.020312554	6.21036297	0.541094921
LOC642751	-0.01384224	6.082422634	0.541699767
LOC650549	0.014629691	6.110627682	0.541713933
FLJ35880	0.01659553	6.134442813	0.54172226
TFG	-0.029305547	10.00660833	0.541834507
C20ORF85	0.017235147	6.156688562	0.541880379
CCL4L2	-0.012879603	6.139693225	0.541922554
TRMU	-0.025477938	7.12851179	0.541925586
C1ORF70	0.022596023	6.03274854	0.541956562
LOC646320	0.014040669	6.050681557	0.541978691
LOC645885	0.014304321	6.074480364	0.542016934
CRX	0.018303439	6.275012522	0.542025364
LOC647031	0.016112977	5.983428126	0.542100764
FREM3	0.013627947	6.081587276	0.542101466
LOC653441	0.009029163	6.011834741	0.542102846
ABCC9	0.020381784	6.746283077	0.542166869
LOC198437	0.017793752	5.915695638	0.542186796
COL12A1	-0.028527096	7.255128384	0.542290018
ZNF776	0.021958711	6.643008008	0.542290284
LOC643045	0.017672664	6.182991193	0.542344722
DHX29	-0.02405121	8.697663228	0.542372237
LOC643692	0.013625779	6.05381788	0.54242867
LOC643899	0.016489568	5.999929046	0.542443795
OR5M11	-0.015236214	5.99361322	0.542461952
OR8G1	-0.0130947	6.018662897	0.542465749
LOC644484	0.015557393	5.895506527	0.542720578
PRX	0.010893523	6.144675424	0.542794463
LOC642615	-0.016257044	6.284747071	0.54279897
DPF2	0.020504614	9.11159844	0.54282685
LOC647850	0.014293914	6.163678875	0.54293539
LOC647046	0.013743735	5.999621037	0.543007049
PRKAA1	0.02353285	8.252540043	0.543072008
OAZ3	0.022572271	6.661681242	0.543216543
LOC643906	0.015499959	5.931204374	0.543326126
LOC286076	-0.022194003	5.959436005	0.543392581
TTBK2	0.016885716	6.159280953	0.543605915
LOC654080	-0.014292479	6.020701825	0.543649523
PAGE3	-0.015766072	6.094505501	0.543720204
ZNF461	-0.020355313	6.022424889	0.543765321
LOC644200	0.015023448	6.05048839	0.543781924
CCDC5	0.016481385	6.451296198	0.543810988
LOC646139	0.016346636	6.018647074	0.543841158
C2ORF51	0.015299412	5.944054677	0.543880824
LOC653980	0.010213799	6.020184404	0.543957029
OR4F3	0.016817917	6.12877761	0.54396448
OR4C13	-0.01519293	6.017164323	0.544058639
MGC34800	0.012971106	6.045897331	0.544184889
GSTZ1	0.019305428	6.714071826	0.544399234

LOC642231	-0.014032916	5.920559188	0.544427949
LOC653722	0.016809815	6.014622304	0.544523445
SAP30BP	-0.023150633	6.801511782	0.544570491
C8A	-0.015797699	5.959757403	0.544590037
IGF1	0.029778611	6.580354562	0.54460468
OR2C3	-0.017783125	5.992244859	0.544717496
GPR158	0.018776322	6.072727388	0.544794522
GZMB	-0.032230299	6.32805225	0.544890794
FRAG1	0.037924796	8.425189384	0.544913172
B4GALNT1	0.02498581	6.318675938	0.544921839
LOC645118	0.016540162	6.175307132	0.544973753
ESPNP	0.013877617	5.914583182	0.545039176
LOC728441	-0.016589748	6.005109517	0.545051825
LOC652168	-0.017353133	6.099443001	0.545064114
C14ORF166	0.029268784	10.49314983	0.545066235
LOC642765	0.014333869	6.247586713	0.545091043
LOC641750	-0.012074148	6.036868311	0.545113317
LOC388882	-0.01969132	6.068680753	0.545130437
GKN1	0.015637296	6.057580097	0.545174334
LOC642731	-0.015966239	6.06441707	0.545242717
CD1C	-0.022410805	6.371955336	0.545451019
LOXHD1	0.021211695	6.190836158	0.545538014
LOC730184	0.015398655	6.314293543	0.545640374
C9ORF50	-0.016208696	6.036927296	0.545728539
LOC650278	0.016445385	6.126492767	0.545931811
GH2	0.008539309	6.03557862	0.545967756
FAM75A3	-0.013766208	6.038651654	0.545968598
CAMKV	0.017132167	6.017490537	0.546002095
TMEM41B	0.030150953	9.204987051	0.546063831
TNKS2	0.022229123	6.058369417	0.54609078
LOC730240	0.017554143	6.238885333	0.546149082
LOC651208	-0.013940988	5.98575428	0.546181534
DPEP3	-0.017093698	6.138042906	0.546358655
TBC1D13	-0.022624666	7.700286781	0.546364254
IGLL3	0.014262597	6.106374526	0.546376226
LOC645701	0.013964244	6.039595218	0.546445068
E2F6	-0.021605456	7.636285006	0.546487712
WBP2NL	-0.016046997	5.93073023	0.546586987
HNRNPCL1	0.011206121	6.041544539	0.546594442
MTPN	-0.039505204	10.31957601	0.546621151
LOC392232	0.01558652	6.055101643	0.546687741
LOC653598	0.015712052	6.17562455	0.546687766
FLJ32955	-0.017160438	6.381972117	0.546703914
LOC642960	-0.015795583	6.428609528	0.546733197
AGXT	-0.016916911	6.114346878	0.546796739
COPS7B	0.020228545	8.267774846	0.546838016
LOC646762	0.021159261	6.070597609	0.546860408
CYP11B1	-0.012179332	5.914785181	0.546898073
FLJ46321	-0.010912697	6.07864333	0.546909985
LOC644122	0.010670289	6.016192581	0.54698993
ABHD9	-0.016130653	6.287538411	0.547058648

KIAA0258	-0.03361194	6.353617734	0.547225849
DDX55	-0.034585415	7.44756432	0.547281029
MORF4	0.016381056	5.978860106	0.547306122
LOC647171	0.012477888	6.116581485	0.547517898
LOC642527	-0.016395265	5.966820853	0.547741082
LOC642830	0.016104474	6.013222147	0.547759653
NFKBIL2	-0.015776805	6.182074699	0.547845692
MFGE8	0.030378166	13.29918216	0.548011073
LOC199882	-0.015773973	5.98499071	0.548022272
PGAM1	0.04332401	9.336114596	0.548040434
LOC388080	0.012314424	6.122697844	0.54806863
C4ORF22	-0.016802656	5.970052763	0.548073769
OTP	0.011232914	6.228119551	0.548269835
LOC651714	-0.015506626	6.210540629	0.548424765
ACTC1	-0.037973533	13.89921021	0.548513978
LOC646179	-0.022370318	6.008771043	0.548522231
LOC644491	0.015662097	6.329181239	0.548546617
GPR32	-0.016336277	6.025938315	0.548649828
RABEPK	-0.049721206	8.608525777	0.548730206
LOC392262	-0.013863203	5.997035041	0.548767457
LOC644783	0.013306554	6.047983191	0.548799517
ISLR2	0.031177658	6.738736084	0.548967653
LOC644701	0.029547252	6.060940634	0.548969527
VAMP4	0.023357456	6.95049175	0.549023678
LOC152578	0.014892154	6.101477656	0.549029553
WT1	-0.01510622	6.352364378	0.54903982
FLJ45079	0.016993339	6.105967395	0.549097005
RNASEH2C	-0.015786012	6.398114027	0.549126127
SEMG2	0.015303843	5.862427853	0.5493234
CHCHD10	-0.026465302	13.21846499	0.549422452
LOC643365	-0.017442773	5.935840982	0.549427658
AKIRIN2	-0.034354869	9.12511092	0.549442459
LOC653664	0.014839659	5.963714541	0.549452801
LOC439965	0.017893326	5.896345974	0.549468607
ASAH2B	0.010985744	6.214714787	0.549472452
LOC654101	0.014175273	6.110156084	0.549492442
PRAMEF4	0.011164583	6.037867124	0.549528462
LOC644887	0.014808451	6.059687819	0.549561804
TSPAN15	0.022027434	6.64732743	0.5495739
PTPRV	-0.015871275	6.032424982	0.549710613
OR6N1	0.014936165	5.977374861	0.549735037
LOC642692	-0.015796467	6.254919392	0.549878835
LOC647096	-0.014655796	5.958291758	0.549967083
LOC442512	0.015073732	6.076332935	0.550104463
LOC440225	0.01202861	5.959415393	0.550108957
OR6C68	-0.015126566	5.901992998	0.55022269
LAMB4	0.015988946	6.17097254	0.550247721
LOC646307	-0.014451021	6.09441654	0.550277929
LOC643998	0.015634903	6.219806432	0.550357847
ITLN2	0.017508714	6.129610248	0.550526854
TMEM180	-0.017383977	6.419132494	0.550604831

COTL1	0.028982278	6.023582436	0.550631659
MYCL1	0.015554729	6.125634456	0.550638927
C7ORF31	-0.014993583	6.023667998	0.550788692
GPR128	0.025119986	6.111010237	0.550804048
INHBA	0.018084121	6.062900382	0.550851727
CTCFL	0.014757691	5.979544647	0.550871732
LOC642257	0.015435382	6.010045103	0.550977365
THBS2	0.068022115	9.02997437	0.551130991
LOC644255	-0.014277422	5.993691515	0.551155391
TMEM176A	-0.062756688	7.879987686	0.551214741
PAPSS1	0.027191025	7.413985199	0.551222278
LUC7L	-0.016920983	6.903368502	0.551244114
MAGEA2	-0.009768633	6.062300049	0.551297613
FAM153C	-0.010692867	6.0284495	0.551391478
LOC648434	0.017826915	6.345922133	0.551452235
C6ORF165	0.009156815	6.130855107	0.551476614
CYP3A43	0.011875965	6.019272846	0.551578657
LOC651112	0.019937951	6.140148237	0.551590211
MCF2L	0.014720103	6.002763166	0.551675284
AIM1	-0.021164177	6.447588901	0.551693281
ANGPTL3	-0.01099181	6.035306752	0.551703832
LOC647215	0.017022505	5.977510367	0.55174537
PRKAG3	-0.015827326	6.082957498	0.551875309
LOC650965	0.014936679	5.979414038	0.551901133
SLC18A1	0.012594287	6.289821997	0.55191543
KRT37	-0.018633261	6.106297623	0.552068365
CENTB2	-0.015599238	6.899141828	0.552200015
FLJ43093	-0.016646534	6.023169793	0.552228061
VPRBP	0.021122227	6.753642106	0.55223218
BAZ2A	-0.015864501	6.218335881	0.55233139
RINL	-0.019368633	6.591246096	0.552436483
SPRR2D	0.010309426	6.033444021	0.552438288
LOC441016	-0.015280909	6.302132051	0.552444363
LOC647584	-0.015001944	5.962644462	0.552580022
LOC645730	-0.01611384	5.943101148	0.552606153
LOC442474	-0.014462126	6.204049522	0.552663857
FBN2	-0.065714129	8.656944323	0.552682553
LOC440687	-0.014260185	5.890955513	0.552739583
C9ORF37	0.029133342	6.881885585	0.552897205
THADA	-0.014330871	6.964107138	0.552993061
LOC648405	0.014764734	6.228374509	0.553115513
AQP1	-0.04127428	7.250773633	0.553160648
LOC649641	-0.01759664	6.149924896	0.553197794
LOC400858	-0.01428862	6.036378403	0.553214497
FTHL11	-0.052656111	10.68608986	0.553402912
LOC651842	0.014563029	6.112224683	0.553445012
LOC648164	0.010748212	6.021650478	0.553479092
LOC390669	0.016992734	6.086016673	0.553497743
LOC641697	0.014735056	6.070832811	0.553525361
KCNQ1	-0.009925952	6.351819694	0.553772534
DKFZP564N2472	-0.016205639	6.099617679	0.553860588

LOC647246	0.014941886	6.045753247	0.553873454
SLC35E2	0.015963031	6.196367223	0.553896238
TRPV5	0.011635647	6.057739262	0.553924294
FAM26C	0.022192308	6.975205941	0.553971956
FLJ44290	0.011681621	6.10455633	0.553981589
MAP2K7	-0.019906035	7.037503262	0.554009218
GRIN2B	0.023181837	5.980820816	0.554015137
SPINK6	-0.010523329	6.053563478	0.554039388
C11ORF49	0.012613114	6.418679255	0.55405048
MB	0.033127858	13.74810705	0.554071171
LOC649212	-0.016761209	6.030352832	0.554096548
KCTD16	0.01602074	5.944245342	0.554123335
LOC442446	0.015927301	5.997915203	0.554190731
CDC20B	-0.015434603	6.217023263	0.554205008
RAB2B	0.027387477	9.740807057	0.554324521
LOC441722	0.014599956	5.957046552	0.554433533
KRT38	-0.015815737	5.925019622	0.554535813
LOC650429	-0.015919047	6.074479673	0.554609813
COX4I2	0.03253817	7.623604617	0.554649114
IL13	-0.017007737	5.857503013	0.554707822
FLJ41481	0.023441037	6.102922183	0.554732554
TNFRSF19	-0.034347345	8.312088582	0.55477581
ATAD4	0.022665169	7.418128711	0.554881378
PNLIP	0.016194349	6.147193548	0.554910108
ZNF74	0.014194794	6.489773562	0.554931218
IYD	-0.016338555	6.004657694	0.554934035
LOC727937	-0.014002396	6.213615411	0.555008392
SPZ1	-0.016440491	6.115993335	0.55511074
TAT	-0.013571075	6.038976145	0.555152099
ENTHD1	0.02074301	6.007401216	0.555298096
LOC645278	-0.016374772	6.118953104	0.555405092
LOC648878	-0.014135348	6.121483132	0.555416056
DIO3	-0.021084116	6.127912698	0.555417623
FTHL17	0.016186245	5.974276912	0.555522922
ALDH1B1	-0.031625181	6.835211185	0.555579638
NFATC2IP	0.012091914	6.337291008	0.555606808
LOC199897	0.02263758	6.037271849	0.555668304
LY6G6C	-0.025906929	6.0001057	0.555668942
PRNPIP	-0.016332559	7.098029403	0.555690471
OR2AT4	0.016129457	6.017739732	0.555706225
MEIS2	-0.025073311	7.390144102	0.555853018
ASAH2	-0.009719693	6.121094546	0.555914077
PKLR	0.008582698	6.098470004	0.555966821
LOC646867	0.015370059	6.048188223	0.556025139
OR2W5	0.016739822	6.476209029	0.556025215
LOC392440	0.015329315	6.12273905	0.556084727
CEACAM19	-0.018183421	6.213589837	0.556130536
NOS1AP	0.014410717	6.196470559	0.556158234
LOC652813	-0.01602858	6.038356444	0.556261843
OR52N5	-0.015730749	6.14648154	0.556270198
SPATA8	-0.017252623	6.169706849	0.556338103

PIP5K1B	-0.020729356	6.772958107	0.55637308
IGFBPL1	0.023546736	6.072150452	0.556402198
SLC7A14	0.016313928	6.059822202	0.556406141
DUSP15	0.011058709	6.085676913	0.556411728
LOC642464	-0.013960475	6.117938152	0.556470318
GGTA1	-0.016379275	6.14760072	0.556493428
TEX2	0.024497096	9.690214276	0.556502853
BEST2	-0.018375269	5.949688372	0.556523246
LOC652757	-0.010952917	5.969710004	0.556583062
DBF4	-0.014593179	6.154904246	0.556596542
NEUROG1	0.015174982	6.115144189	0.556632774
GPR1	-0.039724369	8.970310182	0.556695001
PLXNB2	0.024715507	7.792359832	0.556701279
LOC646237	0.015380004	6.018169757	0.556744853
LOC648604	0.01549924	5.898884219	0.556776891
LCA5L	0.016079206	6.200439887	0.556947131
TMEM186	0.031571042	6.410626323	0.557091931
LOC401398	0.02029886	5.925725539	0.557532076
NFKBIE	-0.016635251	6.451577855	0.557624258
LOC650465	-0.013633582	6.170218971	0.557711133
OR10K1	-0.015186753	6.009267734	0.558025703
KIF25	0.014100285	6.515621736	0.558155006
TAL1	-0.02468844	6.152145385	0.558193423
TAAR1	-0.009990122	6.217543605	0.558297402
CCDC64	0.014018588	6.038225136	0.558326664
C5ORF20	0.016504183	6.242426429	0.558452736
HHLA2	-0.01624098	5.924904651	0.5586646
LOC652747	-0.014974046	5.993183637	0.558671887
LOC650151	0.012692248	6.06178074	0.558709284
PRRG3	-0.014316134	6.170444914	0.55877611
LOC645052	-0.013500467	5.984483965	0.558780832
DPEP1	0.014633361	6.074000415	0.558786741
AKR1CL2	0.014889517	6.087649237	0.558831634
OR2K2	-0.016289931	5.93954759	0.558866907
USP47	0.026367114	8.270913333	0.558929615
NRG4	-0.015184743	5.983235909	0.558934076
LOC645652	-0.013590149	5.994108041	0.558999356
METAP1	0.027658373	10.37227526	0.559125127
LOC643529	-0.014462851	6.066610806	0.559159863
WBSCR28	0.014646212	6.090091501	0.559186667
SCN1B	-0.031380422	7.148054446	0.559188477
NDUFS8	-0.026327625	12.7599795	0.559237897
LOC401620	0.023894867	6.053438515	0.559294533
KIAA0562	0.026056271	7.088687622	0.559368863
LOC652547	0.013691378	6.193321489	0.559390036
OR2G6	-0.012664539	5.957311153	0.55940931
TULP1	-0.017482473	6.019361352	0.55942198
LOC730996	0.025638598	6.629009064	0.55942946
NPEPPS	0.017032886	7.435451521	0.559488511
C3ORF22	0.014110931	6.222281329	0.559626264
CKS2	-0.037969974	7.092432731	0.559724509

OR2W1	0.016201597	5.818951693	0.559745736
LOC644686	0.014383834	6.408514181	0.559899013
LOC649185	-0.014258151	6.032446681	0.559933377
LOC651643	0.012474882	6.203429538	0.559948029
ICHTHYIN	-0.010852493	6.071811438	0.559980255
MGC34821	-0.015421526	6.033220597	0.560182688
LOC647661	0.014845642	6.021073913	0.560370966
TRDMT1	0.012636355	6.3391922	0.560567029
ZC3H7B	0.019304337	6.197067562	0.560628718
NTAN1	-0.024825146	7.835276652	0.560776899
LAPTM4B	0.038656316	11.94405095	0.560787234
GAPDHS	0.00950417	5.962566986	0.56081665
LOC646509	-0.017050739	6.234486431	0.560913619
OR14A16	0.016212612	5.957764307	0.560914418
LOC647149	-0.015728647	5.889681311	0.561020155
LOC221091	0.015419157	6.100127551	0.561081818
LOC644950	-0.015021912	5.961029281	0.561092062
OR4L1	0.010566755	6.111786179	0.56111755
OTX2	0.007615801	6.126738629	0.561132102
ZNF705D	-0.008826541	6.159597933	0.561245316
MTMR9	0.028292626	9.199832898	0.561362134
RANBP17	0.012896844	6.029721546	0.561381858
LOC647989	0.013500845	5.936753683	0.561383591
LOC644473	0.013991948	6.181594984	0.56149747
LOC646862	-0.015082638	6.08013244	0.561835194
LOC652741	0.019109792	5.9898771	0.561841525
LOC651807	0.016868532	6.465002405	0.56192983
LOC642037	0.01312579	6.060227868	0.561955466
LOC642097	0.01877303	6.01835078	0.561972303
DAB1	0.01498283	5.874666916	0.561983921
KLK8	0.011430005	6.085480604	0.562170478
TUBB1	0.016836094	6.135994788	0.562204333
LOC642537	-0.014207307	6.110387129	0.562586981
LOC645238	0.015116732	6.019841436	0.562626458
KLRC4	0.016408365	6.252138664	0.562651429
MASP2	0.008458812	5.998934058	0.562687107
LOC728656	-0.014354956	6.196618625	0.562692263
KLRC2	0.012309051	6.029890301	0.562886349
XAGE-4	0.013007104	6.067077168	0.562890772
LOC285423	-0.010802013	6.139694416	0.562941203
LOC642222	-0.013449162	6.147846002	0.562975267
HTR1E	0.018238121	6.490718334	0.563042385
LOC648863	-0.018853867	6.152938266	0.563044745
LOC651096	0.012589169	6.020440852	0.563123197
PDSS2	-0.026285932	10.33953225	0.56314258
LOC642405	-0.017098668	6.0595761	0.563143766
SUZ12	0.024814809	9.921945988	0.563215675
THUMPD2	0.019214071	7.340801355	0.563238671
FLJ44955	-0.015494687	6.081945401	0.563394672
LOC644856	0.013994079	6.037667912	0.563440701
ZNF774	0.028027052	6.381032248	0.563466618

HOXA7	-0.016810366	6.091604844	0.563490947
LOC390243	-0.011303162	5.991000544	0.563498515
C16ORF73	0.01419193	6.076205665	0.563562577
OR52A1	0.015957785	5.964849799	0.563721895
LOC642974	0.013678904	5.927740016	0.563730973
LOC645522	-0.016360758	6.057711425	0.563786181
SCUBE1	-0.015904954	5.948273946	0.563845891
RAB1B	-0.018385501	7.296015099	0.563961532
MRPS22	0.021656017	10.78543888	0.564043034
RNF183	0.017655545	6.167854476	0.564050124
DDX4	-0.014969917	5.997690193	0.564056731
FLJ44385	0.016099585	6.051629244	0.564060038
KIAA0368	-0.022442282	8.75785257	0.564068352
LOC729966	0.01420301	6.086453908	0.564140806
SLC9A11	0.011431814	6.032607161	0.564187447
MIF	-0.038366431	11.48018994	0.564198937
LOC648568	-0.014658315	6.054889064	0.564381213
LCE2A	0.019553604	5.995104484	0.564544916
LOC653983	-0.015277378	6.118199244	0.564558231
RLN3	0.019113256	6.446766062	0.564571542
LOC390846	-0.014276457	6.155210025	0.564581516
SERPINA11	0.010268983	6.07327973	0.5646
ARPM1	-0.018050333	6.217406999	0.564695048
DNAJC5B	0.014193351	5.889210224	0.564733485
MAGEA12	0.012532612	6.226004041	0.564742401
HISPPD1	0.014191288	6.010177664	0.56490067
LOC646848	-0.01550768	5.944924667	0.564913434
A26B1	0.016245368	6.036981513	0.564914673
LOC643948	0.016559477	5.998910263	0.565033936
MPP7	0.015890304	6.336175579	0.565046569
LOC645232	-0.013403345	5.997920617	0.565181603
RPL5	-0.032541172	12.172336	0.565183149
ZNF205	-0.013527359	6.370878757	0.565211833
ANKRD56	0.016594848	5.926020889	0.565216594
RBM8A	0.017641764	6.286425864	0.565238461
PPYR1	0.014409821	6.000908774	0.565245423
LOC653211	-0.012495418	6.161592965	0.56542217
IBSP	-0.016255494	6.224700001	0.565429934
FLJ43879	0.013865449	6.234402883	0.565573951
LOC648487	0.01309083	6.089197007	0.565588942
BTBD8	0.009924761	6.009171046	0.565612894
LOC133491	-0.017292253	6.191892047	0.565620192
LOC644934	-0.03828146	10.60817059	0.565629517
FAM84B	-0.036152073	7.520154127	0.565630864
LOC648256	-0.012447306	6.290008559	0.565635402
LRRC30	-0.013185992	6.108265993	0.56579778
TMEM168	-0.021591606	7.327629911	0.565880334
LOC644155	-0.014689321	6.058042413	0.565938781
LOC653620	-0.021524981	5.990810208	0.566138728
TAS2R5	-0.016445174	6.130757586	0.566216747
LOC652870	0.01364322	5.995044621	0.566336544

TOMM5	0.03712216	8.756130235	0.566361655
ADRB2	0.03794684	7.810129323	0.566365274
SP6	0.018873326	6.31761359	0.566419223
NFKBID	-0.015404266	6.239834598	0.566427793
LOC646793	-0.016603476	6.031528203	0.566460345
MBNL2	0.041194192	9.073911772	0.566565991
FTMT	-0.014626937	5.989463833	0.56660946
KLHL14	0.01101995	5.961918217	0.566617968
PAX1	0.015251259	6.178381282	0.566645028
LOC644063	0.044571635	7.613661593	0.566650762
LOC643464	-0.013671994	6.082121808	0.566687204
LOC497190	-0.013830909	6.145592411	0.56683836
LOC442245	-0.015244209	6.248840722	0.566886068
HIST1H2BM	-0.017237839	6.182354293	0.566953828
CYP4F22	0.014660187	5.93868774	0.567261479
ARSH	0.012710247	6.016957887	0.567372123
HNRNPA3	-0.041653643	6.756903014	0.567372693
WFDC10A	-0.013996085	6.095161118	0.567487249
DEFA5	0.014682712	6.047770829	0.567494954
LOC651921	0.013689993	5.983973361	0.56760694
ADAM18	-0.014827678	6.038769523	0.567607663
C1ORF27	0.017866888	6.146048544	0.567726833
XAGE2	0.010893307	5.949237488	0.567794344
LOC441632	0.015048409	6.223548483	0.5679196
FLJ46109	0.010268521	5.951558641	0.567921469
NUP43	-0.027642095	8.458303853	0.567934607
GPRC5A	-0.015305517	6.073697361	0.567940139
SLC27A2	-0.015860755	6.165651448	0.567953835
TBX22	-0.01103077	6.22334714	0.56796137
CCDC16	0.020756402	7.908675654	0.568094096
OR2B11	0.013711865	6.019933112	0.568172189
LOC652286	0.014241483	6.12173167	0.568199067
OR13C2	-0.015252416	6.002984603	0.568263713
TSKS	0.013500387	6.048248401	0.568285387
RASL10A	-0.020960969	6.51137498	0.568335594
LOC647946	0.009923129	6.037272581	0.56836792
LOC645138	0.044843953	11.26257482	0.568479353
ARGFXP2	-0.010086519	6.00596689	0.568531597
UGT2B15	-0.009975635	5.961605215	0.568551611
LOC129870	-0.013118869	6.03405602	0.568569475
WBSCR23	-0.018906475	5.928328178	0.568702664
LOC650118	0.01245036	6.016816974	0.568793665
FLJ42953	-0.013680118	6.05114007	0.56882169
SCEL	-0.009841244	6.092979158	0.568989217
OR8B3	0.014418802	5.948242441	0.569036004
HSDL1	-0.0260427	7.558945344	0.569096981
EIF4G3	0.027932996	8.495534032	0.56912278
SNORD42B	-0.01390589	6.10535925	0.569138086
COL1A2	0.070173768	9.99797938	0.56919381
LOC652802	-0.01559697	6.009514296	0.569200666
PPP1R1B	0.010945207	6.2132889	0.569433968

SEMG1	-0.016359671	6.464346811	0.56962648
LOC650862	-0.015744401	5.968585601	0.569650517
KRT83	-0.014900772	6.277977479	0.569675482
PRKAG2	-0.030339815	9.001818762	0.569736724
SLC26A10	-0.015950125	6.275858394	0.569766233
KLHL31	-0.019470041	6.087322808	0.569787914
MINK1	-0.016107772	6.003465746	0.569813117
HIVEP3	-0.015331088	6.094959599	0.569831464
UGT1A1	0.015111998	6.031785314	0.569932766
DNM3	0.017272945	6.261885632	0.570069905
ZSCAN29	0.014955413	6.247609321	0.570100673
LOC641914	-0.01393472	6.050262967	0.570275097
LOC643583	-0.015443003	6.023444627	0.570343092
HIST1H2AM	-0.015026705	6.037944943	0.570345639
LOC641972	-0.018759841	6.143986365	0.570437538
LOC650276	-0.024313226	13.44070924	0.570484448
NGB	0.023764706	7.072494579	0.570560198
LOC731992	0.013400218	6.115089517	0.570578528
RAP1GDS1	0.017166408	6.922639144	0.57063481
FOXL1	-0.014079001	6.086184712	0.570711963
RAB21	0.042538364	10.2798808	0.570740491
TNK1	-0.015662945	5.974241959	0.57085038
LOC653526	-0.014679827	6.066842831	0.570895623
AZI1	0.022262882	7.658770978	0.570931568
ATP1A4	0.010612683	6.044860368	0.571049541
G6PC2	-0.009056179	6.025151607	0.571137927
LOC729766	-0.016449594	6.037598471	0.571173953
CES7	-0.015594895	6.166198924	0.571175673
IFNE1	-0.009894871	6.043964755	0.57121308
LOC652401	-0.013794478	6.086583207	0.57121571
OR56B1	0.020771476	6.069607911	0.571830666
LOC441743	0.025438966	6.762034083	0.571988304
HTN1	-0.017326954	5.835539591	0.57202207
LOC654147	0.017338292	6.256467246	0.57205765
KRTAP12-4	-0.013511219	6.118339905	0.572063708
C17ORF55	-0.015432618	6.146598292	0.572308194
MANEAL	0.008667084	6.000052564	0.572324705
LOC442299	-0.014581908	6.024516584	0.572332909
PTPRCAP	0.016286403	6.375451971	0.572382202
NBPF15	0.008837924	6.003943721	0.572403017
LOC652463	-0.01378777	6.080456532	0.572535637
CLEC4E	0.015772763	6.167815166	0.572623482
CCDC46	0.016580925	7.025888857	0.572714256
TRAIP	0.01887909	6.404817188	0.572718475
LOC653472	-0.015715264	6.037336861	0.573102128
LOC647537	0.012978511	6.024063374	0.573232154
ACY1	-0.025577759	7.990419689	0.573256832
COP1	0.011655437	6.187412196	0.573266298
KLK13	-0.014113315	5.888296949	0.573295236
POU2F3	0.010137279	6.068101465	0.573370059
LOC643547	-0.016200176	5.938860425	0.573511326

KLF8	-0.021146691	6.810454772	0.573577771
WDR65	-0.020999806	6.064582197	0.573798751
LOC651860	-0.014197076	5.993101349	0.573847304
ZPBP2	-0.008811444	5.978313331	0.573882382
LOC647499	0.015072912	6.024893884	0.573899016
GPR62	0.01520123	5.92383821	0.574155112
TGM4	0.015618898	5.979650546	0.574246661
SLC16A2	0.019212513	6.909193369	0.574273448
LOC653883	0.013998938	5.999169601	0.574333218
DPCR1	-0.01878604	6.056911878	0.574362571
DEFB106B	-0.015284337	6.194183741	0.574523834
LOC651868	0.012227561	6.102580802	0.574527573
C20ORF112	-0.017233391	5.970482888	0.574595209
LOC641846	-0.012258641	5.945471479	0.574676471
LOC654091	-0.016522298	5.825795122	0.574682392
PCDHGA7	0.010413397	5.95766377	0.574695316
LOC644732	0.016015427	5.963805293	0.574782872
FAM102B	-0.028313235	7.034437849	0.574814515
FAM82B	-0.015737783	6.311699874	0.574840687
LOC643637	0.012926628	6.051514552	0.574933672
PDHA2	0.012244561	6.079895695	0.574948853
CNTN1	0.011922222	5.985216918	0.574954005
SLC25A3	0.024425178	12.61446785	0.574975345
KIAA1276	0.017945428	6.537108308	0.575023444
TMEM179	0.011957852	6.069126096	0.575050541
C6ORF168	-0.015010636	6.113798104	0.575098835
NDUFS4	0.036075687	11.84085246	0.575183452
LOC653567	0.018088691	6.609319903	0.575253365
BANP	-0.023211359	8.669435737	0.575477513
LOC341346	-0.011927641	6.091134923	0.575485804
LOC642047	-0.016518944	6.237011362	0.575489162
WASL	0.026769915	9.694446658	0.57549965
LOC220115	0.009164229	6.093376586	0.575576271
LOC650631	0.013290709	6.039498803	0.575618042
SFTPC	-0.019844004	6.097145325	0.575660862
LOC643986	-0.014313267	6.287117529	0.575689517
LOC651552	0.016016348	6.043163718	0.575711111
CRH	-0.018647405	6.509062373	0.575845096
C2ORF79	0.021539333	7.76381373	0.576106111
LOC649103	0.013491415	5.948314135	0.576130334
LOC651022	0.014378039	5.940422818	0.576148935
LOC651493	-0.013128062	6.084128857	0.576183155
PROK1	-0.017726174	6.046194137	0.576191646
ATP5EP2	0.025062928	13.69574759	0.576232498
LOC643322	0.012657622	6.097793457	0.576251265
CLCA3	0.016474737	6.024011695	0.576274368
DUSP4	-0.010133115	5.999362135	0.576449937
PAG1	0.024150676	7.352551313	0.576454203
SMA5	0.014444079	6.235229724	0.576492748
EPYC	-0.014753678	6.015944416	0.576515428
OR4A5	0.01448585	5.916247554	0.576644775

MRGPRX3	-0.01373945	6.259414232	0.576787925
PGP	0.020245664	6.982812322	0.57692747
CCT6P1	-0.026139483	7.859603725	0.576963425
NOG	0.013025925	6.03912265	0.576989372
FAM110C	0.012924864	6.065170486	0.577125778
LOC648287	-0.012330529	6.202883716	0.577176752
LOC644197	0.012779288	6.237964578	0.577217101
PUSL1	0.017637036	6.699001466	0.577227203
LOC649160	-0.014495048	6.137388466	0.577299136
ANXA3	-0.042787828	7.091019267	0.577301926
CGB2	0.01585999	5.943878741	0.577349385
PRSS33	0.01178657	5.972215843	0.577355195
FAM19A1	-0.015446893	5.963110291	0.577368423
LOC642173	0.012944325	6.068109412	0.577449082
GUCY1B3	0.023909276	6.895100769	0.577488572
HNRNPA1	0.027855445	6.829000719	0.577578531
LOC649524	-0.01619701	5.998978278	0.57762593
LOC153328	0.012704421	6.345089703	0.577641443
LOC399815	0.011397726	6.088278264	0.57764251
IL1A	-0.013307717	6.030481557	0.577654142
C15ORF53	-0.013882003	5.953683013	0.5776827
PIK3C2A	0.024161877	6.462985965	0.577774771
FLJ10374	-0.038388889	6.465636128	0.577813634
LOC648682	0.014092551	6.105231292	0.57788597
TIMM9	0.034936745	7.698815638	0.577938486
CHRM1	0.017787142	5.910451712	0.577950879
CST5	0.014120595	6.036012717	0.577953089
LOC442208	-0.013864483	6.077408645	0.578153398
MEIS3P1	-0.027088886	8.306075318	0.57825362
PLCH1	0.015702864	6.075823066	0.578427291
SH3BP5	0.009284653	6.078513446	0.578493555
TCL6	0.008488897	6.107283158	0.578806816
SFTA2	0.014631182	6.361436861	0.578899145
EN2	-0.018503251	5.992141935	0.578909742
LOC643227	0.013165151	5.981924278	0.579011819
LOC647706	-0.014025343	5.953313768	0.579057854
LOC641851	-0.015005285	6.106815588	0.579087167
LOC400948	0.047590665	10.00631642	0.579124576
TAOK3	-0.016499225	5.953468431	0.579265561
ESPN	0.014442965	6.12114753	0.579408726
C9ORF4	0.016643965	6.156216525	0.579433376
LOC728973	-0.057586947	10.42828642	0.579448563
LOC400590	-0.01300253	6.16419656	0.579452065
LOC653527	0.016658527	5.848670559	0.579608963
ZNF793	-0.012896239	6.039974084	0.579672584
LOC642344	0.013396179	6.058432571	0.579714003
ZNF354C	0.010462915	5.958433356	0.579839582
LOC652259	-0.01426132	6.151925382	0.579861821
LOC644773	-0.013060626	5.972309994	0.57999513
SALL4	-0.012221023	5.951845945	0.580058791
NWD1	-0.014928266	5.992926842	0.580068215

GPRC5D	-0.01387954	6.008579727	0.58006862
PPM1L	-0.014537498	6.120793662	0.58015705
SSX3	0.00836176	6.100701795	0.580207277
KLHL17	-0.013184661	6.218693816	0.580269935
LOC650849	0.014175104	6.102005757	0.580434794
LOC340096	0.013074825	6.059568165	0.580526834
LASS3	-0.014996734	5.99011598	0.58052696
HIST1H4G	-0.015351566	5.963974047	0.580559255
DDX58	0.031076986	6.694308894	0.580565898
KIAA1467	-0.021016751	6.408964317	0.58059734
EGFR	-0.008333536	6.234080415	0.580612243
DEFB129	-0.015630076	6.107998824	0.580620007
MGC39545	0.014952256	6.074471639	0.580652123
LOC653100	0.014095345	6.189549856	0.580705513
LOC728739	-0.023424978	7.698001294	0.580741263
PFTK2	0.010566902	6.07015803	0.580793818
CUX1	0.011987681	6.189965486	0.580814612
LOC648876	0.024376208	5.988129086	0.580910919
SYCP1	-0.010559801	5.990483237	0.580933269
LOC643496	0.014351039	5.921505234	0.580968113
LOC387870	0.012591669	6.127755334	0.580981097
SHF	0.010145359	6.174103784	0.581020574
LOC221136	-0.012772274	6.151670022	0.581099321
LOC442423	0.014772292	5.910778184	0.581154098
LOC644694	-0.016397719	6.542199287	0.581170619
LOC652417	0.012298404	6.224656178	0.581236292
AIM2	-0.010316682	6.10373394	0.581293214
C6ORF15	0.013783241	6.037747678	0.581422171
LOC441032	0.014026763	6.124918771	0.581504665
FSCN3	-0.013874688	6.062796309	0.581656867
C4ORF33	0.022849797	6.77778127	0.581672179
LOC645038	0.012517049	6.072729035	0.581672951
LOC652602	-0.013193959	6.106629372	0.581738355
BARX1	-0.015704183	6.111460455	0.581863593
OR51G1	0.014974307	5.944985787	0.581957478
TIGD3	0.019267108	6.006665549	0.581968311
LOC389105	-0.013122367	5.950636099	0.581976349
MARLIN1	-0.008904239	6.174220736	0.582129905
FLJ46026	-0.015708513	5.931741342	0.582135411
GJB7	0.015307715	6.023544474	0.582229045
LOC652354	-0.016955469	6.150053792	0.582252491
LOC651789	-0.015947603	5.943835433	0.58229206
LOC649967	-0.013797432	6.071320052	0.582393172
LOC339799	-0.014682522	5.90052604	0.582397276
FAM101A	0.018956372	6.054994254	0.582444887
TNFRSF13B	0.013719715	6.041374992	0.582463815
LOC644402	0.013592266	6.022746037	0.582465434
KLK7	-0.011957235	6.278008099	0.582483283
RCAN3	-0.013060594	6.15853783	0.582518489
FLJ36848	-0.022893412	6.242900574	0.582534753
LOC645882	0.013952688	6.072004645	0.582668056

LOC652424	0.013685142	6.235162673	0.58270364
SUFU	0.02227207	6.072918896	0.582828609
LOC339778	0.013556961	6.165222593	0.582939656
LIMK1	-0.011300467	6.049361135	0.58313606
LOC649527	0.010427634	5.98681693	0.583162196
TTTY9A	0.014531762	5.994837788	0.583279216
LOC650159	-0.013312754	6.037705138	0.58330509
RPS29	-0.02128482	13.38616836	0.583344486
C1QL2	0.017841304	6.244114367	0.583364045
LOC652876	0.013322486	6.138600309	0.583406079
TRIM65	0.01600905	6.390222498	0.583413084
PRAME	-0.00967083	6.389892033	0.583523312
OXT	-0.015005287	6.0689327	0.583639294
ZFP57	0.029049466	6.593310005	0.583641045
UGT2B11	-0.035059599	6.400930316	0.583643723
ABHD8	0.024429816	8.414383613	0.583693811
LOC645837	0.026308312	6.069656076	0.58372758
MAP3K7IP1	0.010037096	6.39386219	0.583817602
RIMBP2	0.014050096	6.070011195	0.583839588
CYB5B	-0.020711501	10.59535502	0.583897234
NEFL	0.015219553	6.142947769	0.58394257
LOC642673	-0.011794363	6.123111408	0.584082353
PSG8	0.0151877	6.098398386	0.584123695
LOC650418	0.011986052	6.138322282	0.584180197
SPATA9	0.011256669	6.107114288	0.584190003
LOC648626	0.018711177	6.135830708	0.584227804
BCORL2	-0.012974599	5.93326853	0.584268473
LOC649264	-0.01311985	5.971481952	0.584279096
NBPF4	-0.013626642	5.882934598	0.584437346
PDZK1IP1	0.015562197	5.940620517	0.584447866
LOC342918	-0.015397539	5.948761336	0.584479803
LOC652156	-0.01403189	6.014148071	0.584540832
LOC642883	-0.01587515	6.006593629	0.584646867
CASR	-0.013569143	5.993457224	0.584650408
C10ORF109	0.015598524	5.998100515	0.584666917
ZDHHC20	-0.01246837	6.119242369	0.584716542
LOC730805	0.012463243	6.085796208	0.58473705
DEFB4	0.016341122	5.962218298	0.584749224
IDUA	0.022385298	6.871773908	0.584786709
HPCA	-0.013944791	6.041607161	0.584806324
KCTD6	0.023986983	7.838998229	0.584975744
LOC260340	-0.013751646	5.981849343	0.584988751
LOC653600	-0.0128875	6.105494033	0.585014068
LOC732007	0.041591496	9.183558143	0.58508103
TTR	-0.017993151	6.341852604	0.58516769
MRPL17	0.023755134	10.12917246	0.585295633
NDE1	0.024201289	8.007051993	0.585386976
LOC340602	0.012860577	5.965847384	0.585396836
HSP90AB1	0.030373126	12.05916039	0.585415314
NPY1R	-0.015379298	6.049691985	0.585494638
CCDC13	-0.009599675	6.124947689	0.585853851

LOC284288	0.013099346	6.022697086	0.585917058
OR5P3	0.009581197	6.012711742	0.586023858
LOC92497	-0.013425129	6.236250561	0.586201528
ZNF37A	-0.01243094	6.162677015	0.586205516
LOC652688	-0.013720408	6.065681826	0.586308058
SALL3	0.029632528	7.98165236	0.586357729
SLC22A6	-0.010232455	6.096734032	0.586439782
NPFFR1	-0.016018335	6.037963424	0.586490713
CCDC26	0.014032046	6.058477946	0.586616473
XIST	0.119914947	7.381035876	0.58672688
LOC649952	0.020649187	5.921145245	0.586764062
FLJ20712	-0.014237334	6.186049362	0.587019595
LOC647371	0.013781053	5.991868182	0.587041332
LOC644327	0.012765874	6.061334697	0.58704551
TXNDC10	-0.020735879	7.54113432	0.587108954
KCNH8	0.014409022	6.378484981	0.587165729
TAC4	0.010067135	6.094162705	0.587242329
CABIN1	0.023898461	7.145450649	0.587281313
PRSS22	0.016799021	6.093035136	0.587415946
GPR110	-0.007202295	6.019374796	0.587421651
LOC651741	-0.013118196	6.207728735	0.587443426
C22ORF15	-0.016169857	6.241573806	0.587515195
C3ORF35	-0.007131582	6.055399898	0.587521019
PPAP2B	-0.039621363	9.855049608	0.587563066
LOC644813	0.014082525	6.031107553	0.587800347
LOC644613	0.018831707	5.928114825	0.588068459
DERA	0.029218767	10.39553092	0.588082522
LOC652317	-0.012292026	6.095801905	0.588152801
C1ORF9	-0.017722715	6.164299139	0.588177472
LOC654206	-0.01409801	5.911506943	0.588235185
LOC728946	0.009364707	5.99026576	0.588270245
C4ORF26	0.015771222	5.983998343	0.588315134
FLJ30719	0.016795267	5.967155401	0.588473902
OR52E2	0.014645781	5.925873615	0.588576658
PAPD5	0.032984891	7.709085104	0.588705699
LOC644330	0.026508197	6.742260007	0.588784897
PIGS	0.032159176	7.822615476	0.588793316
LOC642341	-0.017901446	6.099058811	0.589032514
KIAA1257	0.010872937	6.096215921	0.589046689
LOC642451	-0.014016889	6.188037489	0.589098795
NDUFS6	-0.01479485	5.991904535	0.589243634
LOC653154	0.013416994	6.047307206	0.589244725
RGS22	-0.015648245	6.02649838	0.589378988
FEZF1	-0.01285027	5.978723476	0.589384582
FER1L5	-0.012387292	6.243909467	0.589386795
LOC653244	-0.010487661	6.001551669	0.589415899
LOC150356	-0.028520248	6.46154102	0.589448174
ZNRF3	0.019723844	6.739781822	0.58949206
TREX1	0.009219454	6.189484134	0.589575683
GPR85	0.016217102	5.988189075	0.589680671
TSNARE1	-0.013706224	6.163464441	0.589724182

LOC389730	0.011656026	6.092350144	0.589772031
CXORF55	0.013349025	6.031860252	0.589852138
LOC440558	0.01228569	5.911567056	0.589989171
C20ORF195	-0.01613683	6.057410985	0.590006194
SLC6A11	0.014860766	6.194802778	0.590018934
PNLIPRP3	-0.012326797	5.909204465	0.590083984
DPPA5	0.013235177	5.986233223	0.590187256
SMCR8	-0.01310636	6.014528862	0.590382663
LOC647876	-0.014328167	6.235511075	0.590483004
LOC139046	0.022141344	6.01347089	0.590535167
ARHGAP8	-0.007650111	5.992088052	0.590577207
PLEKHG2	-0.018177166	6.499303188	0.590599109
HTR3D	0.009349763	6.124053389	0.590696515
TRIM31	0.014070286	6.171208444	0.590748588
HSPC105	0.013404605	6.15294104	0.590877754
HERPUD2	-0.019760722	6.940966256	0.59092897
UCN2	-0.016430368	5.941154396	0.591078016
LOC391730	0.013454211	6.021286496	0.591128112
LOC402716	-0.015936956	5.957842074	0.591228839
TAOK2	-0.013871491	6.45603413	0.591254127
CHD2	0.013349362	6.499873451	0.591325145
LOC647646	0.014869339	6.199007763	0.591402137
GIMAP7	0.044261776	7.973759685	0.591446261
LOC642408	-0.013509362	5.958322681	0.591464397
LOC648596	-0.011637304	6.177738261	0.591470151
SERPINB7	-0.009794894	6.01759197	0.591626329
IHH	-0.013090441	6.001283848	0.591732316
LOC647310	-0.013412527	6.086051725	0.591747872
ANKRD7	0.010803367	6.112583593	0.591780851
OR52K2	-0.012786058	6.055276716	0.591819606
LOC652675	0.016556697	6.69629941	0.591866776
C11ORF30	-0.014314177	6.459815428	0.59192513
ARHGEF16	-0.022577874	5.979308857	0.59205802
SAR1P3	-0.013649115	5.97094693	0.592165586
FLJ20674	0.011729971	6.353827478	0.592219225
TSSK4	0.01374207	6.038762366	0.592465162
CTNNA2	-0.016869009	6.131334407	0.592467761
FKSG2	-0.012592858	5.953026219	0.592847819
PLRG1	-0.016875474	8.956446706	0.592890176
PIGR	0.013781576	6.050649144	0.592956314
UBXN7	0.013230981	6.131874746	0.59298712
ARID4B	0.025659834	7.307675471	0.593074256
NFIL3	0.038572823	8.225249394	0.593085938
ZNF81	-0.01564025	5.948348835	0.593213173
OR10S1	-0.014111914	6.182995938	0.593248946
LOC440226	0.01592376	6.384494355	0.593280558
CLLU1	0.01471555	5.96387804	0.593289382
CCT3	-0.025236335	9.188580162	0.593342368
LOC121792	-0.014870361	6.15167681	0.593502978
SMYD1	-0.055019923	11.76443099	0.593534649
RAD51L1	-0.010833538	6.030599966	0.593595033

HEXIM1	0.020288955	6.344717228	0.593801923
LOC651609	0.014173723	6.054875002	0.593859314
LOC648153	-0.012852125	6.174329572	0.593900605
LOC648251	0.011408396	6.162179946	0.593921457
CENPP	0.020560815	6.537576585	0.593935396
LOC439945	-0.014481427	6.216350389	0.594013993
GH1	0.009894961	5.985120489	0.594016392
CNGB3	-0.014818697	5.97618164	0.594116614
FOXQ1	-0.014567644	6.077177476	0.594204928
TERT	-0.010987745	6.036677168	0.594234996
CCNY	-0.024837857	8.598928687	0.594245319
HIST1H4I	0.012880893	6.027366137	0.594249386
GNG13	-0.013252802	5.992858093	0.594367377
PTCH2	0.016655121	6.055595422	0.594377068
ANP32D	-0.011979226	5.92195303	0.594465146
LOC283116	-0.010485399	6.15433402	0.594479388
DKFZP781G0119	0.009828948	5.972832072	0.594526007
BPI	0.012996507	6.113505815	0.594798547
LOC643672	-0.013313073	6.066410127	0.594805087
LOC648085	0.015212713	6.014083865	0.594814256
LOC648277	-0.014569859	6.232791743	0.594918656
FABP4	0.063114419	9.370864006	0.595094125
FLJ26056	0.011425952	6.056909813	0.595117516
SYT3	0.01747226	6.288443362	0.595199799
LOC644300	-0.013701896	6.191472567	0.595337749
TMEM102	-0.011574132	6.095087604	0.595397125
OR4D11	0.012771548	6.080355301	0.59578765
HES2	0.009996127	6.022434226	0.595840631
LOC644766	0.012526118	6.102804349	0.596029958
LOC643171	-0.008571899	6.10709119	0.596126177
NBPF14	0.021218825	6.722390802	0.596133608
LOC390551	-0.017869295	6.080402865	0.59615429
WIPI2	0.014193487	6.748154881	0.596161206
LOC646503	-0.015949339	6.07350874	0.596200182
ROR1	0.02701428	6.873117321	0.596248087
EIF3D	-0.031217992	10.08749556	0.59625519
KCNG3	0.009408578	5.977763333	0.596280835
C2ORF66	0.013442118	6.177770264	0.596563385
LOC647130	-0.012471524	6.020048856	0.59661644
LOC643614	0.013437344	6.131775171	0.596711458
GPR171	-0.014031748	5.931073835	0.596810279
ZNF157	0.017329379	6.038996225	0.596963201
GUCY2F	-0.019165499	6.098083056	0.596993495
C17ORF51	-0.009796834	6.179645857	0.597202253
FLT3	0.015753172	6.173290434	0.597237802
LOC648775	0.01271534	5.985698766	0.597317835
PDLIM2	-0.01065839	6.030690979	0.5973841
PRAMEF6	-0.012371345	5.996884314	0.597426424
HPN	-0.011377582	6.239687722	0.597509341
PLA2G2E	-0.013193182	6.079005751	0.597877412
C11ORF44	-0.015173723	5.992112275	0.597914575

LOC649616	-0.013528425	6.079764631	0.598016834
LOC652454	-0.012775343	5.994463742	0.598056017
LOC653630	0.015273303	5.98182377	0.598110566
FAM3C	0.028409333	7.794230862	0.598149681
LOC441316	0.012865376	5.97554543	0.59820572
LOC649458	0.011439218	6.054890971	0.598206661
AKR1C1	0.014381386	5.877284928	0.598244594
TAF6	-0.027908497	7.646158196	0.5983954
BTN2A3	-0.012567333	5.992138386	0.598487707
SMYD4	-0.025655451	7.763812309	0.598492262
DNAJC10	-0.020451858	8.280100843	0.598557502
LOC647747	-0.017052969	6.086703741	0.598628305
KRTAP20-1	-0.01315712	5.961284414	0.598753059
T1560	-0.01334989	5.955567065	0.598779998
C19ORF30	0.013844211	6.174353111	0.598799955
LOC647920	-0.01282293	6.170390864	0.598800249
MUS81	0.019165046	7.524378423	0.598835296
CYP27A1	-0.026088048	7.625913501	0.5988877
LOC648608	0.023142034	6.080040351	0.598943276
PTK2	-0.022266617	10.01716941	0.598981292
OR1E2	0.009047998	6.01766295	0.598991616
TAS2R60	0.014530856	5.968604834	0.599059908
LOC400954	-0.009645178	5.972079967	0.599075285
LOC728765	0.012958921	6.080191894	0.599090908
SLC46A1	-0.014182887	6.198428322	0.599094871
SLC25A4	-0.021392616	14.25384173	0.5991166
ATG9B	0.013982709	6.068039261	0.599126007
C1ORF113	-0.015218678	6.48474253	0.599151615
C1QTNF3	0.008714922	6.023024915	0.599156221
LOC389852	-0.014310233	5.990821841	0.599325266
LOC643660	0.01104232	6.068373946	0.599357476
LOC388906	-0.012978517	6.108274172	0.59953995
SAMD9L	0.036412587	7.114836428	0.599579206
FBXO33	-0.026095916	8.189398576	0.599631966
MGC24125	0.015091176	6.071048508	0.59973123
CFTR	0.015356567	6.119996898	0.599806203
LRP2	0.012725629	6.003935359	0.60007085
LOC653382	-0.008535501	6.327393367	0.600208735
MTNR1A	0.013759234	6.010404746	0.600216354
PXN	-0.017116732	6.837595992	0.60021699
C1ORF200	0.010869772	6.297667363	0.600237679
OR7E24	0.00949435	6.007368413	0.600245256
LOC255411	-0.011820552	6.060567984	0.600262874
LOC653194	0.016605493	6.127652164	0.600455808
HIST1H2AD	0.014096763	6.011614622	0.600481708
LOC648346	-0.01377821	5.924678628	0.600516706
APOC4	-0.013900582	6.065450919	0.60052737
LOC400807	-0.010015504	6.120345299	0.600581413
USHBP1	0.020610675	6.518857948	0.600683505
RAB39B	-0.014322825	5.87360778	0.600702734
LOC652762	-0.012980264	6.126285534	0.600715054

LOC645402	-0.009612816	6.101789846	0.6007386
LOC644209	-0.012031344	5.919267461	0.60074836
SIGLEC15	0.012334819	6.038405423	0.60097564
LOC649469	-0.014791492	6.236593828	0.601017901
UNQ830	0.013412842	6.396045453	0.601023804
LOC645835	0.015169333	6.124065682	0.601078323
TP53TG5	0.012122738	6.039808244	0.601081654
TEPP	-0.007409466	6.100387345	0.601109471
LOC642718	0.011741227	6.141150248	0.601119868
PMCHL2	0.008997307	6.092298251	0.601149081
LOC653631	0.028988545	6.880339058	0.601154095
C14ORF118	-0.010658849	6.258505677	0.601191399
PCDHAC1	0.009083707	5.960615658	0.601248441
LOC643377	0.0127673	5.981479878	0.6013164
ATXN3L	-0.012905523	5.931331002	0.601349611
FRG2B	-0.009516242	6.004519599	0.601360291
OR7G3	0.016588092	6.066344358	0.601531803
LOC440350	-0.020281221	6.459180595	0.601608636
APCS	-0.015313729	6.524895192	0.601712194
LOC643195	-0.013550643	5.931408317	0.601873358
OR51F1	0.009488981	6.1145989	0.601944187
SNORD21	-0.013756805	6.191101052	0.602004702
STK24	-0.018783239	9.624263111	0.602046287
DKFZP434M131	-0.010213215	6.196585443	0.602060945
SPINK5L2	0.012403746	6.058745775	0.602180411
LOC644035	-0.009257563	5.960087105	0.60223998
LOC653536	-0.013740132	6.004363469	0.602315109
ADH4	0.009941834	6.005693829	0.602421535
PCDP1	0.0127401	6.254554701	0.60251934
C12ORF37	0.015712072	6.258797908	0.602534228
PLA2G1B	0.012606199	5.946703123	0.602645713
LOC646310	-0.012716907	6.160955864	0.602674116
TEAD1	-0.009807738	6.075738978	0.60284888
NDUFB3	0.025929525	12.42770833	0.602926086
C10ORF25	-0.020858741	5.847145155	0.603013133
FLJ45455	-0.014042466	6.037603008	0.603030404
FLJ44313	-0.01684351	6.061853481	0.603137876
KIAA1804	0.010579612	6.163945103	0.603150293
TM6SF2	0.007879974	6.101584223	0.603153685
FLJ38717	0.024155342	7.121859966	0.603188665
FLJ25996	-0.015231276	6.059840316	0.603276906
ASB12	-0.026737368	7.065186503	0.603521163
LOC400299	-0.012758169	6.012209162	0.603550957
LOC650787	0.012456157	6.001906086	0.603558711
LOC401305	0.014734585	6.01925851	0.603630329
SPTLC3	0.019740342	6.408334527	0.603643601
TGM6	0.016779997	6.10847911	0.603696892
LOC441554	-0.012566283	6.252563482	0.603890132
ARL8B	0.024703204	10.12063859	0.603897794
LOC650496	0.012587085	6.011504709	0.603946888
TIMD4	-0.013866877	5.994599829	0.604038925

LOC650339	-0.010550737	6.289739844	0.604057553
DPPA4	0.012513183	6.041807934	0.604167491
MAK	-0.009145086	6.038223001	0.604217504
ZNF575	-0.019689868	6.031732625	0.6042459
SFRS16	-0.019149812	6.58065633	0.604351805
DNMT3L	-0.010395995	6.059311984	0.604388756
MYOD1	0.015079853	6.047850895	0.604480001
NGEF	0.014748646	6.0212066	0.604547916
LOC645228	0.014663697	6.013912437	0.604576786
MRPL38	-0.032350796	8.872216856	0.604653547
OR2B6	-0.012796925	6.031906978	0.604713403
LOC729399	0.010316967	6.058754183	0.604750914
LOC651834	0.012249912	5.91224867	0.605039505
LOC442480	0.012710714	5.996472537	0.605048403
LOC642749	0.02382718	5.938089342	0.605066796
PRAMEF19	0.013332766	6.130511564	0.605108391
MS4A1	0.008982867	6.028273547	0.605147903
PPP3R2	0.013679561	6.010046803	0.605281395
FAM163B	-0.012333871	6.105520763	0.605325134
NAALAD2	0.026039594	6.330663931	0.605381046
LOC651845	0.014955105	6.115750961	0.605405765
LOC401630	-0.013727602	6.007970411	0.605562747
OPRD1	0.018359516	6.142331294	0.605568281
LOC642362	-0.008842564	6.074075383	0.605570212
SOSTDC1	-0.016740954	6.341637427	0.605593154
LOC653461	-0.013549254	5.898900764	0.605620311
LOC644507	-0.012674243	5.972575461	0.60563164
DCX	0.009072432	6.203256701	0.605650858
LOC645393	-0.012274166	6.060053142	0.605664946
CA5A	-0.014315391	5.923348525	0.605699105
NDUFAF1	-0.050110156	8.483956493	0.605828078
C9ORF91	0.01702946	6.483798946	0.605899165
LOC644727	-0.012562798	6.000177548	0.605899545
FAM127A	-0.02071649	11.79551259	0.605904141
LOC389906	-0.010933518	6.008303969	0.605936532
BAAT	-0.013253693	5.935521435	0.605965112
LOC646823	0.012934366	6.018174844	0.606039686
FLJ36144	-0.010321484	6.075649152	0.606070686
GLRA3	-0.010290881	6.025123797	0.606139266
OR5B17	0.012291991	5.949693582	0.60617456
OR2T12	-0.010820051	6.103188133	0.606186685
LOC254312	0.012229912	6.080914825	0.606270051
IGFBP1	-0.010949462	6.135360199	0.606278772
LOC442064	0.013447118	6.200682787	0.606366383
PTK2B	0.010482979	6.315639042	0.606386764
FOXE1	0.009568707	6.147266657	0.606429629
BTN2A2	0.00973431	6.183818364	0.606500994
LOC648123	-0.012713616	6.157214598	0.606588652
ANUBL1	0.015239614	6.171555893	0.606603272
LOC643520	0.012288956	5.960280589	0.60666254
BNC2	-0.017889279	6.576346541	0.606665955

RBMY1A1	0.009287592	5.980040737	0.606724501
CCL25	0.009358608	6.054330482	0.606813329
PDE6D	0.019541517	7.868791238	0.606964193
LOC645176	0.011589105	6.197023811	0.607029753
GLOD5	-0.017942586	6.106869411	0.607238856
LOC648398	0.014049816	6.058742517	0.607268401
GAS2L3	0.016290055	6.489226283	0.607339007
HTR2C	-0.008798411	5.952207482	0.607377687
OPALIN	0.010822815	5.96630243	0.607388988
MTBP	-0.013303494	6.058954799	0.607408047
LOC653254	0.012581876	5.926484902	0.60740945
SLC2A8	-0.035334197	8.730057938	0.60741135
LOC643897	0.012148216	6.055157748	0.607421864
KRTAP9-8	-0.00781599	6.065716325	0.607723021
NDNL2	-0.024956932	6.377921416	0.6077767
LOC645689	-0.011660765	5.932827735	0.608048462
NRARP	0.010572225	6.068238259	0.608146295
LOC649139	0.011187752	6.090065682	0.608321633
LOC652673	-0.016937922	6.322040332	0.608527306
SPRR3	0.014739531	6.125056338	0.608577322
LOC646575	-0.01188217	6.019012317	0.608640783
MASTL	0.011135878	6.237819837	0.608705823
RNF213	0.014945912	6.205757302	0.608721362
C11ORF85	-0.009339492	5.98854566	0.608902822
OLR1	0.027358853	6.572457274	0.609001619
C7ORF51	0.01608852	6.091225781	0.609015909
TAF1A	0.008820411	6.245523949	0.609064153
LOC651386	-0.012127667	6.045607144	0.609180527
LOC646882	-0.013998346	5.948799671	0.609234949
LOC649337	-0.012739025	6.102124007	0.609293678
AGAP2	0.012542736	6.031848026	0.609316799
FLJ41327	-0.014016611	6.090380417	0.609338153
PRKCDBP	-0.032872605	10.26458699	0.609371184
LYZL1	0.01022514	6.166523009	0.60941433
OR8U1	-0.012689732	6.100354663	0.60946444
LOC339209	-0.011941607	6.058461693	0.609509737
KCND3	-0.00960626	6.235415121	0.609573517
LOC642356	-0.011672412	5.940105257	0.609682718
DPP6	-0.008283328	6.012598713	0.609699778
C12ORF69	0.013575867	5.950121194	0.609727712
TMEM126A	0.032649234	10.62984922	0.609847891
LOC649942	0.012692526	6.095374645	0.609869337
LOC649738	-0.012832664	6.038930509	0.609906939
KCNV1	0.012359603	5.945074627	0.610014191
BMPER	-0.016189288	5.907624923	0.610058767
LOC642966	0.017954906	5.992000125	0.610105233
KIAA0853	0.014703123	5.888357441	0.610111819
ITGB7	0.014574069	6.29404836	0.610142768
FGF6	0.013954261	5.980199305	0.61017818
LOC440864	-0.014196597	5.945771588	0.610232815
LOC342933	0.012167152	6.042955745	0.610451431

C12ORF12	-0.013817556	6.033269923	0.610453582
LOC642176	0.013633388	5.902845675	0.610537293
TMC4	-0.017602483	6.19385442	0.610552099
LOC650796	0.017857032	6.088037974	0.610730339
SLC17A6	-0.012315367	5.937840325	0.610786962
HIST1H4J	0.014263533	6.17355585	0.610834987
LOC647500	-0.009293524	6.019268288	0.610854881
RND2	0.014638707	6.503595101	0.610920113
CD9	0.031336952	7.809163822	0.611018913
FLJ21511	-0.014476529	6.05359287	0.611023911
C1ORF151	-0.010553193	6.186184465	0.611096026
PRM3	-0.008544031	6.141373826	0.611134173
ENTPD5	-0.013430121	6.24920135	0.611228837
ETV3L	-0.011931532	6.001508223	0.611250497
PART1	0.013938502	6.026311925	0.611318715
MAP3K15	0.016552832	6.295425225	0.611357764
GAGE12G	0.030201014	6.101775405	0.611387626
FAM21B	-0.007995537	6.251302326	0.611400387
LOC642867	0.012113481	5.987630957	0.611410603
ZNF611	-0.010285499	6.121461075	0.61169267
GNAQ	-0.023052353	6.583408294	0.611748123
TKTL2	0.013207247	5.890359677	0.611819627
ATP6V0E2	0.051369812	10.98769528	0.611907805
SSX7	-0.01242412	5.926160255	0.611932965
C6ORF188	-0.016120841	6.31574603	0.611942806
OR5M3	-0.018159589	6.119898489	0.612039922
LOC648718	0.012593127	6.094460008	0.612046572
LOC653809	-0.011034636	5.931247329	0.612134089
SETD7	-0.011643034	6.119068956	0.612286958
ADAMTS4	-0.017753314	6.272081317	0.612458136
LOC647890	-0.012360669	6.486828625	0.612623698
SCLY	0.013536533	6.606081585	0.612655053
LOC343705	-0.013220751	6.260865047	0.612686828
PTCH1	0.014433199	6.414165213	0.61290084
C1ORF49	-0.014999054	6.172458872	0.612955761
HAO1	0.010244606	6.120176023	0.61295947
TGIF2	-0.021549056	6.752861468	0.613017817
PAQR5	0.009195953	6.06273247	0.61302418
FANCB	0.010006557	6.345851739	0.613107255
LOC644806	0.01227165	6.027601043	0.613150696
CTNNBIP1	-0.031117999	7.649531347	0.613151364
LHX6	-0.024741794	7.273723617	0.613164268
ACSL4	-0.028003563	7.962891057	0.613200322
PPP2R2A	0.026734289	9.595424163	0.613310458
KRTAP21-2	0.012902515	6.043388293	0.613353012
NPFF	0.015036019	6.203733546	0.613382546
FAM63B	0.021250654	6.697737279	0.613543447
LOC648517	-0.012816348	6.140165591	0.613710428
LOC441070	0.01759905	5.937392053	0.613766541
CXCL3	0.021338167	6.057024571	0.613807961
LOC644962	-0.008847676	6.030038643	0.613834773

LOC644829	0.012770289	6.052967408	0.613887859
LOC643729	-0.012418565	5.916661245	0.613986512
LOC649340	0.013547648	6.12800054	0.61409148
LOC644373	0.012954461	6.072660238	0.614148634
RGSL1	0.010335973	5.936862014	0.61417563
LOC643595	-0.013799675	5.978967293	0.614355405
LOC649858	0.012205106	6.0509866	0.614364442
RPP14	0.017103887	6.002595893	0.614404851
LOC643577	0.013233028	6.214635733	0.614483525
RAG2	0.012675679	5.942621551	0.614521501
LOC642866	-0.012381319	5.995273807	0.614546625
SOST	-0.010107435	5.986301553	0.61456416
C1ORF215	0.011089374	6.09746599	0.614574714
CHRND	0.012805689	6.00902529	0.614577185
DND1	0.012165768	6.02607	0.614688638
GPR111	-0.014471649	5.93331731	0.614774826
OR7C1	-0.013486526	5.932898397	0.614864181
AGTR2	0.018616677	6.35171472	0.614989378
FLJ42220	0.014479439	5.947107821	0.614992508
LOC146909	0.011078603	6.240939242	0.615017826
SOX3	-0.010992341	6.004902546	0.615147593
ZRANB3	0.009829235	6.155818851	0.61524897
LOC642817	-0.045396481	10.36176146	0.615377131
ZDHHC6	-0.01819831	8.58442736	0.615378838
KCNIP4	-0.005934296	6.012359425	0.61543618
LOC728242	-0.014682177	6.31296163	0.615480855
LYPLA1	-0.027758635	6.99768596	0.615525811
RIMS1	0.013338356	6.079090636	0.615535908
LOC440792	0.014527127	5.933051688	0.615578864
PLEKHG4B	0.011466074	5.871704862	0.615624619
OLIG3	-0.0155979	6.191561504	0.615803518
FAM84A	0.014527209	6.086568798	0.615813801
ATOX1	-0.026692166	9.377350713	0.61583929
CRYBA4	0.011179916	6.006878012	0.615842877
LOC649313	0.013590746	6.111762217	0.615853751
LOC729034	-0.015161207	5.958233878	0.615891588
OR1K1	-0.018500188	5.922076934	0.615929906
DBF4B	0.009631568	6.022393413	0.615970395
LOC338829	-0.018677898	6.020864908	0.616042123
LOC644586	-0.01151164	6.060359639	0.616074306
PCDHB7	0.01178606	5.938499369	0.616133787
LOC647337	-0.012898163	5.88438356	0.616152475
LOC652128	0.011494935	6.075285021	0.616194273
LOC731501	-0.013506502	6.170478021	0.616229548
SHC2	0.010268873	6.420164568	0.61635128
MFN1	0.014085166	6.705506391	0.616628962
LOC649909	-0.014609897	5.94200826	0.616675273
SPACA3	0.013737872	6.237584127	0.616808625
PPFIA3	0.018696515	6.151092379	0.61688541
DUSP13	0.022082893	7.393080839	0.617071173
HDGFL1	-0.008081881	6.099234608	0.617128123

TMEM200A	-0.014196697	6.095198861	0.617153317
RHBG	-0.014240029	5.996047016	0.617245335
C8ORF54	0.012769875	5.994698512	0.617327675
LOC401002	0.01887212	6.096924087	0.617384996
VTCN1	0.014722686	5.991162349	0.617498541
SLFN13	-0.013699169	6.278838587	0.617510714
LOC645198	-0.010845873	6.084803109	0.617522048
LOC641942	0.012864209	6.045990843	0.617628998
KRTDAP	-0.014261056	6.217017929	0.617634301
FAM124A	0.014871584	6.204074482	0.617695177
LOC642312	-0.012567082	6.077245405	0.617717779
LOC727922	0.013887004	6.035447022	0.617847363
SERINC4	-0.017806304	6.033128298	0.617861836
MKNK1	0.023830475	7.567768546	0.617933019
CHRNA7	0.013014416	6.156658059	0.617962318
LOC652530	0.011574735	6.133313138	0.617997722
REPIN1	0.023843909	9.091453975	0.618029993
OR1S1	0.008838094	6.006672268	0.61803879
NUP210L	0.010280443	5.999407332	0.618146518
RTBDN	0.007162441	6.028972794	0.618173979
C17ORF77	-0.012932252	5.88671752	0.61821043
LOC644625	0.014026368	6.274665505	0.618486203
PSPHL	0.012789212	6.114906137	0.618535595
CRY1	-0.033632795	8.10904556	0.618741789
LOC644038	0.008762277	6.063214202	0.618913508
LOC644011	0.011990459	6.113773096	0.618994658
LOC442519	0.015930035	6.175047644	0.61904644
LOC649101	0.011037679	6.076688691	0.61916119
LOC648424	-0.012225021	5.961294887	0.619173835
NEIL3	-0.010567136	5.97565112	0.619255402
LOC651704	0.012148088	6.022364381	0.619300202
G3BP2	-0.021240379	8.348965668	0.619358675
LOC400723	-0.012170496	5.958426003	0.61939786
LOC400236	-0.012249273	6.128956734	0.619463463
C6ORF97	0.012252109	6.174668193	0.619493025
S100A14	0.017213092	6.159173632	0.61953377
ZNF208	0.0095619	6.006502624	0.619749982
UTF1	-0.013707852	5.953869026	0.619814311
CSAG3B	-0.007963086	6.045740361	0.619947091
PPEF2	0.009723161	5.962078584	0.620084791
ME1	0.025403979	7.562321184	0.620208723
LOC347549	-0.019811819	5.924037778	0.620248825
CHRNA1	-0.012063163	6.149436362	0.620283417
LOC641948	0.012964505	6.1476303	0.620369493
LOC648628	0.01206351	5.945946451	0.620421253
CARD18	-0.013066074	6.03490228	0.620481407
LOC644623	-0.005992454	6.025654926	0.620542897
LOC441171	0.011969465	6.039412613	0.620657377
LOC653521	0.015609774	6.185184301	0.620752814
LOC643866	-0.011937698	5.981302143	0.620825412
URM1	-0.024553414	10.20244675	0.620919967

SV2B	0.039465424	6.834025769	0.620930489
KHDRBS2	0.01197599	6.014608617	0.620940056
FLJ14712	-0.011106125	6.090608787	0.621024957
LOC644031	0.011259189	6.079766519	0.621047225
CCKBR	-0.014115045	5.983915573	0.621069178
FAM19A3	0.010435488	5.923216527	0.621078852
LOC651612	-0.012997522	5.910189644	0.621110937
LOC130576	0.012957892	5.935417491	0.621222631
PIK3CG	0.013709172	6.271000204	0.621265602
LOC653736	0.011866179	6.048783522	0.621273001
PWP2	0.019379934	6.339313528	0.621383644
NUP210	0.016464509	6.310701495	0.621487826
LOC284232	-0.013320436	6.165113582	0.621695075
LOC647625	-0.01395304	6.058679264	0.621792995
HEXDC	-0.024248669	6.698663336	0.621938423
ZNF714	0.015950417	6.078232111	0.621963754
C17ORF38	0.013847293	6.131420379	0.622028598
LOC653313	0.011018393	6.051837929	0.622065356
LOC652615	-0.017851803	6.710123017	0.622065368
ATG2A	-0.01897169	7.476848015	0.622065978
KLHL1	-0.013406195	6.149122312	0.622069452
LOC648342	0.014657728	6.063841342	0.622091812
DMBX1	0.012498447	6.082396537	0.622420115
LHB	-0.013122713	6.026696633	0.622453067
ZNF311	0.015849014	6.023417618	0.622483796
FLJ25328	0.013937242	5.945973567	0.622560279
PEAR1	-0.016025122	6.946949724	0.622568326
LOC344741	0.012038161	6.034885028	0.62260477
BCL2L14	-0.007417116	6.074755577	0.622663957
LOC648001	0.011367536	6.187997175	0.622704041
LOC644753	-0.010166503	5.949114289	0.622811782
WBP5	0.026327103	9.143016588	0.622819638
LOC648744	-0.013285213	6.354824329	0.62294106
MAP7D2	-0.01292665	6.000736302	0.622964445
MYBPC2	0.014937553	5.95887264	0.622971513
LOC644208	0.010971494	5.843000407	0.623184058
HSD17B6	-0.013118346	5.976783038	0.623253753
LOC441155	-0.010251353	6.380269607	0.623598662
LOC283432	-0.012036173	5.87928517	0.623853833
LOC440292	-0.011732123	6.147572581	0.623903341
ITIH5L	0.01265581	5.998577379	0.624027531
KIAA1024	-0.013288236	6.106491286	0.624083463
POFUT2	-0.01535958	7.864982221	0.624132553
SH2D4B	0.009281558	5.964519835	0.624137708
LOC731338	0.011470985	6.039170592	0.624284253
C14ORF21	0.012159815	6.560767981	0.624340105
LOC643102	-0.009244477	6.079740114	0.624352685
TMEM220	0.011141116	6.139068351	0.624370261
LOC346157	-0.012636746	6.06929593	0.624391446
TMEM155	-0.016888044	6.276726886	0.624895272
OTUD3	-0.017809035	6.139283311	0.624939465

LOC647859	0.009899541	6.139288014	0.624950364
PNLIPRP1	-0.012614527	6.067014409	0.6250171
PRSS1	0.011837528	6.096774001	0.625127974
LOC650950	-0.013621533	6.28465847	0.625237319
RNF157	-0.01544849	6.264809144	0.625264607
LOC652470	0.011881731	6.127097888	0.625380252
INCA1	0.015739888	6.561067013	0.625420165
LOC646670	0.011734441	6.122402006	0.62543395
C7ORF33	-0.012083683	5.971243718	0.625447275
LOC650990	0.009165998	6.109960962	0.625478804
TREML1	0.014804582	6.366483054	0.62553427
LOC652662	0.011445325	6.106136271	0.625585269
LOC401074	0.016461806	6.211082238	0.625609771
PRDM12	0.018009951	5.894524237	0.625701234
PCDH8	-0.008725766	5.933520463	0.625735694
PAEP	0.009065883	6.002360502	0.625829941
LOC649971	0.012702979	6.009093266	0.625884162
JMJD3	0.016505354	6.236010746	0.625955304
FGF14	-0.00962163	6.026222871	0.62600349
PLXND1	-0.025969997	6.051483202	0.626015438
LOC644474	0.015756967	5.964087654	0.626017031
TCEB1P3	0.011953794	6.012376214	0.626056317
LOC648874	-0.013462544	6.185639498	0.62616775
TCF2	-0.007428327	6.014321803	0.626222032
TTTY8	0.01331498	6.123732719	0.626264166
FAM82A1	-0.011503354	6.0066735	0.626305596
LOC652054	-0.011735077	5.938099749	0.6263118
OR4D9	0.010845176	5.98557174	0.626386275
GPR120	-0.012557259	5.973383439	0.626436861
KLHL10	0.011997554	5.975059796	0.626463986
MAPK8IP2	0.007328007	6.028849826	0.626507597
VN1R4	0.014020734	6.140410563	0.626518337
ABCC2	-0.013683203	6.151753236	0.626552101
BST1	0.014337456	6.293660446	0.626596194
LOC652849	0.013237743	6.123290789	0.62659626
DEFB127	0.01269923	5.986913122	0.626628496
PDE6H	-0.016547845	6.110225618	0.626779806
LOC644311	0.010229943	6.086958127	0.62684452
SEPT1	0.009147031	6.218413697	0.62700202
NFKBIL1	-0.021706095	6.0478553	0.627030161
ANTXRL	-0.013610893	5.942256211	0.627127289
BAI1	0.007147522	6.060397655	0.62724486
C6ORF195	-0.013269761	6.139093317	0.627255086
NUDCD3	-0.025415067	8.567224891	0.62726344
TTTY17A	-0.007688482	6.060973361	0.627287905
LOC399715	0.007394448	6.076541092	0.627364617
LOC653210	0.006989472	6.097709649	0.627430857
MRGPRE	0.013610909	6.194064569	0.62750966
LOC648138	-0.013109797	6.064909418	0.627643708
ARSF	0.012015131	5.966787136	0.627736513
STK17A	0.013170031	6.252394205	0.627840982

DIRC1	0.012905815	6.031948853	0.627981815
LOC651923	-0.011934232	6.109947963	0.628012502
ACOT9	-0.02419631	8.653597751	0.628033731
LOC652216	0.012119587	5.95745627	0.628083388
ORMDL1	0.02858033	7.976635083	0.628186176
RFK	-0.014493958	6.482410831	0.628396095
SLCO6A1	0.01366362	5.967655039	0.628407216
FCAMR	-0.010299321	6.03895822	0.628439822
SCARF1	-0.009773502	6.138379988	0.628462907
GGT6	-0.011352712	6.109213158	0.628487214
LOC650387	-0.014042743	6.104256585	0.628641932
LOC649504	-0.01187477	6.05752806	0.628933187
HSF2BP	-0.012971958	6.199791865	0.628958101
FLJ31401	0.009128753	5.947248217	0.629008737
C21ORF100	0.014727886	6.063463366	0.629058915
LOC392506	0.010831401	6.079614797	0.629159571
LOC642506	-0.010990739	5.944836456	0.629190062
FCHSD1	0.009617	6.057633718	0.629211104
SYNC1	-0.035593351	7.669705547	0.629304226
LOC646992	0.011115268	6.061251703	0.629310476
KRTAP5-2	0.014166681	5.963544719	0.629522158
RTKN2	0.013709373	5.964547961	0.629595815
FAM3D	0.012519186	6.119880943	0.629632121
FAM47B	-0.008292802	6.086373968	0.629643054
LOC645217	0.013949287	6.345299047	0.629770094
LOC646960	0.010770442	6.016784389	0.629842339
SLC26A5	0.007138155	6.013591659	0.629864262
PCTK2	0.018413872	6.225218809	0.630181793
IQUB	0.01307362	6.102725292	0.630268694
LOC651301	0.012101217	6.099681471	0.630358229
CEACAM4	0.012541097	6.213917222	0.630358494
COL17A1	-0.015604262	6.321507015	0.63036203
CST8	0.011908658	6.050947554	0.63048962
ARL5C	0.012049605	6.112112071	0.630494886
SLC35D1	0.012852827	6.14402086	0.630875594
SLC4A1	-0.012691186	5.933037675	0.630913657
DLX1	0.008043206	6.218901576	0.63096862
CCNB3	0.007877275	6.002390874	0.63106609
LOC647544	0.01108262	6.080908337	0.631066266
ZCCHC12	0.016535322	5.965374463	0.631115316
SLFN5	0.020408374	6.385621162	0.631367531
LOC650680	-0.011662416	6.055549226	0.631372136
AQP8	-0.011329196	5.979000741	0.631380173
TBX19	0.010625085	6.429550495	0.63143378
LOC284998	-0.010850585	6.161045079	0.631625254
LOC728276	-0.010020148	6.028649663	0.63164549
MGC27382	0.02006346	6.092275087	0.631651179
LOC731196	0.010549843	6.171617702	0.631674181
PGLYRP2	-0.012407646	6.017778574	0.631681989
LOC652656	0.010989779	6.165055185	0.631759972
LOC729533	-0.015378029	6.134731537	0.631862018

LOC644596	0.013654888	6.280779638	0.631916423
SMS	-0.031479387	9.588787997	0.631940675
OR4K13	-0.011270242	6.116957742	0.632188251
GOLGA8E	0.008022288	5.963648439	0.632209704
SEPT6	0.013377917	6.469272902	0.632842561
LOC642520	-0.012494278	6.142865851	0.632845435
SOHLH1	-0.012299051	5.892401182	0.632859022
HSPC072	0.012886569	5.992631525	0.632872387
PKNOX1	0.020161503	6.520677697	0.632882885
LOC650212	0.011651893	5.933034082	0.632923948
HORMAD2	-0.011171423	6.025406868	0.632980748
FGFBP1	-0.012018891	5.952402081	0.633153264
LOC644885	0.013553305	5.944836779	0.633179777
LOC644593	-0.013513571	6.089702979	0.633327858
FDPS	-0.028568666	7.515035875	0.633339562
RAB40A	0.013747931	5.964636151	0.633409321
LOC651747	-0.013060818	6.057586904	0.633441032
LOC641953	0.011558317	6.072899559	0.633493738
SLC23A3	0.013490765	6.128773169	0.633507025
FAM48B1	0.010614909	6.08856193	0.633541861
TMEM145	0.016535783	6.430565386	0.633706496
GPR17	-0.012099733	6.062312602	0.633764757
LOC647865	0.014369751	5.942004515	0.633912143
LOC652797	0.011048808	5.969846786	0.633960845
LOC649044	-0.007935263	6.29206669	0.633973794
LOC158318	-0.011985441	6.253163995	0.634116818
LOC650247	0.012676539	6.080166266	0.634163258
CCDC122	0.009646287	6.221043319	0.634195843
GABRG1	-0.01259027	5.969631754	0.634345856
LOC649151	-0.014795702	6.031766201	0.634708934
LOC642083	0.011560077	6.083798408	0.634898261
PRL	-0.012847983	5.915233055	0.634931799
LOC650399	-0.012961863	5.889339926	0.634973367
LYSMD3	-0.013492776	6.252342308	0.634983739
LOC441847	0.011947825	6.022820168	0.635016793
RS1	-0.011373299	6.017474716	0.635237953
KIAA1086	-0.014715809	6.23789812	0.635491929
TFF2	0.013480163	6.017791469	0.635560522
LOC649477	-0.01453138	5.977377023	0.635661908
LOC644338	0.01053216	6.056534069	0.635709021
LOC648585	-0.008057908	6.106771767	0.635826603
UPK3A	-0.013671971	6.118774158	0.635860977
LOC392425	0.01308073	5.936172217	0.635916392
HSP90AB6P	0.011985694	6.049168698	0.636039457
CELSR3	0.013180822	6.149630558	0.636093452
OR1A2	-0.011869516	6.023178892	0.636149361
MYO16	0.04704189	7.473540554	0.636186454
CALM1	0.022731471	12.31425099	0.636289888
OR2S2	-0.01087902	5.931770225	0.636586783
TCHHL1	-0.013597022	5.876856818	0.63661665
LOC652690	0.01154558	5.993193904	0.636663116

NDUFA1	0.018835035	14.1125454	0.63668108
DIS3L2	0.013905768	7.189650691	0.636813832
LOC732040	-0.013850482	6.054157338	0.637037502
CLUL1	0.007405799	6.081702952	0.63703822
C11ORF9	0.012119832	6.006229648	0.637138341
LOC652542	-0.01248636	5.95843528	0.637387994
LOC388327	-0.006094094	6.105208467	0.637560554
LOC729601	0.010782116	6.107805614	0.637567425
CCDC108	0.010612173	5.965671636	0.637807325
LOC642564	-0.01368035	5.954933333	0.637956885
INSIG2	0.01652742	8.241680948	0.638091122
LOC647237	-0.012586027	5.983265892	0.638133068
FRG2	0.007644449	5.998510399	0.638165584
SULT4A1	0.010766568	5.958465725	0.638208077
LOC729792	0.011997426	6.144116774	0.638364106
LOC389827	-0.009530573	6.078705494	0.638368983
LOC651754	-0.010929267	6.099430791	0.638404348
ABCB11	-0.01148356	6.172809025	0.638490259
TAF2	-0.017324858	7.29096696	0.638498812
LOC642948	0.012490113	5.975130585	0.638501759
PGLYRP1	-0.01321595	6.208705486	0.638648178
ZNF831	-0.009140455	5.983963568	0.638668371
LOC390564	-0.011680143	5.936683778	0.638827003
LOC652687	-0.011270891	6.103549412	0.638840036
LOC644411	0.012934446	6.023170539	0.638849297
RGS7	0.013345973	5.991075158	0.63891768
MBD1	0.01492684	7.022004605	0.639123171
LOC644482	0.029550764	7.295460804	0.639132339
HTF9C	0.008568216	6.333527008	0.639134984
LOC643082	-0.012103975	5.931688336	0.639241724
PPY	0.01169485	5.99380467	0.639334366
OR2M5	0.012910583	6.026930295	0.639389185
LOC644371	0.012518574	6.047538335	0.639606703
NUFIP1	0.013005328	6.254140403	0.639620311
OR5K1	0.01226805	6.022607705	0.639663313
LOC646223	-0.015279188	6.02654697	0.639688428
LOC642720	0.012909605	5.994881996	0.639803756
KIAA1958	-0.016857073	6.307930059	0.639826224
PUNC	0.011805755	5.970875228	0.639961417
FLJ43826	-0.010796479	5.937722754	0.639987018
SPEN	0.017343802	9.169806258	0.640039622
LOC651371	0.013245746	6.188507013	0.640083466
LOC642141	-0.011173121	6.020349959	0.64021842
TTTY9B	0.011618485	6.039830221	0.64024108
LOC653648	0.018286183	5.948886369	0.640263588
AFARP1	-0.008561786	6.043173479	0.640657239
LOC652825	0.011113322	6.076165924	0.640658623
LOC642621	0.01257206	6.158602151	0.641004127
SAMD7	0.013446998	6.347108724	0.641078594
EIF4E	-0.015709113	6.30371078	0.641145176
GBP4	0.045251307	8.219388735	0.641178373

DDX49	0.031627608	6.24990381	0.641292084
OXER1	0.012909208	6.080070474	0.641387597
NUDT9	0.016558214	8.23430469	0.641409339
TULP4	0.015706445	7.195138004	0.641492856
DEFB112	0.011508985	5.993914462	0.641649603
TBX18	-0.007911581	6.22915613	0.641714371
CXORF22	0.012322611	6.015495868	0.641917
EP400NL	-0.007788845	6.062781716	0.641976553
ZFAT	0.009547601	6.265453123	0.641982221
LOC441237	0.013120058	6.053840267	0.641988462
DLG1	-0.018421276	6.17154584	0.642061716
FOXP3	0.016342743	5.98396142	0.642116878
LOC647083	0.011847545	6.248035744	0.642127803
LOC643402	0.013228049	6.178265441	0.642216704
FLJ32784	-0.008572138	6.108196201	0.642233358
SLCO1B3	0.011592097	5.958205506	0.642236204
ACPL2	-0.036240867	8.009875199	0.642281259
GJB5	0.013987351	5.894234956	0.642290176
LOC650677	0.007240998	6.12429186	0.642297882
HLA-DOA	-0.028363035	6.780299507	0.642364819
NPIP	0.021358989	8.244609052	0.642386264
LOC646457	-0.012539113	6.066796723	0.642389051
BLOC1S3	0.012241575	6.038880123	0.642420366
CDY2B	0.015522876	5.986849144	0.642451947
PERLD1	0.021225553	8.03748933	0.64275763
HS1BP3	-0.019265325	7.512381796	0.64284385
ABCD2	-0.008210387	6.047191883	0.642928085
MGC24975	0.012934035	6.385443491	0.642935171
MRPL18	0.017489268	10.15201062	0.643058745
GRIK5	0.012197999	6.178750242	0.64312628
LOC400950	0.009457409	6.026258876	0.643165817
SAG	0.011257358	5.976914549	0.64316836
LOC644707	-0.01073473	6.038430237	0.643406859
OR10J5	0.011984115	6.036264584	0.643506251
LOC649703	0.011151899	6.132066778	0.643514133
LOC653303	0.007602204	6.213764629	0.643520133
HSD3B1	-0.011116833	6.087447473	0.643523259
LOC338750	0.016883416	6.116627819	0.643552851
LOC643713	0.012464349	6.39023654	0.643556176
LOC285577	0.010030326	6.324680103	0.643662269
LOC641999	0.011598416	5.866415176	0.643678134
PRIM2A	0.009616548	6.261622154	0.643681343
HOXA1	-0.009249701	6.130016006	0.643756983
NTSR1	0.01220056	6.171414512	0.643789236
LOC504188	-0.011654496	6.150370913	0.643828025
SPANXN4	0.012864089	5.866396935	0.643839285
ARL6IP6	-0.012987344	6.747794686	0.64401345
MAGEA11	-0.008872393	6.056352015	0.644053014
SHOX	0.007236196	6.045182411	0.644108664
FLJ40453	-0.01071934	6.037808112	0.644165048
LOC729373	0.01150096	6.056406501	0.644195371

TTC7A	0.012650008	6.232269533	0.64452084
TNFSF11	0.007701231	6.052919656	0.644550534
LOC646084	-0.010841188	6.15764844	0.644568802
BRD9	0.015613382	8.829314607	0.644591804
PRAMEF12	-0.012397411	6.0042949	0.644596983
CKMT1A	-0.010575465	6.095617352	0.64465861
CALML6	-0.014240033	6.0244141	0.644745619
PRIMA1	-0.009412515	6.066475348	0.644916205
LOC648262	0.011008874	5.974097376	0.644917164
LRRC61	-0.012226405	6.091832631	0.644984471
LOC643590	-0.012998368	6.31479835	0.645218291
LOC653706	-0.015677255	5.95492512	0.64525268
LOC649951	0.011770068	6.000749867	0.64527126
IL22RA1	0.010509465	6.067066444	0.645330407
FRRS1	0.01050207	6.457402991	0.645369497
FNBP1L	-0.034886408	7.70770856	0.645501322
LOC643905	0.015477868	6.117715031	0.645538686
RASA4	0.012071847	6.051964082	0.645743269
C19ORF40	0.013307701	6.293588743	0.645855433
OR8J3	-0.00770118	6.100329963	0.645953151
KRT13	-0.01161301	6.031092201	0.645962171
NR0B1	0.011736833	6.194659857	0.645975752
KIR3DL3	0.007559126	6.075320007	0.6460292
LOC652292	0.012054369	5.9202342	0.646029949
HOXA13	-0.01095903	5.963965744	0.646065274
C10ORF27	0.011251821	6.206238401	0.646104147
PCDHB18	-0.013345139	6.264232671	0.646219255
CHAT	-0.006780106	6.128634635	0.646228657
RP1L1	-0.013281186	6.130680882	0.646276488
PPP1R9B	0.013519179	6.105175989	0.646293041
P11	0.013521555	6.491315722	0.646413761
CCT8	0.019785484	11.13125071	0.646517054
REG3G	0.008522652	6.029735309	0.646596541
HIST1H2BO	0.011838404	6.027251311	0.64659696
LOC650646	-0.028967025	12.45374111	0.64669897
LOC651577	0.01069604	6.05197615	0.646713446
CTAG1A	-0.008616133	5.996440978	0.646728937
C1ORF166	0.018362402	7.783645042	0.646759141
LOC651751	-0.015249879	6.126950803	0.646824986
SIPA1L2	-0.042233446	10.93117603	0.646858529
CCDC69	-0.027539849	7.125374721	0.646919529
LOC647471	-0.012110322	6.033532689	0.646971028
LOC653625	0.012713884	6.306952795	0.647020578
LOC284837	0.012878408	5.929892806	0.647027599
POLK	-0.011453075	6.019649937	0.647092369
IL11	-0.012895919	6.015735526	0.647196915
LOC644532	0.013601912	6.04733297	0.64722096
ADAMTS19	-0.01368793	6.324979622	0.647226586
CRLF1	0.031518645	6.89285869	0.647458572
ZNF696	0.015698509	6.916727341	0.647511429
LOC652418	0.013860603	6.208867651	0.6475138

C21ORF121	0.012913978	6.115047786	0.647521005
LOC651140	0.011142033	6.172751299	0.647643624
LOC645946	-0.011380493	5.959599035	0.647941714
VWA3B	-0.010737753	6.260476192	0.647956482
NEK4	0.011421231	6.205418622	0.647966113
HIP1	-0.017806119	6.917898028	0.647998313
OR6M1	0.012152735	5.977194655	0.648001545
LOC652537	-0.012163752	6.363943324	0.648016915
LRTM2	-0.011940045	6.195707494	0.648084633
FAM154A	0.012046635	6.013134711	0.648221398
LOC651524	-0.011139443	5.875928996	0.648314493
TDRKH	0.012054844	6.200703519	0.648419548
LOC649107	-0.010831681	6.020843467	0.64843335
DLX2	-0.010622832	6.100410311	0.648529561
NEGR1	0.010836819	6.160182203	0.648679709
KRTAP12-2	-0.011322165	6.134996035	0.648846673
LOC93349	0.012585979	6.013348522	0.648933976
ANKRD42	-0.013067906	5.985176544	0.648988156
LOC650227	0.021336341	6.039122326	0.649326033
TRIM9	0.011174044	6.401764032	0.649351349
MYBL2	0.017144385	6.021890038	0.649359571
LOC651324	-0.014015583	6.066599421	0.649368031
GTF2IRD1	-0.014897841	6.678882244	0.649682267
RNASE4	-0.02150158	7.314701295	0.649766934
TEDDM1	0.01384901	6.204123111	0.649809514
KIAA0494	-0.018773364	9.327614167	0.649943976
PC-3	0.01002733	6.113461119	0.650207828
BMI1	0.023239544	9.190245518	0.650279424
C6ORF208	-0.01046065	6.171031145	0.650281489
LOC388255	0.01705686	5.978713705	0.65028779
SLC16A13	-0.01289481	6.15868162	0.650395735
LOC148003	-0.012925767	6.046796132	0.650444486
TRAF4	-0.009485057	5.978143671	0.650491537
RARS	-0.017360357	10.06387979	0.650520802
TRIM54	0.029663839	8.802269964	0.650524873
LOC642888	0.011489626	6.011689154	0.650695916
PTGER1	0.012289558	6.182909086	0.650739179
NXF3	-0.012652626	6.136840456	0.650740579
GTSF1L	0.008176788	6.032849756	0.650748286
LOC652582	-0.010569743	6.118411132	0.650843823
NAIP	-0.006935963	6.114783814	0.650947257
LOC645777	0.011617025	5.924897057	0.650966413
LOC653816	-0.010832358	6.072174923	0.650994354
ACOT12	0.013102174	6.139400541	0.651011471
MUC3B	-0.009077642	6.09772913	0.651309185
LOC649414	0.01051217	5.980592614	0.6513918
LOC644738	-0.010594126	6.112689925	0.651407005
LOC133874	-0.010779946	6.045138239	0.651495476
GPD1L	0.037248471	12.20492147	0.651515141
FLJ12529	0.018980816	6.497167376	0.651553664
LOC643324	-0.012770196	6.143545957	0.651584034

SLC18A3	-0.011789776	5.983844532	0.651609108
FAM98B	-0.007696836	6.141668347	0.651618721
LOC442726	0.012682991	5.926908716	0.651639041
LOC646470	-0.012434302	5.94176914	0.65192849
ARL6IP1	0.023296655	8.51029834	0.651960751
LOC653645	-0.010380837	6.047790474	0.652037207
LOC650458	-0.011151369	6.16780564	0.652214652
LOC646312	-0.01295181	6.160681132	0.652384897
LOC644908	0.012991634	6.067278695	0.652510529
MLL3	-0.008875603	6.678506662	0.652534344
OR5AK2	-0.009935949	5.990219936	0.652660284
LOC645620	0.012974903	5.964449765	0.652722273
FOXJ1	-0.020187785	6.056158685	0.652758905
PRB1	0.006708334	6.064849639	0.652784218
OR6X1	0.008115713	6.01604697	0.6528013
LOC644715	-0.02180742	6.374609448	0.652813212
HRNBP3	0.013325938	5.974931045	0.652850428
RBMY2FP	-0.010816438	6.071889022	0.652917729
AKAP5	-0.012383591	5.988668569	0.653084265
LOC648117	0.009488342	6.040098372	0.653246746
SART1	-0.010980628	6.234498539	0.653559095
IL29	-0.008190595	6.042269671	0.653583331
LOC643927	0.01180423	5.847002753	0.653605642
LOC643604	-0.010411549	5.958584314	0.653619369
VN1R2	-0.00968311	6.148147674	0.653625929
LOC646071	-0.010597212	6.17948626	0.653669792
SASS6	-0.009880715	6.381358446	0.653720737
LOC727958	0.010781193	6.064367769	0.653855195
UEVLD	0.014307576	6.643405715	0.653861346
LOC653832	-0.007825222	6.078861483	0.653875513
C8ORF42	0.010194012	6.13618883	0.653898991
PRKAR2B	-0.012250057	6.129607012	0.65395703
LOC644735	0.012968109	6.150162107	0.653985315
LOC643811	-0.010684021	6.177716979	0.653987809
C14ORF72	0.01057184	6.052802181	0.654229648
B3GAT1	-0.007331176	6.161555942	0.654303914
TPCN2	0.025745269	7.29980524	0.65433907
PQLC2	0.006757788	6.099465572	0.654401024
GINS4	-0.011451472	6.433667486	0.654504872
PROCA1	0.015738392	6.075036008	0.654604334
LOC646549	-0.010244817	6.005798735	0.654715173
LOC644345	0.010654132	6.037909731	0.654806519
LOC642473	0.010125333	6.036793076	0.654816732
AK1	-0.033140521	10.26592912	0.654846239
LOC642196	0.011847565	5.916804209	0.654887371
LOC652512	-0.010618311	6.11922967	0.654888528
LOC646515	0.01179125	6.166115429	0.655013012
LOC646931	0.01050168	6.047009264	0.655353516
GPRIN1	-0.013403209	5.954568869	0.655359778
LOC642922	0.012566774	6.075512814	0.65537928
CXCR3	0.013589438	6.128469189	0.655386079

LOC388438	0.011333742	6.077249215	0.655403591
LOC653111	0.013779463	6.327239586	0.655419087
OR1N2	0.011465765	6.055894982	0.655420184
MMP26	-0.012611908	6.067930796	0.655437067
LOC646654	0.010719779	5.968626692	0.655462397
CD8B	-0.006021837	5.996247904	0.655543525
PARD6A	-0.01366378	6.584162458	0.655774983
KIAA1975	-0.010712534	6.073263354	0.65588383
LOC651128	0.01028007	5.982751674	0.655931211
SLC5A3	-0.014222546	6.131471014	0.655942204
LOC339524	0.010614286	6.068591785	0.655973504
PYDC2	0.012029079	6.08650394	0.655984255
TBC1D22A	0.019515617	6.188518364	0.656032011
RHBDF1	-0.024653598	10.16761758	0.656160202
HLA-DRB1	0.119528937	7.40229684	0.656230829
PAGE2B	-0.012920318	6.031531613	0.656260625
TMEM64	-0.011835193	6.463864496	0.656316592
A1BG	0.009123176	6.217095601	0.656491702
LOC645183	-0.011459133	6.234553386	0.656552289
DUOX2	-0.01291148	5.98398373	0.656623384
LOC145845	0.009973418	6.025791025	0.656664224
MGC15885	0.010473697	5.97971133	0.656764302
CCNO	-0.017169544	6.215319998	0.657001434
LOC645408	-0.010583319	5.998960038	0.657092889
LOC642639	0.011728094	6.031389061	0.657141269
C8ORF13	0.011971648	6.028041701	0.657202022
LOC654020	-0.011179577	6.309266765	0.657243039
TNRC18	-0.009584482	6.244521523	0.657278039
GYS2	0.010368571	6.069889267	0.657420769
PLCL1	0.03126209	8.138925702	0.657496686
LOC389834	0.032300451	6.573234077	0.657567737
OR2A42	0.009581334	6.127686717	0.657585497
KCNJ16	0.009634848	6.408553399	0.657592421
LOC650755	0.01710674	6.039240292	0.657692801
RILPL1	-0.024517751	10.82953157	0.65779428
LOC148203	-0.012315189	5.944242588	0.65785592
NPDC1	-0.024630898	7.057576458	0.65786381
GAGE7	-0.021840969	6.200563253	0.657936605
LOC652763	0.011419708	6.078683116	0.657942257
CERCAM	0.026041535	6.926203585	0.657951451
HSPA9	0.052709625	10.37807853	0.658220228
C10ORF107	0.011294792	6.065704459	0.658324452
LOC206227	0.0104968	5.952474961	0.658427773
DUOXA1	-0.012094849	5.988673301	0.65846706
LOC440414	-0.01052409	6.057578265	0.658481728
LOC644830	-0.011463281	6.052133256	0.658789101
LOC648738	-0.01090817	5.989715117	0.65890977
LOC650241	-0.011057464	6.08197868	0.658978377
LOC649086	-0.010756867	6.091463532	0.659113525
FAM64A	-0.012822754	6.022140435	0.659131549
HTR5A	0.010655891	5.93686408	0.659242468

PARP15	-0.012558507	5.971206556	0.659284769
SLC4A11	-0.010954593	6.233737815	0.659346674
LOC646156	-0.011305577	5.991750146	0.659536237
LOC116349	-0.011407747	6.106253414	0.659676636
STOML3	-0.011613804	6.100025059	0.659749917
LOC644982	-0.010995889	6.011724208	0.659873755
TWIST1	0.019139658	8.349939478	0.659903379
OR56A3	-0.012307637	5.929948451	0.659926656
HIST1H2BJ	-0.021690216	8.438475694	0.659928036
ZNF107	0.008324385	6.037287818	0.660038769
FLJ37453	0.014718211	6.413805514	0.660065067
LOC647568	-0.011262766	6.29100113	0.660322979
UFSP1	-0.01565267	6.275831383	0.660425643
LOC650257	0.011846585	5.987258012	0.660476626
LOC127602	0.005420675	6.080537317	0.660685879
RNASE11	-0.010520915	6.092546496	0.660796103
HRNR	-0.012680499	6.069753006	0.660866971
ABCB5	-0.012400074	5.883988189	0.66088679
LOC650846	-0.009880957	6.134493825	0.660934289
TAS2R8	-0.008522029	6.032986479	0.660954778
LOC651565	-0.014273404	5.983935694	0.660973637
HYPB	0.010192048	6.057962155	0.661034892
OR13C9	-0.007742229	6.135666503	0.661131708
DAZAP2	-0.022468249	12.30728367	0.661278857
LOC644891	0.012564766	6.083596377	0.661315031
LOC649659	-0.010381314	6.077551422	0.661341709
SPAM1	-0.008591958	6.165589083	0.661534304
NMUR1	0.011111823	6.090784783	0.661578384
KIR3DP1	0.012250998	6.220785502	0.661656822
MGC19604	-0.010783493	6.105014781	0.661687629
DEPDC6	0.017039691	6.956757159	0.661719385
LOC653178	-0.008202112	6.081606462	0.661958819
SYDE1	0.019431071	7.353397288	0.661975335
LOC643495	0.010567088	6.032497882	0.662053041
EIF1AD	0.01822028	6.262928344	0.662084392
KRTAP26-1	-0.010438467	6.083432638	0.662166561
C1ORF101	0.011252243	6.084154487	0.662206345
GOLGA8G	0.006854212	5.956607263	0.66223933
NOX5	-0.010418679	6.150636002	0.662288269
LOC440992	0.01021775	5.998196327	0.662328365
CSNK2A1P	-0.013928675	7.467834618	0.66234783
C20ORF70	0.012449541	6.097668501	0.662349387
CRYGN	0.015396594	6.030547454	0.662488397
MGC13098	0.010323471	6.095373729	0.662513388
LOC643330	0.010695269	5.941637854	0.662554935
RPL30	-0.025642246	12.50698755	0.662598947
LOC651630	0.011622625	6.219332552	0.662609839
LINGO1	0.013535065	6.339912727	0.662650481
LOC646407	-0.009071628	6.166517391	0.662713233
GUCY2D	0.010640746	6.102760567	0.66275937
MLANA	-0.0099896	5.869018236	0.66289552

FADS1	-0.024464054	7.938182441	0.662924577
PARD3B	0.008491201	6.100673643	0.663130646
ANKRD31	-0.010396824	5.947655659	0.663255595
PLGLA1	-0.007178149	6.137657419	0.663489307
LOC401354	-0.010708614	6.225137605	0.663551455
SPANXA2	-0.010313263	6.083148947	0.663575786
PAXIP1	-0.013576198	7.78091636	0.663678588
USP27X	0.009856937	6.169806261	0.663800033
CECR2	0.010051252	6.08432055	0.663887826
LOC645552	-0.010276606	5.953113065	0.663928948
MEIG1	-0.00797675	5.967449304	0.663939564
PRSS3	0.010395529	6.10370515	0.663940263
LOC387646	0.012962525	6.058188103	0.664046369
EFCAB6	0.007006315	6.121230877	0.664101689
REG3A	0.009244969	5.979890898	0.66415395
BIRC1	0.015151535	6.051109431	0.664178831
EXTL3	0.025624984	8.575317746	0.66421317
C1ORF140	-0.010874959	6.100867288	0.664236804
OR51A2	0.010043225	5.978969221	0.664251668
SLAMF9	-0.017130585	6.123405219	0.664258077
LOC649990	0.014098357	6.207559809	0.664260633
FLJ20920	0.018367302	6.552023213	0.664341861
LOC391347	0.009851318	6.134003801	0.664460094
LOC650759	-0.011961962	6.257059574	0.664497816
ACTR3B	0.007425965	6.183781263	0.664547659
GOLGA2LY1	0.011525083	6.112861357	0.664636652
SPON2	-0.018166949	6.249148855	0.664668056
SLC26A1	-0.00608112	6.081978729	0.664761972
LOC653596	0.006325553	6.199230781	0.664806386
LOC643787	-0.01074809	5.982323944	0.664940516
CSN3	-0.012075136	6.011129238	0.664973108
LOC648456	-0.009959329	6.157799136	0.664992662
LOC642335	-0.008069626	6.008048969	0.665027372
ZBTB9	0.014514022	7.029591755	0.665180511
LOC652355	0.007591594	5.960694381	0.665199814
LOC642509	0.00973227	6.056735283	0.665234309
C2ORF56	0.016632786	8.204842111	0.665334929
TMEM185A	0.018634584	8.101376029	0.665368705
FLJ22531	0.009968212	6.14829708	0.665439792
MGAT4C	0.022969011	6.255566346	0.665507715
CYP4F2	0.008066016	6.041970664	0.665513858
LOC144678	0.010703188	6.023071254	0.665579753
C21ORF87	0.010846096	6.003285469	0.665611614
ADPRH	-0.025120963	6.273660493	0.665632451
PRODH	0.043556342	7.299531247	0.665681146
TWISTNB	0.013372899	6.956098857	0.665730129
PSPC1	-0.013723437	7.207227779	0.66576331
PTGES3	0.036841727	9.230819555	0.665801479
APOC1	0.022574319	6.444922737	0.665806077
SLC6A8	0.025708678	8.915992119	0.66582032
COL5A2	-0.045667002	8.591724673	0.665848202

HSD17B3	0.011501432	6.283329838	0.66598887
LOC392145	0.010231296	6.450386569	0.666033042
FLJ45422	-0.007898698	6.13722271	0.666037321
C10ORF63	-0.007987599	6.049619496	0.666109644
TCEAL5	-0.014688777	6.10212028	0.666112421
DPPA2	-0.011244213	6.091461737	0.666169093
LOC650488	0.012362574	5.878749594	0.666192648
LOC651133	0.012526614	6.102141568	0.666245898
LOC645139	0.011571556	5.974718783	0.666296327
LOC652575	0.010117843	6.07951202	0.66636158
CCL3L1	-0.010206876	6.145614591	0.666382992
LOC648926	-0.011694144	6.30373624	0.666384627
HOXC9	0.014326869	5.922214045	0.666529604
LOC649618	-0.01008123	6.205179906	0.666551054
KCNA3	-0.01267962	5.911231267	0.66668643
SUPT7L	0.011701411	6.325838419	0.666743946
LOC647444	0.008039182	6.014293807	0.66682249
TTTY7	0.009051911	6.038026118	0.666884598
LOC645868	0.008910435	6.055731716	0.666887216
LOC652211	0.010536252	6.174203555	0.666972952
LOC647655	0.010342697	5.976051298	0.666980177
LEPROT	0.02062051	10.18947297	0.667094931
GGT1	0.005110991	6.136472933	0.667185083
LOC646596	0.010636791	5.98304963	0.667480776
LOC651774	0.009918843	5.987880174	0.667481311
SPEG	-0.018256277	6.09701359	0.667484308
LOC729197	0.00982906	6.013676921	0.66755113
LOC729639	0.012206478	6.133579202	0.667595452
UGT1A5	0.014452669	6.163475264	0.667798846
LOC649823	0.010429765	6.048020191	0.66784244
RHOV	0.013974685	5.975470429	0.667876689
VCY	0.008718166	6.086861727	0.667889057
GJC3	0.013292105	6.110610279	0.667919895
LOC642325	-0.00817736	6.10011477	0.667920519
LOC642938	0.010609521	6.080784898	0.667955445
OR2A20P	-0.009334703	6.161763575	0.667962591
PANK3	0.015450608	6.666815953	0.668131098
LOC646905	0.010304879	5.939182735	0.668144828
SPATA1	-0.012203652	6.082091536	0.668222767
NFASC	0.005749824	6.170458012	0.668262969
LOC284296	-0.015299387	6.072055909	0.668305819
ELMO1	0.008347582	6.519348225	0.668313397
LOC650822	-0.010828162	6.161545032	0.668429421
ISOC2	0.025344248	8.285393848	0.668453349
LOC653143	-0.011173216	6.073379835	0.668457111
LOC651008	0.009852658	6.133486444	0.668486402
ZYG11A	0.007150066	6.017770866	0.668493986
LOC644047	-0.011831941	6.133323834	0.668568447
VAX2	0.014001188	6.173429677	0.668762825
KIAA0495	-0.018239306	7.389388336	0.668950459
CUL1	0.021013216	8.203607021	0.669155291

ACTL6B	-0.011119159	6.113340909	0.669165812
LOC651249	0.011320504	5.889325405	0.669236264
LOC643300	0.021262974	6.595756303	0.669331009
BUB3	-0.016694887	8.609425036	0.669482558
NBPF5	0.010785941	6.066401662	0.66954617
C19ORF55	0.010749338	6.328159635	0.669576266
LOC650491	0.015227935	5.944960188	0.669605495
C3ORF32	0.018610255	6.559961789	0.669630238
NEUROD6	0.010023379	6.087319664	0.669672341
FOXD3	-0.010745255	6.097976921	0.669693366
CXCL13	-0.014387232	5.946974308	0.669762347
LOC255313	0.011174648	6.023450323	0.669803385
TIMM13	0.021997375	6.030049821	0.670036683
LOC644165	0.009999868	5.940533793	0.670114416
CD244	0.01054468	6.173139061	0.670133859
LOC646971	0.009601006	5.981661705	0.670258326
FLG	-0.011866431	5.930483888	0.670351306
CEACAM8	-0.010495701	5.927858224	0.670355484
LOC650975	0.010313024	6.084709681	0.670360919
YKT6	0.015156506	5.975478536	0.670407589
LOC642077	0.011319122	6.19977098	0.670498024
LOC646887	0.009742927	6.105343736	0.670541509
BHLHB2	0.055734657	9.761490894	0.67070875
SOX5	0.006005607	6.023468051	0.670809087
SPRY3	-0.011028198	6.085195546	0.670879172
LOC387701	0.011230679	6.057650591	0.671039071
HTR3B	0.014074752	6.079474134	0.671087686
NETO1	-0.006025005	6.014321067	0.671154466
USE1	0.018923648	7.334829898	0.671185604
OBP2A	-0.012560785	6.190441733	0.671203666
LOC643449	-0.007479509	6.05877039	0.671249932
CNTF	0.01045473	6.024284451	0.671362174
NLE1	-0.008576739	6.266184051	0.671375846
FAM71A	-0.018431579	6.068664093	0.671460174
LOC645769	-0.007441733	5.949249487	0.671577924
GSC	0.01575894	6.143844836	0.671711583
DMRT1	-0.01143943	5.975167522	0.671947904
ANKRD53	-0.010481964	6.170127461	0.672291235
LOC645010	-0.011080053	5.945697852	0.672434849
BCMO1	-0.009606494	5.949476731	0.672492211
KRT82	0.011647499	5.976541548	0.672666798
LOC642106	-0.012862208	5.903412061	0.672687729
LOC648460	-0.009722295	6.129958528	0.672712388
CYP2A13	0.007607856	6.06133133	0.672767036
GPR135	0.008301969	6.070677626	0.673017502
FAS	-0.009881339	6.108790902	0.673057112
H3F3B	0.023412986	9.30178688	0.67306325
LOC651219	-0.012046162	6.021473166	0.673083187
NBR2	0.019621502	6.05147612	0.673110968
LOC643378	-0.011675883	5.939100952	0.673123954
NXPH3	0.014295704	6.364397372	0.673180587

LOC648200	-0.010510232	6.156983986	0.673187225
LOC390738	-0.010594133	5.960419402	0.673188633
LOC732226	0.010004219	5.995844026	0.673351173
LOC642918	0.006769034	5.984498699	0.673378266
C9ORF98	0.013003396	6.101621253	0.673411262
TTC9C	0.011753588	6.677051193	0.673431163
ADAT1	-0.015839349	7.250589098	0.673568464
SNX22	0.01420571	6.270367472	0.673572299
LOC646246	0.010495211	5.89035265	0.673763227
LOC644663	0.010313895	6.096373196	0.673810501
LOC400019	0.012338787	6.164475108	0.673844355
LOC730841	-0.011997845	6.498365432	0.673865607
LOC150297	-0.010851111	6.032442563	0.67390206
IL12B	-0.01191101	5.983664513	0.674011414
TMEM200B	0.012849177	5.906316303	0.674023118
LOC653203	-0.005969844	6.03896653	0.674042066
FAM62A	-0.025096865	8.668456645	0.674139087
LOC158257	-0.009885544	6.200882372	0.674305591
DIAPH3	-0.005780302	6.128889441	0.674351291
HIST1H4F	0.010121927	6.039645247	0.674387278
C2ORF43	-0.012611298	6.696432747	0.674442003
FNDC8	-0.011527385	6.082412788	0.67446816
MMP11	-0.022226197	6.864069262	0.674579074
NUS1	0.015026327	6.792678838	0.674720826
E2F1	0.018146951	5.984131015	0.674844614
VRK2	0.017007474	7.094302442	0.674848156
LOC643133	-0.011502141	5.959038825	0.674868631
SMEK2	0.0202048	7.983758559	0.674904742
CD1A	-0.01128447	6.027817192	0.675008781
TRIM7	-0.00925875	6.436034152	0.67501519
LOC650062	0.010876166	6.00571609	0.675057464
DFFA	0.011400123	7.593782497	0.675150506
DNAH12L	0.009108559	6.006623976	0.67538059
AJAP1	0.008203458	6.190473388	0.675396348
FAM148A	0.009390004	5.87664121	0.675420907
AQP5	-0.010631352	5.976103045	0.675622537
BMP10	-0.013303897	5.958585655	0.675703073
LOC732206	-0.010399299	6.015892223	0.675754657
OR52N2	0.013827163	6.597845095	0.676048953
ACMSD	0.016310166	6.983624579	0.676105341
C6ORF156	-0.010259304	5.925319334	0.676159579
LRRC19	0.012075546	6.157654257	0.676213378
LOC474170	-0.010197643	6.329103205	0.676265381
LOC654191	0.021741245	7.223306543	0.676325067
LOC653658	-0.031856039	11.6994797	0.676406731
FAM136A	0.017530853	8.839390985	0.676565856
C13ORF16	-0.012091712	6.0756652	0.676631749
OR10V1	0.012111623	6.010991402	0.676764585
LRPPRC	0.018747839	8.851774286	0.676877089
PEF1	0.018853248	8.421016685	0.676998464
LOC645963	0.011380207	6.136313337	0.677013894

CAND1	0.019966297	6.596816105	0.677106439
FLJ46836	0.021854993	5.966586657	0.67717787
NARG2	0.008804915	6.599861668	0.677180028
LOC643284	-0.019020726	13.54419521	0.677206757
LOC643432	-0.009435388	6.148004206	0.677245636
DHRS2	0.00881486	6.118346965	0.677307334
HOXD4	-0.010893173	6.186402025	0.677568431
TUSC2	0.014792342	7.292401983	0.677588854
LOC643983	0.010709995	6.030844725	0.677625119
MIER3	0.012189779	6.198010609	0.677690213
LOC344065	0.011662287	6.236930611	0.677754331
C8ORF74	0.007879647	6.05821683	0.677817469
PTPN20B	-0.006571127	6.068360147	0.67784239
C20ORF62	0.009989844	6.295569037	0.677865722
CIC	0.015337427	7.260510859	0.67790347
INSIG1	0.017867933	7.525090514	0.677926212
LOC731042	0.009959549	6.004073556	0.67794113
C16ORF42	0.017266903	7.818276255	0.67802985
DBX1	0.013033416	6.049118475	0.678071945
LOC644433	-0.010796709	6.040111461	0.67809442
LOC441193	0.014929589	6.117345696	0.678171162
LOC643089	-0.010334566	6.055914512	0.678257069
EGLN3	0.03414098	7.926689628	0.678289572
SIX2	-0.0116784	5.995799885	0.678311238
LOC652874	0.010587925	5.906489435	0.678344728
LOC642365	0.009593797	6.121572654	0.67839349
FLJ14186	0.010516708	6.098308909	0.67839947
SLC35F4	-0.011165514	6.156670802	0.678478817
UGT2B28	-0.00936584	6.019495961	0.678536797
C6ORF218	0.010672291	6.133882676	0.678605388
LOC644372	0.012358062	5.960268482	0.678637571
RPL10	-0.007720466	6.06924962	0.678664292
MLL2	0.018956683	6.120709972	0.678665157
LOC644789	-0.011569018	6.301601733	0.678668241
MRPL37	-0.01413472	11.14187412	0.678821655
LRRC43	-0.011423232	6.158754595	0.678915216
SBSN	-0.01013169	6.010875997	0.679047136
FOXH1	0.013564504	6.780715009	0.679083186
INS-IGF2	0.00784681	5.972693463	0.679110536
USP32	-0.010245521	6.123364057	0.679136331
LOC649596	0.011763643	6.439349124	0.679142898
XPO1	0.023998496	9.558545408	0.67926491
CCDC129	0.015634901	6.08273061	0.67935017
C11ORF40	0.012154109	5.945684572	0.679410152
LETM2	-0.010237173	6.095763907	0.679440222
C14ORF104	-0.020437161	7.06317313	0.679500464
FLJ41733	-0.012221699	6.071960238	0.679509836
LOC644046	0.01048265	6.013046504	0.679534143
OR52I2	0.010577825	5.899254734	0.679858786
PTCHD1	0.010694813	6.037068211	0.679917621
LOC651821	-0.010018339	6.031142138	0.679957239

RGL3	0.015719472	5.996125821	0.680051364
LOC619208	0.018087056	6.42607927	0.680144238
LOC649757	0.010513691	5.939249249	0.680289866
RNGTT	-0.020294326	8.677666828	0.680303612
AFP	0.010610666	6.12493856	0.680420115
RNU2-1	0.011136606	6.034455211	0.68045142
LOC644508	-0.011710764	5.91479943	0.680520385
LOC652161	0.008992615	6.219980369	0.68055216
LOC646870	0.009825377	6.141922947	0.680643312
DUSP9	0.015225625	5.923815024	0.680824237
PRG-3	-0.00656531	6.183977753	0.680944656
LOC644958	-0.009585413	6.111717442	0.680982751
BACH1	0.006997021	6.12527469	0.681034378
LOC645040	-0.009429594	6.04723656	0.681072477
LOC646236	-0.009962667	6.029608366	0.681148118
LOC150051	0.009369522	6.228412122	0.681247272
ITSN2	-0.011776716	6.148406073	0.681304248
TTTY11	-0.008747977	6.161186969	0.681335712
CNR1	0.01204169	6.086755989	0.681839359
IPMK	0.015961031	6.099801667	0.68193576
LOC730412	0.013998586	5.93494814	0.682037187
CCR8	-0.011966638	5.953918232	0.682039236
LOC647349	-0.027524712	8.649677339	0.682114299
FAM59B	-0.010631902	6.142530298	0.682287932
LOC645445	0.010843178	6.162416754	0.682335549
DGKZ	-0.007035358	5.991203085	0.682347005
LOC646560	0.009684326	6.12626845	0.682437383
LOC641982	0.009800961	5.998400023	0.682464851
CAMK2N2	0.010820721	6.039902992	0.682489802
LOC644377	-0.009816489	5.983902791	0.682520169
UTRN	0.013901496	6.986848932	0.682526426
ADCK1	0.016872018	7.343533569	0.682660294
PPIC	-0.021818446	9.587975621	0.682725615
AADACL2	0.009279675	6.063072745	0.682840015
LOC391427	0.009362974	6.073465719	0.682888315
LOC644714	-0.005756964	6.064969567	0.682979467
TRIM75	-0.009238389	6.082761468	0.683178074
DTWD1	-0.012771587	6.688459785	0.683282757
SNORD38B	-0.009607851	6.062142661	0.683333536
ZNF862	0.010667516	6.067749749	0.683404634
FLJ21687	0.016702155	6.121791072	0.683479457
LOC644361	-0.012023932	6.003743462	0.683509852
TRIM60	-0.007975903	6.170710841	0.683562021
LOC390811	0.00970617	6.153562674	0.683611529
C4ORF7	-0.010595796	5.991581374	0.683690915
TAS2R16	-0.011013509	6.08086266	0.683693487
LOC643039	-0.010654533	6.017460052	0.683750605
LOC646906	0.017343796	6.000347563	0.683766509
CSRP2	-0.026142543	7.907527232	0.68377742
LOXL1	0.018136621	6.581793264	0.683779779
LOC642853	-0.009253349	6.162611361	0.68380919

BRF1	0.006828174	6.237530035	0.683828984
LOC647306	0.009854098	6.173236682	0.683916017
IFNA2	0.009906817	5.979734052	0.684049474
STUB1	-0.020888082	9.484886234	0.684092217
LOC196120	-0.009147339	6.00275189	0.684117402
C10ORF128	-0.016028384	6.72435051	0.684164268
OR2AE1	0.010330711	6.021919751	0.684179968
GNG8	0.013886162	6.192374476	0.684341254
LOC644151	-0.011608398	6.060598451	0.684421597
CYP2A6	-0.009381404	5.957282676	0.684422176
C6ORF10	0.009796424	6.104854025	0.684479819
HBS1L	-0.007026524	6.165257692	0.68451943
MCM6	0.021962333	8.4200309	0.684534853
LOC651286	0.008453487	6.062156356	0.684560354
C2ORF19	-0.011633002	5.965502725	0.684565547
LOC402677	0.01264688	6.238583396	0.684582636
YSK4	0.006588148	6.002037109	0.684616405
LOC441340	-0.010366917	6.061047012	0.684704036
GBAP	0.012348807	6.391634424	0.684785599
DTYMK	0.010075371	5.929763969	0.684937215
KIAA0913	0.016741317	8.78905234	0.68497648
LOC644865	-0.010832016	6.263746504	0.68512685
PRR4	-0.013619444	6.490775468	0.685199155
LOC654115	-0.011120638	6.289756338	0.685208709
LOC642851	0.010602157	5.927529246	0.685217633
OR4C3	-0.007865484	6.119799922	0.685224792
FOXB2	-0.014987446	6.083302798	0.685233515
LOC646653	0.009736054	6.214624187	0.685273081
HIF3A	-0.006808575	6.158049168	0.685378967
OR6A2	0.011791445	6.050171784	0.685403635
NSUN5	0.011531834	7.103219145	0.685428129
LOC400455	0.019715662	10.28930911	0.68545054
SCAMP1	0.01557926	9.05610775	0.685643352
CNOT8	0.015590473	7.667912654	0.685705554
WFDC12	0.008745264	5.963021886	0.685862441
RBMY1J	0.004730182	5.997722627	0.685890455
C10ORF95	-0.009646857	6.111540395	0.685892167
KRT25	-0.010575829	6.00256204	0.68593789
LOC652217	0.008440528	6.059878671	0.68609428
LOC645307	-0.009987231	5.998446184	0.686170103
CHRNB3	0.01535927	6.027003575	0.686287147
TXNDC3	-0.010078971	6.004767756	0.686316072
LOC643373	0.010364583	6.175625256	0.686436248
KRT33A	0.007425329	6.05761223	0.686463321
GSDMA	0.010062229	6.082428773	0.686623319
TMPRSS9	-0.010918759	6.011517654	0.686657753
EMR3	-0.007937964	5.987108152	0.686760746
LOC644183	0.008488079	5.957187344	0.686814463
OPCML	0.007362746	6.369139589	0.686858467
LOC729012	-0.008884105	5.9566391	0.686912628
ZNF700	0.010548312	7.282581066	0.68694293

BCKDK	-0.014814917	10.9810743	0.686987228
OR52E8	0.010696982	5.899640495	0.687011216
LOC648502	0.010242956	6.0127256	0.687051331
LOC653166	-0.008988189	6.098945784	0.687108876
CDKL1	0.009499004	6.241905826	0.687115627
MAP1LC3C	-0.012545717	6.350580323	0.687116043
LOC441454	-0.011489224	6.106323794	0.687208347
LOC644166	0.008919505	6.046576408	0.687232057
LOC642675	0.010364219	5.932856821	0.687247002
TCTE1	-0.009362889	5.941185996	0.687275053
PSMB2	0.016993143	9.828074694	0.687297752
AGBL1	-0.006973013	6.02195429	0.687366072
ZC3H11A	0.012241394	6.012180468	0.687405342
ZNF274	0.012337321	7.738228078	0.687426867
GUCY2E	0.011059433	6.123828407	0.687619769
UGT1A6	-0.006143505	6.005193996	0.68774373
TIAL1	0.012111644	7.694478815	0.68783454
KRT2	-0.009604915	5.959707835	0.688118618
TAF5	-0.010739196	6.73025756	0.688144603
LOC728904	-0.009558452	6.009847111	0.688539433
LOC441296	-0.010415533	5.976450418	0.688630564
LOC651648	0.011003258	6.187899078	0.688661677
GCGR	0.011102843	5.975313529	0.688908883
LOC642449	-0.012524522	6.129065543	0.689000672
CETN1	-0.007907279	6.169204045	0.689003171
TAF10	0.018545903	9.717081348	0.689139686
LOC652563	0.009513633	6.130239925	0.689178331
SLBP	-0.019841575	7.254534272	0.689237649
WDR17	0.006160054	6.083225816	0.689247994
ANKRD20A3	0.009313316	5.990488869	0.689313121
PLA2G6	0.006765511	6.232114415	0.689338842
SPATA12	-0.010478582	6.049197614	0.689403016
LOC93556	-0.009285048	5.944341164	0.689457042
LOC645649	0.009852558	6.141663413	0.689473402
LOC649999	0.011097431	6.227568265	0.68948652
ZNF526	-0.023274637	6.616179747	0.689525857
INGX	0.010220848	6.157255951	0.68955034
LOC643703	0.009692539	6.086860799	0.689583315
LOC652492	0.009376669	6.285821892	0.689660809
LOC649350	-0.009750412	6.054770933	0.689685313
PP8961	-0.007224735	6.034939366	0.689767885
RHD	-0.009290868	5.976438863	0.689803483
LOC728951	0.007949624	6.122834179	0.68985767
FOXN4	0.010295286	6.014937303	0.690024001
FLJ27365	-0.016271073	6.254368689	0.690121986
RNASE9	-0.009530868	5.865590041	0.690209935
SYT10	0.010409905	5.887327571	0.690215286
LOC147670	-0.009208191	6.17811266	0.690257732
SYT8	0.011418046	6.069213408	0.690322801
LOC730413	0.009656588	6.164738379	0.690415523
CYP4A22	0.006803364	5.985853338	0.69044668

PPM1D	0.018008099	7.091224131	0.690465143
CBLN3	-0.013904117	6.679212771	0.690587405
CYORF15B	0.017171144	6.548674064	0.690619735
KLF17	-0.013520876	6.010185064	0.690816463
LOC727775	-0.011423788	6.072862538	0.690897292
LOC147650	-0.009288064	6.236513406	0.691022403
C14ORF39	-0.006675373	6.12838191	0.691041217
C20ORF79	-0.009182189	6.101709187	0.69130431
HECA	0.032314684	8.32778934	0.691333099
FLJ46284	0.010232234	6.078804439	0.691415967
UBE2CBP	-0.007876028	6.314104494	0.691528542
LOC647357	0.008249463	6.198498748	0.691675582
LOC642434	-0.009858572	6.11708918	0.691721798
MLXIP	-0.009783352	6.116047363	0.691736447
FCRL6	-0.011801627	5.927091885	0.691775125
LRIT2	-0.009853694	6.028783848	0.691843614
C11ORF77	0.011458953	6.39907919	0.691876242
SLPI	-0.009730277	6.024912402	0.691909306
CCDC9	0.010209385	6.170884034	0.692032414
LOC649944	0.007238508	5.936825236	0.692147277
KRT78	0.011146265	6.04372616	0.692240038
C14ORF166B	0.008834488	6.14357522	0.692287245
SLC33A1	0.019109026	6.811625394	0.69229508
BAG1	0.010639479	6.289842824	0.69233456
C1ORF182	-0.010782044	6.159502551	0.692373842
MAGEC1	0.00534421	6.042855107	0.692531886
MSX2	-0.012155606	6.188001038	0.692592062
LOC650407	0.009612153	6.002468289	0.692628079
IRF4	0.014290236	6.224231533	0.692689868
C12ORF31	0.027785905	8.473637265	0.692711609
LOC91948	0.011222391	6.126673539	0.692724204
SLC39A6	-0.013987488	7.073880889	0.692774031
C19ORF34	-0.012374616	5.938168021	0.69281215
LOC652777	0.010938638	6.163228976	0.692815159
SNHG5	0.046473469	10.26556084	0.693009802
MIPOL1	-0.009347909	6.195994912	0.693106536
EME2	0.011230248	6.134998077	0.693106956
LOC643047	0.010538655	6.095640948	0.693197421
HES7	-0.011708039	5.8033181	0.693272352
CNTD1	-0.010513408	6.065492316	0.693296964
LDOC1L	0.019362888	8.29572529	0.693337257
LOC283398	-0.010773655	6.121364169	0.693378097
GTSE1	-0.011568621	6.167531578	0.693392529
SDC1	0.008081199	6.19739275	0.693485029
LOC646429	0.010258338	5.96071926	0.693500408
MUC5AC	-0.009537446	6.218315277	0.693628855
AMELX	0.009028521	5.988596607	0.693693951
LOC650594	-0.009122095	6.099654936	0.693734913
KLHL9	-0.019945087	8.633231098	0.693790346
RPS26	-0.027116691	10.36530461	0.693807806
OR8U9	-0.006852636	5.963300975	0.693995595

LOC642420	-0.009388023	6.078751841	0.694010003
LOC642762	0.009395561	6.112529462	0.694065612
LCN8	-0.009780523	5.923640589	0.694068578
LOC401268	0.015147015	6.0598971	0.694174582
C17ORF66	0.009573186	6.016203396	0.694187142
VNN1	-0.01268533	6.233654646	0.694376645
LOC256483	-0.009294944	6.057775399	0.694437056
MAG	-0.007159365	6.160167601	0.694440288
LOC339529	-0.00837711	6.024501758	0.694553751
RGSL2	0.011032089	6.082234804	0.694622806
APC2	0.009383368	6.099675573	0.694650305
LOC652562	-0.010669499	6.020920203	0.694828505
HDLBP	0.006610287	6.401258868	0.694971919
FAM71C	-0.00881297	6.051790883	0.69499248
SETD1A	0.016326196	7.741041613	0.695120003
EFHC2	-0.040189082	7.511491482	0.695135088
FAM108A1	-0.006950646	6.067338889	0.695240247
OPN1LW	-0.010810203	5.989391908	0.695346132
ADAM9	0.014881143	7.176840674	0.695363069
LOC642743	0.012469439	6.08102862	0.695452479
GPC3	-0.01641016	6.432119158	0.69552876
KRTAP2-2	0.007475878	5.969762077	0.695543536
LOC650852	0.00982834	6.17113482	0.695583693
IL17C	-0.009832556	6.26189477	0.69567622
WDR82	0.01711145	9.351891097	0.695697902
LOC442606	-0.010005158	6.144797755	0.695720016
NCR2	-0.010805788	6.038450436	0.695733143
PHTF2	0.017933444	6.295102303	0.695970917
PRDM14	-0.007207947	6.068693857	0.69600135
MMAA	-0.013107935	6.257647688	0.696208726
LOC648733	-0.009362305	5.996749398	0.696260476
GALNT2	-0.01343817	6.401227665	0.696492801
KCTD9	-0.018054662	8.39778233	0.69650656
LOC649324	0.009820321	6.01413841	0.696514157
LOC644257	0.010708843	5.963787096	0.696541564
FAM119A	-0.023893644	10.47564463	0.696553962
MYO3A	-0.011793022	5.950420301	0.696566951
LOC642515	-0.011547251	5.928034066	0.696607161
LOC644733	0.011588795	6.274283011	0.696611159
POLN	-0.010099794	6.090917611	0.696689063
LOC645033	0.008951796	6.030564467	0.696800314
MOGAT1	-0.015702601	6.518668566	0.696820701
GPR125	-0.008857716	6.52999799	0.696939997
LOC643320	0.01056007	6.10502798	0.696996635
KCNG4	0.006777557	5.934629049	0.697011208
GYPE	-0.005116204	6.074458601	0.697025593
LOC646649	0.008410347	6.002864355	0.697036557
WBP11	0.01448244	8.968359793	0.69706556
LOC651614	-0.01053815	6.033970025	0.697115881
UBE2B	0.012317356	6.664318235	0.697135437
LOC284274	-0.007543661	6.002439655	0.69722414

LCE3C	0.008535903	5.969903593	0.697318992
BAGE5	-0.015178186	6.410753128	0.697402657
LOC642914	0.009931794	6.137999922	0.6974071
TGM1	-0.010448085	6.122693518	0.697412653
LOC648476	-0.007528535	6.181316578	0.697423188
FLJ13224	-0.007233611	6.122392606	0.697586833
OR56A1	0.009046467	6.063003674	0.697593012
LOC441294	-0.006799425	6.113060789	0.697691389
GAGE1	-0.004782788	6.08884967	0.697804908
OVOS	-0.010199507	6.133783513	0.697813347
LOC653241	-0.014210238	5.999835049	0.697930122
TMPRSS11F	0.010024747	6.158331659	0.697943352
FLJ36166	0.009689464	5.936340391	0.698050435
PGCP	0.018413567	8.539367327	0.698074087
PAK3	-0.010961629	6.12764053	0.698163344
CLCN4	0.014167456	6.32372445	0.698221533
RFPL1S	0.007626766	6.149331621	0.698283962
ARMC2	0.007203047	6.121722906	0.698328036
LOC651711	0.010853808	5.970636015	0.698515662
LOC652062	0.010282033	6.176827683	0.698810605
HPSE2	0.009105015	6.057053219	0.698846919
ZNF280C	0.007113049	6.494786883	0.698943408
LOC651280	-0.008492625	6.0891867	0.699007068
LOC646023	0.010225678	6.119799199	0.699095447
MOBK1B	0.017982367	6.173939197	0.699207898
TAS2R39	0.009342924	6.150321448	0.699209601
ACTL8	-0.012566578	6.084405056	0.699284022
GSG1	-0.005607143	6.127743226	0.699393743
GAGE12I	0.020656176	6.095218241	0.699399069
BMX	-0.006522646	6.160628504	0.699444306
LOC652768	0.009827686	6.126921321	0.699476353
OR5I1	-0.009943204	6.0187715	0.699606306
TBX6	-0.006885097	6.136662627	0.699770342
LOC654189	0.016028103	6.932856683	0.699836568
CPA6	0.010045458	5.816817163	0.699919311
LOC730099	-0.009545261	5.907023252	0.700094514
LOC647481	-0.010238384	6.060494658	0.700267586
LOC650612	-0.009651518	5.952878133	0.700278222
FXYD7	-0.010691749	6.129789798	0.700373507
PRPF40A	-0.014346548	7.054322357	0.700412514
MAP3K7IP2	-0.013621219	6.560636972	0.70042867
PRB2	-0.007214143	6.107894496	0.700440535
R3HCC1	-0.013851177	9.327696758	0.700475073
SMTN	-0.024953295	8.741840011	0.700482048
PHF16	-0.01366724	7.454856812	0.700534386
IMPG1	0.00697963	5.985615962	0.700690716
LOC401152	-0.017822759	8.586481745	0.700810386
INSL3	-0.009664751	5.920126527	0.700857095
HSPB2	0.024155014	10.38549751	0.701021247
G0S2	0.048949944	7.966843218	0.701031803
DUSP10	0.007632793	6.232210703	0.701103315

LOC642761	0.008792622	6.012578706	0.701122938
AADAC	0.01018957	6.073549532	0.70116537
MOV10L1	-0.010109491	6.090872827	0.701228688
LOC347119	-0.009864575	5.903663642	0.701305725
OR10Q1	-0.009112111	5.982223621	0.701312081
S100A1L	-0.006711859	6.128014423	0.701320094
FAM55B	0.010298841	5.986463032	0.701353016
LOC645104	0.00778071	6.028379066	0.701387463
LOC654340	0.009348538	6.049737705	0.70144215
CALB1	-0.010196389	6.013495857	0.701508061
INSL5	-0.012010292	6.092659473	0.701560636
LOC389442	0.00885279	6.078591391	0.70156277
LOC340069	0.009933542	6.000905859	0.701637694
LOC642008	-0.009623216	6.050664561	0.701654261
FAM154B	-0.011912315	5.99182163	0.701695552
FOLR1	-0.005411674	6.135538738	0.701750486
LOC648222	0.009423606	5.986750609	0.701754611
RAD23B	0.021343166	9.047892478	0.701883812
LOC644650	0.017192465	6.037725692	0.701895154
TRAF3IP3	0.009340394	6.030231097	0.702000594
OR3A2	0.009871738	6.075912458	0.702049004
C1ORF135	-0.009509996	5.976594903	0.702242432
SUV420H1	-0.009363406	6.85523542	0.702399152
C14ORF181	-0.014022511	6.214232429	0.702510302
C10ORF88	0.015882784	6.956069563	0.702530494
LOC652305	-0.011550774	5.9896263	0.702597272
SOX21	-0.011411369	6.097114271	0.70268766
RSPO4	0.007217236	6.040033981	0.70271299
LOC648801	-0.009032505	6.00340433	0.702756216
LOC643938	-0.009032475	6.084012659	0.70275744
RASGRF1	0.008219653	6.093716883	0.702786166
ANXA8	-0.007716707	6.118091902	0.702833835
FAAH2	0.01273848	6.149034788	0.702859427
LOC642607	0.00936489	6.112770517	0.702924412
HRH1	0.011846521	6.426015791	0.703064096
PNPO	-0.015816069	9.805887235	0.70308526
SLC22A1	-0.008479685	6.213678575	0.703381169
PRUNE2	0.007380339	6.114602337	0.703562973
ZNF229	0.011134355	6.015903828	0.703607854
MYO15B	-0.00868425	6.069767196	0.703695022
RAET1L	-0.008621731	6.05349193	0.703707266
POU4F3	-0.009896254	5.986416343	0.703770144
STAC3	-0.012578067	6.087271407	0.703775772
LOC649198	-0.007046651	5.99777987	0.703790767
LOC389422	-0.009327794	6.00518663	0.70382522
RPA2	-0.020772387	9.95943734	0.70383562
UPRT	-0.017986946	8.17319785	0.703861718
ADAM7	-0.009972123	6.260152608	0.703865995
TAS2R44	0.009146169	5.967144827	0.703867076
LOC643017	0.00972036	6.08832404	0.703876655
INHBB	-0.035635329	6.800565373	0.704044679

LOC645391	0.009494306	5.931246275	0.704069561
LOC168474	0.010052706	6.303023896	0.704113226
LOC440910	-0.006470896	6.073005936	0.70412439
OR6C6	0.009782126	6.014544083	0.704133915
LOC647634	-0.009510101	6.099031046	0.704181954
LOC730952	0.015825926	5.96877798	0.70420896
DMP1	0.007639019	5.996714986	0.704345934
CYP2W1	0.013019227	6.039702908	0.704513945
LOC389267	0.009318105	6.081166024	0.70480908
GSTT1	-0.113097079	9.268842452	0.704823243
CDC6	0.009805153	6.054111657	0.705047266
TCF7L1	-0.012090103	6.512186116	0.705125023
LOC654088	-0.007384718	6.07238039	0.705155861
NDRG3	-0.018708879	8.118856088	0.705296304
FLJ12078	-0.014634651	6.572269685	0.705379618
LOC132707	-0.008153012	6.057000609	0.705490127
SPG21	0.014653453	6.969387369	0.705557855
OR1L4	-0.006964502	6.114044469	0.705562093
PCDHA9	0.005975469	6.101979003	0.705589976
LOC652551	0.009954637	5.954470447	0.705591259
FASTK	0.020562374	8.648694999	0.70563359
RUFY3	-0.012019044	7.028953691	0.705762089
ASTL	0.009755366	6.331532472	0.70584131
GDF1	0.00596585	6.044163281	0.705895755
TBC1D23	0.02721815	7.036018878	0.705931753
CXORF30	-0.010638834	5.979651792	0.706062644
PGLYRP4	-0.009492074	6.441767311	0.706110384
LOC728137	-0.015143738	5.890964302	0.706112676
MUCL1	-0.009729288	6.155089609	0.706114506
LOC644756	0.009885611	6.245957727	0.706140157
ENTPD1	0.013690474	6.987033677	0.706145421
DUOX1	0.006374768	6.136227107	0.706242733
DKFZP686J0529	0.007694194	6.06179968	0.706252326
BUB1B	0.015838763	6.077016085	0.706267789
SLC37A3	-0.0095753	6.660342319	0.706488682
LOC653189	0.01071631	6.053376872	0.7065297
KRTAP21-1	-0.010414125	6.124219315	0.706542799
SSX8	0.009911522	5.898329807	0.706549623
LOC648359	-0.009650136	5.979301324	0.706579562
LOC651515	0.010461389	6.016521276	0.706605161
PAPOLB	-0.008836098	6.020767771	0.706623647
OR52K1	-0.010421832	6.31861706	0.706636686
COL9A3	0.00933843	6.200929701	0.706654291
FTCD	-0.007532984	6.179013976	0.706696779
SLC2A7	-0.009469683	5.961644626	0.706803657
C21ORF58	0.006285872	6.083603672	0.706828439
DPYSL5	0.010395436	6.256163974	0.706911465
FLJ30679	0.008799396	6.103417323	0.706949826
MAP3K7IP3	-0.006396272	6.038809282	0.707008973
LTK	-0.012778329	5.988163857	0.707096807
LOC649587	-0.010838522	6.014876377	0.707191518

GIMAP2	0.014665568	6.437591762	0.707203221
DMRTC2	0.011345706	5.925002159	0.707268784
KRTAP1-5	0.00949237	6.07618417	0.707276435
TSPY2	-0.006548211	6.101789311	0.707315419
ARHGEF5	0.009673772	6.118356917	0.707328922
OR52R1	-0.009668572	6.039161792	0.707385678
LOC645208	0.010034584	5.952651646	0.707677088
LOC647361	-0.016248856	14.01603789	0.707701688
TXNDC6	0.016205432	6.00338134	0.707723172
BTN2A1	-0.015976287	7.799819047	0.707736328
ERGIC2	0.030975684	8.593285961	0.707754076
LOC286467	0.00684409	6.164914566	0.707802505
LRRC52	0.009901121	5.982891809	0.707842304
NCAPH	0.016526442	5.96872899	0.707892864
LOC648834	-0.011187088	6.057342099	0.707910048
PDLIM4	-0.011078344	6.025012278	0.707972621
LOC642475	-0.009801411	5.976386025	0.708089385
TTN	0.024511013	7.401004198	0.708158807
LOC342897	-0.009074672	6.126564835	0.708186072
ZFHX2	-0.010526944	6.068795054	0.708193666
LOC653581	-0.008997636	5.998850673	0.708366241
LOC652499	-0.008967995	5.925015905	0.708456834
LOC652677	-0.009474924	5.928738058	0.708480266
LOC643584	0.009540043	5.989703683	0.708576687
PTPN20A	0.034296869	7.475873937	0.708580677
KRTAP9-3	-0.011123447	5.969467648	0.70865361
LOC649144	0.008672739	6.166964148	0.708665755
C9ORF57	0.010952851	6.209472018	0.708728679
LOC642776	0.0094956	6.074492553	0.708740756
LOC642832	-0.010394148	5.939370768	0.708744402
LOC643993	0.009305185	5.993420713	0.70874887
LOC392781	0.00939335	5.952735197	0.708757479
LOC647229	0.01122326	5.995770043	0.708824829
CCDC114	-0.009517994	5.999300446	0.70884998
OLIG1	0.010603277	6.022744836	0.708893922
AKAP14	-0.005266408	5.955560923	0.708904076
LOC400298	-0.010406444	6.012059051	0.708923642
CDX2	-0.01131244	6.039387726	0.70895321
LOC643634	0.009473471	5.943705292	0.709035055
LOC642707	0.0095788	6.212023245	0.709046386
RNASE10	0.011620894	6.620350266	0.709060339
LOC653286	0.010112004	5.899518173	0.709090568
LOC647134	-0.009136081	6.05046139	0.709108177
PMP2	0.022612804	6.759741482	0.709332874
LOC284988	0.035931194	11.1790947	0.709383424
FBXO36	0.009800762	6.016914825	0.709451423
FAM75A7	-0.009418008	5.972696886	0.709551605
CCHCR1	0.013390181	5.998273691	0.709560521
LOC647493	-0.009129696	6.085178068	0.709732647
RNF13	-0.011409156	7.145520446	0.709835915
C10ORF118	-0.020245725	6.14278583	0.709957906

LOC646769	-0.017120843	6.006929505	0.70996166
LOC139542	-0.009819971	5.982387536	0.709994267
PRY2	-0.010186227	6.166487404	0.710000806
GTF3A	0.022631503	11.50367208	0.710029507
UPK2	0.010168525	6.078630273	0.710166862
LOC644215	0.009331613	6.021297124	0.710220797
DUX2	-0.012278137	6.114041868	0.710257899
LOC642000	0.010554848	5.953165216	0.710265818
HIST1H4A	-0.010329159	5.93536329	0.71033465
OR51B6	-0.008661691	5.931105616	0.710458681
LOC648374	-0.008879254	6.053743793	0.710514158
PDYN	0.011489496	6.27076491	0.710611636
PCDHGB5	-0.010168754	6.270410688	0.710620855
HELB	-0.009520043	6.105351486	0.710671734
DEFB111	0.008918369	6.045658874	0.710686657
WDR31	-0.010661026	6.452794504	0.710707208
LOC651075	-0.010733374	6.443843774	0.710714265
LOC641858	-0.009014948	6.045003213	0.710818122
PSG1	0.009715291	5.919095932	0.710825231
MAMDC4	0.013231076	6.668523086	0.710866856
LOC648154	-0.006770432	6.022258854	0.710880037
LOC646680	0.009479631	6.093759077	0.710908401
ZADH2	-0.021696216	8.878171777	0.710949844
EFCAB4B	0.010526067	6.068471638	0.71099148
TGFB1	0.008652431	6.007496611	0.710997464
LOC653787	-0.008812399	6.074125616	0.711084362
OR2V2	-0.008738429	6.096537484	0.711193113
OVOS2	-0.004887488	6.100569685	0.711354262
LOC648309	-0.009004066	6.164131145	0.711453674
REG1B	0.010537392	5.958641707	0.71148751
C17ORF73	-0.010466147	6.436909312	0.711531514
LOC645123	0.009094357	6.058603379	0.711718277
NKX2-3	-0.009927387	6.103859752	0.711766196
LOC647802	-0.008302345	6.242862219	0.711792292
IGSF9B	0.018941195	7.146778599	0.711836691
LOC643702	-0.009193331	5.947881426	0.711890029
MC5R	-0.010074065	5.969429688	0.711902661
IGSF9	-0.009893441	5.943490536	0.711974848
LRTM1	0.012515753	6.256677612	0.712075474
LOC646706	-0.010143832	5.976302799	0.71208564
CCL1	0.009117827	5.957268054	0.712223247
OR2M1P	-0.006132114	6.023591625	0.712383026
FBXL18	0.009179474	6.09328924	0.71247361
OR52E5	0.008880421	6.096036824	0.712548521
CHD3	0.010329917	6.19618293	0.712560627
LOC388333	-0.008444448	6.031335911	0.712602706
OR6Q1	-0.009678201	6.013565631	0.712619377
LOC642169	0.008148633	6.122839065	0.712913731
CTTNBP2	0.010433715	5.990058223	0.712924676
ZAR1	-0.01061184	5.974153812	0.712971637
C1ORF52	-0.016031306	8.86387503	0.713048131

LOC646631	-0.007893672	6.070050828	0.713067598
LOC643428	0.00889248	5.955108758	0.713068027
LOC643285	0.011901717	6.076617065	0.713111428
CTRL	0.010550107	6.263295343	0.713113865
LOC642965	-0.008700198	6.021947837	0.713122686
TTC22	-0.008733495	5.975251	0.713161337
COMMD7	-0.011399475	10.84557863	0.713175124
MAGEA4	-0.00696008	6.04166202	0.71319227
LOC648761	-0.008068672	6.167748575	0.713402586
INSR	0.017134138	6.311015646	0.713688434
LOC648859	0.008766986	6.065510348	0.713716413
AMPD1	-0.009884055	6.002929347	0.713730269
LOC649379	-0.011096486	5.939238587	0.714006571
LOC650013	0.009918212	5.991044791	0.714161436
NBR1	-0.005978898	6.030943881	0.71422181
LOC653789	0.008182158	5.953752013	0.714225313
SLC36A3	-0.007180665	6.034130728	0.714291448
LOC649747	-0.009263549	6.073249019	0.714320176
LOC648689	0.009765641	6.183410363	0.71439075
LOC652290	0.010148038	6.087045439	0.714469884
KRTAP5-11	-0.01063002	6.079661933	0.714569522
LOC643406	0.009499527	6.018522433	0.714616391
LOC642633	0.00910259	6.060793053	0.714702405
CYP27C1	0.012730775	6.209840593	0.714795247
UGT2B10	0.008412531	5.936921004	0.714889124
LOC646812	-0.008484885	6.050696824	0.714960892
LOC541473	-0.009951544	6.010036407	0.715030947
CEACAM6	0.008894722	5.809927629	0.715125196
ZSCAN22	-0.009958971	6.068092858	0.715181775
EXOSC10	-0.011397736	8.619835985	0.715235797
TPT1	0.011944173	14.18011338	0.715263856
CKMT2	0.018938938	13.88423993	0.715278055
LOC650341	0.009146126	5.985678369	0.715281626
GABRD	0.009153072	6.26304793	0.715286158
C6ORF64	0.013648588	7.100456112	0.715481744
PCDHA7	0.005793596	6.057634842	0.715484752
SPANXB1	0.006529972	6.110820591	0.715516745
GLIPR1L2	-0.009732316	6.191307786	0.715579185
LOC653175	0.008268446	6.061456878	0.715664804
LOC647935	0.00845168	6.133028947	0.715726386
LOC642044	0.009088188	5.928601016	0.715779644
WFDC8	-0.007347794	6.123377976	0.715906444
LOC389036	0.007769301	6.019229948	0.715979359
LOC652049	0.009650228	6.115364275	0.716052745
LOC727837	-0.008730608	6.066987356	0.716124432
LOC645463	-0.008051878	6.026641481	0.716374576
LOC654235	0.008977468	6.226033596	0.716397487
USP44	0.007120553	6.046200317	0.716416606
CPEB4	-0.029130015	8.267129563	0.71649515
C14ORF49	0.009131466	6.198867757	0.716616796
LOC642072	0.010511432	6.184558496	0.716639713

C17ORF65	0.009838681	6.295875304	0.716684567
MGC45800	-0.009056326	6.076638399	0.716797692
MGC34774	0.00955919	6.046283149	0.716870247
NOS2A	-0.006213881	6.100601305	0.717038018
ABCG4	-0.012286537	6.452815779	0.71705538
OR9G1	-0.006428288	6.0296328	0.717128836
GREM1	-0.006288853	6.15305682	0.717157835
PLA2G4E	-0.006686697	6.127533506	0.717186182
LOC652630	-0.009809921	6.197297918	0.717196038
ZNF643	0.007088014	6.1136287	0.717226778
ACAA2	-0.022791377	9.827599796	0.717239179
C12ORF72	0.011636997	6.038499604	0.717356111
LOC652203	0.009844265	6.033191042	0.717520123
TULP3	-0.011022259	6.562821371	0.717572289
GOLM1	-0.009550667	6.29042107	0.717661976
C14ORF73	0.006897627	6.123335377	0.717710217
VSTM3	0.010907093	6.099315953	0.717782378
EFS	-0.007620512	6.293556704	0.717808748
ELSPBP1	-0.008808904	5.984720239	0.717935907
LOC51145	0.008530704	6.045531997	0.717984712
LOC653260	-0.008895705	6.097980959	0.717987199
KLHDC10	0.011439657	6.497135787	0.718106511
CGB	0.00943029	6.33248161	0.7181794
HERC2P4	-0.007456757	5.974660497	0.718181325
C17ORF41	0.004811342	6.096437047	0.718202833
WARS2	0.028774784	6.670439334	0.718214903
LOC642414	0.009509186	5.941511402	0.71821915
HFE	0.006337095	5.975362737	0.718503505
MLF1IP	0.013641889	6.022628702	0.718513021
LOC646918	-0.01008435	5.969256196	0.718556854
LOC402198	0.008982539	5.922657569	0.718672473
LOC653337	-0.006599727	6.172150368	0.718713637
PTH2R	0.008489177	6.074699901	0.718747328
LOC642530	-0.008084671	6.019634908	0.718762654
HS3ST4	0.010511196	6.107453487	0.718929419
DSCC1	-0.016483405	6.241154554	0.71895528
LOC650188	0.009659743	6.127222814	0.718994396
HS6ST3	0.008559356	6.066981662	0.719080678
RBM46	0.006359586	5.925639808	0.719106094
GPR139	-0.009614957	6.096694644	0.719182384
HIST1H2BF	-0.008106311	6.10553003	0.719401942
LOC642733	-0.008597017	6.066988609	0.719734406
LOC440896	-0.008884623	6.038882141	0.719767407
LOC400456	-0.005610935	6.084458462	0.719814923
GAGE6	0.012099715	6.071921323	0.719872577
PCDH10	0.008997634	5.999620134	0.719933973
LOC442465	-0.009985767	5.817777628	0.719936356
LOC642808	-0.008783111	6.015382849	0.719968893
ZNF569	0.008762331	6.001940636	0.720029054
EML1	-0.017042726	7.630900991	0.720124396
KCNH4	-0.010614854	6.211127312	0.720172342

LOC387756	-0.009811906	6.0921426	0.720191078
LOC642316	-0.010723217	6.084765035	0.720203728
LOC646403	0.009837926	6.11409746	0.720600728
OR52W1	-0.009759776	6.156563475	0.720661824
LOC644838	-0.006329584	6.040593576	0.720705134
LOC647315	-0.008486732	5.992072282	0.720706726
OR51M1	-0.010248655	6.096044262	0.720963556
CTNNAL1	0.019994276	9.58083232	0.72108309
FLJ37307	0.009244817	6.171187009	0.721160721
LOC644695	0.007843974	6.018465018	0.721309685
LRCH2	0.010728642	6.666818836	0.721413612
LOC650713	0.008241213	6.03271015	0.721424908
HOXD10	-0.008989003	6.000838229	0.721494739
LOC647360	0.014042535	6.05757684	0.721555469
LRRTM4	-0.010536648	6.544209314	0.721592411
LOC652605	0.009140696	6.096797042	0.72167408
CXADR	-0.023368589	8.959885309	0.721689884
IFT52	0.00906571	6.552139355	0.721758757
UBE2U	0.008463241	6.247191185	0.721794856
LOC441459	0.0087762	6.018772128	0.721897527
IGFL4	0.010575742	6.013334718	0.721949942
BGN	-0.032181841	12.86541915	0.722104962
LOC390956	0.008393115	6.170919629	0.722191535
LOC643338	-0.009051487	6.068662755	0.722217414
CHID1	-0.012084336	6.079477493	0.722276744
C13ORF30	0.021447506	6.267482226	0.72229774
LOC731742	0.013285029	6.19987704	0.722303252
SLC6A7	0.0110332	6.135579435	0.722396771
FOXRED1	0.018388828	8.809708065	0.722482318
EFCAB4A	-0.019202614	8.312632961	0.722517308
LOC651794	-0.00904636	6.024566579	0.722530966
LOC644113	-0.013742755	6.26406464	0.722577973
C4ORF43	0.012430462	6.166646147	0.722638332
EFNB1	0.013492985	7.32356514	0.722782562
CGREF1	0.008910886	6.041952913	0.722796561
UBE2MP1	0.00881557	6.139558233	0.722872588
LOC650879	-0.010292897	5.974963068	0.722901091
LOC442582	0.018000612	6.055962881	0.722943761
TMEM82	-0.008581466	5.99245369	0.722969625
LOC647451	-0.0084974	6.21754691	0.723046768
FGF21	0.006705174	6.094573532	0.723108308
LOC651404	0.008258331	6.060453712	0.723161275
LOC645681	0.008647469	6.040620826	0.72324872
LOC650296	0.008561777	6.391843176	0.723277069
LOC650295	-0.007594054	5.931596929	0.723309976
MGC88374	-0.009127465	6.028284058	0.723396249
TMEM25	-0.015619748	6.425730115	0.723785912
FLJ40235	0.009243363	5.943505492	0.723835527
JOSD3	0.015313696	6.35924699	0.723980822
LOC731396	-0.008649207	6.311264519	0.724023992
MAML3	0.012282535	5.929821403	0.724223803

FAM86B1	-0.008789882	6.53131559	0.724241931
C20ORF12	-0.010480423	6.362012273	0.724246679
C20ORF95	-0.008892646	6.202998714	0.724299874
LOC283914	0.008228477	6.117056655	0.724354711
LOC642656	0.008400376	6.05005731	0.724398218
TLR3	0.007426472	6.182451077	0.724420721
NINJ1	0.019645127	9.618840649	0.724436987
ITGB6	-0.009005313	5.962988115	0.724461295
CDSN	-0.011090627	6.053471242	0.724508527
TMEM141	0.018235481	8.286409904	0.724599189
CKMT1B	0.010834287	5.959922489	0.724798712
LOC647551	0.009506226	6.016126172	0.724809882
LOC648691	0.009098013	6.181724792	0.724839515
TRIM35	-0.006307864	6.262610435	0.724840153
C11ORF53	0.010295914	6.309907065	0.724841188
LOC653487	-0.010823034	5.931593398	0.724845815
C19ORF52	0.012224317	8.08235855	0.724852418
LOC652102	0.00846934	5.956706654	0.724857853
DEFB108B	0.007635561	6.121477015	0.724927263
BLK	-0.010475263	6.037419539	0.724928782
DSCR1L1	0.016780005	6.324628274	0.724991454
LAYN	-0.013799719	6.801995369	0.725011577
C1ORF87	0.006068128	6.07225568	0.725140629
LOC642946	0.009258168	6.298172052	0.725144248
CELSR1	0.011016161	6.770740247	0.725286412
CCDC110	0.01061393	6.168089976	0.725301365
FLJ32810	0.006529027	6.194320955	0.725364903
LOC647034	0.009014826	6.010282933	0.725546349
LOC642252	-0.004525033	6.085935909	0.725636264
LOC641835	-0.009476611	5.997174162	0.725747906
NXF2	-0.006831549	6.078743097	0.725786305
S100B	-0.008505965	5.928689418	0.725874242
GJA5	0.011026593	6.451705902	0.726006471
LOC645037	0.021430114	5.978712389	0.726058856
SNX19	0.015488928	7.836630575	0.72638629
NPHS1	-0.009423681	6.038792444	0.726428108
C21ORF37	-0.009007598	6.306415008	0.726440643
C8ORF51	-0.00831902	6.358623868	0.726454551
BTNL9	0.024171153	7.49653066	0.726514046
LOC652045	-0.009804702	6.244027383	0.726612914
DAPK3	0.034621562	7.924332786	0.726630016
B4GALNT2	-0.008480527	6.003701164	0.72667686
BVES	-0.02053032	8.461654736	0.726840455
LOC643693	-0.008694959	6.062602119	0.726866737
LOC400581	0.009114187	6.023656145	0.727015776
HTATIP	0.01241216	6.65535687	0.727061281
LOC651997	-0.009739685	6.018948941	0.727069775
SYNPO2	-0.012619677	6.803433441	0.727071558
LOC645450	0.008314952	5.898545246	0.727205968
DDC	0.00797988	6.072183026	0.727257633
GSDMC	-0.031040315	6.88335044	0.727331504

LOC653424	-0.008311925	6.016274156	0.727354402
RASAL1	-0.007838083	6.223497512	0.727418544
LOC646981	-0.010477448	6.170127842	0.727438948
CCL5	0.031720942	7.331388441	0.72744357
ZNF552	0.008414184	6.172511045	0.727518156
LOC728320	0.01132471	5.97226273	0.727669215
LOC653934	-0.010036881	6.095350215	0.727676219
TXNDC15	-0.015599933	7.656232547	0.727727957
LOC647193	-0.008501096	5.944797307	0.727812314
LOC647042	0.008751681	6.174071469	0.727824533
LOC651506	0.011403158	6.069265842	0.727845487
CNOT2	0.01521136	8.588962521	0.727880882
PNLDC1	0.011871408	5.928110054	0.727933513
SYCN	-0.008432807	6.12306973	0.727988867
LOC442711	0.011152202	6.373881053	0.72807385
PCTK1	-0.010460769	6.257362317	0.728144636
KCTD1	0.009708127	6.150460292	0.72819068
LOC646026	0.010095855	6.277884631	0.728250779
ANKRD38	-0.010387769	6.502117364	0.728282357
C8ORF80	-0.009251707	6.212265584	0.728362776
SLURP1	-0.009897381	6.010525456	0.728409371
LOC647535	0.009449938	6.365526124	0.72846775
SPINLW1	0.004765905	6.198618407	0.728544509
SIRPD	-0.009416158	6.197413832	0.728726236
PSMB4	0.014655984	10.72915062	0.728769771
LOC341651	-0.008014942	6.002091341	0.728806141
KRTAP13-2	0.008348446	6.012082885	0.728931796
ZAK	-0.022975529	11.42968663	0.728954559
LOC643206	0.010814604	6.520543802	0.728966035
STRC	0.007004072	6.292395078	0.729037633
ELOF1	-0.025737437	7.147586701	0.729038496
SNX30	-0.010481008	6.923001343	0.729140366
STK32C	0.01560344	6.042737245	0.729197684
LOC644048	-0.007633982	6.091434295	0.729233187
LOC653589	0.00845865	5.97402513	0.729315411
OR1G1	-0.009411529	6.044712637	0.729331936
LOC390874	-0.009647571	5.980300674	0.729424251
LOC650799	0.008673698	6.071245995	0.729561938
LOC643615	0.007845775	6.131687016	0.729595801
C2ORF13	0.008324242	6.001786547	0.729629873
LOC647035	0.00886929	6.074161974	0.72976362
FLJ37228	0.009529592	6.256611507	0.729773584
LOC653559	-0.008188979	6.054144687	0.729830965
THAP5	0.005473558	6.017090652	0.729891843
TAF9B	-0.008994607	5.900003233	0.73013969
PANK4	0.014254303	7.601088778	0.73019282
ERBB4	0.010521526	6.534028834	0.730277239
C9ORF90	0.011947923	7.442119219	0.730286136
LOC442147	0.009689061	5.928581079	0.730322592
LOC646486	-0.009370055	5.859501924	0.730373989
LOC400942	0.007246978	6.029613655	0.730416722

OR7E91P	0.006449363	6.01097117	0.73042593
LOC644207	0.007997871	6.094075843	0.730550506
LOC441007	-0.008515542	6.158300568	0.730592361
LOC646360	0.007815956	6.239286449	0.730682077
GPR143	0.011841819	6.155439042	0.730791691
TRAT1	0.006104673	6.101848353	0.730814463
PRO1853	-0.03431334	7.439952826	0.730829063
LOC646332	0.013435019	6.302496366	0.730890237
MINA	0.006904174	6.363774569	0.730918525
LOC650938	-0.009161555	6.203429342	0.731044173
LOC643813	-0.008579008	6.069214346	0.731051387
LOC641819	-0.009602465	6.193743439	0.731106514
LOC650518	-0.012716167	11.21784942	0.731164957
HHIP	-0.005846259	6.036700208	0.731214628
LMBR1L	-0.014469465	6.29046909	0.731215899
LOC652721	-0.009097838	6.320016689	0.731318379
IFNA14	0.007321812	6.177388171	0.731522428
SH2D1B	0.009942823	6.01502477	0.731534134
KCTD21	-0.016987056	8.224314632	0.731594404
SLED1	0.008630312	6.312320711	0.731632095
LOC652803	0.00900125	5.947225644	0.73188505
LOC730455	0.014603404	7.704586728	0.731945273
LOC553158	0.012714259	5.900339544	0.732031383
LOC641995	0.008155995	5.99547803	0.732116314
RAB2A	-0.018886701	7.960522602	0.732253426
OR10G8	0.008782919	5.973337751	0.732280248
SNX8	0.013122338	7.271047027	0.732283395
COL3A1	0.048984452	10.3008275	0.732498634
LOC642756	0.008823281	6.341411393	0.732657059
THRAP3	0.012248295	6.877014533	0.732722626
TREML2	-0.009406941	6.242272324	0.732904451
LOC646383	0.008846125	6.142043604	0.732911738
LOC391429	-0.014725012	6.149759892	0.733047704
FBXL13	0.01162607	6.081405277	0.733171978
OR51B4	-0.00792183	6.052229105	0.733318605
CXORF41	0.008485417	6.032971854	0.73331991
LOC85391	0.011684177	6.105298441	0.733487532
SLC9A2	0.013663525	6.015755065	0.733496975
BIN2	-0.009343504	6.010886611	0.733882714
LOC643985	0.008298249	6.20751206	0.733884915
OR8B2	-0.008746631	6.015654642	0.733897537
LOC647322	-0.011441632	6.194139289	0.73397542
TIMM10	-0.042476065	9.600169527	0.734216468
TIGD4	0.008618738	5.925902584	0.734219285
GDF6	0.007522175	6.017303021	0.734301591
LRPAP1	-0.028064874	8.985398599	0.734419672
FAM46C	0.027860677	7.835534321	0.734443603
C1ORF222	0.007963857	6.191162614	0.734502231
LOC728070	-0.009149947	6.210364952	0.734680712
LOC649947	0.007914484	6.06809772	0.734757586
FGF4	-0.011031761	5.906351223	0.734759864

GAN	0.00723115	6.122339452	0.734834353
XAGE1	0.004164072	5.989937851	0.734869784
SPDYA	0.004526912	6.081829063	0.734994448
ZNF33A	-0.00919775	6.8463591	0.735022911
DAOA	-0.008598064	5.981657465	0.735111045
MGC39606	-0.008744297	5.993067868	0.735193767
OR6F1	0.008777067	6.086344344	0.735205965
LOC653483	-0.005823595	5.957844037	0.735246967
LOC644299	-0.007504607	5.967152371	0.735261973
OPRS1	-0.010491668	6.761705443	0.735274727
DKFZP434E1119	-0.009650689	5.894416411	0.735282962
TRPC5	-0.014953278	6.063193856	0.735412528
OR52E6	0.008358106	6.205590284	0.735464247
RAB40AL	-0.008578415	6.043693415	0.73551288
GRK7	-0.010107484	5.90470425	0.73555888
LOC645897	0.007563425	6.07424584	0.735721996
B3GNT3	0.014550191	6.016069488	0.735783223
MAPKBP1	-0.010683364	6.509493855	0.735812587
MBD3L2	0.008448859	6.070966858	0.735878108
LOC643556	-0.006292736	6.131461211	0.735902327
SLC35E4	0.009478229	6.240705086	0.735903673
R3HDML	-0.010146592	5.935525236	0.73596313
LOC642785	0.008405344	5.949482483	0.7360207
TRIP6	0.013013553	8.551457693	0.736201397
GRB7	-0.005309153	6.076130911	0.736203301
LOC644195	-0.009418848	6.063205256	0.736256964
LOC440981	-0.008425619	5.900383928	0.736275503
MUPCDH	0.006129346	6.029448822	0.736278598
LOC653270	-0.009976751	6.512766186	0.736343088
LOC649282	0.008106044	6.229968297	0.736461608
C10ORF96	-0.00832141	5.953366534	0.736522977
P4HA3	-0.008572936	6.143367779	0.736534998
LOC650611	-0.01060762	6.143870599	0.736598754
LOC400652	0.01156206	6.137916537	0.736646924
LOC729967	-0.009163711	6.016959039	0.736785629
LOC645932	-0.008394992	6.070806813	0.736897948
OR10H5	0.006558911	6.047927801	0.73697257
RNF126P1	-0.006261688	6.548123771	0.73717397
A4GNT	0.013461335	6.037934322	0.737230225
LOC401720	0.01198535	6.426405507	0.737240255
LOC647219	0.009283175	6.184649554	0.737286157
PRAMEF9	0.01078885	6.051602908	0.737293483
GFER	0.015642508	6.020776566	0.73739392
EFNA2	0.008305816	5.93962399	0.737477213
DOCK6	0.016326903	7.956819245	0.737548103
LOC649179	0.006745728	6.156585512	0.737815515
LIN28	-0.008625879	5.889987182	0.73781719
ESCO2	-0.00898896	5.959525249	0.737860834
GAL3ST1	-0.009833253	6.010039399	0.738106565
LOC152586	0.012393979	5.991783049	0.73810886
KIAA0467	-0.009085577	6.201736046	0.738204787

LOC647805	0.010950406	6.258356807	0.738243646
FKBP6	-0.008329336	6.045057044	0.738317561
LOC647311	-0.010760188	6.056408439	0.738384543
UGT3A1	-0.008215972	6.093918259	0.738401434
C9ORF68	-0.0063741	5.977516001	0.738440512
OR51B5	-0.006651242	6.001758253	0.738532943
HSD17B1	-0.011357192	6.333777352	0.738608525
MORN1	0.008124417	5.989017776	0.738611768
LOC643158	-0.007623835	6.007056903	0.738741109
NALP5	0.008436053	6.156031403	0.738764575
TRAF5	-0.006258854	6.220097865	0.738852496
LOC642110	-0.009927249	5.998261153	0.738919792
ZNF689	-0.014271146	8.093189722	0.739010715
LOC441054	0.009183382	6.108730466	0.73910207
OR6Y1	-0.007608805	6.013186203	0.739196136
CA11	0.011920025	6.593513329	0.739295927
PCSK4	0.008791515	6.207237785	0.739332207
MIXL1	-0.008657432	5.905719714	0.73944395
FLJ40330	0.006417185	6.168592901	0.739608486
TTTY1	-0.011001312	6.074165793	0.739701928
PLAC1L	-0.007991144	6.042216838	0.739923231
KCNK15	-0.01197312	6.020597017	0.739941497
LOC651436	0.021602078	12.46548248	0.739957115
HTRA4	0.007955353	6.176036895	0.73996622
MADD	-0.010744968	7.09479984	0.740033788
LOC645093	-0.007869739	5.917762602	0.740038101
LOC90379	0.00985507	6.929231225	0.740042855
DBH	-0.009182592	6.061212178	0.740049138
LOC643763	0.007226158	5.967489746	0.74005228
LOC652668	-0.009089317	6.181859734	0.74006912
LOC642662	-0.008025856	6.102516205	0.740088463
MGC13168	0.008962962	6.456663497	0.740106819
CST11	-0.005769748	5.97187486	0.740134642
ADAMTS12	-0.008431282	5.987314446	0.740226059
LIPL3	0.010992324	5.988965856	0.740245963
LOC646664	0.007668071	6.025020514	0.74027828
LOC642118	0.008502067	5.988748406	0.740443348
CPLX4	-0.007986363	6.013396783	0.740497579
WNT6	-0.01117444	6.066842168	0.74053364
GALNTL5	0.009488442	5.939569093	0.740636703
LOC643327	0.00685095	5.982207007	0.740692354
LOC645236	-0.014771266	6.64804963	0.740775085
FANCE	0.014471737	7.185145461	0.740827873
PPAP2A	0.019068369	9.065418377	0.740844871
NUCB2	0.019023738	9.007345473	0.740868145
TIPIN	-0.012345	6.231101351	0.741048086
LOC652786	-0.0078459	6.069089288	0.741099622
C1ORF43	-0.02354575	8.888891249	0.741117187
ZNF528	-0.006193183	6.143118877	0.741117351
TPP1	0.015364568	9.386308764	0.741180243
GNB4	-0.010689888	6.470607787	0.741488028

SLC22A14	-0.009501694	6.075697232	0.741880007
LOC642566	0.008262342	6.053147327	0.741883063
GALNT5	0.007617159	5.886944413	0.741891375
CREG2	0.008239257	6.007074885	0.741932789
LOC649864	-0.009250669	6.230232451	0.742171761
LOC649136	-0.007379783	6.168032203	0.742222917
LOC402634	-0.008330558	5.877966839	0.742274662
PINK1	0.019898632	9.706340561	0.742307648
IRGQ	-0.008088115	5.97794524	0.742339326
GLB1L2	0.007990343	6.060182692	0.742491456
LOC643038	0.007439753	5.997482093	0.742623213
USP45	0.012505854	6.325490191	0.742659685
DNAH7	0.009355618	6.23206738	0.742697991
CDON	-0.005208313	6.047661915	0.742791294
LOC389832	0.00997761	6.126581314	0.742800631
LOC642345	-0.007389938	6.092034655	0.742865085
OR8B12	-0.009082553	6.163250427	0.742966734
OR1D4	-0.005290509	6.058016859	0.743058797
C15ORF26	0.008709656	6.078471183	0.743129109
GC	0.008166643	6.035363309	0.743165785
LOC645416	0.008686721	6.232156111	0.743193298
LOC652190	0.009594096	6.056943814	0.743216056
RPS11	-0.018126267	13.06982885	0.743225416
KIAA0748	-0.007760264	6.087748748	0.743264947
C1ORF157	0.008335151	6.096222585	0.743314001
FLJ45966	0.006955223	6.447713442	0.743504787
ADAM3A	0.005147429	5.943102095	0.743543546
LOC650076	-0.008420072	6.077529026	0.743633533
LOC642595	-0.008264035	6.130072616	0.7436703
LOC644883	0.007964727	5.995097812	0.743696076
LOC51152	-0.00786926	6.132223773	0.743783691
TAS2R50	-0.008973932	5.907997018	0.743902131
RSPO1	0.008493521	5.89230364	0.743997812
PLA2G12A	-0.01119115	6.46919722	0.744082644
LOC647025	0.007385697	6.154977071	0.744188207
EIF5AL1	-0.008540953	6.345734637	0.744205119
USP34	-0.014486606	7.892321041	0.744326321
HOXD13	0.011030954	7.123900925	0.744375995
LOC651695	0.012782172	6.070000465	0.744441493
LOC649977	0.008355498	6.018099211	0.744544202
LRRN1	-0.007752578	6.129042961	0.744660745
LOC651311	-0.009297588	6.145806576	0.744710357
LOC652141	0.007932406	5.935756613	0.74471491
LOC646372	-0.007572317	6.179238597	0.744762804
ZSCAN10	-0.008799021	5.928234122	0.744795191
LOC440508	-0.011467216	6.242880812	0.744869009
SLC22A7	-0.005154209	6.136914165	0.744887926
FRS2	0.005272322	6.07035922	0.745083144
LOC642804	0.010034483	6.098031926	0.745110213
CD22	-0.007830822	6.065988704	0.745120132
ZNF329	0.010772128	7.928832428	0.745155614

COBLL1	-0.01804558	9.055824695	0.745175451
ATG16L1	-0.010865232	8.187888541	0.745200491
SNORD42A	-0.008845904	5.810977491	0.745366015
LOC727934	0.007910264	6.28110188	0.745392973
LYPD3	0.009473833	6.204895267	0.745397784
LOC440338	0.008923898	5.925996661	0.745465689
HOXA6	-0.009673568	6.337863482	0.745596121
FLJ38973	0.012380861	6.333588486	0.745601394
LOC648302	-0.007248978	6.318853898	0.745610612
SLC12A4	-0.017175667	8.044420679	0.745637159
LOC643647	0.008315067	6.011289731	0.745729977
PTK7	-0.008499841	6.157660674	0.745744287
CAPZA2	0.015596147	11.48711616	0.745890858
CC2D1A	0.009905305	6.583304094	0.745892319
GLYCAM1	-0.004586468	6.051825934	0.745932822
SLC35B2	0.014111629	8.604627487	0.746007022
LOC285484	-0.008143518	6.081531097	0.746081494
LOC652113	-0.008018929	6.043412992	0.74611466
CD177	0.009383546	6.060260288	0.746133296
MGC71805	0.008999878	6.280826656	0.746169099
SPATA21	0.008352421	5.973790818	0.746261505
LOC440386	-0.007431937	6.04011278	0.74629298
LOC647297	0.007351892	6.105139483	0.746488604
C14ORF144	0.005946754	6.123646789	0.746574671
SIX6	0.00827308	5.936487521	0.746622978
OGFOD1	-0.010622369	8.150403279	0.746672622
LOC646584	0.007740856	5.998658866	0.746768516
OR8S1	-0.0082458	5.949687836	0.747059748
PRAMEF8	0.0086763	6.196223733	0.747084891
C6ORF123	-0.008282534	5.934049764	0.747087178
IGF2BP1	0.010272451	6.113082303	0.747114381
FAM69A	-0.009353698	6.74189901	0.74747437
TINAG	0.009356654	6.146698411	0.74754278
P2RX1	0.012836103	6.378791232	0.747547532
FRMPD2	0.003223344	6.022147505	0.747549187
SSTR3	0.008774189	6.107186186	0.747705343
FOXF2	0.008388615	6.149138632	0.747748766
LPA	-0.003686999	6.124217151	0.747791048
LOC731884	0.008561829	6.132036632	0.747828296
LOC642228	0.007661156	6.049699583	0.747950053
LOC646617	0.008308101	6.075479116	0.747953991
SF3B2	-0.017652645	9.008708055	0.748016928
PPAPDC1A	0.011432184	5.947047467	0.748042479
LOC645006	0.00859641	5.957750536	0.748138404
LOC402282	-0.007293243	6.072297525	0.748225045
KIAA0754	-0.007526474	6.032153558	0.748233803
NOX3	0.008519629	6.021233912	0.748372772
PYGM	-0.024013717	7.327364959	0.748451971
MRPS18B	0.016059755	10.03942044	0.748542027
ABCA8	0.026864029	8.944649082	0.748570753
LOC729085	0.009219149	5.96976559	0.748634567

REM2	-0.007887749	6.008240926	0.748766765
MYT1	0.008292148	5.97449656	0.748783702
LOC652848	-0.008415667	6.077314556	0.749004115
LOC644952	-0.009020599	6.014060843	0.749069219
LOC648089	-0.006070749	6.110185744	0.749093831
MTMR7	0.008613057	6.057411178	0.749137124
LOC647987	0.008240044	6.124209419	0.749169012
ATP2A1	-0.00454673	6.059628988	0.749204555
LOC134121	0.007866593	6.027937088	0.749349071
LOC731017	0.0076667	6.064289655	0.749418737
LOC441782	0.007725603	6.25259616	0.749445345
LOC648609	0.007227601	6.12424039	0.749481858
RNPC2	0.009820501	6.246481469	0.749511819
LOC646079	0.008190578	5.974294932	0.749578801
SYT1	0.007510898	6.069500455	0.749605216
OSBPL5	-0.010995463	7.801415682	0.74975302
TR2IT1	-0.005715465	6.016392893	0.749859752
INTS6	0.013259243	7.872871607	0.749986647
LOC653053	0.007246272	6.042148332	0.750043404
LOC651898	0.007568957	6.064588834	0.750049935
C21ORF84	0.008152117	6.006454445	0.750147023
BCL2L13	-0.018855298	10.87836673	0.750186737
ENPP1	-0.009048073	6.672455213	0.750262308
LOC283867	-0.008501877	6.179082952	0.750328429
STRA8	-0.007736782	5.933295125	0.750353121
KRTAP6-1	-0.007858313	5.917086514	0.750357568
OR2T10	0.008415782	5.922164601	0.750444213
LOC339760	-0.007039758	6.258485344	0.750452534
RPS4Y1	0.1435116	8.824464226	0.750714279
CLEC11A	-0.020163952	7.022023199	0.750739351
GTF2H2	-0.009211993	6.224427053	0.750803548
OR4C45	-0.008638979	6.210949631	0.750804106
LOC642536	0.007826903	5.971588269	0.750907169
OR1J1	-0.006210818	6.259784078	0.750922456
ALS2CR11	0.010493024	6.188540693	0.750947962
LOC651951	0.007942438	6.063957097	0.751063437
C10ORF68	0.007342089	5.979847637	0.751113434
LOC340515	-0.007386029	6.143704072	0.751199322
LOC644396	0.007071804	6.061774217	0.751343848
LOC338756	0.007016671	6.144673665	0.751367256
AREG	-0.007881762	6.020799564	0.75146428
CD200R1	-0.006682895	6.098592004	0.751496636
GIMAP1	0.021168197	7.204812055	0.751570546
LOC646160	-0.007442158	6.01079584	0.751795181
SPTBN2	-0.008072596	6.317878681	0.751796793
EFCAB1	0.007805034	6.056959202	0.751831828
LOC643717	0.007189232	6.013409397	0.751891553
LOC644479	0.008528219	5.983845052	0.751891721
GABRR3	-0.007436282	5.977575788	0.751977972
LOC646590	-0.007664397	5.954971743	0.752033919
LOC652009	-0.008878984	5.983842534	0.75207815

GPS1	-0.010130103	8.832771592	0.752164136
PCDH21	0.012754644	6.058937192	0.752200358
KIF6	0.009704712	6.785575677	0.752241265
CTXN1	-0.017200701	6.648320537	0.752306737
LOC731714	0.007001493	6.02570774	0.752330575
LOC642062	-0.008705419	6.23362942	0.752400924
LOC731682	0.018138099	6.128136011	0.752414326
LOC647585	-0.007524644	6.096106777	0.752703364
STARD6	0.007261357	5.930156177	0.752805035
CLDN19	-0.008350213	6.289542869	0.752823584
ANKRD19	-0.007650985	6.03311903	0.752869986
RNU12	-0.008887487	6.118974799	0.752875199
KIAA1586	0.008524521	6.273682057	0.753001743
SSBP1	0.014746773	10.63250691	0.753077959
MUM1	0.014230122	8.833640765	0.753093885
ZNF292	0.009303722	6.15592564	0.753160417
POTE14	-0.008569072	5.912281118	0.753267642
OR51D1	0.00973417	6.264060345	0.753302666
LOC402679	-0.007844704	6.037709594	0.753311348
IL1F5	-0.006190178	5.974219765	0.753406792
IL12RB2	-0.008162691	6.141329153	0.753452654
LOC643825	0.007507892	6.055673893	0.753559161
MGC48628	0.008445983	6.10251696	0.753563413
OPN4	-0.005433424	6.013035832	0.753629778
LOC646550	-0.007495539	5.97707845	0.753644583
CSGALNACT1	0.018445446	7.331920179	0.75364659
OR8B8	-0.008405206	5.851858616	0.75365523
C21ORF125	-0.00851371	6.074045909	0.753760266
SELV	-0.008080149	6.065424121	0.753818393
RG9MTD2	-0.008833758	6.569966588	0.753833553
LOC388743	0.007228231	6.121830245	0.753834849
LOC641820	-0.008168268	6.247000468	0.753846758
FLJ32310	-0.008603396	6.073316065	0.754039794
SERPINA2	0.005665059	5.944205882	0.754103446
LOC643040	0.008084009	6.072312979	0.754110298
CD1D	0.012258158	6.436908834	0.754157644
WASF1	0.00567789	6.063386183	0.754170354
MRPS18A	0.018193681	8.387536278	0.754241447
LOC441920	-0.009387354	6.160793293	0.754262401
NLRP6	-0.008317211	6.116966663	0.754384339
GRIP1	0.00559724	5.979550251	0.754410293
LOC642263	0.00723129	6.032713727	0.754413619
C8ORFK29	0.006884253	6.063141256	0.754539814
LOC387686	-0.008272726	6.279319315	0.754608897
CDCA3	0.009291831	6.061704942	0.754655082
NBPF1	-0.004736395	6.220393857	0.754839158
LOC126147	-0.008246766	5.942985482	0.754883536
LOC732343	-0.006942091	5.969589802	0.754884239
CD96	-0.006927887	6.153061778	0.754935871
H2AFV	-0.009129232	6.791742389	0.754983302
C12ORF28	-0.009588749	6.24207707	0.755020395

LOC642570	0.010836334	6.286701611	0.755040797
LOC642561	0.007325753	6.040485182	0.755084788
SPRR2G	-0.008328254	6.124367295	0.755118485
CACNG2	-0.008273372	6.014957154	0.75533144
ANGPTL6	-0.007110213	6.039729638	0.755359701
FKBP10	-0.011419374	6.190416221	0.755474244
IL1RAPL1	-0.025554154	7.259012395	0.755553793
DEFB103A	0.007771382	6.060611614	0.755619798
LOC732381	0.009035297	6.112815387	0.75577285
FCN2	-0.006454596	5.984807507	0.755790068
LOC647965	0.008753613	6.157590162	0.755838247
NUP98	-0.006759248	6.618280936	0.755869234
DLG7	0.009258808	6.130716579	0.755905744
LOC439936	-0.008735524	5.850125506	0.755962872
GPX7	0.017055481	7.781422266	0.756058564
WNT4	-0.008643446	5.998161071	0.756145642
MGC26647	0.008563393	5.98302789	0.756193361
UFM1	0.015396884	8.129740484	0.756219628
WDR6	0.015696276	9.7764317	0.756387253
CIITA	-0.012898733	6.021153009	0.756400627
LOC729249	-0.008317283	5.947888496	0.756408673
LOC646895	-0.005563469	6.078968298	0.756459934
FLJ25169	0.008268969	6.233426934	0.756523539
LOC649293	0.011820176	6.03193124	0.756601956
TSPAN10	0.008803569	6.124503706	0.756684
USO1	0.013166419	10.4754768	0.756770599
RAB19	0.008112145	5.947469941	0.756851395
CSNK1A1L	0.005975777	6.096588915	0.756981243
MSL3L2	-0.008328847	5.971022946	0.757067772
CSF2RB	0.007478907	6.031482335	0.757144717
PHB2	0.011177067	9.641523087	0.757219713
ELAVL2	-0.00668302	6.042854941	0.757300703
OR2T3	-0.008296596	5.926850052	0.757321976
LOC285636	-0.015284025	8.765418159	0.757429031
LOC653882	0.007937819	5.951063976	0.757441784
LOC642018	0.009088887	6.073611162	0.757549702
TRAF3	0.009204614	6.39921729	0.757591864
C6ORF25	-0.004527289	6.197396405	0.757617805
TRIM14	0.004392349	6.082759881	0.757677992
HOXB8	0.008575106	5.980339582	0.757750983
CARS2	-0.014933504	7.604957358	0.757803437
C12ORF42	0.007859307	5.850149801	0.757888686
LOC641931	0.009952726	6.026468157	0.757981834
OR8B4	-0.008322566	6.027509471	0.758018744
LOC158345	0.022949203	10.59962184	0.758343578
ATCAY	0.008704303	6.075479548	0.758352979
LOC391241	-0.010500058	6.019629411	0.75836022
C10ORF120	-0.007524357	5.931048676	0.75847651
LOC643771	-0.009060567	5.956518782	0.758545011
HIST1H2BB	0.007420958	5.995640511	0.758752218
PAX7	-0.015150563	7.921062912	0.758757899

SPRED3	-0.004850312	6.100267137	0.759034234
PSMD1	0.014961334	9.880474923	0.759145772
LOC652460	-0.008072608	6.038190929	0.759214629
SIAE	-0.009715721	6.000572048	0.759244069
GALR3	-0.005478304	6.019796415	0.759400544
LOC651919	-0.011779337	6.120925161	0.759469323
LOC644019	-0.003514687	6.136917661	0.75955622
LOC732138	0.010795968	6.013004614	0.759614745
USP20	0.007086828	6.268270127	0.759615969
CLDN6	0.008624758	6.0453148	0.759650636
C16ORF69	0.010247209	6.334617053	0.759732689
LOC647645	0.010419067	6.190929664	0.759821483
LOC650830	-0.007849367	6.225682755	0.759913173
LOC388559	-0.006525452	6.073290458	0.760058479
CEBPZ	-0.025378509	10.04562279	0.760101575
EMP2	-0.015896445	6.163043141	0.760148851
MTF2	0.01751881	7.085806214	0.760219352
RHOH	0.010136063	5.956107853	0.760227778
DNAJB4	0.030847086	7.81932014	0.760323897
LOC644136	-0.007656968	5.984986915	0.760426567
PCDHB9	-0.019934659	7.192461865	0.760452085
APOBEC3B	-0.009866136	6.047722942	0.760471918
CCDC85B	-0.017885779	6.330773641	0.76058226
C1ORF75	0.006445725	6.014834324	0.760689048
TMEM76	-0.007702504	5.95322982	0.76074787
DAO	0.008201004	6.070286887	0.760802204
BTC	0.007506525	6.135773649	0.760826236
LOC653701	0.01398087	6.069168915	0.76084642
LOC652589	-0.007951915	6.325453578	0.761067341
POU2F1	0.0141525	6.461464403	0.761224313
GAGE4	0.015598124	6.157936256	0.761227693
CRYBB3	-0.007311744	5.962698345	0.761295498
LOC643116	0.007574322	5.962535846	0.7613846
PRKDC	0.011192938	7.439155342	0.761447093
IL31	0.009582769	5.79010906	0.761476979
LOC653512	0.011607118	5.934733217	0.761798524
RNF187	0.009767392	6.640144374	0.761805686
HOMER2	0.016723797	8.753014124	0.761845241
LOC653971	-0.007475794	5.966549394	0.761867998
KIF4A	0.007436877	5.965796317	0.761870918
LOC650776	0.009854343	5.824989668	0.761912974
LOC650881	0.006849244	6.164131793	0.761913793
ROPN1B	-0.009347538	6.414010118	0.762028563
PTCHD2	0.008331742	6.068150006	0.762042255
ELOVL3	-0.009057961	6.01292907	0.762076594
KIF3A	-0.008114329	6.041448629	0.762099713
VGLL1	0.007814544	6.20227529	0.762141635
SETD5	-0.008914427	6.126248638	0.762157024
LOC649963	-0.007611444	6.033870628	0.762217501
CDH4	0.008169419	6.080301416	0.762223086
LOC649804	0.007189764	6.096810837	0.762392006

LOC149351	0.007956211	6.337168428	0.762451296
ACCN4	0.004950658	6.051126251	0.762567016
FLJ13614	0.008405159	6.167693498	0.762581934
NRXN3	0.004764144	6.052800554	0.762835755
GLI2	-0.008199543	6.239661889	0.76284893
CIB4	-0.007117397	5.908593922	0.762870803
LOC649905	0.008460838	5.965477062	0.762889099
ARHGAP15	-0.015236443	7.368087495	0.762908176
LOC202134	0.00954035	6.186850543	0.762947385
LOC646778	0.008399865	6.152637871	0.76298503
SERPINA12	-0.007474764	6.020925756	0.763034757
CRLS1	0.018484342	11.12064011	0.763125897
BCL11B	0.005934999	6.068550805	0.763157911
LOC642207	0.008305124	6.033033213	0.763247397
LOC644683	-0.00611332	6.072259054	0.763584076
SILV	0.007990911	6.124737593	0.763587901
MFAP5	-0.031339613	9.770758607	0.763677738
FAF1	-0.009427315	8.631976971	0.763769994
TBX10	-0.009109981	6.358218943	0.763812498
FSCN2	-0.006034866	5.976842205	0.7638664
RNF139	0.0112644	5.985694594	0.763932749
ANKRD5	0.00470453	5.957571336	0.764058372
LOC653729	-0.00899844	5.902858929	0.764073137
ABP1	0.009188119	6.169796248	0.764094544
USH3A	0.006870201	6.040861982	0.764155534
NCK1	0.014560563	8.588573723	0.764251707
LSM11	-0.00784862	6.135884687	0.764272178
MAGEA5	0.007625391	6.199430447	0.764275337
SNAP25	0.006083238	6.382400603	0.764296925
ANLN	-0.008654325	6.268082815	0.764314869
HIST1H3I	0.007763563	6.251223817	0.764329556
FLJ27255	-0.008447585	5.980538983	0.764480632
ZNF618	0.006759504	6.692974663	0.764572887
IL26	0.007128921	6.03492996	0.764677094
MC2R	0.008223334	5.885259955	0.764761078
LOC653606	-0.007636412	6.083291766	0.764812593
FLJ44790	0.010921289	6.106450871	0.764828698
CLEC4D	-0.012352839	5.987280877	0.764859828
KEL	0.011482652	6.075440591	0.764904288
LOC392221	0.008743898	6.164024253	0.764988685
OR14C36	-0.006674125	6.022735702	0.765062881
KIAA0284	0.008113536	6.172973896	0.765173615
CSTF2	0.011003928	7.553566806	0.765251258
LOC652298	0.007644875	5.974054853	0.765290326
C6ORF66	-0.015932995	8.629315758	0.765308353
RAB39	0.008266855	6.022561628	0.765322244
EMR4	0.004287057	6.043997714	0.765564849
CATSPER4	0.008399827	6.022153099	0.765598782
LOC646373	-0.006948066	5.981787332	0.765606886
OR5P2	-0.005226459	5.986246169	0.765608629
LOC649495	-0.007315262	5.941669744	0.765651014

OR52N4	-0.009153461	6.01440227	0.765824028
KRTAP2-1	0.005513196	5.956091687	0.76586234
FAM55C	0.018928924	7.084154159	0.76586558
LOC643801	-0.006846697	6.049509689	0.765887309
TMEM99	-0.009452306	7.767394183	0.765917649
PCDHGB1	0.008215659	6.250081946	0.766077864
NDUFB11	-0.014288729	12.10737942	0.766086232
LOC150759	-0.009576498	6.146458478	0.766089234
C9ORF82	0.010007327	6.916859509	0.766136709
IL21	-0.007882393	6.035321711	0.766217287
MMP8	-0.006929262	5.911184428	0.766333659
LOC650781	-0.007312653	6.050876466	0.766410781
LOC644024	0.007276764	6.130286708	0.766556912
LOC652044	-0.013084732	6.08482252	0.766579052
LOC647650	0.011905644	6.380779763	0.766642124
PXK	0.009659002	6.536548527	0.766645241
HIST2H3C	0.00886446	5.954632676	0.766688101
REN	-0.00859995	6.202756019	0.766863766
LOC646632	0.007475819	6.328861119	0.766964094
LOC645373	0.007750216	6.044805801	0.767061712
LOC646003	0.00712583	6.083058713	0.767120836
FBP2	0.00855114	6.056608452	0.767175204
FLJ11795	-0.007953652	6.065287892	0.767247089
7A5	0.006868959	5.975353038	0.767251015
KRT71	0.006959499	6.059013816	0.767345915
LOC650757	0.007463646	6.434760337	0.767346872
LOC642460	-0.007164539	5.949978867	0.767355618
MMP21	0.005178402	6.076816047	0.767474408
KIAA1239	-0.005740251	5.949264476	0.767518913
LOC648453	-0.006758012	6.029235897	0.767630284
MFSD4	0.004501857	5.989616342	0.767679665
CNO	0.018603151	7.707833993	0.767798603
LOC644819	0.008310602	5.868535204	0.767853749
ZNF497	0.00721849	6.091887054	0.76785617
ARR3	0.008912336	6.019460869	0.767876354
LOC728454	0.008481484	6.31667917	0.767915648
KIR2DL5A	0.004047796	5.990916139	0.767917579
EEPD1	0.013268925	6.9249622	0.767936185
TCEB3B	-0.007277476	6.000593597	0.768010492
RG9MTD1	-0.014213904	7.328391107	0.768083051
SLC15A2	-0.00950768	6.218577644	0.768159442
CYORF16	-0.009103908	6.090278218	0.768163829
FAM132A	0.0095047	6.240395975	0.768235411
LGSN	0.005320114	6.010939308	0.768534125
C1ORF158	-0.007861326	6.024715197	0.768632521
CCT6A	0.01617938	8.677873236	0.768651331
FRYL	-0.005926412	6.748444329	0.768950813
GSX1	0.011374721	5.951729327	0.769167103
LOC646055	0.007405122	5.948206342	0.769169555
LOC644111	0.009474162	6.091617021	0.769181565
LOC401677	-0.006773984	6.056319971	0.769245066

LOC152024	-0.007239006	6.083367101	0.769289054
CDCA7	0.008046643	6.352191198	0.769345142
SDCCAG3L	-0.008138126	6.248942873	0.769367832
COL28	0.007305017	6.074129249	0.769463663
SLC26A8	-0.004079424	6.126284418	0.769609658
FOXK1	-0.014472888	7.889547145	0.769705682
SLC35C2	-0.009012545	6.605855021	0.769757553
LOC649242	-0.007198872	6.311879514	0.769831834
LOC647008	0.008955414	5.889010602	0.769896525
LOC651040	-0.007372506	5.999771997	0.770000663
SLC25A33	-0.009085322	6.044339272	0.770110227
RFX4	0.003505548	6.085646079	0.77012446
MAPK8	0.005633115	6.101488361	0.770154183
MYLK3	0.019495193	8.014797674	0.770386702
CNOT1	0.012308385	8.258978806	0.770395482
DPY19L2P4	0.0069323	6.029205482	0.770488999
PTS	0.012938994	9.603859035	0.770510504
C20ORF191	0.013118932	7.750169834	0.770517841
LOC647713	0.006544766	6.085485204	0.770528825
TRIM73	-0.004861014	6.099824693	0.770899168
MPV17L	-0.009128504	6.410475031	0.770922696
OR8G2	-0.008353392	6.133645437	0.770947764
XAGE3	-0.005320877	6.06127623	0.770969737
PDCD6	-0.012261223	10.12429282	0.770982096
PCDH11X	-0.003495982	6.072393631	0.771186854
FLJ39080	-0.007003182	6.30499128	0.771256554
LOC651624	0.007473343	6.151859231	0.771260073
SH2D3A	0.007793017	6.053159894	0.77127242
RPS27A	0.012160197	13.36885685	0.77139452
SPATA4	0.009201618	6.29316454	0.771408872
EPB41L2	0.012587946	7.434420206	0.771481005
FAM90A11P	-0.006693914	6.029149099	0.771483034
OPTC	-0.007508271	6.13211928	0.771551393
CNDP1	0.006156611	6.08544166	0.771587755
GCN1L1	-0.018111098	8.682456338	0.771592562
TMSL8	-0.011866912	6.355072317	0.771595228
GPR173	-0.004163157	6.103829782	0.771662636
LOC648103	-0.010615406	6.211902266	0.771856168
GPR150	-0.00811725	6.084304462	0.772142496
LOC644976	-0.004874978	6.07102842	0.772242628
LOC648879	-0.006664275	6.150228625	0.772366706
DNAH17	0.008248233	6.000683339	0.772495386
C17ORF50	0.007185171	6.038715835	0.772506378
LRRC46	0.010850884	6.226171536	0.772575879
B3GNT7	0.008159293	5.981119181	0.772679364
LOC647435	0.007395995	6.033295126	0.772682263
KCNK5	-0.009928632	6.22992226	0.772894121
DALRD3	0.005348512	6.231258111	0.772924575
BLOC1S2	0.004732346	6.168976381	0.772927611
LOC645757	0.007613273	5.888873036	0.772947728
LOC649984	-0.007641346	6.025725618	0.773042094

LOC374768	-0.006337195	6.096156613	0.773049069
C12ORF8	0.00671187	5.994341642	0.773060963
LOC286528	0.008313297	6.171692464	0.773076628
LOC646357	0.008596348	6.00071333	0.773077385
ST6GALNAC5	0.008786944	6.34692283	0.773200011
LOC652880	-0.007682372	6.112848143	0.773213922
LOC653891	0.006270643	6.110647157	0.773236414
C4ORF31	-0.013540352	6.868432445	0.773325199
LOC653499	-0.007212851	6.067305266	0.773352475
LOC283767	-0.003926674	6.165998649	0.773369958
C14ORF50	0.010438319	6.097713374	0.773389778
TAAR6	-0.007047008	6.068355089	0.773399766
LOC390683	-0.010975546	5.994397607	0.773409949
LOC283412	-0.030939675	9.873215202	0.773522988
ARL11	0.007395596	5.951829036	0.773574362
LOC728929	-0.0063628	6.128888095	0.773700338
LOC641905	-0.007804632	5.887905671	0.773812242
LILRP2	-0.006503298	6.045706365	0.773876196
LOC649355	-0.007210163	6.223212499	0.773876875
C2ORF73	0.006710689	5.890463681	0.773973424
LOC649902	-0.006436941	5.992119054	0.774311587
PSG3	-0.004873302	5.948203639	0.774318054
TTLL13	0.009026105	6.070074755	0.774381093
LOC402100	0.005368395	6.009674094	0.774425376
PLAC1	0.007051028	6.095891423	0.774442319
LYPD6	0.007252625	6.173991671	0.774448996
SERPINC1	0.007527709	6.040875586	0.774452432
RPP30	-0.005535731	6.093639461	0.774593576
MAP2K6	-0.005151403	6.005723264	0.774622078
C9ORF152	0.007692595	6.063037061	0.774624837
RNF2	-0.00803953	6.162961394	0.774731893
CBWD2	-0.008052901	5.959426341	0.774810277
LOC653114	-0.006683311	5.878280243	0.774862218
TOP1P2	-0.017174566	7.003802683	0.774980349
LPO	0.007508253	6.005895931	0.775048487
LOC644072	0.006836436	6.044845618	0.775095593
LOC646861	-0.007729766	6.10411581	0.775225169
C1ORF61	-0.007875036	5.944928636	0.775260868
H2AFB2	-0.010535462	5.93606307	0.775511544
KIAA1345	0.005953475	6.104000128	0.775511564
LOC645225	0.00728758	6.090124102	0.775592464
SLC38A6	-0.011536526	6.623568449	0.775728455
C3ORF72	-0.006679076	6.235964447	0.775799302
LOC645434	0.008279951	6.386205545	0.775819175
TNPO3	0.0139094	8.051192572	0.775832902
IL17A	-0.00750062	5.970809858	0.775940678
LOC654053	0.011035982	6.207075742	0.775957448
RER1	-0.010657407	7.877421853	0.776001891
LOC338588	-0.008844037	6.209716194	0.776349634
LOC644228	-0.006964461	6.088012297	0.776369578
LOC653163	-0.007641268	6.294697261	0.776447381

DERL3	-0.006038092	6.129764574	0.776525246
LOC652327	-0.00637009	6.136799206	0.776607366
LOC643933	-0.00738728	6.092484922	0.776621835
LOC647649	-0.0071087	6.1493223	0.776868951
LOC651463	-0.008255055	6.041176695	0.776917009
TMEM196	0.006220236	6.047664327	0.776954226
STK11IP	-0.00964016	6.932615355	0.777057893
LOC642773	0.009953372	6.05469917	0.777091013
LOC646257	-0.007143794	6.001796111	0.777147603
LOC653198	-0.005915046	6.063156498	0.777172896
GRP	-0.005287166	6.026897866	0.777237515
LOC441239	-0.005638887	6.023905614	0.777261366
PPME1	-0.011552014	7.963093731	0.777277524
LOC646529	-0.006084	6.068000658	0.777407675
SNX9	-0.007819679	5.897946119	0.777552865
SLC32A1	-0.007099519	5.906081445	0.777629247
CSAG1	0.005490208	6.031891702	0.777697859
IREB2	0.009247888	8.068823719	0.777743666
CHCHD1	-0.010215889	7.675973702	0.777806656
RDHE2	-0.009484121	6.07603597	0.777873567
LOC647908	0.00681537	5.947375122	0.777881214
C17ORF47	0.007750523	6.203833641	0.777931043
RIMS2	-0.008146168	6.012933201	0.778095781
LOC391269	0.007850091	6.080595133	0.778106702
ADAMTS20	0.004318045	5.996441041	0.77811624
LOC441958	0.008794746	6.239011949	0.778164913
FOXA1	-0.006875549	5.997295747	0.778182052
LOC646445	-0.006262337	6.004371103	0.778398663
ALDH3B2	0.004483755	6.04108231	0.77853205
LOC648934	0.007337305	6.226659536	0.778542861
CRIPAK	-0.016148237	7.362499297	0.778556914
LOC646764	-0.007400432	6.089164268	0.778585483
TLE1	0.018855955	6.786808229	0.778621199
LOC643884	-0.006788448	6.094719452	0.778685304
GUCY1B2	-0.007915514	6.127134004	0.778741154
EIF1	-0.0154317	11.86439417	0.778899348
FIBP	-0.01012475	8.758661989	0.778937903
PRO0611	0.007015691	5.95961154	0.778989262
LOC646473	0.006890543	6.067831876	0.778989443
TOP2A	-0.01340251	6.142565952	0.778989695
CXYORF2	0.006848321	6.092937123	0.779049953
LOC642685	-0.007249159	5.987888664	0.779090315
PCDH15	-0.006308575	6.09023769	0.77911813
ACVR2B	-0.005373811	5.959651639	0.779131241
MAST2	-0.018923682	6.621981217	0.779430916
SYNGR3	0.007173925	6.032567443	0.77945812
ERCC6L	-0.005070146	5.938260693	0.779576574
CPB1	-0.011488964	6.030629287	0.779605857
FLJ44379	-0.009522851	6.27128059	0.779639718
LOXL2	-0.007124667	5.956546173	0.779690388
FAM26D	0.007068951	5.988201121	0.779725466

LOC402509	-0.008752582	6.325164687	0.779732796
LOC441052	-0.008145108	5.937726115	0.77976551
USP6	-0.013324547	6.235182131	0.779818851
PDXDC2	0.006421024	6.0302049	0.779907671
SEPT12	0.010332271	6.273621803	0.779911232
C20ORF186	-0.00677612	5.962177194	0.779946526
LOC645527	-0.006058326	6.024925947	0.779964758
LOC729317	-0.010780948	11.35480414	0.779981562
LOC651113	0.006411228	6.09671416	0.78012088
ACAN	-0.006779825	5.952898925	0.780134409
TROAP	-0.006874459	6.048840479	0.780155371
LOC648473	-0.008059358	5.926385363	0.780198728
ACSBG2	-0.006899331	6.074100612	0.780462309
ZIM2	0.006477634	5.979935664	0.780463884
LOC649557	-0.006624866	6.092452704	0.780495114
CTAGE6	0.005708518	6.200586671	0.780501576
LOC644954	-0.007227176	6.017189419	0.780605803
LOC440518	0.00707337	6.3427325	0.780804909
LOC651308	-0.008340826	6.05305316	0.780866922
PPFIBP2	-0.012396818	7.454254597	0.780904401
C1ORF14	0.006614054	5.973700269	0.781073981
SERPIND1	-0.006598821	6.014957909	0.781113766
EID2	-0.009967532	7.087225577	0.781167844
MLN	0.006246988	6.047854019	0.781273488
LOC651051	0.006450571	6.162992334	0.781616248
LCTL	-0.012114113	6.43197261	0.781678598
NHEDC1	0.005637915	6.06390676	0.781702207
CECR7	0.016589303	8.083163036	0.781734549
FAM83F	0.008992402	6.211974364	0.781752003
C15ORF2	-0.009614156	5.968742719	0.781771651
CD27	0.009106983	6.051875126	0.781815145
ZNF335	-0.01033595	6.158633561	0.78193142
DDX12	-0.002716053	6.102096237	0.781939827
LOC284861	-0.007580702	6.002424435	0.781948729
BTBD10	0.014694368	7.97082342	0.781998325
NDUFV2	0.014015192	12.03656155	0.782023535
LAPTM4A	-0.019566464	8.14567119	0.782095682
BMP8B	0.010485154	6.298132845	0.782144043
LOC254028	-0.006911292	6.013593456	0.782155318
C19ORF16	0.007998048	6.057852015	0.782266439
WNT7B	0.005304668	6.114880293	0.782323769
LOC641798	0.010005932	7.404637303	0.78232864
DLEU7	0.006926618	6.009416802	0.782513898
ONECUT1	0.005184638	6.065151388	0.782737607
ETHE1	-0.011347679	6.214753657	0.782751684
TAS1R3	-0.007262367	5.896666644	0.782864204
MED31	0.009341822	5.961568394	0.782910742
LOC652846	-0.007561832	5.876995127	0.783129635
JAKMIP1	0.009599752	5.915831208	0.783187804
LOC440491	0.006243374	6.122392402	0.783195232
IFNA1	-0.007833903	6.000729928	0.783241204

FAM78A	-0.008125012	6.379971385	0.78331012
MGC51338	0.007108266	6.032131789	0.783336332
DYNC1I2	0.010742213	9.687763528	0.783358552
LOC650924	0.006629101	6.092411821	0.783384264
ADAMTS14	-0.004390335	6.066377291	0.783452025
C9ORF84	-0.004423007	6.084431991	0.783500298
MGC26733	0.011542624	5.996050078	0.783649087
LOC90342	-0.011054251	6.058560433	0.783806086
ZNF468	0.005992443	6.265199383	0.78383709
LRFN2	0.006389263	5.924760034	0.783868051
NDUFB10	-0.015982195	12.54221666	0.783951959
METTL11A	-0.019230236	7.716768136	0.784055691
OR4F6	0.006923903	5.977057855	0.784108021
LOC644023	-0.006434322	6.0702448	0.784138331
UPP2	-0.007250073	5.98162229	0.784202672
NLGN4Y	0.008414249	6.03921463	0.784214327
LOC126075	0.006813035	6.012226438	0.784612363
C9ORF139	-0.007526855	5.926515865	0.784699839
LOC643982	0.0063086	6.006465018	0.784730932
LOC387939	0.006819722	6.029509153	0.78480583
LRRTM2	-0.00713013	6.213745628	0.784882055
LOC645947	-0.006750048	6.033274551	0.784885923
LOC139363	0.006890272	5.992126349	0.784928008
ETV3	-0.008018689	6.334767159	0.784994911
OTX1	-0.007942001	5.920791177	0.78504592
TPTE	0.004736692	5.978654652	0.785059688
IFNG	-0.007099178	5.984851237	0.785183443
OR5A2	0.008252494	5.866572698	0.785207336
SFRS12IP1	0.007247858	6.157922527	0.785321377
ENY2	-0.011794321	10.72159362	0.785411657
KIAA1462	0.006927265	6.319338856	0.78542506
KLF14	-0.007271117	6.040181706	0.785520354
KTN1	-0.024931394	9.549839151	0.785574048
LOC651361	-0.005796123	6.046452052	0.785610784
TACR3	0.006574998	6.281361476	0.785667314
FLJ44216	0.013339508	5.889470467	0.785676696
C14ORF105	-0.004906631	6.104617918	0.785725912
POMC	-0.015136833	6.757806702	0.78584575
SLC2A2	-0.006999739	6.058662625	0.785868445
C20ORF151	0.009486961	5.909244262	0.785887092
LOC652260	0.011488883	6.038783524	0.78589915
LOC649023	-0.009765905	6.150040139	0.786037416
LOC651465	0.007159476	6.102436969	0.786315874
C3ORF67	-0.00760128	6.226529779	0.786340924
LOC651732	0.006059468	6.000433869	0.786387793
GJD2	0.0064729	5.987353703	0.786539752
LOC649407	0.006213987	6.057983282	0.786567025
ANGEL1	0.0090047	7.417748031	0.786605641
LOC651808	0.006288088	5.969752447	0.786632229
LOC645476	-0.008702997	6.035955538	0.786701642
RPLP1	0.010515691	13.2375227	0.786736377

GCM1	0.008088479	6.297754143	0.786739674
LOC653366	-0.006588525	6.061002832	0.786742957
LOC203235	-0.007157574	5.96113344	0.786746567
GRIN3B	0.007318554	6.110156681	0.786867911
INPP5E	-0.015867418	7.304214211	0.786881403
HIGD1A	-0.012166678	11.68409152	0.787213842
KIAA1147	0.010769198	7.600579926	0.787260251
LOC649191	0.007174618	5.936566413	0.787261376
LOC648830	-0.006738468	5.947229067	0.787402994
ALG6	-0.010310373	7.152982969	0.787433372
CALML5	-0.009239066	6.087486167	0.787660224
MZF1	0.01098151	7.627610066	0.787769085
LOC646714	0.009283726	6.099485718	0.78784095
FBXL16	0.00667288	6.036881801	0.787931485
BTF3	-0.010917472	11.59187671	0.787969157
LOC650534	-0.006385239	6.009552057	0.787993168
GOLGA6	0.003801093	6.08939932	0.788077589
BTBD14B	-0.006778626	6.331329809	0.788086087
LOC728799	0.006191414	6.061901308	0.788144664
LOC650634	-0.006212168	6.129973926	0.788181825
LOC389958	0.007678069	6.089297857	0.788186557
FAM83G	0.007384896	5.958704578	0.788274429
LOC51233	0.007099387	6.066887659	0.788330797
LOC646456	0.006015181	5.947278283	0.788365055
CYSLTR2	-0.010310599	6.032413239	0.788426074
C9ORF79	-0.00661202	6.291864088	0.788518196
LOC642930	-0.00652	5.998974733	0.788590613
LOC387693	0.007696625	6.16061904	0.788603814
KCNQ1DN	0.006996706	5.936860929	0.788605221
SYCP2L	0.007296719	6.159816133	0.788625884
LOC401220	0.006229698	5.98190194	0.788686478
MGLL	-0.010772292	7.932021851	0.788715198
LOC652760	-0.007074071	6.141489766	0.788768666
SLC22A25	-0.007659021	5.911972848	0.788832025
SIGLEC8	0.008014927	6.122971669	0.788841494
ZNF169	-0.006638599	6.117042027	0.788963848
RBM19	-0.00423492	6.148992347	0.789159154
LOC646094	-0.007099493	6.111681251	0.789298013
LOC653493	-0.008032181	5.959837894	0.789364851
SERPINI2	-0.00666088	6.086123078	0.789385731
MGC22014	-0.004661488	5.990903457	0.789429123
TBC1D25	0.006105096	6.525558137	0.78956133
NRP2	-0.004462687	6.040150308	0.789605783
CCDC75	0.006454132	6.28432881	0.789628763
SF3B5	0.015762695	9.928786012	0.789671369
FLJ25439	-0.00799398	6.237905185	0.789867835
HMMR	-0.007361593	6.153191226	0.790007879
LOC653543	0.004861912	6.06654792	0.790035374
LOC648264	0.007058758	5.984435411	0.790123572
ARSA	-0.011715573	6.797594588	0.790155996
ARHGAP5	-0.007797073	6.408353022	0.790194967

LOC652736	-0.006307179	6.134719194	0.790369047
POU6F2	-0.006809401	5.982343763	0.790603226
C1ORF187	0.005091315	6.026337563	0.790713994
GPRASP1	0.00829381	6.141582677	0.790794155
PRTN3	0.006531763	6.255388738	0.79086615
FLJ35282	-0.008639385	6.019242055	0.790867901
RHPN1	-0.00886603	6.433311168	0.790907304
ARL4C	-0.00838789	6.175816824	0.79093133
LOC441347	0.00616331	6.096446416	0.791000438
HBE1	-0.006034399	5.982414557	0.791023433
OR2G3	0.004553556	6.15324779	0.79106111
PARP8	0.005220858	6.290883892	0.791067458
RTN4	-0.025374384	9.232091041	0.791089819
IFNA4	0.005208013	6.150982241	0.791101486
KRT77	-0.007720716	6.004531478	0.791215556
LOC652233	0.006167285	6.117227	0.791276859
FLJ33534	0.006682558	6.071420145	0.791426754
ADAMTS15	0.00754491	6.119631908	0.791459508
CUTL1	-0.018715408	8.998310943	0.79151158
ORM2	0.006405574	5.991208119	0.791556184
GPR27	-0.010144573	7.344529551	0.791615514
C19ORF63	-0.005769044	6.295714464	0.791623542
LOC652644	0.007087966	6.152142426	0.791635847
RPL7A	-0.021607229	8.579354152	0.791814784
FLJ45803	0.006539129	6.023312382	0.791862851
PLA2G4B	-0.015491465	8.09483048	0.791874738
FAM55A	0.006824807	6.019905341	0.791924561
KCNA2	0.007097745	5.908505173	0.79208808
C10ORF92	-0.007280074	6.277857189	0.792121514
OR2A12	0.006979662	5.931275251	0.792164835
DKFZP434J1015	-0.006102964	6.057299469	0.792240302
LOC441120	-0.005647727	6.360079209	0.792598196
LOC652322	0.007442417	6.038149538	0.792665754
LOC401072	0.006820768	6.068711574	0.79275783
MGC45491	0.009580116	6.034218806	0.79282294
STAT1	-0.019532624	8.939505319	0.792823976
SLTM	-0.011779396	8.707537789	0.792853456
LOC645852	0.005813122	5.993675933	0.792979208
LOC392843	0.004646061	6.071687616	0.793006834
MACROD2	0.004894786	6.004293433	0.793154558
KRTAP4-5	0.006835718	5.995720745	0.793163155
LOC648169	0.006629348	6.215227975	0.793424637
LOC652809	-0.006261386	6.12713446	0.793574887
LOC340268	-0.007773728	6.018085176	0.793580964
LOC646795	0.005387585	6.196014348	0.793583204
STARD9	0.008047086	6.115508787	0.793760569
LOC644644	-0.00695932	6.322278231	0.793811482
LOC648674	0.006711893	5.975282942	0.793881326
KCNK16	-0.006556794	6.056036965	0.793977083
FLJ41856	0.007017774	5.992007658	0.793987868
CCDC67	0.006301163	6.016734252	0.794046679

ATOH7	0.00494772	6.049183708	0.794054012
LYPD4	-0.008656272	6.073873777	0.794094747
ITIH3	0.0089485	6.19923181	0.794128155
LOC651991	-0.006979854	5.951665052	0.79414961
KRT79	0.006730116	6.03682735	0.794166291
PSMD6	0.009718241	10.40880853	0.794175015
LOC649771	0.006642653	5.896970257	0.79419741
LOC643381	0.006958791	6.000663269	0.79436857
LOC732391	-0.006251666	5.953749608	0.7944709
LECT1	0.00555564	6.217554833	0.794536949
LOC645365	0.009866686	6.094423262	0.794594985
LOC647786	0.005805687	6.060420249	0.794690467
LOC150207	-0.005927067	6.033562593	0.794697332
PYY2	0.005260613	5.985126351	0.794709361
LOC643240	0.008210961	6.546645473	0.794790469
LOC641957	0.006588162	6.077230688	0.794923271
LOC652779	-0.006276056	6.070584227	0.795150159
LOC391770	0.006237261	6.217205847	0.795178804
HSF5	-0.004999545	5.91991246	0.79526285
B3GALT3	0.006560384	6.185382762	0.795347566
LOC650749	0.007982188	6.156120242	0.795470349
SNX15	-0.006429403	6.451787473	0.795493563
ALOX12	-0.006977248	6.034126653	0.795627649
LCE1E	-0.005367631	5.970474592	0.795644463
LOC651963	-0.007081784	6.116605582	0.795666106
OR52N1	-0.006306827	6.043504962	0.795792836
C1ORF51	0.021827288	6.992196161	0.795794995
CNTNAP5	0.005722225	6.196048024	0.795819614
LOC388922	0.005137542	6.041617122	0.795937334
LOC728758	0.008055047	6.541118884	0.795961752
LOC645953	0.007142531	5.997326684	0.796006868
NR2E1	0.004948183	6.235756523	0.796167017
PLCL4	-0.007638853	6.042970066	0.796428399
LOC643903	0.007295541	6.15737499	0.796454569
SNORD101	0.005990171	6.017852378	0.796533107
OR51I2	-0.006581851	6.059744118	0.796542073
LOC441762	-0.011534279	5.954419076	0.796566734
LIPH	0.00822708	6.202508992	0.796604808
LOC642993	-0.007459087	6.018310128	0.796616522
ARHGEF15	0.0112889	6.495261665	0.79671034
POLD3	-0.005129998	6.3897026	0.796741376
OR11H12	-0.006523449	6.081707936	0.79687016
MREG	0.006598618	6.147102317	0.797034756
LOC652377	-0.010074659	6.559854283	0.797174112
LOC649186	-0.005774991	6.261410505	0.797261157
LOC646215	0.006080274	5.85372425	0.797343992
RABEP2	0.005501506	6.051335669	0.797385234
C11ORF88	-0.006349912	5.993584698	0.797555898
FUT7	-0.009035803	6.109937336	0.797710725
LOC648213	-0.007859624	6.250829409	0.797729452
LOC647251	0.006433949	6.23819438	0.797796073

FAM151A	0.006692909	6.103038207	0.798016147
PRR15	-0.00627603	6.105626771	0.798075567
LOC643757	-0.006784256	6.189872848	0.79809684
FAM70A	0.004636763	6.075381534	0.79819926
PAQR9	0.013189635	7.211966292	0.79826189
MGC40170	0.008117301	6.173821221	0.798346742
SCFD2	-0.00893161	6.343397157	0.798399469
LOC643815	0.011658271	5.968591285	0.798427243
LOC220998	0.005605521	5.989084652	0.798483407
CCT6B	-0.013349317	6.982870981	0.79859421
LRRC3	-0.008093936	6.445519574	0.79860795
KNDC1	0.003945552	6.103977633	0.798678559
LOC653081	0.005496691	6.143708577	0.798882213
VAV2	0.011921707	6.210698963	0.798919441
LOC650632	0.007670913	6.15859982	0.799013268
LPGAT1	0.007595724	6.388076784	0.799065211
NR1I3	-0.004669421	5.9801644	0.799066205
LOC647949	0.005883699	6.022792526	0.799224633
HEATR4	-0.006356367	5.983106941	0.799397836
NAT2	-0.006807375	6.066924433	0.799431259
STX18	0.006829691	6.256376301	0.7994495
LOC647955	-0.007211203	6.123979686	0.799453615
KCNJ9	-0.006534734	5.949975439	0.799454787
WDR88	0.007886019	5.949173217	0.799525761
DCXR	0.01145515	10.16404703	0.799562936
BTN1A1	0.005912164	5.891744876	0.799684275
ADD2	0.003964426	6.052751512	0.799692183
LOC647190	-0.006342044	5.964355701	0.799696389
LOC645797	0.006625527	6.013159075	0.799701772
MYH13	-0.006866123	5.906312724	0.799798998
WNT10A	0.005977677	6.126214696	0.79985782
LOC441056	0.006293945	6.03455843	0.800063125
MBNL3	0.005815767	6.289341906	0.800142909
HARS	-0.010145557	9.528111916	0.800161062
TBC1D2B	-0.012412103	7.784682099	0.800237187
KRT9	-0.007689308	6.58589336	0.800238112
LOC652659	0.006545952	6.220233004	0.800305901
OR10C1	-0.006661441	6.004126712	0.800324009
C20ORF74	-0.006689306	6.094482811	0.800412756
LOC653346	0.006055756	6.101721902	0.800466461
CIDECP	0.008600169	6.29738414	0.800488264
LOC644846	0.005723217	6.119204665	0.800561428
LOC652775	-0.007390896	6.296893863	0.800917901
MAGEH1	0.008638232	6.912718152	0.801151147
KRT7	-0.006994919	6.129255975	0.801226468
LOC643175	-0.006594534	6.136155488	0.801294459
SRCRB4D	0.008371983	6.142758811	0.801408516
LOC730744	-0.010984738	10.53988173	0.801490089
OR5D14	-0.005983234	6.080896873	0.801496089
LOC648370	-0.00606061	6.169157623	0.801512197
CLCN1	-0.007106931	6.030117763	0.801525274

ZNF525	-0.006889548	6.317081624	0.801537085
FLJ37396	-0.007488163	6.460967611	0.801578075
LOC652712	0.008131086	5.962655096	0.801642491
LOC650921	0.006504081	6.156053566	0.801657136
CES2	-0.019277877	8.973727362	0.801803704
LOC646461	0.006022655	6.043242422	0.80182394
LOC653284	0.005613774	5.997209643	0.802141125
CEBPA	0.015448365	6.588261358	0.80214249
DSCR8	0.004173427	6.171374937	0.802412192
IL8RA	-0.008384875	5.993815295	0.802430829
C12ORF56	-0.006018066	6.123059103	0.8024523
TBX20	-0.003800039	6.151970475	0.802500983
OR4D10	0.004762283	6.218266127	0.802546547
LOC390427	-0.005723755	6.005770123	0.802557156
ATP10A	0.007760443	6.270236509	0.802609646
FAM19A5	-0.006977119	6.081492611	0.802712264
RFFL	0.005115841	6.350696006	0.802733288
LOC649184	0.006442958	6.057459452	0.802774255
LOC399706	-0.006308593	5.866797638	0.802882486
DGAT2L3	0.006537968	6.063439702	0.802902722
LOC646176	-0.006437598	5.958209128	0.80293238
LY6D	0.006539777	6.03273813	0.802980816
LOC643444	0.006425703	6.184652028	0.803001842
LOC344167	-0.007195848	6.257304232	0.80306942
EPO	0.00599914	5.903421869	0.803112753
ANK3	0.01620764	7.804019899	0.803132897
SAMM50	0.012875468	10.65643436	0.803191343
CALML3	-0.006564847	6.060129168	0.803298074
ROBO2	-0.00430435	5.987712735	0.803304734
MAPK1	0.006871988	7.188616991	0.803363615
KCTD8	0.006934154	6.184516484	0.803412909
NKAIN4	0.005666714	6.024293897	0.803480328
LOC650851	0.006456519	6.06909867	0.803602916
ABHD12	0.009976054	7.407921502	0.803614593
C18ORF54	-0.004908598	6.419208462	0.803672386
ANKRD34C	-0.00655237	6.144137691	0.803860435
LOC648025	-0.007737104	5.912503012	0.803914509
LOC653978	-0.006513823	6.12560014	0.804004573
RPGRIP1	-0.006988705	5.920551034	0.80401127
OR13F1	0.006291591	5.881348896	0.804020117
CR2	0.003666103	6.021145064	0.804057964
IL1F7	0.004175805	6.092220612	0.804212599
MMP20	-0.004961164	5.967202517	0.804245635
LOC441368	0.006118365	6.101669093	0.80429725
C2ORF57	-0.00605907	6.132963818	0.804334904
MMP27	0.006225556	6.103812754	0.804398667
LOC652483	-0.006163451	6.016765092	0.804466557
PCK1	0.00497513	5.997515402	0.804498372
LOC642147	0.005179792	5.999374142	0.804658084
FLJ22639	-0.008040807	6.297110888	0.804718287
SPECC1	-0.004897489	6.127744798	0.804847966

SPAST	-0.007291365	6.629545403	0.804903144
LIPK	-0.005538339	6.072400564	0.804915783
SLC5A4	-0.00689621	5.949151924	0.804975896
XPNPEP2	-0.006745392	6.588447603	0.805109975
LOC652683	-0.00571483	6.104919262	0.805202081
LOC728660	0.006428773	6.108217456	0.805233144
WDR21A	-0.010189893	7.154351302	0.805372661
TSGA13	0.006293749	6.029416323	0.805376186
ALX4	0.006554951	5.936313045	0.805450603
LOC642624	-0.006852629	5.943245546	0.805485223
LOC390531	0.007389373	5.981324408	0.805619916
APOF	0.006036827	5.992300317	0.805667545
LRRIQ1	0.006279729	5.916148779	0.805808308
LOC647621	0.005448883	6.097147319	0.805889981
MYO7B	0.006349621	5.9649459	0.806003745
KLK4	0.00613159	6.026496437	0.806013257
ODF4	0.006703991	6.149656557	0.806023858
DCBLD2	-0.020515212	9.973692357	0.806112389
LOC653605	-0.006450903	6.020665536	0.80616176
LOC642688	-0.006281075	6.037890429	0.806195299
NXT2	-0.009852227	6.422512963	0.806249552
C10ORF81	-0.005548535	6.097094677	0.806279174
TREML3	0.003950298	6.026838374	0.806520051
LOC650538	0.007267182	6.20412506	0.806635104
HHIPL1	0.006137703	6.098734412	0.806714534
LOC647589	-0.006319104	6.232260656	0.806789979
RNF181	0.012634635	10.27805935	0.806897561
LOC645200	-0.005343212	6.018812302	0.806973296
TRIM11	0.011899749	6.529791162	0.807374173
DLL4	0.008078247	6.3459519	0.807386525
TAKR	-0.004146553	5.979230107	0.807449246
KLRC1	0.003870433	6.009961166	0.807503431
LOC644569	-0.006120383	5.870269532	0.807534955
LOC645320	0.004529617	6.039383899	0.807723835
FGF10	-0.005998367	6.006483634	0.807759705
ZNF124	0.004910959	6.097144098	0.807765501
LOC134541	-0.005977597	6.044337176	0.807834952
TPH1	-0.007343491	6.259781276	0.807902083
MGC52498	-0.005975593	6.263596462	0.807966369
RETNLB	0.007081278	6.177630069	0.808013858
LOC440277	-0.006316855	5.988352002	0.808057587
LOC648748	0.007625168	6.180269339	0.808089725
MGC42630	-0.009599374	6.922408309	0.80820953
ZP1	-0.00641281	6.156542365	0.808293019
RFNG	-0.007960669	7.464111012	0.808304008
RBM35B	0.006529444	6.05811397	0.808328106
ZNF516	0.006251361	6.21899133	0.808351754
SPANXN3	-0.005613518	5.907500026	0.808400219
LOC653110	-0.005283993	5.980179121	0.808430211
LOC651079	-0.005817214	6.010186579	0.808510451
BAHD1	0.010903176	6.051788283	0.808598701

LOC440888	0.006061506	6.119353805	0.808614496
MUC13	-0.007788219	5.983576205	0.808618815
LOC136242	0.005203408	6.054800207	0.808669114
MGC39581	0.005339493	6.019315605	0.808683847
NOX1	0.003678695	6.123722853	0.808763728
CFC1B	0.006930829	6.357999572	0.808782013
TMEM211	-0.006608115	6.01972685	0.808816625
LOC648846	0.006212517	6.188862163	0.808840726
GPR148	0.006152943	6.065682672	0.808930012
LOC648976	0.005505215	6.0772508	0.809042556
LOC649926	0.007049967	6.243509698	0.809245473
ARNT	0.011899676	6.378329168	0.809255124
LOC390507	0.005557459	6.078136434	0.809271808
SIX1	-0.005895385	5.989490269	0.80930396
LOC645790	-0.006022289	6.096367229	0.809304439
FARP2	0.007276982	6.779957382	0.809319346
LINCR	-0.005880482	6.116582177	0.809369545
LOC642237	0.005360038	5.983866334	0.809446956
LOC642846	-0.005442704	6.029722853	0.809603128
LOC649436	-0.005942386	6.051670888	0.809624612
NDRG1	-0.01548039	8.948184949	0.809729063
C16ORF61	-0.011320713	11.93960333	0.809806784
ADAMTS18	0.005102322	6.090231266	0.809814879
DLGAP2	0.008103469	6.639739931	0.8098297
P2RX2	-0.003006402	6.102888762	0.810186509
ELOVL7	-0.005683199	5.998160275	0.810189453
PRPF39	0.005788485	6.34425472	0.810226298
TMEM174	0.0086799	6.060220001	0.810422296
LSMD1	-0.01053554	8.899774141	0.810444901
LOC648905	-0.005514943	6.024521977	0.810468476
KCNA6	-0.008108321	6.049489327	0.810519504
LOC654200	0.006940463	5.918791759	0.810628601
YWHAE	-0.014787977	7.439215637	0.810693302
LOC652367	-0.006170929	6.177208903	0.810786886
ZNF180	0.005771607	5.943762203	0.8108204
CCDC76	0.008019763	7.147699106	0.81083482
ANKRD17	0.007063147	7.597226758	0.81083956
VPS26A	0.010886737	9.886219052	0.810852143
LOC647174	0.007176314	6.115056999	0.810883637
VISA	-0.008521498	6.407761668	0.810920958
LOC647827	0.006183128	6.39877334	0.810944818
NODAL	0.005875395	6.203548878	0.81098689
MGC3196	-0.009670505	7.628217513	0.811114508
ALG10B	-0.004924239	6.182383883	0.811128983
LOC651158	0.006753848	5.956895898	0.811180338
KIF2C	0.008022746	6.298423197	0.811186226
FOXG1	0.009236326	5.952309544	0.811374337
DTNB	0.006174202	6.288261498	0.811414596
ST8SIA3	-0.004561139	6.224955006	0.811458771
LOC729025	-0.006306187	6.060882989	0.811473861
PITPNM3	0.006486023	5.923013955	0.811605899

SDAD1	-0.01070817	9.091640671	0.811713707
ALB	0.004042235	6.059619123	0.811787083
RGS8	0.007198324	5.965985013	0.811861247
LOC650427	0.005366961	6.120394438	0.811870378
CCDC142	-0.006498394	6.045983107	0.811891656
CXORF59	-0.008394313	5.97284702	0.812042433
LOC651763	0.006718458	6.219969888	0.812126421
LOC653751	0.008863365	6.174137262	0.812216341
LOC652234	0.005473011	6.028910684	0.812259918
MYO15A	-0.007745092	5.954864219	0.812331231
PNOC	-0.006746566	5.949384571	0.812401394
CIB2	0.011725889	6.750964832	0.812412773
ITGA6	0.012413804	6.781328886	0.812441873
LOC642693	0.006239475	6.020314762	0.812524005
LOC643140	-0.007619334	5.909359135	0.812537133
LOC85390	0.007595758	6.261484717	0.812594112
LOC643359	-0.00564575	5.995462078	0.812755308
RAB11B	0.008220753	6.728646203	0.812777326
LOC652525	-0.006062085	6.295558657	0.812794751
NEK10	-0.004531123	6.242579283	0.812829286
C1ORF226	-0.004174044	6.081403191	0.812866035
MTAP	-0.010497381	7.765890975	0.812936394
LOC650874	-0.006198465	6.119842025	0.813053262
CD160	-0.009868896	6.800805957	0.81308041
PNPLA1	-0.004265372	6.01721249	0.813105088
LOC642895	-0.00536931	6.14590248	0.813168386
CCDC126	0.009218536	6.757560433	0.813183528
KRTAP19-6	-0.006766628	6.579857497	0.813211218
LOC652048	0.006361209	6.191393385	0.813218201
SPRED2	-0.010497283	7.909500577	0.813252843
WNT1	-0.006502423	6.015402057	0.813277979
MORF4L1	-0.014220766	10.01855426	0.81336229
KCNC2	-0.004011888	6.016827204	0.813472765
MBTPS1	-0.008533137	8.928753658	0.813534925
SIX4	0.008618891	6.667466168	0.813544016
PNMA3	-0.017916044	7.027503396	0.813593775
LOC645043	0.005283992	6.05597837	0.813718941
LOC648758	0.005109831	6.12533446	0.81372919
TFDP3	-0.00623106	6.071105123	0.813807766
ABCB1	0.008136177	6.494097689	0.813997367
SPAG17	-0.005287405	5.986989348	0.814031004
GIMAP4	0.013879433	9.640597701	0.814080192
ZNF192	-0.004887032	6.170006325	0.814096206
LOC642538	0.005681264	6.08328326	0.814124708
ZNF584	-0.005869254	6.446716627	0.814167209
LRRC17	-0.017631641	6.941287048	0.814184711
LOC644899	0.005709365	6.161376171	0.814269859
LOC387931	-0.005241759	6.014873814	0.814340452
MAGEL2	0.00911941	7.327594116	0.814447578
C6ORF134	0.003530966	6.197798953	0.814460308
UXT	-0.01166331	9.623273671	0.814468601

LOC651362	0.005099283	5.958951843	0.814529898
TARBP2	0.006874347	7.431713342	0.814563394
SLC22A11	-0.006024297	6.094860318	0.814583328
TMPRSS11E	-0.005924597	5.962653928	0.814600473
RGPD1	-0.007723962	6.215802103	0.814685511
LOC641945	0.005013543	6.057669878	0.814935293
ZDHHC11B	-0.005585477	6.03543024	0.814984148
NPNT	0.012881335	6.225671731	0.814985148
PRRX2	0.014228212	6.768149681	0.815030489
SNORD28	0.005058092	5.902785436	0.815176484
RABIF	0.004362707	6.291265207	0.815404755
LOC648408	0.005087922	6.156415324	0.815521662
LOC651296	-0.009448095	6.168048399	0.815630019
IGSF11	0.005013667	6.411388156	0.815667205
ZNF131	0.009029298	6.061298666	0.815727804
ATP4B	0.003651451	6.092836023	0.815752455
LIMD2	-0.005876606	6.052884329	0.816005723
LOC642648	-0.005559688	6.23758305	0.816058078
ERN2	0.0061554	6.150937761	0.816090735
ACTRT1	0.021255076	7.151436558	0.816203458
C12ORF66	0.00813504	6.448131822	0.816244336
MARCH9	-0.006796264	6.221813357	0.816289965
MTFMT	-0.007307121	7.387730971	0.816338359
LOC730145	-0.005631456	6.012214512	0.816434647
ZNF354A	0.011377826	6.375567082	0.816578926
LOC647370	0.007966163	6.012479733	0.816769853
PSMD5	-0.007670823	7.293725145	0.816772352
LOC441086	-0.005951809	6.13107724	0.816814621
CTNNBL1	0.017116414	8.56603359	0.816818616
LOC651581	-0.005537572	6.145641893	0.816842884
FCGR3A	0.004707574	6.199340902	0.816853295
ZNF239	-0.007934621	6.823506542	0.816881297
OFCC1	-0.005535353	6.037493367	0.816984962
KLC2	0.005281004	6.33053738	0.817007126
CHP	-0.017516175	8.876386832	0.817012065
N6AMT2	-0.011085051	7.686209853	0.817096078
TRIM10	0.003335092	6.009363189	0.817228777
PLP1	-0.00672788	6.384814057	0.817377568
OR4D6	-0.006008813	6.166458492	0.817461279
LOC200726	0.009742038	6.452651922	0.817532211
GDNF	-0.005714705	5.993858224	0.817549027
NDUFB8	0.007979285	13.30943937	0.81755428
LOC650217	-0.006583869	6.058856047	0.81756213
BCAP29	0.009389953	7.911125061	0.817609835
LCE1F	-0.006034945	6.053770727	0.8176935
GBF1	0.012983369	7.647332702	0.817726124
OR2T1	-0.008476919	6.073996337	0.81775068
LOC643479	0.006494863	6.063088968	0.817810798
LOC653808	0.005105257	6.223604139	0.817901713
BICC1	-0.004998488	6.048297812	0.817917661
FANCF	0.005894756	6.230482119	0.817970181

KLK12	-0.004335036	5.997745602	0.818090245
LOC649676	-0.00597561	5.991734145	0.818187374
UNQ5830	-0.00546218	6.040978612	0.818430605
LOC648603	0.005673433	6.046033554	0.818435464
ITGB1BP2	0.015535213	7.711127883	0.81866198
LOC651287	0.005702998	6.125493738	0.818753003
LOC654114	0.00978699	5.947150172	0.818795539
LOC650010	-0.005875748	6.175395228	0.818840424
LOC650005	0.00960857	5.976538959	0.818849114
DDX27	0.013924303	7.374245779	0.818850833
NARG1L	-0.005560487	6.548542796	0.81891417
LOC647541	0.006562932	6.212284463	0.818922626
RLBP1L1	-0.005591805	5.972084432	0.818955475
LOC643246	0.008315779	6.030022494	0.819069349
MGC10646	0.005193786	6.083117652	0.81909853
MAGEB1	0.004131516	5.971363061	0.819103051
LOC646607	0.004676556	6.14498854	0.81924661
LOC649946	0.023036409	8.783055072	0.819286717
FSTL5	0.004966399	5.88414466	0.81940963
LOC645580	0.005859444	6.036047479	0.819430342
LGR6	-0.004848887	5.983692656	0.819442193
TRIM48	0.006247178	6.026929055	0.819485943
RELN	-0.012460678	6.358232112	0.819581248
SLC7A11	-0.00692961	5.967070422	0.819583923
GRM5	0.009007161	6.07292773	0.819625375
LOC654116	-0.006407498	6.103998949	0.819655344
FLJ31132	-0.00563422	6.114218723	0.819693464
C8G	-0.007251399	6.338742222	0.819794519
LOC401056	0.007096697	6.284509945	0.819956783
DRG1	0.010756356	9.773853389	0.820050835
LOC642616	-0.005056816	6.116115935	0.8201995
LOC644578	0.005213084	6.129037939	0.820224391
CCDC62	-0.004464375	6.221866178	0.820241309
LYRM1	0.010088104	10.3562918	0.820247933
MGC119295	-0.009897446	6.021380939	0.820272883
LOC647723	-0.005762483	5.996637577	0.820351975
KRTAP22-1	-0.006012046	5.972562791	0.820485481
DDAH2	0.015938593	6.900247404	0.820565099
DEFB106A	-0.005545557	5.906898776	0.820648631
LOC402110	0.005783633	6.130113836	0.820764106
LOC152667	-0.003917041	5.952476027	0.820875262
C1ORF192	0.00699404	6.111640309	0.820876066
LOC644826	0.00527218	6.033302527	0.82106255
JPH2	0.009521208	7.705726881	0.821271481
PVALB	-0.005843564	5.962202461	0.82141114
TSPAN1	0.007216143	6.491547464	0.821422829
TMEM1	0.005116473	6.654090009	0.821450633
LOC653080	-0.007334456	6.508383274	0.821480418
CCL4L1	-0.009597034	6.173390946	0.821511513
LIM2	-0.005738313	5.900901665	0.821527588
LOC642856	0.006130125	6.027829823	0.82162073

OR2T2	0.004629154	6.240702386	0.821751426
LOC441066	-0.010727941	6.085133827	0.821947787
LOC648533	-0.005779687	5.934413072	0.82198939
POU3F1	0.005056777	6.023032411	0.822176535
C17ORF54	-0.005515295	6.156112506	0.822265631
EMG1	0.010878227	9.108448596	0.82266009
IPO9	0.005518978	7.50052788	0.822662766
LOC644853	0.0061832	6.063239823	0.822687301
LIMD1	-0.005729145	5.987337239	0.822719093
KIAA1409	-0.004569666	6.110468808	0.822854935
LOC647281	0.004912686	6.068951574	0.822875387
LOC650810	-0.005373052	6.100313407	0.822909276
AATK	0.003921452	6.200842052	0.823048257
MYO5C	-0.013549978	7.068215032	0.823062397
GEMIN5	0.007098295	6.928332911	0.823207942
FLI1	0.009946777	6.293974977	0.823219675
LOC649921	0.005204751	6.012972287	0.823260828
OR10A7	0.005484183	6.053789524	0.823289603
ZNF792	-0.008185863	6.850352678	0.823414882
LOC652379	0.005980908	6.072665031	0.823421884
DLEU2L	0.005758198	6.07230619	0.823454177
LOC647439	0.008385579	6.079052295	0.823481306
PTPN5	-0.007797272	6.081399087	0.823485778
ZNF343	0.009459497	6.082029759	0.823863049
CASC5	-0.004072542	6.038265483	0.823900818
LOC643451	0.005334801	6.207947882	0.824021692
STAR	-0.003815554	6.146455445	0.824046488
LOC644480	0.004712766	6.010022945	0.82424081
LOC649773	-0.005643194	6.062599553	0.824269644
FILIP1	0.012987506	8.644887622	0.824282835
DNAH11	-0.005247779	5.910418633	0.824307705
MYST2	0.006903494	6.704289693	0.82437109
A26C1B	-0.00517609	6.145106783	0.824461599
OR2T35	0.004115086	6.024271901	0.824495954
LOC441799	-0.005174805	6.05688805	0.824508883
DDX20	-0.007784218	6.549918308	0.824510497
TRAPPC1	-0.009686008	9.353927699	0.824521244
LOC645133	-0.006215002	6.232465625	0.824575449
CALML4	-0.008369898	7.796281571	0.82461689
SLC6A14	0.005273505	5.958515216	0.824627634
C15ORF28	0.0080853	6.509072852	0.824654272
MRPL33	-0.011691425	12.16693845	0.824655844
TMEM151	-0.008678384	5.949695619	0.824771282
ZSCAN2	-0.00398873	6.622484425	0.824772488
C11ORF47	0.003621245	6.139483433	0.82477796
SKAP1	-0.005146456	6.156956667	0.825184097
RGPD2	0.008235974	6.289831235	0.82519082
LOC643186	0.005913782	6.016559633	0.825252022
LOC642166	-0.006747597	5.915763403	0.825288473
ANKS4B	0.00572027	6.051665323	0.825343117
MRPL27	-0.010096333	8.210551776	0.825400111

SPANXC	-0.005625356	5.890277903	0.825653678
LOC642032	-0.005411869	6.045830065	0.825668578
LOC649112	0.006844987	6.090589664	0.825681549
LOC641987	-0.005760211	6.274026675	0.825806674
FLJ46010	-0.005769813	6.025070444	0.825905662
SLC43A2	-0.010434442	6.876226425	0.825931024
USP30	-0.005769344	6.566521153	0.825942598
ZNF710	-0.004886395	5.905570571	0.825987074
LOC646779	-0.004919494	5.969187214	0.826029485
CACNG1	0.00631642	5.989920379	0.826236236
OSTALPHA	-0.006293952	6.038174215	0.826243699
NDOR1	0.008322229	5.976676435	0.826320241
MRE11A	0.006279146	6.908764821	0.826524168
B3GNT4	0.007941321	5.987289487	0.826524248
CYP2C18	-0.005127548	6.064967397	0.826555479
WDR44	-0.006492722	6.73546825	0.826622319
LOC644897	0.005169877	6.009945874	0.826727291
LOC653520	-0.005126917	6.258731018	0.826751705
LOC652368	0.005167636	6.028697369	0.826908952
FRG1	0.015195259	9.102248733	0.826992494
LOC642017	-0.007119717	7.073932766	0.827069169
FAM66E	-0.00551559	6.060945905	0.827094577
PLB1	-0.005788247	6.23164644	0.827131115
LOC642701	0.006488958	6.027586947	0.827171602
LOC727752	-0.006107975	6.051017971	0.827179798
KLHL5	0.009928124	7.707526234	0.827304743
TMEM83	0.0067853	6.335972598	0.827351423
LOC646396	0.005551159	6.125096141	0.827372859
FLJ22795	-0.011369308	7.415967998	0.827430253
LOC646799	-0.005126672	5.914764368	0.827440392
GCKR	0.005110821	6.183494593	0.827441159
LOC728493	-0.005532657	5.959321923	0.827471458
LOC649993	0.006086708	6.253532683	0.827537293
DMXL2	0.00672901	6.40184052	0.827720006
LOC651589	-0.005095202	6.034159993	0.827731613
LOC644117	0.006044799	6.452753036	0.827778448
LOC340900	-0.005361371	6.029228811	0.827845714
LOC642843	0.008318741	5.994131583	0.828016759
LOC642204	-0.006160549	5.964619584	0.828254697
C19ORF20	0.005295246	6.263154169	0.82825689
IFITM4P	-0.004461665	6.175701236	0.828304637
MAP3K13	-0.00544412	6.224026959	0.828561899
UQCRFS1	0.010354649	13.45215026	0.828680887
LOC727992	0.004866707	5.976122526	0.828751359
LOC339862	0.004849038	6.106404653	0.828761695
CSMD2	0.009668789	6.120518372	0.828822061
LOC650154	-0.005055512	5.939943584	0.828891968
C15ORF42	-0.005269326	6.022260408	0.829012058
LOC653765	-0.005406114	6.459256376	0.829171135
LOC654164	-0.005409669	6.127487879	0.829608938
PHKG1	0.018092081	6.9674049	0.829676976

TMEM9B	0.007004351	10.2045366	0.829791946
REG1A	0.00492478	5.994056894	0.829815769
LOC650943	0.005249643	6.217659919	0.829989668
ATP5G2	-0.015495424	10.36735897	0.830017854
CPB2	-0.003286578	5.987279053	0.830017867
UVRAG	-0.007224839	6.690215646	0.830162215
OR6B3	0.005960288	6.162031242	0.830174896
LOC729266	0.005434237	6.023290844	0.830312598
BDH1	0.010675362	6.903073451	0.830392583
HNRNPL	0.011619974	8.67287786	0.830449517
LOC728226	-0.003915058	6.16732465	0.830453561
MCM3AP	-0.007888136	8.914639192	0.830548647
LOC652800	0.005359668	6.124893634	0.830667894
LOC728956	-0.00521199	6.067023415	0.830770742
ERAS	0.005613369	6.041920172	0.830782523
FETUB	-0.005794101	6.121104804	0.830812348
OR5C1	-0.00518215	6.175197181	0.830829883
LOC648469	0.006199275	5.928661986	0.830894289
LOC339789	-0.006082452	5.926166628	0.831029264
PITX2	-0.002920215	6.121974193	0.831065197
TTLL8	0.006125381	6.050445738	0.831137026
GGTLC2	-0.006226875	5.90081801	0.831139617
KIF3B	0.007656871	7.333181101	0.831140598
LOC646892	-0.003966633	6.023249353	0.83114942
LOC649238	0.00557033	6.1393784	0.831342072
KRT6C	0.004886241	6.017513373	0.831450677
ORM1	0.005190516	6.044381303	0.831630588
MRGPRD	0.006322091	6.110591478	0.831868325
CPO	0.006508576	5.978315894	0.831878053
LOC652472	0.00492111	5.978060783	0.832142858
LOC648132	-0.005298148	6.049035333	0.832144233
LOC729218	-0.005292878	5.987918568	0.832159177
PASK	-0.004180232	6.155293002	0.832192694
LAMB1	0.012409752	8.322750296	0.832276162
LOC731227	-0.005069399	6.22591903	0.83236886
LOC440836	0.004796	6.105522748	0.832376444
C8B	-0.005783469	5.957592701	0.832376608
LOC652185	-0.005063417	6.106733173	0.832415678
GPR152	0.004810786	5.985582337	0.832542725
FANCA	-0.003373139	6.13142445	0.832660147
CSH2	-0.003869461	6.074532184	0.832733453
LOC652652	-0.005145488	6.086323196	0.832763516
C15ORF55	-0.005351802	6.077180771	0.832779862
LOC338805	0.005236892	5.918295794	0.832815136
CHRNA9	-0.003982793	6.077091025	0.832816268
MEIS3	-0.004767985	6.273002293	0.832820929
ELA2A	0.005628306	5.938328587	0.83282477
LOC730546	0.005016597	6.121867668	0.832920564
LOC643664	0.008628043	5.904038121	0.832929983
TSHR	-0.002703037	6.017078507	0.832939214
SLC45A2	0.004062037	6.034324622	0.832951324

LOC647448	0.00626447	5.956155682	0.832953015
PACAP	-0.006104367	6.167142063	0.833054492
RNFT1	-0.008029895	6.842921904	0.833059139
ZNRF4	-0.005236023	5.959513427	0.833105639
OR52B2	0.005415549	6.018828202	0.833177059
PCDHA10	-0.004001777	5.949013577	0.833263523
CCNL2	0.005306189	6.383036448	0.83328757
SLC25A29	0.006765477	6.785257697	0.833287747
SERPINA7	-0.005908622	6.031431196	0.833348491
FOXE3	-0.004302526	6.074177395	0.833438612
UBXD7	-0.007454304	6.145420864	0.833497272
GPR155	-0.003680417	6.037664509	0.833716638
LOC442015	0.005685977	6.105844778	0.833968596
OR11A1	-0.003691233	6.095307261	0.833979534
ABCA3	-0.010905009	7.09751013	0.834057058
HDAC6	-0.007276752	7.332617613	0.834241612
LOC653496	0.006627228	6.779082738	0.83428178
SFTPD	0.010045695	6.466931092	0.834332622
LRGUK	-0.005079409	6.074069233	0.834386052
LOC100101266	-0.003489733	6.054798777	0.834520655
LOC731431	0.004785007	6.085618314	0.834543103
SEMA4F	-0.00570092	6.927618967	0.834595847
LOC650346	0.005444319	6.213661741	0.83466185
BDKRB1	-0.005971263	6.24008519	0.834716831
LOC283824	-0.003562321	6.034853002	0.834801776
KLKBL4	0.005143842	5.932879837	0.834996186
LMX1B	0.006010897	6.122188076	0.835024798
LOC652038	-0.006980702	6.124870269	0.835076527
LOC222699	0.005137722	6.084339459	0.835108296
LOC650009	0.005883535	6.069036154	0.835249797
SCN11A	0.005068365	5.972797559	0.835449801
TMEM101	0.008987217	8.530915969	0.835510065
WDHD1	0.003724458	6.29002824	0.835606999
LOC643612	-0.005909782	6.314915849	0.83561641
OR10H4	-0.004365393	5.992299654	0.835768019
MGC42638	-0.004487256	6.009848096	0.835845816
ICAM5	-0.005883035	6.00225471	0.835890527
LOC648754	-0.005135096	6.069860425	0.836010848
LOC647279	-0.00561751	6.003345683	0.836046608
FAM55D	-0.002974567	5.967042504	0.836130639
NAPSA	0.005723681	6.081980241	0.836144807
LOC647719	-0.004460743	6.011819336	0.836198315
DHX16	0.007497721	8.869420529	0.836225988
ULBP1	0.005431291	6.164558762	0.836479044
OTUD6A	-0.005680259	6.128584699	0.836491449
CACNB2	-0.014958934	7.295920611	0.836530207
C1QL3	-0.003851821	6.140546482	0.836555349
GALNT8	0.005290178	6.175875623	0.836608709
CTF8	-0.003784523	6.162296978	0.836702285
WFIKKN2	0.005358599	5.941853896	0.836801143
SIPA1L3	0.012059282	7.198890919	0.836816099

GRM4	-0.005466372	6.210241481	0.836908373
SNX32	-0.006379561	6.236086778	0.837079709
COX6B2	-0.003841836	6.077478413	0.837151501
LOC646047	-0.004919521	6.238324528	0.837285002
SPAG4L	0.005463882	6.319272199	0.837343202
LOC653878	-0.00852469	6.153682439	0.837415027
LOC729569	-0.00568888	6.005991039	0.837634851
LOC400891	0.005596852	6.043401681	0.837667848
LOC648548	0.005073989	6.153156991	0.837766504
OR6K6	-0.005708255	5.93110674	0.837793818
HNRNPA3P1	-0.011280254	6.327059181	0.837802377
LOC650883	-0.005893184	5.968862584	0.837835072
CPA4	0.005492858	5.997904838	0.837851229
TMPRSS5	0.005001829	6.021740676	0.837895876
LOC402145	0.005352872	5.984157907	0.837962792
HSP90AB4P	0.00489983	6.025107868	0.838118186
MARK2	-0.006774335	6.496932923	0.838219577
FCGR3B	-0.006200455	6.770587143	0.838224474
TMEM203	-0.006462604	9.474465101	0.838363979
ACOXL	-0.005616103	5.960970027	0.83842559
OR51Q1	0.005168873	6.202497648	0.838494828
VMAC	0.006566263	6.262656798	0.838508072
ZBTB34	0.005114512	6.835347512	0.83862572
YDD19	-0.004398749	6.059407391	0.838705412
LOC650014	0.004420474	5.955611541	0.838868084
LOC644335	-0.005262172	6.111689149	0.838901574
NRXN1	-0.003914173	6.067924444	0.838934999
LOC729164	0.005661476	6.078064911	0.838935598
LOC645253	-0.006827757	6.008712299	0.838994
LOC387647	-0.012778655	7.029116832	0.839082655
FBXO46	0.008533803	6.920679943	0.8391419
GRSF1	-0.00891908	10.20208551	0.839210643
LOC647542	0.004580266	6.131717265	0.839265362
OR5L2	0.003652852	6.083403419	0.839326243
LOC132203	0.005228665	6.098992761	0.839375727
LOC731050	-0.004844248	6.069580206	0.83959701
RASSF10	0.005178147	6.093267236	0.839599411
CARTPT	-0.005786428	6.430203532	0.83964271
LOC390829	0.004990764	6.071071729	0.839643717
LOC652080	0.005185569	5.882227151	0.839646347
NKX2-2	0.005098356	6.046374456	0.83966108
FGF19	-0.006200423	6.142216445	0.839668094
GRLF1	0.004260471	6.126240661	0.839693741
GTF2A1	-0.004454288	6.22469833	0.839824566
SEPT3	0.00376581	6.021656661	0.839849479
OR5B2	0.004987114	6.128168524	0.840061739
LOC645994	0.004879047	6.171838634	0.840199032
LOC401629	0.005752547	6.104605995	0.840258795
IFNA16	-0.00516486	6.008572769	0.840315463
LOC650681	0.006211615	6.109555276	0.840383156
FLJ33590	0.005728202	6.192969181	0.840424355

LOC731170	0.0062139	5.91090727	0.840500881
LOC644045	-0.005653278	6.030185265	0.840644453
LOC646283	-0.004594407	6.07658851	0.840672167
OPRL1	-0.00438989	6.137230286	0.840706913
LOC652894	-0.005285025	6.019049057	0.840808647
LOC441426	0.004358744	6.146524399	0.840903809
PUS3	-0.006264731	7.103048707	0.840942835
LOC648993	-0.007268863	5.99682656	0.840993254
OR4F15	0.004817794	6.017511879	0.841026161
AARS2	0.010414859	8.0217017	0.841060702
KIF14	0.006137471	6.45960815	0.841078479
SLCO1B1	0.003888238	6.094864128	0.841120218
LOC647250	-0.006573056	6.021718013	0.841157706
SERP2	0.013273973	8.626893264	0.841280294
CRB2	0.005238425	5.997100532	0.841380955
C7ORF30	-0.009421086	11.59937564	0.841393906
MRGPRX4	0.004906866	5.98690394	0.841635563
MED18	-0.005937612	6.261374951	0.84188708
LOC646376	-0.00520691	6.061601248	0.841957924
KLK9	0.005317135	5.949089449	0.842031784
OR6B2	0.005477245	5.983298015	0.842236861
GZMM	-0.005073974	6.044758034	0.842338671
LOC651933	0.004913448	6.060730839	0.842369745
COPS5	0.008311219	11.24229399	0.842536664
LOC643328	0.004222279	5.967429861	0.842557962
SFTPA2B	-0.004243507	5.974243877	0.842617696
LOC644662	0.003740735	6.135187902	0.842632561
FOXN2	-0.00943639	8.200974963	0.842696018
C1ORF69	-0.006431321	6.837911096	0.842724878
TTTY4C	0.004705852	6.074892089	0.842949491
LOC731827	-0.004602525	6.123488713	0.842960049
LOC642611	-0.004922999	6.022204655	0.843004572
AGER	0.004400716	6.050065145	0.843040308
ITGB1BP1	0.011903203	9.004665033	0.84312384
NDST3	0.004847811	6.029961063	0.843199338
RND1	0.005138415	6.114201673	0.843226224
LOC645643	0.005466895	5.964520487	0.843272994
LOC402199	-0.004980716	5.967281284	0.843355509
RASGRP4	0.002742651	6.016203453	0.843395304
RHCE	-0.003550189	6.117014901	0.8434547
LOC150383	-0.005469657	6.039227096	0.843770295
RNASEH1	-0.007235701	8.897551633	0.843901221
LOC391574	-0.005054114	6.174665053	0.844039304
KIAA0415	-0.006051949	6.434113704	0.844140403
PPIF	0.007676205	6.796264463	0.844168146
TCTE3	0.004923371	6.006078981	0.844181133
DCLRE1C	0.008238408	7.565341165	0.844264695
LOC646881	0.005218031	6.272145269	0.844341343
PIP5K3	0.002945932	6.004120237	0.844434549
LOC651619	-0.006593759	6.081419014	0.844630668
SLC7A5P1	-0.00355034	6.024417137	0.844870466

ASB8	0.014649836	8.213005727	0.844955493
INPP4A	0.0036212	6.141810817	0.84498965
LOC649094	-0.002856105	6.002181489	0.845059989
ARHGAP11B	-0.005303924	6.128150495	0.845104799
LOC653397	-0.005320838	5.93912889	0.84510692
LOC653684	-0.005066581	5.949569274	0.845190024
LOC645563	0.004901353	6.231580406	0.845246986
IL21R	0.002889657	6.191495387	0.845372761
GDPD1	-0.007378481	6.951973144	0.845446428
HIRA	-0.008144177	6.153839627	0.845462552
GNRHR2	0.006834799	6.029479572	0.845473565
RBMXL2	0.005299127	5.957205025	0.845487118
LOC648541	-0.005149652	6.104536242	0.845559666
SERPINA13	-0.00520203	5.982638338	0.845621243
CXORF42	-0.005461647	6.163605325	0.845736231
DRD3	0.003541412	6.087559889	0.845738229
LOC653635	-0.007413338	6.827374002	0.845771903
LOC732058	-0.004217707	5.965825098	0.845851097
LOC283887	-0.004671422	6.151734999	0.845860455
AURKB	-0.005310118	6.078358514	0.845938402
CD300LB	0.0048955	6.046685188	0.845959841
FLJ33706	-0.004472688	6.087701073	0.846008283
LOC646471	-0.00494253	6.052172847	0.846079595
LOC652184	-0.004298477	6.080109345	0.846176348
DSCR9	-0.004456566	5.994078889	0.84628662
SPIC	0.003899976	6.018173576	0.846316908
SPERT	-0.005764039	6.202223682	0.846355462
FLJ20209	0.005339718	6.348957881	0.846381785
CCDC80	0.007477409	6.613044995	0.846501341
PCDHA8	0.004840274	6.086150098	0.846524829
LOC653574	0.005074192	6.061007209	0.846723558
FGB	-0.003658316	6.138638834	0.846773142
LOC646074	0.004781565	6.402013113	0.84677579
LOC440438	0.005032884	6.277216865	0.846779619
FLJ42957	-0.004867738	6.005065174	0.846788374
TRMT2A	0.005325361	6.164013836	0.846852869
LOC653702	0.015115292	7.355330386	0.846941852
LOC652163	-0.004371323	6.022829215	0.846950963
GPR156	0.006881582	6.110128859	0.846971626
GRXCR1	0.004433188	6.012523699	0.846994073
VTA1	0.006459888	7.558827389	0.847047198
LOC648620	0.005652066	6.04242214	0.847051058
OVCH1	0.006667725	5.963279307	0.847076668
FUNDC2	-0.005003194	6.651100997	0.847091754
CDY1B	0.002490962	5.999469482	0.84720391
SPRR2E	-0.00350066	5.986814613	0.847220622
KBTBD2	-0.012915038	9.189607295	0.847319558
TMEM184A	0.005646544	6.166683877	0.84733138
LOC646521	0.005049424	6.332244198	0.847410939
SGCZ	-0.004835093	6.059327389	0.847473833
ZFATAS	0.003840585	5.956275518	0.847504696

LOC645694	0.004400784	5.958363106	0.847507705
LOC654220	0.004824436	6.164896891	0.84760626
LOC652300	-0.004689068	6.099684186	0.847617573
TAS2R46	0.005314782	6.013536773	0.84767155
RPL23	-0.015894424	11.23164351	0.847673006
LY6G6F	0.004823927	6.086686376	0.847679279
TTLL2	-0.003663883	6.003106658	0.847846384
SPINK5L3	0.004838854	6.076482193	0.847919709
LOC652450	-0.003586031	6.050571442	0.848002019
OR9Q2	0.005287718	5.956152378	0.848031526
C13ORF23	0.005421892	7.188497938	0.848032749
LOC653537	0.003693084	6.083991045	0.848063314
MVD	-0.007206429	6.716280006	0.848206004
LOC646750	-0.004806423	6.133683328	0.848313227
FAM92B	-0.006818875	5.937167077	0.848318105
CRCT1	0.005427754	6.081593624	0.848448946
OLAH	0.005575725	6.2240817	0.848458962
LOC653669	0.00458479	5.924533742	0.848538461
LOC388814	-0.00502952	6.168072208	0.848581582
LOC390530	-0.005493224	5.956726568	0.848590847
LOC642659	0.003947889	6.056297406	0.848624515
C16ORF52	-0.005214725	6.603119335	0.848660237
FLJ46020	-0.004558314	6.068595223	0.848666886
C9ORF11	-0.005094282	5.988025795	0.848667165
NFIC	-0.008618767	7.572640361	0.848678963
C1ORF138	0.004578848	6.0114654	0.848743955
BCKDHB	0.008601869	7.258736438	0.848850174
LOC645915	0.005066702	6.029986116	0.84894257
LOC643438	0.011175789	6.836569161	0.849031314
A2ML1	-0.004795735	5.970533135	0.849116756
LOC647015	0.00475902	6.007344787	0.849194425
TCEAL6	0.004668005	5.995989594	0.849203532
OR7C2	-0.005037592	6.167054269	0.849226583
LOC126860	0.005397071	5.987457254	0.849229761
ACSM1	0.004726956	5.947212737	0.849434399
SERPINA6	0.00502824	5.904244435	0.849435416
PTCD1	-0.008257904	8.561318326	0.849442283
LOC650628	-0.007266387	6.077447757	0.849450124
TPI1	0.008552374	12.15057548	0.849455127
MAN1C1	-0.010586159	7.69871368	0.849515643
RNF8	-0.005410081	6.941318816	0.849661926
LOC649783	-0.005575732	5.972524641	0.849708212
LOC648220	-0.005129573	6.038987442	0.849859544
LOC646400	-0.004842635	6.128203177	0.849991996
NTF5	0.005010247	6.072002445	0.850024605
LOC645737	-0.004949267	6.210391888	0.850048517
AFAR3	0.006049237	6.149681015	0.850082388
LOC652875	-0.004715543	5.99345162	0.850154504
LOC641975	0.006271361	6.201887361	0.850160668
LOC642384	0.004534711	6.331621811	0.850191267
LOC648173	-0.004988329	6.111700923	0.850340397

FLJ35740	-0.005836467	6.037237379	0.850387061
MMP12	0.003810197	5.946055325	0.85039959
DZIP1L	0.009225296	6.414711074	0.850441767
C14ORF101	0.005941011	6.176966219	0.850450222
LOC650843	-0.004840003	6.083425749	0.850457964
LOC644994	0.004489883	6.129190644	0.850516583
LOC642714	0.005290294	6.164943439	0.850663161
C2ORF44	-0.009027728	7.50578824	0.850675955
CLDN16	-0.003211003	6.104209091	0.850724836
BIN1	0.011806804	8.151747688	0.850726987
LOC440059	-0.004624119	6.030859336	0.850778433
LOC729485	0.004889354	6.206944707	0.850783271
OR2T27	0.004822771	6.011733704	0.850868891
LOC402377	-0.004606551	6.095575599	0.850936284
EGR4	0.005000083	6.072886474	0.850983651
ARSE	0.004472161	5.997045203	0.85099324
BRWD2	0.007493788	7.090672943	0.851004182
LOC653580	0.004815653	6.264124692	0.851045821
BLVRB	0.014892411	9.382787096	0.851094084
LOC649248	-0.004978461	6.12563098	0.851137969
LOC652771	-0.005714014	6.140323825	0.851243847
LOC644612	0.00515683	6.018080191	0.851248234
LOC389631	-0.004739161	5.973622987	0.851518221
KRT14	-0.00474661	6.007945879	0.851547347
MPHOSPH6	0.012452488	6.822552727	0.85167565
ARFGAP2	-0.009053	8.137001441	0.851758014
LOC644925	0.005310922	6.112646934	0.852097628
LOC646782	-0.004627832	6.045216925	0.852101594
LOC644143	-0.005321841	5.907714092	0.852115598
LOC149086	-0.004659596	6.250838511	0.852244442
SLITRK2	-0.005197859	6.034739306	0.852286375
TOMM70A	-0.00842159	10.64701649	0.852290789
LOC647806	0.004921607	6.060963233	0.852384814
WNT2	0.004912245	6.355149027	0.852410598
TMEM50B	-0.008185827	9.069983706	0.852526311
DEFB104A	0.004216423	6.043493413	0.852569091
EGFL6	0.003853248	6.003763953	0.85259862
AOX2P	0.004917421	6.175118123	0.852650153
SNORD31	-0.006568219	6.548499374	0.852655359
FAM111B	0.004897782	6.156485007	0.852706328
LOC649974	-0.004184581	6.142766441	0.852779447
ANKRD34B	-0.003881315	5.957088958	0.85278833
LOC645485	0.003412493	6.035146585	0.852871105
PVRL4	-0.004801592	6.002421939	0.852878128
FBXL19	0.006310788	6.103840172	0.852923737
KCNAB3	0.004798755	6.051393213	0.852971571
NUMBL	-0.008092462	6.085879352	0.852983963
LOC645325	0.004775317	6.041998304	0.853027533
LOC645066	-0.004689024	5.931469087	0.853302855
TULP2	0.005054421	5.921404832	0.853360597
SPAG6	-0.00353184	5.979164509	0.853425736

LOC653941	-0.004557753	6.05274733	0.853552007
FLT4	-0.002866685	6.026274464	0.853559365
PABPC4	0.011009044	11.32377283	0.85364155
FLJ90086	0.006949935	6.310205322	0.853805836
LOC644068	0.005067465	5.955629105	0.853998689
AQP11	0.006965762	6.436814618	0.854028224
LOC644420	-0.004777072	5.992745242	0.854105421
C7ORF16	0.004302811	6.118804363	0.854244486
LOC644957	-0.005079891	5.930889043	0.854280655
LOC643339	0.006388104	6.543964469	0.854364174
PMS2L11	0.005146298	6.274960434	0.85443362
GFI1B	-0.004725211	6.11585714	0.854477079
OR10A2	0.004492064	5.937795432	0.854489691
KPNA6	-0.009104068	9.439846278	0.854527802
AGMAT	0.003900897	6.057494115	0.854755148
LOC391013	-0.003008935	6.03841038	0.854841
LOC650845	-0.00530307	6.421164798	0.854846634
NR1I2	0.003582324	6.068260054	0.855117838
CTDSP2	-0.007557961	10.04800211	0.855126651
LOC641944	0.005390186	6.154634461	0.855159217
LOC651289	0.004428268	6.01691916	0.85521809
DLG3	-0.003129436	6.215153867	0.855293107
LOC732139	0.004185236	6.11969241	0.855302051
LOC730656	0.00492685	6.058962743	0.855303388
ADAM28	0.003307961	6.078779396	0.855381057
LOC144383	-0.004385983	6.005775568	0.855414724
LOC648176	0.005602378	6.629861962	0.855566737
LIN28B	0.005603575	5.948137243	0.855572501
LOC649503	-0.004340569	6.00671857	0.855626976
C1ORF84	-0.002371564	6.179733537	0.855641825
LOC645246	0.004691859	6.027793797	0.855648097
GPR141	0.003085876	6.072533222	0.855697712
TCP10L2	-0.004284831	6.053434363	0.855717221
ROBO4	0.006819748	6.631918492	0.855721249
LOC401934	-0.005448853	6.38107256	0.855744263
LOC391124	-0.004588481	6.048861263	0.855750976
MYLPF	0.004178072	5.998827647	0.855798845
LOC643420	0.004971067	6.147896046	0.855807444
LOC648454	0.004556296	6.062575342	0.855834895
ABCB8	-0.00572474	6.18090484	0.855940415
SGOL1	0.003682975	6.072515966	0.856002906
LOC133993	-0.002824938	6.104583861	0.856054899
RRM2	0.005382721	6.074976306	0.856136689
QRFP	0.005352163	5.946997821	0.856220304
NHLH1	-0.005143449	6.096362638	0.856302505
FLJ41649	0.005442688	5.990652561	0.856548789
OPRM1	-0.002158142	6.093149819	0.856558616
GTF2I	-0.004811398	6.33844946	0.856694077
LOC649366	-0.005297895	5.953866708	0.856766341
C1ORF62	-0.003234487	6.057748268	0.856828257
XRCC3	0.004552589	6.294878012	0.856834433

IFNA13	-0.00280703	6.022840331	0.856857521
LOC646780	0.004823721	6.141507801	0.856881423
KCNE2	0.004185866	6.026468098	0.856965594
PRSS2	-0.005916513	6.034280976	0.85698956
MRPL22	-0.006798692	10.44309026	0.857003412
OVOL2	-0.004879785	6.027934453	0.857050874
LOC650454	0.003912639	6.056485047	0.857064211
MST1R	0.004895099	6.096356637	0.857089501
LOC647716	-0.00483696	5.951676006	0.857113216
DKFZP686E2433	0.0056709	6.235583063	0.857171278
TMEM118	-0.006561977	6.593302209	0.857182691
LOC643465	0.004256415	6.0404571	0.857280027
LOC641841	0.004562578	6.037328154	0.857431811
HOXC12	-0.005447908	5.895007313	0.857487636
ACTN3	-0.004155117	6.051937049	0.857491588
LOC643932	0.005294258	6.505874972	0.857549273
FOXJ3	0.008621182	9.69334512	0.857751304
LOC643368	-0.004666696	6.177792803	0.857908874
ZKSCAN1	0.012151326	6.662369913	0.857987682
CDC16	0.005857764	9.857482458	0.857989844
LOC651680	0.005731836	6.335165655	0.858038459
EMID1	0.005027731	6.053619854	0.858087752
PHEX	-0.00506576	6.068615528	0.858099414
LOC732387	0.003704706	6.027779306	0.858265977
KIAA1920	-0.004951051	6.160064994	0.858290797
ARF6	0.007412498	6.156148479	0.858291351
LOC389405	0.004209245	6.122506379	0.858334762
LOC728080	0.004229102	6.112310437	0.858357322
SPINK4	-0.004224882	5.951187337	0.858447556
WFDC9	-0.004299085	6.006931045	0.858511881
LOC651513	-0.004500777	5.961519787	0.858617354
LOC653429	0.003990559	6.037592345	0.858696004
FAM54A	-0.003236095	6.13565148	0.858704207
LOC728216	-0.007881551	6.220365975	0.858773555
LOC389396	-0.004530981	5.980742973	0.858774118
PIGG	0.005652987	6.573182712	0.859134555
WAC	-0.008000349	9.241678395	0.859204527
NYX	0.004128717	6.052736287	0.8592465
EDG7	-0.005508386	6.2461509	0.859310572
LOC641958	-0.004376408	6.016201018	0.859536407
NOVA2	0.005651806	6.225685088	0.859561979
B3GALNT2	-0.006017957	6.804567578	0.859615162
GPR101	0.004274399	6.228974464	0.859737521
SYT12	-0.004399024	6.184230435	0.859793228
LOC649500	-0.004853161	6.16481656	0.859804752
RPL23A	-0.009630793	7.475880583	0.859810802
SLC34A1	-0.006256837	6.104361594	0.859815321
SNORD107	-0.002826366	5.982586343	0.859832539
PSMB7	-0.007517732	11.44542839	0.8598728
DNAJC17	0.00492715	7.159610997	0.8600277
OR1F1	0.004014596	5.985045976	0.860099208

LOC652554	0.004139931	6.011656197	0.860191758
EIF4EBP2	-0.007694793	8.118209952	0.860277073
LOC652842	0.004540378	6.03084065	0.860477227
LOC390667	0.004546473	6.187326888	0.860578602
LOC158572	-0.005606662	6.381189577	0.860627941
LOC647881	0.00295073	6.008117739	0.860630707
CUBN	0.005103661	6.140736956	0.860666545
MS4A12	0.00454112	6.039828349	0.860708238
COPG	-0.009054683	8.912866088	0.860730789
ZNF639	0.004360376	6.137271376	0.860759381
FGF22	-0.004614309	5.944447248	0.860770498
LOC650841	-0.004460369	6.071006434	0.860807305
TAS2R42	0.003681971	5.977756393	0.860856887
LOC152742	0.004098696	5.992577571	0.860859581
PGF	-0.010072343	6.5705022	0.861012888
LOC645446	-0.004775727	6.117410636	0.861020562
MGC27165	0.004518594	6.025460338	0.861087817
C4ORF8	0.006778243	7.437103887	0.861124345
DEPDC1	-0.003274469	5.966099614	0.861200314
ELAC1	-0.004502716	6.264878573	0.861211372
LOC653293	0.003376508	6.096995507	0.861212188
LOC643409	0.004458427	6.041534932	0.86133185
SFTPA1	0.004773586	6.026955	0.861403146
IMP5	0.005542723	6.152580558	0.861468682
CAMK1G	0.005800149	6.195192602	0.861476569
FOXD4L2	-0.002543135	5.998335009	0.861486281
GPR119	0.005184715	6.716534142	0.861637424
HSFY1	0.003088323	5.999702907	0.861693763
LOC401589	-0.003051351	6.193110115	0.861746929
FLJ35424	-0.002490485	6.162627243	0.861804783
KRT85	-0.004200941	6.091729328	0.861894043
LOC644288	-0.00408023	5.904388067	0.86195849
TRDN	0.005481952	6.39285342	0.861971277
LOC645442	-0.004672081	6.120548967	0.861979388
OTOP1	0.004725888	6.226824916	0.862146179
OR10A3	0.004578933	5.937970891	0.86217425
FLJ10120	0.004443102	6.149650849	0.862236511
CRYBB2	-0.013005049	6.624097212	0.862319835
KPRP	0.004192047	6.050434025	0.862361453
LOC644246	0.004620242	5.959347104	0.862498396
UBA2	0.008552714	6.301378002	0.862516004
C19ORF15	0.004018769	6.154250062	0.86252249
PROP1	0.005396199	6.148501817	0.862575247
ST7OT4	-0.004404962	5.986489484	0.862667424
LOC440422	-0.00317532	6.036208343	0.862748101
SLC27A4	0.008636381	5.982347688	0.862900947
C21ORF114	0.004059362	5.990295756	0.862926523
LOC645167	0.004251848	6.270168714	0.862957949
PCDHGB8P	-0.004156725	6.052762864	0.863025182
LOC653866	0.002851121	6.000775388	0.863052137
TOE1	0.004193187	6.378745039	0.863090109

LOC645850	0.00436343	6.075067286	0.863130939
LOC642165	-0.004609401	6.061659174	0.863428197
LOC653137	0.004872733	6.43502665	0.863765271
LOC651537	-0.004164187	6.060620768	0.863771572
RYK	0.005592343	8.910184928	0.863859899
ACBD7	0.003383756	5.966304659	0.863873346
LOC644342	-0.004040009	5.964224126	0.863884005
ACTL7B	0.005041211	6.193894099	0.863890163
LOC652840	0.00616886	5.971516333	0.863957878
C14ORF162	-0.005100908	6.096167405	0.864056493
LOC441662	0.003459614	6.189718765	0.864094535
USP37	0.006073289	7.100922924	0.864293849
LOC644943	-0.004166805	6.094543423	0.864381882
LOC653796	-0.004426305	6.213455184	0.864547611
LOC650687	-0.004462287	5.961942824	0.864612079
LOC339766	-0.004325481	6.110571174	0.864646605
EPHA10	-0.004084249	6.35550902	0.864655447
AHCYL1	0.010166398	9.654900229	0.864715655
DHX38	-0.007894228	7.395507206	0.864724365
LOC652544	-0.004856211	5.912043078	0.86493888
UQCRC1	-0.011777734	11.69052708	0.8649391
SLC25A37	0.008586404	8.106417457	0.865052374
PHACS	-0.006280673	6.599413568	0.865052921
LOC284379	0.003249231	6.077470392	0.865100833
C15ORF39	-0.006710659	6.945367465	0.865120056
LOC391703	-0.004509415	6.193550674	0.865138062
ATP6V1C2	-0.003346021	5.985344082	0.865148696
STEAP2	-0.008845759	6.749943233	0.865198365
FDPSL2A	0.004711873	6.157456019	0.865211703
CDH18	-0.004056615	5.857597287	0.865259154
HTR1A	-0.00494522	5.999453589	0.865532583
LOC642377	-0.004655696	6.480863161	0.865900558
LOC647123	0.00423853	6.082017093	0.865907504
CAPSL	-0.002547907	6.080782821	0.865969626
CBX8	0.004991375	6.439883472	0.865986135
BANF2	-0.004998822	6.14588696	0.866014135
INTS5	-0.005524371	7.214721999	0.866022588
TP53BP1	0.009334549	7.917771091	0.86609973
ALLC	0.002599858	6.053680786	0.86610751
OR52J3	-0.004110427	6.011187614	0.866229535
LOC392979	0.004296541	6.072208738	0.866266997
DDX3Y	-0.006597724	6.050763399	0.866269032
LOC654046	0.004724405	6.087637477	0.866315012
FMO1	-0.006642227	6.285203528	0.866377887
SPTBN4	-0.003082036	6.162290104	0.86640517
OR10G2	-0.004242445	6.058739686	0.866524965
GEFT	0.003186988	6.155905534	0.866535685
DNTTIP2	0.007371085	6.235145361	0.867243457
LPHN2	-0.011075207	8.260220109	0.867385326
LOC654078	0.00672528	6.057706854	0.867405513
FAM35A	0.004238356	6.615776681	0.867418402

SHANK2	0.002788233	6.046274814	0.867435034
HOXB9	-0.004382267	5.970641878	0.867490548
ZFPM1	0.009157448	7.696530803	0.867517661
LOC732300	0.004184797	6.266342469	0.867655072
CHI3L1	0.004257268	6.029959167	0.867680166
LOC649429	-0.004660615	6.0746424	0.867876795
LOC340529	-0.004133724	6.004932547	0.867963791
KRTAP9-4	-0.002941776	6.128796364	0.868038182
FAM159A	-0.003933326	6.063874956	0.868055078
LOC650103	0.003745787	5.943740133	0.868062062
DBX2	-0.003607141	6.078417312	0.868083059
LOC653981	-0.004335031	6.302471257	0.86816217
PPP5C	-0.006352371	6.136059156	0.868347069
ATP5B	-0.007665924	14.65937991	0.86868243
LOC653569	-0.005318143	6.044930495	0.868762482
TEKT1	0.005020193	6.205546812	0.868927263
KIAA1967	0.006423841	6.854732866	0.868937826
CCDC135	-0.003318888	6.368805977	0.868989609
DUS2L	-0.006119522	7.608739499	0.869141457
CXCL14	-0.007834161	6.505035595	0.869181938
LOC650024	-0.004459981	6.047284562	0.869242475
LOC651177	-0.004128858	5.928801603	0.869429452
RNF168	-0.004238326	6.172291979	0.869501034
CLCA1	-0.004543258	5.93100491	0.869643547
LOC652587	-0.003793498	5.9671253	0.869738616
OR9K2	0.004120999	5.969090654	0.869848616
LBP	-0.005422069	6.04817602	0.869913038
LOC651453	-0.009347973	7.118220727	0.869989167
PSMG1	-0.008823036	8.184837979	0.870189731
LRRC37A2	-0.004244011	5.941392162	0.870201392
KCNE1L	0.004888689	6.144041392	0.870215113
LOC647683	-0.004176031	5.88251388	0.870256525
ISL2	0.006134871	6.140617983	0.870346511
LOC729692	0.006090873	6.414999368	0.87046191
KLHDC7A	0.004532844	6.213210444	0.870480428
LOC651116	0.003472047	6.054811954	0.87052143
COPB1	0.006775529	9.091238533	0.870613204
PARD6G	0.002738734	6.142651961	0.870646609
LOC645876	0.004084508	5.932020778	0.870753505
LYZL6	0.004288674	6.238961795	0.870873186
LOC649120	0.005216021	6.001645656	0.871044688
NAV3	0.006891498	6.546520333	0.871202102
SYT6	-0.004012174	6.031602371	0.871227929
GRHL3	-0.002254708	6.022837227	0.871249578
ABHD7	0.007799016	6.250488294	0.871345298
DLC1	-0.010206777	7.581986244	0.87135458
LOC653140	0.00384621	6.051727087	0.871361465
LOC652755	0.010987365	6.582078389	0.871387124
LRFN4	-0.007298913	6.671885143	0.871423494
LOC651403	-0.004769665	5.953748139	0.871488756
TTC9B	-0.004410293	6.025971046	0.871553752

LOC647074	0.005849424	6.163510606	0.871576657
CD80	-0.004053775	6.063426465	0.871639868
LOC400099	0.004112959	6.112002051	0.871733789
LOC652859	0.004926437	6.017586279	0.871837548
LOC652519	0.003520929	6.035959833	0.872042659
LMF1	0.004652543	6.286246729	0.872054425
LOC644472	0.005606925	5.96949774	0.872067632
DUB3	0.004394969	6.025920038	0.872225994
LOC646638	-0.003957344	6.043659694	0.872362209
LOC647624	-0.004111792	6.224048502	0.872465446
HNF4G	0.003992279	6.085600998	0.872714443
PNMA5	-0.003970223	5.891297149	0.872819455
LOC728626	-0.004205559	6.032468105	0.872821861
EYA4	0.007376917	6.682926228	0.872907784
HIST1H3C	0.005139754	6.400187272	0.87291146
LOC643731	0.003791237	6.058474858	0.872944356
LOC647243	-0.004212989	6.43816461	0.872944554
UGT1A3	0.005169235	6.084158937	0.873110501
LOC649137	0.003683392	6.024330714	0.873191491
LOC391742	0.004545065	6.218765185	0.873219737
CYP2D7P1	-0.002860763	6.243258135	0.873267909
LOC642005	-0.003719804	5.979966292	0.873345059
LOC220594	0.0036552	5.895530405	0.87341175
C10ORF85	0.004469225	6.25222362	0.873432014
LOC642011	-0.004153952	5.969928147	0.873493149
MGC16121	-0.008934065	6.885303319	0.87362129
LOC441752	0.004048221	6.050004492	0.873622115
OR6T1	-0.003816779	5.948794523	0.873840625
FAM125B	0.004062606	6.648526126	0.873920951
LOC652734	-0.005772295	6.112921287	0.873946675
LOC648354	0.003490825	6.066362149	0.874004238
LOC642164	0.003636143	5.97023767	0.87403751
TEX14	0.002989988	5.988684428	0.874045958
CBFA2T3	-0.003276204	6.148274537	0.874081652
LOC650520	0.004481823	6.013194203	0.874136442
MAGEA1	0.003343899	6.092690498	0.87416171
LOC644641	0.004412813	6.179889426	0.874219153
LOC650656	-0.003743068	6.061531825	0.874332403
LOC645410	-0.004553281	5.873805566	0.87448359
TM4SF4	-0.004161326	6.003059829	0.874505505
ITM2B	0.009175952	10.97292032	0.874562971
AP4E1	0.0073331	7.791022821	0.874727901
FAAH	-0.004678783	6.155183685	0.874750696
SIGLEC6	-0.003034234	6.155110029	0.874855302
LOC649182	-0.004454661	6.203834369	0.874859324
LOC652003	0.00496693	6.041286499	0.874938845
FLJ45139	0.004066042	6.003286728	0.874941855
IL27	0.004068246	5.985899513	0.875014731
FLJ45032	-0.002721897	6.052867187	0.875139295
LOC643062	0.004216289	5.999621367	0.875154379
LILRA1	-0.004069341	6.094897694	0.875201275

FLJ22447	-0.002508164	6.064150898	0.875228197
RAB31	0.012083574	9.751606103	0.875256586
KIAA1274	0.007939821	6.087196469	0.875262195
LOC652436	-0.003649655	6.041703625	0.875269958
DGKE	-0.004368961	5.952157376	0.875369174
CLDN10	-0.003060718	6.098594202	0.875436666
FAM111A	-0.00621504	7.618148223	0.875440341
CREBL2	-0.006574222	6.138632949	0.875516481
UNC93A	0.003768832	6.089544197	0.875530903
RUTBC2	0.004184729	6.079370637	0.87559804
LOC644929	-0.004674471	6.222961629	0.875648937
LOC649201	0.003626888	5.950825459	0.875732311
CNKSR2	0.003926538	6.262328209	0.875818117
CACNA1A	-0.0029985	5.975320235	0.875885391
LOC650074	0.003790705	6.03837036	0.875967968
PSMC2	-0.004395026	10.80546887	0.875984548
LOC642672	-0.00397925	6.092305607	0.876024153
LOC651520	0.004016112	6.340116046	0.876034163
MOS	0.005551733	6.067353762	0.876066843
LOC648058	0.003622198	6.222362151	0.876372355
LOC732157	0.003609425	6.236037592	0.87642141
LOC388692	-0.00519175	6.076872118	0.876616938
LOC644489	-0.003795281	5.953228814	0.876630504
RNF186	0.005230408	6.064785405	0.876632407
UPF1	0.007176419	7.579766254	0.876663309
NY-REN-7	-0.004876831	6.499079746	0.876863341
TEAD3	-0.006677904	6.718664229	0.87689464
PA2G4P4	0.006711441	6.029333813	0.876916991
DDX25	0.003027992	6.033097125	0.876919157
LOC400509	0.004224338	5.987808762	0.876951626
ZNF273	0.003051363	6.061555977	0.877019026
GPD2	-0.002752536	6.053957683	0.877082634
EPHB6	0.004201492	6.202229345	0.877297205
LOC643767	-0.004396655	6.034827016	0.877357521
OR2AK2	-0.005038293	6.090639827	0.877377804
AMPD2	0.003139757	5.988417538	0.877440305
DSC3	0.003288152	6.067971961	0.877570943
LOC643501	-0.003599788	6.04871733	0.87762994
OR10T2	0.003813742	6.008880938	0.877676654
NR1D1	-0.004219784	6.222631252	0.87778775
USPL1	-0.006188002	7.653995098	0.877841106
LOC650889	-0.003970521	6.251031877	0.877918517
FLJ10404	0.003171231	6.288537199	0.878096915
LOC401399	0.00345289	5.997478263	0.878134444
LOC653711	0.004491102	6.354988132	0.878147885
LOC729252	0.003812852	6.24972447	0.878268959
LOC643857	0.004148956	6.135371957	0.87830392
LOC653665	-0.004057861	5.90753222	0.878325305
C7ORF60	0.004208158	5.969119416	0.878327438
NBL1	0.013093623	9.245524039	0.878359998
ITFG3	0.009665902	9.698691236	0.878372075

WIPF2	-0.004293271	6.825670877	0.878394811
AQP3	-0.005629778	5.922797536	0.878435947
TNIP3	0.004052698	6.097960922	0.878496163
C20ORF144	-0.003815174	5.892383472	0.878528006
PRH1	-0.002893218	6.266213708	0.87858672
LOC653148	-0.004115652	5.974260235	0.878665862
CACNB1	0.003419328	6.250494549	0.878726701
LOC652657	-0.005087051	6.117786942	0.878762965
RGS12	-0.002493219	6.56120342	0.878781953
C18ORF24	0.004281592	6.2548947	0.878817938
RPS24	0.011751081	11.29675862	0.878866441
LOC644444	0.004119638	5.949796387	0.878879429
LOC652215	-0.004573428	5.820908035	0.878903512
LOC641724	0.004425359	6.196793756	0.878961873
ZNF282	0.005355505	7.319229717	0.879062682
FUT1	0.0051333	6.176067763	0.879190535
HOXA3	-0.002089389	6.029215646	0.879199998
LOC644567	0.002934113	6.043377865	0.879214084
CCDC140	0.006149227	7.089997207	0.879416357
PBX3	-0.010473884	10.73631832	0.879451607
OR4Q3	0.00267021	6.006508145	0.879499751
LOC644512	0.003450835	5.962298638	0.879535657
LOC642691	-0.004090336	6.329092838	0.879664467
WNT3A	-0.004093498	5.99271775	0.879729601
LOC541469	-0.005607522	6.154609038	0.879832008
IFNA7	0.003237559	6.06075031	0.879843244
FLJ44635	-0.003143929	6.062895585	0.879874949
USP50	0.003505722	5.941939085	0.879884164
ZFYVE19	0.006650561	7.924971759	0.88002317
LOC284100	0.003484536	6.102378005	0.880061125
CPSF3L	-0.00532498	7.436578067	0.880157781
RBPJL	-0.003779868	6.163975059	0.880162516
ZNF600	-0.008093703	6.898163853	0.880168945
LOC122038	-0.003783275	6.005493787	0.880399482
STMN2	0.003224406	6.370888987	0.880401587
LOC440258	-0.004249969	6.307019897	0.880420175
CABP2	-0.002416888	6.112962942	0.880481302
LOC651610	0.003579383	6.071264175	0.880579189
MKLN1	-0.006424142	9.646669901	0.880739946
LOC653930	-0.002360083	6.133305039	0.880891467
KLK3	-0.002532542	5.982674851	0.880961434
LOC642248	-0.003440166	6.032248084	0.881114805
LOC645492	-0.003737298	6.154957486	0.881295703
LOC649501	0.003417652	6.144874794	0.881319558
P53AIP1	-0.002848338	5.997645834	0.881365345
GALNT14	0.004350705	6.456489022	0.881440371
LOC341356	-0.003735782	5.984872535	0.881493777
SDC3	-0.002609149	6.240547211	0.881504359
FSTL3	-0.017444403	9.095332167	0.881546094
MAPK15	0.00437544	6.236171802	0.881713211
CD180	0.003965302	6.093444424	0.881843819

GM127	0.004385678	6.127334672	0.881847728
LOC647810	-0.004469862	5.993618676	0.881859338
LOC728181	-0.005228931	6.129331945	0.881862619
ZNF678	-0.005471306	5.819936401	0.881901947
TRPV3	-0.004226134	6.214948918	0.881995164
FAM22C	0.003637397	6.049111618	0.882013779
LOC643234	-0.003709891	6.109464067	0.882029699
FLJ42280	0.004342121	6.008347401	0.882052935
LOC149620	0.003742942	6.200200118	0.882326241
DCDC2B	0.003984792	5.954023971	0.882354393
MRPL30	-0.005109162	7.176092577	0.88239276
CDCA5	-0.005067782	6.506671528	0.882470294
KCNK2	0.00200857	6.027577266	0.88258203
FLJ30430	-0.003918002	6.171997834	0.882633534
SPC25	-0.002871868	6.040149586	0.882636874
CDCA1	-0.004468506	5.99457436	0.88265441
LOC645565	0.004226	5.934748421	0.882655848
LOC643296	-0.003775302	5.978022415	0.882672659
BARD1	0.006799169	6.845408169	0.882688271
LOC643648	-0.003743707	6.163261978	0.882691115
ABCG8	0.003486556	6.05380852	0.882748678
OR8H2	0.002654276	5.986215549	0.882756822
FLJ20397	0.001957156	6.080663433	0.882842271
EGFL8	-0.008572092	7.505912111	0.882953706
IHPK2	-0.004119763	6.666156551	0.882969656
C9ORF89	0.006354765	8.080313772	0.882978225
SNORD73A	-0.004000723	6.181257646	0.883072289
POU2AF1	-0.003689133	6.162860894	0.883148901
LOC651830	-0.004206599	5.954627781	0.883168978
LOC647647	0.003393756	6.047778576	0.88319375
CMTM1	-0.002688952	6.06653271	0.883227146
LOC646193	0.003644891	5.948808276	0.883242672
LOC340204	0.004166165	6.036437461	0.883263981
TMEM169	0.003511302	6.125843794	0.883402495
LOC641983	-0.002968849	6.127470455	0.883524373
C9ORF38	-0.004391872	6.199094274	0.883574889
ZNF485	0.003977554	6.168359156	0.88358805
CHRDL1	-0.002715004	6.004611346	0.883623418
LOC163233	0.002529404	6.105844391	0.883644459
TLR7	-0.005784026	6.370977057	0.88365448
LOC653431	0.004744977	5.868784431	0.883766218
ZNF496	0.004224699	6.083952633	0.883883831
LOC646209	0.003854381	6.065803305	0.883888146
ATG2B	0.004522697	6.876495871	0.883888244
C2ORF83	-0.003595484	6.060534925	0.883933838
GRID1	0.003607741	6.002774872	0.883981032
KIAA1755	0.00500591	6.060672908	0.884059304
LOC644599	-0.003790053	6.231506225	0.884104691
LOC653720	-0.00482158	6.545994927	0.884305645
LOC652335	0.003162979	6.121980979	0.884338805
BRI3	0.006992603	10.73656034	0.884348862

LIN7A	0.005864302	6.592730728	0.8844022
BTBD16	0.003914713	6.231503116	0.884623668
LOC402457	-0.00347464	6.064969534	0.884766444
BRUNOL4	-0.002034149	6.070888469	0.884775794
P2RY12	-0.003860039	6.137727113	0.884776886
CRTAP	0.005579734	7.338591158	0.884781366
PIPOX	0.003937059	5.992598982	0.884793791
LOC650187	-0.00353057	5.995358475	0.884868685
LOC729096	0.003586072	6.062979948	0.884988461
IFIT3	0.011644633	7.000682049	0.885117085
LOC642936	-0.003568383	6.234843198	0.885361065
C19ORF46	0.003952186	6.05144935	0.885361878
LOC643640	0.003950693	6.07428393	0.885467126
GPR109A	0.004424737	5.940317126	0.885512376
LOC641943	0.003725164	6.054759045	0.885526761
LOC642255	-0.003462094	6.817333837	0.885637318
TMEM16J	-0.003362597	5.974879216	0.885649695
PHLDA3	-0.007327394	7.112871887	0.886009214
SEBOX	0.003315375	6.194166155	0.886034619
FUT10	0.00250786	6.123758282	0.886046397
LOC728369	-0.002357136	6.125927385	0.88604936
LOC149069	0.003621297	5.98016073	0.886086334
CSMD3	0.002328342	5.9891906	0.886088558
HSP90AB2P	0.002912477	6.147937814	0.886104199
LOC390748	-0.002262619	6.1123136	0.886205227
OR8D1	0.003554094	6.054346079	0.886275839
DSCR4	-0.004317156	6.066200387	0.88630313
FUT2	-0.004019432	5.764011531	0.886360156
LOC652173	-0.003541796	6.083789398	0.886395869
WASPIP	-0.007137163	7.277068958	0.886409513
GAPDH	0.005003363	12.89753697	0.886437506
ATP6V0D2	0.003719758	6.232996914	0.886511466
CFHR1	0.004522169	6.163327148	0.886532354
LOC653043	0.003174203	6.118129215	0.886632026
LOC728185	0.004019417	5.818778632	0.88670286
LOC647788	0.003786495	6.057756084	0.886715453
LOC653192	0.003428302	6.072505357	0.886724771
LOC283029	-0.003518478	6.075066872	0.886810641
LOC651166	-0.00346376	6.413191537	0.886925475
MMRN1	0.00855348	6.36726701	0.886946082
CREBL1	-0.004640086	6.326921453	0.887048901
SYNCRIP	0.007569091	9.227753361	0.887101221
WDR42C	0.003190594	6.007273593	0.887125082
WDR76	0.003236589	5.958694507	0.887162842
DIRC2	-0.006275175	9.16450335	0.887295976
LOC646853	-0.004062878	6.489923372	0.88782476
RRAGA	0.005854487	11.4942548	0.887904021
HMGA2	0.002281283	6.138260039	0.887959906
CTNS	0.005021499	6.891592107	0.887987176
LOC652877	0.003714659	6.09830672	0.88816166
FAM91A2	0.002336777	6.086849124	0.888395344

FAM12A	-0.00404033	6.521167612	0.8884621
CCNK	0.006241659	8.610318806	0.888508491
SNAI3	0.004196686	6.192813752	0.888549194
FBXW10	-0.003677831	6.019722711	0.888554934
NKAIN1	-0.003655637	6.064151049	0.888587873
SOX30	0.002903056	6.093044116	0.888636486
SIDT2	-0.010256027	8.889891639	0.888644017
GABRA2	0.003510256	6.021357747	0.888679667
C21ORF49	-0.004456319	5.968395498	0.888746958
STAC	0.004287204	6.170317776	0.888862373
RHBDD2	0.005995935	9.479593246	0.88900525
RBM12	-0.004219125	7.783029	0.889027177
PAX9	-0.004027423	6.178453796	0.889080762
LOC653686	0.003825447	5.931448227	0.88910517
SPINT1	0.002871775	6.0078993	0.889122188
PSMD11	-0.006481966	6.731728883	0.889152947
BPIL1	0.003754613	5.932545036	0.889310211
OAS2	-0.009904429	7.327663051	0.889353127
ABCA12	0.003246036	6.138866685	0.889450263
LOC651189	0.003558562	5.911928669	0.889457057
LOC389722	-0.003492843	6.115863283	0.889466006
LOC645986	-0.003638924	6.055195707	0.889490887
INSL6	-0.002505642	6.043871209	0.889575537
CCDC61	0.005631746	6.161720564	0.889581795
RFPL2	-0.003857688	5.927891287	0.889589262
LOC646331	0.003613108	6.077457467	0.889619428
FAM152A	-0.005149116	8.660383232	0.889716562
LOC650087	0.003684868	6.206990076	0.889835547
PGBD1	-0.004911227	6.745453811	0.88990065
ACCN3	0.003371082	6.607101243	0.889912453
LOC650977	0.003409395	6.036615691	0.889914497
FAM9B	-0.002512168	5.994678951	0.889919084
LOC645468	0.003254729	5.91125347	0.889954506
LOC646050	0.003565344	6.001329826	0.889957522
LOC645464	-0.005089371	6.487585141	0.890002463
OR52A4	0.003614595	6.02249667	0.890011497
LOC645687	0.003993017	5.975085409	0.89008489
LOC652655	-0.003493455	5.996450274	0.890422618
ZFYVE9	-0.001894361	6.065027356	0.890548701
LOC642214	-0.003305307	6.060825204	0.890552873
LOC340260	-0.003160262	5.994396827	0.89063838
LOC643712	0.003953087	6.375075172	0.890710192
OR1B1	0.003335984	6.047043647	0.890836955
SUSD5	-0.004798323	6.326688535	0.890852433
LOC642553	0.003459226	6.234307012	0.890922233
LOC651907	0.003439055	6.037837892	0.890940467
LOC649432	-0.002827684	6.053366754	0.890984903
LOC642244	-0.003550514	6.053143433	0.890998451
FLVCR1	-0.0059913	6.867826129	0.891270297
LOC401845	0.003376289	6.127225309	0.891274237
LOC732393	0.002191563	6.077584375	0.891296651

TINAGL1	-0.007542269	7.272697966	0.891333488
DYNLRB2	-0.00419114	6.541761171	0.891356392
B4GALNT3	0.004633137	5.903014365	0.891377322
TTTY16	-0.003465886	5.978613125	0.891429397
ADCY6	0.009724484	8.685094568	0.891448955
LOC652101	0.00313616	5.9919242	0.891457805
SIRPB2	0.002846789	5.988508772	0.891470922
VSTM1	-0.003444224	6.031647007	0.891480166
ESSPL	0.003025971	5.920835839	0.891502796
LOC728343	0.00186362	6.087556943	0.891513167
ANKHD1-EIF4EBP3	0.004572507	6.551921039	0.891731476
C9ORF128	0.003561567	6.146528676	0.891756319
POU4F1	0.003080345	5.989239235	0.891823997
ADAMTS2	0.002891298	6.130845624	0.89190345
MED12L	0.003453077	5.966647433	0.89192651
PRDX5	-0.005031634	12.31748172	0.89193649
LOC401387	0.003713295	5.91426801	0.891976698
ANGPT4	0.003653209	5.982506532	0.891982463
LOC653464	0.002551302	6.167773902	0.892159508
LOC646178	-0.0041169	6.516967241	0.892198048
CTSW	0.003696816	6.296422885	0.892262629
MS4A5	-0.004289178	5.956875017	0.892478487
SLC7A13	0.003447951	5.944877311	0.892489506
TAS1R2	0.002502264	6.052738945	0.89249952
LOC646146	-0.004221174	6.010683406	0.892552422
FLJ20850	0.004451162	6.166088901	0.892562726
CNGA4	-0.002663117	6.063300765	0.892582038
LOC121006	-0.002626624	6.095623357	0.892640666
LOC652733	0.004729756	6.297559544	0.892647739
AP1S3	0.003508866	5.898704002	0.892662198
MYH2	-0.003586705	5.95325881	0.892694951
MBNL1	0.00623184	8.915563073	0.892834254
LIN7C	0.005911387	6.436878433	0.892841653
LOC647862	0.003526823	6.108405392	0.893030676
MAS1	-0.003212756	6.044279688	0.893060168
LOC643968	0.0031423	5.960660752	0.893248715
NLN	0.004328999	6.433315728	0.893316215
LOC286177	0.00322212	5.954256799	0.893453031
MAGEB2	0.002697069	5.964978513	0.893583844
RPGRIP1L	-0.003717166	6.168564556	0.893682585
LOC645203	-0.003437322	5.95523396	0.893801173
HIST1H1T	0.003036535	6.077741658	0.893815362
LOC651100	-0.00317154	6.054531284	0.893896737
PLEKHG6	0.003204911	6.052178875	0.893906918
LOC652629	0.00279234	6.021765892	0.894004649
CDC25C	-0.001943304	6.097564623	0.894183153
ARHGAP22	0.006102735	6.915538391	0.89423228
OR2T33	-0.003111035	6.239525885	0.894455902
LOC649470	0.003277103	5.988255491	0.89451752
PPL	-0.004583571	6.484901908	0.894525027
GDA	0.002416834	6.031011791	0.894559282

UNQ9438	0.003656143	5.985380017	0.894705671
ASB2	0.006918905	7.0130871	0.894782358
LOC652258	-0.003141215	6.044859175	0.894785952
KRTAP5-1	0.004121485	6.12409555	0.894793707
LOC729351	0.003671385	6.058836801	0.894845804
HAPLN4	-0.003785894	6.255154373	0.894938122
TCP10	0.001899652	6.022724113	0.895019195
DNA2	-0.003410529	6.654097967	0.895130257
LOC652625	-0.00308388	5.999210964	0.895141662
PRKD3	-0.004206785	6.253787508	0.895169814
KIAA0409	-0.005991131	7.818287398	0.895188906
LOC729162	-0.003419905	6.256484203	0.895189092
MCTP2	0.003580222	5.927450809	0.895257266
LOC648672	-0.003781211	6.037737481	0.895284617
ACR	0.003377209	6.336742195	0.895344552
LOC648815	-0.005779971	6.019528589	0.895526909
LOC374443	0.003382475	6.148446673	0.895541381
SRC	-0.00282952	6.441437804	0.895587792
ZMYM6	-0.004344148	7.708441442	0.895613518
NHSL2	0.003359192	6.120243184	0.895665599
H6PD	-0.002958203	6.251648212	0.895790226
FABP5L3	0.003349864	6.150347563	0.895929929
DDX43	0.004086937	6.098111412	0.895949759
LOC342541	0.003123518	6.033169733	0.896280295
CDH26	0.002057517	6.149174227	0.896358042
FLJ46906	0.004560669	6.333430787	0.896579992
UBE2G2	0.008399566	8.695791003	0.896587098
C4ORF15	0.0031736	6.19107717	0.896637093
LOC650744	0.003586985	5.957552833	0.896666477
NAT8B	0.00351071	6.198205039	0.896707145
SLC25A41	0.003101866	5.992686649	0.896828454
TARP	-0.003333955	6.043780465	0.896838399
GABRG2	0.002371716	6.024522581	0.896861697
PRKCABP	-0.004971564	6.793160422	0.896911283
LOC647082	-0.003172044	5.84490396	0.896927101
GUCA2A	0.003725734	6.169177904	0.89700532
LOC644055	0.002955987	6.255627408	0.897080826
PPP2R4	-0.00627089	8.491766488	0.897146273
GEMIN4	0.005229688	8.700111094	0.897228344
YTHDC2	0.004023405	6.593637316	0.897373106
HRH3	-0.003744625	6.028365862	0.897409301
MAFA	-0.009544035	7.303342571	0.897412035
LOC643566	0.00349035	6.015469876	0.897454303
GRM7	0.001972483	6.019105747	0.897468311
LOC642976	-0.002910828	5.993604551	0.897516506
MBD3L1	-0.003200744	5.956648884	0.897557368
GNGT1	0.004021133	6.002197793	0.897562068
LOC642009	-0.002825014	5.959505294	0.897590821
SNX24	-0.005380586	6.820909256	0.89760873
ATP8A1	0.002912063	6.200767705	0.897704633
PLCB3	-0.002917258	6.166521898	0.897732091

LOC285453	0.002965038	6.2984613	0.897822882
HSP90B3P	0.002956205	6.020523791	0.897882881
KIAA1787	0.004685054	6.993007096	0.897903338
ZDHHC22	-0.005257509	6.097459103	0.897926244
FLJ40243	0.003471882	5.967024297	0.897934445
LOC642269	0.002576699	6.12564968	0.897962015
PIH1D1	-0.005201391	7.553908375	0.898115778
LOC442403	-0.002977863	6.155371256	0.898279645
BBOX1	0.003861014	6.04349904	0.898311214
LOC652684	-0.003059407	6.438183904	0.898427152
SULT6B1	-0.003857115	6.453609151	0.898436259
LOC441005	-0.002827858	6.019784135	0.898452855
ZNF318	0.003670139	7.34615934	0.898570346
LOC645961	-0.003055035	6.279214472	0.898592897
KIF27	0.001858132	6.103544213	0.898662586
GPR176	-0.003346401	6.273548735	0.898833486
IL19	-0.002381409	6.081433977	0.898965974
ZNF782	0.003516918	6.036052982	0.898980627
PHOX2B	0.002438657	6.116295661	0.899005645
LOC348174	0.003460182	6.008496655	0.899114318
LOC653184	0.002307975	6.125927152	0.899194207
VSIG6	-0.003266494	6.473619695	0.899289176
AGBL2	0.003267756	6.070789961	0.89939505
FLJ46257	0.003651964	5.961974898	0.899406532
ZNF707	0.00333279	6.232741764	0.899409341
LOC441424	0.003202306	5.827652333	0.899476732
LOC647753	0.003307688	6.07841555	0.899577161
CBL	-0.004188705	6.655588822	0.899629589
TMEM40	0.003745401	6.19609209	0.89965879
ZNF560	-0.002842958	6.055013592	0.89976444
LOC653587	-0.00338638	5.976878066	0.899789339
LOC283514	-0.002732898	6.095474697	0.899825149
RASA3	-0.003931536	6.646667278	0.899880482
OR5V1	0.003300862	6.148524421	0.899889679
LOC645558	-0.002610723	6.308167823	0.899938975
MTTP	-0.005832735	6.367981311	0.9000666
ZBTB40	0.005850893	7.745995928	0.900146086
CT45-2	0.003490533	5.928223165	0.90018067
LOC648612	0.002910739	6.013796732	0.900201196
OR2J2	0.003381478	5.970572952	0.900229082
LOC649279	0.002847435	6.082323713	0.900280584
SLC17A3	-0.003321205	6.351110671	0.900363539
LOC653160	0.003319605	6.096506788	0.900411033
CD151	0.006266406	11.52491453	0.9005378
LOC642622	0.003264701	6.091021242	0.900609782
RUFY4	-0.003352942	6.158838965	0.900620606
PLG	-0.002885406	5.964732961	0.900731699
MYO18B	0.006938858	7.632818226	0.900809318
KIAA1245	-0.001326537	6.075296075	0.900848
LOC728637	0.002902916	6.037435338	0.901049135
FAM71F1	-0.002802839	5.990626196	0.90106508

KISS1	0.003272069	6.086777782	0.901077859
LOC643274	-0.003146994	6.205762606	0.901079392
FAM128B	0.005286541	5.997747624	0.901238524
OR51G2	0.003788819	5.962104665	0.901253239
OR2G2	-0.003226301	5.978738013	0.901299158
KIAA0319	0.003242167	5.893637613	0.901351949
TMEM173	0.007074326	9.237937422	0.901369818
OR4N4	-0.003187776	6.007791004	0.901409575
OR2A7	-0.002463222	5.978494384	0.901423631
NEUROG3	-0.003209403	6.01412781	0.90145931
OR2L8	0.003217677	6.014123427	0.901589136
LOC643386	-0.003291229	5.949175396	0.901589934
FLJ25770	-0.003673729	5.92589163	0.901667872
BARHL2	0.003906944	6.134550851	0.901709245
LOC440389	0.002988852	6.239873585	0.901743124
CYP2B6	-0.002206547	6.017300629	0.901745109
LOC441320	-0.002940872	6.005784567	0.90185288
PDILT	-0.00289669	5.969846261	0.902005019
C6ORF182	0.003211429	6.213452007	0.902039327
FAM120A	-0.008057735	11.11886497	0.902056743
FLJ45121	0.002914358	6.055996057	0.902081121
AMN	0.003031022	6.082743824	0.902118077
ULK3	-0.004162239	6.810160842	0.902139185
LOC650174	-0.002610895	6.091846135	0.902235612
SYCP2	-0.002353505	6.217078234	0.902252485
LOC649745	-0.002738176	6.108368312	0.902264278
LOC654055	0.00284665	6.13285285	0.902277373
LOC389043	-0.002010438	5.999612866	0.90241983
XKR5	0.003465741	6.051456842	0.902514079
LOC401358	0.0030578	6.052928443	0.902529048
FOXD4	-0.002801983	6.571822808	0.902795349
LOC651131	-0.002622913	6.045726493	0.902799351
LOC651961	0.003054346	5.853416631	0.902903774
LOC652887	0.003245079	6.254813335	0.902998103
LOC650739	-0.002565853	6.073250517	0.90301935
LOC649701	0.003019074	6.033837491	0.903021319
PRR10	0.003296896	5.952661728	0.903099381
LOC649897	0.003885704	6.679095534	0.903166028
LOC645870	0.002917885	5.946879984	0.903184849
CLIC2	-0.006092023	6.836349732	0.903267172
LYAR	0.010214591	8.504247684	0.903272397
GTPBP5	0.004007009	6.379703577	0.903432492
LOC390760	0.003156104	5.983744995	0.903488246
C11ORF37	0.00327544	5.972167949	0.903668075
C9ORF41	-0.00241303	6.114957363	0.903807936
MDH1B	0.003078689	6.152293656	0.903833513
ZC3HAV1L	-0.002670952	6.234010412	0.903876964
LOC653541	0.003406071	6.083914422	0.903889428
PHYHIP	0.003356579	6.668837981	0.903903562
STAT4	0.017798002	6.963426481	0.903971521
FOLH1	-0.002504505	6.290496156	0.903984312

PGDS	0.002785728	6.042669465	0.904010442
CD3D	0.003582723	6.222705481	0.904042911
DKFZP667M2411	-0.003164154	6.464426594	0.904116693
FAM41C	0.002759853	6.107594984	0.904229061
ZNF708	0.003782383	6.03016491	0.904434811
TMEM201	0.003565703	6.814569746	0.904442049
RBP2	-0.003248748	6.199228096	0.904462183
LOC340527	0.002281576	6.042191787	0.904532242
STAB2	0.003227678	5.994201301	0.904532977
TMEM181	-0.005982546	9.313395082	0.904632974
LOC641939	0.003528924	6.175632588	0.904654006
LOC389633	-0.003277005	5.990227524	0.904761199
LOC388938	0.003958654	6.021602332	0.904764554
THRB	-0.003373079	5.986812532	0.904828428
LOC388397	-0.00328548	6.490369306	0.904833335
NDUFA10	0.008518996	9.560883748	0.904866142
LOC649707	0.002998397	6.122161288	0.904887116
CENPH	0.002345124	6.207039424	0.904912993
PSMD7	-0.00723257	10.76578491	0.904923003
LOC391555	0.00303594	6.102280637	0.905028871
CT45-5	0.001785677	5.994984767	0.905051802
ALKBH4	-0.003656619	6.494489058	0.905060973
LOC643009	-0.004533061	5.943965577	0.905323762
TBC1D10C	-0.003624898	6.277303892	0.905333996
TRAP1	0.005051784	8.929236244	0.905425276
LOC649584	0.002768273	6.080819321	0.905465466
LOC283951	-0.007040336	8.118267173	0.905570485
ECH1	-0.007927811	12.46049766	0.905583101
CNTNAP4	-0.002156775	6.10882665	0.905894308
LOC389000	-0.004018131	5.945047236	0.905938859
LOC642516	0.00271243	6.001803771	0.905945727
TTTY2	0.001640699	6.036663667	0.905961395
DGAT2L4	-0.002976425	6.065485387	0.90606839
S100Z	-0.002195239	6.002563758	0.906093357
EXOC8	0.004903411	6.417814707	0.906346298
AKAP4	0.001955227	6.154434911	0.90641107
F11	-0.002143684	6.138041841	0.906618345
LOC440402	0.003125538	5.998559625	0.906685994
FLJ35894	-0.002805343	5.954586634	0.906727809
KCNH5	-0.002369186	6.016394895	0.906914222
ZNF79	0.00407558	6.234939355	0.907021621
IKZF3	0.002771913	6.34096063	0.907074402
A3GALT2	-0.002366481	5.989230817	0.907092153
LOC388114	0.003136504	6.043552678	0.907130717
DHX57	-0.001756137	6.132477438	0.907154919
C20ORF179	0.003146568	6.008779038	0.907213621
SELP	-0.005001697	6.478739801	0.907369545
TMEM167A	-0.00739935	7.11373227	0.907429956
LOC651400	-0.002743863	6.009443471	0.907594651
KRT18	0.002523964	6.135411484	0.90767025
RCSD1	0.003924715	7.982036312	0.907726879

TMEM185B	0.004821808	7.828701682	0.908018626
LOC646046	0.004424844	6.102224853	0.908052554
LOC645418	-0.002929593	5.943687928	0.908276735
FLJ35767	-0.003086089	6.119829918	0.908390099
KIAA0182	0.005585447	8.73544801	0.908540819
TLR9	-0.002972393	6.097997148	0.908697033
FLJ22184	-0.002116294	6.106131585	0.908741937
MSGN1	0.003641096	6.011705905	0.908776937
OR8D4	-0.003131744	6.041385822	0.908792012
MAGEE2	-0.002934919	5.985954556	0.908847773
LOC644824	0.002537228	6.066387252	0.908941125
KCNJ1	0.001811978	6.052612133	0.909027262
LOC728670	-0.003088968	6.183041892	0.909074915
UCHL5	-0.005269446	8.175685792	0.90915167
LOC643493	0.002821611	6.207454378	0.909237349
XIAP	0.0043461	6.531237967	0.909306358
HS3ST1	0.004910362	6.37269806	0.909350062
MORC1	-0.002669406	5.995744722	0.90940444
LOC642243	0.003759379	6.236075315	0.909499536
SEC63	0.003514248	6.436701295	0.909505753
CA10	0.002866081	6.06567203	0.90954867
DAGLA	0.0038649	6.637891977	0.909618276
LOC652811	0.002989043	5.986230953	0.909645765
HAND1	0.007609379	10.83307111	0.909850633
COG5	0.006586074	7.753013076	0.910012333
LOC645733	0.002969642	6.013739523	0.910031816
P2RY13	0.002234523	6.272789417	0.910254352
LOC643807	-0.002766667	6.104942967	0.910289331
FXYD3	-0.001697204	6.015420735	0.910329626
LOC649415	-0.002797405	6.009460462	0.91033051
FBXO47	-0.00283086	5.912128512	0.910417517
GTF3C5	-0.006351652	8.172215836	0.910446613
LOC651900	0.002571631	6.035173268	0.910527789
LOC644093	0.002385571	6.287049844	0.910551939
SNORD15B	-0.002986884	6.184493636	0.910625416
LOC727737	0.002750218	6.243387099	0.910691486
LOC388931	0.00201053	6.043974689	0.910715401
LOC644386	-0.002466186	6.043237401	0.910814903
FLJ32011	-0.003655915	6.350710795	0.910887984
CCDC116	-0.002936838	6.193546015	0.910923094
C14ORF148	0.002967819	5.994449463	0.911134212
LOC645557	-0.003332874	5.876815491	0.911163225
PJCG6	-0.003054848	6.205550461	0.911347049
LOC646703	0.002877585	6.181495128	0.911379972
OR5H14	0.003034089	5.853108305	0.911492622
LOC652252	-0.002952413	5.998207522	0.911511688
LOC651568	-0.003046685	6.010013954	0.911523659
RNASE7	-0.002949741	5.953867721	0.911603728
LOC649979	-0.002542249	6.021126558	0.911652378
TTC15	-0.006377326	9.685957258	0.911722669
LOC647102	0.003574912	6.15118078	0.911867588

C6ORF167	0.003120209	6.185835799	0.91195092
RIBC1	0.003126341	6.13511733	0.911964351
LOC653113	0.002825987	6.339597448	0.911965052
EPHB1	0.00304604	6.215981759	0.9120027
LOC642276	0.003408426	5.997634786	0.912005533
NPCDR1	0.002496447	6.277332957	0.912093228
PCDH11Y	0.001754085	5.924344884	0.912169689
LOC642571	-0.002777488	6.036657027	0.912187327
PNPLA3	-0.002455747	5.998177264	0.912229999
KIAA1841	-0.002743732	5.799107304	0.912368942
OR52L1	-0.002713852	6.051797053	0.912531338
C11ORF59	-0.006022284	10.26475681	0.912542729
LOC649652	0.002950969	6.078370759	0.912554549
LOC645942	0.003094362	6.286481348	0.912675014
LOC652646	-0.002588338	6.11282915	0.912698943
PHF20	-0.004695296	7.357805642	0.91271841
LOC647654	-0.003119768	6.185122672	0.91273208
FAM175B	0.003902495	8.947495134	0.912798618
GALP	-0.002790617	5.955876481	0.912831865
LOC653486	0.002915673	6.031920964	0.91289588
FGG	0.001876908	5.987410901	0.913074139
REXO1L1	-0.003221228	5.968018699	0.913093285
LOC402643	0.002745365	5.940046734	0.913204667
DNHD2	0.002977432	6.025492305	0.913289803
GOLT1A	-0.003135947	5.920039886	0.913338558
GPX6	-0.003290686	6.258001158	0.913342775
IL1F10	0.002087066	6.010090953	0.913428258
LOC649371	-0.00200571	6.062997098	0.913444637
LOC729444	-0.001639527	6.020924187	0.913519065
BCYRN1	-0.008357632	9.864324915	0.913585385
SERF2	0.005663988	12.17112907	0.913596248
DDX6	0.003469019	6.186536695	0.913909027
SLC28A3	0.002636833	6.048551748	0.91396209
ABLIM2	-0.008511248	8.104624139	0.913989963
LOC647558	0.002196349	6.043093621	0.91399176
LAX1	-0.002930011	6.337953866	0.914011267
DDT	-0.006854305	11.34380136	0.914018556
LOC654222	-0.003072136	5.980062462	0.914053959
C8ORF79	0.002372336	6.203211651	0.914106186
LOC442517	-0.002834885	6.180009911	0.914212991
LOC120364	0.00300807	6.16691613	0.91428679
NKX2-1	-0.001826422	5.962503513	0.914488545
OR4M1	-0.001762367	6.101514254	0.914559998
LOC441436	0.002937237	6.012314024	0.914600865
FBXO42	0.005671324	6.150234005	0.914624758
POMT2	-0.004398835	6.972816036	0.914697303
TMEM132D	-0.002551599	5.984083315	0.914729087
RERG	0.004344024	6.888131401	0.91476683
FLJ40194	0.002993254	6.076628995	0.914897797
GABRR2	-0.00271476	5.930823317	0.914963531
UBE2D4	0.005341236	9.255752487	0.914972189

KLK10	0.002147327	6.140292697	0.915087616
LOC392713	0.002478684	6.239789905	0.915122874
GPHA2	0.003653206	6.073746338	0.915307912
SURF2	-0.003319195	6.606127523	0.91543444
C4BPA	-0.002823948	5.961717617	0.915457268
OVCA2	-0.004942436	6.193978943	0.915477273
LOC644303	-0.003014686	5.908616479	0.915592832
HSPH1	-0.008833186	9.849213435	0.915688462
PRMT6	0.004079864	6.968820413	0.915750595
LOC648358	0.002573375	6.077184333	0.915784557
CDK4	0.004645374	9.266630675	0.915785647
LOC652588	-0.00291144	5.9574182	0.915863545
LOC644951	-0.002441731	6.062149793	0.915915425
LOC728032	-0.002644152	6.042719293	0.916084733
LOC731085	0.003300609	5.923276654	0.916124215
HIST1H2AK	0.002800963	6.113170669	0.916268818
LOC643752	0.002633766	5.963140184	0.916306253
DNPEP	-0.007679102	7.22014324	0.916333049
LOC649075	-0.002495725	6.136133588	0.916396063
HNRPDL	-0.003560427	8.798032778	0.916498999
PPY2	-0.003479577	5.916259652	0.916548631
HRASLS2	-0.004326872	6.053372677	0.916627722
LOC728119	-0.002512577	6.137115582	0.916784221
FBXW4	0.00606507	9.318333475	0.916819152
FKBP7	-0.001882082	6.103834502	0.916832408
SLC22A12	0.001872237	6.034101566	0.916904734
H1FOO	0.002458432	5.996272115	0.91692506
CHP2	0.002954468	6.099817016	0.917047094
LOC402665	0.003047015	6.013573796	0.917174281
CCDC4	0.001892337	6.234093332	0.917223066
LOC644573	0.002624826	5.95769677	0.917319391
WNT8B	-0.003104906	5.972401693	0.917325814
TTTY4	0.003207699	5.892912844	0.917340291
NOXA1	0.004484624	6.706134654	0.917542438
LOC644968	0.002234769	6.078282834	0.917616568
PIP5K1A	0.004140827	6.794756279	0.917676353
LOC652349	0.002591138	6.124731108	0.917706803
OR10G9	0.002654114	5.994399333	0.917736364
CACNG5	0.001887575	6.058230484	0.917793487
LOC648000	0.005245789	12.2042365	0.917868765
LOC644021	-0.00252108	6.101112394	0.917936957
EDC4	-0.00364734	8.998404117	0.918060533
EIF2C1	0.003255058	7.583590538	0.918095392
GPC5	-0.004408754	6.347932716	0.918141041
NRN1	0.00504912	6.264764776	0.918153575
RPS26L	-0.007210862	11.80426529	0.918260952
LOC161527	-0.002996205	6.809259769	0.918344995
LOC653427	0.002743302	6.099913443	0.918425926
LOC642902	0.002349117	5.984187156	0.918439143
LOC642628	-0.002628715	5.935687358	0.918501236
MPN2	-0.002776723	5.966261885	0.918598632

C12ORF50	0.00185585	6.045577585	0.918645529
STRADA	-0.003358117	7.295351145	0.918712047
OR4M2	-0.001722692	6.003200062	0.918721991
TRPC4	-0.003312082	6.287681912	0.918728358
CCDC27	0.002839521	6.292211186	0.919021114
MCAT	0.003035505	6.886333438	0.919059679
GPR78	0.002510918	6.186238516	0.919074221
LOC389333	-0.002754271	5.998441864	0.919078348
LOC641518	-0.002665879	6.007118358	0.919081494
OR1J4	0.002399066	6.139500232	0.91910689
CALCR	0.002385076	6.047056481	0.919136121
TOR1A	0.005458546	7.311463348	0.919221729
LOC391033	-0.002510785	6.17890667	0.919253838
SRY	-0.002140434	6.123818177	0.919296488
TENC1	0.002448289	6.549110041	0.919307691
CTSL3	0.003046562	6.162937524	0.91938195
OR8J1	-0.001779199	6.032501883	0.919393117
GPR18	-0.002543907	5.998616927	0.919394199
LOC653652	0.003503641	5.953434181	0.919443841
CST4	0.001867209	6.048044766	0.919495086
GBP6	-0.002206262	6.096427502	0.919526338
LOC649030	0.002442808	6.200047788	0.919610134
LOC286478	-0.002759996	6.072049845	0.91965564
LOC728241	0.002519113	6.125902932	0.919885581
LOC284009	0.002878032	5.910023765	0.920091974
DDX10	0.003891819	7.946706427	0.920131923
LOC388910	0.002722177	5.91018462	0.92025249
KIAA1026	-0.001391738	6.20543287	0.920289683
LOC137107	0.004320357	6.520695715	0.920300502
ARID3C	-0.002639982	6.105372382	0.920305517
LOC644917	-0.002772516	6.068067084	0.920395804
LOC645581	0.001888078	6.026976101	0.920526462
PSG9	-0.002571883	5.979310185	0.920593343
POLH	-0.002015051	6.209717116	0.920623112
EXOSC2	0.003702519	7.746880393	0.920631643
LOC649613	-0.002341536	6.096593451	0.920689795
FLJ36701	0.002455863	6.012683877	0.92083199
HBD	-0.002512436	5.99845908	0.920856562
LOC649542	-0.002594364	6.144487454	0.920877367
LOC645722	0.003728328	6.445804128	0.920894629
LOC649593	-0.002365393	6.038217043	0.92089937
FUT9	0.001678234	5.902651146	0.920910653
HIST1H2AG	0.00194118	6.015744514	0.920966625
ATAD5	-0.002265072	5.9933307	0.921068367
LOC644229	-0.002421896	6.042752638	0.921074661
ATP8A2	0.002539194	6.366175069	0.921131202
PORCN	-0.002997838	6.540084583	0.921196786
LOC653193	-0.002443132	6.003784917	0.921417253
MYADML	0.002762386	6.11069279	0.92159947
MTNR1B	-0.002803827	6.085914002	0.921633467
TRIM59	-0.002675359	5.876595634	0.921664627

CYP26A1	0.001406341	6.101920633	0.921670103
FLJ23356	-0.002773287	6.017221199	0.921699501
C11ORF35	-0.002461398	6.027869394	0.921700702
ACSM2B	0.001373112	6.083011517	0.92175126
LOC645335	-0.002606305	5.952373111	0.921794164
DHCR24	-0.007410415	6.937013169	0.92184923
FSHR	-0.001956133	6.015869923	0.921906444
LOC646713	0.002239942	6.180862664	0.921932784
STX1B	-0.002501703	5.984403541	0.921988172
FLJ44006	0.002437681	5.992876729	0.92201178
LOC645723	0.002650465	6.269437058	0.922127646
TUBA4	0.002630065	5.917085763	0.922183635
LOC284912	0.002638053	6.165480248	0.922188293
LOC652864	-0.006625312	8.655678538	0.922211163
CFHR3	0.001983579	6.021572784	0.922234198
LOC389362	0.002333774	5.898684671	0.922296373
FOXL2	-0.001375648	5.990842314	0.922409415
BBS7	0.001903144	6.390311465	0.922500953
AIFM3	-0.003762686	6.052761144	0.922511526
KCNK7	0.001947511	6.144569214	0.922521822
LOC440585	-0.002568399	6.030088811	0.922607642
LOC440280	-0.003413744	6.749001282	0.922701676
LOC643836	0.001920794	6.115809851	0.922849187
FLJ36031	0.00257547	5.992027642	0.92290094
DNAJC6	-0.002040838	6.07382938	0.922968159
OR10A4	-0.002721604	5.930996677	0.922997267
LOC652585	0.002473734	6.036104795	0.923078666
LOC649181	-0.002461215	6.046240531	0.923101801
LOC648230	-0.002210121	6.065845263	0.923184444
LOC402382	-0.002208361	6.117284017	0.923346552
ZNF765	-0.002321211	5.902780383	0.923445393
FAM19A2	0.001594782	6.089072555	0.923451547
LOC652000	-0.002685189	5.894767899	0.923492637
LOC643191	-0.002388463	5.906925531	0.923511508
LOC391761	-0.002533573	5.987352911	0.923543126
LOC644922	-0.002769625	6.077259093	0.923545424
CFDP1	-0.007583298	7.326408699	0.923654646
ZBTB2	-0.00277261	6.312665936	0.923711634
MITD1	0.003969578	7.855479118	0.923715865
CDH3	-0.002278102	6.015511399	0.924161665
LOC57399	-0.00244788	6.091951419	0.92432513
GOLT1B	0.006195093	7.206004308	0.924408701
TCL1B	0.002030369	6.102460252	0.924467129
C9ORF75	0.00436838	6.583386842	0.924478069
ARID3B	-0.003775328	7.042500035	0.924553987
DMRT2	0.001886783	6.097719553	0.924621237
PRSS16	0.002621225	5.898377526	0.924629496
LRP5L	0.004141509	7.229486545	0.924887677
C1ORF167	0.003044633	6.100916538	0.924898247
TMPRSS2	0.002585592	5.970890719	0.925122853
OR6C75	0.002299242	6.030339207	0.925250353

LOC644341	0.002152203	6.025715157	0.925311462
C6ORF126	0.002421166	6.029659956	0.925324889
LOC731658	0.002489225	6.03850735	0.925383237
CALR3	0.002109411	6.032348828	0.925554088
LOC402573	0.002668079	5.930912586	0.925722126
SFXN1	-0.004216823	7.303051562	0.925725631
SORL1	-0.003390909	6.571095653	0.925799059
AGAP7	-0.003348503	6.180672766	0.925883335
PRR18	-0.0022632	6.253755709	0.925890335
LOC644391	0.002533647	6.330130416	0.925941276
LOC645812	0.002390383	6.098610511	0.925965802
LOC652383	-0.002494299	5.963882819	0.926062386
LOC128102	-0.002096229	5.927947854	0.926204781
LOC154872	-0.002048623	5.94331382	0.926256136
C1ORF127	0.004177176	6.024412205	0.926262595
PAGE2	-0.002380615	5.966388733	0.92628211
MED29	0.005983283	7.808926809	0.926481701
LOC650933	-0.002274975	6.243714132	0.926697647
CLDN7	-0.002249776	6.159412383	0.926868202
LOC647479	0.002002558	6.001245758	0.926972639
SCGB2A1	-0.002254927	6.012342977	0.926987806
LOC645843	-0.002348742	6.028999444	0.927065412
LOC642548	-0.002832025	6.180627219	0.92723729
LOC642220	0.002365903	6.106823482	0.92729815
LOC647942	-0.002419465	6.172279705	0.927323041
PSKH2	0.002285995	5.86828617	0.927323155
C11ORF55	0.002117313	6.122834963	0.927355564
LOC642101	0.002529273	5.996492534	0.927376876
RPS4Y2	0.01363653	7.506693755	0.92744305
MAPBPIP	0.004972629	9.572090839	0.927565903
ZNF398	0.002161638	6.703292826	0.927644526
C1ORF95	0.00302191	5.945125089	0.927691978
KRT40	0.002790495	5.826037298	0.927845112
HIST1H4B	0.002248465	6.112705731	0.927868452
GPR31	-0.00246577	5.954927045	0.927909251
LOC441097	-0.002214279	6.027557253	0.927941834
HOXD8	0.002381291	6.090141335	0.928025714
LOC645660	0.002131679	6.082506901	0.928193201
TRIM17	0.001881691	6.176314638	0.928349848
FADS6	0.003549705	6.021936589	0.928391572
MC4R	0.002079419	5.995217668	0.928428685
FLJ00312	0.001136084	6.05382505	0.92852272
LOC646424	-0.002402062	5.891401092	0.92856073
LOC131055	0.002464122	6.007389988	0.928652711
CTNNA1	0.002587173	11.73090963	0.928821906
AP3B2	0.002611776	5.953935586	0.928868814
LOC644152	0.00209157	6.075547056	0.929033263
LOC646915	-0.002133618	6.262405154	0.929147581
LOC649167	0.00219696	6.023641545	0.929203255
SLC37A2	0.002461557	5.87444961	0.929345443
LOC643755	-0.002000284	6.105327209	0.929354865

LOC146053	-0.002325127	6.163274402	0.929379065
LRRC16	0.003841034	6.536996734	0.929436698
CCDC73	-0.002104684	5.977749302	0.92950487
BNC1	-0.001083361	6.06109481	0.929523885
LOC650195	0.002551125	5.990809818	0.929541377
LOC644064	-0.002358382	6.079008383	0.929556976
ZC3HC1	0.003724415	9.806750985	0.929582443
LOC643879	-0.002219503	6.023305255	0.929728274
LOC645359	0.002916084	5.966297942	0.929882862
LOC652427	0.002232912	6.312305543	0.929929624
LOC646951	-0.001538527	6.027591046	0.929989889
CBLL1	0.00398806	7.466144405	0.930034116
OLFM2	-0.003033229	5.965782979	0.930079499
LOC643081	0.00223981	5.938349245	0.930155977
ATP12A	0.002413167	6.159384977	0.930163726
CCDC52	0.002229742	6.002838164	0.93024355
LOC649071	0.002052285	6.163845582	0.930245629
LOC644379	-0.002360897	6.002463451	0.930334227
LOC644201	-0.002254635	6.078361329	0.930352979
PHOSPHO1	-0.002417238	6.095889884	0.930388528
LOC649722	0.002106422	6.070907205	0.930411008
MGC44328	0.002582953	5.994994415	0.930411474
LOC646716	-0.002100575	6.037377712	0.930495394
KRTAP3-1	0.001866333	6.136368458	0.930511772
LOC642833	0.002482373	6.287947206	0.930535182
TGFBR1	-0.002413846	6.156227102	0.930603977
BLMH	-0.00548709	7.447024175	0.930606011
LOC550631	0.002537154	6.069902265	0.930619967
LY6K	-0.002229954	6.018212085	0.930711503
SORCS3	0.001579753	6.134038395	0.930738688
VTI1A	0.002795243	6.033611748	0.93076382
OR52D1	-0.002824902	6.203028467	0.93081532
VENTXP7	0.00148663	6.032302637	0.930880991
MMP10	-0.002061861	5.994427542	0.93091984
OASL	0.003218361	6.071941851	0.930947275
LOC641753	0.00263804	5.998081652	0.930949594
LOC648795	-0.002378815	6.001815494	0.93098589
LOC646888	0.002270356	6.013444788	0.931131835
PINX1	0.00197649	6.813083814	0.931149306
C21ORF77	-0.002219571	6.146754338	0.931212514
CACNB4	-0.001527697	5.967968138	0.931273576
CLEC1B	-0.002279588	5.977048924	0.931366752
GDF11	0.003810644	6.072031738	0.931503606
NPAL2	-0.001704624	6.208294251	0.931640971
UNC13C	-0.001343971	5.999371741	0.931694652
LOC389072	-0.001986554	6.078892848	0.931755845
SOCS7	-0.001904755	6.091256282	0.931878112
LOC648816	0.002030473	6.102142523	0.931917433
RCC1	0.002454731	6.058070982	0.93191765
TSC2	-0.002672126	6.939278609	0.932011522
PCDHGA1	0.001340303	6.045587594	0.932251278

LOC652015	-0.002009329	6.0631548	0.932320605
LOC641977	0.001537816	6.023733994	0.932337061
OR5T3	0.002312772	6.118016463	0.932386514
LOC653528	0.002863289	5.79980331	0.932429743
LOC646207	0.002188173	6.186193878	0.932465899
ATRN	0.001727046	6.885988622	0.932495845
UNQ9391	-0.002805582	6.11975363	0.932498691
LOC651495	0.002347191	6.102888338	0.932528132
SHC3	-0.002984012	6.016246104	0.932689007
NBPF7	0.00168628	6.027417128	0.932889434
SNORD18B	0.002041758	5.95922487	0.932922705
HRES1	-0.002829557	6.144357269	0.932934089
LOC152118	-0.001933771	6.127436036	0.932937863
LOC653806	0.002080396	6.112586284	0.932998787
IL9	0.001614523	6.07319774	0.933184645
SLC25A31	-0.002655427	6.139992482	0.933279155
KIAA1906	0.001940066	6.166671703	0.933287784
OIT3	0.002960479	6.084168707	0.933310266
LOC646932	-0.001867095	6.180442665	0.933342527
CXORF48	0.00125521	6.088584945	0.933398287
LOC391727	-0.00222935	6.044660494	0.933445916
CCDC146	0.004975272	6.693757924	0.933513761
SLC35D2	-0.003547905	7.329909435	0.933700347
LOC731837	-0.00215129	6.077207811	0.933719661
LOC644231	-0.002100349	5.927122564	0.933915636
C6ORF122	0.002089792	6.10820531	0.933931752
PIP	-0.002297392	6.038577276	0.93396616
LCE2B	-0.002331867	6.054362235	0.933981148
LOC650678	0.002374917	5.97652458	0.934004575
CELP	-0.002246599	5.980266792	0.934025966
TRPS1	-0.002932232	6.285250054	0.934065632
GDF3	-0.002185947	6.096239754	0.93409906
RPS14	-0.003056175	12.92045118	0.934108156
LOC654209	-0.002230092	6.128651516	0.934110793
LOC653554	0.001980481	5.88646053	0.934285322
CYP2C8	0.00298915	6.053727448	0.934404341
DEFB105A	0.002226273	6.082750191	0.934404441
CNTNAP2	0.003960544	6.200699432	0.934441515
LOC650040	0.001945945	6.061138618	0.93445853
DNTT	0.001725354	6.091911644	0.934468955
OR8K1	0.00191008	5.956386946	0.934491277
LOC653088	-0.002148574	6.048383895	0.934635209
PPP2R5C	0.002809027	8.241131511	0.934660424
TAPBPL	-0.004129359	7.072234481	0.934691827
LOC442049	-0.00224452	6.270746675	0.934692844
C1ORF146	-0.001621464	6.007905641	0.934749508
SNORD33	0.002418062	6.541221645	0.934794764
LOC650034	-0.002370694	6.670398364	0.934873849
FAM9A	0.002350749	5.868253558	0.934948347
LOC654121	-0.006240684	9.270287089	0.93495036
FAM75A1	0.002068041	6.081108679	0.935188504

LOC442570	0.001689783	6.064004612	0.93549611
RGS9BP	-0.002766678	6.405026202	0.935500502
GPR97	0.001062515	6.084041087	0.935554549
LOC643033	0.001770238	6.030566473	0.935573547
LOC648240	0.002243286	5.918346525	0.935788782
C16ORF78	-0.001747495	5.962197757	0.935808086
SSTR1	0.001563754	6.05581408	0.935820292
GPR98	-0.001656012	6.027087841	0.93587037
RAB36	0.002340978	5.890286837	0.935907235
DCHS2	-0.00140794	5.980551508	0.935969994
LOC651101	-0.002144433	6.240738361	0.936089509
TSHZ3	-0.003841327	7.443925326	0.936125969
LOC642381	0.002079585	6.090199932	0.936135026
HOXB6	-0.004048625	7.077197959	0.936146241
OR1J2	-0.002232582	6.231878081	0.936172924
LOC647582	-0.002039105	5.958508587	0.936268272
KIR2DS2	0.002183067	6.058026832	0.936270328
LOC647199	0.002412593	6.13256495	0.936328092
S100A7A	0.002165283	5.967417018	0.936422895
LOC729985	0.007164199	6.752372184	0.936520004
LOC646004	-0.00215717	5.968092845	0.936760151
HSPD1	0.003917522	10.24357609	0.936855971
NEK2	-0.001419189	6.073239375	0.936871892
RBP5	-0.002436117	6.438458756	0.93697279
HIST1H2AB	-0.002890513	6.08737973	0.937050263
LOC643502	-0.001882919	6.197558055	0.937160968
LOC653667	-0.001784862	6.108154152	0.937163121
PF4	-0.001870749	5.918498262	0.937232045
PODNL1	0.002322438	6.211766744	0.937239077
CBX6	-0.004019423	9.098162617	0.937309094
CYP2F1	0.001493257	6.00617457	0.937322717
LOC643850	0.002054984	6.104350153	0.937454953
LOC650751	-0.002183771	5.940153424	0.937468032
ICAM1	-0.003635242	6.039228924	0.937502942
FTSJ3	-0.003571656	6.685232898	0.937542743
LGALS14	-0.002154846	6.163724042	0.937592806
RASSF8	0.002142427	6.25278513	0.937664647
OIP5	0.001773658	6.189636665	0.937666652
HOXB3	-0.002182959	5.966157654	0.937743062
LOC647417	0.001922925	6.112707679	0.937825949
LOC285544	0.001830516	6.071938724	0.937860093
C3ORF63	0.002828455	7.046359066	0.937901446
DAPL1	-0.001943535	6.003893896	0.93790272
C20ORF4	0.00343853	8.23112841	0.937980073
OR2T6	0.002171978	6.10971969	0.937984946
BTBD17	0.002248	5.840813093	0.938072338
SLC24A2	-0.001982914	6.103835825	0.938084144
LOC647894	-0.002746926	6.010493313	0.938099779
SLC3A1	-0.001963757	6.095761078	0.938158451
ZNF114	0.001650816	6.097640393	0.938166935
C10ORF79	0.002017958	5.961227798	0.938225467

LOC401164	0.001864695	6.08853511	0.938251911
VKORC1L1	0.004804651	8.421482284	0.938289264
LRRC4	-0.002619117	5.93529259	0.938443908
LOC651175	0.00210139	6.000702713	0.938446381
DIAPH1	0.006024384	8.514243499	0.938505337
MOV10	-0.002672232	6.981867186	0.938738735
KLK6	-0.001379939	6.083731828	0.938875859
CCNH	-0.003006086	7.80825316	0.938925384
LOC643286	0.002062457	6.033075483	0.938959617
COX4I1	0.00343786	14.59557879	0.938981978
LOC644447	-0.001912798	6.107763479	0.938994582
LOC729238	0.001798861	6.083386678	0.939034663
SLC22A3	-0.0014461	6.089644398	0.939076016
MARK4	-0.002916182	5.981524278	0.93921029
RPS26P10	0.004971452	7.054092672	0.939266784
KCTD19	0.001969921	6.031533075	0.939469742
TDH	0.001858101	5.952116348	0.939749575
ENPP3	-0.002110188	6.088367942	0.939757748
LOC649841	-0.005415714	6.906718016	0.939767572
PTCHD3	-0.001212501	6.107490823	0.939825799
CYP3A7	0.002342144	5.98130192	0.939886689
TMEM184B	0.003748794	8.13206331	0.940004682
LPIN1	-0.005449269	10.36867887	0.940021423
TMEM120A	-0.005234345	9.41641285	0.940219645
JMJD2C	-0.001982506	6.328778505	0.940269258
STON2	-0.001894764	5.964913057	0.940308749
LOC651772	0.001984017	6.127559075	0.940339637
MYEOV	-0.001855173	6.063052249	0.940892584
NRTN	0.004925291	7.258279528	0.940971239
HIST2H2BF	0.001818861	6.258585681	0.941017692
SPTA1	0.002227296	6.169037524	0.941031791
ERO1LB	0.002196674	6.53587264	0.941097826
KRT28	-0.001711792	5.953396802	0.941234447
CCDC22	0.003904661	6.597873129	0.941287666
EIF3F	-0.003417995	10.48324182	0.941296906
DEFB123	0.00225902	6.206143117	0.941382131
LOC650330	-0.001703373	5.937964915	0.941437951
LOC643699	0.00219374	6.115622759	0.941491857
LOC647257	0.001800529	6.061630258	0.941590451
LOC441964	0.001692524	6.039127885	0.941610348
SELPLG	0.001805756	6.053422213	0.941618899
C3ORF20	0.002058055	6.007499397	0.941702566
LOC642293	0.001900648	5.853760131	0.941754915
FLJ31568	-0.001208756	6.038105179	0.942039442
GPX2	0.00190376	6.174094444	0.942222856
CYP1A2	-0.001909649	6.017173006	0.942296326
PSD2	0.001777224	6.146034141	0.94251412
LOC645413	-0.001755863	6.108181331	0.942523626
PRO0132	-0.001495141	5.992333928	0.942607506
APOL4	-0.001535801	6.101083566	0.942746364
LOC730419	-0.001408299	5.989874777	0.942849993

LOC644963	0.00169699	6.062639381	0.942904606
LOC643677	-0.001793342	6.003406418	0.942994294
LOC650620	0.002217299	6.372991322	0.942999056
MUC15	-0.001976243	6.028165207	0.943234079
LOC647995	-0.001820918	6.241797923	0.943234158
LOC650256	0.001735869	5.951352974	0.943283491
FLJ34306	0.001545092	5.961053142	0.943318973
LOC643468	0.001701017	5.961707142	0.943323588
SNORD27	0.001820475	5.941320692	0.943349759
LOC653616	0.002010392	6.147202112	0.943362314
MESP2	-0.001891461	6.020500113	0.943379306
LOC644880	0.002166977	6.076928256	0.943383239
UBE2E3	0.002359907	7.360804325	0.94348093
GSTM4	0.00274048	6.666018674	0.943501498
TMEM171	0.001722902	5.944942296	0.943505675
CCT4	-0.002910667	6.976651511	0.943558283
ZNF833	-0.001837668	6.192216566	0.943602283
LOC649276	-0.001820635	6.187177625	0.9437067
HRAS	-0.002579367	7.965258334	0.943711436
RXFP1	-0.001599178	5.910128814	0.943795641
MRPL32	0.003886302	10.24664185	0.943831623
NOTCH2NL	-0.001597849	6.209375259	0.943865749
LOC389936	-0.001499764	5.93552661	0.94400971
LOC642359	0.001719826	6.032823483	0.94415126
C20ORF77	0.00165926	6.035928072	0.944216371
CCDC36	-0.002019515	6.164056302	0.944223563
C21ORF94	-0.001556251	6.039115384	0.944252325
HIST1H4K	0.003757903	6.785104154	0.9443365
UGT1A4	0.001846944	5.983665868	0.944357627
CPN2	-0.00360604	6.581637922	0.94438159
LOC654249	0.001629182	6.180071077	0.94443923
LOC647436	-0.006403763	7.836935636	0.944829222
A1CF	0.001153687	6.108813451	0.944867727
PCSK2	-0.002272853	5.986257256	0.944936862
LOC651820	0.001753705	6.082133868	0.944954339
LOC647979	0.002471383	5.943057161	0.945406371
PGAM4	-0.002749387	9.556409845	0.94551572
LOC401296	0.001957136	6.264786446	0.945524707
SMG7	-0.003166132	7.813375209	0.945528153
ST7OT2	-0.001762726	6.043531745	0.945535477
ISX	-0.001726612	6.049977895	0.945568636
LOC652076	-0.001468602	6.20824146	0.945695661
LOC388117	0.001728227	5.990734023	0.945813117
CFHR2	0.00203554	6.312376819	0.945846416
LOC652505	-0.002066992	5.950250177	0.945853865
HIST1H1E	-0.00162466	5.90658434	0.946020608
SLC6A10P	-0.003749191	8.886529861	0.946135609
COX10	0.003530406	10.5866177	0.946141104
ZNF749	0.002694013	6.041885739	0.946183442
UBXN10	-0.00219017	6.567697828	0.946190468
LOC653362	-0.001771835	6.113195443	0.946201301

ANO9	0.00113908	6.155144246	0.946214795
LOC642360	0.001557987	6.044194258	0.946371799
ACCN1	-0.001257237	6.078033112	0.946480594
LOC648623	0.00173691	6.133788913	0.946500647
LOC642490	-0.001728748	6.075017167	0.946562943
LOC404266	-0.001757187	6.087228454	0.946648442
C17ORF64	0.001997735	6.259724905	0.946982959
DLX4	0.001178161	6.12116362	0.946992425
ZNF8	-0.002444758	6.188646954	0.947262373
GALNT6	-0.002120967	6.479863062	0.947332828
SPTY2D1	-0.00224626	7.067163284	0.947551777
AKR1CL1	-0.001997901	6.193584604	0.94765316
BNIP1	-0.001938039	7.009511615	0.948021472
OR4A47	-0.001676643	5.929226226	0.948071311
LOC651713	-0.001277471	6.060884824	0.94807402
RNF166	-0.002124311	6.550753586	0.94813665
HLA-DPB2	-0.001277354	5.949411067	0.94820353
PCDHGA10	0.001570485	6.351304041	0.948243047
LOC126767	-0.001498079	6.176548885	0.948276301
CCDC153	-0.001151187	6.002706285	0.948303619
C10ORF62	-0.0018106	6.083923834	0.948379408
MYLC2PL	-0.001514835	6.014182439	0.94838505
LOC652094	-0.001732377	5.85734327	0.948385297
ATP6V1C1	0.001759377	6.666661672	0.948404578
FLJ44861	-0.001642147	6.007572244	0.948419379
SP7	0.001592485	5.978154232	0.948585176
LOC727773	0.001049577	6.244951452	0.949045616
PLA2G3	-0.001635626	6.015234445	0.949081674
UNG2	0.001610179	6.29755885	0.94912282
LOC731985	-0.003611953	11.68220322	0.949169518
FOXR2	0.001476709	5.973417481	0.949170673
RIBC2	0.001558974	6.00823226	0.949280476
NPR1	0.002901472	6.115094261	0.94928336
LOC730746	-0.005467939	6.775660301	0.949309478
FLJ20297	-0.001724334	6.015069704	0.94939264
SMYD5	0.001686118	6.52256349	0.949452629
CPA5	0.001742783	6.08630116	0.94947108
LOC643792	0.001695295	6.265292659	0.949883776
RASSF3	-0.002123705	6.140579112	0.94988914
SLC25A2	-0.00159099	6.010646783	0.949950555
HIST1H2AA	0.001295613	6.059850026	0.949957286
SMARCA1	-0.003046635	8.142514621	0.949986051
LOC651758	0.001602143	6.062914356	0.95011824
KRTAP19-5	-0.002297917	5.91464398	0.950170498
CAPN9	0.001196936	6.058011904	0.950241712
MRPS18C	0.003362383	9.496792016	0.950298854
LOC646879	0.001542938	6.116745118	0.950318377
LOC730684	0.001256906	6.04462873	0.950541235
LOC646106	-0.001531808	6.304856317	0.950615549
DSG1	0.002035264	6.054217457	0.950660441
GFRA4	-0.00142275	6.304453815	0.950686831

RB1	0.002712542	7.162203234	0.950724122
ASCL4	-0.00185228	5.91530101	0.950725564
KRT5	-0.001444081	5.956024692	0.950822576
ZNF92	0.001034561	6.089801388	0.95090291
LOC651659	0.001598596	6.101743273	0.950984813
LOC389174	0.001680911	6.114038576	0.950994659
GUCY2C	-0.001662726	5.947514671	0.951037062
FGA	0.001473698	6.741440507	0.951050592
ZBTB39	0.001433937	6.403129121	0.951333933
SAMD3	0.000861673	6.053681479	0.951347249
UMODL1	0.000980058	6.01464602	0.95144706
SMPD2	-0.001957059	6.44573865	0.951578923
LOC390880	-0.001473611	5.982087615	0.951591151
LOC731823	-0.001253309	5.9898119	0.951613567
LOC649970	-0.001528952	6.082613651	0.951689403
LOC647760	0.001455902	6.022684144	0.951738823
FLJ34690	0.001638496	6.077307684	0.951830572
LOC441377	-0.007023705	10.2633253	0.951845187
SRMS	-0.001341447	6.114498507	0.952010756
SPRR2F	-0.001766182	6.076631995	0.952036126
LOC647527	-0.002189153	6.043436782	0.952130011
SFRS11	0.0054861	7.474884523	0.952144648
FMO5	-0.001554254	6.159385995	0.952239667
HRASLS5	-0.001340975	5.984111064	0.952411909
LOC651410	0.001543528	6.029995238	0.952518963
ALOX15	-0.000857128	6.111386912	0.952527696
KIF18A	0.0011943	5.908340302	0.952618305
LOC643369	0.001291972	5.968811819	0.952634618
LOC392447	-0.001271772	6.007428506	0.95265109
NKX6-1	0.002189156	5.848918263	0.952661756
LOC642646	0.001605145	5.988142599	0.952669334
MGC33948	0.001803321	6.481813229	0.952700755
FBS1	-0.00177548	6.49989467	0.952724975
C17ORF46	0.001488924	6.241823872	0.952743689
MS4A15	-0.001788129	6.080512383	0.952782743
LOC652769	-0.001649158	5.93780524	0.952862442
SACS	-0.004788898	7.212349259	0.952900855
SH3RF3	0.002813583	7.079335288	0.952993105
LOC646444	0.002091377	5.950574628	0.953220538
LOC727811	0.001345983	6.019601052	0.953236842
TOP1	0.001411126	6.124665411	0.953271588
TPD52L3	-0.000981585	6.164076683	0.953289841
TMEM160	0.002792434	9.533906493	0.953295744
LOC285307	-0.001479579	6.046211485	0.953377416
ADRB3	0.00154118	6.083485388	0.953508801
SNHG4	-0.00149563	6.102495721	0.953536957
SARDH	-0.001419815	6.011703412	0.95354157
ZNF773	-0.002175972	7.415345984	0.953571115
LOC652175	-0.002241605	5.980291765	0.953597871
CRK	-0.00291205	8.533552826	0.953603421
ZNF101	0.001483755	6.225477986	0.953653554

TMEM222	0.002127213	7.226106333	0.953660278
MATN3	-0.001708062	6.140805944	0.953677798
LOC643454	0.001668553	6.118336851	0.953725007
YPEL4	-0.001638204	6.077044035	0.953845659
WFDC6	0.001535541	5.987018678	0.953901707
LOC646361	-0.001481801	5.992406312	0.953915995
TTL	0.003280346	8.929947637	0.953963481
LOC645679	-0.001332388	6.04825579	0.954002611
FUT5	-0.001549449	6.138355901	0.954155572
STAP2	-0.000915654	6.265156808	0.95417802
C12ORF11	-0.001783061	7.093908278	0.95420042
RPL23AP13	0.00321026	8.25104506	0.954315664
SEC22B	-0.002304536	8.238130038	0.954371645
LOC648955	-0.001461026	6.060941494	0.954479284
LOC729754	0.001568489	5.983665411	0.954613989
LOC642213	-0.001380764	6.03171604	0.954713947
LOC136157	0.000880571	6.082011409	0.954808777
CHERP	0.001701193	7.066303242	0.954811426
COX7B2	0.001589388	5.98079314	0.954925571
LOC646630	0.003588689	12.29780512	0.954941524
PRO1768	-0.00147697	6.012636487	0.955039283
FOXB1	-0.001054667	6.054177732	0.955193224
TBC1D10B	0.00194828	6.724199825	0.95523703
LOC644719	-0.001544406	6.117408949	0.955266193
CKAP2L	-0.001480999	6.050784312	0.955308208
CCDC42	0.00173274	5.826488872	0.955338372
LOC441488	0.001312699	6.06351682	0.955352643
LOC648680	0.001492735	5.890093751	0.955410562
LOC389634	0.001305874	6.094660254	0.955461917
LOC653935	-0.001352745	6.011567089	0.955702636
MYOG	0.001931587	6.075448147	0.955743879
FLJ46156	-0.001436454	6.09504568	0.955848994
FLCN	-0.001116176	6.293729775	0.955879612
STAC2	0.00150782	6.278736387	0.955904153
SMA3	-0.001406805	5.974250577	0.955915459
ZP2	0.001484282	5.971364	0.955961216
LCE2D	-0.001352118	6.059174537	0.956029563
FLJ20184	-0.001945556	5.9396222	0.95613044
PARP1	0.002696676	10.86883872	0.956134646
MARS	0.002569077	7.832099355	0.956155439
TTTY10	-0.002240858	6.123366599	0.956191328
REG4	0.001770652	5.959815563	0.956405793
TPSD1	0.001401896	5.981340657	0.956591251
LOC648615	0.001359588	6.12037745	0.956684554
LOC440145	-0.001722353	7.052435147	0.956800255
FLJ44450	0.001445916	6.203001257	0.956850096
LOC440261	0.000817431	6.131038471	0.956915242
CASP8	-0.001069985	6.056796107	0.956951724
LOC647534	0.001508672	6.131756762	0.957273659
CCAR1	-0.002698946	6.554211186	0.957343257
LOC643747	0.001202513	6.020571372	0.957357057

FERD3L	-0.001439024	6.17062674	0.957434965
SLFN12	0.001612622	6.126979987	0.957651202
LOC653060	-0.00151795	5.999916361	0.957793615
HARBI1	0.001251632	6.279488838	0.957853222
LOC651694	-0.001342423	6.033416125	0.957870947
FLJ12331	-0.001428669	5.929040286	0.958044423
OR3A1	-0.001482183	6.05511932	0.958122706
TSC22D2	0.003790663	8.608512558	0.958147666
GDF2	-0.001295132	5.964829126	0.958154988
CASS4	-0.001221117	5.990726262	0.958738126
C12ORF64	-0.000738621	6.080240007	0.958791056
UGT8	0.00091384	6.02443024	0.95886773
LOC652740	0.001297215	6.000215491	0.958961168
PRAMEF1	-0.000821728	6.004362106	0.958991355
GNAL	-0.000765328	6.063893837	0.95901988
LOC339782	-0.001505497	6.383041939	0.959114348
LOC645941	0.001212063	6.178571087	0.959418165
LOC651118	0.001286918	5.957689497	0.959439905
TXK	0.001257307	5.967411629	0.959468073
LOC642188	-0.001226639	6.09552044	0.959562252
PCDHB16	-0.001493105	6.310391377	0.959594888
ECAT8	0.002165604	6.247927984	0.959605727
PNLIPRP2	0.00126135	5.900018776	0.959630252
ELAVL3	-0.001046982	6.295779537	0.959653932
OR5T2	0.000927064	6.007542784	0.959686565
RXRB	0.003344751	7.751320772	0.95978451
LOC650767	-0.001300819	6.086752668	0.959900159
ITIH1	0.001916625	6.099045394	0.960197759
LOC644087	-0.001135135	5.993262375	0.960337751
TUBAL3	-0.00175854	6.221093981	0.960501599
CIT	0.001548476	5.896218869	0.960555064
COX8C	-0.001259868	6.132015604	0.960668521
CASP10	0.00098682	6.342415958	0.960720449
UBXN6	0.002650881	9.282782327	0.960728242
LOC650243	0.001207627	6.036259994	0.960773226
LOC644331	0.001114373	6.174212361	0.960829217
LOC644187	-0.001243879	6.031479218	0.961026901
TCEA3	-0.003558509	11.04964412	0.961073335
EIF4E2	-0.00431111	7.696066929	0.961142602
PSEN1	-0.000669361	6.138733992	0.961323206
ARFRP1	-0.001352191	6.836971614	0.961358794
GSTM1L	0.001229253	5.955651039	0.961453553
C6ORF141	0.001202996	6.115802863	0.961464641
LOC644298	-0.001263854	5.964044189	0.961567789
ASCL1	-0.001381419	6.118333101	0.961679732
KIF12	0.001319544	6.030565084	0.961739479
ARID1B	-0.000979569	6.111470233	0.961745541
RAB6C	0.00105845	5.961688161	0.961821416
IGFALS	-0.001234163	6.17921869	0.961822112
DSPP	-0.001267027	6.036217141	0.961980866
RNF10	-0.002694819	8.157792242	0.962011378

LOC144817	-0.00157554	5.939281919	0.962026622
OR4K17	-0.000874887	5.994514327	0.962055143
SBDS	0.004776729	9.881869027	0.962058695
TYMS	0.002568493	6.589709856	0.962083895
H2AFB3	-0.001310428	5.869427118	0.962134886
LOC652833	-0.001165215	6.156628608	0.962171613
APOC2	-0.001468977	6.109978603	0.962228395
C11ORF10	0.003305236	11.39417923	0.96225597
LOC651065	-0.00126029	6.045305561	0.962340502
IRF2	-0.000957479	6.267266158	0.962433738
RUNDC3B	-0.001981417	6.557505374	0.962457719
PRSS21	-0.000937027	6.140459137	0.962476645
C1ORF150	-0.001324457	5.975643367	0.962546455
KIRREL2	0.000798136	6.029814495	0.962627386
KRTAP4-12	-0.001118442	6.134006311	0.962650884
LOC644658	0.001159568	5.925773577	0.962717848
SAR1A	-0.003140243	8.971677108	0.962753582
POLA	-0.001248033	6.155366413	0.962758607
NOBOX	0.000840488	6.107546626	0.962776322
DEFB136	-0.001155543	5.950458784	0.962807837
C7ORF52	-0.001287565	6.048681872	0.962957753
MCCD1	0.001499316	6.287536958	0.963108653
C16ORF65	-0.001178001	6.010473602	0.963114654
LOC729900	-0.001023763	6.005917077	0.963118457
LOC643094	0.001221563	5.920756353	0.963192499
LOC643093	-0.001108398	6.041104429	0.963246847
LOC644365	-0.001065622	6.003009636	0.963275442
ZNF221	0.001143525	5.914474461	0.96330673
LOC646786	0.002965469	8.291733802	0.963481225
LOC646241	-0.001129899	5.948667906	0.963547725
CLEC2L	-0.001505542	6.118370737	0.963585234
CACNG4	-0.001296042	6.079354301	0.963611805
LOC442535	0.001202804	6.104452647	0.963636395
TTTY14	-0.003191039	6.299032645	0.963643099
LOC645460	-0.001228301	6.145977774	0.963723154
LOC653056	0.001201358	6.028383435	0.963782504
LOC442096	-0.00104392	6.027900811	0.963789228
ADAM5	-0.000727911	5.998957345	0.963852777
LOC647353	0.001505662	6.190284584	0.96391097
CILP2	-0.001367923	5.964979875	0.963942224
TMC1	0.001121309	6.099874473	0.963959974
GALK2	0.00110107	6.660722976	0.964155822
ITPRIPL1	0.000682632	6.092000756	0.964156783
LOC651122	-0.001105878	6.10007073	0.964182556
LOC651212	-0.001559331	6.053287943	0.964273859
KIAA1432	0.00124428	6.034029989	0.964334851
LOC652845	0.001241456	6.0060521	0.964341211
HMX3	0.001141277	6.03777461	0.964473444
LOC441843	-0.001019449	6.115399889	0.964477353
ITGB1BP3	0.004351058	12.25674853	0.964597071
LOC388630	0.001087992	6.342420859	0.964650243

LOC646481	-0.001620645	6.136826278	0.964692283
ATXN2L	-0.000636307	6.204178989	0.964784629
LOC126661	-0.001100351	6.157241311	0.964836892
CYP2A7	0.000953433	6.060700436	0.964868251
AHI1	0.001878489	7.895209152	0.964949354
LOC653169	0.001128653	6.05056326	0.965026724
ZBTB7A	-0.001602056	6.087342738	0.965045003
LOC645661	0.00106173	6.103719034	0.965196101
AP1S2	0.002229373	11.37224826	0.96523352
MTM	-0.00131722	6.004421704	0.965237004
IER3IP1	0.002978938	8.109346302	0.965257693
LOC643901	0.001211096	6.060609541	0.965276949
INTS12	0.002038291	9.302480476	0.96527927
SLC7A3	0.00081533	6.095695861	0.965384152
LOC648552	-0.001057508	6.089420146	0.96560488
REEP3	0.001410329	6.352937554	0.965674772
PRAMEF21	-0.001089083	6.029472375	0.966152595
NCK2	-0.002149864	8.447004513	0.966232356
LOC645459	0.000999183	5.950947523	0.966297751
CASRL1	-0.000964637	6.018361701	0.96642026
LOC730926	-0.001000143	5.888845287	0.966486828
BTNL3	-0.000584496	6.06788301	0.96654192
LOC727832	0.000837509	6.087938072	0.966571178
PHF21B	0.001333445	6.071047918	0.9665866
DLGAP1	-0.000780073	5.984669164	0.966588521
ADAM32	-0.00059766	6.047536286	0.966589252
LOC652804	0.001157973	6.029399812	0.966713731
PLXNC1	0.001018593	6.106488326	0.966797605
C10ORF136	0.001091057	6.196630854	0.966825191
LOC731106	0.001056881	5.946227335	0.966846628
DUSP14	-0.002530496	8.028361587	0.966880574
INSL4	-0.000992639	6.077693751	0.96706212
WSCD2	-0.001165627	6.247686488	0.967118888
CXORF12	-0.001790257	7.282478616	0.967168256
CDY2A	-0.001113499	5.968749153	0.967182804
LOC642645	0.001121523	5.956837301	0.96720311
OR4E2	-0.001100865	5.958061742	0.967298753
SMR3B	-0.001089984	5.98534026	0.967315079
NOXO1	-0.00082115	6.150573086	0.967548627
LOC392481	-0.000932334	6.105870302	0.967600576
KRTAP5-7	-0.000912555	6.025644008	0.967631465
TBC1D28	-0.00060584	6.10703892	0.96769356
PRDM7	-0.000931608	5.952354563	0.967710647
ATP8B1	-0.00111583	6.273520313	0.967756734
LOC644711	-0.00102929	6.100417904	0.967815216
SNX29	-0.001612947	7.200955321	0.967842029
NSMCE2	-0.00149027	8.393431417	0.967904543
C1ORF161	-0.001085799	6.151720433	0.967985888
RMI1	0.001405614	6.958790491	0.968002931
SIRT3	-0.001112501	6.033471311	0.968016565
LOC651373	-0.000914803	6.125966674	0.968111533

LOC643231	-0.000961776	5.96576125	0.968145302
DARS	-0.001658113	11.56723914	0.968280799
TTLL10	-0.001108545	5.952623911	0.968307929
ANKRD36	0.000800336	6.118174981	0.96831576
KCTD17	0.001921312	6.250823345	0.968374641
APRIN	-0.00107389	5.976873758	0.968601241
HTR3C	-0.001033388	6.117037567	0.96864293
MAGEB6	-0.000723811	6.051185298	0.968702381
IPO13	0.0021821	8.383604363	0.968758426
SCFD1	0.001512983	8.591710577	0.968783916
LOC728709	-0.001087062	6.213476798	0.968940943
RAB3B	-0.001086428	6.119787047	0.969009669
HMOX2	-0.001700937	6.063823664	0.969184838
LOC644628	-0.000987189	6.097362293	0.969420174
C9ORF45	-0.001948695	7.47222528	0.969471153
IL1RAPL2	-0.001012546	6.054449228	0.969492005
C1ORF177	-0.001106152	6.26202101	0.969512358
TTC7B	0.001256774	6.471857692	0.969577848
CLDN23	-0.00187215	6.731460424	0.969702556
LOC647849	-0.001007303	5.933166067	0.969752634
C14ORF121	-0.001080589	6.466255466	0.969839271
SFTPA1B	-0.001048707	6.000124784	0.96991289
ZNF354B	0.000966806	6.020845283	0.969936122
RFX3	0.000544668	6.071476847	0.970029939
LOC650909	-0.00107074	6.468732012	0.970065992
ANXA8L2	-0.000948096	6.288688004	0.97013148
KIAA1826	-0.002256951	8.940322435	0.970174654
FLJ35409	-0.000875342	6.001732509	0.97020103
SPP2	-0.001109935	5.930300606	0.970328328
CECR5	0.00177808	9.823878474	0.970452169
UGT1A10	-0.001031411	5.88659439	0.970521037
LOC653097	-0.000982029	5.990999242	0.97055655
SCT	-0.00132012	6.163679425	0.970556955
SMCP	0.001573269	6.142756638	0.970563461
ZNF551	-0.001024007	6.356839424	0.970565933
PCDHA6	-0.000726994	6.072612103	0.970574907
LOC644787	0.000894401	6.132728615	0.970655258
ADRA2A	-0.001139187	6.463194209	0.970667586
OR1N1	0.001129972	6.132394911	0.970704405
SLC6A16	-0.001546514	6.340281812	0.970987142
HN1L	-0.001706545	6.648391607	0.97115333
SEC11B	0.00085367	6.069692802	0.97116565
CXORF20	0.000747482	5.963728301	0.971305845
HIST1H3B	-0.00098219	5.921436556	0.971326892
XKR9	0.00094658	5.984177836	0.971421266
CASP14	-0.001033931	6.040307736	0.971528739
ARL10	0.000875467	6.032369657	0.971533614
CALCRL	0.002430763	7.391903755	0.971640301
DEFB119	-0.000741643	6.414564577	0.971705457
LLGL2	0.000419845	6.090004316	0.971749113
ZFYVE28	0.001008998	6.001751158	0.971855553

HSD17B2	-0.001001256	5.909079617	0.971867833
LOC652536	0.000774087	6.016668913	0.971879202
SAMD5	0.000929475	6.111593765	0.971935241
FAM169A	-0.000709745	6.094567982	0.971977063
EPHA1	0.000860977	6.043523178	0.972014882
LGALS13	-0.001160334	6.149072145	0.972056391
LOC652676	-0.000954919	6.124741217	0.972106855
SUV420H2	0.001502119	6.006472276	0.972175502
PHKA2	0.001768527	8.06199636	0.972176493
LOC652774	0.00089414	6.016074171	0.972182204
ARL4A	0.001652805	6.991684602	0.972231955
LOC648553	0.000870991	6.003080139	0.972239158
LOC646569	-0.000904483	6.009034557	0.972259746
TMED6	-0.000984625	5.996109116	0.972435824
DNAJB2	-0.001799251	9.522401998	0.972593319
LOC652150	-0.000908553	6.075612878	0.972596633
LOC653321	0.003051068	6.018139502	0.972634828
KCTD11	-0.001055676	6.611544367	0.9727368
C15ORF43	-0.000720488	6.172248357	0.972761838
OVCH2	-0.000937867	5.957329154	0.972832722
LOC647904	-0.000873822	6.089603642	0.972914239
CDH20	0.000926237	6.327664121	0.973069766
MIS12	0.001622809	8.345613609	0.973114925
C2ORF3	0.001412003	6.063652523	0.97317288
CDC2L1	-0.001313906	6.744155201	0.973174902
LOC401860	0.001144149	6.083548337	0.973190797
CHRNA2	0.000925293	6.301568794	0.973191664
LOC643388	0.000816438	6.218276611	0.973315228
WISP3	0.000516185	6.102860926	0.973624938
MAGEC3	-0.000458865	5.992661531	0.973650969
KRTAP12-3	0.000819847	6.052308388	0.973715284
CXORF15	-0.000991118	6.263683538	0.973729239
CCDC87	0.000871597	5.965795347	0.973790054
LOC649425	0.000830293	6.041816289	0.974029751
TBC1D12	0.000804515	6.188055222	0.974042396
PDZK3	0.000874421	6.179636044	0.974081537
CLDN17	-0.000776807	6.113061738	0.974189302
GGTL3	0.000875047	6.475913018	0.974210356
LOC401222	-0.000846393	5.951116623	0.974304175
TMEM16C	-0.000832884	5.948289819	0.974317279
LOC642815	0.000850489	6.540244105	0.974478583
TAS2R40	0.000774973	5.936260756	0.974658505
LOC650422	0.000899307	6.059737517	0.974717027
DOCK7	-0.000997951	6.98512881	0.974760496
ITGB3BP	-0.001194043	6.314754264	0.974859213
AKAP3	0.001009788	6.614751245	0.974880551
LOC653855	0.00083413	6.225778204	0.97489383
LOC652256	0.000677598	6.112047272	0.97502658
LOC644598	-0.000753248	6.062552608	0.975169114
C17ORF56	0.000929962	6.249450996	0.975283801
LOC652735	0.000712618	6.091829256	0.975312568

LOC645334	0.000767366	6.183505734	0.975447194
PLEKHA8	-0.001293201	6.010645054	0.975729601
MAP3K5	0.00194638	7.896732497	0.97595602
C18ORF58	0.00078452	6.039526644	0.975974299
L1CAM	0.000744711	6.573993908	0.976047423
LOC647972	-0.000784403	6.010138477	0.9762552
C20ORF197	-0.00078743	6.176272844	0.976358383
LOC652753	0.000762625	6.056514979	0.976384486
LOC146177	-0.000895325	6.516288206	0.976456168
LOC645438	0.0007524	6.077822271	0.976588506
GSTM3	-0.002541925	8.138034697	0.976607029
LOC400955	-0.000679274	6.059816498	0.976641334
LOC91431	-0.000957619	6.212767621	0.976661068
LOC647206	-0.000873877	6.032581584	0.976687576
LOC647400	0.000679573	6.112691469	0.976703957
LOC388381	0.000714801	6.062399841	0.976750738
LOC283152	0.000706312	6.064643768	0.976835138
LOC648979	-0.000690215	6.158815461	0.97692236
LOC54103	0.000981142	6.265198698	0.977024277
IGFL2	0.000887622	6.009550549	0.97703018
RFX1	0.001766016	7.349574019	0.97703555
OR1I1	0.000735089	5.987707936	0.977051931
NKX1-1	0.000408517	6.087669238	0.977063419
KCNK4	0.000556319	6.153060705	0.977116742
EFNA3	0.000684735	6.024269109	0.977195733
LOC643784	-0.000720427	6.058926547	0.977251019
C10ORF21	0.000752686	6.02886	0.977376929
LOC152663	-0.000730197	5.955569729	0.977627692
MAP7	-0.000903754	6.654922384	0.977773159
C10ORF82	-0.001058173	5.961811501	0.977839788
LOC649320	0.00063448	6.045504715	0.977865225
LOC650867	0.000701251	6.07361862	0.977867516
ADAD2	0.001099945	6.183271879	0.977986643
LOC343384	-0.00063767	6.055764397	0.978000388
RHO	0.00066549	6.004378687	0.978024483
LOC653061	0.00063465	5.886177563	0.978079654
CDR1	-0.00050705	5.978186663	0.978244158
ZCCHC16	0.000544607	6.15607211	0.978248331
LOC644241	-0.000800324	6.068103844	0.978281251
NOSIP	-0.001769432	9.550596945	0.978313343
KRTAP10-5	0.001023998	5.978063372	0.978324977
LOC652641	-0.000686627	5.939033541	0.978388696
LOC642130	-0.000634961	6.058301244	0.978518015
TRIM62	0.001156193	6.069260736	0.978520007
LOC649620	-0.000608121	6.204726958	0.978570204
ZIC3	-0.001076259	5.913070656	0.978642983
EME1	-0.000888924	6.130303537	0.978649509
LOC338586	0.000686864	5.939706425	0.978663482
LOC647961	-0.000790153	6.094486741	0.978788458
H1FNT	-0.000673466	6.112861876	0.97884599
LOC649686	-0.000661954	6.082050872	0.978884377

TNFRSF11A	-0.000813013	5.959962526	0.978906912
LOC654126	0.000368508	6.192360069	0.978927588
SSX4	-0.000330969	6.08475131	0.979169189
LOC652593	-0.000705952	5.978452282	0.979220083
GINS1	-0.000980929	6.01594541	0.979284024
LOC644889	0.000705689	6.172593597	0.979311706
LOC439914	0.000760911	5.960638058	0.979363252
LOC440956	-0.000619736	6.069642959	0.97945059
LOC389458	0.000727481	6.086067513	0.979489834
LOC650028	-0.000659371	6.066964801	0.979662897
EPS8L2	-0.000474732	6.045815898	0.979663916
LOC650210	0.000560866	5.969908861	0.97978151
XCL1	-0.000420808	5.975798661	0.979825101
LOC644222	0.00063637	6.20053963	0.979865741
OR10J3	-0.000657567	5.973526773	0.979869457
KARS	-0.000927843	8.762450459	0.979927929
LOC644782	0.000579163	5.985537449	0.980023947
KLK5	0.000430349	5.953370735	0.980171196
LOC642161	0.000536506	6.141269511	0.98021395
LOC644704	0.000617878	5.979753914	0.98024151
S100A7	0.000658092	5.896279428	0.980254755
MSX2P1	-0.000463041	6.057214992	0.980281502
LOC646041	0.00059964	6.067743704	0.980285263
LOC649466	-0.000617661	6.174579375	0.980342129
NDUFB2	-0.001273894	12.75045984	0.980343265
KRTAP19-1	0.00068934	5.964320205	0.980355114
F3	0.00282099	8.203962681	0.980386344
LOC644802	-0.000743887	5.893023195	0.980397704
LOC642229	-0.000583991	6.189016655	0.980426868
TBX21	0.001146739	5.996051117	0.980468662
FLJ44674	0.000706167	6.123457343	0.980483004
TRIM42	0.000646169	6.105307387	0.980597288
XPO5	-0.001066643	7.432270852	0.980636362
TRAF2	0.000869279	6.497726083	0.98064361
NDUFS5	-0.001023728	13.17157783	0.980643631
HABP2	-0.000608678	5.980137425	0.980665995
SPINK9	0.000481885	6.133185797	0.980695691
LOC642819	-0.000627766	6.088194943	0.980728144
DHRS13	0.001075272	6.193006802	0.980788238
LOC650280	0.000614879	6.023628454	0.98104093
LOC646573	0.00051815	6.140732069	0.981289491
DEPDC5	0.000414169	6.300541146	0.981315035
LOC652839	0.000623562	6.146522661	0.981349172
L1TD1	0.000800443	6.04132169	0.981376531
ABHD14A	0.001351774	8.313014578	0.981441884
GPR113	-0.000689464	6.428978999	0.981515496
OXTR	-0.000924395	6.12485495	0.98153489
NGDN	0.000871155	7.951708356	0.981694499
PIGY	0.001454647	10.3705286	0.981786575
KIAA0888	0.000378171	6.115129288	0.981808669
CMKLR1	-0.000679536	6.239456721	0.981868637

LACRT	0.000545524	5.977099516	0.981921408
HTR1D	0.000741113	6.188086431	0.982230811
ZNF417	-0.000643759	6.408628403	0.982422815
OR11L1	-0.000873436	6.878312399	0.982463335
LOC654259	-0.0006468	6.243133864	0.982503025
LOC389963	0.000565104	5.960111715	0.982595218
LOC342934	-0.000536006	6.218211993	0.982611749
CAPNS2	0.000597734	6.032039647	0.982622841
PAX4	0.000597843	6.185750409	0.982623498
LOC646038	-0.000702323	6.123435417	0.982632656
LOC646200	0.001065711	11.78653733	0.98265399
LOC650293	0.000465151	6.171879631	0.982741456
LOC647841	0.000768418	5.950274207	0.982775368
KIAA1614	-0.000609772	6.147111918	0.982793676
XDH	0.000587707	5.887006327	0.98282376
GLIS2	-0.000562147	6.022645776	0.982913509
NCAPH2	-0.000627546	6.536033468	0.982942569
DKFZP686K1684	-0.00047205	6.30914153	0.983004703
LOC646806	-0.000490255	6.059345477	0.983010694
LOC646040	0.000524799	6.025518623	0.983044278
LOC653428	0.000525542	6.09758501	0.983140693
NEURL	0.000625665	6.138203706	0.983204452
LOC729193	-0.000329034	5.969588872	0.983225644
CNBD1	0.000508189	5.923916889	0.983338149
CRHR1	-0.000623697	6.966080136	0.983614482
TPM1	0.001065935	13.83932126	0.983718973
RNF151	-0.000382459	5.933099781	0.983825573
CSRP2BP	0.000548362	7.413343057	0.983928781
CYP4F8	0.000538555	5.966624108	0.983951736
C1ORF104	0.000325986	6.317626809	0.984001391
LOC653253	0.000590622	6.355658774	0.984052523
KLHL35	-0.000656534	6.75769044	0.984067442
PATL1	-0.000936959	8.974682415	0.98409531
CA6	0.0005681	6.32554212	0.984146786
BRS3	0.000513023	6.030900264	0.984174499
HSPB8	-0.001414812	12.24499653	0.984228731
PCSK9	-0.000567789	6.130177398	0.984249274
KCNQ4	-0.000305613	6.041004643	0.984274079
LOC644074	-0.000498726	5.991096733	0.98427775
LOC388526	0.000455224	6.186290568	0.984327798
LOC127150	0.000520956	6.102739497	0.98438461
LOC284751	0.000443658	5.983339369	0.984386453
LOC646145	-0.000771058	6.218704186	0.98440786
MCCC2	0.001400413	7.383414675	0.984479886
GJB1	0.000583779	6.125946192	0.984503673
BFAR	-0.001224087	7.905420019	0.984509956
FGD1	-0.000760173	7.080161644	0.984713273
ZNF313	-0.001175269	9.825285575	0.984726902
LSR	-0.000410102	6.18637425	0.98477315
RIPK4	0.000527458	6.578501897	0.984779807
PARP6	-0.000470714	6.749103025	0.984932499

UNC5C	-0.000434461	6.46892932	0.984971288
LOC648699	0.000499959	6.113670889	0.985005136
TFAP2B	0.000342197	6.018597066	0.985088635
KLC3	0.00026764	6.039531003	0.985185397
DKFZP434L187	0.000578009	6.063456064	0.985293238
LOC647343	-0.000435902	6.02939884	0.985334021
CBR1	0.00073495	7.348634498	0.985339406
TEKT2	-0.000470775	6.276571505	0.985407901
LOC642194	0.000455966	6.056847406	0.985412058
LOC644262	0.000419117	5.958725751	0.985480337
SLC22A9	-0.00032857	5.999011517	0.985514506
PLEKHH3	-0.001101489	7.525589448	0.985557902
LOC441151	0.000514565	6.468446288	0.985719131
TTTY15	0.00057033	6.061911375	0.985743067
SLC22A10	-0.000252016	5.960743029	0.985744955
FAM156A	-0.000555443	7.265621183	0.985799999
LOC643189	-0.00040454	5.952939594	0.985820804
LOC100101120	0.000387444	6.076722127	0.98590218
LOC646582	0.00043707	6.008976868	0.986118885
ID2B	0.000536803	5.978985293	0.986223377
C5ORF36	0.000331402	5.982901675	0.986241114
LOC646130	0.000412426	6.086398182	0.98643248
NEO1	-0.000821265	7.559363827	0.986478074
MT1L	-0.000510109	6.162307889	0.986561935
DNAJC14	0.00055797	7.219294484	0.986635655
FBXL12	-0.000789112	6.574489959	0.986861243
ST8SIA6	-0.000382523	6.205444301	0.986904467
C14ORF165	-0.000531728	6.053258445	0.98703816
SMEK1	0.000470149	6.003761008	0.987048552
PECR	-0.000717074	6.727684207	0.987176423
LOC652119	-0.000410987	6.077995651	0.987182101
LMOD3	-0.001091063	9.728517649	0.987191887
LOC652077	0.00049942	5.870603806	0.987274971
C20ORF91	-0.000349174	6.130699295	0.987430055
IFIH1	0.001275742	7.394975299	0.987627114
RRH	-0.000343305	5.925913028	0.987669658
EHD2	0.000818767	7.334258062	0.987688332
TRIAP1	-0.000538745	7.982065058	0.987699379
LOC648111	0.000345865	6.024794433	0.987731761
LOC646943	0.000523134	5.942247199	0.987867698
CARD10	-0.000835268	8.258450872	0.987868656
IL2	-0.00037294	6.025631254	0.987977067
LOC645626	-0.0003409	6.112945357	0.988007003
PCDHGA6	-0.00036433	6.12374964	0.988106823
HEPACAM2	-0.000231411	6.038021761	0.988151583
ZNF99	0.000433376	6.230774878	0.9881535
SPANXB2	-0.000304364	6.038115197	0.988181263
LOC650390	-0.000325222	6.128084511	0.988744119
LOC651849	0.000441596	6.101555266	0.988773679
LOC653410	0.000341979	6.109782587	0.988793259
ZNF645	0.000354472	5.941401605	0.988885108

LOC651787	-0.000344205	6.171838645	0.988906928
TRPV6	-0.000200337	6.052616943	0.988929947
ZNF409	-0.000277084	6.008628598	0.989010361
LOC652610	0.000363287	5.986692888	0.989043456
LOC650482	0.000413375	5.971681976	0.989071464
CDH8	0.000392741	5.960622283	0.989178746
ONECUT2	0.00017116	6.050410783	0.989207283
FAM153A	0.000248307	6.127249421	0.989221428
LOC651213	0.00030981	6.234396362	0.989233725
FDXR	0.000477851	5.984984546	0.989289872
CSF2	0.000364789	6.006573136	0.989293061
GAGE2C	-0.000363566	6.086683235	0.989301397
KREMEN2	-0.000186548	6.133297708	0.989356116
HGSNAT	0.00031907	6.254500021	0.989373213
SCAND3	-0.000301347	6.134988944	0.989379716
LOC389217	-0.000282499	5.923535308	0.989409549
DEFA4	0.000367228	6.353741898	0.989435212
LOC441395	0.000374172	5.934185449	0.98944845
PCDHA11	0.000324662	6.204697824	0.989502817
KRTAP19-7	0.000409784	6.186309776	0.989519251
SLC22A8	0.000392597	5.943812243	0.989557209
XCL2	0.000360949	5.930224697	0.989897708
GFRAL	-0.000252411	5.941890469	0.98994447
LOC643426	0.000282138	6.011236326	0.990015723
LOC647335	0.000280178	6.129663683	0.990080536
C11ORF34	0.000219196	6.053631908	0.99010204
LOC729396	0.000317367	5.999104865	0.990179644
LOC651859	0.000426908	5.944862801	0.990336668
PKD2L2	-0.000288663	6.047498014	0.990378778
LIMS3	0.000307236	6.034986235	0.990392204
FAM40A	0.000569745	6.65827677	0.990401406
LOC642606	0.000334677	5.948636935	0.990505925
TAS2R13	-0.000280831	5.946951255	0.990510309
EPPK1	-0.00040386	6.015967992	0.99055174
LOC651137	0.000201553	6.217677983	0.990572444
GPR45	-0.000342819	5.920666487	0.990632877
ODAM	0.000349015	6.08502338	0.990883162
MIST	-0.000262031	6.100873781	0.99107338
FAM184A	0.000333747	6.364902816	0.99121377
LOC647680	-0.000281152	6.126749093	0.991215576
LOC651341	0.000283213	5.94011266	0.99133339
LOC643107	-0.000318066	5.937821237	0.991422006
MYEF2	-0.000425465	5.963946173	0.991503184
LOC400793	-0.000286992	6.042434694	0.991567925
LOC642587	0.000223677	5.974960702	0.991587276
TBC1D1	0.000346215	6.514906817	0.991594681
MRPL42P5	-0.000283146	6.104523508	0.991669612
FLJ16686	0.000233836	6.181002855	0.991824244
C9ORF100	-0.000164494	6.059190036	0.991928961
LOC642869	-0.000236783	6.037025418	0.991993417
SHISA4	0.000356362	6.482682501	0.992044222

LOC652661	0.00023252	6.099388017	0.992062496
ZNF141	0.000262289	6.033746471	0.992091593
RNF152	-0.000191589	6.154091807	0.99214409
SLC26A3	-0.000252131	5.961404059	0.992165591
LOC652350	0.000243927	6.11276418	0.992231415
LOC55908	-0.000251498	6.420210062	0.992311687
LOC651966	-0.000235506	6.052118335	0.992369076
C4BPB	-0.000205549	6.109965821	0.992407011
LOC653437	-0.000247945	5.971214362	0.992610211
NEUROD1	0.000214448	6.067660247	0.9926233
KIAA0125	-0.000224467	6.005637093	0.992658746
KCNMB3	0.000158573	6.330352156	0.992813346
LOC344967	0.000215628	5.941824259	0.992827836
LOC391609	-0.000225933	5.996142453	0.992939757
DEFB114	0.000152159	5.959841599	0.992957554
PGM5P2	0.000202598	6.154849194	0.993011587
FLJ14082	0.000261906	6.289712261	0.993067048
LOC344165	0.000219528	6.074827122	0.993071534
LOC653547	-0.000217259	6.054166249	0.993092249
C20ORF51	-0.000229709	6.232808228	0.993169921
CLPS	0.000217818	5.93972797	0.993180073
LOC442710	-0.00018389	6.020993208	0.993201652
FLJ16331	-0.000194661	6.000119982	0.993253764
C3ORF47	0.000212376	6.047489109	0.993347971
LOC643824	0.000149257	6.110801426	0.993412294
QRICH2	-0.000182342	6.020740038	0.993439825
ZMYND8	0.000275846	7.120878167	0.993508209
MUC20	0.000401505	6.335476089	0.993539684
TMED8	0.000229885	6.131728052	0.993601192
MDC1	-0.000542085	8.078388098	0.993714126
OTUD1	0.000832362	8.151634285	0.993877604
ZIK1	0.000200746	6.630312898	0.994013548
LOC642744	0.000174199	6.014278557	0.99405399
TDGF3	0.000167442	6.002427732	0.994103037
MUC5B	0.000156565	6.101221774	0.99416971
FKSG44	0.000228811	6.05431469	0.994179828
FABP9	-0.000124469	6.01032858	0.994256894
LMAN1L	0.000290889	6.31608676	0.994328078
ZBTB32	0.000165265	6.169072571	0.994346607
LOC645090	0.000179268	6.101334034	0.994348953
HCRT	0.00021323	6.238201142	0.994372536
AACSL	-0.000206736	6.260631164	0.994635557
COPA	0.000342122	10.82282138	0.994646879
LOC391722	-0.000141912	6.03750039	0.994685517
KCNJ4	0.00054249	7.479535715	0.994712928
MMP13	-0.000176494	5.9566592	0.99476609
CCT8L2	-0.00018248	5.878175484	0.994772859
MGC34829	0.000157668	5.995277465	0.994945847
TRIM49	-0.000205355	7.049213823	0.994996934
DCPS	-0.000258295	7.040948278	0.995025876
PLSCR4	0.000397361	7.833396537	0.995114663

NOL9	0.000188552	6.113600618	0.995208621
POF1B	-0.000248901	6.026933872	0.995404626
LOC646603	-0.000118404	6.11290652	0.995466273
VCX2	6.12229E-05	6.058581595	0.99547683
FBXL8	0.000193953	6.350429601	0.995484057
LOC644388	-0.000141275	6.038038457	0.995499311
GDPD2	-0.000138073	6.123005274	0.995586391
LOC643605	0.000137507	6.043953894	0.995905417
LOC401778	0.000117593	6.028785898	0.995929105
PWWP2	-0.000139082	6.205929377	0.995942889
LOC641964	-0.000105378	6.122913465	0.995957204
DNAH8	-0.000128681	6.086291211	0.996100553
STIM1	-0.00026541	7.218790121	0.996134388
C6ORF190	0.000144691	6.016612971	0.996171338
LOC652438	-8.23748E-05	6.099004207	0.996203105
C20ORF75	-0.000112344	6.175321627	0.996296835
ZBTB12	-0.000113122	5.918672511	0.996305357
LOC642348	0.000111249	6.211633986	0.996336639
LOC643799	-0.000116444	5.972033516	0.996608547
GLDC	-9.52367E-05	5.957091573	0.996809146
SMUG1	0.000153887	8.555872589	0.996811639
LOC391352	0.000110918	6.300151921	0.996822122
MGC35030	9.37911E-05	6.043008999	0.996868956
OR10J1	-6.96525E-05	6.213267087	0.996961324
LOC401703	-9.51329E-05	6.109769159	0.996966463
LOC646082	-8.01787E-05	6.152520374	0.996977155
CCDC83	-0.000104871	5.875446954	0.996995364
LOC642233	9.27803E-05	6.273067104	0.997207174
MTL5	-6.87249E-05	5.977786645	0.997290776
OR12D2	-9.63455E-05	6.156099326	0.997343931
TPSG1	-7.80097E-05	6.058652414	0.997660024
DAAM1	0.000166992	8.884410034	0.997745681
LOC643278	-7.47036E-05	5.961548304	0.997746042
CDK3	-6.14412E-05	6.238650482	0.997823761
RPE65	-6.94684E-05	5.973738701	0.997895393
LOC648823	6.5994E-05	5.986208948	0.997899633
MMP3	7.95247E-05	6.25351666	0.997980772
FZD10	7.39207E-05	6.155209412	0.998001425
PLLP	8.33915E-05	6.655410125	0.998171741
KRTAP4-7	-3.81608E-05	6.109131757	0.998187794
LOC645645	7.50302E-05	5.891655868	0.998257615
KCNK10	3.25477E-05	6.19755187	0.998385719
LOC651597	-4.50426E-05	5.967428096	0.998506145
DLGAP4	8.02879E-05	7.103904596	0.998533215
LOC730760	-5.59457E-05	5.982939457	0.998535361
LRRC66	6.03586E-05	5.858038396	0.998615881
LOC653264	3.07822E-05	6.110198389	0.998621785
RHOBTB3	7.83211E-05	9.92935754	0.99869974
LOC653421	-4.88436E-05	6.528790557	0.998794937
LOC646135	-4.83741E-05	7.428804797	0.999007546
ALOX12P2	2.37242E-05	6.032141989	0.999148512

LOC647928	1.95719E-05	6.157428676	0.999373117
TEC	-2.06007E-05	6.217661694	0.99947821
LOC440804	-1.58945E-05	6.193332766	0.999547369
UBAP2	-3.12542E-05	6.784012149	0.999635396
CASP5	7.78138E-06	6.026896231	0.999734311
ZZEF1	-1.46667E-05	8.355932917	0.999764419
NPBWR2	-3.52425E-06	6.204527585	0.999891964
KDELC1	-2.57909E-06	6.303420206	0.999926415

adj.P.Val	group	name
1.31125E-42	down	TUBA3E
1.05801E-41	down	CEBPD
1.21262E-41	down	TUBA3C
1.93398E-41	down	ZFP36
2.02818E-41	down	RASD1
1.7549E-40	down	TUBA3D
9.03533E-40	down	CDC42EP4
1.84907E-38	down	SERPINA3
1.6462E-37	down	AXUD1
8.52808E-37	down	S100A9
9.12961E-37	down	MYH6
1.6114E-36	down	FCN3
3.14881E-36	down	MT1M
7.19057E-36	down	LYVE1
9.01884E-36	down	MAP3K6
7.63454E-35	down	PRKCD
2.33699E-34	down	CD163
5.58981E-34	up	YPEL2
6.38051E-34	down	S1PR3
7.11333E-34	down	C1R
7.34986E-33	down	FPR1
2.26465E-32	down	FCER1G
5.38251E-32	down	FKBP5
6.83616E-32	up	PIK3IP1
8.95267E-32	up	AP3M2
9.17841E-32	down	MYC
1.65032E-31	down	MT1X
1.98916E-31	down	S100A8
5.98534E-31	down	MAFB
9.09577E-31	down	CMTM7
1.28404E-30	down	SLA
1.28404E-30	up	JAK2
1.70417E-30	up	SCN2B
2.02078E-30	down	FES
2.60585E-30	up	DYRK1B
5.87268E-30	down	ZDHHC9
5.87268E-30	down	GALK1
6.06155E-30	up	PROS1
6.16656E-30	up	CHN1
6.6773E-30	down	NFKBIZ
6.81649E-30	up	C7ORF41
8.43126E-30	down	TIPARP
1.11848E-29	down	SLCO4A1
1.40484E-29	down	LOC653888
1.83588E-29	down	CORO1A
3.26025E-29	down	ALOX5
5.03032E-29	down	CTSC
5.03032E-29	up	SORBS2
6.422E-29	down	CD97

6.67241E-29	down	XLKD1
2.23004E-28	down	STAT3
2.99682E-28	stable	SEC14L1
3.48195E-28	down	MT1A
3.69284E-28	down	PIM1
5.99931E-28	down	ARPC1B
8.77874E-28	down	IFITM2
8.96698E-28	down	HCK
9.84662E-28	down	EBI2
1.11032E-27	down	WAS
1.44797E-27	down	ITGB2
1.63945E-27	up	SMOC2
2.10448E-27	down	F13A1
2.18533E-27	down	CD248
2.29467E-27	down	RANGAP1
2.7998E-27	down	LCP1
2.84114E-27	down	IFITM3
3.73716E-27	up	SMYD2
4.16453E-27	down	HCLS1
4.96558E-27	down	SLCO2B1
4.96558E-27	down	IL18R1
5.94841E-27	down	BTG2
7.00224E-27	down	CHRDL2
8.14123E-27	down	MGST1
1.19652E-26	up	C10ORF71
1.30934E-26	up	IRX6
1.52383E-26	down	INPP1
1.77044E-26	down	IL13RA1
1.81072E-26	down	MGC42367
1.81072E-26	up	SAP18
1.96609E-26	down	CHST7
2.26216E-26	down	MFSD10
2.74118E-26	down	BCL6
5.79773E-26	up	LMOD2
5.89469E-26	down	NFKBIA
8.64388E-26	down	TUBB2C
9.09781E-26	down	LOC653879
1.20804E-25	stable	MNS1
1.21671E-25	down	C1QC
1.2368E-25	down	SLC15A3
1.306E-25	down	MT2A
1.36391E-25	down	NNMT
1.39812E-25	down	CYBA
1.77625E-25	down	C1ORF162
1.77625E-25	down	C1QB
1.78362E-25	down	RHBDF2
1.79123E-25	down	DOCK2
1.79123E-25	down	LAPTM5
1.8074E-25	up	ZNF415
2.22683E-25	stable	ZGPAT
2.39252E-25	up	HEG1

2.46458E-25	down	LRRC8A
2.53019E-25	down	CEBPB
3.22668E-25	down	CD14
3.74624E-25	stable	C5ORF4
3.86409E-25	down	FGD2
4.05986E-25	down	CD68
4.95262E-25	up	MBP
5.11258E-25	stable	ZNF627
5.33433E-25	down	S100A10
5.37897E-25	up	MASP1
5.67607E-25	down	TKT
5.76091E-25	down	VAMP8
5.9827E-25	down	TSPYL2
6.52119E-25	down	TDRD9
6.78435E-25	down	SRGN
7.13827E-25	down	ALOX5AP
7.13827E-25	up	FRZB
7.67005E-25	up	IVNS1ABP
7.93134E-25	down	LILRA5
9.03606E-25	up	C14ORF132
9.16317E-25	up	FAM110B
9.47685E-25	down	SLC12A9
1.00394E-24	down	HAVCR2
1.02868E-24	stable	MTCH1
1.04612E-24	down	GPR56
1.19821E-24	up	CCND2
1.47476E-24	stable	ARHGAP4
1.55137E-24	down	CAPN1
1.61349E-24	down	MVP
1.77642E-24	up	LYPLAL1
1.86364E-24	down	OSR1
2.24815E-24	down	CD209
2.35664E-24	stable	SYF2
2.55941E-24	up	CYP2J2
2.69815E-24	down	IFI30
3.44184E-24	up	ACE2
3.5406E-24	up	SORBS1
3.5406E-24	up	DDIT3
3.55761E-24	stable	LILRB5
3.65469E-24	up	ATPIF1
3.75385E-24	up	PRICKLE1
3.75924E-24	down	HES6
4.24537E-24	down	SEMA4B
4.53638E-24	stable	RASSF1
4.56395E-24	down	M6PRBP1
5.0759E-24	up	C8ORF47
5.29108E-24	down	NKD2
5.29108E-24	down	GPR172A
5.30491E-24	up	KIAA1853
5.74285E-24	down	OSMR
6.35782E-24	stable	PUS1

1.07062E-23	stable	ZNF187
1.08818E-23	stable	TCEAL8
1.08818E-23	down	TUBB
1.73407E-23	stable	INTU
1.73407E-23	down	GNA15
1.73407E-23	up	ANKMY2
1.87291E-23	down	SELS
1.879E-23	up	SDSL
1.90489E-23	stable	UCHL5IP
2.33773E-23	stable	GATA2
2.37258E-23	up	HMGN2
2.49546E-23	stable	VPS41
2.80592E-23	up	C5ORF23
3.15773E-23	down	GRASP
3.17883E-23	up	YPEL1
3.25983E-23	stable	CNDP2
3.84129E-23	up	LOC148915
4.12973E-23	up	SVIL
5.03676E-23	down	MAP3K8
6.44551E-23	down	MIDN
7.34439E-23	down	SGK
9.56471E-23	up	RASL11B
9.83475E-23	up	IRX2
1.67243E-22	up	PDE5A
1.77451E-22	down	MPP3
1.91104E-22	stable	C20ORF27
1.91378E-22	down	TUBA1B
2.21806E-22	up	PLCE1
2.47828E-22	stable	CSNK1D
2.53761E-22	stable	IMPDH1
2.78749E-22	down	VSIG4
3.27755E-22	down	FCGR2A
3.2959E-22	down	GFPT2
3.37065E-22	up	KIAA0141
3.39174E-22	down	DCUN1D3
3.42383E-22	down	DDIT4
3.50259E-22	down	LOC255783
3.54076E-22	up	LRRC49
3.72229E-22	up	SLC16A9
3.80146E-22	stable	EGLN2
4.28967E-22	stable	PPARG
4.64449E-22	down	PLEK
5.0338E-22	down	DUSP1
5.2301E-22	down	IRAK3
5.29089E-22	down	ADAP2
5.29089E-22	down	SLC39A1
5.41312E-22	down	FGR
5.44821E-22	down	PLTP
5.47173E-22	stable	TERF1
5.59847E-22	down	MARCO
5.92953E-22	stable	FERMT3

7.47541E-22	stable	SLC25A28
8.10407E-22	down	C1QTNF1
8.24651E-22	up	TRMT5
8.67825E-22	stable	ARS2
1.39244E-21	down	SLC7A1
1.53985E-21	up	CCDC28A
1.57529E-21	down	RARRES1
1.61371E-21	up	LOC730534
1.69758E-21	down	SERPINA5
1.80178E-21	up	SFRP1
1.84784E-21	up	C21ORF34
1.95472E-21	down	TOMM40
2.00829E-21	up	SLC47A1
2.02143E-21	down	SMAP2
2.05277E-21	down	BCL3
2.30284E-21	down	LTBR
2.60378E-21	down	DUSP23
2.7104E-21	down	TYROBP
2.71706E-21	up	CRYM
3.08308E-21	down	HOPX
3.36166E-21	up	DUSP26
3.41162E-21	stable	BATF3
3.5443E-21	stable	PLAUR
3.61022E-21	down	PDIA3P
3.63899E-21	up	RGS4
3.68763E-21	down	MS4A7
4.42784E-21	stable	LOC644128
4.42883E-21	up	CCND1
4.73142E-21	stable	ICK
5.29257E-21	down	TUBA1C
5.48604E-21	stable	ITGAM
5.80352E-21	down	SNRPB
6.10799E-21	down	DDOST
6.13578E-21	up	EDNRA
6.64624E-21	stable	TJP1
7.11517E-21	up	COL21A1
7.12351E-21	stable	CAPRIN1
7.28931E-21	down	ECGF1
7.8606E-21	down	ERRFI1
7.93657E-21	down	METTL7B
8.18698E-21	down	PLEKHF1
9.16924E-21	up	PHLDB2
9.55741E-21	down	SH3BGRL3
1.0374E-20	down	C19ORF10
1.07955E-20	stable	GLA
1.1193E-20	down	MS4A6A
1.38939E-20	stable	FLJ20021
1.48536E-20	stable	HRASLS
1.5173E-20	stable	CDC42EP2
1.57519E-20	stable	NCF4
1.61518E-20	stable	EIF4G1

1.75008E-20	stable	SLC11A1
1.75008E-20	up	FRMD5
1.76833E-20	stable	OMA1
1.81527E-20	down	LOC642031
1.84652E-20	stable	EPB41L5
2.05566E-20	down	DHX37
2.33135E-20	stable	VPS45
2.69011E-20	down	KLF6
2.701E-20	down	SLC2A1
2.83328E-20	down	SEC61A1
2.87318E-20	stable	ZNF20
3.00226E-20	stable	HLTF
3.25651E-20	stable	SLC39A3
3.35085E-20	down	ENO1
3.37155E-20	down	LOC201140
3.47085E-20	stable	KYNU
3.47085E-20	up	ATP1A2
4.83381E-20	down	EML3
5.20707E-20	stable	LOC158376
5.56671E-20	stable	ATP6V1G2
5.85073E-20	down	TEAD4
5.9201E-20	up	KLHDC9
5.9201E-20	stable	FBXO38
5.9201E-20	down	TBXAS1
6.16325E-20	stable	TMEM189
6.211E-20	stable	SLC1A5
6.35751E-20	up	GATM
6.72894E-20	down	TNFRSF1A
6.79829E-20	down	SERPINH1
6.81308E-20	down	ELL2
7.08117E-20	down	ARPC3
8.00868E-20	down	LOC387763
9.04877E-20	down	COL6A2
9.04877E-20	down	HYAL2
9.30923E-20	down	ATP1A1
1.07E-19	stable	ORC3L
1.08072E-19	down	GSDMD
1.12253E-19	stable	RASSF9
1.13538E-19	stable	CRBN
1.16081E-19	down	LCN6
1.49878E-19	stable	CD53
1.50568E-19	stable	ACVR1B
1.61202E-19	down	ZNF787
1.92618E-19	down	ZYX
1.97522E-19	stable	HM13
2.1522E-19	stable	RGS19
2.32511E-19	stable	C3
2.34531E-19	down	RDH10
2.35781E-19	stable	KLHL12
2.46109E-19	up	C5ORF41
2.59105E-19	up	FZD2

2.61916E-19	stable	LOC729843
2.76699E-19	stable	PARVG
2.76699E-19	down	FKBP2
2.78313E-19	down	CYBB
2.90846E-19	down	ENDOGL1
3.0122E-19	stable	SLC37A4
3.08088E-19	up	PTPN11
3.15386E-19	up	DIO2
3.32474E-19	stable	ARHGAP24
3.39749E-19	down	NDUFA4L2
3.4589E-19	stable	ASPSCR1
3.57084E-19	stable	PGD
3.68736E-19	stable	ATP6V0C
3.69756E-19	stable	BEST1
3.69756E-19	stable	C19ORF51
4.1152E-19	stable	MERTK
4.23488E-19	down	GPR4
4.29705E-19	stable	PAFAH2
4.3951E-19	down	TMBIM1
4.71481E-19	up	GRM1
4.8031E-19	stable	CTSB
5.02259E-19	up	SYNPO2L
5.48097E-19	stable	TGFBR2
5.81928E-19	down	CHSY1
5.81928E-19	stable	NLGN2
5.91841E-19	up	HEPH
6.06757E-19	stable	TNKS1BP1
6.09057E-19	down	AGXT2L1
6.14287E-19	stable	ALG3
6.20658E-19	stable	LRRC1
6.74341E-19	up	NUP62CL
7.40277E-19	up	HTRA1
7.56796E-19	stable	RBPMS2
8.01372E-19	stable	TRABD
8.57845E-19	stable	DCLK1
8.99282E-19	stable	MKNK2
9.27285E-19	down	FKBP11
9.49007E-19	up	COL16A1
9.82234E-19	up	NAP1L3
1.00546E-18	stable	RAB28
1.07225E-18	stable	KIAA1191
1.12337E-18	down	DARC
1.23184E-18	stable	MME
1.25837E-18	stable	MAPK13
1.31237E-18	stable	C21ORF51
1.31237E-18	stable	TMBIM6
1.3871E-18	down	NBPF20
1.47364E-18	stable	HSPA1L
1.49814E-18	stable	HPCAL1
1.57748E-18	stable	DNAJC21
1.72105E-18	stable	JAG2

1.75144E-18	stable	NPL
1.7674E-18	stable	TSPYL3
1.77892E-18	stable	ALS2CR13
2.0742E-18	up	HMGB2
2.077E-18	up	LUM
2.13039E-18	stable	ATL3
2.15508E-18	down	PLA2G2A
2.23584E-18	up	VWC2
2.46958E-18	down	RRBP1
2.64084E-18	up	S100A13
2.64546E-18	up	HFE2
2.74784E-18	stable	RPN1
2.86891E-18	down	HGS
3.04929E-18	up	ECM2
3.04929E-18	stable	NIPA2
3.09215E-18	up	EXTL1
3.19374E-18	stable	FAM125A
3.23268E-18	up	CBX7
3.42868E-18	up	SLC35F1
3.53256E-18	up	C14ORF179
3.6365E-18	stable	BATF
3.8234E-18	up	C5ORF46
4.12982E-18	up	KIAA0644
4.43329E-18	stable	ENTPD6
4.48691E-18	down	SNAI2
4.6457E-18	down	OKL38
4.95342E-18	up	APCDD1L
4.99405E-18	stable	MEIS1
5.04749E-18	stable	UNC84B
5.10861E-18	stable	RNF149
5.10861E-18	stable	ZNF211
5.60791E-18	up	C1ORF142
5.64779E-18	down	HCST
5.78976E-18	up	C2ORF64
5.97911E-18	up	LOC728473
6.06737E-18	stable	DYNC2LI1
6.81823E-18	stable	LOC731486
7.0811E-18	stable	FBP1
7.32586E-18	down	RGMA
7.33253E-18	stable	N6AMT1
7.81183E-18	down	C19ORF59
8.10695E-18	down	BTBD14A
8.16669E-18	up	ASAH1
8.31118E-18	stable	CSF3R
8.3742E-18	stable	CIDEB
8.57683E-18	stable	APBB1IP
8.62695E-18	down	LPCAT3
8.87312E-18	stable	EPB41L3
8.87312E-18	stable	LMNB2
9.21908E-18	stable	HBP1
9.62128E-18	up	PLK2

9.68031E-18	stable	SERPINA1
9.93081E-18	stable	IL18BP
1.03591E-17	down	KCNK12
1.05678E-17	up	KLHL34
1.0725E-17	down	CDR2L
1.07601E-17	stable	OSGIN2
1.08531E-17	up	STK38L
1.14827E-17	stable	LILRB2
1.22729E-17	stable	SLC30A1
1.30999E-17	stable	C21ORF63
1.30999E-17	up	FBXO32
1.32436E-17	down	SERPINE1
1.49993E-17	up	LIN9
1.57613E-17	stable	CTPS
1.5843E-17	down	PLAU
1.69941E-17	stable	ZMYND12
1.76084E-17	stable	GPRASP2
1.76084E-17	stable	HK3
1.8696E-17	down	CD59
1.87755E-17	stable	NR2C1
2.05051E-17	down	ADCY3
2.17274E-17	down	LDHA
2.29499E-17	down	FAM107A
2.33442E-17	up	PKD1L2
2.36673E-17	stable	C1RL
2.43368E-17	stable	MED16
2.43368E-17	down	RNASE2
2.45476E-17	stable	PNRC2
2.45546E-17	stable	TICAM1
2.48361E-17	stable	FXR1
2.60803E-17	stable	LNX2
2.76453E-17	stable	C4ORF41
2.799E-17	down	FKSG30
2.799E-17	up	PPP1R3C
2.8123E-17	up	TRAK2
2.98421E-17	up	MDFIC
2.98421E-17	stable	THRA
3.11264E-17	down	CEL
3.27729E-17	down	OBFC2A
3.35145E-17	stable	ZMYND19
3.38753E-17	down	MTHFR
3.4507E-17	down	KTELC1
3.72882E-17	down	LOC285556
3.76248E-17	down	ACTB
4.0666E-17	down	EMILIN1
4.1497E-17	up	HSPA2
4.15752E-17	stable	AMZ2
4.19834E-17	down	PRKCQ
4.58229E-17	stable	PTBP1
4.60538E-17	stable	FAM102A
5.26191E-17	stable	LGMN

5.27196E-17	stable	STXBP2
5.30793E-17	down	AIF1
5.35586E-17	up	FYCO1
5.45739E-17	stable	ARF3
5.57385E-17	down	PDK4
5.58893E-17	up	TNNT1
5.84257E-17	down	ISG20
6.11017E-17	down	FCN1
6.4027E-17	down	KLF4
6.57759E-17	stable	GRB2
6.60019E-17	up	THBS4
6.62294E-17	down	PHGDH
6.6337E-17	down	MTE
6.97395E-17	stable	LILRA2
7.04465E-17	stable	TEAD2
7.23775E-17	stable	ARPC4
7.49147E-17	down	RAC2
7.58271E-17	down	IFI16
7.73725E-17	stable	SLC31A1
7.94882E-17	stable	SBNO2
8.0404E-17	up	IGSF5
8.0404E-17	stable	UBN1
8.12402E-17	stable	FAM46A
8.14565E-17	up	TMEM140
8.28815E-17	stable	APOBEC3F
8.7547E-17	stable	LMNA
8.93504E-17	stable	C17ORF70
8.94906E-17	up	TMEM74
9.08923E-17	stable	ZNF385A
9.15329E-17	stable	ARRDC2
9.17635E-17	up	SOX4
9.19621E-17	stable	LOC654264
9.22085E-17	up	GPNMB
9.30704E-17	stable	POLR3H
9.36184E-17	stable	C5AR1
9.36184E-17	stable	EYA1
9.66991E-17	stable	FCGR1B
9.72768E-17	up	GLRB
1.00005E-16	stable	MKL1
1.00937E-16	up	C22ORF25
1.01339E-16	up	TPD52L1
1.01339E-16	down	ZNF189
1.02725E-16	stable	SLMAP
1.05732E-16	stable	GRRP1
1.10739E-16	stable	MST150
1.14776E-16	stable	ZMPSTE24
1.14776E-16	stable	TNFRSF1B
1.20526E-16	stable	CD276
1.20623E-16	down	CFD
1.24808E-16	up	OMD
1.25788E-16	down	PPP1R14B

1.2873E-16	down	GFOD1
1.32967E-16	up	CENPA
1.34399E-16	stable	ASCC3L1
1.42453E-16	up	C14ORF135
1.46463E-16	stable	PCK2
1.49137E-16	up	TSPAN32
1.49827E-16	stable	SERPINI1
1.50763E-16	up	TM7SF2
1.52743E-16	stable	TMEM14C
1.58768E-16	down	S100A11
1.60211E-16	stable	SIRT5
1.6266E-16	stable	KIAA1429
1.67413E-16	up	LOC401397
1.71081E-16	down	LOC399942
1.84873E-16	stable	TMEM51
2.06363E-16	down	LYN
2.18352E-16	up	SGIP1
2.27952E-16	stable	SPI1
2.29912E-16	stable	RHOG
2.34165E-16	stable	RCOR3
2.44468E-16	stable	STEAP3
2.46708E-16	stable	MGST3
2.47055E-16	down	APOLD1
2.56022E-16	stable	PYGL
2.6005E-16	stable	LOC643783
2.6879E-16	stable	FZD1
2.71399E-16	stable	MUC1
2.77223E-16	stable	HSPA4L
2.8831E-16	stable	CCNYL1
2.8831E-16	stable	TMEM149
2.98581E-16	stable	ZNF536
3.01709E-16	down	S100A12
3.05062E-16	stable	ZNF616
3.07559E-16	down	ATP2A2
3.15014E-16	up	C10ORF110
3.22897E-16	up	KCNA5
3.28245E-16	stable	DNAJC4
3.32421E-16	stable	C12ORF47
3.40572E-16	down	RGS1
3.43443E-16	stable	ARHGAP9
3.71373E-16	down	ID3
3.73484E-16	down	LOC648024
3.77401E-16	up	FRMD3
3.96089E-16	up	IER3
3.96089E-16	down	C13ORF15
4.0824E-16	up	EDC3
4.09767E-16	up	GARNL3
4.0999E-16	stable	FAM129B
4.18223E-16	down	RNASE1
4.29231E-16	down	SMTNL2
4.4479E-16	stable	SNX13

4.4874E-16	stable	FGD3
4.74087E-16	stable	C1ORF105
4.77049E-16	down	PDGFRB
4.87748E-16	stable	CD33
4.97034E-16	down	SLC7A5
5.36556E-16	down	FAM46B
5.49952E-16	stable	PUF60
5.54737E-16	down	CLIC1
6.03709E-16	stable	FPR3
6.10333E-16	stable	B4GALT5
6.10333E-16	stable	GCA
6.44042E-16	down	SOCS1
6.45162E-16	stable	SLC31A2
6.45162E-16	down	SNF1LK
6.55664E-16	stable	LOC642897
6.80617E-16	stable	KLHL13
6.85245E-16	stable	YARS
6.94665E-16	up	TMCO3
6.97906E-16	stable	BRD8
7.2249E-16	stable	CACNA1H
7.41299E-16	stable	BDNF
7.90735E-16	stable	COMMD1
8.0305E-16	stable	FLJ35801
8.06613E-16	up	USP46
8.34185E-16	stable	MLKL
8.51268E-16	stable	PHF3
8.75149E-16	up	TMEM56
8.82769E-16	stable	ATPBD4
8.86913E-16	down	LHFPL2
8.97437E-16	stable	TRIM39
9.20658E-16	stable	DRAM
9.20658E-16	stable	MYADM
9.32595E-16	stable	LILRA3
9.53798E-16	up	XPO4
9.77951E-16	stable	TTYH3
9.81417E-16	down	CCL2
9.87934E-16	stable	PTCD2
1.08125E-15	stable	GPBAR1
1.12475E-15	stable	RNF20
1.15038E-15	stable	UNQ2541
1.15693E-15	stable	ZNF30
1.16333E-15	down	ARPC5L
1.17281E-15	down	ADFP
1.18276E-15	stable	HRK
1.19612E-15	down	SOX18
1.20734E-15	up	CXCR7
1.22192E-15	stable	IL15RA
1.22996E-15	down	ITPR3
1.23549E-15	down	MAGOH
1.24801E-15	stable	NEDD9
1.28708E-15	stable	FLJ23584

1.30404E-15	stable	KIAA1303
1.31602E-15	stable	UPF3B
1.31602E-15	stable	FLJ27354
1.34941E-15	stable	ZNF16
1.3957E-15	stable	PYCARD
1.41643E-15	down	GADD45G
1.41778E-15	stable	LRP5
1.46646E-15	stable	BBS4
1.50458E-15	up	ST8SIA5
1.50679E-15	stable	CSK
1.57478E-15	stable	ZNF502
1.57975E-15	stable	EIF4ENIF1
1.57975E-15	down	PTHR1
1.5824E-15	up	TLL2
1.61434E-15	up	TAF15
1.61678E-15	stable	C6ORF111
1.64605E-15	stable	FURIN
1.65417E-15	stable	POLR1E
1.66814E-15	stable	SUSD1
1.7172E-15	stable	NT5DC3
1.73057E-15	down	NBPF10
1.73059E-15	stable	TUBG1
1.80597E-15	stable	PAFAH1B3
1.80714E-15	down	ARHGDIA
1.91294E-15	down	LY96
1.91449E-15	down	LMCD1
1.95873E-15	stable	SPTBN1
1.96719E-15	up	PROM1
2.10698E-15	stable	FADD
2.14329E-15	stable	ZNF671
2.20651E-15	stable	LOC374395
2.22009E-15	stable	C14ORF28
2.2264E-15	stable	ETS2
2.24718E-15	stable	LARP7
2.29731E-15	down	NINJ2
2.31257E-15	stable	FGF1
2.31426E-15	up	C9ORF24
2.34416E-15	down	ADAMTS9
2.38978E-15	down	LOC647000
2.39006E-15	stable	CCR1
2.39006E-15	stable	MORN4
2.47088E-15	stable	HMOX1
2.67643E-15	stable	LRSAM1
2.88476E-15	down	CXCL2
2.96764E-15	stable	GLIPR1
2.98406E-15	up	MFN2
3.16781E-15	stable	CCND3
3.18804E-15	up	RBM20
3.21758E-15	stable	NLRP3
3.2432E-15	stable	MOAP1
3.70756E-15	stable	SKP1A

3.84109E-15	stable	UBE2J1
4.12481E-15	stable	COL4A5
4.13363E-15	stable	BTK
4.24441E-15	down	SLC7A7
4.26164E-15	stable	CX3CR1
4.30483E-15	down	WARS
4.47247E-15	down	SPOCK1
4.54146E-15	stable	CAPN3
4.67014E-15	stable	TSC1
4.75092E-15	stable	SLC2A6
5.1432E-15	stable	C3AR1
5.45607E-15	stable	KIAA1468
5.89791E-15	stable	PPM1G
6.04526E-15	down	PDGFRA
6.26253E-15	stable	MYD88
6.29769E-15	stable	PPCS
6.37772E-15	down	TIMP4
6.37986E-15	stable	NOL6
6.38232E-15	stable	PMM1
6.43029E-15	stable	C5ORF37
6.43029E-15	down	TUBA1A
6.60762E-15	stable	NMNAT3
6.60807E-15	stable	MYCN
6.83746E-15	stable	ZNF277
6.96469E-15	up	SNCA
6.97548E-15	up	SFRP4
7.25403E-15	stable	LANCL1
7.46702E-15	stable	PRDX6
7.58685E-15	down	AQP9
7.66471E-15	stable	UNC45B
7.71872E-15	stable	PLSCR3
8.09209E-15	stable	NOL1
8.10253E-15	down	FCGBP
8.12147E-15	stable	GAB1
8.1733E-15	stable	ZFP90
8.25748E-15	stable	LRCH4
8.25974E-15	stable	P2RY11
8.25974E-15	stable	IL4R
8.42462E-15	down	MAP2K1
8.51841E-15	up	CPT1B
8.67325E-15	up	GABRB1
8.90662E-15	down	TGFBR3
9.03449E-15	down	RDH5
9.12929E-15	stable	PNPLA4
9.13763E-15	up	MLLT11
9.4783E-15	stable	GRK4
9.70601E-15	up	HLF
9.93388E-15	stable	TMEM42
1.00161E-14	stable	ELL
1.00738E-14	down	EIF4A1
1.03897E-14	stable	LFNG

1.05859E-14	stable	SLC44A3
1.0951E-14	stable	MMP9
1.0951E-14	up	DCUN1D2
1.0951E-14	stable	KIAA1370
1.12214E-14	stable	PLA2G15
1.14283E-14	down	CCDC109B
1.19826E-14	stable	REXO1
1.21297E-14	down	NPTX2
1.23709E-14	stable	PTPRM
1.25307E-14	up	CLIC6
1.25684E-14	stable	SAMSN1
1.2865E-14	stable	CTF1
1.30237E-14	stable	ZNF564
1.30646E-14	stable	MDP-1
1.3083E-14	up	FLJ21986
1.31025E-14	down	PVR
1.31179E-14	down	TAP1
1.3843E-14	stable	GAK
1.38539E-14	stable	C10ORF97
1.38539E-14	stable	CD300C
1.40533E-14	stable	ASB3
1.40903E-14	stable	PSMB8
1.42877E-14	down	SLC16A3
1.42877E-14	stable	YTHDC1
1.43549E-14	stable	MTHFD2
1.43706E-14	stable	VARS
1.46399E-14	stable	UCRC
1.47217E-14	up	TMEM71
1.49447E-14	stable	FAM89A
1.55733E-14	stable	CDKL3
1.56668E-14	down	LRRC32
1.58549E-14	stable	CCDC41
1.60993E-14	stable	AKAP8
1.63512E-14	stable	ZNHIT3
1.68378E-14	down	PDE4D
1.69484E-14	stable	CD86
1.69546E-14	down	FASN
1.70627E-14	stable	SYK
1.70627E-14	stable	DCLK2
1.79253E-14	up	MYH7B
1.79912E-14	stable	STEAP4
1.82137E-14	stable	MS4A4A
1.91032E-14	stable	SH2D3C
1.94839E-14	stable	PDZD2
1.97604E-14	stable	CRSP2
2.0292E-14	stable	C11ORF74
2.04957E-14	down	SPPL2A
2.10394E-14	stable	CHMP5
2.10416E-14	stable	TPD52
2.12557E-14	stable	ABL1
2.14836E-14	stable	ANKRA2

2.17005E-14	stable	RCN1
2.19281E-14	stable	CDKN2D
2.24813E-14	stable	DNAJC15
2.2978E-14	stable	U2AF2
2.33825E-14	up	DPY19L2
2.34305E-14	stable	CLDND2
2.36688E-14	down	NAMPT
2.42326E-14	stable	ZNF664
2.44627E-14	stable	RRAGB
2.46978E-14	stable	CCDC121
2.52031E-14	stable	SEC23B
2.52031E-14	stable	TMUB1
2.52267E-14	down	PDE7A
2.52267E-14	stable	FMO4
2.53443E-14	stable	PVRL2
2.54627E-14	stable	RNASE6
2.60168E-14	stable	C14ORF147
2.72402E-14	down	CAP1
2.7845E-14	stable	NXT1
2.84593E-14	up	OXCT1
2.90573E-14	stable	FAM164A
3.0182E-14	stable	H2AFY
3.18323E-14	stable	SLC25A22
3.22268E-14	stable	PAAF1
3.23337E-14	down	TMED9
3.27479E-14	down	IGFBP4
3.31461E-14	up	CTSF
3.40016E-14	down	EMP3
3.44654E-14	stable	TRIM8
3.47278E-14	stable	ATP1B2
3.52653E-14	stable	RCC2
3.5512E-14	down	RAMP1
3.5512E-14	up	TYRP1
3.57221E-14	up	DUSP28
3.5938E-14	stable	SMARCD2
3.65764E-14	stable	RIN2
3.67593E-14	stable	ZBTB4
3.82885E-14	stable	H2AFZ
3.88969E-14	stable	GPSM2
3.90843E-14	up	SLC2A12
3.94624E-14	stable	C6ORF130
3.94767E-14	stable	C7ORF44
4.00906E-14	down	LOC650832
4.08705E-14	stable	TFDP2
4.09084E-14	stable	MDK
4.12213E-14	stable	LOC606724
4.15371E-14	stable	GMPR2
4.15673E-14	up	NPR3
4.17389E-14	up	KIAA1394
4.24042E-14	stable	APBB3
4.30496E-14	stable	SKAP2

4.41409E-14	stable	MAP2K5
4.45032E-14	down	HLA-DRB4
4.63189E-14	stable	BAIAP2L1
4.63189E-14	stable	FTL
4.63608E-14	stable	WDR48
4.88537E-14	stable	PTP4A2
4.88537E-14	stable	GYS1
5.03288E-14	stable	PPRC1
5.11198E-14	stable	ESRRB
5.19743E-14	up	MUM1L1
5.22648E-14	down	MTP18
5.3716E-14	stable	TMEM16A
5.37663E-14	stable	PSTPIP2
5.71605E-14	down	LOC730525
5.84575E-14	stable	HNRNPAB
6.11885E-14	down	S100A3
6.20232E-14	down	KLHL3
6.31706E-14	stable	RAB3IL1
6.36663E-14	stable	FAIM2
6.36663E-14	stable	DPH2
6.41685E-14	stable	POP5
6.55107E-14	stable	PPWD1
6.75042E-14	up	SSPN
6.80669E-14	stable	BOP1
7.23094E-14	stable	ZDHHC18
7.34561E-14	stable	RASSF2
7.47039E-14	stable	VPS8
7.47039E-14	stable	SLCO3A1
7.48737E-14	stable	KIAA1949
7.52524E-14	stable	TP53INP1
7.81695E-14	up	FNBP4
7.93545E-14	stable	C17ORF58
8.11977E-14	stable	POR
8.19846E-14	stable	PRMT2
8.2407E-14	stable	SLC38A3
8.31249E-14	up	ST8SIA2
8.58177E-14	stable	ZEB1
8.70712E-14	stable	C12ORF44
8.89306E-14	stable	ZBTB25
9.03639E-14	stable	ACYP2
9.21476E-14	stable	LETMD1
9.61113E-14	stable	STAB1
9.81141E-14	stable	ZNF17
9.94613E-14	stable	SLC36A4
9.99615E-14	stable	MPHOSPH8
1.00507E-13	up	LOC723972
1.0084E-13	stable	MYOM1
1.05507E-13	stable	TESK1
1.09949E-13	stable	AGTR1
1.09987E-13	stable	CFL1
1.11223E-13	up	LOC647346

1.12852E-13	stable	ZMAT1
1.14234E-13	stable	RAB7B
1.17396E-13	up	KCNJ2
1.18655E-13	stable	NSL1
1.21723E-13	stable	ICAM2
1.22822E-13	stable	PLAG1
1.2513E-13	stable	CAV3
1.26337E-13	stable	EIF2B4
1.2885E-13	stable	XPC
1.30911E-13	stable	DPM1
1.31819E-13	stable	DYRK4
1.32858E-13	stable	CRLF3
1.35451E-13	stable	GALNT17
1.40562E-13	stable	OCIAD2
1.41603E-13	stable	MFSD1
1.42105E-13	stable	OGFR
1.4708E-13	stable	LOC441019
1.47184E-13	stable	LEFTY2
1.49873E-13	down	TRIB1
1.49873E-13	up	PLA2G4C
1.57836E-13	stable	BAX
1.66368E-13	stable	GGT5
1.67123E-13	stable	FLAD1
1.67775E-13	stable	SLC40A1
1.6833E-13	stable	BLM
1.6905E-13	stable	GATAD2A
1.74612E-13	stable	FRMD4A
1.79791E-13	stable	CLEC10A
1.80865E-13	stable	LOC643888
1.82771E-13	stable	EFR3A
1.82824E-13	stable	RWDD3
1.8343E-13	up	SCRN1
1.84427E-13	stable	ST3GAL1
1.84696E-13	stable	KIAA0427
1.84971E-13	stable	ZCCHC5
1.87646E-13	stable	IK
2.0014E-13	stable	RNF145
2.09893E-13	stable	SOCS3
2.10203E-13	stable	FLJ20273
2.10203E-13	stable	PRR16
2.1099E-13	stable	LLGL1
2.13258E-13	down	ITGB4BP
2.30654E-13	down	LDLR
2.36634E-13	stable	TCTEX1D2
2.48737E-13	stable	SDCCAG1
2.51099E-13	stable	SHISA2
2.53299E-13	stable	ZBED4
2.53299E-13	stable	KIAA1217
2.67259E-13	up	FMOD
2.78102E-13	stable	M160
2.84281E-13	stable	STAMBPL1

2.8807E-13	stable	SCO2
2.96866E-13	stable	ZFP82
3.03115E-13	stable	C14ORF106
3.0634E-13	stable	ZNF404
3.06903E-13	stable	TAF1
3.16327E-13	stable	MTRF1L
3.1801E-13	stable	RCBTB2
3.18287E-13	up	MATN2
3.29273E-13	stable	SLC25A27
3.45811E-13	stable	ZRANB2
3.45996E-13	down	SAT1
3.51518E-13	stable	DOPEY1
3.59179E-13	stable	MAP6
3.61205E-13	stable	ZNF615
3.62287E-13	stable	ISCU
3.66131E-13	stable	GMPPB
3.66131E-13	stable	SLC24A6
3.73984E-13	stable	LMTK2
3.76216E-13	stable	CLEC4G
3.76216E-13	down	SERTAD1
3.76795E-13	down	TUBB4Q
3.94541E-13	stable	TMEM126B
3.98385E-13	stable	C9ORF125
4.04291E-13	stable	CCDC25
4.07024E-13	stable	MPDU1
4.07212E-13	up	LRRC39
4.07212E-13	stable	TBC1D16
4.15285E-13	stable	SH3GL1
4.15622E-13	stable	ISLR
4.26418E-13	up	SLITRK4
4.29306E-13	stable	E2F4
4.54034E-13	stable	SCUBE2
4.63954E-13	stable	RP9
4.78016E-13	stable	LOC730063
4.89426E-13	stable	RAB8A
4.92246E-13	up	PRRX1
4.92246E-13	stable	BRMS1
4.92246E-13	stable	FST
4.96088E-13	down	SPP1
5.0536E-13	stable	CDKN2AIP
5.05542E-13	stable	SEPSECS
5.21413E-13	stable	TMSB10
5.32734E-13	up	GLDN
5.37745E-13	stable	ATPAF1
5.3964E-13	stable	CATSPER2P1
5.40447E-13	stable	TRIM41
5.51423E-13	stable	PSPH
5.67326E-13	up	APOA1
5.84058E-13	stable	BBS2
5.86328E-13	stable	PITPNM1
5.88397E-13	down	LOC402221

6.06496E-13	stable	ZNF816A
6.41521E-13	stable	C17ORF62
6.43307E-13	up	COL7A1
6.43307E-13	stable	SLC11A2
6.4389E-13	stable	SLC3A2
6.489E-13	stable	FAM65A
6.50569E-13	up	C1ORF24
6.72508E-13	down	CYP4B1
6.89907E-13	down	IHPK3
7.03932E-13	stable	FAM98C
7.05533E-13	stable	HCFC1
7.09975E-13	stable	WDR1
7.33361E-13	stable	ZNF433
7.46205E-13	stable	PFN1
7.54557E-13	stable	NDP
7.94264E-13	stable	TMEM2
8.13199E-13	stable	LGTN
8.14736E-13	stable	TNFRSF14
8.1854E-13	up	MYOZ3
8.32635E-13	stable	BIRC3
8.52713E-13	up	ALDH5A1
8.70308E-13	stable	LAT2
8.70881E-13	stable	BCAT1
8.93429E-13	stable	MAP2K3
9.21418E-13	stable	KDELR2
9.39333E-13	stable	ALDH4A1
9.4201E-13	stable	TGIF1
9.46368E-13	stable	IFNGR1
9.63542E-13	stable	ADD3
9.65032E-13	up	GADD45A
9.8384E-13	up	SGCG
9.97808E-13	up	GJA1
1.02993E-12	stable	RAB5A
1.03233E-12	stable	DPH5
1.03484E-12	stable	PEX1
1.06107E-12	stable	SERTAD3
1.07109E-12	down	WWC1
1.08609E-12	stable	THAP6
1.10065E-12	stable	B3GNT8
1.13591E-12	stable	ARHGEF19
1.14358E-12	down	STS-1
1.15475E-12	stable	ZNF248
1.15821E-12	stable	ARMC4
1.15833E-12	down	TMEM43
1.18224E-12	stable	ADAL
1.19099E-12	up	MYOZ2
1.22946E-12	down	MT1G
1.25776E-12	up	IL17D
1.25776E-12	down	RGS2
1.26511E-12	stable	LOC492311
1.2694E-12	stable	AKR7A3

1.36621E-12	stable	LRFN3
1.38548E-12	up	OLFML2A
1.38642E-12	stable	CBR4
1.40604E-12	up	C20ORF26
1.44067E-12	down	ID1
1.45743E-12	stable	DNAJC19
1.47696E-12	stable	COL4A3BP
1.50612E-12	stable	KBTBD3
1.50771E-12	stable	MFNG
1.53602E-12	stable	ZKSCAN4
1.55651E-12	down	TNFAIP8L3
1.55748E-12	stable	TCIRG1
1.5741E-12	stable	LONRF2
1.58334E-12	stable	FBXO8
1.59068E-12	down	ITGA5
1.6126E-12	stable	ST3GAL2
1.61955E-12	stable	LIX1L
1.63378E-12	stable	MN1
1.63773E-12	stable	HYI
1.70907E-12	stable	PTRH2
1.70907E-12	stable	RNF103
1.71548E-12	down	ANKRD13A
1.80202E-12	stable	SPATA7
1.82495E-12	stable	CTNNA3
1.83254E-12	stable	KANK1
1.88567E-12	stable	TNPO1
1.9228E-12	stable	LOC401431
1.9228E-12	stable	U1SNRNPBP
1.92599E-12	stable	C10ORF11
2.03297E-12	stable	CDC37
2.06187E-12	stable	ST13
2.06759E-12	stable	SLC25A43
2.10149E-12	down	ALPL
2.10339E-12	stable	JMJD1C
2.18179E-12	stable	BAZ2B
2.20251E-12	stable	SNAPC5
2.23491E-12	stable	THAP9
2.2484E-12	stable	PEMT
2.31257E-12	stable	ANPEP
2.32002E-12	stable	NCDN
2.33917E-12	stable	PMS2L5
2.35621E-12	up	TPM3
2.38108E-12	stable	WDSUB1
2.53617E-12	stable	SCO1
2.57068E-12	stable	DAND5
2.57383E-12	up	DBC1
2.59352E-12	stable	WBP1
2.59886E-12	stable	RGS11
2.69788E-12	stable	PARP2
2.76063E-12	stable	BHLHB9
2.77211E-12	stable	ECOP

2.80045E-12	stable	AZIN1
2.80765E-12	stable	LRWD1
2.8502E-12	stable	RABGGTB
2.86745E-12	down	XBP1
2.93904E-12	stable	TRIM5
2.98005E-12	stable	MRFAP1L1
2.98914E-12	stable	AP1B1
2.99301E-12	stable	CCPG1
3.00183E-12	stable	PGA5
3.03219E-12	stable	EDEM2
3.10973E-12	down	PTDSS1
3.12861E-12	stable	PQBP1
3.14977E-12	stable	ZDHHC5
3.20143E-12	stable	CLK4
3.20143E-12	stable	RANBP10
3.22438E-12	stable	ARHGEF6
3.22438E-12	stable	SMARCD3
3.23434E-12	stable	FAM131A
3.23434E-12	stable	SETMAR
3.27204E-12	down	PICALM
3.27432E-12	stable	G6PD
3.31471E-12	stable	C9ORF114
3.33696E-12	stable	THSD4
3.37394E-12	stable	RNF214
3.4809E-12	stable	DAGLB
3.53112E-12	stable	TMEM150
3.53124E-12	up	SOX15
3.72468E-12	stable	RUNX1T1
3.7572E-12	stable	CBR3
3.85085E-12	stable	KIAA0040
3.85398E-12	stable	FLJ10986
3.91233E-12	stable	SIGLEC10
3.91466E-12	stable	TTC14
3.98708E-12	up	IFIT2
4.02764E-12	stable	IL17RB
4.03239E-12	stable	RGS6
4.0773E-12	stable	ZNF407
4.12243E-12	stable	ZSCAN5A
4.13865E-12	stable	TBC1D8
4.20044E-12	stable	RIPK2
4.22281E-12	stable	CRIPT
4.2572E-12	stable	KCNE1
4.38948E-12	stable	LOC63920
4.41036E-12	stable	C9ORF119
4.44389E-12	stable	IL7R
4.44794E-12	stable	FAM152B
4.46509E-12	stable	C19ORF22
4.52309E-12	stable	PIK3R1
4.54879E-12	stable	NAAA
4.58016E-12	stable	STOM
4.58432E-12	stable	GTF2F2

4.67579E-12	stable	ELA2
4.7832E-12	stable	ZNF323
4.78553E-12	stable	AGBL5
5.06972E-12	stable	UPF2
5.13441E-12	stable	C3ORF23
5.16603E-12	stable	WDFY2
5.1949E-12	stable	KIAA0831
5.22876E-12	up	C20ORF177
5.25235E-12	stable	TUFT1
5.3054E-12	up	SH3GL2
5.37871E-12	stable	SLC25A18
5.39725E-12	down	HSPA6
5.40485E-12	stable	SERPINB8
5.40529E-12	down	ATP1B3
5.53992E-12	stable	PCBD1
5.5486E-12	stable	PGBD4
5.73247E-12	stable	VSTM2L
5.92533E-12	stable	TUBB2A
6.06136E-12	stable	KIAA1383
6.06633E-12	stable	PLP2
6.09964E-12	stable	C10ORF26
6.36105E-12	down	SERPINB1
6.51419E-12	stable	SNX1
6.51419E-12	stable	SFRS9
6.55293E-12	stable	IAH1
6.55845E-12	stable	WDR4
6.64448E-12	stable	TEX12
6.77261E-12	down	FOS
6.82233E-12	stable	PILRA
6.844E-12	stable	CNKSR3
6.84949E-12	stable	ATM
6.8644E-12	stable	SPATA2L
6.89136E-12	stable	GAS2
6.98156E-12	stable	ZNF598
7.44638E-12	stable	AK3L1
7.44793E-12	stable	NUDT4
7.44988E-12	stable	ZFP3
7.44988E-12	up	FAM129A
7.47077E-12	up	TMEM30B
7.50134E-12	stable	AKR7A2
7.57355E-12	stable	ENAM
7.59326E-12	down	ALDH1A3
7.60436E-12	stable	FUNDC1
7.66315E-12	stable	ZMYND11
7.66996E-12	stable	DHX40
7.67995E-12	stable	VGLL4
7.92693E-12	up	RYR2
7.97982E-12	stable	HSPB7
8.70282E-12	stable	AMPH
8.90947E-12	stable	LOC541471
8.98986E-12	stable	ITGA3

9.13429E-12	stable	C7ORF53
9.24982E-12	stable	PAPD4
9.34935E-12	stable	LOC88523
9.56461E-12	down	NP
9.88056E-12	stable	KIAA1712
1.00375E-11	stable	GJA4
1.00619E-11	stable	C1ORF175
1.01739E-11	up	FNDC1
1.02099E-11	stable	SRPRB
1.03119E-11	stable	CNTNAP1
1.07489E-11	stable	DOK5
1.07733E-11	stable	TXLNA
1.08695E-11	stable	TOB2
1.08828E-11	stable	PKD2
1.10377E-11	stable	LPCAT1
1.10815E-11	stable	KLHDC6
1.10815E-11	stable	NDEL1
1.11667E-11	stable	C11ORF1
1.14974E-11	stable	FNIP1
1.16022E-11	stable	CAMK2B
1.19416E-11	down	MSX1
1.19883E-11	stable	LONRF1
1.21856E-11	stable	C4ORF18
1.22013E-11	stable	ZZZ3
1.22065E-11	stable	LOC390975
1.22487E-11	stable	ZNF706
1.27702E-11	stable	LCN12
1.27702E-11	stable	RARB
1.28007E-11	up	CTSK
1.28667E-11	stable	PRKCB1
1.30689E-11	down	KLF2
1.35109E-11	stable	ERCC5
1.36748E-11	stable	COX15
1.41348E-11	stable	MKRN2
1.42561E-11	stable	BYSL
1.46107E-11	down	COL6A3
1.46217E-11	down	STXBP6
1.46481E-11	up	GPR22
1.49545E-11	stable	GGH
1.49545E-11	stable	ZNF544
1.54731E-11	stable	ATP2C1
1.56734E-11	stable	CHST13
1.58746E-11	stable	WDR46
1.6058E-11	down	BST2
1.65621E-11	stable	TCF21
1.6778E-11	stable	NDFIP1
1.67797E-11	stable	RBPJ
1.73704E-11	stable	ARHGEF12
1.75469E-11	up	LOC644760
1.78222E-11	stable	GPR137C
1.80398E-11	stable	PFDN1

1.81851E-11	stable	SCAP
1.81851E-11	stable	C1ORF63
1.82378E-11	up	CASQ1
1.90145E-11	stable	LIN7B
1.92853E-11	stable	SLC1A7
1.93043E-11	up	RNF165
1.93043E-11	stable	CYFIP1
1.95054E-11	stable	RBM24
1.95188E-11	stable	MGEA5
1.96126E-11	stable	OSCAR
1.9797E-11	stable	UBXN2A
1.99308E-11	stable	TM9SF1
2.04889E-11	stable	FAM59A
2.05023E-11	stable	PGBD3
2.13295E-11	stable	ELF4
2.16937E-11	stable	ZNF540
2.18555E-11	stable	MPPE1
2.18555E-11	stable	CPNE8
2.18555E-11	stable	GTPBP6
2.18612E-11	stable	KIAA0922
2.18612E-11	stable	TIMM44
2.22889E-11	stable	PROK2
2.25451E-11	stable	CDK2
2.26912E-11	up	CA3
2.31396E-11	stable	CENPB
2.4068E-11	stable	TMEM49
2.41769E-11	stable	TMEM167B
2.4244E-11	stable	FBXL22
2.50589E-11	stable	ANKRD46
2.53027E-11	stable	MSRB3
2.61855E-11	stable	BAZ1A
2.7867E-11	stable	GLT25D1
2.78953E-11	stable	C12ORF39
2.81505E-11	down	IFITM1
2.85929E-11	stable	PDK3
2.87627E-11	stable	MYO9B
2.88878E-11	stable	NUP62
2.95438E-11	stable	KCNAB1
2.98593E-11	stable	B4GALT3
2.99911E-11	stable	TMEM144
3.10311E-11	stable	SLC25A44
3.25976E-11	stable	ZBTB47
3.3215E-11	stable	GNL1
3.32339E-11	stable	SEC15L2
3.35085E-11	stable	TNFRSF6B
3.35504E-11	stable	PFKFB3
3.3847E-11	stable	LOC440589
3.42324E-11	down	ANXA2
3.42469E-11	up	CMBL
3.44713E-11	stable	LOC651380
3.45449E-11	stable	LOC391692

3.52141E-11	stable	CX3CL1
3.52715E-11	stable	PSIP1
3.58812E-11	down	IL8
3.63181E-11	stable	VPS13A
3.682E-11	stable	WDR70
3.682E-11	down	CDKN1A
3.82102E-11	stable	GRIA3
3.82102E-11	stable	HDAC5
3.82102E-11	stable	HSF1
3.86564E-11	stable	FBXO3
3.92164E-11	stable	ELF1
3.92753E-11	up	LOC196549
4.00135E-11	stable	QSOX1
4.00715E-11	up	FLJ10781
4.10442E-11	stable	PLEKHH2
4.11852E-11	stable	SIPA1
4.17995E-11	stable	C1QA
4.18526E-11	stable	IRAK1
4.19582E-11	stable	CTSS
4.19582E-11	stable	C14ORF45
4.21685E-11	stable	OSTF1
4.23559E-11	stable	DUT
4.23559E-11	stable	LOC648500
4.33561E-11	stable	MID1IP1
4.49014E-11	stable	KAL1
4.54477E-11	stable	ZFP106
4.56227E-11	stable	TFE3
4.63368E-11	stable	FAM20C
4.68584E-11	stable	NFKB2
5.06629E-11	stable	LEPREL2
5.14858E-11	stable	TRAK1
5.20388E-11	stable	CCM2
5.21898E-11	down	CRISPLD2
5.22224E-11	stable	CYP2U1
5.3598E-11	stable	ANTXR2
5.40758E-11	stable	ACADM
5.43769E-11	up	PROX1
5.43807E-11	down	SCD
5.46757E-11	up	MFAP4
5.50742E-11	stable	KIAA1737
5.57092E-11	stable	NCOA7
5.63917E-11	stable	MAEL
5.65404E-11	stable	PHACTR4
5.73307E-11	stable	OSBPL2
5.76902E-11	down	IL1RL1
5.80464E-11	stable	SH3D19
5.86281E-11	stable	ZC3H12B
6.05509E-11	stable	ACAD8
6.07385E-11	stable	MTMR10
6.1108E-11	stable	DYNC1LI2
6.13598E-11	stable	POLA2

6.1957E-11	down	ATF3
6.26376E-11	stable	ATF7IP2
6.35301E-11	stable	SLC35A2
6.35301E-11	stable	PSMD3
6.37477E-11	stable	FAM13A1
6.55023E-11	up	SLC30A2
6.56474E-11	stable	CDKN1B
6.57091E-11	stable	PREPL
6.72174E-11	stable	ZNF541
6.87034E-11	stable	C20ORF24
6.90199E-11	stable	KHDRBS3
6.90199E-11	stable	GOLSYN
7.01366E-11	up	DKFZP564O0823
7.08347E-11	stable	ZNF763
7.09557E-11	stable	NR2C2AP
7.19418E-11	stable	RABGAP1
7.25519E-11	stable	LIAS
7.27761E-11	stable	LOC285908
7.56016E-11	stable	DARS2
7.59692E-11	down	NME1
7.62206E-11	stable	ACTG1
7.64374E-11	stable	TNIK
7.67654E-11	down	CTSG
7.68542E-11	stable	PACSIN2
7.73974E-11	down	C17ORF91
7.80157E-11	up	SOCS2
7.88903E-11	stable	ABHD11
7.9275E-11	stable	ZNF302
8.00869E-11	stable	SLC9A3R1
8.00869E-11	stable	COPZ2
8.08411E-11	stable	OTUD4
8.21133E-11	stable	UBB
8.48803E-11	stable	IKBKG
8.50128E-11	stable	IRF8
8.54196E-11	stable	ORC5L
8.61842E-11	stable	EPAS1
8.72713E-11	stable	ZCCHC7
8.76911E-11	stable	GPATCH4
8.96151E-11	stable	ALDH6A1
9.01683E-11	stable	NOLA1
9.03282E-11	stable	ORC2L
9.10719E-11	stable	PROCR
9.15459E-11	stable	MARK3
9.21537E-11	stable	ORAOV1
9.37897E-11	stable	RAI2
9.3845E-11	stable	NT5C2
9.56297E-11	stable	SLC25A39
9.6909E-11	stable	TARS2
1.00485E-10	up	ACOX2
1.0249E-10	stable	RBMX2
1.02845E-10	stable	ETV6

1.0343E-10	up	MAP4
1.08765E-10	stable	THNSL1
1.09337E-10	stable	NFYC
1.09597E-10	stable	RGS17
1.10518E-10	stable	RNF144B
1.13764E-10	stable	HTR2B
1.13835E-10	stable	ZMYM4
1.13904E-10	stable	CASP4
1.16669E-10	stable	CMTM8
1.16831E-10	stable	DIXDC1
1.17993E-10	stable	CCDC130
1.18323E-10	stable	ADHFE1
1.18489E-10	stable	TCEAL7
1.19062E-10	stable	SURF4
1.19062E-10	stable	CCDC66
1.19799E-10	stable	NSDHL
1.20725E-10	stable	LOC730024
1.22179E-10	stable	CST3
1.22209E-10	stable	NRSN2
1.25421E-10	stable	KLF12
1.27451E-10	stable	ZNF518A
1.29056E-10	stable	ATAD3A
1.31604E-10	stable	GFM2
1.32168E-10	stable	RNF220
1.32925E-10	stable	OPN3
1.361E-10	stable	GRAP
1.36869E-10	stable	LEAP2
1.43028E-10	stable	PPP3CC
1.43301E-10	stable	CCDC91
1.45129E-10	stable	EPHA3
1.45645E-10	stable	CAPZB
1.53816E-10	stable	SLC6A9
1.54438E-10	stable	LOC203547
1.55634E-10	stable	DPAGT1
1.59551E-10	up	VAT1L
1.59575E-10	stable	TINF2
1.61175E-10	stable	FBXO31
1.62772E-10	stable	SOX17
1.654E-10	stable	SHISA5
1.66322E-10	down	LITAF
1.66508E-10	stable	ARHGEF3
1.66891E-10	stable	CNNM4
1.68715E-10	stable	IGBP1
1.69189E-10	stable	PSCD4
1.75185E-10	stable	ARAF
1.75189E-10	stable	C19ORF54
1.76106E-10	stable	SLC6A1
1.77271E-10	stable	C9ORF61
1.78743E-10	stable	RGS5
1.81809E-10	stable	MTHFD2L
1.8473E-10	up	HBEGF

1.8765E-10	stable	SLU7
1.88005E-10	stable	C17ORF85
1.89006E-10	stable	MAFG
1.9095E-10	stable	EVC
1.91397E-10	stable	AQP10
1.93595E-10	stable	NMB
1.96281E-10	stable	SMARCE1
1.96281E-10	up	FLJ35258
1.97448E-10	stable	TOR1AIP1
1.99274E-10	stable	EBI3
1.99327E-10	stable	C8ORF40
2.0103E-10	stable	CCDC112
2.02902E-10	stable	C6ORF91
2.06527E-10	stable	TSPAN12
2.07562E-10	stable	GLOD4
2.09429E-10	stable	SCAMP2
2.13743E-10	stable	NIP7
2.20975E-10	stable	ACADL
2.27259E-10	stable	ZNF135
2.29215E-10	down	IL6
2.31475E-10	stable	CORO1B
2.31936E-10	stable	PIAS2
2.37268E-10	stable	ITPKC
2.37558E-10	stable	TCEAL4
2.38778E-10	stable	CMIP
2.38778E-10	stable	ATPAF2
2.39911E-10	stable	LOC121952
2.42997E-10	stable	EIF3B
2.46388E-10	stable	ARV1
2.47324E-10	stable	NHLRC3
2.4846E-10	stable	DNAJC13
2.49564E-10	stable	CLPTM1
2.51047E-10	stable	NAT10
2.51047E-10	stable	UNQ1940
2.56439E-10	stable	ZSCAN18
2.59387E-10	stable	CD300A
2.59549E-10	stable	FAM92A1
2.60497E-10	stable	VGLL2
2.60779E-10	stable	C1ORF165
2.63305E-10	stable	SEPT9
2.63305E-10	down	TIMP1
2.63387E-10	stable	LOC400963
2.72397E-10	stable	INTS9
2.73843E-10	stable	ARMCX2
2.7776E-10	stable	LCMT1
2.81007E-10	stable	ATP6V1F
2.81552E-10	stable	PRKCH
2.83599E-10	stable	HARS2
2.83599E-10	down	TSC22D1
2.91156E-10	stable	C1ORF25
2.92912E-10	stable	THUMPD3

3.03645E-10	stable	TIMELESS
3.0609E-10	stable	RNF126
3.0609E-10	stable	YWHAQ
3.14255E-10	stable	GLT25D2
3.19323E-10	stable	CCL11
3.21997E-10	stable	TRUB1
3.27925E-10	stable	TP53INP2
3.28563E-10	stable	PFDN4
3.32149E-10	stable	TNFAIP3
3.33311E-10	stable	IL18RAP
3.33311E-10	stable	ZNF630
3.39276E-10	stable	MT1F
3.39306E-10	stable	C10ORF35
3.43446E-10	stable	SLC38A10
3.43591E-10	stable	C10ORF125
3.47316E-10	stable	KCNIP2
3.57527E-10	stable	EIF4A2
3.58804E-10	stable	CDC42EP1
3.59115E-10	stable	TTF1
3.59158E-10	stable	TUBB3
3.63952E-10	stable	CAMK1D
3.69706E-10	stable	FCGR1A
3.71509E-10	stable	MAPK7
3.74947E-10	stable	RAB23
3.7642E-10	stable	UAP1
3.80512E-10	stable	ENAH
3.83176E-10	stable	NEK1
3.87245E-10	stable	LOC652458
3.87715E-10	stable	PRIC285
3.93587E-10	stable	PDIA4
3.95675E-10	stable	CSDC2
3.99957E-10	stable	RAB5C
4.02426E-10	stable	FBXL2
4.03237E-10	down	ANXA5
4.06427E-10	down	LOC649143
4.11764E-10	stable	TNMD
4.11865E-10	stable	SPNS1
4.39052E-10	stable	CDH19
4.43434E-10	stable	UBE2W
4.45028E-10	stable	TSSC4
4.45723E-10	stable	FAM120B
4.51453E-10	stable	MYO1G
4.52205E-10	up	APP
4.56764E-10	stable	DNTTIP1
4.5716E-10	stable	PPP1R9A
4.57725E-10	stable	LOC199800
4.60625E-10	stable	SPTAN1
4.60625E-10	stable	FBXL6
4.64367E-10	stable	AKR1C4
4.69825E-10	stable	ARHGAP1
4.73503E-10	stable	FYB

4.75152E-10	stable	CCDC86
4.77644E-10	stable	MTMR15
4.79196E-10	stable	CD40
4.83311E-10	stable	HERC5
4.89011E-10	stable	IL1R2
4.89347E-10	stable	CLCC1
5.03173E-10	stable	IFT88
5.03294E-10	stable	CPEB1
5.03294E-10	stable	LOC441408
5.04179E-10	stable	ACOT1
5.07769E-10	stable	BRP44L
5.25225E-10	stable	C11ORF60
5.3772E-10	stable	RECQL
5.40624E-10	up	C21ORF7
5.42484E-10	stable	C2ORF34
5.47295E-10	stable	MGC40489
5.62976E-10	down	LIPG
5.62976E-10	stable	ARD1A
5.6827E-10	stable	VIL2
5.76462E-10	stable	BIRC6
5.7952E-10	stable	PSCD1
5.82903E-10	stable	TTLL7
5.85758E-10	stable	PCMTD2
5.94432E-10	stable	DCTN6
6.01698E-10	stable	ZNF653
6.03536E-10	up	HIST1H2AC
6.03536E-10	stable	KIAA1598
6.03829E-10	stable	TNFRSF10B
6.07642E-10	stable	NFE2L3
6.07642E-10	stable	ZNF235
6.18768E-10	stable	ARRDC3
6.21617E-10	stable	CLASP1
6.25949E-10	stable	C19ORF24
6.25949E-10	stable	CD37
6.32949E-10	stable	MAEA
6.36788E-10	stable	FLJ20444
6.47001E-10	stable	C9ORF6
6.54755E-10	stable	C10ORF76
6.59373E-10	stable	RCOR2
6.6454E-10	stable	SPIN3
6.68409E-10	stable	KIAA0194
6.73848E-10	stable	SSU72
6.73848E-10	stable	LRRC25
6.74032E-10	stable	RFTN1
6.74032E-10	stable	C7ORF36
6.76144E-10	stable	VPS37B
6.76599E-10	stable	ZNF177
6.82297E-10	stable	LOC285550
6.82297E-10	stable	TMEM100
7.01632E-10	stable	KCNA4
7.17752E-10	stable	LOC649679

7.19791E-10	stable	BID
7.25205E-10	stable	GNB3
7.25386E-10	stable	GPR116
7.28886E-10	stable	DDR1
7.32299E-10	down	IQCG
7.36116E-10	stable	PCDHB17
7.39565E-10	stable	TSPAN13
7.41697E-10	stable	TRMT11
7.49024E-10	down	JUNB
7.55113E-10	stable	LOC91561
7.55113E-10	stable	TXNDC1
7.55113E-10	stable	LOC730273
7.60804E-10	stable	ALS2
7.62412E-10	stable	THYN1
7.672E-10	stable	PMM2
7.79829E-10	stable	C13ORF33
7.90535E-10	stable	LOC728014
7.90723E-10	stable	IGSF1
7.96428E-10	stable	C6ORF153
8.00083E-10	stable	C1QTNF7
8.11867E-10	down	MT1E
8.22224E-10	stable	ARHGAP12
8.3327E-10	stable	BCL9
8.33933E-10	stable	AXL
8.35039E-10	up	MXRA5
8.39112E-10	stable	ZNF233
8.42807E-10	stable	GBGT1
8.47295E-10	stable	PAPSS2
8.54511E-10	stable	THAP10
8.6407E-10	stable	CPEB3
8.69382E-10	stable	RAB24
8.75426E-10	stable	DPP3
8.77757E-10	stable	SLC22A5
8.86472E-10	stable	LY86
8.95999E-10	down	CORIN
8.98761E-10	stable	C14ORF159
9.15063E-10	stable	ADAM15
9.15063E-10	stable	MYL1
9.21526E-10	stable	DAK
9.23132E-10	stable	RSC1A1
9.23132E-10	stable	PCCB
9.3632E-10	stable	C3ORF54
9.36722E-10	stable	ATP13A1
9.49659E-10	stable	MBIP
9.6029E-10	stable	DHX32
9.6029E-10	up	IRF6
9.70026E-10	stable	GABRE
9.82243E-10	stable	PTGER2
9.92481E-10	stable	HAPLN1
9.96461E-10	stable	SPG11
1.00133E-09	stable	TRIM33

1.01071E-09	stable	TPM4
1.01288E-09	stable	SEMA4D
1.0165E-09	stable	EPB49
1.02513E-09	stable	PPP1R1A
1.0267E-09	down	HAMP
1.04099E-09	stable	LOC201175
1.04331E-09	stable	SLC5A6
1.04718E-09	down	LYZ
1.04848E-09	stable	SMARCA4
1.07376E-09	stable	SENP5
1.09654E-09	stable	F2RL3
1.11129E-09	stable	KIF5B
1.11901E-09	stable	PSTPIP1
1.15035E-09	stable	RGL1
1.15557E-09	stable	TSPYL1
1.15679E-09	stable	CCL13
1.17377E-09	up	LOC100008589
1.18974E-09	stable	KLHL36
1.19115E-09	stable	GNB1
1.19887E-09	stable	EDNRB
1.19887E-09	stable	NLGN1
1.19915E-09	stable	SETD8
1.22814E-09	stable	SBK1
1.23781E-09	stable	ALG13
1.25399E-09	stable	GPX1
1.25668E-09	stable	PKD1
1.25793E-09	stable	EIF2C2
1.27473E-09	stable	LOC441461
1.28373E-09	stable	TSKU
1.29011E-09	stable	NAPB
1.29222E-09	stable	ARHGEF17
1.30528E-09	stable	RNF146
1.30603E-09	stable	C2ORF33
1.30603E-09	stable	C21ORF69
1.30603E-09	stable	NOTCH3
1.3061E-09	stable	SERTAD4
1.30675E-09	stable	ING4
1.33102E-09	stable	FERMT2
1.33625E-09	stable	MPZL1
1.3692E-09	stable	CRYZL1
1.36936E-09	stable	SLC26A4
1.37892E-09	stable	ZNF23
1.40515E-09	stable	STK19
1.40796E-09	stable	GFPT1
1.43449E-09	stable	ZNF76
1.45169E-09	stable	MAN2B1
1.45368E-09	stable	FHL3
1.46068E-09	stable	ZNF503
1.46413E-09	stable	LAMA2
1.48486E-09	stable	ERMAP
1.48815E-09	stable	KIAA0460

1.5166E-09	stable	TMEM79
1.54948E-09	stable	TSC22D3
1.55637E-09	stable	C14ORF139
1.59571E-09	up	GCOM1
1.60026E-09	up	KLHL24
1.60214E-09	stable	LPAR4
1.61963E-09	stable	PDCD6IP
1.6207E-09	stable	KIDINS220
1.6283E-09	stable	SDF4
1.63073E-09	stable	C15ORF24
1.63702E-09	stable	SSR1
1.67407E-09	stable	C20ORF127
1.67719E-09	stable	CAP2
1.67719E-09	stable	C2ORF28
1.69806E-09	stable	GPR3
1.7009E-09	stable	TTC8
1.70386E-09	stable	FAM133A
1.70797E-09	stable	GABARAPL2
1.71796E-09	stable	RCCD1
1.72521E-09	stable	C16ORF30
1.73529E-09	stable	TTC37
1.73707E-09	stable	PITPNC1
1.75027E-09	stable	BACH2
1.76922E-09	stable	FBXO21
1.78431E-09	stable	LEF1
1.78722E-09	stable	ATXN3
1.80999E-09	stable	HEXA
1.81991E-09	stable	NIN
1.82904E-09	stable	PDE1C
1.86516E-09	stable	RAB8B
1.86564E-09	stable	PLSCR1
1.89783E-09	stable	FBXO18
1.91033E-09	stable	GLCE
1.95655E-09	stable	RPN2
1.95691E-09	stable	ZNF230
1.98193E-09	stable	RTKN
2.00418E-09	stable	MRPS14
2.0045E-09	stable	EPHB4
2.03213E-09	stable	JAM3
2.07913E-09	stable	SLC25A25
2.08192E-09	stable	RPL29
2.10428E-09	stable	TRIM45
2.11114E-09	down	PTPN1
2.13113E-09	stable	MMP23A
2.13184E-09	stable	LIME1
2.18297E-09	down	SDF2L1
2.18297E-09	stable	SPRY1
2.22219E-09	stable	TOB1
2.24879E-09	stable	KIAA2010
2.27788E-09	down	SLC2A3
2.28135E-09	stable	ATP7B

2.28135E-09	stable	PDE4DIP
2.29299E-09	stable	TRPC1
2.30078E-09	stable	MYO19
2.32112E-09	stable	PCMTD1
2.33875E-09	stable	PER3
2.34707E-09	stable	C1ORF212
2.34707E-09	stable	HESX1
2.37409E-09	stable	C1ORF183
2.38873E-09	stable	GSTA4
2.40203E-09	stable	TBC1D4
2.4035E-09	stable	ACLY
2.40487E-09	stable	GALM
2.43541E-09	stable	RECK
2.44301E-09	stable	RELB
2.44665E-09	stable	APBA3
2.47112E-09	down	IRX3
2.47112E-09	stable	ADAM11
2.47218E-09	stable	RASSF7
2.48932E-09	stable	OLFM1
2.48932E-09	stable	POLR3GL
2.49284E-09	stable	NOD2
2.49391E-09	stable	KRIT1
2.54576E-09	stable	PUS7
2.55357E-09	stable	ADC
2.56648E-09	stable	LEPROTL1
2.57898E-09	stable	JOSD1
2.66004E-09	stable	C10ORF33
2.69841E-09	stable	UBL7
2.69881E-09	stable	RWDD2A
2.85409E-09	stable	ACADSB
2.86906E-09	stable	FAM58A
2.8806E-09	stable	SND1
2.8806E-09	stable	HERC6
3.0206E-09	stable	LOC728037
3.07675E-09	stable	ATP1B1
3.08777E-09	up	LOC652097
3.17598E-09	stable	LOC644739
3.18604E-09	stable	LOH3CR2A
3.30163E-09	stable	KCTD18
3.32739E-09	stable	LOC653186
3.3396E-09	stable	GRAMD1A
3.33962E-09	stable	MYO5A
3.34984E-09	stable	DPY19L4
3.38738E-09	stable	ABCF2
3.4184E-09	stable	LMO7
3.49586E-09	stable	TCF25
3.54849E-09	stable	DNAJB12
3.54887E-09	stable	DOCK11
3.56311E-09	down	C3ORF52
3.61183E-09	stable	AGAP3
3.62838E-09	stable	FDFT1

3.62838E-09	stable	POLR2G
3.6407E-09	stable	C4ORF38
3.66484E-09	stable	P2RY2
3.68378E-09	stable	WEE1
3.6993E-09	up	BEX1
3.7037E-09	stable	MRPL35
3.71294E-09	stable	UGCG
3.73017E-09	stable	ZNF654
3.75695E-09	stable	MPP6
3.7871E-09	stable	PTN
3.7871E-09	stable	HACL1
3.79021E-09	stable	C4ORF29
3.79021E-09	stable	GPATCH1
3.79733E-09	up	PXDNL
3.83803E-09	stable	ACTN2
3.91867E-09	stable	ZNF366
3.95957E-09	stable	FAM38A
3.99976E-09	stable	BIVM
4.00882E-09	stable	EXOC2
4.06209E-09	stable	SEMA6B
4.06766E-09	stable	MURC
4.11034E-09	stable	ANK2
4.11219E-09	stable	DET1
4.12389E-09	stable	CBX3
4.14123E-09	stable	IKIP
4.1445E-09	stable	DOCK8
4.15664E-09	stable	NUP37
4.17864E-09	stable	ZNF626
4.17887E-09	stable	GRK5
4.22045E-09	stable	AP2S1
4.28284E-09	stable	LARP5
4.30157E-09	stable	EFHD2
4.32182E-09	stable	RASD2
4.34925E-09	stable	LYRM2
4.3544E-09	stable	RFX5
4.36912E-09	stable	CHI3L2
4.37046E-09	stable	DDX39
4.44939E-09	stable	SLC35A1
4.58044E-09	stable	OSGEPL1
4.59016E-09	stable	ARHGEF9
4.59078E-09	stable	VPS16
4.6432E-09	stable	ANKRD6
4.6432E-09	stable	DOCK1
4.6432E-09	stable	SEC61B
4.6432E-09	stable	AFF3
4.70006E-09	stable	MRTO4
4.70031E-09	up	RABGAP1L
4.71199E-09	stable	EIF4EBP3
4.74977E-09	stable	ABCG2
4.79921E-09	stable	QPCT
4.79921E-09	stable	GEMIN8

4.79921E-09	stable	POLR1C
4.80212E-09	stable	AFMID
4.80255E-09	stable	NCAM2
4.9374E-09	stable	FANCL
4.99864E-09	stable	RARA
5.01428E-09	stable	USP54
5.01428E-09	up	C1ORF188
5.01428E-09	stable	RAB6IP1
5.0832E-09	stable	GLT8D2
5.0903E-09	stable	PFAS
5.10401E-09	stable	NDUFA8
5.10953E-09	stable	C19ORF12
5.13097E-09	stable	CAT
5.21631E-09	stable	TXNDC5
5.27782E-09	stable	AMY2A
5.31233E-09	stable	UBAP1
5.31736E-09	stable	TAP2
5.35258E-09	stable	HCCS
5.39636E-09	stable	VDAC3
5.40871E-09	stable	STARD7
5.41241E-09	stable	IL11RA
5.44621E-09	stable	UNG
5.45623E-09	stable	AMHR2
5.53699E-09	stable	UBTD1
5.55749E-09	stable	C10ORF32
5.59435E-09	stable	ESM1
5.67301E-09	stable	PRR6
5.67345E-09	stable	SH3TC1
5.70279E-09	stable	LOC648147
5.82972E-09	stable	SPAG16
5.83217E-09	stable	SNTA1
5.83833E-09	stable	NUDT6
5.84087E-09	stable	ORAI3
5.86785E-09	stable	ZNF337
6.00012E-09	stable	MGC27348
6.04359E-09	stable	TMLHE
6.09945E-09	stable	PELI1
6.12289E-09	stable	RAPH1
6.15623E-09	stable	CXCL16
6.22021E-09	stable	IRF7
6.37424E-09	stable	TMEM165
6.48306E-09	stable	MLH3
6.50254E-09	down	AZGP1
6.54343E-09	stable	PHYH
6.56637E-09	stable	SPCS2
6.59435E-09	stable	KPNA2
6.7084E-09	stable	PLEKHB2
6.7084E-09	stable	FOLR2
6.71318E-09	stable	SFRS2IP
6.75423E-09	stable	AOX1
6.76157E-09	stable	ZNF594

6.78756E-09	stable	CAND2
6.79355E-09	stable	BMP6
6.82813E-09	stable	DIDO1
6.82813E-09	stable	SREBF1
6.90541E-09	stable	FABP5
7.03592E-09	stable	RRP9
7.05411E-09	stable	CHCHD6
7.05461E-09	up	ENO2
7.12459E-09	stable	VPS13D
7.13262E-09	stable	PRDX1
7.13262E-09	stable	MMP25
7.15783E-09	stable	CCRK
7.22895E-09	stable	AUP1
7.24278E-09	stable	CCRN4L
7.24278E-09	stable	SPC24
7.29409E-09	up	SULF1
7.32227E-09	stable	SDCCAG3
7.36051E-09	stable	B3GNT2
7.38556E-09	stable	DNAJB6
7.42277E-09	stable	FCGRT
7.43705E-09	stable	APITD1
7.43705E-09	stable	UBE2Q2
7.46947E-09	stable	PPP1R3A
7.50685E-09	stable	HOMER1
7.52455E-09	stable	ACYP1
7.5246E-09	stable	MGST2
7.5365E-09	stable	IL6R
7.64559E-09	stable	MRPS36
7.73398E-09	stable	PMPCB
7.80374E-09	stable	SRI
7.86904E-09	stable	FLJ46082
7.89393E-09	stable	SLAMF8
8.01196E-09	stable	ATXN10
8.11438E-09	stable	CYP39A1
8.24397E-09	stable	APLP2
8.31772E-09	stable	CXORF57
8.32396E-09	stable	RPP40
8.40214E-09	stable	GIT2
8.40214E-09	stable	MGC87042
8.40214E-09	down	CD44
8.54345E-09	stable	DUSP16
8.57737E-09	stable	KCTD5
8.68997E-09	stable	LOC344595
8.88547E-09	down	CYR61
8.93198E-09	stable	LOC201229
9.00324E-09	stable	BASP1
9.02733E-09	stable	SLC25A19
9.03854E-09	stable	NPC1
9.09221E-09	stable	FAR2
9.16888E-09	stable	PKP2
9.24827E-09	stable	YWHAH

9.34237E-09	stable	UBE2Q1
9.3757E-09	up	GEM
9.43385E-09	stable	FOXS1
9.45684E-09	stable	USP13
9.47338E-09	stable	CCDC147
9.52132E-09	stable	IGFBP5
9.66295E-09	stable	DTX2
9.66558E-09	stable	TAF9L
9.7082E-09	stable	DMTF1
9.78841E-09	stable	GALT
9.87848E-09	stable	HIP1R
9.97858E-09	up	LDB3
1.00754E-08	stable	SEMA5B
1.01388E-08	stable	C9ORF21
1.02391E-08	stable	KIAA1143
1.03942E-08	stable	TXNDC14
1.04279E-08	stable	RASSF4
1.04354E-08	stable	TNFRSF21
1.04438E-08	stable	CLEC16A
1.04793E-08	stable	CASZ1
1.05553E-08	stable	CCDC6
1.05643E-08	stable	TNRC6A
1.05712E-08	stable	SEMA6D
1.05743E-08	stable	LOC283953
1.06005E-08	stable	IMP4
1.07493E-08	stable	ZNF167
1.08932E-08	stable	C17ORF60
1.08983E-08	stable	RRM1
1.09216E-08	stable	CLTA
1.12811E-08	stable	ZFP2
1.13285E-08	stable	ZNF740
1.1384E-08	down	C1ORF117
1.14725E-08	stable	LARP6
1.15078E-08	stable	MYH9
1.15905E-08	stable	PRKAB2
1.17231E-08	stable	ATHL1
1.17875E-08	stable	TOR3A
1.18283E-08	stable	GMFG
1.19989E-08	stable	FICD
1.20411E-08	stable	CADPS
1.21351E-08	stable	APRT
1.23686E-08	stable	HIF1AN
1.24178E-08	down	ISG20L1
1.25015E-08	stable	ESAM
1.25097E-08	stable	GPAM
1.26935E-08	stable	SUB1
1.29482E-08	stable	TOM1
1.30691E-08	stable	SNX12
1.31627E-08	stable	NUP205
1.32143E-08	stable	DOK6
1.33146E-08	stable	CKLF

1.33359E-08	stable	SLC25A11
1.34049E-08	stable	PSME4
1.34661E-08	stable	SRBD1
1.34871E-08	stable	OSBPL3
1.35707E-08	stable	RHOB
1.36641E-08	stable	S100A16
1.37349E-08	stable	OGFRL1
1.37789E-08	stable	CMAH
1.3841E-08	stable	SLC35F2
1.39651E-08	stable	ZNF839
1.40181E-08	down	HEY2
1.40379E-08	stable	KBTBD7
1.41476E-08	stable	MKL2
1.42216E-08	stable	TNFRSF10A
1.42252E-08	stable	RFWD2
1.42367E-08	stable	LIMCH1
1.45675E-08	down	MUSTN1
1.50416E-08	stable	AACS
1.51706E-08	stable	TTC3
1.51779E-08	stable	PLEKHB1
1.51779E-08	stable	PTPN21
1.54512E-08	stable	KCNS3
1.55963E-08	stable	NFE2L1
1.56045E-08	stable	B4GALT1
1.56079E-08	stable	ADRM1
1.56164E-08	stable	TEX261
1.5738E-08	stable	ZNF641
1.58353E-08	stable	TCTA
1.61094E-08	stable	C9ORF10OS
1.62892E-08	stable	OPTN
1.65173E-08	stable	PFKFB4
1.65782E-08	stable	KIAA1128
1.67618E-08	stable	ANP32C
1.69661E-08	stable	PLCD3
1.70342E-08	stable	HMGCL
1.70372E-08	stable	DDO
1.71029E-08	stable	MGC35361
1.72231E-08	stable	SPIRE1
1.73771E-08	stable	DDX59
1.74376E-08	stable	EFCAB7
1.75742E-08	stable	VAMP1
1.76543E-08	stable	KCTD12
1.77045E-08	stable	CCDC92
1.78316E-08	stable	FHOD3
1.79888E-08	stable	TXNDC13
1.80307E-08	stable	GAS1
1.80986E-08	stable	PAICS
1.81511E-08	stable	THOC5
1.81511E-08	stable	GTF2H5
1.83403E-08	stable	HSPA5
1.84269E-08	stable	CPT2

1.84617E-08	stable	BMP4
1.85061E-08	stable	GLIPR2
1.86195E-08	stable	PPIL1
1.86761E-08	stable	WTIP
1.86761E-08	stable	FTSJ1
1.87258E-08	stable	MNDA
1.88415E-08	stable	CRELD2
1.89396E-08	stable	TUB
1.89797E-08	stable	FOSL1
1.93708E-08	stable	TFPI
1.957E-08	stable	ZNF83
1.96883E-08	stable	UBA3
1.96987E-08	stable	C10ORF78
2.01018E-08	stable	PSMB6
2.02774E-08	stable	FBXO4
2.03754E-08	stable	ZNF471
2.03839E-08	stable	MOBKL2A
2.04339E-08	stable	PFN4
2.06571E-08	stable	DBNL
2.06571E-08	stable	IL1R1
2.06902E-08	stable	RASL11A
2.07882E-08	stable	AGRN
2.0882E-08	stable	FAM173B
2.0882E-08	stable	MARCH5
2.08896E-08	stable	GNB1L
2.09626E-08	up	DPYSL4
2.1003E-08	down	TUBB6
2.11355E-08	stable	FAM96B
2.11355E-08	stable	HTRA3
2.11562E-08	stable	CADM1
2.14416E-08	stable	MYH10
2.14653E-08	stable	IRX5
2.16497E-08	stable	MOBKL2C
2.16835E-08	stable	NDST1
2.2006E-08	stable	CA14
2.24492E-08	stable	CCDC45
2.25139E-08	stable	TPSAB1
2.25935E-08	stable	ATG4D
2.25935E-08	stable	MYL6B
2.27722E-08	stable	SEMA5A
2.28282E-08	stable	BOLA2
2.28353E-08	stable	IPO11
2.32268E-08	stable	MGC4172
2.33866E-08	stable	EMR2
2.34361E-08	stable	LOC149448
2.36698E-08	stable	STARD8
2.36821E-08	stable	FRMPD4
2.39267E-08	stable	TAF6L
2.43447E-08	stable	ADAMTSL5
2.47105E-08	stable	CSF1R
2.48029E-08	stable	ZDHHC8

2.48029E-08	stable	P2RY5
2.52876E-08	stable	TRMT1
2.54727E-08	stable	DCAKD
2.55557E-08	stable	OBSL1
2.57483E-08	stable	PTPN12
2.57483E-08	stable	STX12
2.58246E-08	stable	POLR3F
2.58246E-08	stable	DEGS1
2.62588E-08	stable	PAOX
2.62588E-08	stable	LOC91461
2.67407E-08	stable	NNT
2.68268E-08	stable	SNORA64
2.68966E-08	stable	OGDHL
2.70672E-08	stable	RAD51AP1
2.70737E-08	stable	CLCN7
2.76622E-08	stable	ING3
2.83025E-08	stable	C20ORF46
2.83755E-08	stable	PRDX4
2.8439E-08	stable	CPSF4
2.87025E-08	stable	TESSP2
2.87609E-08	stable	PPP2CB
2.8961E-08	stable	LOC651309
2.93022E-08	stable	GAST
2.93765E-08	stable	GHITM
2.93765E-08	stable	RAB27A
2.93765E-08	stable	TRAF6
2.96932E-08	stable	MRPS6
2.97016E-08	stable	LOC388681
2.97831E-08	stable	ZNF204
3.00078E-08	stable	ADPGK
3.05506E-08	stable	AASS
3.06801E-08	stable	SLC9A9
3.08849E-08	stable	ATF5
3.08849E-08	stable	SYT15
3.08849E-08	stable	RCE1
3.10173E-08	stable	RP5-1022P6.2
3.10173E-08	stable	MLLT10
3.1021E-08	stable	LTA4H
3.16014E-08	stable	CORO6
3.16446E-08	stable	TMEM88
3.16684E-08	stable	LOC92270
3.16884E-08	stable	LACE1
3.17221E-08	stable	UBE2H
3.1799E-08	stable	C1ORF50
3.18715E-08	stable	PIGP
3.1979E-08	stable	PURA
3.22185E-08	stable	TMEM154
3.23564E-08	stable	FRAS1
3.26919E-08	stable	PPP2R5A
3.29191E-08	stable	RPUSD1
3.32275E-08	stable	FBXW11

3.32275E-08	stable	SLC39A11
3.32275E-08	stable	SCYL1BP1
3.34614E-08	down	GADD45B
3.35528E-08	stable	PTEN
3.40397E-08	stable	TNFAIP6
3.45704E-08	stable	RAP1GAP
3.49672E-08	stable	SVEP1
3.50541E-08	stable	ANKAR
3.52151E-08	stable	COMMD8
3.53999E-08	stable	ABCB4
3.54144E-08	stable	GRINL1A
3.55811E-08	stable	FKBP9L
3.61001E-08	stable	VPS26B
3.61693E-08	stable	FPR2
3.651E-08	stable	ENPEP
3.654E-08	stable	VPS52
3.67964E-08	stable	TOP1MT
3.69943E-08	stable	RNF219
3.69943E-08	stable	NCRNA00153
3.70351E-08	stable	NEXN
3.71469E-08	stable	GCNT2
3.76063E-08	down	TMEM158
3.77308E-08	stable	JMJD1A
3.78297E-08	stable	ADPRHL1
3.81595E-08	stable	PTGES2
3.82539E-08	stable	LOC653904
3.83464E-08	stable	CHIC2
3.83464E-08	stable	TMEM11
3.84961E-08	stable	FAM69B
3.87709E-08	stable	GPR34
3.89423E-08	down	TNC
3.93663E-08	stable	ABTB1
3.9683E-08	stable	ITGB3
3.97003E-08	stable	KIAA0672
3.97417E-08	stable	HTATIP2
3.99082E-08	stable	TTPAL
3.99984E-08	stable	GOPC
3.99984E-08	stable	PKIG
4.00197E-08	stable	LOC440733
4.06043E-08	stable	FXYD5
4.09636E-08	stable	TLN2
4.11609E-08	stable	RPL37
4.11653E-08	stable	ARID5A
4.11849E-08	stable	BRD7
4.12118E-08	stable	C11ORF46
4.12455E-08	stable	LYSMD4
4.127E-08	stable	SPHK1
4.19962E-08	stable	KAZALD1
4.21499E-08	stable	ASNS
4.21857E-08	stable	FLJ40142
4.25444E-08	stable	KIAA1160

4.3269E-08	stable	MRPL40
4.33372E-08	stable	SYVN1
4.36385E-08	stable	CTPS2
4.40201E-08	stable	MAGEE1
4.40409E-08	stable	SBF1
4.43484E-08	stable	PKNOX2
4.45532E-08	stable	HNRPUL1
4.46374E-08	stable	CLCF1
4.50507E-08	stable	HLA-B
4.50827E-08	stable	FLJ14213
4.50956E-08	stable	TGFA
4.52658E-08	stable	ZNF684
4.5698E-08	stable	PIP5K1C
4.57866E-08	stable	ZFPM2
4.60287E-08	stable	ALPK1
4.67551E-08	stable	ALDH16A1
4.68843E-08	down	RAB15
4.74707E-08	stable	SMPDL3A
4.81271E-08	stable	NDC80
4.82397E-08	stable	INTS2
4.83392E-08	up	UCHL1
4.84084E-08	stable	LOC652002
4.84484E-08	stable	TMEM147
4.87843E-08	stable	THOP1
4.8814E-08	stable	C17ORF61
4.93949E-08	stable	PSMD8
4.93949E-08	stable	THOC4
4.95552E-08	stable	PAK1
4.99221E-08	stable	PDE6B
5.0549E-08	stable	RAG1AP1
5.07829E-08	stable	LRIG3
5.08582E-08	stable	ACAT1
5.10233E-08	stable	LOC643961
5.22842E-08	stable	LAMA5
5.23757E-08	stable	TNFAIP2
5.33425E-08	stable	SF3B1
5.34511E-08	stable	STK40
5.35618E-08	stable	FLJ10081
5.38432E-08	stable	PRKX
5.40059E-08	stable	PBX1
5.41173E-08	stable	NOV
5.49249E-08	stable	RALB
5.49904E-08	stable	SRGAP1
5.50198E-08	stable	TXNDC12
5.52937E-08	stable	RABL4
5.53E-08	stable	CARM1
5.5951E-08	stable	EHD1
5.60618E-08	stable	CLASP2
5.65465E-08	stable	TADA1L
5.68194E-08	stable	NANS
5.69189E-08	stable	LOC652164

5.73548E-08	stable	BHLHB3
5.75863E-08	up	IFIT1
5.75863E-08	stable	ACAD10
5.77819E-08	stable	MTMR4
5.80215E-08	stable	FAT
5.87141E-08	stable	PPFIA4
5.88881E-08	stable	ZMIZ2
6.08975E-08	stable	DHRS7
6.0981E-08	stable	BSDC1
6.25889E-08	stable	JMJD1B
6.26189E-08	stable	PTPRR
6.29287E-08	stable	PDIK1L
6.31775E-08	stable	MTMR2
6.32902E-08	stable	POLE4
6.35337E-08	stable	STX19
6.36641E-08	stable	RAD17
6.39108E-08	stable	C14ORF133
6.44246E-08	stable	LOC440503
6.44678E-08	stable	EMP1
6.45334E-08	stable	RAGE
6.52344E-08	stable	NPHP4
6.52893E-08	stable	ABCG1
6.52986E-08	stable	MXI1
6.52986E-08	stable	DBP
6.57511E-08	stable	ATP1B4
6.76165E-08	stable	IFNAR2
6.76617E-08	stable	PPP1CA
6.82753E-08	stable	NOL8
6.97345E-08	stable	NAP5
6.99788E-08	stable	COL9A1
6.99788E-08	stable	PTPN4
7.0172E-08	stable	RFC1
7.0871E-08	stable	BMP1
7.08893E-08	stable	SDCBP
7.11529E-08	stable	KIAA0889
7.12387E-08	stable	FCGR2B
7.14435E-08	stable	C15ORF17
7.2016E-08	stable	SLC36A1
7.20248E-08	stable	PSD4
7.20248E-08	stable	REPS2
7.34222E-08	stable	SH3RF1
7.34606E-08	stable	FKBP4
7.36782E-08	stable	C1ORF213
7.55685E-08	stable	CD55
7.61242E-08	stable	SLC9A1
7.63882E-08	stable	CASD1
7.71211E-08	stable	TMEFF2
7.71934E-08	stable	C20ORF200
7.73291E-08	stable	RAB35
7.73291E-08	stable	HEATR1
7.78733E-08	stable	AGL

7.84421E-08	stable	WDR74
7.928E-08	stable	RAB26
8.02904E-08	down	LAD1
8.15288E-08	stable	SLC4A2
8.1554E-08	stable	GTF2E2
8.17792E-08	stable	WDR51A
8.24139E-08	stable	ZCCHC9
8.32187E-08	stable	SYNM
8.40594E-08	stable	MRPL10
8.40594E-08	stable	LOC644615
8.41123E-08	stable	YWHAZ
8.50622E-08	stable	GRWD1
8.56451E-08	stable	EXOC7
8.57842E-08	stable	RNASET2
8.60016E-08	stable	HNRPH3
8.66801E-08	stable	ARFGAP3
8.6832E-08	stable	DUS3L
8.69629E-08	stable	RRS1
8.7318E-08	stable	SDCCAG10
8.7657E-08	stable	FAM43B
8.90716E-08	stable	ICA1L
8.94817E-08	stable	NLRX1
8.95071E-08	stable	LOC646168
9.04018E-08	stable	ZCWPW2
9.2312E-08	up	COMP
9.40608E-08	up	AEBP1
9.45186E-08	stable	AARS
9.51666E-08	stable	ZCRB1
9.54724E-08	stable	SYTL2
9.5917E-08	stable	TNNI3
9.60917E-08	stable	HSD17B10
9.62389E-08	stable	FAM70B
9.6554E-08	stable	ARNTL
9.72623E-08	stable	LIPT1
9.74714E-08	stable	MED12
9.75912E-08	stable	GON4L
9.88298E-08	stable	CARKD
9.98187E-08	down	AMY1C
1.00284E-07	stable	STAG3L4
1.00358E-07	stable	ANAPC10
1.00778E-07	stable	REST
1.0145E-07	stable	ELF3
1.02242E-07	stable	ABHD5
1.02294E-07	stable	NEK6
1.02723E-07	stable	RYR3
1.02762E-07	stable	MIER2
1.03666E-07	stable	SLC6A6
1.04403E-07	stable	PPARGC1B
1.05349E-07	stable	PTGER4
1.06034E-07	stable	SLC30A9
1.07875E-07	stable	UACA

1.07875E-07	stable	TMEM205
1.07895E-07	stable	LGALS9
1.09406E-07	stable	PEX7
1.09511E-07	stable	ERCC6
1.09697E-07	stable	CC2D2A
1.10447E-07	stable	C3ORF1
1.11305E-07	stable	GNL2
1.11341E-07	up	PTPN3
1.11341E-07	stable	C10ORF104
1.11618E-07	stable	TARBP1
1.11975E-07	stable	MTHFD1L
1.15674E-07	stable	FAM108C1
1.16692E-07	stable	F8
1.17378E-07	stable	SASH3
1.21519E-07	stable	PRCC
1.21894E-07	stable	PDZD4
1.22636E-07	stable	OR51E1
1.22636E-07	stable	NACA
1.22726E-07	stable	RAB32
1.22894E-07	stable	ASRGL1
1.25307E-07	stable	ST6GALNAC4
1.25672E-07	stable	MGAT1
1.25981E-07	stable	INADL
1.27161E-07	stable	SOX11
1.27424E-07	stable	CRNKL1
1.28377E-07	stable	CIRH1A
1.29919E-07	stable	ARHGEF18
1.30178E-07	stable	LPHN3
1.30549E-07	stable	FAM73B
1.31092E-07	stable	IMPACT
1.32026E-07	stable	SCYL3
1.32055E-07	stable	C6ORF125
1.3248E-07	stable	ATP6V0A1
1.32493E-07	stable	C20ORF166
1.33178E-07	stable	TBC1D7
1.33616E-07	stable	PLCG1
1.34258E-07	stable	YPEL3
1.34491E-07	stable	LOC285359
1.34553E-07	stable	ZC3H14
1.34976E-07	stable	PI4KAP1
1.34976E-07	stable	PAK2
1.36382E-07	stable	KLHDC2
1.36745E-07	stable	CMTM6
1.36933E-07	stable	STK38
1.37926E-07	stable	WFDC1
1.37966E-07	stable	CHMP4B
1.38101E-07	stable	NRAS
1.40293E-07	stable	ALS2CR8
1.42491E-07	stable	FAM179B
1.43281E-07	stable	AKR1A1
1.43479E-07	stable	OPA1

1.46485E-07	stable	PFKFB2
1.47269E-07	stable	LOC643319
1.47557E-07	stable	CROP
1.48793E-07	stable	DIP2B
1.48863E-07	stable	TMEM116
1.49336E-07	stable	RHEB
1.51199E-07	stable	PRKCSH
1.51944E-07	stable	MPHOSPH9
1.5347E-07	stable	FAM50A
1.5347E-07	stable	MAP4K4
1.53976E-07	stable	FLJ45244
1.54523E-07	stable	PTTG1IP
1.56484E-07	stable	C17ORF44
1.56829E-07	stable	GMPPA
1.5705E-07	stable	RRAS
1.5754E-07	stable	TMTC1
1.58118E-07	stable	AMD1
1.58388E-07	stable	IGSF21
1.58586E-07	stable	IL10RB
1.58661E-07	stable	CD93
1.5909E-07	stable	PDSS1
1.59181E-07	stable	ENG
1.59464E-07	stable	PDZRN3
1.60028E-07	stable	S100A6
1.60174E-07	up	MYL7
1.6025E-07	stable	C11ORF17
1.61852E-07	stable	MMP23B
1.63128E-07	stable	OSBPL9
1.63611E-07	stable	SGPP2
1.64526E-07	stable	VRK1
1.64882E-07	stable	EIF6
1.6529E-07	stable	ATF4
1.66965E-07	stable	LOC653377
1.67012E-07	stable	NFE2
1.67012E-07	stable	FNDC4
1.6826E-07	stable	APOM
1.70721E-07	stable	PLEKHG3
1.71336E-07	down	SLC38A2
1.71336E-07	stable	AP2M1
1.71629E-07	stable	HSZFP36
1.72167E-07	stable	LZTS1
1.72962E-07	stable	WDR62
1.73579E-07	stable	CLNS1A
1.7428E-07	stable	CCNG2
1.74808E-07	stable	AP3S2
1.75587E-07	stable	FGF7
1.75758E-07	stable	AMT
1.76538E-07	stable	ST8SIA4
1.77531E-07	stable	PREX1
1.80029E-07	stable	FZD9
1.82367E-07	stable	LRRN3

1.84302E-07	stable	TRIM52
1.87393E-07	stable	MGRN1
1.88206E-07	down	THBS1
1.88221E-07	stable	TMEM55A
1.90089E-07	stable	C12ORF26
1.91903E-07	stable	OSGEP
1.91903E-07	stable	GCLC
1.94552E-07	stable	LOC648470
1.94592E-07	stable	PRR13
1.9717E-07	stable	CLK1
1.97891E-07	stable	PRPF4
1.98525E-07	stable	TSPAN9
1.99949E-07	stable	EFTUD1
2.00685E-07	stable	HINT3
2.0282E-07	stable	GOLPH3
2.02924E-07	stable	C7ORF23
2.03617E-07	stable	ST3GAL3
2.04901E-07	stable	LOC338758
2.05277E-07	stable	ABCC8
2.06918E-07	stable	LOC731895
2.07931E-07	stable	LST1
2.07931E-07	up	FSD2
2.08932E-07	stable	ABCE1
2.104E-07	stable	BCAS4
2.10454E-07	stable	ABCB6
2.11389E-07	stable	PDPN
2.13071E-07	stable	ACOX1
2.14679E-07	stable	BP75
2.15017E-07	stable	C15ORF58
2.17723E-07	stable	GBAS
2.17782E-07	stable	CITED2
2.18079E-07	stable	LOC647108
2.18288E-07	stable	YTHDF1
2.18406E-07	stable	ALDH7A1
2.19577E-07	stable	TMEM115
2.23039E-07	stable	MT3
2.23192E-07	stable	UFSP2
2.23399E-07	stable	ZNF426
2.23564E-07	stable	GPT
2.243E-07	stable	PCDH1
2.2655E-07	stable	ZNF350
2.27077E-07	stable	HAS2
2.27592E-07	stable	DDIT4L
2.27795E-07	stable	FREM1
2.30293E-07	stable	PANK1
2.3034E-07	stable	C16ORF35
2.33272E-07	stable	CYGB
2.34718E-07	stable	ZNF512
2.36985E-07	stable	C17ORF53
2.36985E-07	stable	CCDC55
2.46412E-07	stable	BMPR1A

2.46918E-07	stable	MAFF
2.51335E-07	down	DUSP5
2.52214E-07	stable	PLOD3
2.52772E-07	stable	GLIS3
2.54935E-07	stable	EVL
2.57542E-07	stable	TNNT3
2.57664E-07	stable	VAMP5
2.5809E-07	stable	ARPC1A
2.60061E-07	stable	MRPL45
2.60973E-07	stable	C9ORF9
2.61705E-07	stable	VNN2
2.61705E-07	stable	FLOT2
2.62759E-07	stable	SYT11
2.65384E-07	stable	PYCR2
2.67678E-07	stable	LOC644310
2.67989E-07	stable	NGF
2.67989E-07	stable	DHRSX
2.70177E-07	stable	DDX21
2.70177E-07	stable	LOC388588
2.71555E-07	stable	VIP
2.71669E-07	stable	DNAJB11
2.71854E-07	stable	PLEKHA2
2.71854E-07	stable	KBTBD4
2.72552E-07	stable	ZXDA
2.72857E-07	stable	HOXB1
2.77224E-07	stable	ABI3
2.77341E-07	stable	ROGDI
2.78913E-07	stable	HNRPM
2.82586E-07	stable	IQWD1
2.83118E-07	stable	GPR44
2.83961E-07	stable	TNFSF10
2.84184E-07	stable	STX2
2.84309E-07	stable	HDAC3
2.84384E-07	stable	NTN4
2.84463E-07	stable	DUS4L
2.85552E-07	stable	LOC93622
2.86137E-07	stable	ZNF550
2.87258E-07	stable	ARMCX6
2.88272E-07	stable	UPP1
2.90517E-07	stable	HAGH
2.90632E-07	stable	DYRK2
2.92906E-07	stable	ALS2CR4
2.92906E-07	stable	GPRIN3
2.94442E-07	stable	FLJ10213
2.96347E-07	stable	EFEMP1
2.96844E-07	stable	CDKN2C
2.98197E-07	stable	ZNF605
2.99581E-07	stable	ZNF493
3.02796E-07	stable	PTPN6
3.03511E-07	stable	ALG9
3.0399E-07	stable	SEPW1

3.18127E-07	stable	DNAJC24
3.20663E-07	stable	C1ORF190
3.21157E-07	stable	CTRC
3.24149E-07	stable	CHRD
3.24593E-07	stable	PEX16
3.25778E-07	stable	HMG20B
3.26622E-07	stable	SLC25A30
3.29082E-07	stable	ZNF133
3.36114E-07	stable	PLA2G7
3.39016E-07	stable	PERP
3.39016E-07	stable	SATB1
3.41123E-07	stable	BCL2
3.45822E-07	stable	PCDHGB4
3.47071E-07	stable	TUBB2B
3.51541E-07	stable	RNF150
3.52819E-07	down	KBTBD5
3.54299E-07	stable	PHF21A
3.56478E-07	stable	CP110
3.58001E-07	stable	EDA2R
3.58623E-07	stable	CNOT6
3.59161E-07	stable	KRTCAP2
3.59358E-07	stable	ZNF385D
3.60561E-07	up	LEPREL1
3.61995E-07	stable	RBM16
3.64214E-07	stable	DGAT1
3.64682E-07	stable	KCTD15
3.65809E-07	stable	PDE1A
3.67083E-07	stable	KLHL21
3.68194E-07	stable	PPAN
3.7074E-07	stable	CRADD
3.717E-07	stable	PDIA6
3.74887E-07	stable	RALGDS
3.75752E-07	stable	FLVCR2
3.7604E-07	stable	ROD1
3.78587E-07	stable	DDX56
3.78893E-07	stable	LOC400214
3.78971E-07	stable	ZNF701
3.80519E-07	stable	SLC25A38
3.81408E-07	stable	C20ORF54
3.82749E-07	stable	KRBA2
3.84024E-07	stable	ACSL5
3.89878E-07	stable	RBMS1
3.90281E-07	stable	NT5C3
3.92412E-07	stable	LACTB
3.96999E-07	stable	PHKB
4.0313E-07	stable	PTPRU
4.04641E-07	stable	TBC1D2
4.05117E-07	stable	SOBP
4.05185E-07	stable	IL20RA
4.05267E-07	stable	NOS3
4.06815E-07	stable	SEMA3F

4.12084E-07	stable	RGS16
4.12969E-07	stable	SH3PXD2B
4.13453E-07	stable	IFT122
4.14611E-07	stable	HIF1A
4.15519E-07	stable	ARID4A
4.15932E-07	stable	RNF14
4.16795E-07	stable	UBE2L6
4.17228E-07	stable	MYOCD
4.20184E-07	stable	CBY1
4.21927E-07	stable	KBTBD10
4.23398E-07	stable	LOC651302
4.28713E-07	stable	NCAPD3
4.29051E-07	stable	NIPSNAP1
4.32448E-07	stable	SLCO5A1
4.34382E-07	stable	RPS6KA1
4.41856E-07	stable	ECE2
4.42585E-07	stable	TPR
4.44092E-07	stable	DNAJC18
4.48122E-07	stable	SRP14P1
4.49651E-07	stable	B3GALTL
4.5189E-07	stable	MED22
4.58552E-07	stable	CIP29
4.6014E-07	stable	PTGES
4.73848E-07	stable	SLC22A4
4.76965E-07	stable	LOC150763
4.79356E-07	stable	SMAD9
4.80661E-07	stable	JAZF1
4.82948E-07	stable	TXLNB
4.95929E-07	stable	TATDN3
4.95929E-07	stable	NECAB1
4.97193E-07	stable	DENND2D
5.0313E-07	stable	POLR3D
5.07825E-07	stable	YIF1B
5.12666E-07	stable	GUCY1A2
5.12893E-07	stable	TM4SF18
5.13989E-07	stable	COL23A1
5.14216E-07	stable	NOSTRIN
5.14216E-07	up	TNNI3K
5.14216E-07	stable	GPHN
5.15412E-07	stable	PRKCZ
5.1793E-07	stable	MPL
5.20598E-07	stable	UBE2Z
5.20911E-07	stable	TDRD6
5.27646E-07	stable	CYP11A1
5.27646E-07	stable	SRRM1L
5.28366E-07	stable	HERC1
5.36235E-07	stable	SLC7A8
5.39045E-07	stable	LOC650737
5.42005E-07	stable	TNIP1
5.42905E-07	stable	GPA33
5.43425E-07	stable	CACNB3

5.43425E-07	stable	DSC1
5.54219E-07	stable	SRR
5.57548E-07	stable	KATNB1
5.57641E-07	stable	GYLTL1B
5.62461E-07	stable	ACOT7
5.66179E-07	stable	FIS1
5.68622E-07	stable	VPS29
5.69284E-07	stable	TLR8
5.72731E-07	stable	SPATA18
5.7438E-07	stable	KIAA1009
5.7438E-07	stable	RORC
5.77783E-07	stable	CABC1
5.78226E-07	stable	PDE4B
5.79798E-07	stable	SCRIB
5.80762E-07	stable	MARK1
5.87306E-07	stable	JTV1
5.90097E-07	stable	CSPG4
5.95014E-07	stable	M6PR
5.95027E-07	stable	BEX5
5.98781E-07	stable	FLJ20125
5.98955E-07	stable	LOC285074
6.04343E-07	stable	P704P
6.06835E-07	stable	C10ORF57
6.08603E-07	stable	ARHGDIB
6.0912E-07	stable	FECH
6.12057E-07	stable	METTL1
6.19213E-07	stable	RNH1
6.28327E-07	stable	GRM8
6.28865E-07	stable	DNMT1
6.36775E-07	stable	SFRS5
6.43855E-07	stable	SH2B1
6.43855E-07	stable	SLC4A7
6.44335E-07	stable	PRICKLE2
6.47417E-07	stable	ADA
6.51431E-07	stable	BRF2
6.58099E-07	stable	HLA-C
6.58807E-07	stable	CCL21
6.59615E-07	stable	IDS
6.6218E-07	stable	SUV39H1
6.63594E-07	stable	TAGLN2
6.65092E-07	stable	FLJ10246
6.67233E-07	stable	UQCR
6.67834E-07	stable	WDR66
6.7196E-07	stable	MCOLN1
6.72924E-07	stable	DHX15
6.72952E-07	stable	LOC643452
6.75057E-07	stable	PIP4K2B
6.75266E-07	stable	ASB5
6.75735E-07	stable	GPR146
6.77656E-07	stable	CCDC90A
6.88639E-07	stable	CAMK2A

6.98561E-07	stable	MRPL46
7.01241E-07	stable	GCLM
7.05016E-07	stable	SLAMF1
7.08738E-07	stable	CEBPG
7.09235E-07	stable	SHPK
7.17525E-07	stable	SNORD68
7.18077E-07	stable	COPE
7.18682E-07	stable	OAF
7.20082E-07	stable	AP4B1
7.2355E-07	stable	TSPAN17
7.2401E-07	stable	XPR1
7.24037E-07	stable	ABCF1
7.33344E-07	stable	MAPK9
7.35608E-07	stable	MRPS27
7.35608E-07	stable	ZFR
7.41715E-07	stable	ZNF442
7.44267E-07	stable	PPP1R3D
7.46096E-07	stable	CCDC115
7.46096E-07	stable	SEC24D
7.47518E-07	stable	HDGF
7.52055E-07	stable	ZNF197
7.56079E-07	stable	SUGT1
7.59984E-07	stable	FOSL2
7.61997E-07	stable	CKAP2
7.62945E-07	stable	STARD13
7.7282E-07	stable	PECI
7.76274E-07	stable	ZNF717
7.76951E-07	stable	RALGPS1
7.77796E-07	stable	COMMD10
7.81208E-07	stable	FAM122B
7.84862E-07	stable	NUDT5
7.8703E-07	stable	GRINA
7.88872E-07	stable	ASCC2
7.89466E-07	stable	S100P
7.90617E-07	stable	CFH
7.91205E-07	stable	MAOB
7.95533E-07	stable	TRIM23
7.99258E-07	stable	MSN
8.01442E-07	stable	TTC23
8.01992E-07	stable	LOC731950
8.04054E-07	stable	C5ORF30
8.07969E-07	stable	NPTXR
8.09905E-07	stable	RPL34
8.10241E-07	stable	ZFP36L2
8.12517E-07	stable	COMMD6
8.14152E-07	stable	SLC16A5
8.23945E-07	stable	CCDC141
8.2807E-07	stable	PYGB
8.29012E-07	stable	SRPX
8.38174E-07	stable	LOC400027
8.40035E-07	stable	COPS4

8.41373E-07	stable	RRP12
8.42536E-07	stable	ABHD10
8.50176E-07	stable	PPP1R1C
8.52993E-07	stable	VASN
8.55818E-07	stable	POGK
8.62726E-07	stable	LOC728452
8.69118E-07	stable	SMARCA2
8.76039E-07	stable	ENOX2
8.78746E-07	stable	ZNF10
8.82793E-07	stable	PRO0628
8.84778E-07	stable	LOC649049
8.90033E-07	stable	STK3
8.90859E-07	stable	SLC8A1
8.93016E-07	stable	LTBP3
8.9679E-07	stable	LOC728519
9.01719E-07	stable	CXCL6
9.14152E-07	stable	RPS6KB2
9.14481E-07	stable	TOMM40L
9.20774E-07	stable	C1ORF83
9.20902E-07	stable	LOC400890
9.21058E-07	stable	KIAA0240
9.2666E-07	stable	EFHD1
9.27636E-07	stable	C20ORF116
9.30723E-07	stable	HDC
9.39152E-07	stable	PEX19
9.39934E-07	stable	ZFP37
9.40069E-07	stable	JUND
9.45129E-07	stable	LOC145814
9.5545E-07	stable	WFDC3
9.60954E-07	stable	MYNN
9.66511E-07	stable	ADAMTS5
9.70368E-07	stable	LONP2
9.71381E-07	stable	ZNF610
9.771E-07	stable	CEP68
9.79499E-07	stable	C6ORF162
9.95612E-07	stable	ZNF175
9.97534E-07	stable	DAZAP1
9.98183E-07	stable	LILRB4
1.00693E-06	stable	ZNF271
1.00772E-06	stable	CTNND2
1.00931E-06	stable	C7ORF54
1.01097E-06	stable	TIMP2
1.01525E-06	stable	LOC645378
1.01525E-06	stable	LIPA
1.01525E-06	stable	NRM
1.01967E-06	stable	RAB4B
1.02812E-06	stable	AGPAT1
1.03213E-06	stable	TPST2
1.04146E-06	stable	C22ORF40
1.04521E-06	stable	LOC653171
1.05871E-06	stable	RALBP1

1.06629E-06	stable	SEH1L
1.07091E-06	stable	RPS2
1.07569E-06	stable	NVL
1.0769E-06	stable	POLR2C
1.07798E-06	stable	HSPC111
1.07991E-06	stable	RBM39
1.08059E-06	stable	ATP6V1D
1.08071E-06	stable	DYNC1H1
1.08377E-06	stable	DBT
1.09908E-06	stable	LOC646849
1.10147E-06	stable	MRPS16
1.10942E-06	stable	TK2
1.12491E-06	stable	VPS37C
1.12648E-06	stable	HDAC9
1.13096E-06	stable	CDC2L6
1.13442E-06	stable	SCRN3
1.13576E-06	stable	PPP2R3A
1.13576E-06	stable	LASP1
1.13706E-06	stable	LRG1
1.14261E-06	stable	MYST4
1.14728E-06	stable	GABARAPL1
1.15498E-06	stable	PIN4
1.15643E-06	stable	RIC3
1.15643E-06	stable	NPM3
1.15878E-06	stable	RHPN2
1.15984E-06	stable	CRIP3
1.17029E-06	stable	HADH
1.17066E-06	stable	MYOM2
1.17395E-06	stable	CREB5
1.18322E-06	stable	EDN3
1.18449E-06	stable	GRAMD4
1.19389E-06	stable	C6ORF203
1.19519E-06	stable	CDC25B
1.197E-06	stable	CYB5D2
1.2072E-06	stable	NCBP2
1.21078E-06	stable	CMYA5
1.21588E-06	stable	KIAA0232
1.24331E-06	stable	SIRT4
1.26391E-06	stable	MTA2
1.28126E-06	stable	BAG3
1.28398E-06	stable	CPA3
1.29179E-06	stable	ZNF680
1.29983E-06	stable	C3ORF60
1.30803E-06	stable	DACT3
1.30863E-06	stable	FBXW5
1.31155E-06	stable	C1ORF2
1.31881E-06	stable	TTC12
1.32874E-06	stable	ZNF285A
1.32989E-06	stable	PIK3C2B
1.33046E-06	stable	F5
1.33523E-06	stable	OLFML3

1.35407E-06	stable	MAD2L2
1.37064E-06	up	GUCA1C
1.37593E-06	stable	ZNF224
1.38067E-06	stable	ADORA3
1.38412E-06	stable	FKRP
1.39947E-06	stable	DLD
1.40137E-06	stable	GFOD2
1.40693E-06	stable	MBOAT7
1.41515E-06	stable	DC2
1.42109E-06	stable	LYPD1
1.42356E-06	stable	OXA1L
1.42857E-06	stable	CECR6
1.44342E-06	stable	CCDC8
1.472E-06	stable	TRPV2
1.47483E-06	stable	GPIHBP1
1.47518E-06	stable	TMEM135
1.47767E-06	stable	GAL
1.47908E-06	stable	PHF5A
1.49058E-06	stable	IRF2BP1
1.49101E-06	stable	KLF15
1.50643E-06	stable	LGALS8
1.50995E-06	stable	CSNK2A2
1.51058E-06	stable	CD69
1.51637E-06	stable	C11ORF31
1.53074E-06	stable	PLGLB1
1.53291E-06	stable	ANTXR1
1.53362E-06	stable	ABHD2
1.56071E-06	stable	GNA11
1.57967E-06	stable	NIPBL
1.58153E-06	stable	ARIH2
1.64882E-06	stable	CETN2
1.6542E-06	stable	FASTKD1
1.66156E-06	stable	FGD6
1.66277E-06	stable	HLA-DRA
1.66485E-06	stable	MSC
1.67597E-06	stable	AP2B1
1.67972E-06	stable	APOL3
1.68162E-06	stable	ANGPTL4
1.69003E-06	stable	C12ORF62
1.69436E-06	stable	SCOC
1.736E-06	stable	FEM1C
1.73809E-06	stable	TRK1
1.74432E-06	stable	MAP4K3
1.7722E-06	stable	GGNBP2
1.77613E-06	stable	DOLPP1
1.77879E-06	stable	CYBRD1
1.78584E-06	stable	WBSCR18
1.8467E-06	stable	TBC1D15
1.86788E-06	up	LOC147804
1.88419E-06	stable	TTC30A
1.90163E-06	stable	POPDC2

1.90236E-06	stable	SRGAP3
1.9042E-06	stable	STRA13
1.92411E-06	stable	LOC641705
1.95463E-06	stable	MANBA
1.95505E-06	stable	HSPC171
1.96114E-06	stable	CARHSP1
1.96866E-06	stable	SSR2
1.98647E-06	down	HIPK2
1.99526E-06	stable	MRGPRF
2.02156E-06	stable	NCKAP1L
2.02956E-06	stable	MRPS33
2.03379E-06	stable	PODN
2.04945E-06	stable	IER5
2.05881E-06	stable	GAB3
2.06232E-06	stable	ASB15
2.07297E-06	stable	SPAG11A
2.07975E-06	stable	EP400
2.09043E-06	stable	HIST1H4C
2.09498E-06	stable	C17ORF90
2.0969E-06	stable	DDX24
2.09758E-06	stable	EEF2K
2.09796E-06	stable	APLP1
2.09959E-06	stable	ARMCX3
2.11917E-06	stable	IFT172
2.13242E-06	stable	TCFL5
2.151E-06	stable	MAD2L1
2.15604E-06	stable	DCUN1D5
2.15705E-06	stable	NXNL1
2.15962E-06	stable	P2RX5
2.16466E-06	stable	CLPTM1L
2.17214E-06	stable	ZNF827
2.20088E-06	stable	LZIC
2.20292E-06	stable	NBN
2.22289E-06	stable	C1ORF35
2.2277E-06	stable	SNORA33
2.23584E-06	stable	SHMT1
2.23584E-06	stable	POMT1
2.24133E-06	stable	AFAP1L1
2.2513E-06	stable	SMCR7L
2.26143E-06	stable	WDR45
2.27176E-06	stable	MPHOSPH10
2.28296E-06	stable	DTX3
2.28751E-06	stable	ENPP5
2.29166E-06	stable	IL32
2.29854E-06	stable	FAM134B
2.31543E-06	stable	FAM100A
2.33376E-06	stable	FUT4
2.34006E-06	stable	PGA3
2.35022E-06	stable	C9ORF102
2.41865E-06	stable	ZNF704
2.42178E-06	stable	ACTR6

2.42762E-06	stable	CCDC53
2.45878E-06	stable	CD38
2.45975E-06	stable	ARHGAP19
2.45989E-06	stable	C17ORF87
2.47576E-06	stable	CFB
2.47691E-06	stable	C2ORF76
2.4772E-06	stable	MAP2
2.4819E-06	stable	ZFP30
2.48844E-06	stable	PDIA5
2.50997E-06	stable	RAMP3
2.5201E-06	stable	PHC1
2.5201E-06	stable	MRPL49
2.52028E-06	stable	USP48
2.52375E-06	stable	SGSM1
2.53288E-06	stable	LOC441268
2.54101E-06	stable	PCBD2
2.54442E-06	stable	WDR5
2.59912E-06	stable	NSF
2.59985E-06	stable	C18ORF10
2.60161E-06	stable	PDHB
2.62941E-06	stable	TCTEX1D1
2.65555E-06	stable	SEC31A
2.65629E-06	stable	QTRT1
2.67945E-06	stable	USP28
2.68407E-06	stable	PTPN14
2.68937E-06	stable	ADAT3
2.69851E-06	stable	PARVA
2.69986E-06	down	TFRC
2.71004E-06	stable	FBXL14
2.72247E-06	stable	ATF6
2.7361E-06	stable	NOPE
2.74356E-06	stable	ADCK2
2.75741E-06	stable	PRMT1
2.75741E-06	stable	WWC2
2.77879E-06	stable	GPS2
2.77879E-06	stable	PER1
2.785E-06	stable	ADD1
2.7871E-06	stable	MGA
2.7871E-06	stable	BZRAP1
2.79077E-06	stable	C17ORF80
2.79077E-06	stable	PPP1R10
2.79077E-06	stable	CRY2
2.79649E-06	stable	ADRA1B
2.79783E-06	stable	CMTM3
2.81596E-06	stable	NEBL
2.81636E-06	stable	GNG11
2.82624E-06	stable	PARP11
2.82675E-06	stable	NUB1
2.83398E-06	stable	TNFSF13B
2.84129E-06	stable	CHRAC1
2.84234E-06	stable	ADH5

2.84805E-06	stable	DBI
2.85905E-06	stable	ACAD11
2.87475E-06	stable	GOLPH4
2.87954E-06	stable	FAM48A
2.88648E-06	stable	LOC57228
2.8889E-06	stable	APEX1
2.89893E-06	stable	ELK1
2.90274E-06	stable	LOC653566
2.93994E-06	stable	GIMAP5
2.94663E-06	stable	GAP43
2.96974E-06	stable	NENF
2.98206E-06	stable	RBP1
2.98244E-06	stable	LOC731878
2.99879E-06	stable	SH3PXD2A
2.99879E-06	stable	ANO5
3.00142E-06	stable	SLC4A4
3.00432E-06	stable	CHN2
3.0221E-06	stable	TIGD7
3.05544E-06	stable	SEPT5
3.05544E-06	stable	RBM4B
3.05544E-06	stable	CHST14
3.0982E-06	stable	C9ORF103
3.11752E-06	stable	TPRG1L
3.13745E-06	stable	RABL3
3.14052E-06	stable	BTD
3.17104E-06	stable	PLAC9
3.17519E-06	stable	LOC644590
3.1765E-06	stable	RAI1
3.19497E-06	stable	SLC25A1
3.19721E-06	stable	WDR34
3.20068E-06	stable	STK10
3.21469E-06	stable	FOXO3
3.21469E-06	stable	CCNL1
3.23969E-06	stable	FNBP1
3.26454E-06	stable	C18ORF22
3.27463E-06	stable	GNAI2
3.29384E-06	stable	CDC7
3.2947E-06	stable	FXYD1
3.29537E-06	stable	C6ORF48
3.30218E-06	stable	ZNF667
3.325E-06	stable	MAD1L1
3.33082E-06	stable	TRQ1
3.47885E-06	stable	DDX42
3.49762E-06	stable	EIF3I
3.51151E-06	stable	HSD17B14
3.53436E-06	stable	PPP1CB
3.55685E-06	stable	HDDC2
3.55685E-06	stable	NSMCE4A
3.58181E-06	stable	CTSL1
3.59538E-06	stable	TAX1BP1
3.61046E-06	stable	SRD5A2L2

3.61934E-06	stable	PIGW
3.6285E-06	stable	SEPT8
3.6384E-06	stable	IFI35
3.6611E-06	stable	TACC1
3.67202E-06	stable	CXORF23
3.68139E-06	stable	DOCK9
3.70191E-06	stable	TWSG1
3.70262E-06	stable	CHAC2
3.7212E-06	stable	CORO7
3.75391E-06	stable	CALB2
3.76241E-06	stable	ZNF439
3.77364E-06	stable	C1ORF149
3.77601E-06	stable	ZBTB43
3.77947E-06	stable	SYT17
3.80021E-06	stable	EGLN1
3.81544E-06	stable	SNRK
3.81839E-06	stable	MESP1
3.86929E-06	stable	CCL19
3.87613E-06	stable	WBSCR22
3.87716E-06	stable	TMEM9
3.87716E-06	stable	PSAP
3.87827E-06	stable	CAMP
3.88864E-06	stable	HIRIP3
3.93608E-06	stable	VASH2
3.95448E-06	stable	EPN2
4.0032E-06	stable	LSM12
4.0262E-06	stable	KIAA1754
4.03432E-06	stable	C1ORF74
4.04564E-06	stable	SHISA3
4.04639E-06	stable	RAD21
4.05753E-06	stable	LAMC2
4.06122E-06	stable	IRF2BP2
4.08564E-06	stable	LOC151579
4.11768E-06	stable	ZDHHC16
4.13221E-06	stable	C16ORF79
4.14008E-06	stable	CLDN15
4.18022E-06	stable	MEOX2
4.20004E-06	stable	PAQR4
4.21107E-06	stable	PHF1
4.23535E-06	stable	RTN1
4.24268E-06	stable	MUTED
4.24556E-06	stable	YAF2
4.26454E-06	stable	ZNF226
4.29519E-06	stable	C11ORF75
4.29776E-06	stable	PRPF19
4.30708E-06	stable	HEXB
4.32471E-06	stable	TMEM18
4.33613E-06	stable	JUB
4.34632E-06	stable	ARG2
4.35563E-06	stable	LOC728411
4.38165E-06	stable	CYP46A1

4.38175E-06	stable	ITGAE
4.40832E-06	stable	LOC652624
4.41247E-06	stable	SIAH2
4.42969E-06	stable	SECISBP2
4.44813E-06	stable	PTGFRN
4.46325E-06	stable	TMEM120B
4.47956E-06	stable	EIF3A
4.4929E-06	stable	C22ORF13
4.51446E-06	stable	C11ORF52
4.52155E-06	stable	C2ORF30
4.53863E-06	stable	CEP63
4.54563E-06	stable	NID1
4.54766E-06	stable	ECE1
4.60598E-06	stable	HSPA12A
4.61078E-06	stable	IL12A
4.61601E-06	stable	CDC42SE1
4.62164E-06	stable	TRA2A
4.62829E-06	stable	DUSP2
4.64614E-06	stable	E2F3
4.66207E-06	stable	FLJ39827
4.67238E-06	stable	OXR1
4.67276E-06	stable	RPSA
4.67435E-06	stable	UNC93B1
4.68377E-06	stable	EVI5L
4.68377E-06	stable	C6ORF108
4.70952E-06	stable	SRPK3
4.71635E-06	stable	PLCXD1
4.71813E-06	stable	IRAK1BP1
4.71972E-06	stable	ADIPOR2
4.72723E-06	stable	PPAT
4.73258E-06	stable	ZNF408
4.74579E-06	stable	KLHL7
4.75791E-06	stable	CAMLG
4.78208E-06	stable	SNRPC
4.81043E-06	stable	UBIAD1
4.81515E-06	stable	SNAPC2
4.81738E-06	stable	ANKRD39
4.82475E-06	stable	APEG1
4.83931E-06	stable	ZNF484
4.86131E-06	stable	ANGPTL1
4.87536E-06	stable	EZH1
4.89221E-06	stable	RBBP6
4.90036E-06	stable	MNAT1
4.91595E-06	stable	SLIT2
4.93206E-06	stable	NRIP1
4.93753E-06	stable	CKAP5
4.95113E-06	stable	HSD3B7
4.95113E-06	stable	SCARB2
4.95113E-06	stable	EPHX2
4.96285E-06	stable	C7ORF49
4.96875E-06	stable	GNA13

5.00079E-06	stable	C21ORF70
5.04698E-06	stable	VPS33B
5.07872E-06	stable	ZNF254
5.08783E-06	stable	TDG
5.10115E-06	stable	SLC39A8
5.10245E-06	stable	AGFG2
5.10245E-06	stable	NELF
5.10245E-06	stable	TRAPPC3
5.12205E-06	stable	ADCK5
5.17107E-06	stable	CHGB
5.19629E-06	stable	PCF11
5.19629E-06	stable	TRPC4AP
5.19629E-06	stable	POLR3B
5.26407E-06	stable	ADSS
5.26645E-06	stable	NAB1
5.27488E-06	stable	KIAA0649
5.31714E-06	stable	LSAMP
5.39551E-06	stable	CHFR
5.41217E-06	stable	SLC46A3
5.45043E-06	stable	STAG2
5.48605E-06	stable	FLJ12684
5.55518E-06	stable	SCGN
5.58449E-06	stable	DHX9
5.59E-06	stable	RBM41
5.63699E-06	stable	PDLIM1
5.66223E-06	stable	E2F5
5.66223E-06	stable	CAPRIN2
5.66223E-06	stable	NOTCH1
5.66223E-06	stable	IKBKAP
5.66427E-06	stable	ATP5L
5.69537E-06	stable	EBNA1BP2
5.69537E-06	stable	ERF
5.70136E-06	stable	MYBPHL
5.74054E-06	stable	C19ORF42
5.74991E-06	stable	GPN2
5.77451E-06	stable	TMEM189-UBE2V1
5.79375E-06	stable	CBLB
5.81226E-06	stable	C21ORF2
5.81726E-06	stable	TXNDC4
5.82655E-06	stable	C20ORF149
5.88652E-06	stable	MCCC1
5.93183E-06	stable	FGFR1OP
5.94888E-06	stable	RUNX3
5.98209E-06	stable	ZNF256
5.99179E-06	stable	PDGFD
5.99386E-06	stable	METTL5
6.03407E-06	stable	SH3RF2
6.05745E-06	stable	EDF1
6.0816E-06	stable	C10ORF137
6.10773E-06	stable	FRY
6.11764E-06	stable	BRP44

6.14083E-06	stable	TNFAIP1
6.18147E-06	stable	ATP5S
6.22969E-06	stable	TMCC1
6.25784E-06	stable	FLJ41603
6.25784E-06	up	FGF18
6.25784E-06	stable	VCP
6.27118E-06	stable	GABBR2
6.27118E-06	stable	METAP2
6.27278E-06	stable	CPD
6.29154E-06	stable	ZFHX3
6.35298E-06	stable	LOC653972
6.36559E-06	down	XIRP1
6.39564E-06	stable	C21ORF59
6.43544E-06	stable	GLUL
6.47919E-06	stable	PRELID1
6.48297E-06	stable	UHRF1
6.5282E-06	stable	RASSF5
6.62112E-06	stable	PDLIM5
6.65679E-06	stable	HVCN1
6.66646E-06	stable	SLC9A4
6.7227E-06	stable	NAT9
6.76332E-06	stable	HDHD3
6.76932E-06	stable	ZNF287
6.78083E-06	stable	PRRG2
6.78747E-06	stable	UNC84A
6.79635E-06	stable	SNRPF
6.82146E-06	stable	SRA1
6.82146E-06	stable	RNF170
6.86418E-06	stable	HLA-E
6.87166E-06	stable	C20ORF103
6.89288E-06	stable	KCNK3
6.89924E-06	stable	C12ORF57
7.00976E-06	stable	LOC653994
7.0149E-06	stable	SH3GLB1
7.0454E-06	stable	FLJ22167
7.04803E-06	stable	RPL39L
7.04983E-06	stable	GBA
7.11155E-06	stable	GADL1
7.11832E-06	stable	VPS24
7.15535E-06	stable	NPHP3
7.20633E-06	stable	ZNF613
7.23385E-06	stable	FKBP3
7.25518E-06	stable	LOC646463
7.27557E-06	stable	PARVB
7.35468E-06	stable	SGCA
7.39004E-06	stable	CD47
7.52284E-06	stable	MAF
7.52371E-06	stable	DMKN
7.52848E-06	stable	SUMO3
7.54599E-06	stable	SAMD4A
7.59958E-06	stable	SNX21

7.59958E-06	stable	TOMM34
7.61114E-06	stable	RNF125
7.64527E-06	stable	PPP6C
7.67238E-06	stable	NME1-NME2
7.68774E-06	stable	ASGR2
7.70908E-06	stable	SNORA10
7.72808E-06	stable	C6ORF57
7.75177E-06	stable	CXXC5
7.75177E-06	stable	ANAPC13
7.75177E-06	stable	RWDD4A
7.75177E-06	stable	TFB1M
7.84694E-06	stable	E2F2
7.86599E-06	stable	DHFRL1
7.86815E-06	stable	IL27RA
7.91938E-06	stable	NR2F6
7.92682E-06	stable	ZFAND6
7.96068E-06	stable	ZCCHC3
7.96952E-06	stable	LETM1
7.98427E-06	stable	MGC12965
7.98427E-06	stable	C18ORF51
8.01539E-06	stable	TUSC4
8.04513E-06	stable	AURKA
8.07174E-06	stable	RAB11FIP3
8.11619E-06	stable	ZMYM2
8.15302E-06	stable	U2AF1
8.155E-06	stable	CCNB1IP1
8.19822E-06	stable	GSN
8.28229E-06	stable	NSMCE1
8.29802E-06	stable	LOC647970
8.30856E-06	stable	ACVRL1
8.32139E-06	stable	THOC6
8.43116E-06	stable	ZFYVE21
8.44848E-06	stable	RCN3
8.59684E-06	stable	SLC25A13
8.6084E-06	stable	MMP28
8.64594E-06	stable	MLSTD1
8.6857E-06	stable	SNORA70
8.6857E-06	stable	CCNI
8.69299E-06	stable	MAP2K1IP1
8.69402E-06	up	PENK
8.72397E-06	stable	SRRM1
8.7283E-06	stable	GABPA
8.74367E-06	stable	STEAP1
8.75417E-06	stable	FLJ39653
8.75671E-06	stable	HRIHFB2122
8.7651E-06	stable	RPL3L
8.77816E-06	stable	LOC729101
8.85969E-06	stable	ZNF365
8.91784E-06	stable	SNRP70
8.93357E-06	down	SLCO2A1
8.94752E-06	stable	TMEM67

8.97062E-06	stable	LAT1-3TM
8.97986E-06	stable	RNF130
8.98683E-06	stable	LOC644743
9.02033E-06	stable	AP1M1
9.12554E-06	stable	KHSRP
9.12554E-06	stable	EI24
9.1618E-06	stable	RBM12B
9.22309E-06	stable	C19ORF18
9.26677E-06	stable	PDE2A
9.27134E-06	stable	KIAA0564
9.36542E-06	stable	LOC339344
9.39746E-06	stable	TMEM47
9.4276E-06	stable	LRRC10
9.44002E-06	stable	NECAB3
9.45618E-06	stable	FOXC1
9.46578E-06	stable	LOC285929
9.50039E-06	stable	UBE2J2
9.5088E-06	stable	CETN3
9.57403E-06	stable	SLC22A18
9.63019E-06	stable	ZNF181
9.65104E-06	stable	TMEM183A
9.65695E-06	stable	FAM167B
9.69604E-06	stable	DNAJA4
9.79157E-06	stable	ARMCX1
9.82353E-06	stable	LOC643446
9.82394E-06	stable	NGLY1
9.83559E-06	stable	C18ORF1
9.94942E-06	stable	STAG3L2
1.00398E-05	stable	FOLR3
1.00487E-05	stable	FAM104B
1.00522E-05	stable	CLEC1A
1.01079E-05	stable	RBBP7
1.01192E-05	stable	C1ORF225
1.01542E-05	stable	LOC401019
1.02106E-05	stable	OPA3
1.02214E-05	stable	ALDH1A2
1.02582E-05	stable	PLAGL1
1.0286E-05	stable	IVD
1.03357E-05	stable	FIS
1.03932E-05	stable	PLCB2
1.03935E-05	stable	KIAA0196
1.0431E-05	stable	EGFL7
1.0431E-05	stable	HIST1H4H
1.05485E-05	stable	SUOX
1.05608E-05	stable	TIAM2
1.07053E-05	stable	ZNF703
1.07053E-05	stable	CDC123
1.07053E-05	stable	RRAGD
1.07232E-05	stable	FBXO9
1.07355E-05	stable	FAP
1.07627E-05	stable	DERL2

1.07683E-05	stable	DECR2
1.07683E-05	stable	ZNF18
1.07886E-05	stable	KIAA0528
1.08221E-05	stable	BMP2
1.08816E-05	stable	CAV1
1.08889E-05	stable	TMEM80
1.09718E-05	stable	NUPR1
1.09718E-05	stable	C9ORF123
1.09846E-05	stable	LOC389203
1.10176E-05	stable	ORMDL3
1.10215E-05	stable	EIF4E3
1.10517E-05	stable	RAB9A
1.10887E-05	stable	IPO4
1.10887E-05	stable	CBFA2T2
1.10887E-05	stable	BBS12
1.11059E-05	stable	PLXNB1
1.11498E-05	stable	HIG2
1.11552E-05	stable	PCDH18
1.12191E-05	stable	ANXA2P3
1.125E-05	stable	FAM45A
1.12865E-05	stable	SMC4
1.12996E-05	stable	HPS1
1.1359E-05	stable	PIGZ
1.14088E-05	stable	MGAT4A
1.14226E-05	stable	PELP1
1.15559E-05	stable	GARS
1.16405E-05	stable	OSM
1.1667E-05	stable	C11ORF45
1.1667E-05	stable	TTC33
1.17315E-05	stable	C11ORF38
1.17811E-05	stable	RAB40B
1.18033E-05	stable	CDH23
1.18802E-05	stable	COQ3
1.19463E-05	stable	CCR2
1.19616E-05	stable	TMEM183B
1.19896E-05	stable	COG6
1.20218E-05	stable	LEMD2
1.20899E-05	stable	SKP2
1.21833E-05	stable	PACSIN3
1.23551E-05	stable	PDS5A
1.2465E-05	stable	AGTPBP1
1.248E-05	stable	UQCRQ
1.25026E-05	stable	CCDC32
1.25135E-05	stable	FBXO30
1.25135E-05	stable	HEATR2
1.25256E-05	stable	PIGO
1.25384E-05	stable	KIAA1244
1.26136E-05	stable	KIAA1267
1.26957E-05	stable	PIAS4
1.28322E-05	stable	BMF
1.28844E-05	stable	WDR13

1.28975E-05	stable	SRPR
1.30092E-05	stable	SUPT3H
1.30119E-05	stable	PLD3
1.30919E-05	stable	ELK4
1.30993E-05	stable	POLR2D
1.31688E-05	stable	ZNF561
1.31798E-05	stable	CFI
1.32129E-05	stable	UBAC1
1.32341E-05	stable	PMVK
1.33267E-05	stable	TCEB3
1.3375E-05	stable	LTB4R
1.34403E-05	stable	PTGDS
1.34584E-05	stable	TPMT
1.35161E-05	stable	SNX10
1.35616E-05	stable	F2RL2
1.36144E-05	stable	CD300LG
1.36969E-05	stable	UBE2O
1.36969E-05	stable	CCBL1
1.37419E-05	stable	RPLP0
1.38578E-05	stable	ZSWIM6
1.39144E-05	stable	AXIN1
1.39431E-05	stable	KIAA1107
1.40297E-05	stable	TJAP1
1.4044E-05	stable	DHTKD1
1.40962E-05	stable	AMY2B
1.42258E-05	stable	POLB
1.42311E-05	stable	ZFP161
1.42575E-05	stable	BOLA1
1.43535E-05	stable	C20ORF43
1.43971E-05	stable	SHE
1.44283E-05	stable	TRAPPC2L
1.46086E-05	stable	C2
1.46691E-05	stable	LRRC51
1.46691E-05	stable	RAB38
1.47635E-05	stable	RIC8A
1.47691E-05	stable	CNBP
1.47969E-05	stable	TMEM159
1.48164E-05	stable	CKAP4
1.48583E-05	stable	SETBP1
1.49998E-05	stable	C10ORF67
1.5003E-05	stable	KIAA1271
1.5003E-05	stable	ILF3
1.50657E-05	stable	TLR4
1.50757E-05	stable	WDR45L
1.50937E-05	stable	EHD3
1.50937E-05	stable	C16ORF72
1.51398E-05	stable	CRYBG3
1.52045E-05	stable	C15ORF40
1.52681E-05	stable	ARNT2
1.52947E-05	stable	LOC641825
1.53084E-05	stable	KCNIP3

1.53718E-05	stable	PH-4
1.5455E-05	stable	ZNF507
1.54727E-05	stable	HSF4
1.54926E-05	stable	ANK1
1.56168E-05	stable	DAB2
1.5676E-05	stable	ABCB7
1.56987E-05	stable	AQP4
1.59235E-05	stable	AKAP8L
1.59694E-05	stable	OSAP
1.60523E-05	stable	PSMB9
1.61338E-05	stable	SLC16A14
1.62529E-05	stable	EFTUD2
1.62696E-05	stable	MSRB2
1.6299E-05	stable	SLC41A3
1.63438E-05	stable	FARP1
1.63476E-05	stable	C15ORF59
1.64042E-05	stable	FAM81A
1.64323E-05	stable	ESD
1.64323E-05	stable	CEP290
1.65088E-05	stable	CPE
1.6528E-05	stable	KLRG1
1.65737E-05	stable	TUBE1
1.67322E-05	stable	HIC2
1.67551E-05	stable	ELOVL5
1.67785E-05	stable	GPR124
1.67802E-05	stable	COQ6
1.69011E-05	stable	LOC283174
1.69175E-05	stable	CETP
1.69861E-05	stable	LOC643293
1.70798E-05	stable	CCDC51
1.70963E-05	stable	FHIT
1.71317E-05	stable	ITFG2
1.73294E-05	stable	FAM155B
1.73735E-05	stable	SMAP1
1.74147E-05	stable	VGLL3
1.74147E-05	stable	C16ORF87
1.74733E-05	stable	RARRES3
1.76886E-05	stable	WDR59
1.76886E-05	stable	FBL
1.78026E-05	stable	C2ORF18
1.79191E-05	stable	HS3ST2
1.7952E-05	stable	ESRRAP2
1.80711E-05	stable	SETD3
1.81269E-05	stable	ZNF234
1.81308E-05	stable	SNRPA1
1.83474E-05	stable	SNCAIP
1.83559E-05	stable	ZDHHC15
1.83642E-05	stable	ZNF491
1.83642E-05	stable	ZER1
1.85031E-05	stable	ZNF12
1.85271E-05	stable	ZNF25

1.85628E-05	stable	C11ORF71
1.86044E-05	stable	EDN1
1.86679E-05	stable	SLAIN1
1.87555E-05	stable	C3ORF33
1.88094E-05	stable	PTRH1
1.88094E-05	stable	GNMT
1.88496E-05	stable	MED23
1.89256E-05	stable	LRRCC1
1.90167E-05	stable	CD52
1.90167E-05	stable	TSNAX
1.90179E-05	stable	KCMF1
1.90499E-05	stable	MYO9A
1.90675E-05	stable	LOC645863
1.90732E-05	stable	LOC440349
1.90985E-05	stable	AGPAT9
1.91127E-05	stable	KRCC1
1.91174E-05	stable	CHKB
1.91876E-05	stable	S100A4
1.92795E-05	stable	PNPLA7
1.928E-05	stable	HKR1
1.93627E-05	stable	FAM57A
1.93867E-05	stable	CEP57
1.94274E-05	stable	CPNE3
1.9574E-05	stable	A4GALT
1.96369E-05	stable	RTN4R
1.97184E-05	stable	BHMT2
1.9893E-05	stable	DOCK10
1.99948E-05	stable	LINGO4
2.00669E-05	stable	GDF10
2.01874E-05	stable	CHMP2A
2.02348E-05	stable	KPNA4
2.02867E-05	stable	KIAA1688
2.03428E-05	stable	CCDC136
2.03661E-05	stable	PEBP4
2.04878E-05	stable	BBS1
2.06363E-05	stable	DYRK3
2.08895E-05	stable	TMEM177
2.10387E-05	stable	STIL
2.10545E-05	stable	MRPL1
2.11099E-05	stable	LAMA4
2.11431E-05	stable	ACBD5
2.12135E-05	stable	CDCA2
2.12782E-05	stable	LOC648057
2.13497E-05	stable	GNRH1
2.13896E-05	stable	SLC38A7
2.16189E-05	stable	CIR
2.1679E-05	stable	SH3BGR
2.174E-05	stable	HIBCH
2.18431E-05	stable	C18ORF8
2.19385E-05	stable	MAT2B
2.22533E-05	stable	TMUB2

2.23511E-05	stable	RBBP9
2.2378E-05	stable	SDC2
2.24868E-05	stable	NPHP1
2.25021E-05	stable	RNF24
2.25021E-05	stable	GNPNAT1
2.25021E-05	stable	SAMD13
2.25154E-05	stable	YIPF1
2.25605E-05	stable	TMEM198
2.25605E-05	stable	UBTD2
2.26913E-05	stable	FKBP14
2.27311E-05	stable	DPF3
2.27363E-05	stable	ZNF71
2.27744E-05	stable	MRPL52
2.28487E-05	stable	PLEKHA5
2.28497E-05	stable	AKAP11
2.28599E-05	stable	KIAA0152
2.3001E-05	stable	SRPK1
2.30171E-05	stable	EDIL3
2.30342E-05	stable	LOC652545
2.30526E-05	stable	LRRC8D
2.31126E-05	stable	YIPF3
2.35161E-05	stable	DBN1
2.39711E-05	stable	HECW2
2.39728E-05	stable	RIOK2
2.39862E-05	stable	C14ORF2
2.41784E-05	stable	LOC642299
2.43199E-05	stable	FAM50B
2.44265E-05	stable	LEPR
2.44896E-05	stable	CRISPLD1
2.4562E-05	stable	ZFYVE16
2.47207E-05	stable	MAGED2
2.47947E-05	stable	U2AF1L4
2.48333E-05	stable	CHODL
2.50913E-05	stable	MARCKSL1
2.51192E-05	stable	SGPL1
2.51755E-05	stable	C1S
2.53265E-05	stable	FAM33A
2.53669E-05	stable	SNAP23
2.54416E-05	stable	EIF4A3
2.54987E-05	stable	GNL3
2.56169E-05	stable	KCNT2
2.56435E-05	stable	MMP15
2.5754E-05	stable	GOLGA4
2.58158E-05	stable	NDRG2
2.58158E-05	stable	GCNT3
2.58195E-05	stable	RPAIN
2.65338E-05	stable	LOC650695
2.65767E-05	up	THY1
2.66311E-05	stable	LMNB1
2.6676E-05	stable	CADPS2
2.67615E-05	stable	BTRC

2.69848E-05	stable	ANKRD23
2.7015E-05	stable	PAWR
2.70783E-05	stable	CRKL
2.71858E-05	stable	CDC5L
2.71858E-05	stable	C20ORF194
2.72066E-05	stable	GTF3C3
2.72315E-05	stable	C4ORF27
2.73599E-05	stable	TGFB1I1
2.73599E-05	stable	GPR162
2.75705E-05	stable	ACVR2A
2.79033E-05	stable	PPP2R3C
2.81635E-05	stable	FOXK2
2.82521E-05	stable	ELP4
2.82521E-05	stable	PGLS
2.82521E-05	stable	EP300
2.83217E-05	stable	PLXNA3
2.83994E-05	stable	WIPI1
2.84392E-05	stable	C11ORF54
2.87159E-05	stable	GRB10
2.87159E-05	stable	SNORD108
2.87499E-05	stable	BCAM
2.87547E-05	stable	PANK2
2.87783E-05	stable	CLEC5A
2.88586E-05	stable	ANXA1
2.89994E-05	stable	ZNF462
2.90139E-05	stable	LSM7
2.93278E-05	stable	NMD3
2.94321E-05	stable	MGC71993
2.95463E-05	stable	PNMT
2.95848E-05	stable	PLEKHG1
2.97056E-05	stable	FABP3
2.97986E-05	stable	TP53I13
2.99076E-05	stable	TRIM32
2.99335E-05	stable	TES
2.99637E-05	stable	SLC25A16
3.00554E-05	stable	RB1CC1
3.01125E-05	stable	PDXP
3.01371E-05	stable	MLH1
3.02145E-05	stable	KIT
3.02588E-05	stable	DVL3
3.026E-05	stable	ZBTB7B
3.02696E-05	stable	HINT2
3.03038E-05	stable	FLJ39632
3.03251E-05	stable	HNRNPU
3.03287E-05	stable	CRSP9
3.03756E-05	stable	HSP90B1
3.04478E-05	stable	ZC3H6
3.08364E-05	stable	LOC402176
3.08933E-05	stable	MFSD7
3.10135E-05	stable	LMAN2L
3.11741E-05	stable	CAMK2G

3.14072E-05	stable	FDX1L
3.14807E-05	stable	EIF5A
3.15837E-05	stable	HAS3
3.15987E-05	stable	TCEA1
3.17957E-05	stable	ITSN1
3.20452E-05	stable	PRKAA2
3.20835E-05	stable	MTERF
3.20928E-05	stable	SLC43A3
3.22051E-05	stable	PKIA
3.22504E-05	stable	DHX33
3.24112E-05	stable	B3GAT3
3.24559E-05	stable	SHROOM4
3.25126E-05	stable	ATP11C
3.25199E-05	stable	LOC144481
3.25991E-05	stable	SHPRH
3.26042E-05	stable	YOD1
3.28571E-05	stable	RAPGEF3
3.28571E-05	stable	PPP1R8
3.29754E-05	stable	CYB561D2
3.29887E-05	stable	DDX23
3.31156E-05	stable	VTN
3.34313E-05	stable	HDAC2
3.34532E-05	stable	CORO1C
3.35265E-05	stable	THAP1
3.3563E-05	stable	FBXL20
3.36265E-05	stable	NAP1L1
3.36584E-05	stable	CHMP1B
3.37876E-05	stable	HEXIM2
3.37876E-05	stable	SELE
3.40023E-05	stable	STBD1
3.43637E-05	stable	ABCA5
3.4469E-05	stable	IARS2
3.45365E-05	stable	PCDHB15
3.48007E-05	stable	C1ORF216
3.50255E-05	stable	MTCP1
3.51726E-05	stable	CCDC43
3.51968E-05	stable	GNPTAB
3.52113E-05	stable	C1QL1
3.53868E-05	stable	BNIP3
3.55099E-05	stable	LZTR1
3.5754E-05	stable	PLEKHO1
3.59208E-05	stable	BMP7
3.59756E-05	stable	SPAG7
3.6137E-05	down	CYP1B1
3.61978E-05	stable	NPEPL1
3.62684E-05	stable	BMPR2
3.63011E-05	stable	SPATA5L1
3.63175E-05	stable	PSMB3
3.63519E-05	stable	LOC642333
3.64004E-05	stable	PSME2
3.64389E-05	stable	OSR2

3.64795E-05	stable	RUNDC2A
3.65374E-05	stable	AIG1
3.65374E-05	stable	ADSSL1
3.67202E-05	stable	PPP1R14A
3.68404E-05	stable	REPS1
3.68404E-05	stable	TMEM27
3.6897E-05	stable	AHCY
3.69004E-05	stable	C14ORF131
3.6982E-05	stable	SRRP35
3.70493E-05	stable	EVI2B
3.71453E-05	stable	RPS6KA2
3.72049E-05	stable	MYL5
3.72492E-05	stable	SH2D5
3.72936E-05	stable	SEPP1
3.72936E-05	stable	CHUK
3.73939E-05	stable	GPSM3
3.75348E-05	stable	WDR67
3.7734E-05	stable	SCG2
3.7844E-05	stable	SEC14L2
3.80606E-05	stable	APCDD1
3.81856E-05	stable	SCRN2
3.83182E-05	stable	TOR1AIP2
3.87115E-05	stable	CNOT4
3.87559E-05	stable	ATG5
3.87885E-05	stable	PDGFC
3.87889E-05	stable	C20ORF132
3.88061E-05	stable	UBE1C
3.89187E-05	stable	PPIL3
3.89432E-05	stable	CXXC1
3.90714E-05	stable	SCAPER
3.93744E-05	stable	LOC645566
3.93801E-05	stable	DVL2
3.94493E-05	stable	GABRB3
3.96195E-05	stable	U2AF1L2
3.9631E-05	stable	ASS1
3.98724E-05	stable	CEACAM3
3.98724E-05	up	POSTN
4.01137E-05	stable	LPCAT4
4.02042E-05	stable	HAND2
4.02572E-05	stable	LOC644436
4.04492E-05	stable	WDR55
4.04739E-05	stable	STK33
4.08837E-05	stable	STX4
4.10091E-05	up	APOE
4.10091E-05	stable	SMARCB1
4.11802E-05	stable	TTC13
4.13708E-05	stable	PNPLA6
4.14931E-05	stable	PTTG1
4.15521E-05	stable	PTHLH
4.15628E-05	down	CNN1
4.161E-05	stable	SIVA

4.16439E-05	stable	ACOT8
4.16465E-05	stable	ANKRD26
4.16628E-05	stable	RN7SK
4.16628E-05	stable	BET1
4.16628E-05	stable	EIF2A
4.17686E-05	stable	TAX1BP3
4.20094E-05	stable	USP10
4.23296E-05	stable	KIAA0247
4.23296E-05	stable	C18ORF21
4.2752E-05	stable	GFM1
4.28154E-05	stable	CASC4
4.28154E-05	stable	HEYL
4.28154E-05	stable	SNX11
4.28732E-05	stable	BEX2
4.29588E-05	stable	P4HA2
4.29988E-05	stable	KCNMA1
4.30323E-05	stable	PPP1R3E
4.30829E-05	stable	LRRC47
4.31188E-05	stable	LUZP1
4.31701E-05	stable	C20ORF107
4.31847E-05	stable	LOC642780
4.32778E-05	stable	USP38
4.32778E-05	stable	ZNF148
4.33333E-05	stable	FLJ43663
4.37833E-05	stable	ARPP-19
4.39817E-05	stable	PFAAP5
4.41836E-05	stable	MPDZ
4.46018E-05	stable	SNUPN
4.46054E-05	stable	FLJ21438
4.47324E-05	stable	LOC642921
4.47679E-05	stable	SDHAP3
4.50167E-05	stable	ME2
4.50604E-05	stable	LONP1
4.51686E-05	stable	SDHD
4.52942E-05	stable	C9ORF16
4.53347E-05	stable	TPP2
4.53386E-05	stable	HRH2
4.53651E-05	stable	SLC25A20
4.57223E-05	stable	CUX2
4.59103E-05	stable	RING1
4.61268E-05	stable	DHX35
4.62125E-05	stable	PI4KB
4.62401E-05	stable	PPP2R1B
4.64431E-05	stable	GOT2
4.64431E-05	stable	LOC647054
4.64431E-05	stable	RASL12
4.68067E-05	stable	ZNF425
4.68991E-05	stable	LOC649604
4.6903E-05	stable	CHCHD5
4.69588E-05	stable	USP22
4.69738E-05	stable	VWF

4.6981E-05	stable	HEBP2
4.70878E-05	stable	C1ORF128
4.74087E-05	stable	TAF1C
4.74984E-05	stable	LOC339804
4.75254E-05	stable	ANP32A
4.75279E-05	stable	DHRS4L2
4.79985E-05	stable	CDK5
4.82348E-05	stable	PARP9
4.84418E-05	stable	RBM5
4.84816E-05	stable	YIPF4
4.85882E-05	stable	GMDS
4.86885E-05	stable	GATA6
4.86925E-05	stable	RPGR
4.87063E-05	stable	PPP1R11
4.88131E-05	stable	TPPP3
4.88208E-05	stable	UBTF
4.88621E-05	stable	C3ORF15
4.90822E-05	stable	PLEKHA6
4.91696E-05	stable	NME5
4.92891E-05	stable	ZFAND1
4.969E-05	stable	GSPT1
5.01539E-05	stable	DKFZP564O0523
5.02442E-05	stable	ZSCAN4
5.02547E-05	stable	SYNE2
5.0588E-05	stable	ENSA
5.06757E-05	stable	ATE1
5.12253E-05	stable	WNT2B
5.12395E-05	stable	GTF2B
5.13425E-05	stable	TMEM182
5.13954E-05	stable	LOC728635
5.15066E-05	stable	ZNF517
5.15601E-05	stable	TPPP
5.15601E-05	stable	RBM26
5.17647E-05	stable	ENPP2
5.17709E-05	stable	ZFYVE26
5.17709E-05	stable	MGC18216
5.18986E-05	stable	LOC648249
5.18986E-05	stable	CISD1
5.21191E-05	stable	LOC651029
5.21977E-05	stable	DKK3
5.21977E-05	stable	MCM2
5.21977E-05	stable	JPH1
5.25816E-05	stable	CCDC131
5.26951E-05	stable	AOAH
5.31068E-05	stable	CNIH4
5.32613E-05	stable	RIPK1
5.35644E-05	stable	NUDC
5.38364E-05	stable	FGL2
5.39028E-05	stable	LZTFL1
5.39535E-05	stable	KIAA0261
5.41481E-05	stable	DYNC1LI1

5.41971E-05	stable	ACTR5
5.42234E-05	stable	TPM2
5.45297E-05	stable	LOC401238
5.46674E-05	stable	ARMET
5.47517E-05	stable	TRADD
5.47726E-05	stable	RENBP
5.52769E-05	stable	NPR2
5.53468E-05	stable	TMEM62
5.56022E-05	stable	GLRX2
5.5625E-05	stable	AK3
5.5671E-05	stable	ARGLU1
5.57018E-05	stable	LOC653308
5.57904E-05	stable	TEF
5.68065E-05	stable	BBS9
5.69816E-05	stable	SLC19A3
5.76488E-05	stable	ENDOG
5.78018E-05	stable	ANKRD32
5.78018E-05	stable	C9ORF23
5.78283E-05	stable	TSPAN4
5.80404E-05	stable	LOC440093
5.82743E-05	stable	MFHAS1
5.84495E-05	stable	STMN1
5.84495E-05	stable	CTBP2
5.84891E-05	stable	ZBTB48
5.85214E-05	stable	LOC643911
5.92365E-05	stable	ARHGAP11A
5.92365E-05	stable	DIABLO
5.94169E-05	stable	LEPRE1
5.96523E-05	stable	NSUN3
5.97432E-05	stable	PDXK
6.00128E-05	stable	ASL
6.00458E-05	stable	SNAPC3
6.00906E-05	stable	AXIN2
6.06581E-05	stable	PNN
6.11294E-05	stable	SCARA5
6.16717E-05	stable	ELOVL4
6.16821E-05	stable	ZNF644
6.24173E-05	stable	EPDR1
6.28154E-05	stable	ERN1
6.3364E-05	stable	CDC42BPB
6.36933E-05	stable	PNO1
6.39018E-05	stable	TNNC2
6.40622E-05	stable	GLTSCR1
6.50315E-05	stable	C11ORF48
6.50999E-05	stable	LOC648605
6.50999E-05	stable	SRM
6.53715E-05	stable	HMG20A
6.55025E-05	stable	DERL1
6.62617E-05	stable	SIRT1
6.68386E-05	stable	LOC644096
6.69125E-05	stable	C4ORF14

6.69125E-05	stable	ISCA1L
6.7757E-05	stable	HOXD12
6.77813E-05	stable	UBR1
6.81825E-05	stable	RHOT2
6.83813E-05	stable	CARD8
6.9213E-05	stable	ELOVL6
6.95094E-05	stable	CSF1
6.96062E-05	stable	SCAMP3
6.96427E-05	stable	NBEAL2
6.99609E-05	stable	IL24
7.01765E-05	stable	GPR64
7.04309E-05	stable	C17ORF75
7.04563E-05	stable	TRIOBP
7.06766E-05	stable	TMEM70
7.07015E-05	stable	MAP1LC3B
7.07015E-05	stable	CDK2AP2
7.07348E-05	stable	SLC39A14
7.08173E-05	stable	ACADVL
7.17686E-05	stable	LOC399888
7.19629E-05	stable	UROD
7.26659E-05	stable	ACSM3
7.32829E-05	stable	ATP5G3
7.37849E-05	stable	MAN2C1
7.39971E-05	stable	COMMD3
7.41069E-05	stable	DDX17
7.44346E-05	stable	DHRS7B
7.46446E-05	stable	C17ORF63
7.50944E-05	stable	CXORF56
7.54045E-05	stable	LOC642489
7.54715E-05	stable	CCT2
7.58566E-05	stable	OR8H3
7.60498E-05	stable	ZBTB8OS
7.61399E-05	stable	ATP6V0A2
7.61827E-05	stable	HNRPC
7.6305E-05	stable	YRDC
7.63524E-05	down	MYOT
7.65145E-05	stable	CLEC4GP1
7.6581E-05	stable	ZNF444
7.68324E-05	stable	AKR1C2
7.6875E-05	stable	HECTD3
7.71429E-05	stable	ZNF529
7.73116E-05	stable	HSPBAP1
7.73171E-05	stable	CD74
7.73738E-05	stable	SCARNA9
7.74901E-05	stable	TTC17
7.79304E-05	stable	TPO
7.82071E-05	stable	PADI4
7.86818E-05	stable	MST1
7.90897E-05	stable	PIK3R4
7.91638E-05	stable	PTTG2
7.92923E-05	stable	ZNF451

7.9973E-05	stable	G6PC3
7.99993E-05	stable	AP3D1
8.01238E-05	stable	IDH2
8.01945E-05	stable	AGT
8.03331E-05	stable	MACF1
8.04765E-05	stable	NEDD1
8.12579E-05	stable	SLC27A1
8.15936E-05	stable	BCL2L12
8.17468E-05	stable	TPCN1
8.21063E-05	stable	IRS2
8.21063E-05	stable	PAQR6
8.21146E-05	stable	MLXIPL
8.24219E-05	stable	LOC440160
8.27742E-05	stable	LOC387867
8.27742E-05	stable	LOC650152
8.34361E-05	stable	CDS2
8.36705E-05	stable	CNPY3
8.38695E-05	stable	IL3RA
8.41455E-05	stable	TP53
8.44708E-05	stable	SNORD36A
8.45426E-05	stable	LRRFIP2
8.48872E-05	stable	PTTG3
8.50473E-05	stable	GHR
8.52216E-05	stable	RASIP1
8.52944E-05	stable	CCDC111
8.52944E-05	stable	LOC729905
8.54913E-05	stable	EPN1
8.55534E-05	stable	C9ORF164
8.55534E-05	stable	AGK
8.56301E-05	stable	STK32B
8.57369E-05	stable	TMEM57
8.6115E-05	stable	NHP2L1
8.63259E-05	stable	TAGLN
8.67473E-05	stable	DOK7
8.70041E-05	stable	SALL2
8.72267E-05	stable	MRPL53
8.73639E-05	stable	GRHPR
8.76637E-05	stable	CEP120
8.77396E-05	stable	PPM1F
8.80124E-05	stable	LOC643720
8.8425E-05	stable	HERC4
8.8609E-05	stable	IIP45
8.86275E-05	stable	IKBKE
8.90051E-05	stable	SF3B4
8.95689E-05	stable	SGCE
8.96583E-05	stable	NME3
8.96583E-05	stable	LOC727751
8.98198E-05	stable	SLC27A6
8.99156E-05	stable	AP1G1
8.99814E-05	stable	MRCL3
9.00309E-05	stable	TACC2

9.06045E-05	stable	CDKN2AIPNL
9.06141E-05	stable	PCDHB4
9.06991E-05	stable	NTNG2
9.10111E-05	stable	ABCA2
9.10111E-05	stable	VAV1
9.11642E-05	stable	DHRS12
9.17201E-05	stable	SERINC2
9.22202E-05	stable	PMS2
9.22202E-05	stable	C16ORF63
9.22557E-05	stable	GBP2
9.22941E-05	stable	LOC645550
9.35956E-05	stable	SPRY2
9.36625E-05	stable	RANBP1
9.43548E-05	stable	ABHD1
9.58204E-05	stable	GALNT11
9.58999E-05	stable	LOC652455
9.59339E-05	stable	FAM171A1
9.70443E-05	stable	CENPM
9.79452E-05	stable	B9D2
9.81869E-05	stable	C6ORF115
9.86004E-05	stable	LOC389137
9.861E-05	stable	SPHKAP
9.87736E-05	stable	MEF2C
9.88911E-05	stable	MEPCE
9.91151E-05	stable	LOC647856
9.9146E-05	stable	IFI27
9.94811E-05	stable	LOC642267
9.99192E-05	stable	ANKZF1
0.000100707	stable	SLC20A1
0.000100999	stable	GLYCTK
0.000102773	stable	FJX1
0.00010397	stable	UST
0.000104334	stable	BAK1
0.000104383	stable	FLJ20718
0.000104472	stable	SMARCA5
0.000104527	stable	ZKSCAN3
0.000105208	stable	RNPC3
0.000105208	stable	HRSP12
0.000105226	stable	KLF9
0.000105692	stable	COLEC11
0.000105736	stable	FGF9
0.000105736	stable	VHL
0.000105763	stable	MICB
0.000105782	stable	EVI1
0.000105869	stable	DLAT
0.000105926	stable	LOC651881
0.000106439	stable	EIF2S3
0.000107435	stable	UCHL3
0.00010764	stable	GPBP1L1
0.00010764	stable	DRD4
0.00010793	stable	TRAPPC2

0.000108227	stable	PLA1A
0.000108471	stable	LOC654069
0.000108989	stable	TLCD1
0.000109193	stable	SLAMF6
0.000109259	stable	PMS2L2
0.000109865	stable	TIA1
0.000110402	stable	SPRYD3
0.000110498	stable	SHMT2
0.000110529	stable	CSGLCA-T
0.000110537	stable	LOC654346
0.000110649	stable	FXR2
0.000110663	stable	THOC2
0.000110885	stable	TTC5
0.00011113	stable	POLE3
0.000112244	stable	ZNF510
0.000112704	stable	LOC400406
0.000112871	stable	KIAA0133
0.000113389	stable	CNN2
0.000113389	stable	C1ORF168
0.000113492	stable	TERF2IP
0.000113492	stable	LOC731314
0.000113983	stable	CD63
0.000114738	stable	COBRA1
0.000115201	stable	C6ORF145
0.000115754	stable	POLE2
0.000115845	stable	KCNJ8
0.000115851	stable	CALCOCO2
0.000115882	stable	LOC648749
0.000116445	stable	YEATS4
0.000116665	stable	AGA
0.000116729	stable	NUPL2
0.000117523	stable	C9ORF142
0.000117814	stable	SLC38A8
0.000117816	stable	ZFAND5
0.000117907	stable	KIF7
0.000118268	stable	ANKRD29
0.000118536	stable	CCDC104
0.000118547	stable	KIAA1324L
0.000118554	stable	ATG4B
0.000118593	stable	SP100
0.000118826	stable	MAP4K2
0.000118963	stable	LOC650215
0.00011924	stable	INPP4B
0.000119401	stable	LRRC2
0.000119414	stable	GATA4
0.000119432	stable	CYBASC3
0.000119432	stable	KIAA0319L
0.000119474	stable	GRIPAP1
0.000119894	stable	LRRC3B
0.000121029	stable	SLC2A11
0.000121152	down	EGR1

0.000121487	stable	EMD
0.0001215	stable	RHBDL1
0.000121649	stable	HIST1H4E
0.00012243	stable	MGC16169
0.00012282	stable	GUCA1B
0.000122847	stable	LOC728689
0.000122915	stable	DDAH1
0.000123264	stable	C14ORF153
0.000123537	stable	TRIM56
0.000123597	stable	DYSFIP1
0.000123793	stable	MLL
0.000123846	stable	NMRAL1
0.00012469	stable	LOC653226
0.00012476	stable	FTO
0.00012635	stable	UTP18
0.000126386	stable	CHD6
0.00012647	stable	PRUNE
0.000127048	stable	HLA-DRB6
0.000127549	stable	ATP6V1A
0.000128097	stable	C5ORF13
0.000128244	stable	RASGRP2
0.000128605	stable	PLEKHA4
0.000128737	stable	C18ORF55
0.000129026	stable	TRAM1
0.000129135	stable	GOLGA6B
0.000129365	stable	C3ORF18
0.000129561	stable	TMC6
0.000129561	stable	C14ORF180
0.000129696	stable	SLFN11
0.000129895	stable	HLA-DMA
0.00013032	stable	IGSF6
0.000130472	stable	ECD
0.00013056	stable	LOC221442
0.000130817	stable	OXSR1
0.000131304	stable	LOC645937
0.000131634	stable	MKRN1
0.000131748	stable	ZKSCAN2
0.000131748	stable	TFF3
0.000132335	stable	PDE6A
0.000132584	stable	LOC653082
0.000133521	stable	S1PR4
0.000133595	stable	WDR27
0.000133979	stable	S100PBP
0.000134404	stable	RBMS3
0.000134424	stable	LOC201725
0.000134424	stable	MAMDC2
0.000134424	down	PCDH20
0.000134464	stable	SNORD4A
0.000134694	stable	JMJD6
0.000135668	stable	CDK6
0.000135727	stable	EXOC6

0.000135882	stable	BCDIN3D
0.000137431	stable	MYO5B
0.000137432	stable	CXCL1
0.000137436	down	CPNE4
0.000137755	stable	EBPL
0.000138304	stable	CCNDBP1
0.00013868	stable	VDAC1
0.000139109	stable	LOC643031
0.000139386	stable	IL1RN
0.000139543	stable	MICALL2
0.000139543	stable	RPS5
0.000139668	stable	LRRC20
0.000139853	stable	TUBG2
0.000140135	stable	SERHL
0.000141275	stable	RBMS2
0.000141633	stable	TRIM24
0.000142183	stable	ERCC1
0.000142643	stable	REXO2
0.000142812	stable	ZNF200
0.000142855	stable	HCG4
0.000142855	stable	SERTAD2
0.000143875	stable	FKBP1A
0.000144059	stable	NME4
0.00014473	stable	NFKBIB
0.000145087	stable	IMMT
0.000145817	stable	HELLS
0.00014612	stable	B4GALT7
0.000146648	stable	LOC650826
0.000146649	stable	PPHLN1
0.000146848	stable	MRPS26
0.000147066	stable	CSNK1E
0.000147091	stable	LOC153561
0.000148206	stable	PDE3B
0.000148271	stable	CABP1
0.000148697	stable	ITGAX
0.000149712	stable	WIBG
0.000149712	stable	TPD52L2
0.000149712	stable	TCAP
0.000149875	stable	ARL1
0.000150193	stable	PLCB4
0.000150805	stable	C2CD2
0.000150818	stable	CHMP4A
0.000151405	stable	RAPSN
0.000151775	stable	COQ5
0.000151973	stable	POMZP3
0.000151973	stable	ZBED5
0.000152314	stable	EMILIN3
0.000152347	stable	TXN
0.000152787	stable	TCEA2
0.000154988	stable	NR4A3
0.000155058	stable	KCNN3

0.000155515	stable	SLC35E3
0.000155515	stable	C10ORF54
0.000155766	stable	NRBP2
0.000155766	stable	BCKDHA
0.000156009	stable	TUSC3
0.000156253	stable	DNLZ
0.000156967	stable	FCER1A
0.000157293	stable	UCK2
0.000157994	stable	MTHFS
0.000158413	stable	MBD5
0.000158433	stable	TMEM139
0.000159043	stable	WBP2
0.000160656	stable	TBRG4
0.000161378	stable	MRPL36
0.000162286	stable	TGFBI
0.000162497	stable	SLC29A1
0.000162667	stable	LOC647834
0.000162736	stable	SNORD36C
0.000163044	stable	KIAA0195
0.000163459	stable	GALNTL4
0.000163494	stable	UBE2D3
0.000163836	stable	CATSPER2
0.00016391	stable	JUP
0.000164539	stable	CGNL1
0.000165034	stable	SOX6
0.000165729	stable	XRN2
0.000166569	stable	HDGFRP3
0.000166796	stable	FYN
0.000166968	stable	QKI
0.000167094	stable	LCN2
0.000167375	stable	C11ORF65
0.000167399	stable	GPAA1
0.000167461	stable	ZNF259
0.00016751	stable	KLHL4
0.000167693	stable	SNX16
0.000167693	stable	MRPL11
0.000167716	stable	RP2
0.000168095	stable	USP12
0.000168095	stable	PRDX3
0.000168095	stable	PLN
0.000168934	stable	CACNA1B
0.000169102	stable	PACS1
0.000170457	stable	TLE4
0.000170854	stable	LACTB2
0.000171024	stable	DCTN1
0.00017107	stable	MRPL3
0.000171327	stable	CAPN2
0.000171436	stable	C10ORF119
0.000171615	stable	LIMS1
0.000171615	stable	LOC390705
0.000171633	stable	PAIP2

0.000172903	stable	LOC650111
0.000173003	stable	COQ10A
0.000173082	stable	COASY
0.000173106	stable	ASB13
0.000173128	stable	WBP4
0.000173136	stable	RHBDL3
0.000174945	stable	ITGA2
0.000175048	stable	ANXA7
0.00017545	stable	BAT1
0.000175594	stable	TNFRSF25
0.000175924	stable	MYL3
0.000176011	stable	NUAK2
0.000176495	stable	KLHL11
0.000176495	stable	DDX3X
0.000176693	stable	USP15
0.000176694	stable	SMARCC1
0.000177315	stable	THAP4
0.000177316	stable	PEBP1
0.000177629	stable	FGFR3
0.00017856	stable	LOC51057
0.000178995	stable	PTPRD
0.0001791	stable	SH3GLB2
0.000179329	stable	LOC388524
0.000180083	stable	LOC729776
0.000180088	stable	TXNL2
0.000180747	stable	LOC643665
0.000181062	stable	TTLL12
0.000181062	stable	ATG7
0.000182	stable	C3ORF34
0.000182478	stable	BCL9L
0.000182758	stable	PAK1IP1
0.000182869	stable	ZBTB17
0.000182978	stable	COL20A1
0.000184491	stable	KIAA0430
0.000184557	stable	ZNF441
0.000185083	stable	FNTB
0.000185314	stable	TLR2
0.000185837	stable	C7ORF50
0.000186548	stable	IQGAP1
0.000186702	stable	DMAP1
0.000187213	stable	HIGD2A
0.000187292	stable	KLHL28
0.000187449	stable	EXOC1
0.000188562	stable	CIDEC
0.000189487	stable	THTPA
0.0001895	stable	ERLIN2
0.000189704	stable	CCNJ
0.000189776	stable	AQP7P2
0.00019007	stable	BBX
0.000190236	stable	GSTP1
0.000190293	stable	TCF20

0.000190707	stable	OXCT2
0.000190707	stable	KIAA1618
0.000191423	stable	FAM14A
0.000191593	stable	CALD1
0.000191642	stable	BCL2L1
0.000192171	stable	UBR5
0.000192177	stable	CEP350
0.000192736	stable	NET1
0.000193718	stable	RETSAT
0.000193766	stable	TK1
0.000193861	stable	NMT2
0.000194008	stable	DEF6
0.000195181	stable	MTX1
0.000197861	stable	RPIA
0.00019916	stable	APEX2
0.00019916	stable	MTX2
0.000199836	stable	LOC648304
0.000199865	stable	LRBA
0.000200138	stable	STK17B
0.000200607	stable	TUBB4
0.000200918	stable	LOC285053
0.000201314	stable	FAM40B
0.000201663	stable	DKK1
0.000201757	stable	PGR
0.000201793	stable	MED27
0.00020181	stable	TEK
0.000202068	stable	SGMS2
0.000202251	stable	SP4
0.000202251	stable	BMS1
0.000202405	stable	C14ORF93
0.000203744	stable	C16ORF80
0.000204063	stable	HMBS
0.000204794	stable	ZFP91
0.000205387	stable	PSMC3IP
0.000205647	stable	ZNF428
0.000206128	stable	ALG8
0.000206287	stable	PPP1R12C
0.000208686	stable	SOD2
0.000209043	stable	IGFBP3
0.000209909	stable	ARC
0.000210323	stable	ERMP1
0.000211548	stable	LOC652815
0.000211616	stable	COX17
0.000212486	stable	CXORF45
0.000212496	stable	BRPF3
0.000212496	stable	AP1G2
0.000213411	stable	SCYL1
0.000213886	stable	DUSP19
0.00021438	stable	KCNC4
0.00021438	stable	LOC647210
0.000215204	stable	LOC149134

0.000215323	stable	MUT
0.00021541	stable	SAFB
0.000215418	stable	TOP2B
0.00021638	stable	KIAA0556
0.000217057	stable	ASIP
0.000217488	stable	DSEL
0.000219049	stable	PGS1
0.000219049	stable	PIGK
0.000219169	stable	DHX34
0.000219589	stable	AKNA
0.000219652	stable	TBX15
0.000219706	stable	SNORD14B
0.000220946	stable	PALLD
0.000220946	stable	RNF11
0.000221763	stable	TNFSF4
0.000222163	stable	PCNA
0.000222515	stable	RPL7L1
0.000222515	stable	P4HB
0.000223955	stable	RUVBL2
0.000226038	stable	SRP14
0.000226082	stable	MAPRE2
0.000226082	stable	CTHRC1
0.000226846	stable	CYP2R1
0.000227181	stable	XPO6
0.000227181	stable	P8
0.000227585	stable	TRIB3
0.000229239	stable	ASXL1
0.000231122	stable	ALKBH2
0.00023175	stable	WDR69
0.000232018	stable	ARL15
0.000232302	stable	C16ORF58
0.000233874	stable	TLL1
0.000234331	stable	MOSPD2
0.000238346	stable	AVEN
0.000238428	stable	FLJ36070
0.000239615	stable	RNASEL
0.000241641	stable	SIRT7
0.000242883	stable	MESDC1
0.000242883	stable	LAMB2
0.000242883	stable	GAS7
0.000242883	stable	SP110
0.000242892	stable	ODF2
0.000243484	stable	KRT10
0.000243484	stable	ZFP1
0.000243484	stable	TBL2
0.000243752	stable	MTERFD1
0.000246548	stable	LOC646567
0.000248803	stable	C12ORF45
0.00024967	stable	FLJ46230
0.00025003	stable	MICAL1
0.000250071	stable	C6ORF173

0.000250438	stable	NUP85
0.000250826	stable	DCUN1D1
0.000252974	stable	RPL18A
0.000253227	stable	XRCC2
0.000253387	stable	GMEB2
0.000253603	stable	UBR2
0.000254111	stable	TUBA8
0.000255097	stable	ABCA10
0.00025522	stable	DCTN4
0.000256104	stable	GNPAT
0.000258483	stable	C6ORF47
0.000259118	stable	FABP6
0.000260818	stable	P2RY6
0.000261166	stable	INPPL1
0.000261727	stable	TMEM14B
0.000263326	stable	PTGS1
0.000263664	stable	EFHC1
0.000263678	stable	PTPRE
0.000263875	stable	C12ORF49
0.000265534	stable	DNAH1
0.000265653	stable	TMED5
0.000265939	stable	ANKRD40
0.000266213	stable	FANCG
0.000266386	stable	HOOK1
0.00026642	stable	CHCHD7
0.000267008	stable	DENND1B
0.000269188	stable	C9ORF86
0.000269771	stable	YARS2
0.000272217	stable	PPAPDC2
0.000272728	stable	IRAK2
0.000273235	stable	C6ORF1
0.00027341	stable	SUPT16H
0.000275146	stable	PUS10
0.00027595	stable	SMCR7
0.000279019	stable	ANGPTL7
0.00027925	stable	TBX3
0.000279323	stable	EPPB9
0.000279492	stable	ITFG1
0.000280005	stable	CASQ2
0.000280066	stable	OAT
0.000280488	stable	TRIM29
0.000280576	stable	PCNP
0.000280852	stable	RLN2
0.000283116	stable	LOC644254
0.000283116	stable	SMC3
0.000283937	stable	WDR35
0.000284531	stable	ANO10
0.000285176	stable	ZNF137
0.000286232	stable	CSNK1G2
0.000287471	stable	PAN2
0.000288575	stable	EIF3C

0.000288611	stable	TIGA1
0.000288611	stable	ARCN1
0.000288847	stable	C15ORF15
0.000289085	stable	MAP3K7
0.000289682	stable	CBARA1
0.000290112	stable	ANO7
0.000291953	stable	SPR
0.000291953	stable	LOC441108
0.000291997	stable	KLHL30
0.000292393	stable	GAS8
0.000293678	stable	C1ORF181
0.000293907	stable	CLEC7A
0.000294118	stable	C1ORF163
0.000294827	stable	PTX3
0.000296227	stable	TMEM86A
0.000298503	stable	SOX13
0.00030145	stable	OAZ1
0.000301691	stable	LOC653505
0.000302056	stable	C3ORF45
0.000302232	stable	ZCWPW1
0.000302865	stable	ANXA2P1
0.000302876	stable	NCOA2
0.000303299	stable	R3HDM2
0.000304491	stable	ZSWIM5
0.000306432	stable	PPP1R16B
0.000307545	stable	BEXL1
0.000310131	stable	CPXM1
0.000310426	stable	CDGAP
0.000312531	stable	LCMT2
0.000313421	stable	RAB7A
0.000314162	stable	KIF1A
0.000314231	stable	IFT81
0.000314233	stable	COL5A3
0.00031486	stable	PSMD2
0.000314879	stable	TCEAL2
0.000315105	stable	LOC728153
0.00031555	stable	UTX
0.000316798	stable	NXN
0.000319451	stable	PMP22
0.000319751	stable	SNORD46
0.000319879	stable	NOL14
0.000320106	stable	LOC648984
0.00032214	stable	MEGF9
0.000322695	stable	C15ORF57
0.000323309	stable	CWF19L2
0.000323691	stable	C1ORF122
0.000323691	stable	ESRRA
0.000323867	stable	N4BP2L1
0.00032447	stable	POLR2L
0.000324868	stable	NAPEPLD
0.000327671	stable	ALDH3B1

0.000327905	stable	SLC6A18
0.000329635	stable	BXDC1
0.000330081	stable	KIAA0391
0.000330523	stable	ZMYM1
0.000331347	stable	PCNX
0.000333361	down	FOSB
0.000333361	stable	SYNJ2
0.000334342	stable	PI4K2B
0.000335949	stable	C7ORF20
0.000336633	stable	TBCC
0.000337382	stable	TRIM68
0.000337572	stable	CSTF3
0.000338942	stable	TCEB1
0.000340209	stable	BRPF1
0.00034335	stable	CENTA1
0.000344221	stable	LOC401115
0.000344923	stable	IRX4
0.000345788	stable	SRXN1
0.000346601	stable	KIAA0892
0.000347089	stable	WDR91
0.000347676	stable	CASP1
0.000348026	stable	NDUFB4
0.000348762	stable	MCM4
0.000348885	stable	HPSE
0.000351086	stable	SLC25A5
0.000355111	stable	DUSP3
0.000355892	stable	RTN4IP1
0.000356383	stable	FLJ39639
0.000356758	stable	KIFAP3
0.000356883	stable	UBD
0.000358181	stable	B4GALT4
0.000358827	stable	NKX2-5
0.000358873	stable	MAPK3
0.000358932	stable	LOC641978
0.000361675	stable	GMIP
0.000361786	stable	GCNT1
0.000362598	stable	GTPBP8
0.000362897	stable	CD4
0.000363481	stable	C3ORF38
0.000365526	stable	C21ORF122
0.000366121	stable	TCN2
0.000367123	stable	CENTG2
0.000367165	stable	LASS6
0.000367165	stable	FLJ23834
0.00036738	stable	REEP1
0.000368182	stable	CHEK2
0.000369967	stable	RAB6A
0.000369967	stable	ITGA10
0.000370046	stable	HIST3H2A
0.000373424	stable	ZNF266
0.000374294	stable	PRPF40B

0.000376024	stable	JTB
0.000376265	stable	MXRA7
0.000378939	stable	METTL9
0.000379045	stable	C3ORF19
0.000379193	stable	LOC644869
0.000379399	stable	TLR5
0.000379755	stable	RXRA
0.000380512	stable	RFTN2
0.000381264	stable	CTSH
0.00038298	stable	CENTD1
0.000387192	stable	KIAA0251
0.000387307	stable	SAV1
0.000388471	stable	RUFY2
0.00038937	stable	UBE3C
0.000390254	stable	GANAB
0.000390763	stable	LYPLA2
0.000391518	stable	SFRS2
0.000392427	stable	ADO
0.000392704	stable	FMO3
0.000392704	stable	RNASEN
0.000392895	stable	C8ORF55
0.000393377	stable	DBNDD2
0.000394611	stable	LOC85389
0.000394611	stable	MON1B
0.000398183	stable	C14ORF149
0.00039947	stable	RGS18
0.000401113	stable	FLJ35429
0.000403379	stable	SEMA4A
0.000403746	stable	REV1
0.000403867	stable	NDUFV1
0.000404172	stable	STAM
0.000404246	stable	TOP3A
0.000404372	stable	C21ORF91
0.000406017	stable	TRIP13
0.000407035	stable	LOC26010
0.000407385	stable	NQO1
0.000409827	stable	TTLL4
0.000411349	stable	LYRM5
0.000411608	stable	SCAND1
0.000412246	stable	TCTN1
0.000413055	stable	LOC730083
0.000417324	stable	SHANK3
0.000418238	stable	ADCY1
0.000418554	stable	ATP5G1
0.000418698	stable	COL9A2
0.000419509	stable	WDR92
0.000421777	stable	JARID1B
0.000422345	stable	EXOC4
0.00042299	stable	DNAL1
0.000423173	stable	SNHG3-RCC1
0.000423794	stable	SMCHD1

0.000423982	stable	LOC401284
0.000425401	stable	LDLRAP1
0.000425929	stable	SSBP2
0.000428042	stable	AKR1C3
0.000428088	stable	ZBTB16
0.000428128	stable	PPARGC1A
0.000429143	stable	SLC16A12
0.000429427	stable	LOC645018
0.00042971	stable	CCDC77
0.00042971	stable	MTX3
0.000431158	stable	ASMTL
0.000433816	stable	GALE
0.000433928	stable	VEZT
0.000434445	stable	SCN3B
0.000437341	stable	MLYCD
0.000437341	stable	MMD
0.000438163	stable	UGDH
0.000438567	stable	NUFIP2
0.000438905	stable	EDARADD
0.000438968	stable	ELP2
0.000439456	stable	C22ORF30
0.000440497	stable	PPAPDC3
0.000440943	stable	HADH2
0.000442054	stable	DNALI1
0.000442163	stable	ARIH1
0.000442163	stable	BTG3
0.000444674	stable	KIAA0802
0.000445417	stable	STAMBP
0.00044604	stable	RIOK3
0.000446199	stable	AMY1A
0.000446928	stable	RPS15
0.000449708	stable	PSD3
0.000449768	stable	TMEM63A
0.000452027	stable	EBF3
0.000453318	stable	TFCP2
0.000453703	stable	LOC440928
0.000453708	stable	DGCR6
0.000454815	stable	GMEB1
0.000456108	stable	MRPL55
0.000456461	stable	NDST2
0.000456566	stable	EPC1
0.000456724	stable	MOCS2
0.000457338	stable	MRPS35
0.000457338	stable	LOC653479
0.000458765	stable	NMNAT1
0.0004602	stable	STAT6
0.000460748	stable	SLC2A14
0.000460748	stable	SPRYD4
0.000461406	stable	PITX3
0.000463185	stable	PIK4CA
0.00046424	stable	VCAN

0.000467425	stable	ZNF416
0.00046842	stable	JUN
0.000468642	stable	SRRD
0.000469178	stable	HIVEP1
0.000470153	stable	AUH
0.000470788	stable	AZI2
0.000474337	stable	ALKBH3
0.000476146	stable	RAB43
0.000477055	stable	SENP6
0.000477055	stable	LMBR1
0.000479268	stable	KIAA0776
0.000480429	stable	LY6E
0.000480976	stable	PARK7
0.000480979	stable	TMEM37
0.000482025	stable	LOC727935
0.00048431	stable	PCYOX1
0.000484822	stable	LOC644390
0.000488982	stable	DCK
0.000489061	stable	AKAP6
0.000489163	stable	PFTK1
0.000489163	stable	LRAP
0.000489741	stable	C1ORF218
0.000490254	stable	NUCB1
0.000493019	stable	LOC644863
0.000494108	stable	MYO1F
0.000498021	stable	NT5DC2
0.00050027	stable	ADNP
0.000500799	stable	PIK3CD
0.000503649	stable	PKN2
0.000503969	stable	OGT
0.000503969	stable	C8ORF4
0.000517679	stable	PCNT
0.000517758	stable	ATP5SL
0.000518599	stable	FOXC2
0.000518815	stable	HCG9
0.000520175	stable	C7ORF63
0.000521683	stable	TMEM216
0.000531414	stable	MCTP1
0.00053511	stable	MGC20983
0.000536762	stable	ECM1
0.000537077	stable	DST
0.00053843	stable	AATF
0.000543144	stable	SOCS4
0.000543144	stable	GOLGA8A
0.000545119	stable	CYB5R1
0.000545864	stable	SETD2
0.000546149	down	ADH1A
0.000548464	stable	NOL5A
0.000548575	stable	RBM28
0.000549474	stable	AMMECR1
0.000549871	stable	LRP3

0.000549871	stable	RASAL2
0.000550099	stable	ZNF658B
0.000550662	stable	KCTD14
0.000551122	stable	SPAG9
0.000551169	stable	ANKRD37
0.000551169	stable	COL14A1
0.000552082	stable	LOC202051
0.000552939	stable	MGC72104
0.000556904	stable	TRIM22
0.000556904	stable	PVRL3
0.000556983	stable	C20ORF39
0.00055841	stable	RPL8
0.000560236	stable	ZMIZ1
0.000560801	stable	STX5
0.000562565	stable	AS3MT
0.000564336	stable	RYBP
0.000565652	stable	NFU1
0.000568588	stable	RNF41
0.000571885	stable	ZNF136
0.000572099	stable	C21ORF124
0.000572653	stable	ADAMTS1
0.000579001	stable	UTP14C
0.000580308	stable	C2ORF49
0.000581218	stable	CSNK1G3
0.000582133	stable	CXORF38
0.000583669	stable	LOC651575
0.000583677	stable	GTF3C1
0.000583924	stable	PACS2
0.00058456	stable	HSPA1B
0.000586539	stable	HOXC11
0.000587977	stable	DMPK
0.00058864	stable	MOCOS
0.00058864	stable	NDUFB5
0.000589067	stable	ZNF691
0.000589103	stable	MED20
0.000589386	stable	MYOZ1
0.000590855	stable	ZNF14
0.00059218	stable	HMGA1
0.00059218	stable	LOC644284
0.000594427	stable	E4F1
0.000594496	stable	MAP3K1
0.000594623	stable	AK2
0.000596655	stable	IMPDH2
0.00059808	stable	MARVELD2
0.000598164	stable	PDHX
0.000598621	stable	VPS11
0.000600285	stable	FAM23B
0.000600285	stable	C14ORF102
0.000601379	stable	TGDS
0.000603764	stable	FLNC
0.000605393	stable	LOC642732

0.000605393	stable	IFNGR2
0.000606469	stable	CHRM2
0.000609537	stable	CBFB
0.000610091	stable	ZNF438
0.000610862	stable	HIST1H1A
0.000612598	stable	MS4A6E
0.000613649	stable	CYHR1
0.000614822	stable	GTF3C2
0.000621558	stable	NOLC1
0.000624916	stable	LOC653066
0.000625521	stable	BEX4
0.000626545	stable	DCP1A
0.000626708	stable	UBL4A
0.000627843	stable	C10ORF6
0.000628251	stable	MOG
0.000628251	stable	GRAMD3
0.000629347	stable	MICAL2
0.000630307	stable	GIMAP8
0.000632273	stable	C9ORF127
0.000633476	stable	THRAP5
0.000634279	stable	GTDC1
0.000634316	stable	PPP1CC
0.000635617	stable	LAP3
0.000638736	stable	C3ORF58
0.00063922	stable	PRKRIP1
0.000639786	stable	NRIP3
0.000640392	stable	PECAM1
0.000640398	stable	ZNF543
0.000641442	stable	PPA1
0.000642075	stable	LOC729148
0.000642582	stable	ZC3H3
0.000643595	stable	ADORA2B
0.000644118	stable	FIGNL1
0.00064734	stable	SHKBP1
0.000648418	stable	CXCL10
0.000648998	stable	FLJ22222
0.000651595	stable	LOC645100
0.000651624	stable	EFHA2
0.00065298	stable	HLA-DRB3
0.000653028	stable	GSDMB
0.000653033	stable	C4ORF30
0.000653081	stable	CPLX3
0.000653391	stable	OXSM
0.000654021	stable	LOC440704
0.000654525	stable	SFRS4
0.000656393	stable	ZNF2
0.000657348	stable	RPL3
0.000660961	stable	FZD7
0.000660961	stable	NDUFB7
0.000661731	stable	DNER
0.000665722	stable	LOC653394

0.000666432	stable	DKFZP686I15217
0.000667622	stable	SERPING1
0.000667748	stable	LOC651149
0.000668154	stable	RAB13
0.000670443	stable	STRBP
0.000671417	stable	SHC1
0.000673235	stable	LOC653342
0.000677371	stable	COPZ1
0.000677626	stable	GTF2E1
0.000678997	stable	GSTK1
0.000679931	stable	ALDOA
0.000685198	stable	LOC652826
0.00068747	stable	CAPN5
0.000687819	stable	LOC653354
0.000691518	stable	BIN3
0.000694178	stable	PUM1
0.000696675	stable	ARPC5
0.000697191	stable	TOR1B
0.000698234	stable	PPM1A
0.000699079	stable	LOC196993
0.000700266	stable	VPS35
0.000702139	stable	RGR
0.000708543	stable	NAGA
0.000711198	stable	MAGT1
0.000712166	stable	ZNF688
0.000712367	stable	PMEPA1
0.000713095	stable	PLCZ1
0.000715983	stable	PCDH9
0.00071669	stable	C14ORF43
0.000717239	stable	OSBP2
0.000719428	stable	EPM2A
0.000720329	stable	AURKAIP1
0.000723947	stable	CACNA2D2
0.000724526	stable	ZNF687
0.000724685	stable	LOC653158
0.000727303	stable	CCNG1
0.000727303	stable	YTHDF3
0.00073076	stable	C12ORF34
0.000732974	stable	FAM83D
0.000733837	stable	PMS2CL
0.000735925	stable	NUDT2
0.000739576	stable	CDK5RAP2
0.000742408	stable	RILPL2
0.000744112	stable	LOC124512
0.000744349	stable	SSFA2
0.000747702	stable	FADS3
0.000748443	stable	RHBDD1
0.000752345	stable	LSM4
0.000752345	stable	CUL4B
0.000753688	stable	CBS
0.000753688	stable	SUSD2

0.000754076	stable	LEO1
0.000754171	stable	RFXANK
0.000754984	stable	POLR2I
0.000756849	stable	ZNF418
0.000756849	stable	FAM114A1
0.000757109	stable	MED25
0.0007577	stable	CLN8
0.0007577	stable	LOC401357
0.000759672	stable	GRB14
0.000765506	stable	HEATR3
0.000765721	stable	SUPT5H
0.000766173	stable	PPIL4
0.000769514	stable	SEL1L2
0.000770889	stable	HK1
0.000773419	stable	C12ORF52
0.000780546	stable	NELL1
0.000780597	stable	FAM10A7
0.000780676	stable	ATP6V1H
0.000784189	stable	PFDN6
0.000785666	stable	NEK7
0.000785883	stable	TBCCD1
0.000786983	stable	ATP6V1E1
0.000790271	stable	COPS8
0.000790453	stable	IGF2R
0.000792313	stable	COL4A6
0.000794835	stable	SSH1
0.000796885	stable	CUL4A
0.000801376	stable	NRP1
0.000802596	stable	DNM1L
0.000805465	stable	CCDC132
0.000805543	stable	PEG3
0.000809465	stable	KRR1
0.000810176	stable	PPP2R2B
0.000813634	stable	PARP16
0.000813695	stable	SELT
0.000815329	stable	NKIRAS1
0.000816185	stable	TSEN2
0.000816185	stable	GRN
0.000820476	stable	RAB40C
0.000821937	stable	GPC1
0.000822424	stable	PHF12
0.000823375	stable	THRSP
0.000823541	stable	NETO2
0.000824581	stable	CPNE1
0.000825604	stable	HSPB6
0.000825604	stable	PPP1R14C
0.000826539	stable	C20ORF196
0.000827519	stable	CENTG3
0.000831897	stable	SLC41A1
0.000832771	stable	STGC3
0.000833434	stable	CCDC90B

0.000836067	stable	HSGT1
0.000837626	stable	STAU1
0.000839802	stable	C21ORF119
0.000839837	stable	FIP1L1
0.000839837	stable	C9ORF169
0.000841354	stable	ALS2CR16
0.000841354	stable	CD274
0.000842035	stable	STAT5A
0.000842326	stable	SH3BP2
0.000843225	stable	KCTD20
0.000843225	stable	FOXRED2
0.000843225	stable	RBP7
0.000843225	stable	TM7SF3
0.000844797	stable	CPAMD8
0.000845864	stable	SMARCD1
0.000845864	stable	MCFD2
0.000845864	stable	CLIP3
0.000846072	stable	HHEX
0.000849138	stable	CYB5A
0.000851787	stable	TJP2
0.000851844	stable	C1ORF198
0.000854411	stable	APPL1
0.000858836	stable	MED8
0.000859036	stable	DEXI
0.000859794	stable	MAPK6
0.000863972	stable	MKS1
0.000864442	stable	CCDC7
0.000868435	stable	NCAM1
0.000874345	stable	LOC728417
0.000879062	stable	IDI2
0.000880427	stable	PSMD10
0.000881523	stable	TRIM2
0.000885808	stable	SLC44A2
0.000887547	stable	MECR
0.000888036	stable	GDF15
0.000888908	stable	C20ORF30
0.000893691	stable	KRAS
0.000894301	stable	YPEL5
0.000894723	stable	KIAA0020
0.000895647	stable	DLK2
0.000898182	stable	PLEKHH1
0.00090098	stable	C14ORF85
0.00090492	stable	AEBP2
0.000905458	stable	ABCC3
0.000906659	stable	EIF4EBP1
0.000907327	stable	LBR
0.000907506	stable	DSG4
0.000908174	stable	HDGF2
0.000911376	stable	FN1
0.000912248	stable	AKR1D1
0.000913742	stable	ZNF673

0.000915129	stable	CSF2RA
0.000915726	stable	NPY6R
0.000922626	stable	LOC90826
0.000922626	stable	CCBL2
0.000927604	stable	SCARB1
0.000929441	stable	LOC648581
0.000931648	stable	BECN1
0.000931931	stable	LOC644322
0.000935341	stable	ARHGAP28
0.000935983	stable	MSTN
0.000940921	stable	MYL9
0.000940921	stable	C1ORF186
0.000940921	stable	CPSF1
0.000941674	stable	LOC652541
0.000943374	stable	C5ORF29
0.000943374	stable	KCNK1
0.000944951	stable	PQLC1
0.000944953	stable	ATAD1
0.00094764	stable	TOMM20
0.000963682	stable	TIGD2
0.000964926	stable	GCS1
0.000972383	stable	MGC70857
0.000972871	stable	AQP7
0.000973378	stable	HLA-DMB
0.000974369	stable	RICTOR
0.000975718	stable	L2HGDH
0.000983208	stable	GLO1
0.000987976	stable	PPOX
0.000989464	stable	CENPN
0.000989906	stable	SUCLA2
0.000994254	stable	FBXO40
0.000994785	stable	NDUFS1
0.001000077	stable	C13ORF34
0.001002059	stable	CCL8
0.00100533	stable	MAP1A
0.001009182	stable	CCT7
0.001011039	stable	PNKD
0.001011252	stable	BTBD1
0.001018031	stable	DNAJC3
0.001018031	stable	C6ORF35
0.001019087	stable	LAS1L
0.001019852	stable	EEF1E1
0.001021242	stable	CDO1
0.001023077	stable	ZNF341
0.001029907	stable	SLC36A2
0.001030719	stable	RHOJ
0.001035658	stable	NLRC4
0.001037542	stable	PRPF31
0.001037929	stable	AASDH
0.001040229	stable	PCDHGA12
0.001042333	stable	USP31

0.001046197	stable	WDR40A
0.00104653	stable	ZNF546
0.001049248	stable	HLA-DQB1
0.001052199	stable	LCN10
0.001053715	stable	CHST3
0.001054095	stable	SLK
0.001055789	stable	NUP133
0.001058019	stable	WDR8
0.001058068	stable	MPZL2
0.001059173	stable	ZNF140
0.00106904	stable	GYG2
0.001069701	stable	CTBS
0.001069701	stable	SH3KBP1
0.00107097	stable	HSPA14
0.00107097	stable	ERCC2
0.001074428	stable	TP53I11
0.001074707	stable	CTDSPL
0.001077543	stable	TLR6
0.001077543	stable	TBL1XR1
0.001081827	stable	MRPL54
0.001081879	stable	LOC390354
0.001083046	stable	EIF1B
0.00108479	stable	LOC134997
0.001091031	stable	IQGAP2
0.001093666	stable	C5ORF35
0.0010955	stable	DIRAS1
0.001097431	stable	CREB1
0.001100263	stable	HLA-H
0.001100263	stable	PCBP2
0.001101926	stable	LOC651316
0.001103151	stable	SLC4A1AP
0.001108569	stable	DHRS3
0.001111611	stable	GCC2
0.001111611	stable	NOTCH4
0.001114262	stable	C12ORF65
0.001114319	stable	FGFRL1
0.001127692	stable	NR3C2
0.001133207	stable	ZNF160
0.001133612	stable	C12ORF4
0.001133612	stable	ISOC1
0.001135195	stable	LOC727848
0.001138313	stable	LOC647389
0.00113875	stable	GOLGA1
0.001140751	stable	HSCB
0.001141525	stable	P2RY1
0.001142894	stable	DPY30
0.001144894	stable	SYDE2
0.001144894	stable	ZBTB33
0.001146246	stable	PHB
0.001147331	stable	ESR1
0.001147421	stable	YY1AP1

0.001147421	stable	C3ORF37
0.001149594	stable	C10ORF59
0.001149933	stable	MAPRE1
0.001150191	stable	POLDIP2
0.00115059	stable	TMEM44
0.001151208	stable	ISCA1
0.001158046	stable	FLJ42875
0.0011635	stable	GMCL1
0.0011698	stable	GAB2
0.001175445	stable	PPP2CA
0.001177194	stable	ICA1
0.001179151	stable	TUSC1
0.001180931	stable	JPH3
0.001185253	stable	C15ORF52
0.001185253	stable	ROCK1
0.00118946	stable	PSCDBP
0.001191103	stable	MGC13057
0.001193354	stable	TUBA4A
0.001194057	stable	HSPG2
0.001194625	stable	VEGFA
0.001198904	stable	COL6A1
0.001198904	stable	ACO1
0.001198904	stable	BCL7B
0.001199527	stable	ATG4C
0.001200118	stable	KIF15
0.001200118	stable	LOC653157
0.001200965	stable	OSGIN1
0.001211483	stable	MAFK
0.001211483	stable	FZD8
0.00121283	stable	PLOD2
0.001212898	stable	D4S234E
0.00121683	stable	STIP1
0.001217852	stable	STX16
0.001218992	stable	C19ORF61
0.001221573	stable	RAPGEFL1
0.001224627	stable	SPOCK2
0.001224627	stable	C18ORF34
0.001230307	stable	UPF3A
0.001235032	stable	WISP1
0.001239666	stable	C13ORF1
0.001240827	stable	NEURL2
0.001243715	stable	PIBF1
0.001244076	stable	SEPN1
0.001248832	stable	GALNTL2
0.001250195	stable	EMCN
0.001252818	stable	PML
0.001254609	stable	LOC651398
0.001255539	stable	RPS3
0.001257358	stable	VPS39
0.001259035	stable	KIAA1219
0.001260242	stable	USP49

0.001260242	stable	ABHD4
0.00126192	stable	DNASE2
0.001262503	stable	LOC650683
0.001271366	stable	PARP3
0.001271537	stable	NDUFS2
0.001272943	stable	C7ORF10
0.001272943	stable	LOC728744
0.001284209	stable	INPP5D
0.001287507	stable	ZDHHC7
0.001289442	stable	BCO2
0.001289558	stable	KCNK6
0.001294613	stable	ZNF334
0.001295724	up	XIRP2
0.001298473	stable	SYPL2
0.001298473	stable	ZMYND17
0.001303412	stable	LOC643722
0.001304579	stable	LOC727762
0.001304802	stable	NUP93
0.001304802	stable	C16ORF57
0.001305175	stable	LASS2
0.001309513	stable	HNRNPUL2
0.001315683	stable	MPST
0.00131698	stable	ANKDD1A
0.001327903	stable	SNRPN
0.00132911	stable	IRF5
0.001332266	stable	TTC35
0.001333267	stable	ZNF383
0.001333582	stable	ZNF275
0.001337729	stable	LTF
0.001338071	stable	LOC730316
0.001344192	stable	ARMCX5
0.001344898	stable	EHHADH
0.001344934	stable	REV3L
0.001346393	stable	DLG4
0.001347375	stable	LOC646144
0.001351131	stable	OBFC2B
0.001358707	stable	CCR4
0.001360764	stable	ZNF606
0.001368281	stable	KCNS1
0.001370812	stable	ZFP112
0.001374167	stable	PSTK
0.00137557	stable	TMEM93
0.00137557	stable	ARSG
0.001377307	stable	FLJ35220
0.001377456	stable	SRPK2
0.001378114	stable	VSIG8
0.001378114	stable	NUDT1
0.001381644	stable	MED11
0.001391716	stable	LOC645743
0.001395449	stable	YEATS2
0.001403919	stable	LRCH1

0.001414039	stable	NCAPG2
0.001424307	stable	NANP
0.001427286	stable	TUBGCP5
0.001430745	stable	HPS4
0.001431677	stable	PCDH12
0.001434465	stable	NXPH4
0.001435368	stable	RBM38
0.001436669	stable	SAAL1
0.001437737	stable	RAPGEF1
0.001438631	stable	ARHGAP10
0.001439046	stable	LOC388474
0.001440827	stable	CD109
0.00144241	stable	LYPD2
0.001443517	stable	GMFB
0.001445	stable	TTLL1
0.001445213	stable	FAF2
0.001445213	stable	COX7A2
0.001446603	stable	LOC646531
0.001454289	stable	TBCE
0.001456948	stable	LOC652968
0.001456948	stable	SETDB2
0.001461384	stable	GNPDA1
0.001464091	stable	GSTM5
0.001468155	stable	ZNF549
0.001468155	stable	KATNAL2
0.001473479	stable	NUCKS1
0.00147355	stable	ZNF7
0.001478106	stable	ACOT4
0.001478106	stable	YBX1
0.001486928	stable	KIAA1328
0.001487815	stable	LOC550112
0.001494192	stable	HABP4
0.001495299	stable	EPHA2
0.001495299	stable	ILF2
0.00149683	stable	TRO
0.001498664	stable	NMI
0.001500043	stable	ARF1
0.00150191	stable	NPAT
0.001502427	stable	NR2C2
0.001503386	stable	LOC341457
0.001503386	stable	FLJ22374
0.001506061	stable	HK2
0.001506921	stable	CPXM2
0.001512271	stable	BCL11A
0.001513012	stable	FAM80A
0.001513185	stable	TMEM106C
0.001513226	stable	POLR2J3
0.001514241	stable	C7
0.001514748	stable	BAI3
0.001519329	stable	ZNF577
0.001519329	stable	OCRL

0.001519611	stable	MFAP1
0.001521291	stable	C6ORF199
0.001526201	stable	PLEC1
0.0015299	stable	TMEM17
0.001533089	stable	PHF10
0.001538385	stable	DDX50
0.001543588	stable	TSR1
0.001547693	stable	SCMH1
0.001547693	stable	PFKM
0.00154776	stable	LOC348262
0.001548729	stable	ARPC2
0.001552845	stable	FAM186B
0.001552912	stable	AMOT
0.001554654	stable	CHST10
0.001554892	stable	TMEM104
0.001554968	stable	C14ORF19
0.001560507	stable	APAF1
0.001563317	stable	LOC442501
0.001565303	stable	BCOR
0.001568107	stable	FLJ46309
0.001571118	stable	KIF21A
0.001575608	stable	NCALD
0.001588592	stable	TNRC15
0.001589643	stable	LOC644039
0.001590797	stable	FLJ37464
0.001603975	stable	GSPT2
0.001606102	stable	NDFIP2
0.001608093	stable	NEDD8
0.001608093	stable	CSDE1
0.001608249	stable	LOC402560
0.001610655	stable	PLXDC2
0.001613034	stable	ALDH1L2
0.001613558	stable	SBF2
0.001619093	stable	LOX
0.001620353	stable	LTA
0.001625056	stable	C16ORF56
0.00163251	stable	TIGD1
0.001632657	stable	IL2RA
0.001633526	stable	GLRX
0.001633954	stable	HSPC268
0.001645285	stable	ORC4L
0.001646133	stable	MTG1
0.001646719	stable	NDUFC1
0.001655847	stable	GPR161
0.001668768	stable	FUCA2
0.001670645	stable	SERPINE2
0.001670645	stable	PLDN
0.001675712	stable	PGBD5
0.001677774	stable	C2ORF63
0.001681647	stable	ARL6IP5
0.001683547	stable	TNFSF13

0.001685621	stable	UBE3B
0.001691604	stable	TMED3
0.001692628	stable	VPS37D
0.001693579	stable	VGF
0.001695789	stable	LOC347544
0.001695789	stable	LYPD5
0.001699109	stable	DPP7
0.001699732	stable	PTPN18
0.001701527	stable	C6ORF85
0.001704047	stable	TSPAN8
0.001709021	stable	NUDT9P1
0.001712399	stable	CYB561
0.001712399	stable	NAP1L2
0.001713887	stable	EBF2
0.001715009	stable	RNMT
0.001715726	stable	MOCS1
0.001717774	stable	C6ORF117
0.001734126	stable	INSC
0.001735695	stable	ZNF669
0.001740796	stable	NIT2
0.001752554	stable	PLEKHN1
0.001754599	stable	TLN1
0.00175911	stable	MCAM
0.00175911	stable	ST6GAL1
0.00175911	stable	ZNF668
0.001759131	stable	NAT14
0.001759131	stable	ALG5
0.001759131	stable	ZNF34
0.001775009	stable	NIF3L1
0.001776363	stable	C7ORF26
0.001776596	stable	SRP72
0.001781077	stable	SRP9
0.001782997	stable	LOC399744
0.001786259	stable	SCCPDH
0.001786259	stable	TMEM129
0.001790305	stable	SPG3A
0.00179263	stable	APIP
0.001792654	stable	RAB17
0.001793724	stable	NEU1
0.001793724	stable	FAM174A
0.00179379	stable	NECAP2
0.001794161	stable	DKFZP686O24166
0.001794518	stable	AHSA2
0.001798505	stable	PMPCA
0.001815454	stable	RORA
0.001819226	stable	DNAJC5
0.00182435	stable	BRD3
0.001826303	stable	C8ORF58
0.001832655	stable	CLPP
0.001833655	stable	C11ORF67
0.001837713	stable	LOC729466

0.001838232	stable	TMEM106B
0.001838306	stable	ASPM
0.001839608	stable	CCR6
0.001842012	stable	IDH3G
0.001847805	stable	TMEM161A
0.001847805	stable	LOC643466
0.001847805	stable	IL1B
0.001847805	stable	TRAPPC4
0.001857532	stable	TTC32
0.001858232	stable	PAK6
0.001859686	stable	GNAO1
0.001861325	stable	ARL2BP
0.001861325	stable	ZNF662
0.001862749	stable	TM6SF1
0.001862749	stable	UBC
0.001862892	stable	WDR24
0.001864372	stable	LOC123688
0.001864401	stable	TCF4
0.001869954	stable	FUBP3
0.001870651	stable	ABAT
0.001870651	stable	B3GALNT1
0.001880153	stable	C12ORF30
0.001880948	stable	C5ORF39
0.00188189	stable	SSR3
0.001885681	stable	HEATR6
0.001890464	stable	LOC642755
0.001895382	stable	GLUD1
0.001895771	stable	C6ORF151
0.001895771	stable	C10ORF84
0.001897631	stable	FCER2
0.001898876	stable	TMEM178
0.001898913	stable	REP15
0.001898967	stable	AKTIP
0.00189931	stable	BNIPL
0.00189931	stable	NIPSNAP3A
0.001901087	stable	DAP3
0.001901265	stable	SNORD13
0.001906514	stable	FAM158A
0.001908997	stable	NAGK
0.001923693	stable	TMEM192
0.001923693	stable	STK4
0.001927682	stable	CANT1
0.001927851	stable	MTMR14
0.001934215	stable	SLC25A36
0.001936771	stable	CPNE5
0.001945678	stable	SNRPA
0.001949858	stable	LOC400879
0.001951322	stable	TMEM54
0.001952745	stable	PGRMC1
0.001957273	stable	MYL6
0.001957273	stable	TRUB2

0.001964437	stable	FAM107B
0.001966501	stable	ULK1
0.001966951	stable	SH3BP4
0.00196934	stable	SIL1
0.001970589	stable	RNF34
0.001971163	stable	IL10RA
0.001971534	stable	FBXL10
0.001975019	stable	COG7
0.001977284	stable	LAMP1
0.001986029	stable	C1ORF123
0.001990541	stable	NCF1
0.001997597	stable	MAST4
0.001997651	stable	P2RX4
0.002005671	stable	LOC728772
0.002009468	stable	LOC653610
0.002013118	stable	NLRP7
0.00201337	stable	ASF1A
0.002015596	stable	MATR3
0.002035392	stable	LOC653506
0.002035392	stable	FAM13C1
0.002040048	stable	ATXN7L3
0.002046336	stable	EDN2
0.002074929	stable	MMP2
0.002075003	stable	VPS36
0.002080781	stable	UBE2A
0.002091409	stable	SIGLEC16
0.002091409	stable	CDK10
0.002096067	stable	TC2N
0.002100724	stable	NUDT3
0.002101564	stable	ZSCAN12
0.00210477	stable	ZNF679
0.002118888	stable	TMEM218
0.002135964	stable	LOC642852
0.002140886	stable	SLC7A6
0.002143733	stable	AASDHPPT
0.002148255	stable	MSH2
0.00215401	stable	C19ORF4
0.002158805	stable	CRYZ
0.002162317	stable	HSF2
0.002162869	stable	KIF13B
0.002163086	stable	AKAP12
0.002175774	stable	LOC654201
0.002185265	stable	NAP1L5
0.002194521	stable	FAM78B
0.00219628	stable	DIS3
0.002202214	stable	PRMT5
0.002205312	stable	LOC441034
0.002213849	stable	SLFNL1
0.002220665	stable	LOC644162
0.00222223	stable	TATDN2
0.002222612	stable	CYP2S1

0.002222612	stable	ANKRD57
0.002229757	stable	STT3A
0.002230011	stable	LPPR4
0.002230476	stable	MGC61598
0.002234294	stable	ASH2L
0.002242266	stable	RALA
0.002244816	stable	FLJ44076
0.002250155	stable	C9ORF72
0.002258	stable	WDR21B
0.002261746	stable	HRASLS3
0.002269198	stable	HP
0.002270301	stable	LOC642412
0.002286829	stable	PTPRA
0.002290843	stable	UBE4B
0.002295431	stable	FAM108A3
0.002300231	stable	BCAR3
0.002300511	stable	C11ORF68
0.002303786	stable	ADI1
0.002304844	stable	CCDC128
0.002309747	stable	COL10A1
0.002310997	stable	CXCL12
0.002312396	stable	VIPR2
0.00231768	stable	FLJ20254
0.002331006	stable	SLC7A2
0.002331642	stable	LSM3
0.002350914	stable	AHNAK
0.002351783	stable	ANAPC4
0.002353177	stable	CCDC101
0.002358335	stable	RAB30
0.00235888	stable	ALS2CL
0.002360816	stable	ASB16
0.002360816	stable	CLIC3
0.002361517	stable	ADCY4
0.002366907	stable	SPIN1
0.002371398	stable	LOC729603
0.002376056	stable	RCBTB1
0.002377378	stable	WHSC1
0.002378075	stable	SLC1A3
0.002379585	stable	CLEC2D
0.002383551	stable	MFAP3
0.002385436	stable	LOC645196
0.002394968	stable	TTC1
0.002394968	stable	MYBBP1A
0.002400771	stable	NMNAT2
0.002404459	stable	E2F8
0.002417195	stable	SF4
0.002425655	stable	ST7
0.002426212	stable	CDCP1
0.002426326	stable	C5ORF44
0.002431936	stable	PXMP3
0.002432381	stable	RPS19

0.002433409	stable	LMBRD2
0.002437716	stable	ADAMTS7
0.002438207	stable	C2ORF29
0.00245661	stable	CA1
0.002463425	stable	MDH2
0.002464847	stable	GPSM1
0.002465215	stable	KLHL6
0.002465928	stable	HCFC1R1
0.002468352	stable	LOC649422
0.002468654	stable	C6ORF205
0.002470825	stable	GRK6
0.002471591	stable	LYNX1
0.002477337	stable	RPS18
0.002486343	stable	NSMAF
0.002493652	stable	SIGLEC5
0.002495306	stable	DDX5
0.00250093	stable	C11ORF51
0.002505244	stable	MYBPH
0.002514514	stable	C10ORF141
0.002514514	stable	SERP1
0.002524146	stable	NFRKB
0.002524601	stable	KALRN
0.002527052	stable	CEACAM1
0.002531257	stable	AKAP1
0.002541102	stable	GRPEL1
0.002542532	stable	HSPBP1
0.00254417	stable	LRRC42
0.002546718	stable	WDR73
0.002556803	stable	C16ORF59
0.002567723	stable	SENP7
0.002577599	stable	STK36
0.002584014	stable	TUBD1
0.002586771	stable	CLYBL
0.002588966	stable	LMOD1
0.002592562	stable	IGSF22
0.002596556	stable	MAGED4B
0.002601867	stable	DEFB103B
0.002625063	stable	TANC1
0.00263331	stable	PMS1
0.002638191	stable	DPY19L1
0.002647594	stable	TBL3
0.002649712	stable	MIB2
0.002651035	stable	PEX6
0.002652194	stable	LRP8
0.002661522	stable	ALG1
0.00266724	stable	PIK3R2
0.00266724	stable	UBE2T
0.00266724	stable	LOC284023
0.002676893	stable	SC5DL
0.002678699	stable	BCAR1
0.002693136	stable	ADARB1

0.0026982	stable	DKK2
0.002701714	stable	CALCB
0.002710718	stable	KIAA1012
0.002711732	stable	IL17RA
0.002714086	stable	RIPK3
0.002714086	stable	CARD11
0.002724657	stable	LOC339123
0.002735739	stable	MAP4K1
0.002739741	stable	NDUFC2
0.002761066	stable	ZNF580
0.002761066	stable	NAT1
0.002761066	stable	STC1
0.002762234	stable	KIAA1202
0.002763741	stable	SDCCAG8
0.002767408	stable	PIGU
0.002774803	stable	SUSD3
0.002775323	stable	RHOBTB1
0.002779239	stable	SPCS3
0.0027818	stable	POLA1
0.002783355	stable	ADAR
0.002786538	stable	SAMD8
0.002787318	stable	FKBP9
0.002791212	stable	SMAD5
0.002803494	stable	ZSWIM3
0.002807005	stable	EIF2S2
0.002807005	stable	PHLPPL
0.002815119	stable	EID1
0.002817045	stable	LOC442421
0.002817045	stable	C19ORF53
0.002823511	stable	RPL26L1
0.002825336	stable	HELZ
0.002825506	stable	CD36
0.002845064	stable	RAB33B
0.002861055	stable	LOC285016
0.002864475	stable	C3ORF21
0.002873342	stable	ABHD14B
0.002875251	stable	ENPP4
0.002881847	stable	SFXN2
0.002892268	stable	TIMM8A
0.002892885	stable	ABLIM1
0.002895219	stable	GPR83
0.002896511	stable	PIF1
0.002897008	stable	BCAT2
0.00290149	stable	COL27A1
0.002903623	stable	LOC647326
0.002908065	stable	PEX11B
0.002912065	stable	C10ORF58
0.002916244	stable	MALL
0.002933679	stable	PIPSL
0.002934854	stable	SMO
0.00293777	stable	KBTBD11

0.002962125	stable	MGC33846
0.002967656	stable	CRIM1
0.002968788	stable	JMJD5
0.002978909	stable	LARGE
0.00298245	stable	PEX13
0.002982595	stable	MAP6D1
0.003000713	stable	UNC50
0.003009552	stable	RABL2B
0.003014046	stable	KIAA0363
0.00301637	stable	HOXB7
0.003019752	stable	COIL
0.003028373	stable	ZBBX
0.003028373	stable	RPA3
0.003028958	stable	CDS1
0.00303044	stable	LOC643011
0.003030968	stable	GOLGA2
0.003057167	stable	SMC6
0.003068607	stable	PCOLCE2
0.003068752	stable	METT10D
0.003069594	stable	PSME3
0.003073504	stable	TTC18
0.003078531	stable	AFF4
0.00308092	stable	SR140
0.003083579	stable	ZNF573
0.00308502	stable	C12ORF10
0.003085397	stable	FEZ2
0.003091222	stable	ANKRD50
0.003097054	stable	SYNJ2BP
0.003097412	stable	BTBD12
0.003108812	stable	SS18
0.003114605	stable	RHOA
0.00311847	stable	LOC643509
0.003127956	stable	PAN3
0.00313065	stable	GTF2H1
0.003139121	stable	DHPS
0.003150407	stable	ODF3L2
0.003150407	stable	LOC731777
0.003153696	stable	EEA1
0.003155649	stable	CFL2
0.003162012	stable	HSPB1
0.00316272	stable	TTF2
0.00316272	stable	FAM127B
0.003164646	stable	LOC136143
0.003166983	stable	OGN
0.003168866	stable	BACE2
0.003174357	down	FGF12
0.003178801	stable	FGFR1
0.003180952	stable	LOC649169
0.003183845	stable	SLC22A16
0.003192851	stable	MGAT2
0.003199748	stable	LOC730092

0.00321783	stable	ST14
0.00321783	stable	PLD1
0.003247963	stable	POU5F1P1
0.003249342	stable	TNIP2
0.003255212	stable	UCP2
0.003269702	stable	SLC35A5
0.003276628	stable	MGC12966
0.003279668	stable	HLA-DOB
0.003279668	stable	ZNF432
0.003282084	stable	CTR9
0.003289959	stable	ZNF513
0.003294247	stable	SLC16A4
0.003296074	stable	ITPKB
0.003296306	stable	CHST1
0.003300474	stable	SPARCL1
0.003304168	stable	HSDL2
0.003305568	stable	LIG3
0.003313251	stable	PLOD1
0.003315595	stable	ZNF260
0.003315595	stable	PHLDA2
0.003315595	stable	QDPR
0.003324566	stable	C3ORF64
0.003344626	stable	GHDC
0.003371723	stable	NOL12
0.003377401	stable	PCBP3
0.003393277	stable	ATRNL1
0.003396755	stable	RSBN1L
0.003400119	stable	ARHGEF4
0.003406198	stable	RGN
0.003407922	stable	ZNF585A
0.003414544	stable	PHF2
0.00341483	stable	LGI1
0.003420951	stable	CASP7
0.003422669	stable	COPG2
0.003425727	stable	CSHL1
0.00343246	stable	ALG2
0.003448779	stable	TSPAN18
0.003452491	stable	FTSJD1
0.003457814	stable	LOC654174
0.003468809	stable	PSAT1
0.003470245	stable	RAB12
0.003476973	stable	DEPDC1B
0.003479672	stable	KIAA0513
0.003490001	stable	SOX8
0.003491193	stable	ARHGAP30
0.003492778	stable	FAM39E
0.003498111	stable	VIT
0.003503662	stable	HIST2H4A
0.00351505	stable	PCIF1
0.00351505	stable	C1ORF170
0.00352457	stable	EBF1

0.00352457	stable	MARCH1
0.003545969	stable	ANXA6
0.003552302	stable	ECT2
0.003554597	stable	SETD1B
0.003559644	stable	PSMA4
0.003568477	stable	LYG1
0.003572375	stable	C1GALT1
0.003581623	stable	MSR1
0.003587249	stable	SLC26A9
0.003609007	stable	CCDC21
0.003611205	stable	SF3A3
0.003611381	stable	TRIM47
0.003612174	stable	LOC346887
0.003612174	stable	NXF1
0.003618277	stable	PIP3-E
0.003619581	stable	LOC653884
0.003649585	stable	CR1
0.003654675	stable	MGP
0.003654675	stable	PLAC8
0.0036557	stable	DIS3L
0.0036557	stable	UBL3
0.003657373	stable	CCDC56
0.003670808	stable	NTN1
0.003672634	stable	C1ORF97
0.003681975	stable	CCL23
0.003681975	stable	KIAA0859
0.003701152	stable	EAF2
0.003702708	stable	PJA1
0.003703042	stable	CRELD1
0.003703042	stable	LOC652671
0.003704272	stable	GART
0.003713468	stable	LOC339879
0.003725935	stable	LOC644584
0.003726996	stable	RIN3
0.003728307	stable	LOC283547
0.003731807	stable	IMPA2
0.003733122	stable	LRRC33
0.003737905	stable	EID2B
0.003738157	stable	CLN3
0.003742	stable	RBM22
0.003749582	stable	RNF217
0.003752399	stable	LRRC28
0.003755754	stable	TERC
0.003757912	stable	LPPR2
0.003763014	stable	HLA-DQA1
0.003763312	stable	ZNF32
0.003764702	stable	CNOT7
0.003778338	stable	MED28
0.003785863	stable	LOC554203
0.003791785	stable	TGM2
0.003796613	stable	TCP11L2

0.003800484	stable	DTNA
0.003802914	stable	PIGA
0.003806043	stable	YIPF5
0.003815053	stable	SLC29A2
0.003815738	stable	SFRS7
0.003826821	stable	LOC642197
0.003829183	stable	LOC648366
0.003841367	stable	ITGA9
0.003854906	stable	RNF40
0.003854906	stable	PELO
0.003855805	stable	LOC440345
0.003856361	stable	COG3
0.003860502	stable	PROSAPIP1
0.00386133	stable	KPNA1
0.003869153	stable	TTC26
0.0038728	stable	PALB2
0.0038728	stable	TRPV1
0.003877561	stable	KIAA1407
0.003886731	stable	SOLH
0.003898394	stable	RPL22
0.003898836	stable	CRSP6
0.003906303	stable	CDT1
0.003930916	stable	HRB
0.0039533	stable	EPSTI1
0.003962184	stable	SNTB1
0.003965043	stable	LRP10
0.003970043	stable	LOC131873
0.003971053	stable	CCDC127
0.003985936	stable	IGFBP6
0.003992852	stable	PNMA1
0.004008166	stable	C9ORF40
0.004008556	stable	ETV4
0.004014822	stable	PSME1
0.004025551	stable	NPSR1
0.004026549	stable	PARP10
0.004033139	stable	ZBED1
0.004037869	stable	FLJ38482
0.004038587	stable	IRF9
0.004065869	stable	LOC400657
0.004066912	stable	RPAP3
0.004071169	stable	PCSK6
0.004078273	stable	BRD4
0.004079419	stable	FEZ1
0.004086066	stable	SMARCC2
0.004094129	stable	C1ORF85
0.004099235	stable	LOC647958
0.004100695	stable	DPP9
0.004120216	stable	C16ORF75
0.004120216	stable	PABPN1
0.004120216	stable	ALKBH5
0.004128461	stable	MYO1C

0.004129478	stable	HLA-F
0.004133778	stable	RAD51
0.004137245	stable	LOC644852
0.004138789	stable	C21ORF33
0.004139848	stable	SP3
0.004158281	stable	MRPS21
0.004187322	stable	KIF13A
0.004210633	stable	CALCOCO1
0.004213404	stable	MPI
0.004215027	stable	KLHL20
0.004217117	stable	GUCY1A3
0.004234418	stable	RPAP1
0.004238531	stable	IRAK4
0.004239393	stable	POLR3A
0.004250885	stable	C3ORF70
0.00425101	stable	GPR160
0.004258994	stable	CCNE2
0.004291148	stable	BMP2K
0.00430008	stable	LDHB
0.004305869	stable	NDUFAF2
0.004305869	stable	PSMC4
0.004312216	stable	AQR
0.004321024	stable	EIF3EIP
0.004321357	stable	SCG5
0.004344456	stable	PMS2L1
0.004351439	stable	MAN1A2
0.004355771	stable	CUGBP1
0.004381154	stable	NUSAP1
0.004381942	stable	LOC389033
0.004384031	stable	VIM
0.004386125	stable	PRNP
0.004389582	stable	PXT1
0.004389582	stable	KIAA0367
0.004389582	stable	NBEA
0.004389582	stable	C21ORF128
0.004393191	stable	PPP1R3B
0.00440628	stable	ICAM3
0.004410292	stable	RAN
0.004416853	stable	HYOU1
0.004431117	stable	PPP3CB
0.004439663	stable	GGA1
0.004440775	stable	KIAA1211
0.004443318	stable	HFM1
0.00444524	stable	RTN3
0.00447258	stable	PCDH17
0.004473626	stable	MGC3207
0.004487426	stable	DCTN2
0.004493326	stable	KLK11
0.004494771	stable	LOC653602
0.004497866	stable	TNK2
0.004499271	stable	NUAK1

0.004499271	stable	SEZ6L
0.004503729	stable	ANKRD12
0.0045042	stable	SNHG11
0.004512464	stable	MRPL16
0.004515788	stable	C14ORF172
0.004525106	stable	FLJ44124
0.004535035	stable	TMEM38A
0.004541323	stable	SFRS12
0.004545183	stable	PDGFB
0.00454799	stable	SHCBP1
0.00454799	stable	PALM
0.004551937	stable	TROVE2
0.004558174	stable	TP73L
0.004558174	stable	PRDM11
0.004559064	stable	CACHD1
0.004563398	stable	USP9X
0.004582298	stable	ZNF649
0.004582602	stable	LOC283683
0.004589342	stable	EDA
0.004592054	stable	C9ORF30
0.004600401	stable	FAIM
0.004607543	stable	TP53RK
0.004612452	stable	ETFA
0.00461601	stable	FLNA
0.004618168	stable	ZNF85
0.00461982	stable	ITGAV
0.004620155	stable	FTHL7
0.004620155	stable	SEMA6C
0.004622057	stable	PTPRN2
0.00463491	stable	ZNF518B
0.004636118	stable	ZSWIM4
0.004637705	stable	TMF1
0.004637705	stable	RN7SL1
0.004644864	stable	OSBPL7
0.004644864	stable	CD34
0.004656393	stable	TRAPPC6B
0.004660912	stable	RALY
0.004668464	stable	TBC1D19
0.004674605	stable	CHRNA5
0.004675419	stable	MPPED2
0.004697879	stable	RPL12
0.004698145	stable	SEMA4C
0.004712096	stable	RABGGTA
0.004716613	stable	VASP
0.00471851	stable	PTGS2
0.004724695	stable	SNORD100
0.004724695	stable	MBD6
0.004736483	stable	KIAA1553
0.00474977	stable	ZNF165
0.004754382	stable	GGPS1
0.004780843	stable	MGC33556

0.004782278	stable	NRCAM
0.004782278	stable	XTP3TPA
0.004795041	stable	MTIF2
0.004797158	stable	IL1RAP
0.004798513	stable	ARHGEF11
0.004799391	stable	MAP1S
0.004828172	stable	PACSIN1
0.004841097	stable	CSE1L
0.004868084	stable	EVI2A
0.004885826	stable	ZNF772
0.00488961	stable	C12ORF5
0.004900574	stable	HOOK2
0.004923066	stable	RGS10
0.004923066	stable	LRRN4CL
0.004933784	stable	PLA2G5
0.004937396	stable	IQCC
0.004937396	stable	LOC134466
0.004937396	stable	PRPF18
0.004941385	stable	SPECC1L
0.004963988	stable	HEBP1
0.004965136	stable	OPLAH
0.004970644	stable	HIST1H1C
0.004983973	stable	BZW1
0.005000824	stable	ZNF91
0.005006932	stable	A2BP1
0.00501119	stable	BCAP31
0.005030466	stable	TRIP4
0.005030466	stable	LOC653717
0.005037934	stable	ABRA
0.00505404	stable	LOC731366
0.00505404	stable	SERINC1
0.005054205	stable	LOC648570
0.005064911	stable	CASK
0.005088592	stable	CHCHD4
0.005089359	stable	CUEDC1
0.005102159	stable	CAPNS1
0.005102159	stable	DYNLT1
0.005104243	stable	LOC653583
0.005120717	stable	SLC16A7
0.005123424	stable	C1ORF54
0.005124922	stable	TINP1
0.005132111	stable	SNTB2
0.00513457	stable	TSPAN3
0.00513457	stable	FKTN
0.005160793	stable	SUPT4H1
0.005161138	stable	UBQLN4
0.005165864	stable	FAT3
0.005175731	stable	SLC30A5
0.005181839	stable	GJC1
0.00518407	stable	WDR43
0.005191415	stable	ASPH

0.005197844	stable	PTH2
0.005197844	stable	APOL1
0.00522182	stable	LAG3
0.005260269	stable	C17ORF39
0.005261229	stable	CTDSPL2
0.005265299	stable	PHYHD1
0.005269726	stable	C1ORF53
0.005274105	stable	C4ORF42
0.005274105	stable	GPR180
0.005275455	stable	MYO1D
0.005275455	stable	CCDC14
0.005279425	stable	C20ORF7
0.005281917	stable	TAOK1
0.005303676	stable	LOC646574
0.005311048	stable	CDV3
0.005311587	stable	DACT2
0.005311587	stable	TSG101
0.005320241	stable	FAM83B
0.005346227	stable	TM2D1
0.005350793	stable	TM4SF1
0.005364926	stable	LOC652837
0.005365469	stable	PPM1J
0.00536554	stable	VSIG2
0.005366714	stable	LOC651143
0.00539305	stable	RNF5
0.005412887	stable	ETFB
0.005420062	stable	C14ORF108
0.005424543	stable	ACACB
0.005429056	stable	STOML1
0.005434794	stable	ASCC3
0.005441281	stable	CD320
0.005458529	stable	SLC19A2
0.005458529	stable	SNORD56
0.005461849	stable	NCOA3
0.00546196	stable	VEGFB
0.00546539	stable	ADH1B
0.005481013	stable	RARS2
0.005501809	stable	GLTPD1
0.00550637	stable	ZNF767
0.005506793	stable	HIST1H2BK
0.005518295	stable	EGF
0.00552262	stable	PARD3
0.005537999	stable	C9ORF80
0.005540556	stable	FOXD2
0.005546264	stable	PRRG1
0.005546973	stable	SIGLECP3
0.005555961	stable	ITLN1
0.00557193	stable	PDE1B
0.005576271	stable	FAM109B
0.005589849	stable	FCHO2
0.005612124	stable	LOC727849

0.005627464	stable	ZNF658
0.005636586	stable	LOC644544
0.00563696	stable	DKFZP586H2123
0.005662705	stable	BEAN
0.005663094	stable	ACTR8
0.005667752	stable	MRPL44
0.005668221	stable	C8ORF33
0.005672302	stable	DNAJB9
0.005693365	stable	ISG20L2
0.00570686	stable	MT1H
0.00570686	stable	SH2B2
0.005711666	stable	TAF4
0.005712665	stable	LOC130773
0.00571648	stable	MED10
0.005717154	stable	TRAFD1
0.005732462	stable	SNORD35B
0.005737589	stable	OLFM4
0.005745508	stable	RABEP1
0.005746148	stable	APOO
0.005746148	stable	FXYD2
0.005746733	stable	C1ORF19
0.005764991	stable	KIAA1542
0.005774471	stable	ANGPTL2
0.005778287	stable	EFNB2
0.005797308	stable	MS4A2
0.005816864	stable	HLA-DPA1
0.005821867	stable	ZNF362
0.00582365	stable	DLL1
0.00582667	stable	MCM5
0.005829682	stable	LOC653489
0.005830215	stable	LOC401317
0.005837139	stable	CYB5D1
0.005845833	stable	GAS2L1
0.00585387	stable	LOC651064
0.005864521	stable	ZNF397
0.005888621	stable	COPS6
0.00589668	stable	CDRT1
0.00589996	stable	LOC642947
0.005902988	stable	GLT8D3
0.005920872	stable	C3ORF59
0.00592393	stable	INDO
0.00592393	stable	C19ORF43
0.00593221	stable	PCGF5
0.005935295	stable	FIG4
0.005943601	stable	QTRTD1
0.005946301	stable	CTDP1
0.005957268	stable	COL13A1
0.00596517	stable	DYNLRB1
0.005965716	stable	LOC648059
0.005974675	stable	ZNF431
0.005981312	stable	JRKL

0.005981312	stable	ZNF747
0.00601011	stable	CHRNA3
0.006019874	stable	LOC286297
0.006020798	stable	ZFYVE1
0.006022785	stable	C17ORF71
0.00603942	stable	MTRF1
0.006041527	stable	LOC645888
0.006050858	stable	SMAD7
0.006056208	stable	ST6GALNAC6
0.006061349	stable	HIST1H3D
0.006072547	stable	REC8
0.006078196	stable	CCDC117
0.006091257	stable	LOC651790
0.006094405	stable	GSTT2
0.006097495	stable	FBXW7
0.006099027	stable	AYP1P1
0.006101667	stable	PTGDR
0.00611114	stable	LCN1
0.006117975	stable	ARHGEF1
0.006119138	stable	IDH1
0.006125622	stable	WHSC2
0.006151102	stable	MXD4
0.00615771	stable	LOC441196
0.006164856	stable	KIAA0355
0.00618373	stable	YIPF6
0.00618373	stable	CLTB
0.006213175	stable	MRPL4
0.006218696	stable	SOD1
0.006226037	stable	DEFA1
0.006228047	stable	MAX
0.006229841	stable	BRWD1
0.006231385	stable	MYRIP
0.006238344	stable	SH2B3
0.006240182	stable	PODXL2
0.006252754	stable	RNF135
0.006252754	stable	C11ORF42
0.006254954	stable	IFRD2
0.006254954	stable	COQ7
0.006259228	stable	SLC25A15
0.006279771	stable	HYPK
0.006279771	stable	WDR68
0.006292247	stable	MCL1
0.006309396	stable	FLYWCH1
0.00631551	stable	LHFP
0.006325367	stable	LTC4S
0.006325367	stable	GLTP
0.006337777	stable	ARMC10
0.006339886	stable	SIGLEC9
0.00634064	stable	SEC23IP
0.006351336	stable	TMEM87A
0.006385824	stable	C4ORF36

0.006392099	stable	SCARF2
0.006402817	stable	CREB3L2
0.006413843	stable	LOC441775
0.006427636	stable	DCBLD1
0.006430219	stable	SOHLH2
0.006439305	stable	RAMP2
0.006442001	stable	MGAT4B
0.006452112	stable	SEMA3E
0.006467894	stable	PBLD
0.006491598	stable	SLC25A14
0.006495728	stable	CYP26B1
0.00650879	stable	PPP1R3F
0.006509458	stable	SAA1
0.006517848	stable	LOC652672
0.006527809	stable	PRKD1
0.006534063	stable	C8ORFK36
0.006547404	stable	UQCC
0.006552174	stable	SIGLEC1
0.006583053	stable	C19ORF28
0.006585883	stable	RAB34
0.006587444	stable	SCARA3
0.006633311	stable	CKM
0.006633311	stable	RNF215
0.006633311	stable	ZNF823
0.006640577	stable	FAM101B
0.006647467	stable	HIST1H3H
0.006648197	stable	LOC645098
0.006648197	stable	SUPT6H
0.006648197	stable	ZNF283
0.006648197	stable	ERH
0.006650136	stable	IFT57
0.0066563	stable	RBM3
0.006656656	stable	RPP21
0.006658625	stable	EEF1G
0.006676064	stable	FEM1B
0.006680078	stable	NLRC5
0.006681968	stable	COG4
0.006702437	stable	LOC653650
0.006704975	stable	VIPR1
0.006714888	stable	KIAA0999
0.00672393	stable	SLC19A1
0.006742919	stable	CREBZF
0.006758604	stable	FLJ45248
0.006767189	stable	STARD3
0.006778634	stable	ZNF443
0.006802708	stable	NY-SAR-48
0.006835728	stable	C15ORF38
0.006837367	stable	ZBTB3
0.006844057	stable	CCDC74A
0.006845878	stable	TM9SF2
0.006864276	stable	C21ORF24

0.006872127	stable	PILRB
0.006874438	stable	CNTNAP3B
0.006883336	stable	VENTX
0.006899882	stable	CAD
0.006911776	stable	SIX5
0.006911776	stable	PLAT
0.006917633	stable	C12ORF60
0.006921707	stable	ZNF134
0.006933788	stable	FMNL2
0.006960849	stable	SEPT2
0.006997602	stable	PURB
0.007002367	stable	BCR
0.007028284	stable	NFIA
0.007052142	stable	TBC1D5
0.007068482	stable	SFRP5
0.007068482	stable	BRCA1
0.007068482	stable	LRRC48
0.007069294	stable	C9ORF66
0.007077863	stable	ACP6
0.007082404	stable	LOC148137
0.007088271	stable	LOC730994
0.007090871	stable	NUDT13
0.007113481	stable	TCP11L1
0.007122207	stable	OR4D1
0.007122207	stable	POP7
0.007140686	stable	NOD1
0.007143752	stable	LOC197350
0.007159301	stable	PKN3
0.007164386	stable	FREQ
0.007197811	stable	BAT3
0.007217347	stable	PEPD
0.007219154	stable	NEIL2
0.007232593	stable	TMEM132A
0.007235682	stable	PAFAH1B2
0.007242316	stable	OGG1
0.007242799	stable	TNFAIP8
0.007267207	stable	ATP13A5
0.007282506	stable	C17ORF69
0.007297561	stable	GPR65
0.007305444	stable	GPR84
0.007308378	stable	LOC643287
0.007314906	stable	LHX2
0.007325582	stable	FAM26F
0.007325582	stable	KL
0.007327772	stable	TPRKB
0.007333063	stable	LOC729196
0.007333063	stable	TBCB
0.007333063	stable	MGC3032
0.00733889	stable	HHAT
0.007363204	stable	AIFM1
0.00736897	stable	HGD

0.007385062	stable	MR1
0.007397855	stable	PSMG2
0.007401576	stable	SLC38A1
0.007418845	stable	RAB9B
0.007423775	stable	WNT5B
0.007428208	stable	LOC205251
0.007429466	stable	LOC375190
0.007437561	stable	LOC401623
0.007438694	stable	TM9SF3
0.007438694	stable	PDK2
0.007446122	stable	FAM8A1
0.007449033	stable	RETN
0.007450259	stable	ZNF628
0.007450259	stable	FAM113A
0.007463115	stable	IQCA1
0.007474184	stable	LPXN
0.007486579	stable	CXXC6
0.007486579	stable	GAL3ST3
0.007490437	stable	CIB1
0.007493868	stable	C14ORF142
0.007499857	stable	SIRPA
0.007499857	stable	EARS2
0.007505691	stable	CCL26
0.00753947	stable	KLHDC5
0.0075618	stable	ZNF652
0.007581401	stable	MGC16703
0.007583825	stable	ZNF367
0.007606798	stable	GSK3A
0.007623833	stable	CCS
0.007630888	stable	VWA1
0.007650305	stable	PRPF4B
0.007683986	stable	C2ORF24
0.007684543	stable	RAB1A
0.007712315	stable	C12ORF43
0.007730607	stable	ZDHHC17
0.007734211	stable	TP53BP2
0.007734767	stable	LOC727726
0.007743347	stable	P2RXL1
0.007743347	stable	MRS2
0.007759214	stable	RPL27
0.007771397	stable	GCHFR
0.007772485	stable	THBD
0.007773111	stable	DJ222E13.2
0.007781868	stable	NGFR
0.007798627	stable	HSD17B8
0.007799586	stable	CHD5
0.007804602	stable	HOXA4
0.007822699	stable	C20ORF29
0.007839691	stable	SLC23A2
0.007844337	stable	LHCGR
0.007848378	stable	PID1

0.007848741	stable	MALT1
0.007851893	stable	KLHDC1
0.007852859	stable	KRTAP20-2
0.007856968	stable	MIER1
0.007866361	stable	FAM71E1
0.007866361	stable	CHMP2B
0.007880951	stable	AMACR
0.007905072	stable	DUS1L
0.007908025	stable	PHTF1
0.007937234	stable	ADAM23
0.00795034	stable	USF2
0.007978545	stable	ARL5A
0.007978545	stable	LOC441246
0.007979602	stable	IL1RL2
0.007986146	stable	PLXNA1
0.008031193	stable	PLD2
0.008031193	stable	LOC653453
0.00806675	stable	CENPO
0.008068844	stable	LOC652844
0.008073141	stable	KCNS2
0.008076108	stable	ZNF436
0.008090963	stable	ITPA
0.008101324	stable	GABBR1
0.008102641	stable	ODZ3
0.008105682	stable	CA13
0.008131557	stable	LOC653513
0.008161461	stable	POL3S
0.008170963	stable	CCDC49
0.008175547	stable	C1ORF77
0.008197032	stable	PRAF2
0.008216071	stable	MND1
0.008242845	stable	C5ORF25
0.008248641	stable	LOC643224
0.008273293	stable	DFNB59
0.008280882	stable	WWP2
0.008280882	stable	NLRP8
0.008284092	stable	LANCL2
0.008300343	stable	SYNGAP1
0.008300343	stable	HSN2
0.008305883	stable	FLJ90757
0.008309041	stable	PPP2R3B
0.008323438	stable	CCDC81
0.008334925	stable	CASP9
0.008344342	stable	MBD3
0.008346861	stable	ATP10D
0.008356629	stable	PLAA
0.008358866	stable	NDN
0.008366409	stable	C3ORF17
0.008372936	stable	TXNDC16
0.00837841	stable	MOSC1
0.008397501	stable	KCNA7

0.008409241	stable	GLB1
0.008419559	stable	DIP2A
0.008455394	stable	MAK10
0.008460971	stable	IHPK1
0.008468584	stable	TTC19
0.008470443	stable	GPX3
0.008474927	stable	NDUFA9
0.008486865	stable	XK
0.008486865	stable	SEPHS1
0.008486865	stable	LOC401093
0.008486865	stable	STRN
0.008494378	stable	ZNF599
0.00850588	stable	CDC42
0.008509326	stable	C9ORF78
0.00851111	stable	PPP1R15A
0.008517535	stable	FZD6
0.008570341	stable	MARCH2
0.00860117	stable	WDR47
0.00860117	stable	GUSBL2
0.008601189	stable	RPS19BP1
0.008622196	stable	DNAJC25
0.008625811	stable	AKIRIN1
0.008646436	stable	C9ORF150
0.008646436	stable	LOC344405
0.008649966	stable	PRMT7
0.008675979	stable	CIAO1
0.008689028	stable	GARNL4
0.008706984	stable	C2ORF42
0.008707739	stable	PCBP1
0.008728852	stable	XRN1
0.008733248	stable	SARM1
0.008771833	stable	TTC27
0.008776156	stable	MAL
0.008828002	stable	KIAA1324
0.008835935	stable	PTPRO
0.008838585	stable	NARF
0.008852023	stable	SF3B14
0.008863581	stable	CHD1
0.008867467	stable	PRKAG1
0.008873676	stable	RIMS3
0.008891682	stable	C8ORF37
0.008891682	stable	LOC554206
0.008896594	stable	SLC39A10
0.008901827	stable	C8ORF45
0.008906873	stable	SPIN4
0.008908562	stable	UROS
0.008915429	stable	FHDC1
0.008926406	stable	ARHGAP23
0.008951943	stable	C19ORF23
0.009002157	stable	PCDHB2
0.009035155	stable	PHF17

0.009048083	stable	GALIG
0.009083474	stable	RNF141
0.009109064	stable	NICN1
0.009111323	stable	MYH4
0.009140912	stable	TMEM209
0.009156309	stable	ACTN4
0.00918602	stable	FVT1
0.009195314	stable	IL4I1
0.009198506	stable	LOC646195
0.009198506	stable	CAMK1
0.009216645	stable	PON2
0.009223283	stable	KRTAP10-12
0.009243065	stable	APOL6
0.009249709	stable	KCNIP1
0.009249709	stable	DHX36
0.009265729	stable	LOC652388
0.009268537	stable	MOSC2
0.009292232	stable	MRPS25
0.009292232	stable	ITIH4
0.009301977	stable	CLDN9
0.009310753	stable	PGBD2
0.009326358	stable	MYO3B
0.009328592	stable	DPM2
0.009333859	stable	XRCC4
0.009357895	stable	C14ORF80
0.009359557	stable	GNAI3
0.009374031	stable	SPTLC1
0.009378623	stable	MGC72080
0.009398857	stable	LOC728358
0.009439007	stable	RNF122
0.009439007	stable	SP140
0.009450158	stable	SPATA20
0.009452629	stable	SELI
0.009458285	stable	GNAT1
0.009478571	stable	SPHK2
0.0094811	stable	DJ341D10.1
0.009483014	stable	JMY
0.009490161	stable	MAL2
0.009503516	stable	GPI
0.009503516	stable	ZDHHC12
0.009503516	stable	ANGPT2
0.009511642	stable	MTMR3
0.009563711	stable	VBP1
0.009601764	stable	SIAH1
0.009654814	stable	RPL18
0.009665505	stable	AADACL1
0.009697993	stable	IKBKB
0.009714664	stable	ZW10
0.009726322	stable	C14ORF32
0.009739433	stable	TTC25
0.009763635	stable	XRCC6BP1

0.009765626	stable	RNASE3
0.009769057	stable	ISYNA1
0.009792065	stable	GLT8D1
0.009797322	stable	RAB11A
0.009798186	stable	HIPK1
0.009829045	stable	ZNF720
0.009836382	stable	UNC5B
0.009836382	stable	RUSC1
0.009861108	stable	BRSK1
0.009862663	stable	TGFB2
0.009866469	stable	TBC1D20
0.009872116	stable	LOC730820
0.009879058	stable	CHM
0.009879413	stable	TYRO3
0.009879413	stable	LGI2
0.009884022	stable	ORAI1
0.009954421	stable	NQO2
0.009965258	stable	CDH15
0.01002941	stable	ZNF276
0.010056752	stable	KIAA1641
0.010060993	stable	APOBEC3C
0.010088076	stable	GBP1
0.010111446	stable	DPH3
0.010122586	stable	LOC441061
0.010127412	stable	MRPL21
0.010162817	stable	FLJ36644
0.010238764	stable	NFKB1
0.010238864	stable	RMND5B
0.010282471	stable	TCEB2
0.01029308	stable	AP3B1
0.010365058	stable	DOK3
0.010370984	stable	TRIM3
0.010374352	stable	TRIM61
0.010401945	stable	SMC2
0.010403906	stable	ZNF394
0.010403906	stable	PAPOLA
0.010462937	stable	LOC731007
0.010479397	stable	BAG4
0.010495285	stable	LOC643396
0.010509928	stable	LOC643872
0.010540367	stable	LOC645688
0.010540937	stable	LRP4
0.010546765	stable	H2AFY2
0.010565415	stable	FRK
0.010578187	stable	PRICKLE4
0.010605163	stable	LOC647195
0.010609832	stable	LOC146517
0.010616635	stable	LOC158301
0.010616635	stable	PBXIP1
0.010616635	stable	SLC25A45
0.010616635	stable	SEC11A

0.010655717	stable	TMEM33
0.010674393	stable	TDRD1
0.01067715	stable	ACTR10
0.010678783	stable	EXOSC4
0.010695865	stable	RTCD1
0.010696898	stable	TTC30B
0.010696898	stable	FZD3
0.010700782	stable	LOC731002
0.010706797	stable	ZNF589
0.010709269	stable	ELOVL1
0.010750478	stable	MRPL19
0.010764085	stable	GARNL1
0.01077428	stable	C20ORF52
0.010817127	stable	LOC646276
0.010818768	stable	GNG7
0.010833683	stable	ITK
0.010846252	stable	USP14
0.010904793	stable	C1ORF144
0.010937857	stable	POLR2B
0.010986024	stable	TSFM
0.010991725	stable	CYFIP2
0.010991725	stable	FLJ14107
0.010997031	stable	FAM119B
0.010997031	stable	FADS2
0.011008583	stable	LOC731835
0.011036463	stable	FAM49B
0.011037639	stable	LOC650831
0.011063287	stable	PLS1
0.011078114	stable	ZNF558
0.011078114	stable	DPY19L3
0.011082045	stable	NONO
0.011083749	stable	CYC1
0.01109382	stable	LOC649801
0.011098669	stable	FRS3
0.011098669	stable	LOC652881
0.011101087	stable	DKC1
0.011112135	stable	LOC732371
0.011112135	stable	GNA12
0.01112755	stable	CORO2A
0.011163808	stable	OCIAD1
0.011178934	stable	DENR
0.011214656	stable	ZNF217
0.011236552	stable	SPRED1
0.011249575	stable	KIAA0753
0.011256766	stable	SBDSP
0.011260654	stable	MGC39584
0.011309447	stable	SPAG1
0.011346944	stable	LPAR1
0.011349153	stable	KIAA1671
0.011353182	stable	CSAD
0.011372329	stable	ZEB2

0.011383915	stable	SCNN1D
0.011392812	stable	LARS2
0.011397425	stable	CD84
0.011412019	stable	LOC387762
0.011417005	stable	UQCRC2
0.011425331	stable	HOXB5
0.011429712	stable	TMED4
0.011439376	stable	FAM98A
0.011445837	stable	DDX11
0.01144899	stable	APM-1
0.011453816	stable	ELOVL2
0.011453816	stable	TEX264
0.011486464	stable	PREP
0.011494234	stable	GPD1
0.01149689	stable	PABPC1
0.01150703	stable	DAPK1
0.01151186	stable	PMS2L3
0.011552552	stable	GK
0.011564209	stable	ATAD3B
0.01157522	stable	PIGH
0.011644752	stable	CHL1
0.011645101	stable	MDH1
0.011645101	stable	SSH2
0.011645101	stable	LOC729759
0.011649001	stable	SIGLEC7
0.011718296	stable	ZFAND2A
0.011718296	stable	SDHA
0.011727649	stable	ZNF596
0.011728658	stable	RHOD
0.011730348	stable	SCGB1D2
0.011799687	stable	LOC642859
0.011821126	stable	RNF144
0.011826117	stable	PEX11G
0.011833235	stable	IKZF5
0.011843113	stable	DYM
0.011852556	stable	PPM1B
0.011899223	stable	LOC730051
0.011939726	stable	CREB3L4
0.011953606	stable	HDHD2
0.011980368	stable	TUG1
0.011980756	stable	TNFSF8
0.011985703	stable	FAM10A4
0.012000275	stable	CNTN4
0.012014264	stable	FAM168A
0.012060231	stable	HPS5
0.012060231	stable	INMT
0.012087394	stable	TCF7L2
0.012099771	stable	C18ORF26
0.012134893	stable	LOC728888
0.012150995	stable	ABCG5
0.012150995	stable	GLI3

0.012152117	stable	LOC152195
0.012184853	stable	KCNQ3
0.012189893	stable	CCDC47
0.012203733	stable	HIVEP2
0.012203733	stable	CALN1
0.012204555	stable	BTNL2
0.012214622	stable	ZNFX1
0.012262705	stable	NAV1
0.012267657	stable	C10ORF41
0.012272286	stable	SENP2
0.012282137	stable	XYLT1
0.01229321	stable	COL4A3
0.012297838	stable	SLC1A4
0.012338228	stable	CACNA2D1
0.012345641	stable	HDHD1A
0.012368006	stable	SPIN2B
0.012375389	stable	C1ORF58
0.012433377	stable	MIB1
0.012437855	stable	GIYD1
0.012469756	stable	PUM2
0.01247908	stable	COMMD9
0.012483024	stable	ALS2CR12
0.012558384	stable	LOC440731
0.012558384	stable	LOC493754
0.012567059	stable	PHF11
0.012570987	stable	WTAP
0.012589217	stable	ECHS1
0.012617786	stable	ECHDC1
0.012617786	stable	ABCA1
0.012620641	stable	SLC26A6
0.012621095	stable	RHAG
0.012644346	stable	UGP2
0.012648342	stable	MGC4677
0.012648359	stable	TIE1
0.012656728	stable	MAP1LC3A
0.012674888	stable	ELP3
0.012693285	stable	LOC644137
0.012765916	stable	ARID1A
0.012782154	stable	KIAA1715
0.012789077	stable	RABGEF1
0.012831542	stable	LOC441191
0.012831542	stable	AIFM2
0.012852622	stable	TNNC1
0.01285704	stable	CCDC23
0.012857641	stable	DISC1
0.012865494	stable	ZNF593
0.012869251	stable	NRK
0.012874684	stable	ACTG2
0.012908366	stable	MEOX1
0.012915947	stable	WRNIP1
0.012947747	stable	POT1

0.012957346	stable	COL22A1
0.012959565	stable	CDCA7L
0.012976656	stable	RNPS1
0.01299891	stable	LOC732075
0.013076269	stable	FAM21C
0.013101888	stable	TNS1
0.013113585	stable	CCDC48
0.013134133	stable	CXORF34
0.013154124	stable	LOC728723
0.013188995	stable	GPR68
0.013204665	stable	CDKN1C
0.013216671	stable	RABAC1
0.013321187	stable	VAMP3
0.013330087	stable	BUD31
0.013355831	stable	RSAD1
0.013368892	stable	GPKOW
0.013368892	stable	MT1JP
0.013384381	stable	FLJ30092
0.01343735	stable	LRRC6
0.01343735	stable	ZNF57
0.01343735	stable	LOC645241
0.013526582	stable	DNAJC9
0.013567985	stable	HCN2
0.013579967	stable	ERBB2IP
0.013579967	stable	KIAA1333
0.013582117	stable	ANGPTL5
0.013583684	stable	RPL36A
0.013583684	stable	AFG3L1
0.013591385	stable	SEPT7
0.013632613	stable	NHLRC2
0.01365742	stable	C3ORF26
0.013659438	stable	CABLES2
0.013668052	stable	RNF19A
0.013687499	stable	PHKG2
0.01369167	stable	CRYAB
0.013695209	stable	KIF17
0.01370149	stable	RAVER1
0.013702973	stable	LOC652242
0.01370447	stable	CCR7
0.013738118	stable	SCAMP5
0.013742912	stable	C13ORF7
0.01374651	stable	SYN2
0.013760814	stable	LOC646588
0.013796553	stable	TMEM14A
0.013800508	stable	SEC16A
0.013800508	stable	IQCK
0.013800508	stable	MYST1
0.013808883	stable	MFSD2
0.013831006	stable	ITGA7
0.013861161	stable	ZC3H4
0.013878511	stable	ZBTB38

0.013889055	stable	SPAG8
0.013965871	stable	PKD1L1
0.013990903	stable	TOX4
0.014029146	stable	DTNBP1
0.014031282	stable	RUNX2
0.014031282	stable	CDC42SE2
0.014031282	stable	CYTL1
0.014043655	stable	CTH
0.014064149	stable	STK39
0.014100404	stable	CD58
0.014104697	stable	SLC16A6
0.014159078	stable	MICALL1
0.014159078	stable	NARG1
0.014170988	stable	TSLP
0.014215409	stable	FMO2
0.014272406	stable	ATL2
0.014284784	stable	SLC9A5
0.014370525	stable	PTGIS
0.014382099	stable	B3GALT4
0.0143859	stable	LOC642443
0.014422043	stable	TST
0.014430719	stable	DGUOK
0.014439351	stable	C6ORF142
0.014457949	stable	KIAA1881
0.014466905	stable	PTP4A1
0.014472028	stable	FARSA
0.014478185	stable	CSGALNACT2
0.01450441	stable	MGC2752
0.014518109	stable	SMOX
0.014559461	stable	FGFR4
0.014559461	stable	SCRG1
0.014581708	stable	LOC647450
0.014588887	stable	PLEKHO2
0.014658819	stable	LOC642393
0.014676005	stable	NEUROG2
0.014712379	stable	EXTL2
0.014718949	stable	PCTK3
0.014768919	stable	LOC644511
0.014770787	stable	LOC651285
0.014800274	stable	PPTC7
0.014847424	stable	KPNB1
0.014852802	stable	LOC401218
0.014874055	stable	LOC647677
0.014933896	stable	KIAA0652
0.014949524	stable	LCP2
0.014954587	stable	XG
0.014971562	stable	RMND5A
0.014986471	stable	ATXN2
0.015129111	stable	DPP4
0.015161787	stable	NR3C1
0.015235704	stable	LOC642423

0.015239869	stable	C16ORF13
0.015251086	stable	DNAJC2
0.01525779	stable	LOC641700
0.015281885	stable	CDAN1
0.015312672	stable	BLZF1
0.015346169	stable	TBX1
0.015391632	stable	MAPKAP1
0.015415832	stable	NR4A1
0.015437443	stable	ANKRD44
0.015447353	stable	C21ORF81
0.015458448	stable	SYTL4
0.015462334	stable	GNS
0.015462379	stable	RBKS
0.015477844	stable	CRMP1
0.015513267	stable	STARD5
0.01552852	stable	FLT1
0.015601175	stable	FLJ20699
0.015601175	stable	USP25
0.015601175	stable	LAMC3
0.015603166	stable	HSPB3
0.015660174	stable	ZNF614
0.015680899	stable	SELK
0.015715666	stable	CYP4V2
0.015715807	stable	NRGN
0.0157574	stable	ACBD3
0.015785509	stable	LOC644670
0.01581432	stable	PRKAR1A
0.015867459	stable	DEDD
0.015931277	stable	TBP
0.015931277	stable	RG9MTD3
0.015946681	stable	ZC3H7A
0.015950598	stable	LOC650128
0.015950598	stable	LOC646609
0.015954422	stable	ADPRHL2
0.016015492	stable	C9
0.01601673	stable	ZWILCH
0.016033411	stable	C20ORF72
0.016033411	stable	TSPAN5
0.016033514	stable	RUNDC1
0.016104879	stable	RAB37
0.016110573	stable	JMJD2A
0.016110573	stable	CNTROB
0.016110573	stable	HPDL
0.016150842	stable	MGC3731
0.016155214	stable	FLJ44112
0.016193531	stable	KIAA0746
0.01622028	stable	RFWD3
0.016241763	stable	PPARA
0.016251694	stable	TRIM25
0.016252423	stable	SLC22A15
0.016253589	stable	FAM110A

0.016256735	stable	WRN
0.01626041	stable	DPYSL3
0.016261466	stable	LOC651337
0.016294857	stable	NADSYN1
0.016295478	stable	GPATCH2
0.016298442	stable	CENPQ
0.016336918	stable	CST6
0.016343635	stable	PLEKHM2
0.016343635	stable	HNRPK
0.016343635	stable	NUDT4P1
0.016361561	stable	GORASP1
0.016363176	stable	TMED2
0.016374223	stable	RNF111
0.016384322	stable	NCOA5
0.016384322	stable	USP16
0.016420742	stable	FGF13
0.016434457	stable	STX7
0.016434457	stable	NUDT12
0.016434816	stable	MAP7D3
0.0164405	stable	SUMO1P3
0.0164405	stable	BAPX1
0.0164405	stable	C1ORF156
0.016441211	stable	CTAGE5
0.016441547	stable	PSMA5
0.016444278	stable	NAGLU
0.01645837	stable	CRYGS
0.016464191	stable	C6ORF89
0.016477187	stable	TMPRSS13
0.016520368	stable	SERPINF2
0.016520368	stable	ACAT2
0.016521684	stable	NEU4
0.016590467	stable	CROCC
0.016634326	stable	C5ORF28
0.016642472	stable	DNASE1L3
0.016673461	stable	LOC651872
0.016675026	stable	TFEB
0.016676115	stable	ALDOC
0.016700155	stable	C13ORF25
0.016711585	stable	NOC4L
0.016734975	stable	FOXO1
0.016762935	stable	CSDA
0.016766334	stable	LOC653438
0.016768915	stable	LOC643310
0.016779776	stable	LOC730272
0.016790542	stable	ARRDC4
0.016858906	stable	TRAPPC6A
0.016877515	stable	DEFB122
0.016970492	stable	ARFGEF1
0.016970492	stable	FAM104A
0.017000614	stable	OSBPL1A
0.017016181	stable	MFSD8

0.017016181	stable	TNRC6B
0.017037205	stable	CAPG
0.017055867	stable	LOC283050
0.017064255	stable	DGKI
0.017075388	stable	C20ORF59
0.017075388	stable	HDAC8
0.017084949	stable	LOC648852
0.017121643	stable	LOC153364
0.017134921	stable	RFX2
0.017146234	stable	PLIN
0.017146234	stable	CYB5R2
0.017147067	stable	OSBP
0.017154746	stable	LOC388654
0.017164736	stable	KCNG1
0.017164736	stable	LRRC40
0.017169311	stable	CCL7
0.017170396	stable	OPHN1
0.017171911	stable	PRPS1
0.017203	stable	LMO4
0.017209889	stable	LOXL3
0.017230242	stable	ROCK2
0.017254128	stable	ZNF250
0.017257612	stable	NGFRAP1
0.017287259	stable	LOC645013
0.017300091	stable	ERP29
0.017313589	stable	EEF1D
0.017320872	stable	ING2
0.017361623	stable	HEMK1
0.017370076	stable	DMD
0.017391072	stable	EPB41L1
0.017395884	stable	CSNK1A1
0.017410529	stable	UFD1L
0.017418305	stable	NFX1
0.017420802	stable	LOC441763
0.01749621	stable	LOC642477
0.01749621	stable	TOP3B
0.017508218	stable	RPS21
0.017508218	stable	LOC652489
0.017630029	stable	GRIK2
0.017630029	stable	PPP1R12A
0.017644054	stable	C4ORF34
0.017650014	stable	C22ORF28
0.017653024	stable	FLJ10996
0.017664447	stable	ASB10
0.017664447	stable	SAP130
0.017670039	stable	AFAP1L2
0.017699888	stable	RRP7A
0.017703569	stable	PRRT1
0.017709163	stable	DNAJC28
0.0177248	stable	TM2D2
0.017763383	stable	PPP1R15B

0.017763719	stable	PDCD2
0.017763719	stable	LOC649509
0.017773519	stable	ARMC1
0.017799148	stable	RMND1
0.017841344	stable	OLFML1
0.017854403	stable	TMTC4
0.017877195	stable	CSN1S2A
0.017883606	stable	SMPX
0.017898904	stable	C6ORF72
0.017914897	stable	CDC34
0.017921211	stable	ZNF768
0.017935648	stable	ACTR2
0.017938841	stable	MRPL34
0.01796992	stable	CXORF26
0.017987424	stable	IPPK
0.017991739	stable	RELL2
0.018003209	stable	UBE4A
0.018004382	stable	APTX
0.018004382	stable	FAM116A
0.018021124	stable	AKR1B10
0.018043427	stable	LMTK3
0.018050724	stable	LOC646109
0.018109035	stable	MON2
0.018123425	stable	VTI1B
0.018123425	stable	DPYD
0.018130253	stable	TAPT1
0.018161916	stable	WDR77
0.018169553	stable	SNORD38A
0.018223704	stable	RNASEH2A
0.018250079	stable	FIGN
0.018260321	stable	RPS27
0.018260321	stable	KCNMB1
0.018286716	stable	KIF3C
0.018295108	stable	FBXO6
0.018372689	stable	EPM2AIP1
0.018442006	stable	AOF2
0.018513807	stable	CLEC12A
0.018564885	stable	ADAM22
0.018625468	stable	EIF4B
0.01863518	stable	HNRNPD
0.018683732	stable	TRAF3IP1
0.018699901	stable	CD82
0.01870432	stable	CLCNKB
0.018748255	stable	LOC652264
0.018748255	stable	NSFL1C
0.018751213	stable	ATP13A2
0.018783093	stable	ZNF501
0.018826983	stable	SSX4B
0.018878517	stable	ITM2C
0.01890285	stable	LOC730358
0.019006796	stable	UBQLN2

0.019017347	stable	IFT74
0.019037373	stable	HNRNPH2
0.019072928	stable	C7ORF47
0.01907456	stable	LOC642816
0.019082343	stable	LOC650037
0.019111765	stable	KLHL2
0.019140451	stable	RBM14
0.019140451	stable	C8ORF32
0.019144707	stable	TAF1B
0.019160719	stable	KATNAL1
0.019162197	stable	TF
0.019185996	stable	NES
0.019217168	stable	HBZ
0.019223379	stable	BOC
0.019232449	stable	SLC25A17
0.019289972	stable	CSPG4LYP1
0.019324783	stable	OBFC1
0.019347043	stable	CLIC4
0.01938478	stable	RAD54B
0.019421945	stable	ELMOD2
0.019432336	stable	RNF5P1
0.019437815	stable	COL5A1
0.019530252	stable	RNF19B
0.019607109	stable	PSMC3
0.019615815	stable	GOLGB1
0.019621288	stable	C11ORF63
0.019640755	stable	TMEM194
0.019710184	stable	C1ORF96
0.019807944	stable	AKR1B1
0.019829696	stable	ANKRD24
0.019917982	stable	PDE4C
0.019963904	stable	P4HA1
0.019992697	stable	WBSCR17
0.020012146	stable	SEC22A
0.020023498	stable	CHX10
0.020043023	stable	LOC644029
0.020061467	stable	PGAM5
0.020061467	stable	SNORD43
0.020078545	stable	IMMP2L
0.020089497	stable	MCF2L2
0.020093303	stable	GAMT
0.0201873	stable	MYH3
0.0201873	stable	PGM3
0.0201873	stable	WDR18
0.02022615	stable	C3ORF55
0.020230834	stable	LOC388002
0.020248094	stable	TXNRD1
0.020252779	stable	C9ORF3
0.020258215	stable	N-PAC
0.020264893	stable	FLJ45337
0.0203291	stable	DAB2IP

0.020337754	stable	ZNF479
0.020350485	stable	LDHD
0.02035737	stable	POMP
0.020382329	stable	TMEM143
0.020409996	stable	LRRC58
0.020421696	stable	OR1M1
0.02042588	stable	HOXA5
0.020444625	stable	YIPF7
0.020449642	stable	RELA
0.020460269	stable	PTPN2
0.020542516	stable	RASA1
0.020545154	stable	ARHGAP21
0.020546074	stable	FAM116B
0.020632082	stable	LOC407835
0.020632082	stable	LOC653119
0.020680827	down	C6
0.020741704	stable	ENTPD4
0.0207797	stable	RFESD
0.0207797	stable	CHIC1
0.020789271	stable	TAS2R9
0.020803814	stable	PLEKHA1
0.020807613	stable	DZIP3
0.020830003	stable	SNRPE
0.02083385	stable	PITRM1
0.02083385	stable	PALM2-AKAP2
0.020836949	stable	USP7
0.020844709	stable	CCDC109A
0.020855671	stable	HSBP1
0.020857555	stable	TNCRNA
0.020932886	stable	CLOCK
0.0209365	stable	C17ORF81
0.0209365	stable	ALDH3A2
0.0209365	stable	SLC2A9
0.0209365	stable	UNC13B
0.020943319	stable	SLC35B1
0.020947331	stable	LOC387841
0.021021844	stable	NOC2L
0.021023329	stable	SNF1LK2
0.021047486	stable	CD48
0.021081131	stable	IGF2BP3
0.021081131	stable	TTC38
0.021084436	stable	DUSP18
0.021086473	stable	NFATC1
0.021092941	stable	FANCI
0.021122712	stable	HOXB4
0.021122712	stable	PXDN
0.021139494	stable	ZNF213
0.021151752	stable	FBXW2
0.02115309	stable	GPR126
0.021158185	stable	SP2
0.021158185	stable	NF2

0.021193985	stable	HLA-A
0.021207886	stable	LOC285033
0.021294736	stable	C21ORF123
0.021316481	stable	C16ORF68
0.021379068	stable	GIMAP6
0.021400502	stable	TOM1L2
0.021424999	stable	GATA5
0.021435056	stable	KCNT1
0.021439454	stable	LOC653291
0.021488321	stable	LOC729446
0.021506799	stable	LOC650251
0.021516425	stable	LOC285216
0.021523878	stable	LYL1
0.021568008	stable	MRPS12
0.021568008	stable	TIAM1
0.021578143	stable	KCNF1
0.021594161	stable	EXT2
0.021642137	stable	SEC61A2
0.021692091	stable	RTF1
0.021714421	stable	NME2
0.021722925	stable	RNF7
0.021777139	stable	SPIRE2
0.021782017	stable	LOC653907
0.021825196	stable	DEFB107A
0.021848665	stable	ZNF26
0.021926849	stable	ARRDC5
0.02193495	stable	LOC647570
0.021943668	stable	TRIM28
0.021990462	stable	KLF10
0.022029386	stable	RCN2
0.022039776	stable	ZFP36L1
0.022078074	stable	ZDHHC14
0.022083779	stable	KRT8
0.022083779	stable	PRKRIR
0.022090096	stable	PRND
0.022121798	stable	NEK3
0.022130618	stable	SPINK1
0.02213332	stable	FAM73A
0.02213332	stable	SLC25A46
0.022133435	stable	BUB1
0.022166048	stable	CDR2
0.022258925	stable	LOC653147
0.022284385	stable	DDX51
0.022372239	stable	SESTD1
0.022374346	stable	ZCCHC14
0.022389646	stable	ODC1
0.022441373	stable	TTMA
0.022498852	stable	GPR89C
0.022500725	stable	PHLPP
0.022501774	stable	HSD17B4
0.022511692	stable	RPUSD4

0.022530951	stable	C21ORF55
0.022571826	stable	LOC643356
0.022610194	stable	FLJ11292
0.022717019	stable	COMMD4
0.022740446	stable	STXBP1
0.022854503	stable	TMIE
0.022894484	stable	TRPC6
0.022913698	stable	XKR8
0.022952644	stable	GDF9
0.022957501	stable	PPM1E
0.022957501	stable	MYO7A
0.022973377	stable	NPPC
0.023008847	stable	LOC728006
0.02303058	stable	PPARD
0.023133287	stable	NUP35
0.023138698	up	NPPA
0.023150856	stable	C1ORF159
0.023208157	stable	KRTCAP3
0.023208157	stable	LTV1
0.02327331	stable	FGF11
0.023278124	stable	BAD
0.023278124	stable	SNURF
0.023309467	stable	ELMO3
0.023312249	stable	PRLH
0.023326978	stable	CREB3L3
0.023355072	stable	ZNF225
0.023376394	stable	CALHM2
0.023409492	stable	UBXN4
0.023410559	stable	SETD4
0.023423536	stable	ZNF775
0.023429281	stable	NANOS3
0.023438562	stable	FUT6
0.023447929	stable	MGC27345
0.023448215	stable	CAMTA1
0.02353447	stable	NUDCD1
0.02355206	stable	EBAG9
0.02355206	stable	C3ORF39
0.023555832	stable	RILP
0.02357301	stable	C18ORF56
0.023575461	stable	ZNF384
0.023575461	stable	CD200R2
0.023575461	stable	ZNF69
0.023588856	stable	CDC14B
0.023629753	stable	STRN4
0.023671097	stable	WWC3
0.023689875	stable	CORO2B
0.023726352	stable	S100A2
0.023779169	stable	FHOD1
0.023791972	stable	CLSTN1
0.023811119	stable	PDCL3
0.023814687	stable	RHBDL2

0.023861847	stable	LOC642277
0.023942916	stable	LOC646667
0.024034226	stable	MTMR11
0.024085088	stable	LOC388282
0.02409508	stable	TMEM128
0.024106786	stable	OR2A9P
0.024106786	stable	FEM1A
0.024135996	stable	PLGLA
0.024136456	stable	NISCH
0.024163604	stable	FLJ40473
0.024203996	stable	DGKA
0.024214222	stable	RUNDC2C
0.024277122	stable	TNFSF15
0.02438802	stable	TSPO
0.024413071	stable	GRIP2
0.024428821	stable	C21ORF62
0.024435911	stable	FKSG24
0.024509613	stable	CXORF36
0.024523741	stable	MGC15763
0.024548461	stable	ACSS1
0.02458316	stable	MTMR12
0.024620641	stable	ZNF184
0.024651185	stable	NDUFS3
0.02467809	stable	SCPEP1
0.02470698	stable	CD302
0.02473997	stable	KCNJ12
0.024786986	stable	GPR89B
0.024817341	stable	IGSF3
0.024831812	stable	CHCHD3
0.024932901	stable	RBM45
0.024932901	stable	MTR
0.02494729	stable	STXBP5
0.02494729	stable	ANGEL2
0.0249545	stable	C3ORF36
0.024967178	stable	LOC727909
0.025049979	stable	TXNDC9
0.025091218	stable	KCNMB2
0.025096367	stable	C1ORF71
0.025131378	stable	TM2D3
0.025142845	stable	LOC644961
0.02521494	stable	C8ORF44
0.02523293	stable	DSCR5
0.025244076	stable	MARCH3
0.025244076	stable	NAB2
0.025250417	stable	C15ORF23
0.025256164	stable	PPEF1
0.025340397	stable	ISL1
0.025352009	stable	LOC646019
0.025369378	stable	MCEE
0.02539281	stable	DKFZP451M2119
0.025428188	stable	LOC728492

0.025437658	stable	TRAF3IP2
0.025482218	stable	FIBCD1
0.025483464	stable	LOC641693
0.02550409	stable	LOC652773
0.025540967	stable	FANCC
0.025558216	stable	LOC644935
0.025605767	stable	AADAT
0.025605937	stable	ORC6L
0.025625704	stable	C4ORF39
0.025635446	stable	C17ORF49
0.02564641	stable	ZC3H8
0.025656008	stable	WRB
0.025668369	stable	TAF8
0.025668369	stable	NCAN
0.025696027	stable	TRIM27
0.025699965	stable	PDCD10
0.025714627	stable	LOC644537
0.025714627	stable	TIMP3
0.025718804	stable	MAGMAS
0.025718804	stable	B2M
0.025726024	stable	RAD51C
0.025763845	stable	RQCD1
0.025763845	stable	FGFR1OP2
0.025848417	stable	CTSA
0.025852221	stable	ZNF264
0.025863913	stable	LOC391771
0.02600496	stable	ST6GALNAC1
0.026050871	stable	RRN3
0.026056529	stable	SYP
0.02615082	stable	C22ORF32
0.026176851	stable	APOB
0.026210722	stable	LOC646119
0.026224785	stable	FBXO34
0.026224785	stable	NIP30
0.026244173	stable	EZH2
0.026244173	stable	ARL6
0.026325935	stable	LOC399900
0.026394761	stable	SH3YL1
0.026429256	stable	PMCH
0.026433676	stable	MPEG1
0.026469067	stable	KLF13
0.026488028	stable	GRPEL2
0.026562484	stable	C3ORF30
0.026582776	stable	C5ORF42
0.026585283	stable	ATAD2
0.026696854	stable	RBM4
0.026696854	stable	ARL3
0.026700581	stable	C14ORF94
0.026722596	stable	MRPL14
0.026730422	stable	LOC648706
0.026736539	stable	ZNF322A

0.026798979	stable	UBL5
0.026834282	stable	SERPINF1
0.026851338	stable	C1QTNF6
0.02685869	stable	USP3
0.026864883	stable	TMEM66
0.026874459	stable	LOC641896
0.026907984	stable	MYLIP
0.026907984	stable	DOT1L
0.026912888	stable	PHF14
0.026976828	stable	SEL1L
0.027038922	stable	ARHGAP26
0.027114368	stable	RPUSD2
0.027114368	stable	LOC439957
0.027114368	stable	TMEM136
0.027128261	stable	TIMM17B
0.027128759	stable	CNOT10
0.027158013	stable	C16ORF86
0.027204003	stable	SSX5
0.027233646	stable	LOC646317
0.027233646	stable	PRPS2
0.027262387	stable	CENPJ
0.027278277	stable	ACTR1A
0.027329975	stable	VAPB
0.027480121	stable	TMTC2
0.027543578	stable	XPOT
0.027587987	stable	LPCAT2
0.027599418	stable	ACOX3
0.027615284	stable	CRTAC1
0.027615284	stable	KCNH2
0.027652674	stable	ORMDL2
0.02765528	stable	KIF9
0.027686013	stable	UTP11L
0.027705728	stable	IWS1
0.027720549	stable	C6ORF120
0.027731843	stable	LOC388181
0.027741363	stable	ADH1C
0.027741909	stable	FLRT3
0.027749143	stable	TRIM21
0.02778958	stable	MRPS34
0.02778958	stable	C9ORF116
0.027798294	stable	WDR61
0.027819721	stable	CCR5
0.027848794	stable	C6ORF222
0.027848794	stable	ZNF227
0.027866404	stable	C19ORF25
0.027976813	stable	PIK3AP1
0.028072932	stable	HIST1H2BC
0.028092649	stable	MAPK8IP1
0.028107903	stable	TRNAU1AP
0.028108205	stable	BCAS3
0.028108205	stable	REEP5

0.028114391	stable	APBA2BP
0.028139013	stable	KLRB1
0.028142749	stable	ERCC3
0.028156117	stable	LOC652634
0.028202248	stable	SFXN5
0.028269589	stable	BRMS1L
0.028291088	stable	PITPNM2
0.028354443	stable	LOC654260
0.028443068	stable	GPR137B
0.028451935	stable	IFIT5
0.028451935	stable	ZNF154
0.028451935	stable	RIF1
0.028451935	stable	LOC728844
0.028451935	stable	LOC648695
0.028451935	stable	HDAC4
0.028486501	stable	DYNLT3
0.028543777	stable	MDM4
0.028543777	stable	DHX30
0.028543777	stable	P2RY8
0.028551019	stable	BAIAP2
0.028624217	stable	P2RY14
0.028629656	stable	AP2A2
0.028813895	stable	LOC144983
0.028826014	stable	ZNF608
0.028853097	stable	STARD3NL
0.028884434	stable	ZNF555
0.028908616	stable	TFPT
0.028908616	stable	NDUFV3
0.028976238	stable	PIP5K2A
0.02900434	stable	TTLL5
0.029019353	stable	SKIV2L2
0.029072048	stable	PHF20L1
0.029072048	stable	NT5C1A
0.029110184	stable	CKB
0.029150718	stable	SAA2
0.029164205	stable	LCAT
0.02917364	stable	PRAM1
0.029206704	stable	NCOA1
0.029226261	stable	HISPPD2A
0.02923091	stable	JAM2
0.02932282	stable	AGPAT2
0.029362274	stable	F12
0.029369363	stable	ACIN1
0.029382056	stable	NOL11
0.029391743	stable	MMS19L
0.029392416	stable	INPP5B
0.029435397	stable	DAG1
0.029473096	stable	ANKRD9
0.029511808	stable	LOC642678
0.029523106	stable	FAHD2A
0.029524074	stable	MAPKAPK5

0.029600387	stable	ZNF519
0.029610181	stable	OPN1MW
0.029654474	stable	SUMO2
0.02967784	stable	C21ORF56
0.029687261	stable	PCMT1
0.029695958	stable	C19ORF47
0.029705793	stable	CKS1B
0.029781976	stable	LOC649555
0.029809005	stable	TXNL4A
0.029817314	stable	EWSR1
0.029828556	stable	KIAA1704
0.029851191	stable	LEP
0.029985024	stable	DEF8
0.030034167	stable	PPFIBP1
0.030081087	stable	ZDHHC24
0.030113329	stable	CEP170
0.030171139	stable	RAE1
0.030225868	stable	ATOH8
0.030263078	stable	SNAPIN
0.030301555	stable	MKX
0.030321022	stable	SLC7A6OS
0.030355017	stable	CNNM3
0.030425112	stable	OR4P4
0.030468789	stable	PDE6C
0.030471152	stable	CDK5RAP3
0.030483721	stable	ABCC4
0.030497609	stable	PIGM
0.030506153	stable	PPM2C
0.030537605	stable	DYNLL1
0.03053879	stable	LOC654042
0.030540413	stable	FBXO28
0.030651785	stable	PHF8
0.030678452	stable	GGA2
0.030678452	stable	MMP16
0.030695185	stable	TASP1
0.030751568	stable	LOC647543
0.030751568	stable	ARRB2
0.030768752	stable	LOC643242
0.030768752	stable	ELFN2
0.030786128	stable	KIAA0241
0.030795054	stable	SUCLG1
0.030871429	stable	OR2Y1
0.03088838	stable	ELN
0.030894063	stable	NARS2
0.030908402	stable	AGPAT3
0.030908402	stable	LBH
0.03091025	stable	C6ORF204
0.030979767	stable	TRIM55
0.030979767	stable	RAB3GAP1
0.030988108	stable	CPSF2
0.031001892	stable	PHC3

0.031009517	stable	LOC643668
0.031016389	stable	LRMP
0.031081211	stable	SELL
0.031097205	stable	ANKRD28
0.031097778	stable	C10ORF4
0.031124745	stable	NIPA1
0.031173528	stable	SIP1
0.031213377	stable	C20ORF45
0.031243278	stable	USP33
0.03134852	stable	OR56B4
0.031356948	stable	EIF3G
0.031452484	stable	SELENBP1
0.031480065	stable	TMEM60
0.03148061	stable	LOC442060
0.031511618	stable	BCL7C
0.031512176	stable	LOC647766
0.031514955	stable	TP53AP1
0.031551106	stable	SCGB1D1
0.031551106	stable	VWA5A
0.031613258	stable	MRAS
0.031620601	stable	SP5
0.031622843	stable	RNASEH2B
0.03165068	stable	FLJ23152
0.03170493	stable	C10ORF47
0.031758239	stable	GIPC1
0.031766041	stable	THUMPD1
0.031766041	stable	PDIA2
0.031826841	stable	ABCD3
0.031850443	stable	RBAK
0.031858398	stable	DDX1
0.031933113	stable	LOC651546
0.03197979	stable	PARN
0.032055363	stable	TSPAN31
0.032070302	stable	LOC728944
0.032102567	stable	KCND2
0.032102567	stable	LOC389257
0.03212473	stable	EBP
0.03212473	stable	LOC96597
0.032154266	stable	ACTR1B
0.03216791	stable	EIF3J
0.032171878	stable	PTGIR
0.032244517	stable	KY
0.03224503	stable	ALDH2
0.032247467	stable	TTTY5
0.032285146	stable	LOC145837
0.032334783	stable	GKAP1
0.032398582	stable	BTBD15
0.032418566	stable	RBBP8
0.032540394	stable	AES
0.032549657	stable	LOC644336
0.032550228	stable	UTP15

0.032551653	stable	KLHL22
0.032552568	stable	CCDC3
0.032624559	stable	FLJ46358
0.032658315	stable	SLC20A2
0.032725692	stable	PNPT1
0.032742863	stable	ST3GAL4
0.032742863	stable	RANBP6
0.032750762	stable	SLC25A40
0.032750762	stable	C1QTNF9
0.032790164	stable	LOC642372
0.032835136	stable	HDAC1
0.032853659	stable	CPEB2
0.032873763	stable	FLOT1
0.032875059	stable	XKR6
0.032929267	stable	ZNF364
0.032998258	stable	DERPC
0.033019311	stable	TRA1P2
0.03302107	stable	DDX54
0.033026533	stable	SCN4B
0.033062472	stable	LRRC59
0.033071187	stable	TMOD4
0.033087677	stable	ICOS
0.033155072	stable	YWHAB
0.033191885	stable	PCDH19
0.033191885	stable	DNAJC5G
0.033191885	stable	C22ORF36
0.033191885	stable	LOC648399
0.033238279	stable	NR0B2
0.033239219	stable	MID1
0.033247758	stable	AFF1
0.033294032	stable	LOC650369
0.033318869	stable	PSMA2
0.033372077	stable	FBN3
0.033379242	stable	KLRG2
0.033386411	stable	TLE3
0.033411697	stable	SSRP1
0.033438759	stable	C6ORF150
0.033463329	stable	PIWIL4
0.033486413	stable	GTF2IRD2P
0.033487971	stable	WNK1
0.033496669	stable	SPHAR
0.033530432	stable	SHOX2
0.033561007	stable	LOC643431
0.033599998	stable	MPP4
0.033608117	stable	HS3ST3A1
0.03362821	stable	LOC402057
0.033648015	stable	ADAM8
0.033665096	stable	WWP1
0.033665096	stable	LOC653182
0.033744684	stable	LOC90586
0.033764023	stable	DEFA3

0.033890502	stable	HRCT1
0.034014715	stable	MFSD3
0.034017217	stable	SLC28A2
0.034029667	stable	RPL13L
0.034077123	stable	GPBP1
0.034117252	stable	NAV2
0.034134245	stable	TRIM74
0.034152804	stable	EIF4H
0.034184895	stable	EXOC3
0.034220262	stable	DDX31
0.034230207	stable	CLCN2
0.034243752	stable	SFRS15
0.034278495	stable	ACP5
0.034282239	stable	LYSMD2
0.034282239	stable	NXF2B
0.034300648	stable	PPIG
0.034436972	stable	PGA4
0.034534735	stable	C19ORF50
0.034558449	stable	BZW2
0.034653465	stable	MOXD1
0.034677748	stable	PARP4
0.034717776	stable	PDZRN4
0.03472257	stable	ANKRD11
0.034747308	stable	ADAMTS13
0.034769678	stable	DNAI2
0.034787987	stable	REL
0.034797241	stable	C22ORF39
0.034805505	stable	CADM3
0.034879764	stable	MAP2K2
0.034904644	stable	PA2G4
0.035035605	stable	CHD1L
0.035035605	stable	ATP5F1
0.03509444	stable	FAM49A
0.035151803	stable	NARS
0.035171735	stable	FBXO5
0.035176402	stable	ZHX2
0.035188459	stable	LOC644538
0.035274579	stable	APPL2
0.035324922	stable	HIGD1B
0.035327444	stable	AFTPH
0.035440761	stable	RIPK5
0.03546982	stable	NOVA1
0.035470253	stable	DACT1
0.035540468	stable	CLCNKA
0.035554672	stable	KIAA1571
0.035724936	stable	PABPC5
0.035737958	stable	FTH1
0.035742489	stable	SPOP
0.035745632	stable	LGALS3BP
0.035755402	stable	PARK2
0.035755823	stable	CDC14A

0.035765531	stable	LOC23117
0.035778135	stable	PPP2R1A
0.035839739	stable	GNB2L1
0.035852588	stable	ARSD
0.035852588	stable	LOC644251
0.035865673	stable	EXOSC1
0.035865673	stable	FLII
0.03586647	stable	PRKCI
0.035912694	stable	LMO2
0.035933047	stable	NF1
0.035955468	stable	LOC652493
0.036030662	stable	SLC44A4
0.036054873	stable	C16ORF53
0.036054873	stable	YES1
0.036085311	stable	GNAI1
0.036085311	stable	FLRT2
0.036156406	stable	NKIRAS2
0.036156406	stable	TMEM110
0.036214472	stable	PRKAB1
0.036214554	stable	PRTFDC1
0.036245438	stable	RBCK1
0.036396601	stable	CREB3
0.036399788	stable	TAZ
0.036402549	stable	ULK2
0.036402549	stable	LOC648710
0.036503887	stable	BRE
0.036505167	stable	PPP1R13B
0.036507266	stable	PLA2G4F
0.036521725	stable	RND3
0.036571258	stable	PSMA3
0.036699412	stable	ANKRD10
0.036703266	stable	PAIP1
0.036810944	stable	LCOR
0.036826176	stable	LRP1B
0.036826176	stable	RHOXF1
0.036853797	stable	FAM100B
0.036869173	stable	GANC
0.036869173	stable	LOC652479
0.036869173	stable	DNMT3B
0.036930602	stable	SLC5A7
0.036950718	stable	FN3K
0.03695852	stable	GCC1
0.036965163	stable	C11ORF82
0.036969408	stable	SOX9
0.037032758	stable	LOC441179
0.037041784	stable	PRPSAP2
0.037075736	stable	PI3
0.037077664	stable	ZNF251
0.037104108	stable	WSB1
0.037154788	stable	FRAP1
0.037171027	stable	MEN1

0.037185436	stable	ATRIP
0.037225833	stable	TGFB3
0.037242674	stable	PAPOLG
0.037471005	stable	JAG1
0.037476186	stable	LOC644530
0.037476186	stable	KIF1B
0.037497173	stable	SORBS3
0.037498683	stable	EHMT1
0.037522696	stable	ASPA
0.037537167	stable	NOMO2
0.037541813	stable	CAST
0.037567768	stable	TMEM22
0.037577632	stable	LOC642578
0.037606381	stable	SERBP1
0.037753601	stable	SAR1B
0.037753601	stable	RPS17
0.037856751	stable	C16ORF62
0.037972282	stable	PRRT3
0.037972282	stable	COX6C
0.038008702	stable	UBE2I
0.038091036	stable	BPHL
0.038143752	stable	FLJ32154
0.038175001	stable	SH3BP1
0.038216287	stable	B3GNT6
0.038250787	stable	LOC150223
0.038333099	stable	RPRD1A
0.038333099	stable	LOC493869
0.038340592	stable	ID2
0.038357484	stable	LSM8
0.03841656	stable	PRDM16
0.038518456	stable	ZNF533
0.038581435	stable	PHKA1
0.038656406	stable	PSMA7
0.03867079	stable	AMDHD1
0.038671985	stable	TMSB4X
0.038738246	stable	PHIP
0.038855644	stable	ITPKA
0.038917852	stable	TWF1
0.038942146	stable	SFRS1
0.038988053	stable	TMED10
0.038988053	stable	RANBP3
0.039005017	stable	TMCO6
0.039041322	stable	RPLP2
0.039068452	stable	LOC642398
0.03916218	stable	NPFFR2
0.039279687	stable	SMAD3
0.039322499	stable	RNPEP
0.039349361	stable	NPTX1
0.039360071	stable	ZNF155
0.039401318	stable	LOC649711
0.039472121	stable	LOC649209

0.039516142	stable	PDCD2L
0.039551888	stable	KCNG2
0.039673644	stable	POLE
0.039692683	stable	LOC644466
0.039693661	stable	GCK
0.039704687	stable	LOC645683
0.039712181	stable	GAPVD1
0.039839497	stable	LOC730360
0.039878948	stable	BUD13
0.039878948	stable	PPA2
0.039884648	stable	CA5B
0.039951316	stable	FBLN5
0.039970376	stable	LOC641710
0.039971108	stable	DNAJB1
0.039976961	stable	ETNK2
0.039996891	stable	C7ORF28A
0.040031577	stable	LOC646342
0.040039205	stable	SLC35C1
0.040057622	stable	FLJ20160
0.040057622	stable	ABO
0.040164712	stable	MTA3
0.040238071	stable	FCRL1
0.040277793	stable	WASF2
0.040351073	stable	GYG1
0.040378201	stable	ADRA2C
0.040387426	stable	RASGRP1
0.040442043	stable	SIM2
0.040560568	stable	GPR132
0.040644669	stable	CCNC
0.040770388	stable	C1ORF210
0.040791447	stable	TICAM2
0.040791447	stable	GP1BA
0.040870753	stable	ATP8B4
0.040896425	stable	MSTP9
0.040980832	stable	CCDC59
0.040999678	stable	GTF2A2
0.041015035	stable	AAA1
0.041054668	stable	POLM
0.041056998	stable	ZC3HAV1
0.041065236	stable	ASGR1
0.041090257	stable	DCTN5
0.041101116	stable	FANCD2
0.041101116	stable	KIAA1797
0.041178916	stable	ZCCHC8
0.041178916	stable	LOC645600
0.041183565	stable	DKFZP761P0423
0.041185741	stable	SAP30L
0.041192465	stable	ZBTB20
0.041272079	stable	MUSK
0.04129246	stable	BAT5
0.041316967	stable	LOC648099

0.041317321	stable	ANKRD43
0.041330811	stable	C1ORF26
0.041332355	stable	BBS5
0.041364277	stable	C9ORF58
0.041417547	stable	PTPN13
0.04143668	stable	LOC401052
0.041451199	stable	ZNF193
0.041464233	stable	CTCF
0.041472312	stable	FARSB
0.041522421	stable	AMDHD2
0.04153401	stable	OR51B2
0.04156248	stable	MLX
0.041566939	stable	S100A1
0.041567602	stable	MAGEA6
0.041657564	stable	MELK
0.041788078	stable	DOC2B
0.041823762	stable	FAM44B
0.041850105	stable	TMEM59
0.041850207	stable	PTGFR
0.041877581	stable	ERO1L
0.041877581	stable	LOC149837
0.042032645	stable	LOC400721
0.042213554	stable	N4BP1
0.042233788	stable	TCF12
0.042296814	stable	TGFBRAP1
0.042302107	stable	LOC652699
0.042302107	stable	LOC646900
0.042341506	stable	PRKCE
0.042362068	stable	LILRB1
0.042368233	stable	NEK8
0.042415591	stable	CBWD6
0.042436456	stable	EIF5
0.042436456	stable	GATC
0.042440238	stable	LOC347376
0.042465027	stable	LOXL4
0.042465027	stable	PCTP
0.042477284	stable	LOC730077
0.042533952	stable	CES4
0.042565336	stable	LOC642660
0.042571201	stable	METTL3
0.042631978	stable	FLJ32252
0.042654799	stable	C17ORF48
0.042733945	stable	SLC35E1
0.042796304	stable	SHC4
0.042806014	stable	EMX1
0.042997351	stable	C10ORF39
0.043001836	stable	CAPN7
0.043001836	stable	HSD17B11
0.043017494	stable	MDM2
0.043021142	stable	EPS15
0.043030586	stable	RPL27A

0.043041904	stable	ZNF429
0.043057558	stable	ZNF607
0.04307193	stable	ABHD3
0.043162643	stable	LOC648494
0.043179488	stable	SYT4
0.043193108	stable	CSTB
0.043193108	stable	CENPE
0.043228119	stable	LIMK2
0.043281018	stable	SULT1A2
0.043424475	stable	APOOL
0.043463508	stable	RGS7BP
0.043480388	stable	DAD1
0.043499962	stable	RAB33A
0.043499962	stable	HNT
0.043616665	stable	SEC11C
0.043622345	stable	C8ORF31
0.043684995	stable	C16ORF90
0.043684995	stable	LOC653895
0.043694624	stable	C1ORF103
0.043845295	stable	CMAS
0.043862807	stable	FER
0.043881709	stable	DENND5B
0.043881709	stable	PSMD14
0.04401032	stable	ATP5A1
0.044012773	stable	FASTKD3
0.044021086	stable	LOC650706
0.04416546	stable	AHCTF1
0.044275667	stable	ASB1
0.044294425	stable	ZNF695
0.044350362	stable	RNF25
0.044485876	stable	LOC653214
0.044513669	stable	ASF1B
0.044579564	stable	FCHO1
0.044663056	stable	ARG1
0.044690755	stable	PCYT2
0.044696446	stable	C1ORF176
0.044720639	stable	CDC27
0.044720639	stable	CRAT
0.044720639	stable	CFLAR
0.044727566	stable	MYCBPAP
0.044941106	stable	PIGV
0.044997942	stable	DCDC5
0.045040158	stable	LSM1
0.04507341	stable	BNIP3L
0.04513037	stable	LOC389672
0.045279262	stable	LOC387934
0.045378533	stable	PSCA
0.045546935	stable	METTL10
0.045566667	stable	CLK2
0.045566667	stable	PPAN-P2RY11
0.045581846	stable	PNPLA2

0.045677297	stable	FRMD6
0.045689682	stable	C1ORF152
0.045706333	stable	L3MBTL
0.045706333	stable	RAC1
0.045706333	stable	THSD1
0.045757368	stable	SDK1
0.045785328	stable	GALNTL1
0.04579003	stable	LOC642771
0.045840095	stable	ITGA11
0.045969698	stable	C4ORF32
0.045988596	stable	UBE2F
0.046176969	stable	LOC642787
0.046176969	stable	C7ORF55
0.046227727	stable	KIF1C
0.046227727	stable	ZNF713
0.046227727	stable	C19ORF48
0.046227727	stable	KIAA0323
0.046250407	stable	CACNA2D3
0.046315678	stable	FAHD1
0.046358169	stable	SLC35A4
0.046359898	stable	ANKRD54
0.046457402	stable	TOR2A
0.046482188	stable	TLR1
0.046503573	stable	RNF208
0.04655421	stable	ZNF347
0.046599099	stable	BSG
0.046599099	stable	PRSS27
0.046620958	stable	ORAI2
0.046660779	stable	GCAT
0.046688974	stable	RHOBTB2
0.046692929	stable	LOC440132
0.046727304	stable	MDN1
0.046741787	stable	LRRC55
0.046777103	stable	LOC645466
0.046791478	stable	ANKH
0.046831249	stable	ZNF294
0.046876977	stable	IL2RG
0.046917125	stable	GUF1
0.046927758	stable	ZNF202
0.046970464	stable	DLEU1
0.046999181	stable	RANBP2L1
0.047060034	stable	LEAP-2
0.047060034	stable	RP11-529I10.4
0.04707906	stable	LOC644012
0.047104847	stable	RPESP
0.047136976	stable	POFUT1
0.047161925	stable	BSN
0.047164173	stable	TMEM219
0.0472055	stable	COX7B
0.047213216	stable	TMSB4Y
0.047264433	stable	TNFAIP8L2

0.047264433	stable	LOC644380
0.047277203	stable	LOC441087
0.047282884	stable	LOC652404
0.047369152	stable	PGM5
0.047396106	stable	FOXP1
0.047396106	stable	DCTN3
0.047419721	stable	RASGEF1C
0.047438791	stable	RGS3
0.047526286	stable	MEF2D
0.047655779	stable	LOC646304
0.047691852	stable	SOD3
0.047722889	stable	CHPT1
0.047726501	stable	TPK1
0.047726501	stable	ACPP
0.047773811	stable	XPA
0.047774305	stable	LOC554208
0.047801765	stable	KIF2A
0.047872498	stable	LOC651723
0.047872498	stable	KIAA0664
0.047934218	stable	GOLPH3L
0.048050755	stable	C9ORF5
0.048086858	stable	HIST1H2BL
0.048116224	stable	ZC3H12A
0.048127442	stable	NTRK2
0.048223181	stable	KIAA1199
0.04823948	stable	PRRG4
0.04823948	stable	DEFB118
0.04823948	stable	GPR177
0.048265734	stable	DUSP11
0.048298731	stable	LOC653227
0.048334884	stable	KCNH3
0.048385572	stable	SLC6A19
0.048438332	stable	KREMEN1
0.048441255	stable	FAM160A2
0.048441255	stable	LOC440396
0.048441255	stable	SQRDL
0.048507426	stable	HAX1
0.048511103	stable	HIBADH
0.048535502	stable	RTP4
0.048560056	stable	VCPIP1
0.048560091	stable	C17ORF74
0.048660718	stable	LASS1
0.048660718	stable	LOC642113
0.048684048	stable	CLTCL1
0.04870022	stable	MAP3K12
0.048724013	stable	RBM25
0.048744733	stable	LOC653748
0.048792083	stable	LOC220729
0.048792083	stable	CBX2
0.048803973	stable	PANX1
0.048831531	stable	MCART1

0.048841034	stable	ALDH18A1
0.049010633	stable	SUDS3
0.049090664	stable	C1QTNF2
0.049128351	stable	UQCRH
0.049189201	stable	KNTC1
0.049195218	stable	ZFX
0.049201097	stable	LOC728069
0.04927505	stable	KIRREL
0.049297454	stable	ATP7A
0.049298721	stable	ATF7IP
0.049306643	stable	PDCD1
0.049309037	stable	MYH14
0.049319503	stable	EPS15L1
0.049329185	stable	ITGAL
0.049420811	stable	RFC3
0.049469926	stable	LOC81691
0.04947163	stable	LOC651407
0.049550076	stable	FAM36A
0.049647613	stable	FAM80B
0.049826374	stable	THEM4
0.04986304	stable	GMPR
0.049915078	stable	SLC44A5
0.049915078	stable	DDR2
0.05006541	stable	ATP6V1G1
0.050081178	stable	SH2D1A
0.050086731	stable	RPS6KA5
0.050090628	stable	ZFAND2B
0.050211675	stable	TWIST2
0.050211675	stable	LAT
0.050216256	stable	MRPL39
0.05025563	stable	SLC12A8
0.050257316	stable	CCDC102B
0.050271054	stable	FBXO16
0.050271461	stable	HSD11B1L
0.050271461	stable	FAM76B
0.0502898	stable	RDH16
0.050316913	stable	OTUD7A
0.050316913	stable	CCL24
0.050326917	stable	AKAP7
0.050331464	stable	KIAA0101
0.050331464	stable	WDR5B
0.050455628	stable	LOC644268
0.050471839	stable	LOC652623
0.050562855	stable	ANAPC7
0.050596942	stable	RAP1A
0.050667123	stable	RBM9
0.050749501	stable	PCBP4
0.050763271	stable	DKK4
0.050824333	stable	CNN3
0.050897478	stable	LOC645070
0.051036445	stable	FALZ

0.051046781	stable	HIAT1
0.051075979	stable	GABARAP
0.051159725	stable	HNRNPF
0.05116885	stable	TMED10P
0.051171085	stable	IL6ST
0.051228079	stable	MMAB
0.051228079	stable	SNRPG
0.051291747	stable	SNPH
0.051291747	stable	CCDC97
0.051389916	stable	PROM2
0.051412775	stable	GDPD3
0.051438119	stable	NT5C3L
0.051438119	stable	CLDND1
0.051473132	stable	ZBTB46
0.051608931	stable	LAIR2
0.051616592	stable	FTHL12
0.05164015	stable	LOC653803
0.051647005	stable	DSC2
0.051647005	stable	LILRB3
0.051724392	stable	RAD51L3
0.051724392	stable	SVOP
0.051785711	stable	LOC649775
0.05180542	stable	LOC220686
0.051838521	stable	DDX28
0.05191112	stable	TMEM134
0.051912636	stable	C19ORF56
0.051939536	stable	SYNPR
0.051994954	stable	USP9Y
0.052074337	stable	SLC13A1
0.052085683	stable	HBXIP
0.052085683	stable	BTG1
0.052173665	stable	MRPS17
0.052343284	stable	GPC4
0.052359952	stable	PUS7L
0.052359952	stable	OR3A4
0.052482921	stable	SELM
0.052593431	stable	TAF7L
0.052593431	stable	KRT19
0.052593431	stable	METTL2A
0.052593431	stable	SHROOM2
0.052627716	stable	GBP5
0.052627716	stable	CECR1
0.0527146	stable	NDUFA11
0.0527146	stable	COMMD5
0.05274359	stable	LOC727825
0.052779847	stable	PLA2G2D
0.052898887	stable	MED24
0.052902098	stable	C17ORF28
0.052922984	stable	LOC646483
0.052922984	stable	CDRT4
0.052953944	stable	PSG6

0.052982421	stable	SLC24A1
0.0530533	stable	CD8A
0.053116858	stable	ACTR3
0.053146951	stable	LOC650885
0.053202552	stable	FSD1CL
0.053219358	stable	ABI3BP
0.053232894	stable	GBE1
0.053237093	stable	MRPS24
0.053239316	stable	NCOR1
0.053344884	stable	NLRP12
0.053394821	stable	CPVL
0.053431112	stable	MAP2K4
0.053481861	stable	OSBPL11
0.05348465	stable	HDDC3
0.05349621	stable	CCDC137
0.05349621	stable	LOC653071
0.05360208	stable	MGC48637
0.05360208	stable	ZBTB5
0.05360208	stable	TRIM16L
0.053626594	stable	PEX5
0.05363198	stable	C19ORF41
0.053686104	stable	MYPN
0.053696956	stable	LOC651816
0.053765883	stable	FAM130A2
0.053765883	stable	SFRS10
0.053807791	stable	C19ORF39
0.053818353	stable	SLC6A12
0.053923	stable	ZBTB45
0.053937776	stable	PRPS1L1
0.053967318	stable	THOC1
0.054039857	stable	KLHL23
0.054071776	stable	SSBP4
0.054103659	stable	DENND1A
0.054164405	stable	CCBE1
0.054193194	stable	PCDHB8
0.054222537	stable	LOC645004
0.054231686	stable	TMED1
0.054371592	stable	NKX1-2
0.054538716	stable	SLC38A4
0.054538716	stable	PGM2
0.054562078	stable	MCART2
0.054592807	stable	C5ORF33
0.054606873	stable	LOC387882
0.054627558	stable	SPEF2
0.054724678	stable	LOC653338
0.054752461	stable	WFIKKN1
0.054752461	stable	PDLIM3
0.054775595	stable	LOC730031
0.054775595	stable	TTC39C
0.0547933	stable	IGFBP2
0.054798085	stable	DKFZP564J102

0.054858479	stable	LOC643821
0.054895277	stable	CENTD2
0.054895277	stable	TBC1D3B
0.054898288	stable	MIA3
0.05493166	stable	LOC202181
0.055005391	stable	CCDC74B
0.055015095	stable	TNFRSF9
0.055198623	stable	TFPI2
0.055212744	stable	STK35
0.055322996	stable	TDP1
0.055327345	stable	BBS10
0.055342197	stable	SLC6A15
0.055420686	stable	USP5
0.055434795	stable	FRMD7
0.055440931	stable	CISD2
0.055526471	stable	C11ORF87
0.055529561	stable	THAP2
0.05553957	stable	PSMC1
0.055585769	stable	LOC613037
0.055588083	stable	OR2L13
0.055664988	stable	MS4A14
0.055678924	stable	HUS1B
0.055710115	stable	MRPS11
0.055747937	stable	HLCS
0.055765786	stable	KCTD3
0.055980555	stable	CCDC106
0.056035881	stable	MORN2
0.05625669	stable	GTPBP2
0.056272194	stable	LRRC14
0.056284986	stable	ADRB1
0.056330319	stable	TDRD3
0.056427613	stable	LOC650263
0.056429397	stable	LOC728715
0.056476662	stable	LOC645058
0.0564945	stable	NDUFA7
0.056649717	stable	TBC1D17
0.056662437	stable	TLK1
0.056705616	stable	MORC3
0.056832309	stable	C7ORF57
0.056940498	stable	COX11P
0.056952492	stable	IL15
0.057039743	stable	SUCLG2
0.057139812	stable	ARMETL1
0.057151368	stable	FBXO22
0.057165597	stable	BMP5
0.057165597	stable	RRP1B
0.057195137	stable	FLJ39061
0.05721547	stable	LOC606495
0.057327163	stable	CCL18
0.057392016	stable	OR2B2
0.057392016	stable	FAM131C

0.057421564	stable	UBAP2L
0.057424894	stable	NOLA3
0.057484229	stable	IRS1
0.05759497	stable	EHD4
0.05759497	stable	PRSS35
0.057713242	stable	LRFN5
0.057768766	stable	LOC646766
0.057778915	stable	LOC652607
0.057834939	stable	LY6G5B
0.058038183	stable	SRRM2
0.05807294	stable	SAMD14
0.05807294	stable	USP2
0.058220802	stable	WBSCR16
0.058254697	stable	IPP
0.058296691	stable	KIF20A
0.058305376	stable	LOC653344
0.058339899	stable	SNORD55
0.058341978	stable	SOX7
0.058375393	stable	OSBPL8
0.058375393	stable	GGCT
0.058375393	stable	LOC644075
0.058449109	stable	POLG2
0.058457058	stable	RCP9
0.058531761	stable	ZNF185
0.058531761	stable	GLT6D1
0.058587712	stable	RASSF6
0.058615022	stable	LOC653108
0.058617935	stable	LOC645983
0.058728886	stable	CLMN
0.058775355	stable	HHLA3
0.058831261	stable	KCNK17
0.058873769	stable	PRPF6
0.058891205	stable	PISD
0.058949262	stable	CGI-96
0.058949262	stable	CRHBP
0.058969761	stable	APC
0.058986576	stable	SAFB2
0.059044479	stable	GNB2
0.059076246	stable	LOC647596
0.059126178	stable	MTA1
0.05915548	stable	GEN1
0.059172695	stable	TBL1X
0.059172695	stable	NFYA
0.059250553	stable	CITED4
0.059250553	stable	PAP2D
0.059296145	stable	MGC62100
0.059346107	stable	NAPRT1
0.059414032	stable	VPS13C
0.059430339	stable	INHBE
0.059544735	stable	LOC134145
0.059576363	stable	ACCN2

0.05959519	stable	IL10
0.059616381	stable	HCN3
0.059734961	stable	PDCD7
0.059735985	stable	C14ORF11
0.059822806	stable	LOC642559
0.059829665	stable	DNCL1
0.059890939	stable	SSH3
0.059914346	stable	EIF2AK2
0.059914346	stable	UGCGL2
0.059914346	stable	RPS6KA6
0.05994144	stable	POPDC3
0.060044097	stable	RNF6
0.060054871	stable	GATS
0.060135896	stable	ZNF770
0.060193626	stable	PVT1
0.060265557	stable	NACAP1
0.060274623	stable	C1ORF41
0.060287731	stable	B3GNT5
0.060317657	stable	TMEM170A
0.060355554	stable	SHB
0.060356653	stable	ZNF420
0.060416138	stable	C9ORF95
0.060417708	stable	DTWD2
0.060423489	stable	SEC14L5
0.060424416	stable	MTCH2
0.060819018	stable	ARSB
0.060950241	stable	SLC10A7
0.061011394	stable	C2ORF47
0.061060763	stable	GZMK
0.061070257	stable	SRGAP2
0.061108427	stable	HNRPH1
0.061145604	stable	TMEM69
0.061145604	stable	IFRD1
0.061206686	stable	ILVBL
0.061475906	stable	LAMB3
0.061523358	stable	LOC643652
0.061552768	stable	LRRC8C
0.061561476	stable	WDR79
0.06156617	stable	FFAR2
0.061616492	stable	ABI2
0.061652661	stable	C19ORF33
0.061747404	stable	LOC641926
0.061796593	stable	MRPL47
0.061815653	stable	CDC14C
0.061883035	stable	LOC646434
0.061917228	stable	PIM3
0.061946591	stable	C22ORF26
0.061946591	stable	ENO3
0.061998178	stable	LOC653269
0.062043261	stable	MAGEF1
0.062107223	stable	UCP3

0.06211066	stable	MCM10
0.062140015	stable	MCM8
0.062177684	stable	CLDN5
0.062199414	stable	HECTD2
0.062304289	stable	BAG5
0.062307231	stable	LOC729559
0.062378127	stable	HIST1H2BG
0.062399149	stable	C20ORF108
0.062400997	stable	LOC652816
0.062463587	stable	ZP3
0.062515287	stable	UBXN1
0.062515287	stable	HSPA12B
0.062530494	stable	LOC375295
0.062585481	stable	MLPH
0.062596284	stable	FAM62B
0.062627603	stable	PHOSPHO2
0.062683474	stable	IKZF2
0.062683474	stable	LOC645508
0.062683474	stable	ZHX1
0.062684877	stable	ANKRD27
0.062684877	stable	KATNA1
0.062727675	stable	MPND
0.062727675	stable	PXMP4
0.062773678	stable	CA8
0.062789435	stable	C9ORF46
0.062876663	stable	LOC649095
0.062931491	stable	EIF3H
0.062939099	stable	SLC46A2
0.063020064	stable	TMEM8
0.063033958	stable	MORG1
0.063307895	stable	GPR175
0.063334344	stable	GGCX
0.063378567	stable	ALOX15B
0.063378567	stable	EPR1
0.063694661	stable	PER2
0.06374169	stable	CLP1
0.06377532	stable	SRL
0.063825179	stable	SLC5A8
0.063872666	stable	SNN
0.06390668	stable	MAD2L1BP
0.063918943	stable	LOC441528
0.063953106	stable	TYRO3P
0.063961987	stable	KLHL25
0.064011197	stable	YIF1A
0.064011197	stable	SCN5A
0.064053552	stable	ATF2
0.064065385	stable	PITX1
0.064124016	stable	LOC652595
0.064130888	stable	LOC654161
0.064137224	stable	MCM3APAS
0.064148982	stable	RAI16

0.064148982	stable	RLN1
0.064310742	stable	PSMC6
0.064387917	stable	PPFIA2
0.064678885	stable	C9ORF106
0.064678885	stable	PTPRZ1
0.064730311	stable	JAK1
0.064736028	stable	NKX3-1
0.064749704	stable	FAM139A
0.064782699	stable	TSR2
0.064876962	stable	TOPORS
0.064985832	stable	MBD4
0.064992713	stable	LOC654074
0.065213434	stable	COQ9
0.065227464	stable	H2AFJ
0.065308611	stable	SLC13A4
0.065329726	stable	CNKSR1
0.065329726	stable	NBEAL1
0.0653396	stable	C6ORF62
0.065370254	stable	ERAP1
0.065428871	stable	UBE2M
0.065540162	stable	ZNHIT1
0.06558195	stable	PCSK1N
0.065600521	stable	TRIM26
0.065664179	stable	GM2A
0.065717768	stable	PMF1
0.065732706	stable	BIRC5
0.065758508	stable	NKAIN3
0.065758508	stable	HBA2
0.065770466	stable	TIRAP
0.065854301	stable	LOC648149
0.065877132	stable	PPIB
0.065878828	stable	CCDC149
0.065883361	stable	LOC197135
0.066039733	stable	TIMM23
0.066047021	stable	DEFB109
0.066064407	stable	CD200
0.066129868	stable	MC1R
0.066129868	stable	ITIH5
0.06614109	stable	SDCBP2
0.066163636	stable	AHSG
0.066172437	stable	HPS6
0.066214961	stable	FBXL3
0.066254976	stable	DLL3
0.066312937	stable	ZNF576
0.06638577	stable	TBCD
0.066475545	stable	ACCN5
0.066491542	stable	LOC642287
0.066690125	stable	DNAJA2
0.066764815	stable	MFSD5
0.066764815	stable	C7ORF25
0.066946407	stable	LOC652577

0.067153781	stable	CDC40
0.067153781	stable	CAMK2D
0.067305746	stable	CHAC1
0.067317378	stable	ICOSLG
0.067368708	stable	RNF38
0.06739553	stable	BICD2
0.06739553	stable	OR5J2
0.067443971	stable	TECTA
0.067480411	stable	DHCR7
0.067696924	stable	GPR89A
0.067764347	stable	MSH3
0.067807605	stable	LOC116236
0.067840588	stable	CDH5
0.067840588	stable	LOC653801
0.067842213	stable	CENPK
0.067902954	stable	HSD17B7
0.067945319	stable	PRR14
0.067955041	stable	HNRPR
0.067959762	stable	DTX3L
0.067991233	stable	FLJ45717
0.068018421	stable	LOC646447
0.068072737	stable	GLI1
0.06815373	stable	BCL2L2
0.06815373	stable	KIR2DS1
0.068175498	stable	COL1A1
0.068175498	stable	TSGA14
0.068175498	stable	GLG1
0.0681767	stable	IL17RC
0.068198448	stable	ARF4
0.068238282	stable	FMNL3
0.068301589	stable	CD79A
0.068317998	stable	GYPC
0.06835387	stable	LOC653326
0.068433418	stable	NANOS1
0.068474616	stable	KIAA1751
0.068476363	stable	ANKRD22
0.06847901	stable	TMEM39A
0.06847901	stable	TTLL6
0.06847901	stable	LOC650968
0.068705043	stable	ARG99
0.068705043	stable	MNT
0.06872034	stable	RAB18
0.068735654	stable	LOC654113
0.068761019	stable	DDX26B
0.068837577	stable	CNR2
0.068837577	stable	TEX10
0.068851249	stable	GAL3ST2
0.068851249	stable	CDKL2
0.06909659	stable	C6ORF163
0.069169222	stable	LOC391322
0.069383254	stable	C8ORF76

0.069468028	stable	LOC645447
0.069482776	stable	EGR2
0.069488881	stable	ZNF430
0.069546214	stable	TBC1D3
0.069546214	stable	BFSP1
0.06960996	stable	MBTPS2
0.06962367	stable	FAM122A
0.069629837	stable	RARG
0.069679783	stable	HOXB2
0.069793994	stable	NEDD4L
0.069831709	stable	HS2ST1
0.0698714	stable	ITGA2B
0.069953274	stable	RDBP
0.069988567	stable	CYP2C9
0.070015074	stable	MAPKAPK2
0.070067457	stable	JMJD4
0.070084086	stable	OR6B1
0.070096033	stable	MORC2
0.070121531	stable	STX8
0.070129115	stable	ARHGAP29
0.070129115	stable	DOK2
0.070158241	stable	RPL17
0.070158241	stable	UNQ9370
0.070258314	stable	LOC650566
0.07029038	stable	GAS6
0.07029038	stable	ZHX3
0.070300015	stable	F8A1
0.070320716	stable	PIGT
0.070331551	stable	SCN9A
0.070418882	stable	FAM65B
0.070442709	stable	ZNF174
0.070446807	stable	SCHIP1
0.070589417	stable	TMEM20
0.070639688	stable	C10ORF111
0.070639688	stable	GPLD1
0.070733612	stable	SGMS1
0.070750541	stable	EML4
0.070920371	stable	PMAIP1
0.071139733	stable	C5ORF5
0.071255186	stable	C10ORF108
0.071290798	stable	C8ORF38
0.071295832	stable	MRPL20
0.071443946	stable	PCGF2
0.071443946	stable	IFFO1
0.07162094	stable	ZSCAN21
0.071785099	stable	ZPLD1
0.072125013	stable	KDELC2
0.07215402	stable	CAPS2
0.072209718	stable	LOC352909
0.072269068	stable	RNF113A
0.072287663	stable	LOC643831

0.072287663	stable	LOC643774
0.072363738	stable	ACE
0.072387097	stable	C22ORF29
0.072387097	stable	LOC646676
0.072616701	stable	SYNJ1
0.072706767	stable	ADIPOQ
0.072728541	stable	LOC647135
0.072728541	stable	C17ORF95
0.072733781	stable	FSIP1
0.072733781	stable	FBXO48
0.072774542	stable	OR14I1
0.072774542	stable	ELF2
0.072830289	stable	CNTNAP3
0.07290369	stable	FSCN1
0.073003848	stable	TMEM191B
0.07303293	stable	SLC39A13
0.073034765	stable	EXOSC3
0.073120056	stable	RAB27B
0.073172102	stable	EIF2AK4
0.073260685	stable	RPL13A
0.073329026	stable	ZNF317
0.073501181	stable	RBX1
0.073601212	stable	DDB2
0.073640258	stable	LOC650577
0.073683764	stable	COL4A4
0.073785558	stable	C2ORF7
0.073911653	stable	NARFL
0.073944447	stable	YIPF2
0.07394734	stable	DFFB
0.073975238	stable	GLE1
0.074037903	stable	SCML2
0.074046292	stable	APBB2
0.074053251	stable	HCG2P7
0.074058314	stable	PIK3R3
0.074058314	stable	KLRF1
0.074058314	stable	LRRC24
0.074158495	stable	FAR1
0.074232666	stable	LRRC34
0.074232666	stable	GHRL
0.074248864	stable	LOC650003
0.074302699	stable	CCR9
0.074315751	stable	LSDP5
0.074403324	stable	VARS2
0.074403324	stable	HMGB4
0.074413443	stable	TAS2R10
0.07448054	stable	GCH1
0.07448054	stable	C14ORF124
0.074509622	stable	MYO1B
0.074515529	stable	CREM
0.074656351	stable	CP
0.074689037	stable	LOC643959

0.074713321	stable	LOC650193
0.074721381	stable	LPIN2
0.074864586	stable	LIN54
0.074879037	stable	TSSK6
0.074922795	stable	NR1H2
0.074931241	stable	RFC5
0.074956881	stable	LOC643790
0.075080679	stable	LOC730479
0.075080679	stable	IL12RB1
0.075113211	stable	NMT1
0.07512878	stable	MATK
0.075145872	stable	CREG1
0.075236024	stable	LOC646437
0.075237824	stable	LOC651326
0.075316081	stable	CCT5
0.075335541	stable	TP53I3
0.075405837	stable	LOC729882
0.075435679	stable	METT5D1
0.075524983	stable	LOC162073
0.075542188	stable	C5ORF45
0.075573014	stable	LOC729137
0.075609886	stable	PSMD12
0.075701184	stable	SLC25A23
0.075744606	stable	LOC729675
0.075802593	stable	IARS
0.075857343	stable	LOC642156
0.075916161	stable	MBOAT2
0.075916161	stable	RHOT1
0.075936085	stable	HES4
0.075987499	stable	C15ORF37
0.076103322	stable	BDH2
0.076140653	stable	C1ORF59
0.076146507	stable	UHMK1
0.076146507	stable	ZNF625
0.076153018	stable	SLC25A42
0.076284712	stable	LOC645015
0.076302461	stable	SV2A
0.076346383	stable	ATP6AP1
0.076381756	stable	LOC653450
0.076405172	stable	HR
0.076417517	stable	LOC387790
0.076438546	stable	CCDC71
0.076536099	stable	RASA2
0.076557884	stable	SEC24B
0.076574695	stable	UBE2L3
0.076649834	stable	SETDB1
0.07666206	stable	MITF
0.076739947	stable	LOC643995
0.076744597	stable	LOC646858
0.076746025	stable	ALKBH7
0.076746025	stable	HERC3

0.076746025	stable	APH1B
0.076746025	stable	LOC653383
0.076746025	stable	SLC27A3
0.076746025	stable	KCNRG
0.076808663	stable	LOC644150
0.07688721	stable	CPT1C
0.076889044	stable	WNK3
0.076889044	stable	SPATA13
0.077000067	stable	MRPS10
0.077140244	stable	DCP2
0.077210806	stable	C6ORF201
0.077210806	stable	PLEKHJ1
0.077241686	stable	LOC391767
0.07734361	stable	PTGER3
0.077417763	stable	LOC127406
0.077422725	stable	ACAP3
0.077652258	stable	ZNF236
0.077722672	stable	DYSF
0.077860572	stable	LOC400464
0.077864808	stable	MRPS9
0.077864808	stable	ADAMTS17
0.078000947	stable	UBQLN1
0.078002494	stable	ZMYM3
0.078162541	stable	ARFIP1
0.078314075	stable	LOC652140
0.078404098	stable	DOLK
0.078423138	stable	ZNF835
0.078423742	stable	C22ORF31
0.078445857	stable	CLEC2B
0.07846416	stable	CD72
0.07846416	stable	ZNF566
0.078537784	stable	POM121
0.078660974	stable	LOC652889
0.078664358	stable	LOC654032
0.078770782	stable	PLS3
0.078770782	stable	TESSP5
0.078911918	stable	PTPN22
0.078912718	stable	TNFRSF4
0.07900312	stable	C15ORF5
0.079366181	stable	GINS3
0.079376582	stable	DAPP1
0.079381135	stable	PIM2
0.079494006	stable	GRIN3A
0.079494006	stable	DAXX
0.079532231	stable	TSN
0.079599549	stable	ENC1
0.07968935	stable	DNAL4
0.07968935	stable	SSR4
0.079754318	stable	WWTR1
0.079758205	stable	FUT8
0.079780816	stable	B3GNT9

0.079873866	stable	RPUSD3
0.079926766	stable	AHDC1
0.079943331	stable	FAM118A
0.080045113	stable	RICS
0.080126795	stable	PICK1
0.080170511	stable	NOC3L
0.080174586	stable	EIF3K
0.080203369	stable	TCF15
0.080212497	stable	MSL2L1
0.080212497	stable	PCYT1B
0.080259601	stable	LOC646938
0.080265438	stable	SFXN4
0.080312486	stable	USP1
0.080354104	stable	NCOR2
0.080354104	stable	SERF1B
0.080354104	stable	CLIP4
0.080354104	stable	LYG2
0.080357006	stable	SCP2
0.080413601	stable	NDUFB1
0.080413601	stable	NNAT
0.080469106	stable	CMC1
0.080484228	stable	LOC255326
0.080552862	stable	KLHDC8B
0.080568862	stable	C6ORF159
0.080576633	stable	YY1
0.080628026	stable	CENPC1
0.080684869	stable	VASH1
0.080738293	stable	SNORD34
0.080760782	stable	RPS6KA4
0.080830109	stable	LOC642418
0.080832787	stable	C2ORF27
0.08099835	stable	CST2
0.081008581	stable	ABCA11
0.081119448	stable	TMPRSS12
0.081155122	stable	POLG
0.081189984	stable	PRELP
0.081274364	stable	GSTTP2
0.081371533	stable	ZNF532
0.081385007	stable	NTSR2
0.081402493	stable	LOC648639
0.081457133	stable	ALMS1
0.081495296	stable	FARSLB
0.08156995	stable	LOC643870
0.081854911	stable	LSG1
0.081867281	stable	UAP1L1
0.081917572	stable	TMEM91
0.082011311	stable	ALAS2
0.082168319	stable	ANXA11
0.082288287	stable	C7ORF59
0.082541874	stable	SRD5A1
0.082623963	stable	TRIM37

0.082652022	stable	LOC646134
0.082715286	stable	RDM1
0.082715596	stable	C1ORF107
0.082872121	stable	IGLL1
0.082886534	stable	LOC389517
0.082892236	stable	ABR
0.082906311	stable	CALR
0.083182124	stable	SPG7
0.083187143	stable	FMNL1
0.083252946	stable	ZNF319
0.083319673	stable	LOC730015
0.083319673	stable	NCL
0.083439577	stable	PCGF6
0.083445508	stable	JARID2
0.083462129	stable	LOC648814
0.0836904	stable	LOC644243
0.083708304	stable	SCGB2A2
0.08374091	stable	NEK9
0.083751526	stable	RECQL4
0.083799738	stable	FAM22A
0.083875716	stable	GABPB2
0.083906501	stable	VPS4B
0.083964439	stable	HERC2P2
0.083964439	stable	SDS
0.084066031	stable	CNTN6
0.084106358	stable	ERV3
0.084152384	stable	TRIM71
0.08420118	stable	LOC646890
0.084383656	stable	BET1L
0.084389054	stable	CRABP2
0.084403363	stable	GALR2
0.084467563	stable	LRRC50
0.084640998	stable	SHRM
0.084658794	stable	CTGF
0.084658794	stable	FAM5C
0.084685195	stable	NT5DC1
0.084732579	stable	TAC3
0.084850632	stable	OAS1
0.084850632	stable	STELLAR
0.084853593	stable	NHEDC2
0.084853593	stable	BAGE3
0.084897617	stable	DGAT2
0.084958614	stable	PRPH2
0.085133933	stable	LOC388789
0.085151279	stable	CTSZ
0.085320031	stable	NOMO1
0.085320031	stable	COX4NB
0.085428876	stable	FARS2
0.085460202	stable	ALG14
0.085523904	stable	FRAT2
0.085565083	stable	ASTN1

0.085603701	stable	ZNF750
0.085630142	stable	CTNND1
0.085641309	stable	C14ORF151
0.085662162	stable	C14ORF173
0.085759128	stable	CUL5
0.085800864	stable	KRT126P
0.085844297	stable	OR11H6
0.085855444	stable	LOC643121
0.085916314	stable	DOCK5
0.086016496	stable	SUMF1
0.086078722	stable	HMHA1
0.086176875	stable	PGM2L1
0.08635706	stable	TMEM39B
0.086466922	stable	TRIP11
0.086486681	stable	TCF3
0.086581311	stable	PDS5B
0.086673896	stable	FXYD4
0.086774559	stable	NKPD1
0.086774559	stable	ACSL3
0.086806766	stable	ATMIN
0.086922343	stable	SCNM1
0.08696133	stable	HCFC2
0.087009602	stable	LOC389435
0.087048554	stable	SYTL3
0.087291735	stable	KIAA1683
0.087372126	stable	STXBP3
0.087461284	stable	C5ORF38
0.08748192	stable	CDH22
0.087556552	stable	LOC653764
0.087561776	stable	IDI1
0.087585721	stable	RWDD1
0.087642664	stable	LOC646254
0.087712097	stable	NSUN5C
0.087792755	stable	C14ORF145
0.087812933	stable	SHBG
0.087812933	stable	RSPH3
0.087897748	stable	F11R
0.087950021	stable	NAT6
0.087962105	stable	LOC643265
0.087962105	stable	CHST9
0.088247577	stable	LOC642486
0.088262451	stable	RSF1
0.088266502	stable	C6ORF105
0.088309418	stable	FBXL17
0.088346903	stable	FLJ20323
0.088489944	stable	NRG2
0.088510629	stable	RPS10
0.088607465	stable	TSP50
0.088623876	stable	LOC643842
0.088702055	stable	C1QL4
0.088728584	stable	LRRK2

0.088770142	stable	SEPT11
0.088837944	stable	SATL1
0.088995199	stable	TPST1
0.089073955	stable	MDGA1
0.089089901	stable	NID2
0.089149163	stable	LOC653275
0.089177486	stable	FAT4
0.08928699	stable	LOC646268
0.08928699	stable	HIP2
0.089328609	stable	LOC728468
0.089332342	stable	IFNAR1
0.089606981	stable	LOC440900
0.089614757	stable	PCDHB19P
0.089675163	stable	FH
0.08993378	stable	DSN1
0.089940781	stable	IGJ
0.089962297	stable	TM7SF4
0.089971753	stable	PHCA
0.090047817	stable	DIP2C
0.090083976	stable	RAB3A
0.090089629	stable	NUPL1
0.09021432	stable	SGK269
0.090254715	stable	SLC22A18AS
0.090340029	stable	PCDHB13
0.090388642	stable	LOC388276
0.090513348	stable	MUC2
0.09051558	stable	ZNF75A
0.09051558	stable	PPP1R13L
0.09051558	stable	TOX
0.090706549	stable	POLL
0.090909222	stable	TRAPPC9
0.090912653	stable	DLG2
0.090982876	stable	GDPD5
0.090982876	stable	ZNF621
0.091015283	stable	LOC653471
0.091032217	stable	PIK3CA
0.091032217	stable	RYR1
0.091032217	stable	LOC145853
0.091041146	stable	ZNF146
0.091082749	stable	PRDM2
0.091132964	stable	ANKRD30B
0.091137259	stable	PLAC4
0.091213336	stable	LOC646562
0.091248378	stable	ARHGEF7
0.091254244	stable	KCTD13
0.091254244	stable	XPNPEP3
0.091282543	stable	FLJ45445
0.091303164	stable	MTERFD3
0.091327841	stable	PDRG1
0.091412598	stable	SLC39A9
0.091476516	stable	UGCGL1

0.091542881	stable	ANKRD2
0.091588295	stable	CDCA4
0.091591586	stable	SLC34A3
0.091613036	stable	FOXI2
0.091618389	stable	FEZF2
0.091647249	stable	RNUXA
0.091715628	stable	FLJ36492
0.091784872	stable	DUSP27
0.09191252	stable	CATSPERB
0.091916131	stable	METTL4
0.092011332	stable	UBOX5
0.092031657	stable	LOC643345
0.092099736	stable	CYLN2
0.092099736	stable	MAPK12
0.092234308	stable	C7ORF28B
0.092260115	stable	SPRR2B
0.092335832	stable	RASGRP3
0.092337373	stable	ZNF548
0.092368445	stable	RNF43
0.092400409	stable	C6ORF224
0.092484718	stable	LMO1
0.092484718	stable	ALDH8A1
0.092648033	stable	COL25A1
0.092666377	stable	C20ORF142
0.09283938	stable	CXORF39
0.09283938	stable	LGALS7
0.092965373	stable	UBQLNL
0.093035454	stable	RPL14
0.093185729	stable	CLCA4
0.093185729	stable	LOC731186
0.093211013	stable	CASP3
0.093285039	stable	ZNF629
0.093285039	stable	TEX13A
0.093348546	stable	CLTC
0.093354637	stable	GDAP2
0.093354637	stable	DCLRE1A
0.093573247	stable	LOC643997
0.093573247	stable	LOC643913
0.093585334	stable	PARP14
0.093585334	stable	FLJ46210
0.093625547	stable	LOC158160
0.093625547	stable	BPNT1
0.093725743	stable	LYSMD1
0.093726594	stable	S1PR5
0.093791702	stable	APEH
0.093828294	stable	PKM2
0.094082267	stable	KIAA0082
0.094133657	stable	C6ORF211
0.094158873	stable	LOC731096
0.094180897	stable	SNAPC4
0.094330483	stable	SENP8

0.094330483	stable	LOC642502
0.094356985	stable	LOC646871
0.094372432	stable	PYCR1
0.094372432	stable	FEN1
0.094386858	stable	LOC652326
0.094404571	stable	ZNF132
0.094448718	stable	LOC646825
0.094454276	stable	NFIB
0.094507409	stable	FRAT1
0.094507409	stable	PPP1R7
0.094624212	stable	ACSM2A
0.094695397	stable	HHATL
0.094720139	stable	LOC400120
0.094773819	stable	ZNF764
0.094775569	stable	ANKRD20A1
0.094848842	stable	LOC651655
0.094891247	stable	NOTUM
0.094934323	stable	LDLRAD3
0.094956706	stable	SERPINB4
0.094961837	stable	LOC152084
0.095136755	stable	RNF123
0.095151535	stable	ZNF521
0.09528172	stable	GVIN1
0.095347544	stable	SSTR5
0.095363325	stable	FLJ32065
0.095363325	stable	TUBGCP4
0.095371987	stable	PTPLAD1
0.095400814	stable	RAET1G
0.095461668	stable	LOC732450
0.095461668	stable	NOL3
0.095461668	stable	MED6
0.095610531	stable	LOC388578
0.095712094	stable	SP140L
0.095712094	stable	ABLIM3
0.095715667	stable	CD99L2
0.095727464	stable	TBC1D24
0.095727464	stable	CGRRF1
0.095751959	stable	LOC652265
0.095838109	stable	CTTN
0.095969434	stable	LOC650006
0.096036002	stable	EGFLAM
0.096167578	stable	IL17RD
0.096256167	stable	SYPL1
0.096280999	stable	CITED1
0.096283493	stable	QPRT
0.09643084	stable	FLJ20489
0.096487643	stable	RGAG1
0.096509915	stable	KRTAP6-2
0.096551799	stable	PKP4
0.09665748	stable	MKI67IP
0.096784447	stable	FLJ40125

0.096787013	stable	ZNF454
0.096787013	stable	LOC645362
0.096821265	stable	LOC440970
0.096826921	stable	LOC642775
0.096844975	stable	SEC24A
0.096905141	stable	ANAPC5
0.097086243	stable	TRIM58
0.097183375	stable	KCNB1
0.097268245	stable	C4B
0.097322545	stable	HNRPA1L-2
0.097345693	stable	LOC648900
0.097355882	stable	KRTAP10-2
0.097370847	stable	OR10H3
0.097384324	stable	ZNF570
0.097386465	stable	TCEAL3
0.097402019	stable	LOC647656
0.097435968	stable	HIST1H2AE
0.097439331	stable	CHST6
0.09745977	stable	LOC646089
0.097485564	stable	LOC113230
0.097534488	stable	TSC22D4
0.097643547	stable	TRIO
0.097643547	stable	BOLA3
0.097669211	stable	NPAS3
0.097800471	stable	RECQL5
0.097844034	stable	BSCL2
0.098079435	stable	SLC13A5
0.098079435	stable	TRPC7
0.098094498	stable	TRIM43
0.098112689	stable	SCLT1
0.098170602	stable	SOX12
0.098239377	stable	EPS8
0.098239377	stable	SOX14
0.098239377	stable	LOC642889
0.09826978	stable	SH3TC2
0.09828667	stable	OS9
0.098354113	stable	LOC728308
0.098368196	stable	LOC339742
0.098377981	stable	MEA1
0.098377981	stable	IFT140
0.098377981	stable	SLC9A6
0.098384113	stable	MAN1B1
0.098581881	stable	ANAPC2
0.098655765	stable	GPR107
0.098696422	stable	TMEM107
0.098735543	stable	CTGLF1
0.098744205	stable	CCDC37
0.098744205	stable	TMEM87B
0.098744205	stable	SLC25A12
0.098861498	stable	LOC644112
0.09888492	stable	SAPS1

0.098898187	stable	LOC643150
0.098960895	stable	ZNF846
0.099006733	stable	NUDT15
0.099079572	stable	LOC728895
0.099220624	stable	C16ORF45
0.099287294	stable	HN1
0.099295743	stable	LOC652246
0.099295743	stable	SLC29A3
0.099340539	stable	CHCHD8
0.099382968	stable	ST5
0.099451188	stable	KRT16
0.099468712	stable	APOB48R
0.099471882	stable	FAM91A1
0.099471882	stable	KIF20B
0.099514505	stable	NAGPA
0.099562326	stable	LRIG1
0.099607089	stable	ISY1
0.099760606	stable	LOC732425
0.099762076	stable	EMILIN2
0.099775027	stable	FERMT1
0.099775027	stable	DNAJB5
0.099791978	stable	LOC646743
0.099792292	stable	LOC401280
0.099792292	stable	DHX8
0.099792292	stable	CT47.7
0.099792292	stable	MLL5
0.099835847	stable	MOSPD3
0.099887797	stable	UNC13A
0.099898217	stable	GSR
0.100090241	stable	MSL3L1
0.100111973	stable	SORT1
0.100273601	stable	COL15A1
0.100273601	stable	RFXAP
0.100283908	stable	GLMN
0.100392686	stable	LOC388969
0.100441596	stable	CCK
0.100637399	stable	HCG18
0.100646481	stable	ZNF295
0.100748095	stable	TTLL3
0.100748095	stable	MTUS1
0.100826346	stable	FIBIN
0.100826346	stable	SEC24C
0.100826346	stable	PRPH
0.100842649	stable	PRKAR1B
0.100868331	stable	ECAT1
0.100924487	stable	PPP4R1
0.100983231	stable	RBPMS
0.101020612	stable	FKBP8
0.101020612	stable	LOC645895
0.101030101	stable	LOC646324
0.101037912	stable	AGPAT4

0.101096874	stable	BAIAP2L2
0.101124598	stable	TRIM38
0.101170949	stable	KCNH6
0.101207068	stable	CPLX2
0.101313492	stable	LOC646519
0.101313492	stable	LRRC37B
0.101329314	stable	C1QTNF5
0.101332886	stable	MYCT1
0.101352998	stable	RFPL4B
0.101429112	stable	LOC441124
0.101473683	stable	PANX2
0.101473683	stable	LOC653741
0.101473683	stable	LOC441584
0.101524228	stable	PTPLB
0.101524228	stable	NEIL1
0.101548824	stable	LOC646210
0.101581276	stable	PRDM6
0.101614866	stable	VEGFC
0.101825606	stable	CGA
0.10192394	stable	LOC646561
0.102392714	stable	CRTC2
0.102540875	stable	CHMP7
0.102601941	stable	PLCG2
0.102602768	stable	LOC647536
0.102719744	stable	C11ORF73
0.102778942	stable	PSORS1C1
0.102819507	stable	FIT1
0.102819507	stable	IL18
0.102849297	stable	KIAA1543
0.1028852	stable	C1ORF112
0.102917713	stable	SIRPG
0.10317081	stable	LOC642342
0.10317081	stable	PGAP1
0.10319964	stable	FLJ21075
0.103349861	stable	ZNF665
0.103363466	stable	ARHGEF2
0.103463168	stable	ZNF511
0.10364301	stable	LOC642302
0.103746526	stable	CASC2
0.103775786	stable	LOC284948
0.103778383	stable	SLC17A7
0.103873474	stable	RFC4
0.10408213	stable	C11ORF39
0.10408213	stable	RBL2
0.10408213	stable	ANKRD36B
0.10408213	stable	LOC653232
0.10408213	stable	CIAPIN1
0.10408213	stable	CHD7
0.104095049	stable	KDELR3
0.104220646	stable	ASAP3
0.104299737	stable	EPC2

0.104299737	stable	AMIGO2
0.104342264	stable	C7ORF27
0.104487222	stable	MT1B
0.104487222	stable	SULF2
0.10452858	stable	MED21
0.104606828	stable	C9ORF163
0.104631328	stable	NCKIPSD
0.104631328	stable	CCDC15
0.104645477	stable	LOC650898
0.104645477	stable	FLJ44606
0.10477632	stable	TRAPPC5
0.104813291	down	HLA-A29.1
0.104898766	stable	FAM177B
0.105046725	stable	ZBTB44
0.105054744	stable	EIF1AX
0.105134098	stable	LOC654202
0.105317342	stable	MLLT6
0.105353592	stable	GOLGA7
0.105508193	stable	VCX-C
0.105508193	stable	BAZ1B
0.105612487	stable	LOC646781
0.105803578	stable	ZACN
0.105856181	stable	TYSND1
0.105864331	stable	HGFAC
0.105953153	stable	NIPSNAP3B
0.105953153	stable	OR51T1
0.105980217	stable	HBB
0.106128116	stable	SF3B3
0.10612819	stable	C20ORF111
0.106175536	stable	LOC154907
0.106175536	stable	BANF1
0.106238539	stable	SIN3A
0.106241717	stable	NR4A2
0.106263459	stable	CCDC78
0.106415691	stable	TNFRSF10D
0.106417341	stable	ELMO2
0.106492801	stable	LOC643714
0.106916195	stable	PTPRQ
0.106916195	stable	NDST4
0.107122725	stable	NFAT5
0.107254635	stable	LOC645893
0.107397472	stable	ETF1
0.107448163	stable	LOC646123
0.107607788	stable	LOC401895
0.107621126	stable	RDH8
0.107624769	stable	LOC653629
0.107751409	stable	ADAM10
0.107810101	stable	CAV2
0.107977148	stable	LUZP4
0.108080569	stable	SEMA3C
0.108236008	stable	PDE10A

0.108320302	stable	ZNF781
0.108359946	stable	DNM2
0.10848328	stable	CD46
0.108496849	stable	LOC652287
0.108867688	stable	C14ORF129
0.108905777	stable	TTLL11
0.108905777	stable	SEDLP
0.109119026	stable	LOC643145
0.109125414	stable	LOC392364
0.109237297	stable	LOC643596
0.109254341	stable	CLDN1
0.109262012	stable	TFAM
0.109262012	stable	RNMTL1
0.109262012	stable	VAMP7
0.10957773	stable	SLC44A1
0.109625826	stable	ZNF524
0.109707127	stable	OR1A1
0.109831906	stable	CCL17
0.109846358	stable	PAF1
0.109849011	stable	IPO5
0.110017059	stable	LOC653204
0.110017059	stable	MGC70863
0.110060394	stable	LOC729264
0.110060394	stable	ZNF434
0.110145003	stable	NPB
0.110145891	stable	LOC653333
0.110193543	stable	CCDC134
0.110249993	stable	ATP6V1B2
0.110397309	stable	LOC653498
0.110447854	stable	GNA14
0.110506449	stable	ZCCHC6
0.110566714	stable	BAI2
0.110642776	stable	ZNF675
0.110650567	stable	ETV7
0.110650567	stable	DNASE1
0.110670923	stable	NIT1
0.110707213	stable	LOC641699
0.110731645	stable	SNORD35A
0.111007657	stable	GPX4
0.111007657	stable	SPA17
0.111023791	stable	ABCC10
0.111106187	stable	NUDT14
0.111106187	stable	SAT2
0.111148025	stable	PARL
0.111148025	stable	EPHA8
0.111239501	stable	KRT72
0.111295287	stable	LOC643262
0.111295287	stable	SYTL1
0.111305188	stable	FAM53B
0.11140591	stable	AMY1B
0.111460933	stable	TTYH1

0.111595994	stable	DNAH9
0.111663303	stable	SMURF2
0.111683111	stable	SLCO1A2
0.111786568	stable	PDCL
0.111980443	stable	HIST1H2BI
0.112013299	stable	CEP152
0.11206917	stable	LOC647839
0.11206917	stable	SFRS17A
0.11206917	stable	THOC7
0.112216557	stable	SNORA32
0.112227039	stable	FZD4
0.11237118	stable	LOC654163
0.112445314	stable	ASXL3
0.112612032	stable	LOC647816
0.112743509	stable	LOC645875
0.11280446	stable	LOC652694
0.112834271	stable	LOC647129
0.112884752	stable	TBC1D14
0.112985734	stable	KIAA1602
0.113056256	stable	LOC441177
0.113072888	stable	SYCE1
0.113198009	stable	GPR114
0.113198009	stable	PAPLN
0.113216956	stable	EFEMP2
0.113244466	stable	LOC90624
0.113428621	stable	LOC643000
0.113448715	stable	LOC650914
0.113453507	stable	LOC651730
0.113563366	stable	ZNF320
0.113610285	stable	LOC642698
0.113626495	stable	ABCA13
0.11365866	stable	PATE
0.113688568	stable	TOMM7
0.113692638	stable	ITPRIPL2
0.113706372	stable	LOC646723
0.114216549	stable	UBE1DC1
0.114249623	stable	SFMBT1
0.114308123	stable	RBED1
0.114360646	stable	LOC145820
0.114474685	stable	CHGA
0.114562646	stable	LOC285047
0.114588254	stable	NRG3
0.114786994	stable	ATP5H
0.114828673	stable	LOC643334
0.114828673	stable	CXCL9
0.114896443	stable	LOC389293
0.114906943	stable	ADIG
0.115032913	stable	SCN3A
0.115032913	stable	LOC650526
0.115032913	stable	CCNF
0.115151728	stable	RPL10A

0.115180072	stable	SPPL2B
0.115190877	stable	LOC650548
0.115316426	stable	TAF5L
0.115316426	stable	PCGF1
0.115395745	stable	ATP13A3
0.115395745	stable	LOC653086
0.115505862	stable	ODF2L
0.11564563	stable	CBWD5
0.11573219	stable	SDR-O
0.115735551	stable	ETV1
0.115758042	stable	C16ORF33
0.115820442	stable	ZNF207
0.115821525	stable	GNGT2
0.11584653	stable	LOC643740
0.115913277	stable	GFRA2
0.115945528	stable	LOC647954
0.116042108	stable	TIPRL
0.116077424	stable	KIAA1279
0.116123715	stable	PARS2
0.116136506	stable	KLHL26
0.116136506	stable	C2ORF32
0.116234525	stable	C20ORF100
0.116290277	stable	SIDT1
0.116431281	stable	PEX3
0.116517521	stable	LOC389983
0.116532435	stable	TSPAN11
0.116534189	stable	SNORD64
0.116610135	stable	CMTM5
0.116662123	stable	LOC728825
0.116662123	stable	LOC646111
0.116767113	stable	RORB
0.116767113	stable	DQX1
0.116767113	stable	C14ORF79
0.116767113	stable	NAE1
0.116799582	stable	FPGT
0.116862607	stable	ADSL
0.116958247	stable	ERBB3
0.117044464	stable	TOX3
0.117081391	stable	C2ORF40
0.117116379	stable	MPFL
0.117216438	stable	PRKG1
0.117406434	stable	ZNF419
0.11746553	stable	LOC441440
0.117481571	stable	SYT5
0.117517332	stable	JARID1C
0.117922654	stable	TCHP
0.118006647	stable	RGP1
0.118033113	stable	MMEL1
0.118060711	stable	PSMD4
0.118060711	stable	MAP1B
0.118060711	stable	LOC649456

0.1181155	stable	SDPR
0.1181155	stable	NAG18
0.1181155	stable	LOC730012
0.118117429	stable	DRAP1
0.118176612	stable	TMEM84
0.118228236	stable	ARL4D
0.118260062	stable	DKFZP586I1420
0.118519898	stable	FLJ43752
0.11860204	stable	LOC729310
0.118671628	stable	RBP4
0.118931114	stable	LRRC31
0.119018212	stable	CNPY4
0.119113821	stable	FLJ45831
0.119155551	stable	LOC644457
0.119179807	stable	ZNF460
0.119197919	stable	LOC643453
0.119305849	stable	LOC554175
0.119305849	stable	DOPEY2
0.119369906	stable	ATP5J
0.11941043	stable	RSBN1
0.119424707	stable	G6PC
0.119428898	stable	METTL8
0.119430662	stable	LOC652204
0.119619829	stable	N4BP2
0.119655713	stable	LOC651986
0.119768857	stable	LOC442382
0.119778308	stable	SIGLEC12
0.119785924	stable	FZD5
0.119785924	stable	NCAPD2
0.119785924	stable	LOC643382
0.119919949	stable	SIX3
0.119976555	stable	LOC441046
0.120000693	stable	UBE2V2
0.120043918	stable	MOSPD1
0.120067204	stable	NUDT18
0.120426036	stable	LOC651850
0.12043648	stable	MX2
0.120449536	stable	RHOC
0.120557332	stable	FCRLB
0.120557332	stable	TSSC1
0.120569894	stable	MSH5
0.120690712	stable	HIST1H2BH
0.120750998	stable	C6ORF54
0.120852999	stable	AGXT2L2
0.12095414	stable	LOC728352
0.121007937	stable	PES1
0.121054514	stable	KRT6B
0.121193878	stable	KIAA0256
0.121352545	stable	MCMDC1
0.121390462	stable	SNORD56B
0.121396686	stable	AKT2

0.121622534	stable	LOC652857
0.121624788	stable	FLYWCH2
0.121658231	stable	P15RS
0.121705016	stable	OR10G7
0.121705016	stable	LOC390259
0.121721508	stable	ZNF35
0.121856082	stable	C18ORF32
0.121963076	stable	LOC441996
0.121991461	stable	FLJ20628
0.12201037	stable	EIF2C4
0.122072764	stable	LOC652046
0.122117499	stable	KRT3
0.122150805	stable	ICT1
0.122166075	stable	HMGB1
0.12232491	stable	CACNA2D4
0.122453672	stable	CTNNB1
0.122546003	stable	SPATA2
0.122546003	stable	ATP6V0D1
0.122596622	stable	MCF2
0.122638447	stable	GOLGA8B
0.122638447	stable	PPIA
0.122660503	stable	C4ORF19
0.122786675	stable	GPT2
0.122927391	stable	GOLGA3
0.122927391	stable	BCS1L
0.123014115	stable	DENND3
0.123091128	stable	PCYOX1L
0.123221565	stable	CMYA3
0.123271641	stable	HP1BP3
0.123583743	stable	ARSK
0.123671104	stable	LOC651745
0.123671104	stable	LOC645364
0.123679974	stable	FAM153B
0.1237766	stable	CAMKK2
0.1237766	stable	PLEKHA3
0.124010029	stable	RPL28
0.124063672	stable	CUTA
0.124096738	stable	F8A3
0.124173857	stable	DCDC2
0.124267355	stable	LOC153684
0.124419428	stable	CSRP1
0.124824414	stable	LOC653197
0.124824414	stable	MX1
0.125006034	stable	TLX2
0.125327092	stable	OR2M7
0.12543442	stable	SAMHD1
0.125523489	stable	D2HGDH
0.125538389	stable	TSHZ1
0.125702206	stable	CACNA1E
0.125702206	stable	RPS9
0.125721133	stable	ZNF572

0.125721133	stable	CLEC9A
0.125725215	stable	VPS25
0.125727956	stable	PFDN5
0.125964278	stable	OR10Z1
0.12603363	stable	ZNF142
0.12614063	stable	LOC643686
0.126170266	stable	LOC645752
0.126197985	stable	CXXC4
0.126233655	stable	BRI3BP
0.126233655	stable	LOC642666
0.126314847	stable	CNFN
0.126491847	stable	LOC645535
0.126526113	stable	COX6BP1
0.126582858	stable	ZNF791
0.126626152	stable	PIGB
0.126671229	stable	TMEM48
0.126671229	stable	BAT2
0.12678834	stable	AMZ1
0.126827023	stable	NKRF
0.126981976	stable	CC2D2B
0.127053443	stable	C11ORF80
0.127178363	stable	RPL39
0.127178363	stable	TSPAN7
0.127178694	stable	LOC643430
0.127264172	stable	MARCH8
0.127264172	stable	RAB42
0.127264172	stable	KIAA1033
0.12727106	stable	AP2A1
0.127275927	stable	NPAS2
0.127351197	stable	MADCAM1
0.127380758	stable	EIF3IP1
0.127443078	stable	SLC2A13
0.127463561	stable	FAM114A2
0.127467624	stable	FASTKD2
0.127591067	stable	LOC646585
0.127592389	stable	SASH1
0.127686768	stable	SLC4A8
0.127835322	stable	PRPSAP1
0.127835322	stable	SFRS8
0.127835322	stable	TMEM77
0.127878075	stable	LOC388237
0.127878075	stable	ATP6V0B
0.127878075	stable	LOC286208
0.127933183	stable	PPIH
0.128015791	stable	TMEM176B
0.128035904	stable	APOA4
0.128097648	stable	LOC440055
0.128224352	stable	ARSJ
0.128224352	stable	CD99
0.128224352	stable	EXOD1
0.128325785	stable	TMEM117

0.128420279	stable	C20ORF86
0.128471113	stable	LOC731102
0.128568731	stable	C6ORF114
0.128580159	stable	LOC649639
0.128899567	stable	TOLLIP
0.129026587	stable	FAM103A1
0.129242842	stable	SLC12A6
0.129242842	stable	CCDC124
0.129329485	stable	FLJ10490
0.129464214	stable	PHF6
0.129504778	stable	TRAM1L1
0.129659731	stable	TFIP11
0.129693374	stable	LOC654103
0.129693374	stable	PRY
0.129693374	stable	ARMCX4
0.129841521	stable	NSUN2
0.129841933	stable	SNX17
0.129899122	stable	HMGN3
0.130027316	stable	OR10A5
0.130468512	stable	CPNE6
0.130555296	stable	LOC642953
0.130591392	stable	THAP3
0.130977037	stable	C16ORF14
0.130978308	stable	LOC643138
0.13102998	stable	TAS2R38
0.131054517	stable	CD207
0.131066436	stable	RNF4
0.131349089	stable	MAN2A1
0.131399149	stable	CCDC24
0.131470073	stable	LOC645602
0.131562992	stable	FAM39DP
0.131562992	stable	HLA-DPB1
0.131578102	stable	CRYL1
0.131630665	stable	FAM131B
0.131630665	stable	KHK
0.13168308	stable	GAS2L2
0.131796721	stable	OR5M10
0.131820345	stable	FSTL1
0.131820345	stable	C21ORF42
0.131835383	stable	GALNT10
0.132360022	stable	LOC126536
0.13238925	stable	C20ORF20
0.13238925	stable	FLJ41170
0.132390836	stable	NPC2
0.132466134	stable	LOC647006
0.132472744	stable	PXMP2
0.132580177	stable	PPIL6
0.132630692	stable	ESPNL
0.132667521	stable	KRT17
0.132667521	stable	LOC440737
0.132781195	stable	LOC643951

0.133036292	stable	STH
0.133036292	stable	LOC650321
0.133369306	stable	COX5A
0.133445769	stable	ZNF574
0.133445769	stable	LMF2
0.133499347	stable	C19ORF2
0.133574366	stable	ZNF771
0.133594083	stable	LOC652586
0.133594083	stable	LOC644094
0.133745437	stable	MTERFD2
0.133745437	stable	MS4A8B
0.133890624	stable	LOC653689
0.133894541	stable	SAMD9
0.134144302	stable	NELL2
0.134147497	stable	CDC20
0.134556888	stable	LBX1
0.134606642	stable	LOC653643
0.134606642	stable	LIMS2
0.134713315	stable	CLCN5
0.134755585	stable	CCDC40
0.134976403	stable	ITGA8
0.135017761	stable	LOC645914
0.135052019	stable	C17ORF59
0.135115216	stable	ZFPL1
0.135251935	stable	LOC641804
0.135251935	stable	LOC647691
0.135288836	stable	LOC375010
0.135429726	stable	VPS37A
0.135429726	stable	ART4
0.135429726	stable	LOC641741
0.135429726	stable	ARL14
0.135429726	stable	SEZ6L2
0.135429726	stable	WDR85
0.135578214	stable	RABL5
0.13560078	stable	LOC728248
0.135669045	stable	LOC644744
0.136023216	stable	LOC644624
0.136037507	stable	SFPQ
0.136084894	stable	YME1L1
0.136208034	stable	MAGED1
0.136290376	stable	NUDT21
0.136452385	stable	LOC652814
0.136551332	stable	OPN1SW
0.136560315	stable	KPNA3
0.136571331	stable	MRPS28
0.136589647	stable	GAA
0.136589647	stable	C9ORF130
0.136590657	stable	LMO3
0.136590657	stable	ARFGEF2
0.136653921	stable	SSBP3
0.136740075	stable	CCDC72

0.136819742	stable	ADCY7
0.136846653	stable	TTC24
0.137055705	stable	CD164
0.137212235	stable	ETS1
0.137422633	stable	PDE9A
0.13751862	stable	C19ORF31
0.137642256	stable	LRIG2
0.137659508	stable	RHOF
0.137669561	stable	TMEM68
0.137855953	stable	TRIM36
0.137900365	stable	MRPL24
0.137997265	stable	SEPX1
0.138110101	stable	ADCYAP1R1
0.138165642	stable	POU5F1
0.138289087	stable	LOC643817
0.138292157	stable	CAPN14
0.138352519	stable	INO80B
0.13836888	stable	LOC641815
0.13836888	stable	NEB
0.138407622	stable	PTMA
0.138467014	stable	METRNL
0.138625473	stable	DNAH10
0.138655366	stable	FLJ32679
0.138655366	stable	LOC402160
0.138855388	stable	LOC642432
0.138874607	stable	FLJ22662
0.13889635	stable	NSBP1
0.138939914	stable	CD247
0.138985286	stable	CTSO
0.138985286	stable	HAGHL
0.139100287	stable	LOC441873
0.139116498	stable	CIDEA
0.139173688	stable	SLC30A7
0.139181851	stable	SAE1
0.139584165	stable	SCGB1D4
0.139627201	stable	DAPK2
0.139781523	stable	SNX27
0.139915201	stable	ACVR1
0.139915201	stable	BRIP1
0.13992732	stable	C9ORF64
0.140042743	stable	SYMPK
0.140074149	stable	CSNK2A1
0.140121816	stable	FBXW9
0.140267273	stable	NDUFA5
0.140267273	stable	EPGN
0.140295471	stable	NHS
0.140295471	stable	RBM18
0.140295471	stable	SNORA66
0.140362023	stable	ZMAT3
0.140800958	stable	CNTN5
0.140959893	stable	VPS28

0.141058044	stable	EYA3
0.141058044	stable	LOC646358
0.141192611	stable	NUP54
0.141249828	stable	LOC285941
0.141258094	stable	LOC643687
0.141276394	stable	IL9R
0.141331239	stable	LOC653145
0.141384348	stable	HIPK3
0.141422545	stable	C21ORF45
0.141632131	stable	NR1H3
0.141705572	stable	C14ORF4
0.141708248	stable	IL25
0.141754746	stable	LOC389895
0.141792648	stable	GEMIN6
0.141869017	stable	LOC730740
0.141869017	stable	BTN3A2
0.141869017	stable	OR2L3
0.141976777	stable	SCN10A
0.142009397	stable	WISP2
0.142097482	stable	LOC644276
0.142097482	stable	KEAP1
0.142097482	stable	ADAMTS8
0.142207518	stable	LOC652152
0.142351388	stable	SMN1
0.142499685	stable	LOC400304
0.142499685	stable	CGGBP1
0.142499685	stable	FBXO10
0.142586156	stable	CGB7
0.142624994	stable	KIR2DL1
0.142678477	stable	ZNF721
0.142678477	stable	LOC643329
0.142843781	stable	VAV3
0.142948931	stable	SEPT4
0.143058889	stable	SMARCAL1
0.143065254	stable	LOC653886
0.143087108	stable	AHSA1
0.14311042	stable	ATOH1
0.143188712	stable	ENTPD3
0.14327241	stable	ST7L
0.14363913	stable	LOC654000
0.143645962	stable	LRRC67
0.143733307	stable	MSLN
0.143771749	stable	SUMO1
0.143776305	stable	C1ORF102
0.143874222	stable	PSMB5
0.143874222	stable	AUTS2
0.143905095	stable	WDSOF1
0.144104034	stable	COL4A2
0.144116835	stable	STAM2
0.144131741	stable	ZNF280D
0.14415209	stable	ATP2B4

0.144188459	stable	FLJ43763
0.144303063	stable	CARD9
0.144450592	stable	LOC646129
0.144526347	stable	LGALS9B
0.144630121	stable	LOC727927
0.144630121	stable	RBMY2EP
0.144639155	stable	MGC52000
0.144777447	stable	FLJ21865
0.144871421	stable	ALDH9A1
0.144880231	stable	LOC653107
0.145101252	stable	MED13
0.145337536	stable	C6ORF192
0.145364966	stable	TSPYL5
0.14549473	stable	GRIN2D
0.145632142	stable	NCF1B
0.145926935	stable	FOXM1
0.146002095	stable	CLN6
0.14600616	stable	OR2A2
0.146106269	stable	LOC650325
0.146391883	stable	RPLP0P2
0.146431693	stable	TMEM31
0.146431693	stable	LOC642821
0.146433657	stable	PFKP
0.146485973	stable	ARID3A
0.146485973	stable	ZNF818
0.146580283	stable	DCHS1
0.146580283	stable	RBMX
0.146818785	stable	ZDHHC13
0.146914509	stable	GLB1L
0.146966982	stable	RSU1
0.147139337	stable	USP41
0.147261824	stable	LOC645927
0.147261824	stable	THSD1P
0.147261824	stable	LOC642757
0.147261824	stable	GPR50
0.147261824	stable	TSPY1
0.147284651	stable	C9ORF96
0.147349841	stable	MGC57346
0.147351096	stable	LRRC44
0.147504677	stable	TRIM63
0.147685425	stable	LOC728005
0.147708317	stable	CDK9
0.147874993	stable	SEC14L4
0.147913896	stable	CYYR1
0.147977777	stable	CHRFAM7A
0.148112193	stable	PNPLA8
0.148260436	stable	TOM1L1
0.148344202	stable	TCP10L
0.148495215	stable	AAK1
0.148558754	stable	PRODH2
0.148615559	stable	GABRP

0.148719532	stable	MINPP1
0.148722929	stable	PDCD5
0.148760622	stable	CNOT3
0.148778323	stable	NOL7
0.148831734	stable	SUSD4
0.148883252	stable	LOC643109
0.149025425	stable	SERINC3
0.149025425	stable	SALL1
0.149025425	stable	HES5
0.149252861	stable	ALKBH8
0.149291268	stable	TBX4
0.149291268	stable	C19ORF6
0.149347889	stable	RCAN2
0.149356057	stable	EXOSC7
0.149382394	stable	POP1
0.14962464	stable	LOC390712
0.149702886	stable	HYDIN
0.149702886	stable	LOC645351
0.149710317	stable	ACSL1
0.149849015	stable	LOC644403
0.150068349	stable	KIN
0.150150077	stable	ZNF28
0.150156817	stable	KIAA1377
0.150214016	stable	LOC652565
0.150308543	stable	LASS4
0.150344595	stable	NRXN2
0.150344595	stable	DENND2A
0.150344595	stable	BRUNOL6
0.150349697	stable	DGCR2
0.150349697	stable	LOC643222
0.150349697	stable	OR6W1P
0.150469208	stable	DMRT3
0.150471477	stable	MESDC2
0.150474603	stable	ATF1
0.150474603	stable	APBB1
0.150474603	stable	EPHB3
0.150474603	stable	KRTAP10-4
0.150474603	stable	CEP70
0.150534887	stable	NKAPL
0.150534887	stable	C1ORF89
0.150552566	stable	HTR6
0.150604102	stable	PELI3
0.150699128	stable	MS4A13
0.150809095	stable	SLCO1C1
0.150936996	stable	ZNF559
0.151034686	stable	DGKQ
0.151054407	stable	CDC73
0.151190962	stable	ITIH2
0.151213978	stable	FLJ33544
0.151274912	stable	SGSM3
0.151357131	stable	LOC645848

0.151363201	stable	PDP2
0.151572957	stable	LOC647571
0.151648753	stable	C10ORF73
0.151697309	stable	LOC256374
0.151751921	stable	LOC730456
0.151751921	stable	NTRK1
0.151841182	stable	SERPINB6
0.151841182	stable	CCDC18
0.151841182	stable	GK2
0.151841182	stable	LOC729047
0.15185354	stable	SLC6A5
0.151885144	stable	GORASP2
0.151885144	stable	ALKBH6
0.151996584	stable	EFCBP1
0.152025336	stable	KIR2DS3
0.152025336	stable	EIF2B5
0.1520721	stable	LOC647340
0.152074602	stable	GTF2H4
0.152097687	stable	FAM29A
0.152243505	stable	LOC646817
0.15227071	stable	MAN2B2
0.152329647	stable	RSPRY1
0.152510486	stable	ZNF212
0.152632809	stable	LOC647974
0.152797119	stable	C10ORF53
0.15282562	stable	CXORF40B
0.15289188	stable	C15ORF29
0.152997299	stable	DHRS9
0.153104633	stable	NLRP14
0.153104633	stable	FAM86C
0.153277449	stable	CPXCR1
0.153372793	stable	HIST2H2AB
0.153388262	stable	CCDC44
0.153427384	stable	SLC9A7
0.153487648	stable	ZUFSP
0.153508583	stable	C1ORF86
0.153544441	stable	BCAS2
0.153737664	stable	ERAL1
0.15376248	stable	PIAS3
0.153874251	stable	KIRREL3
0.153947625	stable	LOC285733
0.153947625	stable	ZNF483
0.153947625	stable	LOC729715
0.153947625	stable	ATG16L2
0.154115815	stable	LOC645218
0.154115815	stable	LOC442251
0.154243696	stable	ABCC13
0.154354998	stable	FLJ34503
0.154401107	stable	PPAPDC1B
0.154428632	stable	LOC649923
0.154531545	stable	ZNF222

0.154556686	stable	ABCC5
0.154674587	stable	ZFP64
0.154696518	stable	DNAJA3
0.154737859	stable	LOC645048
0.154766425	stable	LOC730262
0.154777268	stable	RNF128
0.154881384	stable	FLJ10916
0.154947598	stable	GNE
0.155008733	stable	RBMY1E
0.1550279	stable	KIAA1539
0.155143596	stable	C16ORF84
0.155143596	stable	PPFIA1
0.15524969	stable	LOC652637
0.155263458	stable	EYA2
0.155351723	stable	LOC142937
0.155495882	stable	CELSR2
0.155682631	stable	LOC642602
0.155683226	stable	SLITRK6
0.155797451	stable	SKIV2L
0.155931284	stable	CEP27
0.155985884	stable	WDR51B
0.15599969	stable	C10ORF116
0.156179647	stable	P76
0.156182541	stable	AMBN
0.156295194	stable	KRI1
0.156295194	stable	LOC283932
0.156295194	stable	LOC646009
0.156381296	stable	CCR3
0.15652216	stable	PRR19
0.156833501	stable	SERPINB2
0.156955562	stable	LOC644026
0.156955562	stable	C11ORF57
0.15713117	stable	LOC387820
0.157150666	stable	GALNT1
0.157217283	stable	CD300LF
0.157246369	stable	GPR108
0.157271562	stable	ARL16
0.157282915	stable	H1FX
0.157545046	stable	OR51V1
0.157569778	stable	KIAA2018
0.157821889	stable	ANKS1B
0.15789393	stable	FTSJ2
0.15789393	stable	ALPP
0.157949293	stable	MPPED1
0.157985346	stable	PRKAR2A
0.157985346	stable	RAD50
0.157985346	stable	CROT
0.158023646	stable	RBL1
0.158023646	stable	RFC2
0.158191277	stable	LOC642809
0.15826408	stable	PRKD2

0.158323339	stable	LOC401068
0.158375511	stable	TMEM30A
0.158381308	stable	OR4F5
0.158384578	stable	LOC644413
0.158472615	stable	MRPS30
0.158506885	stable	RPL21
0.158556953	stable	MET
0.158556953	stable	ERC1
0.158607141	stable	RC3H2
0.158764399	stable	HYAL4
0.158805237	stable	RPL31
0.159004451	stable	PQLC3
0.159052341	stable	TYW1
0.159132596	stable	RSHL3
0.159173411	stable	SMEK3P
0.159222944	stable	PEX10
0.159228852	stable	HRG
0.159270042	stable	SUZ12P
0.159362687	stable	LOC649260
0.159367134	stable	DPT
0.159428334	stable	FAM177A1
0.159428334	stable	DMRTA1
0.159428334	stable	LOC642104
0.159484169	stable	NECAB2
0.159509966	stable	LOC732172
0.159509966	stable	FAM169B
0.159593595	stable	LUZP5
0.159737185	stable	LOC391092
0.159740539	stable	LOC652616
0.159785147	stable	HACE1
0.159785147	stable	LOC652344
0.159885674	stable	LOC647200
0.159891402	stable	IER5L
0.159891402	stable	LOC132706
0.160197389	stable	LOC651017
0.160226572	stable	MRAP
0.16028269	stable	LOC645336
0.160463908	stable	FLJ11151
0.160474761	stable	FKBP1B
0.160508438	stable	LOC651686
0.160590496	stable	CCDC84
0.160683347	stable	POLR2K
0.160704693	stable	LOC388564
0.160807357	stable	LOC641992
0.160942689	stable	ZNF3
0.160942689	stable	GRIK4
0.161004186	stable	H19
0.161033189	stable	SLIT3
0.16105001	stable	BOAT
0.161065286	stable	RTP1
0.161126486	stable	STRN3

0.161126486	stable	SS18L1
0.161460012	stable	WFDC11
0.161466107	stable	PLXNA4B
0.161468147	stable	PPP1R16A
0.161468147	stable	KANK2
0.161588952	stable	MRGPRX1
0.16167371	stable	TREM1
0.161697684	stable	FLJ45256
0.161700042	stable	IZUMO1
0.161703914	stable	LOC643550
0.161751599	stable	LOC653238
0.161797193	stable	FTHL8
0.161838893	stable	UBXN11
0.162075974	stable	FLJ10324
0.162175196	stable	SULT1C3
0.162193978	stable	TNKS
0.162201842	stable	LOC642212
0.162251529	stable	DEFB131
0.162296819	stable	CPLX1
0.162296819	stable	LOC731432
0.162380113	stable	C1ORF132
0.162478198	stable	ANXA4
0.162667906	stable	LOC645968
0.16281075	stable	ATP2B1
0.162860116	stable	ALDH1A1
0.162959564	stable	DUXAP3
0.162960929	stable	LOC645197
0.162980129	stable	KIAA1622
0.163060301	stable	KITLG
0.163060301	stable	CCDC144A
0.163133877	stable	VPS18
0.163155272	stable	GLRA1
0.163155272	stable	BAT4
0.163182012	stable	FAM149A
0.163185634	stable	LOC645436
0.163185634	stable	LOC642424
0.163222121	stable	SNORD116-2
0.163434812	stable	VLDLR
0.163477978	stable	HES1
0.163799224	stable	CAPZA1
0.163799224	stable	USP6NL
0.163799224	stable	LOC344382
0.16388575	stable	COQ10B
0.16395923	stable	C15ORF33
0.164014008	stable	SLC10A3
0.164092773	stable	LOC652783
0.16431044	stable	LCT
0.164390909	stable	MAGEB4
0.164390909	stable	LOC653564
0.164390909	stable	PRIM1
0.164419032	stable	LOC645367

0.164427229	stable	AHR
0.164427229	stable	LOC653034
0.16450525	stable	LOC649987
0.164587707	stable	LOC644577
0.164748124	stable	LOC644343
0.164790066	stable	PRDM5
0.164964632	stable	VCX
0.165037707	stable	LOC388494
0.165123154	stable	MTM1
0.165139619	stable	S1PR2
0.165172943	stable	LOC641806
0.165187045	stable	HEY1
0.165201638	stable	GINS2
0.165586275	stable	TSSK1B
0.165586275	stable	GIYD2
0.165586275	stable	LAMA3
0.165586275	stable	HOXC4
0.165586275	stable	LOC650137
0.165586275	stable	WDR25
0.165586275	stable	GCDH
0.165594351	stable	FOXI1
0.165622345	stable	SYNPO
0.165909247	stable	EPHA7
0.165965959	stable	SPPL3
0.166155076	stable	ATP6V0E1
0.166162464	stable	LOC255809
0.166298828	stable	LONRF3
0.166299669	stable	ARF5
0.16630365	stable	C10ORF115
0.166536621	stable	LOC649632
0.166633535	stable	KCNMB4
0.166797782	stable	GNLY
0.167130026	stable	LOC645582
0.167130026	stable	OR2J3
0.167200748	stable	LOC646783
0.167200748	stable	AFG3L2
0.167551409	stable	LOC652373
0.167961288	stable	RIT1
0.168051643	stable	CRB3
0.168066648	stable	ZC3H18
0.168149243	stable	LOC441655
0.168180884	stable	TBC1D8B
0.168218259	stable	NKX6-3
0.168219109	stable	MATN1
0.168219109	stable	SAMD1
0.168267786	stable	LOC441511
0.168288935	stable	DHRS4
0.168298768	stable	PIAS1
0.168483836	stable	ATP11B
0.168522058	stable	FLJ46380
0.168523923	stable	HRH4

0.168579775	stable	RRAD
0.168621406	stable	LOC643002
0.168830067	stable	DKFZP761E198
0.168854059	stable	SLC25A26
0.168978279	stable	MAPKAPK3
0.169039295	stable	COPS2
0.169125926	stable	LASS5
0.169125926	stable	LOC643043
0.169202358	stable	DSCR6
0.169230048	stable	CD1B
0.169245987	stable	OR9G4
0.16930678	stable	MOBP
0.169474699	stable	LOC647384
0.169490758	stable	LOC650363
0.169683972	stable	LOC645669
0.169683972	stable	LOC646746
0.169845699	stable	FLJ26850
0.169868663	stable	TRIM50
0.169868663	stable	SPRR4
0.169934496	stable	ILK
0.169947028	stable	PPID
0.169995658	stable	GALNS
0.170081324	stable	CCDC65
0.170260967	stable	ZC3H10
0.170374247	stable	PELI2
0.170381702	stable	FLJ45224
0.170440714	stable	WDR20
0.170631164	stable	LOC554223
0.170772039	stable	FLJ42291
0.170820953	stable	LOC284064
0.170894883	stable	ZXDC
0.170947813	stable	LOC402508
0.171096511	stable	C21ORF67
0.171096511	stable	NKAP
0.171120715	stable	WHDC1L1
0.171186809	stable	SNX5
0.171305606	stable	C4ORF44
0.171309546	stable	CPZ
0.171365572	stable	LOC387941
0.171439231	stable	LOC645711
0.171439231	stable	DGCR5
0.171657942	stable	CNOT6L
0.171710447	stable	CA12
0.171890812	stable	LOC338797
0.171890812	stable	CAMSAP1
0.171890812	stable	LOC148766
0.171890812	stable	FABP7
0.171890812	stable	LOC646309
0.171890812	stable	SLC34A2
0.171890812	stable	PDDC1
0.171927242	stable	GRIA2

0.172039663	stable	CEP250
0.172064066	stable	RNF175
0.172101492	stable	TMC7
0.172158068	stable	INO80C
0.172171437	stable	GOSR2
0.17217705	stable	INTS4
0.172268172	stable	NRIP2
0.172299017	stable	CXORF1
0.172299017	stable	TLE6
0.172385608	stable	ANKRD55
0.172489183	stable	HMG2L1
0.172505906	stable	TBC1D3G
0.172545321	stable	DSCR3
0.172545321	stable	DMC1
0.172755148	stable	C18ORF2
0.172822528	stable	RNF26
0.172936503	stable	LOC284821
0.173186799	stable	ZDHHC21
0.173186799	stable	PRAMEF16
0.173186799	stable	PHLDB1
0.173241327	stable	RCL1
0.173501162	stable	CASC1
0.173517013	stable	C6ORF160
0.173517013	stable	CBLN2
0.173517013	stable	SMC5
0.173555155	stable	SDHALP1
0.173604319	stable	KIF21B
0.173627693	stable	IGSF2
0.173748624	stable	BXDC5
0.173816524	stable	WDR22
0.173816524	stable	LOC120376
0.173829277	stable	SPRN
0.173851021	stable	C15ORF56
0.173855043	stable	LOC402694
0.174124395	stable	FAM21A
0.174124395	stable	CDA
0.174164158	stable	PCDHGC4
0.174267511	stable	C20ORF135
0.174267511	stable	TMEM188
0.174267511	stable	RPS6KC1
0.174417027	stable	INTS1
0.1746061	stable	CAPN11
0.174608823	stable	GTPBP4
0.17461805	stable	LOC400968
0.17461805	stable	QRICH1
0.174679889	stable	FAM19A4
0.174679889	stable	RBM7
0.174679889	stable	ANXA9
0.174711986	stable	CSNK1G1
0.174775389	stable	MRPL12
0.174855127	stable	CLRN1

0.174855127	stable	PTCD3
0.175149899	stable	HNRNPC
0.175164788	stable	SRF
0.175164788	stable	PITPNA
0.175164788	stable	B4GALT6
0.175226947	stable	INTS10
0.175226947	stable	BAALC
0.175271621	stable	LOC652392
0.175311583	stable	SHANK1
0.175407812	stable	LOC648638
0.175518574	stable	MTHFD1
0.17559695	stable	LOC641772
0.175620534	stable	KRTAP10-7
0.175649516	stable	CWC15
0.175649516	stable	RPA1
0.175837352	stable	CUGBP2
0.175890088	stable	RPPH1
0.175890088	stable	LOC255374
0.175930708	stable	DBR1
0.175930708	stable	LOC649404
0.175960977	stable	LOC552891
0.176009236	stable	COG2
0.176009236	stable	ZNF324B
0.176065481	stable	AAAS
0.176182517	stable	ITGA1
0.176222213	stable	C7ORF11
0.176308711	stable	NRAP
0.176425131	stable	PPIL5
0.176521299	stable	LOC647103
0.176738644	stable	PRSS8
0.176738644	stable	RNF44
0.176738644	stable	LOC200420
0.176738644	stable	CDH1
0.176852995	stable	LOC647919
0.176895076	stable	GRIK3
0.176908806	stable	LOC653127
0.17701107	stable	LRRC15
0.177078959	stable	VPS72
0.177228709	stable	SNX14
0.177234276	stable	CACNA1D
0.177454485	stable	LOC647121
0.177704075	stable	CLEC3B
0.177704075	stable	LOC652251
0.177704075	stable	DUX4C
0.177863189	stable	NUP107
0.177876312	stable	TESC
0.177913599	stable	EXOC3L
0.177936388	stable	HYMAI
0.177950973	stable	LOC401915
0.177962412	stable	CXORF24
0.177962412	stable	LOC650008

0.177962412	stable	SULT1A1
0.178126664	stable	SCNN1B
0.178225645	stable	VAPA
0.178225645	stable	FLJ40722
0.178451809	stable	LOC390378
0.178451809	stable	SIGLEC11
0.178754253	stable	TUFM
0.1787553	stable	POLR2J
0.178856147	stable	H2BFWT
0.178877424	stable	DNASE1L1
0.178938036	stable	NBPF3
0.179006273	stable	ZNF568
0.179068607	stable	FGF2
0.179068607	stable	HSPE1
0.179124154	stable	CSMD1
0.179154522	stable	CHES1
0.179392076	stable	RGNEF
0.17963131	stable	SNX33
0.179634141	stable	TBC1D22B
0.179686549	stable	LOC642154
0.179893529	stable	GAL3ST4
0.179937542	stable	ZNF414
0.179947038	stable	ATG9A
0.179963464	stable	WWOX
0.179963464	stable	FAM43A
0.179977182	stable	LOC730426
0.179977182	stable	LOC644655
0.180158512	stable	LOC91661
0.180260989	stable	HSH2D
0.180301393	stable	SEPT10
0.18030785	stable	C14ORF126
0.18030785	stable	LOC143666
0.18030785	stable	LOC645032
0.180314196	stable	NFIX
0.180353921	stable	FTHL3P
0.180353921	stable	WNT5A
0.180357202	stable	OR6N2
0.180408169	stable	ATXN1
0.18042582	stable	SFT2D3
0.180573357	stable	LOC648851
0.180573357	stable	FAM7A1
0.180682162	stable	LOC642387
0.180760208	stable	C3ORF62
0.180760208	stable	SPON1
0.180777258	stable	PLAC8L1
0.180828269	stable	CAMK2N1
0.180959007	stable	GBA3
0.180968956	stable	SNORD25
0.180968956	stable	SNORA25
0.180970927	stable	RPL35
0.180985947	stable	DYNLL2

0.181022054	stable	PLA2R1
0.181022054	stable	APH1A
0.18105029	stable	PLSCR2
0.18105029	stable	ADAMTSL4
0.181130266	stable	UBE2N
0.181177101	stable	HCG27
0.181200816	stable	STAT5B
0.181560177	stable	LOC390367
0.181725482	stable	NHEJ1
0.181747115	stable	DSE
0.181835117	stable	FAM5B
0.181846388	stable	LOC647166
0.181872016	stable	LOC646507
0.18195791	stable	CLCN3
0.18195791	stable	CNTN2
0.18198699	stable	PADI3
0.18198699	stable	LOC642855
0.182280856	stable	WDR89
0.182280856	stable	MTIF3
0.182286134	stable	ZFP28
0.182713439	stable	GPHB5
0.182713439	stable	GALNT3
0.182744697	stable	C10ORF12
0.182819978	stable	LOC653766
0.182926452	stable	PIWIL2
0.18311247	stable	UBR4
0.183302724	stable	FLJ10357
0.183302724	stable	FASTKD5
0.183321695	stable	GTF2IRD2B
0.183403575	stable	LOC390466
0.183524908	stable	FLJ43980
0.183568815	stable	LEREPO4
0.183692896	stable	LOC652126
0.183713262	stable	DUOXA2
0.183716122	stable	C8ORF22
0.183794715	stable	PDE8A
0.183794715	stable	TRPM4
0.183960314	stable	COQ2
0.184003961	stable	TDRD7
0.184126219	stable	C13ORF18
0.184176564	stable	LDHC
0.184176564	stable	CDH17
0.184176564	stable	TUSC5
0.184254538	stable	ACSL6
0.184414292	stable	LIN37
0.184453121	stable	USP53
0.184453121	stable	RPS15A
0.184453121	stable	C6ORF103
0.184453121	stable	GRIA1
0.184617782	stable	TELO2
0.184751634	stable	SLC17A4

0.184803438	stable	SLC7A10
0.184882721	stable	KRTAP13-3
0.184891117	stable	QSER1
0.184981868	stable	SLC17A2
0.184981868	stable	BHLHB5
0.185042849	stable	RFXDC1
0.185159536	stable	LOC651556
0.185164558	stable	VSIG1
0.185170935	stable	EPN3
0.185342478	stable	ARHGEF10
0.185612117	stable	CAPN12
0.185809294	stable	FBXO27
0.185863812	stable	PLCL2
0.186111779	stable	HMG1L1
0.186199412	stable	LOC644227
0.186269447	stable	LOC642669
0.186426453	stable	DEPDC4
0.186432707	stable	GNG5
0.186505596	stable	C10ORF132
0.186549144	stable	CBLN1
0.186549144	stable	LOC400506
0.186706173	stable	LOC642282
0.186725964	stable	PNKP
0.186747382	stable	CSTF2T
0.186757697	stable	RNF121
0.186757697	stable	LOC649957
0.186814669	stable	HSP90AA1
0.186842767	stable	CST9
0.186904788	stable	LOC652517
0.186923554	stable	LOC648415
0.186923554	stable	TMEM148
0.187003469	stable	NRSN1
0.187214108	stable	TNXA
0.18728528	stable	LOC645312
0.187449635	stable	LOC648997
0.187479757	stable	USMG5
0.187479757	stable	SLC35B4
0.187615772	stable	LOC647104
0.187615772	stable	LOC652534
0.18766756	stable	LOC652704
0.18766756	stable	PIGF
0.187715206	stable	RAB3IP
0.187768913	stable	LOC729021
0.187825697	stable	SUPV3L1
0.187838897	stable	RBM44
0.187862685	stable	CORT
0.187862685	stable	LOC391811
0.187911822	stable	C14ORF143
0.187953804	stable	LOC389748
0.188001582	stable	RAD52
0.188033804	stable	SYT7

0.188102196	stable	GTSF1
0.18819818	stable	LTB
0.188244704	stable	HOXA2
0.188276932	stable	IL3
0.188551152	stable	STT3B
0.188551152	stable	ETV5
0.188551152	stable	LOC647592
0.188669832	stable	C6ORF106
0.188753936	stable	OR2D2
0.18882171	stable	TEKT4
0.188872646	stable	LDOC1
0.188877821	stable	CDC42EP3
0.188877821	stable	PGM1
0.189134877	stable	VN1R1
0.189257444	stable	MARS2
0.189257444	stable	RPL24
0.189336852	stable	ZNF682
0.189336852	stable	TRMT6
0.189336852	stable	TNFSF9
0.189355222	stable	RDH13
0.189355222	stable	LOC646266
0.189355222	stable	RFP
0.189355222	stable	LOC653778
0.189362325	stable	DNAJB14
0.189362325	stable	LOC285382
0.189362325	stable	LOC441582
0.18951272	stable	TEKT3
0.18951272	stable	MFSD11
0.189643239	stable	LOC642321
0.189733845	stable	UBA6
0.189733845	stable	ZSWIM2
0.189838246	stable	PI4K2A
0.189860934	stable	LOC643201
0.189929156	stable	LOC402251
0.190013823	stable	CABP7
0.190038048	stable	DEK
0.190091237	stable	APOC3
0.190396588	stable	ASMT
0.190439139	stable	LIF
0.190693688	stable	LOC644979
0.190849532	stable	OR13C8
0.190884098	stable	NXF5
0.190970252	stable	ADCYAP1
0.19098404	stable	LOC647778
0.191074993	stable	LOC652898
0.191077564	stable	ANP32B
0.191153121	stable	LOC643176
0.191282431	stable	DENND2C
0.191399361	stable	TTK
0.191446401	stable	GPR42
0.191446401	stable	NOTCH2

0.191491537	stable	PON1
0.19153569	stable	C15ORF44
0.191673159	stable	RPH3A
0.191711555	stable	SLC22A17
0.191766952	stable	FAM24A
0.191766952	stable	TMEM16B
0.191766952	stable	OR6C76
0.19183959	stable	DCT
0.192000317	stable	SNORD83A
0.192182698	stable	ME3
0.192525786	stable	TSTA3
0.192525786	stable	ELF5
0.192525798	stable	FAM109A
0.192525798	stable	ZNF681
0.192834755	stable	OSTBETA
0.193004101	stable	LOC388755
0.193044017	stable	LOC651073
0.193044017	stable	FAM162A
0.193081243	stable	NPPB
0.193084094	stable	GPN1
0.193093103	stable	SNORD109B
0.19315834	stable	RUSC2
0.193255381	stable	LTBP4
0.193410679	stable	USP11
0.193629457	stable	LOC730510
0.193629457	stable	LOC644412
0.19365611	stable	POLR3C
0.193894618	stable	PRM1
0.193894618	stable	LCE5A
0.193983915	stable	RSHL1
0.194001917	stable	C22ORF9
0.194119446	stable	TMEM98
0.194158752	stable	NKX2-8
0.194158752	stable	CACNA1I
0.194158752	stable	OR7E37P
0.194200283	stable	AAMP
0.194236951	stable	LOC440978
0.194254408	stable	CDKN2B
0.194254408	stable	PTPRC
0.194409803	stable	RALGPS2
0.194745372	stable	AP4S1
0.194786685	stable	DNAJC8
0.194786685	stable	LOC728653
0.194787587	stable	SLC4A5
0.194883825	stable	MEGF8
0.194892545	stable	PSMD13
0.194912568	stable	CHMP6
0.194969929	stable	FAM115A
0.195117552	stable	TMEM151A
0.195277814	stable	VEPH1
0.19529315	stable	ASB14

0.195326837	stable	LOC651333
0.195384684	stable	C15ORF27
0.195477069	stable	ELL3
0.195477069	stable	BPY2
0.1955549	stable	ADAMTSL1
0.195608736	stable	UTP14A
0.195697655	stable	CCL4
0.195707957	stable	BRSK2
0.195775561	stable	LOC643669
0.195779059	stable	LOC653050
0.195846306	stable	C9ORF117
0.19586323	stable	FBLN7
0.19586323	stable	LOC96610
0.195911617	stable	SFT2D1
0.195911617	stable	LOC642573
0.195911617	stable	LOC646302
0.196201152	stable	CLCN6
0.196269525	stable	FNDC5
0.196269525	stable	RNF216
0.196335339	stable	HSPC047
0.196413385	stable	EEF2
0.196413385	stable	NOMO3
0.196413385	stable	SREBF2
0.196548076	stable	LOC442448
0.196548076	stable	GDE1
0.196635207	stable	PIGN
0.196877499	stable	SEP15
0.196895556	stable	LOC283392
0.197258101	stable	ZNF800
0.197258101	stable	DKFZP451A211
0.197258101	stable	MFAP3L
0.197317435	stable	APOA2
0.197343211	stable	GPM6B
0.197493749	stable	AGGF1
0.197503473	stable	IFNA21
0.197531177	stable	C7ORF43
0.197680702	stable	FAM58B
0.197728324	stable	PRKACB
0.197853037	stable	TCF23
0.198068015	stable	MYO18A
0.198068015	stable	LOC643311
0.198080486	stable	LOC642236
0.198091609	stable	FCF1
0.198138232	stable	LOC652278
0.198247635	stable	CUEDC2
0.198310078	stable	LOC644642
0.198472186	stable	PHF23
0.198514827	stable	LOC644764
0.198552711	stable	LOC647415
0.198638142	stable	ZNF813
0.198723557	stable	TMCO4

0.198802088	stable	ZNF778
0.198882805	stable	LOC254559
0.199022425	stable	RAP1B
0.199072314	stable	LOC653807
0.199078677	stable	C21ORF57
0.199100438	stable	LOC653480
0.199328575	stable	LOC646533
0.19941806	stable	CYP20A1
0.199451581	stable	OR8D2
0.199568718	stable	LOC644264
0.199568718	stable	LOC645151
0.199751226	stable	TTYH2
0.199795297	stable	MBD2
0.199795297	stable	CCDC107
0.199795297	stable	LOC646348
0.199795297	stable	LOH12CR1
0.2002194	stable	LOC388780
0.200261359	stable	ZNF423
0.200353185	stable	ATP2B3
0.200390129	stable	C5ORF22
0.200668298	stable	FLJ36208
0.20069544	stable	LOC643032
0.200732932	stable	NAP1L4
0.200732932	stable	ADAM21
0.201156871	stable	HEPACAM
0.201461464	stable	LOC388153
0.20159018	stable	C3ORF42
0.201648019	stable	CEP135
0.201752214	stable	LOC645478
0.201893453	stable	RPL6
0.201893453	stable	CNNM2
0.20207789	stable	APOBEC3G
0.202131646	stable	DDX18
0.202131922	stable	ZNF623
0.202153294	stable	LOC389833
0.20224069	stable	DTD1
0.202370448	stable	LOC642178
0.202417863	stable	RPP25
0.202520151	stable	XRCC1
0.202520151	stable	LOC642653
0.202560612	stable	LOC654002
0.202560612	stable	ZNF514
0.202625357	stable	SYT13
0.202625357	stable	MKRN3
0.202759095	stable	ZNF702P
0.202759095	stable	LOC647308
0.202759095	stable	DC36
0.202854842	stable	LOC652570
0.202991545	stable	FILIP1L
0.203198491	stable	GSC2
0.203202522	stable	PHOX2A

0.203265077	stable	KIAA1600
0.203265077	stable	KLK1
0.203357582	stable	SEMA7A
0.20369848	stable	PCOLCE
0.203742431	stable	SATB2
0.203742431	stable	LOC731365
0.203830138	stable	CDK8
0.204363708	stable	LOC389607
0.204363708	stable	STX17
0.204373877	stable	LOC642558
0.204659628	stable	LCK
0.204704173	stable	C8ORF77
0.204704173	stable	WDR72
0.204704173	stable	APOBEC3D
0.204778166	stable	LOC647910
0.204778166	stable	LOC440359
0.204778166	stable	FAM62C
0.204778166	stable	HSPA1A
0.20498067	stable	LOC643018
0.205011282	stable	ZNF445
0.205011282	stable	C8ORF59
0.205082772	stable	SEMA4G
0.205100694	stable	LOC440067
0.205158401	stable	LOC648278
0.205303545	stable	C4ORF12
0.205569697	stable	HYLS1
0.205572621	stable	LCE1A
0.20560265	stable	COL8A2
0.20575051	stable	LOC651429
0.205751781	stable	LOC654230
0.205755237	stable	LOC643085
0.205864755	stable	LOC645210
0.206284034	stable	NUP88
0.206379032	stable	VSNL1
0.206456905	stable	RAC3
0.206464299	stable	LOC652357
0.206472395	stable	LOC400796
0.206576672	stable	LOC644066
0.206807535	stable	FLJ37201
0.206814867	stable	FRMPD2L2
0.206857356	stable	CNIH
0.206862253	stable	PDE6G
0.206862253	stable	LOC644294
0.207325609	stable	DNAJC11
0.207325609	stable	OR5W2
0.207325609	stable	FBN1
0.207325609	stable	FLJ40292
0.207325609	stable	AURKAPS1
0.207325609	stable	GTSCR1
0.20761332	stable	CCDC102A
0.207764798	stable	NEU2

0.207764798	stable	LOC646114
0.207764798	stable	LOC643837
0.20784406	stable	GNG12
0.20784406	stable	SAPS2
0.207846802	stable	LOC652578
0.207954688	stable	CCDC103
0.207954688	stable	ALDOB
0.208061224	stable	LCA5
0.208253726	stable	WBSCR27
0.208273326	stable	PLEKHF2
0.208489811	stable	LOC442028
0.20864872	stable	LOC650840
0.20875296	stable	HSD17B13
0.208776857	stable	ITGB4
0.208857232	stable	ARL13A
0.208890025	stable	LOC652614
0.208921262	stable	TLK2
0.209084286	stable	FLJ16369
0.209247372	stable	AIRE
0.209247372	stable	C10ORF10
0.209599576	stable	LOC652568
0.209648398	stable	APOBEC2
0.209680049	stable	TNRC6C
0.209725627	stable	IDH3B
0.20994751	stable	AKT1S1
0.210078581	stable	WNK2
0.210114421	stable	SCGB3A1
0.210114421	stable	LOC441737
0.210135477	stable	IL16
0.210152054	stable	TBCA
0.210157555	stable	LOC644705
0.210282953	stable	VPS53
0.210282953	stable	OR4C16
0.210315585	stable	LOC646128
0.210369412	stable	LTB4R2
0.210446071	stable	MBTD1
0.210626345	stable	IL17B
0.210672428	stable	FBXO44
0.210961081	stable	C2ORF77
0.210972254	stable	ZNF41
0.210972254	stable	ERP27
0.211001576	stable	LOC158381
0.211006991	stable	CLIP1
0.211006991	stable	MRPS23
0.211006991	stable	HINT1
0.211166767	stable	ARID5B
0.211314393	stable	LOC401622
0.211314393	stable	OR4K15
0.2113574	stable	STK11
0.211388962	stable	INPP5F
0.211518537	stable	C1ORF56

0.21167386	stable	LOC646422
0.211677428	stable	LYPLA2P1
0.211677428	stable	C1ORF189
0.211755958	stable	FAHD2B
0.211755958	stable	INTS8
0.211755958	stable	LIG1
0.211827762	stable	CCDC85A
0.211993329	stable	NPLOC4
0.211993329	stable	SNX4
0.212298156	stable	ASCL2
0.212396943	stable	LOC283970
0.212494221	stable	PLEKHM1
0.212672037	stable	PLEKHM3
0.212720295	stable	OR6C4
0.212818703	stable	SUMO1P1
0.212818703	stable	LOC391045
0.212903496	stable	RBP3
0.212903496	stable	TRIP12
0.213025192	stable	LOC402644
0.213025192	stable	LOC653276
0.213038517	stable	NUP153
0.213072818	stable	LOC653125
0.21308422	stable	LIN52
0.21311039	stable	GPR25
0.213207443	stable	LOC652342
0.213268296	stable	LOC650905
0.213348338	stable	LOC652796
0.213415103	stable	ABT1
0.213465872	stable	ANKRD47
0.213525578	stable	LOC442454
0.213526118	stable	PCGF3
0.21374849	stable	LOC652453
0.213797288	stable	SULT1C4
0.213938064	stable	LOC645322
0.213938064	stable	GALC
0.213952879	stable	BGLAP
0.214102875	stable	CBX4
0.214165584	stable	FCAR
0.214274087	stable	PMCHL1
0.21448623	stable	LGALS1
0.214705432	stable	KAT2A
0.214705432	stable	TMEM162
0.214746523	stable	STAG3
0.214779954	stable	GIP
0.214837058	stable	LOC643977
0.214906493	stable	STOML2
0.214988873	stable	NAGS
0.215085482	stable	MPP2
0.215314405	stable	MSRA
0.215314405	stable	SDHB
0.215501086	stable	SCML1

0.215568827	stable	SLC1A1
0.215568827	stable	SPACA5B
0.215616096	stable	C16ORF82
0.215713088	stable	OR5AR1
0.215727234	stable	LOC654253
0.215727234	stable	COL8A1
0.215734271	stable	KIAA1660
0.215734271	stable	LRRC37A
0.21609402	stable	NME7
0.21609402	stable	DCAMKL3
0.216169829	stable	COL19A1
0.21624836	stable	CYP7B1
0.216252847	stable	C4ORF16
0.216298527	stable	MYL4
0.216315813	stable	C1ORF115
0.216329095	stable	SLITRK3
0.216560271	stable	HNMT
0.216634769	stable	OR13C3
0.216678228	stable	ZBTB6
0.216697545	stable	C1ORF100
0.21673724	stable	SLC2A4
0.21673724	stable	C1ORF125
0.216801664	stable	MPRIP
0.216880694	stable	MMP14
0.2170335	stable	DSCAML1
0.217222994	stable	TNFSF12
0.217222994	stable	LOC286239
0.217565716	stable	NOX4
0.217668718	stable	F2RL1
0.217694834	stable	MGC57359
0.217694834	stable	PSMC5
0.217889435	stable	RPS27L
0.217958832	stable	LOC442572
0.218142332	stable	LOC647107
0.218142332	stable	OR2AG1
0.218149045	stable	PCDHGA8
0.218149045	stable	FAM63A
0.218169422	stable	LOC643422
0.218353352	stable	LOC644634
0.218353352	stable	OR7A10
0.218353352	stable	ASTE1
0.218574263	stable	JAKMIP2
0.218598615	stable	LOC645435
0.218618832	stable	ASPHD1
0.218742542	stable	SUGT1P
0.218917026	stable	BCAN
0.218917026	stable	LOC652068
0.219016134	stable	MGC3020
0.219024895	stable	LOC647333
0.219024895	stable	ECHDC2
0.219024895	stable	FUSIP1

0.219038425	stable	MGC40168
0.219068124	stable	PBOV1
0.219112565	stable	LOC644242
0.219243602	stable	DZIP1
0.219243602	stable	LOC440456
0.219243602	stable	LRCH3
0.219243602	stable	HOMER3
0.2192575	stable	ASB4
0.219319016	stable	LOC644886
0.219395155	stable	MRPL50
0.219395155	stable	ATRX
0.219407874	stable	ZNF324
0.219458984	stable	RUNX1
0.219499725	stable	MYSM1
0.219659813	stable	CCDC113
0.219659813	stable	RAB22A
0.219659813	stable	AGPAT6
0.219659813	stable	PLD5
0.219659813	stable	LOC143188
0.219852102	stable	ATG10
0.219866805	stable	IFNA10
0.220178086	stable	CYP24A1
0.220251606	stable	STS
0.220369753	stable	GSX2
0.220369753	stable	SLC13A2
0.22041269	stable	PPP3CA
0.220591341	stable	CD5L
0.220591341	stable	ZNF280B
0.220591341	stable	LGR8
0.220730704	stable	SMAD2
0.220737625	stable	ANGPT1
0.220889288	stable	SPRR1B
0.220923423	stable	LOC646374
0.22093559	stable	PTK9
0.221015995	stable	BRAP
0.221021858	stable	ETAA1
0.221021858	stable	MUCDHL
0.221324548	stable	HOXD9
0.221324548	stable	PIN1
0.221324548	stable	SNX25
0.221324548	stable	APOH
0.221574236	stable	SPESP1
0.221631567	stable	SLMO1
0.221631567	stable	ABCC11
0.221631567	stable	TMBIM4
0.221631567	stable	GPR82
0.221794049	stable	PCSK1
0.221845585	stable	LOC285479
0.221856877	stable	NPHS2
0.221888408	stable	ZNF821
0.222058886	stable	KIFC1

0.222145824	stable	PHACTR1
0.222355342	stable	LRRC37A4
0.222632816	stable	SGTA
0.222773374	stable	GUSB
0.223138068	stable	HBBP1
0.223138068	stable	LOC441601
0.223139095	stable	LOC728863
0.223139095	stable	LOC644424
0.223276664	stable	GJD3
0.223276664	stable	TMPRSS6
0.223391733	stable	XPNPEP1
0.223585962	stable	CD3EAP
0.223850917	stable	TMEM45A
0.223850917	stable	LOC653497
0.223899119	stable	MBOAT1
0.223980244	stable	LOC442366
0.224021124	stable	ARMC9
0.224021124	stable	LOC440053
0.224081707	stable	LOC653876
0.224188212	stable	MRPL41
0.224209657	stable	ZNF219
0.224297031	stable	H2AFX
0.224297709	stable	BEGAIN
0.224326622	stable	LOC732111
0.224418473	stable	LOC648841
0.224455541	stable	LOC374920
0.224481495	stable	FLJ41200
0.224723805	stable	NOL4
0.224836382	stable	PRSS7
0.224836382	stable	LOC650803
0.224836382	stable	FAM22B
0.224836382	stable	FLJ11235
0.224910718	stable	USP21
0.225340612	stable	EFCAB2
0.225349055	stable	NADK
0.225364219	stable	C17ORF42
0.225488379	stable	LYST
0.225564981	stable	LOC651483
0.225564981	stable	LOC728789
0.225564981	stable	WDR37
0.225678802	stable	LOC728936
0.225951544	stable	RPL36AL
0.2260733	stable	LOC646064
0.22609898	stable	SULT2B1
0.226115903	stable	NEFM
0.226284744	stable	CHRNE
0.226392964	stable	LOC643346
0.226392964	stable	LOC644266
0.226464256	stable	CD40LG
0.226475829	stable	FLJ44005
0.226475829	stable	PCSK5

0.226591201	stable	TRIM4
0.226591201	stable	OR7A5
0.226650418	stable	XAB2
0.226803925	stable	LOC653874
0.226803925	stable	TRPM6
0.226886467	stable	THEG
0.226964713	stable	ZNF826
0.226977982	stable	ADAMTSL3
0.226977982	stable	RTL1
0.227199155	stable	UTP6
0.227359113	stable	RPS7
0.2274705	stable	WDR19
0.227513941	stable	KIAA1609
0.227572425	stable	RACGAP1
0.227572425	stable	C16ORF54
0.227572425	stable	LOC642397
0.227721066	stable	LCN9
0.227759122	stable	DEFB121
0.227766159	stable	CYSLTR1
0.227790764	stable	PCDHA3
0.227798587	stable	SLC41A2
0.227852265	stable	OR4F21
0.227895686	stable	GJA10
0.227899984	stable	EEF1B2
0.227901686	stable	GALNT13
0.227901686	stable	PHPT1
0.228031167	stable	LBA1
0.228031167	stable	THBS3
0.228276013	stable	MON1A
0.228276013	stable	ASH1L
0.228419939	stable	LOC729744
0.228419939	stable	LOC648827
0.22846868	stable	LOC642373
0.228495091	stable	ZNF280A
0.2284976	stable	LOC642968
0.228525996	stable	HSFX1
0.228525996	stable	LOC127295
0.228746991	stable	LOC146439
0.228763464	stable	OR4X2
0.228797781	stable	AKAP13
0.228797781	stable	LOC654010
0.228921877	stable	CATSPER3
0.228966061	stable	ATP5I
0.229000895	stable	GLP2R
0.229115065	stable	IMP3
0.229151178	stable	LOC391378
0.22917938	stable	CDC42BPA
0.229284927	stable	ASB11
0.229340914	stable	LOC388210
0.22944372	stable	MUC16
0.22944372	stable	VANGL2

0.229565761	stable	LOC652553
0.229565761	stable	SLC5A10
0.229565761	stable	IRS4
0.2298908	stable	KPNA5
0.229894536	stable	LOC652674
0.230023597	stable	CAPS
0.230097425	stable	CRYGB
0.230158888	stable	SPTB
0.230158888	stable	LOC647855
0.230158888	stable	AMOTL2
0.230294273	stable	LOC652620
0.230394652	stable	RPRC1
0.230466211	stable	NCF2
0.230500862	stable	ART1
0.230520708	stable	PODXL
0.230520708	stable	SMURF1
0.230520708	stable	LOC647122
0.230556356	stable	OR5K4
0.230649281	stable	RBM34
0.230655693	stable	LOC340113
0.230655693	stable	INHA
0.230794243	stable	CT47.1
0.230957253	stable	MOGAT2
0.23099604	stable	LOC642183
0.2309989	stable	ALPI
0.231076457	stable	CES1
0.231095545	stable	PPT1
0.231095545	stable	LOC643258
0.231095545	stable	EDEM3
0.231095545	stable	COX6B1
0.231135584	stable	RY1
0.231135584	stable	NSD1
0.231135584	stable	RAB41
0.231189726	stable	ZBTB10
0.23143971	stable	SLC14A1
0.231473439	stable	LOC642933
0.231483756	stable	CCDC60
0.231483756	stable	HOXC6
0.231744746	stable	FGD4
0.231977048	stable	LOC642834
0.232244545	stable	C17ORF79
0.232306194	stable	SNTG1
0.232306818	stable	FRMD4B
0.232318419	stable	MRRF
0.232318419	stable	SKIP
0.232318419	stable	DGKD
0.232342112	stable	PROSC
0.232419151	stable	TRERF1
0.23244742	stable	LIPI
0.232555888	stable	TTRAP
0.232561284	stable	PRKACG

0.232624839	stable	LOC644297
0.232624839	stable	LCE3D
0.232688634	stable	C16ORF47
0.232697849	stable	LOC646699
0.232796965	stable	FBXO39
0.232819037	stable	CRP
0.232883297	stable	LOC645931
0.232988991	stable	SMAD4
0.233362177	stable	ALCAM
0.233561069	stable	LOC653889
0.233685222	stable	PPM1H
0.233742129	stable	KRTAP3-2
0.233800537	stable	TMCO1
0.233817157	stable	KLHL8
0.233848206	stable	SDC4
0.233848206	stable	LOC399939
0.234082585	stable	TMEM125
0.234109317	stable	LOC390594
0.234125025	stable	LOC642795
0.234149494	stable	CSPG5
0.234172735	stable	SNORA7B
0.234172735	stable	QSOX2
0.23427401	stable	ATPBD3
0.234319546	stable	LPL
0.234362309	stable	HOXC5
0.234362309	stable	NAALADL1
0.234362309	stable	MRC2
0.23436825	stable	TMEM109
0.234403089	stable	NOLA2
0.234405764	stable	IQCF1
0.234452288	stable	CASC3
0.234508752	stable	TRRAP
0.234651226	stable	HRC
0.234713924	stable	LOC650361
0.234881768	stable	LOC654194
0.235081322	stable	INVS
0.235268354	stable	CLDN14
0.235291773	stable	CXORF27
0.235298276	stable	EEF1A2
0.235444784	stable	IKZF1
0.235444784	stable	IGFL3
0.235550936	stable	LOC645904
0.235555964	stable	LOC653217
0.235611987	stable	FBXO2
0.235625798	stable	ATXN7L1
0.235789677	stable	MUTYH
0.235789677	stable	CD2BP2
0.235829089	stable	EXOSC9
0.236040595	stable	ZNF624
0.236247667	stable	SAPS3
0.236258155	stable	OR51L1

0.23625825	stable	LCE2C
0.236408534	stable	LOC647009
0.236419411	stable	LOC653265
0.236538214	stable	OR1Q1
0.236538214	stable	C1ORF185
0.236538214	stable	MCOLN3
0.236538214	stable	OR5D16
0.236741876	stable	ZNF655
0.236741876	stable	CAB39L
0.236909788	stable	DIRAS2
0.236909788	stable	RBM33
0.236930629	stable	CYLC2
0.236930629	stable	CCNA1
0.237022653	stable	LOC643401
0.237165435	stable	TCP1
0.237165435	stable	CLDN18
0.237180418	stable	PDGFRL
0.237180418	stable	FAM71D
0.237180418	stable	SMAD1
0.237180418	stable	LOC283584
0.23729748	stable	SMPD3
0.237328985	stable	FLJ40113
0.237437962	stable	CS
0.237449409	stable	ZNF195
0.237449409	stable	KLRD1
0.237561556	stable	MAPK4
0.237849329	stable	TBC1D9
0.237860941	stable	BLVRA
0.237860941	stable	MFF
0.237860941	stable	MMD2
0.237860941	stable	LOC652667
0.238366096	stable	DLST
0.238388832	stable	GML
0.238417792	stable	DES
0.238417792	stable	LOC649583
0.238443749	stable	LOC441081
0.238545153	stable	FLJ11783
0.23858022	stable	C9ORF126
0.238623846	stable	SNHG10
0.238701313	stable	LOC649975
0.238701313	stable	MGC16075
0.238712711	stable	LOC646982
0.238842921	stable	GPR133
0.23890847	stable	ITM2A
0.239121923	stable	LOC654187
0.23913807	stable	C5ORF48
0.23913807	stable	MED15
0.23917436	stable	KRTAP10-11
0.23923346	stable	TFAP2A
0.23923346	stable	CCNT1
0.239316349	stable	OR5AP2

0.239344451	stable	PFKFB1
0.239344451	stable	C2ORF52
0.239344451	stable	LOC646036
0.239375606	stable	LOC439992
0.239608561	stable	GRAMD1C
0.23964634	stable	LOC651894
0.239684037	stable	KCNE4
0.23979454	stable	LOC286411
0.239896731	stable	GPR115
0.239896731	stable	GPR20
0.239988698	stable	HERC2
0.239988698	stable	SLC27A5
0.240274106	stable	C9ORF144
0.240275598	stable	SRP19
0.240280018	stable	LOC401321
0.240542891	stable	TSEN34
0.240587044	stable	RBM15
0.240615273	stable	C1QTNF4
0.240615273	stable	CHRNA6
0.240620946	stable	TCN1
0.2407092	stable	ZNF562
0.240751849	stable	BCL2L11
0.240770421	stable	ANKS3
0.240776068	stable	LOC90835
0.24079159	stable	LOC643690
0.240985636	stable	LOC650546
0.241237451	stable	SARS
0.241237451	stable	LOC729764
0.241635458	stable	BIRC7
0.241684405	stable	LOC441178
0.242033943	stable	CRAMP1L
0.242177494	stable	LIPC
0.242263162	stable	HECTD1
0.242303648	stable	ANKRD58
0.242612047	stable	PTPN23
0.242705553	stable	PLUNC
0.242762578	stable	LOC648293
0.242906603	stable	HTR1B
0.242979576	stable	ZNF45
0.243077664	stable	C14ORF177
0.243116669	stable	TMEM55B
0.243121876	stable	OR5T1
0.243295587	stable	FAM53C
0.243297087	stable	ADAMTS3
0.243297087	stable	ACSS3
0.243407705	stable	TAAR2
0.24349069	stable	ART3
0.24354868	stable	LOC388177
0.243707307	stable	GIPR
0.244070869	stable	LOC652543
0.244177203	stable	STRAP

0.24420359	stable	LOC650140
0.244262356	stable	TREX2
0.244418154	stable	MSH6
0.244449906	stable	IQCD
0.244449906	stable	FLJ45300
0.244480988	stable	LOC653451
0.244480988	stable	PCCA
0.244480988	stable	SFN
0.244550992	stable	C4ORF35
0.244634219	stable	CCL3L3
0.244667059	stable	ATP9A
0.245016592	stable	CA2
0.245175	stable	SDHC
0.245268622	stable	APLN
0.245567718	stable	TRY6
0.245707399	stable	C1ORF109
0.245725302	stable	LOC645609
0.245786775	stable	LOC641849
0.245821422	stable	DEGS2
0.24583756	stable	MST4
0.24587818	stable	ZNF90
0.246138651	stable	LOC729185
0.246273669	stable	HIST3H2BB
0.246630148	stable	FSD1L
0.24672864	stable	EXO1
0.246947549	stable	LOC339692
0.247029725	stable	LOC645590
0.247029725	stable	LOC644423
0.247029725	stable	KDELR1
0.247058658	stable	MRC1L1
0.247058658	stable	ZAP70
0.247058658	stable	STAT2
0.247229491	stable	LOC727761
0.247229491	stable	SNORA65
0.247229491	stable	CT47.6
0.247234312	stable	CNGB1
0.247460073	stable	DDX19B
0.247493565	stable	LOC647038
0.24762801	stable	LOC643922
0.247641548	stable	DEDD2
0.24765818	stable	DDX60
0.248092615	stable	MCTS1
0.248446682	stable	WDR93
0.24850539	stable	MTF1
0.248515723	stable	PKMYT1
0.248515723	stable	LOC91664
0.24858054	stable	ACVR1C
0.24858054	stable	C16ORF7
0.248633127	stable	FBXO25
0.248708858	stable	KRTAP13-4
0.24881394	stable	LOC440361

0.248831786	stable	LOC642820
0.248970429	stable	GJA3
0.248970429	stable	LOC388161
0.248970429	stable	NAG
0.24904463	stable	POLR2J4
0.249128965	stable	LOC644519
0.249197348	stable	COPS3
0.249269268	stable	RFXDC2
0.249293695	stable	ZNF642
0.249363859	stable	LOC645342
0.249497472	stable	LOC492303
0.2495851	stable	LOC390688
0.249617291	stable	CSF3
0.249618245	stable	LOC652051
0.249655472	stable	MIZF
0.249934933	stable	TAS2R45
0.250056459	stable	CCDC88A
0.25008434	stable	MBL1P1
0.25008434	stable	AICDA
0.250085253	stable	RREB1
0.250085253	stable	LOC646482
0.250124505	stable	ARTN
0.250124505	stable	CDC45L
0.25015814	stable	ZNF330
0.250252305	stable	FLJ14166
0.250300589	stable	ZNF121
0.250323551	stable	LOC653057
0.250414979	stable	SLC45A4
0.250414979	stable	VPS33A
0.250440011	stable	ASB6
0.25051508	stable	SLC26A11
0.250579634	stable	CYP4Z1
0.25082538	stable	LOC652767
0.251077708	stable	CDH24
0.25120067	stable	USP29
0.251466289	stable	ZNF268
0.251466289	stable	LOC643008
0.251587232	stable	GATA3
0.251780777	stable	LOC652726
0.251818301	stable	MPV17
0.251900647	stable	MAT2A
0.251943661	stable	TNRC4
0.251943661	stable	KHDRBS1
0.251957629	stable	PPM1K
0.251989657	stable	PDGFA
0.252058345	stable	UBE2D2
0.25207561	stable	GPR21
0.25223036	stable	FLJ42133
0.252468961	stable	PRKCA
0.2525035	stable	STX10
0.252523965	stable	LOC440297

0.252527835	stable	FCRL5
0.252535945	stable	LOC649057
0.252664403	stable	CLSPN
0.252718839	stable	HIST2H2AC
0.252718839	stable	LCE3A
0.252718839	stable	LRP12
0.252718839	stable	LOC143543
0.252763276	stable	ABCF3
0.252828582	stable	REEP6
0.252828582	stable	LOC374491
0.252845639	stable	DDEF2
0.25287629	stable	LOC400958
0.252992644	stable	LOC652606
0.253089579	stable	LOC729751
0.253148826	stable	TMC8
0.25333662	stable	RAB1C
0.25333662	stable	LOC285141
0.25335333	stable	ZNF263
0.253446126	stable	ICMT
0.253533586	stable	RASGRF2
0.253573449	stable	ZMYND10
0.253607855	stable	LOC728876
0.253611494	stable	LOC651357
0.253611494	stable	EDEM1
0.25425314	stable	BTN3A1
0.254301806	stable	IL5RA
0.254556517	stable	SLC6A17
0.254556517	stable	C20ORF82
0.254556517	stable	RRM2B
0.254635421	stable	DENND4A
0.254740397	stable	GPR52
0.254964758	stable	PIK3R5
0.255355103	stable	SPSB3
0.255464867	stable	XPO7
0.255464867	stable	CALM3
0.255464867	stable	YBX2
0.255501842	stable	LOC115648
0.255533718	stable	CLSTN2
0.255576759	stable	SLC4A10
0.255759465	stable	NECAP1
0.256000198	stable	DAZL
0.256105531	stable	LOC652197
0.256184011	stable	DCD
0.256250818	stable	LOC144776
0.256250818	stable	FAM106A
0.256423907	stable	POLR2H
0.256432446	stable	MAMLD1
0.256432446	stable	DCST2
0.256432446	stable	EN1
0.256432446	stable	LOC653262
0.256432446	stable	CDCA8

0.256432446	stable	LPHN1
0.25659277	stable	PASD1
0.25660789	stable	TWF2
0.256630633	stable	SURF1
0.256631542	stable	NLF2
0.256659376	stable	C14ORF112
0.256701186	stable	TBXA2R
0.256773396	stable	BCL8
0.256955203	stable	LOC390192
0.257136523	stable	TAS2R49
0.257215702	stable	ANKHD1
0.257215702	stable	ZCCHC17
0.257303653	stable	PSMA1
0.257303653	stable	GOLGA5
0.257307799	stable	LOC728518
0.257367064	stable	CCDC82
0.257367064	stable	ADCY2
0.257375453	stable	COX19
0.257406416	stable	LOC387804
0.257564151	stable	ATG12
0.257627676	stable	NUDT17
0.257978254	stable	CT45-1
0.258074765	stable	LOC653604
0.258074765	stable	REEP4
0.258112565	stable	DOK4
0.258134687	stable	PIR
0.25814267	stable	BAMBI
0.258560532	stable	LOC652722
0.25865203	stable	LOC654135
0.25872709	stable	FBXL15
0.258871367	stable	LOC389669
0.259282087	stable	FKBPL
0.259325113	stable	SLC30A3
0.259341862	stable	HIST1H3J
0.259475873	stable	TYW3
0.259531245	stable	ADRA1A
0.259531245	stable	LOC644556
0.259591581	stable	GTPBP10
0.259591581	stable	TTC31
0.259787792	stable	LOC643980
0.259814629	stable	LOC647065
0.25989675	stable	ZBTB26
0.259919194	stable	XRRA1
0.259940773	stable	LOC643403
0.260011035	stable	NPAL1
0.260014962	stable	TPH2
0.26002776	stable	PDE11A
0.260150519	stable	LOC651059
0.260167232	stable	LOC647339
0.260167232	stable	LGALS12
0.260187809	stable	C6ORF78

0.260307122	stable	MLF2
0.260554673	stable	HIST1H2AI
0.260648489	stable	LOC648526
0.260706537	stable	GFRA1
0.260706992	stable	LOC653059
0.260756737	stable	FLJ25778
0.260756737	stable	PRC1
0.260993406	stable	PM20D2
0.260993406	stable	RAP2C
0.261058907	stable	MGC39900
0.261118714	stable	SV2C
0.261137347	stable	INSRR
0.261253704	stable	LOC653733
0.261298769	stable	KHDC1
0.261675072	stable	MTHFSD
0.261676027	stable	XAGE1E
0.261731115	stable	BTLA
0.261869609	stable	PC
0.261907254	stable	LOC653420
0.262538581	stable	ZIM3
0.26301442	stable	LOC642778
0.263117422	stable	USP4
0.263120248	stable	LOC645851
0.263238419	stable	LOC730919
0.263238419	stable	GOT1L1
0.263429453	stable	CDKN3
0.263437306	stable	LOC388122
0.26351318	stable	CASP2
0.26353655	stable	HSPC152
0.263674266	stable	ANKFN1
0.263682575	stable	LOC647269
0.263732657	stable	LOC440313
0.26388045	stable	PPP2R5D
0.26388045	stable	STX3
0.26388045	stable	RDX
0.264198529	stable	LGALS9C
0.264203603	stable	PDLIM7
0.264203603	stable	RCAN1
0.2642232	stable	LOC643226
0.264246658	stable	CAPN13
0.264325107	stable	IL7
0.264369634	stable	SMU1
0.264441765	stable	ACAP1
0.264454814	stable	TSGA10IP
0.264545349	stable	ADIPOR1
0.264767044	stable	CYCS
0.264825772	stable	FLJ12949
0.264829798	stable	BCL6B
0.264902796	stable	LOC93463
0.264902796	stable	BTBD9
0.264984407	stable	TSEN54

0.265040225	stable	SLC9A3
0.265210469	stable	LOC642468
0.26521951	stable	LOC441956
0.2652226	stable	RAI14
0.265617725	stable	TTTY21
0.265640277	stable	RASGEF1B
0.265707158	stable	LOC646508
0.265736092	stable	LOC643962
0.265872564	stable	LOC641989
0.266040172	stable	C12ORF59
0.266151092	stable	GP5
0.266183276	stable	LOC645981
0.266184931	stable	KLF3
0.266338793	stable	NTF3
0.266401432	stable	UCKL1
0.266401432	stable	LOC730686
0.266401432	stable	CD83
0.266474415	stable	BTG4
0.266763846	stable	MGC29506
0.266772834	stable	ARRDC1
0.266772834	stable	LOC645732
0.267094445	stable	EVI5
0.267111924	stable	LOC440926
0.267111924	stable	FAM155A
0.2672413	stable	OR51S1
0.26729658	stable	ZNF699
0.26732169	stable	SNORD15A
0.26732169	stable	EHMT2
0.267428376	stable	HMGN1
0.267428549	stable	UTS2D
0.267480856	stable	RSPO2
0.267606364	stable	RPL41
0.267810218	stable	PDZD7
0.26781321	stable	MUC12
0.268031985	stable	LOC400831
0.26813145	stable	OTOR
0.268243105	stable	DEAF1
0.268293922	stable	SEC16B
0.268299938	stable	KCNA10
0.268318404	stable	LOC644919
0.268318404	stable	USP19
0.268379667	stable	BPTF
0.268379667	stable	NXPH1
0.268379667	stable	LOC647022
0.268420811	stable	C12ORF53
0.268486402	stable	TTC21A
0.26849371	stable	MPO
0.268551807	stable	LOC653419
0.268556871	stable	LOC728351
0.26869248	stable	MSI1
0.26869248	stable	WDR57

0.268694971	stable	GZMA
0.268715357	stable	TRIM67
0.268891788	stable	UIMC1
0.268891876	stable	TMEM26
0.269017067	stable	LOC648196
0.269356592	stable	SETX
0.269364956	stable	SPRY4
0.269408057	stable	TRIM34
0.269444976	stable	C1ORF120
0.269444976	stable	LOC644250
0.269570327	stable	ADAM17
0.270041184	stable	LOC648622
0.270234623	stable	METTL2B
0.270267372	stable	LOC653734
0.270291909	stable	LNPEP
0.270480934	stable	ABCA4
0.270480934	stable	LOC646428
0.270718039	stable	GJB3
0.270739931	stable	LOC643626
0.270739931	stable	DOM3Z
0.270820669	stable	TESK2
0.270820669	stable	CEACAM21
0.271492821	stable	C7ORF45
0.271596154	stable	LOC727759
0.271678148	stable	GABPAP
0.272080043	stable	MGC10701
0.272293394	stable	HEL308
0.272293394	stable	PPP4R1L
0.272347668	stable	A2M
0.272579719	stable	LOC641955
0.272590227	stable	LHX1
0.272590227	stable	LOC732316
0.272590227	stable	CIZ1
0.272592543	stable	RNF39
0.272618828	stable	KRT6A
0.273026465	stable	LOC645168
0.273094018	stable	EXPH5
0.273137009	stable	LOC653495
0.273329189	stable	BLCAP
0.273460082	stable	PPAP2C
0.273460082	stable	LOC401233
0.273481866	stable	RAB11FIP5
0.273500492	stable	ATP5C1
0.273507962	stable	PPP1R14D
0.273616308	stable	ACP1
0.273616308	stable	LOC391157
0.273629678	stable	PLA2G4A
0.273629678	stable	LOC645302
0.273742768	stable	DIRAS3
0.273755518	stable	TCF7
0.273800567	stable	PRLHR

0.273905968	stable	LOC253724
0.27395682	stable	LOC338799
0.273960851	stable	RPL29P2
0.274019621	stable	SON
0.274042237	stable	FUK
0.274061715	stable	KRTAP12-1
0.274072554	stable	LOC284757
0.274072554	stable	RPL13
0.274467176	stable	PRG2
0.274467176	stable	ZNF486
0.274467176	stable	LOC644120
0.27448505	stable	LMLN
0.27448505	stable	LOC202459
0.274540323	stable	SLC12A1
0.27468699	stable	BARHL1
0.274709943	stable	LOC649365
0.274890766	stable	ZCCHC13
0.275000411	stable	LOC644334
0.275099145	stable	CNGA1
0.275138695	stable	STK25
0.275214013	stable	CHR415SYT
0.275269774	stable	SLC6A2
0.275276579	stable	C7ORF42
0.27531055	stable	NT5C
0.275327221	stable	MYL2
0.275395028	stable	LOC283202
0.275395028	stable	TSPAN16
0.275483374	stable	LOC643847
0.275491382	stable	LOC650122
0.275625473	stable	PEX26
0.275631556	stable	POLD4
0.275655342	stable	KRTAP10-1
0.275655342	stable	FLJ34931
0.275655342	stable	LOC646366
0.275655342	stable	CTXN3
0.275655342	stable	LOC283788
0.275746909	stable	CRYGA
0.275755539	stable	LOC648509
0.275855344	stable	PCDHA12
0.275872641	stable	SPACA1
0.276097114	stable	LOC643253
0.276097114	stable	PIWIL3
0.27613966	stable	LOC643235
0.276263241	stable	HOM-TES-103
0.276272427	stable	SNAP29
0.276300767	stable	UBE2G1
0.276300767	stable	LOC644362
0.276313014	stable	RCVRN
0.276321635	stable	OR2Z1
0.276321635	stable	AK5
0.276403146	stable	ABCD4

0.276562123	stable	CLEC17A
0.276660031	stable	MYST3
0.276774041	stable	RBBP5
0.276775861	stable	PAPPA
0.276775861	stable	FLJ40852
0.276775861	stable	OR3A3
0.276783117	stable	LOC643581
0.276859776	stable	GSTO2
0.276932028	stable	LOC440080
0.276985308	stable	LOC652571
0.277102216	stable	SGSH
0.277154564	stable	WNT11
0.277246011	stable	RPL11
0.277436949	stable	LOC652011
0.277436949	stable	EIF2AK1
0.277472332	stable	FLJ46838
0.277484544	stable	FLJ20309
0.277562528	stable	GPR109B
0.27769883	stable	KLHL32
0.277707869	stable	KCNAB2
0.27773086	stable	GFRA3
0.27773223	stable	UNQ9433
0.277829722	stable	LOC645705
0.278051141	stable	LOC647286
0.278102128	stable	LOC644973
0.278161177	stable	CXCR6
0.278168759	stable	GSS
0.278174183	stable	LOC641694
0.278174183	stable	LOC642891
0.278174183	stable	CRIP2
0.278174183	stable	KIAA0090
0.278545006	stable	KRT24
0.278553985	stable	DKFZP686C2281
0.278570268	stable	CHST4
0.278570268	stable	RNF185
0.278570268	stable	TADA3L
0.27865026	stable	OR14J1
0.27865026	stable	ZNHIT2
0.27865026	stable	LOC728564
0.278660514	stable	NKG7
0.27873501	stable	HMGB3
0.27876263	stable	TP53TG3
0.278969918	stable	LGALS2
0.279157965	stable	LOC728729
0.27965542	stable	LOC730432
0.27965542	stable	PRR12
0.27965542	stable	RAF1
0.279663195	stable	SPATA19
0.279663195	stable	SYN3
0.279799866	stable	CXORF50
0.279823546	stable	CLEC4F

0.279969423	stable	PCDH24
0.279993781	stable	LOC642644
0.280034029	stable	ABCC6
0.280397448	stable	LOC644008
0.280397448	stable	LOC440820
0.280397448	stable	ZIC2
0.280397448	stable	SPATS1
0.280397448	stable	COX7A1
0.280397448	stable	NLRP9
0.280475639	stable	LOC646243
0.280593408	stable	LOC653609
0.280642959	stable	TBKBP1
0.280646347	stable	MED30
0.280885475	stable	C1ORF106
0.280969157	stable	TMEM132C
0.281009469	stable	ZIC5
0.281064297	stable	MRVI1
0.281419441	stable	ZWINT
0.281439538	stable	SBNO1
0.281502786	stable	SSX1
0.281672032	stable	ELK3
0.281706161	stable	SAC3D1
0.281851467	stable	RSRC2
0.281947595	stable	LOC642290
0.282195682	stable	ADAM2
0.282241883	stable	RCHY1
0.282272313	stable	REXO4
0.282376808	stable	C19ORF36
0.283137117	stable	GPR35
0.283164752	stable	KIFC2
0.283241763	stable	PKD1L3
0.283351762	stable	FAM24B
0.283383789	stable	SCGB1A1
0.283551411	stable	HPGD
0.283551411	stable	OSBPL6
0.283610815	stable	MGC39372
0.283610815	stable	GABRA4
0.283774478	stable	RIMS4
0.283912808	stable	LOC643558
0.284044763	stable	EIF2C3
0.284347453	stable	LOC647947
0.284561077	stable	RGS14
0.284925866	stable	C6ORF206
0.284925866	stable	LEMD3
0.284975756	stable	H3F3A
0.285067314	stable	FLJ43950
0.285122049	stable	FAM166A
0.285122049	stable	PRR11
0.285122049	stable	KLKB1
0.285122049	stable	WDR63
0.285189626	stable	LOC642716

0.285191232	stable	LOC642954
0.285216306	stable	SCRT1
0.285243734	stable	RPL36
0.28529494	stable	FLJ38969
0.285325716	stable	MMP19
0.285325716	stable	GABRA1
0.285345141	stable	ENTPD2
0.285365108	stable	TSSK2
0.285598984	stable	HOXD3
0.285722445	stable	LOC644725
0.285755018	stable	EFNA4
0.285833532	stable	C12ORF35
0.285997248	stable	CHEK1
0.286222473	stable	C20ORF152
0.286372042	stable	ZFY
0.286372042	stable	LOC647274
0.28658611	stable	SAMD10
0.286753648	stable	LOC652209
0.286808601	stable	FAM83E
0.286905599	stable	LOC389102
0.286905599	stable	NDUFA12
0.286906017	stable	C20ORF94
0.286926971	stable	C14ORF100
0.286926971	stable	LOC646717
0.286928358	stable	SEC62
0.287128533	stable	BRCC3
0.287403945	stable	CICE
0.287470402	stable	LMX1A
0.287580572	stable	LOC649199
0.287639571	stable	OR5K2
0.287657481	stable	LOC441326
0.287678468	stable	LOC642250
0.287842053	stable	NLP
0.287922937	stable	CCL3
0.287996311	stable	LOC647466
0.288142473	stable	LOC641716
0.288142473	stable	NFATC4
0.288219041	stable	SNORD14A
0.288412623	stable	FOXP2
0.288846403	stable	LOC643389
0.288951631	stable	PCM1
0.289034454	stable	TXNDC2
0.289034454	stable	ATP5O
0.28908379	stable	KRT75
0.28908379	stable	LOC653463
0.289147302	stable	GDAP1
0.289360524	stable	CARD14
0.289429863	stable	FAIM3
0.289521035	stable	TNRC9
0.289578577	stable	LOC643482
0.289589165	stable	SLC4A9

0.289650228	stable	LOC644188
0.28970569	stable	LOC646431
0.289747654	stable	LOC284067
0.289747654	stable	C6ORF184
0.289747654	stable	NDUFB6
0.289747654	stable	UBLCP1
0.289954129	stable	CRKRS
0.290143297	stable	LOC729393
0.290272568	stable	LOC653651
0.290401071	stable	LOC650721
0.290490863	stable	INSM1
0.290519695	stable	MGC15705
0.290745765	stable	MRLC2
0.290919302	stable	OR10AG1
0.29098161	stable	NSUN6
0.29098161	stable	KCNN4
0.291045797	stable	KIF26B
0.291069791	stable	IPO8
0.29125713	stable	LOC653781
0.291263812	stable	LOC654109
0.291360179	stable	CYCSL1
0.291360179	stable	LOC196913
0.291659004	stable	LOC388923
0.291691083	stable	POU2F2
0.291853316	stable	LOC145783
0.291863963	stable	LOC652481
0.291931561	stable	GALNT12
0.291961299	stable	SEMA3D
0.292097254	stable	PTPRK
0.292097254	stable	BSND
0.292211897	stable	HIATL1
0.292211897	stable	LOC647566
0.292211897	stable	LOC442590
0.292253465	stable	LOC648377
0.292428575	stable	UNKL
0.292428575	stable	FBXW8
0.292520862	stable	ZNF215
0.292745294	stable	HAO2
0.292831171	stable	LOC642969
0.292859942	stable	SNX3
0.292922361	stable	CDC42BPG
0.292922361	stable	NTRK3
0.29347688	stable	FAM27L
0.29371099	stable	FLJ37078
0.293861618	stable	KLKP1
0.293897526	stable	KLC1
0.294023198	stable	SH2D4A
0.294035038	stable	C5
0.294035038	stable	ANAPC11
0.294102568	stable	LOC651060
0.294102568	stable	LOC285176

0.294182273	stable	HPRT1
0.294182273	stable	OR5B21
0.294182273	stable	PSMB1
0.294186687	stable	SLC17A8
0.294328786	stable	SULT1B1
0.294344616	stable	VDR
0.29465235	stable	ZC3H15
0.294687235	stable	PTBP2
0.294687235	stable	TNS3
0.294704463	stable	LOC650132
0.294723179	stable	GBA2
0.294918074	stable	SKI
0.295030793	stable	RNF148
0.295089305	stable	MRPL48
0.295103995	stable	STOX2
0.295180046	stable	NLRP1
0.295226915	stable	CLEC4C
0.295273486	stable	LOC728990
0.295500968	stable	STRA6
0.295514528	stable	RNASE8
0.295531186	stable	LOC644065
0.295531186	stable	NCOA6IP
0.295580381	stable	FCRL2
0.295580381	stable	LOC643923
0.295596492	stable	AARSD1
0.295596492	stable	C20ORF11
0.295784402	stable	RIC8B
0.295841617	stable	AKAP9
0.295873399	stable	LOC647169
0.295873399	stable	ANO4
0.296660704	stable	SEC31B
0.29675899	stable	SNORD4B
0.296809062	stable	LOC649712
0.296809062	stable	C1ORF76
0.297161951	stable	SPINT4
0.297171414	stable	HCRTR2
0.29728744	stable	RABL2A
0.297321354	stable	FGFBP2
0.297321354	stable	LOC642903
0.297321798	stable	RPS16
0.297321798	stable	PHLDA1
0.297329107	stable	SHFM1
0.297329107	stable	EED
0.297430431	stable	CD3E
0.297484328	stable	TAAR8
0.297505571	stable	SCGB1C1
0.297505571	stable	FAM123A
0.297505571	stable	GNG4
0.297548015	stable	STOX1
0.297548015	stable	CDH2
0.297613436	stable	LOC653092

0.297613436	stable	NT5C1B
0.297613436	stable	DUSP12
0.297661476	stable	KIAA1875
0.297874824	stable	OR11H4
0.297903582	stable	UMOD
0.297992724	stable	SNORD54
0.298159933	stable	ZNF321
0.298466877	stable	CD79B
0.298923565	stable	LOC644799
0.298923565	stable	SCIN
0.298923565	stable	LOC133185
0.299086641	stable	CLEC6A
0.299094916	stable	NUP160
0.299499744	stable	EIF2B1
0.299503923	stable	GLI4
0.299667604	stable	KLRK1
0.29976269	stable	RFPL3S
0.300002093	stable	LRRC41
0.300015229	stable	LOC130678
0.300137468	stable	LOC442316
0.300248471	stable	MCHR1
0.300301032	stable	ARX
0.300397352	stable	LOC387856
0.300527049	stable	SCYL2
0.300613246	stable	USP24
0.300614331	stable	TMEM53
0.300667774	stable	LOC653663
0.300776121	stable	UQCRB
0.300776121	stable	NPAS4
0.300780778	stable	LOC653103
0.300780778	stable	LOC646663
0.300873743	stable	BEST3
0.301085228	stable	LOC652745
0.301468363	stable	TUBGCP2
0.301685834	stable	LOC645126
0.301725057	stable	C15ORF48
0.301779261	stable	APOD
0.301801005	stable	MARCH7
0.301819064	stable	GPR61
0.301905338	stable	PI15
0.301925738	stable	LOC376693
0.302065535	stable	LOC400752
0.30209842	stable	LOC652439
0.30212046	stable	LOC440092
0.302242194	stable	LOC647080
0.302275291	stable	MRPL13
0.30228311	stable	C20ORF38
0.302716357	stable	LOC648133
0.302716357	stable	LOC651829
0.302894694	stable	POU4F2
0.302911773	stable	OLFML2B

0.303030368	stable	LOC644167
0.303053165	stable	PKP3
0.303196131	stable	DGAT2L6
0.303210852	stable	TMEM45B
0.303213114	stable	CCDC70
0.303273789	stable	TMEM4
0.303273789	stable	LOC646449
0.303296883	stable	ENOSF1
0.30335891	stable	SLC39A7
0.303375322	stable	LOC643695
0.303481676	stable	LOC650162
0.303481676	stable	LOC644500
0.303495439	stable	JMJD2B
0.303687159	stable	ZDHHC19
0.303729849	stable	LOC645644
0.303733555	stable	LOC649351
0.303749171	stable	LILRA6
0.303779352	stable	LOC650528
0.303779352	stable	TRIMP1
0.303928711	stable	OSTM1
0.303931067	stable	MYO1E
0.304116481	stable	PTCRA
0.304466558	stable	FAM9C
0.304510586	stable	NAT5
0.304703249	stable	C16ORF48
0.305375242	stable	LOC401097
0.305509727	stable	ANKRD30A
0.305509727	stable	GRK1
0.305509727	stable	PRKRA
0.305732008	stable	BATF2
0.305883416	stable	EPB41
0.305883416	stable	LOC643304
0.305892994	stable	KGFLP1
0.305971935	stable	LOC651721
0.305988581	stable	KIAA0226
0.306034605	stable	ZNF650
0.306034605	stable	NOD3
0.30604	stable	THEM5
0.306089774	stable	CNGA3
0.306214339	stable	PLAGL2
0.306574339	stable	PAQR8
0.306683097	stable	LOC647041
0.306915055	stable	LOC440356
0.306956689	stable	ROBO1
0.306983098	stable	TRIM6
0.307155315	stable	F10
0.307197095	stable	ZNF571
0.307553982	stable	LOC654118
0.307612053	stable	PTPLAD2
0.307731195	stable	TPRX1
0.307755369	stable	TH

0.307877191	stable	LOC731158
0.307891439	stable	EVX1
0.308001668	stable	LOC642447
0.308011332	stable	LOC440233
0.308138249	stable	LOC388946
0.308378358	stable	PEX11A
0.308423957	stable	LOC440509
0.308423957	stable	DPEP2
0.308496957	stable	CHRNA4
0.308496957	stable	LOC643854
0.308598027	stable	OR5K3
0.308598027	stable	CHRM5
0.308668699	stable	LOC651905
0.308672153	stable	LOC643154
0.30871457	stable	USP39
0.308782631	stable	HJURP
0.308819923	stable	PWP1
0.308985368	stable	FRMPD1
0.309423256	stable	MYLK2
0.309423256	stable	FLJ38379
0.309494551	stable	STAG1
0.309494551	stable	SNX6
0.309795464	stable	IL28B
0.309795464	stable	ETV2
0.309795464	stable	C14ORF37
0.309873842	stable	BHLHB8
0.309873842	stable	PTPRF
0.309957823	stable	NLK
0.309978938	stable	CILP
0.310330713	stable	LOC650148
0.310330713	stable	BIRC2
0.310391851	stable	CHMP4C
0.310518722	stable	LOC641801
0.310541854	stable	C18ORF19
0.310563181	stable	LOC730818
0.310599705	stable	CAMK4
0.310785892	stable	DKFZP434N035
0.310785927	stable	TEX11
0.310800299	stable	CEACAM16
0.310843737	stable	LOC643869
0.310901169	stable	FAM173A
0.310921958	stable	LOC643256
0.310983926	stable	LOC644992
0.310983926	stable	ARL17P1
0.310983926	stable	SMPDL3B
0.31110818	stable	TXNDC17
0.31138929	stable	TRIM69
0.311392887	stable	ZNF98
0.311503438	stable	TNFAIP8L1
0.311525535	stable	SLC35D3
0.311550425	stable	CACNA1C

0.311581095	stable	C3ORF57
0.311581095	stable	LSM14B
0.311632112	stable	C19ORF70
0.311642656	stable	HNRNPA0
0.311878268	stable	C1ORF114
0.311911028	stable	LOC652784
0.311911028	stable	OR6V1
0.312245344	stable	OBP2B
0.312415954	stable	NCF1C
0.312461969	stable	LOC644763
0.312461969	stable	SERPINA10
0.312461969	stable	SOS1
0.31250678	stable	NMUR2
0.31254461	stable	LOC649037
0.312619057	stable	GCSH
0.312672965	stable	LOC650812
0.3128165	stable	COL2A1
0.312917038	stable	RGL4
0.312974483	stable	LOC650708
0.313079807	stable	PWWP2A
0.313129953	stable	LOC642712
0.313181007	stable	DLK1
0.313181007	stable	LOC201164
0.313244866	stable	PDAP1
0.313308276	stable	CCBP2
0.313329241	stable	PBX4
0.313518578	stable	MGC5457
0.313620594	stable	GLCCI1
0.314097886	stable	OR51I1
0.31412047	stable	C8ORF49
0.314232523	stable	NAT13
0.314250368	stable	HADHA
0.314250368	stable	LOC645262
0.314250368	stable	CYS1
0.314309835	stable	XKRY
0.314471316	stable	SNIP1
0.314475413	stable	CFP
0.314529182	stable	PCP4
0.314529182	stable	LOC651192
0.31466188	stable	OR9I1
0.314796258	stable	C2ORF25
0.314863136	stable	LOC646388
0.314883485	stable	C11ORF41
0.314883485	stable	ZFHX4
0.314883485	stable	EIF3M
0.314967222	stable	LOC650428
0.315055744	stable	TXNL1
0.315121097	stable	LOC652883
0.315213556	stable	LOC647520
0.315253163	stable	LOC643147
0.315310755	stable	PCDHB3

0.315310755	stable	LOC285900
0.315444538	stable	FAM134C
0.315484395	stable	RET
0.315541621	stable	LOC644974
0.315747634	stable	CEP55
0.315822766	stable	TTPA
0.315868733	stable	KLB
0.315868733	stable	PCDHGC3
0.316174333	stable	LOC650274
0.316174333	stable	FLJ12993
0.316229633	stable	LOC646256
0.316229633	stable	FBLIM1
0.316231208	stable	ECEL1
0.316242306	stable	LOC390349
0.31628013	stable	LOC647037
0.316333499	stable	NBPF12
0.31636461	stable	LOC649385
0.316428762	stable	FAM18B
0.316433182	stable	CST9L
0.316528963	stable	TPX2
0.3167926	stable	LOC650985
0.3167926	stable	ZNF565
0.316848141	stable	GLS
0.316863795	stable	LOC730196
0.316977198	stable	CNTN3
0.317016027	stable	LOC643416
0.317094592	stable	LOC646625
0.317239385	stable	LOC730243
0.317239385	stable	IKZF4
0.317239385	stable	CHST2
0.317414293	stable	BNIP2
0.317425273	stable	LOC642419
0.317468097	stable	C9ORF156
0.317766485	stable	BAGE2
0.317940836	stable	B4GALT2
0.31797181	stable	CCL28
0.317986627	stable	TARDBP
0.318357984	stable	GTF2H3
0.318424125	stable	PRRC1
0.318495529	stable	OR4D2
0.318567898	stable	NFYB
0.31866935	stable	KIF5A
0.318713581	stable	LOC644450
0.318713581	stable	FLJ36157
0.318846075	stable	LOC399898
0.318919078	stable	FBXO15
0.319037092	stable	SNIP
0.319141268	stable	LOC146325
0.31928167	stable	KRTHB6
0.31928167	stable	SESN3
0.319407434	stable	FAM168B

0.319417316	stable	AVPR1B
0.319436064	stable	LOC652435
0.319626005	stable	CEP110
0.319626005	stable	LOC441031
0.319647212	stable	POLD1
0.319788696	stable	SLC9A10
0.319788696	stable	ABCB9
0.31998119	stable	LOC653674
0.320062067	stable	LOC727791
0.320062067	stable	R3HDM1
0.320164583	stable	ZNRF2
0.320164583	stable	C9ORF131
0.320194612	stable	LOC644204
0.320254104	stable	KIAA2022
0.320457328	stable	LOC650684
0.320457328	stable	IPF1
0.320528061	stable	LOC644808
0.320688816	stable	LOC641941
0.320688816	stable	SH2D6
0.320720942	stable	RTN4RL1
0.320818391	stable	PAM
0.320902436	stable	LOC645454
0.320961899	stable	KBTBD8
0.321009048	stable	OR6K2
0.321297839	stable	NFS1
0.321302621	stable	GAD2
0.321591209	stable	DNAJC22
0.32166307	stable	SSX2IP
0.321681551	stable	CCL15
0.321801508	stable	NOTO
0.321801508	stable	ABCC1
0.321809603	stable	OR7A17
0.321894147	stable	LOC642788
0.32190545	stable	LOC388199
0.32190545	stable	SQSTM1
0.321953606	stable	CHST12
0.321953606	stable	INS
0.321953606	stable	HNRPA1P4
0.321978812	stable	PCDHGB6
0.322167485	stable	TTTY20
0.322352884	stable	LOC647819
0.322469632	stable	KLF11
0.322520833	stable	UGT2B4
0.322521672	stable	DHH
0.322609517	stable	LOC648226
0.322698867	stable	NUDT22
0.322770155	stable	KIAA0692
0.32278428	stable	GNRH2
0.322813328	stable	SPACA4
0.322896042	stable	RAD23A
0.322944586	stable	C6ORF138

0.323218437	stable	WIF1
0.323561944	stable	LMAN2
0.323589218	stable	LOC390231
0.323592091	stable	MAOA
0.323671214	stable	PAQR7
0.32380176	stable	LOC645971
0.323811023	stable	GGA3
0.323905048	stable	FGF17
0.323905048	stable	LOC402538
0.323905048	stable	SAP30
0.323970127	stable	XRCC6
0.324034004	stable	ST6GAL2
0.32404787	stable	DNAJC1
0.324128631	stable	LOC647785
0.324211467	stable	MMP1
0.324236366	stable	LGALS3
0.324316447	stable	CYP8B1
0.324316447	stable	ZSCAN16
0.324668929	stable	GZF1
0.324668929	stable	LOC442206
0.324668929	stable	KIAA0773
0.324782284	stable	C9ORF93
0.324792833	stable	EIF2AK3
0.324900772	stable	PCSK7
0.324900772	stable	POLR3K
0.324900772	stable	ZBTB37
0.325162526	stable	ADAM6
0.325162526	stable	DUSP22
0.325283399	stable	GTF3C6
0.325305837	stable	SEC14L3
0.32551416	stable	AFAP1
0.325554627	stable	ERGIC1
0.325588156	stable	LOC402217
0.325612777	stable	ADAMTSL2
0.32562352	stable	BRDG1
0.325810644	stable	ATP2B2
0.325898758	stable	BIRC8
0.325952026	stable	LOC647044
0.326097417	stable	CDC2L2
0.326097417	stable	RERE
0.326129792	stable	COMMD2
0.326155672	stable	AMFR
0.326155672	stable	C20ORF173
0.326200717	stable	SLC9A8
0.326200717	stable	DRD1IP
0.326268085	stable	ADM
0.326302323	stable	TRIM64
0.326325064	stable	LOC642187
0.326325064	stable	LOC654165
0.326325064	stable	ST8SIA1
0.326380088	stable	TH1L

0.326544292	stable	TAS2R43
0.326676952	stable	PRNT
0.326783315	stable	C14ORF68
0.326783315	stable	LOC132241
0.326861798	stable	LOC729008
0.326861798	stable	LOC643814
0.326935553	stable	SERINC5
0.327008006	stable	LOC347487
0.327117697	stable	RAB3C
0.327242073	stable	LOC283710
0.3273574	stable	FBXO7
0.3273574	stable	FLJ43276
0.327409069	stable	HIST2H2BE
0.327409069	stable	KRTAP10-10
0.327409069	stable	KLRA1
0.327456667	stable	MAML2
0.327494437	stable	INCENP
0.327513103	stable	ACRBP
0.327590196	stable	FLJ40712
0.327616523	stable	IRX1
0.327616523	stable	LOC648223
0.327631233	stable	C16ORF50
0.327647844	stable	FTHL2
0.327722752	stable	CES3
0.327746559	stable	LOC165186
0.327781702	stable	CDH16
0.327867238	stable	LOC388532
0.327920808	stable	MAGEA2B
0.327964636	stable	SOS2
0.327979426	stable	SPACA5
0.327979426	stable	SNORD32A
0.327979426	stable	LOC647254
0.327979426	stable	LOC646615
0.328034387	stable	LOC643621
0.328095411	stable	LOC441212
0.328095411	stable	LOC652764
0.328142315	stable	TMEM213
0.3281822	stable	EFNA5
0.328274886	stable	ACTL6A
0.328361823	stable	SPSB1
0.328583141	stable	MGC26718
0.328583141	stable	TMEM195
0.328640361	stable	LOC644313
0.328893004	stable	PRRT2
0.328893004	stable	LOC399978
0.328893004	stable	LOC648829
0.328928817	stable	OR1C1
0.329019243	stable	C1ORF124
0.329031652	stable	LOC649389
0.329134249	stable	HHCM
0.329134249	stable	LOC652526

0.329372272	stable	KRT74
0.329743326	stable	LOC390414
0.329743326	stable	PRELID2
0.329979986	stable	SDF2
0.330060726	stable	LOC728636
0.330122081	stable	FLJ41821
0.330289668	stable	PLCH2
0.330500175	stable	SAMD11
0.330673394	stable	CRYBB1
0.3307387	stable	MXRA8
0.330744776	stable	PET112L
0.330876952	stable	C16ORF74
0.330955282	stable	SPTLC2
0.331072338	stable	IBTK
0.331072338	stable	CBWD1
0.331072338	stable	LOC642245
0.33113694	stable	SOX2
0.331512782	stable	NGRN
0.331596225	stable	PLK1
0.331722316	stable	LOC648987
0.331921398	stable	ZDHHC1
0.332158804	stable	CDKL4
0.332158804	stable	LOC644602
0.332168996	stable	PRDM8
0.332493486	stable	ABCB10
0.33250604	stable	LOC643475
0.332533033	stable	LOC652640
0.332533033	stable	TCTEX1D4
0.332533382	stable	KIR2DL3
0.332643844	stable	CIRBP
0.332707384	stable	GRM3
0.33296835	stable	PROL1
0.333073213	stable	OR5H15
0.333073213	stable	ARMC6
0.333352769	stable	LOC643060
0.3333712	stable	TMPRSS3
0.333421336	stable	LOC643161
0.333426763	stable	TMCC2
0.333461047	stable	PAQR3
0.333568419	stable	ZNF214
0.333568419	stable	LOC339746
0.333762222	stable	POLRMT
0.333762222	stable	TIMM50
0.333819949	stable	LOC641704
0.333924479	stable	HADHB
0.333938205	stable	LOC442597
0.333952624	stable	OTOP3
0.334128928	stable	LOC645089
0.334137934	stable	RANBP3L
0.334137934	stable	RHCG
0.334301066	stable	C2ORF67

0.334390079	stable	AKT1
0.334583745	stable	BICD1
0.334612686	stable	ATP6V1E2
0.334668883	stable	MCHR2
0.334668883	stable	LOC646097
0.334742105	stable	RIT2
0.334742781	stable	LOC644005
0.334768474	stable	IRF1
0.334888402	stable	DIMT1L
0.335051788	stable	ODZ2
0.335187558	stable	CGB8
0.335234735	stable	SORCS2
0.335269536	stable	LOC647673
0.335324381	stable	C1ORF137
0.335380791	stable	DNMT3A
0.335586738	stable	LOC646100
0.335688722	stable	LOC653841
0.335706299	stable	KIAA0408
0.335728396	stable	TBCEL
0.335728396	stable	KIAA0907
0.33582873	stable	LOC651603
0.336115359	stable	TIGD6
0.336239693	stable	OR4K2
0.336324303	stable	LOC649667
0.336402045	stable	ANKRD16
0.336402045	stable	HAS1
0.336614769	stable	MEX3C
0.336614769	stable	LOC203510
0.336633565	stable	LOC647468
0.336813854	stable	DOHH
0.336857666	stable	DUSP21
0.336905778	stable	NR6A1
0.336905778	stable	LOC645254
0.337098976	stable	RAB11FIP2
0.337098976	stable	DNASE2B
0.337170344	stable	C8ORF16
0.337194521	stable	LOC642003
0.337194521	stable	C5ORF24
0.337194521	stable	LOC730109
0.337281717	stable	ARHGAP18
0.337351635	stable	CNP
0.337402817	stable	LOC644669
0.337498211	stable	WDR7
0.337500623	stable	C10ORF2
0.337633055	stable	SSX9
0.337844568	stable	TMEM86B
0.337844568	stable	IFI44L
0.337844568	stable	LOC730347
0.337900065	stable	DLG5
0.338020465	stable	RHBDD3
0.338165818	stable	RNF169

0.338277054	stable	KCNB2
0.338334615	stable	SOCS5
0.338334615	stable	FAT2
0.338334615	stable	EPRS
0.338439616	stable	JOSD2
0.338714925	stable	LOC652664
0.33884571	stable	BPIL3
0.338870622	stable	TAL2
0.338909975	stable	C15ORF32
0.338909975	stable	ASNA1
0.338920843	stable	IL1F8
0.338926535	stable	STARD4
0.33893611	stable	ITGBL1
0.339022066	stable	RBM10
0.339022066	stable	SPDEF
0.339022066	stable	GABRB2
0.339022066	stable	H2BFM
0.339053496	stable	SERGEF
0.339061702	stable	C1ORF116
0.339061702	stable	SNHG8
0.339061702	stable	RESP18
0.339324357	stable	CCDC96
0.339372464	stable	LOC646155
0.339402981	stable	NR2F2
0.339585557	stable	C12ORF41
0.3398798	stable	PDE12
0.340044845	stable	SERPINB3
0.34011808	stable	SIPA1L1
0.3401225	stable	CCDC50
0.340357814	stable	TTC16
0.34042974	stable	TMEM41A
0.34042974	stable	DHFR
0.340442555	stable	PMFBP1
0.340442555	stable	MEG3
0.340442555	stable	TAS1R1
0.340459534	stable	ZIC1
0.340534542	stable	HTRA2
0.340624721	stable	HAR1A
0.340624721	stable	TMEM95
0.340624721	stable	LOC652523
0.340732285	stable	C17ORF97
0.340934682	stable	NPM1
0.340980341	stable	COMT
0.340980341	stable	ARSI
0.340999949	stable	CYP11B2
0.341028489	stable	LOC389523
0.341315403	stable	LOC648999
0.341315403	stable	MC3R
0.34137375	stable	PRG3
0.341490688	stable	LOC650771
0.341490688	stable	OR4K1

0.341539554	stable	LOC643844
0.341575724	stable	LOC645712
0.341802258	stable	FMR1NB
0.342034662	stable	LOC124685
0.342086627	stable	LOC728554
0.342285949	stable	ARRB1
0.342404671	stable	AP4M1
0.342420378	stable	KTI12
0.342462602	stable	MLL4
0.342462602	stable	YAP1
0.342505182	stable	BRD2
0.342625764	stable	LOC645648
0.342765591	stable	FAM117A
0.342842406	stable	BAP1
0.343041716	stable	NHLH2
0.343054063	stable	LOC652759
0.343279896	stable	NSUN4
0.343350524	stable	RANBP2
0.343459212	stable	LOC646194
0.34346211	stable	FSCB
0.343463133	stable	ZNF43
0.343463133	stable	HOXC8
0.343546197	stable	ARL2
0.343546197	stable	LOC645116
0.343546197	stable	C9ORF7
0.343546197	stable	TKTL1
0.343546197	stable	OFD1
0.343666786	stable	LOC400145
0.343666786	stable	NUP188
0.343701097	stable	LOC648667
0.343701097	stable	NCAPG
0.343857709	stable	LOC644873
0.343857709	stable	LOC644098
0.34399556	stable	ZSWIM1
0.344152756	stable	UCK1
0.344152756	stable	C2ORF65
0.344152756	stable	MYO10
0.344274669	stable	LOC652227
0.344274669	stable	PIGC
0.344283406	stable	C21ORF90
0.344334387	stable	LOC645330
0.344518769	stable	MAPK14
0.344589361	stable	LOC440927
0.344589361	stable	MGC16025
0.344893517	stable	SPINK7
0.344898685	stable	ZDHHC4
0.344898685	stable	PARP12
0.344901036	stable	LOC387940
0.345210909	stable	LOC652696
0.345210909	stable	WFDC2
0.345210909	stable	NPM2

0.345210909	stable	LOC284672
0.345210909	stable	AMN1
0.345215461	stable	KRTAP17-1
0.345245545	stable	TCEB3C
0.345245545	stable	GTPBP3
0.345245545	stable	PCDHGA11
0.345254895	stable	CAB39
0.345280877	stable	LOC647854
0.345353989	stable	LOC651126
0.345368076	stable	LOC653920
0.34543754	stable	LOC401957
0.345504484	stable	GNB5
0.345554764	stable	TRPM1
0.345554764	stable	LOC644710
0.345568934	stable	PLAC2
0.345568934	stable	PRAMEF10
0.345568934	stable	YDJC
0.345864765	stable	LOC387723
0.345899539	stable	MGC87631
0.345912266	stable	C6ORF65
0.346054323	stable	LOC648745
0.3463018	stable	LOC653188
0.346440135	stable	SPATS2
0.346440135	stable	OVOL1
0.346512763	stable	TREML2P
0.346556235	stable	LOC648003
0.346625024	stable	SMG5
0.346736046	stable	SHROOM1
0.346736046	stable	LOC649493
0.346969403	stable	LOC643072
0.346969403	stable	LOC392563
0.346969403	stable	OR2AG2
0.346969403	stable	PAPPA2
0.346969403	stable	LOC643266
0.346976558	stable	TAS2R1
0.346999344	stable	GOLGA8F
0.347170021	stable	WDR81
0.347357115	stable	SPATA22
0.347357115	stable	TMEM108
0.347375066	stable	LHX3
0.347375066	stable	C9ORF167
0.347451709	stable	C10ORF99
0.347451709	stable	FUBP1
0.347451709	stable	RPS26L1
0.347459931	stable	FLJ46361
0.347515197	stable	LOC388344
0.347687455	stable	SF3A1
0.347739883	stable	OAS3
0.347739883	stable	OR10W1
0.34788817	stable	KRTAP24-1
0.34788817	stable	POU6F1

0.34788817	stable	FLJ35816
0.34788817	stable	NTHL1
0.348330507	stable	CDK7
0.348330507	stable	CEP72
0.348393287	stable	C9ORF14
0.348501531	stable	IL1F6
0.348569291	stable	CXORF40A
0.34857817	stable	HTR3A
0.34869738	stable	GSK3B
0.34869738	stable	PLA2G4D
0.348781976	stable	NR2F1
0.348868204	stable	RPL26
0.349079702	stable	PCDHA4
0.349220828	stable	TERF2
0.349220828	stable	LOC650203
0.349409993	stable	PKD2L1
0.349417969	stable	LOC647710
0.349417969	stable	RGMB
0.349537713	stable	OR10H1
0.349537713	stable	OR4F16
0.349537713	stable	TMEM81
0.349649519	stable	OXGR1
0.349736517	stable	FLJ45983
0.349760309	stable	ZNF24
0.349760309	stable	PAX5
0.349760309	stable	GPR174
0.350084469	stable	LOC100101121
0.350084469	stable	FLJ43860
0.35029604	stable	RP13-360B22.2
0.35029604	stable	LOC441150
0.350375191	stable	LOC644989
0.350405536	stable	IQCF3
0.350492012	stable	UBA52
0.350922163	stable	AMPD3
0.350922163	stable	C1ORF110
0.350949253	stable	LOC642916
0.350951307	stable	LOC402569
0.351051417	stable	LSM10
0.351376098	stable	LOC730953
0.351382041	stable	LOC650061
0.351588321	stable	LOC644841
0.351690924	stable	KRTAP10-3
0.351706179	stable	TBPL1
0.351730769	stable	LOC391766
0.351892213	stable	CPM
0.352027938	stable	LOC730049
0.352042958	stable	FAM135A
0.352108344	stable	C20ORF55
0.352115186	stable	ESCO1
0.352136773	stable	BTBD7
0.352315661	stable	GPR55

0.352475265	stable	FLJ25037
0.352681933	stable	LANCL3
0.352702183	stable	MRPS7
0.352702183	stable	KIAA1486
0.352770775	stable	MAGEB5
0.353391872	stable	PNRC1
0.353407231	stable	MGC4294
0.353407231	stable	LOC645045
0.353407231	stable	FLJ44048
0.353407231	stable	ST3GAL6
0.353407231	stable	QARS
0.353624967	stable	SGEF
0.353624967	stable	HPS3
0.353624967	stable	MGC102966
0.353682126	stable	OMG
0.353789626	stable	STYX
0.353859218	stable	LALBA
0.354163389	stable	POLDIP3
0.354169799	stable	C10ORF51
0.354226943	stable	LOC441925
0.354262528	stable	UMPS
0.354262528	stable	CHAF1B
0.354296491	stable	LOC649683
0.354296491	stable	BIK
0.354296491	stable	AOC3
0.354482945	stable	FAM86A
0.354489364	stable	ANO6
0.354575523	stable	CYP4X1
0.354606728	stable	LOC643608
0.35468633	stable	LOC442204
0.35501047	stable	KIF5C
0.355239874	stable	COMTD1
0.355398053	stable	KIAA0895
0.355435884	stable	SSNA1
0.355548811	stable	LOC643169
0.355571873	stable	SS18L2
0.355722951	stable	LOC651752
0.355742212	stable	LOC642355
0.355742212	stable	TMEM50A
0.35582793	stable	CXCL11
0.355859869	stable	UBE2D1
0.356015016	stable	LRRC29
0.356067769	stable	ILDR1
0.356145569	stable	DRGX
0.356193532	stable	ATPBD1B
0.356199209	stable	LOC653524
0.356199471	stable	LOC642863
0.356199471	stable	WDR87
0.356201506	stable	LOC644291
0.35651808	stable	LOC645282
0.356540268	stable	OR4A16

0.356645444	stable	ZNF500
0.356818857	stable	DCTD
0.356935926	stable	LOC641742
0.356988566	stable	LOC440368
0.357043503	stable	LOC729732
0.357065509	stable	OGDH
0.357103049	stable	SPAG4
0.357383283	stable	LOC641744
0.357383283	stable	GPR137
0.357405681	stable	FLT3LG
0.357463447	stable	SPINK2
0.357499964	stable	NOP5/NOP58
0.357501231	stable	KRT32
0.357501471	stable	BCL2L10
0.357581065	stable	PSMAL
0.357728798	stable	MRGPRX2
0.357783811	stable	GULP1
0.357811015	stable	HSD17B12
0.357854016	stable	C1ORF66
0.357920018	stable	LOC644656
0.357920018	stable	ELAVL4
0.357960478	stable	THOC3
0.357999249	stable	ZNF619
0.357999249	stable	LOC644986
0.358003817	stable	PROZ
0.358045002	stable	CENPF
0.358075312	stable	GLYATL2
0.358254787	stable	RP11-49G10.8
0.358499121	stable	TNFRSF11B
0.358537076	stable	HIST1H2BE
0.358611932	stable	LOC650780
0.358683784	stable	LOC644286
0.358862002	stable	DNMBP
0.358862002	stable	UCN3
0.359170575	stable	TRY1
0.359191282	stable	LOC645141
0.35927007	stable	KIAA1549
0.359324522	stable	LOC401237
0.359324522	stable	LOC645958
0.359647718	stable	C10ORF91
0.359671122	stable	SPAG11B
0.359790913	stable	KRTAP5-3
0.359998805	stable	MRPS5
0.360028542	stable	KIF19
0.3600413	stable	SCARNA15
0.3600413	stable	LOC646568
0.3600413	stable	C9ORF85
0.360114795	stable	LOC390110
0.360139491	stable	LOC646008
0.360573937	stable	UNC5CL
0.360578796	stable	MID2

0.360729929	stable	LOC643761
0.360754172	stable	HAL
0.360806755	stable	LOC392196
0.360884804	stable	LOC645718
0.361099978	stable	NSUN5B
0.36114594	stable	COLEC10
0.361146333	stable	LOC647181
0.361229949	stable	LOC647513
0.361321737	stable	LOC644516
0.361337475	stable	FXYD6
0.361337475	stable	DDX19-DDX19L
0.36134007	stable	LOC139735
0.361499058	stable	LOC644144
0.361624626	stable	FKBP15
0.361643088	stable	ACTN1
0.361643088	stable	LOC388481
0.361757158	stable	PDK1
0.36185303	stable	CEACAM7
0.362008752	stable	PRED57
0.362008752	stable	RBMY1A3P
0.362055719	stable	LOC642989
0.362084961	stable	BPGM
0.362215088	stable	ZNF587
0.362305255	stable	FLJ13611
0.362415858	stable	LOC642934
0.362611413	stable	PREI3
0.362611413	stable	LOC654258
0.362629468	stable	WDR78
0.362773681	stable	LOC641823
0.362850487	stable	OR2A5
0.362850487	stable	RPL7
0.362863716	stable	LOC647347
0.363224905	stable	FLG2
0.36327644	stable	XKR3
0.363468768	stable	ALPK3
0.363468768	stable	DNAI1
0.363468768	stable	SYT2
0.363468768	stable	ATP6V0A4
0.363468768	stable	DISC2
0.363835692	stable	SNORD18C
0.363882728	stable	DUSP7
0.363998848	stable	LOC646813
0.364074194	stable	NUDT8
0.364089237	stable	GGT3P
0.364178044	stable	MAT1A
0.364359062	stable	MIPEP
0.364569722	stable	LOC652831
0.364644232	stable	SERPINA4
0.364659872	stable	LOC646734
0.364677188	stable	GIT1
0.364677188	stable	MRC1

0.364700613	stable	LOC644175
0.364839057	stable	STXBP4
0.364839057	stable	MYB
0.364993433	stable	LOC644621
0.364993433	stable	CHMP1A
0.365054269	stable	TMOD2
0.365129892	stable	LOC644948
0.365403014	stable	CALCA
0.365509187	stable	CARD6
0.365602128	stable	LOC285442
0.365602128	stable	KIR2DL5B
0.365650559	stable	MGC50722
0.365731072	stable	EPX
0.365854523	stable	C11ORF72
0.36587743	stable	ZNF296
0.36587743	stable	PREB
0.366010726	stable	SMC1A
0.366298042	stable	LOC442180
0.366313906	stable	LOC649292
0.366390426	stable	LENG1
0.366475991	stable	FLJ40411
0.366503062	stable	LOC651511
0.366980261	stable	OR2A25
0.367026291	stable	LOC440918
0.367052167	stable	C12ORF61
0.367174501	stable	ZNF75
0.367273587	stable	MICALCL
0.367341589	stable	TAS2R41
0.367453352	stable	SRFBP1
0.367501633	stable	ATP9B
0.367501633	stable	C10ORF55
0.367616782	stable	LOC284417
0.367758415	stable	ELA2B
0.36789705	stable	GLYATL1
0.367940196	stable	LOC401180
0.367956779	stable	TFCP2L1
0.367956779	stable	MYBPC1
0.367956779	stable	GGTLC1
0.367956779	stable	LOC442124
0.367956779	stable	EPHA5
0.367956779	stable	CCL16
0.368122978	stable	TMEM52
0.368211245	stable	LOC650342
0.368211245	stable	LOC642148
0.368211245	stable	LOC644086
0.368211245	stable	PADI1
0.36822038	stable	LOC642399
0.368242785	stable	ASZ1
0.368402667	stable	KRTAP19-3
0.368510382	stable	LOC646570
0.368548546	stable	LOC647225

0.368548546	stable	MMP24
0.368608319	stable	AMMECR1L
0.368703925	stable	MAML1
0.368703925	stable	CGB1
0.368780927	stable	FOXJ2
0.368780927	stable	THAP8
0.368780927	stable	TAF7
0.368788776	stable	TMEM166
0.36879878	stable	PSMA6
0.368876253	stable	NKX6-2
0.368902771	stable	TIAF1
0.369012064	stable	LOC652501
0.369055617	stable	SVOPL
0.369149642	stable	TNFRSF10C
0.369149642	stable	LOC643868
0.369149642	stable	AGBL3
0.369173426	stable	BTBD3
0.369202344	stable	LOC647460
0.369203596	stable	GMCL1L
0.369203596	stable	C14ORF78
0.369254216	stable	LOC643273
0.36927641	stable	LOC643248
0.369364461	stable	LOC442560
0.36944233	stable	LIPJ
0.36944233	stable	KRTAP5-4
0.369674853	stable	LOC645078
0.370076928	stable	PDZD8
0.370139609	stable	CASP12
0.370218571	stable	DEPDC7
0.370237673	stable	LOC651197
0.370237673	stable	KIAA1161
0.370237673	stable	KRT36
0.370237673	stable	GABRA3
0.370335428	stable	LOC650804
0.370391468	stable	NUDCD2
0.370391468	stable	AGR3
0.370391468	stable	PRAMEF7
0.370391468	stable	SC4MOL
0.370391468	stable	FLJ32255
0.370458134	stable	SEZ6
0.37047109	stable	LOC653570
0.370665433	stable	KLF1
0.370755712	stable	LOC652869
0.370845635	stable	ZDHHC23
0.370872332	stable	LOC646190
0.370994107	stable	CYP2D6
0.371117653	stable	DSTN
0.371308375	stable	LOC643344
0.371360304	stable	LOC652078
0.371360304	stable	ATPBD1C
0.371439757	stable	RAD9B

0.37146575	stable	SCN1A
0.371848432	stable	LOC642210
0.371876125	stable	BEST4
0.372227815	stable	LOC648645
0.372227815	stable	NANOS2
0.372311702	stable	LGI3
0.372313996	stable	LOC645545
0.372355686	stable	LOC650095
0.372360629	stable	ESPL1
0.372675132	stable	LOC652153
0.372677335	stable	ZDHHC11
0.372709075	stable	AZU1
0.372715814	stable	TMOD1
0.372741975	stable	CHRM4
0.372812466	stable	GIPC3
0.372958885	stable	LOC441876
0.373140594	stable	ZNF711
0.373183751	stable	PCP2
0.373212379	stable	LOC441709
0.373227701	stable	C6ORF128
0.373242182	stable	LOC652882
0.373499631	stable	LOC648148
0.373537442	stable	LOC650770
0.373627426	stable	LOC643738
0.373627426	stable	HMBOX1
0.373676157	stable	C10ORF65
0.373762165	stable	MSMB
0.373762165	stable	UTY
0.373762165	stable	LOC649826
0.373762165	stable	OR2M4
0.373830105	stable	LOC642521
0.373981329	stable	C6ORF148
0.374038956	stable	IRF3
0.374047438	stable	CACNA1S
0.374047438	stable	NRN1L
0.374047438	stable	LOC650517
0.374047438	stable	OR4C15
0.374047438	stable	LOC648205
0.374162336	stable	SRCAP
0.374172636	stable	IGSF10
0.374199485	stable	OTUD5
0.37421999	stable	LOC644536
0.374242429	stable	LOC642620
0.374242429	stable	LOC647488
0.37436384	stable	FUCA1
0.37436384	stable	CCL27
0.374728746	stable	FLJ21767
0.374902465	stable	COBL
0.375181144	stable	MIOX
0.375181144	stable	ZNF578
0.375272968	stable	LOC652147

0.375327604	stable	LOC648982
0.375327604	stable	MFAP2
0.375327604	stable	CLCA2
0.375343512	stable	RBBP4
0.375441783	stable	MGC50273
0.375494874	stable	LOC647531
0.375497753	stable	VWCE
0.375500064	stable	MT1P2
0.375592794	stable	LOC641743
0.37573485	stable	PRDM10
0.375835722	stable	RPL32P3
0.376001887	stable	LOC646442
0.376100584	stable	LOC646149
0.376253428	stable	STK32A
0.376451083	stable	FBXL5
0.376451083	stable	LOC647028
0.376507437	stable	FLJ45721
0.376507437	stable	MYH15
0.376691638	stable	ZNRF1
0.376933993	stable	C2CD2L
0.37698213	stable	C17ORF92
0.37698213	stable	LOC732165
0.377080869	stable	LOC644033
0.37711216	stable	RAB5B
0.37718014	stable	LOC644768
0.377328393	stable	SLC8A2
0.377328393	stable	LOC653352
0.377343757	stable	KCNQ2
0.37735065	stable	WDR60
0.377667596	stable	LOC653200
0.377754504	stable	CNNM1
0.378049779	stable	KRTAP5-6
0.378134393	stable	OR56A4
0.378203704	stable	CDH7
0.378203704	stable	C9ORF27
0.378203704	stable	LOC653632
0.378429952	stable	LOC645289
0.378641486	stable	LOC644631
0.378641486	stable	RWDD2B
0.378641486	stable	KCNJ11
0.378693629	stable	LOC653887
0.378693629	stable	HDAC10
0.378693629	stable	TFAP2D
0.378813173	stable	TUT1
0.378816624	stable	UHRF1BP1L
0.379048073	stable	ZNF557
0.379158309	stable	VMO1
0.379288904	stable	ITGB5
0.379319204	stable	TET3
0.379364719	stable	FER1L4
0.379391268	stable	GCET2

0.379437358	stable	LOC645553
0.379437358	stable	LOC650584
0.379437358	stable	LOC650463
0.379487661	stable	ZNRD1
0.379578214	stable	CHML
0.379635672	stable	KIAA0565
0.379720165	stable	LOC728601
0.379846119	stable	LOC728910
0.379926096	stable	ITGB1
0.379944462	stable	CLDN22
0.380072306	stable	LOC643570
0.380099374	stable	NUP50
0.380105962	stable	ZNF646
0.380105962	stable	LOC440776
0.380105962	stable	MGC45922
0.380105962	stable	GUK1
0.380107095	stable	TMEM190
0.380208101	stable	ADRA1D
0.380217157	stable	MAB21L1
0.380335557	stable	JMJD2D
0.380424633	stable	FLJ23754
0.380468121	stable	LOC652708
0.380468121	stable	TMIGD1
0.380468121	stable	LOC646227
0.380632624	stable	CD164L2
0.380944238	stable	C12ORF36
0.381032523	stable	COG1
0.381095098	stable	LRRC8B
0.381174645	stable	KIAA1604
0.381181949	stable	SNORD16
0.381221774	stable	ZNF789
0.381221774	stable	LOC644647
0.381679341	stable	MOBKL2B
0.381683121	stable	LOC283278
0.381683121	stable	ZBP1
0.381683121	stable	BLNK
0.381683121	stable	MYOHD1
0.381707408	stable	FLJ42393
0.381707408	stable	LOC440525
0.381731954	stable	PATZ1
0.381921189	stable	SUCNR1
0.381921189	stable	MYCBP
0.382006042	stable	CACNG7
0.382133658	stable	LOC652713
0.382133658	stable	LOC642471
0.382133658	stable	OR1L6
0.382225363	stable	LOC339240
0.382233411	stable	LOC653905
0.382300111	stable	CAMTA2
0.382482113	stable	LOC401087
0.38250722	stable	KRTAP5-5

0.38250722	stable	FLJ31813
0.38250722	stable	LOC650433
0.38250722	stable	LOC200493
0.382614002	stable	DOCK4
0.382614002	stable	AGXT2
0.382614002	stable	GTF2IRD2
0.382614002	stable	SC65
0.382614002	stable	NAT12
0.382624793	stable	C2ORF39
0.382751637	stable	MFSD9
0.382789958	stable	PSG5
0.382840816	stable	RAB4A
0.383022964	stable	LOC642181
0.383022964	stable	LOC155060
0.383022964	stable	LOC649021
0.383037245	stable	ANKRD41
0.38330586	stable	LOC653155
0.383395963	stable	PADI2
0.383400053	stable	SFRS14
0.383400053	stable	CDH6
0.383400053	stable	PLXNA2
0.383598048	stable	KLHL18
0.383842171	stable	PDE4A
0.383842171	stable	C20ORF96
0.383842171	stable	LOC196752
0.383903453	stable	LOC642343
0.383903453	stable	LOC647331
0.384299109	stable	LOC375748
0.384299109	stable	LOC441136
0.3843933	stable	LRRC23
0.3843933	stable	DKFZP434K191
0.384682435	stable	LOC644193
0.384993363	stable	FLJ10803
0.385013761	stable	RBM17
0.385029666	stable	LOC646426
0.385072988	stable	F7
0.385072988	stable	FLJ43987
0.385072988	stable	CCDC12
0.385156637	stable	AGBL4
0.38518576	stable	TMEM163
0.38518576	stable	RSPH10B
0.38548481	stable	LOC389676
0.38548481	stable	LOC646345
0.38548481	stable	LOC653563
0.385514037	stable	CT45-4
0.385517105	stable	PLEKHG5
0.385805749	stable	LOC647079
0.385805749	stable	LOC730249
0.385805749	stable	LOC158730
0.38652741	stable	LOC341604
0.38660459	stable	LOC651562

0.38660459	stable	IQCE
0.38660459	stable	OR13G1
0.386607282	stable	LOC653355
0.386611824	stable	DDHD2
0.38667008	stable	LIPL4
0.386677153	stable	EMX2
0.386677153	stable	RPL15
0.386710235	stable	LOC654335
0.386727662	stable	LOC644116
0.386773775	stable	ROS1
0.386773775	stable	LOC340156
0.386878918	stable	LOC646589
0.386915241	stable	KIR3DL1
0.386940303	stable	GRIK1
0.387168968	stable	CD5
0.387210343	stable	RNF112
0.387210343	stable	HERV-FRD
0.387210343	stable	STARD10
0.387227322	stable	NCKAP1
0.387253938	stable	LOC649200
0.38728237	stable	TSPYL4
0.387318174	stable	LOC641721
0.387350999	stable	LOC646970
0.387553418	stable	LOC647606
0.387553418	stable	FLRT1
0.387553418	stable	LOC644001
0.387681558	stable	LOC646835
0.387825977	stable	LOC651344
0.387888863	stable	KCNJ5
0.387979448	stable	LOC653392
0.388173282	stable	FAM151B
0.388270899	stable	ALPK2
0.388353165	stable	DRG2
0.38851653	stable	NR5A2
0.388582181	stable	LOC647406
0.388625844	stable	MGAT5B
0.388699232	stable	LOC260339
0.388741487	stable	LOC653225
0.388785751	stable	SLC5A1
0.388785751	stable	ART5
0.388807983	stable	LY6G5C
0.389378486	stable	ZXDB
0.389393111	stable	DEFB137
0.389694867	stable	RTP2
0.389888616	stable	CABLES1
0.389888616	stable	LRRK1
0.390006385	stable	ASAH3
0.390006385	stable	PPP3R1
0.390006385	stable	CLK3
0.390056642	stable	LRRTM1
0.390124508	stable	LOC645967

0.390243622	stable	LOC649431
0.390322101	stable	SOCS6
0.390590953	stable	LOC641726
0.390647718	stable	LCE3B
0.390653003	stable	MYCBP2
0.390819331	stable	HIST1H2BD
0.390857054	stable	MTMR1
0.390898623	stable	LILRA4
0.390912974	stable	LOC647060
0.391054928	stable	OR4K5
0.39124957	stable	GOT1
0.39137399	stable	LOC654117
0.391499771	stable	LOC643491
0.391733035	stable	LOC648145
0.391733035	stable	KLRC3
0.391733035	stable	TSHB
0.391733035	stable	BAGE4
0.391733035	stable	LRRC37A3
0.391733035	stable	LOC645783
0.391762451	stable	TMEM119
0.391762451	stable	LOC646939
0.391946753	stable	LIPF
0.391972124	stable	LYRM4
0.391991315	stable	CWF19L1
0.392054586	stable	OR10H2
0.392063271	stable	MTFR1
0.392063865	stable	MIA
0.392063865	stable	ARMC8
0.392096577	stable	LOC440348
0.392096577	stable	LOC644083
0.392153128	stable	MORN3
0.392153128	stable	CHSY3
0.392367629	stable	ARL6IP4
0.392367629	stable	LOC390033
0.392367629	stable	HES3
0.392414576	stable	GNPDA2
0.392531738	stable	DNAJA1
0.392629406	stable	SYT16
0.392771988	stable	FLJ23172
0.392854561	stable	ESX1
0.392952579	stable	LOC283585
0.393366836	stable	LOC728395
0.393418284	stable	WDR26
0.39344438	stable	AMIGO1
0.393654608	stable	MRFAP1
0.393743295	stable	C16ORF70
0.393839619	stable	AGTRAP
0.393839619	stable	LOC390714
0.393917985	stable	CLEC14A
0.393940811	stable	LOC649821
0.393940811	stable	CTBP1

0.394091405	stable	C19ORF44
0.394186924	stable	IL17F
0.394253886	stable	LOC440993
0.394540596	stable	LOC652271
0.39467073	stable	CA9
0.39467073	stable	NMU
0.394978994	stable	RP1
0.395103775	stable	FGF3
0.395103775	stable	LCORL
0.395103775	stable	PRR7
0.395103775	stable	LMAN1
0.395103775	stable	EPHX1
0.395269659	stable	MGAT5
0.395270819	stable	PAGE1
0.395288595	stable	LAMP3
0.395408458	stable	FAM126A
0.395432103	stable	NAT15
0.395476485	stable	TSNAXIP1
0.395700244	stable	DKFZP434B061
0.395700338	stable	LOC644672
0.395800737	stable	PSITPTE22
0.395807761	stable	CLIP2
0.395840599	stable	KRT23
0.395850398	stable	LOC650689
0.395946691	stable	AP3S1
0.396129891	stable	LENEP
0.396730773	stable	MGC52282
0.396856885	stable	ZNF579
0.396856885	stable	ADAMTS16
0.396859802	stable	C20ORF165
0.396859802	stable	LOC643904
0.396868124	stable	TDGF1
0.396924919	stable	SFMBT2
0.396924919	stable	PIWIL1
0.396924919	stable	LOC644097
0.396924919	stable	C13ORF3
0.396924919	stable	EFCAB5
0.396924919	stable	LDB2
0.396924919	stable	TFAP4
0.396938575	stable	LOC730417
0.396986133	stable	C20ORF185
0.397014401	stable	CLN5
0.397014401	stable	SCUBE3
0.397095384	stable	FER1L3
0.397274881	stable	SKIL
0.397348265	stable	LOC643895
0.397348265	stable	ZNF585B
0.397348265	stable	DUSP6
0.397348265	stable	CRYBA1
0.397400983	stable	SNCG
0.397636275	stable	TGM3

0.397662674	stable	RNF31
0.397662674	stable	LOC651141
0.397662674	stable	SPOCK3
0.397662674	stable	NAPA
0.397662674	stable	RAD54L
0.397739673	stable	ALK
0.397888741	stable	LOC650167
0.39793032	stable	FBXL7
0.398009144	stable	LOC645212
0.398022404	stable	SLC5A2
0.398022404	stable	LOC391358
0.398469179	stable	GPATCH3
0.398469179	stable	IQCH
0.39868497	stable	LOC139587
0.398761613	stable	LOC653199
0.398791951	stable	KRT26
0.398822723	stable	EDG1
0.398822723	stable	WNK4
0.398957379	stable	MANSC1
0.399011684	stable	MORC4
0.399033195	stable	ZNF527
0.399113264	stable	PREX2
0.399196504	stable	ROR2
0.399368746	stable	LOC440742
0.399398939	stable	METTL6
0.399398939	stable	FSBP
0.399520063	stable	NAPG
0.399520063	stable	TSPYL6
0.399520063	stable	C14ORF115
0.399563898	stable	SEC61G
0.399563898	stable	INO80E
0.399563898	stable	LOC652789
0.399575967	stable	LOC645520
0.399575967	stable	ARFGAP1
0.399758168	stable	LOC644347
0.39987878	stable	LOC652490
0.39987878	stable	LOC649859
0.400005639	stable	DDN
0.400010079	stable	LOC651914
0.400010079	stable	LOC730877
0.400034152	stable	MAGEB16
0.400114278	stable	C1ORF147
0.400198645	stable	LOC651115
0.400280017	stable	C2ORF15
0.400363527	stable	HIST1H2BN
0.400363527	stable	CDC42EP5
0.400648301	stable	LOC440354
0.400648301	stable	LOC645073
0.400730378	stable	NEFH
0.40082318	stable	PTPRB
0.400836294	stable	RPL22L1

0.40085199	stable	ZNF746
0.400990617	stable	PYY
0.400990617	stable	LIFR
0.400990617	stable	LOC643718
0.401354419	stable	WASF3
0.401458977	stable	DSCAM
0.401476555	stable	STYXL1
0.401496626	stable	GCG
0.401496626	stable	SPARC
0.401643489	stable	LOC649680
0.401643489	stable	C8ORF78
0.401757888	stable	IL23A
0.401761554	stable	NASP
0.401761554	stable	RAXL1
0.401761554	stable	LOC730411
0.401761554	stable	CYB5R3
0.401871117	stable	HCN4
0.401922879	stable	CT45-6
0.401922879	stable	G3BP1
0.401922879	stable	S100G
0.401922879	stable	NUDT11
0.401943029	stable	ZNF804A
0.401982268	stable	LOC652400
0.402033576	stable	WDR54
0.402039546	stable	HIST1H3F
0.402050355	stable	STAG3L1
0.402119177	stable	OR1L1
0.40223036	stable	PTP4A3
0.402283267	stable	CNIH2
0.402321639	stable	LOC402279
0.402321639	stable	LOC654256
0.402438411	stable	SSB
0.402438411	stable	THPO
0.402645962	stable	SLC1A2
0.402761063	stable	AMTN
0.403153013	stable	TNFRSF18
0.403156926	stable	PRTG
0.403174483	stable	TNFRSF8
0.403174483	stable	LOC728505
0.403174483	stable	AMH
0.40327277	stable	LOC652645
0.4034722	stable	WDR40B
0.4034722	stable	LOC646774
0.403563282	stable	MT4
0.403598669	stable	PLK3
0.40362288	stable	CD24
0.403689077	stable	HIST4H4
0.403712925	stable	C4ORF6
0.40397819	stable	CLEC4A
0.404047096	stable	ZNF581
0.404099976	stable	ANKRD13D

0.404280967	stable	LOC650494
0.404336226	stable	LOC441127
0.404389991	stable	LOC652680
0.404389991	stable	BCHE
0.404389991	stable	NAT8
0.404389991	stable	CHDH
0.404389991	stable	OR52H1
0.404420784	stable	LOC644916
0.404461259	stable	CCNE1
0.404464147	stable	LOC643318
0.404608569	stable	C2ORF37
0.404608569	stable	LOC727948
0.40463342	stable	ZBTB11
0.404638945	stable	LOC442406
0.404698635	stable	TRPC3
0.404699642	stable	PTPDC1
0.404699642	stable	MOCS3
0.404699642	stable	TAF9
0.404782838	stable	STX1A
0.404985905	stable	HYAL1
0.405051003	stable	CMTM4
0.405068483	stable	LOC653579
0.405080052	stable	GDI2
0.405080052	stable	ANP32E
0.405080052	stable	C15ORF60
0.405128257	stable	GPR63
0.405265415	stable	LGI4
0.405265415	stable	C21ORF66
0.405316551	stable	DKFZP434B0335
0.40532027	stable	ZNF331
0.40532027	stable	FAM71F2
0.405427984	stable	SLC12A5
0.405469892	stable	LOC732372
0.405535965	stable	RTDR1
0.405760808	stable	NPY
0.405760808	stable	LOC644196
0.40576211	stable	LOC641765
0.405916288	stable	FAM118B
0.406033964	stable	ZFP41
0.406055543	stable	LOC650329
0.406150121	stable	TRPC2
0.406150121	stable	FLJ45832
0.406151133	stable	ULBP3
0.406243865	stable	AOC2
0.406364166	stable	LOC441119
0.406370597	stable	PSG7
0.406527954	stable	C12ORF24
0.406527954	stable	HCG3
0.406613008	stable	LOC388813
0.406679802	stable	C10ORF93
0.406703383	stable	LOC642167

0.406754923	stable	TP73
0.40681894	stable	LOC647049
0.406977347	stable	SPNS3
0.407031438	stable	GGN
0.407031438	stable	LOC254398
0.407031438	stable	FOXP4
0.407031438	stable	LOC647843
0.407072465	stable	LOC645227
0.407343584	stable	LOC644931
0.40736708	stable	NEK5
0.40736708	stable	ADH7
0.407399765	stable	ATP5J2
0.407417936	stable	IDH3A
0.407506258	stable	NRD1
0.407506258	stable	FOXO4
0.407630356	stable	LOC650562
0.407703925	stable	MAP3K11
0.407827405	stable	SFRP2
0.407933117	stable	FEV
0.408075249	stable	CRTC1
0.408368682	stable	LOC730256
0.408562148	stable	LOC652612
0.408684378	stable	DUX5
0.408684378	stable	PPT2
0.408723829	stable	LOC648127
0.408723829	stable	LOC647519
0.40885069	stable	LOC650498
0.408882455	stable	CARS
0.409533057	stable	LOC283999
0.409551262	stable	FAM161B
0.409733098	stable	RANBP9
0.410169529	stable	SGCB
0.410183453	stable	LOC646132
0.410183453	stable	PRDM4
0.410352438	stable	DPM3
0.410352438	stable	ANKRD13B
0.410401244	stable	LOC646772
0.410491485	stable	LOC728481
0.410529695	stable	TMEM156
0.410907811	stable	GPR157
0.41105205	stable	LOC645551
0.411414589	stable	KIAA0586
0.411857704	stable	LOC643696
0.411911679	stable	TBPL2
0.411911679	stable	CEACAM20
0.411911679	stable	LOC649817
0.411911679	stable	GNL3L
0.411911679	stable	LOC642185
0.411947089	stable	C10ORF37
0.41195924	stable	SLC12A2
0.412016028	stable	SLC4A3

0.412016028	stable	LOC650961
0.412115646	stable	SLC24A5
0.41238732	stable	SRP68
0.412544853	stable	LRRTM3
0.412590371	stable	KIFC3
0.412849241	stable	NCOA6
0.412886895	stable	CENPL
0.412886895	stable	PAX6
0.412886895	stable	TXNL4B
0.412893523	stable	ZNF143
0.412913123	stable	IL17REL
0.413174051	stable	GLRX5
0.413476911	stable	LOC645186
0.41350504	stable	LOC644172
0.413552933	stable	SCYE1
0.413649127	stable	NR5A1
0.413729867	stable	PSKH1
0.413729867	stable	TM9SF4
0.413889857	stable	SOAT1
0.413981674	stable	C6ORF27
0.413993224	stable	RAB20
0.413993224	stable	CDC37L1
0.414257122	stable	C10ORF49
0.414257122	stable	LOC643225
0.414401932	stable	LDB1
0.414475574	stable	TMEM164
0.414475574	stable	MYT1L
0.414492877	stable	KRT1
0.414627991	stable	ACOT11
0.414670935	stable	LOC402066
0.414723694	stable	LOC340228
0.41483338	stable	LOC389365
0.41483338	stable	ACN9
0.41483338	stable	C20ORF160
0.414986715	stable	OR5AN1
0.415194309	stable	LOC283487
0.415284337	stable	IFNA8
0.415329053	stable	LCE1B
0.415329053	stable	ZNF345
0.415351582	stable	LRAT
0.415351582	stable	SLC5A12
0.415351582	stable	SULT1A4
0.415351582	stable	OR2T29
0.415428516	stable	LOC399746
0.4155109	stable	PSMD9
0.415542182	stable	RBM15B
0.415600705	stable	EPS8L1
0.415600705	stable	SEMA3G
0.415600705	stable	XKR7
0.415600705	stable	DBNDD1
0.415620633	stable	LSM5

0.415688467	stable	TBR1
0.415823171	stable	ACSF3
0.415823171	stable	LOC390876
0.416038941	stable	RNF17
0.416114728	stable	C1D
0.416117366	stable	DAZ2
0.416140593	stable	IMPAD1
0.416211054	stable	LOC644168
0.416289048	stable	TLX1
0.41647081	stable	LOC645839
0.41653447	stable	ARVP6125
0.416694051	stable	LOC643125
0.416732221	stable	LOC644769
0.416732221	stable	LOC400197
0.416843708	stable	ZFAND3
0.416894486	stable	LOC644427
0.417018215	stable	SLC2A10
0.417098444	stable	CCDC138
0.417098444	stable	HCP5
0.417098444	stable	MEI1
0.417167989	stable	RASGEF1A
0.417324142	stable	DACH2
0.417333785	stable	CDRT15
0.417708806	stable	LOC641719
0.417986764	stable	C14ORF125
0.417986764	stable	IFNK
0.418017124	stable	L3MBTL3
0.418381297	stable	LOC646359
0.418381297	stable	GPC6
0.418654145	stable	TNFSF14
0.4186682	stable	TMCC3
0.418965049	stable	LOC642879
0.419107905	stable	LOC401137
0.419112167	stable	UBAC2
0.41911472	stable	LOC388565
0.419123263	stable	MCOLN2
0.41913039	stable	LOC650724
0.419214326	stable	IFRG15
0.419325098	stable	LOC255620
0.419424809	stable	AMAC1
0.419454522	stable	P2RY4
0.419585584	stable	SLC39A5
0.419585584	stable	LOC654056
0.41962	stable	CAPN10
0.419677162	stable	LOC648576
0.419768288	stable	C8ORF48
0.419848662	stable	LOC643146
0.420015587	stable	CNIH3
0.420015587	stable	FLJ20433
0.420024527	stable	TUBB8
0.420059782	stable	LOC643505

0.420088571	stable	C14ORF70
0.420224027	stable	DPPA3
0.420314955	stable	IL22RA2
0.42039845	stable	LRP1
0.420450997	stable	FLJ35776
0.420450997	stable	LOC652183
0.420450997	stable	PRDM13
0.420450997	stable	POU5F2
0.420450997	stable	LOC650919
0.420468037	stable	SLC16A8
0.420468037	stable	LOC652808
0.420468037	stable	GALNT4
0.420510318	stable	MIF4GD
0.420510318	stable	YTHDF2
0.42053937	stable	LOC649318
0.420589836	stable	KIAA1522
0.420589836	stable	LOC643012
0.421054866	stable	LOC402269
0.421115926	stable	LOC652886
0.421117539	stable	ADARB2
0.421430389	stable	LOC649214
0.421430389	stable	LOC643805
0.42150179	stable	ZKSCAN5
0.42162867	stable	C10ORF18
0.421831473	stable	LOC649203
0.421831473	stable	C3ORF65
0.422435857	stable	LOC641810
0.422435857	stable	MAPRE3
0.422435857	stable	AQP6
0.422664435	stable	GATA1
0.422892851	stable	LOC653349
0.423030387	stable	LOC647147
0.423361305	stable	LARP4
0.423367327	stable	FLJ32867
0.423395437	stable	SMN2
0.423395437	stable	LOC645261
0.423516929	stable	LOC729378
0.423595893	stable	LOC642563
0.423710831	stable	YY2
0.423749887	stable	AP3M1
0.423937951	stable	LOC651004
0.424227472	stable	WNT9A
0.42450443	stable	MTO1
0.42450443	stable	LOC647546
0.424604109	stable	PSG4
0.424666126	stable	LOC648217
0.424734104	stable	LOC222967
0.424734104	stable	CYB5R4
0.424734104	stable	KLHDC4
0.424774948	stable	LOC644876
0.424819989	stable	CSTF1

0.424819989	stable	BAT2D1
0.424819989	stable	LOC650254
0.424825753	stable	LOC653829
0.424825753	stable	SF1
0.424832552	stable	REEP2
0.424867244	stable	FLJ42177
0.424937284	stable	AMBP
0.424937284	stable	NME2P1
0.425042616	stable	TRH
0.425184414	stable	PLVAP
0.425299259	stable	TMEM199
0.425299259	stable	LOC648210
0.425360156	stable	HEMGN
0.425432232	stable	LOC441208
0.425559933	stable	L3MBTL4
0.425651782	stable	LOC731999
0.425685299	stable	UGT2B7
0.425769555	stable	GLT1D1
0.425799671	stable	TNPO2
0.426142341	stable	LOC652838
0.426403706	stable	LOC653618
0.426403706	stable	LOC654192
0.426850521	stable	KIF11
0.426898224	stable	LOC647474
0.426898224	stable	SMPD1
0.426904803	stable	ARHGDIG
0.427033668	stable	LOC221710
0.42707415	stable	LOC348840
0.427158747	stable	BAIAP3
0.427158747	stable	LOC646554
0.42746121	stable	LOC653641
0.42746121	stable	LOC643341
0.427596351	stable	OR4C46
0.427618868	stable	PROKR2
0.427668887	stable	LOC729786
0.427813922	stable	LOC401252
0.427890885	stable	LOC650235
0.428095294	stable	LOC401101
0.428196546	stable	PRIM2
0.428282446	stable	ACY3
0.428484176	stable	NCSTN
0.428522856	stable	IDE
0.428612908	stable	DCUN1D4
0.428746513	stable	LOC388503
0.428746513	stable	PRH2
0.428746513	stable	CD300E
0.428746513	stable	ZCCHC11
0.428824573	stable	LOC442388
0.428963165	stable	LOC653576
0.428963165	stable	C3ORF31
0.428993219	stable	LOC651231

0.428993219	stable	KIR2DL4
0.428993219	stable	OCA2
0.429175851	stable	CYORF15A
0.429365636	stable	OR7D2
0.429365636	stable	LOC644452
0.429365636	stable	ANKRD35
0.429375462	stable	FAM90A1
0.429486097	stable	C16ORF46
0.429503512	stable	ERCC8
0.429590627	stable	LOC651007
0.429747812	stable	CD81
0.429869598	stable	GATAD1
0.429954728	stable	LOC653507
0.429954728	stable	TCHH
0.430057836	stable	C8ORF46
0.430057836	stable	LOC643937
0.430057836	stable	XKR4
0.430057836	stable	LOC642767
0.430251055	stable	LOC649060
0.430265736	stable	LOC654254
0.430423221	stable	LOC646926
0.430438347	stable	FGF5
0.430472861	stable	LEKR1
0.430472861	stable	FAM44A
0.430544715	stable	LOC642592
0.430585523	stable	AKAP2
0.430669752	stable	LOC651150
0.430846155	stable	BTBD11
0.430846155	stable	GNG10
0.431075064	stable	A26C3
0.431109921	stable	LOC643808
0.431109921	stable	CDC23
0.431189978	stable	SUMF2
0.431189978	stable	TFDP1
0.431217998	stable	LOC641996
0.431223469	stable	LOC646627
0.431223469	stable	PARC
0.431299347	stable	SPRR2C
0.431562137	stable	SLC28A1
0.431672746	stable	BCCIP
0.431672746	stable	LOC388849
0.43176959	stable	TMPRSS11D
0.43176959	stable	LOC644360
0.43176959	stable	C22ORF23
0.43194165	stable	LOC644003
0.432063486	stable	CERKL
0.432316199	stable	RFT1
0.432316199	stable	TAF12
0.43248404	stable	SLC25A34
0.43248404	stable	F9
0.432575886	stable	ARID2

0.432626058	stable	KIAA1530
0.432626058	stable	PCAF
0.432708957	stable	PYDC1
0.432708957	stable	LOC339674
0.432708957	stable	LOC641365
0.432708957	stable	PPP1R2P3
0.432768489	stable	TRAM2
0.43288283	stable	LOC644884
0.43288283	stable	C4ORF23
0.432939817	stable	C1ORF129
0.433058639	stable	FAM81B
0.433100718	stable	LOC285768
0.433278422	stable	LOC654252
0.433363743	stable	STX11
0.433397384	stable	CHD9
0.433405416	stable	LOC388963
0.433426036	stable	MRPS31
0.433630449	stable	SGTB
0.433632738	stable	LOC643949
0.433658339	stable	LOC402617
0.433720363	stable	ELMOD1
0.433806326	stable	COX11
0.434160079	stable	C19ORF35
0.434402184	stable	FAM83C
0.434402184	stable	ZNF446
0.434402184	stable	FLJ46300
0.434630808	stable	MEGF11
0.434630808	stable	LOC284422
0.434662911	stable	LOC220433
0.434740811	stable	WDR41
0.434751229	stable	ITPR1
0.434868421	stable	RGPD7
0.434924192	stable	LOC645294
0.434930333	stable	LOC643131
0.434939265	stable	LOC643070
0.434953758	stable	LOC728516
0.435001682	stable	ZNF582
0.435027919	stable	F2R
0.435227917	stable	PRAP1
0.435229931	stable	LOC728285
0.435229931	stable	LOC388915
0.435237069	stable	ZNF620
0.435278017	stable	ZNF609
0.435412235	stable	LOC388312
0.435552103	stable	LOC728811
0.435555949	stable	LOC647711
0.435568822	stable	LOC651892
0.435568822	stable	LOC643662
0.435666082	stable	CASKIN1
0.435722367	stable	DISP1
0.436229244	stable	GLT8D4

0.436232523	stable	MRPL23
0.436265631	stable	RPS6KB1
0.436269907	stable	MAPK10
0.436326314	stable	DEFB104B
0.436361476	stable	UFC1
0.436494427	stable	CTSD
0.436556761	stable	LOC647208
0.43675013	stable	LOC642995
0.43675013	stable	LOC651503
0.437014332	stable	DDI1
0.437024373	stable	LOC649978
0.437027899	stable	CCDC35
0.437118899	stable	DYNC2H1
0.43714629	stable	C1ORF21
0.43714629	stable	LOC650853
0.43714629	stable	LOC651738
0.437173364	stable	LOC441205
0.437173364	stable	VIL1
0.437257469	stable	LGR5
0.43732206	stable	FLJ45684
0.43732206	stable	PDCL2
0.437366962	stable	DMRTA2
0.437366962	stable	VKORC1
0.43741694	stable	FMR1
0.437659906	stable	OR13A1
0.437659906	stable	MCRS1
0.437784422	stable	MAP4K5
0.437784422	stable	LOC613206
0.437990192	stable	OLIG2
0.438009255	stable	GPM6A
0.438014265	stable	ZNF597
0.438285526	stable	LOC651731
0.438313161	stable	LOC651202
0.438315962	stable	LOC729758
0.438315962	stable	ZNF480
0.438315962	stable	TCBA1
0.438315962	stable	SIRPB1
0.43836974	stable	MGC70870
0.438435956	stable	LOC651314
0.438474689	stable	LTBP1
0.438774746	stable	RPS6
0.438774746	stable	ODF3L1
0.438774746	stable	TMPRSS11A
0.438833499	stable	OR9A2
0.438833499	stable	GPR112
0.438862208	stable	EXOC5
0.438862208	stable	LOC648041
0.438862208	stable	AQP2
0.438934566	stable	FAM126B
0.438934566	stable	LOC645899
0.438941884	stable	TMEM89

0.438983158	stable	SERPINB5
0.439027263	stable	LOC649910
0.439161737	stable	CALM2
0.439227469	stable	HSD11B1
0.439299627	stable	C8ORF41
0.439299627	stable	UNC45A
0.439336946	stable	LOC389671
0.439336946	stable	PSMG3
0.439425424	stable	COG8
0.43945909	stable	LOC647328
0.439490568	stable	EIF3E
0.439490568	stable	DNAJC27
0.439760735	stable	MAK16
0.439762871	stable	SMG1
0.439955779	stable	KRTAP10-8
0.439967035	stable	HBG1
0.440183269	stable	LOC648943
0.44028995	stable	C4ORF17
0.440381874	stable	PTPRG
0.44066527	stable	KIAA0317
0.440678238	stable	LOC440706
0.440678238	stable	CCL20
0.441042491	stable	ANKRD13C
0.441050947	stable	PARG
0.441107351	stable	LOC642987
0.441107351	stable	XAGE1B
0.441107351	stable	HS3ST3B1
0.441127045	stable	LOC388900
0.441132999	stable	SELO
0.441460313	stable	TMEM161B
0.441472256	stable	PTPN7
0.441527873	stable	C1ORF141
0.441538221	stable	LOC441870
0.441538221	stable	PIGQ
0.441538221	stable	LOC646279
0.441562968	stable	LOC113386
0.441562968	stable	UBXD6
0.441593718	stable	C22ORF33
0.441823539	stable	RPL38
0.441823539	stable	GJA9
0.441909073	stable	CDIPT
0.442264024	stable	CHAF1A
0.442264024	stable	CLDN12
0.442500652	stable	DNHL1
0.442500652	stable	LOC440577
0.44251815	stable	BTN3A3
0.442730682	stable	HIT-40
0.442794287	stable	ANXA13
0.442820961	stable	SOX1
0.442976704	stable	MGC33407
0.443002212	stable	GPR37L1

0.443130389	stable	LOC645686
0.44313606	stable	LOC389787
0.443223161	stable	ADAM20
0.443244991	stable	CSTA
0.443244991	stable	LOC400713
0.443264497	stable	ZBTB41
0.443313185	stable	RAPGEF6
0.443313185	stable	LOC643350
0.443317208	stable	LOC653146
0.443487204	stable	HDAC11
0.443489236	stable	LOC389842
0.443636721	stable	LOC651848
0.443665476	stable	LOC645576
0.4436716	stable	HIST1H2BA
0.44399888	stable	UBL4B
0.44399888	stable	LOC390561
0.44399888	stable	KCNH1
0.444014021	stable	LOC647089
0.444065462	stable	NME6
0.444293095	stable	KRTAP19-2
0.444307891	stable	LOC650599
0.444465882	stable	OCEL1
0.444556527	stable	LOC402562
0.444556527	stable	LOC652111
0.444812408	stable	ZMAT5
0.444849714	stable	C21ORF86
0.444919362	stable	DAZ1
0.444944179	stable	GJA8
0.445198077	stable	CABYR
0.445201919	stable	ZNF556
0.445201919	stable	IL5
0.445201919	stable	LOC648392
0.445201919	stable	RTEL1
0.445201919	stable	ZNF223
0.445201919	stable	KLHDC3
0.445201919	stable	FLJ30058
0.445201919	stable	TAF3
0.445201919	stable	DYNC1I1
0.445201919	stable	LOC644652
0.445285781	stable	IGF2AS
0.445285781	stable	LOC646010
0.445321993	stable	KIAA1666
0.445321993	stable	TMPRSS11B
0.445343187	stable	IL22
0.445381031	stable	LOC653037
0.445475457	stable	GPR6
0.445518554	stable	LOC648590
0.445518554	stable	C12ORF71
0.445943094	stable	LOC645012
0.446141296	stable	LOC651102
0.446141296	stable	LOC440995

0.446185356	stable	LOC650712
0.446286246	stable	LOC646675
0.446292909	stable	LOC646198
0.446338391	stable	KIAA1922
0.446338391	stable	KRT73
0.446349625	stable	C1ORF172
0.446423884	stable	FLJ43582
0.446512073	stable	LOC387873
0.446592779	stable	ATXN8OS
0.446623437	stable	SLC5A11
0.446728256	stable	UGT2A1
0.44674895	stable	LOC645363
0.446822198	stable	LOC649428
0.447167554	stable	DOK1
0.447343405	stable	APPBP2
0.447589668	stable	LOC389830
0.447638883	stable	LOC730130
0.447724048	stable	C16ORF5
0.447724048	stable	SLIC1
0.447913849	stable	METT11D1
0.447972011	stable	PHF19
0.448031786	stable	LOC642629
0.448064288	stable	LOC402483
0.448153624	stable	TFAMP1
0.448196873	stable	LOC652200
0.448225139	stable	PRKCG
0.448277176	stable	BDP1
0.448305278	stable	TGOLN2
0.448416104	stable	LOC653533
0.448480435	stable	TNFSF12-TNFSF13
0.44862163	stable	SNRPD1
0.448654417	stable	LOC389641
0.448756801	stable	CPSF3
0.448889978	stable	KIR3DX1
0.448907927	stable	FBXL21
0.448907927	stable	KIAA0514
0.448907927	stable	LOC651430
0.448914522	stable	IFNA17
0.448955885	stable	PRR3
0.449025851	stable	AGAP1
0.449025851	stable	KRT86
0.449092026	stable	PBRM1
0.449325306	stable	FLJ90650
0.449350658	stable	ACSM5
0.449849773	stable	LOC644468
0.450017228	stable	FAM174B
0.450117707	stable	CHURC1
0.450314705	stable	LOC643770
0.450396102	stable	CYLC1
0.450645216	stable	LOC651728
0.450731708	stable	STCH

0.450775206	stable	LOC442020
0.450871818	stable	PLCXD2
0.450871818	stable	FAM128A
0.450871818	stable	DMWD
0.451003244	stable	ATF7
0.4510437	stable	SLC2A4RG
0.4510437	stable	APLNR
0.451046557	stable	LOC729602
0.451152956	stable	MGC33894
0.451171243	stable	AADACL3
0.451281365	stable	CPNE7
0.451300439	stable	GRIN2C
0.451326247	stable	WHDC1L2
0.451426526	stable	MGC42105
0.451452833	stable	RAP2B
0.451862666	stable	SCML4
0.452245102	stable	LOC124216
0.452847166	stable	LOC728183
0.452918719	stable	LOC149224
0.452918719	stable	CATSPER1
0.453061868	stable	LIX1
0.453061868	stable	RNF212
0.453184528	stable	GNG3
0.453185398	stable	GMNN
0.453193104	stable	IL23R
0.453243792	stable	NPAL3
0.453398914	stable	CD28
0.453403864	stable	TOMM22
0.453473575	stable	ACAA1
0.453510865	stable	CCDC88B
0.453510865	stable	SNW1
0.453570642	stable	LOC652288
0.453603087	stable	LOC401410
0.453867605	stable	LOC650314
0.453909445	stable	SLC39A4
0.453909445	stable	USH1G
0.453936134	stable	LOC644558
0.453936134	stable	C20ORF133
0.453980412	stable	RNF180
0.454163132	stable	KCNA1
0.454163132	stable	PGAM2
0.454287778	stable	MYEOV2
0.454309575	stable	MORN5
0.454309575	stable	LOC653325
0.454581935	stable	LOC642433
0.454734156	stable	CLDN2
0.455102517	stable	PRDM9
0.455397443	stable	MTRR
0.455626811	stable	SLC2A5
0.455641316	stable	TRIT1
0.455641316	stable	PRB4

0.455641316	stable	LOC653962
0.455706339	stable	LOC651272
0.455815468	stable	LOC652127
0.45593415	stable	RAET1E
0.455976514	stable	USH2A
0.455976514	stable	VAX1
0.455976514	stable	LOC653280
0.455976514	stable	SSTR4
0.456429224	stable	LOC345630
0.456519672	stable	GTPBP1
0.456755267	stable	VCX3A
0.457008749	stable	DUXA
0.457008749	stable	LOC441251
0.457013291	stable	LOC646491
0.457013291	stable	C19ORF66
0.457187705	stable	TFAP2C
0.457187705	stable	SNAPC1
0.457399473	stable	HPR
0.45755166	stable	LOC652850
0.457715118	stable	MIA2
0.457723393	stable	LOC645992
0.457892864	stable	LOC728621
0.458082146	stable	XKRX
0.45817621	stable	RAD1
0.45817621	stable	ADAM30
0.458287507	stable	C16ORF3
0.4583048	stable	LOC649317
0.458475858	stable	COL24A1
0.458475858	stable	PTPRS
0.45851064	stable	LOC649956
0.458654602	stable	TMEM202
0.458711362	stable	DHDDS
0.458860526	stable	LOC644399
0.458883013	stable	LOC645869
0.458883013	stable	HOXC13
0.45893277	stable	MGC27121
0.45893277	stable	ANXA10
0.45903633	stable	CD6
0.45903633	stable	LOC388720
0.459165478	stable	LOC116412
0.459165478	stable	LOC642730
0.459248613	stable	HPCAL4
0.459329317	stable	PCDHB10
0.459329317	stable	PFN3
0.459334717	stable	CYP3A5
0.459346949	stable	NPY2R
0.45970752	stable	LOC345643
0.459733827	stable	LOC441771
0.459788897	stable	RAB3D
0.459829955	stable	LOC641805
0.46001799	stable	LOC642380

0.460032463	stable	FAM75A2
0.460126442	stable	MSI2
0.460331568	stable	C8ORFK32
0.460436219	stable	LOC649948
0.460660854	stable	HOXD1
0.460895351	stable	LOC648705
0.460895351	stable	GRIA4
0.460895351	stable	LOC652513
0.461036297	stable	TMEM132E
0.461146452	stable	AK7
0.461146452	stable	OCLN
0.461146452	stable	POLR1A
0.461203391	stable	C9ORF31
0.461268347	stable	TIMM8B
0.461387433	stable	ZMYM5
0.461468658	stable	RBM11
0.461561242	stable	XGPY2
0.461689109	stable	RNF138
0.461690987	stable	LOC642505
0.461690987	stable	CUZD1
0.461762108	stable	GRAMD2
0.461975816	stable	ANKRD33
0.461975816	stable	LOC650261
0.461975816	stable	RTP3
0.462100448	stable	KRTAP4-3
0.462104172	stable	LOC389901
0.462155447	stable	LOC642961
0.462155447	stable	CLU
0.462155447	stable	LOC646997
0.462253737	stable	ERVK6
0.462286675	stable	HMCN1
0.462349205	stable	FAM38B
0.462431347	stable	LOC283755
0.462518463	stable	C8ORF15
0.462538759	stable	KDR
0.462703927	stable	UGT3A2
0.462757561	stable	LOC650311
0.462861074	stable	NDUFAB1
0.462930583	stable	LOC643953
0.463058122	stable	SLC45A1
0.463123074	stable	KCNJ10
0.463123074	stable	RAD51AP2
0.46313216	stable	C9ORF153
0.46313216	stable	GADD45GIP1
0.463222804	stable	REM1
0.463222804	stable	KERA
0.463227651	stable	LOC652369
0.463243243	stable	LOC644477
0.463277633	stable	TTC39B
0.463277633	stable	LOC652819
0.463282286	stable	LOC646334

0.463282286	stable	INE1
0.463305597	stable	LOC440352
0.463305597	stable	LOC651125
0.463305597	stable	LOC728095
0.463305597	stable	FUZ
0.463379681	stable	LOC642583
0.463379681	stable	FLJ35848
0.463386696	stable	RARRES2
0.463578649	stable	LOC653324
0.463578649	stable	LOC392197
0.463578649	stable	LOC649091
0.463578649	stable	SSTR2
0.463605461	stable	C4ORF21
0.463722688	stable	RKHD1
0.463722688	stable	TARS
0.463741016	stable	LOC650543
0.463770972	stable	MGC5139
0.463909565	stable	TRIM72
0.463960912	stable	LOC652594
0.463994243	stable	ZNF138
0.464048127	stable	LOC441617
0.464152774	stable	RNPEPL1
0.464196214	stable	COX8A
0.464196214	stable	LOC730194
0.464196214	stable	TNNI1
0.464196214	stable	MAGI1
0.464329311	stable	LOC728229
0.464329311	stable	UNC119
0.464360203	stable	RPL4
0.464361926	stable	LOC642453
0.464361926	stable	LOC285444
0.464425954	stable	LOC728654
0.464523857	stable	APOBEC3A
0.464523857	stable	RPL35A
0.464552993	stable	STATH
0.464552993	stable	MPP5
0.464654619	stable	LOC642591
0.46484713	stable	PPARBP
0.464872296	stable	RPS6KL1
0.464898172	stable	LOC653135
0.464899807	stable	C1QBP
0.46500511	stable	ZNF586
0.46500511	stable	KENAE
0.465102054	stable	FLJ45974
0.465102054	stable	LY6G6D
0.465102054	stable	COL18A1
0.46526837	stable	C3ORF14
0.465630508	stable	LOC644767
0.465630508	stable	CEMP1
0.465630508	stable	LOC648432
0.465786436	stable	NFAM1

0.465807454	stable	ACHE
0.465807454	stable	RAD9A
0.46599797	stable	DRD1
0.466007943	stable	ARHGEF10L
0.466160412	stable	LOC342931
0.46642232	stable	OR1S2
0.46642232	stable	LOC642168
0.466536975	stable	SRD5A3
0.466623287	stable	MGC46336
0.466623287	stable	LOC645836
0.46672804	stable	LOC647748
0.46672804	stable	LOC649128
0.466797322	stable	MFI2
0.46680766	stable	LYZL2
0.467004617	stable	ZAN
0.467219735	stable	LOC440550
0.467377755	stable	LOC441773
0.467391917	stable	TMOD3
0.467400039	stable	SLC10A6
0.467400039	stable	GFI1
0.467400039	stable	IFNW1
0.467400039	stable	IQSEC1
0.467420862	stable	SPATA3
0.467420862	stable	LOC642278
0.467582823	stable	LOC648253
0.468033596	stable	ZNF469
0.468033596	stable	WDR42B
0.468069327	stable	LOC645153
0.468084463	stable	RLF
0.468084463	stable	ZSCAN1
0.468084463	stable	MARVELD3
0.468084463	stable	LOC642608
0.468084463	stable	MBL2
0.468193428	stable	LOC649063
0.468219085	stable	RNF190
0.468373752	stable	LOC285407
0.468414878	stable	LOC649571
0.468440759	stable	LOC649800
0.46845923	stable	RPL19
0.46845923	stable	PSRC1
0.468610989	stable	FIGLA
0.468615211	stable	FAM7A3
0.468615211	stable	LOC644591
0.468660998	stable	LOC441476
0.468719487	stable	C21ORF29
0.468719487	stable	LOC647413
0.468793497	stable	ASAM
0.468959784	stable	PRMT3
0.469001647	stable	XAF1
0.469001647	stable	ADORA2A
0.469001647	stable	LOC653696

0.469001647	stable	LOC647058
0.469102354	stable	VCAM1
0.469102354	stable	TCERG1
0.469164193	stable	LOC642981
0.469180799	stable	ZFYVE27
0.469210833	stable	LOC646399
0.469210833	stable	DEFB134
0.469318232	stable	LOC285697
0.469366538	stable	LOC648257
0.469366538	stable	SFXN3
0.469477489	stable	THEM2
0.469477489	stable	BTNL8
0.469477489	stable	OR13J1
0.469477489	stable	LOC440030
0.469655398	stable	COL4A1
0.469672765	stable	PI16
0.469863376	stable	HOOK3
0.469863376	stable	MTSS1
0.469863376	stable	NRF1
0.470110776	stable	FLJ44653
0.470111159	stable	ACADS
0.470111159	stable	LOC646439
0.470170344	stable	LOC730302
0.470328998	stable	PLEKHG4
0.470328998	stable	MAN2A2
0.470664532	stable	OSBPL10
0.470664532	stable	DUX4
0.470713608	stable	FLJ39739
0.470884318	stable	POGZ
0.470939841	stable	CYP27B1
0.471042897	stable	LOC643700
0.471229255	stable	SLC9A3R2
0.471229255	stable	SLC1A6
0.471318191	stable	LOC642428
0.47134345	stable	LOC653852
0.471386651	stable	TMEM97
0.471514206	stable	LOC643943
0.47152305	stable	LOC647374
0.471527073	stable	CPSF6
0.471527073	stable	LOC647797
0.471527073	stable	CIB3
0.471527073	stable	MYH7
0.471649194	stable	LOC651319
0.471706987	stable	LOC643772
0.471723204	stable	CDH11
0.471976746	stable	B3GN-T6
0.472053998	stable	ATN1
0.472053998	stable	DYX1C1
0.472053998	stable	LOC643132
0.472053998	stable	CLDN11
0.472053998	stable	LOC650214

0.472053998	stable	SLC47A2
0.472053998	stable	LOC649009
0.472053998	stable	LOC389816
0.472053998	stable	CST7
0.472086619	stable	LOC652282
0.472140634	stable	DKKL1
0.472140634	stable	GPR19
0.472146058	stable	LOC643930
0.47215636	stable	OR4C11
0.47215636	stable	LOC283440
0.47215636	stable	CTRB1
0.47215636	stable	LOC391656
0.47215636	stable	MAZ
0.47215636	stable	FBXL4
0.47215636	stable	KCNJ13
0.47215636	stable	C18ORF25
0.47234795	stable	LOC649991
0.472450695	stable	LOC440848
0.472450695	stable	STAU2
0.472561608	stable	ZNF322B
0.472561608	stable	UGT2B17
0.472561608	stable	TNF
0.472561608	stable	LOC643853
0.47259835	stable	LOC127099
0.472866536	stable	LOC441811
0.472918769	stable	LOC441098
0.472994976	stable	C1ORF194
0.473108229	stable	FLJ33996
0.473348864	stable	RHOQ
0.473372163	stable	PIP5K2B
0.473585678	stable	C2ORF60
0.473585678	stable	C17ORF76
0.473624832	stable	GPER
0.473905837	stable	RUNDC3A
0.473955183	stable	C16ORF85
0.474004177	stable	TMIGD2
0.474033419	stable	ASCC1
0.474223808	stable	DSG2
0.474246587	stable	ARNTL2
0.474293544	stable	FAM108B1
0.474478407	stable	ROBO3
0.474538993	stable	LOC653387
0.474601489	stable	DPYS
0.474601489	stable	FLJ41423
0.474601489	stable	ACPT
0.474740156	stable	SAMD12
0.474827731	stable	AP1S1
0.474844059	stable	CDH13
0.474844059	stable	LOC652100
0.474844059	stable	LOC647213
0.474844059	stable	LOC646067

0.474844059	stable	LOC648170
0.474844059	stable	UGT2A3
0.474844059	stable	CBX5
0.474844059	stable	NEUROD2
0.474946067	stable	PTPN20
0.474954179	stable	CLUAP1
0.474954179	stable	NT5E
0.47497094	stable	LOC646512
0.47497094	stable	AMICA1
0.475133776	stable	BRCA2
0.475133776	stable	LOC646299
0.475166494	stable	B3GALT6
0.475171016	stable	APOA1BP
0.475220352	stable	LOC51149
0.475280229	stable	SRPX2
0.475294231	stable	DKFZP781N1041
0.475294231	stable	LOC651530
0.475330369	stable	TTC29
0.475401493	stable	DGKK
0.475751831	stable	ARVCF
0.475751831	stable	SLC38A5
0.475751831	stable	LOC653316
0.475751831	stable	TTTY6B
0.475751831	stable	LOC339902
0.475809183	stable	INPP5A
0.475809183	stable	LOC653577
0.475809183	stable	FLJ32569
0.475893694	stable	TMEM92
0.475970168	stable	TMEM121
0.476074559	stable	LOC151234
0.47611031	stable	LOC440829
0.47611031	stable	AR
0.476255756	stable	MAST1
0.476344009	stable	C20ORF134
0.476344009	stable	LOC653319
0.476344009	stable	LOC643599
0.476344009	stable	CSAG3A
0.476415439	stable	MAPK8IP3
0.476471922	stable	LOC651617
0.476514651	stable	LOC650568
0.476536616	stable	LOC730118
0.476536616	stable	LOC729826
0.476570536	stable	SLC30A10
0.476685567	stable	ANO8
0.47688764	stable	SNORD22
0.477008386	stable	ABHD13
0.477008386	stable	C12ORF40
0.477008386	stable	CDYL
0.477173677	stable	FLJ45994
0.477377588	stable	LOC595101
0.477377588	stable	PPP2R2D

0.477639712	stable	CTTNBP2NL
0.477683675	stable	LOC653434
0.477683675	stable	LOC200810
0.477683675	stable	TMPRSS7
0.477725733	stable	IMPA1
0.477725733	stable	OR1E1
0.478015199	stable	LOC651554
0.478096902	stable	PITPNB
0.478110619	stable	ENOX1
0.478110619	stable	ABL2
0.478110619	stable	CDKAL1
0.478116021	stable	LOC649192
0.478116021	stable	KLC4
0.478262633	stable	KIAA1109
0.478262633	stable	C8ORF71
0.478262633	stable	PGC
0.478262633	stable	MEF2A
0.478315699	stable	FLJ21839
0.478315699	stable	LOC644668
0.478408718	stable	LOC650930
0.47854694	stable	LHPP
0.47854694	stable	ACOT2
0.47857653	stable	FLJ30851
0.478577827	stable	TBC1D21
0.47861264	stable	GSTA2
0.47861264	stable	ZNF100
0.478719356	stable	LOC653820
0.478719356	stable	PAK4
0.478746336	stable	CSNK2B
0.4788045	stable	MECP2
0.478833184	stable	SYNGR4
0.478833184	stable	LOC90120
0.478884065	stable	TMEM19
0.478911356	stable	ADK
0.47902385	stable	LOC728556
0.479041938	stable	CYP4A11
0.479041938	stable	LOC644734
0.479083109	stable	LOC731670
0.479155821	stable	LOC646903
0.479417079	stable	DPP10
0.479417079	stable	WNT9B
0.479417079	stable	ASAH3L
0.479431001	stable	LOC652469
0.479431001	stable	LOC645276
0.479652356	stable	LOC647309
0.479654379	stable	LOC652761
0.479654379	stable	UBFD1
0.479791533	stable	ODZ1
0.479890849	stable	LOC285588
0.480093247	stable	LOC645427
0.480136151	stable	EHBP1

0.480171276	stable	KIAA1843
0.480219341	stable	C12ORF29
0.480222263	stable	MYOM3
0.480323329	stable	LOC727805
0.480729811	stable	LOC642978
0.480730417	stable	ATP6V1G3
0.480975368	stable	LOC644135
0.481123338	stable	LOC642503
0.481123338	stable	LOC645481
0.481123338	stable	C2ORF16
0.481148357	stable	LOC643582
0.481148357	stable	FHL2
0.481148357	stable	ODF1
0.481148357	stable	FLJ20972
0.481148357	stable	CEBPE
0.481225711	stable	LOC653423
0.481247587	stable	KRT27
0.481420792	stable	ADAMDEC1
0.481571741	stable	LOC653094
0.48180912	stable	LOC340598
0.481902709	stable	HLX
0.481918193	stable	OR7D4
0.482177477	stable	LOC649848
0.482392982	stable	LOC649908
0.482403529	stable	ATP5D
0.482436836	stable	C2ORF50
0.482436836	stable	ILKAP
0.483002499	stable	SNORA62
0.483167242	stable	GZMH
0.483268922	stable	PAGE5
0.483316603	stable	LHX9
0.483316603	stable	LOC643878
0.483316603	stable	TAF1L
0.483378212	stable	GSTCD
0.483378212	stable	FAM134A
0.483378212	stable	LINGO2
0.483378212	stable	TRPM5
0.483378212	stable	LOC650717
0.483591529	stable	DTX1
0.483595255	stable	CMA1
0.483732844	stable	GALR1
0.483895436	stable	LOC440567
0.483895436	stable	TBC1D3C
0.483895436	stable	TTTY3
0.483895436	stable	NCBP1
0.483895436	stable	LOC649945
0.483895436	stable	LOC642411
0.483895436	stable	C14ORF128
0.484332797	stable	TDRD10
0.484423866	stable	LIMA1
0.484423866	stable	RINT1

0.484423866	stable	CSPP1
0.484423866	stable	LOC646917
0.484464797	stable	LOC646162
0.484464797	stable	KIR2DS4
0.484464797	stable	LOC643559
0.484464797	stable	TMED7
0.484705057	stable	LOC652856
0.484804058	stable	ZNF467
0.484804058	stable	EXOSC6
0.484804058	stable	RAD18
0.485162687	stable	SLN
0.485169535	stable	LOC652647
0.485175605	stable	LOC642759
0.485459923	stable	RAB6B
0.485679484	stable	ACBD4
0.4857648	stable	LMBRD1
0.485767713	stable	LOC643697
0.485767713	stable	IAPP
0.485803864	stable	TRIM16
0.485935198	stable	LOC440895
0.485948833	stable	LOC220429
0.486029751	stable	PCDHA5
0.486065647	stable	MCM3
0.48607067	stable	LOC644157
0.486149866	stable	ZYG11B
0.486149866	stable	OR4S2
0.486177199	stable	PNMA6A
0.486321687	stable	DDHD1
0.486531606	stable	PAH
0.486690001	stable	LOC645966
0.48685929	stable	FLJ33708
0.487077263	stable	LOC645487
0.48717509	stable	LOC645676
0.487190897	stable	SLC38A9
0.487226195	stable	LOC728081
0.487227874	stable	LOC645441
0.487678743	stable	ZNF326
0.487787435	stable	HERPUD1
0.487801127	stable	LOC644953
0.487853	stable	KBTBD6
0.487858652	stable	FLJ12355
0.48786248	stable	EPHB2
0.487919133	stable	FOXD4L1
0.487919133	stable	LOC644828
0.488082592	stable	LOC641994
0.488083003	stable	TYK2
0.488083003	stable	MEFV
0.488276063	stable	FLJ41841
0.488276063	stable	PCDHB11
0.488342716	stable	LOC729486
0.488467608	stable	RAX

0.488624011	stable	DEFB1
0.488631808	stable	LOC653673
0.488631808	stable	HYAL3
0.488631808	stable	RAB7L1
0.488686348	stable	TTC4
0.489081356	stable	LOC649937
0.489081356	stable	SPEF1
0.489291854	stable	LOC644679
0.48941927	stable	GPR149
0.489585081	stable	PKP1
0.489782313	stable	LOC653899
0.489794522	stable	POTE2
0.489798294	stable	LOC652828
0.489807783	stable	SMPD4
0.489811471	stable	LOC652765
0.489811471	stable	LOC646103
0.489811471	stable	C1ORF180
0.489811471	stable	LOC390595
0.489811471	stable	FUS
0.489811471	stable	LOC643950
0.489851008	stable	SPRYD5
0.489856638	stable	SYAP1
0.489856638	stable	B3GALT5
0.48987585	stable	FAM12B
0.489900978	stable	SCTR
0.490091869	stable	ZNF512B
0.490091869	stable	LOC649159
0.490285371	stable	LOC644580
0.490296425	stable	PLEKHA7
0.490296425	stable	SLC30A6
0.490296425	stable	PCDHA13
0.490298146	stable	SNRPD2
0.490417706	stable	P2RY10
0.490511547	stable	LOC440748
0.490511547	stable	LOC645494
0.490635307	stable	LOC283157
0.49065597	stable	FBLN2
0.49076978	stable	LOC649935
0.490898511	stable	LOC652903
0.490898511	stable	MAF1
0.491008133	stable	SIGIRR
0.491187277	stable	HSPA8
0.491381136	stable	LOC642350
0.491381136	stable	GLUD2
0.491438446	stable	ERBB2
0.4917092	stable	LOC644316
0.491797275	stable	ZNF808
0.49180998	stable	C19ORF21
0.49180998	stable	DKFZP761O2018
0.491970299	stable	LOC648044
0.491985131	stable	OR51F2

0.492239643	stable	C1ORF64
0.492239643	stable	SPTBN5
0.492239643	stable	FLJ34969
0.492406565	stable	LOC643143
0.492461153	stable	KIAA0947
0.492461153	stable	TBC1D26
0.492556356	stable	RPS12
0.49257348	stable	LOC644779
0.492605551	stable	LOC649088
0.492605551	stable	LOC730357
0.492605551	stable	LOC642452
0.492605551	stable	TRIM46
0.492627909	stable	LOC649133
0.492627909	stable	LOC654244
0.492629309	stable	KCNC1
0.492749217	stable	ZNF473
0.492749217	stable	OR6C1
0.492749217	stable	OR2T11
0.49280481	stable	LOC647873
0.493011069	stable	ARMC7
0.493050083	stable	GOLGA2L1
0.493197049	stable	DNAH2
0.493488389	stable	BRWD3
0.494273809	stable	WDFY3
0.494483369	stable	LOC643197
0.494521403	stable	FAM18B2
0.494606458	stable	VRK3
0.49462741	stable	POLR2F
0.494648905	stable	ZNF281
0.495110419	stable	THG1L
0.495185639	stable	BOK
0.495236747	stable	LOC647888
0.495418631	stable	SIT1
0.495444355	stable	CER1
0.495444355	stable	NEK11
0.495444355	stable	CDH9
0.495444355	stable	LOC440925
0.495531223	stable	LRRC38
0.495574731	stable	AVP
0.495582474	stable	MORF4L2
0.495598781	stable	FAM164C
0.495598781	stable	LOC653242
0.495623147	stable	ARL5B
0.495765131	stable	LOC652663
0.49579186	stable	WDR16
0.495800775	stable	LOC158830
0.496012759	stable	LOC388275
0.496196877	stable	C20ORF71
0.496402674	stable	FOXA2
0.496685934	stable	LOC646628
0.496685934	stable	NRG1

0.496689242	stable	POLR2A
0.496689242	stable	LOC390933
0.496964724	stable	LOC646494
0.496996274	stable	WDR21C
0.497005058	stable	LOC643834
0.497011343	stable	ASB9
0.497089963	stable	LOC650410
0.497349055	stable	ZNF284
0.497656309	stable	SEC22C
0.497661868	stable	LOC440334
0.497966359	stable	SDK2
0.498011544	stable	ZNF232
0.498036414	stable	CYP4Z2P
0.498075424	stable	LSP1
0.49812956	stable	PCDH7
0.498408512	stable	FLJ16734
0.498408512	stable	SLC7A4
0.498870041	stable	LOC441682
0.498997562	stable	LOC389904
0.499024846	stable	ALAS1
0.499024846	stable	PPP1R2
0.499161274	stable	LOC647176
0.499223441	stable	LOC643912
0.499223572	stable	LOC653481
0.499223572	stable	RAB11FIP1
0.499223572	stable	LOC649455
0.499359363	stable	SLFN12L
0.499423338	stable	CCRL1
0.499423338	stable	LOC644646
0.499423338	stable	ZNF93
0.499586517	stable	LOC644539
0.49960355	stable	LOC652468
0.499672565	stable	DFNA5
0.499704133	stable	FBXO11
0.499704133	stable	LOC402670
0.499747116	stable	LOC648907
0.500146867	stable	KCNQ5
0.500146867	stable	MAS1L
0.500182301	stable	PPCDC
0.500399681	stable	LOC642671
0.500532707	stable	APOL2
0.500641552	stable	LOC402693
0.500641552	stable	LOC650553
0.500641552	stable	LOC647461
0.500641552	stable	ZNF592
0.500641552	stable	LOC650098
0.500641552	stable	CLEC2A
0.500711854	stable	THSD7B
0.500913937	stable	CHRNA10
0.500913937	stable	BCL2A1
0.501100587	stable	GBP7

0.501100587	stable	SH3BP5L
0.501419496	stable	IGFBP7
0.501419496	stable	LOC646845
0.501473785	stable	ANG
0.501636028	stable	MRPL43
0.501821971	stable	MAGEC2
0.501983307	stable	C13ORF28
0.502037333	stable	LOC439949
0.502037333	stable	SLC35F3
0.502365981	stable	C15ORF21
0.502365981	stable	FAM53A
0.502416676	stable	ACBD6
0.502525201	stable	MRPL51
0.502525201	stable	GJC2
0.502615965	stable	C1ORF88
0.502615965	stable	LOC727851
0.502691567	stable	LOC645273
0.502711218	stable	RAB25
0.502711218	stable	CCDC17
0.502711218	stable	UNK
0.502711218	stable	OR10AD1
0.502711218	stable	GPATC4
0.502711218	stable	ATP11A
0.502719836	stable	NTS
0.502719836	stable	TACC3
0.502719836	stable	PDE3A
0.502832433	stable	LOC400986
0.503155363	stable	LOC648167
0.503203653	stable	LOC389394
0.503203653	stable	TEX9
0.503204191	stable	KRTAP27-1
0.503204191	stable	LOC646262
0.50328868	stable	LOC652522
0.50328868	stable	NFATC3
0.50328868	stable	TEX15
0.503524927	stable	HTR7P
0.503741355	stable	LOC647941
0.503851734	stable	MIAT
0.50388419	stable	FLJ16323
0.504074673	stable	THEX1
0.504233403	stable	COPB2
0.504310347	stable	FOXD4L3
0.50436062	stable	TM4SF5
0.504433928	stable	TXNIP
0.504486868	stable	OR51E2
0.504486868	stable	GPRC5C
0.504598583	stable	LOC646197
0.504615842	stable	RNF167
0.504615842	stable	RXFP3
0.504615842	stable	AVPR1A
0.504615842	stable	C14ORF119

0.504615842	stable	LOC647868
0.504681075	stable	ZNF490
0.504681075	stable	GAGE8
0.504681075	stable	GLIPR1L1
0.504798128	stable	LOC390211
0.504801877	stable	FIZ1
0.504801877	stable	TMEM138
0.504804932	stable	RBM23
0.504894771	stable	OR4C6
0.504894771	stable	LOC388476
0.505022141	stable	MSH4
0.505022141	stable	LOC650794
0.505078455	stable	RTTN
0.505205949	stable	LOC389599
0.505215696	stable	SCAND2
0.505215696	stable	LSS
0.505496767	stable	FLNB
0.505597919	stable	DRD2
0.505735739	stable	KRT76
0.505767632	stable	SNX7
0.505978486	stable	ZNF677
0.505978486	stable	LOC644635
0.505978486	stable	PALMD
0.505978486	stable	WIT-1
0.506050806	stable	LOC728939
0.506050806	stable	LOC646748
0.506050806	stable	LOC651441
0.506050806	stable	C14ORF48
0.506050806	stable	LOC732162
0.506050806	stable	QPCTL
0.506050806	stable	MXD1
0.506321709	stable	PHF15
0.506383995	stable	CYP51A1
0.506387315	stable	ALX3
0.506487037	stable	ZNF117
0.506487037	stable	FLJ25006
0.506487037	stable	LOC729647
0.506504551	stable	CDC2
0.506584353	stable	TRIM15
0.506623899	stable	HNF4A
0.506773014	stable	LOC651561
0.506912094	stable	SCNN1A
0.506922415	stable	CA7
0.507167659	stable	BTBD6
0.507167659	stable	UBP1
0.507167659	stable	SLC14A2
0.507167659	stable	LOC284402
0.507258084	stable	SPRR2A
0.507266763	stable	LOC645708
0.507267626	stable	SLC5A5
0.507295962	stable	SNRPD3

0.507295962	stable	SET
0.507441423	stable	SCAF1
0.507567941	stable	ROM1
0.507567941	stable	C18ORF20
0.507606035	stable	ATP2C2
0.507606035	stable	SETD6
0.50818274	stable	LOC653800
0.50818274	stable	LOC652906
0.50818274	stable	LSM2
0.50818274	stable	LOC728219
0.50818274	stable	RNASE13
0.50818274	stable	LOC728919
0.508602802	stable	LOC730862
0.508633188	stable	ELA1
0.508657093	stable	KRT80
0.509123306	stable	LOC401447
0.509123306	stable	LOC643149
0.509179862	stable	LOC647344
0.509402112	stable	EEF1A1
0.509402112	stable	ING5
0.509508184	stable	HSPB9
0.509568582	stable	RPS23
0.509568582	stable	ISG15
0.509807963	stable	LOC643326
0.509807963	stable	FSIP2
0.509837069	stable	LYZL4
0.510001739	stable	C6ORF58
0.510121051	stable	NDUFS7
0.510377585	stable	PTOV1
0.510556772	stable	LOC400965
0.510556772	stable	SLC12A7
0.510578741	stable	JAK3
0.510656423	stable	LOC647050
0.510656423	stable	LOC728912
0.510712256	stable	GDF7
0.510723562	stable	LOC652389
0.510745926	stable	LOC642711
0.510745926	stable	SYTL5
0.510887221	stable	ARHGAP6
0.510910505	stable	GHSR
0.511023158	stable	TAS2R14
0.511131373	stable	LOC648981
0.51115066	stable	FBXO22OS
0.51115066	stable	PACRG
0.51115066	stable	LOC727987
0.51115066	stable	PSENEN
0.51115066	stable	LOC654123
0.51115066	stable	DRP2
0.51115066	stable	ECSIT
0.51115066	stable	TMEM130
0.511240874	stable	LOC649540

0.511332743	stable	SERHL2
0.511332743	stable	LOC649851
0.511332743	stable	PAPD1
0.511383317	stable	LOC650869
0.511383317	stable	LOC643090
0.511426916	stable	LOC646090
0.511650579	stable	LOC644921
0.511650579	stable	KISS1R
0.511663977	stable	HSD17B7P2
0.511688365	stable	LOC653657
0.511688365	stable	LOC653272
0.51174003	stable	LOC121981
0.51174003	stable	LOC644305
0.51174003	stable	TAAR5
0.511941961	stable	LOC649305
0.512167962	stable	C2CD3
0.512209414	stable	LOC347292
0.512209414	stable	LOC647783
0.512230194	stable	NR2E3
0.51233562	stable	IFP38
0.512404259	stable	LOC651825
0.512404259	stable	LOC652730
0.512484574	stable	LOC650114
0.512563653	stable	LOC644548
0.512587626	stable	PRKG2
0.512588668	stable	FLJ25404
0.512820471	stable	LOC642144
0.512896617	stable	SUV39H2
0.513031877	stable	FAH
0.513033514	stable	MDGA2
0.513045204	stable	ZNF785
0.513053515	stable	OR8A1
0.513053515	stable	GHRH
0.513053515	stable	LOC652781
0.513420102	stable	KRTAP9-5
0.513722038	stable	LOC389286
0.513783329	stable	OR10K2
0.51384957	stable	POLR1D
0.513951463	stable	PLCXD3
0.514270943	stable	LRRC27
0.514391266	stable	LOC645724
0.514490154	stable	PIK3CB
0.514629545	stable	LOC653425
0.514728634	stable	LOC646665
0.514818383	stable	ENPP6
0.514972014	stable	RUVBL1
0.514972014	stable	TBC1D9B
0.514972014	stable	LHX8
0.515104184	stable	OR8K5
0.515104184	stable	LOC652255
0.515104184	stable	LOC645385

0.515371958	stable	DNAJB8
0.515371958	stable	LOC653601
0.515409013	stable	GAD1
0.515423063	stable	PTPLA
0.515423063	stable	HDX
0.51545901	stable	OR5L1
0.515493171	stable	LOC651468
0.515493755	stable	CRB1
0.515650402	stable	LOC645799
0.515650402	stable	MGC40499
0.515704212	stable	SYN1
0.515744011	stable	LOC641733
0.515785396	stable	LOC645651
0.515802108	stable	C2ORF48
0.515802108	stable	BMPR1B
0.515982757	stable	C1ORF220
0.516120068	stable	PRR8
0.516235401	stable	LOC92017
0.516352941	stable	KIF2B
0.516364747	stable	OR5H6
0.516520046	stable	LRRC4C
0.516552415	stable	LOC653104
0.516601203	stable	LOC441257
0.516960857	stable	KCNN1
0.516960857	stable	ENOPH1
0.517114032	stable	LOC647707
0.517139732	stable	SESN1
0.517189405	stable	LOC392395
0.517228261	stable	LOC641950
0.517438955	stable	TACSTD2
0.517829415	stable	SNAG1
0.517833787	stable	NKTR
0.517885688	stable	ARFIP2
0.517898701	stable	LOC652618
0.517910796	stable	C20ORF106
0.518168778	stable	ERGIC3
0.518168778	stable	C20ORF117
0.518168778	stable	OTOA
0.518189789	stable	DDX46
0.518303436	stable	DEADC1
0.518303436	stable	SLC6A20
0.51862211	stable	C3ORF24
0.518714289	stable	LOC286187
0.518966576	stable	TRPM8
0.51899739	stable	AURKC
0.519089473	stable	GPR75
0.519089564	stable	PI4KAP2
0.519089564	stable	PNMA2
0.519089564	stable	LOC401351
0.519089564	stable	SLC8A3
0.519089564	stable	UGT1A7

0.519118536	stable	CLK2P
0.519118536	stable	CACNG3
0.519120023	stable	C2ORF21
0.519220655	stable	LOC643272
0.519284389	stable	ATP6V1B1
0.519537597	stable	DDX41
0.51955679	stable	OR10X1
0.51955679	stable	LOC652790
0.51955679	stable	LOC728340
0.519584975	stable	TRAF7
0.519692226	stable	MATN4
0.519878421	stable	ERLIN1
0.519891594	stable	TTC28
0.519891594	stable	LOC647391
0.519891594	stable	GJB4
0.519891594	stable	IGFN1
0.519891594	stable	ABHD12B
0.519891594	stable	PPP1R12B
0.519891594	stable	LOC646897
0.519891594	stable	LOC652685
0.520323206	stable	LOC649327
0.520323206	stable	ERAP2
0.520323206	stable	LOC644131
0.520323206	stable	LOC643623
0.520323206	stable	LOC649497
0.520347639	stable	LOC652860
0.520520168	stable	TDRD5
0.520520168	stable	CDRT15P
0.520682577	stable	LOC283553
0.520712734	stable	ZNF182
0.520777425	stable	NLRP2
0.520796588	stable	LOC388022
0.520900205	stable	EIF2B2
0.521284126	stable	LOC642149
0.521284126	stable	ZFYVE20
0.521361142	stable	LOC554235
0.521384397	stable	CDKL5
0.521400372	stable	H2BFXP
0.521519905	stable	KIAA2013
0.521528757	stable	FRMPD3
0.521632509	stable	LARP1
0.521632509	stable	LOC654106
0.521760068	stable	LOC647983
0.521760068	stable	LOC650030
0.522125219	stable	GSDM1
0.522125219	stable	OTOL1
0.522125219	stable	LOC646303
0.522125219	stable	LDHAL6B
0.522163007	stable	LOC283874
0.522416549	stable	HHIPL2
0.522522929	stable	LOC731548

0.522522929	stable	LOC645284
0.52257581	stable	LOC652235
0.522584917	stable	GTF2IP1
0.522609961	stable	LOC342892
0.522609961	stable	P2RX7
0.522609961	stable	SAC
0.522644227	stable	LOC439951
0.522653638	stable	LOC648496
0.522816759	stable	ADAM5P
0.522816759	stable	NKD1
0.522816759	stable	SEMA3B
0.522816759	stable	LOC347475
0.522817324	stable	RIOK1
0.522817324	stable	LOC652557
0.522817324	stable	GPRC6A
0.522817324	stable	LOC650733
0.522817324	stable	PVRL1
0.523024313	stable	ANKRD45
0.523267644	stable	PAX2
0.523267644	stable	LOC652697
0.523267644	stable	GRHL2
0.523267644	stable	EVX2
0.523267644	stable	OCLM
0.523267644	stable	OR5AC2
0.523304918	stable	DYRK1A
0.523417277	stable	SRP54
0.523417277	stable	BTAF1
0.523417277	stable	FANK1
0.523528575	stable	HUWE1
0.523568891	stable	MEX3B
0.523616037	stable	LOC646484
0.523616037	stable	LOC647718
0.523616037	stable	LOC649563
0.523616037	stable	C6ORF154
0.523616037	stable	GLS2
0.523797428	stable	VN1R5
0.523828192	stable	TLX3
0.523828192	stable	LOC728608
0.523910732	stable	C13ORF39
0.524036665	stable	LOC643007
0.524036665	stable	LOC642374
0.524036665	stable	LOC651169
0.524052736	stable	KAAG1
0.524052736	stable	LOC392489
0.524141936	stable	LOC653353
0.524313107	stable	DR1
0.524313107	stable	ANKFY1
0.524362445	stable	LOC652451
0.524561158	stable	PEA15
0.524638662	stable	TIMM22
0.524701784	stable	LOC391165

0.52473502	stable	CBLC
0.524957958	stable	HEATR5B
0.524957958	stable	IFI44
0.525132895	stable	LOC642369
0.525247888	stable	STX6
0.525359811	stable	LOC644749
0.525359811	stable	FLJ38377
0.525359811	stable	ATP13A4
0.525359811	stable	MLF1
0.525404864	stable	LOC646803
0.525404864	stable	KLF16
0.525643316	stable	DGKB
0.525682547	stable	PAX8
0.525682547	stable	LRRC45
0.525682547	stable	C9ORF43
0.525682547	stable	LOC654159
0.525811881	stable	LOC641947
0.525848368	stable	LOC645871
0.525848368	stable	AOF1
0.52601011	stable	GABRR1
0.526022467	stable	LOC729269
0.526062046	stable	LOC654092
0.52607236	stable	HMGN4
0.526302326	stable	ACRC
0.526480836	stable	LOC647366
0.526685646	stable	LOC255649
0.526721872	stable	CMPK1
0.526838329	stable	LOC650557
0.526838329	stable	GJB6
0.527001492	stable	WDR86
0.527008162	stable	LOC648271
0.527023427	stable	FAM90A10
0.527023427	stable	CD1E
0.527055715	stable	C8ORF34
0.527094609	stable	OR1F2P
0.527335332	stable	UNCX
0.527480008	stable	LOC651864
0.527487485	stable	EXT1
0.527509933	stable	TFB2M
0.527822319	stable	HIST1H4L
0.527910199	stable	LOC440795
0.528049274	stable	KCNK9
0.528057984	stable	C10ORF114
0.528120461	stable	SDC4P
0.52815798	stable	LOC651091
0.528187654	stable	AP1GBP1
0.528187654	stable	ATP8B2
0.528354893	stable	LOC652238
0.528444739	stable	LOC642153
0.528444739	stable	FKSG49
0.528444739	stable	NRBP1

0.528496549	stable	LOC440291
0.528524073	stable	LOC441548
0.52855638	stable	LOC642781
0.528747252	stable	CHD4
0.528752577	stable	FAM20A
0.528752577	stable	SCRT2
0.52896339	stable	HLA-DQB2
0.52896339	stable	LOC644680
0.52896339	stable	VPREB3
0.529003443	stable	LOC653149
0.529158341	stable	LOC644666
0.529158341	stable	C14ORF169
0.529235488	stable	LOC387885
0.529235488	stable	LOC648790
0.529476295	stable	MEX3D
0.529476295	stable	LOC283104
0.529597622	stable	LOC646796
0.529597622	stable	LOC619207
0.529664649	stable	ZNF784
0.529675915	stable	NXPH2
0.529845803	stable	LOC255130
0.529846973	stable	C9ORF140
0.529846973	stable	C3ORF10
0.529846973	stable	VWA2
0.529953111	stable	HS6ST1
0.529953111	stable	RSPO3
0.530006855	stable	HCG4P6
0.530006855	stable	HUNK
0.530013821	stable	HTR1F
0.530013821	stable	LOC728117
0.530013821	stable	CADM4
0.530050374	stable	ABTB2
0.530141399	stable	LOC653735
0.530141399	stable	LOC286260
0.530141399	stable	LOC652510
0.530141399	stable	NFE2L2
0.530266875	stable	FZR1
0.530384648	stable	LOC643918
0.530510657	stable	KRTAP4-2
0.530628116	stable	LOC650107
0.53067946	stable	SLC6A13
0.530720725	stable	LOC652787
0.530835061	stable	LOC644717
0.530990089	stable	IFI6
0.531089144	stable	USP40
0.531089144	stable	APOBEC3H
0.531097021	stable	LOC650412
0.531097021	stable	LOC643990
0.531097021	stable	MLLT4
0.531141215	stable	LOC732424
0.531141215	stable	SCAMP4

0.531141215	stable	CLEC3A
0.531141215	stable	LOC652022
0.531141215	stable	CYP3A4
0.531240039	stable	PSMB10
0.531249187	stable	POMGNT1
0.531249187	stable	TRPM7
0.531285768	stable	NUTF2
0.531472819	stable	TG
0.531500256	stable	LOC648080
0.531581146	stable	ACP2
0.531581146	stable	FAM176B
0.531581146	stable	TAS2R4
0.531581146	stable	AGRP
0.531604852	stable	C1ORF126
0.531604852	stable	KIAA1632
0.531604852	stable	PRDX2
0.531623898	stable	LOC388242
0.531623898	stable	LBXCOR1
0.531629037	stable	HKDC1
0.531643023	stable	LOC650716
0.531753489	stable	SGK2
0.53208029	stable	PCDHGC5
0.532177263	stable	LOC649813
0.532446327	stable	S100A5
0.532547949	stable	EFCAB3
0.532594836	stable	CPS1
0.53272795	stable	RPTN
0.53272795	stable	CFHR5
0.53272795	stable	OR2H1
0.53272795	stable	LOC441546
0.532797044	stable	AMAC1L1
0.5328053	stable	LOC644325
0.5328053	stable	ENDOD1
0.532973898	stable	ATP8B3
0.532980262	stable	ZMAT2
0.532980262	stable	MUC7
0.532980262	stable	C13ORF27
0.533112258	stable	LOC401242
0.533196977	stable	CD70
0.533255826	stable	OR13H1
0.533255826	stable	LOC642637
0.533255826	stable	LOC731408
0.533255826	stable	LOC645291
0.533426226	stable	PPIL2
0.533426226	stable	LOC728747
0.533426226	stable	LOC651167
0.533426226	stable	DEFB124
0.533426226	stable	RGPD4
0.533426226	stable	VAMP2
0.533426226	stable	PCDHA1
0.533520568	stable	RSPH1

0.533520568	stable	XYLB
0.533564474	stable	RHOXF2B
0.533622988	stable	C1ORF57
0.533722346	stable	LOC643965
0.533722346	stable	LOC642338
0.533730558	stable	LOC727866
0.533906252	stable	CLLU1OS
0.533966615	stable	SIN3B
0.533966615	stable	MAGEA3
0.534215582	stable	ATP1A3
0.534252115	stable	EPB41L4B
0.53428369	stable	DKFZP434I0714
0.534345492	stable	C20ORF175
0.534377498	stable	LOC647348
0.534390527	stable	HNRPLL
0.534416832	stable	HMGCS1
0.534544739	stable	GPR37
0.534705054	stable	HBQ1
0.534804183	stable	LOC649162
0.534804183	stable	NBPF11
0.534804183	stable	LOC646804
0.534804183	stable	OR11H1
0.534804183	stable	TRPA1
0.534818741	stable	C3ORF44
0.534818741	stable	OR5AU1
0.534890373	stable	LOC647283
0.535065551	stable	PSMF1
0.535067018	stable	LOC645811
0.535161798	stable	LOC646350
0.535217486	stable	LOC441795
0.535217486	stable	SMARCAD1
0.535217486	stable	CLEC12B
0.535549165	stable	LOC641875
0.53583064	stable	LOC646098
0.53583064	stable	LOC652821
0.536496125	stable	LOC90113
0.536520827	stable	LOC441759
0.536520827	stable	OR2T34
0.536567354	stable	CCDC34
0.536567354	stable	FGF23
0.536601243	stable	VMD2L3
0.536666601	stable	LOC729408
0.536690237	stable	THAP11
0.536933517	stable	SYNGR1
0.536964958	stable	ENPP7
0.536970161	stable	TCL1A
0.536998181	stable	GRIN2A
0.536998181	stable	CRABP1
0.537068549	stable	LOC649857
0.537086673	stable	UNC5A
0.537122473	stable	LOC644348

0.537122473	stable	LOC402635
0.537122473	stable	ACTL7A
0.537237528	stable	ZNF805
0.537237528	stable	LOC646033
0.537237528	stable	LOC644827
0.53730241	stable	RAPGEF4
0.537478078	stable	C6ORF12
0.537478078	stable	TFAP2E
0.537575645	stable	MGMT
0.537582684	stable	LOC647359
0.537582684	stable	LOC645293
0.53760259	stable	CYP21A2
0.537782592	stable	LOC139116
0.537888847	stable	ZNF19
0.53800839	stable	GREB1
0.538179947	stable	LOC650698
0.538179947	stable	HIST1H4D
0.538255026	stable	DNAJC7
0.538255026	stable	SGPP1
0.538255026	stable	C9ORF29
0.53828223	stable	ZBTB24
0.53828223	stable	TRPV4
0.53828223	stable	LOC645349
0.538314058	stable	LOC440337
0.53833935	stable	GIPC2
0.538559257	stable	LOC388621
0.538559257	stable	LOC387826
0.538648021	stable	KRTAP4-1
0.538652459	stable	LOC643882
0.538916305	stable	OR10G4
0.538916305	stable	COX18
0.539190352	stable	LOC646908
0.539431427	stable	RGL2
0.539481835	stable	C6ORF79
0.539601855	stable	SCN7A
0.53965302	stable	LOC284801
0.539700332	stable	CYORF14
0.53994818	stable	FNDC7
0.539964142	stable	H1F0
0.539964142	stable	TTC21B
0.539964142	stable	LOC644814
0.539964142	stable	WIPF1
0.539964142	stable	LOC648064
0.539964142	stable	TAPBP
0.540017289	stable	OTOF
0.54016939	stable	HIST3H3
0.540183607	stable	LOC441126
0.540204439	stable	USP42
0.540204439	stable	LOC653123
0.540397292	stable	KRT12
0.540441052	stable	FLJ22269

0.540511273	stable	CCNB2
0.540556138	stable	ATG4A
0.540746537	stable	LOC643580
0.540985447	stable	KCNN2
0.541007152	stable	SLITRK5
0.541021551	stable	FLJ44881
0.541095691	stable	LOC649812
0.541095691	stable	GPSN2
0.541095691	stable	DCC
0.541095691	stable	ZNF828
0.541255451	stable	MRM1
0.541261196	stable	LOC645304
0.541408223	stable	LY9
0.541515033	stable	LOC642528
0.541515033	stable	LOC653165
0.541587162	stable	THAP7
0.541587162	stable	HOMEZ
0.541998612	stable	KCNH7
0.542069692	stable	TMEFF1
0.542069692	stable	TNR
0.542069692	stable	C3ORF56
0.542069692	stable	FLJ45202
0.542074888	stable	OR4D5
0.542190768	stable	SULT1E1
0.542344775	stable	ORC1L
0.542359174	stable	SPANXN1
0.542434115	stable	PAK7
0.542600767	stable	CUTC
0.542600767	stable	LOC653539
0.542600767	stable	LOC651769
0.542752906	stable	PRICKLE3
0.542752906	stable	DCDC1
0.542752906	stable	LOC651044
0.542754927	stable	CRTAM
0.542819456	stable	LOC648066
0.543214358	stable	LOC652274
0.543214358	stable	LOC648763
0.543250898	stable	LOC649362
0.54332015	stable	RRAS2
0.54332015	stable	KPTN
0.543454884	stable	LOC644978
0.543804381	stable	DGKG
0.54381059	stable	VANGL1
0.54381059	stable	LOC648966
0.544168841	stable	MED9
0.544201067	stable	RBMY1B
0.544437575	stable	ZNF333
0.544508128	stable	LOC644267
0.544669007	stable	PHACTR3
0.544672688	stable	LOC645099
0.544911918	stable	LOC728004

0.544911918	stable	SIKE
0.545007318	stable	CTLA4
0.545272052	stable	LOC645956
0.545272251	stable	ST18
0.545272251	stable	TMEM75
0.545458481	stable	QRSL1
0.545458481	stable	POLQ
0.545524593	stable	BACE1
0.54565412	stable	LOC652070
0.545712641	stable	UBE2S
0.545755086	stable	ADRA2B
0.546071857	stable	LOC649784
0.546233342	stable	FLJ39660
0.546233342	stable	FAM90A12
0.546308071	stable	LOC646719
0.546340357	stable	LIPL2
0.546398092	stable	LOC731640
0.54640221	stable	LOC652579
0.546435041	stable	POLR1B
0.54653546	stable	LY75
0.546549256	stable	TNN
0.546560526	stable	LOC393076
0.546571675	stable	LOC284964
0.546579973	stable	LOC643237
0.546676912	stable	LOC647485
0.546715319	stable	FAM10A6
0.546715319	stable	LOC644554
0.546982409	stable	OR4K14
0.547022993	stable	PLXDC1
0.547022993	stable	SNORD48
0.547022993	stable	LOC387895
0.547022993	stable	ANRIL
0.547022993	stable	ATIC
0.547022993	stable	PPP1R2P9
0.547022993	stable	ZNF474
0.547022993	stable	HAT1
0.547022993	stable	KLF7
0.547022993	stable	LOC732150
0.547022993	stable	PLEKHA9
0.547022993	stable	DAP
0.547131837	stable	GPR151
0.547131837	stable	TMC2
0.547131837	stable	FLJ41766
0.54728695	stable	LOC649217
0.54728695	stable	FAM181A
0.54728695	stable	XCR1
0.54728695	stable	OR4B1
0.54742106	stable	LPAL2
0.54742106	stable	ARMC5
0.547513931	stable	LOC648403
0.547714159	stable	FLJ46154

0.547720522	stable	LOC440105
0.547737889	stable	PCDHGA4
0.547802014	stable	ZNF660
0.547843769	stable	ZNF396
0.547947078	stable	MGAT3
0.547947078	stable	ALDH1L1
0.548013868	stable	ZNF542
0.548013868	stable	LOC644058
0.548013868	stable	GSG2
0.548013868	stable	AGPS
0.548013868	stable	SART3
0.548013868	stable	AMOTL1
0.548176475	stable	LOC653206
0.548176475	stable	AMAC1L2
0.548176475	stable	C19ORF26
0.548246471	stable	TBC1D10A
0.548299115	stable	ALS2CR14
0.548369354	stable	LOC646740
0.548369354	stable	LOC402096
0.548401236	stable	KIAA1862
0.548401682	stable	PFKL
0.548401682	stable	LOC400499
0.548548353	stable	AIM1L
0.548548353	stable	TRPT1
0.548548353	stable	LOC642576
0.548634845	stable	SMAD6
0.548851978	stable	CXCL5
0.548893015	stable	CYP4F12
0.548958194	stable	RANGRF
0.54900804	stable	LOC644682
0.54900804	stable	FMO6P
0.549042725	stable	IL20RB
0.549104084	stable	LOC645663
0.549430287	stable	WDR3
0.549465263	stable	LOC647247
0.549465263	stable	ASTN2
0.549465263	stable	FAM14B
0.549465263	stable	LOC400696
0.549563905	stable	SCD5
0.549584157	stable	LOC650155
0.549605341	stable	OR2B3P
0.549717623	stable	LOC642684
0.549965373	stable	PRB3
0.54999101	stable	DHODH
0.54999101	stable	RGS9
0.54999101	stable	KSR2
0.550191414	stable	NUDT16
0.550227173	stable	RHEBL1
0.550325857	stable	HPD
0.550388644	stable	LOC652179
0.55047572	stable	DDEF1

0.550487131	stable	LOC440104
0.550631539	stable	EID3
0.550664147	stable	ITGA4
0.550664147	stable	ANKS6
0.550676683	stable	HS3ST6
0.550735369	stable	LOC645504
0.55081477	stable	ZNF595
0.550980468	stable	LOC644816
0.55111123	stable	FCRL3
0.551119282	stable	LOC651195
0.551119608	stable	MAN1A1
0.551126233	stable	GPR142
0.551193082	stable	OR2A1
0.551193082	stable	RGAG4
0.551432232	stable	FAM90A6P
0.55144851	stable	TNP2
0.551468865	stable	FLJ25076
0.551562755	stable	TMEM207
0.551562755	stable	PDPR
0.551562755	stable	LOC645458
0.551562755	stable	TCEAL1
0.551562755	stable	FGFBP3
0.551562755	stable	TEKT5
0.55160934	stable	ZNF553
0.55160934	stable	OR13D1
0.55160934	stable	C9ORF97
0.551730265	stable	LOC646731
0.551797534	stable	CFC1
0.551801554	stable	DYDC2
0.551854201	stable	S100A7L2
0.551916979	stable	SLC23A1
0.552030533	stable	LOC643423
0.552232508	stable	MYF6
0.552232508	stable	FAM22G
0.552232508	stable	NR1H4
0.552424965	stable	SLC35F5
0.552513613	stable	ZNF449
0.552617886	stable	UROC1
0.552617886	stable	LOC642950
0.552617886	stable	CABP5
0.552617886	stable	LOC646096
0.552797714	stable	CDH12P
0.552829561	stable	OLFM3
0.552837424	stable	FBLN1
0.552837424	stable	PAGE4
0.552840498	stable	RGPD5
0.552847022	stable	HIST2H2AA3
0.552847022	stable	PPP4R4
0.552874356	stable	LOC136288
0.552874356	stable	DLX3
0.553020357	stable	LOC728504

0.553020357	stable	LOC645279
0.553063425	stable	NDUFA4
0.553250311	stable	TTLL9
0.553250311	stable	KLK15
0.553250311	stable	LHFPL3
0.553250311	stable	LOC649270
0.553250311	stable	RBMY1F
0.553250311	stable	BPESC1
0.553250311	stable	MRPL28
0.553250311	stable	HMGCR
0.553250311	stable	AP1M2
0.553250311	stable	AVIL
0.553293246	stable	IQCB1
0.553293246	stable	DNCL2A
0.553293246	stable	DIO1
0.553293246	stable	OTUD6B
0.55339632	stable	LOC728403
0.553468757	stable	LOC653115
0.553648294	stable	LOC641929
0.553687037	stable	LOC645172
0.553840885	stable	KIAA1772
0.553898554	stable	TAAR9
0.553898554	stable	LOC642702
0.553993771	stable	JDP2
0.554072588	stable	ITGB8
0.554317841	stable	C19ORF62
0.554725476	stable	NPBWR1
0.554772284	stable	SNAP91
0.554805398	stable	TFR2
0.554989184	stable	CCR10
0.555062275	stable	KIF23
0.555194349	stable	PLD4
0.555194349	stable	LOC646933
0.555194349	stable	GBX2
0.555200346	stable	HCG22
0.555399892	stable	LOC441282
0.555515912	stable	COL11A2
0.555604503	stable	ARGFX
0.555604503	stable	LOC648600
0.555604503	stable	KRT84
0.555616815	stable	FAM7A2
0.555628949	stable	LOC732416
0.555709816	stable	FLJ44817
0.555713332	stable	XRCC5
0.555763788	stable	SULT1C2
0.555851489	stable	LOC648364
0.555851489	stable	NUP155
0.555874939	stable	LRP6
0.555887098	stable	COPS7A
0.556101731	stable	OR10R2
0.556154162	stable	LOC392215

0.556207454	stable	TAGAP
0.556222015	stable	SLC6A4
0.556222015	stable	LOC651777
0.556222015	stable	B3GALT1
0.556222015	stable	PLXNB3
0.556222015	stable	LOC391276
0.556222015	stable	LOC651268
0.556393552	stable	VCL
0.556418538	stable	C4ORF37
0.556418538	stable	CLECL1
0.556547796	stable	LOC642265
0.556547796	stable	HILS1
0.556604646	stable	APBA2
0.556711944	stable	FBXO41
0.556711944	stable	CLGN
0.556711944	stable	CXORF58
0.556863291	stable	ERAF
0.556863291	stable	DMXL1
0.557138488	stable	LINS1
0.557207405	stable	CAMKK1
0.55723888	stable	LOC647336
0.557384321	stable	RPS25
0.557810727	stable	LOC645194
0.557901678	stable	LOC651381
0.558008099	stable	LOC645879
0.558008099	stable	ADNP2
0.558112263	stable	LOC652160
0.558155995	stable	UGT1A9
0.55886166	stable	ATP2A3
0.558862349	stable	ECHDC3
0.558862349	stable	SPCS1
0.558867131	stable	NPY5R
0.558949088	stable	LOC126520
0.558968931	stable	LOC652405
0.559034339	stable	C16ORF55
0.55909277	stable	LOC646855
0.559109192	stable	LOC284701
0.559125392	stable	SHD
0.559125392	stable	KAT5
0.559125392	stable	LOC650475
0.559226677	stable	PIK3C3
0.559276599	stable	KCTD7
0.559566302	stable	LOC730076
0.559880188	stable	SST
0.559880188	stable	GLYAT
0.560096863	stable	MGC24103
0.560096863	stable	LOC644057
0.560230357	stable	OR52I1
0.560384237	stable	LOC645333
0.560420963	stable	LOC649092
0.560477919	stable	RPA4

0.560477919	stable	LOC653249
0.560549966	stable	LOC641908
0.560840228	stable	LOC651031
0.560888843	stable	SMA4
0.560910882	stable	LOC648963
0.560937704	stable	LOC645659
0.560957952	stable	GYPA
0.560976979	stable	RPE
0.560976979	stable	OR8K3
0.560976979	stable	LOC653073
0.560976979	stable	DHRS1
0.561092141	stable	LOC645179
0.561092141	stable	LOC442057
0.561152007	stable	LOC401498
0.561288567	stable	DEC1
0.561306623	stable	TSPAN33
0.561345539	stable	LOC731898
0.561378416	stable	MICAL3
0.561589765	stable	CRHR2
0.561589765	stable	FBXO43
0.561589765	stable	CASKIN2
0.561589765	stable	OLA1
0.561654614	stable	RPL23AP7
0.561669825	stable	ALOX12B
0.561742534	stable	LOC652086
0.561742534	stable	LOC285299
0.561742534	stable	LOC648329
0.561762363	stable	MEP1A
0.561762363	stable	TMEM175
0.561762363	stable	FLJ35773
0.561789206	stable	OR51A4
0.561847408	stable	LNP1
0.561847408	stable	UTP20
0.561969756	stable	LOC644285
0.561991666	stable	LOC650509
0.562036117	stable	LOC389370
0.562073353	stable	LOC389813
0.562073353	stable	HSD11B2
0.562278625	stable	LOC138652
0.562300493	stable	MAGEA9
0.562348081	stable	LOC728565
0.56240207	stable	LOC389457
0.562623841	stable	IFIT1L
0.562623841	stable	LOC648796
0.562929867	stable	SLC6A3
0.56334233	stable	ZNF385C
0.563357368	stable	LOC646492
0.563392234	stable	LOC652743
0.563480946	stable	LOC644090
0.56359321	stable	DPH1
0.563656224	stable	LOC645317

0.563697161	stable	LOC652529
0.563795341	stable	BTF3L4
0.563806212	stable	LOC647154
0.563806212	stable	LOC440917
0.563937862	stable	CDX1
0.564003762	stable	LOC647960
0.564012529	stable	LOC728103
0.564012529	stable	MUC4
0.564211932	stable	ERICH1
0.564274143	stable	NEDD4
0.564469762	stable	DEFA6
0.564483159	stable	OR6C65
0.564758908	stable	PRLR
0.564915241	stable	EEFSEC
0.564915241	stable	CASP8AP2
0.564915241	stable	LOC642347
0.564915241	stable	KCNJ14
0.564915241	stable	OPRK1
0.564915241	stable	OR4A15
0.564949922	stable	LOC641311
0.565074026	stable	MAGEA10
0.565157057	stable	LOC650185
0.565186368	stable	PRPF3
0.56537294	stable	BMP3
0.56537294	stable	LOC648480
0.565386702	stable	WDR12
0.565405453	stable	SLC5A9
0.565454524	stable	PSG2
0.565454524	stable	LOC729837
0.565459076	stable	CYP17A1
0.565533353	stable	KRT15
0.565533353	stable	FAM46D
0.565591299	stable	RFX6
0.565981796	stable	CHST5
0.565981796	stable	LOC645636
0.566213674	stable	GPC2
0.566362419	stable	HSPA4
0.566370538	stable	DVL1
0.566399713	stable	C11ORF84
0.566487727	stable	LOC652805
0.566487727	stable	SPIN2A
0.566487727	stable	GRIN1
0.566487727	stable	LOC652566
0.566753123	stable	CEP192
0.566776639	stable	APOBEC1
0.566978763	stable	USP51
0.567109581	stable	METTL7A
0.567138617	stable	LOC641802
0.567463845	stable	LOC653591
0.567463845	stable	C1ORF38
0.567569806	stable	FAM96A

0.567569806	stable	FGF16
0.567633982	stable	LOC391282
0.567676457	stable	LOC644099
0.567722834	stable	PDE8B
0.567723156	stable	KNG1
0.567789598	stable	SLC25A10
0.567789598	stable	DAZ4
0.56783239	stable	DDA1
0.56783239	stable	TCF19
0.56783239	stable	LOC441261
0.56783239	stable	LOC731444
0.567853114	stable	LOC284577
0.567874278	stable	ZNF804B
0.56818006	stable	LOC643006
0.568218636	stable	GPR144
0.568279699	stable	LOC402117
0.568353533	stable	CC2D1B
0.568353533	stable	CD7
0.568628432	stable	ROPN1
0.568696722	stable	C18ORF45
0.568748716	stable	MFRP
0.568863866	stable	NAPSB
0.568901086	stable	LOC440934
0.568975245	stable	MMP7
0.569048745	stable	LOC650298
0.569048745	stable	NEUROD4
0.569126607	stable	CCDC28B
0.569131168	stable	HLXB9
0.569185558	stable	LOC727778
0.569185558	stable	LOC441249
0.569185558	stable	C7ORF34
0.569416973	stable	CLDN4
0.569416973	stable	SQLE
0.569416973	stable	OR2W3
0.569469728	stable	ANKRD21
0.569469728	stable	GRHL1
0.569469728	stable	OTOP2
0.569507468	stable	LOC644043
0.569507468	stable	LOC653917
0.569555813	stable	LOC649546
0.56961553	stable	FLJ16171
0.569730533	stable	RNF32
0.569805596	stable	CLRN3
0.569819319	stable	CLPX
0.569819319	stable	LOC648039
0.569819319	stable	GATAD2B
0.569898356	stable	FMN2
0.569898356	stable	LOC653623
0.57000605	stable	LOC651979
0.570035854	stable	HAP1
0.570106567	stable	RLBP1

0.570106567	stable	LOC654134
0.570304031	stable	SNF8
0.57054259	stable	CRISP1
0.570614843	stable	LOC644975
0.570614843	stable	LOC401876
0.570614843	stable	LOC728832
0.570614843	stable	C6ORF170
0.570614843	stable	SNCB
0.570664783	stable	LOC643355
0.570764016	stable	PRPF38A
0.570764016	stable	DDX47
0.570817323	stable	LYCAT
0.571115468	stable	LOC730027
0.571152602	stable	ZNF786
0.571152602	stable	LOC648700
0.571152602	stable	GTF3C4
0.571189477	stable	HMHB1
0.571315292	stable	SFT2D2
0.571315292	stable	CRYGD
0.571589588	stable	GOSR1
0.571589588	stable	C6ORF191
0.571875846	stable	PROC
0.571961013	stable	TMEM131
0.572111106	stable	LOC643152
0.572385128	stable	CCDC63
0.572645706	stable	LOC400707
0.572645706	stable	LOC649723
0.572645706	stable	ZNF670
0.572645706	stable	TLE2
0.572802882	stable	LOC442283
0.572812616	stable	LOC644059
0.573005033	stable	PTF1A
0.573128903	stable	LOC644845
0.573351846	stable	LOC642313
0.574096921	stable	MRPL2
0.574131494	stable	SEPHS2
0.574237529	stable	B3GNTL1
0.574259422	stable	LOC644234
0.574340234	stable	FLJ43806
0.574366441	stable	DHDH
0.574482294	stable	OR2A14
0.574482294	stable	LOC440341
0.574543044	stable	UBA7
0.574605315	stable	LOC644319
0.574783085	stable	ITPK1
0.57483475	stable	ICAM4
0.57483475	stable	KRT222P
0.574841707	stable	LOC642985
0.574841707	stable	SP8
0.575177412	stable	SHH
0.575177412	stable	FOXD1

0.575390275	stable	LOC644044
0.575390275	stable	KCNJ3
0.575395442	stable	LOC651668
0.575555137	stable	LOC653759
0.5755761	stable	LOC652793
0.575611136	stable	MEP1B
0.575611136	stable	LOC728116
0.575611136	stable	MRPL42
0.575611136	stable	LOC285095
0.575781197	stable	LOC649150
0.575860126	stable	LOC402641
0.576096289	stable	CHD8
0.576258964	stable	SERAC1
0.576258964	stable	LOC441874
0.576258964	stable	LOC390877
0.576334067	stable	BLID
0.576334067	stable	NOL10
0.576437982	stable	HTR7
0.57643872	stable	LOC340094
0.576590492	stable	ISCA2
0.576697378	stable	RTN2
0.576735948	stable	AVPI1
0.576815579	stable	LOC646920
0.57704719	stable	TRHR
0.57704719	stable	ASCL3
0.57704719	stable	H2AFB1
0.57704719	stable	SLC25A32
0.577140079	stable	KCTD10
0.577154917	stable	TRIM13
0.577154917	stable	LOC642939
0.577201041	stable	CHORDC1
0.577201041	stable	LOC648684
0.577372654	stable	PEX12
0.577372654	stable	IGF1R
0.577515141	stable	LOC653867
0.577569728	stable	CD19
0.577569728	stable	TNNI2
0.577702634	stable	LOC651987
0.577762413	stable	TGM7
0.577762413	stable	LOC343296
0.577762413	stable	OTOS
0.577834722	stable	UBQLN3
0.577959516	stable	LOC646946
0.578069632	stable	SLC43A1
0.578111479	stable	SH3GL3
0.578111479	stable	LOC729957
0.578111479	stable	FLJ40504
0.578111479	stable	FLJ31945
0.578111479	stable	EIF5B
0.578283762	stable	ESR2
0.578758025	stable	LOC255025

0.579013154	stable	LOC644421
0.579137899	stable	SPSB4
0.57919181	stable	LECT2
0.57919181	stable	MGC13005
0.579291849	stable	LOC642680
0.579291849	stable	DLEU2
0.579291849	stable	FHL1
0.579291849	stable	C2ORF58
0.579307179	stable	C3ORF48
0.579307179	stable	LOC652466
0.579362447	stable	LOC650815
0.579451286	stable	GPR123
0.579451286	stable	SNED1
0.579535309	stable	OR1L8
0.579535309	stable	FLJ40448
0.579535309	stable	CYP19A1
0.579535309	stable	BCL2L15
0.579535309	stable	UBE2C
0.579535309	stable	C10ORF28
0.579535309	stable	LOC649765
0.579535309	stable	LOC650455
0.579955522	stable	ZBED3
0.580015943	stable	PLA2G2F
0.580038434	stable	NUMA1
0.580043685	stable	LOC401525
0.58012505	stable	BXDC2
0.58012505	stable	IRGM
0.58012505	stable	WNT10B
0.58012505	stable	LOC652396
0.58012505	stable	ERG
0.58012505	stable	CD226
0.580138075	stable	KCNC3
0.580313024	stable	LOC647630
0.580332409	stable	C1ORF91
0.58036885	stable	CNGA2
0.580379398	stable	LOC652341
0.580379398	stable	PSEN2
0.580619158	stable	LOC643734
0.580895232	stable	LOC648803
0.580917099	stable	CYP4F3
0.581056051	stable	C5ORF34
0.581256968	stable	CNTLN
0.581293075	stable	CCDC148
0.581293075	stable	LOC646580
0.581379409	stable	MPG
0.581379409	stable	TFF1
0.581573124	stable	BFSP2
0.581573124	stable	FLJ16124
0.58171211	stable	RIN1
0.581793071	stable	MAGEB6B
0.581822227	stable	LGALS4

0.581849752	stable	DOC2A
0.581849752	stable	WDR23
0.581849752	stable	HCCA2
0.582040406	stable	LOC645529
0.582093732	stable	ZP4
0.58213773	stable	LOC646443
0.58213773	stable	LOC653653
0.582158004	stable	ADAM12
0.582158004	stable	FAM87B
0.582189889	stable	TTTY6
0.582368951	stable	ADCK4
0.582434982	stable	SFTPB
0.582542499	stable	MYOC
0.582657658	stable	HNRPUL2
0.582657658	stable	PSD
0.582881152	stable	LOC643988
0.582881152	stable	RBM35A
0.582881152	stable	WNT3
0.582881152	stable	GPR87
0.582881152	stable	GSTA5
0.582888787	stable	RGS20
0.583029111	stable	LGR4
0.583029111	stable	DDX19A
0.583277946	stable	RASL10B
0.583472332	stable	CTAGE1
0.58348859	stable	LST-3TM12
0.58348859	stable	GABRA6
0.583506813	stable	LOC651876
0.583932679	stable	ETFDH
0.583932679	stable	LOC650942
0.584015064	stable	LOC645039
0.584315268	stable	C10ORF113
0.584315268	stable	GPR182
0.58433638	stable	C10ORF90
0.58433638	stable	LOC728160
0.58446527	stable	SEMA3A
0.584509596	stable	FOXA3
0.584693777	stable	CCDC68
0.584696768	stable	ERCC4
0.585242998	stable	OR8G5
0.585508526	stable	LOC652202
0.585511003	stable	LDLRAD1
0.58557518	stable	LOC648792
0.585594307	stable	PKHD1
0.585626798	stable	LOC143941
0.585626798	stable	RPRML
0.585671608	stable	NLGN3
0.585760639	stable	DCP1B
0.585783835	stable	GUCA1A
0.585826618	stable	LOC648752
0.5859184	stable	DHX58

0.5859184	stable	TAS2R48
0.5859184	stable	CFHR4
0.586163823	stable	MOGAT3
0.586173995	stable	SLC37A1
0.586241984	stable	ZDHHC3
0.586259789	stable	CSN2
0.586867765	stable	LOC650020
0.58698557	stable	LOC642594
0.58710375	stable	FSTL4
0.58710375	stable	NTN2L
0.58710375	stable	ZNF683
0.58710375	stable	LCE1D
0.58710375	stable	KIAA1627
0.58710375	stable	CCDC123
0.587134192	stable	LOC644277
0.587182252	stable	FGL1
0.587266947	stable	BPIL2
0.587333396	stable	XAGE1D
0.587333396	stable	DCN
0.587333396	stable	WNT8A
0.587336468	stable	MCC
0.587369565	stable	TATDN1
0.58741848	stable	ZNF358
0.58755776	stable	LOC389142
0.58755776	stable	UNC5D
0.58755776	stable	LOC497256
0.58755776	stable	LOC143678
0.587567787	stable	OTC
0.587567787	stable	LOC441135
0.587576964	stable	FMO9P
0.587678936	stable	TTTY19
0.587840402	stable	PKIB
0.588030573	stable	LOC390364
0.588048194	stable	LOC644278
0.588226661	stable	LOC651353
0.588237326	stable	TTTY18
0.588399417	stable	CPN1
0.588410285	stable	LOC653093
0.588602309	stable	PRAMEF5
0.588602309	stable	MED4
0.588602309	stable	LOC389111
0.588689027	stable	LOC643083
0.588818249	stable	LOC338667
0.589096129	stable	LOC651390
0.589096467	stable	TPTE2
0.589183183	stable	FUT11
0.589505959	stable	HBM
0.589515758	stable	HMGCS2
0.589516847	stable	MLNR
0.589559495	stable	MTMR6
0.590030702	stable	LOC652670

0.59004392	stable	LOC642082
0.59004392	stable	SFRS2B
0.590234875	stable	DSCR10
0.590313617	stable	LOC643841
0.590346739	stable	LOC647456
0.590346739	stable	SLC24A3
0.590822974	stable	LOC728643
0.59085179	stable	C12ORF32
0.59098915	stable	RELT
0.59098915	stable	PCDHB1
0.591049316	stable	OR13C4
0.591049316	stable	LOC642574
0.591049316	stable	LOC649067
0.591049316	stable	MCM7
0.591049316	stable	CCDC105
0.591049316	stable	GPR103
0.591150449	stable	SLC38A11
0.591178634	stable	LOC648716
0.591181405	stable	LOC390372
0.591181405	stable	LOC643433
0.591497023	stable	ANKRD49
0.591497023	stable	LOC441114
0.591708746	stable	LOC644280
0.591791107	stable	TEX13B
0.591791107	stable	LOC440295
0.591888236	stable	CCDC54
0.591889084	stable	COLEC12
0.591889084	stable	LOC652651
0.592159898	stable	LOC649227
0.592313769	stable	BANK1
0.592387998	stable	FAM171B
0.592603491	stable	LOC645079
0.592630004	stable	EIF5A2
0.592630004	stable	LOC285500
0.592630004	stable	CENPI
0.592876226	stable	LOC641751
0.592979905	stable	DENND1C
0.592980151	stable	HOXA9
0.592980151	stable	LOC647579
0.593076045	stable	TMEM38B
0.593263702	stable	MUC17
0.593434431	stable	FLJ42986
0.593434431	stable	OR4F4
0.593434431	stable	LOC644326
0.593434431	stable	SPANXN5
0.593434431	stable	SYS1
0.593546123	stable	C1ORF92
0.593546123	stable	LOC644964
0.593718682	stable	CHRNG
0.593720779	stable	ATP4A
0.593822269	stable	C15ORF20

0.593935981	stable	LOC651809
0.593950585	stable	ZNF253
0.594214725	stable	KCNJ6
0.594387066	stable	PWWP2B
0.594397221	stable	ZMYND15
0.594408686	stable	LOC147975
0.594425667	stable	POU1F1
0.594456611	stable	IFITM5
0.59461208	stable	TMEM35
0.594636977	stable	ZNF648
0.594860423	stable	RLTPR
0.594992069	stable	KIAA0329
0.595319285	stable	LOC652552
0.595611166	stable	CH25H
0.595611166	stable	DHRS8
0.595611166	stable	C6ORF136
0.595830643	stable	LOC647244
0.59585541	stable	GAB4
0.595856219	stable	CREBBP
0.595856219	stable	NFATC2
0.595873302	stable	KRT33B
0.59596772	stable	GP6
0.59596772	stable	TRMT2B
0.59596772	stable	LOC653584
0.59596772	stable	LOC641860
0.595976037	stable	LOC345051
0.595976037	stable	MICA
0.595976037	stable	SOAT2
0.596531852	stable	LOC642580
0.596531852	stable	LRP11
0.596531852	stable	LOC651553
0.596537996	stable	LOC643900
0.596701265	stable	LOC388160
0.596714115	stable	LOC643233
0.59681185	stable	LBX2
0.596853738	stable	C10ORF122
0.59700943	stable	LOC647191
0.597010628	stable	ACACA
0.597076183	stable	NXF4
0.59711074	stable	CDK5R1
0.59711074	stable	ZNF554
0.597421731	stable	INF2
0.59756772	stable	TANK
0.59767179	stable	FLJ25715
0.597982287	stable	ANKRD1
0.597982287	stable	ALPPL2
0.597982287	stable	LOC643721
0.597982287	stable	PLCD1
0.598004415	stable	UHRF2
0.598057027	stable	INA
0.598064097	stable	LOC644302

0.598064097	stable	LOC650836
0.598118043	stable	LOC651012
0.598203219	stable	LOC644225
0.598230293	stable	IL28A
0.598522788	stable	LOC652294
0.598522788	stable	LOC641714
0.598522906	stable	OBSCN
0.598674184	stable	UBE2E2
0.598674184	stable	LOC651965
0.598674184	stable	SSX6
0.598674184	stable	LOC652134
0.598748041	stable	LOC653240
0.599002893	stable	LOC649548
0.599010298	stable	MPP1
0.599156337	stable	LOC653817
0.599343473	stable	TMEM137
0.599349746	stable	MYO6
0.599349746	stable	FAM90A20
0.599360133	stable	LOC645634
0.599363169	stable	ZNF33B
0.599478522	stable	LOC643533
0.599478522	stable	LOC642425
0.599494296	stable	CPA2
0.599774704	stable	FLJ41046
0.600021024	stable	MXD3
0.600059654	stable	LOC442329
0.600113184	stable	LOC647648
0.600398865	stable	NHLRC1
0.600583642	stable	SLC45A3
0.600583642	stable	LOC643594
0.600583642	stable	LOC642626
0.600583642	stable	LOC731052
0.600583642	stable	LOC644923
0.600730534	stable	TM4SF19
0.601169353	stable	TEX28
0.601195993	stable	ITPR2
0.601244656	stable	LOC644351
0.60126817	stable	RALYL
0.601376188	stable	PHYHIPL
0.601376188	stable	ADCY8
0.601674447	stable	LOC644126
0.60201795	stable	LOC652476
0.602095796	stable	HTR2A
0.602101169	stable	INSM2
0.602167259	stable	LOC648569
0.602270133	stable	CSRP3
0.602377125	stable	TRMT12
0.602444674	stable	FGD5
0.602444674	stable	LOC644981
0.602691191	stable	VNN3
0.602691191	stable	LOC643624

0.602778607	stable	LOC645627
0.602883837	stable	PTDSS2
0.603015865	stable	LOC339047
0.603015865	stable	LOC642160
0.603021542	stable	LOC642103
0.603021542	stable	LOC729370
0.603021542	stable	CTSE
0.603046628	stable	RBM
0.6032169	stable	KIAA0953
0.603283114	stable	LOC642049
0.603630039	stable	CLSTN3
0.603677526	stable	MAGI3
0.603677526	stable	TEP1
0.603857691	stable	C14ORF82
0.603991385	stable	DISP2
0.603998637	stable	DONSON
0.604070309	stable	SCG3
0.604070309	stable	LOC644785
0.604292788	stable	LOC731049
0.604340705	stable	OR51A7
0.604584448	stable	LOC643492
0.604584448	stable	LOC642268
0.604830595	stable	TMEM65
0.604830595	stable	STYK1
0.604830595	stable	NPTN
0.60489812	stable	TM4SF20
0.605240983	stable	MGC40574
0.605248709	stable	LOC645891
0.605298908	stable	PTGR2
0.605535909	stable	LOC646698
0.605535909	stable	LOC644359
0.605569109	stable	LOC730274
0.605657822	stable	AADACL4
0.605721813	stable	C11ORF76
0.605898878	stable	CDC26
0.605938744	stable	LOC652491
0.605938744	stable	TMEM85
0.605938744	stable	FHL5
0.606065539	stable	LOC648050
0.606095335	stable	MEPE
0.606104473	stable	RDH12
0.606104473	stable	DKFZP686E2158
0.606104473	stable	POLS
0.606117498	stable	PCNXL3
0.606175244	stable	VAC14
0.606229177	stable	RRAGC
0.606269596	stable	LOC728066
0.606312557	stable	FABP1
0.606312557	stable	PRSS23
0.606355416	stable	LOC651196
0.606355416	stable	LOC643236

0.606488499	stable	GYPB
0.606488499	stable	LRDD
0.606860378	stable	PNCK
0.606860378	stable	MARCH6
0.606860378	stable	SIM1
0.60697403	stable	KIF16B
0.60697403	stable	LOC642198
0.607121805	stable	REG1P
0.607154156	stable	ITCH
0.607154156	stable	LOC643120
0.607156471	stable	LOC643313
0.607161112	stable	TBC1D3E
0.607218027	stable	LOC646501
0.607521149	stable	RPS20
0.607581334	stable	LOC642772
0.607591634	stable	LOC650788
0.607597069	stable	TTC39A
0.607639295	stable	CYP2C19
0.607678597	stable	HOXD11
0.60780976	stable	TIGD5
0.60780976	stable	SFTPA2
0.607830807	stable	DCLRE1B
0.60789923	stable	PRKACA
0.60789923	stable	LOC642295
0.60789923	stable	LOC390547
0.608039546	stable	LOC648174
0.608039546	stable	LOC647295
0.608154346	stable	LOC439985
0.608219591	stable	TOPBP1
0.608219591	stable	CHST8
0.608248385	stable	FLJ35785
0.608269939	stable	CREB3L1
0.608270087	stable	OR5AY1
0.60836722	stable	LOC652043
0.60836722	stable	FLJ38773
0.60845555	stable	PYGO1
0.608566071	stable	LOC644056
0.60858062	stable	LOC644843
0.608641341	stable	SCN2A
0.608760864	stable	DGKH
0.608760864	stable	GPATCH8
0.608760864	stable	TSGA10
0.608761128	stable	HAPLN3
0.608834724	stable	LOC149643
0.608834724	stable	RNASE12
0.608847047	stable	OR5H1
0.608973832	stable	SLC15A1
0.608973832	stable	C1ORF131
0.609051147	stable	LOC441792
0.609130587	stable	LOC441907
0.60924947	stable	LOC653968

0.60924947	stable	LOC649770
0.60924947	stable	LATS2
0.609256805	stable	MKI67
0.609567414	stable	LOC644408
0.609647359	stable	LOC400927
0.609673519	stable	TBX5
0.609723189	stable	TAS2R3
0.609723189	stable	FAM130A1
0.609723189	stable	KIAA1984
0.610166405	stable	TAF4B
0.610166405	stable	LOC643630
0.610481346	stable	HIGD2BP
0.610481346	stable	PBK
0.610640442	stable	DEFB125
0.610640442	stable	LOC653252
0.610640442	stable	LOC728499
0.610814805	stable	LOC390570
0.610814805	stable	LOC642980
0.610814805	stable	TMC5
0.610904323	stable	TMEM123
0.611006294	stable	EVC2
0.611066709	stable	LOC648069
0.611066709	stable	GJD4
0.611191012	stable	FA2H
0.6113037	stable	LOC730995
0.61154006	stable	TMEM105
0.61154006	stable	PHLDB3
0.611684297	stable	C18ORF16
0.611740073	stable	LOC123876
0.611789079	stable	LOC253573
0.611789079	stable	KU-MEL-3
0.611789079	stable	LRRFIP1
0.611902684	stable	ALOXE3
0.611902684	stable	DKFZP686O1327
0.611973847	stable	GTF2F1
0.611973847	stable	CLDN8
0.612085206	stable	LOC651476
0.612085206	stable	LOC649396
0.612085206	stable	CA4
0.612124333	stable	FLJ30375
0.612124333	stable	LOC649025
0.612124333	stable	LOC401010
0.612124333	stable	GSTA1
0.612554225	stable	LOC643391
0.612554225	stable	LOC649749
0.612554225	stable	ANKK1
0.612554225	stable	RPS4X
0.612554225	stable	NOS1
0.61264316	stable	GPR88
0.612717556	stable	LOC652231
0.612745336	stable	LOC650146

0.612936253	stable	ABCC12
0.612941609	stable	OR9A4
0.612968078	stable	CDX4
0.612968078	stable	PIGL
0.612968078	stable	BAGE
0.612968078	stable	LOC646496
0.612968078	stable	SGOL2
0.613118395	stable	KIAA1166
0.613118395	stable	GBL
0.613118395	stable	EMR1
0.613194935	stable	KRTAP15-1
0.613194935	stable	ZNF709
0.613231217	stable	LOC645201
0.613237578	stable	VPREB1
0.613391387	stable	C1ORF65
0.613482477	stable	SNRPB2
0.613489505	stable	LOC650850
0.613646099	stable	SLC29A4
0.613646099	stable	LOC644422
0.613669545	stable	C17ORF82
0.613669545	stable	LOC731000
0.613732793	stable	DPF1
0.614024081	stable	OR5AS1
0.614059733	stable	NCOA4
0.614059971	stable	LOC650239
0.614059971	stable	C1GALT1C1
0.614111261	stable	DLX6
0.614121995	stable	IPO7
0.614205195	stable	GABRA5
0.614225453	stable	LRRC7
0.614225453	stable	DKFZP434I1020
0.614262363	stable	MAP3K4
0.61434377	stable	CCDC120
0.61434377	stable	LOC651936
0.614368878	stable	ZSCAN20
0.614368878	stable	DENND4C
0.614480299	stable	OR5B12
0.614498798	stable	LOC119358
0.614498798	stable	MDS1
0.614559253	stable	LOC388965
0.614559253	stable	LOC642439
0.614736645	stable	FIGF
0.614782822	stable	STC2
0.614782822	stable	LOC646226
0.614782822	stable	SLC30A8
0.614782822	stable	CCDC33
0.614858286	stable	ZNF705A
0.61488932	stable	TMTC3
0.614914942	stable	LOC647971
0.615156269	stable	LOC387761
0.615173193	stable	DNAJB13

0.615219685	stable	CRISP3
0.615219685	stable	LOC728127
0.615219685	stable	POU3F2
0.615219685	stable	C9ORF71
0.615232698	stable	SHQ1
0.615414556	stable	GPR15
0.615438736	stable	CYP26C1
0.615438736	stable	GRTP1
0.615495169	stable	LOC650268
0.615495169	stable	ZNF382
0.615495169	stable	SMYD3
0.615495169	stable	FLJ33360
0.615495169	stable	LOC650238
0.615495169	stable	INHBC
0.615859492	stable	MAGI2
0.615859492	stable	HMG4L
0.615922485	stable	PDZK1
0.615922485	stable	LOC653680
0.615922485	stable	LOC647131
0.615922485	stable	LOC650651
0.615922485	stable	LOC646037
0.616200701	stable	ZNF563
0.6162328	stable	FLJ45537
0.616297603	stable	LOC390856
0.616306297	stable	HIC1
0.616384556	stable	LOC644571
0.616467449	stable	PHF7
0.616467449	stable	HOXC10
0.616681825	stable	USP26
0.616681825	stable	EDAR
0.616681825	stable	LOC441869
0.616727868	stable	LOC652806
0.616727868	stable	LOC731035
0.616748426	stable	NOB1
0.616748426	stable	VEZF1
0.616748426	stable	UPK1A
0.616790986	stable	TMEM16D
0.616790986	stable	LOC728089
0.616883346	stable	TRNT1
0.616883346	stable	LOC647591
0.616883346	stable	PROKR1
0.617156163	stable	LOC730804
0.617192755	stable	TNNT2
0.617192755	stable	LOC649189
0.617192755	stable	DSG3
0.617221133	stable	MAGEB10
0.617221133	stable	LOC728269
0.617221133	stable	FLJ35934
0.617496178	stable	OR2T8
0.617598968	stable	LOC647832
0.617615586	stable	CDYL2

0.61765222	stable	C1ORF94
0.617660701	stable	TAC1
0.617663244	stable	UXS1
0.617697512	stable	LOC653894
0.617723773	stable	PDCD4
0.617729057	stable	IL33
0.617729057	stable	KRTHA5
0.617933993	stable	AKT3
0.617998094	stable	C4ORF11
0.618237936	stable	CD2
0.618237936	stable	PFDN2
0.618359704	stable	C20ORF141
0.618359704	stable	LOC652540
0.618423532	stable	GK5
0.618423532	stable	LOC647911
0.618492288	stable	TBX2
0.618573449	stable	C6ORF155
0.618718994	stable	HIST1H2AH
0.618718994	stable	LOC646029
0.618862354	stable	CDY1
0.619051518	stable	KRT34
0.619051518	stable	LOC645029
0.619051518	stable	LOC651291
0.619114741	stable	NUDT10
0.619287231	stable	UPK1B
0.619328817	stable	PTMS
0.619368715	stable	ATR
0.619423175	stable	GSTM2
0.619478011	stable	LOC647011
0.619485425	stable	WFDC13
0.619547465	stable	CYP2B7P1
0.619565605	stable	APOL5
0.619565605	stable	LOC400163
0.619584713	stable	CINP
0.619590749	stable	FAM178B
0.619590749	stable	LOC653412
0.619851263	stable	USP8
0.619919643	stable	LOC643467
0.619919643	stable	ACSS2
0.619919643	stable	C17ORF45
0.619919643	stable	LOC643210
0.619919643	stable	ASXL2
0.619919643	stable	BMP15
0.619919643	stable	MGC25181
0.619920022	stable	OR2F2
0.620042831	stable	NUDT16L1
0.620129203	stable	LOC648490
0.620223896	stable	LOC390282
0.620227795	stable	C3ORF41
0.620227795	stable	PABPC1L2B
0.620323422	stable	NUMB

0.620363826	stable	C9ORF25
0.620382111	stable	TXNRD2
0.620512339	stable	CRLF2
0.620512339	stable	LOC653047
0.620695643	stable	KSR1
0.62080672	stable	DICER1
0.62080672	stable	COX7C
0.62080672	stable	LOC648289
0.62080672	stable	LOC92154
0.620869698	stable	LOC441868
0.620869698	stable	LOC643791
0.620903109	stable	ANAPC1
0.620954234	stable	LOC440905
0.620954234	stable	FLJ46347
0.621129292	stable	LOC400174
0.621150985	stable	EXDL1
0.621212016	stable	WDR33
0.621629542	stable	ESF1
0.621744565	stable	LOC645781
0.621897007	stable	FNTA
0.621897007	stable	LOC389118
0.621944646	stable	FATE1
0.621944646	stable	FLJ38723
0.621944646	stable	LOC440786
0.621944646	stable	LOC643424
0.621944646	stable	PRPF8
0.62237466	stable	CCDC99
0.62237466	stable	LOC652682
0.622451989	stable	LOC440131
0.622492575	stable	C10ORF46
0.622579255	stable	C6ORF59
0.622588561	stable	LOC643598
0.622588561	stable	LOC653039
0.622588561	stable	MRPS15
0.622590827	stable	HIPK4
0.622590827	stable	LUZP2
0.622803989	stable	CSPG4LYP2
0.622932614	stable	SLC35B3
0.622942881	stable	ATG3
0.623039362	stable	C12ORF54
0.62307282	stable	SYT14
0.62307282	stable	TXN2
0.62307282	stable	RC3H1
0.62307282	stable	LOC647651
0.62307282	stable	LOC646278
0.62307282	stable	ZCCHC24
0.623078949	stable	LOC645084
0.62316491	stable	LOC651062
0.623203643	stable	LOC652224
0.623262545	stable	CTGLF3
0.623311001	stable	DUSP5P

0.623349114	stable	SPOCD1
0.623349114	stable	ELA3B
0.623386725	stable	LOC654128
0.623407317	stable	TCTN2
0.623418367	stable	IL4
0.623713636	stable	PHF13
0.623850319	stable	LOC653857
0.623850319	stable	LOC729777
0.623876133	stable	LOC646012
0.623914821	stable	LOC728763
0.623914821	stable	LOC646452
0.623914821	stable	SPG20
0.623914821	stable	DGCR14
0.623980474	stable	CCNT2
0.62406359	stable	RUFY1
0.624073696	stable	LOC731641
0.624073868	stable	ANKRD34A
0.624073868	stable	LOC652715
0.624073868	stable	SORCS1
0.624097373	stable	DEFB32
0.624097373	stable	LOC650556
0.624231973	stable	IER2
0.624432453	stable	LOC343927
0.624432453	stable	SULT1A3
0.624577301	stable	LOC645465
0.624620891	stable	FAM105B
0.624754184	stable	LOC648362
0.624819166	stable	LOC644800
0.624820244	stable	LOC646708
0.624921009	stable	BDKRB2
0.624974339	stable	C6ORF129
0.625054222	stable	LOC400682
0.625054222	stable	CTSL2
0.625162871	stable	KRTAP7-1
0.625514519	stable	NSUN7
0.625534648	stable	LOC642797
0.625551947	stable	SORD
0.625636165	stable	MSTO1
0.625690016	stable	MGC40069
0.625739347	stable	BBC3
0.625739347	stable	TNFRSF12A
0.625739347	stable	CSTL1
0.62580462	stable	GLTPD2
0.62580462	stable	PPP4R2
0.625840605	stable	LOC643216
0.625840605	stable	AQP12A
0.62596261	stable	LOC647704
0.626061362	stable	FAM127C
0.6263659	stable	LOC648489
0.626374708	stable	LOC729254
0.626374708	stable	TDO2

0.626655499	stable	LOC644774
0.626722125	stable	GALNT9
0.626722125	stable	PSMA8
0.626722125	stable	F13B
0.626722125	stable	LOC728760
0.626826718	stable	NLRP4
0.626830046	stable	IGFL1
0.626922083	stable	MARCKS
0.627102457	stable	LOC650580
0.627164569	stable	LOC343052
0.627214413	stable	FLJ12688
0.627440834	stable	LOC644781
0.627443604	stable	CDH12
0.627443604	stable	MRP63
0.627490928	stable	LY6H
0.627553947	stable	LOC644611
0.627641534	stable	PTRF
0.627641534	stable	LOC642504
0.627641534	stable	LOC646300
0.627641534	stable	TRR1
0.627729336	stable	PTGR1
0.627729336	stable	LOC644184
0.627729336	stable	LOC729003
0.627729336	stable	C10ORF129
0.627729336	stable	LOC649417
0.627793536	stable	HMX1
0.627935612	stable	LOC646566
0.627935612	stable	FLJ40244
0.627935612	stable	UPB1
0.627935612	stable	PVRIG
0.627935612	stable	PLA2G10
0.627936481	stable	CCDC150
0.627956682	stable	LOC440607
0.627966408	stable	KRTAP10-9
0.628106124	stable	FBF1
0.628300141	stable	LOC654155
0.628378462	stable	TBC1D29
0.628482051	stable	CDCP2
0.628572676	stable	LOC651746
0.628616511	stable	IFLTD1
0.62883127	stable	KIF18B
0.62883127	stable	C5ORF15
0.62883127	stable	CHCHD2
0.62883127	stable	C20ORF3
0.62883127	stable	LOC644999
0.628957652	stable	GSTM1
0.629060369	stable	CEP76
0.629100415	stable	OR1D2
0.629107183	stable	LOC642782
0.629107183	stable	PCDHGB3
0.629107183	stable	WDR75

0.629148636	stable	TYR
0.629210666	stable	C6ORF174
0.629210666	stable	LOC644844
0.629210666	stable	ARPM2
0.629343719	stable	TPRXL
0.629343719	stable	DNAJC12
0.629405365	stable	GDAP1L1
0.629465917	stable	LOC128322
0.629465917	stable	LOC642655
0.629465917	stable	EML5
0.629555415	stable	LOC391509
0.629555415	stable	SARS2
0.629990277	stable	FUT3
0.630183779	stable	COCH
0.630183779	stable	TRIB2
0.63033925	stable	LOC651401
0.63033925	stable	LOC440498
0.63033925	stable	WDR64
0.630387322	stable	TTC6
0.630421864	stable	GRID2
0.630429909	stable	LOC650036
0.630499361	stable	ZNF22
0.630969209	stable	OR52B6
0.630969209	stable	AHRR
0.630969209	stable	AIP
0.630969209	stable	AIPL1
0.630969209	stable	PANX3
0.630969209	stable	LOC654023
0.630969209	stable	LOC645574
0.630969209	stable	C17ORF68
0.630969209	stable	GP9
0.630969209	stable	FLJ13305
0.630969209	stable	BHMT
0.63107641	stable	CISH
0.631120263	stable	C1ORF55
0.631120263	stable	PLCD4
0.631120263	stable	CPT1A
0.631120263	stable	RP11-45B20.2
0.631426366	stable	TMEM133
0.631456248	stable	RGS13
0.631683879	stable	DRD5
0.631852093	stable	FAM122C
0.631892999	stable	LOC652441
0.631892999	stable	GABRG3
0.631892999	stable	LOC642588
0.631892999	stable	UNC13D
0.631892999	stable	OR2T5
0.631892999	stable	LOC642334
0.631923746	stable	CTRB2
0.63199817	stable	LOC643719
0.632098018	stable	TMC3

0.632098018	stable	PPM1M
0.632098018	stable	LOC644366
0.632148254	stable	USP35
0.632156511	stable	LOC648794
0.632547265	stable	KRTAP4-4
0.632623799	stable	TMEM34
0.632663852	stable	JSRP1
0.632666422	stable	TIFAB
0.632852563	stable	C21ORF74
0.63292555	stable	FLJ34870
0.63293799	stable	GSTTP1
0.632949587	stable	FAM113B
0.633088268	stable	MTMR8
0.633338697	stable	LOC441241
0.633338697	stable	KIAA0406
0.633338697	stable	LOC389791
0.633338697	stable	RLBP1L2
0.633338697	stable	C8ORF17
0.633476594	stable	C21ORF93
0.633549036	stable	ARL13B
0.633554199	stable	RPL32
0.633554199	stable	FLJ90680
0.633578276	stable	LOC643412
0.633717582	stable	FLJ27465
0.633914997	stable	DCST1
0.633914997	stable	LOC651974
0.63398167	stable	LOC653830
0.634021464	stable	LOC652222
0.634355735	stable	TMEM63C
0.634413668	stable	ACTA1
0.63442397	stable	ZNF395
0.634707645	stable	LOC651638
0.634720877	stable	OGFOD2
0.634929262	stable	LOC642456
0.635006203	stable	IQCF2
0.635016691	stable	C1ORF118
0.63505311	stable	LOC387720
0.635115678	stable	LOC652065
0.635410074	stable	LUC7L2
0.635410074	stable	LOC643935
0.635410074	stable	CD2AP
0.63546887	stable	UCN
0.63577054	stable	EFNB3
0.63577054	stable	PDE7B
0.63577054	stable	PCDHGA9
0.63577054	stable	GLTSCR2
0.63577054	stable	IFNA5
0.635905759	stable	LOC650976
0.635905759	stable	C12ORF48
0.635915636	stable	ZCCHC4
0.636096333	stable	JPH4

0.636135154	stable	LXN
0.636198767	stable	CD3G
0.63620721	stable	LOC653216
0.636247573	stable	LOC390332
0.636247573	stable	ALG11
0.636291248	stable	LOC284371
0.636291248	stable	CRIP1
0.636291248	stable	LOC389199
0.636291248	stable	COX6A2
0.63639657	stable	FRMPD2L1
0.636428453	stable	CBX1
0.636454681	stable	C3ORF25
0.636527406	stable	MEMO1
0.636583354	stable	LOC651123
0.636629906	stable	SLCO4C1
0.636629906	stable	MGC12760
0.636672556	stable	CHST11
0.636881635	stable	CDADC1
0.636944875	stable	DNAH3
0.636944875	stable	LOC645964
0.63697086	stable	WDR40C
0.63697086	stable	FCRL4
0.63704202	stable	HCRTR1
0.637099916	stable	OR2L1P
0.637099916	stable	DPRX
0.637213322	stable	LOC727815
0.637213322	stable	LOC641901
0.637339055	stable	XYLT2
0.63735852	stable	SPATA5
0.637420652	stable	EIF1AY
0.637737899	stable	SLC22A13
0.637840646	stable	NRL
0.637840646	stable	LOC646191
0.637842376	stable	LOC257358
0.637842376	stable	LOC647993
0.637842376	stable	PRKY
0.637843678	stable	OR6C70
0.637948791	stable	C2ORF62
0.637990119	stable	FAM3A
0.637990119	stable	LOC642351
0.638059351	stable	TBK1
0.638059351	stable	FLJ43692
0.638059351	stable	LOC652331
0.638070708	stable	TTBK1
0.638077177	stable	FLJ46363
0.638077177	stable	CUL3
0.638077177	stable	FAM3B
0.638108517	stable	PKN1
0.638140555	stable	IL17RE
0.638140555	stable	LOC651916
0.638292731	stable	PRDM15

0.638334741	stable	ST6GALNAC3
0.638613758	stable	LOC648245
0.638613758	stable	LOC651647
0.638613758	stable	LOC643035
0.638613758	stable	OR12D3
0.638613758	stable	OR4S1
0.638613758	stable	LOC642904
0.638613758	stable	LOC728145
0.638613758	stable	LOC643883
0.638613758	stable	ZNF300
0.638613758	stable	LOC651701
0.638613758	stable	CICK0721Q.1
0.638834517	stable	LOC651055
0.638957754	stable	ZNF530
0.638972655	stable	MIP
0.638972655	stable	OMP
0.638972655	stable	MVK
0.638972655	stable	LOC645974
0.639188233	stable	CACNG8
0.639270281	stable	C11ORF66
0.639273488	stable	HIST1H1D
0.639505923	stable	KIAA1545
0.639518289	stable	FPGS
0.639832728	stable	LOC652742
0.639832728	stable	TIMM17A
0.639832728	stable	CCNB1
0.639837823	stable	LOC400388
0.639943293	stable	FLJ42842
0.639955609	stable	RPP38
0.64000323	stable	LOC650659
0.64000323	stable	VHLL
0.64000323	stable	HIST1H3G
0.64000323	stable	LOC642864
0.64000876	stable	SYCP3
0.640047733	stable	WDR49
0.640321722	stable	SEMA6A
0.640345543	stable	TALDO1
0.640345543	stable	LOC653773
0.640347374	stable	LOC653715
0.640609995	stable	LOC644638
0.640609995	stable	MED13L
0.640628991	stable	LOC647844
0.640849064	stable	CCDC57
0.640849064	stable	LOC642134
0.640849064	stable	P2RX3
0.641016589	stable	LOC652437
0.641021404	stable	ABCA6
0.641055747	stable	LHFPL5
0.641055747	stable	GBP3
0.641055747	stable	ADAMTS10
0.641055747	stable	CLIC5

0.641055747	stable	ZBTB1
0.641055747	stable	LRP2BP
0.641055747	stable	C9ORF62
0.641055747	stable	NCR1
0.641099451	stable	CCNA2
0.641214915	stable	SIRT2
0.641282541	stable	MMRN2
0.641346073	stable	LOC641834
0.641404531	stable	WFS1
0.641404531	stable	LOC391004
0.641404531	stable	LOC652508
0.641404531	stable	CSAG2
0.641404531	stable	LOC643958
0.641404531	stable	SYT9
0.641404531	stable	SLC16A11
0.641404531	stable	LOC651952
0.641404531	stable	LOC732107
0.641464522	stable	LOC649635
0.641466063	stable	CERK
0.641542005	stable	LOC644295
0.641542005	stable	LOC348021
0.641544317	stable	FABP5L7
0.64157277	stable	TMEM61
0.641674893	stable	TREH
0.641674893	stable	TRIM40
0.641674893	stable	LOC643325
0.64169611	stable	ENTPD8
0.64177872	stable	HBG2
0.641918492	stable	LENG8
0.641978775	stable	FLJ32682
0.641978775	stable	NKX2-6
0.641978775	stable	USP36
0.641978775	stable	LOC646381
0.641978775	stable	ANKS1A
0.641986597	stable	LOC729487
0.641986597	stable	LOC647275
0.641995966	stable	PRPF38B
0.641995966	stable	RBM42
0.641995966	stable	C20ORF114
0.641995966	stable	LOC644404
0.641995966	stable	TET2
0.642046342	stable	ATXN7
0.642151257	stable	ACSM4
0.642151257	stable	LOC644912
0.642151257	stable	LOC391859
0.642181691	stable	NUBP2
0.642243099	stable	LOC645721
0.642243099	stable	UBASH3A
0.642243099	stable	LOC652879
0.642309676	stable	LOC642105
0.642353642	stable	KCTD4

0.642353642	stable	C10ORF61
0.642353642	stable	LOC650761
0.642353642	stable	LOC391037
0.642353642	stable	LOC728534
0.642672757	stable	LOC653305
0.642721077	stable	LOC643936
0.642739232	stable	LOC652533
0.642739232	stable	TSPAN2
0.64286742	stable	LOC653683
0.642908957	stable	LOC650116
0.643103511	stable	GDPD4
0.643103511	stable	LOC645451
0.643103511	stable	ST3GAL5
0.643313128	stable	LAMC1
0.643500102	stable	RDH14
0.643597004	stable	HDAC7A
0.643602193	stable	LOC648274
0.643629168	stable	LOC440268
0.643629168	stable	THSD7A
0.643774712	stable	SH2D2A
0.643846044	stable	LOC401914
0.643846044	stable	PMP22CD
0.643846044	stable	RPS6KA3
0.643846044	stable	SLA2
0.643846044	stable	LOC729919
0.643873002	stable	LOC649896
0.643886724	stable	RBM43
0.644016173	stable	LOC643748
0.644016173	stable	FLJ44477
0.644034646	stable	C6ORF52
0.644040567	stable	LOC649578
0.644040567	stable	ZNF567
0.644040567	stable	LOC652628
0.644073332	stable	SLITRK1
0.644073332	stable	C9ORF36
0.644201372	stable	LOC646537
0.644272164	stable	DULLARD
0.64427591	stable	DDI2
0.644431163	stable	LOC643055
0.644447027	stable	RD3
0.644626063	stable	RSRC1
0.644674575	stable	HMX2
0.644843388	stable	FYTTD1
0.645007012	stable	LOC730340
0.645007012	stable	LDHAL6A
0.645007012	stable	EPB41L4A
0.645007012	stable	LOC652792
0.645007012	stable	LOC642681
0.645007012	stable	FFAR3
0.645007012	stable	OR4C12
0.645007012	stable	LOC730833

0.645007012	stable	LOC647666
0.645022388	stable	OCM
0.645022388	stable	DPYSL2
0.64518888	stable	EVPL
0.645485466	stable	LOC649397
0.645966108	stable	LOC400221
0.645970433	stable	GPR39
0.645973637	stable	LOC651896
0.646138549	stable	GGT7
0.64621017	stable	C9ORF110
0.64621017	stable	NUF2
0.64621017	stable	LOC644690
0.64621017	stable	LOC392467
0.64621017	stable	LOC644607
0.64621017	stable	LOC642340
0.646445804	stable	LOC649443
0.646455846	stable	FAM26A
0.646469607	stable	D21S2056E
0.646469607	stable	C1ORF111
0.646515523	stable	ZNF780A
0.646515523	stable	FLJ44815
0.646649841	stable	C20ORF123
0.646716494	stable	TNFSF18
0.646748676	stable	LOC440013
0.646752356	stable	LOC646604
0.646860451	stable	LOC729355
0.646914962	stable	AFF2
0.646914962	stable	MS4A10
0.646914962	stable	LOC651760
0.647048602	stable	FOXR1
0.647048602	stable	TMEM127
0.647069165	stable	LOC652448
0.647111377	stable	ZNF44
0.647117712	stable	CCDC38
0.647129321	stable	LOC401923
0.647228387	stable	LOC650459
0.64725027	stable	ACRV1
0.647315766	stable	LOC653582
0.647364983	stable	ZNF286A
0.64743893	stable	FOXF1
0.647726985	stable	LOC150577
0.648167852	stable	KRT4
0.648597764	stable	FLJ34047
0.648726414	stable	LOC731283
0.648829677	stable	MAGOHB
0.648829677	stable	LOC653380
0.648829677	stable	ESRRG
0.648829677	stable	ARHGAP27
0.648829677	stable	C20ORF118
0.648829677	stable	LOC648438
0.648829677	stable	IL28RA

0.648835734	stable	VSTM2
0.648857026	stable	GTF2A1L
0.648910195	stable	TRAF1
0.648950603	stable	UBE2NL
0.648950603	stable	RPH3AL
0.648950603	stable	SAMD4B
0.64931168	stable	LOC650180
0.649532291	stable	LOC643203
0.649532291	stable	PTH
0.649638054	stable	VSX1
0.649638054	stable	LOC645306
0.649638054	stable	MAGEB18
0.649813978	stable	PIP5KL1
0.649813978	stable	SLC26A7
0.649823467	stable	LOC649073
0.649823467	stable	GPRC5B
0.650033007	stable	KIAA2026
0.650034816	stable	GNG2
0.650056488	stable	KRT20
0.650162232	stable	DCI
0.650162232	stable	MEGF10
0.650162232	stable	C15ORF51
0.650162232	stable	C12ORF67
0.650162232	stable	FLJ45964
0.650162232	stable	DIAPH2
0.650162232	stable	LSM6
0.65029276	stable	LOC647156
0.650376048	stable	LOC643563
0.650376048	stable	PCNXL2
0.650376048	stable	LOC651293
0.650376048	stable	LOC648855
0.650408948	stable	LOC653750
0.650408948	stable	DNM1
0.650462996	stable	MDFI
0.650557683	stable	LOC727735
0.650557683	stable	CDH10
0.650557683	stable	OR52A5
0.650842044	stable	UBE2R2
0.650909166	stable	LOC646864
0.650909166	stable	LARS
0.650909166	stable	GALNAC4S-6ST
0.650995018	stable	LOC641785
0.651090663	stable	ARL8A
0.651104846	stable	LOC646011
0.651104846	stable	BRAF
0.651104846	stable	LOC643261
0.651217194	stable	LOC220416
0.651343427	stable	MPZL3
0.651343427	stable	FCHSD2
0.651350862	stable	MMP17
0.651372337	stable	HTR4

0.651415665	stable	LOC647726
0.651628758	stable	AKAP10
0.651696185	stable	LOC647480
0.651696185	stable	LOC650468
0.651848232	stable	LOC648732
0.651862152	stable	LOC649856
0.651862152	stable	LOC650922
0.651862152	stable	FGF8
0.651867713	stable	DNAHL1
0.651867713	stable	IL31RA
0.651960664	stable	SLC39A12
0.651960664	stable	TIFA
0.651960664	stable	NLGN4X
0.652102223	stable	NAF1
0.652317119	stable	PYGO2
0.652471653	stable	RSL1D1
0.652557687	stable	SERPINB13
0.652648831	stable	LOC642014
0.652696965	stable	LOC169834
0.652696965	stable	KCNK18
0.652699039	stable	OR5F1
0.652699039	stable	SLC18A2
0.652728404	stable	LOC729973
0.652728404	stable	LOC648556
0.652751881	stable	C8ORF30A
0.652751881	stable	LOC650200
0.652826348	stable	GRAP2
0.652955874	stable	PKHD1L1
0.65304749	stable	KLK14
0.65304749	stable	LOC643194
0.653091902	stable	MUC6
0.653146748	stable	ANKRD20B
0.65333624	stable	LOC390483
0.653400471	stable	LOC642366
0.653400471	stable	LOC389031
0.653419857	stable	ZBTB8
0.653534449	stable	LOC654096
0.653704763	stable	PTPN9
0.65371819	stable	LOC643441
0.653761314	stable	LOC643142
0.653761314	stable	CDK5R2
0.653761314	stable	JARID1A
0.653761314	stable	EXOSC8
0.653761314	stable	TTTY13
0.653786094	stable	LOC652191
0.653786094	stable	LOC651576
0.653786094	stable	LOC651959
0.653797646	stable	PRSS12
0.653854414	stable	PRR17
0.653889549	stable	LOC644105
0.653944813	stable	CDC2L5

0.653944813	stable	LOC646552
0.653944813	stable	NDRG4
0.654076227	stable	FNDC3A
0.65412205	stable	LOC650029
0.65412205	stable	P2RX6P
0.654221756	stable	LOC642033
0.654221756	stable	GLP1R
0.654319825	stable	IL1F9
0.654319825	stable	HOXA11
0.654339405	stable	LOC653234
0.654373544	stable	AGR2
0.654401784	stable	ADAD1
0.654401784	stable	DMGDH
0.654401784	stable	C14ORF1
0.654565515	stable	LOC650285
0.654565515	stable	LRRC8E
0.654588753	stable	ZNF663
0.654630286	stable	LOC340549
0.654630286	stable	CLDN3
0.65467022	stable	ADCY5
0.65467022	stable	C10ORF72
0.654702558	stable	LOC338328
0.654782658	stable	FAM22F
0.654891971	stable	CRIM2
0.654913559	stable	MOBKL1A
0.654913559	stable	WFDC5
0.655110839	stable	SPATA17
0.655212135	stable	PCDHGA3
0.655212135	stable	LOC389124
0.655212135	stable	SEC23A
0.655212135	stable	C19ORF57
0.655212135	stable	IL2RB
0.655246431	stable	KIAA0738
0.655246431	stable	LOC653261
0.655404637	stable	OR4N5
0.655404637	stable	LOC645671
0.655404637	stable	LOC642361
0.655404637	stable	ONECUT3
0.655455646	stable	DKFZP779B1634
0.655481378	stable	CXORF61
0.655481378	stable	CEP170L
0.655767244	stable	SLC26A2
0.65592623	stable	LOC654085
0.656047899	stable	SH3MD4
0.656047899	stable	DMRTB1
0.65620424	stable	GP2
0.65620424	stable	SNTG2
0.656399033	stable	TRIP10
0.656407967	stable	LOC653051
0.656407967	stable	LOC648189
0.656407967	stable	FLJ42418

0.656508491	stable	ADAM29
0.656602002	stable	LOC728734
0.656806363	stable	DMBT1
0.656806363	stable	INOC1
0.656806363	stable	C10ORF31
0.65682567	stable	LIPE
0.656859757	stable	LOC652843
0.656945711	stable	SYNE1
0.656945711	stable	PDC
0.656945711	stable	LOC643275
0.656945711	stable	LOC642120
0.65697094	stable	PCDHB14
0.65697094	stable	LOC652226
0.657190292	stable	SSSCA1
0.657387047	stable	CHTF18
0.657568762	stable	KLHL15
0.657569243	stable	TPRG1
0.657660154	stable	LOC645088
0.657660154	stable	TMPO
0.657691942	stable	C2ORF78
0.657724773	stable	LOC654208
0.657724773	stable	MED26
0.657989434	stable	LOC649486
0.658165271	stable	ING1
0.658165271	stable	WDTC1
0.658165271	stable	IGF2
0.658287007	stable	MAPK11
0.658447876	stable	PIH1D2
0.658629996	stable	LOC644133
0.658672276	stable	C6ORF107
0.658699208	stable	LOC342979
0.658711646	stable	NOM1
0.658720417	stable	LOC651621
0.658720417	stable	LOC653937
0.658758206	stable	LEFTY1
0.658888074	stable	LOC727944
0.658942999	stable	LAGE3
0.659229016	stable	LOC645789
0.659357762	stable	LOC641767
0.659522469	stable	FREM2
0.660034427	stable	TNXB
0.660104652	stable	ZNF622
0.660104652	stable	LOC643357
0.660391	stable	LOC399753
0.660434409	stable	LOC651427
0.660450965	stable	LOC440157
0.660502547	stable	LOC648343
0.660519618	stable	ELAVL1
0.660519618	stable	LOC649125
0.660519618	stable	LOC642140
0.660750758	stable	PPP2R5E

0.660803583	stable	LOC642119
0.660803583	stable	SLC12A3
0.660803583	stable	LOC645745
0.660803583	stable	DPY19L2P2
0.660803583	stable	SP1
0.660803583	stable	CEP164
0.660863921	stable	DUX1
0.661229696	stable	LOC647407
0.661336089	stable	CANX
0.661336089	stable	SIRT6
0.661409527	stable	TFEC
0.661409527	stable	SUNC1
0.661454052	stable	PCDHB6
0.661515922	stable	GPR77
0.661605547	stable	LOC648897
0.661654224	stable	LOC642849
0.661749492	stable	LOC344875
0.661749492	stable	CENTD3
0.661749492	stable	DLEC1
0.661749492	stable	FLJ20581
0.661749492	stable	AGC1
0.66204358	stable	FAM47C
0.662233787	stable	LOC389120
0.66225298	stable	USH1C
0.66225298	stable	POU3F4
0.662443485	stable	LOC286094
0.662443485	stable	C13ORF35
0.662443485	stable	FOXN3
0.662531339	stable	ZNF829
0.662536683	stable	C5ORF21
0.662640734	stable	GAGE5
0.662717429	stable	LRRN2
0.662721889	stable	LOC641522
0.662784166	stable	ERC2
0.662850843	stable	SNX2
0.662850843	stable	LOC644524
0.663271861	stable	GEMIN7
0.663400008	stable	LOC648659
0.663400008	stable	SEPT14
0.663668901	stable	OPN5
0.663668901	stable	GCM2
0.663668901	stable	GPR179
0.663668901	stable	SWAP70
0.663728113	stable	SPINK8
0.663782903	stable	CDC25A
0.663782903	stable	LOC648751
0.663800716	stable	LOC400578
0.663958848	stable	SPATA16
0.664430081	stable	C1ORF201
0.664430081	stable	LOC643397
0.664430081	stable	LOC387683

0.664430081	stable	SNORD30
0.664430081	stable	TRHDE
0.664430081	stable	SSX2
0.664430081	stable	TXNDC8
0.66451481	stable	WDR52
0.664632976	stable	OR8H1
0.664706234	stable	LOC285735
0.664706234	stable	C19ORF29
0.664706234	stable	LOC440407
0.664737658	stable	LOC647742
0.664737658	stable	LOC649965
0.664737658	stable	HSD3B2
0.664737658	stable	LOC652324
0.664737658	stable	MLLT3
0.664737658	stable	LOC645287
0.664737658	stable	LOC644944
0.665102367	stable	LOC642740
0.665102367	stable	LOC645682
0.66519246	stable	C1ORF67
0.665319862	stable	KIAA0146
0.665319862	stable	HTN3
0.665319862	stable	MARCH4
0.665338417	stable	PTER
0.665378772	stable	EGR3
0.665437168	stable	CHCHD9
0.665515962	stable	LOC644224
0.665758789	stable	LOC647891
0.665842523	stable	MAP9
0.665873827	stable	CTAG2
0.665873827	stable	RAVER2
0.665873827	stable	ARL9
0.665939399	stable	KIAA0174
0.665963001	stable	TACSTD1
0.66603201	stable	KRTAP11-1
0.66603201	stable	RNF138P1
0.666066783	stable	OR11G2
0.666066783	stable	FAM20B
0.666066783	stable	LOC648481
0.666066783	stable	STK31
0.666066783	stable	KLHDC7B
0.666066783	stable	FLJ90036
0.666066783	stable	CACNA1F
0.666341026	stable	LOC648054
0.666358895	stable	DKFZP564C196
0.666537762	stable	LOC652139
0.666713063	stable	IRGC
0.66677288	stable	APOBEC4
0.666782858	stable	LOC729260
0.666782858	stable	ID4
0.666782858	stable	BOLL
0.66682241	stable	LOC729476

0.666825757	stable	FLJ37673
0.66682973	stable	LOC255187
0.666879335	stable	LOC120824
0.666908781	stable	SLC39A2
0.666992739	stable	FLJ36116
0.666992739	stable	TRPM3
0.667311135	stable	TOP1P1
0.667398256	stable	LOC649452
0.667398256	stable	ABI1
0.667497723	stable	LOC651454
0.667747821	stable	LOC644689
0.667789268	stable	PEX14
0.667809914	stable	LOC648668
0.667810635	stable	SGK3
0.66791738	stable	LOC650638
0.667991324	stable	PZP
0.668079767	stable	AHCYL2
0.66814134	stable	LOC651886
0.668155958	stable	LOC441378
0.668155958	stable	LOC653219
0.668155958	stable	ZIC4
0.668155958	stable	LOC652679
0.668155958	stable	LOC731496
0.668155958	stable	OR10G3
0.668155958	stable	LOC645995
0.668155958	stable	C3ORF46
0.668327751	stable	NLRP10
0.668425163	stable	ZNF509
0.66843395	stable	C14ORF178
0.66843395	stable	PDF
0.66843395	stable	MGC10997
0.66848202	stable	LOC284441
0.66848202	stable	GUCA2B
0.66848202	stable	LOC650823
0.66860157	stable	DSP
0.66860758	stable	LOC644592
0.668682382	stable	MTDH
0.668754959	stable	LOC440082
0.668866026	stable	CTAG1B
0.668947125	stable	CADM2
0.669001692	stable	RCOR1
0.669017771	stable	WFDC10B
0.669017771	stable	LARP2
0.669068834	stable	IGHMBP2
0.669147552	stable	ZPBP
0.669280505	stable	LOC647509
0.669280505	stable	PRMT8
0.669334893	stable	MCART6
0.669431019	stable	UTP3
0.669431019	stable	LOC347411
0.669433772	stable	LOC653555

0.669670528	stable	LOC653562
0.669670528	stable	RNF12
0.669670528	stable	LOC402715
0.669670528	stable	ZNF304
0.669781594	stable	ZNF470
0.669781594	stable	LOC149950
0.669857055	stable	DPRXP4
0.669857055	stable	EIF2S1
0.670035589	stable	ZNF75D
0.670065739	stable	WBSCR19
0.670065739	stable	PIP4K2C
0.670221359	stable	CCDC58
0.670489401	stable	LOC646829
0.670489401	stable	CHRNB1
0.670489401	stable	LOC645662
0.670877004	stable	KMO
0.670926727	stable	KLK2
0.670926727	stable	C1ORF99
0.670926727	stable	LOC642109
0.671052519	stable	CUL2
0.671052519	stable	LOC653514
0.671072186	stable	C9ORF109
0.671369053	stable	DKFZP434P211
0.671420796	stable	MGC35440
0.671802855	stable	USP43
0.671802855	stable	EPS8L3
0.671814792	stable	LOC650323
0.671814792	stable	C9ORF135
0.671814792	stable	ZNF70
0.671915977	stable	GNRHR
0.671955784	stable	ZCCHC10
0.671955784	stable	LOC643429
0.671955784	stable	LOC648629
0.672018701	stable	LATS1
0.672058173	stable	LOC653746
0.672058173	stable	FFAR1
0.672087696	stable	RAB9P1
0.672194407	stable	MAP3K10
0.672244141	stable	TRPM2
0.67231671	stable	CHKA
0.67256781	stable	LOC649891
0.672634986	stable	LOC400759
0.672634986	stable	CLEC4M
0.672634986	stable	GREM2
0.672706876	stable	C17ORF78
0.672706876	stable	LOC649280
0.672706876	stable	LOC643920
0.672706876	stable	TAS2R7
0.672706876	stable	LOC441050
0.672706876	stable	OR2C1
0.672706876	stable	LIG4

0.672706876	stable	LOC644499
0.672706876	stable	LOC644748
0.672761455	stable	LOC645165
0.672812913	stable	FLJ40288
0.672812913	stable	OR8U8
0.672894767	stable	FLJ20464
0.672989852	stable	BRD1
0.672994927	stable	GNAT3
0.673174466	stable	PNPLA5
0.673236431	stable	GRM6
0.673236431	stable	LOC652798
0.673236431	stable	NDUFA3
0.673296318	stable	LOC390387
0.673296318	stable	DLGAP3
0.673595979	stable	KLF5
0.673778628	stable	FOXN1
0.673778628	stable	LOC645655
0.673778628	stable	SLAMF7
0.673945895	stable	LOC653297
0.673945895	stable	LOC728012
0.673945895	stable	SLC7A9
0.673945895	stable	WSCD1
0.673945895	stable	LPP
0.674023334	stable	LOC284428
0.674103754	stable	LOC441362
0.674174212	stable	LOC400301
0.6742201	stable	LAMP2
0.674319341	stable	LOC642426
0.674330287	stable	MACROD1
0.674546259	stable	HIST1H2AJ
0.674546259	stable	JRK
0.674897401	stable	PSCD2
0.674897401	stable	SFRS3
0.674897401	stable	TBRG1
0.674897401	stable	LOC649416
0.674897401	stable	TCP11
0.674897401	stable	PDPK1
0.674897401	stable	CUL7
0.674897401	stable	PCDHAC2
0.674897401	stable	OR9Q1
0.674897401	stable	TPPP2
0.674897401	stable	KIAA1210
0.674897401	stable	OR13C5
0.675003633	stable	SPINT2
0.675036062	stable	FLJ40296
0.675064346	stable	PGPEP1
0.675461274	stable	PCID2
0.675562269	stable	LOC402571
0.675562269	stable	LOC647163
0.67567417	stable	ALG12
0.675724507	stable	SAMD6

0.675724507	stable	EAF1
0.675724507	stable	LOC651635
0.675724507	stable	LOC651186
0.675724507	stable	FAM83H
0.675780425	stable	HORMAD1
0.675942232	stable	PPIE
0.675942232	stable	LOC644504
0.676072154	stable	C11ORF61
0.676072154	stable	ZFP14
0.676091045	stable	KCND1
0.676231711	stable	ARHGAP20
0.676234876	stable	IMAA
0.676300435	stable	TREML4
0.676488548	stable	GDI1
0.676535723	stable	UBR7
0.676535952	stable	RPS3A
0.676603889	stable	MEF2B
0.67668314	stable	CLINT1
0.67668314	stable	LOC648394
0.67668314	stable	RIG
0.676792167	stable	EXOC3L2
0.676792167	stable	INTS3
0.676916879	stable	LOC647869
0.676916879	stable	LOC653105
0.6769263	stable	LOC646448
0.6769263	stable	LOC649768
0.676942529	stable	LOC283551
0.676980032	stable	LOC389316
0.677021805	stable	LOC441376
0.677052352	stable	LOC731159
0.677443392	stable	LOC645444
0.677550382	stable	WDR38
0.677789557	stable	ZBED2
0.677818331	stable	LOC643461
0.677874696	stable	ZNF692
0.678066407	stable	LOC652082
0.67849329	stable	MYO1A
0.678513212	stable	PRSSL1
0.67854658	stable	RDH11
0.67854658	stable	LOC649374
0.67854658	stable	LOC645635
0.67854658	stable	LOC653515
0.678636277	stable	NTNG1
0.678636277	stable	LOC645755
0.678701414	stable	PGK2
0.678721286	stable	LOC652801
0.678896448	stable	ADAM19
0.679017953	stable	LOC342346
0.679017953	stable	SERPINB9
0.679017953	stable	RASEF
0.679036089	stable	NPC1L1

0.679036089	stable	FGF20
0.679050789	stable	LOC644993
0.679050789	stable	LOC730713
0.679059929	stable	HOXB13
0.679243358	stable	LOC648735
0.679290967	stable	LOC730704
0.679290967	stable	SLC10A5
0.679327122	stable	VPS4A
0.679327122	stable	OVGP1
0.679327122	stable	LOC375323
0.679493587	stable	ALDH3A1
0.679719859	stable	LOC653257
0.679815117	stable	LOC642480
0.679824414	stable	LOC346950
0.679824414	stable	LOC650253
0.679824414	stable	LOC647323
0.679824414	stable	LOC653492
0.679824414	stable	DEFB110
0.679824414	stable	NUP214
0.679824414	stable	LOC648998
0.67983688	stable	HELT
0.680063429	stable	TTC36
0.680063429	stable	OR2T4
0.680073481	stable	LAMA1
0.680154957	stable	LOC652067
0.680296055	stable	C16ORF71
0.6804163	stable	LOC642736
0.6804163	stable	ELA3A
0.6804163	stable	NUDT19
0.680510285	stable	ATXN7L2
0.680524821	stable	LOC440944
0.680586725	stable	HNRPA2B1
0.680692066	stable	LOC652495
0.680696956	stable	NLRP13
0.680719019	stable	MGC15634
0.680719019	stable	SERPINB12
0.680719019	stable	ADAMTS6
0.680813055	stable	LOC642093
0.680824572	stable	CRYGC
0.680886786	stable	WDR90
0.680962503	stable	LOC392559
0.680962503	stable	LOC649886
0.680962503	stable	FLJ16165
0.681046234	stable	LOC642216
0.681046234	stable	LOC649383
0.681049374	stable	MYH1
0.681049374	stable	ZNF238
0.681049374	stable	LOC730344
0.681293186	stable	KRTAP8-1
0.681559041	stable	TMPRSS4
0.681782881	stable	TXNDC11

0.681804596	stable	LOC441344
0.681804596	stable	LOC646365
0.681815035	stable	OR8I2
0.681815035	stable	NUBPL
0.681815035	stable	LOC653314
0.681815035	stable	LOC647741
0.681994379	stable	FBXO17
0.682117551	stable	FLJ43870
0.682126111	stable	FAM76A
0.682128918	stable	PRAMEF17
0.682141017	stable	PON3
0.682311959	stable	CART1
0.682316859	stable	ZFP95
0.682345113	stable	LOC150786
0.682345113	stable	LOC642514
0.682345113	stable	TECTB
0.682345113	stable	FAM89B
0.682345113	stable	C2ORF61
0.682345113	stable	KIAA0492
0.682345113	stable	DNHD1
0.682345113	stable	LOC645818
0.682345113	stable	LOC654203
0.682484109	stable	C5ORF32
0.682484109	stable	FLJ33651
0.682501333	stable	KIR2DS5
0.6825349	stable	LOC646458
0.682568661	stable	LOC642450
0.682702122	stable	HGF
0.682873927	stable	C4A
0.682873927	stable	SPAG5
0.682873927	stable	SGCD
0.682950247	stable	VPS54
0.682950247	stable	TARSL2
0.683101623	stable	LOC253820
0.683149917	stable	SERPINB11
0.683171139	stable	CPNE2
0.683376943	stable	PLEK2
0.683437999	stable	PLA2G12B
0.68356538	stable	C2ORF53
0.68358866	stable	SPATA6
0.683796552	stable	MYF5
0.683803734	stable	ARPP-21
0.683806534	stable	ABCD1
0.683817606	stable	LOC649041
0.684012721	stable	ZNF766
0.684082993	stable	SFI1
0.684095064	stable	HLA-DQA2
0.684283986	stable	LOC644171
0.684328211	stable	LZTS2
0.684353172	stable	C17ORF57
0.684353172	stable	LOC338579

0.684462445	stable	LOC643283
0.684618727	stable	LOC286016
0.684828839	stable	NEU3
0.684925113	stable	SH3BGRL2
0.685051065	stable	LOC644898
0.685061087	stable	ASNSD1
0.685096925	stable	CMTM2
0.685330232	stable	C7ORF29
0.68536137	stable	ABCA7
0.685361815	stable	STIM2
0.685619363	stable	FAM120C
0.68568299	stable	MAP3K9
0.68568299	stable	PLCB1
0.68568299	stable	CST1
0.685820755	stable	LOC652071
0.686103679	stable	LOC400708
0.686304054	stable	ZCCHC2
0.686354489	stable	FGFR2
0.68645029	stable	MGC34761
0.686557305	stable	LOC646183
0.686699889	stable	WIPF3
0.686699889	stable	LOC648447
0.686699889	stable	LOC649754
0.686699889	stable	LOC642280
0.686699889	stable	LOC649296
0.686829642	stable	DYDC1
0.686925943	stable	MAGEB3
0.686925943	stable	ASB17
0.686937796	stable	OR5M8
0.68694007	stable	LOC644429
0.68694007	stable	MGAM
0.68694007	stable	LOC652433
0.68694007	stable	LOC730920
0.68694007	stable	OR5M9
0.68694007	stable	KIF24
0.686962327	stable	MYBPC3
0.68711322	stable	FLJ42102
0.68711322	stable	PSORS1C2
0.687152018	stable	LOC646869
0.687152018	stable	LOC391475
0.687152018	stable	FLJ42289
0.687357333	stable	PIB5PA
0.687411887	stable	HS3ST5
0.687465491	stable	LOC651729
0.687506437	stable	RXRG
0.687506437	stable	LOC643363
0.687570567	stable	WHDC1
0.687570567	stable	LOC649702
0.68761481	stable	LOC652698
0.68761481	stable	BLR1
0.687703158	stable	LOC133609

0.68777383	stable	LOC441931
0.687935901	stable	CNTFR
0.688046909	stable	DEFB126
0.688318096	stable	KRTAP6-3
0.688318096	stable	LOC642219
0.688318096	stable	LOC642962
0.688369198	stable	LOC642133
0.688426902	stable	DNAJB7
0.688430187	stable	LOC646280
0.688430187	stable	INDOL1
0.688430187	stable	PKDREJ
0.688430187	stable	DDX53
0.688430187	stable	KCNE3
0.688532173	stable	LOC652548
0.68857715	stable	LOC127011
0.688652302	stable	LOC650861
0.688672079	stable	C1ORF108
0.688686223	stable	SMNDC1
0.688710149	stable	RBM6
0.688810611	stable	LOC653381
0.68884537	stable	LOC644681
0.68884537	stable	ADORA1
0.688924638	stable	LOC653752
0.689007701	stable	LOC645929
0.689249956	stable	LOC441812
0.689274191	stable	GPR153
0.689323699	stable	LOC648972
0.689323699	stable	CAPZA3
0.689323699	stable	IVL
0.689362462	stable	LOC339809
0.689386097	stable	POU3F3
0.689386097	stable	SUMO4
0.689386097	stable	CLPB
0.689419438	stable	SAA4
0.68949663	stable	C9ORF53
0.68949663	stable	NMS
0.689613956	stable	TMCO5A
0.689613956	stable	LOC649360
0.689626936	stable	LOC728334
0.689693607	stable	CRYBA2
0.689744676	stable	FLJ90231
0.689744676	stable	LOC652353
0.689755645	stable	LOC648704
0.689779638	stable	GDF5
0.689877854	stable	LOC391817
0.689877998	stable	LOC652905
0.689877998	stable	LOC730034
0.689877998	stable	CCDC11
0.689877998	stable	SLC25A21
0.689877998	stable	KRT39
0.689877998	stable	GHRHR

0.690061027	stable	LOC647681
0.690367765	stable	LOC646498
0.690430757	stable	GNAS
0.690434302	stable	CYP7A1
0.690449473	stable	ASPRV1
0.690609561	stable	TMEM5
0.690735144	stable	FLJ25758
0.690856814	stable	GLYBP
0.690856814	stable	LOC648657
0.690928555	stable	ZC3H5
0.691194085	stable	LOC646990
0.691237063	stable	PYCRL
0.691237063	stable	C2ORF54
0.691280858	stable	LOC649174
0.691373329	stable	HUS1
0.691373329	stable	LOC730809
0.691397055	stable	FLJ33387
0.691548104	stable	ALAD
0.691548104	stable	LOC647003
0.691548104	stable	LOC650373
0.691548104	stable	LOC400768
0.691668279	stable	BCAS1
0.691749506	stable	ABCA9
0.691749506	stable	LOC646363
0.691749506	stable	LOC652213
0.691749506	stable	RAG1
0.691749506	stable	ADRBK1
0.691749506	stable	LOC649853
0.691875884	stable	FAM105A
0.691931209	stable	C6ORF26
0.691931209	stable	PHACTR2
0.691931209	stable	LOC730132
0.692164066	stable	CHPF
0.692371133	stable	FLJ14816
0.692371133	stable	LOC649580
0.69253505	stable	LOC647197
0.692663288	stable	SCN8A
0.692681872	stable	LOC653118
0.692681872	stable	FBXW12
0.692720196	stable	UBE3A
0.692720196	stable	LOC652608
0.692720196	stable	LOC646173
0.692720196	stable	EREG
0.692720196	stable	UTS2
0.692761378	stable	GLIS1
0.692771453	stable	LOC642597
0.692870157	stable	IFT80
0.692973972	stable	FDX1
0.693009697	stable	LOC646401
0.693042556	stable	GALNT7
0.693042556	stable	LOC440248

0.693042556	stable	PCYT1A
0.693042556	stable	LOC728044
0.693042556	stable	KRTAP1-3
0.693065558	stable	FAU
0.693065978	stable	LOC652599
0.693092796	stable	PJA2
0.693092796	stable	URG4
0.693181207	stable	HTR3E
0.69327252	stable	SURF6
0.693284039	stable	LOC650392
0.693284039	stable	OR2M3
0.693284039	stable	COX6A1
0.693284039	stable	CALU
0.693363624	stable	LOC641852
0.693547909	stable	FLJ42709
0.693754015	stable	C1ORF32
0.693754015	stable	OR5A1
0.693791923	stable	LOC644091
0.693921804	stable	ST6GALNAC2
0.694102154	stable	C11ORF58
0.694110578	stable	KIAA0527
0.694143024	stable	LPIN3
0.694143024	stable	SI
0.694143024	stable	C22ORF34
0.694143024	stable	NFXL1
0.694389547	stable	PTPRJ
0.694430668	stable	SPANXE
0.694562667	stable	LOC155100
0.694805396	stable	AMELY
0.695028916	stable	RRP15
0.695098791	stable	C6ORF118
0.69546395	stable	LOC653052
0.69546395	stable	MAP1D
0.69546395	stable	LOC645489
0.695609876	stable	MYH11
0.695617937	stable	LOC649406
0.695786815	stable	LOC650144
0.6958106	stable	C21ORF41
0.696018456	stable	LOC651467
0.696089884	stable	ETNK1
0.696299945	stable	HSPCAL3
0.696357662	stable	LOC164380
0.696395741	stable	LOC401131
0.696395741	stable	DTL
0.696395741	stable	ATP10B
0.696395741	stable	LOC650436
0.696458322	stable	PRDM1
0.696495345	stable	LOC642769
0.696562567	stable	PRCP
0.696660024	stable	MAP3K3
0.696673762	stable	PDIA3

0.69667904	stable	GUSBL1
0.696695926	stable	OR6C2
0.696804154	stable	DACH1
0.696859731	stable	TMEM32
0.696867875	stable	LOC729335
0.696957056	stable	LOC651957
0.696957056	stable	LOC645396
0.696957056	stable	ITGAD
0.696957056	stable	SFRS6
0.696957056	stable	SLC22A2
0.696957056	stable	LOC652701
0.696957056	stable	LOC643684
0.696984761	stable	WDR36
0.696984761	stable	SERPINA9
0.696984761	stable	SF3A2
0.697059615	stable	ACTA2
0.697064877	stable	CHRNB2
0.697064877	stable	SLC25A35
0.69711758	stable	DNAH5
0.697191302	stable	LOC652703
0.697191302	stable	CHAD
0.697191302	stable	DGCR8
0.697221996	stable	AMBRA1
0.697221996	stable	C14ORF156
0.697245544	stable	LOC648527
0.697359418	stable	SPO11
0.697431751	stable	LOC151121
0.697449247	stable	BPY2B
0.697453295	stable	PTK6
0.697724037	stable	LOC728094
0.697726794	stable	RAB10
0.697754972	stable	C6ORF94
0.697864402	stable	LOC340511
0.697988475	stable	FLJ36032
0.698009067	stable	OR5D18
0.698009067	stable	SLC17A5
0.698039771	stable	CEPT1
0.698039771	stable	DPP8
0.698152645	stable	PSPN
0.698152645	stable	GRM2
0.698212479	stable	OR6C74
0.69827479	stable	B3GAT2
0.698312062	stable	STMN4
0.698312062	stable	TREM2
0.698398238	stable	KRTAP13-1
0.698661047	stable	MAP3K2
0.698682936	stable	C14ORF23
0.698752367	stable	SERF1A
0.698817693	stable	FRMD1
0.698817693	stable	VPS26
0.698826608	stable	FAM45B

0.698826608	stable	DUPD1
0.698826608	stable	RXFP4
0.698983089	stable	LOC732275
0.698983089	stable	C4ORF45
0.698983089	stable	ACO2
0.698983089	stable	DTX4
0.699034117	stable	EXOSC5
0.699065868	stable	CDK5RAP1
0.699137335	stable	TNP1
0.699216404	stable	ZNF80
0.699245414	stable	ZG16
0.699246899	stable	LOC649447
0.699249007	stable	LOC650657
0.699315147	stable	DKFZP686D0972
0.699315147	stable	C21ORF88
0.699315147	stable	STON1
0.699315147	stable	SNX26
0.699315147	stable	C6ORF185
0.699315147	stable	LOC641738
0.699315147	stable	LOC389300
0.699698857	stable	LDLRAD2
0.699698857	stable	SNHG3
0.699716735	stable	LOC255275
0.69995577	stable	C6ORF61
0.700002149	stable	ACOT6
0.700002149	stable	FAM124B
0.700157086	stable	TSPAN14
0.700157086	stable	PEX5L
0.700348831	stable	B4GALNT4
0.700423801	stable	LOC440309
0.700423801	stable	HHLA1
0.700423801	stable	LOC645690
0.700423801	stable	EPHA6
0.700423801	stable	LOC440421
0.700423801	stable	IL13RA2
0.700423801	stable	TUBGCP3
0.700473219	stable	APOA5
0.700473219	stable	SPANXN2
0.700473219	stable	LOC90925
0.70064755	stable	OR7G1
0.70064755	stable	GPR26
0.700727645	stable	BMP8A
0.700727645	stable	LOC645401
0.700759956	stable	PDCD1LG2
0.700769473	stable	LOC644088
0.700987152	stable	SHARPIN
0.701121581	stable	MAB21L2
0.701129946	stable	LOC284215
0.701200658	stable	KRT81
0.701203963	stable	LOC652416
0.70120647	stable	C16ORF89

0.701257847	stable	DEFB128
0.701257847	stable	RNF113B
0.701257847	stable	DUX3
0.701282424	stable	LOC651345
0.701312271	stable	LHX5
0.701312271	stable	OR10A6
0.701331664	stable	FLJ35946
0.701331664	stable	PCDHB12
0.701331664	stable	LOC649341
0.701331664	stable	NAALADL2
0.701331664	stable	HTATSF1
0.701331664	stable	C6ORF68
0.701331664	stable	PALM2
0.701331664	stable	KCTD2
0.701331664	stable	PTAFR
0.701413792	stable	PPP2R5B
0.701422081	stable	PCDHGB7
0.701468738	stable	FAM90A8
0.701639363	stable	LOC651675
0.701639363	stable	HIST1H3A
0.7017424	stable	KRTAP5-10
0.701764037	stable	LOC644014
0.701764037	stable	CEACAM5
0.701806734	stable	PCDHGA5
0.701813796	stable	PAX3
0.701813796	stable	SLIT1
0.701813796	stable	LOC727787
0.701813796	stable	LOC728003
0.701892758	stable	TUBGCP6
0.701916956	stable	PPP2R2C
0.701938105	stable	TNFRSF17
0.701956676	stable	RPL10L
0.7019704	stable	LOC653692
0.702229826	stable	CYLD
0.702255976	stable	LOC645268
0.702255976	stable	PRR5
0.702376046	stable	GNAT2
0.70238104	stable	LOC652669
0.702461202	stable	TEX101
0.702518615	stable	ZNF492
0.702528409	stable	FAM129C
0.702539911	stable	FLJ31222
0.702554238	stable	SLC10A1
0.702641297	stable	LOC645177
0.702654093	stable	KCNJ15
0.702958421	stable	UGT1A8
0.702958421	stable	LOC646686
0.702969759	stable	FLJ41352
0.702969759	stable	LOC644629
0.703155462	stable	LOC653411
0.703155462	stable	OC90

0.703222326	stable	LEMD1
0.703222326	stable	LOC647943
0.703222326	stable	LOC388458
0.703432588	stable	UPK3B
0.703432588	stable	PGGT1B
0.703432588	stable	LOC646043
0.703672842	stable	CDKN2A
0.703793731	stable	C12ORF23
0.703793731	stable	LOC645164
0.703939712	stable	BHLHB4
0.704177677	stable	LOC652854
0.704177677	stable	LOC400095
0.704177677	stable	LOC286310
0.704177677	stable	OR5D13
0.704177677	stable	MRO
0.704179456	stable	ABHD6
0.704188196	stable	LOC652272
0.704188196	stable	GNPTG
0.704202373	stable	C11ORF16
0.704401766	stable	LOC440871
0.704401766	stable	TGM5
0.704444071	stable	VDAC2
0.704510705	stable	LHX4
0.704536523	stable	FLJ42258
0.704536523	stable	LOC440551
0.704536523	stable	PHC2
0.704536523	stable	LOC441864
0.704620244	stable	CTDSP1
0.704641583	stable	BMP2KL
0.704641583	stable	LOC647533
0.704678968	stable	LOC650995
0.704678968	stable	KIAA1529
0.704692008	stable	HOXA10
0.70507765	stable	SENP3
0.705161985	stable	LOC646249
0.705371083	stable	SPEM1
0.705627641	stable	TJP3
0.705674068	stable	HAAO
0.705705507	stable	CBLN4
0.705843269	stable	LOC283849
0.705843269	stable	SOX10
0.705843269	stable	KBTBD9
0.705843269	stable	PGK1
0.705850238	stable	DNAJC16
0.705892874	stable	LOC643100
0.706016848	stable	FXN
0.706040351	stable	TCF1
0.706040351	stable	ERMN
0.706040351	stable	LOC653885
0.706171566	stable	OR2H2
0.706175266	stable	NAT11

0.706230796	stable	LOC648974
0.706230796	stable	ZFP42
0.706521599	stable	LOC441698
0.706521599	stable	MAST3
0.706521599	stable	LOC388259
0.706531943	stable	SULT2A1
0.706724803	stable	ROPN1L
0.706738493	stable	LELP1
0.706738493	stable	FAM150A
0.706738493	stable	OSTN
0.707205917	stable	LOC647540
0.707208562	stable	FAM133B
0.707208562	stable	LOC642112
0.707208562	stable	MKKS
0.707294411	stable	L3MBTL2
0.707294411	stable	LOC648840
0.707294411	stable	LOC645955
0.707294411	stable	OR52B4
0.707294411	stable	LOC442261
0.707294411	stable	OR2M2
0.707294411	stable	MANEA
0.707521287	stable	LOC653418
0.707845623	stable	OR6C3
0.70787133	stable	NCR3
0.70787133	stable	FLJ37587
0.70787133	stable	DNASE1L2
0.70787133	stable	TADA2L
0.707901851	stable	FLJ16793
0.707965941	stable	WDFY1
0.707980423	stable	LOC652456
0.707983207	stable	ZNF77
0.708041428	stable	BARX2
0.708081747	stable	FABP2
0.708185155	stable	UNQ6975
0.708185155	stable	LOC730241
0.708185155	stable	EXDL2
0.708185155	stable	LOC645585
0.708185155	stable	SLC15A4
0.708201632	stable	HECW1
0.708202764	stable	KIAA0100
0.708238936	stable	LOC642497
0.708284723	stable	LOC158825
0.708284723	stable	LRRC36
0.708284723	stable	LOC441233
0.708284723	stable	CASP6
0.708284723	stable	LOC389786
0.708284723	stable	LOC554226
0.708346447	stable	NUBP1
0.708503873	stable	OR6S1
0.70856465	stable	ERVWE1
0.708567939	stable	NMBR

0.708567939	stable	LOC644640
0.708735648	stable	LOC652148
0.708749683	stable	LOC732313
0.708834356	stable	LOC652633
0.708834356	stable	CPNE9
0.708834356	stable	ZNF547
0.708834356	stable	SMTNL1
0.708834356	stable	HMP19
0.708834356	stable	ZNF498
0.708834356	stable	LOC644419
0.708834356	stable	TMCO2
0.708842931	stable	LOC374973
0.708865928	stable	LOC652515
0.708922495	stable	SLC16A10
0.709119929	stable	LOC650887
0.709119929	stable	LOC652034
0.709119929	stable	LOC646110
0.709174733	stable	OR7G2
0.709174733	stable	LNX1
0.709262387	stable	EPHA4
0.709483466	stable	LOC652635
0.709503955	stable	ACAD9
0.709601889	stable	LOC442132
0.709750347	stable	LOC650668
0.709906689	stable	GFAP
0.709906689	stable	LOC646691
0.709972589	stable	ZNF780B
0.710004682	stable	LOC646976
0.710127967	stable	CEND1
0.710127967	stable	CCDC89
0.710127967	stable	CXCR4
0.710127967	stable	OR4N2
0.710127967	stable	NDUFA13
0.710127967	stable	OR4X1
0.710127967	stable	CLC
0.710127967	stable	C7ORF13
0.710176064	stable	FAM47A
0.710176064	stable	C15ORF54
0.710176064	stable	LOC402232
0.710392674	stable	RFPL3
0.710399328	stable	KRTAP4-8
0.710487352	stable	SERPINB10
0.710487352	stable	CAGE1
0.710696016	stable	UBE2V1
0.710696016	stable	GRPR
0.710774379	stable	LOC646730
0.710851612	stable	SGSM2
0.710928211	stable	AVPR2
0.710928211	stable	TBL1Y
0.710928211	stable	NPVF
0.710928211	stable	ZDHHC2

0.710994781	stable	ADAM33
0.711184608	stable	NXNL2
0.711269109	stable	WSB2
0.711269109	stable	LOC651580
0.711269109	stable	HAPLN2
0.711308906	stable	LOC647234
0.711308906	stable	PLSCR5
0.711308906	stable	COL11A1
0.711361234	stable	FN3KRP
0.711384805	stable	LOC649940
0.711389365	stable	LOC643619
0.711402337	stable	LOC645537
0.711404479	stable	TTC23L
0.711690284	stable	LOC221981
0.711690284	stable	LOC652871
0.711759351	stable	LOC646335
0.711759351	stable	C9ORF165
0.711762574	stable	PADI6
0.711839881	stable	NALCN
0.71188938	stable	NACA2
0.711971277	stable	FAM162B
0.711971277	stable	OR6K3
0.712057502	stable	GCUD2
0.712057502	stable	LOC649878
0.71232867	stable	SH3BGRL
0.712329383	stable	LOC641912
0.712329383	stable	LOC642132
0.712524752	stable	LOC652678
0.712524752	stable	FSHB
0.712524752	stable	ZNF391
0.712540689	stable	LOC643855
0.712578334	stable	PCDHB5
0.712623591	stable	ACSBG1
0.712651452	stable	LOC642403
0.712776855	stable	LOC650120
0.712776855	stable	NR1D2
0.712776855	stable	LOC388955
0.712836847	stable	LOC642111
0.713012107	stable	MLC1
0.713140047	stable	CYP2E1
0.713213083	stable	LOC646947
0.713238946	stable	LOC651578
0.713238946	stable	KRTAP19-4
0.713238946	stable	TNFRSF13C
0.713238946	stable	LOC147710
0.713238946	stable	LOC642539
0.713238946	stable	LOC650673
0.713267233	stable	ASB7
0.713435965	stable	LOC389888
0.713436849	stable	ADH6
0.713480901	stable	NBPF9

0.713603093	stable	GABRQ
0.713701231	stable	LOC652115
0.713800419	stable	FAM90A2P
0.713800419	stable	RAB14
0.713915728	stable	LOC652195
0.713915728	stable	ADCY9
0.713915728	stable	IQGAP3
0.713944091	stable	TAF11
0.714268076	stable	LOC650439
0.714277991	stable	FASLG
0.71428976	stable	C7ORF46
0.714343238	stable	ANKMY1
0.714343238	stable	LOC652799
0.714453386	stable	GJB2
0.714558925	stable	SCNN1G
0.714598531	stable	PCDHGB2
0.714749171	stable	SPN
0.714819524	stable	LOC401442
0.714972578	stable	ZNF346
0.714972578	stable	LOC651558
0.714972578	stable	RPS13
0.715108061	stable	WHSC1L1
0.715205374	stable	EOMES
0.715205374	stable	SESN2
0.715303117	stable	LOC644677
0.715303117	stable	PABPC3
0.71530405	stable	LOC283711
0.71530405	stable	LOC642383
0.71530405	stable	TRIML1
0.71530405	stable	LOC648791
0.7153076	stable	LOC643037
0.7153076	stable	LOC728498
0.715394908	stable	LOC645162
0.715606068	stable	LOC392583
0.715606068	stable	ZNF672
0.715606068	stable	MAPT
0.715606068	stable	VPS13B
0.715606068	stable	TRIM44
0.715606068	stable	LOC645785
0.715633968	stable	POP4
0.715655526	stable	RPL9
0.715655526	stable	LOC643809
0.715655526	stable	MMPL1
0.715655526	stable	LOC653636
0.715655526	stable	LOR
0.715679461	stable	C13ORF31
0.715679461	stable	LOC729558
0.715789442	stable	ADM2
0.715789442	stable	LOC440570
0.715789442	stable	LENG9
0.715789442	stable	LOC645355

0.715789442	stable	LOC154449
0.715789442	stable	BCLAF1
0.715998696	stable	SRD5A2
0.715998696	stable	CAMSAP1L1
0.715998696	stable	C17ORF37
0.716123901	stable	C6ORF146
0.716215956	stable	NANOG
0.716363697	stable	LRIT1
0.716363697	stable	SLC25A24
0.716363697	stable	SLC10A2
0.716479394	stable	LOC642155
0.716582741	stable	LOC729175
0.716582741	stable	ARHGAP17
0.716582741	stable	KIAA1919
0.716582741	stable	LOC388885
0.716582741	stable	LOC401911
0.716978181	stable	LOC649210
0.717080071	stable	LOC647836
0.717222303	stable	C13ORF26
0.717453001	stable	LOC731438
0.717453001	stable	GSG1L
0.717455661	stable	LOC643749
0.717455661	stable	FAM99A
0.717480943	stable	PRM2
0.717594886	stable	LOC645113
0.717594886	stable	LOC653136
0.717626744	stable	RTN4RL2
0.717878781	stable	SPOPL
0.717885721	stable	PIK3C2G
0.717926982	stable	FLJ32658
0.718102628	stable	PCP4L1
0.718152741	stable	PIGX
0.718152741	stable	PLEKHG7
0.718206548	stable	POLR3G
0.718328826	stable	HS6ST2
0.718328826	stable	MED19
0.718328826	stable	LOC646677
0.718328826	stable	WNT7A
0.718328826	stable	BCORL1
0.718328826	stable	LOC648040
0.718328826	stable	CHIA
0.718328826	stable	ICF45
0.718328826	stable	ARHGAP25
0.718328826	stable	LOC401206
0.718328826	stable	CLDN20
0.718328826	stable	LOC441551
0.718554067	stable	LOC390998
0.718554067	stable	SMG6
0.718554067	stable	USF1
0.718554067	stable	FLJ44186
0.718554067	stable	CRYAA

0.718615573	stable	LRRC4B
0.718615573	stable	MALAT1
0.718615573	stable	OR2L2
0.718615573	stable	LOC651006
0.718769386	stable	LOC650038
0.718819491	stable	KIAA1524
0.718819491	stable	LOC283340
0.718819491	stable	TBN
0.718819491	stable	PGLYRP3
0.718819491	stable	PTPRT
0.718827977	stable	INCA
0.718827977	stable	IL8RB
0.718830085	stable	TRIM6-TRIM34
0.718952234	stable	CXCR5
0.718952234	stable	CRISP2
0.718952234	stable	LOC613266
0.718952234	stable	MYLK
0.719020991	stable	CXCL17
0.719020991	stable	IL34
0.719135922	stable	EPOR
0.719199425	stable	LOC652710
0.719299882	stable	GSTO1
0.719353373	stable	ALKBH1
0.719353373	stable	PBX2
0.719417631	stable	EIF4E1B
0.719417631	stable	LOC644010
0.719417631	stable	C9ORF122
0.719430269	stable	VAT1
0.719518657	stable	LY6G6E
0.71956888	stable	LOC647784
0.71956888	stable	SLC17A1
0.719602965	stable	XAGE5
0.719627416	stable	IQSEC2
0.719627416	stable	UBE1
0.719627416	stable	LOC642370
0.719627416	stable	LOC641788
0.719627416	stable	LOC652142
0.719924992	stable	THSD3
0.720010118	stable	C1QTNF8
0.720010118	stable	LOC648898
0.720113192	stable	LOC643213
0.720294274	stable	PDHA1
0.720353036	stable	LOC648130
0.720361801	stable	OR52M1
0.720433642	stable	LPAR5
0.720454424	stable	LOC645448
0.720482053	stable	FAM82A2
0.720743324	stable	MEST
0.720801855	stable	KRTAP5-9
0.720837742	stable	LCE3E
0.721004759	stable	C11ORF24

0.721004759	stable	COLQ
0.721008984	stable	RPRM
0.721129468	stable	C3ORF27
0.721159132	stable	ZNF718
0.721178397	stable	SAGE1
0.721223332	stable	LOC644617
0.721223332	stable	PURG
0.721269437	stable	IMPG2
0.721311393	stable	ELAC2
0.721340976	stable	MAP3K14
0.721437374	stable	E2F7
0.721516743	stable	TSPAN19
0.721525884	stable	AFM
0.721553076	stable	LOC651315
0.721553076	stable	GPR158L1
0.721553076	stable	FKSG83
0.721553076	stable	ZNF440
0.721963078	stable	TCERG1L
0.721998138	stable	GPR12
0.721998138	stable	LOC727994
0.721998138	stable	CXORF21
0.721998138	stable	TCOF1
0.722003706	stable	LOC647363
0.72207201	stable	C6ORF70
0.722087816	stable	ACD
0.722097654	stable	SLC16A1
0.722124221	stable	LOC646863
0.722172103	stable	RPAP2
0.722285333	stable	LOC648237
0.722285333	stable	EFHB
0.722285333	stable	LOC642650
0.722285333	stable	B3GALT2
0.722285333	stable	LOC642354
0.722285333	stable	ATAD3C
0.722285333	stable	LOC650406
0.722376974	stable	OR5B3
0.72239982	stable	LCAP
0.72239982	stable	HSFY2
0.722452343	stable	LOC652692
0.722452343	stable	ST7OT1
0.722452343	stable	C11ORF70
0.72269877	stable	LAIR1
0.72269877	stable	SPINK5
0.722800999	stable	LOC642677
0.722877716	stable	GNAZ
0.722906126	stable	LOC643331
0.722906126	stable	LOC643069
0.723186449	stable	LOC648365
0.723186449	stable	LOC652281
0.723186449	stable	GPR172B
0.723217784	stable	WBP11P1

0.723371081	stable	PPBP
0.723380815	stable	ZNF267
0.723380815	stable	SLC13A3
0.723380815	stable	STRADB
0.723380815	stable	TMEM103
0.723380815	stable	HCN1
0.723380815	stable	C9ORF47
0.723380815	stable	LOC644574
0.723380815	stable	LOC643576
0.723380815	stable	LOC283682
0.723380815	stable	LOC148709
0.723447708	stable	LOC341112
0.723623995	stable	RPS28
0.723906215	stable	OR9G9
0.724026296	stable	LOC643910
0.724026296	stable	SACM1L
0.724026296	stable	TRIML2
0.724029317	stable	LOC643130
0.724029317	stable	LOC387927
0.724029317	stable	BCL10
0.724029317	stable	MRPS2
0.724104329	stable	LOC392617
0.724104329	stable	APBA1
0.724104329	stable	SYNGR2
0.724120128	stable	PRF1
0.724155039	stable	C14ORF138
0.724156237	stable	MCPH1
0.724156237	stable	WNT16
0.724311069	stable	BCL7A
0.724311069	stable	LOC648521
0.724311069	stable	KCNK13
0.724311069	stable	TESSP1
0.724649312	stable	LOC642568
0.72479928	stable	FANCM
0.724830292	stable	LOC650058
0.724830292	stable	GAS5
0.724830292	stable	LOC728193
0.724830292	stable	C11ORF36
0.724830292	stable	DDB1
0.724830292	stable	ZNF452
0.724869205	stable	LOC644736
0.724869205	stable	MRPL15
0.72503902	stable	OTUB2
0.72503902	stable	GLRA2
0.725091861	stable	IFNB1
0.725091861	stable	IL8RBP
0.725091861	stable	LOC124220
0.725091861	stable	LRRC18
0.725195561	stable	LOC440434
0.725240538	stable	PDZD11
0.725288238	stable	MS4A3

0.725288238	stable	LOC345537
0.725288238	stable	LOC399829
0.725288238	stable	LOC644762
0.725288238	stable	LOC389151
0.725310781	stable	TTTY12
0.725315887	stable	PARD6B
0.725315887	stable	PTPRN
0.72544728	stable	LHFPL1
0.72555192	stable	LOC643075
0.72555192	stable	RAB11FIP4
0.72555192	stable	C17ORF67
0.72558048	stable	LOC200261
0.72559698	stable	LOC643302
0.72559698	stable	LOC643435
0.725655795	stable	LOC649255
0.725655795	stable	LOC641938
0.725769549	stable	VCY1B
0.725774702	stable	LOC284293
0.72592567	stable	LOC652155
0.72592567	stable	LOC730226
0.72592567	stable	FLJ37543
0.725952406	stable	LOC642121
0.726229063	stable	OR7E156P
0.726229063	stable	NLRP11
0.726282437	stable	LOC652597
0.726334374	stable	SPIB
0.726338212	stable	SPSB2
0.726358446	stable	LOC646697
0.726513279	stable	LOC401497
0.7265433	stable	OR2F1
0.726623303	stable	ENTPD7
0.726715966	stable	HSPC159
0.72672313	stable	CCKAR
0.72672469	stable	SCN4A
0.726751926	stable	SNAI1
0.727151842	stable	LOC650147
0.727288583	stable	LSM14A
0.727288583	stable	OR10P1
0.727406773	stable	OR52E4
0.727709223	stable	TMEM63B
0.727709223	stable	LOC440005
0.727709223	stable	LOC442117
0.727728336	stable	LOC284260
0.727728336	stable	LOC642861
0.727728336	stable	LOC727855
0.727728336	stable	PGRMC2
0.7277689	stable	LOC442229
0.7277689	stable	TSSK3
0.7277689	stable	CPA1
0.7277689	stable	LOC642726
0.7277689	stable	LOC392275

0.7277689	stable	CACYBP
0.727819375	stable	LOC727732
0.727851855	stable	LOC347364
0.727956523	stable	PSG11
0.727956523	stable	CACNA1G
0.727956523	stable	LRIT3
0.727956523	stable	ZNF761
0.727967225	stable	KIAA1957
0.727967225	stable	LOC730242
0.72805001	stable	LOC647212
0.728132278	stable	LOC649246
0.728132278	stable	LOC654172
0.728239675	stable	LOC647447
0.728260975	stable	PBEF1
0.728376979	stable	LOC653676
0.728376979	stable	CGB5
0.728486558	stable	LOC644100
0.728486558	stable	TSPAN6
0.728486558	stable	LOC648868
0.728731578	stable	SMR3A
0.728731578	stable	INTS7
0.728731578	stable	EIF4G2
0.728731578	stable	SPATC1
0.728731578	stable	BRUNOL5
0.728731578	stable	FXC1
0.728890754	stable	C19ORF45
0.728939434	stable	KIAA1644
0.728939434	stable	DDX52
0.728939434	stable	HPX
0.728939434	stable	METRN
0.728939434	stable	MGC16291
0.728941797	stable	LOC653310
0.728967759	stable	CSN1S1
0.728967759	stable	GIF
0.728967759	stable	CMPK2
0.728967759	stable	LOC642035
0.728967759	stable	LOC646296
0.728967759	stable	KRTAP23-1
0.729017009	stable	LOC648894
0.729017009	stable	PYHIN1
0.72914343	stable	LOC652408
0.729180092	stable	RAP2A
0.729286705	stable	LOC641959
0.729286705	stable	EFNA1
0.729286705	stable	LOC647521
0.729286705	stable	LOC652471
0.729286705	stable	LOC645143
0.729294761	stable	GIGYF1
0.729294761	stable	LOC399937
0.729475461	stable	LOC345222
0.729575455	stable	LOC642223

0.729618807	stable	DLX5
0.729629489	stable	FLJ46481
0.729629489	stable	TNS4
0.729629489	stable	LOC653853
0.729656455	stable	LOC654127
0.729656455	stable	C6ORF81
0.729656455	stable	SMOC1
0.72965673	stable	OTOG
0.729658327	stable	KIAA1285
0.729658327	stable	IGSF8
0.729666324	stable	TACR1
0.729746892	stable	KRTAP4-11
0.729748027	stable	BRDT
0.729761305	stable	SNX20
0.729761305	stable	OTUB1
0.729761305	stable	PF4V1
0.729864973	stable	UBE2E1
0.729919863	stable	LOC641848
0.729919863	stable	C15ORF41
0.729919863	stable	LOC648625
0.729919863	stable	MYH16
0.730165577	stable	LOC644431
0.730174932	stable	LOC645411
0.730181642	stable	IFNA6
0.730245801	stable	NT5M
0.730245801	stable	CSH1
0.730245801	stable	EPB42
0.730333137	stable	LOC732156
0.73043721	stable	LOC648931
0.730498999	stable	LOC647529
0.730514396	stable	RPL37A
0.730514396	stable	LOC390205
0.730514396	stable	CRNN
0.730514396	stable	LOC728302
0.73060859	stable	FCGR2C
0.730758608	stable	GSTA3
0.730831983	stable	LOC256223
0.730831983	stable	SLC24A4
0.730861664	stable	LOC646818
0.730861664	stable	T
0.730997599	stable	EHF
0.730997599	stable	LOC651481
0.730997599	stable	LOC649661
0.730997599	stable	DECR1
0.730997599	stable	SLC35A3
0.731057874	stable	MDM1
0.731057874	stable	LOC651536
0.731189242	stable	STON1-GTF2A1L
0.731207751	stable	LOC727942
0.731207751	stable	CCL22
0.731207751	stable	LOC642943

0.731207751	stable	MMACHC
0.731207751	stable	KRTAP5-8
0.731207751	stable	SLC10A4
0.731207751	stable	GKN2
0.731255931	stable	LOC644946
0.731275362	stable	TSHZ2
0.731341059	stable	LOC731236
0.731372752	stable	AANAT
0.731397708	stable	POLI
0.731439051	stable	LOC653129
0.731548879	stable	PCDHGA2
0.731558284	stable	TACR2
0.731614269	stable	MAGEA8
0.731614812	stable	COX5B
0.731614812	stable	AMIGO3
0.731635988	stable	LOC730050
0.73171664	stable	SMC1B
0.731729257	stable	KIR3DL2
0.731729257	stable	SHOC2
0.731729257	stable	YJEFN3
0.732098118	stable	LOC401507
0.732109896	stable	CYTH1
0.732156006	stable	C10ORF64
0.732184539	stable	OR2D3
0.732270854	stable	LOC654133
0.732270854	stable	CACNG6
0.732322559	stable	LRRC26
0.732322559	stable	PAFAH1B1
0.732415028	stable	ASPHD2
0.732480967	stable	AGPAT5
0.732521964	stable	CYP4F11
0.732760917	stable	LOC728056
0.732760917	stable	HIST1H1B
0.732760917	stable	PLK4
0.732815024	stable	SPANXA1
0.732836408	stable	OR5H2
0.732836408	stable	LOC401286
0.732908153	stable	DFNB31
0.733144933	stable	LOC646914
0.733144933	stable	WDR53
0.733518602	stable	FLJ12334
0.733618263	stable	DAAM2
0.73363006	stable	LOC646154
0.73393823	stable	FLJ32214
0.734138916	stable	FLJ44060
0.734138916	stable	LOC647998
0.734138916	stable	ZNF410
0.734138916	stable	RNF133
0.73439037	stable	ASPN
0.734455129	stable	PEG10
0.734455129	stable	LOC650795

0.734455129	stable	HLA-DRB5
0.734466749	stable	API5
0.734536811	stable	SPDYC
0.734536811	stable	LOC644528
0.73467951	stable	MPZ
0.734692914	stable	CCIN
0.734889706	stable	TAGLN3
0.734889706	stable	LRRC56
0.734936917	stable	DOCK3
0.734946397	stable	SPRR1A
0.734946397	stable	MANBAL
0.735059335	stable	LOC644836
0.735059335	stable	LOC727820
0.735059335	stable	UCP1
0.735068987	stable	LOC644320
0.735068987	stable	DUSP8
0.735075085	stable	IQSEC3
0.735091942	stable	GPR81
0.735091942	stable	LOC645908
0.735091942	stable	LOC644809
0.735295273	stable	LOC653089
0.735295273	stable	OR5R1
0.735311114	stable	LOC654342
0.735330535	stable	PTPRH
0.735356711	stable	CEP97
0.735428757	stable	LOC388948
0.735615621	stable	CYTH3
0.735641183	stable	LOC651450
0.735641183	stable	FAM83A
0.735703794	stable	ZRANB1
0.735854109	stable	CNTD2
0.735858311	stable	VILL
0.735858311	stable	C10ORF30
0.735858311	stable	TAF13
0.735858311	stable	CABP4
0.735858311	stable	LHFPL4
0.735858311	stable	YWHAG
0.735937632	stable	SLC30A4
0.735939575	stable	C1ORF173
0.735949295	stable	ZNF257
0.736051877	stable	PFN2
0.736093561	stable	LOC649442
0.736462558	stable	LRRIQ4
0.736498196	stable	LOC147645
0.736498196	stable	STK16
0.736498196	stable	NPAS1
0.736498196	stable	C21ORF129
0.736535775	stable	EDG4
0.736535775	stable	LOC647264
0.736577866	stable	FBXO24
0.736646204	stable	HMGCLL1

0.736646204	stable	HLA-G
0.736676105	stable	LOC646720
0.736676105	stable	ATP6AP2
0.736993739	stable	GPX5
0.736993739	stable	TLR10
0.736993739	stable	LOC342900
0.736993739	stable	LOC645144
0.737003554	stable	N4BP3
0.73708653	stable	MRPL9
0.73708653	stable	C1ORF31
0.737171056	stable	KIAA0774
0.737171056	stable	PDZD3
0.737171056	stable	JARID1D
0.737171056	stable	BZRPL1
0.737171056	stable	SNORD116-4
0.737219952	stable	LOC440287
0.737219952	stable	FSD1
0.737229556	stable	ADRBK2
0.737300178	stable	UTS2R
0.737300178	stable	LOC391025
0.737300178	stable	LOC391209
0.737300178	stable	LOC653180
0.737326402	stable	RSAD2
0.737326402	stable	UTP23
0.737326402	stable	SCGB3A2
0.737370372	stable	LRRC57
0.737370372	stable	CGN
0.737421109	stable	LOC648211
0.737435658	stable	PCDHA2
0.737673019	stable	C4ORF28
0.737673019	stable	LOC646999
0.737694723	stable	MLLT1
0.737741355	stable	LOC649700
0.737741355	stable	LOC652707
0.737893909	stable	SNX31
0.737916241	stable	MYH8
0.737978288	stable	OR1L3
0.737978288	stable	FCRLA
0.737978288	stable	LOC653799
0.738002675	stable	IGF2BP2
[truncated: 237,846 more chars]
